# Formation of the Vasculogenic Mimicry Phenotype in Melanoma Mel Z Cells Is Coupled with Changes in Inter-Chromosomal Contacts of Developmental Genes with rDNA Clusters

**DOI:** 10.3390/ijms26168085

**Published:** 2025-08-21

**Authors:** Nickolai A. Tchurikov, Elena S. Klushevskaya, Viktoriya N. Lukicheva, Antonina N. Kretova, Elizaveta N. Poperekova, Vladimir R. Chechetkin, Galina I. Kravatskaya, Amalia A. Vartanian, Vyacheslav S. Kosorukov, Ildar R. Alembekov, Yuri V. Kravatsky

**Affiliations:** 1 Department of Epigenetic Mechanisms of Gene Expression Regulation, Engelhardt Institute of Molecular Biology Russian Academy of Sciences, 119334 Moscow, Russia; 2 Department of Experimental Diagnosis and Therapy of Tumors, N.N. Blokhin National Medical Research Center of Oncology of the Ministry of Health of Russia, 115478 Moscow, Russia

**Keywords:** melanoma, vasculogenic mimicry, Matrigel, 4C-rDNA, development, co-regulated genes

## Abstract

Upon transferal from plastic to Matrigel, melanoma cells demonstrate growth in three dimensions and form de novo vascular networks—known as vasculogenic mimicry—that are characteristic of the stemness phenotype of aggressive tumors. It has been reported that during malignant transformation, stress, or differentiation, the long-range inter-chromosomal interactions between numerous developmental genes and nucleoli are changed. The aim of this work was to study the potential mechanisms behind the development of the vasculogenic mimicry phenotype in melanoma cells and whether the formation of these 3D structures is connected with the reorganization of inter-chromosomal contacts of rDNA clusters. Here, we show that after 15 h of growth on Matrigel, and following the formation of the vasculogenic mimicry phenotype, dramatic changes occur in Mel Z cells in rDNA contacts with different genomic regions that possess mainly developmental genes. Approximately 400 genes that retained stable contacts with nucleoli were co-expressed with different lincRNAs and were highly associated with H3K27me3 marks and simultaneously regulated by different transcription factors. These genes are involved in development and cell adhesion and may control the basic stage of differentiation. The genes that acquired or increased contacts with rDNA clusters during growth on Matrigel are associated with cell morphogenesis, cell junctions, and the cytoskeleton. Here, we present the first evidence that nucleoli may be involved in both the activation and repression of particular groups of developmental rDNA-contacting genes in melanoma cells forming the vasculogenic mimicry phenotype. We conclude that the inter-chromosomal interactions between developmental genes and rDNA clusters are dynamic, and that nucleoli play an important role in the development of vasculogenic mimicry and stemness phenotypes in aggressive tumor genes.

## 1. Introduction

Vasculogenic mimicry (VM) is an adaptive mechanism developed by malignant tumors in response to hypoxia [1,2]. As a result, vascular channels, cords, and sinuses that lack endothelial cells are formed, while in the course of normal angiogenesis, vessels are formed by endothelial cells [3,4]. The development of such 3D capillary-like structures provides malignant cells with oxygen and nutrition, and as a result, cancer cells survive. The VM phenotype is associated with the stemness and aggressive behavior of cancer cells [5]. Melanoma, the most aggressive type of skin cancer, originates from melanocytes. The pattern of melanoma cells in tumors resembles a honeycomb structure characteristic of the VM phenotype [6]. Aggressive melanoma Mel Z cells are known to quickly create 3D capillary-like structures when grown on Matrigel [7]. This model provides an opportunity to study the underlying molecular mechanisms involved in the formation of this phenotype and examine the stemness behavior of melanoma cells.

Changes in cell phenotype are determined by changes in gene expression patterns that are triggered by different endogenous and exogenous factors. Previously, it was discovered that during malignant progression, rDNA clusters reorganize their inter-chromosomal contacts, and these changes are correlated with gene expression changes [8]. rDNA clusters located in five human acrocentric chromosomes (13, 14, 15, 21, and 22) form in each cell cycle nucleoli. It has also been demonstrated that multiple types of dynamic contacts between nucleoli and developmental genes occur upon stress and development [9,10], while data suggest that the formation of VM phenotypes in melanoma cells is coupled with changes in the pattern of rDNA-contacting genes. Transcriptome analysis has revealed extreme epigenetic changes in the expression patterns of Mel Z melanoma cells grown in the same medium after being transferred from a plastic surface to Matrigel [11].

The aim of this research was to study the possible changes in the patterns of rDNA-contacting genes in the same Mel Z cells that, under different growth conditions, either did or did not form capillary-like structures. The cells were isolated at N.N. Blokhin National Medical Research Center of Oncology of the Ministry of Health, Russia, from metastasis in a lymph node from a patient with disseminated melanoma [12]. The cells were grown either in 2D monolayers on plastic or in 3D on Matrigel. In the latter case, the cells formed capillary-like structures. Matrigel is used as an extracellular matrix for 3D cell cultures and is known to alter gene expression patterns and the development of VM phenotypes in cancer cells [13].

Here, we used the 4C (circular chromosome conformation capture) approach to compare the whole-genome inter-chromosomal contacts of rDNA clusters in Mel Z cells grown on either plastic or Matrigel. Our data strongly indicate that the development of the VM phenotype is coupled with dramatic changes in these contacts. We found that genes that form new contacts or increase the number of contacts with nucleoli are highly associated with cell morphogenesis, the cell junction, and the cytoskeleton. On the other hand, genes that maintain stable contacts with nucleoli are highly associated with development, lincRNAs, and H3K27me3 marks, and are simultaneously regulated by different transcription factors. We conclude that rDNA clusters play a role in the development of VM and stemness phenotypes in cancer cells.

## 2. Results

### 2.1. Transferring Melanoma Cells from a Plastic Surface to Matrigel Leads to the Formation of the VM Phenotype and Changes in rDNA Inter-Chromosomal Contacts

**Figure 1:**
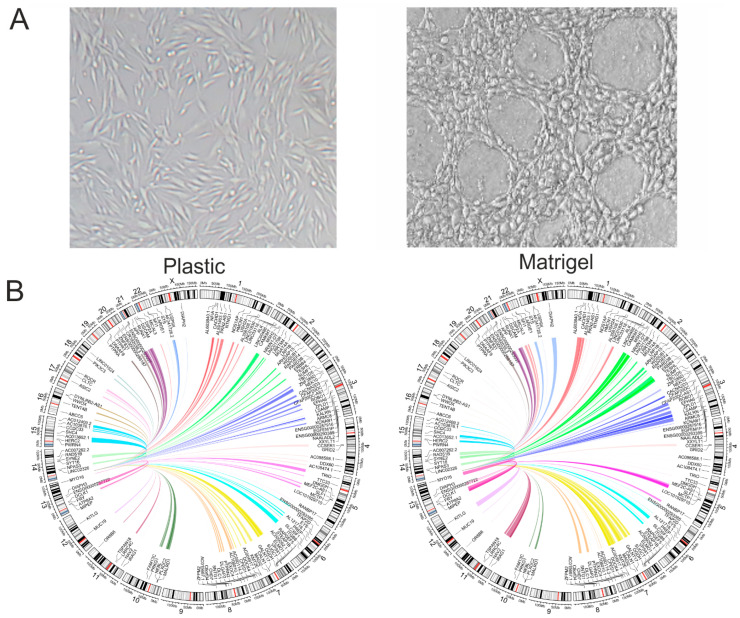
Effects of growth conditions on the behavior of Mel Z cells. (**A**) Melanoma cells grown for 15 h on plastic or on Matrigel. (**B**) Circos presentations of rDNA contacts representing the top 150 genes with the highest statistically significant differences in the number of contacts in cells growing either on plastic or on Matrigel. Only one rDNA unit was included at the tip of chr14. The contacts in chr5 and chr11 are shown in fuchsia and purple colors, respectively.

We expected to observe changes in rDNA contacts induced by transferring cells from plastic to Matrigel and subsequent growth for 15 h (see Methods Section). This transfer and rather short incubation time are sufficient to allow cells to develop 3D capillary-like structures [12,13,14,15,16]. Figure 1A shows the phenotypes of melanoma Mel Z cells grown on plastic and Matrigel. When in a monolayer on the plastic surface, the Mel Z cells appeared to consist of spindle-shaped cells. On Matrigel, the cells formed capillary-like structures, which are associated with cells acquiring a partly rounded shape. Obviously, this phenotype is formed under the control of a specific set of genes and factors in response to cultivation on Matrigel. To check whether this response to growth in three dimensions could induce changes in the inter-chromosomal contacts of specific patterns of genes with rDNA clusters, we used the 4C-rDNA approach (see Methods Section).

Figure 1B shows that in our 4C-rDNA experiments, the transfer to Matrigel induced changes in particular regions in all chromosomes. For example, in the 14M region of chr5, there were many contacts inside the *TRIO* gene in the cells grown on plastic. The number of contacts with rDNA greatly reduced during the 15 h of growth on Matrigel. The gene is involved in coordinating actin remodeling, which is necessary for cell migration and growth [17]. Conversely, in the same chromosome at the 94M region, the *SLF1* gene grown on Matrigel strongly increased the number of contacts with rDNA clusters. The gene plays a role in the DNA damage response [18]. Similarly, in chr11, the *NAV2* gene exhibited very frequent contacts during 15 h of growth on Matrigel. This gene plays a role in cellular growth, cytoskeleton reorganization, and migration. Table S1 provides a full list of rDNA-contacting genes in Mel Z cells grown either on plastic or on Matrigel. To systematically study the nature of rDNA-contacting genes in melanoma cells during the formation of the VM phenotype, we used the search function in the Gene Ontology database.

### 2.2. Analysis of Biological Processes in Which rDNA-Contacting Genes in Mel Z Cells Are Involved

**Figure 2:**
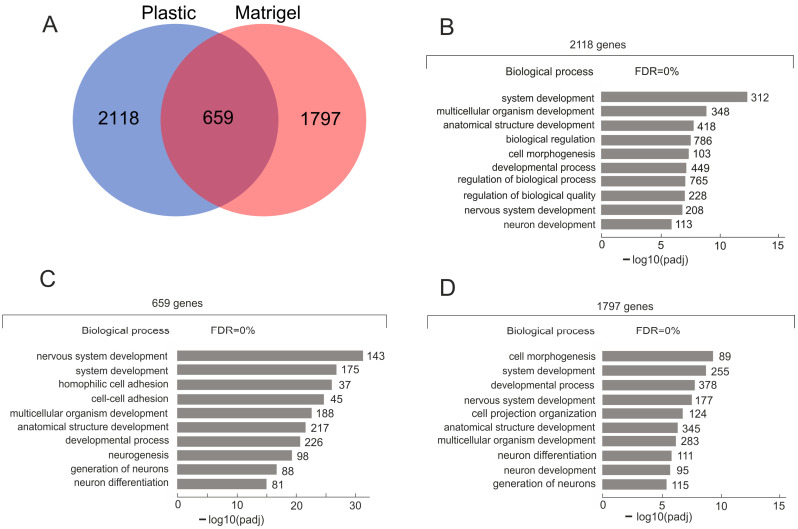
Characterization of rDNA-contacting genes in Mel Z cells growing either on plastic or on Matrigel. (**A**) Venn diagram showing intersections with rDNA-contacting genes in Mel Z cells growing either on plastic or on Matrigel. The complete list of genes is shown in Table S2. (**B**) Top 10 Gene Ontology (GO) biological process associations of 2118 genes using g:Profiler (https://biit.cs.ut.ee/gprofiler/gost, accessed on 1 August 2025). Values to the right of bars show the number of genes associated with a process. The complete list of GO items and the corresponding genes are shown in Table S3. (**C**) Top 10 Gene Ontology (GO) biological process associations of 659 genes that are shared by cells cultivated on plastic or on Matrigel. The complete list of GO items and the corresponding genes are shown in Table S4. (**D**) Top 10 Gene Ontology (GO) biological process associations of 1797 genes that are characteristic of cells cultivated on Matrigel. The complete list of GO items and the corresponding genes are shown in Table S5.

The Venn diagram in Figure 2A shows that during the formation of the VM phenotype, the reorganization of inter-chromosomal contacts of nucleoli occurred. For this study, we selected only the genes with the most frequent contacts—those possessing ≥ 30 contacts with rDNA (Table S1). We found that 659 genes preserved the contacts and kept their interactions with rDNA clusters in cells while growing on Matrigel (Table S2). A search of these three groups of genes, shown in Figure 2A, was performed in the GO database. We found that 2118 genes that are characteristic only of cells growing on plastic are associated with development (Figure 2B; Table S3). At the same time, a new set of 1797 genes increased or acquired contacts during the formation of the VM phenotype.

All three sets of rDNA-contacting genes are highly associated with development. Half of the GO items overlap in Figure 2B–D. Nevertheless, the lists of genes do not overlap. This means that members of the same GO item can behave differently during the formation of the VM phenotype. For example, *ZNF10* from the group of 2118 genes is classified as a “biological regulation” item (GO: 0065007) and behaves as a transcriptional repressor. At the same time, *ZNF229* from the group of 1797 genes also corresponds to the same GO item, which is a transcriptional activator.

All three groups of genes shown in Figure 2A have one property in common: they are enriched with rDNA-contacting lincRNA genes. Among the 2118 rDNA-contacting genes that are only characteristic of the cells grown on plastic, there are 276 lincRNA genes (13%). Similarly, among the 1797 genes that make contact with rDNA only in Mel Z cells grown on Matrigel, there are 276 other lincRNA genes (15%). The 659 overlapping genes possess 83 genes for lincRNAs (12.6%). These data confirm previous observations that rDNA-contacting genes are associated with lincRNAs [9,10].

To understand the main functions, processes, and cellular components of particular GO items, we performed a search using Gene Ontology driver terms (https://biit.cs.ut.ee/gprofiler/gost, accessed on 1 August 2025) [19]. Figure S1 shows that for the genes that are only characteristic of cells grown on plastic, the driver is “system development”. The rDNA-contacting genes shared by cells growing on plastic and Matrigel are driven by “nervous system development” (*p*-value = 2.7 × 10^−30^), “hemophilic cell adhesion”, and “cell junction” (*p*-value = 3.8 × 10^−24^). The genes frequently making contact with rDNA clusters only in the cells grown on Matrigel are driven by “cell morphogenesis”, “cytoplasm”, and “cytoskeleton”.

### 2.3. The Amount of Contacts Between Genes and rDNA Clusters in Mel Z Cells Changes Significantly

**Figure 3:**
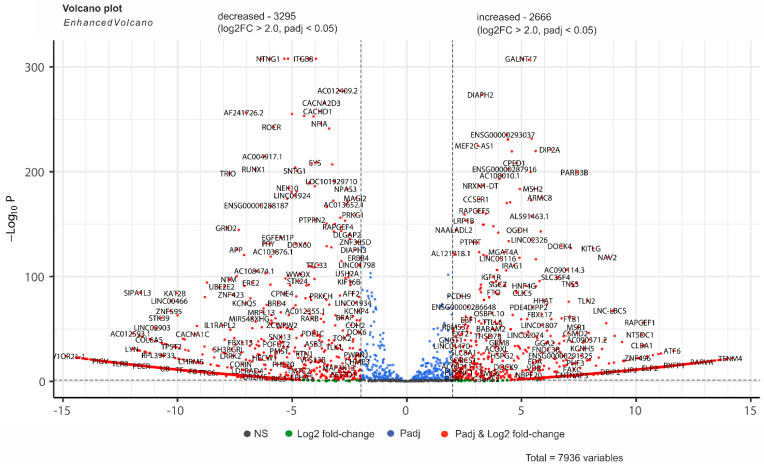
Differential 4C-rDNA analysis. Melanoma cells grown for 15 h on plastic or on Matrigel. The volcano plot presents statistically significant log_2_ fold changes in the number of contacts of genes with rDNA clusters. The *p*-values were corrected using the Benjamini–Hochberg method for multiple testing that is built into DESeq2. A complete list of genes is shown in Table S6.

Figure 2 does not illustrate the range in which the changes in the number of contacts occur during the growth of Mel Z cells on Matrigel. This is why we present the data using differential 4C-rDNA analysis (see Methods Section). We found that, under log2FC and padj < 0.05, there are 3295 genes whose number of contacts with nucleoli decreased and 2666 genes whose contacts increased (Figure 3). The width of log2 fold changes shown in Figure 3 is up to ±15. Table S6 presents the corresponding data. Interestingly, under these conditions, there were 188 lincRNAs and 83 ZNF genes whose number of contacts with nucleoli decreased. This is coupled with an increase in contacts of 186 lincRNAs and 31 ZNF genes. For example, both ZNF595 and ZNF496 are transcriptional regulators, but they behave differently in cells growing on Matrigel: ZNF595’s number of contacts with nucleoli decreased (log2FC = −10), while, at the same level, ZNF496’s increased. These data suggest that rDNA-contacting genes are strongly associated with lincRNAs and with the regulation of the transcription of ZNF genes.

### 2.4. Only 398 Genes That Stably Retain the Numbers of Their Contacts with rDNA Clusters Are Co-Expressed with Numerous lincRNAs and Are Highly Associated with H3K27me3 Marks

**Figure 4:**
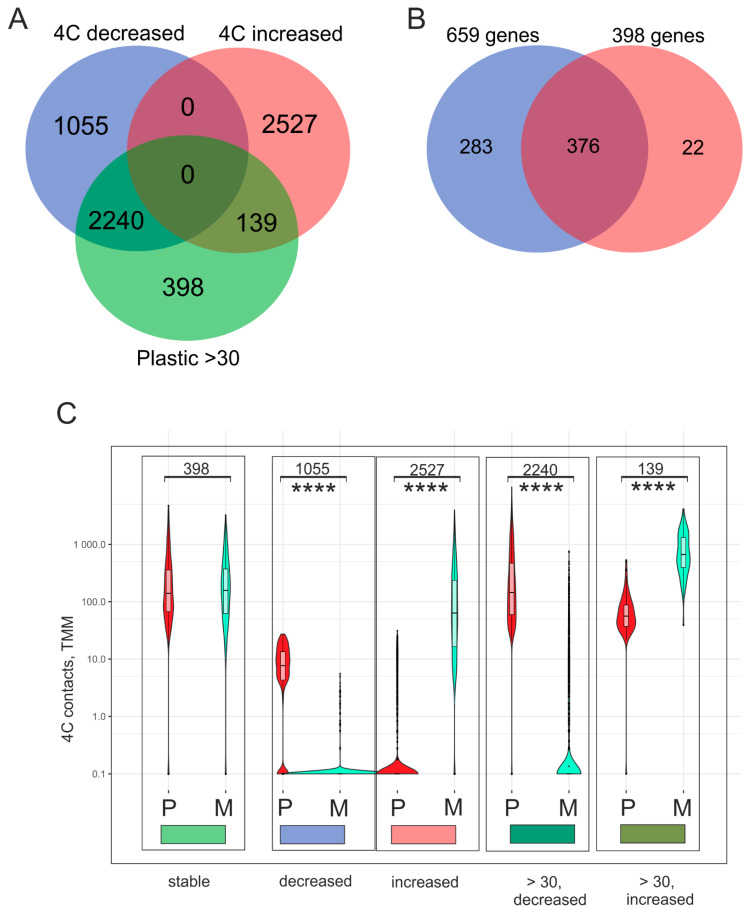
Identification of genes that retained their contacts with rDNA clusters while growing on plastic (P) or Matrigel (M). (**A**) The Venn diagram shows the intersections of rDNA-contacting genes detected in Mel Z cells grown on plastic (≥30 contacts) with genes whose number of contacts either decreased or increased after growth on Matrigel for 15 h. Lists of the corresponding genes are presented in Tables S6 and S7. (**B**) Venn diagram showing the intersections between the common rDNA-contacting genes detected in Mel Z cells grown on plastic and Matrigel (Figure 2) and with the 398 genes shown in (**A**). (**C**) Violin diagrams showing the number of 4C contacts in the five groups of genes shown in the Venn diagram (TMMs—trimmed means of M-values—are shown in Table S1). The numbers of genes shown in the Venn diagram are indicated at the top. **** *p*-values < 10^−6^.

Next, we were interested in determining the nature of genes that maintain stable contacts with nucleoli in Mel Z cells that do not change when grown on Matrigel. It is likely that they are involved in controlling the remaining state of development in Mel Z cells when forming the VM phenotype. A volcano plot was created that shows that there were 3295 genes whose contacts decreased and 2666 genes whose contacts increased. We found that in the cells cultivated on plastic, there were 2777 genes possessing ≥30 contacts with rDNA clusters (Figure 2A). To identify the genes that possessed stable contacts with nucleoli, we used a Venn diagram to present the results (Figure 4). We found that 398 rDNA-contacting genes preserved their association with rDNA clusters, and the number of contacts remained the same. Clearly, these genes are a major part of common rDNA, contacting 659 genes shared by the cells growing on both plastic and Matrigel (Figure 4B).

Figure 4C illustrates the shifts in the number of contacts for these five groups of genes with rDNA clusters. Violin plots show the distribution of normalized 4C contacts of these genes in the cells growing on plastic (P) or on Matrigel (M). The number of contacts did not change for 398 genes. A group of 1055 genes possessed fewer than 30 contacts, and this number decreased dramatically. In addition, the number of contacts decreased for 2527 genes in the cells cultivated on plastic, but highly increased in those grown on Matrigel. In contrast, 2240 genes had ≥30 contacts that were mainly lost, while 139 genes that also had ≥30 contacts obtained more. To understand the reason for these events, we searched the GO database for these groups of genes.

**Figure 5:**
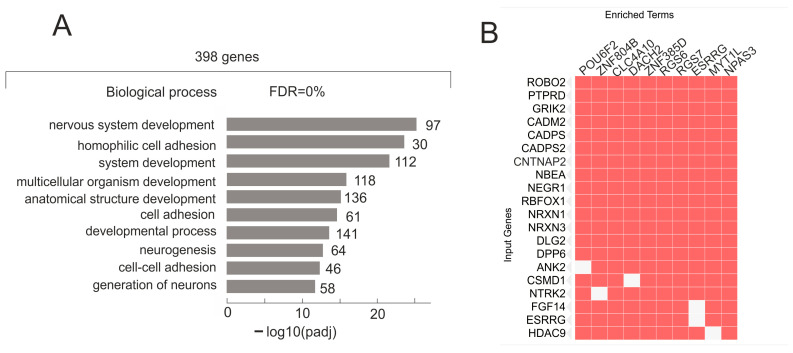
Characterization of 398 rDNA-contacting genes exhibiting no change in contacts with nucleoli in Mel Z cells grown on Matrigel. (**A**) Top 10 Gene Ontology (GO) biological process associations of 398 genes using g:Profiler (https://biit.cs.ut.ee/gprofiler/gost, accessed on 1 August 2025). Values to the right of bars show the number of genes associated with a process. A complete list of GO items and corresponding genes is shown in Tables S7 and S8. (**B**) A total of 398 genes are simultaneously regulated by different transcription factors (“enriched terms”). The top 20 genes are shown (“input genes”). Data were obtained by searching for corresponding genes in Enrichr Submissions TF-Gene Cooccurrence (https://maayanlab.cloud/Enrichr, accessed on 1 August 2025). A complete list of the corresponding TFs is shown in Table S12.

A total of 398 genes retained their contacts when grown for 15 h on Matrigel during the formation of the VM phenotype. This group contains genes highly involved in “nervous system development”, “homophilic cell adhesion via plasma membrane adhesion molecules”, “cell junction”, and “neuron projection” (Figure 5A and Figure S2). The associations of these genes with GO terms are shown in more detail in Table S8. The group included 27 lincRNAs (Table S7), and 91 of the 398 genes were co-expressed in different combinations with 55 different lincRNAs (Table S9). Among them were 15 *PCDHA* genes from the Protocadherin Alpha Cluster that are involved in cell–cell connections in the brain [20]. This result is consistent with the GO data for this group of genes, indicating their role in cell junctions and adhesion (Table S8).

Only this group of 398 genes that form stable contacts with nucleoli is highly associated with the repressive H3K27me3 mark in differently cultured and normally differentiated human cells, including bronchial epithelial cells, CD14-positive monocytes, kidney epithelial cells, and cardiac mesoderm cells (Table S10). The 174 genes from this group that are associated with this H3K27me3 mark are listed in Table S11. It is likely that this silencing mark in a big set of rDNA-contacting genes is essential for maintaining the differentiation state of these cell types. Figure 5B shows that these 398 genes are simultaneously regulated by 391 different transcription factors, including 93 ZNFs and 10 POU genes. One particular gene corresponds to between 13 and 79 transcription factors (*p*-value up to 2.9^−63^). Data were obtained using a search of Enrichr Submissions TF-Gene Cooccurrence (https://maayanlab.cloud/Enrichr/enrich, accessed on 1 June 2025).

The remaining four groups of rDNA-contacting genes shown in the Venn diagram in Figure 4A, as a whole, exhibited no associations with co-expressing lincRNAs or with H3K27me3 marks.

### 2.5. Numerous Genes Whose Number of Contacts with rDNA Clusters Increased After Growth on Matrigel Are Involved in Development and Morphogenesis and Are Subject to Silencing

**Figure 6:**
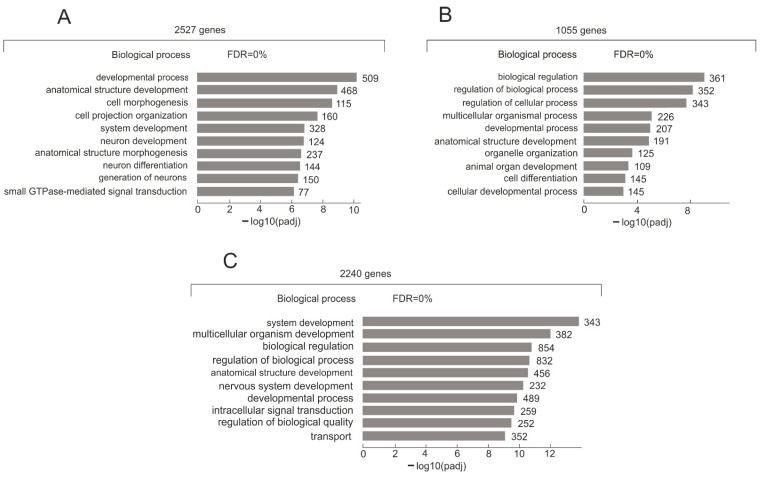
Characterization of three groups of rDNA-contacting genes shown in Figure 4A. (**A**) Top 10 Gene Ontology (GO) biological process associations of 2527 genes using g:Profiler (https://biit.cs.ut.ee/gprofiler/gost, accessed on 1 August 2025). Values to the right of bars show the number of genes associated with a process. A complete list of GO items and corresponding genes is shown in Table S13. (**B**) Top 10 Gene Ontology (GO) biological process associations of 1055 genes using g:Profiler (https://biit.cs.ut.ee/gprofiler/gost, accessed on 1 August 2025). Values to the right of bars show the number of genes associated with a process. A complete list of GO items and corresponding genes is shown in Table S14. (**C**) Top 10 Gene Ontology (GO) biological process associations of 2240 genes using g:Profiler (https://biit.cs.ut.ee/gprofiler/gost, accessed on 1 August 2025). Values to the right of bars show the number of genes associated with a process. A complete list of GO items and corresponding genes is shown in Table S15.

To understand the biological functions associated with 2527 genes whose contacts with nucleoli increased during the formation of the VM phenotype by Mel Z cells, we performed a Gene Ontology (GO) search. Figure 6A shows the top ten GO biological process items for these 2725 genes. Large groups of genes are highly associated with development and morphogenesis. A complete list of GO molecular functions, biological processes, and cellular components is provided in Table S13. The group includes 168 lincRNA genes and 32 ZNF genes.

**Figure 7:**
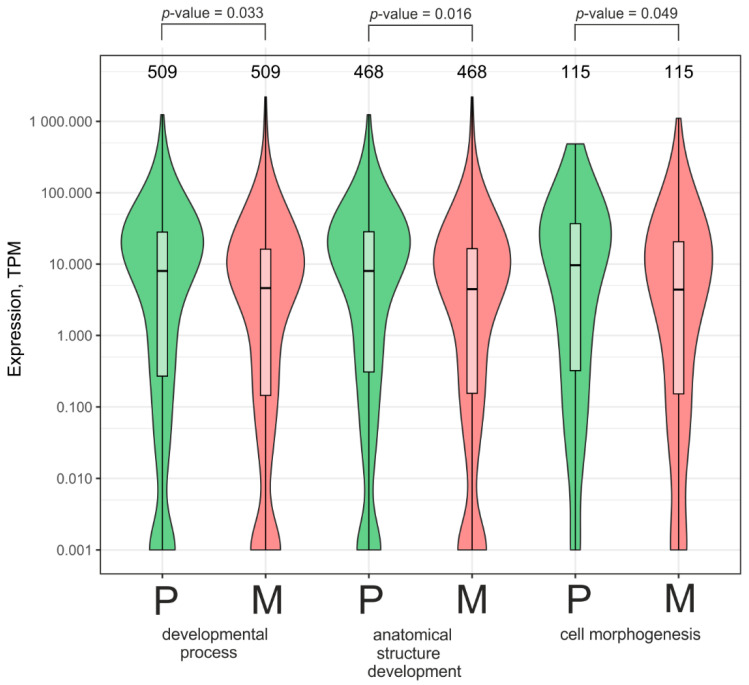
Expression of genes whose number of contacts with nucleoli increased (group of 2527 genes in Figure 4A). Violin plot of the expression of three sets of genes controlling developmental processes, anatomical structure development, and cell morphogenesis in MelZ cells cultivated either on plastic (P) or on Matrigel (M). The numbers of corresponding genes are indicated at the top. Related to Figure 6A.

It is known that growth on Matrigel can induce either the dedifferentiation of some cell types or the further differentiation of other cell types [21,22,23]. To check whether the expression of the rDNA-contacting genes associated with development and morphogenesis from the group of 2527 genes whose number of contacts with nucleoli increased is activated or repressed when grown on Matrigel, we studied their expression using RNA-Seq data for Mel Z cells cultivated on plastic and Matrigel. We found that large groups of genes associated with developmental and anatomical processes and structural development (509 and 468 genes, respectively) are repressed approximately twofold on Matrigel (Figure 7). Similarly, a smaller group of genes involved in morphogenesis (115 genes) is also repressed more than twofold. This conclusion is in agreement with the data on the formation of capillary-like structures by malignant Mel Z cells (Figure 1A). It might follow that the more frequent contacts between genes controlling development, anatomical structure development, and cell morphogenesis and nucleoli led to their silencing.

Interestingly, 115 rDNA-contacting genes that control morphogenesis are highly associated with 133 lincRNAs and 126 human miRNAs (Tables S16 and S17, respectively). These small non-coding RNAs post-transcriptionally silence different mRNAs [24]. Taken together, the data suggest that these genes that control morphogenesis are subjected to silencing. Therefore, Mel Z cells cultivated on Matrigel should be in a less differentiated state than those grown on plastic.

### 2.6. Numerous Genes Whose Number of Contacts with rDNA Clusters Decreased While Growing on Matrigel Are Involved in Development and Biological Regulation

Figure 4 shows that there are two large groups of genes whose number of contacts with rDNA clusters decreased. The first group of 2240 genes possessed 30 or more contacts each when Mel Z cells were cultivated on plastic and included 132 lincRNA genes and 57 ZNF genes. In contrast to the 398 genes with stable contacts, this group, as a whole set, exhibited no associations with lincRNAs and H3K27me3 marks.

The same is true for the second-largest group of 1055 rDNA-contacting genes that also exhibited a decrease in the number of contacts. This group included 52 lincRNA genes and 25 ZNF genes.

Both groups of 2240 and 1055 genes have 4 of the top 10 GO items in common for biological processes (Figure 6B,C). However, as shown in the Venn diagram (Figure 4), these groups possess different genes, including different genes for the same GO items. Therefore, they might be involved in different mechanisms of “biological regulation” in the course of VM phenotype development. For example, the group of 1055 genes possessing 361 members of the “biological regulation” item includes the *TWIST1* gene that promotes tumor cell invasion and metastasis (Table S13). At the same time, the group of 2240 genes has 854 different genes from the same GO item, including the *GATAD2B* gene, which encodes a zinc finger protein transcriptional repressor (Table S15).

**Figure 8:**
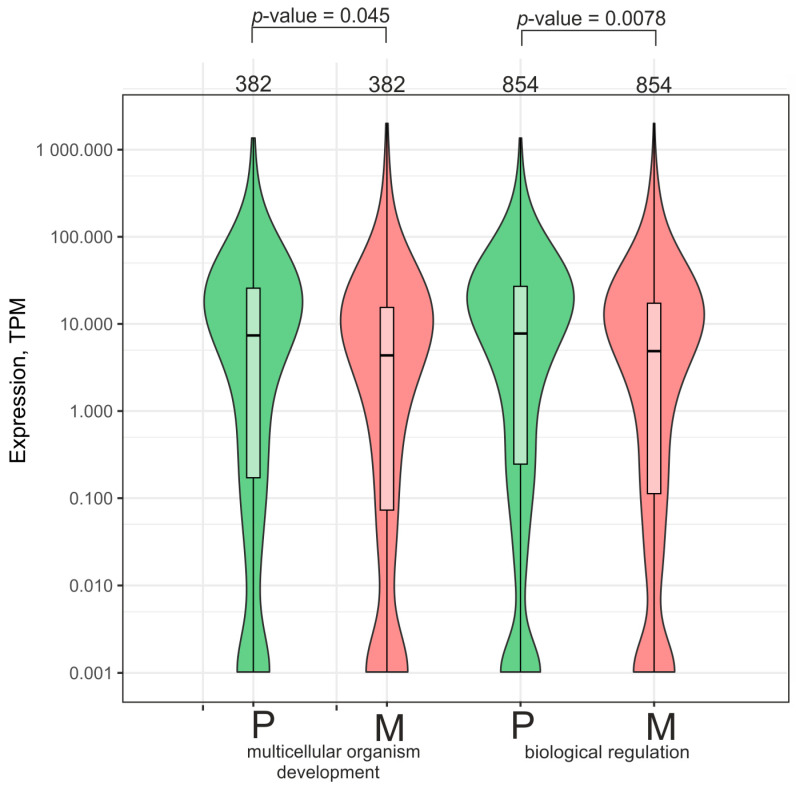
Violin plot of the expression of genes controlling multicellular organism development and biological regulation in Mel Z cells cultivated either on plastic (P) or on Matrigel (M). The numbers of corresponding rDNA-contacting genes in a GO item are indicated at the top. The groups of 382 and 854 genes correspond to the group of 2240 genes whose number of contacts with nucleoli decreased (Figure 4; Table S14). Related to Figure 6C.

To check whether the decrease in contacts with nucleoli could affect the expression of rDNA-contacting genes, we analyzed a large set of 2240 genes associated with two GO items: “multicellular organism development” and “biological regulation” (Table S15). We observed that genes in both sets (382 and 854 genes) decreased their activity approximately twofold (Figure 8). It follows that the loss of contact resulted in a reduction in the expression of the corresponding genes. This means that the more frequent contacts in cells grown on plastic provided more active transcription of these genes controlling development and regulation.

It has been proposed that rDNA clusters may be involved in both the repression and activation of developmental genes [25]. Our data agree with this supposition. Figure 7 shows that the genes whose number of contacts with rDNA increased are subjected to repression. Figure 8 shows that the genes whose number of contacts decreased are also subjected to silencing. However, this can be explained if we assume that when the genes are repressed by the contacts, the increase in their numbers leads to more efficient repression. Similarly, if the genes are activated while in contact with nucleoli, the loss of contact leads to a reduction in activation and also leads to silencing.

## 3. Discussion

### 3.1. Development of Vasculogenic Mimicry Is Connected with the Functions of rDNA-Contacting Genes

The VM phenotype is associated with the stemness and aggressive behavior of cancer cells [1]. Current anti-angiogenic drugs induce hypoxia in both the tumor and its microenvironment, and hypoxia, in turn, induces VM [1]. This is why it is important to uncover the basic molecular mechanisms behind the development of the VM phenotype.

Our data strongly indicate that the VM phenotype formed by Mel Z cells cultivated on both plastic and Matrigel is associated with multiple sets of genes controlling different biological functions, including development and morphogenesis. Three groups of genes that retained, lost, or gained contacts with nucleoli are highly associated with development.

In recent decades, the novel functions of nucleoli beyond their obvious role in the synthesis and formation of ribosomes have been discussed [25,26,27]. The first experimental evidence of direct contacts between nucleoli and particular chromosomal regions in *Drosophila* was demonstrated in [28]. Later, the use of the 4C approach confirmed these data, and numerous rDNA contacts were detected within 2.5–20 kb precision in Drosophila and human cells [8,9,10,29]. These contacts are described as being dynamic and often occur in developmental genes [8,9,10,29]. This is why, for Mel Z cells, we expected the most frequent contacts with rDNA clusters to be detected in these genes.

Based on previous results suggesting that developmental genes often form contacts with nucleoli, we hypothesize that rDNA-contacting genes should be detected in both the initial Mel Z cells cultivated on plastic and in the cells growing on Matrigel, mainly corresponding to developmental genes. Our data strongly confirmed this supposition. We found that during the rather short 15 h incubation time of the cells on Matrigel, dramatic changes in the pattern of these contacts occurred. It follows that during the formation of the VM phenotype induced by Matrigel, deep alterations in inter-chromosomal contacts occur.

At present, we do not know whether these changes in Mel Z cells are reversible. It is known that the cultivation of one type of cell on Matrigel can be reversed by replating uncoated plastic dishes [30], while other types of cells are only partially reversible [31]. Our data indicate that the number of contacts between numerous rDNA-contacting genes controlling developmental processes, anatomical structure development, and morphogenesis and nucleoli increases, and these genes are silenced. It follows that Mel Z cells grown on Matrigel become less differentiated.

### 3.2. Genes Forming Stable Contacts with Nucleoli Are Strongly Associated with lincRNAs and H3K27ac Marks

Our data indicate that only a small part of rDNA-contacting genes are common for cells grown on plastic and Matrigel. These common genes do not change the number of contacts with rDNA (Figure 2A and Figure 4B). They form stable contacts with nucleoli and are likely involved in maintaining the achieved state of differentiation in Mel Z cells. These genes are simultaneously regulated by different transcription factors.

Previously, it was demonstrated that rDNA-contacting genes are associated with lincRNAs, miRNAs, and H3K27me3 marks in HEK293T and K562 cells [9,11,32]. Here, we found that this is true for the genes that form stable contacts with rDNA clusters in Mel Z cells cultivated on Matrigel.

It is known that lincRNAs play important roles in the epigenetic regulation of key molecular processes, including histone modification and chromatin dynamics [33]. LincRNAs reside in chromatin and play different roles there, including recruiting transcription factors, activating or repressing gene expression, and participating in the formation of 3D chromatin structures [34].

The H3K27me3 mark is associated with the epigenetic silencing of developmental genes [35]. Previously, an analysis of rDNA-contacting genes in HEK293T, K652, and hESM01 cells revealed that they have a large group of genes in common that are associated with development and morphogenesis [36]. Our data on Mel Z cells agree with this finding. We observed that parts of rDNA-contacting genes maintain stable contacts with nucleoli, are apparently involved in maintaining the differentiation of the cell, and are associated with the H3K27me3 mark in different cultured and differentiated human cells. The putative role of the contacts with nucleoli in the origin of this mark is not yet clear.

### 3.3. Possible Role of lincRNAs and ZNF Genes in Inter-Chromosomal Interactions

There are data suggesting that lincRNAs might serve as navigators and play a targeting role in the inter-chromosomal interactions between developmental genes and nucleoli [32]. Many lincRNA genes belong to rDNA-containing genes. For example, Figure 2 shows 2120 genes that are only characteristic of cells grown on plastic and 1797 genes found only in Mel Z cells cultivated on Matrigel. Each group includes approximately 300 lincRNA genes. It has been proposed that the co-localization of coding developmental rDNA-contacting genes with lincRNAs may make it possible to guide particular chromosomal regions to nucleoli [32]. Recently, it was found that ZNF274 anchors target DNA sequences at the nucleolus and facilitates their compartmentalization [37]. The above-mentioned groups of genes are also enriched through contact between ZNF genes and nucleoli.

There are examples of the targeting role of small RNA molecules of different classes. siRNAs, piRNAs, and miRNAs possess nucleotide stretches complementary to DNA or RNA molecules. Within RISC complexes, small RNAs recognize particular regions in the genome or transcriptome and play important roles in gene expression regulation. LincRNAs also could possess complementary nucleotide stretches to DNA or RNA sequences. For example, enhancer RNAs (eRNAs) are longer molecules, up to 1000 nt long, that often act locally in the proximity of corresponding genes, may originate from foreign DNA located close to enhancer sequences, and work efficiently with target promoters [38].

Short sequences in lincRNAs, resembling seeds in miRNAs, may be complementary to short stretches in nascent rRNA molecules and, in this way, may recognize nucleoli with the aid of putative protein complexes. Our preliminary search revealed complementary sequences between different lincRNAs associated with rDNA-contacting genes and different sense and antisense rRNAs, including pRNA, PAPAS, and ETS (a detailed analysis will be published separately). Currently, we are investigating the capacity of particular lincRNAs and ZNF genes to play a role in targeting chromosomal regions possessing developmental genes to nucleoli.

### 3.4. How Nucleoli May Be Involved in Both the Activation and Repression of rDNA-Contacting Genes

It is now obvious that nucleoli interact with different chromosomal regions in a non-random way. The propensity of these interactions with developmental genes is well proven [8,9,10,28,39]. However, the possible mechanism by which nucleoli mediate the regulation of gene expression is not yet clear.

Based on the fact that rDNA-containing genes are regulated simultaneously by many transcription factors [9,10,11], we speculate that micro-drops are formed via liquid–liquid phase separation mechanisms around nucleoli containing numerous transcription factors. Nucleoli, which are built up by liquid–liquid and liquid–solid phase transitions [26], may form micro-drops of a similar nature around themselves.

The second argument in favor of this supposition comes from the observation that long stretches of H3K27ac marks, from 5 to 50 kb, along human chromosomes precisely coincide with numerous rDNA contact sites [39]. These regions of broad H3K27ac marks are considered super-enhancers [40]. Recently, it was postulated that such regions are phase-separated [41].

A local unusually high density of interacting factors should change the properties of the solution and lead to phase separation. We suppose that this might happen with factors that induce silencing through the deposition of repressive marks, e.g., the H3K27me3 mark by *Polycomb* repressive complex 2. Previously, it was demonstrated that about 500 common rDNA-contacting genes controlling development and morphogenesis in three different human cell lines are highly associated with the H3K27me3 mark [36]. Here, we observed that in different cells, including differentiated human cells, the rDNA-contacting genes detected in Mel Z cells are also associated with the H3K27me3 mark.

Taken together, these data support the view that nucleoli have the capacity to activate and repress developmental genes. Our data on the repression of rDNA-contacting genes detected in several GO items (Figure 7 and Figure 8) are clearly consistent with this supposition. Currently, we are investigating whether some factors are concentrated around the nucleoli.

## 4. Materials and Methods

### 4.1. Cell Culture

The derivation and preparation of the Mel Z melanoma cell line has been described previously [11]. The cells were obtained from the N.N. Blokhin National Medical Research Center at the Oncology of the Ministry of Health, Russia. The cells were initially propagated on a plastic surface in an RPMI-1640 medium supplemented with 10% fetal calf serum, 2 mM glutamine, and 0.1% gentamicin sulfate at 37 °C in a humidified atmosphere containing 5% CO_2_ and up to 10 million cells with 70–75% confluency. Then, the cells were divided into two equal samples and seeded either on plastic or Matrigel. They were allowed to grow under identical conditions for 15 h; then, the medium above the cells was removed, and the cells from plastic and Matrigel were liberated by 20 min incubation at 37 °C in 5 mL of Versene solution in Dulbecco’s phosphate-buffered saline containing 0.05% trypsin. The cells were then used for 4C experiments and for RNA isolation.

Petri dishes coated with Matrigel (BD Bioscience, Bedford, MA, USA) were prepared as follows: the Matrigel (8.7 mg/mL) was thawed at 4 °C, and 6 mL was quickly added to each 10 cm dish and allowed to solidify for 30 min at room temperature; then, it was placed in a humidified 5% CO_2_ incubator for 1 h at 37 °C.

The RNA-Seq analysis process for cells grown on plastic and Matrigel was described previously [11].

### 4.2. 4C-rDNA Procedure

The DNA samples used for the 4C experiments were isolated according to the procedures previously described [9]. The cells were then fixed in 1.5% formaldehyde, and the nuclei were isolated. Then, digestion with a 6-cutter EcoRI enzyme and the ligation of extensively diluted DNA to favor intramolecular ligations were performed. To shorten the ligated DNA fragments, digestion with a 4-cutter FaeI endonuclease was performed, followed by the ligation of diluted DNA samples to favor circularization and to minimize dimerization. The primers 5′ TCTTTGAAAAAAATCCCAGAAGTGGT 3′ and 5′ AAGTCCAGAAATCAACTCGCCAGT 3′ for 4C-rDNA were selected inside the IGS (intergenic spacer in rDNA genes), as previously described [9]. The final DNA samples were used for the preparation of DNA libraries that were subjected to deep sequencing using Illumina HiSeq 2500 Rapid v2 (Illumina, San Diego, CA, USA), using 150 nt long reads.

The numbers of reads obtained were as follows: for Mel Z cells grown on the plastic substrate, replicate 1 was 11,906,125 and replicate 2 was 11,165,136; for Mel Z cells grown on Matrigel, replicate 1 was 15,399,177 and replicate 2 was 14,262,711. The 4C-rDNA raw data, corresponding to two pairs of biological replicates under both conditions (plastic and Matrigel), were deposited in the GEO database under accession number GSE295545.

### 4.3. 4C Mapping and Processing

The processing of Mel Z 4C-rDNA-associated region reads was conducted as follows: Since 4C-specific adapters are relatively long sequences, their complete trimming from the reads is a crucial step to ensure accurate downstream genome mapping. Cutadapt [42] 4.9 was used to trim 4C-specific adapters separately from direct (R1) and reverse (R2) reads through the following multi-step procedure:

1. 4C full-length adapters (both direct and reverse complement (RC)):
  >A1D TTCACTTCTGACATCCCAGATTTGATCTCCCTACAGAATGCTGTACAGAACTGGCGAGTTGATTTCTGGACTT
  >A1RC AAGTCCAGAAATCAACTCGCCAGTTCTGTACAGCATTCTGTAGGGAG
  ATCAAATCTGGGATGTCAGAAGTGAA
  >A2D TCTTTGAAAAAAATCCCAGAAGTGGTTTTGGCTTTTTGGCTAGGAGGC
  CTAAGCCTGCTGAGAACTTTCCTGCCCAGGATCCT
  >A2RC AGGATCCTGGGCAGGAAAGTTCTCAGCAGGCTTAGGCCTCCTAGCCA
  AAAAGCCAAAACCACTTCTGGGATTTTTTTCAAAGA
  were removed at 5′ ends with the options -O 10 (minimal overlap adapter with the read), --trim-n (omit Ns at the ends of reads), --times = 4 (search for the adapter up to 4 times in the read consequently), --minimum-length 20 (minimum acceptable read length after trimming), and -q 24 (minimal acceptable quality). All untrimmed reads were separated into a particular file for further processing.
2. Illumina 3′ adapter arrays from AGATCGGAAGAGC to AGATCGGAAGAGCNNNNNNNNNN and from GATCGGAAGAGC to GATCGGAAGAGCNNNNNNNNNN anchored to the 3′ end of reads were deleted using cutadapt with the following options: -O 10 (minimal overlap adapter with the read), --times = 4 (search for the adapter up to 4 times in the read), --minimum-length 20 (minimum acceptable read length after trimming), and -q 24 (minimal acceptable quality).
3. Incomplete 5′ 4C full-length adapter arrays from TTCACTTCTGACATCCCAGATTTGATCTCCCTACAGAATGCTGTACAGAACTGGCGAGTTGATTTCTGGACTT to TTCACTTCTGACATCCCAGA (minimal length = 20) and from TCTTTGAAAAAAATCCCAGAAGTGGTTTTGGCTTTTTGGCTAGGAGGCCTAAGCCTGCTGAGAACTTTCCTGCCCAGGATCCT to TCTTTGAAAAAAATCCCAGA (minimal length = 20), both direct and reverse complement, anchored to the 5′ end of reads, were filtered out using cutadapt with the same options as above.
4. Illumina adapter GATCGGAAGAGC and IlluminaPE adapter AGATCGGAAGAGC were removed using cutadapt from the 3′ ends of reads with the following options: -O 5 (minimal overlap adapter with the read), --times = 4 (search for the adapter up to 4 times in the read), --minimum-length 20 (minimum acceptable read length after trimming), and -q 24 (minimal acceptable quality).
5. Incomplete 3′ 4C full-length adapter arrays from TTCACTTCTGACATCCCAGATTTGATCTCCCTACAGAATGCTGTACAGAACTGGCGAGTTGATTTCTGGACTT to GCGAGTTGATTTCTGGACTT (minimal length = 20) and from TCTTTGAAAAAAATCCCAGAAGTGGTTTTGGCTTTTTGGCTAGGAGGCCTAAGCCTGCTGAGAACTTTCCTGCCCAGGATCCT to ACTTTCCTGCCCAGGATCCT (minimal length = 20), both direct and reverse complement, were deleted using cutadapt from the 3′ end of reads with the same options as in points 2 and 3 of this protocol.
6. All untrimmed reads separated at the first step of the procedure (i.e., the reads where no adapters were found) were trimmed again by the described procedure (points 1–5) with the following differences: at the first step, the changed set of cutadapt options was applied: -e 0.2 (this option raises the error rate to 0.2, thus enabling researchers to find adapters that were read with more errors) and -O 15 (instead of -O 10, thus requiring longer overlap of adapters with the reads).

The BBTools [43] 39.01 repair.sh tool was employed to re-pair direct and reverse reads with the “repair” option, while all singletons were excluded from further analysis.

The number of filtered (adapter-trimmed and length- and quality-filtered) and re-paired reads obtained was as follows: 9,120,357 (replicate 1, plastic substrate), 8,444,912 (replicate 2, plastic substrate), 9,462,207 (replicate 1, Matrigel substrate), and 8,795,250 (replicate 2, Matrigel substrate).

Paired-end filtered reads were aligned to the GRCh38/hg38 genome (https://ftp.ensembl.org/pub/release-112/fasta/homo_sapiens/dna/, accessed on 18 August 2025) using the bwa 0.7.17-r1188 [44] mem method. Post-alignment processing of BAM files was performed using SAMtools v1.17 [45], which filtered out unaligned reads and supplementary alignments (-F 2052) and sorted alignments by coordinate (samtools sort). Alignments entirely overlapping low-complexity genomic regions from the DFAM v3.8 database [46] were removed using BEDTools 2.31.0 [47] (intersect -wa -v -f 1.0), ensuring that only alignments that were aligned 100% into low-complexity regions were filtered out. deepTools2 3.5.5 [48] was employed to perform replicate quality control: alignment BAM files from the different replicates were RPKM-normalized (bamCoverage --effectiveGenomeSize 2913022398 --normalizeUsing RPKM --ignoreForNormalization chr21 chrY chrMT chrGL000220 --exactScaling --skipNAs) and then the plotCorrelation tool with options “--removeOutliers --skipZeros --log1p -p scatterplot” was applied to generate scatterplots and assess both Pearson and Spearman correlation coefficients. The assessed correlation coefficients (Pearson value of 0.99 and Spearman value of 0.94 for both datasets) indicated that replicates obtained for 4C-rDNA experiments, both for plastic- and Matrigel-grown Mel Z cells, showed consistency. The scatterplots presented in Figure S3, along with the assessed correlation coefficients, confirm the high consistency between replicates from plastic- and Matrigel-grown Mel Z cells, supporting the reliability of the results.

Aligned 4C-rDNA-associated reads were intersected across replicates using an in-house R script employing data.table 1.15.2 [49] and dplyr 1.1.4 [50] libraries, with only intersecting alignments, were retained for further analysis.

Gene quantification was performed using featureCounts [51] with the following parameters: -a hg38.112.gtf -t gene -g gene_id -O -p --countReadPairs --fraction --readExtension5 2500 --readExtension3 2500. This reflects the ±2.5 kb resolution of the 6-cutter EcoRI restriction enzyme used in the 4C-rDNA experiments.

Gene IDs were mapped to ISO gene names using an in-house R script for downstream Gene Ontology (GO) analysis. GO analysis of rDNA-contacting genes was performed using g:Profiler software (https://biit.cs.ut.ee/gprofiler/gost, accessed on 1 August 2025) using the “Highlight driver terms in GO” option [19].

### 4.4. Differential 4C Analysis

Differential 4C analysis was performed using the DESeq2 1.38.3 [52] R library with 4C replicates of plastic-grown cells as the “control” and 4C replicates of Matrigel-grown cells as the “experimental” group. The parameter fitType = “local” provided the minimal median of absolute approximation residues (0.055) across all possible fitType parameter values (“parametric”, “local”, and “mean”). A volcano plot was generated using the EnhancedVolcano 1.16.0 R library [53].

Since the direct comparison of datasets with varying read depths is not appropriate, we performed normalization using the trimmed mean of M-values (TMM) method [54] and implemented it using the edgeR 3.40.2 [55] R library. Through this method, gene counts are normalized across all datasets, allowing for a direct comparison of 4C contacts associated with genes between different experiments.

### 4.5. Violin Plots for 4C-rDNA Data

Violin plots were generated from TMM-normalized data via R scripts that utilized dplyr 1.1.4 [50] and ggplot2 3.5.0 [56] R libraries. The non-parametric independent two-group Mann–Whitney U-test was used to assess whether different subsets of 4C-rDNA-associated genes originated from the same distribution. The obtained *p*-values were adjusted for multiple comparisons using the most stringent Holm’s family-wise error rate (FWER) correction [57] via the standard pairwise.wilcox.test() function in R. The analysis confirmed that, in each pairwise comparison, the subsets did not originate from the same distribution (*p* ≪ 1 × 10^−6^), indicating their statistical independence.

Circular plots displaying the top 150 most statistically significantly differentially contacted genes, ranked by adjusted *p*-values, were generated based on the results of differential 4C analysis, using the plyranges 1.18.0 [58], Polychrome 1.5.1 [59], and circlize 0.4.16 [60] R libraries.

Mel Z 4C-rDNA-associated RPKM-normalized genome-wide profiles were created via deepTools2 [48] bamcoverage utility with the following options: --bs 10 --normalizeUsing RPKM --exactScaling --ignoreForNormalization chr21 chrY chrGL000220 chrMT --skipNAs. The intersection between genome-wide bedGraph profiles corresponding to different replicates was created using an in-house bash script utilizing BEDtools v.2.31.0 [47] and partition, bedmap, and map-id-uniq methods from bedops v.2.4.41 [61].

### 4.6. Violin Plots for Gene Expression Data

Normalized gene expression data for plastic-grown and Matrigel-grown cells were obtained from the GEO database under accession numbers GSE221876 and GSE221872, respectively. Processing of the raw data to TPM (transcripts per million) values was described in detail in [11]. Gene lists for each functional category were obtained using the g:Profiler functional enrichment analysis web server [19]. Violin plots were generated from TPM-normalized data using an in-house R script incorporating the dplyr 1.1.4 [50], ggplot2 3.5.0 [56], and tidyR 1.3.1 [62] R packages.

To assess whether gene expression datasets corresponding to different gene lists originated from the same distribution, the non-parametric Mann–Whitney U test was applied. The resulting *p*-values were adjusted for multiple comparisons using Holm’s FWER correction [57] as implemented in the standard pairwise.wilcox.test() function in R.

### 4.7. Code Accessibility

All scripts and appropriate data files were deposited in the public GitHub repository (https://github.com/lokapal/MelZ.4C.2025, accessed on 18 August 2025).

## Data Availability

4C-rDNA data were deposited in the Gene Expression Omnibus (GEO) repository under accession number GSE295545. RNA-Seq data were deposited in the GEO repository under accession numbers GSE221876 and GSE221872.

## Supplementary Materials

The following supporting information can be downloaded at https://www.mdpi.com/article/10.3390/ijms26168085/s1.
