# Supplementary material for: Formation of the Vasculogenic Mimicry Phenotype in Melanoma Mel Z Cells Is Coupled with Changes in Inter-Chromosomal Contacts of Developmental Genes with rDNA Clusters
Source: Int J Mol Sci. 2025 Aug 21;26(16):8085. doi: 10.3390/ijms26168085 (PMC12386866; doi:10.3390/ijms26168085)
Supplement: Supplementary file 1 [file ijms-26-08085-s001.zip › Supplemental Information 1-8--2025.pdf]

-Supplemental Information for:

Formation of the vasculogenic mimicry phenotype in melanoma cells is coupled with changes in inter-chromosomal contacts of developmental genes with rDNA clusters

Nickolai A. Tchurikov, [1*] Elena S. Klushevskaya [1], Viktoriya N. Lukicheva [1], Antonina N. Kretova [1], Elizaveta N. Poperekova [1], Vladimir R. Chechetkin [1], Galina I. Kravatskaya [1], Amalia A. Vartanian [2], Vyacheslav S. Kosorukov [2] . Ildar R. Alembekov [1], and Yuri V. Kravatsky [1]

[1] Department of Epigenetic Mechanisms of Gene Expression Regulation, Engelhardt Institute of Molecular Biology Russian Academy of Sciences, Moscow, 119334, Russia
[2] Department of Experimental Diagnosis and Therapy of Tumors, N.N. Blokhin National Medical Research Center of Oncology of the Ministry of Health of Russia, Moscow, 115478, Russia

This PDF file includes:

Figures S1–S3

Tables S1–S17

Figures S1-3

Drivers

2118 genes

ID	Source	Term ID	Term Name	p_{adj} (query_1)
1	GO:MF	GO:0005515	protein binding	8.552×10^{-13}
2	GO:MF	GO:0016301	kinase activity	1.740×10^{-3}
3	GO:MF	GO:0022835	transmitter-gated channel activity	3.320×10^{-3}
4	GO:MF	GO:0003779	actin binding	2.178×10^{-2}
5	GO:BP	GO:0048731	system development	8.565×10^{-10}
6	GO:BP	GO:0051179	localization	2.647×10^{-6}
7	GO:BP	GO:0048013	ephrin receptor signaling pathway	7.429×10^{-4}
8	GO:BP	GO:0007167	enzyme-linked receptor protein signaling pathway	8.773×10^{-4}
9	GO:BP	GO:0007155	cell adhesion	1.277×10^{-3}
10	GO:BP	GO:0030029	actin filament-based process	3.518×10^{-3}
11	GO:BP	GO:0003012	muscle system process	9.311×10^{-3}
12	GO:BP	GO:0080090	regulation of primary metabolic process	3.323×10^{-2}
13	GO:BP	GO:0032535	regulation of cellular component size	3.354×10^{-2}
14	GO:BP	GO:0060078	regulation of postsynaptic membrane potential	3.419×10^{-2}
15	GO:CC	GO:0005737	cytoplasm	6.119×10^{-13}
16	GO:CC	GO:0043235	receptor complex	1.660×10^{-2}

659 genes

ID	Source	Term ID	Term Name	p_{adj} (query_1)
1	GO:MF	GO:0005509	calcium ion binding	1.900×10^{-14}
2	GO:MF	GO:0050839	cell adhesion molecule binding	6.295×10^{-6}
3	GO:MF	GO:0005001	transmembrane receptor protein tyrosine phospha...	1.567×10^{-5}
4	GO:MF	GO:0099106	ion channel regulator activity	1.229×10^{-3}
5	GO:MF	GO:0030695	GTPase regulator activity	3.928×10^{-3}
6	GO:MF	GO:0030554	adenyl nucleotide binding	4.721×10^{-3}
7	GO:BP	GO:0007399	nervous system development	2.722×10^{-30}
8	GO:BP	GO:0007156	homophilic cell adhesion via plasma membrane a...	5.518×10^{-25}
9	GO:BP	GO:0030029	actin filament-based process	1.564×10^{-4}
10	GO:BP	GO:0097120	receptor localization to synapse	1.192×10^{-3}
11	GO:BP	GO:0007010	cytoskeleton organization	1.499×10^{-3}
12	GO:BP	GO:0006941	striated muscle contraction	6.033×10^{-3}
13	GO:BP	GO:0035418	protein localization to synapse	8.170×10^{-3}
14	GO:BP	GO:0036211	protein modification process	1.022×10^{-2}
15	GO:BP	GO:1903779	regulation of cardiac conduction	1.413×10^{-2}
16	GO:BP	GO:0010842	retina layer formation	1.807×10^{-2}
17	GO:BP	GO:0086001	cardiac muscle cell action potential	2.240×10^{-2}
18	GO:BP	GO:0007612	learning	2.584×10^{-2}
19	GO:BP	GO:0040013	negative regulation of locomotion	2.716×10^{-2}
20	GO:BP	GO:0070588	calcium ion transmembrane transport	2.716×10^{-2}
21	GO:BP	GO:0010880	regulation of release of sequestered calcium ion i...	3.547×10^{-2}
22	GO:BP	GO:0098660	inorganic ion transmembrane transport	4.125×10^{-2}
23	GO:BP	GO:0001964	startle response	4.361×10^{-2}
24	GO:BP	GO:0070085	glycosylation	4.540×10^{-2}
25	GO:BP	GO:0050890	cognition	4.836×10^{-2}
26	GO:BP	GO:0030001	metal ion transport	4.913×10^{-2}
27	GO:CC	GO:0030054	cell junction	3.857×10^{-24}
28	GO:CC	GO:0005856	cytoskeleton	1.480×10^{-5}
29	GO:CC	GO:0016010	dystrophin-associated glycoprotein complex	6.084×10^{-3}
30	GO:CC	GO:0034703	cation channel complex	1.150×10^{-2}
31	GO:CC	GO:0012505	endomembrane system	4.477×10^{-2}

1797 genes

ID	Source	Term ID	Term Name	p_{adj} (query_1)
1	GO:MF	GO:0005515	protein binding	1.572×10^{-7}
2	GO:MF	GO:0005085	guanyl-nucleotide exchange factor activity	3.941×10^{-5}
3	GO:MF	GO:0005547	phosphatidylinositol-3,4,5-trisphosphate binding	7.754×10^{-3}
4	GO:MF	GO:0035091	phosphatidylinositol binding	4.283×10^{-2}
5	GO:BP	GO:0000902	cell morphogenesis	6.313×10^{-7}
6	GO:BP	GO:0051056	regulation of small GTPase mediated signal transd...	3.559×10^{-4}
7	GO:BP	GO:0065007	biological regulation	9.671×10^{-4}
8	GO:BP	GO:0034330	cell junction organization	1.117×10^{-2}
9	GO:BP	GO:0051179	localization	2.732×10^{-2}
10	GO:BP	GO:0043491	phosphatidylinositol 3-kinase/protein kinase B sig...	3.802×10^{-2}
11	GO:BP	GO:0007010	cytoskeleton organization	4.038×10^{-2}
12	GO:BP	GO:0006810	transport	4.938×10^{-2}
13	GO:CC	GO:0005737	cytoplasm	3.613×10^{-19}
14	GO:CC	GO:0005856	cytoskeleton	1.369×10^{-8}
15	GO:CC	GO:0098588	bounding membrane of organelle	1.789×10^{-2}
16	GO:CC	GO:0032421	stereocilium bundle	3.811×10^{-2}

Figure S1. Gene Ontology driver terms for the three groups of rDNA-contacting genes shown in Figure 2. Group of 2118 genes is only characteristic of rDNA-contacting genes detected in Mel Z cells grown on plastic. Group of 659 genes was detected in the cells cultivated on both plastic and Matrigel. Group of 1797 genes was found only in the cells cultivated on Matrigel. The search was performed using Gene Ontology driver terms (https://biit.cs.ut.ee/gprofiler).

Drivers - 398

ID	Source	Term ID	Term Name	p_{adj} (query_1)
1	GO:MF	GO:0005509	calcium ion binding	3.220×10^{-11}
2	GO:MF	GO:0050839	cell adhesion molecule binding	7.925×10^{-5}
3	GO:MF	GO:0008569	minus-end-directed microtubule motor activity	2.208×10^{-2}
4	GO:MF	GO:0099106	ion channel regulator activity	3.782×10^{-2}
5	GO:MF	GO:0003774	cytoskeletal motor activity	4.442×10^{-2}
6	GO:BP	GO:0007399	nervous system development	2.018×10^{-24}
7	GO:BP	GO:0007156	homophilic cell adhesion via plasma membrane a...	1.601×10^{-23}
8	GO:BP	GO:0097120	receptor localization to synapse	1.581×10^{-4}
9	GO:BP	GO:0099536	synaptic signaling	3.869×10^{-4}
10	GO:BP	GO:0001964	startle response	2.380×10^{-3}
11	GO:BP	GO:1903779	regulation of cardiac conduction	1.925×10^{-2}
12	GO:BP	GO:0006942	regulation of striated muscle contraction	3.371×10^{-2}
13	GO:BP	GO:0007043	cell-cell junction assembly	4.002×10^{-2}
14	GO:CC	GO:0030054	cell junction	9.536×10^{-15}
15	GO:CC	GO:0043005	neuron projection	1.264×10^{-11}

Figure S2. Gene Ontology driver terms for rDNA-contacting genes that form stable contacts with nucleoli. This group of 398 genes was detected in the cells cultivated on both plastic and Matrigel. The search was performed using Gene Ontology driver terms (https://biit.cs.ut.ee/gprofiler).

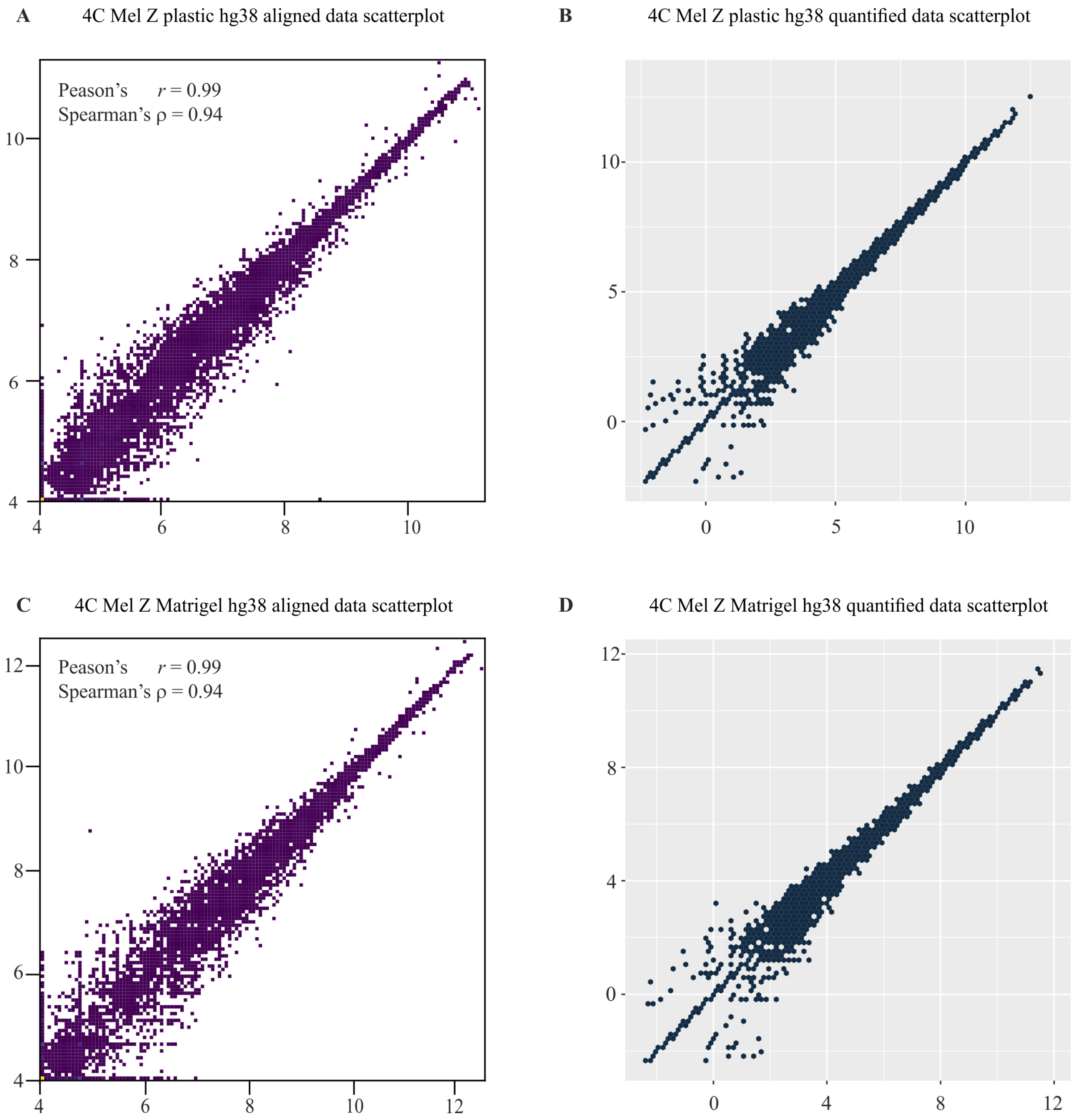

Figure S3. Scatterplots of 4C-rDNA data aligned to the hg38 genome (panels A and C) and quantified using the hg38 Ensembl v106 annotation (panels B and D). The x-axis represents replicate 1, and the y-axis represents replicate 2.

Tables S1–S17

Table S1. List of rDNA-contacting genes in Mel Z cells grown either on plastic or on Matrigel. 4C-rDNA reads were processed as described in the Materials and Methods section. The Excel file is attached separately. Related to Figure 2.

Table S2. Venn diagram showing the intersections between rDNA-contacting genes detected in Mel Z cells grown on plastic (2777 genes) or on Matrigel (2456 genes). The genes exhibiting ≥ 30 contacts were selected. Related to Figure 2A.

Names	total	elements
Plastic Matrigel	659	*AC092634.2 PAX7 LINC01201 RALYL FP236241.1 ABCC6 PTPRR DLEU7 EPB41L4B ENSG00000286540 AC012409.2 LRMDA EPN2 PIR MED13L NFIA SUGCT DIP2A PRH1-PRR4 LINC03007 TRPM6 SLC8A1 RNF103-CHMP3 AL356108.1 ANO5 PCDHA13 ZNF385B LOC102724843 PREP AF127577.4 RSRC1 GRIK2 DPYD OTX2-AS1 SLC22A14 LINC03000 KDM4B FGF1 AC090809.1 ZBTB20 ARHGAP15 LOC101929710 LINC00486 AHR LOC107984685 AC022523.1 FSTL5 TTC3 CADPS2 TLK1 ASTN2 FAM9B LRP1B KALRN MKLN1 AC009262.1 SUMF1 USH2A NEGR1 XXYLT1 FGF12 BTBD9 PARP4 FRG1-DT PCDHA12 AC069228.1 MAST4 NRXN1 AC079801.1 AGBL1 MAGI1 GNGT1 AC036214.3 FARS2 ST6GALNAC3 DPP6 AC098588.1 FAM13A RNF217-AS1 DAAM2 STK24 AL365295.1 LMO7 GRM3 U8 EXOC4 LINC02822 AC078828.1 DNAH7 BBS9 ENSG00000290711 NTRK2 ANXA10 ANKRD34C-AS1 CHD7 ARL17B NBEA AL512598.2 RASGEF1B TMX3 LINC00273 MIR99AHG ENSG00000293415 NRG3 PTPRG CALCR ELMO1 ENSG00000287881 NCOA1 IRAG1 LINC01684 AL390957.1 ATP2B4 ROCR TOX2 PHEX SOX6 LINC03104 PDE4DIP TRPM3 FBLN7 TMEM64 DNAH8 CNTNAP5 PRANCR RAPGEF5 FGF13 DRAIC PATJ LINC01798 GPC3 DMD AL512380.2 ADCY2 DYNLRB2-AS1 KCNQ3 LINC02566 CDH19 LINC02966 ADAMTS9-AS2 GNG12-AS1 ATRNL1 CHRM3 ARHGAP24 LINC00907 SLIT2 TPTE2P6 ZNF804A FAM20C ENSG00000291181 AC010235.1 TTC33 LINC00347 AC098588.2 NALF1 KAZN DNAAF9 BRAF BABAM2 ENSG00000289084 KMT2C FKBP9P1 CU638689.2 LINC01320 STEAP2-AS1 ASMER1 B3GNT5 ERBB4 KIF26B GPHN ENSG00000293389 MEF2C-AS1 ZNF385D ATXN7L1 RAD51B DOP1B SH2D1A MYO3B ENSG00000287722 SH3RF3 EXT2 DIAPH2 MAP3K5 AL138720.1 STAG2 SPAG16 DSCAML1 HTR2C GALNT13 LOC101927609 TBC1D5 CNTN4 RAP1GAP2 LINC01830 LOC105369165 DMBT1L1 AC013652.1 CCSER1 PIK3C3 HMCN1 ENSG00000286033 LINC01695 LOC105373436 ITGA1 TCF12 SGIP1 ARMC8 ENSG00000289694 RUNX1 MYO16 ESRRG AC058822.1 AL161757.2 PCDHA2 FRG1HP PCDHA11 PCLO PRH1 ENSG00000290357 SLC38A4-AS1 CDH18 FRMD6 ANK2 LOC101927314 MIR100HG ANO2 PCDHA9 SUPT3H ROBO1 LINC01091 ENSG00000289002 NEK1 LINC01924 DGKB PWRN1 CDC42 EBF1 OBI1-AS1 PRRC2C PCDHAC2 CDH6 AC109830.1 ENSG00000290397 DOK6 WDR64 C8orf34 JAZF1 BNIP3P41 CHODL ZNF267 LINC01692 CPED1 FGF14 MIR325HG CACNA2D3 LINC03116 SLC8A1-AS1 PCDHA10 MUC19 EDIL3 LRRTM4 PTPRN2 INPP4B SOX5 DSCAM DGKI LINC01151 COL28A1 DENND1B COLEC10 LARGE1 SDK1 PUS7 CCDC33 LINC02694 EPHA6 LINC02328 IGF1R WDR72 SPTB NLGN1 TMEM260 IQSEC1 ENSG00000287916 ANK1 PPM1L CASZ1 CNTNAP2 TRIM71 ENSG00000286523 KCNIP4 SOX2-OT CFTR DYNC1I1 NOS1AP OTOGL DOP1A AC027031.1 PCDH9 NKAIN2 PKN2-AS1 RBMS3 OFCC1 AL603840.1 FP700111.1 SGO1-AS1 CFAP20DC CIBAR1-DT ATP6V0D2 PCDHA1 ZNF800 Y_RNA PCDHA8 CU638689.4 ENSG00000290551 TBCK AC108010.1 CDH12 SHC4 LINC02112 AC098650.1 FRMD6-AS2 TESHL CBR3-AS1 RPS3AP27 DDR2 ADAM22 LARS2 HULC GPC6 MCF2L2 TRPS1 ENSG00000293394 CDH4 ENSG00000288187 DPP10 CELF4 ST8SIA6 CNTN6 PARM1 AC005670.2 CU633906.2 FBLN5 SPEF2 ENSG00000286745 WDSUB1 ZNF638 ATP9B MITF CACNA2D1 NSMCE2 PCSK1 MIR4435-2HG PCDHAC1 HERC2 LRFN5 UTRN GPC5 ENSG00000290523 AGBL4 OGDH GHR ULK4 RBFOX1 PCDH7 LRRC4C AC084149.1 PPP1R9A TMEM117 AP001341.1 RNA5-8SN5 SMAD9 NKAIN3 DYNC1H1 CRPPA NEBL AC006148.1 NAALADL2 AC007161.3 OSBPL10 LANCL2 ZFPM2 MRPS28 SLC35F1 TRD-AS1 LINC02306 FRMPD4 MEIS1 GALNT17 EDAR PMS2P4 CFAP20DC-AS1 BRINP3 RIMBP2 FHIT AC009093.10 WIPI2 DNMT3A TOX PCDH15 SGCZ ACE2 CHMP3 KCNAB1 PDE4D CNTN5 AL121718.1 ITGB8 PDE3A RIMS1 TRANK1 WWOX EGFEM1P PCDHA7 NEK10 BEND5 ANAPC10 LSAMP MIPEP LINC01681 PWRN4 AC007100.1 DIAPH3 LINC01440 TMEM178B CPLANE1 NOL4 PCDHA5 PSG11-AS1 PLCB4 RNF216 FTO UGGT2 TNFSF11 LINC02326 PPFIA2 ENTPD1-AS1 ENOX1 WNT2 ENSG00000292991 OR8B8 STEAP1B UMAD1 LINC00299 POU6F2 SFI1 MGAT4C DIRC3 ANKRD44 CALN1 UXS1 LINC01584 NTNG1 ZNF804B SGCD GRM7 AC007848.1 STS NAV3*

		AC091489.1 AC092343.1 AL110292.1 AC079943.2 LOC124903324 PTPRM ENSG00000286225 PRKCH NRXN3 DLC1 DENND2B LINC01500 MIR663AHG ATP8A2 CU633906.4 CERS6 AL591463.1 LPIN1 PLEKHA5 MACROD2 AP005328.1 ENSG00000287635 ADAM7-AS1 AUTS2 AC092042.3 LOC102724701 SYT16 BCAS3 ADARB2 ENSG00000288723 COMT PCDHA6 MCTP1 ENSG00000286110 NBAS ZPLD1 KMT2E LINC03060 SYNE2 DOCK2 SDCCAG8 SSH2 AL035078.4 LMNTD1 CDH13 MDGA2 AC103796.1 BDNF-AS 5_8S_rRNA DANT2 LINC01934 FBN2 DAB1 TTLL6 RFTN1 PCDHA4 SNTG1 ALK CACHD1 SEMA3A AC068051.1 YEATS2 CCDC26 SLC8A3 ENSG00000290983 MYO5B CADM2 MALRD1 TENT4B MAD2L1-DT AC010601.1 DCLK1 AC004917.1 FAM135B KIF16B CDH2 TENM2 VPS41 LINC00879 TEX13D HDAC9 KIAA1549L USP9X KCNB2 USP13 NBPF1 CDK6 ADGRL4 AC090376.1 LYST CADPS DDX60 LINC03105 RGS7 ENSG00000293037 LINC01470 SRGAP2 TFAP2D AC067956.1 PPP2R2C HEATR4 SPECC1 PTPRT GABPB1 CSMD1 ENSG00000286746 SCEL CTNNA3 FREM2 RAPGEF4 CLTC THSD7B ENSG00000287404 DACH2 AC093515.1 SORCS1 CAMKMT RNF220 ARHGEF37 DLG2 SACS AFF2 PTPRD RORA AL355499.2 PLCB1 NPEPPS PRKG1 BRIP1 MIR924HG LOC107986400 MAGI2 NELL1 MIR4300HG CYP4F62P PLCL1 LINC02215 GALNTL6 PXDNL SCN2A ASIC2 DOCK10 MICAL3 ADK TMEM164 KMT2CP4 RANBP17 GTDC1 RYR2 SLX4IP LDLRAD3 ENSG00000286648 CU633904.3 PTPRA AC011287.1 FAT3 MSH2 PCDHA3 MGAT4A AC078845.1 KLHDC10 RGS9 AF241726.2 MPDZ AC016766.1 EYS SLF1 ROBO2 CR392039.3 ENSG00000288891 LINC02055 SLC39A11 LINC00378 AC026167.1 AC015687.1 PAX3 LOC105379109 KSR2 DLGAP2 BMPR1B PDE8B DCC CTNNA2 PEX5L ARL17A DNER ETV6 CEP112 NRG1 PGM3 DLEU1 SLC25A48 ENSG00000293385 AC092167.1 NPAS3 DNAH5 SATB1-AS1 FANCB LINC02208 NRXN1-DT
Plastic	2118	AL157886.1 FSTL1 CDH12P2 CASC20 C10orf90 APBB2 AC243829.5 PFKP MIR3171HG AC012501.1 ESYT1 STYK1 RPL19 RTN1 HS3ST3A1 RNU7-188P AC100775.1 RGN TRGC2 CDK14 ZSWIM1 MSRB3 PDE1C ENSG00000293331 MREG HNRNPCL4 NOSTRIN ING3 ENSG00000290149 NINL MAP3K3 WSCD1 ENSG00000287515 OSBPL8 SLC12A8 LRRN1 HERC2P2 ENSG00000286309 ENSG00000291144 LINC00698 POTED STX8 AC032019.1 PHF20 FOXK2 CPNE4 TXNDC8 RBM17 AC021517.1 SMYD3 ARHGEF26-AS1 GARS1 RAPGEF6 ARHGEF9 TAFA2 ABR KRT8P32 TLR8-AS1 ISM1 MAOA BLTP3B LINC01163 AC012355.1 LINC02438 HYDIN2 ENSG00000287308 HCCAT5 TRAPPC9 PCDHB1-AS1 SLC16A5 LINC01545 FNDC1 TMEM255A MAP3K13 EIF1AX PPM1A ARL13B RPL15P21 SAMSN1 RN7SL556P C12orf75 AC010291.1 THADA LIMD1 IMMP1L TF SNX10-AS1 CHST11 THRB MYBPC1 USP42 IPP RCSD1 SPTBN1 UBTFL3 AF228730.2 CDC42EP3 ENSG00000286111 AC013472.2 AC021269.2 MIR3117 MYO5C LINC00841 MAPK10 ITPRID1 CACNB1 USP32 AHCYL2 DAPP1 CD101-AS1 ATF7 GNG12 CHRNB2 CDC14C SEZ6 CLDN6 LINC01967 CDH8 KCNQ5 GNB4 AGTPBP1 AL356490.1 ATF7-NPFF FHL1 TRMT6 PPP1R37 RBPMS ATP10A LINC02457 SH3PXD2A SPINK5 MRPS24 OPA3 SKAP2 PRND PIGL ATP7B PPM1H GPBP1L1 RPS6P19 KCNJ6 PCAT1 RPL21P82 BRD4 ZAN DOK4 AC007405.2 LINC00517 AC092378.1 TNRC18 MIR9-3HG LOC102724019 LINC02828 CAMK2B GRIP1 CASC19 ELMO1-AS1 ZNF962P OR4N3BP ARHGAP11A-SCG5 LOC124901321 URGCP-MRPS24 DDI2 AC008764.4 GPATCH8 PGM1 DCT AC073071.1 DLGAP1 FAM193A LNCATV PECR LRP5L SLC38A9 LINC01982 CDRT15P9 LINC00348 ZNF264 QRSL1P2 LINC02133 HBP1 SDR16C5 RGPD2 ABCG8 XPO4 OTUD7A AC060234.3 AL583785.1 AC107909.2 OPN3 MAP4K3 AC092078.2 FAM157A LINC01194 GALNT9 FPGT-TNNI3K SNORD114-10 LOC442028 NOTCH2 CD93 LOC105379362 FKBP5 TMOD2 SLC44A5 SDK2 HMBOX1 SIM1-AS1 AP003900.1 ZNF551 LINC00882 RNU2-49P NPNT AC019211.1 DSC1 EPB41L3 MAP6 AC012363.2 MORC1 PARVB CDH11 RN7SL266P SHANK3 WDR26 NR4A3 CYP2U1-AS1 SPTLC3 GAS1RR AGAP12P LINC00494 AC005154.4 ARID1B LOC101927948 LOC105375387 DPY19L2P2 ENSG00000289752 AMYP1 E2F7 ACVR2B-AS1 L3MBTL4-AS1 SLC14A2 ENSG00000291166 AC091304.2 FAM169BP FOXO1 LINC02311 ZNF611 STYXL1 AC244517.9 LINC02156 STK39 PDE11A-AS1 SMURF2 SRL ADAMTS12 FER1L6 TBC1D16 PLXNA4 AL592078.1 LINC02542 LINC00504 LOC124900810 CDK18 URB2 YBX1 ARMT1 DIPK2A CFAP70 DCDC1 CACNA1C

		FAM20B ENSG00000287410 AC046195.1 FAM3C SNORA5B ASB4 IDO1 AC026786.1 AC015922.1 ARB2A LOC105370489 PRKN ENSG00000286937 CNN3-DT NLGN4X RMND5A LINC02044 HSD17B2-AS1 PRDX3P3 PNPLA3 LINC00320 AC092447.10 F13A1 ENSG00000288041 TAFA1 PIK3C2B XPO6 AC103876.1 KDM7A SLC44A3 SLC5A7 GNA14-AS1 MARCHF9 FAM83B IFT172 AL136317.2 RNU6-258P GPSM1 AL121900.2 CBLL2 AL136372.2 PTBP3 ZBTB44-DT FAM117A CDON SERPINI1 SLC17A6 NLK SDF4 THSD7A OVCH1-AS1 GNAI1 LINC02405 SPATA31D2P MRPL13 AC138932.2 MAPKAPK3 NCAM2 CHML PAPOLG H3P38 TIAM2 INTS6-AS1 CFAP54 ZNF10 RORA-AS1 AC015804.1 TRAK2 AL139383.1 ENSG00000293038 CNMD ENSG00000286229 HERC2P3 LRP8 TAGLN3 AC018767.3 LINC00992 CNTN1 AC002451.2 AGL MEOX2 MAPRE3-AS1 STAT5B ENPEP LINC01242 NUDT19 ARHGEF4 LOC102724428 UBE2O MUC17 DDIAS AC025884.1 MTUS1-DT DUXAP9 AC092100.1 CPEB4 EIF4ENIF1 HEATR9 LOC124900848 CHRFAM7A ERBIN RN7SL430P CR383658.2 ZNF121 HERC2P9 MBTPS2 RGS22 PLS3-AS1 LINC00598 CD247 ENSG00000290948 ENSG00000287621 CEP192 MCC TRIM52-AS1 PTH GEMIN8P1 ENSG00000287008 ADAR DNAH10 PDE10A TM9SF2 ENSG00000286250 PDHX CLCN3 RHOJ CDC37 FGF10 ZFYVE16 JAZF1-AS1 MECOM AC009878.1 RSPH10B COL4A6 CATSPERB ENSG00000287474 SAMD15 TSPAN18 NLRP8 FRG1JP ENSG00000286800 STK32B PLG NEURL4 POLR2J3 HERC5 LINC02505 LOC124900957 AC008109.1 NUDT13 CDH7 LOC101929457 ENSG00000290585 PGPEP1L UBE2E2 SCEL-AS1 ILDR2 AC034229.1 MGC27382 NCK1 PUDP CD84 ENSG00000287744 FAM168A NONO RARRES1 AC138512.1 KLHL42 NREP AF064860.1 EXO1 GABRA5 CCDC71L AL591684.2 ARHGAP23P1 EBNA1BP2 SNORD114-9 NEXMIF ENSG00000286274 LOC401913 ENSG00000288755 MTCP1 ASTN1 SH3BGR TRAJ17 MROH6 AC104248.1 AC004492.1 AC231532.1 KDM2B HEY2-AS1 PREX1 RGPD3 PRIM2BP AL133500.1 AIG1 ERICH1 IGLV1-41 ZNF404 PMS1 CDIN1 WRNIP1 ABCC13 COL24A1 ANO3 TYW1 GOLGA8R AC009511.1 COL11A1 AC119677.1 AC112721.2 AC113391.2 RSF1 AC022509.1 AC063949.2 KCTD3 NCOA7 C2orf88 RNU2-33P SERTM2 SEPTIN10 CIDEB MARCHF1 OR4N3P ENSG00000286376 SLC45A1 ENSG00000286922 MCPH1 AL773545.1 ENSG00000289143 ENSG00000287045 MYO1D SESTD1 ENSG00000291338 LINC01661 ENSG00000288553 ZNF214 COL1A2 DTNA ENSG00000286020 TMOD3 LOC340512 GEMIN5 EEF2KMT MRTFB ENSG00000286147 XPO7 KCNS3 AC011447.3 ENSG00000288902 VWA3B RPL34P11 FREM2-AS1 ENSG00000287608 ENSG00000290114 COL12A1 ABLIM1 ENSG00000287763 AC009226.1 RBMS1 ALPK2 CHRNA7 HECW1 HMGB1 ZNF304 DYNLT2B AC021733.1 CDIP1 AFF3 RFLNA AL158198.1 AC062039.1 LPP HIVEP1 ATRX TNIK STK24-AS1 UBTFL5 LOC105371956 TGFB2 MDFIC EPB41L4A LAMA2 ADD3 TRIO FBXO16 ZNF85 LINC00269 ENSG00000291054 LINC00366 HOXC13 AL049875.1 SMARCC1 NDST1 LINC00355 MUSK KRT223P ZNF73P KCNJ18 JAM3 ENSG00000287741 KCNIP3 SFPQ AC027338.2 PNPLA4 AC004485.1 ALKBH1 SNORA5A ARPP21 AF130359.1 RGS3 ZNF299P DUXAP8 NUP93 LDHAL6DP AC108673.3 ZCCHC4 MRPS9-AS2 ZNF407-AS1 RERG G2E3-AS1 ESRRAP2 OR5AU1 PRKAR2A FILNC1 RBM33 AL604028.1 CTIF CFAP57 ENSG00000286902 FADS2B CALCRL-AS1 ATP11A AC023389.1 MRPL33 RBM23 AC008695.1 ZNF154 ATP4A CSTF3 SLC15A4 HIGD1AP9 ENSG00000288087 AL024474.2 AC244517.11 CERS6-AS1 CDH12P4 PCDHB8 GOLPH3 AC015908.7 PRKG2 MBTPS1 NCF2 PIGV RNU6-1216P USP10 SND1 EPIC1 ENSG00000288692 RN7SKP141 IGFBP7 RSKR EPHA5 MACROD2-IT1 RABGAP1L-DT LINC03076 PTPN20 ENSG00000290849 AC087501.3 LRRK2-DT SKOR2 AC130650.1 CBR4 CRLF3P2 ATP13A4 AL162718.1 AC009055.2 ENAH SNX10 ATP2C1 TMEM230 ALDH1A2 AL354718.2 PPTC7 STON2 MIR3681HG PTGFR IGHV1OR21-1 AL138895.2 FAM171A1 STRADA LINC01725 HUWE1 ZNF595 ASPH RRAS2 SH3BGRL ZDHHC4 UBR5 AP000894.1 AC114781.3 AC096558.1 GTF2F2 AC105031.2 SIM1 KIAA1217 ABTB2 SOX30 AC244131.2 TMEM132C ANKS1B CHL1 TRAJ16 SPOCK3 GSN PLS1 AC007991.3 TCP11L1 SPON1 MAPRE3 MICB-DT TLR8 HPS3 AL391361.1 AC011447.2 KIF21A LOC124900600 SLIT3 ARMH3 FMO8P CACUL1 NLRP7 ARHGEF39 CD226 PHC2 MITA1 AC142086.6 ZNF578 ENSG00000290721

		ENSG00000287021 ANKRD11 ITPR1 AL162254.1 PKP4 AL160286.3 CPM AC105052.4 DNAAF11 ENSG00000288069 PAK3 LINC01033 NDUFAF7 AC007846.2 ABI3BP RNU1-83P KLF12 LOC124900504 AXDND1 AC011824.3 IPMK LINC03042 AC073575.2 LINC01376 CYP2A13 RPL23AP51 TMEM183A RERG-AS1 AC126763.1 ENSG00000289956 CAMK1G PRUNE2 CASC2 SERPINB9P1 LINC01122 RNA5SP96 ENSG00000288694 AC073130.2 PRKAG2 LINC02965 ENSG00000290490 LINC00158 MLLT3 CCNT2 BTG4 HAVCR1 DRG1 RSPH10B2 TRAPPC10 LINC02942 ENSG00000288714 AC034206.1 CPQ CNOT10 CPEB1-AS1 MR1 KRTAP21-2 LOC102724710 F8 TNFRSF12A OASL GDPD1 LINC00237 CPT2 AP000320.1 TAS2R14 VPS8 AC010329.5 MTX3 LINC00703 AC092121.1 ENSG00000286878 C4orf50 NXT1-AS1 LINC01732 AC020743.2 ISY1 ENSG00000287211 ZNF831 ZNF429 AC242426.2 VN1R31P CXCR5 NCKAP1 PRPF40A CLDN12 KRTAP13-6P DTD1 CNTN3 MGAM ENSG00000289085 LINC02427 LINC01060 CILK1 HOXC13-AS FOSL2 PACRG IL1RAPL2 ADGRD1 HNRNPCL3 EPHA3 AC020912.1 CHCHD3 ZNF337-AS1 ENSG00000289131 LATS2 LINC02141 TYW3 MYH14 AC008581.2 APCDD1L-DT SPDYE2B TPRG1 MRPL32 ENSG00000286168 KLHL33 ERP29 CDYL2 ARHGAP18 AC013652.2 UVRAG CACNB4 MYOM2 SMIM10L2B-AS1 AC113386.1 LYN USP6 ADGRB3 LOC100131779 KLF7 MIR4636 LINC00390 SUMO2 LOC102723883 GRIK1 MTA3 CTSLP4 HSD11B1-AS1 LINC02203 UBL3 DIP2B LOC107985126 NME7 SEC24C AC068234.1 AC073869.5 MIR9-1HG AC109583.3 PRAMEF9 ENSG00000286686 ATP6V1H AC009135.1 NUP58 FAM177B FIGNL1 SLC12A1 LINC01538 AC068547.1 PDZD8 PAMR1 AC003006.1 CA10 LINC01878 RRM2 CRYBB1 SIL1 LDB2 AC008035.1 MYRFL AC024610.2 UNC93A C21orf62 MIR3159 LOC84214 ALDH1A1 NMNAT3 FMO5 RNASEL AC010082.1 NAV1 LINC02008 CMKLR2-AS PAQR5 DBF4B DSCAS CORIN TMCC1 LRRC7 PCDHB16 TEX41 LINC00458 AC060765.1 AC027288.1 DCP2 CDH12P1 RBPMSLP PDZRN4 SGMS2 FAM151B-DT TMED7-TICAM2 STX17-DT ENSG00000288055 SLC41A2 FER1L6-AS2 NOS3 AL355306.2 LIPE-AS1 LOC105378402 HMGB3P30 VSX1 ZNF280B EIF3L NOMO2 NALCN-AS1 TNRC6C ZNF90P3 PRAMEF27 LINC01208 PGAM1P5 MLLT10 BPHL ERCC6 EIF2AK4 LINC00840 RN7SL738P IGKV2OR2-2 KIAA1614 NIHCOLE AC016027.4 MSRA AL031599.1 LOC100419786 SYNPR RAB12 SIMC1P1 LINC02235 LINC01505 C21orf62-AS1 IMPG1 HMGCLL1 LINC01911 DSCR4 XKR6 LOC101928335 ENSG00000287682 MAGEL2 AF165147.1 MATN2 TGFBR2 AC141257.2 ITGA11 CMKLR2 CFAP77 MARK4 DDX60L MDN1 AC092131.1 LINC01901 SYNJ1 TTC6 TCF7L2 LOC102724421 LINC02192 AC114781.2 CHAF1B ENSG00000290067 KIAA1328 ENSG00000286326 ZNF462 MTRF1 ECHDC2 AC090386.2 OVCH1 CRTAC1 E2F6 ZFHX3 ENSG00000291336 POLR2C CUX1 UNC119 SHISA2 GPM6B RRAGD ULK3 C2orf50 AC083902.1 LINC02484 LOC339166 SPDYE2 GPR156 ZMAT4 CNOT10-AS1 ENSG00000289949 AP000529.1 AL445224.1 TUBBP3 LINC01596 GKAP1 CACNA2D4 LHFPL3 RGPD1 PPP2R5E AL161751.1 ENSG00000288954 RPL39P33 KPNB1 INHBA-AS1 ENSG00000290385 AC010332.2 TTN-AS1 GSDMD ADGRV1 ST7 RASGRF2 SERBP1 DNM1P33 AC013401.1 QTRT2 EVA1C AL161716.1 EPB41L5 GAD1 SCG5 AL035078.2 NUDCD1 AC068254.1 MIR603 AL050327.1 WAC WHRN OCA2 AC093668.1 DAPK1 TDP2 ULK4P2 ALDH9A1 LOC100419045 RAB6C-AS1 LINC01362 ZNF678 ACSM2B PRUNE1 TUBB8P6 SAR1B TTC7B INSYN2A BTG3 APP GATA4 ATF7IP2 SMAD5 IGF2BP1 PPIL6 AC116634.1 MAMLD1 LINC02610 RPL9P14 RNLS AC009093.4 ENSG00000291100 ANKRD20A7P AL163932.1 GSKIP AL136985.3 NEPRO-AS1 RNF38 ADAM12 LOC107986837 UBE2G1 NBPF12 AC138969.2 PELI2 MIR1273F BTG3-AS1 DHX35 ENSG00000287347 SP140L HCN1 CRACDL ENSG00000286288 CHRM5 SLC13A1 PI4KB AC129926.2 FRY LINC00670 RHOT1 ELOVL5 AC010809.1 AL354810.1 USE1 HOOK3 AL513323.1 PIK3R5 PALLD LYZL1 TLR1 LINC02767 CECR2 TAS2R30 TMCO3 CASC16 RFESDP1 BMT2 AC233699.1 OR52P2P RIPK4 LINC01876 AVPR1B LMAN1L ENSG00000286406 AC091073.1 GOLGA6L10 QDPR FRRS1 IQCM PTK2 AP002373.1 AC006482.1 ENSG00000293467 AC079414.1 NOP9 USP7 IGLV3-2 TICAM2 ENSG00000293472 SCP2 NPEPPSP1 SH2B2 LINC02518 ATP1B4 AL031963.1 AVEN AC019270.1

		AC006153.1 ZNF41 LINC02250 AC046185.1 GATD3 CELF1 HYCC1 DNAH12 POTEG POGZ LINC01090 PIP4K2B PRRC2B SYNM-AS1 UQCC1 ENSG00000288996 LRRK2 INO80D AF279873.3 ZNF395 IKZF3 GET1-SH3BGR SLC38A4 AC006043.1 AC244517.4 MIR548XHG FRY-AS1 CLSTN2 APRG1 ARIH1 SAMD12-AS1 CFAP298 PLD1 AC015908.2 VTCN1 ITFG2 PKHD1 ENSG00000287039 MEG8 TENT5C RARB UBXN7 KIF23-AS1 GDNF-AS1 LINC02435 AC104169.1 ENSG00000290429 PJA2 SNORD114-7 TCF4 AC079742.1 ENSG00000287334 MGST1 RBM44 ARHGEF38 PAEP COP1 TIAM1 PCA3 CSRP3 PHACTR1 MLIP IQSEC2 ACVR2A GNAO1 AC243830.3 AC034232.2 AL033530.1 ASAP2 LINC02309 TIPIN VIT LINC01324 AC091078.1 BAALC-AS1 AL606760.2 RASSF4 C6orf58 LOC124902439 ENSG00000288635 MIR6882 AC092979.1 SNORA5C SNTG2 AC060834.2 NTM AC091230.1 AC091133.4 AMMECR1 AC004805.1 NEDD4L FP236315.2 CYP1D1P LINC00639 TMED11P ENSG00000289368 BRCC3 CDH12P3 ENSG00000288683 AC093010.2 LINC00636 FECHP1 DUSP14 LINC01237 PRMT8 SNX13 AC090365.1 AC093893.1 RABGEF1 AC068724.3 KIT CYB5R4 TBC1D8 ENSG00000273937 EPHA7 ENSG00000291067 OSBPL3 SPAG9 AC104574.2 ANTXR1 ENSG00000293304 RMEL3 LOC100652967 AC131254.2 AC069257.3 LINC02256 C19orf47 AC068299.1 AC007326.2 SPDL1 WNT5A TRABD2B PEX26 TEX2 ARFGEF2 CTNNB1 AL671862.1 ANKRD17 EFCAB5 POTEKP AL096701.3 ENSG00000286811 COL19A1 RUNX1T1 CTSK LOC102723341 PRKDC AGAP4 AC097626.1 LINC00507 IPO5 ESR1 AC026124.1 LOC124900584 TNFRSF19 ZNF479 AC091045.1 LUZP2 RPL3P1 PTPN14 LINC02240 SCFD1 ERC2 POFUT2 LINC02973 DMGDH BCAR3 TPD52L1 L3MBTL4 PPM1E ZXDC AD000090.1 VPS53 LOC100129404 FRMD4A AC099788.1 CTSLP6 LMX1A DCAF8L1 MIR3670-3 LOC128462377 TPST2 FIGN CIMAP2 BTBD8 PARS2 AC103740.2 BNIPL HUNK TRAJ19 LINC01448 LINC00824 SNX29 FILIP1 LERFS TMEM62 SLC44A3-AS1 COPB2-DT FBXO15 EEDP1 PTGER4 AC018467.1 GREB1L AC015574.1 ZNF215 ENSG00000287392 PDE7B BBX AC096711.2 USP40 PPP1R15B-AS1 RASSF8-AS1 VWC2L BEST3 LYPD6B DOCK1 HDDC2 ENSG00000287180 AC131902.1 AC027644.4 CCDC107 TARDBPP2 AC005580.1 GPC4 BPGM PKIG ENSG00000286614 LINC00276 RPL21P10 EFNA5 LINC00536 AC073316.1 IGF2BP2 AL035420.1 AL391869.1 ACTR3B AC018638.8 CDC14B ZNF722 SYNE3 ENSG00000287684 LINC02808 PLCH1 OR1M1 HECTD1 VKORC1L1 NGEF TRPC5 NEUROD2 FNIP2 ZIM2-AS1 AC097478.1 AASS JAML KAT14 CCNJ UBE3B ADORA2A-AS1 PTPRF LINC01473 CASC15 IKBKB AC010280.1 DUX4L51 FXR2 SNHG33 SH3BP5 AC010809.2 AC068413.1 AKAP6 MIR3670-4 LOC101927690 ENSG00000293257 AC103409.1 TRBV10-2 USF3 AC002074.1 LYVE1 APOH AC004870.4 ARHGEF28 RAB3GAP2 CLCN1 TASOR CCPG1 AC104781.2 GPRIN1 EML6 OPCML LINGO2 CEMIP XIST AC074286.1 ATF2 LINC00877 AC140481.3 AC091151.1 RNU6-986P KCNJ12 ZNF426 HDAC8 ACVR1 CHRNB3 HLA-DQA2 PID1 COL6A5 HYAL4 EBF2 CCDC91 GRID2 ZNF423 ZCCHC17 KCNJ3 AC008632.1 AP000487.2 GLDC PLPPR1 SNORA63D SLC17A6-DT RB1 LINC00466 NSMCE1 AC078923.1 MPV17 LOC100131635 IFNLR1 SMIM2-AS1 RNU6-826P GOLGA6L4 PDE4B AL365214.3 MYHAS DDX10 LINC02994 PKD1L3 KIF13A NLRP4 ADIPOR1 ENSG00000286655 SLC23A4P LOC124900205 GABRB3 ZNF143 GOSR2 PET117 SHROOM4 AC093865.1 ZSCAN23 SMG7 PLPP3 UNC80 HNF1B RNU6-1311P IMMP2L ZNF615 AP001599.1 LAMP2 CCDC54 SSPN SPSB1 KIRREL3 GABRG2 F5 SCG5-AS1 CFAP95 AK5 CPS1 NCOA2 TMEM120A AC023300.1 AC104984.2 BNIP3L ENSG00000287614 ACACB AC021088.1 AGPAT5 RABGAP1L LINC01948 ENSG00000286476 LOC101927141 LINC00603 XRCC5 TBRG4 LOC107984536 RGS17 HMGB3P2 LINC01965 SHLD2 GFRA1 SPHKAP KCND2 INPP4A BMX OR5W1P TRAJ18 ZNF418 SBF2-AS1 AC012593.1 SLC24A3 RN7SL563P RAD54L2 MPRIP LINC02351 TAF15 TPST1 GRIA4 RNA5SP489 RAB18 POLD3 AP000526.1 ZNF860 TPH2 POU2F1 AL079305.1 AL445648.1 LINC00508 PRPF40B AC244517.6 AC004870.3 TMTC1 AL592490.1 ETV1 NRK RGS7BP BACH2 MGRN1 LOC339298 AC010745.2 TEX11 LINC02384 MID2 AC087683.2 RBM28 MIR198 LRTM2 LOC105375972 SIK1 ZCWPW2 FBXL13 OR10J4 GLOD5 ERCC6L2 ENSG00000291293 ENSG00000286779 PIPOX RPS2P1 AP002336.2 ZNF850

		TP53BP2 LINC00592 KAT2B ESPNP LSINCT5 GOLPH3L TMTC2 WLS ACTR3C ANKS1A IGKV1-6 RYR3 TMPRSS11B PRCP SUPV3L1 AL035446.1 MEF2D MYO1B MGAM2 MYL4 RPL39P31 AC091588.2 GTF2A1 SPTLC1 AC015922.2 AL137076.1 AC008758.4 AGR3 CDK5RAP2 FLVCR1 AC023830.3 LINC02343 FSIP2LP UBE2E3 RPL15P18 SAMD3 EPN2-AS1 RFC3 ERICH6B FARSB ARHGEF7 CCDC54-AS1 AL445430.1 ANKRD26P1 SAMD12 CU634019.3 DACH1 ENSG00000293462 RPL21P5 ATF3 LINC03082 TRDN LOC105377043 LINC01117 GASK1A ENSG00000290597 NPHP1 AL136146.2 LINC00499 AC111152.2 TEKT4P2 ENSG00000290412 IKZF1 AC005154.5 DAOA-AS1 SLC26A8 CAPZA2 GGT8P AC004147.4 AC011405.1 AC002066.1 TRAJ20 SRI ATP1B1 GREB1 AL158154.2 ADAT2 ARNT BDNF LDLRAD4 TFCP2L1 ACBD6 SEMA3E BCLAF3 HAS2-AS1 SCIN AL163195.3 ENSG00000293265 AP004609.1 MACO1 ZNF665 TLR6 ENSG00000293512 CTBP2P10 ENSG00000287443 NDST4 ADAMTS9 ADGRF2P ITPR2-AS1 STIM1 TSEN15 TMEM237 TMEM161B ENSG00000288563 HAUS6 RAB11FIP2 EEFSEC COX7B2 LAG3 DPP10-AS1 ENSG00000291189 Z95331.1 PPARG LOC124902888 AC016573.1 TMEM72-AS1 TANC1 PAPPA LOC101927605 ENSG00000290217 IRAG1-AS1 ASB3 AC239585.2 MTTP GPR84-AS1 LOC102724934 CHRNB4 AC019322.1 AGAP9 CTBP2P9 AP000282.1 TGM5 NEK7 INTS15 ZBED3-AS1 ZNF80 PDXDC2P TENM1 IL13RA2 ENSG00000293489 LINC01322 RORB AL136441.1 GABRB1 LINC02903 MYOF AP001116.1 LINC01754 AC142384.1 LTB4R2 ATG12P1 JAKMIP1 SH3KBP1 NALCN AC116903.2 SMOX AC020718.1 AVIL LPCAT2 ENSG00000286163 GDAP1 AMY2A AC023509.6 AC126755.4 ENSG00000287801 ENSG00000289870 LINC00398 ISY1-RAB43 EIF2AP4 TTC39C MYLK4 MIR3670-1 BEND7 DIO2-AS1 Z98043.1 TPRXL USP25 KIF2C AA06 MYO5A SIPA1L3 CTSLP1 LINC00393 SLC6A13 ZNF286A-TBC1D26 LRFN2 WDFY4 SPTBN5 AC068138.1 AC092862.1 EIF1B-AS1 SFRP4 BLTP1 SPDYE16 LINC01239 OR4Q2 PNRC1 IL1RAP ULK4P3 SEMA6A-AS1 AC015922.3 ENSG00000286662 AL772307.1 JCAD VAV2 TFRC DANT1 VPS13A TES OSBPL6 SH3TC1 GRIA3 PNPLA8 SPDYE6 AC087564.1 FBN1 AC108474.1 HAPSTR1 SERPINA7P1 RAB31 ENSG00000291283 EXO5-DT DIPK2B ZFP82 MAP3K1 DDX4 IQCB1 COA1 ZNF292 CLEC6A BMPER FUT10 AC109779.1 LINC00922 RNU6-984P CLASP2 ZNF521 RPRD2 ZNF761 PDXDC2P-NPIPB14P AC024230.1 ANGPT1 DUXAP10 SEMA6A VIPR2 ZNF567-DT EIF4G1 PARAIL TMPRSS15 AL133372.2 EEF1A1P11 B3GAT2 ATP6V0A4 ACSM5 PHLPP1 ZNF618 RBKS ITGA4 DNAJC6 CXXC4-AS1 ROCK1 SCIRT AL390860.1 NCAPH M1AP ENSG00000286072 TAX1BP1 AC008739.5 OR7A15P AL445430.2 TBC1D4 AC093843.1 APIP AC012456.1 MMP28 FAM13C NECTIN1-DT LINC02971 ZNF701 KCNT2 CRYBB2P1 NUP98 CLEC3A IKZF2 PML BX571673.1 FOXO3B AC012616.1 LAYN MGLL SCFD2 SCARA5 LONRF3 SLC5A12 PLEK2 TNFSF4 ENSG00000293021 AFAP1L2 DGKG SPATA25 LINC00923 SP100 ENSG00000287616 RNA5SP260 PLXDC2 ELAVL4 SBF2 CDH9 RF01880 FOXRED2 AL731556.2 AL359736.1 NDUFAF2 CFAP298-TCP10L LINC01524 AC022335.1 UMODL1 RFPL4B TTC28 TSEN2 GALK2 PCDH19 ENSG00000287783 NIN LINC02060 SPG21 ZNF286A GARS1-DT ANKFY1 ST7-OT4 ZNF69 CCDC144CP PRSS50 MIR4645 GMDS-DT ENSA ENSG00000286259 MIR9-2HG PPP4R1L FNDC3A KHDRBS3 AIMP1 SLC30A10 AP4S1 TPK1 MYO5BP1 FOXG1-AS1 PRKD1 HSPBP1 CALCRL POT1-AS1 CASC17 SLC20A2 USP34 AC091053.1 STK4 AC138305.1 PCDH10 AC023300.3 ENSG00000289174 TNNI3K AL646090.2 ENSG00000287184 DNAAF4-CCPG1 RAB6D TMEM131 SPC25 AC112493.1 ENSG00000287378 TMEM161B-DT RNASE11 ST6GAL2 AC018742.1 AC244205.1 SCAMP1 PTCHD4 TXNL4AP1 RPL27A LINC01002 ANPEP AC021979.1 LINC01821 PRLR ZNF569 GOLGA8O LINC01697 ARL2BPP10 ENSG00000289397 AP005436.1 ZNF180 AC008758.3 PDE11A NMI RAB6C PCCA CLVS1 YLPM1 IL16 LINC00972 AC012485.1 ENSG00000291276 DIPK1A LINC02814 SULT1C2P2 ZSWIM7 ZNF470 ENSG00000286932 AC107373.1 POTEH LINC02339 EDARADD LINC02511 LINC00558 GNA14 OXNAD1 CR2 LPAR6 ENSG00000286152 ZNF56P CTBP2 TTN LINC01721 NCALD RPS10P13 HHIP DYRK1A LOC105377209 PPFIA1 TMEM30A LINC01705 MYRIP ERICH2-DT LINC00343 ZNF286B PPP1R1C AL355838.1 UNC13C KCNE2 RGS10 CUL4A

		LINC02882 NDUFAF4P1 AC105052.3 SERINC1 KCTD16 LINC00265 CHSY3 TXNRD3 EFCAB13 AC026358.1 TRAM2-AS1 ZNF888 EYA4 AL391361.2 LINC01748 AC069287.3 AL163953.1 ARL15 SPATA13 LINC01288 ZNF736 AL031432.1 MAP7D3 SEMA5A ENSG00000288035 5S_rRNA AL354861.2 LINC02762 ENSG00000290805 EIF3FP1 ELL2 SAMMSON LINC00886 LAMA4 PMEPA1 LINC01811 UBE3C ATE1 ENSG00000293330 ETNK1 AC110772.1 TTPA MYO5BP2 CYP4F30P PVT1 SLC2A1-DT GCNT1P3 CHAC2 GATAD2B FMNL2 METTL3 KRT85 UAP1 ZKSCAN7-AS1 PARD3 LINC00691 KCNN4 LINC01104 TRDC ZNF33A AL353132.1 CP FUNDC1 RNU6-849P COL6A6 BMAL2 ATP8B1 PGPEP1 AC026462.4 BZW1-AS1 RNF6 SEMA3C VAV1 SMYD1 PEPD ENSG00000289871 DPY19L1 GRM3-AS1 GRIA2 VDAC2 KIAA0825 AC002127.2 MIR3670-2 ENSG00000276197 VPS13B SPART AC018697.1 LOC102723684 IGLV4-3 LINC02315
Matrigel	1797	*SLC18A1 OR2AF1P KLHL13 ABCB7 AL355499.1 PBX4 ACOT12 NHLRC3 LOC124903770 ENO1P2 OR11P1P MYO9B LINC00683 COL4A5 LINC01708 ERG ENSG00000293384 PARN ENSG00000291325 AL390816.1 STK16 XK SEMA4D MET MAX TNS3 PDE4DIPP4 SCYL3 TEAD1 LOC105370954 EIF4G3 ACSM3 AC231532.2 SNX16 AC116035.1 WWC3-AS1 LINC02476 LINC02487 SPAG11B LINC01915 IFTAP EOLA2 ZNG1F SUMO1P2 LINC00261 LINC01944 LINC02997 SIAH3 DUS3L CETN3 AC116424.1 RASA4B ARL14EP-DT GAS2L1 RPL4P1 AC108025.1 CHMP1B MMP16 OR7E25P ENSG00000286104 METTL25 AC091231.1 ELAPOR2 FAXC PRORP ZNF534 TOX3 POLR2J4 LINC02334 DNAH11 SLC9A9 RALY-AS1 CDC42BPA POLR2J POLG AC092957.1 EXOC2 DBIP2 AL358934.1 MYLK-AS1 TACR1 FSIP1 ENSG00000289332 SNX25 AC068672.2 LINC02064 SDC2 AC131571.1 ABHD12 AP002954.1 AC005999.1 COX10 CIITA FANCC AP006219.1 ARAP1 UQCRHL LINC00305 AC073488.11 HEPHL1 RN7SL354P ANKLE2 FAM218A GACAT3 CEP85 RPRD1B CR381653.1 LINC01828 CIP2A LOC105371855 AHNAK ACTR5 PPP1R3B-DT YBX1P5 EIF4A3 DLEC1 COX10-DT NFE2L2 AC004943.3 AC069335.1 SCHIP1 AC004594.1 SLC46A2-AS1 TFEC ENSG00000286830 NFYC BLOC1S5-TXNDC5 H2BC18 UPP2 CYP19A1 LOC105374367 SLC46A2 UBTD1 AC073488.10 LINC00313 DTNB GPR158 VRK2 ITSN1 AC092552.1 R3HDM2 ATP2B2 AC073488.2 PREX2 ESPNL SCRN1 AC007314.1 FAM177A1 ELAVL2 AC073488.5 TANC2 MAP9-AS1 PSKH2 ENSG00000286332 AC135507.1 NAV2-AS4 CSMD3 FBF1 NTN4 SERP1 AC090888.1 POLR1A PLEKHM3 ENKUR LOC105375146 CENPBD2P TPTE2 AC017002.6 RAB11FIP4 AC010307.2 CLMAT3 AC116353.4 C10orf53 HSFX4 NPIPB5 SNTA1 PPCDC CHD1-DT RPL7AP83 ENSG00000289178 ENSG00000293110 LINC02578 TTC28-AS1 CCDC144A CBX3P10 ASZ1 CU638689.3 ENSG00000289699 NUB1 ENSG00000293482 SRGAP2C MISFA WIF1 FLI1 DYM-AS1 LINC02197 SPATS2L PABPC5-AS1 CLIC5 ESRRB ARHGAP26 ZFHX2 LINC-PINT RBP7 NR2F2-AS1 TRHDE RAB44 PTPN4 AC105180.1 LEMD1-AS1 SLC16A11 SETBP1 ZBTB7C ITGA2 ENSG00000288643 AL442647.1 ENSG00000290070 KARS1P2 MIR5702 AC093459.1 AC026316.5 IL1RAPL1 AC091564.3 KIF5C BLK GPM6A SPATA16 AC068205.2 AC034195.1 WNT16 RERGL KRT86 SETD3 MRPS35P2 ACOX3 ADCY1 GRID1 RPS6KA2 EFR3B PLPPR5 LINC01790 TRIM24 ZDHHC17 HLCS FIG4 AC106864.2 LINC01621 AP1S3 CFAP47 FOXP2 LOC102724452 RNU6-389P FP325331.1 ENSG00000288016 FBXO34 ENSG00000286069 KMT2D GUSBP5 LPAR3 C3orf49 CKMT1B SHE ANO4 GIPC2 AC008825.1 GFOD1 ENSG00000288106 TXLNG SP110 SH3BP2 SH3TC2 CEP290 FAM171B CNTNAP3P2 AC239859.1 LRIG3-DT TACC1 SH3PXD2B OCLN AC092484.1 CFAP91 AC024598.1 FASTKD5 NETO1 EGFR PTPRQ GBE1 TNPO1 AC097634.4 SNX32 PCGEM1 DNPEP LOC100506321 LINC00589 AF130417.1 NEO1 LINC02894 GLT8D1 MYT1L SRGAP2B STAG1 LINC02428 CLDN14 IQCJ-SCHIP1 AC004702.1 CARM1P1 GUCY1B2 LINC01968 LINC01829 PDE3A-AS1 TIMM23 PLEKHB2 CAPN11 UBOX5 TCERG1 LINC01608 AC024257.1 LINC00869 F10 SLC7A14-AS1 CKMT1A LINC01323 LINC00970 CFAP20DC-DT DNPEP-AS1 LINC00370 SCN1A-AS1 LINC03099 FAM184A ARHGEF35-AS1 AC073325.1 CABIN1 PSMD10 FBXO47 KRT89P CCNO-DT FBXL17 PDZD2 EOLA1 SLC9A4 DHX29 STPG2 AP004833.1 GPAM MSRB3-AS1 IRF2 HEATR6 GET1P1 SPATA6L LRP12 TCF12-DT WEE2-AS1 AC093766.1 ENSG00000291120 LOC100287944*

		AL353133.2 TBC1D22A LINC01492 ESRP1 HSFX3 NPIPB4 UBE2A BRMS1L GOLGA6L1 RBM41 AC099329.2 AC011477.4 RGS12 LNCOG AC245102.2 LINC02963 AC007529.2 ACOXL CBX3 LINC02955 ACAP2 SLC39A10 PRKG1-AS1 AC020687.1 ENSG00000289788 ZNF496 CHCHD6 ZNF550 MPHOSPH9 KIZ LOC102724289 RNF10 MGC4859 FAT1 ENSG00000287051 SLC2A2 PIK3R1 AL078621.3 IL17B AC139143.1 LOC101928253 AGMO NOVA1 SARNP SHTN1 AC100818.1 ATG12 AC073488.4 FNDC3B MASP1 AC034114.2 TMEM232 CD200R1 LINC00587 BTD CALD1 CCDC144NL-AS1 SH3GL2 ZNF847P CLBA1 HIBADH ENSG00000288577 LINC02233 CRTC1 VCF1 FHIP1A N4BP2L1 PITPNC1 SLC30A3 LINC00663 AL137009.1 ENSG00000286353 DOCK7 AC106706.1 PDK3 PTCSC2 XKR4 FBXL4 ROR1 SMAD3-DT RAPGEF4-AS1 CCDC178 ADAMTSL1 PAX5 AAK1 AC090023.2 RTL8B LINC02755 CRB1 LINC02436 UTP15 AC116353.5 ARID1A THEMIS LOC124900945 SMAD6 RYBP NYAP2 PSD3 AL355922.4 ABCA13 AC012368.1 ZNF519 MEF2C FZR1 ST8SIA5 DBNL TBC1D19 PPP1R9A-AS1 AC005381.1 ELP4 RYK MACF1 AP2B1P1 PXT1 STARD4-AS1 DENND11 AC079466.2 TUBGCP3 ITIH5 WEE2 IQCK SLC15A5 TFDP2 MRPS10P2 AC110296.1 NDUFAF4P3 LINC02930 PTPRE MFSD6 SERINC3 AL035401.1 TBC1D31 ZNF347 PTPN12 PTPRZ1 LINC01749 AC105450.1 ACACA AC026786.2 PLN EML1 CTDSPL2 DDX39BP1 SLC66A3 SLC16A7 ALDH7A1P2 NEDD4 OCLNP1 NEK4 CACNA1E AC073488.9 AC024559.1 LOC105375297 ENSG00000293315 FLYWCH1 LRRC38 OVAAL AC078777.1 VWA8 CYSLTR2 PARVA LRRC37A2 AC002064.1 AC106729.1 LINC02237 SECISBP2 URI1 LOC127903862 SLC9C1 MARK3P1 SCML2 ANKRD28 AL031847.2 SNAP25-AS1 CHD6 PDE4DIPP2 ZNF609 PRDX1 NDUFAF6 PACS2 AL121782.1 ATG4B AEBP2 SLC7A14 AL133255.1 DSC2 TRG-AS1 NPSR1-AS1 GLB1L SERPINA1 LOC100132172 LINC00376 OLFM1 RN7SL77P MID1 AC020897.1 TSHZ2 POGK LINC00862 SLC12A2 EFNB3 OTX1 TRIM37 LOC101926964 ENSG00000286432 LINC02458 LINC02775 DLG3 LOC105376219 ENSG00000291284 PROSER1 PVRIG FLOT2 SRP68 SV2B LINC01879 ILK LEF1 EYA1 DAAM2-AS1 MAPK14 ZNF816-ZNF321P MYBPC3 WDR17 ENSG00000290921 AC011499.1 ZFHX4 RAPGEF1 ALG13 EFCAB14 GPRC6A AC068633.1 AC009139.2 EOLA2-DT UNC79 LOC100129616 ZNF516 SLC1A2 HOMER2P1 AC027228.2 ZEB1-AS1 EIF2S3B ARHGEF3 BTBD9-AS1 GABRB2 LOC101927293 AC010196.1 GRIN2B UBXN10 SVOP CRISPLD1 NOP16 IFNGR2 DENND4A ROR1-AS1 TBC1D20 AC083939.1 PLD5 OXR1 LINC00700 AC245748.2 SFMBT2 SNX2 LOC729732 GRHPR AL929601.1 SCAMP5 IL17RB PARP2 MINDY3 MYL1 ZSWIM6 AC006041.1 KLHL4 TTC3-AS1 LINC03096 EIF3F RDH16 FCHSD2 SLC4A7 CFAP418-AS1 RNU6-1117P F10-AS1 DNAJB6 LINC02612 LINC02279 NPIPB3 RPL23AP7 ENSG00000289530 ENSG00000290967 LINC02683 ZNF724 SPCS1 PRR4 MTUS1 LEMD3 PLEKHF2 B3GLCT PIEZO1 OR2T11 GGT4P SPTLC1P2 ENSG00000287042 NEB AC103719.1 AL157359.2 AC090888.3 CSRNP3 AC087762.1 KANSL1L MRGPRX1 AL157944.1 KLHL32 AC018618.1 TMEM100 LINC00861 LINC02253 NUP210 PDIA5 VDR LOC127814297 C16orf74 AC097625.1 CCDC149 NEK11 CREB3L2 RTN4 ENSG00000286962 GNL3L AC096887.1 CACNA1D PRKAA2 ARHGEF11 IVNS1ABP SGPP2 CD163 DPT BBS2 CLSTN1 TMEM150C ZNF155 SERGEF SCYL2 AOPEP SKAP1 AC190387.1 CU634019.5 LINC02375 KRT18P35 AC022816.1 CHM AC026415.1 SKIC3 MRPL3 MYO3A SERPINE2 ITGA2-AS1 ZNG1E LOC101928866 NUBPL PLAAT3 GGNBP1 IL12RB2 LINC01221 LINC00363 LOC100507336 PIK3CG ANOS1 COL4A4 TPP1 LOC100130691 STIL ANKFN1 TRGV5 ATP6V1E2 NBPF10 NUP160 AL935212.2 FGF14-IT1 MCCD1P1 AC132803.1 AC137810.1 CDHR17P AC121757.1 IQGAP1 MYCBP2 WIPI1 MIR6841 LATS1 AC098850.3 NUP153-AS1 PHYHIPL ZSCAN25 ANKRD20A21P LINC01442 AC007881.2 LOC124903099 ENSG00000293441 ENSG00000286215 KLHL12 LOC349160 ENSG00000286097 SNX30 AC007092.1 LINC00942 FANCI THNSL1 OR9Q1 ACAA1 ENSG00000290589 ITGA9-AS1 CPEB2-DT PRSS23 WNT2B CPHL1P LRRC37A3 ENSG00000287299 EXD3 SPNS3 TNFRSF10B IUR1 MAP2 RNU6-973P AC023078.5 GRM5P1 ENSG00000286982 NPIPB13 ENSG00000287771 FBXW11 MTCL1 HSPH1 AC008268.1 SUGP2 AZIN2 AC108517.1 IYD AC026992.1 SLC29A4P1 HNRNPA1P36 MIR646HG NHSL1 SMG6 HLA-DMA

		AC093802.1 CEACAM16-AS1 AC010343.3 TCERG1P2 LOC105370500 ENSG00000286041 PEMT ARHGAP6 LINC01845 ENSG00000289503 PPP2R2B CHD5 LINC02196 JAK1 GPD2 IL12A-AS1 ITGA9 RADIL KCNB1 LINC00298 TRIM39-RPP21 AC084200.1 AC105919.1 GNPAT HSD17B12 HS6ST3 GRM4 ANKRD27 LOC100887080 AIM2 ANK3 LINC01283 LINC00911 RF02271 LINC00578 ZNF19 TEX21P TMEM132B MARK2P11 AL391095.1 AMT ENPP7P10 KITLG SATL1 ARID4B GMDS NDUFS5P5 PLEKHM2 NR1I2 THSD4 ZNF266 PENK-AS1 LINC01471 ENSG00000286371 LINC01414 AC073488.8 AC004852.2 AC068313.1 RFX7 RELN SRP14P2 AF107885.2 ENSG00000286556 LRRC37A ENSG00000286239 STAG3L4 PDHB ADAMTSL3 NXPH1 KMT2CP1 AC012467.2 CRKL LINC00871 TENT5A ARHGEF10 SYTL3 NDC1 AC009093.2 SULT1C4 LINC02742 SNAP91 LINC02074 ZNF816 CDKAL1 AC104452.1 VAV3 RPS6KC1 CD40LG VRK1 AC100802.1 PAPOLB ENSG00000287950 URM1 LINC03041 GPAT3 ENSG00000287862 DPYSL3 ZNF420 SCN9A AC073488.3 C1orf146 SPRING1 AL807742.1 SLC16A10 PLCE1-AS2 LINC01252 ATRN CCDC88A NDUFB9 SNORA36C SLC35B4 UGT3A2 ARNT2 SPOCK1 RPS20P32 PITX1-AS1 HPSE2 PLCE1 AC007262.2 TNIP3 FAM106A TRMT2B-AS1 ENSG00000288799 TACC2 AC073488.6 AC009950.1 NT5DC1 CYP39A1 AC099511.1 AC006927.5 FRAS1 AC022568.1 DCAF5 LINC01423 GNAL AP004607.6 CSTF1 STK32C MIR7110 AC090114.3 OR7A1P RNU6-10P MSH3 UBE2F-SCLY LINC02664 BAK1 MRPL45 LRRC37A4P AC034268.2 DIS3L2P1 LAX1 AC013644.1 LINC00705 PTPRG-AS1 STK26 AC002428.1 ENOX2 TENM4 AC068152.1 GOLGA6L25 TTC9-DT LOC102723446 RPS12P20 PPP3CA CHRNA5 CES2 AC133065.1 DUX4 MKNK1 DYSF NKD1 AL157762.1 ENSG00000286856 ENSG00000287849 RBPJ KMT2CP3 AL356807.1 RIN2 PDE6C AL137247.1 AC016629.1 PLEKHG7 DLX6-AS1 ASCC1 CDH10 DENND1A MZT1 LINC02068 ENSG00000290317 PDZRN3 ADAMTS16 TASP1 LINC00381 NBPF19 MICU1 AC008700.1 TAF10 ENSG00000287329 MBD5 BACE2 KDM5A AC009154.1 ZNF549 AC010328.3 AP002761.2 LINC02026 LOC102724354 LOC101927855 NOMO1 AL133375.1 ARL6IP6 LINC01579 CNOT6 PKIB SPANXA2-OT1 LINC02284 TBX2-AS1 ASAH2 FRYL LINC01145 TBC1D9B STON1 PXMP2 ASH1L RSPO2 LINC02266 AC016598.2 AC010636.2 ZNF805 ACSF2 ENSG00000288632 OR5K1 OR13C9 DISC1 PROX1 MAP3K7CL FMN1 ZNF891 RALGPS1 CCDC6 PAQR5-DT PIEZO2 AC026474.1 AL133353.1 LINC00613 Z96074.1 VSIG1 CU633906.3 LRRC42 ITGB1 RBM26 AP003108.3 AC022031.2 RNA5SP29 CYB561 AC010632.3 DRC7 ZNF827 AL109837.2 UBA5P1 LINC00240 DSE LINC02346 LINC01823 ENSG00000291175 RHCE CORO1C LHFPL6 SPIDR CCDST C17orf80 AP001180.5 NEFL NAV2 TMEM185B STK3 CHN2 RNF150 NBEAP3 FCAR FSIP2-AS1 C1GALT1 HNF4G SMCO4 MSR1 EXOSC3 MED15P7 GABRG3 ENSG00000290790 PSMD7-DT AL138752.2 LINC03095 DPY19L1P1 AC245060.5 SH2D3C APTX TRPC4 RAMP3 GNG2 ZNF705A FRS3 PCDH11X CPB1 LIMS1 LINC01456 ENPP2 DOCK4 ACKR2 ARPC1A LINC01900 LINC00382 MCTP2 ENSG00000286458 BCL11B CERS3 GLCE PLS3 AC062021.1 TSPAN2 PCSK2 KCNIP1 PWWP2A LOC105373170 SLC29A4P2 ZFPM2-AS1 TBC1D10A ENSG00000287188 SSBP2 REEP1 RPH3A CTTNBP2 LINC01088 NME9 CYTH4 NAP1L1 SLC38A6 AC245297.3 AL121594.1 HDAC2-AS2 VPS29 PLPP4 CCDC102B LINC01985 AC132219.1 COPB2 UBR1 AC005972.3 CNN2 COL4A2 SLC1A6 KMT2CP2 ARHGAP32 DYRK3 IP6K2 AC114501.2 AC069444.2 LNC-LBCS CHRNA6 AP000688.3 AP002765.1 LINC02929 AC002428.2 ENSG00000287478 TLN2 HDAC4 F11-AS1 LINC01276 FYB1 RIOK3P1 AL935212.1 RBM45 SEM1 AC073488.1 GYS2 IQCE LVRN RPGRIP1L MYLK SPRY4-AS1 UNC5C CFAP69 MINDY4 CSMD2 TLR7 FLG2 PABIR3 P3H2 AC064859.1 CFL1 BAZ2B LARS2-AS1 PPIAP76 ENSG00000288067 LINC01793 DTYMK ENSG00000290821 WDR7 DIAPH2-AS1 SLAIN2 HMG20A CEP85L TUSC3 COPG2 PLEKHG4B ZFHX2-AS1 RBBP8 AC090517.4 MED13 RNASEH2B PHF2 TAAR2 RAI2 AFDN CAPS2 DEPDC1-AS1 LRP2 CYTIP MTDH ENSG00000290606 ENSG00000287918 ENSG00000288804 GOLGA6L6 LIG1 ZDHHC13 WWC3 LINC02715 CAMK4 SERPINA9 GALNT14 CLPX AC009084.1 VWC2 NCAL1 LINC01170 REPS1 FBXL7 RPA3 TOMM40P2 RNU6-1229P TMEM108 RPL29P26 CC2D2B CLUHP10 CHMP1B-AS1 DOCK7-DT MB21D2 SEZ6L AC005828.4 PCYOX1L SLC39A8

		AFG2B MANCR AC009242.1 LINC00412 MAP4K3-DT UPRT ATXN1 TMEM59L ADAM17 LINC00968 ENSG00000286185 FAM78B SEMA4F AL133353.2 ELAVL1 LIPJ ZFHX3-AS1 AC004922.1 TRAK1 STXBP1 OR6N1 ENSG00000287923 AC008696.2 IDH1 AL109914.1 LMOD2 AC092329.1 LINC00424 ENSG00000289846 LOC112268173 MIR4713HG TSNAX-DISC1 LINC01958 HDGFL2 MUC13 NUP205 PLEKHG1 NHLH1 AP005203.1 ELP2 SLCO5A1 RPSAP37 AC021443.1 SNTB1 ORC4 LINC01723 AC135586.2 AC022031.1 AC021231.1 CYP8B1 ENSG00000286500 ZNF350-AS1 APC PEX5L-AS1 LINC00540 AC090371.2 TTLL5 INO80 AL513325.1 NAA11 LOC728554 MOCS2 ERGIC2 PEAR1 CDA LINC01591 BANCR PPARGC1B STON1-GTF2A1L CTRB1 COL27A1 AC093523.1 ESR2 AC239727.1 AC005225.3 AC091551.1 AL359237.1 AC124254.1 AC108081.1 AC092650.1 NOL10 AC068759.1 LNX1 AC087386.1 LOC105374191 ERC1 ENSG00000287694 SMIM13 AF228730.5 MIR8058 PARM1-AS1 ZNF440 RUBCNL AGO3 UBOX5-AS1 C9 PVR CYYR1-AS1 CASTOR3P CKAP4 RPL39L ACER1 IQGAP2 CDYL RLF AC087289.4 ENSG00000290507 AC092645.1 TNPO1-DT ANKRA2 C12orf42 TAF5L AP000265.1 JPT1 CTNND2 CU633904.2 DNAH14 LARRPM AC010197.2 DELEC1 RASSF3 UGGT1 PACSIN2 RPL36AP47 MIR181A1HG DPYSL2 ENSG00000288700 NEK2P4 ENSG00000287169 NUP210L AC008574.1 CNTNAP3C GIMAP8 AL049775.2 RAB8B METTL8 AL023574.1 NSD2 ZNF516-DT GRM8 PHACTR3 AL139352.1 AC107023.1 EIF3J-DT AC005261.3 ENSG00000291209 AARS1 KCND1 ENSG00000286499 TMEM267 JADE3 CARD18 PHF3 PSMC2 ENSG00000287831 MAPKAP1 LINC01853 EXOC6B LINC01950 EVC2 LINC03017 GON4L ENSG00000293214 AC007179.2 AC007780.1 ENSG00000286812 DNAH6 MGAT5 ACOX1 IGHV3-42 HAO1 USP24 NUP153 LINC01581 FBXO38-DT HSD17B4 EFHD1 LCN12 AL500522.1 TRAV32 TRMT10B UFL1-AS1 COL21A1 RAI14 ENSG00000292277 LOC285626 GPATCH2L PIGR NRP2 GTF3A SIPA1L2 CAVIN1 MIR548AD GYPE MSL3-DT C1orf21 METTL15 TMEM165 AC016745.2 LINC01229 FOXJ2 RNU4-69P CEP162 HECTD4 ELOVL7 AC092111.3 NR3C1 DLX6 KMT2CP5 BORCS5 AC011466.2 FSTL4 ZC3HAV1 MTOR XACT LINC01797 ENSG00000287907 TRAV5 NBPF20 KSR1 LINC02217 FAF1 SNAP25 AC004691.1 DEFB124 SGSM1 ARL10 MFSD4B-DT CHST4 CCDC144BP AC005050.1 OR11H6 ENSG00000287258 AC083795.2 ENSG00000290565 GOLGA8A ZNF221 CLDND1 CR381670.1 PIP5K1A GH2 BTBD2 MMACHC AC131025.1 RAB6B HS6ST2 AC008539.1 KYNU AC015468.3 LOC105370906 ADAL TBXAS1 ASCC2 AC046136.1 SPRED1 ENSG00000289744 GALC GNG4 CNTNAP4 NUDT19P4 TUBA3D CDH26 ENSG00000293242 RNF32-DT SNX19P4 PDS5B CRIM1 ENSG00000289487 NALF2 MEI4 RBM46 ABCB5 RNF115 TRIM61 CYYR1 ESD CDHR3 LINC00689 PER2 LINC01076 ANKRD24 GLI3 IPO5P1 LHPP KAT6A BCOR KCNK12 NTRK3 RXFP1 LINC02932 LINC01493 ENSG00000287729 LINC01807 TADA1 GAB2 DIS3L2 ENSG00000290702 SNTB2 ENSG00000287291 ENSG00000286954 AC073263.1 PDCD10 PDE1A RSBN1L LOC107985643 PRAMEF11 LMTK2 AC023055.1 AL133268.3 HHAT LINC00632 CREB5 PSMA3P1 CCDC68 FAM227B ADGRG6 COPG2IT1 AL357153.3 GOLGA7 FERMT2 GCNA AC245519.1 GPR82 AC068587.3 AC130710.1 GLUD1 AC008056.2 TSPOAP1 ST3GAL2 DNM3 AC016885.1 SREK1IP1 PHIP EPB41 DENND5A LINC00309 SS18 LOC124900792 SFRP5 DOCK9 RPL23AP38 ADAM6 GOLGA6L24 AGBL3 ZYG11B PPARA NCKAP5 CD244 AP000311.1 LINC01643 RN7SKP173 LINC01933 SHISA6 IGLV3-1 NFASC AC122134.1 OR2A1-AS1 HDX RAI1 CPEB2 AC104339.1 ZNF229 LINC00513 GORAB-AS1 NXPE4 PHF6 HMGB4 ENSG00000288075 ENSG00000287533 FGF7 TENT5D MARCHF10 TBCA STXBP6 MGA DENND2B-AS1 AL137782.1 FOXP4-AS1 AC021660.2 RTTN CACNA1A AL596087.2 RN7SKP284 EDA LOC100533679 LIMCH1 TNFSF13B Z93403.1 AC006288.1 ENSG00000291170 MTMR7 CNTNAP3 PTH2R AC005909.1 LOC100420587 IAH1 COL14A1 AC073488.7 RNU2-38P RBM47 AC005828.3 ENSG00000286209 EIF2A JARID2 CYP1B1-AS1 CYTOR FIRRM HDAC2 TPO TGOLN2 FGD1 AC008415.1 TRIM39 INSYN2B TRRAP AC009090.6 AC105213.1 SEMA3D ENSG00000286134 LINC01422 NPSR1 GLIDR EIF4E3 USP48 KCNH5 RRM1 MAGEA1 CADM1 KLHL29 INPP5D CFAP161 THUMPD1 AL356309.1 CLYBL OR4C6 RN7SL797P ATF6 PRKAR1B GBF1 IPO11

		LINC02805 SERPINE1 CCDC88C ABHD15-AS1 BACH1 CUL9 PRAMEF4 ENSG00000289228 INMT-MINDY4 EWSR1 TM7SF3 SLC26A5 AKAP13 AC091047.1 PKM GPAT4-AS1 GCG MRPL45P2 ATP12A LINC01735 CCDC150 ENSG00000289293 TKFC LINC02218 SMARCAD1-DT PTPN3 ENSG00000292979 SEPHS1P1 FOXP1 STAC SCN8A AC010547.2 AF241728.1 RTL4 NNT SSX2IP ST8SIA2 TTC17 ELP1 IRF8 HIVEP3 AC074386.1 PHF5AP1 AC005999.2 AC018629.1 AL008638.3 HEPACAM2 KAT7P1 AC023824.1 RFX4 CCDC198 OR6K6 ZNF30 ZRANB2-DT REM1 LINC01482 AGAP1 FREM1 AC091564.7 RIPOR2 NDUFA10 CBR1-AS1 CD69 DBR1 FUNDC2 EPS15L1 CYB561A3 PARD3B AC021660.3 ENSG00000291188 XIRP2 Z99571.1 AC079950.1 LINC01248 AL356272.1 KDM3A ENSG00000293483 MZT2A CAST AC005394.1 KBTBD11-OT1 ADGRA3 GPR39 CASK HSPG2 RPS29P9 SLCO4C1 GCNT1 BPIFB3 SLC35F4 AF121898.1 GGA2 AC008277.1 USH1C AC118758.3 LOC101927468 LINC02307 AC136428.4 NLRP14 DNAJC13 FRMD4B LOC345471 PSME3IP1 GOLGA8B TRDV3 LINC01075 ENSG00000287469 LINC01478

Table S3. GO associations with biological processes (BP), molecular functions (MF), and cellular components (CC) of 2118 rDNA-contacting genes detected only in Mel Z cells grown on plastic. Related to Figure 2A.

GO.ID	Description	padj	Genes
		MF	
GO:0005515	protein binding	8.552439183814447e-13	*FSTL1,C10ORF90,APBB2,PFKP,ESYT1,STYK1,RPL19,RTN1,CDK14,MSRB3,PDE1C,MREG,NOSTRIN,ING3,NINL,MAP3K3,OSBPL8,SLC12A8,LRRN1,POTED,STX8,PHF20,FOXK2,CPNE4,RBM17,SMYD3,GARS1,RAPGEF6,ARHGEF9,TAFA2,ABR,MAOA,BLTP3B,TRAPPC9,SLC16A5,FNDC1,MAP3K13,EIF1AX,PPM1A,ARL13B,SAMSN1,THADA,LIMD1,TF,THRB,MYBPC1,USP42,IPP,RCSD1,SPTBN1,CDC42EP3,MYO5C,MAPK10,ITPRID1,CACNB1,USP32,AHCYL2,DAPP1,ATF7,GNG12,CHRNB2,SEZ6,CLDN6,CDH8,KCNQ5,GNB4,AGTPBP1,FHL1,TRMT6,PPP1R37,RBPMS,ATP10A,SH3PXD2A,SPINK5,SKAP2,PRND,ATP7B,PPM1H,GPBP1L1,KCNJ6,BRD4,ZAN,DOK4,CAMK2B,GRIP1,DDI2,GPATCH8,PGM1,DCT,DLGAP1,PECR,SLC38A9,ZNF264,HBP1,SDR16C5,RGPD2,ABCG8,XPO4,OTUD7A,MAP4K3,FPGT-TNNI3K,NOTCH2,CD93,FKBP5,TMOD2,SLC44A5,SDK2,HMBOX1,NPNT,DSC1,EPB41L3,MAP6,PARVB,CDH11,SHANK3,WDR26,NR4A3,SPTLC3,ARID1B,E2F7,SLC14A2,FOXO1,STYXL1,STK39,SMURF2,SRL,ADAMTS12,TBC1D16,PLXNA4,CDK18,YBX1,ARMT1,CFAP70,DCDC1,CACNA1C,FAM20B,FAM3C,ASB4,ARB2A,PRKN,NLGN4X,RMND5A,F13A1,TAFA1,PIK3C2B,XPO6,KDM7A,SLC44A3,FAM83B,IFT172,GPSM1,CBLL2,CDON,NLK,THSD7A,GNAI1,MRPL13,MAPKAPK3,NCAM2,TIAM2,ZNF10,TRAK2,CNMD,LRP8,TAGLN3,CNTN1,AGL,MEOX2,STAT5B,ARHGEF4,UBE2O,MUC17,CPEB4,EIF4ENIF1,HEATR9,ERBIN,ZNF121,MBTPS2,RGS22,CD247,CEP192,MCC,PTH,ADAR,DNAH10,PDE10A,PDHX,CLCN3,RHOJ,CDC37,FGF10,ZFYVE16,MECOM,COL4A6,SAMD15,TSPAN18,NLRP8,PLG,NEURL4,HERC5,CDH7,UBE2E2,NCK1,PUDP,CD84,FAM168A,NONO,RARRES1,KLHL42,NREP,EXO1,GABRA5,EBNA1BP2,MTCP1,ASTN1,SH3BGR,KDM2B,PREX1,RGPD3,AIG1,ERICH1,ZNF404,PMS1,CDIN1,WRNIP1,COL24A1,ANO3,GO*

			LGA8R,RSF1,KCTD3,NCOA7,C2ORF88,SEPTIN10,CIDEB,MARCHF1,MCPH1,MYO1D,SESTD1,COL1A2,DTNA,TMOD3,GEMIN5,EEF2KMT,MRTFB,XPO7,KCNS3,COL12A1,ABLIM1,RBMS1,CHRNA7,HECW1,HMGB1,ZNF304,DYNLT2B,CDIP1,RFLNA,LPP,HIVEP1,ATRX,TNIK,TGFB2,MDFIC,EPB41L4A,LAMA2,ADD3,TRIO,FBXO16,ZNF85,HOXC13,SMARCC1,NDST1,MUSK,KCNJ18,JAM3,KCNIP3,SFPQ,ALKBH1,ARPP21,RGS3,NUP93,RERG,PRKAR2A,CTIF,CFAP57,ATP11A,RBM23,CSTF3,SLC15A4,PCDHB8,GOLPH3,PRKG2,MBTPS1,NCF2,PIGV,USP10,SND1,IGFBP7,RSKR,EPHA5,SKOR2,CBR4,ENAH,SNX10,TMEM230,PPTC7,STON2,FAM171A1,STRADA,HUWE1,ZNF595,ASPH,RRAS2,SH3BGRL,ZDHHC4,UBR5,GTF2F2,SIM1,KIAA1217,ABTB2,SOX30,ANKS1B,CHL1,SPOCK3,GSN,PLS1,TCP11L1,SPON1,MAPRE3,TLR8,HPS3,KIF21A,SLIT3,ARMH3,CACUL1,NLRP7,ARHGEF39,CD226,PHC2,ZNF578,ANKRD11,ITPR1,PKP4,DNAAF11,PAK3,NDUFAF7,ABI3BP,KLF12,IPMK,TMEM183A,CAMK1G,PRUNE2,PRKAG2,MLLT3,CCNT2,BTG4,HAVCR1,TRAPPC10,CPQ,CNOT10,MR1,KRTAP21-2,F8,TNFRSF12A,OASL,CPT2,VPS8,ISY1,CXCR5,NCKAP1,PRPF40A,CLDN12,CNTN3,MGAM,CILK1,FOSL2,PACRG,ADGRD1,EPHA3,CHCHD3,LATS2,TYW3,MYH14,SPDYE2B,TPRG1,MRPL32,KLHL33,ERP29,CDYL2,ARHGAP18,UVRAG,MYOM2,LYN,USP6,ADGRB3,SUMO2,MTA3,UBL3,DIP2B,NME7,SEC24C,ATP6V1H,NUP58,FIGNL1,PDZD8,RRM2,CRYBB1,SIL1,LDB2,UNC93A,C21ORF62,ALDH1A1,RNASEL,NAV1,PAQR5,DBF4B,CORIN,TMCC1,LRRC7,DCP2,PDZRN4,SGMS2,SLC41A2,NOS3,VSX1,EIF3L,NOMO2,TNRC6C,MLLT10,BPHL,ERCC6,EIF2AK4,KIAA1614,MSRA,SYNPR,MAGEL2,MATN2,TGFBR2,ITGA11,CMKLR2,CFAP77,MARK4,MDN1,SYNJ1,TTC6,TCF7L2,CHAF1B,KIAA1328,ZNF462,CRTAC1,E2F6,ZFHX3,POLR2C,UNC119,SHISA2,GPM6B,RRAGD,ULK3,C2ORF50,SPDYE2,ZMAT4,GKAP1,LHFPL3,RGPD1,PPP2R5E,KPNB1,GSDMD,ADGRV1,ST7,RASGRF2,SERBP1,QTRT2,EPB41L5,GAD1,SCG5,NUDCD1,WAC,WHRN,OCA2,DAPK1,TDP2,ACSM2B,PRUNE1,SAR1B,TTC7B,INSYN2A,BTG3,APP,GATA4,ATF7IP2,SMAD5,IGF2BP1,PPIL6,RNLS,GSKIP,RNF38,ADAM12,UBE2G1,PELI2,SP140L,HCN1,CHRM5,PI4KB,RHOT1,ELOVL5,USE1,HOOK3,PALLD,TLR1,CECR2,RIPK4,AVPR1B,IQCM,PTK2,USP7,TICAM2,SCP2,SH2B2,ATP1B4,AVEN,ZNF41,CELF1,HYCC1,DNAH12,POTEG,POGZ,PIP4K2B,PRRC2B,UQCC1,LRRK2,IKZF3,SLC38A4,CLSTN2,ARIH1,CFAP298,PLD1,VTCN1,ITFG2,PKHD1,TENT5C,RARB,UBXN7,PJA2,TCF4,MGST1,RBM44,ARHGEF38,PAEP,COP1,TIAM1,CSRP3,PHACTR1,MLIP,IQSEC2,ACVR2A,GNAO1,ASAP2,TIPIN,RASSF4,C6ORF58,SNTG2,NTM,AMMECR1,NEDD4L,BRCC3,DUSP14,PRMT8,RABGEF1,KIT,TBC1D8,EPHA7,OSBPL3,SPAG9,ANTXR1,C19ORF47,SPDL1,WNT5A,TRABD2B,PEX26,ARFGEF2,CTNNB1,ANKRD17,RUNX1T1,CTSK,PRKDC,AGAP4,IPO5,ESR1,TNFRSF19,ZNF479,PTPN14,SCFD1,ERC2,DMGDH,BCAR3,TPD52L1,L3MBTL4,PPM1E,ZXDC,VPS53,LMX1A,DCAF8L1,TPST2,FIGN,BTBD8,PARS2,BNIPL,HUNK,FBXO15,PTGER4,BBX,VWC2L,BEST3,DOCK1,HDDC2,CCDC107,GPC4,BPGM,PKIG,EFNA5,IGF2BP2,ACTR3B,CDC14B,SYNE3,HECTD1,VKORC1L1,NGEF,TRPC5,NEUROD2,F

			NIP2,AASS,JAML,KAT14,UBE3B,PTPRF,IKBKB,FXR2,SH3BP5,AKAP6,USF3,LYVE1,APOH,ARHGEF28,RAB3GAP2,CLCN1,TASOR,CCPG1,GPRIN1,EML6,OPCML,LINGO2,CEMIP,ATF2,KCNJ12,ZNF426,HDAC8,ACVR1,HLA-DQA2,PID1,COL6A5,EBF2,GRID2,ZNF423,ZCCHC17,KCNJ3,GLDC,PLPPR1,RB1,NSMCE1,MPV17,IFNLR1,PDE4B,PKD1L3,KIF13A,NLRP4,ADIPOR1,GABRB3,ZNF143,GOSR2,SHROOM4,ZSCAN23,SMG7,PLPP3,HNF1B,IMMP2L,LAMP2,SPSB1,KIRREL3,GABRG2,F5,CPS1,NCOA2,TMEM120A,BNIP3L,ACACB,AGPAT5,RABGAP1L,XRCC5,TBRG4,RGS17,SHLD2,GFRA1,SPHKAP,KCND2,INPP4A,BMX,ZNF418,RAD54L2,MPRIP,TAF15,TPST1,GRIA4,RAB18,POLD3,ZNF860,POU2F1,PRPF40B,TMTC1,ETV1,BACH2,MGRN1,TEX11,MID2,LRTM2,SIK1,ZCWPW2,FBXL13,ERCC6L2,PIPOX,TP53BP2,KAT2B,GOLPH3L,TMTC2,WLS,ACTR3C,ANKS1A,RYR3,PRCP,SUPV3L1,MEF2D,MYO1B,MYL4,GTF2A1,SPTLC1,AGR3,CDK5RAP2,FLVCR1,UBE2E3,SAMD3,RFC3,FARSB,ARHGEF7,SAMD12,DACH1,ATF3,TRDN,NPHP1,IKZF1,SLC26A8,CAPZA2,SRI,ATP1B1,ADAT2,ARNT,BDNF,LDLRAD4,ACBD6,SEMA3E,SCIN,MACO1,TLR6,STIM1,TSEN15,TMEM237,HAUS6,RAB11FIP2,LAG3,PPARG,TANC1,PAPPA,ASB3,MTTP,CHRNB4,TGM5,NEK7,ZNF80,TENM1,IL13RA2,RORB,GABRB1,MYOF,JAKMIP1,SH3KBP1,NALCN,AVIL,LPCAT2,GDAP1,TTC39C,MYLK4,BEND7,USP25,KIF2C,MYO5A,SIPA1L3,SLC6A13,LRFN2,WDFY4,SPTBN5,SFRP4,BLTP1,SPDYE16,PNRC1,IL1RAP,VAV2,TFRC,VPS13A,TES,OSBPL6,SH3TC1,SPDYE6,FBN1,HAPSTR1,RAB31,MAP3K1,IQCB1,COA1,CLEC6A,BMPER,CLASP2,ZNF521,RPRD2,ANGPT1,SEMA6A,EIF4G1,TMPRSS15,ATP6V0A4,PHLPP1,ZNF618,RBKS,ITGA4,DNAJC6,ROCK1,NCAPH,M1AP,TAX1BP1,TBC1D4,APIP,MMP28,FAM13C,NUP98,IKZF2,PML,LAYN,MGLL,SCARA5,LONRF3,TNFSF4,AFAP1L2,SPATA25,SP100,PLXDC2,ELAVL4,SBF2,CDH9,FOXRED2,NDUFAF2,UMODL1,RFPL4B,TTC28,TSEN2,NIN,SPG21,ZNF286A,ANKFY1,ZNF69,ENSA,FNDC3A,KHDRBS3,AIMP1,SLC30A10,TPK1,PRKD1,HSPBP1,CALCRL,SLC20A2,USP34,STK4,TNNI3K,SPC25,RNASE11,ST6GAL2,SCAMP1,RPL27A,ANPEP,PRLR,ZNF569,ZNF180,PDE11A,NMI,RAB6C,PCCA,CLVS1,YLPM1,IL16,DIPK1A,ZSWIM7,POTEH,EDARADD,GNA14,OXNAD1,CR2,LPAR6,CTBP2,TTN,NCALD,HHIP,DYRK1A,PPFIA1,TMEM30A,MYRIP,PPP1R1C,UNC13C,KCNE2,RGS10,CUL4A,SERINC1,KCTD16,EYA4,ARL15,MAP7D3,SEMA5A,ELL2,LAMA4,PMEPA1,UBE3C,ATE1,ETNK1,TTPA,GATAD2B,FMNL2,METTL3,KRT85,UAP1,PARD3,KCNN4,CP,FUNDC1,BMAL2,ATP8B1,PGPEP1,RNF6,SEMA3C,VAV1,SMYD1,PEPD,GRIA2,VDAC2,KIAA0825,SPART
GO:0043167	ion binding	3.998808915058052e-9	*FSTL1,PFKP,ESYT1,STYK1,RGN,CDK14,ZSWIM1,MSRB3,PDE1C,ING3,ENSG00000290149,NINL,MAP3K3,PHF20,FOXK2,CPNE4,SMYD3,GARS1,RAPGEF6,MAOA,MAP3K13,PPM1A,ARL13B,LIMD1,TF,THRB,MYO5C,MAPK10,USP32,DAPP1,ATF7,CHRNB2,CDH8,AGTPBP1,FHL1,ATP10A,SH3PXD2A,PRND,ATP7B,CAMK2B,GPATCH8,PGM1,DCT,SLC38A9,ZNF264,ABCG8,OTUD7A,MAP4K3,GALNT9,FPGT-TNNI3K,NOTCH2,CD93,ZNF551,NPNT,DSC1,MORC1,CDH11,NR4A3,SPTLC3,ZNF611,STK39,SMURF2,SRL,ADAMTS12,FER1L6,CDK18,ARMT1,CACNA1C*

			,FAM20B,IDO1,PRKN,NLGN4X,RMND5A,PNPLA3,F13A1,PIK3C2B,KDM7A,SLC5A7,MARCHF9,CBLL2,NLK,SDF4,GNAI1,MAPKAPK3,PAPOLG,ZNF10,LRP8,ENPEP,NUDT19,UBE2O,CPEB4,CHRFAM7A,ZNF121,MBTPS2,MCC,ADAR,DNAH10,PDE10A,CLCN3,RHOJ,ZFYVE16,MECOM,NLRP8,STK32B,NUDT13,CDH7,UBE2E2,PUDP,EXO1,KDM2B,ZNF404,PMS1,WRNIP1,TYW1,COL11A1,RSF1,SEPTIN10,CIDEB,MARCHF1,MYO1D,SESTD1,ZNF214,COL1A2,DTNA,ABLIM1,ALPK2,CHRNA7,ZNF304,CDIP1,LPP,HIVEP1,ATRX,TNIK,TRIO,ZNF85,MUSK,KCNIP3,ALKBH1,ZCCHC4,RERG,PRKAR2A,ATP11A,ZNF154,ATP4A,PCDHB8,PRKG2,RSKR,EPHA5,CBR4,ATP13A4,ATP2C1,PPTC7,STRADA,ZNF595,ASPH,RRAS2,UBR5,SPOCK3,GSN,PLS1,SPON1,KIF21A,SLIT3,NLRP7,PHC2,ZNF578,ITPR1,CPM,PAK3,KLF12,IPMK,CYP2A13,CAMK1G,PRUNE2,PRKAG2,CPQ,F8,GDPD1,VPS8,ZNF831,ZNF429,DTD1,CILK1,EPHA3,LATS2,MYH14,LYN,KLF7,MTA3,NME7,SEC24C,FIGNL1,PDZD8,PAMR1,CA10,RRM2,NMNAT3,FMO5,RNASEL,PAQR5,DBF4B,PCDHB16,DCP2,PDZRN4,NOS3,ZNF280B,MLLT10,ERCC6,EIF2AK4,RAB12,HMGCLL1,MATN2,TGFBR2,ITGA11,MARK4,DDX60L,MDN1,ZNF462,OVCH1,CRTAC1,ZFHX3,RRAGD,ULK3,ZMAT4,CACNA2D4,KPNB1,GSDMD,ADGRV1,QTRT2,GAD1,SCG5,DAPK1,TDP2,ZNF678,ACSM2B,PRUNE1,SAR1B,APP,GATA4,SMAD5,RNLS,RNF38,ADAM12,UBE2G1,DHX35,SP140L,HCN1,PI4KB,RHOT1,BMT2,RIPK4,QDPR,FRRS1,PTK2,SCP2,ZNF41,DNAH12,POGZ,PIP4K2B,LRRK2,ZNF395,IKZF3,CLSTN2,ARIH1,RARB,PJA2,COP1,CSRP3,ACVR2A,GNAO1,ASAP2,BRCC3,PRMT8,RABGEF1,KIT,CYB5R4,EPHA7,ANTXR1,TRABD2B,EFCAB5,RUNX1T1,PRKDC,AGAP4,ESR1,ZNF479,L3MBTL4,PPM1E,ZXDC,LMX1A,FIGN,PARS2,HUNK,ZNF215,PDE7B,HDDC2,ACTR3B,ZNF722,PLCH1,HECTD1,TRPC5,IKBKB,ARHGEF28,ATF2,ZNF426,HDAC8,ACVR1,CHRNB3,EBF2,ZNF423,ZCCHC17,GLDC,NSMCE1,PDE4B,DDX10,KIF13A,NLRP4,ADIPOR1,ZNF143,ZSCAN23,ZNF615,F5,AK5,CPS1,ACACB,XRCC5,KCND2,BMX,ZNF418,RAD54L2,TAF15,RAB18,ZNF860,TPH2,NRK,MGRN1,MID2,SIK1,ZCWPW2,ERCC6L2,PIPOX,ZNF850,ACTR3C,RYR3,SUPV3L1,MYO1B,MYL4,SPTLC1,UBE2E3,FARSB,IKZF1,SRI,ADAT2,ACBD6,SCIN,ZNF665,ADAMTS9,STIM1,EEFSEC,PPARG,PAPPA,ENSG00000290217,AGAP9,TGM5,NEK7,ZNF80,RORB,MYOF,AVIL,LPCAT2,AMY2A,MYLK4,KIF2C,MYO5A,VAV2,TES,PNPLA8,FBN1,RAB31,ZFP82,MAP3K1,DDX4,ZNF292,CLEC6A,ZNF521,ZNF761,EIF4G1,B3GAT2,ACSM5,PHLPP1,ZNF618,RBKS,ITGA4,ROCK1,TAX1BP1,APIP,MMP28,ZNF701,KCNT2,IKZF2,PML,LONRF3,PLEK2,DGKG,SP100,CDH9,FOXRED2,UMODL1,RFPL4B,GALK2,PCDH19,NIN,ZNF286A,ANKFY1,ZNF69,TPK1,PRKD1,STK4,PCDH10,TNNI3K,RAB6D,ANPEP,PRLR,ZNF569,ZNF180,PDE11A,RAB6C,PCCA,CLVS1,ZSWIM7,ZNF470,GNA14,TTN,NCALD,HHIP,DYRK1A,MYRIP,UNC13C,CHSY3,TXNRD3,ZNF888,EYA4,ARL15,ZNF736,ETNK1,TTPA,GATAD2B,METTL3,PARD3,ZNF33A,CP,ATP8B1,RNF6,VAV1,SMYD1,PEPD,VDAC2
GO:0036094	small molecule binding	1.1324694145364215e-8	*FSTL1,PFKP,ESYT1,STYK1,RGN,CDK14,ZSWIM1,MSRB3,PDE1C,ING3,ENSG00000290149,NINL,MAP3K3,OSBPL8,PHF20,FOXK2,CPNE4,SMYD3,GARS1,RAPGEF6,MAOA,MAP3K13,PPM1A,ARL13B,LIMD*

			1,TF,THRB,MYO5C,MAPK10,USP32,DAPP1,ATF7,CHRNB2,CDH8,AGTPBP1,FHL1,ATP10A,SH3PXD2A,PRND,ATP7B,CAMK2B,GPATCH8,PGM1,DCT,SLC38A9,ZNF264,ABCG8,OTUD7A,MAP4K3,GALNT9,FPGT-TNNI3K,NOTCH2,CD93,ZNF551,NPNT,DSC1,MORC1,CDH11,NR4A3,SPTLC3,ZNF611,STK39,SMURF2,SRL,ADAMTS12,FER1L6,CDK18,ARMT1,CACNA1C,FAM20B,IDO1,PRKN,NLGN4X,RMND5A,PNPLA3,F13A1,PIK3C2B,KDM7A,SLC5A7,MARCHF9,CBLL2,NLK,SDF4,GNAI1,MAPKAPK3,PAPOLG,ZNF10,LRP8,ENPEP,NUDT19,UBE2O,CPEB4,CHRFAM7A,ZNF121,MBTPS2,MCC,ADAR,DNAH10,PDE10A,CLCN3,RHOJ,ZFYVE16,MECOM,NLRP8,STK32B,NUDT13,CDH7,UBE2E2,PUDP,EXO1,KDM2B,ZNF404,PMS1,WRNIP1,TYW1,COL11A1,RSF1,SEPTIN10,CIDEB,MARCHF1,MYO1D,SESTD1,ZNF214,COL1A2,DTNA,ABLIM1,ALPK2,CHRNA7,ZNF304,CDIP1,LPP,HIVEP1,ATRX,TNIK,TRIO,ZNF85,MUSK,KCNIP3,ALKBH1,ZCCHC4,RERG,PRKAR2A,ATP11A,ZNF154,ATP4A,PCDHB8,PRKG2,RSKR,EPHA5,CBR4,ATP13A4,ATP2C1,ALDH1A2,PPTC7,STRADA,ZNF595,ASPH,RRAS2,UBR5,SPOCK3,GSN,PLS1,SPON1,KIF21A,SLIT3,NLRP7,PHC2,ZNF578,ITPR1,CPM,PAK3,KLF12,IPMK,CYP2A13,CAMK1G,PRUNE2,PRKAG2,CPQ,F8,GDPD1,VPS8,ZNF831,ZNF429,DTD1,CILK1,EPHA3,LATS2,MYH14,LYN,KLF7,MTA3,NME7,SEC24C,FIGNL1,PDZD8,PAMR1,CA10,RRM2,ALDH1A1,NMNAT3,FMO5,RNASEL,PAQR5,DBF4B,PCDHB16,DCP2,PDZRN4,NOS3,ZNF280B,MLLT10,ERCC6,EIF2AK4,RAB12,HMGCLL1,MATN2,TGFBR2,ITGA11,MARK4,DDX60L,MDN1,ZNF462,OVCH1,CRTAC1,ZFHX3,RRAGD,ULK3,ZMAT4,CACNA2D4,KPNB1,GSDMD,ADGRV1,QTRT2,GAD1,SCG5,DAPK1,TDP2,ALDH9A1,ZNF678,ACSM2B,PRUNE1,SAR1B,APP,GATA4,SMAD5,RNLS,RNF38,ADAM12,UBE2G1,DHX35,SP140L,HCN1,PI4KB,RHOT1,BMT2,RIPK4,LMAN1L,QDPR,FRRS1,PTK2,SCP2,ZNF41,DNAH12,POGZ,PIP4K2B,LRRK2,ZNF395,IKZF3,CLSTN2,ARIH1,RARB,PJA2,PAEP,COP1,CSRP3,ACVR2A,GNAO1,ASAP2,BRCC3,PRMT8,RABGEF1,KIT,CYB5R4,EPHA7,OSBPL3,ANTXR1,TRABD2B,EFCAB5,RUNX1T1,PRKDC,AGAP4,ESR1,ZNF479,L3MBTL4,PPM1E,ZXDC,LMX1A,FIGN,PARS2,HUNK,ZNF215,PDE7B,HDDC2,ACTR3B,ZNF722,PLCH1,HECTD1,VKORC1L1,TRPC5,IKBKB,ARHGEF28,ATF2,ZNF426,HDAC8,ACVR1,CHRNB3,EBF2,ZNF423,ZCCHC17,GLDC,NSMCE1,PDE4B,DDX10,KIF13A,NLRP4,ADIPOR1,ZNF143,ZSCAN23,ZNF615,F5,AK5,CPS1,ACACB,XRCC5,KCND2,BMX,ZNF418,RAD54L2,TAF15,RAB18,ZNF860,TPH2,NRK,MGRN1,MID2,SIK1,ZCWPW2,ERCC6L2,PIPOX,ZNF850,ACTR3C,RYR3,SUPV3L1,MYO1B,MYL4,SPTLC1,UBE2E3,FARSB,IKZF1,SRI,ADAT2,ACBD6,SCIN,ZNF665,ADAMTS9,STIM1,EEFSEC,PPARG,PAPPA,ENSG00000290217,AGAP9,TGM5,NEK7,ZNF80,RORB,MYOF,AVIL,LPCAT2,AMY2A,MYLK4,KIF2C,MYO5A,VAV2,TES,OSBPL6,PNPLA8,FBN1,RAB31,ZFP82,MAP3K1,DDX4,ZNF292,CLEC6A,ZNF521,ZNF761,EIF4G1,B3GAT2,ACSM5,PHLPP1,ZNF618,RBKS,ITGA4,ROCK1,TAX1BP1,APIP,MMP28,ZNF701,KCNT2,IKZF2,PML,LONRF3,PLEK2,DGKG,SP100,CDH9,FOXRED2,UMODL1,RFPL4B,GALK2,PCDH19,NIN,ZNF286A,ANKFY1,ZNF69,TPK1,PRKD1,STK4,PCDH10,TNNI3K,RAB6D,A

			NPEP,PRLR,ZNF569,ZNF180,PDE11A,RAB6C,PCCA,CLVS1,ZSWIM7,ZNF470,GNA14,CTBP2,TTN,NCALD,HHIP,DYRK1A,MYRIP,UNC13C,CHSY3,TXNRD3,ZNF888,EYA4,ARL15,ZNF736,ETNK1,TTPA,GATAD2B,METTL3,PARD3,ZNF33A,CP,ATP8B1,RNF6,VAV1,SMYD1,PEPD,VDAC2
GO:0043169	cation binding	0.00000213698110249545	*FSTL1,PFKP,ESYT1,RGN,ZSWIM1,MSRB3,PDE1C,ING3,ENSG00000290149,NINL,MAP3K3,PHF20,FOXK2,CPNE4,SMYD3,MAP3K13,PPM1A,LIMD1,TF,THRB,USP32,ATF7,CHRNB2,CDH8,AGTPBP1,FHL1,ATP10A,PRND,ATP7B,GPATCH8,PGM1,DCT,SLC38A9,ZNF264,ABCG8,OTUD7A,GALNT9,NOTCH2,CD93,ZNF551,NPNT,DSC1,MORC1,CDH11,NR4A3,ZNF611,SMURF2,ADAMTS12,FER1L6,ARMT1,CACNA1C,FAM20B,IDO1,PRKN,RMND5A,F13A1,KDM7A,SLC5A7,MARCHF9,CBLL2,NLK,SDF4,GNAI1,PAPOLG,ZNF10,LRP8,ENPEP,NUDT19,CPEB4,CHRFAM7A,ZNF121,MBTPS2,MCC,ADAR,PDE10A,ZFYVE16,MECOM,STK32B,NUDT13,CDH7,PUDP,EXO1,KDM2B,ZNF404,WRNIP1,TYW1,COL11A1,RSF1,MARCHF1,ZNF214,COL1A2,DTNA,ABLIM1,CHRNA7,ZNF304,CDIP1,LPP,HIVEP1,ATRX,ZNF85,MUSK,KCNIP3,ALKBH1,ZCCHC4,ATP11A,ZNF154,ATP4A,PCDHB8,ATP13A4,ATP2C1,PPTC7,ZNF595,ASPH,UBR5,SPOCK3,GSN,PLS1,SPON1,SLIT3,PHC2,ZNF578,ITPR1,CPM,PAK3,KLF12,IPMK,CYP2A13,PRUNE2,PRKAG2,CPQ,F8,GDPD1,VPS8,ZNF831,ZNF429,DTD1,CILK1,LATS2,KLF7,MTA3,NME7,SEC24C,FIGNL1,PDZD8,PAMR1,CA10,RRM2,RNASEL,PAQR5,DBF4B,PCDHB16,DCP2,PDZRN4,NOS3,ZNF280B,MLLT10,HMGCLL1,MATN2,TGFBR2,ITGA11,ZNF462,OVCH1,CRTAC1,ZFHX3,ZMAT4,CACNA2D4,KPNB1,ADGRV1,QTRT2,TDP2,ZNF678,ACSM2B,PRUNE1,SAR1B,APP,GATA4,SMAD5,RNLS,RNF38,ADAM12,SP140L,RHOT1,BMT2,FRRS1,ZNF41,POGZ,LRRK2,ZNF395,IKZF3,CLSTN2,ARIH1,RARB,PJA2,COP1,CSRP3,ACVR2A,GNAO1,ASAP2,BRCC3,PRMT8,RABGEF1,KIT,CYB5R4,ANTXR1,TRABD2B,EFCAB5,RUNX1T1,AGAP4,ESR1,ZNF479,L3MBTL4,PPM1E,ZXDC,LMX1A,ZNF215,PDE7B,HDDC2,ZNF722,PLCH1,HECTD1,ARHGEF28,ATF2,ZNF426,HDAC8,ACVR1,CHRNB3,EBF2,ZNF423,ZCCHC17,GLDC,NSMCE1,PDE4B,ADIPOR1,ZNF143,ZSCAN23,ZNF615,F5,CPS1,ACACB,KCND2,BMX,ZNF418,TAF15,ZNF860,TPH2,MGRN1,MID2,SIK1,ZCWPW2,ZNF850,RYR3,MYL4,FARSB,IKZF1,SRI,ADAT2,SCIN,ZNF665,ADAMTS9,STIM1,PPARG,PAPPA,ENSG00000290217,AGAP9,TGM5,NEK7,ZNF80,RORB,MYOF,LPCAT2,AMY2A,VAV2,TES,FBN1,ZFP82,MAP3K1,ZNF292,CLEC6A,ZNF521,ZNF761,B3GAT2,ACSM5,PHLPP1,ZNF618,RBKS,ITGA4,ROCK1,TAX1BP1,APIP,MMP28,ZNF701,IKZF2,PML,LONRF3,DGKG,SP100,CDH9,UMODL1,RFPL4B,PCDH19,NIN,ZNF286A,ANKFY1,ZNF69,TPK1,PRKD1,STK4,PCDH10,TNNI3K,ANPEP,PRLR,ZNF569,ZNF180,PDE11A,PCCA,ZSWIM7,ZNF470,GNA14,TTN,NCALD,HHIP,MYRIP,UNC13C,CHSY3,ZNF888,EYA4,ZNF736,GATAD2B,METTL3,ZNF33A,CP,ATP8B1,RNF6,VAV1,SMYD1,PEPD,VDAC2*
GO:0005488	binding	0.0000241713181881757 5	*FSTL1,C10ORF90,APBB2,PFKP,ESYT1,STYK1,RPL19,RTN1,RGN,CDK14,ZSWIM1,MSRB3,PDE1C,MREG,HNRNPCL4,NOSTRIN,ING3,ENSG00000290149,NINL,MAP3K3,OSBPL8,SLC12A8,LRRN1,POTED,STX8,PHF20,FOXK2,CPNE4,RBM17,SMYD3,GARS1*

			,RAPGEF6,ARHGEF9,TAFA2,ABR,MAOA,BLTP3B,TRAPPC9,SLC16A5,FNDC1,MAP3K13,EIF1AX,PPM1A,ARL13B,SAMSN1,THADA,LIMD1,TF,THRB,MYBPC1,USP42,IPP,RCSD1,SPTBN1,CDC42EP3,MYO5C,MAPK10,ITPRID1,CACNB1,USP32,AHCYL2,DAPP1,ATF7,GNG12,CHRNB2,SEZ6,CLDN6,CDH8,KCNQ5,GNB4,AGTPBP1,ATF7-NPFF,FHL1,TRMT6,PPP1R37,RBPMS,ATP10A,SH3PXD2A,SPINK5,MRPS24,SKAP2,PRND,ATP7B,PPM1H,GPBP1L1,KCNJ6,BRD4,ZAN,DOK4,TNRC18,CAMK2B,GRIP1,DDI2,GPATCH8,PGM1,DCT,DLGAP1,PECR,SLC38A9,ZNF264,HBP1,SDR16C5,RGPD2,ABCG8,XPO4,OTUD7A,MAP4K3,GALNT9,FPGT-TNNI3K,NOTCH2,CD93,FKBP5,TMOD2,SLC44A5,SDK2,HMBOX1,ZNF551,RNU2-49P,NPNT,DSC1,EPB41L3,MAP6,MORC1,PARVB,CDH11,SHANK3,WDR26,NR4A3,SPTLC3,ARID1B,E2F7,SLC14A2,FOXO1,ZNF611,STYXL1,STK39,SMURF2,SRL,ADAMTS12,FER1L6,TBC1D16,PLXNA4,CDK18,YBX1,ARMT1,CFAP70,DCDC1,CACNA1C,FAM20B,FAM3C,ASB4,IDO1,ARB2A,PRKN,NLGN4X,RMND5A,PNPLA3,F13A1,TAFA1,PIK3C2B,XPO6,KDM7A,SLC44A3,SLC5A7,MARCHF9,FAM83B,IFT172,GPSM1,CBLL2,PTBP3,CDON,NLK,SDF4,THSD7A,GNAI1,MRPL13,MAPKAPK3,NCAM2,PAPOLG,TIAM2,ZNF10,TRAK2,CNMD,LRP8,TAGLN3,CNTN1,AGL,MEOX2,STAT5B,ENPEP,NUDT19,ARHGEF4,UBE2O,MUC17,CPEB4,EIF4ENIF1,HEATR9,CHRFAM7A,ERBIN,ZNF121,MBTPS2,RGS22,CD247,CEP192,MCC,PTH,ADAR,DNAH10,PDE10A,PDHX,CLCN3,RHOJ,CDC37,FGF10,ZFYVE16,MECOM,COL4A6,SAMD15,TSPAN18,NLRP8,STK32B,PLG,NEURL4,HERC5,NUDT13,CDH7,UBE2E2,NCK1,PUDP,CD84,FAM168A,NONO,RARRES1,KLHL42,NREP,EXO1,GABRA5,EBNA1BP2,MTCP1,ASTN1,SH3BGR,KDM2B,PREX1,RGPD3,AIG1,ERICH1,ZNF404,PMS1,CDIN1,WRNIP1,COL24A1,ANO3,TYW1,GOLGA8R,COL11A1,RSF1,KCTD3,NCOA7,C2ORF88,RNU2-33P,SEPTIN10,CIDEB,MARCHF1,MCPH1,MYO1D,SESTD1,ZNF214,COL1A2,DTNA,TMOD3,GEMIN5,EEF2KMT,MRTFB,XPO7,KCNS3,COL12A1,ABLIM1,RBMS1,ALPK2,CHRNA7,HECW1,HMGB1,ZNF304,DYNLT2B,CDIP1,AFF3,RFLNA,LPP,HIVEP1,ATRX,TNIK,TGFB2,MDFIC,EPB41L4A,LAMA2,ADD3,TRIO,FBXO16,ZNF85,HOXC13,SMARCC1,NDST1,MUSK,KCNJ18,JAM3,KCNIP3,SFPQ,ALKBH1,ARPP21,RGS3,NUP93,ZCCHC4,RERG,OR5AU1,PRKAR2A,RBM33,CTIF,CFAP57,ATP11A,RBM23,ZNF154,ATP4A,CSTF3,SLC15A4,PCDHB8,GOLPH3,PRKG2,MBTPS1,NCF2,PIGV,RNU6-1216P,USP10,SND1,IGFBP7,RSKR,EPHA5,SKOR2,CBR4,ATP13A4,ENAH,SNX10,ATP2C1,TMEM230,ALDH1A2,PPTC7,STON2,IGHV1OR21-1,FAM171A1,STRADA,HUWE1,ZNF595,ASPH,RRAS2,SH3BGRL,ZDHHC4,UBR5,GTF2F2,SIM1,KIAA1217,ABTB2,SOX30,ANKS1B,CHL1,SPOCK3,GSN,PLS1,TCP11L1,SPON1,MAPRE3,TLR8,HPS3,KIF21A,SLIT3,ARMH3,CACUL1,NLRP7,ARHGEF39,CD226,PHC2,ZNF578,ANKRD11,ITPR1,PKP4,CPM,DNAAF11,PAK3,NDUFAF7,ABI3BP,RNU1-83P,KLF12,IPMK,CYP2A13,TMEM183A,CAMK1G,PRUNE2,PRKAG2,MLLT3,CCNT2,BTG4,HAVCR1,TRAPPC10,CPQ,CNOT10,MR1,KRTAP21-2,F8,TNFRSF12A,OASL,GDPD1,CPT2,VPS8,ISY1

			,ZNF831,ZNF429,CXCR5,NCKAP1,PRPF40A,CLDN12,DTD1,CNTN3,MGAM,CILK1,FOSL2,PACRG,ADGRD1,HNRNPCL3,EPHA3,CHCHD3,LATS2,TYW3,MYH14,SPDYE2B,TPRG1,MRPL32,KLHL33,ERP29,CDYL2,ARHGAP18,UVRAG,MYOM2,LYN,USP6,ADGRB3,KLF7,SUMO2,MTA3,UBL3,DIP2B,NME7,SEC24C,ATP6V1H,NUP58,FIGNL1,PDZD8,PAMR1,CA10,RRM2,CRYBB1,SIL1,LDB2,MYRFL,UNC93A,C21ORF62,ALDH1A1,NMNAT3,FMO5,RNASEL,NAV1,PAQR5,DBF4B,CORIN,TMCC1,LRRC7,PCDHB16,DCP2,PDZRN4,SGMS2,SLC41A2,NOS3,VSX1,ZNF280B,EIF3L,NOMO2,TNRC6C,MLLT10,BPHL,ERCC6,EIF2AK4,KIAA1614,MSRA,SYNPR,RAB12,IMPG1,HMGCLL1,MAGEL2,MATN2,TGFBR2,ITGA11,CMKLR2,CFAP77,MARK4,DDX60L,MDN1,SYNJ1,TTC6,TCF7L2,CHAF1B,KIAA1328,ZNF462,MTRF1,OVCH1,CRTAC1,E2F6,ZFHX3,POLR2C,CUX1,UNC119,SHISA2,GPM6B,RRAGD,ULK3,C2ORF50,SPDYE2,ZMAT4,GKAP1,CACNA2D4,LHFPL3,RGPD1,PPP2R5E,KPNB1,GSDMD,ADGRV1,ST7,RASGRF2,SERBP1,QTRT2,EVA1C,EPB41L5,GAD1,SCG5,NUDCD1,WAC,WHRN,OCA2,DAPK1,TDP2,ALDH9A1,ZNF678,ACSM2B,PRUNE1,SAR1B,TTC7B,INSYN2A,BTG3,APP,GATA4,ATF7IP2,SMAD5,IGF2BP1,PPIL6,RNLS,GSKIP,RNF38,ADAM12,UBE2G1,PELI2,DHX35,SP140L,HCN1,CHRM5,PI4KB,RHOT1,ELOVL5,USE1,HOOK3,PIK3R5,PALLD,TLR1,CECR2,BMT2,RIPK4,AVPR1B,LMAN1L,QDPR,FRRS1,IQCM,PTK2,NOP9,USP7,TICAM2,SCP2,SH2B2,ATP1B4,AVEN,ZNF41,CELF1,HYCC1,DNAH12,POTEG,POGZ,PIP4K2B,PRRC2B,UQCC1,LRRK2,ZNF395,IKZF3,SLC38A4,CLSTN2,ARIH1,CFAP298,PLD1,VTCN1,ITFG2,PKHD1,TENT5C,RARB,UBXN7,PJA2,TCF4,MGST1,RBM44,ARHGEF38,PAEP,COP1,TIAM1,CSRP3,PHACTR1,MLIP,IQSEC2,ACVR2A,GNAO1,ASAP2,TIPIN,VIT,RASSF4,C6ORF58,SNTG2,NTM,AMMECR1,NEDD4L,BRCC3,ENSG00000288683,DUSP14,PRMT8,SNX13,RABGEF1,KIT,CYB5R4,TBC1D8,EPHA7,OSBPL3,SPAG9,ANTXR1,C19ORF47,SPDL1,WNT5A,TRABD2B,PEX26,TEX2,ARFGEF2,CTNNB1,ANKRD17,EFCAB5,RUNX1T1,CTSK,PRKDC,AGAP4,IPO5,ESR1,TNFRSF19,ZNF479,PTPN14,SCFD1,ERC2,DMGDH,BCAR3,TPD52L1,L3MBTL4,PPM1E,ZXDC,VPS53,LMX1A,DCAF8L1,TPST2,FIGN,BTBD8,PARS2,BNIPL,HUNK,SNX29,FBXO15,PTGER4,ZNF215,PDE7B,BBX,VWC2L,BEST3,DOCK1,HDDC2,CCDC107,GPC4,BPGM,PKIG,EFNA5,IGF2BP2,ACTR3B,CDC14B,ZNF722,SYNE3,PLCH1,HECTD1,VKORC1L1,NGEF,TRPC5,NEUROD2,FNIP2,AASS,JAML,KAT14,UBE3B,PTPRF,IKBKB,FXR2,SH3BP5,AKAP6,USF3,LYVE1,APOH,ARHGEF28,RAB3GAP2,CLCN1,TASOR,CCPG1,GPRIN1,EML6,OPCML,LINGO2,CEMIP,ATF2,RNU6-986P,KCNJ12,ZNF426,HDAC8,ACVR1,CHRNB3,HLA-DQA2,PID1,COL6A5,EBF2,GRID2,ZNF423,ZCCHC17,KCNJ3,GLDC,PLPPR1,RB1,NSMCE1,MPV17,IFNLR1,RNU6-826P,PDE4B,DDX10,PKD1L3,KIF13A,NLRP4,ADIPOR1,GABRB3,ZNF143,GOSR2,SHROOM4,ZSCAN23,SMG7,PLPP3,HNF1B,RNU6-1311P,IMMP2L,ZNF615,LAMP2,SPSB1,KIRREL3,GABRG2,F5,AK5,CPS1,NCOA2,TMEM120A,BNIP3L,ACACB,AGPAT5,RABGAP1L,XRCC5,TBRG4,RGS17,SHLD2,GFRA1,SPHKAP,KCND2,INPP4A,BMX,ZNF

			418,RAD54L2,MPRIP,TAF15,TPST1,GRIA4,RAB18,POLD3,ZNF860,TPH2,POU2F1,PRPF40B,TMTC1,ETV1,NRK,BACH2,MGRN1,TEX11,MID2,RBM28,LRTM2,SIK1,ZCWPW2,FBXL13,ERCC6L2,PIPOX,ZNF850,TP53BP2,KAT2B,GOLPH3L,TMTC2,WLS,ACTR3C,ANKS1A,RYR3,PRCP,SUPV3L1,MEF2D,MYO1B,MGAM2,MYL4,GTF2A1,SPTLC1,AGR3,CDK5RAP2,FLVCR1,UBE2E3,SAMD3,RFC3,FARSB,ARHGEF7,SAMD12,DACH1,ATF3,TRDN,NPHP1,IKZF1,SLC26A8,CAPZA2,SRI,ATP1B1,ADAT2,ARNT,BDNF,LDLRAD4,TFCP2L1,ACBD6,SEMA3E,BCLAF3,SCIN,MACO1,ZNF665,TLR6,ADAMTS9,STIM1,TSEN15,TMEM237,TMEM161B,HAUS6,RAB11FIP2,EEFSEC,LAG3,PPARG,TANC1,PAPPA,ENSG00000290217,ASB3,MTTP,CHRNB4,AGAP9,TGM5,NEK7,ZNF80,TENM1,IL13RA2,RORB,GABRB1,MYOF,JAKMIP1,SH3KBP1,NALCN,AVIL,LPCAT2,GDAP1,AMY2A,TTC39C,MYLK4,BEND7,USP25,KIF2C,MYO5A,SIPA1L3,SLC6A13,LRFN2,WDFY4,SPTBN5,SFRP4,BLTP1,SPDYE16,PNRC1,IL1RAP,VAV2,TFRC,VPS13A,TES,OSBPL6,SH3TC1,GRIA3,PNPLA8,SPDYE6,FBN1,HAPSTR1,RAB31,ZFP82,MAP3K1,DDX4,IQCB1,COA1,ZNF292,CLEC6A,BMPER,RNU6-984P,CLASP2,ZNF521,RPRD2,ZNF761,ANGPT1,SEMA6A,VIPR2,EIF4G1,TMPRSS15,B3GAT2,ATP6V0A4,ACSM5,PHLPP1,ZNF618,RBKS,ITGA4,DNAJC6,ROCK1,NCAPH,M1AP,TAX1BP1,TBC1D4,APIP,MMP28,FAM13C,ZNF701,KCNT2,NUP98,CLEC3A,IKZF2,PML,FOXO3B,LAYN,MGLL,SCARA5,LONRF3,PLEK2,TNFSF4,AFAP1L2,DGKG,SPATA25,SP100,PLXDC2,ELAVL4,SBF2,CDH9,FOXRED2,NDUFAF2,UMODL1,RFPL4B,TTC28,TSEN2,GALK2,PCDH19,NIN,SPG21,ZNF286A,ANKFY1,ZNF69,ENSA,FNDC3A,KHDRBS3,AIMP1,SLC30A10,TPK1,PRKD1,HSPBP1,CALCRL,SLC20A2,USP34,STK4,PCDH10,TNNI3K,RAB6D,SPC25,RNASE11,ST6GAL2,SCAMP1,RPL27A,ANPEP,PRLR,ZNF569,ZNF180,PDE11A,NMI,RAB6C,PCCA,CLVS1,YLPM1,IL16,DIPK1A,ZSWIM7,ZNF470,POTEH,EDARADD,GNA14,OXNAD1,CR2,LPAR6,CTBP2,TTN,NCALD,HHIP,DYRK1A,PPFIA1,TMEM30A,MYRIP,PPP1R1C,UNC13C,KCNE2,RGS10,CUL4A,SERINC1,KCTD16,CHSY3,TXNRD3,ZNF888,EYA4,ARL15,ZNF736,MAP7D3,SEMA5A,ELL2,LAMA4,PMEPA1,UBE3C,ATE1,ETNK1,TTPA,PVT1,GATAD2B,FMNL2,METTL3,KRT85,UAP1,PARD3,KCNN4,ZNF33A,CP,FUNDC1,RNU6-849P,BMAL2,ATP8B1,PGPEP1,RNF6,SEMA3C,VAV1,SMYD1,PEPD,GRIA2,VDAC2,KIAA0825,VPS13B,SPART
GO:0046872	metal ion binding	0.00003110333221592608	*FSTL1,PFKP,ESYT1,RGN,ZSWIM1,MSRB3,PDE1C,ING3,ENSG00000290149,NINL,MAP3K3,PHF20,FOXK2,CPNE4,SMYD3,MAP3K13,PPM1A,LIMD1,TF,THRB,USP32,ATF7,CDH8,AGTPBP1,FHL1,ATP10A,PRND,ATP7B,GPATCH8,PGM1,DCT,SLC38A9,ZNF264,ABCG8,OTUD7A,GALNT9,NOTCH2,CD93,ZNF551,NPNT,DSC1,MORC1,CDH11,NR4A3,ZNF611,SMURF2,ADAMTS12,FER1L6,ARMT1,CACNA1C,FAM20B,IDO1,PRKN,RMND5A,F13A1,KDM7A,MARCHF9,CBLL2,NLK,SDF4,GNAI1,PAPOLG,ZNF10,LRP8,ENPEP,NUDT19,CPEB4,ZNF121,MBTPS2,MCC,ADAR,PDE10A,ZFYVE16,MECOM,STK32B,NUDT13,CDH7,PUDP,EXO1,KDM2B,ZNF404,WRNIP1,TYW1,COL11A1,RSF1,MARCHF1,ZNF214,COL1A2,DTNA,ABLIM1,ZNF304,CDIP1,LPP,HIVEP1,ATRX,ZNF85,MUS*

			K,KCNIP3,ALKBH1,ZCCHC4,ATP11A,ZNF154,ATP4A,PCDHB8,ATP13A4,ATP2C1,PPTC7,ZNF595,ASPH,UBR5,SPOCK3,GSN,PLS1,SPON1,SLIT3,PHC2,ZNF578,ITPR1,CPM,PAK3,KLF12,IPMK,CYP2A13,PRUNE2,CPQ,F8,GDPD1,VPS8,ZNF831,ZNF429,DTD1,CILK1,LATS2,KLF7,MTA3,NME7,SEC24C,FIGNL1,PDZD8,PAMR1,CA10,RRM2,RNASEL,PAQR5,DBF4B,PCDHB16,DCP2,PDZRN4,NOS3,ZNF280B,MLLT10,HMGCLL1,MATN2,TGFBR2,ITGA11,ZNF462,OVCH1,CRTAC1,ZFHX3,ZMAT4,CACNA2D4,KPNB1,ADGRV1,QTRT2,TDP2,ZNF678,ACSM2B,PRUNE1,SAR1B,APP,GATA4,SMAD5,RNF38,ADAM12,SP140L,RHOT1,FRRS1,ZNF41,POGZ,LRRK2,ZNF395,IKZF3,CLSTN2,ARIH1,RARB,PJA2,COP1,CSRP3,ACVR2A,GNAO1,ASAP2,BRCC3,RABGEF1,KIT,CYB5R4,ANTXR1,TRABD2B,EFCAB5,RUNX1T1,AGAP4,ESR1,ZNF479,L3MBTL4,PPM1E,ZXDC,LMX1A,ZNF215,PDE7B,HDDC2,ZNF722,PLCH1,HECTD1,ARHGEF28,ATF2,ZNF426,HDAC8,ACVR1,EBF2,ZNF423,ZCCHC17,NSMCE1,PDE4B,ADIPOR1,ZNF143,ZSCAN23,ZNF615,F5,CPS1,ACACB,KCND2,BMX,ZNF418,TAF15,ZNF860,TPH2,MGRN1,MID2,SIK1,ZCWPW2,ZNF850,RYR3,MYL4,FARSB,IKZF1,SRI,ADAT2,SCIN,ZNF665,ADAMTS9,STIM1,PPARG,PAPPA,ENSG00000290217,AGAP9,TGM5,NEK7,ZNF80,RORB,MYOF,LPCAT2,AMY2A,VAV2,TES,FBN1,ZFP82,MAP3K1,ZNF292,CLEC6A,ZNF521,ZNF761,B3GAT2,ACSM5,PHLPP1,ZNF618,RBKS,ITGA4,ROCK1,TAX1BP1,APIP,MMP28,ZNF701,IKZF2,PML,LONRF3,DGKG,SP100,CDH9,UMODL1,RFPL4B,PCDH19,NIN,ZNF286A,ANKFY1,ZNF69,PRKD1,STK4,PCDH10,TNNI3K,ANPEP,PRLR,ZNF569,ZNF180,PDE11A,PCCA,ZSWIM7,ZNF470,GNA14,TTN,NCALD,HHIP,MYRIP,UNC13C,CHSY3,ZNF888,EYA4,ZNF736,GATAD2B,ZNF33A,CP,ATP8B1,RNF6,VAV1,SMYD1,PEPD
GO:0016301	kinase activity	0.0017396232064444113	PFKP,STYK1,CDK14,MAP3K3,ABR,MAP3K13,MAPK10,BRD4,CAMK2B,MAP4K3,FPGT-TNNI3K,STK39,CDK18,FAM20B,PIK3C2B,NLK,MAPKAPK3,STK32B,ALPK2,TNIK,TRIO,MUSK,PRKAR2A,PRKG2,RSKR,EPHA5,STRADA,PAK3,IPMK,CAMK1G,PRKAG2,CILK1,EPHA3,LATS2,LYN,NME7,RNASEL,SGMS2,EIF2AK4,TGFBR2,MARK4,ULK3,GKAP1,DAPK1,PI4KB,PIK3R5,RIPK4,PTK2,PIP4K2B,LRRK2,ACVR2A,KIT,EPHA7,PRKDC,HUNK,IKBKB,ACVR1,AK5,BMX,NRK,SIK1,NEK7,SH3KBP1,MYLK4,MAP3K1,RBKS,ROCK1,DGKG,GALK2,TPK1,PRKD1,STK4,TNNI3K,TTN,DYRK1A,ETNK1
GO:0030554	adenyl nucleotide binding	0.0022302702627667896	PFKP,STYK1,CDK14,MAP3K3,GARS1,MAP3K13,MYO5C,MAPK10,ATP10A,ATP7B,CAMK2B,ABCG8,MAP4K3,FPGT-TNNI3K,STK39,CDK18,FAM20B,PIK3C2B,NLK,MAPKAPK3,PAPOLG,UBE2O,DNAH10,PDE10A,CLCN3,NLRP8,STK32B,UBE2E2,PMS1,WRNIP1,MYO1D,ALPK2,ATRX,TNIK,TRIO,MUSK,PRKAR2A,ATP11A,ATP4A,PRKG2,RSKR,EPHA5,CBR4,ATP13A4,ATP2C1,STRADA,KIF21A,NLRP7,PAK3,IPMK,CAMK1G,PRKAG2,CILK1,EPHA3,LATS2,MYH14,LYN,NME7,FIGNL1,ALDH1A1,NMNAT3,FMO5,RNASEL,NOS3,ERCC6,EIF2AK4,TGFBR2,MARK4,DDX60L,MDN1,ULK3,DAPK1,ACSM2B,RNLS,UBE2G1,DHX35,HCN1,PI4KB,RIPK4,QDPR,PTK2,DNAH12,PIP4K2B,LRRK2,ACVR2A,KIT,EPHA7,PRKDC,FIGN,PARS2,HUNK,ACTR3B,IKBKB,ACVR1,PDE4B,DDX10,KIF13A,NL

			RP4,AK5,CPS1,ACACB,XRCC5,BMX,RAD54L2,NRK,SIK1,ERCC6L2,ACTR3C,SUPV3L1,MYO1B,UBE2E3,FARSB,NEK7,MYLK4,KIF2C,MYO5A,PNPLA8,MAP3K1,DDX4,EIF4G1,ACSM5,RBKS,ROCK1,KCNT2,DGKG,GALK2,TPK1,PRKD1,STK4,TNNI3K,PCCA,CTBP2,TTN,DYRK1A,ETNK1,ATP8B1
GO:0016773	phosphotransferase activity, alcohol group as acceptor	0.0027483562828115216	*PFKP,STYK1,CDK14,MAP3K3,ABR,MAP3K13,MAPK10,BRD4,CAMK2B,MAP4K3,FPGT-TNNI3K,STK39,CDK18,FAM20B,PIK3C2B,NLK,MAPKAPK3,STK32B,ALPK2,TNIK,TRIO,MUSK,PRKG2,RSKR,EPHA5,STRADA,PAK3,IPMK,CAMK1G,PRKAG2,CILK1,EPHA3,LATS2,LYN,NME7,RNASEL,EIF2AK4,TGFBR2,MARK4,ULK3,DAPK1,PI4KB,RIPK4,PTK2,PIP4K2B,LRRK2,ACVR2A,KIT,EPHA7,PRKDC,HUNK,IKBKB,ACVR1,BMX,NRK,SIK1,NEK7,MYLK4,MAP3K1,RBKS,ROCK1,DGKG,GALK2,PRKD1,STK4,TNNI3K,TTN,DYRK1A,ETNK1*
GO:0043168	anion binding	0.0030240788185976926	*PFKP,STYK1,CDK14,MAP3K3,GARS1,RAPGEF6,MAOA,MAP3K13,ARL13B,MYO5C,MAPK10,DAPP1,ATP10A,SH3PXD2A,ATP7B,CAMK2B,SLC38A9,ABCG8,MAP4K3,FPGT-TNNI3K,SPTLC3,STK39,SRL,CDK18,FAM20B,NLGN4X,PNPLA3,PIK3C2B,NLK,GNAI1,MAPKAPK3,PAPOLG,UBE2O,DNAH10,PDE10A,CLCN3,RHOJ,ZFYVE16,NLRP8,STK32B,UBE2E2,PMS1,WRNIP1,TYW1,COL11A1,SEPTIN10,CIDEB,MYO1D,SESTD1,ALPK2,ATRX,TNIK,TRIO,MUSK,RERG,PRKAR2A,ATP11A,ATP4A,PRKG2,RSKR,EPHA5,CBR4,ATP13A4,ATP2C1,STRADA,RRAS2,GSN,KIF21A,NLRP7,ITPR1,PAK3,IPMK,CAMK1G,PRKAG2,CILK1,EPHA3,LATS2,MYH14,LYN,NME7,FIGNL1,NMNAT3,FMO5,RNASEL,NOS3,ERCC6,EIF2AK4,RAB12,TGFBR2,MARK4,DDX60L,MDN1,RRAGD,ULK3,GSDMD,GAD1,SCG5,DAPK1,ACSM2B,SAR1B,RNLS,UBE2G1,DHX35,HCN1,PI4KB,RHOT1,RIPK4,QDPR,PTK2,SCP2,DNAH12,PIP4K2B,LRRK2,ACVR2A,GNAO1,KIT,EPHA7,PRKDC,FIGN,PARS2,HUNK,ACTR3B,TRPC5,IKBKB,ACVR1,GLDC,PDE4B,DDX10,KIF13A,NLRP4,AK5,CPS1,ACACB,XRCC5,BMX,RAD54L2,RAB18,NRK,SIK1,ERCC6L2,PIPOX,ACTR3C,SUPV3L1,MYO1B,SPTLC1,UBE2E3,FARSB,ACBD6,SCIN,EEFSEC,PPARG,NEK7,AVIL,AMY2A,MYLK4,KIF2C,MYO5A,PNPLA8,RAB31,MAP3K1,DDX4,EIF4G1,ACSM5,RBKS,ROCK1,KCNT2,PLEK2,DGKG,FOXRED2,GALK2,NIN,TPK1,PRKD1,STK4,TNNI3K,RAB6D,PDE11A,RAB6C,PCCA,CLVS1,GNA14,TTN,DYRK1A,TXNRD3,ARL15,ETNK1,TTPA,PARD3,ATP8B1*
GO:0032559	adenyl ribonucleotide binding	0.0030494435852238913	*PFKP,STYK1,CDK14,MAP3K3,GARS1,MAP3K13,MYO5C,MAPK10,ATP10A,ATP7B,CAMK2B,ABCG8,MAP4K3,FPGT-TNNI3K,STK39,CDK18,FAM20B,PIK3C2B,NLK,MAPKAPK3,PAPOLG,UBE2O,DNAH10,PDE10A,CLCN3,NLRP8,STK32B,UBE2E2,PMS1,WRNIP1,MYO1D,ALPK2,ATRX,TNIK,TRIO,MUSK,PRKAR2A,ATP11A,ATP4A,PRKG2,RSKR,EPHA5,ATP13A4,ATP2C1,STRADA,KIF21A,NLRP7,PAK3,IPMK,CAMK1G,PRKAG2,CILK1,EPHA3,LATS2,MYH14,LYN,NME7,FIGNL1,NMNAT3,RNASEL,ERCC6,EIF2AK4,TGFBR2,MARK4,DDX60L,MDN1,ULK3,DAPK1,ACSM2B,UBE2G1,DHX35,HCN1,PI4KB,RIPK4,PTK2,DNAH12,PIP4K2B,LRRK2,ACVR2A,KIT,EPHA7,PRKDC,FIGN,PARS2,HUNK,ACTR3B,IKBKB,ACVR1,PDE4B,DDX10,KIF13A,NLRP4,AK5,CPS1,ACACB,XRCC5,BMX,RAD54L2,NRK,SIK1,ERCC6L2,ACTR3C,SUPV3L1,MYO1*

			B,UBE2E3,FARSB,NEK7,MYLK4,KIF2C,MYO5A,PNPLA8,MAP3K1,DDX4,EIF4G1,ACSM5,RBKS,ROCK1,KCNT2,DGKG,GALK2,TPK1,PRKD1,STK4,TNNI3K,PCCA,TTN,DYRK1A,ETNK1,ATP8B1
GO:0022824	transmitter-gated monoatomic ion channel activity	0.0033201821805503395	*CHRNB2,CHRFAM7A,GABRA5,CHRNA7,GRIK1,CHRNB3,GRID2,GABRB3,GABRG2,GRIA4,CHRNB4,GABRB1,GRIA3,GRIA2*
GO:0022835	transmitter-gated channel activity	0.0033201821805503395	*CHRNB2,CHRFAM7A,GABRA5,CHRNA7,GRIK1,CHRNB3,GRID2,GABRB3,GABRG2,GRIA4,CHRNB4,GABRB1,GRIA3,GRIA2*
GO:0032553	ribonucleotide binding	0.004975576467303199	*PFKP,STYK1,CDK14,MAP3K3,GARS1,MAP3K13,ARL13B,MYO5C,MAPK10,ATP10A,ATP7B,CAMK2B,ABCG8,MAP4K3,FPGT-TNNI3K,STK39,SRL,CDK18,FAM20B,PIK3C2B,NLK,GNAI1,MAPKAPK3,PAPOLG,UBE2O,DNAH10,PDE10A,CLCN3,RHOJ,NLRP8,STK32B,UBE2E2,PMS1,WRNIP1,TYW1,SEPTIN10,MYO1D,ALPK2,ATRX,TNIK,TRIO,MUSK,RERG,PRKAR2A,ATP11A,ATP4A,PRKG2,RSKR,EPHA5,ATP13A4,ATP2C1,STRADA,RRAS2,KIF21A,NLRP7,PAK3,IPMK,CAMK1G,PRKAG2,CILK1,EPHA3,LATS2,MYH14,LYN,NME7,FIGNL1,NMNAT3,RNASEL,NOS3,ERCC6,EIF2AK4,RAB12,TGFBR2,MARK4,DDX60L,MDN1,RRAGD,ULK3,SCG5,DAPK1,ACSM2B,SAR1B,UBE2G1,DHX35,HCN1,PI4KB,RHOT1,RIPK4,PTK2,DNAH12,PIP4K2B,LRRK2,ACVR2A,GNAO1,KIT,EPHA7,PRKDC,FIGN,PARS2,HUNK,ACTR3B,IKBKB,ACVR1,PDE4B,DDX10,KIF13A,NLRP4,AK5,CPS1,ACACB,XRCC5,BMX,RAD54L2,RAB18,NRK,SIK1,ERCC6L2,ACTR3C,SUPV3L1,MYO1B,UBE2E3,FARSB,EEFSEC,NEK7,MYLK4,KIF2C,MYO5A,PNPLA8,RAB31,MAP3K1,DDX4,EIF4G1,ACSM5,RBKS,ROCK1,KCNT2,DGKG,GALK2,NIN,TPK1,PRKD1,STK4,TNNI3K,RAB6D,PDE11A,RAB6C,PCCA,GNA14,TTN,DYRK1A,ARL15,ETNK1,ATP8B1*
GO:1904315	transmitter-gated monoatomic ion channel activity involved in regulation of postsynaptic membrane potential	0.0050140753124882885	*CHRNB2,CHRFAM7A,GABRA5,CHRNA7,GRIK1,CHRNB3,GRID2,GABRG2,GRIA4,CHRNB4,GABRB1,GRIA3,GRIA2*
GO:0017076	purine nucleotide binding	0.005202191334731892	*PFKP,STYK1,CDK14,MAP3K3,GARS1,MAP3K13,ARL13B,MYO5C,MAPK10,ATP10A,ATP7B,CAMK2B,ABCG8,MAP4K3,FPGT-TNNI3K,STK39,SRL,CDK18,FAM20B,PIK3C2B,NLK,GNAI1,MAPKAPK3,PAPOLG,UBE2O,DNAH10,PDE10A,CLCN3,RHOJ,NLRP8,STK32B,UBE2E2,PMS1,WRNIP1,SEPTIN10,MYO1D,ALPK2,ATRX,TNIK,TRIO,MUSK,RERG,PRKAR2A,ATP11A,ATP4A,PRKG2,RSKR,EPHA5,CBR4,ATP13A4,ATP2C1,STRADA,RRAS2,KIF21A,NLRP7,PAK3,IPMK,CAMK1G,PRKAG2,CILK1,EPHA3,LATS2,MYH14,LYN,NME7,FIGNL1,ALDH1A1,NMNAT3,FMO5,RNASEL,NOS3,ERCC6,EIF2AK4,RAB12,TGFBR2,MARK4,DDX60L,MDN1,RRAGD,ULK3,SCG5,DAPK1,ACSM2B,SAR1B,RNLS,UB*

			E2G1,DHX35,HCN1,PI4KB,RHOT1,RIPK4,QDPR,PTK2,DNAH12,PIP4K2B,LRRK2,ACVR2A,GNAO1,KIT,EPHA7,PRKDC,FIGN,PARS2,HUNK,ACTR3B,IKBKB,ACVR1,PDE4B,DDX10,KIF13A,NLRP4,AK5,CPS1,ACACB,XRCC5,BMX,RAD54L2,RAB18,NRK,SIK1,ERCC6L2,ACTR3C,SUPV3L1,MYO1B,UBE2E3,FARSB,EEFSEC,NEK7,MYLK4,KIF2C,MYO5A,PNPLA8,RAB31,MAP3K1,DDX4,EIF4G1,ACSM5,RBKS,ROCK1,KCNT2,DGKG,GALK2,NIN,TPK1,PRKD1,STK4,TNNI3K,RAB6D,PDE11A,RAB6C,PCCA,GNA14,CTBP2,TTN,DYRK1A,ARL15,ETNK1,ATP8B1
GO:0005524	ATP binding	0.005357351039394697	*PFKP,STYK1,CDK14,MAP3K3,GARS1,MAP3K13,MYO5C,MAPK10,ATP10A,ATP7B,CAMK2B,ABCG8,MAP4K3,FPGT-TNNI3K,STK39,CDK18,FAM20B,PIK3C2B,NLK,MAPKAPK3,PAPOLG,UBE2O,DNAH10,CLCN3,NLRP8,STK32B,UBE2E2,PMS1,WRNIP1,MYO1D,ALPK2,ATRX,TNIK,TRIO,MUSK,ATP11A,ATP4A,PRKG2,RSKR,EPHA5,ATP13A4,ATP2C1,STRADA,KIF21A,NLRP7,PAK3,IPMK,CAMK1G,PRKAG2,CILK1,EPHA3,LATS2,MYH14,LYN,NME7,FIGNL1,NMNAT3,RNASEL,ERCC6,EIF2AK4,TGFBR2,MARK4,DDX60L,MDN1,ULK3,DAPK1,ACSM2B,UBE2G1,DHX35,PI4KB,RIPK4,PTK2,DNAH12,PIP4K2B,LRRK2,ACVR2A,KIT,EPHA7,PRKDC,FIGN,PARS2,HUNK,ACTR3B,IKBKB,ACVR1,DDX10,KIF13A,NLRP4,AK5,CPS1,ACACB,XRCC5,BMX,RAD54L2,NRK,SIK1,ERCC6L2,ACTR3C,SUPV3L1,MYO1B,UBE2E3,FARSB,NEK7,MYLK4,KIF2C,MYO5A,PNPLA8,MAP3K1,DDX4,EIF4G1,ACSM5,RBKS,ROCK1,KCNT2,DGKG,GALK2,TPK1,PRKD1,STK4,TNNI3K,PCCA,TTN,DYRK1A,ETNK1,ATP8B1*
GO:0032555	purine ribonucleotide binding	0.007178287572990105	*PFKP,STYK1,CDK14,MAP3K3,GARS1,MAP3K13,ARL13B,MYO5C,MAPK10,ATP10A,ATP7B,CAMK2B,ABCG8,MAP4K3,FPGT-TNNI3K,STK39,SRL,CDK18,FAM20B,PIK3C2B,NLK,GNAI1,MAPKAPK3,PAPOLG,UBE2O,DNAH10,PDE10A,CLCN3,RHOJ,NLRP8,STK32B,UBE2E2,PMS1,WRNIP1,SEPTIN10,MYO1D,ALPK2,ATRX,TNIK,TRIO,MUSK,RERG,PRKAR2A,ATP11A,ATP4A,PRKG2,RSKR,EPHA5,ATP13A4,ATP2C1,STRADA,RRAS2,KIF21A,NLRP7,PAK3,IPMK,CAMK1G,PRKAG2,CILK1,EPHA3,LATS2,MYH14,LYN,NME7,FIGNL1,NMNAT3,RNASEL,ERCC6,EIF2AK4,RAB12,TGFBR2,MARK4,DDX60L,MDN1,RRAGD,ULK3,SCG5,DAPK1,ACSM2B,SAR1B,UBE2G1,DHX35,HCN1,PI4KB,RHOT1,RIPK4,PTK2,DNAH12,PIP4K2B,LRRK2,ACVR2A,GNAO1,KIT,EPHA7,PRKDC,FIGN,PARS2,HUNK,ACTR3B,IKBKB,ACVR1,PDE4B,DDX10,KIF13A,NLRP4,AK5,CPS1,ACACB,XRCC5,BMX,RAD54L2,RAB18,NRK,SIK1,ERCC6L2,ACTR3C,SUPV3L1,MYO1B,UBE2E3,FARSB,EEFSEC,NEK7,MYLK4,KIF2C,MYO5A,PNPLA8,RAB31,MAP3K1,DDX4,EIF4G1,ACSM5,RBKS,ROCK1,KCNT2,DGKG,GALK2,NIN,TPK1,PRKD1,STK4,TNNI3K,RAB6D,PDE11A,RAB6C,PCCA,GNA14,TTN,DYRK1A,ARL15,ETNK1,ATP8B1*
GO:0099529	neurotransmitter receptor activity involved in regulation of	0.007605958724217555	*CHRNB2,CHRFAM7A,GABRA5,CHRNA7,GRIK1,CHRNB3,GRID2,GABRG2,GRIA4,CHRNB4,GABRB1,GRIA3,GRIA2*

	postsynaptic membrane potential		
GO:0004672	protein kinase activity	0.008329016579996261	*STYK1,CDK14,MAP3K3,ABR,MAP3K13,MAPK10,BRD4,CAMK2B,MAP4K3,FPGT-TNNI3K,STK39,CDK18,NLK,MAPKAPK3,STK32B,ALPK2,TNIK,TRIO,MUSK,PRKG2,RSKR,EPHA5,STRADA,PAK3,CAMK1G,PRKAG2,CILK1,EPHA3,LATS2,LYN,NME7,RNASEL,EIF2AK4,TGFBR2,MARK4,ULK3,DAPK1,RIPK4,PTK2,LRRK2,ACVR2A,KIT,EPHA7,PRKDC,HUNK,IKBKB,ACVR1,BMX,NRK,SIK1,NEK7,MYLK4,MAP3K1,ROCK1,PRKD1,STK4,TNNI3K,TTN,DYRK1A*
GO:0140096	catalytic activity, acting on a protein	0.011002703407369306	*C10ORF90,STYK1,CDK14,ING3,MAP3K3,SMYD3,ABR,MAP3K13,PPM1A,IMMP1L,USP42,MAPK10,USP32,CDC14C,AGTPBP1,PPM1H,BRD4,CAMK2B,DDI2,OTUD7A,MAP4K3,GALNT9,FPGT-TNNI3K,FKBP5,STYXL1,STK39,SMURF2,ADAMTS12,CDK18,ARMT1,ASB4,PRKN,RMND5A,F13A1,KDM7A,MARCHF9,CBLL2,NLK,MAPKAPK3,ENPEP,UBE2O,MBTPS2,MECOM,STK32B,PLG,NEURL4,HERC5,PGPEP1L,UBE2E2,KLHL42,KDM2B,MARCHF1,EEF2KMT,ALPK2,HECW1,TNIK,TRIO,MUSK,PRKG2,MBTPS1,USP10,RSKR,EPHA5,PTPN20,PPTC7,STRADA,HUWE1,ASPH,ZDHHC4,UBR5,CPM,PAK3,NDUFAF7,CAMK1G,PRKAG2,CPQ,CILK1,EPHA3,LATS2,LYN,USP6,SUMO2,NME7,FIGNL1,PAMR1,RNASEL,CORIN,EIF2AK4,MAGEL2,TGFBR2,MARK4,OVCH1,ULK3,DAPK1,PPIL6,RNF38,ADAM12,UBE2G1,PELI2,RIPK4,PTK2,USP7,LRRK2,ARIH1,PJA2,COP1,ACVR2A,NEDD4L,BRCC3,DUSP14,PRMT8,RABGEF1,KIT,EPHA7,TRABD2B,CTSK,PRKDC,PTPN14,POFUT2,PPM1E,TPST2,FIGN,HUNK,USP40,CDC14B,HECTD1,KAT14,UBE3B,PTPRF,IKBKB,ATF2,HDAC8,ACVR1,NSMCE1,IMMP2L,CPS1,BMX,TPST1,TMTC1,NRK,MGRN1,SIK1,KAT2B,TMTC2,TMPRSS11B,PRCP,UBE2E3,ADAMTS9,PAPPA,TGM5,NEK7,MYLK4,USP25,MAP3K1,TMPRSS15,PHLPP1,DNAJC6,ROCK1,MMP28,NUP98,PML,LONRF3,RFPL4B,PRSS50,PRKD1,USP34,STK4,TNNI3K,ANPEP,TTN,DYRK1A,CUL4A,TXNRD3,EYA4,UBE3C,ATE1,PGPEP1,RNF6,SMYD1,PEPD*
GO:0097367	carbohydrate derivative binding	0.012464927309331401	*FSTL1,PFKP,STYK1,CDK14,MAP3K3,GARS1,MAP3K13,ARL13B,MYO5C,MAPK10,ATP10A,ATP7B,CAMK2B,ABCG8,MAP4K3,FPGT-TNNI3K,STK39,SRL,CDK18,FAM20B,PNPLA3,PIK3C2B,NLK,GNAI1,MAPKAPK3,PAPOLG,UBE2O,DNAH10,PDE10A,CLCN3,RHOJ,FGF10,NLRP8,STK32B,UBE2E2,PMS1,WRNIP1,TYW1,COL11A1,SEPTIN10,MYO1D,ALPK2,HMGB1,ATRX,TNIK,TRIO,MUSK,RERG,PRKAR2A,ATP11A,ATP4A,PRKG2,RSKR,EPHA5,ATP13A4,ATP2C1,STRADA,RRAS2,SPOCK3,KIF21A,SLIT3,NLRP7,PAK3,ABI3BP,IPMK,CAMK1G,PRKAG2,CILK1,EPHA3,LATS2,MYH14,LYN,NME7,FIGNL1,NMNAT3,RNASEL,NOS3,ERCC6,EIF2AK4,RAB12,IMPG1,TGFBR2,MARK4,DDX60L,MDN1,RRAGD,ULK3,EVA1C,SCG5,DAPK1,ACSM2B,SAR1B,APP,UBE2G1,DHX35,HCN1,PI4KB,RHOT1,RIPK4,PTK2,DNAH12,PIP4K2B,LRRK2,ACVR2A,GNAO1,VIT,KIT,EPHA7,CTSK,PRKDC,FIGN,PARS2,HUNK,ACTR3B,PTPRF,IKBKB,LYVE1,APOH,CEMIP,ACVR1,PDE4B,DDX10,KIF13A,NLRP4,AK5,CPS1,ACACB,XRCC5,BMX,RAD54L2,RAB18,NRK,LRTM2,SIK1,*

			ERCC6L2,ACTR3C,SUPV3L1,MYO1B,UBE2E3,FARSB,EEFSEC,NEK7,TENM1,MYLK4,KIF2C,MYO5A,PNPLA8,FBN1,RAB31,MAP3K1,DDX4,EIF4G1,ACSM5,RBKS,ROCK1,KCNT2,LAYN,DGKG,GALK2,NIN,TPK1,PRKD1,STK4,TNNI3K,RAB6D,PDE11A,RAB6C,PCCA,GNA14,TTN,DYRK1A,ARL15,SEMA5A,ETNK1,ATP8B1
GO:0035639	purine ribonucleoside triphosphate binding	0.015639842784962436	*PFKP,STYK1,CDK14,MAP3K3,GARS1,MAP3K13,ARL13B,MYO5C,MAPK10,ATP10A,ATP7B,CAMK2B,ABCG8,MAP4K3,FPGT-TNNI3K,STK39,SRL,CDK18,FAM20B,PIK3C2B,NLK,GNAI1,MAPKAPK3,PAPOLG,UBE2O,DNAH10,CLCN3,RHOJ,NLRP8,STK32B,UBE2E2,PMS1,WRNIP1,SEPTIN10,MYO1D,ALPK2,ATRX,TNIK,TRIO,MUSK,RERG,ATP11A,ATP4A,PRKG2,RSKR,EPHA5,ATP13A4,ATP2C1,STRADA,RRAS2,KIF21A,NLRP7,PAK3,IPMK,CAMK1G,PRKAG2,CILK1,EPHA3,LATS2,MYH14,LYN,NME7,FIGNL1,NMNAT3,RNASEL,ERCC6,EIF2AK4,RAB12,TGFBR2,MARK4,DDX60L,MDN1,RRAGD,ULK3,SCG5,DAPK1,ACSM2B,SAR1B,UBE2G1,DHX35,PI4KB,RHOT1,RIPK4,PTK2,DNAH12,PIP4K2B,LRRK2,ACVR2A,GNAO1,KIT,EPHA7,PRKDC,FIGN,PARS2,HUNK,ACTR3B,IKBKB,ACVR1,DDX10,KIF13A,NLRP4,AK5,CPS1,ACACB,XRCC5,BMX,RAD54L2,RAB18,NRK,SIK1,ERCC6L2,ACTR3C,SUPV3L1,MYO1B,UBE2E3,FARSB,EEFSEC,NEK7,MYLK4,KIF2C,MYO5A,PNPLA8,RAB31,MAP3K1,DDX4,EIF4G1,ACSM5,RBKS,ROCK1,KCNT2,DGKG,GALK2,NIN,TPK1,PRKD1,STK4,TNNI3K,RAB6D,RAB6C,PCCA,GNA14,TTN,DYRK1A,ARL15,ETNK1,ATP8B1*
GO:1901265	nucleoside phosphate binding	0.019475284171291705	*PFKP,STYK1,CDK14,MAP3K3,GARS1,MAOA,MAP3K13,ARL13B,MYO5C,MAPK10,ATP10A,ATP7B,CAMK2B,ABCG8,MAP4K3,FPGT-TNNI3K,STK39,SRL,CDK18,FAM20B,PNPLA3,PIK3C2B,NLK,GNAI1,MAPKAPK3,PAPOLG,UBE2O,DNAH10,PDE10A,CLCN3,RHOJ,NLRP8,STK32B,UBE2E2,PMS1,WRNIP1,TYW1,SEPTIN10,MYO1D,ALPK2,ATRX,TNIK,TRIO,MUSK,RERG,PRKAR2A,ATP11A,ATP4A,PRKG2,RSKR,EPHA5,CBR4,ATP13A4,ATP2C1,STRADA,RRAS2,KIF21A,NLRP7,PAK3,IPMK,CAMK1G,PRKAG2,CILK1,EPHA3,LATS2,MYH14,LYN,NME7,FIGNL1,ALDH1A1,NMNAT3,FMO5,RNASEL,NOS3,ERCC6,EIF2AK4,RAB12,TGFBR2,MARK4,DDX60L,MDN1,RRAGD,ULK3,SCG5,DAPK1,ACSM2B,SAR1B,RNLS,UBE2G1,DHX35,HCN1,PI4KB,RHOT1,RIPK4,QDPR,PTK2,SCP2,DNAH12,PIP4K2B,LRRK2,ACVR2A,GNAO1,KIT,EPHA7,PRKDC,FIGN,PARS2,HUNK,ACTR3B,IKBKB,ACVR1,PDE4B,DDX10,KIF13A,NLRP4,AK5,CPS1,ACACB,XRCC5,BMX,RAD54L2,RAB18,NRK,SIK1,ERCC6L2,PIPOX,ACTR3C,SUPV3L1,MYO1B,UBE2E3,FARSB,ACBD6,EEFSEC,NEK7,MYLK4,KIF2C,MYO5A,PNPLA8,RAB31,MAP3K1,DDX4,EIF4G1,ACSM5,RBKS,ROCK1,KCNT2,DGKG,FOXRED2,GALK2,NIN,TPK1,PRKD1,STK4,TNNI3K,RAB6D,PDE11A,RAB6C,PCCA,GNA14,CTBP2,TTN,DYRK1A,TXNRD3,ARL15,ETNK1,ATP8B1,VDAC2*
GO:0003779	actin binding	0.021779715151039037	*MYBPC1,IPP,RCSD1,SPTBN1,MYO5C,CAMK2B,TMOD2,EPB41L3,PARVB,PRKN,TAGLN3,MYO1D,TMOD3,MRTFB,ABLIM1,ADD3,ENAH,GSN,PLS1,PACRG,MYH14,NOS3,PALLD,PTK2,LRRK2,CSRP3,PHACTR1,SNTG2,ANTXR1,ACTR3B,SYNE3,TRPC5,SHROOM4,MPRIP,ACTR3C,MYO1B,MYL4,CAPZA2,SCIN,AVIL,MYO5A,SPTBN5,TTN,NCALD,DYRK1A,MYRIP,FM*

			NL2
GO:0106310	protein serine kinase activity	0.022530888506393832	*CDK14,MAP3K3,MAP3K13,MAPK10,CAMK2B,MAP4K3,STK39,CDK18,NLK,MAPKAPK3,STK32B,ALPK2,TNIK,TRIO,PRKG2,RSKR,PAK3,CAMK1G,CILK1,LATS2,EIF2AK4,MARK4,ULK3,DAPK1,RIPK4,LRRK2,PRKDC,HUNK,IKBKB,NRK,SIK1,NEK7,MYLK4,MAP3K1,ROCK1,PRKD1,STK4,TNNI3K,TTN,DYRK1A*
GO:0098960	postsynaptic neurotransmitter receptor activity	0.02450712509007624	*CHRNB2,CHRFAM7A,GABRA5,CHRNA7,GRIK1,CHRM5,CHRNB3,GRID2,GABRG2,GRIA4,CHRNB4,GABRB1,GRIA3,GRIA2*
GO:1901363	heterocyclic compound binding	0.025057141781594575	*PFKP,STYK1,CDK14,MAP3K3,GARS1,MAOA,MAP3K13,ARL13B,MYO5C,MAPK10,CHRNB2,ATP10A,ATP7B,CAMK2B,ABCG8,MAP4K3,FPGT-TNNI3K,SPTLC3,STK39,SRL,CDK18,FAM20B,PNPLA3,PIK3C2B,NLK,GNAI1,MAPKAPK3,PAPOLG,UBE2O,DNAH10,PDE10A,CLCN3,RHOJ,NLRP8,STK32B,UBE2E2,PMS1,WRNIP1,TYW1,SEPTIN10,MYO1D,ALPK2,ATRX,TNIK,TRIO,MUSK,RERG,PRKAR2A,ATP11A,ATP4A,PRKG2,RSKR,EPHA5,CBR4,ATP13A4,ATP2C1,STRADA,RRAS2,KIF21A,NLRP7,PAK3,IPMK,CAMK1G,PRKAG2,CILK1,EPHA3,LATS2,MYH14,LYN,NME7,FIGNL1,ALDH1A1,NMNAT3,FMO5,RNASEL,NOS3,ERCC6,EIF2AK4,RAB12,TGFBR2,MARK4,DDX60L,MDN1,RRAGD,ULK3,GAD1,SCG5,DAPK1,ACSM2B,SAR1B,RNLS,UBE2G1,DHX35,HCN1,PI4KB,RHOT1,RIPK4,QDPR,PTK2,SCP2,DNAH12,PIP4K2B,LRRK2,RARB,ACVR2A,GNAO1,KIT,EPHA7,PRKDC,FIGN,PARS2,HUNK,ACTR3B,IKBKB,ACVR1,CHRNB3,GLDC,PDE4B,DDX10,KIF13A,NLRP4,AK5,CPS1,ACACB,XRCC5,BMX,RAD54L2,RAB18,NRK,SIK1,ERCC6L2,PIPOX,ACTR3C,SUPV3L1,MYO1B,SPTLC1,UBE2E3,FARSB,ACBD6,EEFSEC,NEK7,MYLK4,KIF2C,MYO5A,PNPLA8,RAB31,MAP3K1,DDX4,EIF4G1,ACSM5,RBKS,ROCK1,KCNT2,DGKG,FOXRED2,GALK2,NIN,TPK1,PRKD1,STK4,TNNI3K,RAB6D,PDE11A,RAB6C,PCCA,GNA14,CTBP2,TTN,DYRK1A,TXNRD3,ARL15,ETNK1,TTPA,ATP8B1,VDAC2*
GO:0004674	protein serine/threonine kinase activity	0.02550897727640399	*CDK14,MAP3K3,ABR,MAP3K13,MAPK10,BRD4,CAMK2B,MAP4K3,STK39,CDK18,NLK,MAPKAPK3,STK32B,ALPK2,TNIK,TRIO,PRKG2,RSKR,PAK3,CAMK1G,PRKAG2,CILK1,LATS2,EIF2AK4,TGFBR2,MARK4,ULK3,DAPK1,RIPK4,LRRK2,ACVR2A,PRKDC,HUNK,IKBKB,ACVR1,NRK,SIK1,NEK7,MYLK4,MAP3K1,ROCK1,PRKD1,STK4,TNNI3K,TTN,DYRK1A*
GO:0015276	ligand-gated monoatomic ion channel activity	0.030277114185069853	*CHRNB2,KCNJ6,CHRFAM7A,GABRA5,ANO3,CHRNA7,KCNJ18,ITPR1,GRIK1,HCN1,KCNJ12,CHRNB3,GRID2,KCNJ3,GABRB3,GABRG2,GRIA4,RYR3,CHRNB4,GABRB1,GRIA3,KCNT2,KCNE2,KCNN4,GRIA2*
GO:0005230	extracellular ligand-gated monoatomic ion channel activity	0.033620350990259965	*CHRNB2,CHRFAM7A,GABRA5,CHRNA7,GRIK1,CHRNB3,GRID2,GABRB3,GABRG2,GRIA4,CHRNB4,GABRB1,GRIA3,GRIA2*
GO:0022834	ligand-gated channel	0.03614493530844068	*CHRNB2,KCNJ6,CHRFAM7A,GABRA5,ANO3,CHRNA7,KCNJ18,ITPR1,GRIK1,HCN1,KCNJ12,CHRNB3,GRID2,KCNJ3,GABRB3,GABRG2,GRIA4,RYR3,CHRNB4,GABRB1,GRIA3,KCNT2,KCNE2,KCNN4,GRIA2*

	activity		
GO:0000166	nucleotide binding	0.03849725935478797	*PFKP,STYK1,CDK14,MAP3K3,GARS1,MAOA,MAP3K13,ARL13B,MYO5C,MAPK10,ATP10A,ATP7B,CAMK2B,ABCG8,MAP4K3,FPGT-TNNI3K,STK39,SRL,CDK18,FAM20B,PIK3C2B,NLK,GNAI1,MAPKAPK3,PAPOLG,UBE2O,DNAH10,PDE10A,CLCN3,RHOJ,NLRP8,STK32B,UBE2E2,PMS1,WRNIP1,TYW1,SEPTIN10,MYO1D,ALPK2,ATRX,TNIK,TRIO,MUSK,RERG,PRKAR2A,ATP11A,ATP4A,PRKG2,RSKR,EPHA5,CBR4,ATP13A4,ATP2C1,STRADA,RRAS2,KIF21A,NLRP7,PAK3,IPMK,CAMK1G,PRKAG2,CILK1,EPHA3,LATS2,MYH14,LYN,NME7,FIGNL1,ALDH1A1,NMNAT3,FMO5,RNASEL,NOS3,ERCC6,EIF2AK4,RAB12,TGFBR2,MARK4,DDX60L,MDN1,RRAGD,ULK3,SCG5,DAPK1,ACSM2B,SAR1B,RNLS,UBE2G1,DHX35,HCN1,PI4KB,RHOT1,RIPK4,QDPR,PTK2,DNAH12,PIP4K2B,LRRK2,ACVR2A,GNAO1,KIT,EPHA7,PRKDC,FIGN,PARS2,HUNK,ACTR3B,IKBKB,ACVR1,PDE4B,DDX10,KIF13A,NLRP4,AK5,CPS1,ACACB,XRCC5,BMX,RAD54L2,RAB18,NRK,SIK1,ERCC6L2,PIPOX,ACTR3C,SUPV3L1,MYO1B,UBE2E3,FARSB,EEFSEC,NEK7,MYLK4,KIF2C,MYO5A,PNPLA8,RAB31,MAP3K1,DDX4,EIF4G1,ACSM5,RBKS,ROCK1,KCNT2,DGKG,FOXRED2,GALK2,NIN,TPK1,PRKD1,STK4,TNNI3K,RAB6D,PDE11A,RAB6C,PCCA,GNA14,CTBP2,TTN,DYRK1A,TXNRD3,ARL15,ETNK1,ATP8B1,VDAC2*
GO:0016772	transferase activity, transferring phosphorus-containing groups	0.04060528428460517	*PFKP,STYK1,CDK14,MAP3K3,ABR,MAP3K13,MAPK10,BRD4,CAMK2B,MAP4K3,FPGT-TNNI3K,STK39,CDK18,FAM20B,PIK3C2B,NLK,MAPKAPK3,PAPOLG,STK32B,ALPK2,TNIK,TRIO,MUSK,PRKAR2A,PRKG2,RSKR,EPHA5,STRADA,PAK3,IPMK,CAMK1G,PRKAG2,OASL,CILK1,EPHA3,LATS2,LYN,NME7,NMNAT3,RNASEL,SGMS2,EIF2AK4,TGFBR2,MARK4,POLR2C,ULK3,GKAP1,DAPK1,PI4KB,PIK3R5,RIPK4,PTK2,PIP4K2B,LRRK2,TENT5C,ACVR2A,KIT,EPHA7,PRKDC,HUNK,IKBKB,ACVR1,AK5,BMX,POLD3,NRK,SIK1,NEK7,SH3KBP1,MYLK4,MAP3K1,RBKS,ROCK1,DGKG,GALK2,TPK1,PRKD1,STK4,TNNI3K,TTN,DYRK1A,ETNK1,UAP1*
GO:0019899	enzyme binding	0.0420856354029582	*STX8,SMYD3,RAPGEF6,MAP3K13,THRB,SPTBN1,CDC42EP3,ATF7,SH3PXD2A,BRD4,XPO4,NOTCH2,NR4A3,FOXO1,STYXL1,YBX1,ARMT1,ASB4,PRKN,XPO6,FAM83B,NLK,MAPKAPK3,EIF4ENIF1,CD247,CEP192,RHOJ,CDC37,PLG,NCK1,MTCP1,PREX1,PMS1,COL1A2,XPO7,HMGB1,ADD3,SFPQ,PRKAR2A,GOLPH3,PRKG2,NCF2,SKOR2,SNX10,STRADA,CHL1,GSN,MAPRE3,CACUL1,NLRP7,CD226,PAK3,NDUFAF7,PRKAG2,CCNT2,NCKAP1,PRPF40A,PACRG,CHCHD3,SPDYE2B,MYOM2,LYN,SUMO2,MTA3,LDB2,DBF4B,TCF7L2,ZFHX3,RRAGD,SPDYE2,KPNB1,WAC,APP,GATA4,SMAD5,GSKIP,UBE2G1,AVPR1B,PTK2,TICAM2,SH2B2,LRRK2,IKZF3,ARIH1,UBXN7,PJA2,TIAM1,PRMT8,RABGEF1,KIT,SPAG9,SPDL1,PEX26,CTNNB1,PRKDC,IPO5,ESR1,PTPN14,BCAR3,DOCK1,TRPC5,IKBKB,AKAP6,RAB3GAP2,ATF2,ACVR1,RB1,ADIPOR1,SMG7,LAMP2,RABGAP1L,XRCC5,MID2,SIK1,ERCC6L2,KAT2B,MEF2D,CDK5RAP2,ARHGEF7,SRI,ATP1B1,STIM1,RAB11FIP2,PPARG,JAKMIP1,SH3KBP1,USP25,MYO5A,SPDYE16,TFRC,SPDYE6,HAPSTR1,MAP3K1,IQCB1,CLEC6A,CLASP2,RPRD2,ANGPT1,ATP6V0A4,ROCK1,TAX1BP1,PML,SP100,SBF2,TTC28,NIN,ANKFY1,ENSA,AIMP1,PRKD1,HSPBP1,PRLR,PCCA,CTBP2,TTN,*

			MYRIP,CUL4A,FMNL2,KCNN4,SPART
GO:0003824	catalytic activity	0.046725030642384695	C10ORF90,PFKP,STYK1,HS3ST3A1,RGN,CDK14,MSRB3,PDE1C,ING3,MAP3K3,WSCD1,SMYD3,GARS1,ABR,MAOA,MAP3K13,PPM1A,ARL13B,IMMP1L,CHST11,USP42,MAPK10,USP32,AHCYL2,CDC14C,AGTPBP1,ATP10A,PIGL,ATP7B,PPM1H,BRD4,CAMK2B,DDI2,PGM1,DCT,PECR,SDR16C5,ABCG8,OTUD7A,MAP4K3,GALNT9,FPGT-TNNI3K,FKBP5,SPTLC3,STYXL1,STK39,SMURF2,ADAMTS12,CDK18,ARMT1,FAM20B,ASB4,IDO1,PRKN,RMND5A,PNPLA3,F13A1,PIK3C2B,KDM7A,MARCHF9,CBLL2,NLK,GNAI1,MAPKAPK3,PAPOLG,AGL,ENPEP,NUDT19,UBE2O,MBTPS2,ADAR,DNAH10,PDE10A,PDHX,RHOJ,MECOM,STK32B,PLG,NEURL4,HERC5,NUDT13,PGPEP1L,UBE2E2,PUDP,KLHL42,EXO1,KDM2B,AIG1,PMS1,WRNIP1,TYW1,RSF1,SEPTIN10,MARCHF1,EEF2KMT,ALPK2,HECW1,HMGB1,ATRX,TNIK,TRIO,NDST1,MUSK,PNPLA4,ALKBH1,RGS3,ZCCHC4,RERG,PRKAR2A,ATP11A,ATP4A,PRKG2,MBTPS1,NCF2,PIGV,USP10,SND1,RSKR,EPHA5,PTPN20,CBR4,ATP13A4,ATP2C1,ALDH1A2,PPTC7,STRADA,HUWE1,ASPH,RRAS2,ZDHHC4,UBR5,KIF21A,CPM,PAK3,NDUFAF7,IPMK,CYP2A13,CAMK1G,PRUNE2,PRKAG2,CPQ,F8,OASL,GDPD1,CPT2,DTD1,MGAM,CILK1,IL1RAPL2,EPHA3,LATS2,TYW3,LYN,USP6,SUMO2,NME7,ATP6V1H,FIGNL1,PAMR1,CA10,RRM2,ALDH1A1,NMNAT3,FMO5,RNASEL,NAV1,CORIN,DCP2,SGMS2,NOS3,BPHL,ERCC6,EIF2AK4,MSRA,RAB12,HMGCLL1,MAGEL2,TGFBR2,MARK4,DDX60L,MDN1,SYNJ1,ECHDC2,OVCH1,POLR2C,RRAGD,ULK3,GKAP1,ADGRV1,QTRT2,GAD1,DAPK1,TDP2,ALDH9A1,ACSM2B,PRUNE1,SAR1B,PPIL6,RNLS,RNF38,ADAM12,UBE2G1,PELI2,DHX35,CHRM5,PI4KB,RHOT1,ELOVL5,PIK3R5,LYZL1,TLR1,CECR2,BMT2,RIPK4,QDPR,FRRS1,PTK2,USP7,SCP2,DNAH12,PIP4K2B,LRRK2,ARIH1,PLD1,TENT5C,PJA2,MGST1,COP1,ACVR2A,GNAO1,NEDD4L,BRCC3,DUSP14,PRMT8,RABGEF1,KIT,CYB5R4,EPHA7,TRABD2B,CTSK,PRKDC,AGAP4,PTPN14,POFUT2,DMGDH,PPM1E,TPST2,FIGN,PARS2,HUNK,TMEM62,PDE7B,USP40,HDDC2,BPGM,CDC14B,PLCH1,HECTD1,VKORC1L1,AASS,KAT14,UBE3B,PTPRF,IKBKB,CEMIP,ATF2,HDAC8,ACVR1,HYAL4,GLDC,PLPPR1,NSMCE1,PDE4B,DDX10,KIF13A,PLPP3,IMMP2L,AK5,CPS1,ACACB,AGPAT5,XRCC5,RGS17,INPP4A,BMX,RAD54L2,TPST1,RAB18,POLD3,TPH2,TMTC1,NRK,MGRN1,MID2,SIK1,ERCC6L2,PIPOX,KAT2B,TMTC2,TMPRSS11B,PRCP,SUPV3L1,MGAM2,SPTLC1,UBE2E3,RFC3,FARSB,ADAT2,TLR6,NDST4,ADAMTS9,TSEN15,EEFSEC,PAPPA,AGAP9,TGM5,NEK7,SH3KBP1,SMOX,LPCAT2,AMY2A,MYLK4,USP25,KIF2C,IL1RAP,PNPLA8,RAB31,MAP3K1,DDX4,FUT10,TMPRSS15,B3GAT2,ACSM5,PHLPP1,RBKS,DNAJC6,ROCK1,APIP,MMP28,NUP98,PML,MGLL,LONRF3,DGKG,FOXRED2,RFPL4B,TSEN2,GALK2,PRSS50,TPK1,PRKD1,USP34,STK4,TNNI3K,RAB6D,RNASE11,ST6GAL2,ANPEP,PDE11A,RAB6C,PCCA,GNA14,OXNAD1,CTBP2,TTN,DYRK1A,RGS10,CUL4A,CHSY3,TXNRD3,EYA4,ARL15,UBE3C,ATE1,ETNK1,CHAC2,METTL3,UAP1,CP,ATP8B1,PGPEP1,RNF6,SMYD1,PEPD,DPY19L1
BP			
GO:0048	system	8.564589750	APBB2,RTN1,HS3ST3A1,RGN,MAP3K3,LRRN1,ARHGEF9,ISM1,TRAPPC9,MAP3K13,ARL13B,CHST11,

731	development	701057e-10	*THRB,SPTBN1,CHRNB2,SEZ6,GNB4,AGTPBP1,SPINK5,DOK4,CAMK2B,GRIP1,DCT,NOTCH2,TMOD2,SDK2,NPNT,EPB41L3,MAP6,CDH11,SHANK3,ARID1B,E2F7,FOXO1,STYXL1,ADAMTS12,PLXNA4,DIPK2A,CACNA1C,ASB4,IDO1,PRKN,NLGN4X,TAFA1,KDM7A,IFT172,GPSM1,CDON,SERPINI1,SLC17A6,THSD7A,NCAM2,TIAM2,TRAK2,CNMD,LRP8,TAGLN3,CNTN1,MEOX2,STAT5B,ENPEP,EIF4ENIF1,MBTPS2,PTH,ADAR,RHOJ,FGF10,TSPAN18,PLG,NCK1,NREP,EXO1,GABRA5,NEXMIF,ASTN1,KDM2B,PREX1,COL11A1,MCPH1,COL1A2,ALPK2,CHRNA7,HECW1,HMGB1,ZNF304,RFLNA,ATRX,TNIK,TGFB2,LAMA2,TRIO,SMARCC1,NDST1,MUSK,JAM3,ALKBH1,SLC15A4,PCDHB8,PRKG2,EPHA5,SKOR2,ENAH,SNX10,ALDH1A2,RRAS2,SIM1,KIAA1217,CHL1,GSN,PLS1,SLIT3,ANKRD11,DNAAF11,PAK3,ABI3BP,BTG4,TNFRSF12A,CXCR5,NCKAP1,FOSL2,IL1RAPL2,EPHA3,MYOM2,LYN,ADGRB3,KLF7,GRIK1,DIP2B,CA10,CRYBB1,LDB2,NAV1,PCDHB16,NOS3,VSX1,ERCC6,EIF2AK4,MATN2,TGFBR2,MARK4,TCF7L2,CRTAC1,ZFHX3,CUX1,UNC119,GPM6B,ADGRV1,EPB41L5,WHRN,TDP2,PRUNE1,APP,GATA4,SMAD5,IGF2BP1,MAMLD1,RNF38,ADAM12,HCN1,FRY,HOOK3,PALLD,CECR2,PTK2,SH2B2,CELF1,HYCC1,LRRK2,CLSTN2,PKHD1,RARB,TCF4,TIAM1,CSRP3,PHACTR1,ACVR2A,VIT,SNTG2,NTM,NEDD4L,KIT,EPHA7,SPAG9,ANTXR1,WNT5A,CTNNB1,ANKRD17,COL19A1,CTSK,PRKDC,ESR1,PTPN14,BCAR3,LMX1A,PTGER4,GREB1L,BBX,VWC2L,GPC4,BPGM,EFNA5,IGF2BP2,HECTD1,NGEF,TRPC5,NEUROD2,PTPRF,FXR2,AKAP6,APOH,ARHGEF28,GPRIN1,OPCML,LINGO2,ATF2,ACVR1,GRID2,ZNF423,PLPPR1,RB1,MPV17,PKD1L3,GABRB3,SHROOM4,PLPP3,HNF1B,IMMP2L,KIRREL3,GABRG2,CPS1,ACACB,XRCC5,SHLD2,GFRA1,RAB18,ETV1,TEX11,LRTM2,SIK1,KAT2B,WLS,ANKS1A,PRCP,MEF2D,CDK5RAP2,FLVCR1,ARHGEF7,NPHP1,SRI,BDNF,TFCP2L1,SEMA3E,SCIN,MACO1,ADAMTS9,STIM1,PPARG,TENM1,RORB,GABRB1,AVIL,SIPA1L3,SFRP4,IL1RAP,JCAD,VAV2,TFRC,VPS13A,FBN1,BMPER,FUT10,CLASP2,ZNF521,ANGPT1,SEMA6A,EIF4G1,PHLPP1,ITGA4,ROCK1,CLEC3A,PML,TNFSF4,DGKG,SP100,ELAVL4,SBF2,CDH9,UMODL1,NIN,FNDC3A,AIMP1,PRKD1,CALCRL,STK4,PCDH10,ANPEP,PRLR,TTN,HHIP,DYRK1A,TMEM30A,SEMA5A,LAMA4,TTPA,METTL3,PARD3,ATP8B1,RNF6,SEMA3C,SMYD1,VPS13B,SPART*
GO:0007275	multicellular organism development	1.1755215564883352e-8	*APBB2,RTN1,HS3ST3A1,RGN,MAP3K3,LRRN1,ARHGEF9,ISM1,TRAPPC9,MAP3K13,ARL13B,CHST11,THRB,SPTBN1,CHRNB2,SEZ6,GNB4,AGTPBP1,SH3PXD2A,SPINK5,BRD4,DOK4,CAMK2B,GRIP1,DCT,NOTCH2,TMOD2,SDK2,NPNT,EPB41L3,MAP6,CDH11,SHANK3,NR4A3,ARID1B,E2F7,FOXO1,STYXL1,SMURF2,ADAMTS12,PLXNA4,YBX1,DIPK2A,CACNA1C,ASB4,IDO1,PRKN,NLGN4X,TAFA1,KDM7A,SLC5A7,IFT172,GPSM1,CDON,SERPINI1,SLC17A6,THSD7A,NCAM2,TIAM2,TRAK2,CNMD,LRP8,TAGLN3,CNTN1,MEOX2,STAT5B,ENPEP,EIF4ENIF1,MBTPS2,PTH,ADAR,RHOJ,FGF10,TSPAN18,PLG,NCK1,NREP,EXO1,GABRA5,NEXMIF,ASTN1,KDM2B,PREX1,COL11A1,MCPH1,COL1A2,COL12A1,ALPK2,CHRNA7,HECW1,HMGB1,ZNF304,AFF3,RFLNA,ATRX,TNIK,TGFB2,LAMA2,TRIO,HOXC13,SMARCC1,NDST1,MUSK,JAM3,ALKBH1,ATP11A,SLC15A4,PCDHB8*

			,*PRKG2,IGFBP7,EPHA5,SKOR2,ENAH,SNX10,ALDH1A2,ASPH,RRAS2,SIM1,KIAA1217,CHL1,GSN,PLS1,SLIT3,ANKRD11,ITPR1,DNAAF11,PAK3,ABI3BP,MLLT3,BTG4,TNFRSF12A,CPT2,CXCR5,NCKAP1,FOSL2,IL1RAPL2,EPHA3,LATS2,MYOM2,LYN,ADGRB3,KLF7,GRIK1,DIP2B,SEC24C,CA10,RRM2,CRYBB1,LDB2,NAV1,PCDHB16,SGMS2,NOS3,VSX1,ERCC6,EIF2AK4,MATN2,TGFBR2,MARK4,TCF7L2,CRTAC1,ZFHX3,CUX1,UNC119,GPM6B,ADGRV1,EPB41L5,WHRN,TDP2,PRUNE1,APP,GATA4,SMAD5,IGF2BP1,MAMLD1,RNF38,ADAM12,DHX35,HCN1,FRY,HOOK3,PALLD,CECR2,PTK2,SH2B2,CELF1,HYCC1,LRRK2,INO80D,IKZF3,CLSTN2,PKHD1,TENT5C,RARB,TCF4,TIAM1,CSRP3,PHACTR1,ACVR2A,VIT,SNTG2,NTM,NEDD4L,KIT,EPHA7,SPAG9,ANTXR1,WNT5A,CTNNB1,ANKRD17,COL19A1,CTSK,PRKDC,ESR1,PTPN14,POFUT2,BCAR3,LMX1A,PTGER4,GREB1L,BBX,VWC2L,GPC4,BPGM,EFNA5,IGF2BP2,HECTD1,NGEF,TRPC5,NEUROD2,KAT14,PTPRF,IKBKB,FXR2,AKAP6,APOH,ARHGEF28,TASOR,GPRIN1,OPCML,LINGO2,ATF2,ACVR1,GRID2,ZNF423,PLPPR1,RB1,MPV17,PKD1L3,GABRB3,SHROOM4,PLPP3,HNF1B,IMMP2L,KIRREL3,GABRG2,CPS1,ACACB,XRCC5,SHLD2,GFRA1,RAB18,ETV1,TEX11,LRTM2,SIK1,KAT2B,WLS,ANKS1A,PRCP,MEF2D,CDK5RAP2,FLVCR1,ARHGEF7,DACH1,NPHP1,SRI,ARNT,BDNF,TFCP2L1,SEMA3E,SCIN,MACO1,ADAMTS9,STIM1,LAG3,PPARG,TENM1,RORB,GABRB1,AVIL,TTC39C,SIPA1L3,SFRP4,BLTP1,IL1RAP,JCAD,VAV2,TFRC,VPS13A,FBN1,BMPER,FUT10,CLASP2,ZNF521,ANGPT1,SEMA6A,EIF4G1,PHLPP1,ITGA4,ROCK1,CLEC3A,PML,TNFSF4,DGKG,SP100,ELAVL4,SBF2,CDH9,UMODL1,NIN,FNDC3A,AIMP1,PRKD1,CALCRL,SLC20A2,STK4,PCDH10,ANPEP,PRLR,LPAR6,TTN,HHIP,DYRK1A,TMEM30A,CUL4A,EYA4,SEMA5A,LAMA4,TTPA,METTL3,PARD3,ATP8B1,RNF6,SEMA3C,SMYD1,VPS13B,SPART*
GO:0048856	anatomical structure development	1.1896738069959778e-7	*FSTL1,APBB2,RTN1,HS3ST3A1,RGN,MAP3K3,LRRN1,SMYD3,ARHGEF9,ISM1,TRAPPC9,MAP3K13,ARL13B,LIMD1,TF,CHST11,THRB,SPTBN1,CDC42EP3,CHRNB2,SEZ6,CDH8,GNB4,AGTPBP1,FHL1,ATP10A,SH3PXD2A,SPINK5,ATP7B,BRD4,DOK4,CAMK2B,GRIP1,DCT,NOTCH2,TMOD2,SDK2,NPNT,EPB41L3,MAP6,PARVB,CDH11,SHANK3,NR4A3,ARID1B,E2F7,FOXO1,STYXL1,SMURF2,ADAMTS12,PLXNA4,YBX1,DIPK2A,CACNA1C,ASB4,IDO1,ARB2A,PRKN,NLGN4X,TAFA1,KDM7A,SLC5A7,IFT172,GPSM1,PTBP3,CDON,SERPINI1,SLC17A6,THSD7A,NCAM2,TIAM2,CFAP54,TRAK2,CNMD,LRP8,TAGLN3,CNTN1,MEOX2,STAT5B,ENPEP,EIF4ENIF1,HEATR9,MBTPS2,PTH,ADAR,RHOJ,FGF10,MECOM,TSPAN18,PLG,CDH7,ILDR2,NCK1,NREP,EXO1,GABRA5,NEXMIF,ASTN1,KDM2B,PREX1,CDIN1,COL11A1,MCPH1,COL1A2,TMOD3,MRTFB,COL12A1,ABLIM1,ALPK2,CHRNA7,HECW1,HMGB1,ZNF304,AFF3,RFLNA,ATRX,TNIK,TGFB2,LAMA2,TRIO,HOXC13,SMARCC1,NDST1,MUSK,JAM3,ALKBH1,ATP11A,SLC15A4,PCDHB8,PRKG2,IGFBP7,EPHA5,SKOR2,ENAH,SNX10,ATP2C1,ALDH1A2,FAM171A1,HUWE1,ASPH,RRAS2,SIM1,KIAA1217,SOX30,CHL1,GSN,PLS1,SLIT3,ANKRD11,ITPR1,CPM,DNAAF11,PAK3,ABI3BP,AXDND1,MLLT3,CCNT2,BTG4,CPQ,MR1,TNFRSF12A,CPT2,CXCR5,NCKAP1,PRPF40A,FOSL2,PACRG,IL1RAPL2,EPHA3,LATS2,MYH14,ARHGAP18,M*

			YOM2,LYN,ADGRB3,KLF7,GRIK1,DIP2B,SEC24C,PDZD8,CA10,RRM2,CRYBB1,LDB2,NAV1,PAQR5,PCDHB16,SGMS2,NOS3,VSX1,ERCC6,EIF2AK4,XKR6,MATN2,TGFBR2,ITGA11,MARK4,TCF7L2,CRTAC1,ZFHX3,CUX1,UNC119,GPM6B,ADGRV1,EPB41L5,WHRN,OCA2,TDP2,PRUNE1,APP,GATA4,SMAD5,IGF2BP1,MAMLD1,RNF38,ADAM12,DHX35,HCN1,PI4KB,FRY,HOOK3,PALLD,CECR2,RIPK4,PTK2,SH2B2,CELF1,HYCC1,LRRK2,INO80D,IKZF3,CLSTN2,ITFG2,PKHD1,TENT5C,RARB,TCF4,PAEP,TIAM1,CSRP3,PHACTR1,ACVR2A,VIT,SNTG2,NTM,NEDD4L,KIT,CYB5R4,EPHA7,SPAG9,ANTXR1,WNT5A,CTNNB1,ANKRD17,COL19A1,CTSK,PRKDC,ESR1,TNFRSF19,PTPN14,SCFD1,POFUT2,BCAR3,LMX1A,PTGER4,GREB1L,BBX,VWC2L,DOCK1,GPC4,BPGM,EFNA5,IGF2BP2,SYNE3,HECTD1,NGEF,TRPC5,NEUROD2,KAT14,PTPRF,IKBKB,FXR2,AKAP6,USF3,LYVE1,APOH,ARHGEF28,TASOR,GPRIN1,OPCML,LINGO2,ATF2,ACVR1,PID1,EBF2,GRID2,ZNF423,PLPPR1,RB1,MPV17,DDX10,PKD1L3,ADIPOR1,GABRB3,SHROOM4,PLPP3,HNF1B,IMMP2L,KIRREL3,GABRG2,CPS1,NCOA2,ACACB,AGPAT5,XRCC5,SHLD2,GFRA1,BMX,SLC24A3,RAB18,ETV1,TEX11,LRTM2,SIK1,KAT2B,WLS,ANKS1A,PRCP,MEF2D,CDK5RAP2,FLVCR1,ARHGEF7,DACH1,ATF3,NPHP1,IKZF1,SRI,ARNT,BDNF,LDLRAD4,TFCP2L1,SEMA3E,SCIN,MACO1,ADAMTS9,STIM1,LAG3,PPARG,TANC1,TGM5,TENM1,RORB,GABRB1,SH3KBP1,AVIL,TTC39C,SIPA1L3,SFRP4,BLTP1,IL1RAP,JCAD,VAV2,TFRC,VPS13A,FBN1,IQCB1,BMPER,FUT10,CLASP2,ZNF521,ANGPT1,SEMA6A,EIF4G1,PHLPP1,ITGA4,ROCK1,CLEC3A,PML,TNFSF4,DGKG,SP100,ELAVL4,SBF2,CDH9,UMODL1,NIN,FNDC3A,AIMP1,PRKD1,CALCRL,SLC20A2,STK4,PCDH10,ANPEP,PRLR,CR2,LPAR6,TTN,HHIP,DYRK1A,TMEM30A,KCNE2,CUL4A,EYA4,SEMA5A,LAMA4,TTPA,FMNL2,METTL3,KRT85,PARD3,ATP8B1,RNF6,SEMA3C,VAV1,SMYD1,VPS13B,SPART
GO:0065007	biological regulation	1.68173859078962e-7	FSTL1,C10ORF90,APBB2,STYK1,RTN1,RGN,CDK14,PDE1C,NOSTRIN,ING3,MAP3K3,OSBPL8,SLC12A8,LRRN1,STX8,PHF20,FOXK2,SMYD3,RAPGEF6,ARHGEF9,TAFA2,ABR,ISM1,MAOA,MAP3K13,PPM1A,ARL13B,SAMSN1,LIMD1,TF,CHST11,THRB,USP42,SPTBN1,CDC42EP3,MAPK10,CACNB1,USP32,DAPP1,ATF7,GNG12,CHRNB2,CDC14C,SEZ6,CDH8,KCNQ5,GNB4,AGTPBP1,ATF7-NPFF,FHL1,RBPMS,ATP10A,SPINK5,OPA3,SKAP2,ATP7B,GPBP1L1,KCNJ6,BRD4,DOK4,CAMK2B,GRIP1,ARHGAP11A-SCG5,DDI2,DCT,DLGAP1,SLC38A9,ZNF264,HBP1,SDR16C5,ABCG8,XPO4,MAP4K3,NOTCH2,TMOD2,HMBOX1,ZNF551,NPNT,EPB41L3,MAP6,MORC1,PARVB,CDH11,SHANK3,NR4A3,ARID1B,E2F7,FOXO1,ZNF611,STYXL1,STK39,SMURF2,ADAMTS12,FER1L6,TBC1D16,PLXNA4,CDK18,URB2,YBX1,DIPK2A,DCDC1,CACNA1C,FAM3C,ASB4,IDO1,ARB2A,PRKN,NLGN4X,F13A1,TAFA1,PIK3C2B,KDM7A,SLC5A7,FAM83B,IFT172,GPSM1,CBLL2,PTBP3,CDON,SERPINI1,SLC17A6,NLK,GNAI1,MRPL13,MAPKAPK3,TIAM2,ZNF10,CNMD,LRP8,TAGLN3,CNTN1,MEOX2,STAT5B,ENPEP,ARHGEF4,UBE2O,DDIAS,CPEB4,EIF4ENIF1,CHRFAM7A,ERBIN,ZNF121,MBTPS2,RGS22,CD247,MCC,PTH,ADAR,PDE10A,TM9SF2,CLCN3,RHOJ,CDC37,FGF10,ZFYVE16,MECOM,CO

			L4A6,TSPAN18,NLRP8,STK32B,PLG,HERC5,UBE2E2,ILDR2,NCK1,CD84,FAM168A,NONO,RARRES1,KLHL42,NREP,GABRA5,NEXMIF,MTCP1,KDM2B,PREX1,ZNF404,WRNIP1,ANO3,RSF1,NCOA7,CIDEB,MCPH1,SESTD1,ZNF214,COL1A2,DTNA,TMOD3,GEMIN5,MRTFB,ALPK2,CHRNA7,HECW1,HMGB1,ZNF304,DYNLT2B,CDIP1,AFF3,RFLNA,HIVEP1,ATRX,TNIK,TGFB2,MDFIC,LAMA2,ADD3,TRIO,ZNF85,HOXC13,SMARCC1,NDST1,MUSK,KCNJ18,JAM3,KCNIP3,SFPQ,PNPLA4,ALKBH1,RGS3,NUP93,ZCCHC4,RERG,OR5AU1,PRKAR2A,RBM33,CTIF,ATP11A,RBM23,ZNF154,ATP4A,SLC15A4,GOLPH3,PRKG2,MBTPS1,USP10,SND1,IGFBP7,EPHA5,SKOR2,ATP2C1,ALDH1A2,PPTC7,STON2,PTGFR,FAM171A1,STRADA,HUWE1,ZNF595,ASPH,RRAS2,SH3BGRL,UBR5,GTF2F2,SIM1,SOX30,ANKS1B,CHL1,SPOCK3,GSN,PLS1,TCP11L1,SPON1,MAPRE3,TLR8,SLIT3,ARMH3,CACUL1,NLRP7,ARHGEF39,CD226,PHC2,ZNF578,ITPR1,PKP4,PAK3,ABI3BP,KLF12,TMEM183A,PRKAG2,MLLT3,CCNT2,BTG4,HAVCR1,CPQ,CNOT10,MR1,F8,TNFRSF12A,OASL,CPT2,TAS2R14,VPS8,ZNF429,CXCR5,NCKAP1,PRPF40A,DTD1,CILK1,FOSL2,IL1RAPL2,ADGRD1,EPHA3,LATS2,MYH14,SPDYE2B,ERP29,CDYL2,ARHGAP18,UVRAG,LYN,USP6,ADGRB3,KLF7,SUMO2,GRIK1,MTA3,DIP2B,NME7,ATP6V1H,NUP58,FIGNL1,SLC12A1,PDZD8,RRM2,LDB2,MYRFL,ALDH1A1,FMO5,RNASEL,DBF4B,CORIN,PCDHB16,DCP2,SGMS2,TMED7-TICAM2,NOS3,VSX1,ZNF280B,TNRC6C,PRAMEF27,MLLT10,ERCC6,EIF2AK4,KIAA1614,RAB12,XKR6,MAGEL2,TGFBR2,ITGA11,CMKLR2,MARK4,SYNJ1,TCF7L2,ZNF462,E2F6,ZFHX3,CUX1,UNC119,SHISA2,GPM6B,RRAGD,ULK3,SPDYE2,GPR156,GKAP1,PPP2R5E,GSDMD,ADGRV1,ST7,RASGRF2,SERBP1,EPB41L5,GAD1,SCG5,WAC,WHRN,OCA2,DAPK1,TDP2,ZNF678,PRUNE1,SAR1B,INSYN2A,BTG3,APP,GATA4,ATF7IP2,SMAD5,IGF2BP1,MAMLD1,RNLS,GSKIP,ADAM12,PELI2,SP140L,HCN1,CHRM5,PI4KB,FRY,RHOT1,ELOVL5,HOOK3,PIK3R5,TLR1,TAS2R30,BMT2,RIPK4,AVPR1B,PTK2,USP7,TICAM2,SCP2,SH2B2,ATP1B4,AVEN,ZNF41,CELF1,POGZ,PIP4K2B,LRRK2,INO80D,ZNF395,IKZF3,CLSTN2,ARIH1,CFAP298,PLD1,VTCN1,ITFG2,PKHD1,TENT5C,RARB,PJA2,TCF4,PAEP,COP1,TIAM1,CSRP3,PHACTR1,MLIP,IQSEC2,ACVR2A,GNAO1,ASAP2,TIPIN,VIT,RASSF4,NEDD4L,BRCC3,SNX13,RABGEF1,KIT,CYB5R4,TBC1D8,EPHA7,SPAG9,ANTXR1,SPDL1,WNT5A,TRABD2B,TEX2,ARFGEF2,CTNNB1,ANKRD17,RUNX1T1,CTSK,PRKDC,IPO5,ESR1,TNFRSF19,ZNF479,PTPN14,SCFD1,ERC2,POFUT2,BCAR3,TPD52L1,L3MBTL4,PPM1E,ZXDC,FRMD4A,LMX1A,BNIPL,HUNK,PTGER4,ZNF215,PDE7B,BBX,VWC2L,BEST3,DOCK1,GPC4,PKIG,EFNA5,IGF2BP2,CDC14B,ZNF722,SYNE3,PLCH1,OR1M1,HECTD1,NGEF,TRPC5,NEUROD2,FNIP2,AASS,JAML,KAT14,PTPRF,IKBKB,FXR2,SH3BP5,AKAP6,TRBV10-2,USF3,LYVE1,APOH,ARHGEF28,RAB3GAP2,CLCN1,TASOR,CCPG1,LINGO2,CEMIP,XIST,ATF2,KCNJ12,ZNF426,HDAC8,ACVR1,CHRNB3,HLA-DQA2,PID1,EBF2,GRID2,ZNF423,ZCCHC17,KCNJ3,PLPPR1,RB1,NSMCE1,MPV17,IFNLR1,PDE4B,KIF13A,NLRP4,ADIPOR1,GABRB3,ZNF143,ZSCAN23,SMG7,PLPP3,HNF1B,ZNF615,LAMP2,GABRG2,F

			5,CPS1,NCOA2,BNIP3L,ACACB,RABGAP1L,XRCC5,TBRG4,RGS17,SHLD2,GFRA1,KCND2,INPP4A,BMX,ZNF418,SLC24A3,TAF15,GRIA4,RAB18,ZNF860,POU2F1,ETV1,RGS7BP,BACH2,MGRN1,TEX11,MID2,MIR198,LRTM2,SIK1,ZNF850,TP53BP2,KAT2B,GOLPH3L,WLS,ANKS1A,RYR3,PRCP,SUPV3L1,MEF2D,MYL4,GTF2A1,SPTLC1,CDK5RAP2,FLVCR1,RFC3,ARHGEF7,SAMD12,DACH1,ATF3,TRDN,NPHP1,IKZF1,CAPZA2,SRI,ATP1B1,ARNT,BDNF,LDLRAD4,TFCP2L1,SEMA3E,BCLAF3,SCIN,MACO1,ZNF665,TLR6,ADAMTS9,STIM1,TMEM237,TMEM161B,HAUS6,RAB11FIP2,EEFSEC,LAG3,PPARG,TANC1,PAPPA,ASB3,MTTP,CHRNB4,NEK7,ZNF80,TENM1,IL13RA2,RORB,GABRB1,MYOF,LTB4R2,SH3KBP1,NALCN,AVIL,USP25,KIF2C,MYO5A,SIPA1L3,ZNF286A-TBC1D26,LRFN2,SPTBN5,SFRP4,BLTP1,SPDYE16,PNRC1,IL1RAP,JCAD,VAV2,TFRC,TES,OSBPL6,GRIA3,PNPLA8,SPDYE6,FBN1,HAPSTR1,RAB31,ZFP82,MAP3K1,DDX4,IQCB1,ZNF292,CLEC6A,BMPER,CLASP2,ZNF521,ZNF761,ANGPT1,SEMA6A,VIPR2,EIF4G1,ATP6V0A4,PHLPP1,ZNF618,ITGA4,DNAJC6,ROCK1,NCAPH,TAX1BP1,TBC1D4,APIP,MMP28,ZNF701,NUP98,IKZF2,PML,FOXO3B,MGLL,PLEK2,TNFSF4,AFAP1L2,DGKG,SP100,ELAVL4,NDUFAF2,CFAP298-TCP10L,UMODL1,RFPL4B,TTC28,NIN,SPG21,ZNF286A,ANKFY1,ZNF69,ENSA,MIR9-2HG,KHDRBS3,AIMP1,SLC30A10,PRKD1,HSPBP1,CALCRL,SLC20A2,USP34,STK4,TNNI3K,SPC25,PRLR,ZNF569,ZNF180,PDE11A,NMI,RAB6C,YLPM1,IL16,ZSWIM7,ZNF470,EDARADD,GNA14,CR2,LPAR6,CTBP2,TTN,NCALD,HHIP,DYRK1A,PPFIA1,TMEM30A,MYRIP,PPP1R1C,UNC13C,KCNE2,RGS10,CUL4A,SERINC1,KCTD16,ZNF888,EYA4,ZNF736,SEMA5A,ELL2,LAMA4,PMEPA1,TTPA,PVT1,GATAD2B,FMNL2,METTL3,UAP1,PARD3,KCNN4,ZNF33A,CP,BMAL2,ATP8B1,RNF6,SEMA3C,VAV1,SMYD1,PEPD,GRIA2,VDAC2,SPART
GO:0000902	cell morphogenesis	1.7136448534117308e-7	*APBB2,MAP3K13,ARL13B,LIMD1,CDC42EP3,CHRNB2,CDH8,ATP10A,CAMK2B,GRIP1,NOTCH2,EPB41L3,MAP6,PARVB,CDH11,SHANK3,PLXNA4,PRKN,TIAM2,LRP8,CNTN1,RHOJ,CDH7,CHRNA7,HECW1,TNIK,TGFB2,LAMA2,TRIO,MUSK,EPHA5,SKOR2,ENAH,FAM171A1,CHL1,PLS1,SLIT3,ITPR1,PAK3,NCKAP1,PRPF40A,FOSL2,EPHA3,LATS2,MYH14,ARHGAP18,ADGRB3,KLF7,DIP2B,PDZD8,EIF2AK4,MATN2,CUX1,EPB41L5,WHRN,APP,IGF2BP1,FRY,PALLD,PTK2,LRRK2,PKHD1,TIAM1,PHACTR1,NEDD4L,KIT,EPHA7,SPAG9,WNT5A,CTNNB1,PRKDC,SCFD1,LMX1A,EFNA5,SYNE3,NGEF,TRPC5,ARHGEF28,RB1,KIRREL3,ETV1,LRTM2,ARHGEF7,BDNF,TFCP2L1,SEMA3E,SH3KBP1,SIPA1L3,CLASP2,SEMA6A,ITGA4,ROCK1,ELAVL4,CDH9,NIN,STK4,SEMA5A,FMNL2,METTL3,PARD3,RNF6,SEMA3C,SPART*
GO:0032502	developmental process	1.8937821752803053e-7	*FSTL1,APBB2,RTN1,HS3ST3A1,RGN,MREG,MAP3K3,OSBPL8,LRRN1,TXNDC8,SMYD3,ARHGEF9,ISM1,TRAPPC9,MAP3K13,ARL13B,LIMD1,TF,CHST11,THRB,USP42,SPTBN1,CDC42EP3,CHRNB2,SEZ6,CDH8,GNB4,AGTPBP1,FHL1,ATP10A,SH3PXD2A,SPINK5,ATP7B,BRD4,DOK4,CAMK2B,GRIP1,DCT,NOTCH2,TMOD2,SDK2,NPNT,EPB41L3,MAP6,MORC1,PARVB,CDH11,SHANK3,NR4A3,ARID1B,E2F7,FOXO1,STYXL1,SMURF2,ADAMTS12,PLXNA4,YBX1,DI*

			PK2A,CACNA1C,ASB4,IDO1,ARB2A,PRKN,NLGN4X,PNPLA3,TAFA1,KDM7A,SLC5A7,IFT172,GPSM1,PTBP3,CDON,SERPINI1,SLC17A6,SDF4,THSD7A,NCAM2,TIAM2,CFAP54,TRAK2,CNMD,LRP8,TAGLN3,CNTN1,MEOX2,STAT5B,ENPEP,EIF4ENIF1,HEATR9,MBTPS2,PTH,ADAR,CLCN3,RHOJ,FGF10,MECOM,CATSPERB,TSPAN18,PLG,CDH7,ILDR2,NCK1,NREP,EXO1,GABRA5,NEXMIF,ASTN1,KDM2B,PREX1,CDIN1,COL11A1,MCPH1,COL1A2,TMOD3,MRTFB,COL12A1,ABLIM1,ALPK2,CHRNA7,HECW1,HMGB1,ZNF304,AFF3,RFLNA,ATRX,TNIK,TGFB2,LAMA2,TRIO,HOXC13,SMARCC1,NDST1,MUSK,JAM3,ALKBH1,ATP11A,SLC15A4,PCDHB8,PRKG2,SND1,IGFBP7,EPHA5,SKOR2,ENAH,SNX10,ATP2C1,ALDH1A2,FAM171A1,HUWE1,ASPH,RRAS2,SIM1,KIAA1217,SOX30,CHL1,GSN,PLS1,SLIT3,PHC2,ANKRD11,ITPR1,CPM,DNAAF11,PAK3,ABI3BP,AXDND1,MLLT3,CCNT2,BTG4,CPQ,MR1,TNFRSF12A,CPT2,CXCR5,NCKAP1,PRPF40A,FOSL2,PACRG,IL1RAPL2,EPHA3,LATS2,MYH14,ARHGAP18,MYOM2,LYN,ADGRB3,KLF7,GRIK1,MTA3,DIP2B,SEC24C,ATP6V1H,FIGNL1,PDZD8,CA10,RRM2,CRYBB1,LDB2,RNASEL,NAV1,PAQR5,PCDHB16,SGMS2,NOS3,VSX1,PRAMEF27,ERCC6,EIF2AK4,XKR6,MATN2,TGFBR2,ITGA11,MARK4,TCF7L2,CRTAC1,ZFHX3,CUX1,UNC119,GPM6B,ADGRV1,ST7,EPB41L5,WHRN,OCA2,TDP2,PRUNE1,APP,GATA4,SMAD5,IGF2BP1,MAMLD1,RNF38,ADAM12,DHX35,HCN1,PI4KB,FRY,HOOK3,PALLD,CECR2,RIPK4,PTK2,SH2B2,CELF1,HYCC1,PRRC2B,LRRK2,INO80D,IKZF3,CLSTN2,ITFG2,PKHD1,TENT5C,RARB,TCF4,PAEP,TIAM1,CSRP3,PHACTR1,ACVR2A,VIT,SNTG2,NTM,NEDD4L,KIT,CYB5R4,EPHA7,SPAG9,ANTXR1,WNT5A,CTNNB1,ANKRD17,COL19A1,RUNX1T1,CTSK,PRKDC,ESR1,TNFRSF19,PTPN14,SCFD1,POFUT2,BCAR3,LMX1A,PTGER4,GREB1L,BBX,VWC2L,DOCK1,GPC4,BPGM,EFNA5,IGF2BP2,SYNE3,HECTD1,NGEF,TRPC5,NEUROD2,KAT14,PTPRF,IKBKB,FXR2,AKAP6,USF3,LYVE1,APOH,ARHGEF28,TASOR,GPRIN1,OPCML,LINGO2,ATF2,ACVR1,PID1,EBF2,GRID2,ZNF423,PLPPR1,RB1,MPV17,DDX10,PKD1L3,ADIPOR1,GABRB3,SHROOM4,PLPP3,HNF1B,IMMP2L,KIRREL3,GABRG2,CPS1,NCOA2,TMEM120A,ACACB,AGPAT5,XRCC5,SHLD2,GFRA1,BMX,SLC24A3,RAB18,ETV1,TEX11,LRTM2,SIK1,KAT2B,WLS,ANKS1A,PRCP,MEF2D,CDK5RAP2,FLVCR1,ARHGEF7,DACH1,ATF3,NPHP1,IKZF1,SLC26A8,SRI,ARNT,BDNF,LDLRAD4,TFCP2L1,SEMA3E,SCIN,MACO1,ADAMTS9,STIM1,COX7B2,LAG3,PPARG,TANC1,TGM5,TENM1,RORB,GABRB1,SH3KBP1,AVIL,TTC39C,SIPA1L3,SFRP4,BLTP1,IL1RAP,JCAD,VAV2,TFRC,VPS13A,FBN1,DDX4,IQCB1,BMPER,FUT10,CLASP2,ZNF521,ANGPT1,SEMA6A,EIF4G1,ATP6V0A4,PHLPP1,ITGA4,ROCK1,M1AP,CLEC3A,PML,TNFSF4,DGKG,SPATA25,SP100,ELAVL4,SBF2,CDH9,UMODL1,NIN,FNDC3A,AIMP1,PRKD1,CALCRL,SLC20A2,STK4,PCDH10,ANPEP,PRLR,EDARADD,CR2,LPAR6,CTBP2,TTN,HHIP,DYRK1A,TMEM30A,UNC13C,KCNE2,CUL4A,TXNRD3,EYA4,SEMA5A,LAMA4,TTPA,GATAD2B,FMNL2,METTL3,KRT85,PARD3,ATP8B1,RNF6,SEMA3C,VAV1,SMYD1,VPS13B,SPART
GO:0050789	regulation of biological	2.2979313235846572e-7	FSTL1,C10ORF90,APBB2,STYK1,RTN1,RGN,CDK14,PDE1C,NOSTRIN,ING3,MAP3K3,OSBPL8,LRRN1,STX8,PHF20,FOXK2,SMYD3,RAPGEF6,ARHGEF9,

	process		*TAFA2,ABR,ISM1,MAOA,MAP3K13,PPM1A,ARL13B,SAMSN1,LIMD1,TF,CHST11,THRB,USP42,SPTBN1,CDC42EP3,MAPK10,CACNB1,USP32,DAPP1,ATF7,GNG12,CHRNB2,CDC14C,SEZ6,CDH8,GNB4,AGTPBP1,ATF7-NPFF,FHL1,RBPMS,ATP10A,SPINK5,OPA3,SKAP2,GPBP1L1,KCNJ6,BRD4,DOK4,CAMK2B,GRIP1,ARHGAP11A-SCG5,DCT,DLGAP1,SLC38A9,ZNF264,HBP1,SDR16C5,ABCG8,XPO4,MAP4K3,NOTCH2,TMOD2,HMBOX1,ZNF551,NPNT,EPB41L3,MAP6,MORC1,PARVB,CDH11,SHANK3,NR4A3,ARID1B,E2F7,FOXO1,ZNF611,STYXL1,STK39,SMURF2,ADAMTS12,FER1L6,TBC1D16,PLXNA4,CDK18,URB2,YBX1,DIPK2A,DCDC1,CACNA1C,FAM3C,ASB4,IDO1,ARB2A,PRKN,NLGN4X,TAFA1,PIK3C2B,KDM7A,SLC5A7,FAM83B,IFT172,GPSM1,CBLL2,PTBP3,CDON,SERPINI1,SLC17A6,NLK,GNAI1,MRPL13,MAPKAPK3,TIAM2,ZNF10,CNMD,LRP8,TAGLN3,CNTN1,MEOX2,STAT5B,ENPEP,ARHGEF4,UBE2O,DDIAS,CPEB4,EIF4ENIF1,CHRFAM7A,ERBIN,ZNF121,MBTPS2,RGS22,CD247,MCC,PTH,ADAR,PDE10A,TM9SF2,CLCN3,RHOJ,CDC37,FGF10,ZFYVE16,MECOM,COL4A6,TSPAN18,NLRP8,STK32B,PLG,HERC5,UBE2E2,ILDR2,NCK1,CD84,FAM168A,NONO,RARRES1,KLHL42,NREP,GABRA5,NEXMIF,MTCP1,KDM2B,PREX1,ZNF404,WRNIP1,RSF1,NCOA7,CIDEB,MCPH1,SESTD1,ZNF214,COL1A2,DTNA,TMOD3,GEMIN5,MRTFB,ALPK2,CHRNA7,HECW1,HMGB1,ZNF304,DYNLT2B,CDIP1,AFF3,RFLNA,HIVEP1,ATRX,TNIK,TGFB2,MDFIC,LAMA2,ADD3,TRIO,ZNF85,HOXC13,SMARCC1,NDST1,MUSK,KCNJ18,JAM3,KCNIP3,SFPQ,ALKBH1,RGS3,NUP93,ZCCHC4,RERG,OR5AU1,PRKAR2A,RBM33,CTIF,ATP11A,RBM23,ZNF154,ATP4A,SLC15A4,GOLPH3,PRKG2,MBTPS1,USP10,SND1,IGFBP7,EPHA5,SKOR2,ATP2C1,ALDH1A2,PPTC7,STON2,PTGFR,FAM171A1,STRADA,HUWE1,ZNF595,ASPH,RRAS2,SH3BGRL,UBR5,GTF2F2,SIM1,SOX30,ANKS1B,CHL1,SPOCK3,GSN,PLS1,TCP11L1,SPON1,MAPRE3,TLR8,SLIT3,ARMH3,CACUL1,NLRP7,ARHGEF39,CD226,PHC2,ZNF578,ITPR1,PKP4,PAK3,ABI3BP,KLF12,PRKAG2,MLLT3,CCNT2,BTG4,HAVCR1,CNOT10,MR1,TNFRSF12A,OASL,CPT2,TAS2R14,VPS8,ZNF429,CXCR5,NCKAP1,PRPF40A,CILK1,FOSL2,IL1RAPL2,ADGRD1,EPHA3,LATS2,MYH14,SPDYE2B,ERP29,CDYL2,ARHGAP18,UVRAG,LYN,USP6,ADGRB3,KLF7,SUMO2,GRIK1,MTA3,DIP2B,NME7,ATP6V1H,NUP58,FIGNL1,PDZD8,RRM2,LDB2,MYRFL,ALDH1A1,FMO5,RNASEL,DBF4B,CORIN,PCDHB16,DCP2,SGMS2,TMED7-TICAM2,NOS3,VSX1,ZNF280B,TNRC6C,PRAMEF27,MLLT10,ERCC6,EIF2AK4,KIAA1614,RAB12,MAGEL2,TGFBR2,ITGA11,CMKLR2,MARK4,SYNJ1,TCF7L2,ZNF462,E2F6,ZFHX3,CUX1,UNC119,SHISA2,GPM6B,RRAGD,ULK3,SPDYE2,GPR156,GKAP1,PPP2R5E,GSDMD,ADGRV1,ST7,RASGRF2,SERBP1,EPB41L5,GAD1,SCG5,WAC,WHRN,DAPK1,TDP2,ZNF678,PRUNE1,SAR1B,INSYN2A,BTG3,APP,GATA4,ATF7IP2,SMAD5,IGF2BP1,MAMLD1,RNLS,GSKIP,ADAM12,PELI2,SP140L,HCN1,CHRM5,PI4KB,FRY,RHOT1,ELOVL5,HOOK3,PIK3R5,TLR1,TAS2R30,BMT2,RIPK4,AVPR1B,PTK2,USP7,TICAM2,SCP2,SH2B2,ATP1B4,AVEN,ZNF41,CELF1,POGZ,PIP4K2B,LRRK2,INO80D,ZNF395,IKZF3,CLSTN2,ARIH1*

			,CFAP298,PLD1,VTCN1,ITFG2,PKHD1,TENT5C,RARB,PJA2,TCF4,PAEP,COP1,TIAM1,CSRP3,PHACTR1,MLIP,IQSEC2,ACVR2A,GNAO1,TIPIN,VIT,RASSF4,NEDD4L,BRCC3,SNX13,RABGEF1,KIT,CYB5R4,TBC1D8,EPHA7,SPAG9,ANTXR1,SPDL1,WNT5A,TRABD2B,TEX2,ARFGEF2,CTNNB1,ANKRD17,RUNX1T1,CTSK,PRKDC,IPO5,ESR1,TNFRSF19,ZNF479,PTPN14,SCFD1,ERC2,POFUT2,BCAR3,TPD52L1,L3MBTL4,PPM1E,ZXDC,FRMD4A,LMX1A,BNIPL,HUNK,PTGER4,ZNF215,PDE7B,BBX,VWC2L,BEST3,DOCK1,GPC4,PKIG,EFNA5,IGF2BP2,CDC14B,ZNF722,SYNE3,PLCH1,OR1M1,HECTD1,NGEF,TRPC5,NEUROD2,FNIP2,AASS,JAML,KAT14,PTPRF,IKBKB,FXR2,SH3BP5,AKAP6,TRBV10-2,USF3,LYVE1,APOH,ARHGEF28,RAB3GAP2,TASOR,CCPG1,LINGO2,CEMIP,XIST,ATF2,KCNJ12,ZNF426,HDAC8,ACVR1,CHRNB3,HLA-DQA2,PID1,EBF2,GRID2,ZNF423,ZCCHC17,KCNJ3,PLPPR1,RB1,NSMCE1,MPV17,IFNLR1,PDE4B,KIF13A,NLRP4,ADIPOR1,GABRB3,ZNF143,ZSCAN23,SMG7,PLPP3,HNF1B,ZNF615,LAMP2,GABRG2,NCOA2,BNIP3L,ACACB,RABGAP1L,XRCC5,TBRG4,RGS17,SHLD2,GFRA1,KCND2,INPP4A,BMX,ZNF418,SLC24A3,TAF15,GRIA4,RAB18,ZNF860,POU2F1,ETV1,RGS7BP,BACH2,MGRN1,TEX11,MID2,MIR198,LRTM2,SIK1,ZNF850,TP53BP2,KAT2B,GOLPH3L,WLS,ANKS1A,RYR3,PRCP,SUPV3L1,MEF2D,MYL4,GTF2A1,SPTLC1,CDK5RAP2,FLVCR1,RFC3,ARHGEF7,SAMD12,DACH1,ATF3,TRDN,NPHP1,IKZF1,CAPZA2,SRI,ATP1B1,ARNT,BDNF,LDLRAD4,TFCP2L1,SEMA3E,BCLAF3,SCIN,MACO1,ZNF665,TLR6,ADAMTS9,STIM1,TMEM237,TMEM161B,HAUS6,RAB11FIP2,EEFSEC,LAG3,PPARG,TANC1,PAPPA,ASB3,MTTP,CHRNB4,NEK7,ZNF80,TENM1,IL13RA2,RORB,GABRB1,MYOF,LTB4R2,SH3KBP1,NALCN,AVIL,USP25,KIF2C,MYO5A,SIPA1L3,ZNF286A-TBC1D26,LRFN2,SPTBN5,SFRP4,BLTP1,SPDYE16,PNRC1,IL1RAP,JCAD,VAV2,TFRC,TES,OSBPL6,GRIA3,PNPLA8,SPDYE6,FBN1,HAPSTR1,RAB31,ZFP82,MAP3K1,DDX4,IQCB1,ZNF292,CLEC6A,BMPER,CLASP2,ZNF521,ZNF761,ANGPT1,SEMA6A,VIPR2,EIF4G1,PHLPP1,ZNF618,ITGA4,DNAJC6,ROCK1,NCAPH,TAX1BP1,TBC1D4,APIP,MMP28,ZNF701,NUP98,IKZF2,PML,FOXO3B,MGLL,PLEK2,TNFSF4,AFAP1L2,DGKG,SP100,ELAVL4,NDUFAF2,CFAP298-TCP10L,UMODL1,RFPL4B,TTC28,NIN,SPG21,ZNF286A,ANKFY1,ZNF69,ENSA,MIR9-2HG,KHDRBS3,AIMP1,SLC30A10,PRKD1,HSPBP1,CALCRL,SLC20A2,USP34,STK4,TNNI3K,SPC25,PRLR,ZNF569,ZNF180,PDE11A,NMI,RAB6C,YLPM1,IL16,ZNF470,EDARADD,GNA14,CR2,LPAR6,CTBP2,TTN,NCALD,HHIP,DYRK1A,PPFIA1,TMEM30A,MYRIP,PPP1R1C,UNC13C,KCNE2,RGS10,CUL4A,KCTD16,ZNF888,EYA4,ZNF736,SEMA5A,ELL2,LAMA4,PMEPA1,TTPA,PVT1,GATAD2B,FMNL2,METTL3,UAP1,PARD3,KCNN4,ZNF33A,CP,BMAL2,ATP8B1,RNF6,SEMA3C,VAV1,SMYD1,PEPD,GRIA2,VDAC2,SPART*
GO:0065008	regulation of biological quality	4.991742113385064e-7	*C10ORF90,SLC12A8,LRRN1,MAP3K13,LIMD1,TF,THRB,SPTBN1,CDC42EP3,CHRNB2,SEZ6,CDH8,KCNQ5,FHL1,ATP10A,ATP7B,CAMK2B,GRIP1,DDI2,SDR16C5,TMOD2,EPB41L3,PARVB,SHANK3,FOXO1,STK39,PLXNA4,YBX1,CACNA1C,PRKN,NLGN4X,F*

			13A1,SLC17A6,NLK,LRP8,STAT5B,ENPEP,DDIAS,EIF4ENIF1,CHRFAM7A,ERBIN,CLCN3,RHOJ,CDC37,FGF10,TSPAN18,PLG,ILDR2,NCK1,GABRA5,PREX1,ANO3,COL1A2,TMOD3,CHRNA7,HMGB1,ADD3,MUSK,JAM3,PNPLA4,RBM33,ATP11A,EPHA5,ALDH1A2,PTGFR,FAM171A1,ASPH,GSN,PLS1,ITPR1,PAK3,TMEM183A,CPQ,CNOT10,F8,CXCR5,NCKAP1,PRPF40A,DTD1,FOSL2,IL1RAPL2,MYH14,ARHGAP18,LYN,ADGRB3,KLF7,GRIK1,DIP2B,ATP6V1H,SLC12A1,ALDH1A1,RNASEL,CORIN,DCP2,NOS3,TNRC6C,EIF2AK4,XKR6,TCF7L2,ADGRV1,RASGRF2,SERBP1,SCG5,OCA2,INSYN2A,APP,IGF2BP1,RNLS,HCN1,RHOT1,AVPR1B,PTK2,USP7,SCP2,CELF1,LRRK2,CLSTN2,TENT5C,TIAM1,CSRP3,IQSEC2,NEDD4L,KIT,CYB5R4,EPHA7,WNT5A,CTNNB1,CTSK,PRKDC,ESR1,ERC2,PTGER4,GPC4,EFNA5,IGF2BP2,SYNE3,NGEF,TRPC5,NEUROD2,FXR2,AKAP6,APOH,CLCN1,LINGO2,ATF2,HDAC8,CHRNB3,PID1,GRID2,ZCCHC17,KCNJ3,PLPP3,HNF1B,LAMP2,GABRG2,F5,CPS1,BNIP3L,TBRG4,KCND2,TAF15,GRIA4,RGS7BP,LRTM2,KAT2B,PRCP,MYL4,ARHGEF7,TRDN,CAPZA2,SRI,ATP1B1,ARNT,BDNF,SEMA3E,SCIN,TMEM161B,RAB11FIP2,PPARG,TANC1,CHRNB4,TENM1,GABRB1,SH3KBP1,NALCN,AVIL,USP25,LRFN2,SPTBN5,IL1RAP,VAV2,GRIA3,CLASP2,ANGPT1,SEMA6A,EIF4G1,ATP6V0A4,ITGA4,ROCK1,PML,DGKG,ELAVL4,NDUFAF2,ENSA,AIMP1,PRKD1,STK4,TNNI3K,PRLR,ZSWIM7,GNA14,NCALD,PPFIA1,TMEM30A,MYRIP,UNC13C,KCNE2,SEMA5A,FMNL2,METTL3,KCNN4,ATP8B1,RNF6,SEMA3C,VAV1,GRIA2,VDAC2,SPART
GO:0007399	nervous system development	5.904155320743422e-7	*APBB2,RTN1,LRRN1,ARHGEF9,TRAPPC9,MAP3K13,ARL13B,THRB,SPTBN1,CHRNB2,SEZ6,GNB4,AGTPBP1,SPINK5,DOK4,CAMK2B,GRIP1,DCT,NOTCH2,TMOD2,SDK2,EPB41L3,MAP6,CDH11,SHANK3,ARID1B,STYXL1,PLXNA4,PRKN,NLGN4X,TAFA1,KDM7A,IFT172,GPSM1,CDON,SERPINI1,NCAM2,TIAM2,TRAK2,LRP8,TAGLN3,CNTN1,EIF4ENIF1,FGF10,NCK1,NREP,GABRA5,NEXMIF,ASTN1,KDM2B,PREX1,MCPH1,CHRNA7,HECW1,HMGB1,ATRX,TNIK,TGFB2,LAMA2,TRIO,SMARCC1,NDST1,MUSK,JAM3,ALKBH1,PCDHB8,EPHA5,SKOR2,ENAH,ALDH1A2,RRAS2,SIM1,CHL1,GSN,PLS1,SLIT3,PAK3,BTG4,NCKAP1,IL1RAPL2,EPHA3,LYN,ADGRB3,KLF7,GRIK1,DIP2B,CA10,LDB2,NAV1,PCDHB16,VSX1,ERCC6,EIF2AK4,MATN2,TGFBR2,MARK4,CRTAC1,ZFHX3,CUX1,UNC119,GPM6B,ADGRV1,WHRN,TDP2,PRUNE1,APP,IGF2BP1,HCN1,FRY,HOOK3,PALLD,CECR2,PTK2,SH2B2,CELF1,HYCC1,LRRK2,CLSTN2,RARB,TCF4,TIAM1,PHACTR1,VIT,SNTG2,NTM,NEDD4L,KIT,EPHA7,SPAG9,WNT5A,CTNNB1,PRKDC,LMX1A,VWC2L,GPC4,BPGM,EFNA5,IGF2BP2,NGEF,TRPC5,NEUROD2,PTPRF,FXR2,ARHGEF28,GPRIN1,OPCML,LINGO2,ATF2,GRID2,ZNF423,PLPPR1,RB1,GABRB3,SHROOM4,PLPP3,HNF1B,IMMP2L,KIRREL3,GABRG2,XRCC5,GFRA1,RAB18,ETV1,LRTM2,KAT2B,WLS,ANKS1A,MEF2D,CDK5RAP2,ARHGEF7,BDNF,SEMA3E,SCIN,MACO1,PPARG,TENM1,RORB,GABRB1,AVIL,IL1RAP,VPS13A,FUT10,CLASP2,ZNF521,SEMA6A,EIF4G1,ITGA4,ROCK1,DGKG,ELAVL4,SBF2,CDH9,NIN,PRKD1,STK4,PCDH10,HHIP,DYRK1A,TMEM30A,SEMA5A,TTPA,METTL3,PARD3,ATP8B1,RNF6,SEMA3C,VPS13B,SPART*
GO:0048	neuron	0.000002219	*APBB2,MAP3K13,THRB,CHRNB2,SEZ6,AGTPBP1,C*

666	development	80102987905 4	AMK2B,GRIP1,NOTCH2,EPB41L3,MAP6,CDH11,SHANK3,STYXL1,PLXNA4,PRKN,SERPINI1,NCAM2,TIAM2,LRP8,CNTN1,NCK1,NREP,GABRA5,PREX1,CHRNA7,HECW1,HMGB1,TNIK,TGFB2,LAMA2,TRIO,MUSK,ALKBH1,EPHA5,SKOR2,ENAH,CHL1,PLS1,SLIT3,PAK3,NCKAP1,EPHA3,LYN,ADGRB3,KLF7,DIP2B,VSX1,ERCC6,EIF2AK4,MATN2,CRTAC1,CUX1,GPM6B,ADGRV1,WHRN,TDP2,APP,IGF2BP1,HCN1,FRY,PALLD,CECR2,PTK2,LRRK2,TIAM1,PHACTR1,NTM,NEDD4L,KIT,EPHA7,SPAG9,WNT5A,CTNNB1,LMX1A,EFNA5,NGEF,TRPC5,NEUROD2,PTPRF,ARHGEF28,GPRIN1,OPCML,GRID2,RB1,KIRREL3,GFRA1,ETV1,LRTM2,KAT2B,ANKS1A,BDNF,SEMA3E,TENM1,RORB,GABRB1,AVIL,CLASP2,SEMA6A,ITGA4,ROCK1,DGKG,ELAVL4,NIN,PRKD1,TMEM30A,SEMA5A,PARD3,ATP8B1,RNF6,SEMA3C,VPS13B,SPART
GO:0035 556	intracellular signal transduction	0.000002606 99247686418	APBB2,RGN,MAP3K3,OSBPL8,RAPGEF6,ARHGEF9,ABR,MAP3K13,PPM1A,LIMD1,TF,THRB,SPTBN1,CDC42EP3,MAPK10,USP32,SEZ6,RBPMS,BRD4,DOK4,GRIP1,SLC38A9,MAP4K3,NOTCH2,NPNT,SHANK3,NR4A3,E2F7,FOXO1,STYXL1,STK39,URB2,DIPK2A,DCDC1,CACNA1C,ASB4,PRKN,PIK3C2B,CDON,NLK,GNAI1,MAPKAPK3,TIAM2,ARHGEF4,DDIAS,ERBIN,MBTPS2,PTH,PDE10A,RHOJ,FGF10,MECOM,STK32B,HERC5,NCK1,NONO,MTCP1,PREX1,CIDEB,COL1A2,CHRNA7,HMGB1,CDIP1,ATRX,TNIK,TGFB2,MDFIC,TRIO,NDST1,MUSK,SFPQ,NUP93,RERG,PRKAR2A,SLC15A4,GOLPH3,MBTPS1,USP10,EPHA5,ATP2C1,ALDH1A2,ASPH,RRAS2,UBR5,TLR8,ITPR1,PAK3,PRKAG2,OASL,CXCR5,NCKAP1,CILK1,LATS2,ERP29,ARHGAP18,LYN,FIGNL1,NOS3,ERCC6,EIF2AK4,RAB12,TGFBR2,MARK4,TCF7L2,RRAGD,ADGRV1,RASGRF2,WAC,DAPK1,APP,GATA4,SMAD5,GSKIP,PELI2,PI4KB,RHOT1,PIK3R5,BMT2,AVPR1B,PTK2,USP7,TICAM2,SH2B2,LRRK2,ARIH1,PLD1,ITFG2,PKHD1,RARB,PJA2,TIAM1,CSRP3,IQSEC2,ACVR2A,TIPIN,BRCC3,RABGEF1,KIT,EPHA7,SPAG9,SPDL1,WNT5A,ARFGEF2,CTNNB1,ANKRD17,PRKDC,IPO5,ESR1,TNFRSF19,BCAR3,TPD52L1,HUNK,PTGER4,PDE7B,DOCK1,CDC14B,PLCH1,NGEF,NEUROD2,FNIP2,IKBKB,SH3BP5,AKAP6,ARHGEF28,ATF2,ACVR1,RB1,ADIPOR1,LAMP2,NCOA2,BMX,RAB18,MGRN1,MID2,SIK1,TP53BP2,WLS,RYR3,PRCP,CDK5RAP2,ARHGEF7,ATF3,ATP1B1,ARNT,LDLRAD4,SEMA3E,TLR6,PPARG,ASB3,NEK7,TENM1,RORB,MYO5A,SIPA1L3,JCAD,VAV2,TFRC,PNPLA8,HAPSTR1,MAP3K1,CLEC6A,BMPER,ANGPT1,SEMA6A,PHLPP1,ROCK1,TAX1BP1,APIP,PML,PLEK2,DGKG,SP100,SLC30A10,PRKD1,STK4,SPC25,PDE11A,NMI,RAB6C,LPAR6,CTBP2,TTN,NCALD,DYRK1A,PPP1R1C,CUL4A,SEMA5A,PMEPA1,RNF6,VAV1,VDAC2
GO:0051 179	localization	0.000002646 81812880397 44	ESYT1,MREG,NOSTRIN,OSBPL8,SLC12A8,STX8,SMYD3,RAPGEF6,BLTP3B,TRAPPC9,SLC16A5,PPM1A,ARL13B,RN7SL556P,IMMP1L,TF,CHST11,RCSD1,SPTBN1,MYO5C,CACNB1,CHRNB2,KCNQ5,AGTPBP1,FHL1,ATP10A,ATP7B,KCNJ6,CAMK2B,GRIP1,SLC38A9,RGPD2,ABCG8,XPO4,CD93,SLC44A5,NPNT,EPB41L3,MAP6,SHANK3,NR4A3,SLC14A2,FOXO1,STK39,YBX1,CACNA1C,PRKN,NLGN4X,XPO6,SLC44A3,SLC5A7,IFT172,GPSM1,SLC17A6,GNAI1,MAPKAPK3,INTS6-AS1,CFAP54,TRAK2,LRP8,CNTN1,STAT5B,UBE2O

			,*EIF4ENIF1,CHRFAM7A,ERBIN,CD247,CEP192,MCC,PTH,ADAR,TM9SF2,CLCN3,RHOJ,CDC37,FGF10,ZFYVE16,TSPAN18,ILDR2,CD84,GABRA5,RGPD3,ANO3,CIDEB,SLC45A1,MCPH1,MYO1D,SESTD1,XPO7,KCNS3,CHRNA7,HMGB1,DYNLT2B,ATRX,TNIK,TGFB2,MDFIC,MUSK,KCNJ18,JAM3,KCNIP3,NUP93,RBM33,ATP11A,ATP4A,SLC15A4,GOLPH3,PRKG2,MBTPS1,NCF2,EPHA5,ATP13A4,SNX10,ATP2C1,TMEM230,STON2,STRADA,HUWE1,ASPH,ZDHHC4,UBR5,SOX30,GSN,PLS1,MAPRE3,HPS3,ITPR1,DNAAF11,PRKAG2,HAVCR1,TRAPPC10,CPT2,VPS8,MTX3,CILK1,FOSL2,PACRG,EPHA3,LATS2,ERP29,UVRAG,LYN,USP6,KLF7,GRIK1,SEC24C,ATP6V1H,NUP58,SLC12A1,PDZD8,SIL1,C21ORF62,RNASEL,CORIN,TMCC1,DCP2,TMED7-TICAM2,SLC41A2,NOS3,NOMO2,RN7SL738P,KIAA1614,XKR6,MAGEL2,TGFBR2,MARK4,MDN1,SYNJ1,TCF7L2,CUX1,UNC119,GPM6B,RRAGD,CACNA2D4,RGPD1,KPNB1,GSDMD,ADGRV1,EPB41L5,SCG5,WHRN,OCA2,DAPK1,SAR1B,TTC7B,APP,IGF2BP1,HCN1,CHRM5,SLC13A1,PI4KB,RHOT1,USE1,HOOK3,CECR2,TMCO3,AVPR1B,LMAN1L,PTK2,NOP9,USP7,TICAM2,SCP2,ATP1B4,HYCC1,PIP4K2B,LRRK2,GET1-SH3BGR,SLC38A4,PLD1,PKHD1,MGST1,TIAM1,CSRP3,IQSEC2,NEDD4L,ENSG00000288683,SNX13,RABGEF1,KIT,CYB5R4,OSBPL3,SPAG9,SPDL1,WNT5A,PEX26,TEX2,ARFGEF2,CTNNB1,IPO5,ESR1,PTPN14,SCFD1,ERC2,POFUT2,VPS53,FRMD4A,BEST3,DOCK1,GPC4,BPGM,PKIG,EFNA5,IGF2BP2,SYNE3,PLCH1,TRPC5,IKBKB,AKAP6,LYVE1,APOH,RAB3GAP2,CLCN1,TASOR,CEMIP,XIST,ATF2,KCNJ12,CHRNB3,PID1,CCDC91,GRID2,ZNF423,KCNJ3,RB1,MPV17,PDE4B,PKD1L3,KIF13A,GABRB3,GOSR2,SMG7,PLPP3,UNC80,HNF1B,IMMP2L,LAMP2,GABRG2,TMEM120A,BNIP3L,ACACB,RABGAP1L,XRCC5,KCND2,SLC24A3,GRIA4,RAB18,BACH2,MGRN1,MID2,SIK1,KAT2B,GOLPH3L,WLS,RYR3,MYO1B,CDK5RAP2,FLVCR1,ARHGEF7,TRDN,NPHP1,SLC26A8,SRI,ATP1B1,BDNF,SCIN,ADAMTS9,STIM1,RAB11FIP2,PPARG,ASB3,MTTP,CHRNB4,TENM1,IL13RA2,GABRB1,MYOF,JAKMIP1,SH3KBP1,NALCN,GDAP1,KIF2C,MYO5A,SLC6A13,SPTBN5,SFRP4,BLTP1,VAV2,TFRC,VPS13A,OSBPL6,GRIA3,PNPLA8,FBN1,RAB31,BMPER,FUT10,CLASP2,ANGPT1,EIF4G1,ATP6V0A4,ITGA4,DNAJC6,ROCK1,TAX1BP1,TBC1D4,KCNT2,NUP98,PML,SCFD2,SCARA5,SLC5A12,SP100,NDUFAF2,NIN,ANKFY1,ENSA,AIMP1,SLC30A10,AP4S1,PRKD1,CALCRL,SLC20A2,STK4,RAB6D,SPC25,SCAMP1,PRLR,RAB6C,IL16,CTBP2,TTN,NCALD,PPFIA1,TMEM30A,MYRIP,UNC13C,KCNE2,TTPA,PARD3,KCNN4,CP,ATP8B1,VAV1,GRIA2,VDAC2,VPS13B*
GO:0006810	transport	0.000003960927830477829	*ESYT1,MREG,NOSTRIN,OSBPL8,SLC12A8,STX8,BLTP3B,TRAPPC9,SLC16A5,PPM1A,TF,RCSD1,SPTBN1,MYO5C,CACNB1,CHRNB2,KCNQ5,AGTPBP1,FHL1,ATP10A,ATP7B,KCNJ6,CAMK2B,GRIP1,SLC38A9,RGPD2,ABCG8,XPO4,CD93,SLC44A5,MAP6,NR4A3,SLC14A2,FOXO1,STK39,YBX1,CACNA1C,PRKN,NLGN4X,XPO6,SLC44A3,SLC5A7,IFT172,SLC17A6,MAPKAPK3,CFAP54,TRAK2,LRP8,CNTN1,STAT5B,UBE2O,EIF4ENIF1,CHRFAM7A,PTH,ADAR,CLCN3,RHOJ,FGF10,ZFYVE16,TSPAN18,ILDR2,CD84,GABRA5,RGPD3,ANO3,CIDEB,SLC45A1,MYO1D,*

			SESTD1,XPO7,KCNS3,CHRNA7,HMGB1,DYNLT2B,TGFB2,MDFIC,KCNJ18,KCNIP3,NUP93,RBM33,ATP11A,ATP4A,SLC15A4,GOLPH3,PRKG2,MBTPS1,NCF2,EPHA5,ATP13A4,SNX10,ATP2C1,TMEM230,STON2,STRADA,ASPH,UBR5,SOX30,GSN,HPS3,ITPR1,DNAAF11,PRKAG2,HAVCR1,TRAPPC10,CPT2,VPS8,MTX3,CILK1,FOSL2,EPHA3,ERP29,UVRAG,LYN,USP6,KLF7,GRIK1,SEC24C,ATP6V1H,NUP58,SLC12A1,PDZD8,SIL1,C21ORF62,RNASEL,CORIN,TMCC1,TMED7-TICAM2,SLC41A2,NOS3,XKR6,MAGEL2,TGFBR2,MDN1,SYNJ1,TCF7L2,CUX1,UNC119,GPM6B,CACNA2D4,RGPD1,KPNB1,GSDMD,SCG5,OCA2,DAPK1,SAR1B,APP,IGF2BP1,HCN1,CHRM5,SLC13A1,PI4KB,RHOT1,USE1,HOOK3,CECR2,TMCO3,AVPR1B,LMAN1L,PTK2,NOP9,USP7,TICAM2,SCP2,ATP1B4,PIP4K2B,LRRK2,SLC38A4,PLD1,MGST1,TIAM1,NEDD4L,ENSG00000288683,SNX13,RABGEF1,KIT,CYB5R4,OSBPL3,SPAG9,WNT5A,PEX26,TEX2,ARFGEF2,CTNNB1,IPO5,PTPN14,SCFD1,ERC2,POFUT2,VPS53,FRMD4A,BEST3,DOCK1,BPGM,PKIG,EFNA5,IGF2BP2,SYNE3,PLCH1,TRPC5,AKAP6,LYVE1,APOH,RAB3GAP2,CLCN1,CEMIP,ATF2,KCNJ12,CHRNB3,PID1,CCDC91,GRID2,KCNJ3,MPV17,PDE4B,PKD1L3,KIF13A,GABRB3,GOSR2,SMG7,PLPP3,UNC80,HNF1B,LAMP2,GABRG2,TMEM120A,BNIP3L,ACACB,RABGAP1L,KCND2,SLC24A3,GRIA4,RAB18,BACH2,MGRN1,SIK1,GOLPH3L,WLS,RYR3,MYO1B,FLVCR1,ARHGEF7,TRDN,SLC26A8,SRI,ATP1B1,SCIN,ADAMTS9,STIM1,RAB11FIP2,PPARG,MTTP,CHRNB4,TENM1,IL13RA2,GABRB1,MYOF,JAKMIP1,SH3KBP1,NALCN,MYO5A,SLC6A13,SPTBN5,SFRP4,BLTP1,VAV2,TFRC,VPS13A,OSBPL6,GRIA3,PNPLA8,RAB31,CLASP2,ANGPT1,ATP6V0A4,ITGA4,DNAJC6,ROCK1,TBC1D4,KCNT2,NUP98,PML,SCFD2,SCARA5,SLC5A12,SP100,NDUFAF2,ANKFY1,ENSA,AIMP1,SLC30A10,AP4S1,PRKD1,CALCRL,SLC20A2,STK4,RAB6D,SCAMP1,PRLR,RAB6C,IL16,CTBP2,TTN,NCALD,TMEM30A,MYRIP,UNC13C,KCNE2,TTPA,KCNN4,CP,ATP8B1,VAV1,GRIA2,VDAC2,VPS13B
GO:0048699	generation of neurons	0.000005313263878500116	*APBB2,RTN1,TRAPPC9,MAP3K13,THRB,CHRNB2,SEZ6,AGTPBP1,SPINK5,CAMK2B,GRIP1,DCT,NOTCH2,SDK2,EPB41L3,MAP6,CDH11,SHANK3,STYXL1,PLXNA4,PRKN,NLGN4X,TAFA1,IFT172,CDON,SERPINI1,NCAM2,TIAM2,LRP8,CNTN1,EIF4ENIF1,NCK1,NREP,GABRA5,NEXMIF,ASTN1,PREX1,CHRNA7,HECW1,HMGB1,TNIK,TGFB2,LAMA2,TRIO,MUSK,ALKBH1,EPHA5,SKOR2,ENAH,ALDH1A2,CHL1,PLS1,SLIT3,PAK3,BTG4,NCKAP1,EPHA3,LYN,ADGRB3,KLF7,DIP2B,NAV1,VSX1,ERCC6,EIF2AK4,MATN2,CRTAC1,ZFHX3,CUX1,GPM6B,ADGRV1,WHRN,TDP2,APP,IGF2BP1,HCN1,FRY,PALLD,CECR2,PTK2,LRRK2,TCF4,TIAM1,PHACTR1,NTM,NEDD4L,KIT,EPHA7,SPAG9,WNT5A,CTNNB1,LMX1A,VWC2L,EFNA5,NGEF,TRPC5,NEUROD2,PTPRF,ARHGEF28,GPRIN1,OPCML,GRID2,RB1,KIRREL3,GFRA1,ETV1,LRTM2,KAT2B,ANKS1A,CDK5RAP2,BDNF,SEMA3E,TENM1,RORB,GABRB1,AVIL,CLASP2,ZNF521,SEMA6A,EIF4G1,ITGA4,ROCK1,DGKG,ELAVL4,NIN,PRKD1,HHIP,TMEM30A,SEMA5A,METTL3,PARD3,ATP8B1,RNF6,SEMA3C,VPS13B,SPART*
GO:0009653	anatomical structure	0.00000660221509574280	*APBB2,HS3ST3A1,MAP3K3,ISM1,MAP3K13,ARL13B,LIMD1,CHST11,THRB,SPTBN1,CDC42EP3,CHRN*

	morphogenesis	7	*B2,CDH8,AGTPBP1,FHL1,ATP10A,SH3PXD2A,SPINK5,CAMK2B,GRIP1,NOTCH2,TMOD2,SDK2,NPNT,EPB41L3,MAP6,PARVB,CDH11,SHANK3,NR4A3,E2F7,ADAMTS12,PLXNA4,YBX1,CACNA1C,ASB4,PRKN,IFT172,PTBP3,CDON,SLC17A6,THSD7A,TIAM2,CNMD,LRP8,CNTN1,MEOX2,ENPEP,RHOJ,FGF10,TSPAN18,CDH7,KDM2B,COL11A1,COL1A2,TMOD3,COL12A1,ABLIM1,ALPK2,CHRNA7,HECW1,ZNF304,AFF3,RFLNA,ATRX,TNIK,TGFB2,LAMA2,TRIO,HOXC13,SMARCC1,NDST1,MUSK,JAM3,EPHA5,SKOR2,ENAH,SNX10,ALDH1A2,FAM171A1,HUWE1,ASPH,SOX30,CHL1,PLS1,SLIT3,ANKRD11,ITPR1,CPM,PAK3,TNFRSF12A,NCKAP1,PRPF40A,FOSL2,EPHA3,LATS2,MYH14,ARHGAP18,MYOM2,ADGRB3,KLF7,DIP2B,PDZD8,NOS3,VSX1,EIF2AK4,MATN2,TGFBR2,CUX1,EPB41L5,WHRN,APP,GATA4,SMAD5,IGF2BP1,ADAM12,HCN1,FRY,PALLD,CECR2,RIPK4,PTK2,LRRK2,PKHD1,RARB,TIAM1,CSRP3,PHACTR1,ACVR2A,NEDD4L,KIT,EPHA7,SPAG9,WNT5A,CTNNB1,PRKDC,ESR1,PTPN14,SCFD1,POFUT2,BCAR3,LMX1A,GREB1L,DOCK1,EFNA5,IGF2BP2,SYNE3,NGEF,TRPC5,LYVE1,APOH,ARHGEF28,TASOR,ATF2,ACVR1,PID1,GRID2,RB1,PLPP3,HNF1B,KIRREL3,ETV1,LRTM2,WLS,PRCP,MEF2D,FLVCR1,ARHGEF7,NPHP1,BDNF,TFCP2L1,SEMA3E,ADAMTS9,STIM1,PPARG,TANC1,RORB,SH3KBP1,TTC39C,SIPA1L3,SFRP4,JCAD,VAV2,TFRC,FBN1,BMPER,CLASP2,ANGPT1,SEMA6A,ITGA4,ROCK1,PML,SP100,ELAVL4,CDH9,NIN,AIMP1,PRKD1,CALCRL,STK4,ANPEP,TTN,HHIP,EYA4,SEMA5A,FMNL2,METTL3,PARD3,ATP8B1,RNF6,SEMA3C,VPS13B,SPART*
GO:0051234	establishment of localization	0.000007557147890921029	*ESYT1,MREG,NOSTRIN,OSBPL8,SLC12A8,STX8,SMYD3,BLTP3B,TRAPPC9,SLC16A5,PPM1A,RN7SL556P,IMMP1L,TF,RCSD1,SPTBN1,MYO5C,CACNB1,CHRNB2,KCNQ5,AGTPBP1,FHL1,ATP10A,ATP7B,KCNJ6,CAMK2B,GRIP1,SLC38A9,RGPD2,ABCG8,XPO4,CD93,SLC44A5,NPNT,MAP6,NR4A3,SLC14A2,FOXO1,STK39,YBX1,CACNA1C,PRKN,NLGN4X,XPO6,SLC44A3,SLC5A7,IFT172,GPSM1,SLC17A6,MAPKAPK3,INTS6-AS1,CFAP54,TRAK2,LRP8,CNTN1,STAT5B,UBE2O,EIF4ENIF1,CHRFAM7A,ERBIN,MCC,PTH,ADAR,CLCN3,RHOJ,CDC37,FGF10,ZFYVE16,TSPAN18,ILDR2,CD84,GABRA5,RGPD3,ANO3,CIDEB,SLC45A1,MCPH1,MYO1D,SESTD1,XPO7,KCNS3,CHRNA7,HMGB1,DYNLT2B,TGFB2,MDFIC,KCNJ18,KCNIP3,NUP93,RBM33,ATP11A,ATP4A,SLC15A4,GOLPH3,PRKG2,MBTPS1,NCF2,EPHA5,ATP13A4,SNX10,ATP2C1,TMEM230,STON2,STRADA,HUWE1,ASPH,ZDHHC4,UBR5,SOX30,GSN,HPS3,ITPR1,DNAAF11,PRKAG2,HAVCR1,TRAPPC10,CPT2,VPS8,MTX3,CILK1,FOSL2,EPHA3,ERP29,UVRAG,LYN,USP6,KLF7,GRIK1,SEC24C,ATP6V1H,NUP58,SLC12A1,PDZD8,SIL1,C21ORF62,RNASEL,CORIN,TMCC1,TMED7-TICAM2,SLC41A2,NOS3,NOMO2,RN7SL738P,XKR6,MAGEL2,TGFBR2,MDN1,SYNJ1,TCF7L2,CUX1,UNC119,GPM6B,CACNA2D4,RGPD1,KPNB1,GSDMD,ADGRV1,EPB41L5,SCG5,WHRN,OCA2,DAPK1,SAR1B,APP,IGF2BP1,HCN1,CHRM5,SLC13A1,PI4KB,RHOT1,USE1,HOOK3,CECR2,TMCO3,AVPR1B,LMAN1L,PTK2,NOP9,USP7,TICAM2,SCP2,ATP1B4,PIP4K2B,LRRK2,GET1-SH3BGR,SLC38A4,PLD1,PKHD1,MGST1,TIAM1,NEDD4L,ENSG00000288683,SNX13,RABGEF1,KIT,C*

			YB5R4,OSBPL3,SPAG9,SPDL1,WNT5A,PEX26,TEX2,ARFGEF2,CTNNB1,IPO5,PTPN14,SCFD1,ERC2,POFUT2,VPS53,FRMD4A,BEST3,DOCK1,BPGM,PKIG,EFNA5,IGF2BP2,SYNE3,PLCH1,TRPC5,AKAP6,LYVE1,APOH,RAB3GAP2,CLCN1,CEMIP,XIST,ATF2,KCNJ12,CHRNB3,PID1,CCDC91,GRID2,KCNJ3,RB1,MPV17,PDE4B,PKD1L3,KIF13A,GABRB3,GOSR2,SMG7,PLPP3,UNC80,HNF1B,IMMP2L,LAMP2,GABRG2,TMEM120A,BNIP3L,ACACB,RABGAP1L,KCND2,SLC24A3,GRIA4,RAB18,BACH2,MGRN1,SIK1,KAT2B,GOLPH3L,WLS,RYR3,MYO1B,CDK5RAP2,FLVCR1,ARHGEF7,TRDN,SLC26A8,SRI,ATP1B1,SCIN,ADAMTS9,STIM1,RAB11FIP2,PPARG,MTTP,CHRNB4,TENM1,IL13RA2,GABRB1,MYOF,JAKMIP1,SH3KBP1,NALCN,GDAP1,KIF2C,MYO5A,SLC6A13,SPTBN5,SFRP4,BLTP1,VAV2,TFRC,VPS13A,OSBPL6,GRIA3,PNPLA8,RAB31,FUT10,CLASP2,ANGPT1,ATP6V0A4,ITGA4,DNAJC6,ROCK1,TBC1D4,KCNT2,NUP98,PML,SCFD2,SCARA5,SLC5A12,SP100,NDUFAF2,ANKFY1,ENSA,AIMP1,SLC30A10,AP4S1,PRKD1,CALCRL,SLC20A2,STK4,RAB6D,SPC25,SCAMP1,PRLR,RAB6C,IL16,CTBP2,TTN,NCALD,TMEM30A,MYRIP,UNC13C,KCNE2,TTPA,PARD3,KCNN4,CP,ATP8B1,VAV1,GRIA2,VDAC2,VPS13B*
GO:0050793	regulation of developmental process	0.0000094050322875597 32	*RGN,LRRN1,ISM1,MAP3K13,LIMD1,CDC42EP3,CHRNB2,SEZ6,ATP10A,SPINK5,BRD4,CAMK2B,GRIP1,DCT,NOTCH2,NPNT,EPB41L3,MAP6,PARVB,SHANK3,ARID1B,FOXO1,SMURF2,ADAMTS12,PLXNA4,YBX1,ASB4,PRKN,PTBP3,CDON,TIAM2,CNMD,LRP8,STAT5B,EIF4ENIF1,PTH,RHOJ,FGF10,TSPAN18,NREP,KDM2B,PREX1,MRTFB,ALPK2,CHRNA7,HECW1,HMGB1,ZNF304,RFLNA,TNIK,TGFB2,LAMA2,TRIO,SMARCC1,MUSK,ATP11A,PRKG2,SKOR2,FAM171A1,HUWE1,PLS1,PAK3,ABI3BP,CCNT2,PRPF40A,FOSL2,EPHA3,LATS2,MYH14,ARHGAP18,LYN,ADGRB3,KLF7,MTA3,DIP2B,PDZD8,SGMS2,NOS3,PRAMEF27,EIF2AK4,TGFBR2,TCF7L2,ZFHX3,CUX1,GPM6B,ADGRV1,ST7,PRUNE1,APP,GATA4,SMAD5,ADAM12,HOOK3,PTK2,CELF1,LRRK2,INO80D,IKZF3,CLSTN2,PKHD1,TENT5C,RARB,TCF4,PAEP,TIAM1,CSRP3,ACVR2A,NEDD4L,KIT,EPHA7,SPAG9,WNT5A,CTNNB1,ANKRD17,RUNX1T1,CTSK,PRKDC,ESR1,POFUT2,LMX1A,VWC2L,EFNA5,SYNE3,NGEF,TRPC5,NEUROD2,KAT14,PTPRF,IKBKB,AKAP6,USF3,APOH,LINGO2,ATF2,ACVR1,PID1,GRID2,RB1,ADIPOR1,HNF1B,NCOA2,ACACB,XRCC5,SHLD2,TEX11,LRTM2,SIK1,KAT2B,CDK5RAP2,FLVCR1,ARHGEF7,ARNT,BDNF,LDLRAD4,SEMA3E,SCIN,ADAMTS9,STIM1,LAG3,PPARG,RORB,SH3KBP1,SFRP4,BLTP1,IL1RAP,JCAD,TFRC,FBN1,IQCB1,BMPER,CLASP2,SEMA6A,EIF4G1,ROCK1,PML,TNFSF4,DGKG,SP100,ELAVL4,UMODL1,NIN,PRKD1,SLC20A2,STK4,PRLR,CUL4A,SEMA5A,LAMA4,TTPA,GATAD2B,FMNL2,METTL3,PARD3,RNF6,SEMA3C,SMYD1,SPART*
GO:0030182	neuron differentiation	0.0000117283484532974 95	*APBB2,RTN1,TRAPPC9,MAP3K13,THRB,CHRNB2,SEZ6,AGTPBP1,SPINK5,CAMK2B,GRIP1,NOTCH2,SDK2,EPB41L3,MAP6,CDH11,SHANK3,STYXL1,PLXNA4,PRKN,NLGN4X,IFT172,CDON,SERPINI1,NCAM2,TIAM2,LRP8,CNTN1,EIF4ENIF1,NCK1,NREP,GABRA5,PREX1,CHRNA7,HECW1,HMGB1,TNIK,TGFB2,LAMA2,TRIO,MUSK,ALKBH1,EPHA5,SKOR2,ENAH,ALDH1A2,CHL1,PLS1,SLIT3,PAK3,BTG4,NCKAP1,EPHA3,LYN,ADGRB3,KLF7,DIP2B,VSX1,ERC*

			C6,EIF2AK4,MATN2,CRTAC1,ZFHX3,CUX1,GPM6B,ADGRV1,WHRN,TDP2,APP,IGF2BP1,HCN1,FRY,PALLD,CECR2,PTK2,LRRK2,TCF4,TIAM1,PHACTR1,NTM,NEDD4L,KIT,EPHA7,SPAG9,WNT5A,CTNNB1,LMX1A,VWC2L,EFNA5,NGEF,TRPC5,NEUROD2,PTPRF,ARHGEF28,GPRIN1,OPCML,GRID2,RB1,KIRREL3,GFRA1,ETV1,LRTM2,KAT2B,ANKS1A,CDK5RAP2,BDNF,SEMA3E,TENM1,RORB,GABRB1,AVIL,CLASP2,ZNF521,SEMA6A,EIF4G1,ITGA4,ROCK1,DGKG,ELAVL4,NIN,PRKD1,TMEM30A,SEMA5A,PARD3,ATP8B1,RNF6,SEMA3C,VPS13B,SPART
GO:0050794	regulation of cellular process	0.00001190444212591023	*FSTL1,C10ORF90,APBB2,STYK1,RTN1,RGN,CDK14,PDE1C,NOSTRIN,ING3,MAP3K3,OSBPL8,LRRN1,STX8,PHF20,FOXK2,SMYD3,RAPGEF6,ARHGEF9,TAFA2,ABR,MAOA,MAP3K13,PPM1A,ARL13B,SAMSN1,LIMD1,TF,CHST11,THRB,USP42,SPTBN1,CDC42EP3,MAPK10,CACNB1,USP32,DAPP1,ATF7,GNG12,CHRNB2,CDC14C,SEZ6,CDH8,GNB4,AGTPBP1,ATF7-NPFF,FHL1,RBPMS,ATP10A,SPINK5,OPA3,SKAP2,GPBP1L1,KCNJ6,BRD4,DOK4,CAMK2B,GRIP1,ARHGAP11A-SCG5,DCT,SLC38A9,ZNF264,HBP1,SDR16C5,ABCG8,XPO4,MAP4K3,NOTCH2,TMOD2,HMBOX1,ZNF551,NPNT,MAP6,MORC1,CDH11,SHANK3,NR4A3,ARID1B,E2F7,FOXO1,ZNF611,STYXL1,STK39,SMURF2,ADAMTS12,FER1L6,TBC1D16,PLXNA4,CDK18,URB2,YBX1,DIPK2A,DCDC1,CACNA1C,FAM3C,ASB4,IDO1,ARB2A,PRKN,NLGN4X,TAFA1,PIK3C2B,KDM7A,FAM83B,IFT172,GPSM1,CBLL2,PTBP3,CDON,SERPINI1,NLK,GNAI1,MRPL13,MAPKAPK3,TIAM2,ZNF10,CNMD,LRP8,TAGLN3,CNTN1,MEOX2,STAT5B,ARHGEF4,UBE2O,DDIAS,CPEB4,EIF4ENIF1,CHRFAM7A,ERBIN,ZNF121,MBTPS2,RGS22,CD247,MCC,PTH,ADAR,PDE10A,TM9SF2,CLCN3,RHOJ,CDC37,FGF10,ZFYVE16,MECOM,COL4A6,STK32B,PLG,HERC5,UBE2E2,ILDR2,NCK1,CD84,FAM168A,NONO,RARRES1,KLHL42,NREP,GABRA5,NEXMIF,MTCP1,KDM2B,PREX1,ZNF404,WRNIP1,RSF1,NCOA7,CIDEB,MCPH1,SESTD1,ZNF214,COL1A2,DTNA,TMOD3,GEMIN5,MRTFB,ALPK2,CHRNA7,HECW1,HMGB1,ZNF304,DYNLT2B,CDIP1,AFF3,RFLNA,HIVEP1,ATRX,TNIK,TGFB2,MDFIC,LAMA2,ADD3,TRIO,ZNF85,HOXC13,SMARCC1,NDST1,MUSK,KCNJ18,JAM3,KCNIP3,SFPQ,ALKBH1,RGS3,NUP93,ZCCHC4,RERG,OR5AU1,PRKAR2A,RBM33,CTIF,ATP11A,RBM23,ZNF154,ATP4A,SLC15A4,GOLPH3,PRKG2,MBTPS1,USP10,SND1,IGFBP7,EPHA5,SKOR2,ATP2C1,ALDH1A2,PPTC7,STON2,PTGFR,STRADA,HUWE1,ZNF595,ASPH,RRAS2,SH3BGRL,UBR5,GTF2F2,SIM1,SOX30,ANKS1B,CHL1,SPOCK3,GSN,PLS1,TCP11L1,SPON1,MAPRE3,TLR8,SLIT3,ARMH3,CACUL1,NLRP7,ARHGEF39,CD226,PHC2,ZNF578,ITPR1,PKP4,PAK3,ABI3BP,KLF12,PRKAG2,MLLT3,CCNT2,BTG4,HAVCR1,CNOT10,MR1,TNFRSF12A,OASL,CPT2,TAS2R14,VPS8,ZNF429,CXCR5,NCKAP1,PRPF40A,CILK1,FOSL2,IL1RAPL2,ADGRD1,EPHA3,LATS2,SPDYE2B,ERP29,CDYL2,ARHGAP18,UVRAG,LYN,USP6,ADGRB3,KLF7,SUMO2,GRIK1,MTA3,DIP2B,NME7,ATP6V1H,NUP58,FIGNL1,RRM2,LDB2,MYRFL,ALDH1A1,FMO5,RNASEL,DBF4B,DCP2,TMED7-TICAM2,NOS3,VSX1,ZNF280B,TNRC6C,PRAMEF27,MLLT10,ERCC6,EIF2AK4,KIAA1614,RAB12,MAG*

			EL2,TGFBR2,ITGA11,CMKLR2,MARK4,SYNJ1,TCF7L2,ZNF462,E2F6,ZFHX3,CUX1,UNC119,SHISA2,GPM6B,RRAGD,ULK3,SPDYE2,GPR156,GKAP1,PPP2R5E,GSDMD,ADGRV1,ST7,RASGRF2,SERBP1,EPB41L5,SCG5,WAC,WHRN,DAPK1,TDP2,ZNF678,PRUNE1,SAR1B,INSYN2A,BTG3,APP,GATA4,ATF7IP2,SMAD5,IGF2BP1,MAMLD1,GSKIP,ADAM12,PELI2,SP140L,HCN1,CHRM5,PI4KB,FRY,RHOT1,ELOVL5,HOOK3,PIK3R5,TLR1,TAS2R30,BMT2,RIPK4,AVPR1B,PTK2,USP7,TICAM2,SCP2,SH2B2,ATP1B4,AVEN,ZNF41,CELF1,POGZ,PIP4K2B,LRRK2,INO80D,ZNF395,IKZF3,CLSTN2,ARIH1,CFAP298,PLD1,VTCN1,ITFG2,PKHD1,TENT5C,RARB,PJA2,TCF4,PAEP,COP1,TIAM1,CSRP3,PHACTR1,MLIP,IQSEC2,ACVR2A,GNAO1,TIPIN,VIT,RASSF4,NEDD4L,BRCC3,SNX13,RABGEF1,KIT,TBC1D8,EPHA7,SPAG9,ANTXR1,SPDL1,WNT5A,TRABD2B,TEX2,ARFGEF2,CTNNB1,ANKRD17,RUNX1T1,CTSK,PRKDC,IPO5,ESR1,TNFRSF19,ZNF479,PTPN14,SCFD1,ERC2,POFUT2,BCAR3,TPD52L1,L3MBTL4,PPM1E,ZXDC,FRMD4A,LMX1A,BNIPL,HUNK,PTGER4,ZNF215,PDE7B,BBX,VWC2L,DOCK1,GPC4,PKIG,EFNA5,IGF2BP2,CDC14B,ZNF722,PLCH1,OR1M1,HECTD1,NGEF,TRPC5,NEUROD2,FNIP2,AASS,JAML,KAT14,PTPRF,IKBKB,FXR2,SH3BP5,AKAP6,TRBV10-2,USF3,LYVE1,APOH,ARHGEF28,RAB3GAP2,TASOR,CCPG1,LINGO2,CEMIP,XIST,ATF2,KCNJ12,ZNF426,HDAC8,ACVR1,CHRNB3,HLA-DQA2,PID1,EBF2,GRID2,ZNF423,ZCCHC17,KCNJ3,PLPPR1,RB1,NSMCE1,MPV17,IFNLR1,PDE4B,KIF13A,ADIPOR1,GABRB3,ZNF143,ZSCAN23,SMG7,PLPP3,HNF1B,ZNF615,LAMP2,NCOA2,BNIP3L,ACACB,RABGAP1L,XRCC5,TBRG4,RGS17,SHLD2,GFRA1,INPP4A,BMX,ZNF418,SLC24A3,TAF15,GRIA4,RAB18,ZNF860,POU2F1,ETV1,RGS7BP,BACH2,MGRN1,TEX11,MID2,MIR198,LRTM2,SIK1,ZNF850,TP53BP2,KAT2B,GOLPH3L,WLS,ANKS1A,RYR3,PRCP,SUPV3L1,MEF2D,GTF2A1,SPTLC1,CDK5RAP2,RFC3,ARHGEF7,SAMD12,DACH1,ATF3,TRDN,NPHP1,IKZF1,CAPZA2,SRI,ATP1B1,ARNT,BDNF,LDLRAD4,TFCP2L1,SEMA3E,BCLAF3,SCIN,MACO1,ZNF665,TLR6,ADAMTS9,STIM1,TMEM237,HAUS6,RAB11FIP2,EEFSEC,LAG3,PPARG,TANC1,PAPPA,ASB3,CHRNB4,NEK7,ZNF80,TENM1,IL13RA2,RORB,GABRB1,MYOF,LTB4R2,SH3KBP1,NALCN,AVIL,USP25,KIF2C,MYO5A,SIPA1L3,ZNF286A-TBC1D26,LRFN2,SPTBN5,SFRP4,BLTP1,SPDYE16,PNRC1,IL1RAP,JCAD,VAV2,TFRC,TES,GRIA3,PNPLA8,SPDYE6,FBN1,HAPSTR1,RAB31,ZFP82,MAP3K1,DDX4,IQCB1,ZNF292,CLEC6A,BMPER,CLASP2,ZNF521,ZNF761,ANGPT1,SEMA6A,VIPR2,EIF4G1,PHLPP1,ZNF618,ITGA4,DNAJC6,ROCK1,NCAPH,TAX1BP1,TBC1D4,APIP,MMP28,ZNF701,NUP98,IKZF2,PML,FOXO3B,MGLL,PLEK2,TNFSF4,AFAP1L2,DGKG,SP100,ELAVL4,NDUFAF2,CFAP298-TCP10L,UMODL1,RFPL4B,TTC28,NIN,SPG21,ZNF286A,ANKFY1,ZNF69,ENSA,MIR9-2HG,KHDRBS3,AIMP1,SLC30A10,PRKD1,HSPBP1,CALCRL,USP34,STK4,SPC25,PRLR,ZNF569,ZNF180,PDE11A,NMI,RAB6C,YLPM1,IL16,ZNF470,EDARADD,GNA14,CR2,LPAR6,CTBP2,TTN,NCALD,HHIP,DYRK1A,PPFIA1,TMEM30A,MYRIP,PPP1R1C,UNC13C,KCNE2,RGS10,CUL4A,KCTD16,ZNF888,EYA4,ZNF736,SEMA5A,ELL2,LAMA4,PMEPA1,TTPA,

			PVT1,GATAD2B,METTL3,UAP1,PARD3,KCNN4,ZNF33A,CP,BMAL2,ATP8B1,RNF6,SEMA3C,VAV1,SMYD1,PEPD,GRIA2,VDAC2,SPART
GO:0031175	neuron projection development	0.0000150880824519811 73	*APBB2,MAP3K13,CHRNB2,SEZ6,CAMK2B,GRIP1,NOTCH2,EPB41L3,MAP6,CDH11,SHANK3,STYXL1,PLXNA4,PRKN,SERPINI1,NCAM2,TIAM2,LRP8,CNTN1,NCK1,NREP,PREX1,CHRNA7,HECW1,HMGB1,TNIK,LAMA2,TRIO,MUSK,ALKBH1,EPHA5,SKOR2,ENAH,CHL1,PLS1,SLIT3,PAK3,NCKAP1,EPHA3,LYN,ADGRB3,KLF7,DIP2B,ERCC6,EIF2AK4,MATN2,CRTAC1,CUX1,GPM6B,ADGRV1,WHRN,APP,IGF2BP1,FRY,PALLD,CECR2,PTK2,LRRK2,TIAM1,PHACTR1,NEDD4L,KIT,EPHA7,SPAG9,WNT5A,CTNNB1,LMX1A,EFNA5,NGEF,TRPC5,NEUROD2,PTPRF,ARHGEF28,GPRIN1,GRID2,RB1,KIRREL3,GFRA1,ETV1,LRTM2,KAT2B,BDNF,SEMA3E,AVIL,CLASP2,SEMA6A,ITGA4,ROCK1,DGKG,ELAVL4,NIN,PRKD1,TMEM30A,SEMA5A,PARD3,RNF6,SEMA3C,VPS13B,SPART*
GO:0006796	phosphate-containing compound metabolic process	0.0000172498384777889 57	*PFKP,STYK1,RGN,CDK14,MAP3K3,OSBPL8,FOXK2,SMYD3,GARS1,ABR,MAP3K13,PPM1A,SAMSN1,LIMD1,TF,MAPK10,DAPP1,CDC14C,PIGL,PPM1H,CAMK2B,PGM1,MAP4K3,FPGT-TNNI3K,SLC44A5,STYXL1,STK39,CDK18,DIPK2A,FAM20B,IDO1,PRKN,PNPLA3,PIK3C2B,SLC44A3,CDON,NLK,MAPKAPK3,LRP8,NUDT19,PTH,ADAR,PDE10A,PDHX,CDC37,FGF10,STK32B,HERC5,NUDT13,NCK1,PUDP,MTCP1,ALPK2,CHRNA7,TNIK,TGFB2,TRIO,MUSK,PRKAR2A,PRKG2,PIGV,RSKR,EPHA5,PTPN20,PPTC7,STRADA,MAPRE3,TLR8,CACUL1,PAK3,IPMK,CAMK1G,PRKAG2,CCNT2,GDPD1,CILK1,EPHA3,LATS2,SPDYE2B,ERP29,UVRAG,LYN,NME7,FIGNL1,RRM2,LDB2,NMNAT3,FMO5,RNASEL,SGMS2,NOS3,ERCC6,EIF2AK4,TGFBR2,MARK4,SYNJ1,UNC119,ULK3,SPDYE2,GKAP1,DAPK1,ACSM2B,TTC7B,APP,GSKIP,PELI2,CHRM5,PI4KB,ELOVL5,PIK3R5,RIPK4,AVPR1B,PTK2,SCP2,HYCC1,PIP4K2B,LRRK2,PLD1,ACVR2A,DUSP14,RABGEF1,KIT,EPHA7,SPAG9,WNT5A,PRKDC,IPO5,PTPN14,BCAR3,TPD52L1,PPM1E,TPST2,HUNK,PDE7B,BPGM,PKIG,EFNA5,CDC14B,TRPC5,FNIP2,AASS,PTPRF,IKBKB,FXR2,CEMIP,ATF2,ACVR1,PID1,PLPPR1,RB1,PDE4B,SMG7,PLPP3,AK5,CPS1,ACACB,AGPAT5,XRCC5,GFRA1,INPP4A,BMX,TPST1,NRK,SIK1,PIPOX,KAT2B,SPTLC1,ATP1B1,ARNT,BDNF,TLR6,PPARG,NEK7,TENM1,SH3KBP1,LPCAT2,MYLK4,SPDYE16,TFRC,PNPLA8,SPDYE6,MAP3K1,CLEC6A,ANGPT1,EIF4G1,ACSM5,RBKS,DNAJC6,ROCK1,DGKG,GALK2,TPK1,PRKD1,STK4,TNNI3K,PRLR,TTN,DYRK1A,SERINC1,ETNK1,UAP1,PARD3*
GO:0006793	phosphorus metabolic process	0.0000189510986363377 9	*PFKP,STYK1,RGN,CDK14,MAP3K3,OSBPL8,FOXK2,SMYD3,GARS1,ABR,MAP3K13,PPM1A,SAMSN1,LIMD1,TF,MAPK10,DAPP1,CDC14C,PIGL,PPM1H,CAMK2B,PGM1,MAP4K3,FPGT-TNNI3K,SLC44A5,STYXL1,STK39,CDK18,DIPK2A,FAM20B,IDO1,PRKN,PNPLA3,PIK3C2B,SLC44A3,CDON,NLK,MAPKAPK3,LRP8,NUDT19,PTH,ADAR,PDE10A,PDHX,CDC37,FGF10,STK32B,HERC5,NUDT13,NCK1,PUDP,MTCP1,ALPK2,CHRNA7,TNIK,TGFB2,TRIO,MUSK,PRKAR2A,PRKG2,PIGV,RSKR,EPHA5,PTPN20,PPTC7,STRADA,MAPRE3,TLR8,CACUL1,PAK3,IPMK,CAMK1G,PRKAG2,CCNT2,GDPD1,CILK1,EPHA3,LATS2,SPDYE2B,ERP29,UVRAG,LYN,NME7,FIGNL1,RRM2,LDB2,NMNAT3,FMO5,RNASE*

			L,SGMS2,NOS3,ERCC6,EIF2AK4,TGFBR2,MARK4,SYNJ1,UNC119,ULK3,SPDYE2,GKAP1,DAPK1,ACSM2B,TTC7B,APP,GSKIP,PELI2,CHRM5,PI4KB,ELOVL5,PIK3R5,RIPK4,AVPR1B,PTK2,SCP2,HYCC1,PIP4K2B,LRRK2,PLD1,ACVR2A,DUSP14,RABGEF1,KIT,EPHA7,SPAG9,WNT5A,PRKDC,IPO5,PTPN14,BCAR3,TPD52L1,PPM1E,TPST2,HUNK,PDE7B,BPGM,PKIG,EFNA5,CDC14B,TRPC5,FNIP2,AASS,PTPRF,IKBKB,FXR2,CEMIP,ATF2,ACVR1,PID1,PLPPR1,RB1,PDE4B,SMG7,PLPP3,AK5,CPS1,ACACB,AGPAT5,XRCC5,GFRA1,INPP4A,BMX,TPST1,NRK,SIK1,PIPOX,KAT2B,SPTLC1,ATP1B1,ARNT,BDNF,TLR6,PPARG,NEK7,TENM1,SH3KBP1,LPCAT2,MYLK4,SPDYE16,TFRC,PNPLA8,SPDYE6,MAP3K1,CLEC6A,ANGPT1,EIF4G1,ACSM5,RBKS,DNAJC6,ROCK1,DGKG,GALK2,TPK1,PRKD1,STK4,TNNI3K,PRLR,TTN,DYRK1A,SERINC1,ETNK1,UAP1,PARD3
GO:0016310	phosphorylation	0.00003096789358501955	*PFKP,STYK1,RGN,CDK14,MAP3K3,SMYD3,ABR,MAP3K13,SAMSN1,LIMD1,TF,MAPK10,CAMK2B,MAP4K3,FPGT-TNNI3K,STK39,CDK18,DIPK2A,FAM20B,PRKN,PIK3C2B,CDON,NLK,MAPKAPK3,LRP8,ADAR,CDC37,FGF10,STK32B,HERC5,NCK1,MTCP1,ALPK2,CHRNA7,TNIK,TGFB2,TRIO,MUSK,PRKAR2A,PRKG2,RSKR,EPHA5,STRADA,MAPRE3,TLR8,CACUL1,PAK3,IPMK,CAMK1G,PRKAG2,CCNT2,CILK1,EPHA3,LATS2,SPDYE2B,ERP29,UVRAG,LYN,NME7,LDB2,RNASEL,SGMS2,ERCC6,EIF2AK4,TGFBR2,MARK4,UNC119,ULK3,SPDYE2,GKAP1,DAPK1,APP,GSKIP,PELI2,PI4KB,PIK3R5,RIPK4,PTK2,PIP4K2B,LRRK2,ACVR2A,RABGEF1,KIT,EPHA7,SPAG9,WNT5A,PRKDC,IPO5,BCAR3,TPD52L1,PPM1E,HUNK,PKIG,EFNA5,TRPC5,FNIP2,IKBKB,FXR2,CEMIP,ATF2,ACVR1,PID1,RB1,PLPP3,AK5,XRCC5,GFRA1,BMX,NRK,SIK1,BDNF,TLR6,PPARG,NEK7,TENM1,SH3KBP1,MYLK4,SPDYE16,TFRC,SPDYE6,MAP3K1,CLEC6A,ANGPT1,EIF4G1,RBKS,ROCK1,DGKG,GALK2,TPK1,PRKD1,STK4,TNNI3K,PRLR,TTN,DYRK1A,ETNK1,PARD3*
GO:0048518	positive regulation of biological process	0.00009291241620752101	*APBB2,RGN,ING3,MAP3K3,OSBPL8,LRRN1,PHF20,FOXK2,SMYD3,MAOA,MAP3K13,PPM1A,LIMD1,TF,THRB,SPTBN1,CDC42EP3,CACNB1,USP32,ATF7,CHRNB2,CDC14C,AGTPBP1,FHL1,RBPMS,ATP10A,GPBP1L1,BRD4,DOK4,CAMK2B,GRIP1,DCT,SLC38A9,XPO4,NOTCH2,TMOD2,HMBOX1,NPNT,MAP6,SHANK3,NR4A3,ARID1B,E2F7,FOXO1,STYXL1,STK39,SMURF2,FER1L6,PLXNA4,YBX1,DIPK2A,ASB4,IDO1,PRKN,KDM7A,FAM83B,IFT172,GPSM1,CDON,SERPINI1,GNAI1,MAPKAPK3,TIAM2,LRP8,CNTN1,MEOX2,STAT5B,UBE2O,EIF4ENIF1,ERBIN,MBTPS2,CD247,PTH,ADAR,CLCN3,RHOJ,FGF10,MECOM,PLG,UBE2E2,NCK1,CD84,FAM168A,NONO,MTCP1,KDM2B,PREX1,RSF1,NCOA7,CIDEB,TMOD3,MRTFB,CHRNA7,HMGB1,ZNF304,HIVEP1,ATRX,TNIK,TGFB2,MDFIC,LAMA2,ADD3,HOXC13,SMARCC1,NDST1,MUSK,JAM3,SFPQ,NUP93,ZCCHC4,CTIF,ATP11A,RBM23,SLC15A4,GOLPH3,PRKG2,SKOR2,ATP2C1,ALDH1A2,PTGFR,STRADA,HUWE1,ASPH,RRAS2,SH3BGRL,UBR5,GTF2F2,SOX30,GSN,PLS1,SPON1,MAPRE3,TLR8,CACUL1,ARHGEF39,CD226,ITPR1,PKP4,PAK3,ABI3BP,KLF12,PRKAG2,MLLT3,CCNT2,HAVCR1,CNOT10,MR1,TNFRSF12A,OASL,CPT2,CXCR5,NCKAP1,FOSL2,EPHA3,LATS2,SPDYE2B,ERP29,UVRAG,LYN,ADGRB3,KLF7,SUMO2,MTA3*

			,DIP2B,RRM2,LDB2,MYRFL,RNASEL,DBF4B,DCP2,TMED7-TICAM2,NOS3,TNRC6C,PRAMEF27,MLLT10,ERCC6,EIF2AK4,MAGEL2,TGFBR2,MARK4,SYNJ1,TCF7L2,ZNF462,ZFHX3,CUX1,UNC119,GPM6B,RRAGD,ULK3,SPDYE2,GKAP1,GSDMD,ADGRV1,RASGRF2,EPB41L5,WAC,WHRN,DAPK1,SAR1B,APP,GATA4,SMAD5,IGF2BP1,GSKIP,ADAM12,PELI2,ELOVL5,PIK3R5,TLR1,BMT2,AVPR1B,PTK2,TICAM2,SCP2,SH2B2,CELF1,POGZ,PIP4K2B,LRRK2,INO80D,ZNF395,IKZF3,CLSTN2,ARIH1,PLD1,VTCN1,PKHD1,TENT5C,RARB,PJA2,TCF4,PAEP,COP1,TIAM1,CSRP3,MLIP,IQSEC2,ACVR2A,TIPIN,VIT,NEDD4L,BRCC3,RABGEF1,KIT,TBC1D8,EPHA7,SPAG9,WNT5A,TRABD2B,ARFGEF2,CTNNB1,ANKRD17,CTSK,PRKDC,IPO5,ESR1,TNFRSF19,ERC2,POFUT2,BCAR3,TPD52L1,PPM1E,ZXDC,FRMD4A,LMX1A,PTGER4,VWC2L,DOCK1,EFNA5,IGF2BP2,CDC14B,HECTD1,TRPC5,NEUROD2,FNIP2,JAML,IKBKB,FXR2,AKAP6,USF3,LYVE1,APOH,RAB3GAP2,CCPG1,LINGO2,CEMIP,ATF2,ACVR1,HLA-DQA2,PID1,EBF2,GRID2,ZNF423,RB1,IFNLR1,PDE4B,ADIPOR1,ZNF143,PLPP3,HNF1B,ZNF615,LAMP2,NCOA2,BNIP3L,ACACB,XRCC5,TBRG4,SHLD2,GFRA1,BMX,SLC24A3,TAF15,POU2F1,ETV1,MID2,LRTM2,SIK1,ZNF850,TP53BP2,KAT2B,GOLPH3L,WLS,SUPV3L1,MEF2D,GTF2A1,SPTLC1,CDK5RAP2,RFC3,ARHGEF7,ATF3,TRDN,NPHP1,SRI,ATP1B1,ARNT,BDNF,TFCP2L1,SEMA3E,BCLAF3,SCIN,TLR6,ADAMTS9,STIM1,RAB11FIP2,LAG3,PPARG,CHRNB4,NEK7,TENM1,RORB,SH3KBP1,NALCN,AVIL,SFRP4,SPDYE16,IL1RAP,JCAD,VAV2,TFRC,SPDYE6,RAB31,ZNF292,CLEC6A,BMPER,CLASP2,ZNF521,ANGPT1,SEMA6A,EIF4G1,ITGA4,ROCK1,NCAPH,TAX1BP1,NUP98,PML,PLEK2,TNFSF4,AFAP1L2,SP100,ELAVL4,NIN,SPG21,ANKFY1,AIMP1,SLC30A10,PRKD1,HSPBP1,CALCRL,SLC20A2,USP34,STK4,PRLR,NMI,IL16,CR2,LPAR6,CTBP2,TTN,DYRK1A,TMEM30A,MYRIP,KCNE2,CUL4A,EYA4,SEMA5A,ELL2,TTPA,PVT1,GATAD2B,METTL3,UAP1,PARD3,KCNN4,ZNF33A,BMAL2,RNF6,SEMA3C,VAV1,SMYD1*
GO:0022603	regulation of anatomical structure morphogenesis	0.00012254861586037953	*ISM1,MAP3K13,LIMD1,CDC42EP3,CHRNB2,ATP10A,SPINK5,CAMK2B,GRIP1,EPB41L3,MAP6,PARVB,SHANK3,ADAMTS12,PLXNA4,PRKN,TIAM2,CNMD,LRP8,RHOJ,FGF10,TSPAN18,CHRNA7,HECW1,ZNF304,TNIK,TGFB2,SKOR2,FAM171A1,HUWE1,PAK3,PRPF40A,MYH14,ARHGAP18,ADGRB3,DIP2B,PDZD8,NOS3,TGFBR2,CUX1,GATA4,ADAM12,PTK2,LRRK2,PKHD1,TIAM1,NEDD4L,KIT,EPHA7,SPAG9,WNT5A,CTNNB1,PRKDC,ESR1,EFNA5,SYNE3,NGEF,TRPC5,APOH,ATF2,PID1,ARHGEF7,BDNF,SEMA3E,ADAMTS9,STIM1,PPARG,SH3KBP1,JCAD,TFRC,BMPER,CLASP2,SEMA6A,ROCK1,PML,SP100,NIN,PRKD1,SEMA5A,FMNL2,RNF6,SEMA3C,SPART*
GO:0048522	positive regulation of cellular process	0.00012954561931580272	*APBB2,RGN,ING3,MAP3K3,OSBPL8,LRRN1,PHF20,FOXK2,SMYD3,MAOA,MAP3K13,PPM1A,TF,THRB,SPTBN1,CDC42EP3,USP32,ATF7,CHRNB2,CDC14C,AGTPBP1,RBPMS,ATP10A,GPBP1L1,BRD4,DOK4,CAMK2B,GRIP1,DCT,SLC38A9,XPO4,NOTCH2,TMOD2,HMBOX1,NPNT,MAP6,SHANK3,NR4A3,ARID1B,E2F7,FOXO1,STYXL1,STK39,SMURF2,FER1L6,PLXNA4,YBX1,DIPK2A,ASB4,IDO1,PRKN,KDM7A,FAM83B,IFT172,GPSM1,CDON,SERPINI1,GNAI1,TI*

			AM2,LRP8,CNTN1,MEOX2,STAT5B,UBE2O,EIF4ENIF1,MBTPS2,CD247,PTH,ADAR,CLCN3,RHOJ,FGF10,MECOM,PLG,UBE2E2,NCK1,CD84,FAM168A,MTCP1,KDM2B,PREX1,RSF1,NCOA7,CIDEB,TMOD3,MRTFB,CHRNA7,HMGB1,ZNF304,HIVEP1,ATRX,TNIK,TGFB2,MDFIC,LAMA2,ADD3,HOXC13,SMARCC1,NDST1,MUSK,JAM3,SFPQ,NUP93,ZCCHC4,CTIF,ATP11A,RBM23,SLC15A4,GOLPH3,PRKG2,SKOR2,ATP2C1,ALDH1A2,PTGFR,STRADA,HUWE1,ASPH,RRAS2,SH3BGRL,UBR5,GTF2F2,SOX30,GSN,PLS1,SPON1,MAPRE3,TLR8,CACUL1,ARHGEF39,CD226,ITPR1,PKP4,ABI3BP,KLF12,PRKAG2,MLLT3,CCNT2,HAVCR1,CNOT10,MR1,TNFRSF12A,OASL,CPT2,CXCR5,NCKAP1,FOSL2,EPHA3,LATS2,SPDYE2B,ERP29,UVRAG,LYN,ADGRB3,KLF7,SUMO2,MTA3,DIP2B,RRM2,LDB2,MYRFL,RNASEL,DBF4B,DCP2,NOS3,TNRC6C,PRAMEF27,MLLT10,ERCC6,EIF2AK4,MAGEL2,TGFBR2,MARK4,SYNJ1,TCF7L2,ZNF462,ZFHX3,CUX1,UNC119,RRAGD,ULK3,SPDYE2,GKAP1,GSDMD,ADGRV1,RASGRF2,EPB41L5,WAC,WHRN,DAPK1,SAR1B,APP,GATA4,SMAD5,IGF2BP1,GSKIP,PELI2,ELOVL5,PIK3R5,TLR1,BMT2,AVPR1B,PTK2,TICAM2,SCP2,CELF1,POGZ,PIP4K2B,LRRK2,INO80D,ZNF395,IKZF3,CLSTN2,ARIH1,PLD1,VTCN1,PKHD1,TENT5C,RARB,PJA2,TCF4,PAEP,COP1,TIAM1,CSRP3,MLIP,IQSEC2,ACVR2A,TIPIN,VIT,NEDD4L,BRCC3,KIT,TBC1D8,EPHA7,SPAG9,WNT5A,TRABD2B,ARFGEF2,CTNNB1,ANKRD17,CTSK,PRKDC,IPO5,ESR1,TNFRSF19,ERC2,POFUT2,BCAR3,TPD52L1,PPM1E,ZXDC,FRMD4A,LMX1A,PTGER4,VWC2L,DOCK1,EFNA5,IGF2BP2,CDC14B,HECTD1,TRPC5,NEUROD2,FNIP2,JAML,IKBKB,FXR2,AKAP6,USF3,LYVE1,RAB3GAP2,CCPG1,LINGO2,CEMIP,ATF2,ACVR1,HLA-DQA2,PID1,EBF2,GRID2,ZNF423,RB1,IFNLR1,PDE4B,ADIPOR1,ZNF143,PLPP3,HNF1B,ZNF615,NCOA2,BNIP3L,XRCC5,TBRG4,SHLD2,GFRA1,SLC24A3,TAF15,POU2F1,ETV1,MID2,LRTM2,SIK1,ZNF850,TP53BP2,KAT2B,GOLPH3L,WLS,SUPV3L1,MEF2D,GTF2A1,SPTLC1,CDK5RAP2,RFC3,ARHGEF7,ATF3,TRDN,NPHP1,SRI,ATP1B1,ARNT,BDNF,TFCP2L1,SEMA3E,BCLAF3,SCIN,TLR6,ADAMTS9,STIM1,RAB11FIP2,LAG3,PPARG,CHRNB4,NEK7,TENM1,RORB,SH3KBP1,NALCN,AVIL,SFRP4,SPDYE16,IL1RAP,JCAD,VAV2,TFRC,SPDYE6,RAB31,ZNF292,CLEC6A,BMPER,CLASP2,ZNF521,ANGPT1,SEMA6A,EIF4G1,ITGA4,ROCK1,NCAPH,NUP98,PML,PLEK2,TNFSF4,AFAP1L2,SP100,ELAVL4,NIN,ANKFY1,AIMP1,SLC30A10,PRKD1,HSPBP1,CALCRL,USP34,STK4,PRLR,NMI,IL16,LPAR6,CTBP2,TTN,DYRK1A,TMEM30A,MYRIP,KCNE2,CUL4A,EYA4,SEMA5A,ELL2,PVT1,GATAD2B,METTL3,UAP1,PARD3,KCNN4,ZNF33A,BMAL2,RNF6,SEMA3C,VAV1,SMYD1
GO:0048812	neuron projection morphogenesis	0.0001792603326197466	APBB2,MAP3K13,CHRNB2,CAMK2B,GRIP1,NOTCH2,EPB41L3,MAP6,CDH11,SHANK3,PLXNA4,PRKN,TIAM2,LRP8,CNTN1,CHRNA7,HECW1,TNIK,LAMA2,TRIO,MUSK,EPHA5,SKOR2,ENAH,CHL1,SLIT3,PAK3,NCKAP1,EPHA3,ADGRB3,KLF7,DIP2B,EIF2AK4,MATN2,CUX1,APP,IGF2BP1,PALLD,PTK2,LRRK2,TIAM1,PHACTR1,NEDD4L,KIT,EPHA7,SPAG9,WNT5A,CTNNB1,LMX1A,EFNA5,NGEF,TRPC5,ARHGEF28,KIRREL3,ETV1,LRTM2,BDNF,SEMA3E,CLASP2,SEMA6A,ITGA4,ROCK1,ELAVL4,NIN,SEMA5A,P

			ARD3,RNF6,SEMA3C,SPART
GO:0006468	protein phosphorylation	0.0001886968998702434	*STYK1,RGN,CDK14,MAP3K3,SMYD3,ABR,MAP3K13,SAMSN1,MAPK10,CAMK2B,MAP4K3,FPGT-TNNI3K,STK39,CDK18,DIPK2A,PRKN,CDON,NLK,MAPKAPK3,LRP8,ADAR,CDC37,FGF10,STK32B,HERC5,NCK1,MTCP1,ALPK2,CHRNA7,TNIK,TGFB2,TRIO,MUSK,PRKAR2A,PRKG2,RSKR,EPHA5,STRADA,MAPRE3,TLR8,CACUL1,PAK3,CAMK1G,PRKAG2,CCNT2,CILK1,EPHA3,LATS2,SPDYE2B,ERP29,UVRAG,LYN,RNASEL,ERCC6,EIF2AK4,TGFBR2,MARK4,UNC119,ULK3,SPDYE2,DAPK1,APP,GSKIP,PELI2,PIK3R5,RIPK4,PTK2,LRRK2,ACVR2A,RABGEF1,KIT,EPHA7,SPAG9,WNT5A,PRKDC,IPO5,BCAR3,TPD52L1,PPM1E,HUNK,PKIG,EFNA5,TRPC5,FNIP2,IKBKB,FXR2,CEMIP,ATF2,ACVR1,PID1,RB1,PLPP3,XRCC5,GFRA1,BMX,NRK,SIK1,BDNF,TLR6,PPARG,NEK7,TENM1,MYLK4,SPDYE16,TFRC,SPDYE6,MAP3K1,CLEC6A,ANGPT1,EIF4G1,ROCK1,PRKD1,STK4,TNNI3K,PRLR,TTN,DYRK1A,PARD3*
GO:0022008	neurogenesis	0.00020568978375778703	*APBB2,RTN1,TRAPPC9,MAP3K13,THRB,CHRNB2,SEZ6,AGTPBP1,SPINK5,CAMK2B,GRIP1,DCT,NOTCH2,SDK2,EPB41L3,MAP6,CDH11,SHANK3,STYXL1,PLXNA4,PRKN,NLGN4X,TAFA1,IFT172,CDON,SERPINI1,NCAM2,TIAM2,TRAK2,LRP8,CNTN1,EIF4ENIF1,FGF10,NCK1,NREP,GABRA5,NEXMIF,ASTN1,PREX1,CHRNA7,HECW1,HMGB1,TNIK,TGFB2,LAMA2,TRIO,MUSK,ALKBH1,EPHA5,SKOR2,ENAH,ALDH1A2,RRAS2,CHL1,PLS1,SLIT3,PAK3,BTG4,NCKAP1,EPHA3,LYN,ADGRB3,KLF7,DIP2B,NAV1,VSX1,ERCC6,EIF2AK4,MATN2,CRTAC1,ZFHX3,CUX1,GPM6B,ADGRV1,WHRN,TDP2,PRUNE1,APP,IGF2BP1,HCN1,FRY,HOOK3,PALLD,CECR2,PTK2,LRRK2,RARB,TCF4,TIAM1,PHACTR1,NTM,NEDD4L,KIT,EPHA7,SPAG9,WNT5A,CTNNB1,LMX1A,VWC2L,EFNA5,NGEF,TRPC5,NEUROD2,PTPRF,ARHGEF28,GPRIN1,OPCML,GRID2,RB1,PLPP3,KIRREL3,XRCC5,GFRA1,ETV1,LRTM2,KAT2B,ANKS1A,CDK5RAP2,BDNF,SEMA3E,PPARG,TENM1,RORB,GABRB1,AVIL,CLASP2,ZNF521,SEMA6A,EIF4G1,ITGA4,ROCK1,DGKG,ELAVL4,NIN,PRKD1,HHIP,TMEM30A,SEMA5A,METTL3,PARD3,ATP8B1,RNF6,SEMA3C,VPS13B,SPART*
GO:0120039	plasma membrane bounded cell projection morphogenesis	0.00021104521590210117	*APBB2,MAP3K13,CHRNB2,CAMK2B,GRIP1,NOTCH2,EPB41L3,MAP6,CDH11,SHANK3,PLXNA4,PRKN,TIAM2,LRP8,CNTN1,CHRNA7,HECW1,TNIK,LAMA2,TRIO,MUSK,EPHA5,SKOR2,ENAH,CHL1,SLIT3,PAK3,NCKAP1,EPHA3,ADGRB3,KLF7,DIP2B,EIF2AK4,MATN2,CUX1,APP,IGF2BP1,PALLD,PTK2,LRRK2,TIAM1,PHACTR1,NEDD4L,KIT,EPHA7,SPAG9,WNT5A,CTNNB1,LMX1A,EFNA5,NGEF,TRPC5,ARHGEF28,KIRREL3,ETV1,LRTM2,ARHGEF7,BDNF,SEMA3E,CLASP2,SEMA6A,ITGA4,ROCK1,ELAVL4,NIN,SEMA5A,PARD3,RNF6,SEMA3C,SPART*
GO:0032501	multicellular organismal process	0.00024022828931002891	*FSTL1,APBB2,RTN1,HS3ST3A1,RGN,MAP3K3,LRRN1,ARHGEF9,TAFA2,ISM1,TRAPPC9,MAP3K13,ARL13B,SAMSN1,LIMD1,TF,CHST11,THRB,RCSD1,SPTBN1,CACNB1,CHRNB2,SEZ6,GNB4,AGTPBP1,SH3PXD2A,SPINK5,OPA3,SKAP2,BRD4,DOK4,CAMK2B,GRIP1,DCT,ABCG8,NOTCH2,CD93,TMOD2,SDK2,NPNT,EPB41L3,MAP6,MORC1,CDH11,SHANK3,NR4A3,ARID1B,E2F7,FOXO1,STYXL1,STK39,SMURF2,ADAMTS12,PLXNA4,YBX1,DIPK2A,CACNA1C,ASB4,IDO1,PRKN,NLGN4X,F13A1,TAFA1,KDM7A,SLC5A7,IFT172,GPSM1,PTBP3,CDON,SERPINI1,SL*

			C17A6,THSD7A,NCAM2,TIAM2,CFAP54,TRAK2,CNMD,LRP8,TAGLN3,CNTN1,MEOX2,STAT5B,ENPEP,EIF4ENIF1,CHRFAM7A,ERBIN,MBTPS2,CD247,MCC,PTH,ADAR,CLCN3,RHOJ,FGF10,TSPAN18,PLG,ILDR2,NCK1,CD84,NREP,EXO1,GABRA5,NEXMIF,ASTN1,KDM2B,PREX1,CDIN1,COL11A1,CIDEB,MCPH1,COL1A2,DTNA,TMOD3,MRTFB,COL12A1,ABLIM1,ALPK2,CHRNA7,HECW1,HMGB1,ZNF304,AFF3,RFLNA,ATRX,TNIK,TGFB2,LAMA2,ADD3,TRIO,HOXC13,SMARCC1,NDST1,MUSK,JAM3,ALKBH1,OR5AU1,ATP11A,SLC15A4,PCDHB8,PRKG2,SND1,IGFBP7,EPHA5,SKOR2,ENAH,SNX10,ALDH1A2,PTGFR,ASPH,RRAS2,SIM1,KIAA1217,CHL1,GSN,PLS1,TLR8,SLIT3,NLRP7,CD226,ANKRD11,ITPR1,DNAAF11,PAK3,ABI3BP,MLLT3,BTG4,HAVCR1,MR1,F8,TNFRSF12A,CPT2,TAS2R14,CXCR5,NCKAP1,CLDN12,FOSL2,IL1RAPL2,EPHA3,LATS2,MYH14,MYOM2,LYN,ADGRB3,KLF7,GRIK1,DIP2B,SEC24C,FIGNL1,CA10,RRM2,CRYBB1,LDB2,ALDH1A1,NAV1,CORIN,PCDHB16,SGMS2,NOS3,VSX1,ERCC6,EIF2AK4,IMPG1,MATN2,TGFBR2,ITGA11,MARK4,SYNJ1,TCF7L2,CRTAC1,ZFHX3,CUX1,UNC119,GPM6B,ULK3,CACNA2D4,LHFPL3,GSDMD,ADGRV1,EPB41L5,GAD1,WHRN,TDP2,PRUNE1,INSYN2A,APP,GATA4,SMAD5,IGF2BP1,MAMLD1,RNLS,RNF38,ADAM12,DHX35,HCN1,CHRM5,FRY,HOOK3,PALLD,TLR1,CECR2,TAS2R30,AVPR1B,PTK2,TICAM2,SH2B2,CELF1,HYCC1,LRRK2,INO80D,IKZF3,CLSTN2,VTCN1,ITFG2,PKHD1,TENT5C,RARB,PJA2,TCF4,PAEP,TIAM1,CSRP3,PHACTR1,MLIP,ACVR2A,GNAO1,VIT,SNTG2,NTM,NEDD4L,RABGEF1,KIT,TBC1D8,EPHA7,SPAG9,ANTXR1,WNT5A,ARFGEF2,CTNNB1,ANKRD17,COL19A1,CTSK,PRKDC,ESR1,TNFRSF19,PTPN14,POFUT2,BCAR3,LMX1A,PTGER4,GREB1L,BBX,VWC2L,DOCK1,GPC4,BPGM,EFNA5,IGF2BP2,OR1M1,HECTD1,NGEF,TRPC5,NEUROD2,JAML,KAT14,PTPRF,IKBKB,FXR2,AKAP6,USF3,APOH,ARHGEF28,CLCN1,TASOR,GPRIN1,OPCML,LINGO2,CEMIP,ATF2,KCNJ12,ACVR1,CHRNB3,HLA-DQA2,EBF2,GRID2,ZNF423,KCNJ3,PLPPR1,RB1,MPV17,PDE4B,PKD1L3,ADIPOR1,GABRB3,SHROOM4,PLPP3,HNF1B,IMMP2L,SSPN,KIRREL3,GABRG2,F5,CPS1,NCOA2,TMEM120A,ACACB,XRCC5,SHLD2,GFRA1,KCND2,SLC24A3,RAB18,ETV1,TEX11,LRTM2,SIK1,KAT2B,WLS,ANKS1A,RYR3,PRCP,MEF2D,MYL4,CDK5RAP2,FLVCR1,ARHGEF7,DACH1,TRDN,NPHP1,IKZF1,SRI,ATP1B1,ARNT,BDNF,LDLRAD4,TFCP2L1,SEMA3E,SCIN,MACO1,TLR6,ADAMTS9,STIM1,TMEM161B,LAG3,PPARG,TANC1,PAPPA,ASB3,MTTP,CHRNB4,TENM1,IL13RA2,RORB,GABRB1,MYOF,LTB4R2,JAKMIP1,SH3KBP1,AVIL,AMY2A,TTC39C,SIPA1L3,SLC6A13,WDFY4,SFRP4,BLTP1,IL1RAP,JCAD,VAV2,TFRC,VPS13A,FBN1,IQCB1,CLEC6A,BMPER,FUT10,CLASP2,ZNF521,ANGPT1,SEMA6A,EIF4G1,ATP6V0A4,PHLPP1,ITGA4,ROCK1,CLEC3A,PML,MGLL,TNFSF4,AFAP1L2,DGKG,SP100,ELAVL4,SBF2,CDH9,UMODL1,NIN,FNDC3A,AIMP1,PRKD1,CALCRL,SLC20A2,STK4,PCDH10,TNNI3K,ANPEP,PRLR,NMI,IL16,CR2,LPAR6,TTN,NCALD,HHIP,DYRK1A,TMEM30A,KCNE2,CUL4A,EYA4,SEMA5A,LAMA4,TTPA,METTL3,KRT85,UAP1,PARD3,KCNN4,ATP8B1,RNF6,SEMA3C,VAV1,SMYD1,VPS13B,SPART
GO:0048	cell	0.000281343	*APBB2,MAP3K13,CHRNB2,CAMK2B,GRIP1,NOTCH2*

858	projection morphogenesis	6417238336	,EPB41L3,MAP6,CDH11,SHANK3,PLXNA4,PRKN,TIAM2,LRP8,CNTN1,CHRNA7,HECW1,TNIK,LAMA2,TRIO,MUSK,EPHA5,SKOR2,ENAH,CHL1,SLIT3,PAK3,NCKAP1,EPHA3,ADGRB3,KLF7,DIP2B,EIF2AK4,MATN2,CUX1,APP,IGF2BP1,PALLD,PTK2,LRRK2,TIAM1,PHACTR1,NEDD4L,KIT,EPHA7,SPAG9,WNT5A,CTNNB1,LMX1A,EFNA5,NGEF,TRPC5,ARHGEF28,KIRREL3,ETV1,LRTM2,ARHGEF7,BDNF,SEMA3E,CLASP2,SEMA6A,ITGA4,ROCK1,ELAVL4,NIN,SEMA5A,PARD3,RNF6,SEMA3C,SPART
GO:0048667	cell morphogenesis involved in neuron differentiation	0.0005837625685134452	APBB2,MAP3K13,CHRNB2,CAMK2B,NOTCH2,MAP6,CDH11,SHANK3,PLXNA4,TIAM2,LRP8,CNTN1,CHRNA7,HECW1,TNIK,LAMA2,TRIO,MUSK,EPHA5,SKOR2,ENAH,CHL1,PLS1,SLIT3,PAK3,EPHA3,ADGRB3,KLF7,DIP2B,MATN2,CUX1,WHRN,APP,IGF2BP1,PALLD,PTK2,LRRK2,TIAM1,PHACTR1,NEDD4L,KIT,EPHA7,WNT5A,LMX1A,EFNA5,NGEF,TRPC5,ARHGEF28,RB1,ETV1,LRTM2,BDNF,SEMA3E,CLASP2,SEMA6A,ITGA4,ELAVL4,NIN,SEMA5A,PARD3,RNF6,SEMA3C,SPART
GO:0048013	ephrin receptor signaling pathway	0.0007429481860978024	NCK1,EPHA5,ANKS1B,PAK3,EPHA3,LYN,PTK2,TIAM1,EPHA7,EFNA5,NGEF,ARHGEF28,ANKS1A,ARHGEF7
GO:0051641	cellular localization	0.0007442961561518678	MREG,OSBPL8,STX8,SMYD3,RAPGEF6,BLTP3B,TRAPPC9,PPM1A,ARL13B,RN7SL556P,IMMP1L,RCSD1,SPTBN1,MYO5C,AGTPBP1,ATP7B,GRIP1,RGPD2,XPO4,NPNT,EPB41L3,MAP6,SHANK3,NR4A3,FOXO1,YBX1,CACNA1C,PRKN,NLGN4X,XPO6,IFT172,GPSM1,SLC17A6,GNAI1,INTS6-AS1,CFAP54,TRAK2,UBE2O,EIF4ENIF1,ERBIN,CD247,CEP192,MCC,ADAR,TM9SF2,CLCN3,CDC37,FGF10,ZFYVE16,TSPAN18,ILDR2,CD84,RGPD3,ANO3,CIDEB,MCPH1,MYO1D,XPO7,DYNLT2B,ATRX,TNIK,TGFB2,MDFIC,MUSK,JAM3,KCNIP3,NUP93,RBM33,SLC15A4,GOLPH3,PRKG2,EPHA5,SNX10,ATP2C1,TMEM230,STON2,STRADA,HUWE1,ASPH,ZDHHC4,UBR5,GSN,PLS1,MAPRE3,HPS3,ITPR1,DNAAF11,TRAPPC10,CPT2,VPS8,MTX3,CILK1,PACRG,EPHA3,LATS2,ERP29,UVRAG,LYN,KLF7,SEC24C,ATP6V1H,NUP58,SIL1,TMCC1,TMED7-TICAM2,NOS3,NOMO2,RN7SL738P,KIAA1614,MAGEL2,MARK4,MDN1,SYNJ1,TCF7L2,UNC119,GPM6B,RRAGD,RGPD1,KPNB1,GSDMD,ADGRV1,EPB41L5,SCG5,WHRN,SAR1B,TTC7B,APP,HCN1,RHOT1,USE1,HOOK3,LMAN1L,NOP9,USP7,SCP2,HYCC1,LRRK2,GET1-SH3BGR,PLD1,PKHD1,TIAM1,CSRP3,IQSEC2,NEDD4L,ENSG00000288683,SNX13,RABGEF1,KIT,CYB5R4,SPAG9,SPDL1,WNT5A,PEX26,ARFGEF2,CTNNB1,IPO5,ESR1,PTPN14,SCFD1,ERC2,VPS53,FRMD4A,GPC4,PKIG,EFNA5,SYNE3,PLCH1,IKBKB,AKAP6,RAB3GAP2,TASOR,CEMIP,XIST,ATF2,PID1,CCDC91,GRID2,ZNF423,RB1,KIF13A,GOSR2,SMG7,HNF1B,IMMP2L,LAMP2,BNIP3L,RABGAP1L,XRCC5,RAB18,BACH2,MGRN1,MID2,KAT2B,GOLPH3L,WLS,RYR3,MYO1B,CDK5RAP2,TRDN,NPHP1,SRI,ATP1B1,BDNF,SCIN,ADAMTS9,RAB11FIP2,PPARG,ASB3,MTTP,CHRNB4,TENM1,IL13RA2,MYOF,JAKMIP1,GDAP1,KIF2C,MYO5A,SLC6A13,SPTBN5,BLTP1,TFRC,VPS13A,FBN1,RAB31,BMPER,FUT10,CLASP2,ANGPT1,EIF4G1,ATP6V0A4,ITGA4,DNAJC6,ROCK1,TAX1BP1,NUP98,PML,SCFD2,SP100,ND

			UFAF2,NIN,ANKFY1,ENSA,AP4S1,PRKD1,CALCRL,STK4,RAB6D,SPC25,SCAMP1,RAB6C,CTBP2,TTN,PPFIA1,TMEM30A,MYRIP,UNC13C,PARD3,KCNN4,ATP8B1,VPS13B
GO:0120036	plasma membrane bounded cell projection organization	0.0007700297779015243	*APBB2,RAPGEF6,MAP3K13,ARL13B,CDC42EP3,CHRNB2,CDC14C,SEZ6,CAMK2B,GRIP1,NOTCH2,EPB41L3,MAP6,PARVB,CDH11,SHANK3,STYXL1,PLXNA4,CFAP70,PRKN,IFT172,SERPINI1,NCAM2,TIAM2,CFAP54,LRP8,CNTN1,ARHGEF4,NCK1,NREP,PREX1,ABLIM1,CHRNA7,HECW1,HMGB1,DYNLT2B,TNIK,LAMA2,TRIO,MUSK,ALKBH1,GOLPH3,EPHA5,SKOR2,ENAH,SNX10,CHL1,GSN,PLS1,SLIT3,DNAAF11,PAK3,NCKAP1,CILK1,EPHA3,LYN,ADGRB3,KLF7,DIP2B,ERCC6,EIF2AK4,MATN2,MARK4,CRTAC1,CUX1,GPM6B,ADGRV1,WHRN,APP,IGF2BP1,FRY,PALLD,CECR2,PTK2,LRRK2,CFAP298,PLD1,PKHD1,TIAM1,PHACTR1,NEDD4L,KIT,EPHA7,SPAG9,WNT5A,CTNNB1,LMX1A,EFNA5,CDC14B,NGEF,TRPC5,NEUROD2,PTPRF,FXR2,ARHGEF28,GPRIN1,GRID2,ZNF423,RB1,KIRREL3,GFRA1,ETV1,LRTM2,KAT2B,ARHGEF7,BDNF,SEMA3E,TMEM237,TANC1,TENM1,AVIL,TTC39C,VAV2,IQCB1,CLASP2,SEMA6A,ITGA4,ROCK1,PLEK2,DGKG,ELAVL4,NIN,PRKD1,TMEM30A,SEMA5A,PARD3,ATP8B1,RNF6,SEMA3C,VPS13B,SPART*
GO:0007167	enzyme-linked receptor protein signaling pathway	0.0008773172260983451	*FSTL1,OSBPL8,PPM1A,CHST11,SPTBN1,RBPMS,DOK4,NOTCH2,NPNT,NR4A3,FOXO1,SMURF2,ADAMTS12,FAM83B,NLK,MAPKAPK3,STAT5B,UBE2O,ERBIN,FGF10,COL4A6,NCK1,NREP,COL1A2,HIVEP1,TGFB2,TRIO,SMARCC1,NDST1,MUSK,NUP93,EPHA5,SKOR2,ANKS1B,PAK3,EPHA3,LATS2,LYN,TGFBR2,SHISA2,GKAP1,GATA4,SMAD5,PTK2,SH2B2,PIP4K2B,TIAM1,CSRP3,ACVR2A,RABGEF1,KIT,EPHA7,WNT5A,CTNNB1,BCAR3,VWC2L,EFNA5,NGEF,PTPRF,ARHGEF28,ATF2,ACVR1,PID1,ZNF423,ADIPOR1,GFRA1,ANKS1A,ARHGEF7,SAMD12,ARNT,BDNF,LDLRAD4,PPARG,SFRP4,JCAD,VAV2,FBN1,BMPER,ANGPT1,SEMA6A,PML,AFAP1L2,SLC30A10,PRKD1,PRLR,HHIP,PMEPA1,VAV1,SPART*
GO:0048583	regulation of response to stimulus	0.0010932184630335405	*FSTL1,RGN,CDK14,ING3,MAP3K3,OSBPL8,ARHGEF9,ABR,MAOA,MAP3K13,PPM1A,SAMSN1,LIMD1,CHST11,THRB,SPTBN1,MAPK10,USP32,SEZ6,SPINK5,BRD4,DOK4,CAMK2B,SLC38A9,NOTCH2,TMOD2,NPNT,SHANK3,NR4A3,ARID1B,FOXO1,STYXL1,STK39,SMURF2,ADAMTS12,PLXNA4,URB2,DIPK2A,IDO1,PRKN,NLGN4X,TAFA1,FAM83B,IFT172,CDON,NLK,GNAI1,MAPKAPK3,TIAM2,LRP8,STAT5B,ARHGEF4,UBE2O,DDIAS,ERBIN,MBTPS2,RGS22,CD247,MCC,PTH,ADAR,PDE10A,CDC37,FGF10,MECOM,NLRP8,PLG,HERC5,NCK1,FAM168A,NONO,NREP,PREX1,NCOA7,MCPH1,ALPK2,CHRNA7,HECW1,HMGB1,TNIK,TGFB2,MDFIC,LAMA2,TRIO,SMARCC1,NDST1,MUSK,JAM3,SFPQ,RGS3,NUP93,SLC15A4,GOLPH3,USP10,IGFBP7,SKOR2,ATP2C1,UBR5,SOX30,TLR8,SLIT3,NLRP7,CD226,ITPR1,PAK3,MLLT3,MR1,TNFRSF12A,OASL,FOSL2,LATS2,ERP29,ARHGAP18,LYN,KLF7,FIGNL1,TMED7-TICAM2,NOS3,ERCC6,EIF2AK4,TGFBR2,MARK4,TCF7L2,SHISA2,RRAGD,ULK3,GKAP1,GSDMD,ADGRV1,RASGRF2,WAC,DAPK1,APP,GATA4,SMAD5,GSKIP,PELI2,PIK3R5,TLR1,BMT2,AVPR1B,PTK2,USP7,TICAM2,SH2B2,CELF1,PIP4K2B,LRRK2,INO80D,VTCN1,ITFG2,PKHD1,PJA2,TIAM1,MLIP,IQSEC2,ACVR2A,BRCC3,SNX13,RABGEF1,KIT,EPHA7*

			,*SPAG9,WNT5A,TRABD2B,ARFGEF2,CTNNB1,ANKRD17,CTSK,PRKDC,IPO5,ESR1,TNFRSF19,BCAR3,TPD52L1,PTGER4,VWC2L,GPC4,NGEF,NEUROD2,FNIP2,PTPRF,IKBKB,AKAP6,APOH,ARHGEF28,ACVR1,HLA-DQA2,PID1,GRID2,ZNF423,RB1,IFNLR1,PDE4B,NLRP4,ADIPOR1,PLPP3,HNF1B,LAMP2,NCOA2,ACACB,XRCC5,RGS17,SHLD2,BMX,RGS7BP,MGRN1,MID2,KAT2B,WLS,PRCP,CDK5RAP2,ATF3,ARNT,BDNF,LDLRAD4,SEMA3E,TLR6,TMEM237,RAB11FIP2,LAG3,PPARG,NEK7,TENM1,USP25,SIPA1L3,SFRP4,JCAD,VAV2,TFRC,PNPLA8,FBN1,HAPSTR1,MAP3K1,CLEC6A,BMPER,CLASP2,ANGPT1,SEMA6A,EIF4G1,PHLPP1,ROCK1,TAX1BP1,APIP,MMP28,PML,MGLL,TNFSF4,AFAP1L2,DGKG,SP100,NDUFAF2,SPG21,SLC30A10,PRKD1,USP34,STK4,PRLR,PDE11A,NMI,IL16,CR2,LPAR6,CTBP2,HHIP,DYRK1A,RGS10,CUL4A,KCTD16,EYA4,SEMA5A,PMEPA1,METTL3,KCNN4,RNF6,SEMA3C,VAV1,VDAC2,SPART*
GO:0044087	regulation of cellular component biogenesis	0.0011475882745811405	*C10ORF90,LRRN1,ARHGEF9,SPTBN1,CDC42EP3,CHRNB2,TMOD2,SHANK3,STYXL1,PRKN,NCK1,PREX1,TMOD3,HMGB1,DYNLT2B,ADD3,MUSK,JAM3,USP10,GSN,MAPRE3,PAK3,VPS8,NCKAP1,IL1RAPL2,EPHA3,ARHGAP18,ADGRB3,NME7,LDB2,MARK4,GPM6B,EPB41L5,PRUNE1,SAR1B,APP,PTK2,PIP4K2B,LRRK2,CLSTN2,PLD1,BRCC3,KIT,EPHA7,ANTXR1,WNT5A,TRABD2B,CTNNB1,SCFD1,PPM1E,PTGER4,GPC4,EFNA5,FNIP2,IKBKB,FXR2,RAB3GAP2,LINGO2,GRID2,RB1,LAMP2,XRCC5,LRTM2,KAT2B,CDK5RAP2,ARHGEF7,NPHP1,CAPZA2,BDNF,SCIN,TLR6,HAUS6,PPARG,NEK7,TENM1,AVIL,SPTBN5,IL1RAP,TFRC,CLASP2,EIF4G1,DNAJC6,ROCK1,PLEK2,PRKD1,DYRK1A,PPFIA1,ATP8B1,VDAC2*
GO:0030154	cell differentiation	0.0011562901031853879	*FSTL1,APBB2,RTN1,MREG,OSBPL8,TXNDC8,SMYD3,TRAPPC9,MAP3K13,LIMD1,TF,CHST11,THRB,USP42,CHRNB2,SEZ6,AGTPBP1,FHL1,SH3PXD2A,SPINK5,BRD4,CAMK2B,GRIP1,DCT,NOTCH2,TMOD2,SDK2,NPNT,EPB41L3,MAP6,MORC1,CDH11,SHANK3,NR4A3,ARID1B,E2F7,FOXO1,STYXL1,ADAMTS12,PLXNA4,YBX1,ASB4,ARB2A,PRKN,NLGN4X,PNPLA3,TAFA1,IFT172,GPSM1,PTBP3,CDON,SERPINI1,SDF4,THSD7A,NCAM2,TIAM2,CFAP54,TRAK2,CNMD,LRP8,CNTN1,STAT5B,EIF4ENIF1,HEATR9,PTH,ADAR,FGF10,MECOM,CATSPERB,PLG,ILDR2,NCK1,NREP,GABRA5,NEXMIF,ASTN1,PREX1,CDIN1,COL11A1,TMOD3,MRTFB,COL12A1,ALPK2,CHRNA7,HECW1,HMGB1,RFLNA,ATRX,TNIK,TGFB2,LAMA2,TRIO,SMARCC1,MUSK,JAM3,ALKBH1,ATP11A,PRKG2,SND1,EPHA5,SKOR2,ENAH,SNX10,ALDH1A2,HUWE1,RRAS2,SIM1,SOX30,CHL1,PLS1,SLIT3,PAK3,ABI3BP,AXDND1,MLLT3,CCNT2,BTG4,MR1,TNFRSF12A,NCKAP1,FOSL2,PACRG,EPHA3,LATS2,MYOM2,LYN,ADGRB3,KLF7,MTA3,DIP2B,FIGNL1,RNASEL,NAV1,PAQR5,VSX1,PRAMEF27,ERCC6,EIF2AK4,MATN2,TGFBR2,ITGA11,TCF7L2,CRTAC1,ZFHX3,CUX1,GPM6B,ADGRV1,ST7,EPB41L5,WHRN,OCA2,TDP2,PRUNE1,APP,GATA4,SMAD5,IGF2BP1,ADAM12,HCN1,FRY,HOOK3,PALLD,CECR2,PTK2,SH2B2,CELF1,PRRC2B,LRRK2,IKZF3,ITFG2,PKHD1,TENT5C,RARB,TCF4,PAEP,TIAM1,CSRP3,PHACTR1,ACVR2A,NTM,NEDD4L,KIT,CYB5R4,EPHA7,SPAG9,WNT5A,CTNNB1,ANKRD17,COL19A1,RUNX1T1,CTSK,PRKDC,ESR1,POFUT2,LMX1A,PTGE*

			R4,VWC2L,DOCK1,BPGM,EFNA5,NGEF,TRPC5,NEUROD2,PTPRF,IKBKB,AKAP6,USF3,ARHGEF28,TASOR,GPRIN1,OPCML,ATF2,ACVR1,EBF2,GRID2,ZNF423,RB1,ADIPOR1,PLPP3,HNF1B,KIRREL3,CPS1,TMEM120A,AGPAT5,XRCC5,GFRA1,ETV1,TEX11,LRTM2,SIK1,KAT2B,ANKS1A,MEF2D,CDK5RAP2,FLVCR1,ARHGEF7,ATF3,NPHP1,IKZF1,SLC26A8,ARNT,BDNF,LDLRAD4,TFCP2L1,SEMA3E,SCIN,ADAMTS9,LAG3,PPARG,TANC1,TENM1,RORB,GABRB1,AVIL,SIPA1L3,SFRP4,BLTP1,TFRC,FBN1,DDX4,IQCB1,FUT10,CLASP2,ZNF521,ANGPT1,SEMA6A,EIF4G1,ITGA4,ROCK1,M1AP,PML,TNFSF4,DGKG,SPATA25,ELAVL4,NIN,FNDC3A,PRKD1,STK4,ANPEP,PRLR,EDARADD,CR2,CTBP2,TTN,HHIP,TMEM30A,CUL4A,TXNRD3,EYA4,SEMA5A,LAMA4,TTPA,GATAD2B,METTL3,KRT85,PARD3,ATP8B1,RNF6,SEMA3C,VAV1,SMYD1,VPS13B,SPART
GO:0048869	cellular developmental process	0.0011812291883329187	*FSTL1,APBB2,RTN1,MREG,OSBPL8,TXNDC8,SMYD3,TRAPPC9,MAP3K13,LIMD1,TF,CHST11,THRB,USP42,CHRNB2,SEZ6,AGTPBP1,FHL1,SH3PXD2A,SPINK5,BRD4,CAMK2B,GRIP1,DCT,NOTCH2,TMOD2,SDK2,NPNT,EPB41L3,MAP6,MORC1,CDH11,SHANK3,NR4A3,ARID1B,E2F7,FOXO1,STYXL1,ADAMTS12,PLXNA4,YBX1,ASB4,ARB2A,PRKN,NLGN4X,PNPLA3,TAFA1,IFT172,GPSM1,PTBP3,CDON,SERPINI1,SDF4,THSD7A,NCAM2,TIAM2,CFAP54,TRAK2,CNMD,LRP8,CNTN1,STAT5B,EIF4ENIF1,HEATR9,PTH,ADAR,FGF10,MECOM,CATSPERB,PLG,ILDR2,NCK1,NREP,GABRA5,NEXMIF,ASTN1,PREX1,CDIN1,COL11A1,TMOD3,MRTFB,COL12A1,ALPK2,CHRNA7,HECW1,HMGB1,RFLNA,ATRX,TNIK,TGFB2,LAMA2,TRIO,SMARCC1,MUSK,JAM3,ALKBH1,ATP11A,PRKG2,SND1,EPHA5,SKOR2,ENAH,SNX10,ALDH1A2,HUWE1,RRAS2,SIM1,SOX30,CHL1,PLS1,SLIT3,PAK3,ABI3BP,AXDND1,MLLT3,CCNT2,BTG4,MR1,TNFRSF12A,NCKAP1,FOSL2,PACRG,EPHA3,LATS2,MYOM2,LYN,ADGRB3,KLF7,MTA3,DIP2B,FIGNL1,RNASEL,NAV1,PAQR5,VSX1,PRAMEF27,ERCC6,EIF2AK4,MATN2,TGFBR2,ITGA11,TCF7L2,CRTAC1,ZFHX3,CUX1,GPM6B,ADGRV1,ST7,EPB41L5,WHRN,OCA2,TDP2,PRUNE1,APP,GATA4,SMAD5,IGF2BP1,ADAM12,HCN1,FRY,HOOK3,PALLD,CECR2,PTK2,SH2B2,CELF1,PRRC2B,LRRK2,IKZF3,ITFG2,PKHD1,TENT5C,RARB,TCF4,PAEP,TIAM1,CSRP3,PHACTR1,ACVR2A,NTM,NEDD4L,KIT,CYB5R4,EPHA7,SPAG9,WNT5A,CTNNB1,ANKRD17,COL19A1,RUNX1T1,CTSK,PRKDC,ESR1,POFUT2,LMX1A,PTGER4,VWC2L,DOCK1,BPGM,EFNA5,NGEF,TRPC5,NEUROD2,PTPRF,IKBKB,AKAP6,USF3,ARHGEF28,TASOR,GPRIN1,OPCML,ATF2,ACVR1,EBF2,GRID2,ZNF423,RB1,ADIPOR1,PLPP3,HNF1B,KIRREL3,CPS1,TMEM120A,AGPAT5,XRCC5,GFRA1,ETV1,TEX11,LRTM2,SIK1,KAT2B,ANKS1A,MEF2D,CDK5RAP2,FLVCR1,ARHGEF7,ATF3,NPHP1,IKZF1,SLC26A8,ARNT,BDNF,LDLRAD4,TFCP2L1,SEMA3E,SCIN,ADAMTS9,LAG3,PPARG,TANC1,TENM1,RORB,GABRB1,AVIL,SIPA1L3,SFRP4,BLTP1,TFRC,FBN1,DDX4,IQCB1,FUT10,CLASP2,ZNF521,ANGPT1,SEMA6A,EIF4G1,ITGA4,ROCK1,M1AP,PML,TNFSF4,DGKG,SPATA25,ELAVL4,NIN,FNDC3A,PRKD1,STK4,ANPEP,PRLR,EDARADD,CR2,CTBP2,TTN,HHIP,TMEM30A,CUL4A,TXNRD3,EYA4,SEMA5A,LAMA4,TTPA,GATAD2B,METTL3,KRT85,PARD3,ATP8B1,RNF6,SEMA3C,VAV1,SMYD1,VPS13B,SPART*

GO:0030030	cell projection organization	0.0011932718426614166	*APBB2,RAPGEF6,MAP3K13,ARL13B,CDC42EP3,CHRNB2,CDC14C,SEZ6,CAMK2B,GRIP1,NOTCH2,EPB41L3,MAP6,PARVB,CDH11,SHANK3,STYXL1,PLXNA4,CFAP70,PRKN,IFT172,SERPINI1,NCAM2,TIAM2,CFAP54,LRP8,CNTN1,ARHGEF4,NCK1,NREP,PREX1,ABLIM1,CHRNA7,HECW1,HMGB1,DYNLT2B,TNIK,LAMA2,TRIO,MUSK,ALKBH1,GOLPH3,EPHA5,SKOR2,ENAH,SNX10,CHL1,GSN,PLS1,SLIT3,DNAAF11,PAK3,NCKAP1,CILK1,EPHA3,LYN,ADGRB3,KLF7,DIP2B,ERCC6,EIF2AK4,MATN2,MARK4,CRTAC1,CUX1,GPM6B,ADGRV1,WHRN,APP,IGF2BP1,FRY,PALLD,CECR2,PTK2,LRRK2,CFAP298,PLD1,PKHD1,TIAM1,PHACTR1,NEDD4L,KIT,EPHA7,SPAG9,WNT5A,CTNNB1,LMX1A,EFNA5,CDC14B,NGEF,TRPC5,NEUROD2,PTPRF,FXR2,ARHGEF28,GPRIN1,GRID2,ZNF423,RB1,KIRREL3,GFRA1,ETV1,LRTM2,KAT2B,ARHGEF7,NPHP1,BDNF,SEMA3E,SCIN,TMEM237,TANC1,TENM1,AVIL,TTC39C,VAV2,IQCB1,CLASP2,SEMA6A,ITGA4,ROCK1,PLEK2,DGKG,ELAVL4,NIN,PRKD1,TMEM30A,SEMA5A,PARD3,ATP8B1,RNF6,SEMA3C,VPS13B,SPART*
GO:0007155	cell adhesion	0.001276534484374044	*ENSG00000290149,MYBPC1,CLDN6,CDH8,SPINK5,BRD4,ZAN,CD93,SDK2,NPNT,DSC1,PARVB,CDH11,NR4A3,ARID1B,ADAMTS12,PLXNA4,IDO1,NLGN4X,CBLL2,CDON,NCAM2,CNTN1,STAT5B,ERBIN,COL4A6,PLG,CDH7,ILDR2,NCK1,CD84,NEXMIF,ASTN1,PREX1,TMOD3,COL12A1,HMGB1,LPP,TGFB2,LAMA2,SMARCC1,MUSK,JAM3,PCDHB8,GOLPH3,IGFBP7,ATP2C1,CHL1,SPON1,CD226,PKP4,ABI3BP,TNFRSF12A,CLDN12,CNTN3,EPHA3,LYN,PCDHB16,TGFBR2,ITGA11,ZFHX3,GPM6B,ADGRV1,EPB41L5,APP,ADAM12,PALLD,PTK2,CLSTN2,VTCN1,PKHD1,TIAM1,VIT,NTM,KIT,EPHA7,ANTXR1,WNT5A,CTNNB1,COL19A1,DOCK1,GPC4,EFNA5,JAML,PTPRF,LYVE1,OPCML,ACVR1,HLA-DQA2,COL6A5,GRID2,PLPP3,SSPN,KIRREL3,BMX,ARHGEF7,NPHP1,ATP1B1,SEMA3E,ADAMTS9,LAG3,TENM1,IL1RAP,JCAD,TFRC,FBN1,CLASP2,ANGPT1,SEMA6A,ITGA4,ROCK1,PML,TNFSF4,CDH9,PCDH19,FNDC3A,STK4,PCDH10,PRLR,PPFIA1,SEMA5A,LAMA4,METTL3,PARD3,COL6A6,VAV1*
GO:0010646	regulation of cell communication	0.0013842073263396599	*FSTL1,RGN,CDK14,MAP3K3,OSBPL8,ARHGEF9,ABR,MAOA,MAP3K13,PPM1A,LIMD1,CHST11,THRB,SPTBN1,USP32,CHRNB2,SEZ6,BRD4,DOK4,CAMK2B,SLC38A9,NOTCH2,TMOD2,NPNT,CDH11,SHANK3,FOXO1,STYXL1,STK39,SMURF2,ADAMTS12,URB2,DIPK2A,PRKN,NLGN4X,TAFA1,FAM83B,IFT172,CDON,NLK,GNAI1,TIAM2,LRP8,ARHGEF4,UBE2O,DDIAS,ERBIN,RGS22,MCC,PTH,ADAR,PDE10A,CDC37,FGF10,MECOM,PLG,NCK1,NONO,NREP,PREX1,ALPK2,CHRNA7,HECW1,HMGB1,TNIK,TGFB2,MDFIC,LAMA2,TRIO,NDST1,MUSK,SFPQ,RGS3,NUP93,SLC15A4,GOLPH3,USP10,IGFBP7,EPHA5,SKOR2,ATP2C1,ASPH,UBR5,SOX30,SLIT3,CD226,ITPR1,PAK3,MLLT3,TNFRSF12A,OASL,LATS2,ERP29,ARHGAP18,LYN,KLF7,GRIK1,FIGNL1,NOS3,ERCC6,EIF2AK4,TGFBR2,MARK4,TCF7L2,SHISA2,RRAGD,ULK3,GKAP1,ADGRV1,RASGRF2,SCG5,WAC,DAPK1,APP,GATA4,SMAD5,GSKIP,PELI2,PIK3R5,TLR1,BMT2,AVPR1B,PTK2,USP7,TICAM2,SH2B2,PIP4K2B,LRRK2,CLSTN2,ITFG2,PKHD1,PJA2,TIAM1,IQSEC2,ACVR2A,BRCC3,SNX13,RABGEF1,KIT,EPHA7,SPAG9,WNT5A,TRABD2B,ARFGEF2,CTNNB1,ANKRD17,CTSK,PRKDC,ESR1,TNFRSF19,ERC2,B*

			CAR3,TPD52L1,VWC2L,GPC4,EFNA5,NGEF,NEUROD2,FNIP2,IKBKB,AKAP6,ARHGEF28,ACVR1,CHRNB3,PID1,GRID2,ZNF423,RB1,PDE4B,ADIPOR1,PLPP3,HNF1B,LAMP2,RGS17,GRIA4,RGS7BP,MGRN1,MID2,WLS,PRCP,CDK5RAP2,ARHGEF7,ATF3,TRDN,SRI,ARNT,BDNF,LDLRAD4,SEMA3E,TLR6,TMEM237,PPARG,CHRNB4,NEK7,TENM1,MYOF,NALCN,SIPA1L3,LRFN2,SFRP4,JCAD,VAV2,TFRC,GRIA3,FBN1,HAPSTR1,CLEC6A,BMPER,ANGPT1,SEMA6A,PHLPP1,ROCK1,TAX1BP1,APIP,PML,MGLL,AFAP1L2,DGKG,SP100,NDUFAF2,ENSA,AIMP1,SLC30A10,PRKD1,USP34,STK4,PRLR,PDE11A,NMI,LPAR6,CTBP2,HHIP,DYRK1A,MYRIP,UNC13C,RGS10,CUL4A,KCTD16,EYA4,SEMA5A,PMEPA1,METTL3,KCNN4,RNF6,VAV1,GRIA2,VDAC2,SPART
GO:0051649	establishment of localization in cell	0.0014953336759999916	*MREG,STX8,BLTP3B,TRAPPC9,PPM1A,RCSD1,MYO5C,AGTPBP1,ATP7B,GRIP1,RGPD2,XPO4,MAP6,NR4A3,PRKN,NLGN4X,XPO6,IFT172,GPSM1,SLC17A6,CFAP54,TRAK2,UBE2O,EIF4ENIF1,ADAR,CLCN3,FGF10,ZFYVE16,TSPAN18,CD84,RGPD3,ANO3,CIDEB,MCPH1,MYO1D,XPO7,DYNLT2B,MDFIC,NUP93,RBM33,EPHA5,SNX10,TMEM230,STON2,STRADA,ASPH,UBR5,HPS3,DNAAF11,TRAPPC10,CPT2,VPS8,CILK1,ERP29,LYN,KLF7,SEC24C,ATP6V1H,NUP58,SIL1,TMCC1,NOS3,MAGEL2,MDN1,SYNJ1,GPM6B,RGPD1,KPNB1,SCG5,WHRN,SAR1B,APP,RHOT1,USE1,HOOK3,LMAN1L,NOP9,USP7,SCP2,LRRK2,PLD1,PKHD1,TIAM1,ENSG00000288683,SNX13,RABGEF1,KIT,SPAG9,SPDL1,PEX26,CTNNB1,IPO5,PTPN14,SCFD1,ERC2,VPS53,PKIG,EFNA5,SYNE3,RAB3GAP2,XIST,ATF2,CCDC91,RB1,KIF13A,SMG7,LAMP2,RAB18,BACH2,MGRN1,KAT2B,WLS,MYO1B,CDK5RAP2,TRDN,SRI,ATP1B1,RAB11FIP2,MTTP,CHRNB4,TENM1,IL13RA2,MYOF,KIF2C,MYO5A,SLC6A13,SPTBN5,BLTP1,VPS13A,RAB31,CLASP2,ANGPT1,ATP6V0A4,ITGA4,DNAJC6,ROCK1,NUP98,PML,SCFD2,SP100,NDUFAF2,ANKFY1,AP4S1,PRKD1,STK4,RAB6D,SPC25,SCAMP1,RAB6C,CTBP2,TMEM30A,MYRIP,UNC13C,PARD3,KCNN4,VPS13B*
GO:0023051	regulation of signaling	0.0017290216233711469	*FSTL1,RGN,CDK14,MAP3K3,OSBPL8,ARHGEF9,ABR,MAOA,MAP3K13,PPM1A,LIMD1,CHST11,THRB,SPTBN1,USP32,CHRNB2,SEZ6,BRD4,DOK4,CAMK2B,SLC38A9,NOTCH2,TMOD2,NPNT,CDH11,SHANK3,FOXO1,STYXL1,STK39,SMURF2,ADAMTS12,TBC1D16,URB2,DIPK2A,PRKN,NLGN4X,TAFA1,FAM83B,IFT172,CDON,NLK,GNAI1,TIAM2,LRP8,ARHGEF4,UBE2O,DDIAS,ERBIN,RGS22,MCC,PTH,ADAR,PDE10A,CDC37,FGF10,MECOM,PLG,NCK1,NONO,NREP,PREX1,ALPK2,CHRNA7,HECW1,HMGB1,TNIK,TGFB2,MDFIC,LAMA2,TRIO,NDST1,MUSK,SFPQ,RGS3,NUP93,SLC15A4,GOLPH3,USP10,IGFBP7,EPHA5,SKOR2,ATP2C1,UBR5,SOX30,SLIT3,CD226,ITPR1,PAK3,MLLT3,TNFRSF12A,OASL,LATS2,ERP29,ARHGAP18,LYN,KLF7,GRIK1,FIGNL1,NOS3,ERCC6,EIF2AK4,TGFBR2,MARK4,TCF7L2,SHISA2,RRAGD,ULK3,GKAP1,ADGRV1,RASGRF2,SCG5,WAC,DAPK1,APP,GATA4,SMAD5,GSKIP,PELI2,PIK3R5,TLR1,BMT2,AVPR1B,PTK2,USP7,TICAM2,SH2B2,PIP4K2B,LRRK2,CLSTN2,ITFG2,PKHD1,PJA2,TIAM1,IQSEC2,ACVR2A,BRCC3,SNX13,RABGEF1,KIT,EPHA7,SPAG9,WNT5A,TRABD2B,ARFGEF2,CTNNB1,ANKRD17,CTSK,PRKDC,ESR1,TNFRSF19,ERC2,BCAR3,TPD52L1,VWC2L,GPC4,EFNA5,NGEF,NE*

			UROD2,FNIP2,IKBKB,AKAP6,ARHGEF28,ACVR1,CHRNB3,PID1,GRID2,ZNF423,RB1,PDE4B,ADIPOR1,PLPP3,HNF1B,LAMP2,RGS17,GRIA4,RGS7BP,MGRN1,MID2,WLS,PRCP,CDK5RAP2,ARHGEF7,ATF3,SRI,ARNT,BDNF,LDLRAD4,SEMA3E,TLR6,TMEM237,PPARG,CHRNB4,NEK7,TENM1,MYOF,NALCN,SIPA1L3,LRFN2,SFRP4,JCAD,VAV2,TFRC,GRIA3,FBN1,HAPSTR1,CLEC6A,BMPER,ANGPT1,SEMA6A,PHLPP1,ROCK1,TAX1BP1,APIP,PML,MGLL,AFAP1L2,DGKG,SP100,NDUFAF2,ENSA,AIMP1,SLC30A10,PRKD1,USP34,STK4,PRLR,PDE11A,NMI,LPAR6,CTBP2,HHIP,DYRK1A,MYRIP,UNC13C,RGS10,CUL4A,KCTD16,EYA4,SEMA5A,PMEPA1,METTL3,KCNN4,RNF6,VAV1,GRIA2,VDAC2,SPART
GO:0007154	cell communication	0.002577383624261571	*FSTL1,APBB2,RGN,CDK14,PDE1C,NOSTRIN,MAP3K3,OSBPL8,RAPGEF6,ARHGEF9,TAFA2,ABR,MAOA,MAP3K13,PPM1A,ARL13B,LIMD1,TF,CHST11,THRB,SPTBN1,CDC42EP3,MAPK10,CACNB1,USP32,DAPP1,GNG12,CHRNB2,SEZ6,CDH8,GNB4,RBPMS,SKAP2,BRD4,DOK4,CAMK2B,GRIP1,ARHGAP11A-SCG5,DLGAP1,SLC38A9,HBP1,ABCG8,MAP4K3,NOTCH2,TMOD2,NPNT,CDH11,SHANK3,NR4A3,E2F7,FOXO1,STYXL1,STK39,SMURF2,ADAMTS12,PLXNA4,URB2,DIPK2A,DCDC1,CACNA1C,FAM3C,ASB4,PRKN,NLGN4X,TAFA1,PIK3C2B,SLC5A7,FAM83B,IFT172,CDON,SLC17A6,NLK,GNAI1,MAPKAPK3,TIAM2,LRP8,CNTN1,STAT5B,ENPEP,ARHGEF4,UBE2O,DDIAS,CPEB4,CHRFAM7A,ERBIN,MBTPS2,RGS22,CD247,MCC,PTH,ADAR,PDE10A,CLCN3,RHOJ,CDC37,FGF10,ZFYVE16,MECOM,COL4A6,STK32B,PLG,HERC5,ILDR2,NCK1,NONO,NREP,GABRA5,MTCP1,PREX1,CIDEB,COL1A2,DTNA,ALPK2,CHRNA7,HECW1,HMGB1,ZNF304,CDIP1,HIVEP1,ATRX,TNIK,TGFB2,MDFIC,LAMA2,TRIO,SMARCC1,NDST1,MUSK,JAM3,KCNIP3,SFPQ,RGS3,NUP93,RERG,OR5AU1,PRKAR2A,SLC15A4,GOLPH3,PRKG2,MBTPS1,USP10,IGFBP7,EPHA5,SKOR2,ATP2C1,ALDH1A2,PTGFR,ASPH,RRAS2,UBR5,SOX30,ANKS1B,CHL1,TCP11L1,TLR8,SLIT3,CD226,ITPR1,PKP4,PAK3,PRKAG2,MLLT3,TNFRSF12A,OASL,TAS2R14,CXCR5,NCKAP1,CILK1,FOSL2,IL1RAPL2,ADGRD1,EPHA3,LATS2,MYH14,ERP29,ARHGAP18,LYN,ADGRB3,KLF7,GRIK1,FIGNL1,PCDHB16,TMED7-TICAM2,NOS3,ERCC6,EIF2AK4,RAB12,TGFBR2,ITGA11,CMKLR2,MARK4,SYNJ1,TCF7L2,UNC119,SHISA2,RRAGD,ULK3,GPR156,GKAP1,PPP2R5E,ADGRV1,RASGRF2,GAD1,SCG5,WAC,DAPK1,TDP2,INSYN2A,APP,GATA4,SMAD5,GSKIP,ADAM12,PELI2,HCN1,CHRM5,PI4KB,RHOT1,PIK3R5,TLR1,TAS2R30,BMT2,AVPR1B,PTK2,USP7,TICAM2,SH2B2,PIP4K2B,LRRK2,CLSTN2,ARIH1,PLD1,VTCN1,ITFG2,PKHD1,RARB,PJA2,TIAM1,CSRP3,IQSEC2,ACVR2A,GNAO1,TIPIN,RASSF4,BRCC3,SNX13,RABGEF1,KIT,CYB5R4,EPHA7,SPAG9,SPDL1,WNT5A,TRABD2B,TEX2,ARFGEF2,CTNNB1,ANKRD17,CTSK,PRKDC,IPO5,ESR1,TNFRSF19,ERC2,BCAR3,TPD52L1,HUNK,PTGER4,PDE7B,VWC2L,DOCK1,GPC4,EFNA5,CDC14B,PLCH1,OR1M1,NGEF,NEUROD2,FNIP2,PTPRF,IKBKB,SH3BP5,AKAP6,TRBV10-2,ARHGEF28,CLCN1,ATF2,ACVR1,CHRNB3,PID1,GRID2,ZNF423,KCNJ3,PLPPR1,RB1,IFNLR1,PDE4B,ADIPOR1,GABRB3,PLPP3,HNF1B,LAMP2,GABRG2,NCOA2,BNIP3L,RGS17,GFRA1,KCND2,INPP4A,BMX,GRIA4,RAB18,RGS7BP,MGRN1,MID2,SIK1,*

			TP53BP2,WLS,ANKS1A,RYR3,PRCP,CDK5RAP2,ARHGEF7,SAMD12,ATF3,TRDN,NPHP1,SRI,ATP1B1,ARNT,BDNF,LDLRAD4,SEMA3E,MACO1,TLR6,TMEM237,RAB11FIP2,LAG3,PPARG,PAPPA,ASB3,CHRNB4,NEK7,TENM1,IL13RA2,RORB,GABRB1,MYOF,LTB4R2,SH3KBP1,NALCN,MYO5A,SIPA1L3,LRFN2,SFRP4,IL1RAP,JCAD,VAV2,TFRC,GRIA3,PNPLA8,FBN1,HAPSTR1,MAP3K1,CLEC6A,BMPER,ANGPT1,SEMA6A,VIPR2,PHLPP1,ITGA4,ROCK1,TAX1BP1,APIP,PML,MGLL,PLEK2,TNFSF4,AFAP1L2,DGKG,SP100,NDUFAF2,SPG21,ENSA,AIMP1,SLC30A10,PRKD1,CALCRL,USP34,STK4,TNNI3K,SPC25,PRLR,PDE11A,NMI,RAB6C,IL16,EDARADD,GNA14,CR2,LPAR6,CTBP2,TTN,NCALD,HHIP,DYRK1A,PPFIA1,MYRIP,PPP1R1C,UNC13C,RGS10,CUL4A,KCTD16,EYA4,SEMA5A,PMEPA1,METTL3,KCNN4,RNF6,SEMA3C,VAV1,GRIA2,VDAC2,SPART
GO:0030029	actin filament-based process	0.00351756765129002	NOSTRIN,TF,SPTBN1,CDC42EP3,MYO5C,TMOD2,EPB41L3,PARVB,SHANK3,CACNA1C,PRKN,THSD7A,TAGLN3,RHOJ,FGF10,NCK1,PREX1,MYO1D,TMOD3,RFLNA,TNIK,EPB41L4A,ADD3,JAM3,EPHA5,ENAH,ATP2C1,FAM171A1,GSN,PLS1,PAK3,NCKAP1,EPHA3,MYH14,ARHGAP18,MYOM2,MAGEL2,GPM6B,EPB41L5,GATA4,RHOT1,PALLD,SH2B2,CSRP3,PHACTR1,IQSEC2,NEDD4L,KIT,ANTXR1,PPM1E,PTGER4,EFNA5,IKBKB,KCNJ3,PDE4B,SHROOM4,ACTR3C,MYO1B,NPHP1,CAPZA2,SRI,SCIN,TENM1,SH3KBP1,AVIL,MYO5A,SPTBN5,CLASP2,ROCK1,PLEK2,PRKD1,TTN,PPFIA1,KCNE2,SEMA5A,FMNL2
GO:0141124	intracellular signaling cassette	0.005538352028688843	RGN,MAP3K3,OSBPL8,RAPGEF6,ARHGEF9,ABR,MAP3K13,PPM1A,TF,SPTBN1,CDC42EP3,MAPK10,RBPMS,BRD4,DOK4,MAP4K3,NOTCH2,NPNT,SHANK3,FOXO1,STK39,DIPK2A,CACNA1C,PRKN,PIK3C2B,CDON,NLK,GNAI1,MAPKAPK3,TIAM2,ARHGEF4,PTH,PDE10A,RHOJ,FGF10,MECOM,HERC5,NCK1,PREX1,COL1A2,CHRNA7,HMGB1,TNIK,TGFB2,MDFIC,TRIO,NDST1,MUSK,NUP93,RERG,USP10,EPHA5,ATP2C1,ASPH,RRAS2,TLR8,ITPR1,PAK3,CXCR5,NCKAP1,ERP29,ARHGAP18,LYN,NOS3,ERCC6,EIF2AK4,RAB12,TGFBR2,TCF7L2,RASGRF2,APP,SMAD5,PELI2,RHOT1,PIK3R5,AVPR1B,PTK2,TICAM2,SH2B2,LRRK2,ARIH1,PLD1,PKHD1,PJA2,TIAM1,IQSEC2,ACVR2A,RABGEF1,KIT,EPHA7,SPAG9,WNT5A,ARFGEF2,CTNNB1,ANKRD17,ESR1,TNFRSF19,BCAR3,TPD52L1,PTGER4,PDE7B,DOCK1,NGEF,NEUROD2,IKBKB,AKAP6,ARHGEF28,ATF2,ACVR1,RB1,ADIPOR1,RAB18,MGRN1,MID2,WLS,RYR3,ARHGEF7,ATF3,ATP1B1,LDLRAD4,SEMA3E,TLR6,PPARG,TENM1,SIPA1L3,JCAD,VAV2,TFRC,MAP3K1,CLEC6A,BMPER,ANGPT1,SEMA6A,PHLPP1,ROCK1,APIP,PML,SLC30A10,PRKD1,STK4,PDE11A,NMI,RAB6C,LPAR6,NCALD,SEMA5A,PMEPA1,VAV1
GO:0030036	actin cytoskeleton organization	0.005837453120505308	NOSTRIN,TF,SPTBN1,CDC42EP3,MYO5C,TMOD2,EPB41L3,PARVB,SHANK3,PRKN,THSD7A,TAGLN3,RHOJ,FGF10,NCK1,PREX1,MYO1D,TMOD3,RFLNA,TNIK,EPB41L4A,ADD3,JAM3,EPHA5,ENAH,ATP2C1,FAM171A1,GSN,PLS1,PAK3,NCKAP1,EPHA3,MYH14,ARHGAP18,MYOM2,MAGEL2,GPM6B,EPB41L5,RHOT1,PALLD,SH2B2,CSRP3,PHACTR1,IQSEC2,KIT,ANTXR1,PPM1E,PTGER4,EFNA5,IKBKB,SHROOM4,ACTR3C,MYO1B,NPHP1,CAPZA2,SCIN,TENM1,SH3KBP1,AVIL,MYO5A,SPTBN5,CLASP2,ROCK1,PLEK2,PRKD1,TTN,PPFIA1,SEMA5A,FMNL2

GO:0051128	regulation of cellular component organization	0.007585304586217583	*C10ORF90,LRRN1,ARHGEF9,MAP3K13,TF,SPTBN1,CDC42EP3,CHRNB2,SEZ6,CDH8,FHL1,ATP10A,CAMK2B,GRIP1,TMOD2,HMBOX1,MAP6,SHANK3,ARID1B,STYXL1,PLXNA4,PRKN,SERPINI1,GNAI1,TIAM2,LRP8,CNTN1,ZFYVE16,NCK1,KDM2B,PREX1,MCPH1,TMOD3,CHRNA7,HECW1,HMGB1,DYNLT2B,ATRX,TNIK,TGFB2,LAMA2,ADD3,SMARCC1,MUSK,JAM3,RERG,GOLPH3,USP10,IGFBP7,EPHA5,SKOR2,STON2,HUWE1,ASPH,GSN,PLS1,MAPRE3,SLIT3,ARMH3,PAK3,MLLT3,VPS8,NCKAP1,IL1RAPL2,EPHA3,ARHGAP18,UVRAG,LYN,ADGRB3,DIP2B,NME7,DCP2,MAGEL2,MARK4,SYNJ1,CUX1,UNC119,GPM6B,EPB41L5,PRUNE1,SAR1B,APP,RHOT1,PTK2,USP7,PIP4K2B,LRRK2,INO80D,CLSTN2,PLD1,PKHD1,TIAM1,NEDD4L,BRCC3,RABGEF1,KIT,EPHA7,SPAG9,ANTXR1,SPDL1,WNT5A,TRABD2B,CTNNB1,SCFD1,ERC2,PPM1E,LMX1A,PTGER4,GPC4,EFNA5,NGEF,TRPC5,NEUROD2,FNIP2,PTPRF,IKBKB,FXR2,AKAP6,RAB3GAP2,LINGO2,HDAC8,PID1,GRID2,RB1,NSMCE1,ADIPOR1,LAMP2,XRCC5,LRTM2,KAT2B,SUPV3L1,CDK5RAP2,ARHGEF7,NPHP1,CAPZA2,BDNF,SEMA3E,SCIN,TLR6,HAUS6,PPARG,TANC1,NEK7,TENM1,AVIL,LRFN2,SPTBN5,SFRP4,BLTP1,IL1RAP,TFRC,RAB31,CLASP2,ANGPT1,SEMA6A,EIF4G1,DNAJC6,ROCK1,NCAPH,TBC1D4,PML,PLEK2,DGKG,ELAVL4,NIN,ANKFY1,PRKD1,SPC25,RAB6C,YLPM1,DYRK1A,PPFIA1,TMEM30A,SEMA5A,ATP8B1,RNF6,SEMA3C,VDAC2,SPART*
GO:0023052	signaling	0.007789568973753834	*FSTL1,APBB2,RGN,CDK14,PDE1C,NOSTRIN,MAP3K3,OSBPL8,RAPGEF6,ARHGEF9,TAFA2,ABR,MAOA,MAP3K13,PPM1A,ARL13B,LIMD1,TF,CHST11,THRB,SPTBN1,CDC42EP3,MAPK10,CACNB1,USP32,DAPP1,GNG12,CHRNB2,SEZ6,CDH8,GNB4,RBPMS,SKAP2,BRD4,DOK4,CAMK2B,GRIP1,ARHGAP11A-SCG5,DLGAP1,SLC38A9,HBP1,ABCG8,MAP4K3,NOTCH2,TMOD2,NPNT,CDH11,SHANK3,NR4A3,E2F7,FOXO1,STYXL1,STK39,SMURF2,ADAMTS12,TBC1D16,PLXNA4,URB2,DIPK2A,DCDC1,CACNA1C,FAM3C,ASB4,PRKN,NLGN4X,TAFA1,PIK3C2B,SLC5A7,FAM83B,IFT172,CDON,SLC17A6,NLK,GNAI1,MAPKAPK3,TIAM2,LRP8,CNTN1,STAT5B,ENPEP,ARHGEF4,UBE2O,DDIAS,CPEB4,CHRFAM7A,ERBIN,MBTPS2,RGS22,CD247,MCC,PTH,ADAR,PDE10A,CLCN3,RHOJ,CDC37,FGF10,ZFYVE16,MECOM,COL4A6,STK32B,PLG,HERC5,ILDR2,NCK1,NONO,NREP,GABRA5,MTCP1,PREX1,CIDEB,COL1A2,DTNA,ALPK2,CHRNA7,HECW1,HMGB1,ZNF304,CDIP1,HIVEP1,ATRX,TNIK,TGFB2,MDFIC,LAMA2,TRIO,SMARCC1,NDST1,MUSK,KCNIP3,SFPQ,RGS3,NUP93,RERG,OR5AU1,PRKAR2A,SLC15A4,GOLPH3,PRKG2,MBTPS1,USP10,IGFBP7,EPHA5,SKOR2,ATP2C1,ALDH1A2,PTGFR,ASPH,RRAS2,UBR5,SOX30,ANKS1B,CHL1,TCP11L1,TLR8,SLIT3,CD226,ITPR1,PKP4,PAK3,PRKAG2,MLLT3,TNFRSF12A,OASL,TAS2R14,CXCR5,NCKAP1,CILK1,FOSL2,IL1RAPL2,ADGRD1,EPHA3,LATS2,ERP29,ARHGAP18,LYN,ADGRB3,KLF7,GRIK1,FIGNL1,PCDHB16,TMED7-TICAM2,NOS3,ERCC6,EIF2AK4,RAB12,TGFBR2,ITGA11,CMKLR2,MARK4,SYNJ1,TCF7L2,UNC119,SHISA2,RRAGD,ULK3,GPR156,GKAP1,PPP2R5E,ADGRV1,RASGRF2,GAD1,SCG5,WAC,DAPK1,TDP2,INSYN2A,APP,GATA4,SMAD5,GSKIP,ADAM12,PELI2,CHRM5,PI4KB,RHOT1,PIK3R5,TLR1,TAS2R30,BMT2,AVPR1B,PTK2,USP7,TICAM2,SH2B2,PIP4K2*

			B,LRRK2,CLSTN2,ARIH1,PLD1,VTCN1,ITFG2,PKHD1,RARB,PJA2,TIAM1,CSRP3,IQSEC2,ACVR2A,GNAO1,TIPIN,RASSF4,BRCC3,SNX13,RABGEF1,KIT,CYB5R4,EPHA7,SPAG9,SPDL1,WNT5A,TRABD2B,TEX2,ARFGEF2,CTNNB1,ANKRD17,CTSK,PRKDC,IPO5,ESR1,TNFRSF19,ERC2,BCAR3,TPD52L1,HUNK,PTGER4,PDE7B,VWC2L,DOCK1,GPC4,EFNA5,CDC14B,PLCH1,OR1M1,NGEF,NEUROD2,FNIP2,PTPRF,IKBKB,SH3BP5,AKAP6,TRBV10-2,ARHGEF28,ATF2,ACVR1,CHRNB3,PID1,GRID2,ZNF423,KCNJ3,PLPPR1,RB1,IFNLR1,PDE4B,ADIPOR1,GABRB3,PLPP3,HNF1B,LAMP2,GABRG2,NCOA2,BNIP3L,RGS17,GFRA1,KCND2,INPP4A,BMX,GRIA4,RAB18,RGS7BP,MGRN1,MID2,SIK1,TP53BP2,WLS,ANKS1A,RYR3,PRCP,CDK5RAP2,ARHGEF7,SAMD12,ATF3,NPHP1,SRI,ATP1B1,ARNT,BDNF,LDLRAD4,SEMA3E,MACO1,TLR6,TMEM237,RAB11FIP2,LAG3,PPARG,PAPPA,ASB3,CHRNB4,NEK7,TENM1,IL13RA2,RORB,GABRB1,MYOF,LTB4R2,SH3KBP1,NALCN,MYO5A,SIPA1L3,LRFN2,SFRP4,IL1RAP,JCAD,VAV2,TFRC,GRIA3,PNPLA8,FBN1,HAPSTR1,MAP3K1,CLEC6A,BMPER,ANGPT1,SEMA6A,VIPR2,PHLPP1,ITGA4,ROCK1,TAX1BP1,APIP,PML,MGLL,PLEK2,TNFSF4,AFAP1L2,DGKG,SP100,NDUFAF2,SPG21,ENSA,AIMP1,SLC30A10,PRKD1,CALCRL,USP34,STK4,SPC25,PRLR,PDE11A,NMI,RAB6C,IL16,EDARADD,GNA14,CR2,LPAR6,CTBP2,TTN,NCALD,HHIP,DYRK1A,PPFIA1,MYRIP,PPP1R1C,UNC13C,RGS10,CUL4A,KCTD16,EYA4,SEMA5A,PMEPA1,METTL3,KCNN4,RNF6,SEMA3C,VAV1,GRIA2,VDAC2,SPART*
GO:0010604	positive regulation of macromolecule metabolic process	0.008184075499643242	*APBB2,RGN,ING3,PHF20,FOXK2,SMYD3,MAP3K13,PPM1A,TF,THRB,SPTBN1,ATF7,AGTPBP1,RBPMS,GPBP1L1,BRD4,NOTCH2,HMBOX1,NPNT,NR4A3,ARID1B,E2F7,FOXO1,FER1L6,YBX1,DIPK2A,IDO1,PRKN,KDM7A,CDON,LRP8,CNTN1,MEOX2,STAT5B,EIF4ENIF1,MBTPS2,PTH,ADAR,FGF10,MECOM,NCK1,CD84,FAM168A,MTCP1,RSF1,NCOA7,MRTFB,CHRNA7,HMGB1,ZNF304,HIVEP1,ATRX,TNIK,TGFB2,MDFIC,HOXC13,SMARCC1,MUSK,SFPQ,ZCCHC4,CTIF,RBM23,ALDH1A2,PTGFR,STRADA,HUWE1,ASPH,SH3BGRL,UBR5,GTF2F2,SOX30,GSN,SPON1,MAPRE3,TLR8,CACUL1,CD226,KLF12,PRKAG2,MLLT3,CCNT2,CNOT10,FOSL2,SPDYE2B,ERP29,LYN,KLF7,SUMO2,MTA3,DIP2B,LDB2,MYRFL,RNASEL,DBF4B,DCP2,NOS3,TNRC6C,MLLT10,ERCC6,EIF2AK4,TGFBR2,TCF7L2,ZNF462,ZFHX3,UNC119,SPDYE2,GSDMD,WAC,WHRN,APP,GATA4,SMAD5,IGF2BP1,PELI2,PIK3R5,TLR1,PTK2,TICAM2,CELF1,POGZ,LRRK2,INO80D,ZNF395,IKZF3,ARIH1,PLD1,VTCN1,TENT5C,RARB,TCF4,PAEP,COP1,CSRP3,MLIP,ACVR2A,NEDD4L,BRCC3,KIT,EPHA7,WNT5A,TRABD2B,ARFGEF2,CTNNB1,PRKDC,ESR1,BCAR3,TPD52L1,ZXDC,LMX1A,PTGER4,EFNA5,IGF2BP2,CDC14B,HECTD1,TRPC5,NEUROD2,FNIP2,IKBKB,FXR2,USF3,RAB3GAP2,CCPG1,CEMIP,ATF2,ACVR1,PID1,EBF2,ZNF423,RB1,PDE4B,ZNF143,PLPP3,HNF1B,ZNF615,NCOA2,XRCC5,SHLD2,GFRA1,SLC24A3,TAF15,POU2F1,ETV1,MID2,ZNF850,KAT2B,SUPV3L1,MEF2D,GTF2A1,CDK5RAP2,RFC3,ATF3,ARNT,BDNF,TFCP2L1,BCLAF3,TLR6,PPARG,NEK7,TENM1,RORB,SFRP4,SPDYE16,IL1RAP,TFRC,SPDYE6,ZNF292,CLEC6A,ZNF521,ANGPT1,EIF4G1,ROCK1,NUP98,PML,TNFSF4,AFAP1L2,SP1*

			00,ELAVL4,PRKD1,HSPBP1,STK4,PRLR,NMI,IL16,CTBP2,TTN,DYRK1A,KCNE2,CUL4A,EYA4,ELL2,PVT1,GATAD2B,METTL3,UAP1,ZNF33A,BMAL2,RNF6
GO:0009966	regulation of signal transduction	0.008433769926545591	*FSTL1,RGN,CDK14,MAP3K3,OSBPL8,ARHGEF9,ABR,MAOA,MAP3K13,PPM1A,LIMD1,CHST11,THRB,SPTBN1,USP32,SEZ6,BRD4,DOK4,SLC38A9,NOTCH2,TMOD2,NPNT,SHANK3,FOXO1,STYXL1,STK39,SMURF2,ADAMTS12,URB2,DIPK2A,PRKN,NLGN4X,TAFA1,FAM83B,IFT172,CDON,NLK,GNAI1,TIAM2,ARHGEF4,UBE2O,DDIAS,ERBIN,RGS22,MCC,PTH,ADAR,PDE10A,CDC37,FGF10,MECOM,NCK1,NONO,NREP,PREX1,ALPK2,CHRNA7,HECW1,HMGB1,TNIK,TGFB2,MDFIC,LAMA2,TRIO,NDST1,MUSK,SFPQ,RGS3,NUP93,SLC15A4,GOLPH3,USP10,IGFBP7,SKOR2,ATP2C1,UBR5,SOX30,SLIT3,CD226,ITPR1,PAK3,MLLT3,TNFRSF12A,OASL,LATS2,ERP29,ARHGAP18,LYN,FIGNL1,NOS3,ERCC6,TGFBR2,MARK4,TCF7L2,SHISA2,RRAGD,ULK3,GKAP1,ADGRV1,RASGRF2,WAC,DAPK1,APP,GATA4,SMAD5,GSKIP,PELI2,PIK3R5,TLR1,BMT2,AVPR1B,PTK2,USP7,TICAM2,SH2B2,PIP4K2B,LRRK2,ITFG2,PKHD1,PJA2,TIAM1,IQSEC2,ACVR2A,BRCC3,SNX13,RABGEF1,KIT,EPHA7,SPAG9,WNT5A,TRABD2B,ARFGEF2,CTNNB1,ANKRD17,CTSK,PRKDC,ESR1,TNFRSF19,BCAR3,TPD52L1,VWC2L,GPC4,NGEF,NEUROD2,FNIP2,IKBKB,AKAP6,ARHGEF28,ACVR1,PID1,ZNF423,RB1,PDE4B,ADIPOR1,PLPP3,HNF1B,LAMP2,RGS17,RGS7BP,MGRN1,MID2,WLS,PRCP,CDK5RAP2,ATF3,ARNT,BDNF,LDLRAD4,SEMA3E,TLR6,TMEM237,PPARG,NEK7,TENM1,SIPA1L3,SFRP4,JCAD,VAV2,TFRC,FBN1,HAPSTR1,CLEC6A,BMPER,ANGPT1,SEMA6A,PHLPP1,ROCK1,TAX1BP1,APIP,PML,MGLL,AFAP1L2,DGKG,SP100,SLC30A10,PRKD1,USP34,STK4,PRLR,PDE11A,NMI,LPAR6,CTBP2,HHIP,DYRK1A,RGS10,CUL4A,KCTD16,EYA4,SEMA5A,PMEPA1,METTL3,KCNN4,RNF6,VAV1,VDAC2,SPART*
GO:0003012	muscle system process	0.00931106816803071	*APBB2,RCSD1,CACNB1,CHRNB2,CAMK2B,TMOD2,NPNT,NR4A3,FOXO1,CACNA1C,DTNA,TMOD3,ASPH,GSN,MYH14,MYOM2,NOS3,GATA4,SMAD5,TIAM1,CSRP3,MLIP,GNAO1,NEDD4L,KIT,AKAP6,CLCN1,KCNJ12,KCNJ3,PDE4B,SSPN,ACACB,KCND2,RYR3,MYL4,TRDN,SRI,ATP1B1,PPARG,ASB3,CHRNB4,MYOF,ROCK1,PRKD1,TNNI3K,TTN,KCNE2*
GO:0050808	synapse organization	0.00980541420291577	*APBB2,LRRN1,ARHGEF9,CACNB1,CHRNB2,SEZ6,CDH8,CAMK2B,SDK2,SHANK3,PLXNA4,NLGN4X,LRP8,CHRNA7,MUSK,PAK3,IL1RAPL2,ADGRB3,PCDHB16,APP,LRRK2,CLSTN2,TIAM1,EPHA7,WNT5A,CTNNB1,ERC2,LMX1A,FILIP1,GPC4,EFNA5,NGEF,NEUROD2,PTPRF,LINGO2,GRID2,GABRB3,KIRREL3,GABRG2,LRTM2,BDNF,SEMA3E,TANC1,LRFN2,IL1RAP,EIF4G1,ROCK1,CDH9,CTBP2,PPFIA1,UNC13C*
GO:0007165	signal transduction	0.01438558766427421	*FSTL1,APBB2,RGN,CDK14,PDE1C,NOSTRIN,MAP3K3,OSBPL8,RAPGEF6,ARHGEF9,TAFA2,ABR,MAOA,MAP3K13,PPM1A,ARL13B,LIMD1,TF,CHST11,THRB,SPTBN1,CDC42EP3,MAPK10,USP32,DAPP1,GNG12,CHRNB2,SEZ6,GNB4,RBPMS,SKAP2,BRD4,DOK4,CAMK2B,GRIP1,ARHGAP11A-SCG5,SLC38A9,HBP1,ABCG8,MAP4K3,NOTCH2,TMOD2,NPNT,SHANK3,NR4A3,E2F7,FOXO1,STYXL1,STK39,SMURF2,ADAMTS12,PLXNA4,URB2,DIPK2A,DCDC1,CACNA1C,FAM3C,ASB4,PRKN,NLGN4X,TA*

			FA1,PIK3C2B,FAM83B,IFT172,CDON,NLK,GNAI1,MAPKAPK3,TIAM2,LRP8,CNTN1,STAT5B,ARHGEF4,UBE2O,DDIAS,CPEB4,CHRFAM7A,ERBIN,MBTPS2,RGS22,CD247,MCC,PTH,ADAR,PDE10A,RHOJ,CDC37,FGF10,ZFYVE16,MECOM,COL4A6,STK32B,HERC5,NCK1,NONO,NREP,GABRA5,MTCP1,PREX1,CIDEB,COL1A2,DTNA,ALPK2,CHRNA7,HECW1,HMGB1,ZNF304,CDIP1,HIVEP1,ATRX,TNIK,TGFB2,MDFIC,LAMA2,TRIO,SMARCC1,NDST1,MUSK,KCNIP3,SFPQ,RGS3,NUP93,RERG,OR5AU1,PRKAR2A,SLC15A4,GOLPH3,PRKG2,MBTPS1,USP10,IGFBP7,EPHA5,SKOR2,ATP2C1,ALDH1A2,PTGFR,ASPH,RRAS2,UBR5,SOX30,ANKS1B,CHL1,TCP11L1,TLR8,SLIT3,CD226,ITPR1,PAK3,PRKAG2,MLLT3,TNFRSF12A,OASL,TAS2R14,CXCR5,NCKAP1,CILK1,FOSL2,IL1RAPL2,ADGRD1,EPHA3,LATS2,ERP29,ARHGAP18,LYN,ADGRB3,GRIK1,FIGNL1,TMED7-TICAM2,NOS3,ERCC6,EIF2AK4,RAB12,TGFBR2,ITGA11,CMKLR2,MARK4,TCF7L2,UNC119,SHISA2,RRAGD,ULK3,GPR156,GKAP1,PPP2R5E,ADGRV1,RASGRF2,SCG5,WAC,DAPK1,TDP2,INSYN2A,APP,GATA4,SMAD5,GSKIP,ADAM12,PELI2,CHRM5,PI4KB,RHOT1,PIK3R5,TLR1,TAS2R30,BMT2,AVPR1B,PTK2,USP7,TICAM2,SH2B2,PIP4K2B,LRRK2,ARIH1,PLD1,VTCN1,ITFG2,PKHD1,RARB,PJA2,TIAM1,CSRP3,IQSEC2,ACVR2A,GNAO1,TIPIN,RASSF4,BRCC3,SNX13,RABGEF1,KIT,EPHA7,SPAG9,SPDL1,WNT5A,TRABD2B,TEX2,ARFGEF2,CTNNB1,ANKRD17,CTSK,PRKDC,IPO5,ESR1,TNFRSF19,BCAR3,TPD52L1,HUNK,PTGER4,PDE7B,VWC2L,DOCK1,GPC4,EFNA5,CDC14B,PLCH1,OR1M1,NGEF,NEUROD2,FNIP2,PTPRF,IKBKB,SH3BP5,AKAP6,TRBV10-2,ARHGEF28,ATF2,ACVR1,CHRNB3,PID1,GRID2,ZNF423,PLPPR1,RB1,IFNLR1,PDE4B,ADIPOR1,GABRB3,PLPP3,HNF1B,LAMP2,NCOA2,BNIP3L,RGS17,GFRA1,INPP4A,BMX,GRIA4,RAB18,RGS7BP,MGRN1,MID2,SIK1,TP53BP2,WLS,ANKS1A,RYR3,PRCP,CDK5RAP2,ARHGEF7,SAMD12,ATF3,NPHP1,SRI,ATP1B1,ARNT,BDNF,LDLRAD4,SEMA3E,MACO1,TLR6,TMEM237,RAB11FIP2,LAG3,PPARG,PAPPA,ASB3,CHRNB4,NEK7,TENM1,IL13RA2,RORB,GABRB1,LTB4R2,MYO5A,SIPA1L3,SFRP4,IL1RAP,JCAD,VAV2,TFRC,GRIA3,PNPLA8,FBN1,HAPSTR1,MAP3K1,CLEC6A,BMPER,ANGPT1,SEMA6A,VIPR2,PHLPP1,ITGA4,ROCK1,TAX1BP1,APIP,PML,MGLL,PLEK2,TNFSF4,AFAP1L2,DGKG,SP100,SPG21,AIMP1,SLC30A10,PRKD1,CALCRL,USP34,STK4,SPC25,PRLR,PDE11A,NMI,RAB6C,IL16,EDARADD,GNA14,CR2,LPAR6,CTBP2,TTN,NCALD,HHIP,DYRK1A,PPFIA1,PPP1R1C,RGS10,CUL4A,KCTD16,EYA4,SEMA5A,PMEPA1,METTL3,KCNN4,RNF6,SEMA3C,VAV1,GRIA2,VDAC2,SPART*
GO:0007010	cytoskeleton organization	0.019151116100950397	*C10ORF90,NOSTRIN,NINL,LIMD1,TF,SPTBN1,CDC42EP3,MYO5C,CDC14C,TMOD2,EPB41L3,MAP6,PARVB,SHANK3,PRKN,IFT172,GPSM1,THSD7A,GNAI1,TAGLN3,ERBIN,CEP192,RHOJ,FGF10,NCK1,PREX1,MCPH1,MYO1D,TMOD3,ABLIM1,RFLNA,ATRX,TNIK,EPB41L4A,ADD3,JAM3,EPHA5,ENAH,ATP2C1,FAM171A1,GSN,PLS1,MAPRE3,DNAAF11,PAK3,NCKAP1,PRPF40A,EPHA3,MYH14,ARHGAP18,UVRAG,MYOM2,NME7,FIGNL1,PDZD8,NAV1,KIAA1614,MAGEL2,MARK4,GPM6B,KPNB1,EPB41L5,PRUNE1,RHOT1,HOOK3,PALLD,CECR2,PTK2,SH2B2,PKHD1,CSRP3,PHACTR1,IQSEC2,KIT,ANTXR1,SPDL1,*

			CTNNB1,PPM1E,FIGN,PTGER4,EFNA5,CDC14B,SYNE3,IKBKB,ARHGEF28,ATF2,SHROOM4,KAT2B,ACTR3C,MYO1B,CDK5RAP2,ARHGEF7,TRDN,NPHP1,CAPZA2,SCIN,HAUS6,NEK7,TENM1,SH3KBP1,AVIL,KIF2C,MYO5A,SIPA1L3,SPTBN5,CLASP2,SEMA6A,ROCK1,PLEK2,NIN,PRKD1,SPC25,RAB6C,TTN,DYRK1A,PPFIA1,MAP7D3,SEMA5A,FMNL2,KRT85,PARD3
GO:0010975	regulation of neuron projection development	0.020387367090139268	*MAP3K13,CHRNB2,SEZ6,CAMK2B,MAP6,SHANK3,STYXL1,PLXNA4,SERPINI1,TIAM2,LRP8,CNTN1,NCK1,PREX1,HECW1,TNIK,MUSK,SKOR2,PAK3,EPHA3,LYN,ADGRB3,DIP2B,CUX1,LRRK2,TIAM1,NEDD4L,EPHA7,WNT5A,EFNA5,NGEF,TRPC5,PTPRF,GRID2,KAT2B,BDNF,SEMA3E,AVIL,SEMA6A,DGKG,ELAVL4,NIN,PRKD1,TMEM30A,SEMA5A,RNF6,SEMA3C,SPART*
GO:0022604	regulation of cell morphogenesis	0.02140712356504779	*MAP3K13,LIMD1,CDC42EP3,ATP10A,CAMK2B,GRIP1,EPB41L3,PARVB,PLXNA4,PRKN,LRP8,RHOJ,FAM171A1,PRPF40A,MYH14,ARHGAP18,PDZD8,CUX1,PTK2,TIAM1,NEDD4L,KIT,SPAG9,WNT5A,PRKDC,EFNA5,SYNE3,ARHGEF7,SEMA3E,SH3KBP1,FMNL2*
GO:0019220	regulation of phosphate metabolic process	0.02545133617638085	*RGN,SMYD3,MAP3K13,SAMSN1,TF,DIPK2A,PRKN,CDON,LRP8,PTH,ADAR,CDC37,FGF10,HERC5,NCK1,MTCP1,CHRNA7,TNIK,TGFB2,MUSK,PRKAR2A,STRADA,MAPRE3,TLR8,CACUL1,PRKAG2,CCNT2,LATS2,SPDYE2B,ERP29,UVRAG,LYN,LDB2,NOS3,ERCC6,TGFBR2,UNC119,SPDYE2,APP,GSKIP,PELI2,CHRM5,PIK3R5,AVPR1B,PTK2,SCP2,LRRK2,ACVR2A,RABGEF1,KIT,EPHA7,SPAG9,WNT5A,PRKDC,IPO5,BCAR3,TPD52L1,PPM1E,PKIG,EFNA5,TRPC5,FNIP2,IKBKB,FXR2,CEMIP,ATF2,ACVR1,PID1,RB1,SMG7,PLPP3,XRCC5,GFRA1,KAT2B,ARNT,BDNF,TLR6,PPARG,TENM1,SPDYE16,TFRC,SPDYE6,CLEC6A,ANGPT1,EIF4G1,ROCK1,PRKD1,STK4,PRLR,TTN,PARD3*
GO:0045937	positive regulation of phosphate metabolic process	0.02626076389141991	*SMYD3,MAP3K13,TF,DIPK2A,CDON,LRP8,PTH,FGF10,MTCP1,CHRNA7,TNIK,TGFB2,MUSK,STRADA,MAPRE3,CACUL1,PRKAG2,SPDYE2B,ERP29,LYN,NOS3,ERCC6,TGFBR2,UNC119,SPDYE2,APP,PELI2,PIK3R5,AVPR1B,PTK2,LRRK2,ACVR2A,KIT,EPHA7,WNT5A,BCAR3,TPD52L1,EFNA5,TRPC5,FNIP2,FXR2,CEMIP,ATF2,ACVR1,PID1,PLPP3,XRCC5,GFRA1,KAT2B,ARNT,BDNF,TLR6,TENM1,SPDYE16,TFRC,SPDYE6,CLEC6A,ANGPT1,EIF4G1,ROCK1,PRKD1,STK4,PRLR*
GO:0010562	positive regulation of phosphorus metabolic process	0.02626076389141991	*SMYD3,MAP3K13,TF,DIPK2A,CDON,LRP8,PTH,FGF10,MTCP1,CHRNA7,TNIK,TGFB2,MUSK,STRADA,MAPRE3,CACUL1,PRKAG2,SPDYE2B,ERP29,LYN,NOS3,ERCC6,TGFBR2,UNC119,SPDYE2,APP,PELI2,PIK3R5,AVPR1B,PTK2,LRRK2,ACVR2A,KIT,EPHA7,WNT5A,BCAR3,TPD52L1,EFNA5,TRPC5,FNIP2,FXR2,CEMIP,ATF2,ACVR1,PID1,PLPP3,XRCC5,GFRA1,KAT2B,ARNT,BDNF,TLR6,TENM1,SPDYE16,TFRC,SPDYE6,CLEC6A,ANGPT1,EIF4G1,ROCK1,PRKD1,STK4,PRLR*
GO:0051174	regulation of phosphorus metabolic process	0.026394099418004597	*RGN,SMYD3,MAP3K13,SAMSN1,TF,DIPK2A,PRKN,CDON,LRP8,PTH,ADAR,CDC37,FGF10,HERC5,NCK1,MTCP1,CHRNA7,TNIK,TGFB2,MUSK,PRKAR2A,STRADA,MAPRE3,TLR8,CACUL1,PRKAG2,CCNT2,LATS2,SPDYE2B,ERP29,UVRAG,LYN,LDB2,NOS3,ERCC6,TGFBR2,UNC119,SPDYE2,APP,GSKIP,PELI2,CHRM5,PIK3R5,AVPR1B,PTK2,SCP2,LRRK2,ACV*

			R2A,RABGEF1,KIT,EPHA7,SPAG9,WNT5A,PRKDC,IPO5,BCAR3,TPD52L1,PPM1E,PKIG,EFNA5,TRPC5,FNIP2,IKBKB,FXR2,CEMIP,ATF2,ACVR1,PID1,RB1,SMG7,PLPP3,XRCC5,GFRA1,KAT2B,ARNT,BDNF,TLR6,PPARG,TENM1,SPDYE16,TFRC,SPDYE6,CLEC6A,ANGPT1,EIF4G1,ROCK1,PRKD1,STK4,PRLR,TTN,PARD3
GO:0042325	regulation of phosphorylation	0.02647391071317086	*RGN,SMYD3,MAP3K13,SAMSN1,TF,DIPK2A,PRKN,CDON,LRP8,ADAR,CDC37,FGF10,HERC5,NCK1,MTCP1,CHRNA7,TNIK,TGFB2,MUSK,PRKAR2A,STRADA,MAPRE3,TLR8,CACUL1,PRKAG2,CCNT2,LATS2,SPDYE2B,ERP29,UVRAG,LYN,LDB2,ERCC6,TGFBR2,UNC119,SPDYE2,APP,GSKIP,PELI2,PIK3R5,PTK2,LRRK2,ACVR2A,RABGEF1,KIT,EPHA7,SPAG9,WNT5A,PRKDC,IPO5,BCAR3,TPD52L1,PPM1E,PKIG,EFNA5,TRPC5,FNIP2,IKBKB,FXR2,CEMIP,ATF2,ACVR1,PID1,RB1,PLPP3,XRCC5,GFRA1,BDNF,TLR6,PPARG,TENM1,SPDYE16,TFRC,SPDYE6,CLEC6A,ANGPT1,EIF4G1,ROCK1,PRKD1,STK4,PRLR,TTN,PARD3*
GO:0007416	synapse assembly	0.0266408911384385	*LRRN1,ARHGEF9,CHRNB2,SDK2,SHANK3,NLGN4X,MUSK,IL1RAPL2,ADGRB3,PCDHB16,APP,CLSTN2,EPHA7,WNT5A,CTNNB1,GPC4,EFNA5,LINGO2,GRID2,GABRB3,KIRREL3,GABRG2,LRTM2,BDNF,IL1RAP,EIF4G1,CDH9*
GO:0050803	regulation of synapse structure or activity	0.02970488143454014	*LRRN1,CHRNB2,CDH8,CAMK2B,SHANK3,SLC17A6,LRP8,MUSK,PAK3,IL1RAPL2,ADGRB3,APP,LRRK2,CLSTN2,TIAM1,EPHA7,WNT5A,CTNNB1,GPC4,EFNA5,NGEF,NEUROD2,LINGO2,GRID2,LRTM2,BDNF,TANC1,LRFN2,IL1RAP,EIF4G1,ROCK1*
GO:0001932	regulation of protein phosphorylation	0.03277906968242873	*RGN,SMYD3,MAP3K13,SAMSN1,DIPK2A,PRKN,CDON,LRP8,ADAR,CDC37,FGF10,HERC5,NCK1,MTCP1,CHRNA7,TNIK,TGFB2,MUSK,PRKAR2A,STRADA,MAPRE3,TLR8,CACUL1,PRKAG2,CCNT2,LATS2,SPDYE2B,ERP29,UVRAG,LYN,ERCC6,TGFBR2,UNC119,SPDYE2,APP,GSKIP,PELI2,PIK3R5,PTK2,LRRK2,ACVR2A,RABGEF1,KIT,EPHA7,SPAG9,WNT5A,PRKDC,IPO5,BCAR3,TPD52L1,PPM1E,PKIG,EFNA5,TRPC5,FNIP2,FXR2,CEMIP,ATF2,ACVR1,PID1,RB1,PLPP3,XRCC5,GFRA1,BDNF,TLR6,PPARG,TENM1,SPDYE16,TFRC,SPDYE6,CLEC6A,ANGPT1,EIF4G1,PRKD1,STK4,PRLR,TTN,PARD3*
GO:0080090	regulation of primary metabolic process	0.033225470964168176	*APBB2,RTN1,RGN,NOSTRIN,ING3,PHF20,FOXK2,SMYD3,MAP3K13,PPM1A,SAMSN1,LIMD1,TF,THRB,ATF7,CHRNB2,AGTPBP1,ATF7-NPFF,RBPMS,SPINK5,OPA3,GPBP1L1,BRD4,ZNF264,HBP1,SDR16C5,NOTCH2,HMBOX1,ZNF551,NPNT,MORC1,NR4A3,ARID1B,E2F7,FOXO1,ZNF611,SMURF2,YBX1,DIPK2A,ARB2A,PRKN,KDM7A,PTBP3,CDON,NLK,MRPL13,ZNF10,LRP8,TAGLN3,MEOX2,STAT5B,CPEB4,EIF4ENIF1,ERBIN,ZNF121,MBTPS2,PTH,ADAR,TM9SF2,CDC37,FGF10,MECOM,HERC5,ILDR2,NCK1,CD84,FAM168A,NONO,MTCP1,KDM2B,ZNF404,WRNIP1,RSF1,NCOA7,CIDEB,MCPH1,ZNF214,GEMIN5,MRTFB,CHRNA7,HMGB1,ZNF304,AFF3,HIVEP1,ATRX,TNIK,TGFB2,MDFIC,ZNF85,HOXC13,SMARCC1,MUSK,KCNIP3,SFPQ,ALKBH1,ZCCHC4,PRKAR2A,RBM33,CTIF,RBM23,ZNF154,SLC15A4,MBTPS1,IGFBP7,EPHA5,SKOR2,STRADA,HUWE1,ZNF595,ASPH,SH3BGRL,GTF2F2,SIM1,SOX30,SPOCK3,GSN,SPON1,MAPRE3,TLR8,CACUL1,NLRP7,PHC2,ZNF578,KLF12,PRKAG2,MLLT3,CCNT2,CNOT10,ZNF429,FOSL2,LATS2,SPDYE2B,ERP29,CDYL2,UVRAG,LYN,KLF7,SUMO2,MTA3,DIP2*

			B,FIGNL1,LDB2,MYRFL,FMO5,RNASEL,DBF4B,DCP2,NOS3,VSX1,ZNF280B,TNRC6C,PRAMEF27,MLLT10,ERCC6,EIF2AK4,MAGEL2,TGFBR2,TCF7L2,ZNF462,E2F6,ZFHX3,CUX1,UNC119,SPDYE2,SERBP1,WAC,DAPK1,TDP2,ZNF678,APP,GATA4,ATF7IP2,SMAD5,IGF2BP1,MAMLD1,GSKIP,PELI2,SP140L,CHRM5,FRY,ELOVL5,PIK3R5,RIPK4,AVPR1B,PTK2,USP7,SCP2,ATP1B4,ZNF41,CELF1,POGZ,LRRK2,INO80D,ZNF395,IKZF3,ARIH1,PLD1,PKHD1,TENT5C,RARB,TCF4,PAEP,COP1,CSRP3,MLIP,ACVR2A,TIPIN,NEDD4L,BRCC3,RABGEF1,KIT,EPHA7,SPAG9,WNT5A,TRABD2B,CTNNB1,ANKRD17,RUNX1T1,PRKDC,IPO5,ESR1,ZNF479,BCAR3,TPD52L1,L3MBTL4,PPM1E,ZXDC,LMX1A,ZNF215,BBX,PKIG,EFNA5,IGF2BP2,CDC14B,ZNF722,HECTD1,TRPC5,NEUROD2,FNIP2,AASS,KAT14,IKBKB,FXR2,USF3,RAB3GAP2,TASOR,CCPG1,CEMIP,ATF2,ZNF426,HDAC8,ACVR1,PID1,EBF2,ZNF423,ZCCHC17,RB1,NSMCE1,MPV17,ADIPOR1,ZNF143,ZSCAN23,PLPP3,HNF1B,ZNF615,NCOA2,ACACB,XRCC5,TBRG4,SHLD2,GFRA1,ZNF418,TAF15,ZNF860,POU2F1,ETV1,BACH2,MID2,SIK1,ZNF850,KAT2B,SUPV3L1,MEF2D,GTF2A1,CDK5RAP2,RFC3,DACH1,ATF3,IKZF1,ARNT,BDNF,TFCP2L1,BCLAF3,ZNF665,TLR6,EEFSEC,PPARG,NEK7,ZNF80,TENM1,RORB,AVIL,USP25,ZNF286A-TBC1D26,SFRP4,SPDYE16,TFRC,SPDYE6,ZFP82,ZNF292,CLEC6A,ZNF521,ZNF761,ANGPT1,EIF4G1,ZNF618,ROCK1,TAX1BP1,ZNF701,NUP98,IKZF2,PML,FOXO3B,TNFSF4,AFAP1L2,SP100,ELAVL4,ZNF286A,ZNF69,KHDRBS3,PRKD1,HSPBP1,STK4,PRLR,ZNF569,ZNF180,NMI,YLPM1,ZNF470,CTBP2,TTN,DYRK1A,KCNE2,CUL4A,ZNF888,EYA4,ZNF736,ELL2,PVT1,GATAD2B,METTL3,PARD3,ZNF33A,CP,BMAL2,ATP8B1,RNF6,SMYD1
GO:0032535	regulation of cellular component size	0.03354020053642215	*SLC12A8,MAP3K13,SPTBN1,CDC42EP3,TMOD2,SHANK3,STK39,PLXNA4,CLCN3,NCK1,PREX1,TMOD3,ADD3,GSN,PLS1,PAK3,NCKAP1,ARHGAP18,DIP2B,SLC12A1,EPHA7,WNT5A,EFNA5,TRPC5,CAPZA2,BDNF,SEMA3E,SCIN,TENM1,AVIL,SPTBN5,VAV2,CLASP2,SEMA6A,PRKD1,SEMA5A,KCNN4,RNF6,SEMA3C,VAV1,SPART*
GO:0060078	regulation of postsynaptic membrane potential	0.03419463250259722	*CHRNB2,SEZ6,SHANK3,NLGN4X,CHRFAM7A,GABRA5,CHRNA7,GRIK1,INSYN2A,APP,HCN1,LRRK2,CHRNB3,GRID2,GABRG2,KCND2,GRIA4,RGS7BP,CHRNB4,GABRB1,GRIA3,GRIA2*
GO:0034330	cell junction organization	0.03777207916275553	*APBB2,LRRN1,ARHGEF9,CACNB1,CHRNB2,SEZ6,CLDN6,CDH8,CAMK2B,SDK2,EPB41L3,CDH11,SHANK3,PLXNA4,NLGN4X,LRP8,CDH7,CHRNA7,TGFB2,MUSK,JAM3,PKP4,PAK3,IL1RAPL2,EPHA3,ADGRB3,PCDHB16,GPM6B,EPB41L5,WHRN,APP,PTK2,LRRK2,CLSTN2,PKHD1,TIAM1,EPHA7,WNT5A,CTNNB1,ERC2,LMX1A,FILIP1,GPC4,EFNA5,NGEF,NEUROD2,PTPRF,IKBKB,LINGO2,GRID2,GABRB3,KIRREL3,GABRG2,LRTM2,ARHGEF7,NPHP1,BDNF,SEMA3E,TANC1,LRFN2,IL1RAP,CLASP2,EIF4G1,ROCK1,CDH9,CTBP2,PPFIA1,UNC13C,PARD3*
GO:0050807	regulation of synapse organization	0.04423989952252186	*LRRN1,CHRNB2,CDH8,CAMK2B,SHANK3,LRP8,MUSK,PAK3,IL1RAPL2,ADGRB3,APP,LRRK2,CLSTN2,TIAM1,EPHA7,WNT5A,CTNNB1,GPC4,EFNA5,NGEF,NEUROD2,LINGO2,GRID2,LRTM2,BDNF,TANC1,LRFN2,IL1RAP,EIF4G1,ROCK1*

GO:0009893	positive regulation of metabolic process	0.04448818732639642	*APBB2,RGN,ING3,PHF20,FOXK2,SMYD3,MAP3K13,PPM1A,TF,THRB,SPTBN1,ATF7,AGTPBP1,RBPMS,GPBP1L1,BRD4,NOTCH2,HMBOX1,NPNT,NR4A3,ARID1B,E2F7,FOXO1,FER1L6,YBX1,DIPK2A,IDO1,PRKN,KDM7A,GPSM1,CDON,LRP8,CNTN1,MEOX2,STAT5B,EIF4ENIF1,MBTPS2,PTH,ADAR,CLCN3,FGF10,MECOM,NCK1,CD84,FAM168A,MTCP1,RSF1,NCOA7,MRTFB,CHRNA7,HMGB1,ZNF304,HIVEP1,ATRX,TNIK,TGFB2,MDFIC,HOXC13,SMARCC1,MUSK,SFPQ,ZCCHC4,CTIF,RBM23,ALDH1A2,PTGFR,STRADA,HUWE1,ASPH,SH3BGRL,UBR5,GTF2F2,SOX30,GSN,SPON1,MAPRE3,TLR8,CACUL1,CD226,KLF12,PRKAG2,MLLT3,CCNT2,CNOT10,CPT2,FOSL2,SPDYE2B,ERP29,UVRAG,LYN,KLF7,SUMO2,MTA3,DIP2B,LDB2,MYRFL,RNASEL,DBF4B,DCP2,NOS3,TNRC6C,MLLT10,ERCC6,EIF2AK4,TGFBR2,TCF7L2,ZNF462,ZFHX3,UNC119,SPDYE2,GSDMD,WAC,WHRN,DAPK1,APP,GATA4,SMAD5,IGF2BP1,PELI2,ELOVL5,PIK3R5,TLR1,AVPR1B,PTK2,TICAM2,SCP2,CELF1,POGZ,PIP4K2B,LRRK2,INO80D,ZNF395,IKZF3,ARIH1,PLD1,VTCN1,TENT5C,RARB,TCF4,PAEP,COP1,CSRP3,MLIP,ACVR2A,NEDD4L,BRCC3,KIT,EPHA7,WNT5A,TRABD2B,ARFGEF2,CTNNB1,PRKDC,ESR1,BCAR3,TPD52L1,ZXDC,LMX1A,PTGER4,EFNA5,IGF2BP2,CDC14B,HECTD1,TRPC5,NEUROD2,FNIP2,IKBKB,FXR2,USF3,RAB3GAP2,CCPG1,CEMIP,ATF2,ACVR1,PID1,EBF2,ZNF423,RB1,IFNLR1,PDE4B,ADIPOR1,ZNF143,PLPP3,HNF1B,ZNF615,NCOA2,BNIP3L,XRCC5,SHLD2,GFRA1,SLC24A3,TAF15,POU2F1,ETV1,MID2,ZNF850,KAT2B,SUPV3L1,MEF2D,GTF2A1,SPTLC1,CDK5RAP2,RFC3,ATF3,ARNT,BDNF,TFCP2L1,BCLAF3,TLR6,PPARG,NEK7,TENM1,RORB,SFRP4,SPDYE16,IL1RAP,TFRC,SPDYE6,ZNF292,CLEC6A,ZNF521,ANGPT1,EIF4G1,ROCK1,NUP98,PML,TNFSF4,AFAP1L2,SP100,ELAVL4,PRKD1,HSPBP1,STK4,PRLR,NMI,IL16,CTBP2,TTN,DYRK1A,KCNE2,CUL4A,EYA4,ELL2,PVT1,GATAD2B,METTL3,UAP1,ZNF33A,BMAL2,RNF6*
GO:0036211	protein modification process	0.045215422496999794	*C10ORF90,STYK1,RGN,CDK14,MAP3K3,SMYD3,ABR,MAP3K13,PPM1A,SAMSN1,USP42,MAPK10,USP32,DAPP1,CDC14C,AGTPBP1,PPM1H,CAMK2B,OTUD7A,MAP4K3,GALNT9,FPGT-TNNI3K,FOXO1,STYXL1,STK39,SMURF2,CDK18,DIPK2A,ASB4,PRKN,RMND5A,F13A1,MARCHF9,CBLL2,CDON,NLK,MAPKAPK3,LRP8,UBE2O,ADAR,CDC37,FGF10,STK32B,HERC5,UBE2E2,NCK1,KLHL42,MTCP1,NCOA7,MARCHF1,EEF2KMT,ALPK2,CHRNA7,HECW1,TNIK,TGFB2,TRIO,MUSK,PRKAR2A,PRKG2,USP10,RSKR,EPHA5,PTPN20,PPTC7,STRADA,HUWE1,ASPH,UBR5,MAPRE3,TLR8,CACUL1,PAK3,NDUFAF7,CAMK1G,PRKAG2,CCNT2,CILK1,EPHA3,LATS2,SPDYE2B,ERP29,UVRAG,LYN,USP6,SUMO2,DIP2B,RNASEL,ERCC6,EIF2AK4,MSRA,MAGEL2,TGFBR2,MARK4,UNC119,ULK3,SPDYE2,DAPK1,APP,PPIL6,GSKIP,RNF38,UBE2G1,PELI2,FRY,PIK3R5,RIPK4,PTK2,USP7,LRRK2,ARIH1,PJA2,COP1,ACVR2A,NEDD4L,BRCC3,DUSP14,PRMT8,RABGEF1,KIT,EPHA7,SPAG9,WNT5A,TRABD2B,CTNNB1,PRKDC,IPO5,PTPN14,POFUT2,BCAR3,TPD52L1,PPM1E,TPST2,HUNK,USP40,PKIG,EFNA5,CDC14B,HECTD1,VKORC1L1,TRPC5,NEUROD2,FNIP2,KAT14,UBE3B,PTPRF,IKBKB,FXR2,RAB3GAP2,CEMIP,ATF2,HDAC8,ACVR1,PID1,RB1,NSMCE1,PLPP3,S*

			PSB1,XRCC5,GFRA1,BMX,TPST1,TMTC1,NRK,MGRN1,MID2,SIK1,KAT2B,TMTC2,UBE2E3,ARNT,BDNF,TLR6,PPARG,ASB3,TGM5,NEK7,TENM1,MYLK4,USP25,SPDYE16,TFRC,SPDYE6,MAP3K1,CLEC6A,FUT10,ANGPT1,EIF4G1,B3GAT2,ROCK1,PML,RFPL4B,PRKD1,HSPBP1,USP34,STK4,TNNI3K,ST6GAL2,PRLR,NMI,TTN,DYRK1A,CUL4A,UBE3C,ATE1,PARD3,RNF6,DPY19L1
GO:0048468	cell development	0.04685719756535345	*APBB2,SMYD3,MAP3K13,LIMD1,TF,CHST11,THRB,CHRNB2,SEZ6,AGTPBP1,SH3PXD2A,SPINK5,BRD4,CAMK2B,GRIP1,DCT,NOTCH2,TMOD2,EPB41L3,MAP6,CDH11,SHANK3,ARID1B,STYXL1,PLXNA4,ARB2A,PRKN,PTBP3,CDON,SERPINI1,NCAM2,TIAM2,CFAP54,LRP8,CNTN1,STAT5B,HEATR9,ADAR,MECOM,NCK1,NREP,GABRA5,PREX1,CDIN1,COL11A1,TMOD3,ALPK2,CHRNA7,HECW1,HMGB1,RFLNA,ATRX,TNIK,TGFB2,LAMA2,TRIO,SMARCC1,MUSK,JAM3,ALKBH1,EPHA5,SKOR2,ENAH,SNX10,ALDH1A2,SOX30,CHL1,PLS1,SLIT3,PAK3,AXDND1,MLLT3,MR1,NCKAP1,FOSL2,PACRG,EPHA3,MYOM2,LYN,ADGRB3,KLF7,DIP2B,PAQR5,VSX1,ERCC6,EIF2AK4,MATN2,TGFBR2,CRTAC1,CUX1,GPM6B,ADGRV1,EPB41L5,WHRN,OCA2,TDP2,PRUNE1,APP,GATA4,SMAD5,IGF2BP1,HCN1,FRY,HOOK3,PALLD,CECR2,PTK2,CELF1,LRRK2,IKZF3,ITFG2,PKHD1,RARB,PAEP,TIAM1,CSRP3,PHACTR1,ACVR2A,NTM,NEDD4L,KIT,CYB5R4,EPHA7,SPAG9,WNT5A,CTNNB1,CTSK,PRKDC,ESR1,LMX1A,PTGER4,DOCK1,BPGM,EFNA5,NGEF,TRPC5,NEUROD2,PTPRF,IKBKB,AKAP6,ARHGEF28,GPRIN1,OPCML,ATF2,ACVR1,GRID2,RB1,KIRREL3,AGPAT5,XRCC5,GFRA1,ETV1,TEX11,LRTM2,KAT2B,ANKS1A,FLVCR1,ARHGEF7,IKZF1,ARNT,BDNF,TFCP2L1,SEMA3E,SCIN,LAG3,PPARG,TENM1,RORB,GABRB1,AVIL,SIPA1L3,TFRC,FBN1,FUT10,CLASP2,ANGPT1,SEMA6A,ITGA4,ROCK1,PML,TNFSF4,DGKG,ELAVL4,NIN,FNDC3A,PRKD1,STK4,CR2,TTN,TMEM30A,CUL4A,SEMA5A,TTPA,METTL3,PARD3,ATP8B1,RNF6,SEMA3C,VAV1,VPS13B,SPART*
		CC	
GO:0005737	cytoplasm	6.118863082424545e-13	*FSTL1,C10ORF90,APBB2,PFKP,ESYT1,RPL19,RTN1,HS3ST3A1,RGN,CDK14,MSRB3,PDE1C,MREG,NOSTRIN,ENSG00000290149,NINL,MAP3K3,WSCD1,OSBPL8,STX8,PHF20,FOXK2,TXNDC8,SMYD3,GARS1,RAPGEF6,ARHGEF9,TAFA2,ABR,MAOA,BLTP3B,TRAPPC9,MAP3K13,EIF1AX,PPM1A,ARL13B,SAMSN1,RN7SL556P,THADA,LIMD1,IMMP1L,TF,CHST11,MYBPC1,USP42,IPP,RCSD1,SPTBN1,CDC42EP3,MYO5C,MAPK10,USP32,AHCYL2,DAPP1,ATF7,CDC14C,SEZ6,KCNQ5,GNB4,AGTPBP1,FHL1,RBPMS,ATP10A,SH3PXD2A,SPINK5,MRPS24,OPA3,SKAP2,PIGL,ATP7B,PPM1H,KCNJ6,DOK4,TNRC18,CAMK2B,GRIP1,ARHGAP11A-SCG5,DDI2,PGM1,DCT,PECR,SLC38A9,SDR16C5,RGPD2,XPO4,OTUD7A,MAP4K3,GALNT9,NOTCH2,CD93,FKBP5,TMOD2,HMBOX1,NPNT,DSC1,EPB41L3,MAP6,PARVB,CDH11,SHANK3,WDR26,SPTLC3,ARID1B,FOXO1,STYXL1,STK39,SMURF2,SRL,TBC1D16,CDK18,YBX1,DIPK2A,CFAP70,DCDC1,CACNA1C,FAM20B,FAM3C,ASB4,IDO1,ARB2A,PRKN,RMND5A,PNPLA3,F13A1,TAFA1,PIK3C2B,XPO6,SLC5A7,MARCHF9,FAM83B,IFT172,GPSM1,CBLL2,SERPINI1,SLC17A6,NLK,SDF4,GNAI1,MRPL13,MAPKAPK3,PAPOLG,TIAM2,INTS6-*

			AS1,CFAP54,TRAK2,AGL,MEOX2,STAT5B,ENPEP,NUDT19,ARHGEF4,UBE2O,MUC17,DDIAS,CPEB4,EIF4ENIF1,ERBIN,MBTPS2,RGS22,CD247,CEP192,MCC,ADAR,DNAH10,PDE10A,TM9SF2,PDHX,CLCN3,CDC37,FGF10,ZFYVE16,MECOM,RSPH10B,COL4A6,NLRP8,PLG,NEURL4,HERC5,NUDT13,PGPEP1L,ILDR2,NCK1,PUDP,KLHL42,NREP,GABRA5,NEXMIF,ASTN1,SH3BGR,MROH6,PREX1,RGPD3,CDIN1,WRNIP1,COL24A1,GOLGA8R,COL11A1,SEPTIN10,CIDEB,MARCHF1,MCPH1,MYO1D,COL1A2,DTNA,TMOD3,GEMIN5,EEF2KMT,XPO7,KCNS3,VWA3B,COL12A1,ABLIM1,RBMS1,HECW1,HMGB1,DYNLT2B,CDIP1,AFF3,RFLNA,LPP,HIVEP1,TNIK,TGFB2,MDFIC,EPB41L4A,ADD3,TRIO,SMARCC1,NDST1,JAM3,KCNIP3,SFPQ,PNPLA4,ALKBH1,ARPP21,RGS3,NUP93,ZCCHC4,RERG,PRKAR2A,RBM33,CTIF,ATP11A,MRPL33,SLC15A4,GOLPH3,PRKG2,MBTPS1,NCF2,PIGV,USP10,SND1,IGFBP7,EPHA5,PTPN20,SKOR2,CBR4,ATP13A4,ENAH,SNX10,ATP2C1,TMEM230,ALDH1A2,PPTC7,STON2,PTGFR,STRADA,HUWE1,ASPH,RRAS2,SH3BGRL,ZDHHC4,UBR5,KIAA1217,SOX30,ANKS1B,GSN,PLS1,SPON1,MAPRE3,TLR8,HPS3,KIF21A,SLIT3,ARMH3,NLRP7,ANKRD11,ITPR1,PKP4,DNAAF11,PAK3,NDUFAF7,KLF12,AXDND1,IPMK,CYP2A13,CAMK1G,PRUNE2,PRKAG2,MLLT3,CCNT2,BTG4,TRAPPC10,CPQ,CNOT10,MR1,KRTAP21-2,F8,OASL,GDPD1,CPT2,VPS8,MTX3,NCKAP1,DTD1,MGAM,CILK1,PACRG,ADGRD1,EPHA3,CHCHD3,LATS2,TYW3,MYH14,TPRG1,MRPL32,ERP29,ARHGAP18,UVRAG,MYOM2,LYN,USP6,KLF7,MTA3,DIP2B,NME7,SEC24C,ATP6V1H,FIGNL1,PDZD8,RRM2,SIL1,MYRFL,ALDH1A1,NMNAT3,FMO5,RNASEL,NAV1,DBF4B,TMCC1,LRRC7,DCP2,SGMS2,TMED7-TICAM2,NOS3,EIF3L,NOMO2,TNRC6C,PRAMEF27,MLLT10,BPHL,EIF2AK4,RN7SL738P,KIAA1614,MSRA,SYNPR,HMGCLL1,MAGEL2,TGFBR2,CFAP77,MARK4,DDX60L,MDN1,SYNJ1,CHAF1B,MTRF1,ECHDC2,ZFHX3,POLR2C,CUX1,UNC119,SHISA2,RRAGD,ULK3,GKAP1,PPP2R5E,KPNB1,GSDMD,ADGRV1,RASGRF2,SERBP1,QTRT2,EPB41L5,GAD1,SCG5,NUDCD1,WHRN,OCA2,DAPK1,TDP2,ALDH9A1,ACSM2B,PRUNE1,SAR1B,TTC7B,BTG3,APP,SMAD5,IGF2BP1,PPIL6,MAMLD1,GSKIP,UBE2G1,NBPF12,PELI2,PI4KB,FRY,RHOT1,ELOVL5,USE1,HOOK3,PIK3R5,PALLD,TLR1,BMT2,RIPK4,AVPR1B,LMAN1L,QDPR,PTK2,USP7,TICAM2,SCP2,SH2B2,AVEN,GATD3,CELF1,HYCC1,DNAH12,POGZ,PIP4K2B,UQCC1,LRRK2,ZNF395,IKZF3,GET1-SH3BGR,CLSTN2,ARIH1,CFAP298,PLD1,ITFG2,PKHD1,TENT5C,RARB,UBXN7,PJA2,MGST1,RBM44,ARHGEF38,COP1,TIAM1,CSRP3,PHACTR1,MLIP,IQSEC2,ACVR2A,ASAP2,TIPIN,SNTG2,AMMECR1,NEDD4L,BRCC3,ENSG00000288683,SNX13,RABGEF1,KIT,CYB5R4,OSBPL3,SPAG9,ANTXR1,SPDL1,WNT5A,PEX26,TEX2,ARFGEF2,CTNNB1,ANKRD17,COL19A1,CTSK,PRKDC,IPO5,ESR1,PTPN14,SCFD1,ERC2,POFUT2,DMGDH,BCAR3,TPD52L1,PPM1E,VPS53,FRMD4A,DCAF8L1,TPST2,FIGN,BTBD8,PARS2,BNIPL,HUNK,FILIP1,FBXO15,PDE7B,USP40,DOCK1,GPC4,BPGM,PKIG,IGF2BP2,ACTR3B,CDC14B,SYNE3,PLCH1,VKORC1L1,NGEF,FNIP2,AASS,KAT14,CCNJ,IKBKB,FXR2,SH3BP5,AKAP6,APOH,ARHGEF28,RAB3GAP2,EML6,CEMIP,ATF2,HDAC8,

			HLA-DQA2,PID1,HYAL4,CCDC91,ZCCHC17,GLDC,RB1,MPV17,PDE4B,KIF13A,NLRP4,GABRB3,GOSR2,PET117,SHROOM4,SMG7,PLPP3,IMMP2L,LAMP2,SSPN,SPSB1,KIRREL3,GABRG2,F5,CFAP95,AK5,CPS1,NCOA2,BNIP3L,ACACB,AGPAT5,RABGAP1L,XRCC5,TBRG4,RGS17,GFRA1,SPHKAP,INPP4A,BMX,MPRIP,TAF15,TPST1,GRIA4,RAB18,POLD3,TPH2,POU2F1,TMTC1,RGS7BP,BACH2,MGRN1,MID2,SIK1,FBXL13,ERCC6L2,PIPOX,TP53BP2,KAT2B,GOLPH3L,TMTC2,WLS,ANKS1A,RYR3,PRCP,SUPV3L1,MEF2D,MYO1B,MYL4,GTF2A1,SPTLC1,AGR3,CDK5RAP2,FLVCR1,UBE2E3,FARSB,ARHGEF7,DACH1,TRDN,GASK1A,NPHP1,IKZF1,CAPZA2,SRI,ARNT,BDNF,LDLRAD4,TFCP2L1,ACBD6,BCLAF3,SCIN,MACO1,TLR6,NDST4,ADAMTS9,STIM1,HAUS6,RAB11FIP2,EEFSEC,COX7B2,PPARG,ENSG00000290217,ASB3,MTTP,CHRNB4,TGM5,NEK7,TENM1,MYOF,JAKMIP1,SH3KBP1,SMOX,AVIL,LPCAT2,GDAP1,USP25,KIF2C,MYO5A,SIPA1L3,WDFY4,SPTBN5,SFRP4,BLTP1,PNRC1,JCAD,VAV2,TFRC,VPS13A,TES,OSBPL6,GRIA3,PNPLA8,FBN1,HAPSTR1,RAB31,MAP3K1,DDX4,IQCB1,COA1,FUT10,CLASP2,EIF4G1,B3GAT2,ATP6V0A4,ACSM5,PHLPP1,RBKS,DNAJC6,ROCK1,NCAPH,M1AP,TAX1BP1,TBC1D4,APIP,MMP28,NUP98,PML,FOXO3B,MGLL,LONRF3,PLEK2,AFAP1L2,DGKG,SP100,ELAVL4,SBF2,FOXRED2,NDUFAF2,UMODL1,RFPL4B,TTC28,TSEN2,GALK2,NIN,SPG21,ANKFY1,PRSS50,ENSA,FNDC3A,AIMP1,SLC30A10,AP4S1,TPK1,PRKD1,HSPBP1,CALCRL,USP34,STK4,TNNI3K,RAB6D,SPC25,ST6GAL2,SCAMP1,RPL27A,ANPEP,PRLR,PDE11A,NMI,RAB6C,PCCA,CLVS1,IL16,DIPK1A,EDARADD,CTBP2,TTN,NCALD,HHIP,DYRK1A,PPFIA1,TMEM30A,MYRIP,PPP1R1C,UNC13C,KCNE2,RGS10,CUL4A,SERINC1,CHSY3,TXNRD3,EYA4,MAP7D3,PMEPA1,ATE1,ETNK1,TTPA,CHAC2,FMNL2,METTL3,KRT85,UAP1,PARD3,KCNN4,CP,FUNDC1,BMAL2,ATP8B1,PGPEP1,RNF6,VAV1,SMYD1,DPY19L1,GRIA2,VDAC2,VPS13B,SPART
GO:0016020	membrane	1.6042457718753464e-10	*C10ORF90,APBB2,PFKP,ESYT1,STYK1,RPL19,RTN1,HS3ST3A1,TRGC2,CDK14,MREG,NOSTRIN,WSCD1,OSBPL8,SLC12A8,LRRN1,POTED,STX8,PHF20,CPNE4,RAPGEF6,ABR,MAOA,SLC16A5,TMEM255A,MAP3K13,PPM1A,ARL13B,THADA,LIMD1,IMMP1L,TF,CHST11,SPTBN1,CDC42EP3,MAPK10,CACNB1,USP32,DAPP1,GNG12,CHRNB2,CDC14C,SEZ6,CLDN6,CDH8,KCNQ5,GNB4,FHL1,ATP10A,SPINK5,MRPS24,SKAP2,PRND,PIGL,ATP7B,KCNJ6,ZAN,TNRC18,CAMK2B,GRIP1,DCT,DLGAP1,PECR,SLC38A9,SDR16C5,ABCG8,GALNT9,NOTCH2,CD93,FKBP5,SLC44A5,SDK2,NPNT,DSC1,EPB41L3,MAP6,PARVB,CDH11,SHANK3,SPTLC3,ARID1B,SLC14A2,STK39,SMURF2,SRL,FER1L6,PLXNA4,YBX1,DIPK2A,CACNA1C,FAM20B,PRKN,NLGN4X,PNPLA3,PIK3C2B,XPO6,SLC44A3,SLC5A7,MARCHF9,FAM83B,GPSM1,CDON,SLC17A6,THSD7A,GNAI1,MRPL13,NCAM2,PAPOLG,TIAM2,CFAP54,TRAK2,CNMD,LRP8,CNTN1,ENPEP,ARHGEF4,MUC17,EIF4ENIF1,CHRFAM7A,ERBIN,MBTPS2,CD247,MCC,ADAR,DNAH10,TM9SF2,CLCN3,RHOJ,FGF10,ZFYVE16,COL4A6,CATSPERB,TSPAN18,PLG,CDH7,ILDR2,NCK1,CD84,NONO,RARRES1,EXO1,GABRA5,ASTN1,PREX1,AIG1,WRNIP1,ANO3,TYW1,GOLGA8R,KCTD3,C2ORF88*

			,SERTM2,CIDEB,MARCHF1,SLC45A1,MYO1D,SESTD1,DTNA,GEMIN5,KCNS3,ALPK2,CHRNA7,HMGB1,CDIP1,LPP,TNIK,MDFIC,LAMA2,ADD3,TRIO,NDST1,MUSK,KCNJ18,JAM3,KCNIP3,PNPLA4,RGS3,NUP93,RERG,OR5AU1,PRKAR2A,ATP11A,MRPL33,RBM23,ATP4A,SLC15A4,PCDHB8,GOLPH3,PRKG2,MBTPS1,NCF2,PIGV,SND1,EPHA5,PTPN20,ATP13A4,ENAH,SNX10,ATP2C1,TMEM230,STON2,PTGFR,IGHV1OR21-1,FAM171A1,HUWE1,ASPH,RRAS2,SH3BGRL,ZDHHC4,UBR5,TMEM132C,ANKS1B,CHL1,GSN,PLS1,TLR8,ARMH3,ARHGEF39,CD226,ITPR1,PKP4,CPM,PAK3,CYP2A13,TMEM183A,CAMK1G,PRUNE2,PRKAG2,CCNT2,HAVCR1,CNOT10,MR1,F8,TNFRSF12A,OASL,GDPD1,CPT2,TAS2R14,VPS8,MTX3,CXCR5,NCKAP1,PRPF40A,CLDN12,CNTN3,MGAM,PACRG,IL1RAPL2,ADGRD1,EPHA3,CHCHD3,MYH14,MRPL32,ERP29,ARHGAP18,UVRAG,LYN,USP6,ADGRB3,GRIK1,UBL3,DIP2B,SEC24C,ATP6V1H,NUP58,SLC12A1,PDZD8,LDB2,MYRFL,UNC93A,FMO5,PAQR5,CORIN,TMCC1,LRRC7,PCDHB16,SGMS2,TMED7-TICAM2,SLC41A2,NOS3,EIF3L,NOMO2,BPHL,KIAA1614,MSRA,SYNPR,RAB12,HMGCLL1,XKR6,MAGEL2,TGFBR2,ITGA11,CMKLR2,MDN1,SYNJ1,CUX1,SHISA2,GPM6B,RRAGD,ULK3,GPR156,CACNA2D4,LHFPL3,KPNB1,GSDMD,ADGRV1,ST7,RASGRF2,SERBP1,QTRT2,EVA1C,EPB41L5,GAD1,WHRN,OCA2,DAPK1,ACSM2B,SAR1B,TTC7B,APP,ADAM12,HCN1,CHRM5,SLC13A1,PI4KB,RHOT1,ELOVL5,USE1,HOOK3,PIK3R5,PALLD,TLR1,TAS2R30,TMCO3,RIPK4,AVPR1B,LMAN1L,FRRS1,PTK2,TICAM2,SCP2,SH2B2,ATP1B4,AVEN,CELF1,HYCC1,PIP4K2B,UQCC1,LRRK2,GET1-SH3BGR,SLC38A4,CLSTN2,PLD1,VTCN1,ITFG2,PKHD1,UBXN7,PJA2,MGST1,COP1,TIAM1,MLIP,ACVR2A,GNAO1,ASAP2,ENSG00000288635,SNTG2,NTM,NEDD4L,ENSG00000288683,PRMT8,SNX13,RABGEF1,KIT,CYB5R4,TBC1D8,EPHA7,OSBPL3,SPAG9,ANTXR1,WNT5A,TRABD2B,PEX26,TEX2,ARFGEF2,CTNNB1,ANKRD17,CTSK,PRKDC,IPO5,ESR1,TNFRSF19,SCFD1,ERC2,POFUT2,BCAR3,VPS53,TPST2,BTBD8,FILIP1,TMEM62,PTGER4,GREB1L,VWC2L,BEST3,LYPD6B,DOCK1,CCDC107,GPC4,EFNA5,CDC14B,SYNE3,PLCH1,OR1M1,VKORC1L1,NGEF,TRPC5,FNIP2,JAML,PTPRF,IKBKB,FXR2,SH3BP5,AKAP6,TRBV10-2,LYVE1,ARHGEF28,RAB3GAP2,CLCN1,CCPG1,GPRIN1,OPCML,LINGO2,CEMIP,ATF2,KCNJ12,ACVR1,CHRNB3,HLA-DQA2,HYAL4,CCDC91,GRID2,KCNJ3,GLDC,PLPPR1,MPV17,IFNLR1,PDE4B,PKD1L3,KIF13A,ADIPOR1,GABRB3,GOSR2,SHROOM4,PLPP3,UNC80,IMMP2L,LAMP2,SSPN,KIRREL3,GABRG2,F5,CFAP95,CPS1,TMEM120A,BNIP3L,ACACB,AGPAT5,RABGAP1L,XRCC5,RGS17,GFRA1,KCND2,INPP4A,BMX,SLC24A3,TPST1,GRIA4,RAB18,TMTC1,NRK,RGS7BP,MGRN1,LRTM2,GOLPH3L,TMTC2,WLS,IGKV1-6,RYR3,TMPRSS11B,PRCP,MYO1B,MGAM2,SPTLC1,CDK5RAP2,FLVCR1,FARSB,ARHGEF7,SAMD12,TRDN,GASK1A,NPHP1,SLC26A8,CAPZA2,SRI,ATP1B1,GREB1,LDLRAD4,TFCP2L1,MACO1,TLR6,NDST4,STIM1,TMEM237,TMEM161B,RAB11FIP2,COX7B2,LAG3,ENSG00000290217,ASB3,MTTP,CHRNB4,TGM5,TENM1,IL13RA2,GABRB1,MYOF,LTB4R2,JAK

			MIP1,SH3KBP1,NALCN,SMOX,LPCAT2,GDAP1,MYLK4,KIF2C,MYO5A,SIPA1L3,SLC6A13,LRFN2,SPTBN5,BLTP1,IL1RAP,JCAD,VAV2,TFRC,VPS13A,TES,OSBPL6,GRIA3,PNPLA8,RAB31,COA1,CLEC6A,FUT10,CLASP2,ANGPT1,SEMA6A,VIPR2,EIF4G1,TMPRSS15,B3GAT2,ATP6V0A4,PHLPP1,ITGA4,DNAJC6,ROCK1,NCAPH,M1AP,KCNT2,NUP98,PML,LAYN,MGLL,SCARA5,SLC5A12,PLEK2,TNFSF4,AFAP1L2,DGKG,SPATA25,PLXDC2,SBF2,CDH9,NDUFAF2,UMODL1,PCDH19,NIN,SPG21,ZNF286A,ANKFY1,FNDC3A,AIMP1,SLC30A10,AP4S1,PRKD1,CALCRL,SLC20A2,PCDH10,TMEM131,ST6GAL2,SCAMP1,PTCHD4,RPL27A,ANPEP,PRLR,NMI,CLVS1,IL16,DIPK1A,POTEH,GNA14,CR2,LPAR6,NCALD,HHIP,TMEM30A,UNC13C,KCNE2,RGS10,SERINC1,KCTD16,CHSY3,MAP7D3,SEMA5A,LAMA4,PMEPA1,ETNK1,UAP1,PARD3,KCNN4,TRDC,CP,FUNDC1,ATP8B1,RNF6,VAV1,DPY19L1,GRIA2,VDAC2,VPS13B,SPART,IGLV4-3
GO:0071944	cell periphery	8.029122470478862e-9	*C10ORF90,ESYT1,STYK1,TRGC2,CDK14,MREG,NOSTRIN,OSBPL8,LRRN1,POTED,STX8,CPNE4,RAPGEF6,SLC16A5,PPM1A,ARL13B,LIMD1,TF,SPTBN1,CDC42EP3,MAPK10,CACNB1,DAPP1,GNG12,CHRNB2,SEZ6,CLDN6,CDH8,KCNQ5,GNB4,FHL1,ATP10A,SPINK5,SKAP2,PRND,ATP7B,KCNJ6,ZAN,GRIP1,DCT,DLGAP1,SDR16C5,ABCG8,NOTCH2,CD93,SLC44A5,SDK2,NPNT,DSC1,EPB41L3,PARVB,CDH11,SHANK3,ARID1B,SLC14A2,STK39,SMURF2,ADAMTS12,PLXNA4,YBX1,CACNA1C,NLGN4X,F13A1,PIK3C2B,XPO6,SLC44A3,SLC5A7,GPSM1,CDON,SLC17A6,THSD7A,GNAI1,NCAM2,TRAK2,LRP8,CNTN1,ENPEP,ARHGEF4,MUC17,CHRFAM7A,ERBIN,CD247,MCC,TM9SF2,CLCN3,RHOJ,FGF10,COL4A6,CATSPERB,PLG,CDH7,NCK1,CD84,EXO1,GABRA5,ASTN1,PREX1,AIG1,COL24A1,ANO3,COL11A1,KCTD3,C2ORF88,SEPTIN10,MARCHF1,MYO1D,COL1A2,DTNA,KCNS3,COL12A1,ALPK2,CHRNA7,HMGB1,LPP,TNIK,TGFB2,MDFIC,LAMA2,ADD3,MUSK,KCNJ18,JAM3,KCNIP3,RGS3,RERG,OR5AU1,PRKAR2A,ATP11A,ATP4A,SLC15A4,PCDHB8,GOLPH3,PRKG2,NCF2,IGFBP7,EPHA5,PTPN20,ATP13A4,ENAH,ATP2C1,STON2,PTGFR,IGHV1OR21-1,FAM171A1,ASPH,RRAS2,SH3BGRL,ZDHHC4,ANKS1B,CHL1,SPOCK3,GSN,PLS1,SPON1,TLR8,ARHGEF39,CD226,ITPR1,PKP4,CPM,PAK3,ABI3BP,CAMK1G,CCNT2,HAVCR1,MR1,F8,TNFRSF12A,TAS2R14,CXCR5,NCKAP1,CLDN12,CNTN3,MGAM,IL1RAPL2,ADGRD1,EPHA3,ARHGAP18,LYN,USP6,ADGRB3,GRIK1,UBL3,ATP6V1H,SLC12A1,LDB2,UNC93A,PAQR5,CORIN,LRRC7,PCDHB16,SGMS2,SLC41A2,NOS3,KIAA1614,MSRA,IMPG1,XKR6,MATN2,TGFBR2,ITGA11,CMKLR2,SYNJ1,GPM6B,GPR156,CACNA2D4,LHFPL3,GSDMD,ADGRV1,RASGRF2,EPB41L5,GAD1,WHRN,DAPK1,TTC7B,APP,ADAM12,HCN1,CHRM5,SLC13A1,FRY,PIK3R5,PALLD,TLR1,TAS2R30,AVPR1B,LMAN1L,PTK2,TICAM2,SH2B2,ATP1B4,HYCC1,PIP4K2B,LRRK2,SLC38A4,CLSTN2,PLD1,VTCN1,PKHD1,PJA2,MGST1,TIAM1,MLIP,ACVR2A,GNAO1,ASAP2,VIT,SNTG2,NTM,NEDD4L,PRMT8,KIT,EPHA7,OSBPL3,ANTXR1,WNT5A,TRABD2B,CTNNB1,COL19A1,CTSK,ESR1,TNFRSF19,SCFD1,ERC2,BTBD8,FILIP1,PTGER4,VWC2L,BEST3,LYPD6B,DOCK1,GPC4,EFNA5,PLCH1,OR1M1,TRPC5,JAML,PTPRF,IKBKB,AKAP6,TRBV10-*

			2,LYVE1,APOH,ARHGEF28,RAB3GAP2,CLCN1,GPRIN1,OPCML,LINGO2,CEMIP,KCNJ12,ACVR1,CHRNB3,HLA-DQA2,COL6A5,GRID2,KCNJ3,GLDC,PLPPR1,IFNLR1,PDE4B,PKD1L3,ADIPOR1,GABRB3,SHROOM4,PLPP3,UNC80,LAMP2,SSPN,KIRREL3,GABRG2,F5,CFAP95,CPS1,TMEM120A,XRCC5,RGS17,GFRA1,KCND2,INPP4A,BMX,SLC24A3,GRIA4,RAB18,RGS7BP,MGRN1,WLS,IGKV1-6,RYR3,TMPRSS11B,PRCP,MYO1B,FLVCR1,ARHGEF7,SAMD12,TRDN,GASK1A,SLC26A8,CAPZA2,SRI,ATP1B1,SCIN,TLR6,ADAMTS9,STIM1,TMEM161B,RAB11FIP2,LAG3,ASB3,MTTP,CHRNB4,TGM5,TENM1,IL13RA2,GABRB1,MYOF,LTB4R2,SH3KBP1,NALCN,LPCAT2,SIPA1L3,SLC6A13,LRFN2,SPTBN5,BLTP1,IL1RAP,JCAD,VAV2,TFRC,TES,OSBPL6,GRIA3,FBN1,RAB31,CLEC6A,BMPER,CLASP2,ANGPT1,SEMA6A,VIPR2,ATP6V0A4,PHLPP1,ITGA4,DNAJC6,ROCK1,MMP28,KCNT2,MGLL,SCARA5,SLC5A12,PLEK2,TNFSF4,AFAP1L2,DGKG,CDH9,UMODL1,PCDH19,NIN,SLC30A10,PRKD1,CALCRL,SLC20A2,PCDH10,SCAMP1,ANPEP,PRLR,IL16,GNA14,CR2,LPAR6,CTBP2,HHIP,TMEM30A,MYRIP,UNC13C,KCNE2,RGS10,SERINC1,KCTD16,SEMA5A,LAMA4,PMEPA1,UAP1,PARD3,KCNN4,TRDC,CP,COL6A6,ATP8B1,VAV1,GRIA2,SPART,IGLV4-3
GO:0045202	synapse	2.365891402160184e-7	APBB2,RPL19,RTN1,ARHGEF9,ABR,EIF1AX,SPTBN1,CACNB1,CHRNB2,SEZ6,CDH8,KCNQ5,PPM1H,CAMK2B,GRIP1,DLGAP1,TMOD2,SDK2,CDH11,SHANK3,PLXNA4,YBX1,CACNA1C,PRKN,NLGN4X,SLC5A7,SLC17A6,TIAM2,LRP8,CNTN1,CPEB4,CHRFAM7A,ERBIN,CLCN3,PLG,GABRA5,DTNA,CHRNA7,TNIK,LAMA2,ADD3,TRIO,MUSK,ENAH,TMEM230,STON2,ANKS1B,ITPR1,PKP4,PAK3,IL1RAPL2,LYN,ADGRB3,GRIK1,ATP6V1H,ALDH1A1,PCDHB16,EIF3L,SYNPR,SYNJ1,UNC119,GAD1,WHRN,DAPK1,INSYN2A,APP,IGF2BP1,HCN1,CHRM5,PALLD,PTK2,LRRK2,CLSTN2,PLD1,PJA2,TIAM1,PHACTR1,IQSEC2,EPHA7,WNT5A,ARFGEF2,CTNNB1,ERC2,BTBD8,FILIP1,PDE7B,VWC2L,GPC4,EFNA5,FXR2,CHRNB3,GRID2,KCNJ3,PDE4B,GABRB3,SSPN,KIRREL3,GABRG2,RGS17,KCND2,INPP4A,GRIA4,RGS7BP,ARHGEF7,BDNF,MACO1,TANC1,CHRNB4,GABRB1,MYOF,SH3KBP1,SLC6A13,LRFN2,BLTP1,IL1RAP,GRIA3,EIF4G1,ATP6V0A4,DNAJC6,ROCK1,ELAVL4,CDH9,SCAMP1,RPL27A,CTBP2,PPFIA1,MYRIP,UNC13C,RGS10,KCTD16,LAMA4,GRIA2,SPART
GO:0005886	plasma membrane	2.4402222702197173e-7	C10ORF90,ESYT1,STYK1,TRGC2,CDK14,MREG,NOSTRIN,POTED,STX8,CPNE4,RAPGEF6,SLC16A5,PPM1A,ARL13B,LIMD1,TF,SPTBN1,CDC42EP3,MAPK10,CACNB1,DAPP1,GNG12,CHRNB2,SEZ6,CLDN6,CDH8,KCNQ5,GNB4,FHL1,ATP10A,SKAP2,PRND,ATP7B,KCNJ6,ZAN,GRIP1,DCT,DLGAP1,SDR16C5,ABCG8,NOTCH2,CD93,SLC44A5,SDK2,DSC1,EPB41L3,PARVB,CDH11,SHANK3,ARID1B,SLC14A2,STK39,SMURF2,PLXNA4,YBX1,CACNA1C,NLGN4X,PIK3C2B,XPO6,SLC44A3,SLC5A7,GPSM1,CDON,SLC17A6,THSD7A,GNAI1,NCAM2,TRAK2,LRP8,CNTN1,ENPEP,ARHGEF4,MUC17,CHRFAM7A,ERBIN,CD247,MCC,TM9SF2,CLCN3,RHOJ,FGF10,CATSPERB,PLG,CDH7,NCK1,CD84,EXO1,GABRA5,ASTN1,PREX1,AIG1,ANO3,KCTD3,C2ORF88,MARCHF1,MYO1D,DTNA,KCNS3,ALPK2,CHRNA7,HMGB1,LPP,TNIK,MDFIC,LAMA2,ADD3,MUSK,KCNJ18,JAM3,KCNIP3

			,*RGS3,RERG,OR5AU1,PRKAR2A,ATP11A,ATP4A,SLC15A4,PCDHB8,GOLPH3,PRKG2,NCF2,EPHA5,PTPN20,ATP13A4,ENAH,ATP2C1,STON2,PTGFR,IGHV1OR21-1,FAM171A1,ASPH,RRAS2,SH3BGRL,ZDHHC4,ANKS1B,CHL1,GSN,PLS1,TLR8,ARHGEF39,CD226,ITPR1,PKP4,CPM,PAK3,CAMK1G,CCNT2,HAVCR1,MR1,F8,TNFRSF12A,TAS2R14,CXCR5,NCKAP1,CLDN12,CNTN3,MGAM,IL1RAPL2,ADGRD1,EPHA3,ARHGAP18,LYN,USP6,ADGRB3,GRIK1,UBL3,ATP6V1H,SLC12A1,LDB2,UNC93A,PAQR5,CORIN,LRRC7,PCDHB16,SGMS2,SLC41A2,NOS3,KIAA1614,MSRA,XKR6,TGFBR2,ITGA11,CMKLR2,SYNJ1,GPM6B,GPR156,CACNA2D4,LHFPL3,GSDMD,ADGRV1,RASGRF2,EPB41L5,GAD1,WHRN,DAPK1,TTC7B,APP,ADAM12,HCN1,CHRM5,SLC13A1,PIK3R5,PALLD,TLR1,TAS2R30,AVPR1B,PTK2,TICAM2,SH2B2,ATP1B4,HYCC1,PIP4K2B,LRRK2,SLC38A4,CLSTN2,PLD1,VTCN1,PKHD1,PJA2,MGST1,TIAM1,MLIP,ACVR2A,GNAO1,ASAP2,SNTG2,NTM,NEDD4L,PRMT8,KIT,EPHA7,OSBPL3,ANTXR1,WNT5A,TRABD2B,CTNNB1,CTSK,ESR1,TNFRSF19,SCFD1,ERC2,BTBD8,FILIP1,PTGER4,VWC2L,BEST3,LYPD6B,DOCK1,GPC4,EFNA5,PLCH1,OR1M1,TRPC5,JAML,PTPRF,IKBKB,AKAP6,TRBV10-2,LYVE1,ARHGEF28,RAB3GAP2,CLCN1,GPRIN1,OPCML,CEMIP,KCNJ12,ACVR1,CHRNB3,HLA-DQA2,GRID2,KCNJ3,GLDC,PLPPR1,IFNLR1,PDE4B,PKD1L3,ADIPOR1,GABRB3,SHROOM4,PLPP3,UNC80,LAMP2,SSPN,KIRREL3,GABRG2,F5,CFAP95,CPS1,TMEM120A,XRCC5,RGS17,GFRA1,KCND2,INPP4A,BMX,SLC24A3,GRIA4,RAB18,RGS7BP,MGRN1,WLS,IGKV1-6,RYR3,TMPRSS11B,PRCP,MYO1B,FLVCR1,ARHGEF7,SAMD12,TRDN,GASK1A,SLC26A8,SRI,ATP1B1,TLR6,STIM1,TMEM161B,RAB11FIP2,LAG3,ASB3,MTTP,CHRNB4,TGM5,TENM1,IL13RA2,GABRB1,MYOF,LTB4R2,SH3KBP1,NALCN,LPCAT2,SIPA1L3,SLC6A13,LRFN2,SPTBN5,BLTP1,IL1RAP,JCAD,VAV2,TFRC,TES,OSBPL6,GRIA3,RAB31,CLEC6A,CLASP2,ANGPT1,SEMA6A,VIPR2,ATP6V0A4,PHLPP1,ITGA4,DNAJC6,ROCK1,KCNT2,MGLL,SCARA5,SLC5A12,PLEK2,TNFSF4,AFAP1L2,DGKG,CDH9,UMODL1,PCDH19,NIN,SLC30A10,PRKD1,CALCRL,SLC20A2,PCDH10,SCAMP1,ANPEP,PRLR,IL16,GNA14,CR2,LPAR6,HHIP,TMEM30A,UNC13C,KCNE2,RGS10,SERINC1,KCTD16,SEMA5A,PMEPA1,UAP1,PARD3,KCNN4,TRDC,CP,ATP8B1,VAV1,GRIA2,SPART,IGLV4-3*
GO:0043005	neuron projection	3.465855689537182e-7	*APBB2,RTN1,GARS1,ABR,SPTBN1,AHCYL2,CHRNB2,SEZ6,CDH8,KCNQ5,AGTPBP1,CAMK2B,GRIP1,EPB41L3,MAP6,SHANK3,CACNA1C,PRKN,NLGN4X,SLC5A7,SLC17A6,NCAM2,TIAM2,TRAK2,LRP8,CNTN1,CPEB4,CHRFAM7A,CLCN3,GABRA5,PREX1,MYO1D,DTNA,CHRNA7,TGFB2,LAMA2,EPHA5,STON2,ANKS1B,CHL1,PLS1,KIF21A,PACRG,EPHA3,MYH14,DIP2B,ALDH1A1,NMNAT3,NAV1,SYNPR,IMPG1,CMKLR2,MARK4,SYNJ1,ADGRV1,GAD1,WHRN,APP,IGF2BP1,HCN1,CHRM5,ELOVL5,PALLD,PTK2,HYCC1,LRRK2,CLSTN2,TIAM1,RABGEF1,EPHA7,ARFGEF2,ERC2,BTBD8,NGEF,TRPC5,PTPRF,FXR2,GPRIN1,CHRNB3,GRID2,PLPPR1,PDE4B,GABRB3,UNC80,KIRREL3,GABRG2,RGS17,GFRA1,KCND2,GRIA4,TPH2,RGS7BP,ANKS1A,ARHGEF7,NPHP1,BDNF,M*

			ACO1,TMEM237,TANC1,CHRNB4,TENM1,GABRB1,SH3KBP1,AVIL,MYO5A,SPTBN5,GRIA3,IQCB1,CLASP2,SEMA6A,ITGA4,ELAVL4,SBF2,NIN,DYRK1A,PPFIA1,MYRIP,UNC13C,PARD3,KCNN4,ATP8B1,RNF6,GRIA2
GO:0030054	cell junction	0.0000014657977175881 89	*APBB2,RPL19,RTN1,ARHGEF9,ABR,EIF1AX,LIMD1,SPTBN1,CACNB1,CHRNB2,SEZ6,CLDN6,CDH8,KCNQ5,FHL1,SH3PXD2A,PPM1H,CAMK2B,GRIP1,DLGAP1,TMOD2,SDK2,DSC1,EPB41L3,PARVB,CDH11,SHANK3,PLXNA4,YBX1,CACNA1C,PRKN,NLGN4X,SLC5A7,SLC17A6,TIAM2,LRP8,CNTN1,CPEB4,CHRFAM7A,ERBIN,CLCN3,PLG,CDH7,ILDR2,NCK1,GABRA5,DTNA,TMOD3,CHRNA7,LPP,TNIK,LAMA2,ADD3,TRIO,MUSK,JAM3,PRKAR2A,ENAH,TMEM230,STON2,RRAS2,ANKS1B,GSN,ITPR1,PKP4,PAK3,NCKAP1,CLDN12,IL1RAPL2,LYN,ADGRB3,GRIK1,ATP6V1H,ALDH1A1,PCDHB16,DCP2,EIF3L,SYNPR,ITGA11,SYNJ1,UNC119,EPB41L5,GAD1,WHRN,DAPK1,PRUNE1,INSYN2A,APP,IGF2BP1,HCN1,CHRM5,PALLD,PTK2,LRRK2,CLSTN2,PLD1,PJA2,TIAM1,PHACTR1,IQSEC2,KIT,EPHA7,WNT5A,ARFGEF2,CTNNB1,ERC2,BCAR3,FRMD4A,BTBD8,FILIP1,PDE7B,VWC2L,GPC4,EFNA5,JAML,FXR2,AKAP6,CHRNB3,GRID2,KCNJ3,PDE4B,GABRB3,SHROOM4,PLPP3,SSPN,KIRREL3,GABRG2,RGS17,KCND2,INPP4A,MPRIP,GRIA4,RGS7BP,TP53BP2,CDK5RAP2,ARHGEF7,NPHP1,ATP1B1,BDNF,SCIN,MACO1,TANC1,CHRNB4,GABRB1,MYOF,SH3KBP1,AVIL,SIPA1L3,SLC6A13,LRFN2,SPTBN5,BLTP1,IL1RAP,JCAD,TES,GRIA3,CLASP2,EIF4G1,ATP6V0A4,ITGA4,DNAJC6,ROCK1,LAYN,ELAVL4,CDH9,PRKD1,SCAMP1,RPL27A,IL16,CTBP2,PPFIA1,MYRIP,UNC13C,RGS10,KCTD16,LAMA4,PARD3,VAV1,GRIA2,SPART*
GO:0005829	cytosol	0.0000046431773746814 95	*C10ORF90,PFKP,RPL19,CDK14,MSRB3,PDE1C,NINL,MAP3K3,OSBPL8,STX8,PHF20,SMYD3,GARS1,RAPGEF6,ARHGEF9,ABR,MAOA,BLTP3B,TRAPPC9,EIF1AX,PPM1A,ARL13B,SAMSN1,LIMD1,MYBPC1,USP42,RCSD1,SPTBN1,CDC42EP3,MAPK10,USP32,AHCYL2,DAPP1,GNB4,AGTPBP1,FHL1,RBPMS,SH3PXD2A,SPINK5,SKAP2,DOK4,TNRC18,CAMK2B,GRIP1,DDI2,PGM1,DCT,PECR,XPO4,OTUD7A,FKBP5,HMBOX1,EPB41L3,PARVB,WDR26,ARID1B,FOXO1,STK39,SMURF2,TBC1D16,YBX1,ASB4,IDO1,PRKN,PIK3C2B,XPO6,GPSM1,NLK,MAPKAPK3,PAPOLG,TIAM2,TRAK2,AGL,STAT5B,NUDT19,ARHGEF4,UBE2O,EIF4ENIF1,RGS22,CEP192,MCC,ADAR,PDE10A,CDC37,ZFYVE16,MECOM,HERC5,PGPEP1L,NCK1,PUDP,KLHL42,GABRA5,NEXMIF,PREX1,CIDEB,MYO1D,GEMIN5,EEF2KMT,KCNS3,VWA3B,RBMS1,HECW1,AFF3,LPP,HIVEP1,TNIK,ADD3,TRIO,KCNIP3,SFPQ,PNPLA4,RGS3,NUP93,RERG,PRKAR2A,CTIF,GOLPH3,PRKG2,NCF2,USP10,SND1,ENAH,ALDH1A2,STON2,STRADA,HUWE1,SH3BGRL,UBR5,SOX30,ANKS1B,GSN,PLS1,HPS3,KIF21A,ARMH3,ANKRD11,DNAAF11,PAK3,KLF12,PRUNE2,PRKAG2,MLLT3,CCNT2,TRAPPC10,CNOT10,KRTAP21-2,OASL,NCKAP1,CILK1,PACRG,ADGRD1,EPHA3,LATS2,MYH14,ARHGAP18,LYN,KLF7,NME7,SEC24C,ATP6V1H,RRM2,ALDH1A1,FMO5,RNASEL,TMCC1,LRRC7,DCP2,NOS3,EIF3L,NOMO2,TNRC6C,MLLT10,EIF2AK4,MSRA,HMGCLL1,MAGEL2,TGFBR2,MARK4,MDN1,SYNJ1,CHAF1B,POLR2C,CUX1,UNC119,RRAGD,ULK3,PPP2R5E,KPNB1,GSDMD,RASGRF2,S*

			ERBP1,EPB41L5,NUDCD1,ALDH9A1,PRUNE1,SAR1B,TTC7B,APP,SMAD5,IGF2BP1,UBE2G1,PELI2,PI4KB,HOOK3,PIK3R5,PALLD,BMT2,QDPR,PTK2,USP7,SCP2,SH2B2,AVEN,HYCC1,POGZ,PIP4K2B,LRRK2,ZNF395,IKZF3,ARIH1,CFAP298,ITFG2,UBXN7,COP1,TIAM1,CSRP3,PHACTR1,MLIP,ASAP2,NEDD4L,BRCC3,RABGEF1,OSBPL3,SPAG9,SPDL1,PEX26,ARFGEF2,CTNNB1,PRKDC,IPO5,ESR1,SCFD1,VPS53,BNIPL,FILIP1,FBXO15,PDE7B,USP40,DOCK1,BPGM,IGF2BP2,PLCH1,NGEF,FNIP2,AASS,IKBKB,FXR2,ARHGEF28,RAB3GAP2,CCDC91,ZCCHC17,RB1,MPV17,PDE4B,NLRP4,GOSR2,SMG7,SPSB1,AK5,ACACB,XRCC5,INPP4A,BMX,MPRIP,RAB18,TPH2,BACH2,MGRN1,FBXL13,ERCC6L2,PIPOX,TP53BP2,KAT2B,GOLPH3L,WLS,ANKS1A,MYL4,GTF2A1,CDK5RAP2,UBE2E3,FARSB,ARHGEF7,DACH1,TRDN,NPHP1,IKZF1,CAPZA2,SRI,ACBD6,HAUS6,PPARG,ASB3,MTTP,SH3KBP1,SMOX,GDAP1,USP25,KIF2C,MYO5A,SPTBN5,VAV2,VPS13A,TES,OSBPL6,RAB31,MAP3K1,IQCB1,COA1,CLASP2,EIF4G1,PHLPP1,RBKS,DNAJC6,ROCK1,NCAPH,TAX1BP1,TBC1D4,APIP,NUP98,PML,FOXO3B,MGLL,AFAP1L2,DGKG,SBF2,RFPL4B,TSEN2,GALK2,SPG21,ANKFY1,FNDC3A,AIMP1,TPK1,PRKD1,USP34,STK4,RAB6D,SPC25,RPL27A,PDE11A,NMI,RAB6C,PCCA,IL16,EDARADD,CTBP2,TTN,NCALD,PPFIA1,RGS10,TXNRD3,ETNK1,TTPA,CHAC2,FMNL2,METTL3,KRT85,UAP1,PARD3,KCNN4,ATP8B1,PGPEP1,VAV1,SPART
GO:0042995	cell projection	0.000007964326012378543	APBB2,RTN1,GARS1,ABR,ARL13B,SAMSN1,SPTBN1,AHCYL2,CHRNB2,SEZ6,CDH8,KCNQ5,AGTPBP1,SH3PXD2A,CAMK2B,GRIP1,NOTCH2,EPB41L3,MAP6,PARVB,SHANK3,CFAP70,CACNA1C,PRKN,NLGN4X,SLC5A7,IFT172,SLC17A6,THSD7A,NCAM2,TIAM2,CFAP54,TRAK2,LRP8,CNTN1,ARHGEF4,CPEB4,CHRFAM7A,MCC,DNAH10,CLCN3,RSPH10B,CATSPERB,GABRA5,PREX1,SEPTIN10,MYO1D,DTNA,ABLIM1,CHRNA7,DYNLT2B,TGFB2,LAMA2,TRIO,JAM3,PRKAR2A,EPHA5,ENAH,STON2,ANKS1B,CHL1,GSN,PLS1,KIF21A,DNAAF11,HAVCR1,NCKAP1,CILK1,PACRG,EPHA3,MYH14,ARHGAP18,DIP2B,NME7,ALDH1A1,NMNAT3,NAV1,SYNPR,IMPG1,CMKLR2,CFAP77,MARK4,SYNJ1,ULK3,ADGRV1,EPB41L5,GAD1,WHRN,APP,IGF2BP1,RNF38,HCN1,CHRM5,ELOVL5,PALLD,PTK2,TICAM2,SH2B2,HYCC1,DNAH12,LRRK2,SLC38A4,CLSTN2,CFAP298,PKHD1,PJA2,TIAM1,RABGEF1,EPHA7,OSBPL3,ANTXR1,ARFGEF2,CTNNB1,ERC2,BTBD8,ACTR3B,NGEF,TRPC5,PTPRF,FXR2,GPRIN1,CHRNB3,GRID2,PLPPR1,PDE4B,GABRB3,UNC80,KIRREL3,GABRG2,CFAP95,RABGAP1L,RGS17,GFRA1,KCND2,BMX,GRIA4,TPH2,RGS7BP,FBXL13,ANKS1A,MYO1B,ARHGEF7,NPHP1,SLC26A8,ATP1B1,BDNF,SCIN,MACO1,TMEM237,RAB11FIP2,TANC1,MTTP,CHRNB4,TENM1,GABRB1,SH3KBP1,AVIL,MYO5A,SPTBN5,JCAD,VPS13A,GRIA3,IQCB1,CLASP2,ANGPT1,SEMA6A,ATP6V0A4,ITGA4,ROCK1,LAYN,PLEK2,ELAVL4,SBF2,NIN,SLC20A2,HHIP,DYRK1A,PPFIA1,MYRIP,UNC13C,KCTD16,PARD3,KCNN4,ATP8B1,RNF6,GRIA2,VDAC2
GO:0120025	plasma membrane bounded	0.000012303594055068176	APBB2,RTN1,GARS1,ABR,ARL13B,SAMSN1,SPTBN1,AHCYL2,CHRNB2,SEZ6,CDH8,KCNQ5,AGTPBP1,CAMK2B,GRIP1,NOTCH2,EPB41L3,MAP6,PARVB,SHANK3,CFAP70,CACNA1C,PRKN,NLGN4X,SLC5A7,

	cell projection		*IFT172,SLC17A6,NCAM2,TIAM2,CFAP54,TRAK2,LRP8,CNTN1,ARHGEF4,CPEB4,CHRFAM7A,MCC,DNAH10,CLCN3,RSPH10B,CATSPERB,GABRA5,PREX1,SEPTIN10,MYO1D,DTNA,ABLIM1,CHRNA7,DYNLT2B,TGFB2,LAMA2,JAM3,PRKAR2A,EPHA5,ENAH,STON2,ANKS1B,CHL1,GSN,PLS1,KIF21A,DNAAF11,HAVCR1,NCKAP1,CILK1,PACRG,EPHA3,MYH14,ARHGAP18,DIP2B,NME7,ALDH1A1,NMNAT3,NAV1,SYNPR,IMPG1,CMKLR2,CFAP77,MARK4,SYNJ1,ULK3,ADGRV1,EPB41L5,GAD1,WHRN,APP,IGF2BP1,RNF38,HCN1,CHRM5,ELOVL5,PALLD,PTK2,SH2B2,HYCC1,DNAH12,LRRK2,SLC38A4,CLSTN2,CFAP298,PKHD1,PJA2,TIAM1,RABGEF1,EPHA7,OSBPL3,ANTXR1,ARFGEF2,CTNNB1,ERC2,BTBD8,NGEF,TRPC5,PTPRF,FXR2,GPRIN1,CHRNB3,GRID2,PLPPR1,PDE4B,GABRB3,UNC80,KIRREL3,GABRG2,CFAP95,RABGAP1L,RGS17,GFRA1,KCND2,BMX,GRIA4,TPH2,RGS7BP,FBXL13,ANKS1A,MYO1B,ARHGEF7,NPHP1,SLC26A8,ATP1B1,BDNF,MACO1,TMEM237,TANC1,MTTP,CHRNB4,TENM1,GABRB1,SH3KBP1,AVIL,MYO5A,SPTBN5,JCAD,VPS13A,GRIA3,IQCB1,CLASP2,ANGPT1,SEMA6A,ATP6V0A4,ITGA4,ROCK1,LAYN,PLEK2,ELAVL4,SBF2,NIN,SLC20A2,HHIP,DYRK1A,PPFIA1,MYRIP,UNC13C,PARD3,KCNN4,ATP8B1,RNF6,GRIA2,VDAC2*
GO:1990351	transporter complex	0.0000240335611392692	*CACNB1,CHRNB2,KCNQ5,ATP10A,KCNJ6,ABCG8,CACNA1C,SLC17A6,CHRFAM7A,CATSPERB,GABRA5,SESTD1,KCNS3,CHRNA7,KCNJ18,KCNIP3,ATP11A,ATP4A,ASPH,GRIK1,ATP6V1H,CACNA2D4,HCN1,ATP1B4,VWC2L,BEST3,TRPC5,AKAP6,CLCN1,KCNJ12,CHRNB3,GRID2,KCNJ3,PDE4B,PKD1L3,GABRB3,UNC80,GABRG2,KCND2,GRIA4,RYR3,ATP1B1,COX7B2,CHRNB4,GABRB1,NALCN,GRIA3,ATP6V0A4,TMEM30A,KCNE2,KCNN4,ATP8B1,GRIA2*
GO:0098794	postsynapse	0.00002409871145250991	*RTN1,ARHGEF9,ABR,SPTBN1,CHRNB2,SEZ6,GRIP1,DLGAP1,SHANK3,CACNA1C,PRKN,NLGN4X,CNTN1,CPEB4,CHRFAM7A,ERBIN,GABRA5,CHRNA7,TNIK,LAMA2,ADD3,TRIO,MUSK,ANKS1B,ITPR1,PKP4,PAK3,LYN,ADGRB3,GRIK1,DAPK1,INSYN2A,APP,IGF2BP1,HCN1,CHRM5,PTK2,LRRK2,CLSTN2,PJA2,TIAM1,WNT5A,ARFGEF2,CTNNB1,FXR2,CHRNB3,GRID2,PDE4B,GABRB3,SSPN,GABRG2,KCND2,INPP4A,GRIA4,RGS7BP,ARHGEF7,TANC1,CHRNB4,GABRB1,LRFN2,GRIA3,EIF4G1,DNAJC6,ELAVL4,CDH9,PPFIA1,KCTD16,GRIA2*
GO:0012505	endomembrane system	0.0000455574946473972 4	*FSTL1,APBB2,ESYT1,RTN1,HS3ST3A1,MSRB3,MREG,WSCD1,OSBPL8,STX8,PHF20,TXNDC8,GARS1,BLTP3B,TRAPPC9,THADA,TF,CHST11,RCSD1,CDC42EP3,USP32,AHCYL2,CDC14C,SEZ6,ATP10A,SPINK5,PIGL,ATP7B,KCNJ6,TNRC18,CAMK2B,GRIP1,ARHGAP11A-SCG5,PGM1,SLC38A9,SDR16C5,RGPD2,XPO4,GALNT9,NOTCH2,CD93,DSC1,MAP6,SPTLC3,SRL,TBC1D16,YBX1,DIPK2A,FAM20B,FAM3C,ARB2A,PRKN,PNPLA3,F13A1,TAFA1,PIK3C2B,SLC5A7,MARCHF9,GPSM1,SERPINI1,SLC17A6,SDF4,TRAK2,CNMD,AGL,MUC17,CPEB4,ERBIN,MBTPS2,CD247,TM9SF2,CLCN3,ZFYVE16,COL4A6,PLG,ILDR2,NCK1,ASTN1,RGPD3,AIG1,COL24A1,GOLGA8R,COL11A1,CIDEB,MARCHF1,MYO1D,COL1A2,XPO7,KCNS3,COL12A1,HMGB1,CDIP1,TNIK,TGFB2,NDST1,JAM3,KCNIP3,ALKBH1,NUP93,ATP11A,SLC15A4,GOLPH3,PRKG2,MBTPS1,NCF2,PIGV,USP10,IGFBP7,EPHA5,ATP13A4,SNX10,ATP2C1,TMEM230,STON2,*

			HUWE1,ASPH,RRAS2,ZDHHC4,GSN,SPON1,TLR8,HPS3,ARMH3,ITPR1,CYP2A13,CAMK1G,TRAPPC10,CPQ,MR1,F8,GDPD1,VPS8,MGAM,EPHA3,ERP29,UVRAG,LYN,USP6,SEC24C,ATP6V1H,NUP58,PDZD8,SIL1,MYRFL,FMO5,TMCC1,SGMS2,TMED7-TICAM2,NOS3,NOMO2,SYNPR,HMGCLL1,MAGEL2,SYNJ1,CUX1,SHISA2,GKAP1,KPNB1,GSDMD,RASGRF2,SCG5,OCA2,SAR1B,APP,MAMLD1,PI4KB,ELOVL5,USE1,HOOK3,TLR1,AVPR1B,LMAN1L,TICAM2,SCP2,ATP1B4,AVEN,PIP4K2B,LRRK2,GET1-SH3BGR,CLSTN2,PLD1,ITFG2,PKHD1,UBXN7,PJA2,MGST1,MLIP,ASAP2,NEDD4L,SNX13,RABGEF1,KIT,CYB5R4,OSBPL3,SPAG9,ANTXR1,WNT5A,TEX2,ARFGEF2,ANKRD17,COL19A1,CTSK,IPO5,ESR1,SCFD1,POFUT2,VPS53,TPST2,BTBD8,GPC4,SYNE3,VKORC1L1,AKAP6,APOH,RAB3GAP2,CEMIP,HLA-DQA2,CCDC91,PDE4B,KIF13A,GOSR2,PLPP3,LAMP2,SSPN,KIRREL3,F5,TMEM120A,BNIP3L,AGPAT5,RABGAP1L,XRCC5,GFRA1,INPP4A,TPST1,RAB18,POU2F1,TMTC1,MGRN1,GOLPH3L,TMTC2,WLS,RYR3,PRCP,MYO1B,SPTLC1,AGR3,CDK5RAP2,DACH1,TRDN,GASK1A,SRI,BDNF,LDLRAD4,MACO1,TLR6,NDST4,ADAMTS9,STIM1,RAB11FIP2,ENSG00000290217,MTTP,CHRNB4,TENM1,GABRB1,MYOF,SMOX,LPCAT2,USP25,MYO5A,SIPA1L3,WDFY4,BLTP1,TFRC,VPS13A,OSBPL6,PNPLA8,FBN1,RAB31,FUT10,CLASP2,B3GAT2,ATP6V0A4,ROCK1,NUP98,PML,MGLL,ELAVL4,SBF2,FOXRED2,SPG21,ANKFY1,PRSS50,FNDC3A,AIMP1,SLC30A10,AP4S1,PRKD1,HSPBP1,CALCRL,RAB6D,ST6GAL2,SCAMP1,RPL27A,ANPEP,PRLR,RAB6C,CLVS1,DIPK1A,NCALD,PPFIA1,TMEM30A,MYRIP,UNC13C,SERINC1,CHSY3,TXNRD3,PMEPA1,TTPA,METTL3,PARD3,CP,ATP8B1,RNF6,DPY19L1,GRIA2,VDAC2,VPS13B
GO:1902495	transmembrane transporter complex	0.00012692264788380814	*CACNB1,CHRNB2,KCNQ5,KCNJ6,ABCG8,CACNA1C,SLC17A6,CHRFAM7A,CATSPERB,GABRA5,SESTD1,KCNS3,CHRNA7,KCNJ18,KCNIP3,ATP4A,ASPH,GRIK1,ATP6V1H,CACNA2D4,HCN1,ATP1B4,VWC2L,BEST3,TRPC5,AKAP6,CLCN1,KCNJ12,CHRNB3,GRID2,KCNJ3,PDE4B,PKD1L3,GABRB3,UNC80,GABRG2,KCND2,GRIA4,RYR3,ATP1B1,COX7B2,CHRNB4,GABRB1,NALCN,GRIA3,ATP6V0A4,KCNE2,KCNN4,GRIA2*
GO:0005783	endoplasmic reticulum	0.00023684346307452494	*FSTL1,APBB2,ESYT1,RTN1,MSRB3,OSBPL8,STX8,TRAPPC9,THADA,TF,AHCYL2,CDC14C,SEZ6,ATP10A,SPINK5,PIGL,CAMK2B,GRIP1,SDR16C5,NOTCH2,SPTLC3,SRL,YBX1,ARB2A,PRKN,PNPLA3,TAFA1,PIK3C2B,GPSM1,AGL,CPEB4,COL4A6,ILDR2,NCK1,COL24A1,COL11A1,CIDEB,MARCHF1,MYO1D,COL1A2,COL12A1,HMGB1,KCNIP3,ALKBH1,ATP11A,MBTPS1,PIGV,IGFBP7,EPHA5,ATP13A4,SNX10,ATP2C1,TMEM230,ASPH,RRAS2,ZDHHC4,SPON1,TLR8,ITPR1,CYP2A13,CPQ,MR1,F8,GDPD1,ERP29,UVRAG,SEC24C,PDZD8,SIL1,MYRFL,FMO5,TMCC1,TMED7-TICAM2,NOMO2,HMGCLL1,SHISA2,KPNB1,RASGRF2,OCA2,SAR1B,APP,PI4KB,ELOVL5,USE1,LMAN1L,TICAM2,SCP2,PIP4K2B,LRRK2,GET1-SH3BGR,CLSTN2,PLD1,PKHD1,UBXN7,PJA2,MGST1,CYB5R4,OSBPL3,WNT5A,TEX2,COL19A1,SCFD1,POFUT2,TPST2,SYNE3,VKORC1L1,AKAP6,RAB3GAP2,CEMIP,HLA-DQA2,GOSR2,PLPP3,F5,BNIP3L,AGPAT5,RAB18,*

			POU2F1,TMTC1,MGRN1,TMTC2,WLS,RYR3,SPTLC1,AGR3,TRDN,GASK1A,SRI,BDNF,MACO1,ADAMTS9,STIM1,MTTP,TENM1,LPCAT2,USP25,MYO5A,WDFY4,BLTP1,VPS13A,OSBPL6,PNPLA8,FBN1,FUT10,PML,MGLL,FOXRED2,PRSS50,AIMP1,HSPBP1,CALCRL,RPL27A,DIPK1A,TMEM30A,SERINC1,TXNRD3,CP,ATP8B1,DPY19L1,GRIA2
GO:0110165	cellular anatomical structure	0.0002389781440947051	*FSTL1,C10ORF90,APBB2,PFKP,ESYT1,STYK1,RPL19,RTN1,HS3ST3A1,RGN,TRGC2,CDK14,ZSWIM1,MSRB3,PDE1C,MREG,HNRNPCL4,NOSTRIN,ING3,ENSG00000290149,NINL,MAP3K3,WSCD1,OSBPL8,SLC12A8,LRRN1,POTED,STX8,PHF20,FOXK2,CPNE4,TXNDC8,RBM17,SMYD3,GARS1,RAPGEF6,ARHGEF9,TAFA2,ABR,ISM1,MAOA,BLTP3B,TRAPPC9,SLC16A5,FNDC1,TMEM255A,MAP3K13,EIF1AX,PPM1A,ARL13B,SAMSN1,RN7SL556P,THADA,LIMD1,IMMP1L,TF,CHST11,THRB,MYBPC1,USP42,IPP,RCSD1,SPTBN1,CDC42EP3,MYO5C,MAPK10,CACNB1,USP32,AHCYL2,DAPP1,ATF7,GNG12,CHRNB2,CDC14C,SEZ6,CLDN6,CDH8,KCNQ5,GNB4,AGTPBP1,ATF7-NPFF,FHL1,TRMT6,RBPMS,ATP10A,SH3PXD2A,SPINK5,MRPS24,OPA3,SKAP2,PRND,PIGL,ATP7B,PPM1H,GPBP1L1,KCNJ6,BRD4,ZAN,DOK4,TNRC18,CAMK2B,GRIP1,ARHGAP11A-SCG5,DDI2,GPATCH8,PGM1,DCT,DLGAP1,PECR,SLC38A9,ZNF264,HBP1,SDR16C5,RGPD2,ABCG8,XPO4,OTUD7A,MAP4K3,GALNT9,SNORD114-10,NOTCH2,CD93,FKBP5,TMOD2,SLC44A5,SDK2,HMBOX1,ZNF551,RNU2-49P,NPNT,DSC1,EPB41L3,MAP6,MORC1,PARVB,CDH11,SHANK3,WDR26,NR4A3,SPTLC3,ARID1B,E2F7,SLC14A2,FOXO1,ZNF611,STYXL1,STK39,SMURF2,SRL,ADAMTS12,FER1L6,TBC1D16,PLXNA4,CDK18,URB2,YBX1,DIPK2A,CFAP70,DCDC1,CACNA1C,FAM20B,FAM3C,SNORA5B,ASB4,IDO1,ARB2A,PRKN,NLGN4X,RMND5A,PNPLA3,F13A1,TAFA1,PIK3C2B,XPO6,KDM7A,SLC44A3,SLC5A7,MARCHF9,FAM83B,IFT172,GPSM1,CBLL2,PTBP3,CDON,SERPINI1,SLC17A6,NLK,SDF4,THSD7A,GNAI1,MRPL13,MAPKAPK3,NCAM2,PAPOLG,TIAM2,INTS6-AS1,CFAP54,ZNF10,TRAK2,CNMD,LRP8,TAGLN3,CNTN1,AGL,MEOX2,STAT5B,ENPEP,NUDT19,ARHGEF4,UBE2O,MUC17,DDIAS,CPEB4,EIF4ENIF1,CHRFAM7A,ERBIN,ZNF121,MBTPS2,RGS22,CD247,CEP192,MCC,PTH,ADAR,DNAH10,PDE10A,TM9SF2,PDHX,CLCN3,RHOJ,CDC37,FGF10,ZFYVE16,MECOM,RSPH10B,COL4A6,CATSPERB,TSPAN18,NLRP8,PLG,NEURL4,HERC5,NUDT13,CDH7,PGPEP1L,UBE2E2,ILDR2,NCK1,PUDP,CD84,NONO,RARRES1,KLHL42,NREP,EXO1,GABRA5,EBNA1BP2,SNORD114-9,NEXMIF,ASTN1,SH3BGR,MROH6,KDM2B,PREX1,RGPD3,AIG1,ZNF404,PMS1,CDIN1,WRNIP1,COL24A1,ANO3,TYW1,GOLGA8R,COL11A1,RSF1,KCTD3,NCOA7,C2ORF88,RNU2-33P,SERTM2,SEPTIN10,CIDEB,MARCHF1,SLC45A1,MCPH1,MYO1D,SESTD1,ZNF214,COL1A2,DTNA,TMOD3,GEMIN5,EEF2KMT,MRTFB,XPO7,KCNS3,VWA3B,COL12A1,ABLIM1,RBMS1,ALPK2,CHRNA7,HECW1,HMGB1,ZNF304,DYNLT2B,CDIP1,AFF3,RFLNA,LPP,HIVEP1,ATRX,TNIK,TGFB2,MDFIC,EPB41L4A,LAMA2,ADD3,TRIO,ZNF85,HOXC13,SMARCC1,NDST1,MUSK,KCNJ18,JAM3,KCNIP3,SFPQ,PNPLA4,ALKBH1,SNORA5A,ARPP21,RGS3,NUP93,ZCCH*

			C4,RERG,OR5AU1,PRKAR2A,RBM33,CTIF,ATP11A,MRPL33,RBM23,ZNF154,ATP4A,CSTF3,SLC15A4,PCDHB8,GOLPH3,PRKG2,MBTPS1,NCF2,PIGV,RNU6-1216P,USP10,SND1,IGFBP7,EPHA5,PTPN20,SKOR2,CBR4,ATP13A4,ENAH,SNX10,ATP2C1,TMEM230,ALDH1A2,PPTC7,STON2,PTGFR,IGHV1OR21-1,FAM171A1,STRADA,HUWE1,ZNF595,ASPH,RRAS2,SH3BGRL,ZDHHC4,UBR5,GTF2F2,SIM1,KIAA1217,SOX30,TMEM132C,ANKS1B,CHL1,SPOCK3,GSN,PLS1,TCP11L1,SPON1,MAPRE3,TLR8,HPS3,KIF21A,SLIT3,ARMH3,NLRP7,ARHGEF39,CD226,PHC2,ZNF578,ANKRD11,ITPR1,PKP4,CPM,DNAAF11,PAK3,NDUFAF7,ABI3BP,RNU1-83P,KLF12,LOC124900504,AXDND1,IPMK,CYP2A13,TMEM183A,CAMK1G,PRUNE2,PRKAG2,MLLT3,CCNT2,BTG4,HAVCR1,TRAPPC10,CPQ,CNOT10,MR1,KRTAP21-2,F8,TNFRSF12A,OASL,GDPD1,CPT2,TAS2R14,VPS8,MTX3,ISY1,ZNF429,CXCR5,NCKAP1,PRPF40A,CLDN12,DTD1,CNTN3,MGAM,CILK1,FOSL2,PACRG,IL1RAPL2,ADGRD1,HNRNPCL3,EPHA3,CHCHD3,LATS2,TYW3,MYH14,TPRG1,MRPL32,ERP29,CDYL2,ARHGAP18,UVRAG,MYOM2,LYN,USP6,ADGRB3,KLF7,SUMO2,GRIK1,MTA3,UBL3,DIP2B,NME7,SEC24C,ATP6V1H,NUP58,FIGNL1,SLC12A1,PDZD8,PAMR1,RRM2,SIL1,LDB2,MYRFL,UNC93A,C21ORF62,ALDH1A1,NMNAT3,FMO5,RNASEL,NAV1,PAQR5,DBF4B,CORIN,TMCC1,LRRC7,PCDHB16,DCP2,SGMS2,TMED7-TICAM2,SLC41A2,NOS3,VSX1,ZNF280B,EIF3L,NOMO2,TNRC6C,PRAMEF27,MLLT10,BPHL,ERCC6,EIF2AK4,RN7SL738P,KIAA1614,MSRA,SYNPR,RAB12,IMPG1,HMGCLL1,XKR6,MAGEL2,MATN2,TGFBR2,ITGA11,CMKLR2,CFAP77,MARK4,DDX60L,MDN1,SYNJ1,TCF7L2,CHAF1B,ZNF462,MTRF1,ECHDC2,OVCH1,CRTAC1,E2F6,ZFHX3,POLR2C,CUX1,UNC119,SHISA2,GPM6B,RRAGD,ULK3,GPR156,ZMAT4,GKAP1,CACNA2D4,LHFPL3,PPP2R5E,KPNB1,GSDMD,ADGRV1,ST7,RASGRF2,SERBP1,QTRT2,EVA1C,EPB41L5,GAD1,SCG5,NUDCD1,WAC,WHRN,OCA2,DAPK1,TDP2,ALDH9A1,ZNF678,ACSM2B,PRUNE1,SAR1B,TTC7B,INSYN2A,BTG3,APP,GATA4,ATF7IP2,SMAD5,IGF2BP1,PPIL6,MAMLD1,RNLS,GSKIP,RNF38,ADAM12,UBE2G1,NBPF12,PELI2,DHX35,SP140L,HCN1,CHRM5,SLC13A1,PI4KB,FRY,RHOT1,ELOVL5,USE1,HOOK3,PIK3R5,PALLD,LYZL1,TLR1,CECR2,TAS2R30,TMCO3,BMT2,RIPK4,AVPR1B,LMAN1L,QDPR,FRRS1,PTK2,NOP9,USP7,TICAM2,SCP2,SH2B2,ATP1B4,AVEN,ZNF41,GATD3,CELF1,HYCC1,DNAH12,POGZ,PIP4K2B,UQCC1,LRRK2,INO80D,ZNF395,IKZF3,GET1-SH3BGR,SLC38A4,CLSTN2,ARIH1,CFAP298,PLD1,VTCN1,ITFG2,PKHD1,MEG8,TENT5C,RARB,UBXN7,PJA2,SNORD114-7,TCF4,MGST1,RBM44,ARHGEF38,PAEP,COP1,TIAM1,CSRP3,PHACTR1,MLIP,IQSEC2,ACVR2A,GNAO1,ASAP2,TIPIN,VIT,C6ORF58,ENSG00000288635,SNORA5C,SNTG2,NTM,AMMECR1,NEDD4L,BRCC3,ENSG00000288683,PRMT8,SNX13,RABGEF1,KIT,CYB5R4,TBC1D8,EPHA7,OSBPL3,SPAG9,ANTXR1,C19ORF47,SPDL1,WNT5A,TRABD2B,PEX26,TEX2,ARFGEF2,CTNNB1,ANKRD17,COL19A1,RUNX1T1,CTSK,PRKDC,IPO5,ESR1,TNFRSF19,ZNF479,LU

			ZP2,PTPN14,SCFD1,ERC2,POFUT2,DMGDH,BCAR3,TPD52L1,L3MBTL4,PPM1E,ZXDC,VPS53,FRMD4A,LMX1A,DCAF8L1,TPST2,FIGN,BTBD8,PARS2,BNIPL,HUNK,FILIP1,TMEM62,FBXO15,PTGER4,GREB1L,ZNF215,PDE7B,BBX,USP40,VWC2L,BEST3,LYPD6B,DOCK1,CCDC107,GPC4,BPGM,PKIG,EFNA5,IGF2BP2,ACTR3B,CDC14B,ZNF722,SYNE3,PLCH1,OR1M1,HECTD1,VKORC1L1,NGEF,TRPC5,NEUROD2,FNIP2,AASS,JAML,KAT14,CCNJ,PTPRF,IKBKB,FXR2,SH3BP5,AKAP6,TRBV10-2,USF3,LYVE1,APOH,ARHGEF28,RAB3GAP2,CLCN1,TASOR,CCPG1,GPRIN1,EML6,OPCML,LINGO2,CEMIP,ATF2,RNU6-986P,KCNJ12,ZNF426,HDAC8,ACVR1,CHRNB3,HLA-DQA2,PID1,COL6A5,HYAL4,EBF2,CCDC91,GRID2,ZNF423,ZCCHC17,KCNJ3,GLDC,PLPPR1,RB1,NSMCE1,MPV17,IFNLR1,RNU6-826P,PDE4B,DDX10,PKD1L3,KIF13A,NLRP4,ADIPOR1,LOC124900205,GABRB3,ZNF143,GOSR2,PET117,SHROOM4,ZSCAN23,SMG7,PLPP3,UNC80,HNF1B,RNU6-1311P,IMMP2L,ZNF615,LAMP2,SSPN,SPSB1,KIRREL3,GABRG2,F5,CFAP95,AK5,CPS1,NCOA2,TMEM120A,BNIP3L,ACACB,AGPAT5,RABGAP1L,ENSG00000286476,XRCC5,TBRG4,RGS17,SHLD2,GFRA1,SPHKAP,KCND2,INPP4A,BMX,ZNF418,SLC24A3,RAD54L2,MPRIP,TAF15,TPST1,GRIA4,RNA5SP489,RAB18,POLD3,ZNF860,TPH2,POU2F1,PRPF40B,TMTC1,ETV1,NRK,RGS7BP,BACH2,MGRN1,TEX11,MID2,RBM28,LRTM2,SIK1,ZCWPW2,FBXL13,ERCC6L2,PIPOX,ZNF850,TP53BP2,KAT2B,GOLPH3L,TMTC2,WLS,ACTR3C,ANKS1A,IGKV1-6,RYR3,TMPRSS11B,PRCP,SUPV3L1,MEF2D,MYO1B,MGAM2,MYL4,GTF2A1,SPTLC1,AGR3,CDK5RAP2,FLVCR1,UBE2E3,RFC3,FARSB,ARHGEF7,SAMD12,DACH1,ATF3,TRDN,GASK1A,NPHP1,IKZF1,SLC26A8,CAPZA2,SRI,ATP1B1,GREB1,ADAT2,ARNT,BDNF,LDLRAD4,TFCP2L1,ACBD6,SEMA3E,BCLAF3,SCIN,MACO1,ZNF665,TLR6,NDST4,ADAMTS9,STIM1,TSEN15,TMEM237,TMEM161B,HAUS6,RAB11FIP2,EEFSEC,COX7B2,LAG3,PPARG,TANC1,PAPPA,ENSG00000290217,ASB3,MTTP,CHRNB4,TGM5,NEK7,INTS15,ZNF80,TENM1,IL13RA2,RORB,GABRB1,MYOF,LTB4R2,JAKMIP1,SH3KBP1,NALCN,SMOX,AVIL,LPCAT2,GDAP1,AMY2A,MYLK4,BEND7,USP25,KIF2C,MYO5A,SIPA1L3,SLC6A13,LRFN2,WDFY4,SPTBN5,SFRP4,BLTP1,PNRC1,IL1RAP,JCAD,VAV2,TFRC,VPS13A,TES,OSBPL6,GRIA3,PNPLA8,FBN1,HAPSTR1,RAB31,DIPK2B,ZFP82,MAP3K1,DDX4,IQCB1,COA1,ZNF292,CLEC6A,BMPER,FUT10,RNU6-984P,CLASP2,ZNF521,RPRD2,ZNF761,ANGPT1,SEMA6A,VIPR2,EIF4G1,TMPRSS15,B3GAT2,ATP6V0A4,ACSM5,PHLPP1,ZNF618,RBKS,ITGA4,DNAJC6,ROCK1,NCAPH,M1AP,TAX1BP1,TBC1D4,APIP,MMP28,ZNF701,KCNT2,NUP98,CLEC3A,IKZF2,PML,FOXO3B,LAYN,MGLL,SCARA5,LONRF3,SLC5A12,PLEK2,TNFSF4,AFAP1L2,DGKG,SPATA25,SP100,RNA5SP260,PLXDC2,ELAVL4,SBF2,CDH9,FOXRED2,NDUFAF2,UMODL1,RFPL4B,TTC28,TSEN2,GALK2,PCDH19,NIN,SPG21,ZNF286A,ANKFY1,ZNF69,PRSS50,ENSA,FNDC3A,KHDRBS3,AIMP1,SLC30A10,AP4S1,TPK1,PRKD1,HSPBP1,CALCRL,SLC20A2

			,USP34,STK4,PCDH10,TNNI3K,RAB6D,TMEM131,SPC25,TMEM161B-DT,RNASE11,ST6GAL2,SCAMP1,PTCHD4,RPL27A,ANPEP,PRLR,ZNF569,ZNF180,PDE11A,NMI,RAB6C,PCCA,CLVS1,YLPM1,IL16,DIPK1A,ZSWIM7,ZNF470,POTEH,EDARADD,GNA14,CR2,LPAR6,CTBP2,TTN,NCALD,HHIP,DYRK1A,PPFIA1,TMEM30A,MYRIP,PPP1R1C,UNC13C,KCNE2,RGS10,CUL4A,SERINC1,KCTD16,CHSY3,TXNRD3,ZNF888,EYA4,ARL15,ZNF736,MAP7D3,SEMA5A,ELL2,LAMA4,PMEPA1,ATE1,ETNK1,TTPA,PVT1,CHAC2,GATAD2B,FMNL2,METTL3,KRT85,UAP1,PARD3,KCNN4,TRDC,ZNF33A,CP,FUNDC1,RNU6-849P,COL6A6,BMAL2,ATP8B1,PGPEP1,RNF6,SEMA3C,VAV1,SMYD1,PEPD,DPY19L1,GRIA2,VDAC2,VPS13B,SPART,IGLV4-3
GO:0098588	bounding membrane of organelle	0.0002886997413284381	RTN1,HS3ST3A1,MREG,NOSTRIN,WSCD1,MAOA,ARL13B,TF,CHST11,USP32,GNB4,ATP7B,CAMK2B,DCT,PECR,SLC38A9,GALNT9,NOTCH2,CD93,DSC1,MAP6,SRL,DIPK2A,FAM20B,PRKN,SLC5A7,MARCHF9,GPSM1,SLC17A6,GNAI1,ENPEP,MBTPS2,TM9SF2,CLCN3,ZFYVE16,CIDEB,MARCHF1,CDIP1,NDST1,ATP11A,SLC15A4,GOLPH3,MBTPS1,ATP13A4,SNX10,ATP2C1,STON2,HUWE1,ASPH,RRAS2,ZDHHC4,TLR8,ARMH3,ITPR1,CAMK1G,MR1,F8,MTX3,MGAM,CHCHD3,UVRAG,LYN,SEC24C,ATP6V1H,SGMS2,TMED7-TICAM2,NOS3,BPHL,SYNPR,CUX1,RRAGD,QTRT2,GAD1,OCA2,SAR1B,PI4KB,RHOT1,TLR1,LMAN1L,TICAM2,LRRK2,CLSTN2,PLD1,ITFG2,PJA2,MGST1,ENSG00000288683,SNX13,RABGEF1,SPAG9,ANTXR1,WNT5A,PEX26,ARFGEF2,SCFD1,VPS53,TPST2,BTBD8,SYNE3,FNIP2,AKAP6,CEMIP,ATF2,HLA-DQA2,MPV17,KIF13A,GOSR2,PLPP3,LAMP2,F5,BNIP3L,ACACB,AGPAT5,INPP4A,TPST1,GRIA4,RAB18,GOLPH3L,WLS,RYR3,PRCP,MYO1B,TRDN,SRI,LDLRAD4,MACO1,TLR6,NDST4,STIM1,RAB11FIP2,ENSG00000290217,CHRNB4,MYOF,LPCAT2,GDAP1,TFRC,VPS13A,OSBPL6,GRIA3,PNPLA8,RAB31,FUT10,B3GAT2,ATP6V0A4,ROCK1,PML,SBF2,SPG21,ANKFY1,FNDC3A,SLC30A10,PRKD1,ST6GAL2,SCAMP1,ANPEP,CLVS1,NCALD,HHIP,TMEM30A,UNC13C,CHSY3,PMEPA1,CP,FUNDC1,GRIA2,VDAC2,VPS13B,SPART
GO:0098796	membrane protein complex	0.0002944500680438749	TRGC2,STX8,IMMP1L,TF,CACNB1,GNG12,CHRNB2,CDH8,KCNQ5,GNB4,KCNJ6,SLC38A9,ABCG8,CDH11,DIPK2A,CACNA1C,PIK3C2B,SLC17A6,GNAI1,CHRFAM7A,CD247,CATSPERB,CDH7,GABRA5,CIDEB,SESTD1,KCNS3,CHRNA7,HMGB1,KCNJ18,KCNIP3,ATP4A,NCF2,STON2,ASPH,MR1,VPS8,MTX3,CHCHD3,UVRAG,LYN,GRIK1,SEC24C,ATP6V1H,MAGEL2,TGFBR2,ITGA11,SYNJ1,GPR156,CACNA2D4,SAR1B,HCN1,USE1,HOOK3,PIK3R5,TLR1,ATP1B4,UBXN7,ACVR2A,GNAO1,SNTG2,CTNNB1,BTBD8,VWC2L,BEST3,SYNE3,TRPC5,IKBKB,AKAP6,TRBV10-2,CLCN1,KCNJ12,ACVR1,CHRNB3,HLA-DQA2,GRID2,KCNJ3,IFNLR1,PDE4B,PKD1L3,GABRB3,GOSR2,UNC80,IMMP2L,LAMP2,SSPN,GABRG2,GFRA1,KCND2,GRIA4,RYR3,ATP1B1,TLR6,COX7B2,CHRNB4,GABRB1,NALCN,TFRC,GRIA3,ATP6V0A4,ITGA4,CDH9,ANKFY1,AP4S1,CALCRL,GNA14,NCALD,KCNE2,KCNN4,GRIA2,VDAC2
GO:0030	axon	0.000571872	APBB2,GARS1,ABR,SPTBN1,CDH8,KCNQ5,AGTPBP

424		0181793255	*1,EPB41L3,MAP6,SLC5A7,NCAM2,TIAM2,TRAK2,LRP8,CNTN1,CPEB4,CLCN3,PREX1,MYO1D,DTNA,TGFB2,EPHA5,KIF21A,MYH14,DIP2B,ALDH1A1,NMNAT3,NAV1,SYNJ1,GAD1,WHRN,APP,IGF2BP1,HCN1,PALLD,LRRK2,TIAM1,ERC2,BTBD8,NGEF,TRPC5,FXR2,GPRIN1,UNC80,KIRREL3,GABRG2,GFRA1,RGS7BP,BDNF,MACO1,TANC1,AVIL,MYO5A,CLASP2,SEMA6A,ITGA4,ELAVL4,SBF2,NIN,DYRK1A,PPFIA1,UNC13C,PARD3,RNF6*
GO:0034702	monoatomic ion channel complex	0.0006186202006056819	*CACNB1,CHRNB2,KCNQ5,KCNJ6,CACNA1C,SLC17A6,CHRFAM7A,CATSPERB,GABRA5,SESTD1,KCNS3,CHRNA7,KCNJ18,KCNIP3,ASPH,GRIK1,CACNA2D4,HCN1,VWC2L,BEST3,TRPC5,AKAP6,CLCN1,KCNJ12,CHRNB3,GRID2,KCNJ3,PDE4B,PKD1L3,GABRB3,UNC80,GABRG2,KCND2,GRIA4,RYR3,CHRNB4,GABRB1,NALCN,GRIA3,KCNE2,KCNN4,GRIA2*
GO:0005794	Golgi apparatus	0.0010203442666577769	*APBB2,RTN1,HS3ST3A1,WSCD1,STX8,TXNDC8,TRAPPC9,CHST11,USP32,ATP7B,KCNJ6,GALNT9,NOTCH2,MAP6,DIPK2A,FAM20B,FAM3C,PRKN,MARCHF9,GPSM1,SDF4,MUC17,MBTPS2,CD247,TM9SF2,CLCN3,GOLGA8R,CIDEB,MARCHF1,KCNS3,NDST1,JAM3,KCNIP3,ATP11A,GOLPH3,MBTPS1,ATP2C1,TMEM230,HUWE1,RRAS2,ZDHHC4,TLR8,ARMH3,CAMK1G,TRAPPC10,CPQ,MR1,F8,LYN,USP6,ATP6V1H,SGMS2,TMED7-TICAM2,NOS3,CUX1,GKAP1,SAR1B,APP,MAMLD1,PI4KB,HOOK3,TLR1,AVPR1B,LMAN1L,TICAM2,LRRK2,CLSTN2,PLD1,ITFG2,PKHD1,PJA2,ASAP2,NEDD4L,WNT5A,ARFGEF2,IPO5,ESR1,SCFD1,POFUT2,VPS53,TPST2,GPC4,HLA-DQA2,CCDC91,KIF13A,GOSR2,PLPP3,LAMP2,RABGAP1L,GFRA1,TPST1,RAB18,GOLPH3L,WLS,MYO1B,CDK5RAP2,DACH1,GASK1A,LDLRAD4,TLR6,NDST4,MTTP,TENM1,LPCAT2,SIPA1L3,VPS13A,RAB31,FUT10,CLASP2,B3GAT2,ROCK1,SPG21,FNDC3A,AIMP1,SLC30A10,AP4S1,PRKD1,RAB6D,ST6GAL2,SCAMP1,RAB6C,CLVS1,NCALD,TMEM30A,CHSY3,PMEPA1,METTL3,ATP8B1,VPS13B*
GO:0099572	postsynaptic specialization	0.0011899603939566753	*RTN1,ARHGEF9,SPTBN1,CHRNB2,GRIP1,DLGAP1,SHANK3,CACNA1C,PRKN,NLGN4X,CPEB4,ERBIN,GABRA5,TNIK,ADD3,ANKS1B,ITPR1,PKP4,PAK3,LYN,ADGRB3,GRIK1,DAPK1,INSYN2A,CLSTN2,PJA2,TIAM1,CTNNB1,FXR2,GRID2,PDE4B,KCND2,INPP4A,GRIA4,RGS7BP,TANC1,GABRB1,GRIA3,DNAJC6,GRIA2*
GO:0005769	early endosome	0.003200872412281204	*APBB2,STX8,BLTP3B,TF,RCSD1,TBC1D16,SLC5A7,TRAK2,CLCN3,ZFYVE16,MARCHF1,MYO1D,ATP11A,SLC15A4,USP10,ATP13A4,TMEM230,HPS3,MR1,VPS8,EPHA3,UVRAG,MAGEL2,APP,TICAM2,SNX13,RABGEF1,RABGAP1L,INPP4A,MGRN1,WLS,MYO1B,LDLRAD4,MYO5A,WDFY4,TFRC,OSBPL6,RAB31,PML,ANKFY1,SLC30A10,CLVS1,TMEM30A,PMEPA1,VPS13B*
GO:0098797	plasma membrane protein complex	0.0038264545111126418	*TRGC2,TF,CACNB1,GNG12,CHRNB2,CDH8,KCNQ5,GNB4,KCNJ6,ABCG8,CDH11,CACNA1C,GNAI1,CHRFAM7A,CD247,CATSPERB,CDH7,KCNS3,CHRNA7,HMGB1,KCNIP3,ATP4A,NCF2,STON2,MR1,LYN,GRIK1,TGFBR2,ITGA11,SYNJ1,GPR156,CACNA2D4,HCN1,TLR1,ATP1B4,ACVR2A,GNAO1,SNTG2,CTNNB1,BTBD8,VWC2L,IKBKB,TRBV10-2,ACVR1,CHRNB3,HLA-DQA2,GRID2,KCNJ3,IFNLR1,PDE4B,SSPN,GFRA1,KCND2,GRIA4,ATP1B1,TLR6,CHRNB4,TFRC,GRIA3,ITGA4,CDH9,CALCRL,GNA14,KCNE2,KCNN4,G*

			RIA2
GO:0098590	plasma membrane region	0.005259673086568985	*MREG,RAPGEF6,ARL13B,TF,SPTBN1,CHRNB2,CLDN6,CDH8,KCNQ5,ATP7B,GRIP1,ABCG8,EPB41L3,SLC14A2,STK39,CACNA1C,NLGN4X,SLC5A7,LRP8,CNTN1,ENPEP,ARHGEF4,MUC17,ERBIN,CLCN3,GABRA5,MYO1D,ALPK2,CHRNA7,TNIK,MUSK,PRKAR2A,ATP4A,PRKG2,NCKAP1,MGAM,ADGRB3,GRIK1,SLC12A1,NOS3,KIAA1614,TGFBR2,SYNJ1,ADGRV1,EPB41L5,WHRN,HCN1,CHRM5,SLC13A1,LRRK2,SLC38A4,CLSTN2,PLD1,PKHD1,TIAM1,ANTXR1,CTNNB1,CTSK,ERC2,EFNA5,AKAP6,CHRNB3,GRID2,KCNJ3,GABRB3,SHROOM4,PLPP3,SSPN,GABRG2,KCND2,BMX,GRIA4,RAB18,RGS7BP,GASK1A,ATP1B1,STIM1,MTTP,CHRNB4,GABRB1,MYOF,SIPA1L3,SLC6A13,LRFN2,JCAD,TFRC,GRIA3,CLASP2,ATP6V0A4,DNAJC6,SLC5A12,PLEK2,CDH9,SLC20A2,HHIP,TMEM30A,UNC13C,KCNE2,KCTD16,PARD3,ATP8B1,GRIA2*
GO:0031090	organelle membrane	0.016008568768072603	*ESYT1,RTN1,HS3ST3A1,MREG,NOSTRIN,WSCD1,OSBPL8,PHF20,MAOA,ARL13B,THADA,IMMP1L,TF,CHST11,USP32,CDC14C,GNB4,ATP10A,SPINK5,MRPS24,PIGL,ATP7B,TNRC18,CAMK2B,GRIP1,DCT,PECR,SLC38A9,SDR16C5,GALNT9,NOTCH2,CD93,DSC1,MAP6,SPTLC3,SRL,DIPK2A,FAM20B,PRKN,PNPLA3,SLC5A7,MARCHF9,GPSM1,SLC17A6,GNAI1,MRPL13,ENPEP,ERBIN,MBTPS2,TM9SF2,CLCN3,ZFYVE16,ILDR2,NCK1,GOLGA8R,CIDEB,MARCHF1,CDIP1,NDST1,NUP93,ATP11A,MRPL33,SLC15A4,GOLPH3,PRKG2,MBTPS1,PIGV,ATP13A4,SNX10,ATP2C1,STON2,HUWE1,ASPH,RRAS2,ZDHHC4,TLR8,ARMH3,ITPR1,CYP2A13,CAMK1G,MR1,F8,GDPD1,CPT2,MTX3,MGAM,EPHA3,CHCHD3,MRPL32,UVRAG,LYN,SEC24C,ATP6V1H,NUP58,PDZD8,MYRFL,FMO5,TMCC1,SGMS2,TMED7-TICAM2,NOS3,NOMO2,BPHL,SYNPR,HMGCLL1,SYNJ1,CUX1,SHISA2,RRAGD,KPNB1,GSDMD,RASGRF2,QTRT2,GAD1,OCA2,SAR1B,APP,PI4KB,RHOT1,ELOVL5,USE1,TLR1,LMAN1L,TICAM2,ATP1B4,PIP4K2B,UQCC1,LRRK2,GET1-SH3BGR,CLSTN2,PLD1,ITFG2,UBXN7,PJA2,MGST1,ASAP2,ENSG00000288683,SNX13,RABGEF1,CYB5R4,OSBPL3,SPAG9,ANTXR1,WNT5A,TRABD2B,PEX26,TEX2,ARFGEF2,ANKRD17,IPO5,SCFD1,POFUT2,VPS53,TPST2,BTBD8,SYNE3,VKORC1L1,FNIP2,SH3BP5,AKAP6,RAB3GAP2,CEMIP,ATF2,HLA-DQA2,MPV17,KIF13A,GABRB3,GOSR2,PLPP3,IMMP2L,LAMP2,GABRG2,F5,CPS1,TMEM120A,BNIP3L,ACACB,AGPAT5,INPP4A,TPST1,GRIA4,RAB18,GOLPH3L,TMTC2,WLS,RYR3,PRCP,MYO1B,SPTLC1,FLVCR1,TRDN,SRI,ATP1B1,LDLRAD4,MACO1,TLR6,NDST4,STIM1,RAB11FIP2,COX7B2,ENSG00000290217,CHRNB4,MYOF,SH3KBP1,SMOX,LPCAT2,GDAP1,SPTBN5,BLTP1,TFRC,VPS13A,OSBPL6,GRIA3,PNPLA8,RAB31,COA1,FUT10,B3GAT2,ATP6V0A4,ROCK1,NUP98,PML,MGLL,SBF2,NDUFAF2,SPG21,ANKFY1,FNDC3A,SLC30A10,AP4S1,PRKD1,ST6GAL2,SCAMP1,ANPEP,CLVS1,DIPK1A,NCALD,HHIP,TMEM30A,UNC13C,SERINC1,CHSY3,PMEPA1,CP,FUNDC1,RNF6,DPY19L1,GRIA2,VDAC2,VPS13B,SPART*
GO:0032279	asymmetric synapse	0.016430124676028057	*RTN1,ARHGEF9,SPTBN1,GRIP1,DLGAP1,SHANK3,PLXNA4,CACNA1C,PRKN,NLGN4X,CPEB4,TNIK,ADD3,ANKS1B,ITPR1,PKP4,PAK3,ADGRB3,GRIK1,DAPK1,INSYN2A,CLSTN2,PJA2,TIAM1,ARFGEF2,C*

			TNNB1,FXR2,GRID2,PDE4B,INPP4A,GRIA4,RGS7BP,TANC1,GRIA3,DNAJC6,GRIA2
GO:0043235	receptor complex	0.016598280408426962	*TRGC2,TF,CHRNB2,ABCG8,NOTCH2,PLXNA4,LRP8,CHRFAM7A,CD247,GABRA5,CHRNA7,MUSK,LYN,GRIK1,TGFBR2,ITGA11,GPR156,ADGRV1,APP,TLR1,ACVR2A,KIT,VWC2L,IKBKB,TRBV10-2,ACVR1,CHRNB3,GRID2,IFNLR1,PKD1L3,GABRB3,GABRG2,GFRA1,GRIA4,ARNT,TLR6,PPARG,MTTP,CHRNB4,IL13RA2,GABRB1,TFRC,GRIA3,ITGA4,CALCRL,PRLR,CR2,KCTD16,BMAL2,GRIA2*
GO:0031984	organelle subcompartment	0.016779529184761437	*ESYT1,RTN1,OSBPL8,STX8,TRAPPC9,THADA,CDC14C,ATP10A,SPINK5,PIGL,ATP7B,CAMK2B,GRIP1,SDR16C5,NOTCH2,SPTLC3,SRL,PNPLA3,MARCHF9,GPSM1,ILDR2,GOLGA8R,CIDEB,MARCHF1,NDST1,ATP11A,GOLPH3,MBTPS1,PIGV,ATP13A4,ATP2C1,TMEM230,ASPH,ZDHHC4,TLR8,ITPR1,CYP2A13,MR1,GDPD1,SEC24C,PDZD8,MYRFL,FMO5,TMCC1,TMED7-TICAM2,NOMO2,HMGCLL1,SHISA2,KPNB1,RASGRF2,OCA2,SAR1B,APP,PI4KB,ELOVL5,USE1,LMAN1L,PIP4K2B,LRRK2,GET1-SH3BGR,CLSTN2,PLD1,UBXN7,PJA2,MGST1,ASAP2,CYB5R4,OSBPL3,TEX2,ARFGEF2,SCFD1,POFUT2,VPS53,VKORC1L1,AKAP6,RAB3GAP2,HLA-DQA2,CCDC91,KIF13A,GOSR2,PLPP3,LAMP2,AGPAT5,RAB18,GOLPH3L,TMTC2,WLS,RYR3,MYO1B,SPTLC1,TRDN,SRI,MACO1,STIM1,LPCAT2,BLTP1,VPS13A,OSBPL6,PNPLA8,RAB31,FUT10,CLASP2,PML,MGLL,AP4S1,PRKD1,ST6GAL2,SCAMP1,CLVS1,DIPK1A,SERINC1,CHSY3,ATP8B1,DPY19L1,GRIA2,VPS13B*
GO:0005768	endosome	0.023576658016504236	*APBB2,MREG,STX8,BLTP3B,TRAPPC9,TF,RCSD1,ATP7B,SLC38A9,TBC1D16,SLC5A7,SDF4,TRAK2,TM9SF2,CLCN3,ZFYVE16,ASTN1,MARCHF1,MYO1D,HMGB1,CDIP1,TNIK,ATP11A,SLC15A4,GOLPH3,USP10,ATP13A4,SNX10,TMEM230,TLR8,HPS3,TRAPPC10,MR1,VPS8,EPHA3,UVRAG,USP6,ATP6V1H,MAGEL2,OCA2,APP,PI4KB,AVPR1B,TICAM2,LRRK2,PLD1,NEDD4L,SNX13,RABGEF1,ANTXR1,ARFGEF2,CTSK,VPS53,HLA-DQA2,KIF13A,GOSR2,LAMP2,RABGAP1L,GFRA1,INPP4A,MGRN1,WLS,MYO1B,LDLRAD4,RAB11FIP2,MYO5A,WDFY4,TFRC,VPS13A,OSBPL6,RAB31,ATP6V0A4,PML,SBF2,SPG21,ANKFY1,SLC30A10,AP4S1,CALCRL,SCAMP1,PRLR,CLVS1,TMEM30A,PMEPA1,TTPA,VPS13B*
GO:0014069	postsynaptic density	0.03107303717030402	*RTN1,ARHGEF9,SPTBN1,GRIP1,DLGAP1,SHANK3,CACNA1C,PRKN,NLGN4X,CPEB4,TNIK,ADD3,ANKS1B,ITPR1,PKP4,PAK3,ADGRB3,GRIK1,DAPK1,INSYN2A,CLSTN2,PJA2,TIAM1,CTNNB1,FXR2,GRID2,PDE4B,INPP4A,GRIA4,RGS7BP,TANC1,GRIA3,DNAJC6,GRIA2*
GO:0030427	site of polarized growth	0.046022364821349936	*APBB2,TIAM2,CPEB4,PREX1,MYH14,WHRN,APP,IGF2BP1,FRY,PALLD,LRRK2,TIAM1,ERC2,NGEF,TRPC5,FXR2,GPRIN1,MYO5A,CLASP2,ITGA4,ELAVL4,NIN*

Table S4. GO associations with biological processes (BP), molecular functions (MF), and cellular components (CC) of 659 rDNA-contacting genes common in Mel Z cells grown on either plastic or Matrigel. Related to Figure 2A and C.

GO.ID	Description	padj	Genes
		MF	
GO:0005509	calcium ion binding	1.9003958808377105e-14	*SLC8A1,PCDHA13,FSTL5,ASTN2,LRP1B,PCDHA12,NRXN1,DNAH7,ANXA10,FBLN7,CDH19,SLIT2,FAM20C,BRAF,HMCN1,RUNX1,PCDHA2,PCDHA11,PCLO,CDH18,PCDHA9,DGKB,PCDHAC2,CDH6,PCDHA10,EDIL3,KCNIP4,PCDH9,PCDHA1,PCDHA8,CDH12,CDH4,FBLN5,PCDHAC1,PCDH7,PCDH15,PCDHA7,PCDHA5,PLCB4,CALN1,GRM7,PCDHA6,MCTP1,CDH13,FBN2,PCDHA4,CDH2,TENM2,ADGRL4,PLCB1,NELL1,RYR2,FAT3,PCDHA3,EYS,DNER*
GO:0043169	cation binding	9.775360646731358e-9	*PIR,TRPM6,SLC8A1,PCDHA13,ZNF385B,DPYD,KDM4B,ZBTB20,FSTL5,TTC3,CADPS2,ASTN2,LRP1B,KALRN,SUMF1,XXYLT1,PCDHA12,MAST4,NRXN1,AGBL1,STK24,LMO7,DNAH7,NTRK2,ANXA10,ATP2B4,PHEX,PDE4DIP,FBLN7,DMD,ADCY2,CDH19,CHRM3,SLIT2,ZNF804A,FAM20C,BRAF,KMT2C,GPHN,ZNF385D,SH3RF3,EXT2,MAP3K5,HTR2C,GALNT13,HMCN1,ITGA1,RUNX1,ESRRG,PCDHA2,PCDHA11,PCLO,CDH18,PCDHA9,NEK1,DGKB,EBF1,PCDHAC2,CDH6,JAZF1,ZNF267,CACNA2D3,PCDHA10,EDIL3,DGKI,LARGE1,PPM1L,CASZ1,TRIM71,KCNIP4,PCDH9,PCDHA1,ZNF800,PCDHA8,CDH12,TRPS1,CDH4,FBLN5,ZNF638,ATP9B,CACNA2D1,NSMCE2,PCDHAC1,HERC2,UTRN,AGBL4,OGDH,PCDH7,SMAD9,ZFPM2,GALNT17,DNMT3A,PCDH15,ACE2,CHMP3,PDE4D,ITGB8,PDE3A,RIMS1,PCDHA7,NEK10,MIPEP,PCDHA5,PLCB4,RNF216,FTO,MGAT4C,CALN1,ZNF804B,GRM7,PRKCH,NRXN3,ATP8A2,ADARB2,COMT,PCDHA6,MCTP1,KMT2E,CDH13,FBN2,TTLL6,PCDHA4,SLC8A3,TENT4B,CDH2,TENM2,VPS41,TEX13D,HDAC9,USP13,ADGRL4,CADPS,SCEL,FREM2,RNF220,RORA,PLCB1,NPEPPS,BRIP1,NELL1,GALNTL6,PXDNL,MICAL3,ADK,RYR2,FAT3,MSH2,PCDHA3,MGAT4A,EYS,KSR2,BMPR1B,DNER,PGM3*
GO:0046872	metal ion binding	1.43484184950939e-8	*PIR,TRPM6,SLC8A1,PCDHA13,ZNF385B,DPYD,KDM4B,ZBTB20,FSTL5,TTC3,CADPS2,ASTN2,LRP1B,KALRN,SUMF1,XXYLT1,PCDHA12,MAST4,NRXN1,AGBL1,STK24,LMO7,DNAH7,NTRK2,ANXA10,ATP2B4,PHEX,PDE4DIP,FBLN7,DMD,ADCY2,CDH19,SLIT2,ZNF804A,FAM20C,BRAF,KMT2C,GPHN,ZNF385D,SH3RF3,EXT2,MAP3K5,GALNT13,HMCN1,ITGA1,RUNX1,ESRRG,PCDHA2,PCDHA11,PCLO,CDH18,PCDHA9,NEK1,DGKB,EBF1,PCDHAC2,CDH6,JAZF1,ZNF267,CACNA2D3,PCDHA10,EDIL3,DGKI,LARGE1,PPM1L,CASZ1,TRIM71,KCNIP4,PCDH9,PCDHA1,ZNF800,PCDHA8,CDH12,TRPS1,CDH4,FBLN5,ZNF638,ATP9B,CACNA2D1,NSMCE2,PCDHAC1,HERC2,UTRN,AGBL4,OGDH,PCDH7,SMAD9,ZFPM2,GALNT17,DNMT3A,PCDH15,ACE2,PDE4D,ITGB8,PDE3A,RIMS1,PCDHA7,NEK10,MIPEP,PCDHA5,PLCB4,RNF216,FTO,MGAT4C,CALN1,ZNF804B,GRM7,PRKCH,NRXN3,ATP8A2,ADARB2,COMT,PCDHA6,MCTP1,KMT2E,CDH13,FBN2,TTLL6,PCDHA4,SLC8A3,TENT4B,CDH2,TENM2,VPS41,TEX13D,HDAC9,USP13,ADGRL4,CADPS,SCEL,FREM2,RNF220,RORA,PLCB1,NPEPPS,BRIP1,NELL1,GALNTL6,PXDNL,MICAL3,ADK,RYR2,FAT3,MSH2,PCDHA3,MGAT4A,EYS,KSR2,BMPR1B,DNER,PGM3*
GO:0043167	ion binding	4.5158112693025055e-8	*ABCC6,PIR,TRPM6,SLC8A1,PCDHA13,ZNF385B,DPYD,KDM4B,ZBTB20,FSTL5,TTC3,CADPS2,TLK1,ASTN2,LRP1B,KALRN,SUMF1,XXYLT1,PCDHA12,MAST4,NRXN1,AGBL1,MAGI1,FARS2,STK24,LMO7,DNAH7,NTRK2,ANXA10,CHD7,ATP2B4,PHEX,PDE4DIP,F*

			BLN7,DNAH8,DMD,ADCY2,CDH19,CHRM3,SLIT2,ZNF804A,FAM20C,BRAF,KMT2C,ERBB4,KIF26B,GPHN,ZNF385D,RAD51B,MYO3B,SH3RF3,EXT2,MAP3K5,HTR2C,GALNT13,PIK3C3,HMCN1,ITGA1,ENSG00000289694,RUNX1,MYO16,ESRRG,PCDHA2,PCDHA11,PCLO,CDH18,PCDHA9,NEK1,DGKB,EBF1,PCDHAC2,CDH6,JAZF1,ZNF267,CACNA2D3,PCDHA10,EDIL3,DGKI,LARGE1,EPHA6,IGF1R,PPM1L,CASZ1,TRIM71,KCNIP4,CFTR,PCDH9,PCDHA1,ZNF800,PCDHA8,TBCK,CDH12,DDR2,LARS2,TRPS1,CDH4,FBLN5,ZNF638,ATP9B,CACNA2D1,NSMCE2,PCDHAC1,HERC2,UTRN,AGBL4,OGDH,ULK4,PCDH7,SMAD9,DYNC1H1,LANCL2,ZFPM2,FRMPD4,GALNT17,WIPI2,DNMT3A,PCDH15,ACE2,CHMP3,KCNAB1,PDE4D,ITGB8,PDE3A,RIMS1,PCDHA7,NEK10,MIPEP,PCDHA5,PLCB4,RNF216,FTO,MGAT4C,CALN1,UXS1,ZNF804B,GRM7,NAV3,PRKCH,NRXN3,ATP8A2,PLEKHA5,ADARB2,COMT,PCDHA6,MCTP1,KMT2E,CDH13,FBN2,TTLL6,PCDHA4,ALK,SLC8A3,MYO5B,TENT4B,DCLK1,KIF16B,CDH2,TENM2,VPS41,TEX13D,HDAC9,USP13,CDK6,ADGRL4,CADPS,DDX60,SCEL,FREM2,RAPGEF4,RNF220,RORA,PLCB1,NPEPPS,PRKG1,BRIP1,NELL1,GALNTL6,PXDNL,MICAL3,ADK,RANBP17,RYR2,FAT3,MSH2,PCDHA3,MGAT4A,EYS,KSR2,BMPR1B,DNER,PGM3,DNAH5
GO:0036094	small molecule binding	1.3612079475331344e-7	*ABCC6,PIR,TRPM6,SLC8A1,PCDHA13,ZNF385B,DPYD,KDM4B,ZBTB20,FSTL5,TTC3,CADPS2,TLK1,ASTN2,LRP1B,KALRN,SUMF1,XXYLT1,PCDHA12,MAST4,NRXN1,AGBL1,MAGI1,FARS2,STK24,LMO7,DNAH7,NTRK2,ANXA10,CHD7,ATP2B4,PHEX,PDE4DIP,FBLN7,DNAH8,DMD,ADCY2,CDH19,CHRM3,SLIT2,ZNF804A,FAM20C,BRAF,KMT2C,ERBB4,KIF26B,GPHN,ZNF385D,RAD51B,MYO3B,SH3RF3,EXT2,MAP3K5,HTR2C,GALNT13,PIK3C3,HMCN1,ITGA1,ENSG00000289694,RUNX1,MYO16,ESRRG,PCDHA2,PCDHA11,PCLO,CDH18,PCDHA9,NEK1,DGKB,EBF1,PCDHAC2,CDH6,JAZF1,ZNF267,CACNA2D3,PCDHA10,EDIL3,DGKI,COLEC10,LARGE1,EPHA6,IGF1R,PPM1L,CASZ1,TRIM71,KCNIP4,CFTR,PCDH9,PCDHA1,ZNF800,PCDHA8,TBCK,CDH12,DDR2,LARS2,TRPS1,CDH4,FBLN5,ZNF638,ATP9B,CACNA2D1,NSMCE2,PCDHAC1,HERC2,UTRN,AGBL4,OGDH,ULK4,PCDH7,SMAD9,DYNC1H1,OSBPL10,LANCL2,ZFPM2,FRMPD4,GALNT17,FHIT,WIPI2,DNMT3A,PCDH15,ACE2,CHMP3,KCNAB1,PDE4D,ITGB8,PDE3A,RIMS1,PCDHA7,NEK10,MIPEP,PCDHA5,PLCB4,RNF216,FTO,MGAT4C,CALN1,UXS1,ZNF804B,GRM7,NAV3,PRKCH,NRXN3,ATP8A2,PLEKHA5,ADARB2,COMT,PCDHA6,MCTP1,KMT2E,CDH13,FBN2,TTLL6,PCDHA4,ALK,SLC8A3,MYO5B,TENT4B,DCLK1,KIF16B,CDH2,TENM2,VPS41,TEX13D,HDAC9,USP13,CDK6,ADGRL4,CADPS,DDX60,SCEL,FREM2,RAPGEF4,RNF220,RORA,PLCB1,NPEPPS,PRKG1,BRIP1,NELL1,GALNTL6,PXDNL,MICAL3,ADK,RANBP17,RYR2,FAT3,MSH2,PCDHA3,MGAT4A,EYS,KSR2,BMPR1B,DNER,PGM3,DNAH5*
GO:0050839	cell adhesion molecule binding	0.000006294861908954311	*EPN2,FGF1,NRXN1,STK24,DMD,CDH19,DSCAML1,CNTN4,ITGA1,CDH18,CDH6,EDIL3,DSCAM,NLGN1,CDH12,ADAM22,CDH4,CNTN6,FBLN5,UTRN,LRRC4C,CNTN5,ITGB8,DIAPH3,NTNG1,PTPRM,NRXN3,CDH13,CDH2,TENM2,PTPRT,CTNNA3,PTPRD,CTNNA2,NRG1*
GO:0005001	transmembrane	0.00001566898184961895	*PTPRR,PTPRG,PTPRN2,PTPRM,PTPRT,PTPRD,PTPRA*

	receptor protein tyrosine phosphatase activity	6	
GO:0019198	transmembrane receptor protein phosphatase activity	0.0000156689818496189 56	*PTPRR,PTPRG,PTPRN2,PTPRM,PTPRT,PTPRD,PTPRA*
GO:0099106	ion channel regulator activity	0.001229426049811206	*FGF12,NRXN1,DPP6,GRM3,ATP2B4,FGF13,FGF14,KCNIP4,CFTR,DPP10,KCNAB1,PDE4D,NRXN3,PRKG1*
GO:0016247	channel regulator activity	0.001843694866857856	*FGF12,NRXN1,DPP6,GRM3,ATP2B4,FGF13,FGF14,KCNIP4,CFTR,DPP10,KCNAB1,PDE4D,NRXN3,PRKG1*
GO:0141108	transporter regulator activity	0.001995126394459651	*FGF12,NRXN1,DPP6,GRM3,ATP2B4,FGF13,FGF14,KCNIP4,CFTR,DPP10,KCNAB1,PDE4D,NRXN3,PRKG1*
GO:0060589	nucleoside-triphosphatase regulator activity	0.003928399959292593	*ARHGAP15,KALRN,FAM13A,RASGEF1B,ELMO1,RAPGEF5,ARHGAP24,SLIT2,TBC1D5,RAP1GAP2,DGKI,DENND1B,IQSEC1,TBCK,MCF2L2,HERC2,RIMS1,DLC1,DENND2B,DOCK2,RGS7,SRGAP2,RAPGEF4,ARHGEF37,PLCB1,DOCK10,RGS9*
GO:0030695	GTPase regulator activity	0.003928399959292593	*ARHGAP15,KALRN,FAM13A,RASGEF1B,ELMO1,RAPGEF5,ARHGAP24,SLIT2,TBC1D5,RAP1GAP2,DGKI,DENND1B,IQSEC1,TBCK,MCF2L2,HERC2,RIMS1,DLC1,DENND2B,DOCK2,RGS7,SRGAP2,RAPGEF4,ARHGEF37,PLCB1,DOCK10,RGS9*
GO:0030554	adenyl nucleotide binding	0.0047210934173129455	*ABCC6,TRPM6,DPYD,TLK1,KALRN,MAST4,MAGI1,FARS2,STK24,DNAH7,NTRK2,CHD7,ATP2B4,DNAH8,ADCY2,FAM20C,BRAF,ERBB4,KIF26B,GPHN,RAD51B,MYO3B,MAP3K5,PIK3C3,RUNX1,MYO16,NEK1,DGKB,DGKI,EPHA6,IGF1R,CFTR,TBCK,DDR2,LARS2,ATP9B,ULK4,DYNC1H1,LANCL2,KCNAB1,PDE4D,NEK10,UXS1,NAV3,PRKCH,ATP8A2,TTLL6,ALK,MYO5B,DCLK1,KIF16B,CDK6,DDX60,RAPGEF4,PRKG1,BRIP1,ADK,MSH2,KSR2,BMPR1B,DNAH5*
GO:0032559	adenyl ribonucleotide binding	0.006379699250517072	*ABCC6,TRPM6,TLK1,KALRN,MAST4,MAGI1,FARS2,STK24,DNAH7,NTRK2,CHD7,ATP2B4,DNAH8,ADCY2,FAM20C,BRAF,ERBB4,KIF26B,GPHN,RAD51B,MYO3B,MAP3K5,PIK3C3,RUNX1,MYO16,NEK1,DGKB,DGKI,EPHA6,IGF1R,CFTR,TBCK,DDR2,LARS2,ATP9B,ULK4,DYNC1H1,LANCL2,PDE4D,NEK10,NAV3,PRKCH,ATP8A2,TTLL6,ALK,MYO5B,DCLK1,KIF16B,CDK6,DDX60,RAPGEF4,PRKG1,BRIP1,ADK,MSH2,KSR2,BMPR1B,DNAH5*
GO:0005524	ATP binding	0.011583080235336801	*ABCC6,TRPM6,TLK1,KALRN,MAST4,MAGI1,FARS2,STK24,DNAH7,NTRK2,CHD7,ATP2B4,DNAH8,ADCY2,FAM20C,BRAF,ERBB4,KIF26B,GPHN,RAD51B,MYO3B,MAP3K5,PIK3C3,RUNX1,MYO16,NEK1,DGKB,DGKI,EPHA6,IGF1R,CFTR,TBCK,DDR2,LARS2,ATP9B,ULK4,DYNC1H1,LANCL2,NEK10,NAV3,PRKCH,ATP8A2,TTLL6,ALK,MYO5B,DCLK1,KIF16B,CDK6,DDX60,PRKG1,BRIP1,ADK,MSH2,KSR2,BMPR1B,DNAH5*
GO:0005	protein	0.029278966	*PAX7,RALYL,PTPRR,EPB41L4B,LRMDA,EPN2,PIR,*

515	binding	733245163	*NFIA,DIP2A,TRPM6,SLC8A1,ANO5,ZNF385B,PREP,RSRC1,GRIK2,DPYD,SLC22A14,FGF1,ZBTB20,AHR,FSTL5,TTC3,TLK1,ASTN2,FAM9B,LRP1B,KALRN,MKLN1,SUMF1,USH2A,NEGR1,FGF12,BTBD9,PARP4,MAST4,NRXN1,AGBL1,MAGI1,GNGT1,FARS2,ST6GALNAC3,DAAM2,STK24,LMO7,GRM3,EXOC4,DNAH7,BBS9,NTRK2,ANXA10,CHD7,NBEA,RASGEF1B,TMX3,NRG3,PTPRG,CALCR,ELMO1,NCOA1,IRAG1,ATP2B4,TOX2,PHEX,SOX6,PDE4DIP,FBLN7,DNAH8,RAPGEF5,FGF13,PATJ,GPC3,DMD,ADCY2,KCNQ3,CDH19,ATRNL1,CHRM3,ARHGAP24,SLIT2,FAM20C,TTC33,KAZN,DNAAF9,BRAF,BABAM2,KMT2C,B3GNT5,ERBB4,KIF26B,GPHN,ATXN7L1,RAD51B,DOP1B,SH2D1A,MYO3B,SH3RF3,EXT2,DIAPH2,MAP3K5,STAG2,SPAG16,DSCAML1,HTR2C,TBC1D5,CNTN4,RAP1GAP2,CCSER1,PIK3C3,HMCN1,ITGA1,TCF12,SGIP1,ARMC8,RUNX1,MYO16,ESRRG,PCLO,CDH18,FRMD6,ANK2,ANO2,SUPT3H,ROBO1,NEK1,EBF1,PRRC2C,CDH6,DOK6,WDR64,JAZF1,CHODL,FGF14,EDIL3,LRRTM4,INPP4B,SOX5,DSCAM,DGKI,DENND1B,COLEC10,LARGE1,SDK1,PUS7,CCDC33,EPHA6,IGF1R,WDR72,SPTB,NLGN1,IQSEC1,ANK1,CNTNAP2,TRIM71,KCNIP4,CFTR,DYNC1I1,NOS1AP,OTOGL,ATP6V0D2,TBCK,CDH12,SHC4,DDR2,ADAM22,GPC6,TRPS1,CDH4,DPP10,CELF4,CNTN6,PARM1,FBLN5,SPEF2,WDSUB1,MITF,NSMCE2,PCSK1,HERC2,LRFN5,UTRN,GPC5,AGBL4,OGDH,GHR,RBFOX1,LRRC4C,PPP1R9A,SMAD9,DYNC1H1,CRPPA,NEBL,NAALADL2,OSBPL10,LANCL2,ZFPM2,SLC35F1,FRMPD4,MEIS1,EDAR,RIMBP2,FHIT,WIPI2,DNMT3A,TOX,SGCZ,ACE2,CHMP3,KCNAB1,PDE4D,CNTN5,ITGB8,PDE3A,RIMS1,TRANK1,WWOX,PCDHA7,BEND5,ANAPC10,LSAMP,DIAPH3,CPLANE1,NOL4,PLCB4,RNF216,FTO,UGGT2,TNFSF11,PPFIA2,ENOX1,WNT2,STEAP1B,UMAD1,POU6F2,SFI1,ANKRD44,CALN1,UXS1,NTNG1,GRM7,NAV3,PTPRM,PRKCH,NRXN3,DLC1,DENND2B,ATP8A2,CERS6,LPIN1,PLEKHA5,AUTS2,SYT16,BCAS3,COMT,NBAS,KMT2E,SYNE2,DOCK2,SDCCAG8,SSH2,CDH13,MDGA2,FBN2,DAB1,TTLL6,PCDHA4,SNTG1,ALK,SEMA3A,YEATS2,SLC8A3,MYO5B,MALRD1,TENT4B,DCLK1,KIF16B,CDH2,TENM2,VPS41,HDAC9,USP9X,KCNB2,USP13,NBPF1,CDK6,LYST,CADPS,DDX60,RGS7,SRGAP2,TFAP2D,PPP2R2C,HEATR4,SPECC1,PTPRT,GABPB1,CSMD1,SCEL,CTNNA3,FREM2,RAPGEF4,CLTC,THSD7B,SORCS1,CAMKMT,RNF220,ARHGEF37,DLG2,SACS,PTPRD,RORA,PLCB1,PRKG1,BRIP1,MAGI2,NELL1,PXDNL,SCN2A,ASIC2,DOCK10,MICAL3,RANBP17,RYR2,SLX4IP,LDLRAD3,PTPRA,MSH2,MGAT4A,KLHDC10,MPDZ,SLF1,ROBO2,PAX3,KSR2,DLGAP2,BMPR1B,DCC,CTNNA2,PEX5L,DNER,ETV6,CEP112,NRG1,SLC25A48,NPAS3,DNAH5,FANCB*
GO:0098631	cell adhesion mediator activity	0.0440496625166709	*DSCAML1,CNTN4,ITGA1,DSCAM,CNTN6,LRRC4C,CNTN5,NTNG1*
		BP	
GO:0007399	nervous system development	2.7223814098461145e-30	*NFIA,DIP2A,PCDHA13,KDM4B,ASTN2,KALRN,USH2A,NEGR1,FGF12,PCDHA12,NRXN1,GNGT1,DAAM2,STK24,NTRK2,CHD7,NRG3,PTPRG,NCOA1,ATP2B4,SOX6,RAPGEF5,FGF13,DMD,KCNQ3,CHRM3,SLIT2,ZNF804A,BRAF,B3GNT5,ERBB4,DSCAML1,CNTN4,ITGA1,TCF12,RUNX1,MYO16,PCDHA2,PCDHA11,PCLO*

			,ANK2,PCDHA9,ROBO1,PCDHAC2,CHODL,FGF14,PCDHA10,SOX5,DSCAM,LARGE1,SDK1,EPHA6,IGF1R,NLGN1,IQSEC1,CASZ1,CNTNAP2,TRIM71,PCDH9,PCDHA1,PCDHA8,DDR2,ADAM22,CDH4,CNTN6,SPEF2,PCSK1,PCDHAC1,LRFN5,AGBL4,OGDH,ULK4,RBFOX1,LRRC4C,PPP1R9A,CRPPA,MEIS1,BRINP3,DNMT3A,TOX,PCDH15,CNTN5,RIMS1,PCDHA7,LSAMP,DIAPH3,CPLANE1,PCDHA5,PPFIA2,WNT2,POU6F2,NTNG1,GRM7,NAV3,PTPRM,PRKCH,NRXN3,DLC1,ATP8A2,LPIN1,MACROD2,AUTS2,COMT,PCDHA6,SYNE2,SDCCAG8,MDGA2,DAB1,PCDHA4,ALK,SEMA3A,SLC8A3,DCLK1,CDH2,TENM2,HDAC9,USP9X,CDK6,SRGAP2,TFAP2D,RNF220,AFF2,PTPRD,RORA,PLCB1,PRKG1,MAGI2,NELL1,SCN2A,ASIC2,DOCK10,FAT3,PCDHA3,RGS9,ROBO2,PAX3,BMPR1B,DCC,CTNNA2,DNER,ETV6,NRG1,DNAH5
GO:0048731	system development	2.481577761452292e-25	EPN2,NFIA,DIP2A,SLC8A1,PCDHA13,KDM4B,FGF1,AHR,ASTN2,KALRN,USH2A,NEGR1,FGF12,PCDHA12,NRXN1,GNGT1,DAAM2,STK24,NTRK2,CHD7,NRG3,PTPRG,NCOA1,ATP2B4,PHEX,SOX6,RAPGEF5,FGF13,GPC3,DMD,KCNQ3,CHRM3,ARHGAP24,SLIT2,ZNF804A,FAM20C,BRAF,B3GNT5,ERBB4,KIF26B,EXT2,DSCAML1,CNTN4,ITGA1,TCF12,RUNX1,MYO16,PCDHA2,PCDHA11,PCLO,ANK2,PCDHA9,ROBO1,PCDHAC2,CHODL,FGF14,PCDHA10,SOX5,DSCAM,LARGE1,SDK1,EPHA6,IGF1R,NLGN1,IQSEC1,CASZ1,CNTNAP2,TRIM71,PCDH9,PCDHA1,ZNF800,PCDHA8,DDR2,ADAM22,TRPS1,CDH4,CELF4,CNTN6,SPEF2,MITF,PCSK1,PCDHAC1,LRFN5,AGBL4,OGDH,GHR,ULK4,RBFOX1,LRRC4C,PPP1R9A,CRPPA,NEBL,ZFPM2,MEIS1,EDAR,BRINP3,DNMT3A,TOX,PCDH15,SGCZ,CNTN5,ITGB8,RIMS1,WWOX,PCDHA7,LSAMP,DIAPH3,CPLANE1,PCDHA5,TNFSF11,PPFIA2,WNT2,POU6F2,NTNG1,SGCD,GRM7,NAV3,PTPRM,PRKCH,NRXN3,DLC1,ATP8A2,LPIN1,PLEKHA5,MACROD2,AUTS2,BCAS3,COMT,PCDHA6,SYNE2,SDCCAG8,CDH13,MDGA2,FBN2,DAB1,PCDHA4,ALK,SEMA3A,SLC8A3,DCLK1,CDH2,TENM2,HDAC9,USP9X,CDK6,SRGAP2,TFAP2D,CSMD1,FREM2,RNF220,AFF2,PTPRD,RORA,PLCB1,PRKG1,BRIP1,MAGI2,NELL1,SCN2A,ASIC2,DOCK10,RYR2,FAT3,MSH2,PCDHA3,RGS9,ROBO2,PAX3,BMPR1B,DCC,CTNNA2,DNER,ETV6,NRG1,DNAH5
GO:0007156	homophilic cell adhesion via plasma membrane adhesion molecules	5.518392844395093e-25	PCDHA13,PCDHA12,CDH19,DSCAML1,CNTN4,HMCN1,PCDHA2,PCDHA11,CDH18,PCDHA9,ROBO1,PCDHAC2,CDH6,PCDHA10,DSCAM,SDK1,PCDH9,PCDHA1,PCDHA8,CDH12,CDH4,CNTN6,PCDHAC1,PCDH7,PCDH15,PCDHA7,PCDHA5,PTPRM,PCDHA6,CDH13,PCDHA4,CADM2,CDH2,PTPRT,FAT3,PCDHA3,ROBO2
GO:0098742	cell-cell adhesion via plasma-membrane adhesion molecules	1.1038957988092012e-24	PCDHA13,PCDHA12,NRXN1,CDH19,DSCAML1,CNTN4,HMCN1,PCDHA2,PCDHA11,CDH18,PCDHA9,ROBO1,PCDHAC2,CDH6,PCDHA10,DSCAM,SDK1,NLGN1,PCDH9,PCDHA1,PCDHA8,CDH12,CDH4,CNTN6,PCDHAC1,LRFN5,PCDH7,LRRC4C,PCDH15,PCDHA7,PCDHA5,NTNG1,PTPRM,PCDHA6,CDH13,DAB1,PCDHA4,CADM2,CDH2,TENM2,PTPRT,PTPRD,FAT3,PCDHA3,ROBO2
GO:0007275	multicellular organism development	2.7967843680798984e-23	PTPRR,EPN2,NFIA,DIP2A,SLC8A1,PCDHA13,KDM4B,FGF1,AHR,ASTN2,KALRN,USH2A,NEGR1,FGF12,PCDHA12,NRXN1,GNGT1,DAAM2,STK24,EXOC4,NTRK2,CHD7,NRG3,PTPRG,NCOA1,ATP2B4,PHEX,SOX6,TMEM64,RAPGEF5,FGF13,GPC3,DMD,KCNQ3,CHRM

			3,ARHGAP24,SLIT2,ZNF804A,FAM20C,BRAF,B3GNT5,ERBB4,KIF26B,RAD51B,DOP1B,MYO3B,EXT2,DSCAML1,CNTN4,ITGA1,TCF12,RUNX1,MYO16,PCDHA2,PCDHA11,PCLO,ANK2,PCDHA9,ROBO1,PCDHAC2,CHODL,FGF14,PCDHA10,SOX5,DSCAM,LARGE1,SDK1,EPHA6,IGF1R,NLGN1,IQSEC1,CASZ1,CNTNAP2,TRIM71,CFTR,PCDH9,PCDHA1,ZNF800,PCDHA8,DDR2,ADAM22,TRPS1,CDH4,CELF4,ST8SIA6,CNTN6,SPEF2,MITF,PCSK1,PCDHAC1,LRFN5,AGBL4,OGDH,GHR,ULK4,RBFOX1,LRRC4C,PPP1R9A,CRPPA,NEBL,ZFPM2,MEIS1,EDAR,BRINP3,DNMT3A,TOX,PCDH15,SGCZ,CNTN5,ITGB8,RIMS1,WWOX,PCDHA7,LSAMP,DIAPH3,CPLANE1,PCDHA5,TNFSF11,PPFIA2,WNT2,POU6F2,NTNG1,SGCD,GRM7,NAV3,PTPRM,PRKCH,NRXN3,DLC1,ATP8A2,LPIN1,PLEKHA5,MACROD2,AUTS2,BCAS3,COMT,PCDHA6,SYNE2,SDCCAG8,CDH13,MDGA2,FBN2,DAB1,PCDHA4,ALK,SEMA3A,YEATS2,SLC8A3,DCLK1,KIF16B,CDH2,TENM2,HDAC9,USP9X,CDK6,SRGAP2,TFAP2D,SPECC1,CSMD1,SCEL,FREM2,DACH2,RNF220,AFF2,PTPRD,RORA,PLCB1,PRKG1,BRIP1,MAGI2,NELL1,SCN2A,ASIC2,DOCK10,RYR2,FAT3,MSH2,PCDHA3,RGS9,ROBO2,PAX3,BMPR1B,DCC,CTNNA2,DNER,ETV6,NRG1,DNAH5
GO:0048856	anatomical structure development	8.722353756601696e-23	*PAX7,PTPRR,EPN2,PIR,NFIA,DIP2A,SLC8A1,PCDHA13,SLC22A14,KDM4B,FGF1,ARHGAP15,AHR,ASTN2,FAM9B,KALRN,MKLN1,USH2A,NEGR1,FGF12,PCDHA12,NRXN1,GNGT1,DAAM2,STK24,EXOC4,NTRK2,CHD7,NRG3,PTPRG,CALCR,NCOA1,ATP2B4,PHEX,SOX6,TMEM64,RAPGEF5,FGF13,GPC3,DMD,KCNQ3,CDH19,CHRM3,ARHGAP24,SLIT2,ZNF804A,FAM20C,KAZN,BRAF,B3GNT5,ERBB4,KIF26B,RAD51B,DOP1B,MYO3B,EXT2,DIAPH2,MAP3K5,SPAG16,DSCAML1,CNTN4,ITGA1,TCF12,RUNX1,MYO16,PCDHA2,PCDHA11,PCLO,CDH18,FRMD6,ANK2,PCDHA9,ROBO1,PRRC2C,PCDHAC2,CDH6,CHODL,FGF14,PCDHA10,SOX5,DSCAM,COLEC10,LARGE1,SDK1,PUS7,EPHA6,IGF1R,WDR72,NLGN1,IQSEC1,CASZ1,CNTNAP2,TRIM71,CFTR,PCDH9,PCDHA1,ZNF800,PCDHA8,CDH12,DDR2,ADAM22,TRPS1,CDH4,CELF4,ST8SIA6,CNTN6,SPEF2,MITF,PCSK1,PCDHAC1,LRFN5,UTRN,AGBL4,OGDH,GHR,ULK4,RBFOX1,LRRC4C,PPP1R9A,SMAD9,CRPPA,NEBL,ZFPM2,MEIS1,EDAR,BRINP3,DNMT3A,TOX,PCDH15,SGCZ,PDE4D,CNTN5,ITGB8,PDE3A,RIMS1,WWOX,PCDHA7,LSAMP,DIAPH3,CPLANE1,PCDHA5,FTO,TNFSF11,PPFIA2,WNT2,POU6F2,NTNG1,SGCD,GRM7,NAV3,PTPRM,PRKCH,NRXN3,DLC1,ATP8A2,LPIN1,PLEKHA5,MACROD2,AUTS2,BCAS3,COMT,PCDHA6,KMT2E,SYNE2,DOCK2,SDCCAG8,CDH13,MDGA2,FBN2,DAB1,PCDHA4,ALK,SEMA3A,YEATS2,SLC8A3,DCLK1,KIF16B,CDH2,TENM2,HDAC9,USP9X,CDK6,SRGAP2,TFAP2D,SPECC1,CSMD1,SCEL,FREM2,DACH2,RNF220,AFF2,PTPRD,RORA,PLCB1,PRKG1,BRIP1,MAGI2,NELL1,SCN2A,ASIC2,DOCK10,RYR2,FAT3,MSH2,PCDHA3,RGS9,EYS,ROBO2,PAX3,BMPR1B,DCC,CTNNA2,DNER,ETV6,NRG1,PGM3,DNAH5*
GO:0032502	developmental process	3.2038834423764702e-21	*PAX7,PTPRR,LRMDA,EPN2,PIR,NFIA,DIP2A,SLC8A1,PCDHA13,SLC22A14,KDM4B,FGF1,ARHGAP15,AHR,FSTL5,ASTN2,FAM9B,KALRN,MKLN1,USH2A,NEGR1,FGF12,PCDHA12,NRXN1,GNGT1,DAAM2,STK24,EXOC4,BBS9,NTRK2,CHD7,NRG3,PTPRG,CALCR,NCOA1,ATP2B4,PHEX,SOX6,TMEM64,RAPGEF5,FGF13,GPC3,DMD,KCNQ3,CDH19,CHRM3,ARHGAP24,SLIT2,ZNF804A,FAM20C,KAZN,BRAF,B3GNT5,ERBB4,*

			KIF26B,RAD51B,DOP1B,MYO3B,EXT2,DIAPH2,MAP3K5,SPAG16,DSCAML1,HTR2C,CNTN4,ITGA1,TCF12,RUNX1,MYO16,PCDHA2,PCDHA11,PCLO,CDH18,FRMD6,ANK2,PCDHA9,ROBO1,PRRC2C,PCDHAC2,CDH6,CHODL,FGF14,PCDHA10,SOX5,DSCAM,COLEC10,LARGE1,SDK1,PUS7,EPHA6,IGF1R,WDR72,NLGN1,IQSEC1,CASZ1,CNTNAP2,TRIM71,CFTR,PCDH9,PCDHA1,ZNF800,PCDHA8,CDH12,SHC4,DDR2,ADAM22,TRPS1,CDH4,CELF4,ST8SIA6,CNTN6,SPEF2,MITF,PCSK1,PCDHAC1,HERC2,LRFN5,UTRN,AGBL4,OGDH,GHR,ULK4,RBFOX1,LRRC4C,PPP1R9A,SMAD9,CRPPA,NEBL,ZFPM2,MEIS1,EDAR,BRINP3,DNMT3A,TOX,PCDH15,SGCZ,PDE4D,CNTN5,ITGB8,PDE3A,RIMS1,WWOX,PCDHA7,LSAMP,DIAPH3,CPLANE1,PCDHA5,FTO,TNFSF11,PPFIA2,WNT2,POU6F2,NTNG1,SGCD,GRM7,NAV3,PTPRM,PRKCH,NRXN3,DLC1,ATP8A2,LPIN1,PLEKHA5,MACROD2,AUTS2,BCAS3,COMT,PCDHA6,KMT2E,SYNE2,DOCK2,SDCCAG8,SSH2,CDH13,MDGA2,FBN2,DAB1,PCDHA4,ALK,SEMA3A,YEATS2,SLC8A3,DCLK1,KIF16B,CDH2,TENM2,HDAC9,USP9X,USP13,CDK6,SRGAP2,TFAP2D,SPECC1,CSMD1,SCEL,FREM2,CLTC,DACH2,RNF220,AFF2,PTPRD,RORA,PLCB1,PRKG1,BRIP1,MAGI2,NELL1,SCN2A,ASIC2,DOCK10,RYR2,FAT3,MSH2,PCDHA3,RGS9,EYS,ROBO2,PAX3,BMPR1B,DCC,CTNNA2,DNER,ETV6,NRG1,PGM3,DNAH5
GO:0022008	neurogenesis	9.76221730487862e-19	*NFIA,DIP2A,ASTN2,KALRN,USH2A,NEGR1,NRXN1,GNGT1,DAAM2,STK24,NTRK2,CHD7,NRG3,PTPRG,NCOA1,SOX6,FGF13,DMD,KCNQ3,SLIT2,ZNF804A,BRAF,ERBB4,DSCAML1,CNTN4,ITGA1,TCF12,RUNX1,MYO16,ROBO1,PCDHAC2,CHODL,SOX5,DSCAM,LARGE1,SDK1,EPHA6,IGF1R,NLGN1,IQSEC1,CASZ1,CNTNAP2,DDR2,ADAM22,CDH4,CNTN6,PCSK1,AGBL4,OGDH,ULK4,LRRC4C,PPP1R9A,CRPPA,MEIS1,BRINP3,DNMT3A,TOX,PCDH15,CNTN5,RIMS1,DIAPH3,PPFIA2,WNT2,NTNG1,GRM7,NAV3,PTPRM,PRKCH,NRXN3,ATP8A2,AUTS2,SYNE2,SDCCAG8,MDGA2,DAB1,ALK,SEMA3A,SLC8A3,DCLK1,CDH2,TENM2,HDAC9,USP9X,CDK6,SRGAP2,RNF220,PTPRD,RORA,PRKG1,MAGI2,DOCK10,FAT3,ROBO2,BMPR1B,DCC,CTNNA2,DNER,ETV6*
GO:0048699	generation of neurons	2.483074796057173e-17	*NFIA,DIP2A,ASTN2,KALRN,USH2A,NEGR1,NRXN1,GNGT1,STK24,NTRK2,NRG3,PTPRG,NCOA1,FGF13,DMD,KCNQ3,SLIT2,ZNF804A,BRAF,ERBB4,DSCAML1,CNTN4,ITGA1,TCF12,RUNX1,MYO16,ROBO1,PCDHAC2,CHODL,SOX5,DSCAM,LARGE1,SDK1,EPHA6,IGF1R,NLGN1,IQSEC1,CASZ1,CNTNAP2,DDR2,CDH4,CNTN6,AGBL4,OGDH,ULK4,LRRC4C,PPP1R9A,CRPPA,MEIS1,BRINP3,DNMT3A,TOX,PCDH15,CNTN5,RIMS1,DIAPH3,PPFIA2,WNT2,NTNG1,GRM7,PTPRM,NRXN3,ATP8A2,AUTS2,SDCCAG8,MDGA2,DAB1,ALK,SEMA3A,DCLK1,CDH2,TENM2,HDAC9,USP9X,CDK6,SRGAP2,RNF220,PTPRD,RORA,PRKG1,MAGI2,DOCK10,FAT3,ROBO2,BMPR1B,DCC,CTNNA2,DNER*
GO:0030182	neuron differentiation	5.7824857249817755e-15	*NFIA,DIP2A,KALRN,USH2A,NEGR1,NRXN1,GNGT1,STK24,NTRK2,PTPRG,NCOA1,FGF13,DMD,KCNQ3,SLIT2,ZNF804A,BRAF,ERBB4,DSCAML1,CNTN4,ITGA1,TCF12,RUNX1,MYO16,ROBO1,PCDHAC2,CHODL,DSCAM,SDK1,EPHA6,IGF1R,NLGN1,IQSEC1,CASZ1,CNTNAP2,DDR2,CDH4,CNTN6,AGBL4,OGDH,ULK4,LRRC4C,PPP1R9A,CRPPA,MEIS1,BRINP3,DNMT3A,TOX,PCDH15,CNTN5,RIMS1,DIAPH3,PPFIA2,WNT2,NTNG1,GRM7,PTPRM,NRXN3,ATP8A2,AUTS2,MDGA2,DAB1,ALK,SEMA3A,DCLK1,CDH2,TENM2,HDAC9,*

			USP9X,SRGAP2,RNF220,PTPRD,RORA,PRKG1,MAGI2,DOCK10,FAT3,ROBO2,BMPR1B,DCC,CTNNA2
GO:0032501	multicellular organismal process	7.046360296695635e-15	*ABCC6,PTPRR,EPB41L4B,EPN2,PIR,NFIA,DIP2A,SLC8A1,PCDHA13,GRIK2,KDM4B,FGF1,ZBTB20,AHR,ASTN2,KALRN,USH2A,NEGR1,FGF12,BTBD9,PCDHA12,NRXN1,GNGT1,DAAM2,STK24,EXOC4,BBS9,NTRK2,CHD7,NBEA,NRG3,PTPRG,CALCR,NCOA1,ATP2B4,PHEX,SOX6,TMEM64,RAPGEF5,FGF13,GPC3,DMD,KCNQ3,CHRM3,ARHGAP24,SLIT2,ZNF804A,FAM20C,KAZN,BRAF,B3GNT5,ERBB4,KIF26B,RAD51B,DOP1B,MYO3B,EXT2,SPAG16,DSCAML1,HTR2C,CNTN4,HMCN1,ITGA1,TCF12,SGIP1,RUNX1,MYO16,ESRRG,PCDHA2,PCDHA11,PCLO,ANK2,PCDHA9,ROBO1,DGKB,PCDHAC2,CHODL,FGF14,PCDHA10,SOX5,DSCAM,DGKI,DENND1B,LARGE1,SDK1,EPHA6,IGF1R,NLGN1,IQSEC1,CASZ1,CNTNAP2,TRIM71,CFTR,NOS1AP,PCDH9,PCDHA1,ZNF800,PCDHA8,DDR2,ADAM22,TRPS1,CDH4,CELF4,ST8SIA6,CNTN6,SPEF2,MITF,CACNA2D1,PCSK1,PCDHAC1,LRFN5,UTRN,AGBL4,OGDH,GHR,ULK4,RBFOX1,LRRC4C,PPP1R9A,DYNC1H1,CRPPA,NEBL,ZFPM2,MEIS1,EDAR,BRINP3,DNMT3A,TOX,PCDH15,SGCZ,ACE2,PDE4D,CNTN5,ITGB8,PDE3A,RIMS1,WWOX,PCDHA7,NEK10,LSAMP,DIAPH3,CPLANE1,PCDHA5,RNF216,FTO,TNFSF11,PPFIA2,WNT2,OR8B8,POU6F2,NTNG1,SGCD,GRM7,NAV3,PTPRM,PRKCH,NRXN3,DLC1,ATP8A2,LPIN1,PLEKHA5,MACROD2,AUTS2,BCAS3,COMT,PCDHA6,KMT2E,SYNE2,DOCK2,SDCCAG8,CDH13,MDGA2,FBN2,DAB1,RFTN1,PCDHA4,ALK,SEMA3A,YEATS2,SLC8A3,MYO5B,DCLK1,KIF16B,CDH2,TENM2,HDAC9,USP9X,KCNB2,CDK6,SRGAP2,TFAP2D,SPECC1,CSMD1,SCEL,CTNNA3,FREM2,CLTC,DACH2,RNF220,AFF2,PTPRD,RORA,PLCB1,PRKG1,BRIP1,MAGI2,NELL1,PLCL1,SCN2A,ASIC2,DOCK10,RYR2,FAT3,MSH2,PCDHA3,RGS9,EYS,ROBO2,PAX3,KSR2,BMPR1B,DCC,CTNNA2,DNER,ETV6,NRG1,DNAH5*
GO:0007155	cell adhesion	3.7064696798303377e-14	*EPB41L4B,PCDHA13,ASTN2,MKLN1,NEGR1,PCDHA12,NRXN1,MAGI1,LMO7,FBLN7,CNTNAP5,DMD,CDH19,BRAF,KIF26B,DSCAML1,CNTN4,HMCN1,ITGA1,RUNX1,PCDHA2,PCDHA11,CDH18,PCDHA9,ROBO1,PCDHAC2,CDH6,PCDHA10,EDIL3,DSCAM,COL28A1,SDK1,NLGN1,CNTNAP2,PCDH9,PCDHA1,PCDHA8,CDH12,DDR2,ADAM22,CDH4,CNTN6,FBLN5,PCDHAC1,LRFN5,UTRN,PCDH7,LRRC4C,PCDH15,CNTN5,ITGB8,PCDHA7,LSAMP,PCDHA5,TNFSF11,PPFIA2,NTNG1,PTPRM,NRXN3,DLC1,PCDHA6,CDH13,DAB1,PCDHA4,CADM2,CDH2,TENM2,CDK6,SRGAP2,PTPRT,CTNNA3,FREM2,DLG2,PTPRD,PRKG1,PTPRA,FAT3,PCDHA3,ROBO2,DCC,CTNNA2,NRG1*
GO:0034330	cell junction organization	1.8648202471101176e-12	*NFIA,DIP2A,KALRN,NEGR1,NRXN1,NTRK2,NBEA,NRG3,FGF13,PATJ,CDH19,ZNF804A,ERBB4,GPHN,PCLO,CDH18,ANK2,DGKB,CDH6,DSCAM,LARGE1,SDK1,IGF1R,SPTB,NLGN1,IQSEC1,CNTNAP2,NOS1AP,CDH12,CDH4,LRFN5,LRRC4C,FRMPD4,ACE2,CNTN5,PPFIA2,NTNG1,PRKCH,NRXN3,DLC1,CDH13,SLC8A3,CDH2,SRGAP2,PTPRT,PTPRD,ASIC2,DOCK10,PTPRA,MPDZ,ROBO2,CTNNA2,DNER*
GO:0030154	cell differentiation	2.043309613521661e-12	*LRMDA,PIR,NFIA,DIP2A,SLC8A1,SLC22A14,FGF1,FSTL5,ASTN2,FAM9B,KALRN,USH2A,NEGR1,NRXN1,GNGT1,DAAM2,STK24,BBS9,NTRK2,CHD7,NRG3,PTPRG,CALCR,NCOA1,SOX6,TMEM64,FGF13,GPC3,DMD,KCNQ3,ARHGAP24,SLIT2,ZNF804A,FAM20C,KAZN,BRAF,ERBB4,EXT2,DIAPH2,MAP3K5,SPAG16,DSCAML1,HTR2C,CNTN4,ITGA1,TCF12,RUNX1,MYO*

			16,FRMD6,ANK2,ROBO1,PRRC2C,PCDHAC2,CHODL,SOX5,DSCAM,LARGE1,SDK1,PUS7,EPHA6,IGF1R,NLGN1,IQSEC1,CASZ1,CNTNAP2,CFTR,ZNF800,SHC4,DDR2,ADAM22,TRPS1,CDH4,CELF4,CNTN6,SPEF2,MITF,PCSK1,AGBL4,OGDH,GHR,ULK4,RBFOX1,LRRC4C,PPP1R9A,SMAD9,CRPPA,NEBL,ZFPM2,MEIS1,EDAR,BRINP3,DNMT3A,TOX,PCDH15,SGCZ,PDE4D,CNTN5,ITGB8,PDE3A,RIMS1,WWOX,DIAPH3,FTO,TNFSF11,PPFIA2,WNT2,POU6F2,NTNG1,SGCD,GRM7,NAV3,PTPRM,PRKCH,NRXN3,ATP8A2,AUTS2,KMT2E,SYNE2,DOCK2,SDCCAG8,SSH2,MDGA2,FBN2,DAB1,ALK,SEMA3A,SLC8A3,DCLK1,CDH2,TENM2,HDAC9,USP9X,USP13,CDK6,SRGAP2,SCEL,CLTC,RNF220,PTPRD,RORA,PLCB1,PRKG1,BRIP1,MAGI2,NELL1,DOCK10,FAT3,MSH2,ROBO2,BMPR1B,DCC,CTNNA2,DNER,ETV6,NRG1,PGM3
GO:0048869	cellular developmental process	2.0865730511378647e-12	*LRMDA,PIR,NFIA,DIP2A,SLC8A1,SLC22A14,FGF1,FSTL5,ASTN2,FAM9B,KALRN,USH2A,NEGR1,NRXN1,GNGT1,DAAM2,STK24,BBS9,NTRK2,CHD7,NRG3,PTPRG,CALCR,NCOA1,SOX6,TMEM64,FGF13,GPC3,DMD,KCNQ3,ARHGAP24,SLIT2,ZNF804A,FAM20C,KAZN,BRAF,ERBB4,EXT2,DIAPH2,MAP3K5,SPAG16,DSCAML1,HTR2C,CNTN4,ITGA1,TCF12,RUNX1,MYO16,FRMD6,ANK2,ROBO1,PRRC2C,PCDHAC2,CHODL,SOX5,DSCAM,LARGE1,SDK1,PUS7,EPHA6,IGF1R,NLGN1,IQSEC1,CASZ1,CNTNAP2,CFTR,ZNF800,SHC4,DDR2,ADAM22,TRPS1,CDH4,CELF4,CNTN6,SPEF2,MITF,PCSK1,AGBL4,OGDH,GHR,ULK4,RBFOX1,LRRC4C,PPP1R9A,SMAD9,CRPPA,NEBL,ZFPM2,MEIS1,EDAR,BRINP3,DNMT3A,TOX,PCDH15,SGCZ,PDE4D,CNTN5,ITGB8,PDE3A,RIMS1,WWOX,DIAPH3,FTO,TNFSF11,PPFIA2,WNT2,POU6F2,NTNG1,SGCD,GRM7,NAV3,PTPRM,PRKCH,NRXN3,ATP8A2,AUTS2,KMT2E,SYNE2,DOCK2,SDCCAG8,SSH2,MDGA2,FBN2,DAB1,ALK,SEMA3A,SLC8A3,DCLK1,CDH2,TENM2,HDAC9,USP9X,USP13,CDK6,SRGAP2,SCEL,CLTC,RNF220,PTPRD,RORA,PLCB1,PRKG1,BRIP1,MAGI2,NELL1,DOCK10,FAT3,MSH2,ROBO2,BMPR1B,DCC,CTNNA2,DNER,ETV6,NRG1,PGM3*
GO:0048666	neuron development	4.054138963946856e-12	*DIP2A,KALRN,NEGR1,NRXN1,GNGT1,STK24,NTRK2,PTPRG,FGF13,DMD,KCNQ3,SLIT2,ZNF804A,BRAF,DSCAML1,CNTN4,ITGA1,RUNX1,MYO16,ROBO1,PCDHAC2,CHODL,DSCAM,SDK1,EPHA6,IGF1R,NLGN1,IQSEC1,CNTNAP2,DDR2,CDH4,CNTN6,AGBL4,OGDH,ULK4,LRRC4C,PPP1R9A,CRPPA,TOX,PCDH15,CNTN5,RIMS1,PPFIA2,NTNG1,GRM7,PTPRM,NRXN3,ATP8A2,AUTS2,DAB1,ALK,SEMA3A,DCLK1,CDH2,TENM2,USP9X,SRGAP2,RNF220,PTPRD,PRKG1,MAGI2,DOCK10,FAT3,ROBO2,BMPR1B,DCC,CTNNA2*
GO:0098609	cell-cell adhesion	6.418698862048176e-12	*PCDHA13,ASTN2,NEGR1,PCDHA12,NRXN1,MAGI1,CDH19,KIF26B,DSCAML1,CNTN4,HMCN1,ITGA1,RUNX1,PCDHA2,PCDHA11,CDH18,PCDHA9,ROBO1,PCDHAC2,CDH6,PCDHA10,DSCAM,SDK1,NLGN1,PCDH9,PCDHA1,PCDHA8,CDH12,CDH4,CNTN6,PCDHAC1,LRFN5,PCDH7,LRRC4C,PCDH15,CNTN5,PCDHA7,PCDHA5,TNFSF11,NTNG1,PTPRM,NRXN3,PCDHA6,CDH13,DAB1,PCDHA4,CADM2,CDH2,TENM2,PTPRT,CTNNA3,DLG2,PTPRD,PRKG1,FAT3,PCDHA3,ROBO2,DCC,CTNNA2*
GO:0048468	cell development	1.0634759036358065e-11	*PIR,DIP2A,SLC8A1,SLC22A14,FAM9B,KALRN,NEGR1,NRXN1,GNGT1,DAAM2,STK24,NTRK2,CHD7,PTPRG,CALCR,TMEM64,FGF13,GPC3,DMD,KCNQ3,SLIT2,ZNF804A,FAM20C,BRAF,ERBB4,DIAPH2,SPAG16,DSCAML1,CNTN4,ITGA1,RUNX1,MYO16,FRMD6,AN*

			K2,ROBO1,PRRC2C,PCDHAC2,CHODL,DSCAM,LARGE1,SDK1,PUS7,EPHA6,IGF1R,NLGN1,IQSEC1,CNTNAP2,CFTR,DDR2,ADAM22,CDH4,CELF4,CNTN6,SPEF2,MITF,AGBL4,OGDH,ULK4,LRRC4C,PPP1R9A,CRPPA,NEBL,MEIS1,DNMT3A,TOX,PCDH15,SGCZ,PDE4D,CNTN5,ITGB8,PDE3A,RIMS1,DIAPH3,TNFSF11,PPFIA2,WNT2,NTNG1,SGCD,GRM7,PTPRM,PRKCH,NRXN3,ATP8A2,AUTS2,KMT2E,DOCK2,DAB1,ALK,SEMA3A,SLC8A3,DCLK1,CDH2,TENM2,HDAC9,USP9X,CDK6,SRGAP2,RNF220,PTPRD,RORA,PLCB1,PRKG1,BRIP1,MAGI2,DOCK10,FAT3,MSH2,ROBO2,BMPR1B,DCC,CTNNA2,DNER,ETV6,NRG1,PGM3
GO:0007417	central nervous system development	2.7728189776245463e-11	*KDM4B,NRXN1,DAAM2,NTRK2,CHD7,NRG3,NCOA1,ATP2B4,SOX6,FGF13,DMD,SLIT2,B3GNT5,ERBB4,DSCAML1,CNTN4,MYO16,ROBO1,LARGE1,IGF1R,CNTNAP2,PCDH9,ADAM22,CNTN6,SPEF2,PCSK1,AGBL4,OGDH,MEIS1,BRINP3,TOX,CNTN5,CPLANE1,WNT2,POU6F2,DLC1,MACROD2,COMT,SYNE2,MDGA2,DAB1,ALK,SEMA3A,SLC8A3,DCLK1,CDH2,CDK6,SRGAP2,TFAP2D,AFF2,RORA,PLCB1,PRKG1,SCN2A,ASIC2,ROBO2,BMPR1B,DCC,CTNNA2,DNER,DNAH5*
GO:0009653	anatomical structure morphogenesis	3.379256117903962e-11	*PAX7,EPN2,NFIA,DIP2A,FGF1,ARHGAP15,ASTN2,KALRN,MKLN1,NRXN1,GNGT1,EXOC4,NTRK2,CHD7,NRG3,NCOA1,ATP2B4,PHEX,SOX6,FGF13,GPC3,DMD,CDH19,ARHGAP24,SLIT2,FAM20C,BRAF,ERBB4,KIF26B,MYO3B,EXT2,DSCAML1,CNTN4,ITGA1,RUNX1,MYO16,CDH18,FRMD6,ANK2,ROBO1,PCDHAC2,CDH6,CHODL,SOX5,DSCAM,LARGE1,SDK1,EPHA6,IGF1R,WDR72,NLGN1,CNTNAP2,TRIM71,CFTR,CDH12,CDH4,CNTN6,SPEF2,GHR,LRRC4C,SMAD9,CRPPA,NEBL,ZFPM2,MEIS1,EDAR,PCDH15,CNTN5,ITGB8,RIMS1,WWOX,CPLANE1,TNFSF11,PPFIA2,WNT2,NTNG1,SGCD,PTPRM,NRXN3,DLC1,ATP8A2,AUTS2,BCAS3,COMT,DOCK2,SDCCAG8,CDH13,FBN2,DAB1,SEMA3A,DCLK1,KIF16B,CDH2,HDAC9,USP9X,SRGAP2,CSMD1,FREM2,PTPRD,RORA,PRKG1,DOCK10,RYR2,FAT3,ROBO2,PAX3,BMPR1B,DCC,CTNNA2,NRG1*
GO:0031175	neuron projection development	3.4722308436084725e-11	*DIP2A,KALRN,NEGR1,NRXN1,STK24,NTRK2,PTPRG,FGF13,DMD,SLIT2,ZNF804A,BRAF,DSCAML1,CNTN4,ITGA1,MYO16,ROBO1,PCDHAC2,CHODL,DSCAM,SDK1,EPHA6,IGF1R,NLGN1,IQSEC1,CNTNAP2,DDR2,CDH4,CNTN6,ULK4,LRRC4C,PPP1R9A,CRPPA,TOX,PCDH15,CNTN5,RIMS1,PPFIA2,NTNG1,GRM7,PTPRM,NRXN3,ATP8A2,AUTS2,DAB1,ALK,SEMA3A,DCLK1,CDH2,USP9X,SRGAP2,PTPRD,PRKG1,MAGI2,DOCK10,FAT3,ROBO2,BMPR1B,DCC,CTNNA2*
GO:0050808	synapse organization	1.777313663052832e-10	*NFIA,DIP2A,KALRN,NEGR1,NRXN1,NTRK2,NBEA,NRG3,FGF13,ZNF804A,ERBB4,GPHN,PCLO,DGKB,CDH6,DSCAM,LARGE1,SDK1,IGF1R,SPTB,NLGN1,NOS1AP,LRFN5,LRRC4C,FRMPD4,CNTN5,PPFIA2,NTNG1,NRXN3,SLC8A3,CDH2,SRGAP2,PTPRT,PTPRD,ASIC2,DOCK10,ROBO2,CTNNA2,DNER*
GO:0000902	cell morphogenesis	4.510103245058977e-10	*NFIA,DIP2A,ARHGAP15,KALRN,MKLN1,NRXN1,NTRK2,FGF13,DMD,CDH19,SLIT2,BRAF,DSCAML1,CNTN4,ITGA1,MYO16,CDH18,FRMD6,ROBO1,PCDHAC2,CDH6,CHODL,DSCAM,EPHA6,IGF1R,NLGN1,CNTNAP2,CDH12,CDH4,CNTN6,LRRC4C,CRPPA,PCDH15,CNTN5,RIMS1,PPFIA2,NTNG1,PTPRM,NRXN3,DLC1,ATP8A2,AUTS2,CDH13,DAB1,SEMA3A,DCLK1,CDH2,USP9X,SRGAP2,PTPRD,PRKG1,DOCK10,FAT3,ROBO2,BMPR1B,DCC,CTNNA2*
GO:0060322	head developme	5.235311819325515e-10	*KDM4B,NRXN1,NTRK2,CHD7,NRG3,NCOA1,ATP2B4,SOX6,FGF13,DMD,SLIT2,BRAF,ERBB4,DSCAML1,C*

	nt		*NTN4,MYO16,ROBO1,LARGE1,IGF1R,CNTNAP2,PCDH9,SPEF2,PCSK1,OGDH,MEIS1,TOX,CNTN5,DIAPH3,CPLANE1,WNT2,DLC1,MACROD2,COMT,SYNE2,DAB1,ALK,SEMA3A,DCLK1,CDH2,CDK6,SRGAP2,TFAP2D,AFF2,RORA,PLCB1,PRKG1,SCN2A,ROBO2,CTNNA2,DNAH5*
GO:0048812	neuron projection morphogenesis	5.296640486944385e-10	*DIP2A,KALRN,NRXN1,NTRK2,FGF13,DMD,SLIT2,BRAF,DSCAML1,CNTN4,ITGA1,MYO16,ROBO1,PCDHAC2,CHODL,DSCAM,EPHA6,IGF1R,NLGN1,CNTNAP2,CDH4,CNTN6,LRRC4C,CRPPA,CNTN5,RIMS1,PPFIA2,NTNG1,PTPRM,NRXN3,ATP8A2,AUTS2,DAB1,SEMA3A,DCLK1,CDH2,USP9X,SRGAP2,PTPRD,PRKG1,DOCK10,ROBO2,BMPR1B,DCC,CTNNA2*
GO:0007420	brain development	6.695899926551759e-10	*KDM4B,NRXN1,NTRK2,CHD7,NRG3,NCOA1,ATP2B4,SOX6,FGF13,DMD,SLIT2,ERBB4,DSCAML1,CNTN4,MYO16,ROBO1,LARGE1,IGF1R,CNTNAP2,PCDH9,SPEF2,PCSK1,OGDH,MEIS1,TOX,CNTN5,CPLANE1,WNT2,DLC1,MACROD2,COMT,SYNE2,DAB1,ALK,SEMA3A,DCLK1,CDH2,CDK6,SRGAP2,TFAP2D,AFF2,RORA,PLCB1,PRKG1,SCN2A,ROBO2,CTNNA2,DNAH5*
GO:0120036	plasma membrane bounded cell projection organization	9.139921852179782e-10	*DIP2A,KALRN,NEGR1,NRXN1,DAAM2,STK24,DNAH7,BBS9,NTRK2,PTPRG,DNAH8,FGF13,DMD,ARHGAP24,SLIT2,ZNF804A,BRAF,MYO3B,SPAG16,DSCAML1,CNTN4,ITGA1,MYO16,ROBO1,NEK1,PCDHAC2,CHODL,DSCAM,SDK1,EPHA6,IGF1R,NLGN1,IQSEC1,CNTNAP2,DDR2,CDH4,CNTN6,SPEF2,ULK4,LRRC4C,PPP1R9A,CRPPA,TOX,PCDH15,CNTN5,RIMS1,CPLANE1,PPFIA2,NTNG1,GRM7,PTPRM,NRXN3,ATP8A2,AUTS2,SYNE2,SDCCAG8,CDH13,DAB1,ALK,SEMA3A,DCLK1,CDH2,TENM2,USP9X,SRGAP2,PTPRD,PRKG1,MAGI2,DOCK10,FAT3,ROBO2,BMPR1B,DCC,CTNNA2,DNAH5*
GO:0120039	plasma membrane bounded cell projection morphogenesis	1.23054913078581e-9	*DIP2A,KALRN,NRXN1,NTRK2,FGF13,DMD,SLIT2,BRAF,DSCAML1,CNTN4,ITGA1,MYO16,ROBO1,PCDHAC2,CHODL,DSCAM,EPHA6,IGF1R,NLGN1,CNTNAP2,CDH4,CNTN6,LRRC4C,CRPPA,CNTN5,RIMS1,PPFIA2,NTNG1,PTPRM,NRXN3,ATP8A2,AUTS2,DAB1,SEMA3A,DCLK1,CDH2,USP9X,SRGAP2,PTPRD,PRKG1,DOCK10,ROBO2,BMPR1B,DCC,CTNNA2*
GO:0030030	cell projection organization	1.2342849212267481e-9	*DIP2A,KALRN,NEGR1,NRXN1,DAAM2,STK24,DNAH7,BBS9,NTRK2,PTPRG,DNAH8,FGF13,DMD,ARHGAP24,SLIT2,ZNF804A,BRAF,MYO3B,SPAG16,DSCAML1,CNTN4,ITGA1,MYO16,ROBO1,NEK1,PCDHAC2,CHODL,DSCAM,SDK1,EPHA6,IGF1R,NLGN1,IQSEC1,CNTNAP2,DDR2,CDH4,CNTN6,SPEF2,ULK4,LRRC4C,PPP1R9A,CRPPA,TOX,PCDH15,CNTN5,RIMS1,DIAPH3,CPLANE1,PPFIA2,NTNG1,GRM7,PTPRM,NRXN3,ATP8A2,AUTS2,SYNE2,SDCCAG8,CDH13,DAB1,ALK,SEMA3A,DCLK1,CDH2,TENM2,USP9X,SRGAP2,PTPRD,PRKG1,MAGI2,DOCK10,FAT3,ROBO2,BMPR1B,DCC,CTNNA2,DNAH5*
GO:0048858	cell projection morphogenesis	1.5922698540491346e-9	*DIP2A,KALRN,NRXN1,NTRK2,FGF13,DMD,SLIT2,BRAF,DSCAML1,CNTN4,ITGA1,MYO16,ROBO1,PCDHAC2,CHODL,DSCAM,EPHA6,IGF1R,NLGN1,CNTNAP2,CDH4,CNTN6,LRRC4C,CRPPA,CNTN5,RIMS1,PPFIA2,NTNG1,PTPRM,NRXN3,ATP8A2,AUTS2,DAB1,SEMA3A,DCLK1,CDH2,USP9X,SRGAP2,PTPRD,PRKG1,DOCK10,ROBO2,BMPR1B,DCC,CTNNA2*
GO:0048667	cell morphogenesis involved	6.2518340664192104e-9	*DIP2A,KALRN,NRXN1,NTRK2,FGF13,SLIT2,BRAF,DSCAML1,CNTN4,ROBO1,PCDHAC2,CHODL,DSCAM,EPHA6,IGF1R,NLGN1,CDH4,CNTN6,LRRC4C,CRPPA,PCDH15,CNTN5,PPFIA2,NTNG1,PTPRM,NRXN3,ATP*

	in neuron differentiation		*8A2,AUTS2,DAB1,SEMA3A,DCLK1,CDH2,USP9X,PTPRD,PRKG1,DOCK10,FAT3,ROBO2,BMPR1B,DCC,CTNNA2*
GO:0048513	animal organ development	2.075618198274932e-8	*PAX7,NFIA,SLC8A1,KDM4B,FGF1,KALRN,USH2A,FGF12,NRXN1,GNGT1,EXOC4,NTRK2,CHD7,NRG3,NCOA1,ATP2B4,PHEX,SOX6,FGF13,GPC3,DMD,SLIT2,FAM20C,KAZN,BRAF,ERBB4,KIF26B,MYO3B,EXT2,DSCAML1,CNTN4,TCF12,RUNX1,MYO16,ANK2,ROBO1,CHODL,SOX5,DSCAM,LARGE1,SDK1,IGF1R,WDR72,CNTNAP2,CFTR,PCDH9,ZNF800,DDR2,TRPS1,CELF4,SPEF2,MITF,PCSK1,UTRN,OGDH,GHR,RBFOX1,NEBL,ZFPM2,MEIS1,EDAR,TOX,PCDH15,SGCZ,CNTN5,ITGB8,WWOX,DIAPH3,CPLANE1,FTO,TNFSF11,WNT2,SGCD,PTPRM,PRKCH,DLC1,ATP8A2,LPIN1,MACROD2,COMT,SYNE2,FBN2,DAB1,ALK,SEMA3A,DCLK1,CDH2,HDAC9,CDK6,SRGAP2,TFAP2D,CSMD1,SCEL,FREM2,AFF2,RORA,PLCB1,PRKG1,BRIP1,MAGI2,NELL1,SCN2A,RYR2,FAT3,MSH2,ROBO2,PAX3,BMPR1B,CTNNA2,DNER,NRG1,DNAH5*
GO:0007409	axonogenesis	2.172266886610224e-8	*KALRN,NRXN1,NTRK2,FGF13,SLIT2,BRAF,DSCAML1,CNTN4,ROBO1,PCDHAC2,CHODL,DSCAM,EPHA6,IGF1R,CDH4,CNTN6,LRRC4C,CRPPA,CNTN5,NTNG1,PTPRM,NRXN3,ATP8A2,AUTS2,DAB1,SEMA3A,DCLK1,CDH2,USP9X,PTPRD,PRKG1,ROBO2,BMPR1B,DCC,CTNNA2*
GO:0034329	cell junction assembly	3.905262534645343e-8	*NEGR1,NRXN1,NTRK2,NRG3,FGF13,PATJ,CDH19,ERBB4,PCLO,CDH18,ANK2,CDH6,DSCAM,LARGE1,SDK1,NLGN1,CNTNAP2,CDH12,CDH4,LRFN5,ACE2,CNTN5,PRKCH,NRXN3,DLC1,CDH13,CDH2,SRGAP2,PTPRD,ASIC2,PTPRA,MPDZ,ROBO2,DNER*
GO:0061564	axon development	4.204654493653484e-8	*KALRN,NRXN1,STK24,NTRK2,FGF13,SLIT2,BRAF,DSCAML1,CNTN4,ROBO1,PCDHAC2,CHODL,DSCAM,EPHA6,IGF1R,CDH4,CNTN6,LRRC4C,CRPPA,CNTN5,NTNG1,GRM7,PTPRM,NRXN3,ATP8A2,AUTS2,DAB1,SEMA3A,DCLK1,CDH2,USP9X,PTPRD,PRKG1,ROBO2,BMPR1B,DCC,CTNNA2*
GO:0010975	regulation of neuron projection development	6.700309337230185e-8	*KALRN,NEGR1,NRXN1,STK24,NTRK2,PTPRG,FGF13,DMD,SLIT2,ZNF804A,BRAF,ROBO1,CHODL,DSCAM,IGF1R,NLGN1,DDR2,CDH4,ULK4,LRRC4C,TOX,PPFIA2,NTNG1,ATP8A2,DAB1,ALK,SEMA3A,CDH2,PTPRD,MAGI2,FAT3,ROBO2,DCC,CTNNA2*
GO:0120035	regulation of plasma membrane bounded cell projection organization	1.2622864491024714e-7	*KALRN,NEGR1,NRXN1,DAAM2,STK24,NTRK2,PTPRG,FGF13,DMD,ARHGAP24,SLIT2,ZNF804A,BRAF,MYO3B,ROBO1,CHODL,DSCAM,IGF1R,NLGN1,DDR2,CDH4,ULK4,LRRC4C,TOX,PPFIA2,NTNG1,ATP8A2,AUTS2,SYNE2,SDCCAG8,DAB1,ALK,SEMA3A,CDH2,TENM2,PTPRD,MAGI2,FAT3,ROBO2,DCC,CTNNA2*
GO:0098916	anterograde trans-synaptic signaling	2.2320284349813187e-7	*GRIK2,CADPS2,FGF12,BTBD9,NRXN1,GRM3,EXOC4,NTRK2,NRG3,DMD,KCNQ3,CHRM3,BRAF,HTR2C,CNTN4,PCLO,DGKB,PTPRN2,DGKI,LARGE1,NLGN1,CELF4,LRRC4C,PPP1R9A,RIMBP2,RIMS1,PLCB4,PPFIA2,NTNG1,GRM7,NRXN3,COMT,MCTP1,SLC8A3,CDH2,CADPS,DLG2,PTPRD,PLCB1,PLCL1,ASIC2,PTPRA,DLGAP2,DCC*
GO:0007268	chemical synaptic	2.2320284349813187e-7	*GRIK2,CADPS2,FGF12,BTBD9,NRXN1,GRM3,EXOC4,NTRK2,NRG3,DMD,KCNQ3,CHRM3,BRAF,HTR2C,CN*

	transmission		TN4,PCLO,DGKB,PTPRN2,DGKI,LARGE1,NLGN1,CELF4,LRRC4C,PPP1R9A,RIMBP2,RIMS1,PLCB4,PPFIA2,NTNG1,GRM7,NRXN3,COMT,MCTP1,SLC8A3,CDH2,CADPS,DLG2,PTPRD,PLCB1,PLCL1,ASIC2,PTPRA,DLGAP2,DCC
GO:0099536	synaptic signaling	2.3128894876999869e-7	GRIK2,CADPS2,FGF12,BTBD9,NRXN1,GRM3,EXOC4,NTRK2,NRG3,DMD,KCNQ3,CHRM3,BRAF,HTR2C,CNTN4,PCLO,DGKB,PTPRN2,DGKI,LARGE1,NLGN1,CELF4,UTRN,LRRC4C,PPP1R9A,RIMBP2,RIMS1,PLCB4,PPFIA2,NTNG1,GRM7,NRXN3,COMT,MCTP1,SLC8A3,CDH2,CADPS,DLG2,PTPRD,PLCB1,PLCL1,ASIC2,PTPRA,DLGAP2,DCC
GO:0031344	regulation of cell projection organization	2.635342358429765e-7	KALRN,NEGR1,NRXN1,DAAM2,STK24,NTRK2,PTPRG,FGF13,DMD,ARHGAP24,SLIT2,ZNF804A,BRAF,MYO3B,ROBO1,CHODL,DSCAM,IGF1R,NLGN1,DDR2,CDH4,ULK4,LRRC4C,TOX,PPFIA2,NTNG1,ATP8A2,AUTS2,SYNE2,SDCCAG8,DAB1,ALK,SEMA3A,CDH2,TENM2,PTPRD,MAGI2,FAT3,ROBO2,DCC,CTNNA2
GO:0099537	trans-synaptic signaling	2.8748020549404666e-7	GRIK2,CADPS2,FGF12,BTBD9,NRXN1,GRM3,EXOC4,NTRK2,NRG3,DMD,KCNQ3,CHRM3,BRAF,HTR2C,CNTN4,PCLO,DGKB,PTPRN2,DGKI,LARGE1,NLGN1,CELF4,LRRC4C,PPP1R9A,RIMBP2,RIMS1,PLCB4,PPFIA2,NTNG1,GRM7,NRXN3,COMT,MCTP1,SLC8A3,CDH2,CADPS,DLG2,PTPRD,PLCB1,PLCL1,ASIC2,PTPRA,DLGAP2,DCC
GO:0030900	forebrain development	4.933882347043253e-7	NTRK2,CHD7,NRG3,NCOA1,ATP2B4,FGF13,DMD,SLIT2,ERBB4,ROBO1,LARGE1,IGF1R,CNTNAP2,PCDH9,PCSK1,OGDH,TOX,DLC1,SYNE2,DAB1,ALK,SEMA3A,DCLK1,CDH2,CDK6,SRGAP2,PLCB1,PRKG1,SCN2A,ROBO2,DNAH5
GO:0021537	telencephalon development	6.08089767481867e-7	NTRK2,CHD7,NRG3,NCOA1,ATP2B4,FGF13,DMD,SLIT2,ERBB4,ROBO1,LARGE1,IGF1R,CNTNAP2,OGDH,SYNE2,DAB1,ALK,SEMA3A,CDH2,CDK6,SRGAP2,PLCB1,SCN2A,ROBO2,DNAH5
GO:0007154	cell communication	6.680067793944816e-7	PTPRR,EPN2,NFIA,SLC8A1,ZNF385B,GRIK2,FGF1,ARHGAP15,AHR,FSTL5,CADPS2,TLK1,KALRN,MKLN1,FGF12,BTBD9,MAST4,NRXN1,MAGI1,GNGT1,FAM13A,DAAM2,STK24,GRM3,EXOC4,NTRK2,CHD7,RASGEF1B,NRG3,PTPRG,CALCR,ELMO1,NCOA1,IRAG1,ATP2B4,PHEX,TMEM64,RAPGEF5,FGF13,PATJ,GPC3,DMD,ADCY2,KCNQ3,ATRNL1,CHRM3,ARHGAP24,SLIT2,FAM20C,BRAF,BABAM2,ERBB4,SH2D1A,SH3RF3,MAP3K5,HTR2C,CNTN4,RAP1GAP2,PIK3C3,ITGA1,ENSG00000289694,MYO16,ESRRG,PCLO,FRMD6,ANK2,ROBO1,DGKB,CDH6,DOK6,FGF14,PTPRN2,INPP4B,DSCAM,DGKI,DENND1B,COLEC10,LARGE1,EPHA6,IGF1R,NLGN1,IQSEC1,ANK1,PPM1L,CNTNAP2,TRIM71,CFTR,NOS1AP,RBMS3,TBCK,SHC4,DDR2,GPC6,CELF4,CNTN6,MITF,CACNA2D1,PCSK1,UTRN,GPC5,GHR,ULK4,LRRC4C,PPP1R9A,TMEM117,SMAD9,LANCL2,EDAR,RIMBP2,FHIT,ACE2,PDE4D,ITGB8,PDE3A,RIMS1,WWOX,NEK10,DIAPH3,PLCB4,TNFSF11,PPFIA2,WNT2,OR8B8,NTNG1,SGCD,GRM7,PTPRM,PRKCH,NRXN3,DLC1,DENND2B,AUTS2,BCAS3,COMT,MCTP1,DOCK2,CDH13,FBN2,DAB1,RFTN1,SNTG1,ALK,SEMA3A,SLC8A3,DCLK1,KIF16B,CDH2,TENM2,USP9X,CDK6,ADGRL4,CADPS,DDX60,RGS7,SRGAP2,PTPRT,SCEL,CTNNA3,FREM2,RAPGEF4,SORCS1,CAMKMT,RNF220,DLG2,PTPRD,RORA,PLCB1,PRKG1,BRIP1,MAGI2,PLCL1,SCN2A,ASIC2,DOCK10,RYR2,PTPRA,MSH2,KLHDC10,RGS9,ROBO2,KSR2,DLGAP2,BMPR1B,DCC,PEX5L,DNER,NRG1
GO:0035	intracell	0.000001551	PTPRR,SLC8A1,ZNF385B,GRIK2,FGF1,ARHGAP15,

556	ular signal transduction	3156234070727	*AHR,TLK1,KALRN,FGF12,MAST4,NRXN1,FAM13A,STK24,NTRK2,RASGEF1B,NRG3,CALCR,ELMO1,NCOA1,IRAG1,ATP2B4,RAPGEF5,FGF13,PATJ,DMD,ADCY2,CHRM3,ARHGAP24,SLIT2,BRAF,BABAM2,ERBB4,SH3RF3,MAP3K5,HTR2C,RAP1GAP2,PIK3C3,ITGA1,ENSG00000289694,MYO16,ESRRG,FRMD6,ANK2,ROBO1,DGKB,DOK6,FGF14,DGKI,IGF1R,NLGN1,IQSEC1,PPM1L,NOS1AP,TBCK,SHC4,DDR2,GHR,ULK4,PPP1R9A,TMEM117,SMAD9,EDAR,FHIT,ACE2,PDE4D,PDE3A,WWOX,NEK10,PLCB4,TNFSF11,SGCD,PRKCH,DLC1,DENND2B,AUTS2,MCTP1,DOCK2,CDH13,DAB1,RFTN1,SEMA3A,DCLK1,CDH2,USP9X,DDX60,RGS7,SRGAP2,RAPGEF4,RORA,PLCB1,PRKG1,BRIP1,MAGI2,PLCL1,SCN2A,DOCK10,RYR2,MSH2,KLHDC10,RGS9,KSR2,PEX5L,NRG1*
GO:0007267	cell-cell signaling	0.00000168629754847865 46	*GRIK2,CADPS2,KALRN,FGF12,BTBD9,NRXN1,GRM3,EXOC4,NTRK2,CHD7,NRG3,PHEX,FGF13,DMD,KCNQ3,CHRM3,BRAF,SH2D1A,HTR2C,CNTN4,PCLO,ANK2,DGKB,FGF14,PTPRN2,DGKI,LARGE1,NLGN1,CFTR,CELF4,CACNA2D1,PCSK1,UTRN,LRRC4C,PPP1R9A,RIMBP2,RIMS1,PLCB4,TNFSF11,PPFIA2,WNT2,NTNG1,GRM7,NRXN3,BCAS3,COMT,MCTP1,SLC8A3,CDH2,CADPS,RAPGEF4,DLG2,PTPRD,PLCB1,PLCL1,ASIC2,RYR2,PTPRA,DLGAP2,DCC*
GO:0023052	signaling	0.00000317090288478889 93	*PTPRR,EPN2,NFIA,SLC8A1,ZNF385B,GRIK2,FGF1,ARHGAP15,AHR,FSTL5,CADPS2,TLK1,KALRN,MKLN1,FGF12,BTBD9,MAST4,NRXN1,MAGI1,GNGT1,FAM13A,DAAM2,STK24,LMO7,GRM3,EXOC4,NTRK2,CHD7,RASGEF1B,NRG3,PTPRG,CALCR,ELMO1,NCOA1,IRAG1,ATP2B4,PHEX,TMEM64,RAPGEF5,FGF13,PATJ,GPC3,DMD,ADCY2,KCNQ3,ATRNL1,CHRM3,ARHGAP24,SLIT2,FAM20C,BRAF,BABAM2,ERBB4,SH2D1A,SH3RF3,MAP3K5,HTR2C,CNTN4,RAP1GAP2,PIK3C3,ITGA1,ENSG00000289694,MYO16,ESRRG,PCLO,FRMD6,ANK2,ROBO1,DGKB,CDH6,DOK6,FGF14,PTPRN2,INPP4B,DSCAM,DGKI,DENND1B,COLEC10,LARGE1,EPHA6,IGF1R,NLGN1,IQSEC1,ANK1,PPM1L,TRIM71,CFTR,NOS1AP,RBMS3,TBCK,SHC4,DDR2,GPC6,CELF4,CNTN6,MITF,CACNA2D1,PCSK1,UTRN,GPC5,GHR,ULK4,LRRC4C,PPP1R9A,TMEM117,SMAD9,LANCL2,EDAR,RIMBP2,FHIT,ACE2,PDE4D,ITGB8,PDE3A,RIMS1,WWOX,NEK10,DIAPH3,PLCB4,TNFSF11,PPFIA2,WNT2,OR8B8,NTNG1,SGCD,GRM7,PTPRM,PRKCH,NRXN3,DLC1,DENND2B,AUTS2,BCAS3,COMT,MCTP1,DOCK2,CDH13,FBN2,DAB1,RFTN1,ALK,SEMA3A,SLC8A3,DCLK1,KIF16B,CDH2,TENM2,USP9X,CDK6,ADGRL4,CADPS,DDX60,RGS7,SRGAP2,PTPRT,SCEL,RAPGEF4,SORCS1,CAMKMT,RNF220,DLG2,PTPRD,RORA,PLCB1,PRKG1,BRIP1,MAGI2,PLCL1,SCN2A,ASIC2,DOCK10,RYR2,PTPRA,MSH2,KLHDC10,RGS9,ROBO2,KSR2,DLGAP2,BMPR1B,DCC,PEX5L,DNER,NRG1*
GO:0007416	synapse assembly	0.00000618120875898774 2	*NEGR1,NRXN1,NTRK2,NRG3,FGF13,ERBB4,PCLO,DSCAM,LARGE1,SDK1,NLGN1,LRFN5,CNTN5,NRXN3,CDH2,SRGAP2,PTPRD,ASIC2,ROBO2,DNER*
GO:0001764	neuron migration	0.00000691664279734669 7	*ASTN2,NTRK2,NRG3,FGF13,LARGE1,ULK4,NTNG1,AUTS2,SDCCAG8,DAB1,SEMA3A,DCLK1,USP9X,SRGAP2,PRKG1,FAT3,DCC,CTNNA2,DNER*
GO:0141124	intracellular signaling cassette	0.00001351111734747072 1	*PTPRR,SLC8A1,GRIK2,FGF1,ARHGAP15,AHR,KALRN,FGF12,NRXN1,FAM13A,NTRK2,RASGEF1B,CALCR,ELMO1,IRAG1,ATP2B4,RAPGEF5,FGF13,DMD,ADCY2,CHRM3,ARHGAP24,SLIT2,BRAF,ERBB4,SH3RF3,MAP3K5,HTR2C,RAP1GAP2,ITGA1,ENSG00000289*

			694,MYO16,ANK2,ROBO1,DOK6,FGF14,DGKI,IGF1R,IQSEC1,PPM1L,NOS1AP,DDR2,GHR,ULK4,PPP1R9A,SMAD9,EDAR,ACE2,PDE4D,PDE3A,NEK10,TNFSF11,SGCD,DLC1,DENND2B,AUTS2,MCTP1,DOCK2,CDH13,DAB1,SEMA3A,CDH2,SRGAP2,RAPGEF4,RORA,PLCB1,PRKG1,MAGI2,DOCK10,RYR2,KLHDC10,KSR2,PEX5L,NRG1
GO:0021543	pallium development	0.000019972245130799405	*NTRK2,NCOA1,ATP2B4,FGF13,DMD,SLIT2,ROBO1,LARGE1,IGF1R,CNTNAP2,OGDH,SYNE2,DAB1,ALK,CDH2,CDK6,SRGAP2,PLCB1,SCN2A*
GO:0051128	regulation of cellular component organization	0.00003487888532075704	*RNF103-CHMP3,TLK1,KALRN,MKLN1,NEGR1,BTBD9,NRXN1,DAAM2,STK24,NTRK2,NRG3,PTPRG,PDE4DIP,FGF13,PATJ,GPC3,DMD,ARHGAP24,SLIT2,ZNF804A,BRAF,MYO3B,TBC1D5,SGIP1,RUNX1,ROBO1,DGKB,CHODL,DSCAM,COLEC10,IGF1R,SPTB,NLGN1,IQSEC1,ANK1,CNTNAP2,DDR2,CDH4,NSMCE2,LRFN5,ULK4,LRRC4C,DYNC1H1,FRMPD4,TOX,ACE2,CHMP3,PDE3A,RIMS1,DIAPH3,PPFIA2,NTNG1,NAV3,PRKCH,DLC1,ATP8A2,AUTS2,BCAS3,MCTP1,SYNE2,DOCK2,SDCCAG8,SSH2,CDH13,DAB1,ALK,SEMA3A,TENT4B,CDH2,TENM2,VPS41,PTPRT,CLTC,SACS,PTPRD,PLCB1,MAGI2,ASIC2,PTPRA,FAT3,MPDZ,SLF1,ROBO2,DCC,CTNNA2,NRG1*
GO:0010646	regulation of cell communication	0.00003660443809853938	*PTPRR,EPN2,SLC8A1,GRIK2,FGF1,ARHGAP15,FSTL5,KALRN,FGF12,BTBD9,NRXN1,FAM13A,DAAM2,GRM3,NTRK2,CHD7,NRG3,CALCR,NCOA1,ATP2B4,TMEM64,GPC3,DMD,KCNQ3,CHRM3,ARHGAP24,SLIT2,FAM20C,BRAF,BABAM2,ERBB4,SH2D1A,SH3RF3,MAP3K5,HTR2C,CNTN4,RAP1GAP2,ITGA1,FRMD6,ANK2,ROBO1,DGKB,DOK6,DGKI,LARGE1,IGF1R,NLGN1,IQSEC1,CFTR,NOS1AP,RBMS3,TBCK,DDR2,GPC6,CELF4,CNTN6,GPC5,GHR,ULK4,LRRC4C,PPP1R9A,LANCL2,EDAR,ACE2,PDE4D,PDE3A,RIMS1,WWOX,NEK10,PLCB4,TNFSF11,PPFIA2,NTNG1,GRM7,PRKCH,NRXN3,DLC1,DENND2B,AUTS2,MCTP1,DOCK2,CDH13,FBN2,DAB1,ALK,SEMA3A,SLC8A3,CDH2,USP9X,DDX60,RGS7,SRGAP2,PTPRT,SCEL,RAPGEF4,CAMKMT,RNF220,PTPRD,RORA,PLCB1,MAGI2,PLCL1,RYR2,PTPRA,KLHDC10,RGS9,ROBO2,KSR2,BMPR1B,DCC,PEX5L,NRG1*
GO:0048870	cell motility	0.00008085098163600824	*PTPRR,EPB41L4B,SLC22A14,FGF1,ASTN2,DAAM2,STK24,DNAH7,NTRK2,NRG3,PTPRG,ELMO1,ATP2B4,DNAH8,FGF13,GPC3,ARHGAP24,SLIT2,BRAF,ERBB4,SPAG16,ITGA1,ROBO1,LARGE1,IGF1R,IQSEC1,DDR2,GPC6,SPEF2,MITF,GPC5,OGDH,ULK4,TNFSF11,NTNG1,NAV3,PTPRM,DLC1,AUTS2,BCAS3,MCTP1,SYNE2,DOCK2,SDCCAG8,SSH2,CDH13,DAB1,TTLL6,SEMA3A,DCLK1,CDH2,HDAC9,USP9X,CDK6,LYST,SRGAP2,PTPRT,CTNNA3,PLCB1,PRKG1,MAGI2,DOCK10,FAT3,DCC,CTNNA2,DNER,DNAH5*
GO:0050804	modulation of chemical synaptic transmission	0.00008491627597442192	*GRIK2,BTBD9,NRXN1,GRM3,NTRK2,NRG3,KCNQ3,BRAF,CNTN4,DGKB,DGKI,LARGE1,NLGN1,CELF4,LRRC4C,PPP1R9A,RIMS1,PLCB4,PPFIA2,NTNG1,GRM7,NRXN3,MCTP1,SLC8A3,CDH2,PTPRD,PLCB1,PLCL1,PTPRA,DCC*
GO:0007610	behavior	0.00008600975925853068	*GRIK2,KALRN,NEGR1,FGF12,BTBD9,NRXN1,NTRK2,CHD7,NCOA1,FGF13,DMD,KCNQ3,BRAF,HTR2C,SGIP1,DSCAM,DGKI,LARGE1,SDK1,NLGN1,CNTNAP2,ADAM22,MEIS1,PCDH15,ACE2,NRXN3,ATP8A2,COMT,DAB1,ALK,SLC8A3,SPECC1,CSMD1,AFF2,PLCB1,SCN2A*

GO:0099177	regulation of trans-synaptic signaling	0.00008883382133495141	*GRIK2,BTBD9,NRXN1,GRM3,NTRK2,NRG3,KCNQ3,BRAF,CNTN4,DGKB,DGKI,LARGE1,NLGN1,CELF4,LRRC4C,PPP1R9A,RIMS1,PLCB4,PPFIA2,NTNG1,GRM7,NRXN3,MCTP1,SLC8A3,CDH2,PTPRD,PLCB1,PLCL1,PTPRA,DCC*
GO:0031346	positive regulation of cell projection organization	0.00009123059907930074	*KALRN,NEGR1,NRXN1,STK24,NTRK2,DMD,SLIT2,ZNF804A,BRAF,MYO3B,ROBO1,CHODL,DSCAM,IGF1R,NLGN1,DDR2,CDH4,TOX,ATP8A2,AUTS2,ALK,TENM2,PTPRD,MAGI2,ROBO2*
GO:0023051	regulation of signaling	0.00012300008549098247	*PTPRR,EPN2,GRIK2,FGF1,ARHGAP15,FSTL5,KALRN,BTBD9,NRXN1,FAM13A,DAAM2,LMO7,GRM3,NTRK2,CHD7,NRG3,CALCR,NCOA1,ATP2B4,TMEM64,GPC3,DMD,KCNQ3,CHRM3,ARHGAP24,SLIT2,FAM20C,BRAF,BABAM2,ERBB4,SH2D1A,SH3RF3,MAP3K5,HTR2C,CNTN4,RAP1GAP2,ITGA1,FRMD6,ROBO1,DGKB,DOK6,DGKI,LARGE1,IGF1R,NLGN1,IQSEC1,CFTR,NOS1AP,RBMS3,TBCK,DDR2,GPC6,CELF4,CNTN6,GPC5,GHR,ULK4,LRRC4C,PPP1R9A,LANCL2,EDAR,ACE2,PDE4D,PDE3A,RIMS1,WWOX,NEK10,PLCB4,TNFSF11,PPFIA2,NTNG1,GRM7,PRKCH,NRXN3,DLC1,DENND2B,AUTS2,MCTP1,DOCK2,CDH13,FBN2,DAB1,ALK,SEMA3A,SLC8A3,KIF16B,CDH2,USP9X,DDX60,RGS7,SRGAP2,PTPRT,SCEL,RAPGEF4,CAMKMT,RNF220,PTPRD,RORA,PLCB1,MAGI2,PLCL1,PTPRA,KLHDC10,RGS9,ROBO2,KSR2,BMPR1B,DCC,PEX5L,NRG1*
GO:0048522	positive regulation of cellular process	0.00014023259386819137	*EPB41L4B,EPN2,NFIA,DIP2A,GRIK2,FGF1,ZBTB20,AHR,CADPS2,KALRN,NEGR1,NRXN1,MAGI1,DAAM2,STK24,LMO7,NTRK2,CHD7,CALCR,NCOA1,ATP2B4,TOX2,SOX6,PDE4DIP,TMEM64,FGF13,GPC3,DMD,CHRM3,SLIT2,ZNF804A,FAM20C,BRAF,BABAM2,KMT2C,ERBB4,KIF26B,RAD51B,SH2D1A,MYO3B,SH3RF3,MAP3K5,HTR2C,TBC1D5,ITGA1,TCF12,SGIP1,RUNX1,ESRRG,FRMD6,ANK2,SUPT3H,ROBO1,DOK6,CHODL,ZNF267,EDIL3,SOX5,DSCAM,DGKI,DENND1B,COLEC10,LARGE1,IGF1R,NLGN1,IQSEC1,ANK1,CASZ1,CNTNAP2,TRIM71,CFTR,NOS1AP,RBMS3,SHC4,DDR2,ADAM22,CDH4,DPP10,CELF4,CNTN6,MITF,CACNA2D1,NSMCE2,PCSK1,UTRN,GPC5,AGBL4,GHR,SMAD9,DYNC1H1,LANCL2,ZFPM2,FRMPD4,MEIS1,EDAR,BRINP3,DNMT3A,TOX,ACE2,CHMP3,PDE4D,ITGB8,PDE3A,RIMS1,WWOX,NEK10,FTO,TNFSF11,WNT2,NAV3,PRKCH,DLC1,DENND2B,ATP8A2,LPIN1,AUTS2,BCAS3,NBAS,KMT2E,SYNE2,DOCK2,SSH2,CDH13,FBN2,DAB1,TTLL6,RFTN1,ALK,SEMA3A,SLC8A3,TENT4B,CDH2,TENM2,HDAC9,USP9X,USP13,CDK6,CADPS,DDX60,RGS7,TFAP2D,GABPB1,SCEL,RAPGEF4,RNF220,PTPRD,RORA,PLCB1,NPEPPS,MAGI2,NELL1,ASIC2,TMEM164,RYR2,MSH2,KLHDC10,SLF1,ROBO2,PAX3,KSR2,BMPR1B,ETV6,NRG1,NPAS3,FANCB*
GO:0030029	actin filament-based process	0.0001563745678255539	*EPB41L4B,MKLN1,FGF12,DAAM2,ELMO1,FGF13,SLIT2,BRAF,MYO3B,DIAPH2,HMCN1,FRMD6,ANK2,SPTB,IQSEC1,NOS1AP,TBCK,CACNA2D1,PPP1R9A,NEBL,FRMPD4,PCDH15,PDE4D,DIAPH3,SGCD,DLC1,AUTS2,BCAS3,SYNE2,DOCK2,SSH2,MYO5B,SRGAP2,SPECC1,CTNNA3,THSD7B,PRKG1,MICAL3,RYR2,CTNNA2*
GO:0097485	neuron projectio	0.00020148079786303687	*KALRN,NRXN1,SLIT2,DSCAML1,CNTN4,ROBO1,PCDHAC2,DSCAM,EPHA6,CDH4,CNTN6,CRPPA,CNTN5,P*

	n guidance		*TPRM,NRXN3,SEMA3A,PTPRD,ROBO2,BMPR1B,DCC*
GO:0007411	axon guidance	0.00020148079786303687	*KALRN,NRXN1,SLIT2,DSCAML1,CNTN4,ROBO1,PCDHAC2,DSCAM,EPHA6,CDH4,CNTN6,CRPPA,CNTN5,PTPRM,NRXN3,SEMA3A,PTPRD,ROBO2,BMPR1B,DCC*
GO:0009887	animal organ morphogenesis	0.00042666436636378094	*FGF1,GNGT1,EXOC4,NTRK2,CHD7,NRG3,PHEX,SOX6,GPC3,SLIT2,FAM20C,BRAF,ERBB4,KIF26B,MYO3B,EXT2,DSCAML1,ROBO1,SOX5,DSCAM,LARGE1,SDK1,WDR72,CFTR,SPEF2,GHR,ZFPM2,MEIS1,EDAR,PCDH15,WWOX,TNFSF11,WNT2,PTPRM,DLC1,ATP8A2,FBN2,CDH2,CSMD1,RYR2,FAT3,ROBO2,PAX3,BMPR1B,CTNNA2,NRG1*
GO:0050789	regulation of biological process	0.0005207303384060373	*PAX7,PTPRR,EPB41L4B,EPN2,MED13L,NFIA,DIP2A,SLC8A1,RNF103-CHMP3,ZNF385B,GRIK2,KDM4B,FGF1,ZBTB20,ARHGAP15,AHR,FSTL5,CADPS2,TLK1,ASTN2,KALRN,MKLN1,USH2A,NEGR1,FGF12,BTBD9,MAST4,NRXN1,MAGI1,GNGT1,DPP6,FAM13A,DAAM2,STK24,LMO7,GRM3,EXOC4,NTRK2,CHD7,NBEA,RASGEF1B,MIR99AHG,NRG3,PTPRG,CALCR,ELMO1,NCOA1,IRAG1,ATP2B4,TOX2,PHEX,SOX6,PDE4DIP,TMEM64,RAPGEF5,FGF13,PATJ,GPC3,DMD,ADCY2,KCNQ3,ATRNL1,CHRM3,ARHGAP24,SLIT2,ZNF804A,FAM20C,BRAF,BABAM2,KMT2C,ERBB4,KIF26B,RAD51B,SH2D1A,MYO3B,SH3RF3,MAP3K5,HTR2C,TBC1D5,CNTN4,RAP1GAP2,PIK3C3,ITGA1,TCF12,SGIP1,ENSG00000289694,RUNX1,MYO16,ESRRG,PCLO,FRMD6,ANK2,SUPT3H,ROBO1,DGKB,EBF1,CDH6,DOK6,JAZF1,CHODL,ZNF267,FGF14,EDIL3,PTPRN2,INPP4B,SOX5,DSCAM,DGKI,LINC01151,DENND1B,COLEC10,LARGE1,SDK1,PUS7,EPHA6,IGF1R,SPTB,NLGN1,IQSEC1,ANK1,PPM1L,CASZ1,CNTNAP2,TRIM71,KCNIP4,CFTR,NOS1AP,NKAIN2,RBMS3,ATP6V0D2,ZNF800,TBCK,SHC4,DDR2,ADAM22,HULC,GPC6,MCF2L2,TRPS1,CDH4,DPP10,CELF4,CNTN6,FBLN5,ZNF638,MITF,CACNA2D1,NSMCE2,PCSK1,LRFN5,UTRN,GPC5,AGBL4,GHR,ULK4,RBFOX1,LRRC4C,PPP1R9A,TMEM117,SMAD9,NKAIN3,DYNC1H1,LANCL2,ZFPM2,FRMPD4,MEIS1,EDAR,BRINP3,RIMBP2,FHIT,DNMT3A,TOX,ACE2,CHMP3,KCNAB1,PDE4D,ITGB8,PDE3A,RIMS1,WWOX,NEK10,BEND5,ANAPC10,DIAPH3,PLCB4,RNF216,FTO,TNFSF11,PPFIA2,WNT2,OR8B8,POU6F2,NTNG1,SGCD,GRM7,NAV3,PTPRM,PRKCH,NRXN3,DLC1,DENND2B,MIR663AHG,ATP8A2,LPIN1,AUTS2,BCAS3,COMT,MCTP1,NBAS,KMT2E,SYNE2,DOCK2,SDCCAG8,SSH2,CDH13,FBN2,DAB1,TTLL6,RFTN1,ALK,SEMA3A,YEATS2,SLC8A3,MALRD1,TENT4B,DCLK1,KIF16B,CDH2,TENM2,VPS41,HDAC9,USP9X,KCNB2,USP13,CDK6,ADGRL4,CADPS,DDX60,RGS7,SRGAP2,TFAP2D,PTPRT,GABPB1,SCEL,CTNNA3,RAPGEF4,CLTC,DACH2,SORCS1,CAMKMT,RNF220,DLG2,SACS,AFF2,PTPRD,RORA,PLCB1,NPEPPS,PRKG1,BRIP1,MAGI2,NELL1,PLCL1,SCN2A,ASIC2,DOCK10,TMEM164,GTDC1,RYR2,LDLRAD3,PTPRA,FAT3,MSH2,KLHDC10,RGS9,MPDZ,SLF1,ROBO2,PAX3,KSR2,DLGAP2,BMPR1B,DCC,CTNNA2,PEX5L,DNER,ETV6,NRG1,NPAS3,FANCB*
GO:0016339	calcium-dependent cell-cell adhesion via plasma	0.0006681583092867225	*NRXN1,CDH19,CDH18,CDH6,NLGN1,CDH12,CDH4,CDH13,CDH2*

	membrane cell adhesion molecules		
GO:0006810	transport	0.0006752234652529716	*ABCC6,EPN2,TRPM6,SLC8A1,RNF103-CHMP3,ANO5,RSRC1,GRIK2,SLC22A14,CADPS2,TLK1,ASTN2,LRP1B,KALRN,MKLN1,FGF12,BTBD9,NRXN1,DPP6,EXOC4,BBS9,NTRK2,CHD7,ELMO1,NCOA1,ATP2B4,TRPM3,FGF13,GPC3,DMD,KCNQ3,CHRM3,NALF1,BRAF,ERBB4,DOP1B,EXT2,SPAG16,HTR2C,TBC1D5,PIK3C3,SGIP1,PCLO,ANK2,ANO2,CACNA2D3,PTPRN2,DGKI,DENND1B,COLEC10,LARGE1,IGF1R,NLGN1,ANK1,KCNIP4,CFTR,DYNC1I1,NOS1AP,DOP1A,NKAIN2,ATP6V0D2,DPP10,FBLN5,SPEF2,ATP9B,CACNA2D1,PCSK1,HERC2,UTRN,AGBL4,GHR,RBFOX1,NKAIN3,DYNC1H1,OSBPL10,SLC35F1,ACE2,CHMP3,KCNAB1,PDE4D,RIMS1,NEK10,DIAPH3,PLCB4,TNFSF11,PPFIA2,GRM7,NRXN3,ATP8A2,COMT,MCTP1,NBAS,SYNE2,DOCK2,CDH13,RFTN1,CACHD1,SLC8A3,MYO5B,DCLK1,KIF16B,CDH2,VPS41,USP9X,KCNB2,LYST,CADPS,RGS7,RAPGEF4,CLTC,SORCS1,DLG2,PLCB1,PRKG1,MAGI2,SCN2A,ASIC2,MICAL3,RANBP17,RYR2,LDLRAD3,RGS9,SLC39A11,PEX5L,DNER,NRG1,SLC25A48,DNAH5*
GO:0016477	cell migration	0.0010184431053622013	*PTPRR,EPB41L4B,FGF1,ASTN2,DAAM2,STK24,NTRK2,NRG3,PTPRG,ATP2B4,FGF13,GPC3,ARHGAP24,SLIT2,BRAF,ERBB4,ITGA1,ROBO1,LARGE1,IGF1R,IQSEC1,DDR2,GPC6,MITF,GPC5,OGDH,ULK4,TNFSF11,NTNG1,NAV3,PTPRM,DLC1,AUTS2,BCAS3,MCTP1,SYNE2,DOCK2,SDCCAG8,SSH2,CDH13,DAB1,SEMA3A,DCLK1,CDH2,HDAC9,USP9X,LYST,SRGAP2,PTPRT,CTNNA3,PLCB1,PRKG1,MAGI2,DOCK10,FAT3,DCC,CTNNA2,DNER*
GO:0010976	positive regulation of neuron projection development	0.0010809400424914343	*KALRN,NEGR1,NRXN1,STK24,NTRK2,DMD,ZNF804A,BRAF,IGF1R,NLGN1,DDR2,TOX,ATP8A2,ALK,MAGI2*
GO:0097120	receptor localization to synapse	0.0011916804061731017	*NRXN1,NBEA,ERBB4,GPHN,NLGN1,ADAM22,GPC6,NRXN3,DLG2,CEP112*
GO:0050896	response to stimulus	0.001454088297531555	*ABCC6,PTPRR,EPB41L4B,EPN2,NFIA,TRPM6,SLC8A1,RNF103-CHMP3,ANO5,ZNF385B,RSRC1,GRIK2,FGF1,ZBTB20,ARHGAP15,AHR,FSTL5,TLK1,KALRN,MKLN1,USH2A,FGF12,PARP4,MAST4,NRXN1,MAGI1,GNGT1,FAM13A,DAAM2,STK24,GRM3,BBS9,NTRK2,CHD7,RASGEF1B,NRG3,PTPRG,CALCR,ELMO1,NCOA1,IRAG1,ATP2B4,PHEX,SOX6,TMEM64,RAPGEF5,FGF13,PATJ,GPC3,DMD,ADCY2,KCNQ3,ATRNL1,CHRM3,ARHGAP24,SLIT2,FAM20C,BRAF,BABAM2,KMT2C,ERBB4,GPHN,RAD51B,SH2D1A,MYO3B,SH3RF3,EXT2,MAP3K5,HTR2C,TBC1D5,RAP1GAP2,PIK3C3,HMCN1,ITGA1,TCF12,SGIP1,ENSG00000289694,MYO16,ESRRG,PCLO,FRMD6,ANK2,SUPT3H,ROBO1,DGKB,CDH6,DOK6,FGF14,PTPRN2,INPP4B,SOX5,DSCAM,DGKI,DENND1B,COLEC10,LARGE1,SDK1,EPHA6,IGF1R,NLGN1,IQSEC1,ANK1,PPM1L,CNTNAP2,TRIM71,CFTR,NOS1AP,RBMS3,TBCK,SHC4,DDR2,GPC6,MCF2L2*

			,CELF4,CNTN6,FBLN5,MITF,CACNA2D1,NSMCE2,PCSK1,HERC2,LRFN5,GPC5,AGBL4,GHR,ULK4,PPP1R9A,TMEM117,SMAD9,NAALADL2,LANCL2,EDAR,BRINP3,FHIT,WIPI2,DNMT3A,PCDH15,ACE2,CHMP3,PDE4D,ITGB8,PDE3A,RIMS1,WWOX,NEK10,DIAPH3,PLCB4,RNF216,FTO,TNFSF11,WNT2,OR8B8,SGCD,GRM7,PTPRM,PRKCH,NRXN3,DLC1,DENND2B,ATP8A2,CERS6,LPIN1,MACROD2,AUTS2,BCAS3,COMT,MCTP1,ZPLD1,DOCK2,CDH13,FBN2,DAB1,RFTN1,ALK,SEMA3A,SLC8A3,DCLK1,KIF16B,CDH2,TENM2,VPS41,HDAC9,USP9X,USP13,CDK6,ADGRL4,LYST,DDX60,RGS7,SRGAP2,PTPRT,CSMD1,SCEL,RAPGEF4,SORCS1,CAMKMT,RNF220,DLG2,PTPRD,RORA,PLCB1,NPEPPS,PRKG1,BRIP1,MAGI2,PLCL1,PXDNL,SCN2A,ASIC2,DOCK10,RYR2,PTPRA,MSH2,KLHDC10,RGS9,EYS,SLF1,ROBO2,KSR2,BMPR1B,CTNNA2,PEX5L,DNER,NRG1,FANCB*
GO:0034762	regulation of transmembrane transport	0.001477954131622381	*SLC8A1,FGF12,NRXN1,DPP6,CHD7,ATP2B4,FGF13,GPC3,DMD,CHRM3,BRAF,ANK2,NLGN1,KCNIP4,CFTR,NOS1AP,DPP10,CACNA2D1,UTRN,ACE2,KCNAB1,PDE4D,RGS7,ASIC2,RYR2,RGS9*
GO:0007010	cytoskeleton organization	0.001499197426642179	*EPB41L4B,RNF103-CHMP3,MKLN1,MAST4,DAAM2,DNAH7,ELMO1,PDE4DIP,DNAH8,FGF13,PATJ,DMD,SLIT2,BRAF,MYO3B,DIAPH2,STAG2,SPAG16,HMCN1,PCLO,ANK2,LARGE1,SPTB,NLGN1,IQSEC1,ANK1,NOS1AP,TBCK,SPEF2,ULK4,PPP1R9A,DYNC1H1,NEBL,FRMPD4,PCDH15,CHMP3,DIAPH3,NAV3,DLC1,ATP8A2,AUTS2,BCAS3,SYNE2,DOCK2,SDCCAG8,SSH2,TTLL6,MYO5B,DCLK1,SRGAP2,SPECC1,CLTC,THSD7B,PRKG1,MICAL3,MPDZ,CTNNA2,DNAH5*
GO:0050793	regulation of developmental process	0.001598519536069716	*EPN2,SLC8A1,FGF1,ARHGAP15,KALRN,MKLN1,USH2A,NRXN1,DAAM2,STK24,NTRK2,CHD7,NCOA1,ATP2B4,SOX6,TMEM64,FGF13,DMD,SLIT2,FAM20C,BRAF,ERBB4,MAP3K5,HTR2C,CNTN4,TCF12,RUNX1,ROBO1,CHODL,SOX5,DSCAM,SDK1,PUS7,IGF1R,NLGN1,CASZ1,CFTR,DDR2,TRPS1,CDH4,CELF4,MITF,AGBL4,GHR,RBFOX1,LRRC4C,ZFPM2,MEIS1,BRINP3,TOX,ITGB8,PDE3A,RIMS1,FTO,TNFSF11,PPFIA2,WNT2,NTNG1,PTPRM,PRKCH,DLC1,ATP8A2,LPIN1,FBN2,DAB1,ALK,SEMA3A,YEATS2,CDH2,HDAC9,CDK6,PTPRD,RORA,PLCB1,NELL1,ASIC2,FAT3,MSH2,ROBO2,BMPR1B,DCC,NRG1*
GO:0048518	positive regulation of biological process	0.0016006213269473917	*EPB41L4B,EPN2,NFIA,DIP2A,SLC8A1,GRIK2,FGF1,ZBTB20,AHR,CADPS2,KALRN,NEGR1,FGF12,NRXN1,MAGI1,DAAM2,STK24,LMO7,NTRK2,CHD7,CALCR,NCOA1,ATP2B4,TOX2,SOX6,PDE4DIP,TMEM64,FGF13,GPC3,DMD,CHRM3,SLIT2,ZNF804A,FAM20C,BRAF,BABAM2,KMT2C,ERBB4,KIF26B,RAD51B,SH2D1A,MYO3B,SH3RF3,MAP3K5,HTR2C,TBC1D5,ITGA1,TCF12,SGIP1,RUNX1,ESRRG,FRMD6,ANK2,SUPT3H,ROBO1,DOK6,CHODL,ZNF267,EDIL3,SOX5,DSCAM,DGKI,DENND1B,COLEC10,LARGE1,IGF1R,NLGN1,IQSEC1,ANK1,CASZ1,CNTNAP2,TRIM71,CFTR,NOS1AP,RBMS3,SHC4,DDR2,ADAM22,CDH4,DPP10,CELF4,CNTN6,MITF,CACNA2D1,NSMCE2,PCSK1,UTRN,GPC5,AGBL4,GHR,SMAD9,DYNC1H1,LANCL2,ZFPM2,FRMPD4,MEIS1,EDAR,BRINP3,DNMT3A,TOX,ACE2,CHMP3,PDE4D,ITGB8,PDE3A,RIMS1,WWOX,NEK10,FTO,TNFSF11,WNT2,NAV3,PRKCH,DLC1,DENND2B,ATP8A2,LPIN1,AUTS2,BCAS3,NBAS,KMT2E,SYNE2,DOCK2,SSH2,CDH13,FBN2,DAB1,TTLL6,RFTN*

			1,ALK,SEMA3A,SLC8A3,TENT4B,CDH2,TENM2,HDAC9,USP9X,USP13,CDK6,CADPS,DDX60,RGS7,TFAP2D,GABPB1,SCEL,RAPGEF4,RNF220,PTPRD,RORA,PLCB1,NPEPPS,PRKG1,MAGI2,NELL1,ASIC2,TMEM164,RYR2,MSH2,KLHDC10,SLF1,ROBO2,PAX3,KSR2,BMPR1B,ETV6,NRG1,NPAS3,FANCB
GO:0065007	biological regulation	0.001719845484446115	PAX7,PTPRR,EPB41L4B,EPN2,MED13L,NFIA,DIP2A,SLC8A1,RNF103-CHMP3,ZNF385B,GRIK2,KDM4B,FGF1,ZBTB20,ARHGAP15,AHR,FSTL5,CADPS2,TLK1,ASTN2,KALRN,MKLN1,USH2A,NEGR1,FGF12,BTBD9,MAST4,NRXN1,MAGI1,GNGT1,DPP6,FAM13A,DAAM2,STK24,LMO7,GRM3,EXOC4,NTRK2,CHD7,NBEA,RASGEF1B,MIR99AHG,NRG3,PTPRG,CALCR,ELMO1,NCOA1,IRAG1,ATP2B4,TOX2,PHEX,SOX6,PDE4DIP,TMEM64,RAPGEF5,FGF13,PATJ,GPC3,DMD,ADCY2,KCNQ3,ATRNL1,CHRM3,ARHGAP24,SLIT2,ZNF804A,FAM20C,BRAF,BABAM2,KMT2C,ERBB4,KIF26B,GPHN,RAD51B,SH2D1A,MYO3B,SH3RF3,EXT2,MAP3K5,HTR2C,TBC1D5,CNTN4,RAP1GAP2,PIK3C3,ITGA1,TCF12,SGIP1,ENSG00000289694,RUNX1,MYO16,ESRRG,PCLO,FRMD6,ANK2,SUPT3H,ROBO1,DGKB,EBF1,CDH6,DOK6,JAZF1,CHODL,ZNF267,FGF14,EDIL3,PTPRN2,INPP4B,SOX5,DSCAM,DGKI,LINC01151,DENND1B,COLEC10,LARGE1,SDK1,PUS7,EPHA6,IGF1R,SPTB,NLGN1,IQSEC1,ANK1,PPM1L,CASZ1,CNTNAP2,TRIM71,KCNIP4,CFTR,NOS1AP,NKAIN2,RBMS3,ATP6V0D2,ZNF800,TBCK,SHC4,DDR2,ADAM22,LARS2,HULC,GPC6,MCF2L2,TRPS1,CDH4,DPP10,CELF4,CNTN6,FBLN5,ZNF638,ATP9B,MITF,CACNA2D1,NSMCE2,PCSK1,LRFN5,UTRN,GPC5,AGBL4,GHR,ULK4,RBFOX1,LRRC4C,PPP1R9A,TMEM117,SMAD9,NKAIN3,DYNC1H1,LANCL2,ZFPM2,FRMPD4,MEIS1,EDAR,BRINP3,RIMBP2,FHIT,DNMT3A,TOX,ACE2,CHMP3,KCNAB1,PDE4D,ITGB8,PDE3A,RIMS1,WWOX,NEK10,BEND5,ANAPC10,DIAPH3,PLCB4,RNF216,FTO,TNFSF11,PPFIA2,WNT2,OR8B8,POU6F2,NTNG1,SGCD,GRM7,NAV3,PTPRM,PRKCH,NRXN3,DLC1,DENND2B,MIR663AHG,ATP8A2,LPIN1,AUTS2,BCAS3,COMT,MCTP1,NBAS,KMT2E,SYNE2,DOCK2,SDCCAG8,SSH2,CDH13,FBN2,DAB1,TTLL6,RFTN1,ALK,SEMA3A,YEATS2,SLC8A3,MYO5B,MALRD1,TENT4B,DCLK1,KIF16B,CDH2,TENM2,VPS41,HDAC9,USP9X,KCNB2,USP13,CDK6,ADGRL4,CADPS,DDX60,RGS7,SRGAP2,TFAP2D,PTPRT,GABPB1,SCEL,CTNNA3,RAPGEF4,CLTC,DACH2,SORCS1,CAMKMT,RNF220,DLG2,SACS,AFF2,PTPRD,RORA,PLCB1,NPEPPS,PRKG1,BRIP1,MAGI2,NELL1,PLCL1,SCN2A,ASIC2,DOCK10,TMEM164,GTDC1,RYR2,LDLRAD3,PTPRA,FAT3,MSH2,KLHDC10,RGS9,MPDZ,SLF1,ROBO2,PAX3,KSR2,DLGAP2,BMPR1B,DCC,CTNNA2,PEX5L,DNER,ETV6,NRG1,NPAS3,FANCB
GO:0007165	signal transduction	0.002099816400343617	PTPRR,EPN2,NFIA,SLC8A1,ZNF385B,GRIK2,FGF1,ARHGAP15,AHR,FSTL5,TLK1,KALRN,MKLN1,FGF12,MAST4,NRXN1,MAGI1,GNGT1,FAM13A,DAAM2,STK24,GRM3,NTRK2,RASGEF1B,NRG3,PTPRG,CALCR,ELMO1,NCOA1,IRAG1,ATP2B4,TMEM64,RAPGEF5,FGF13,PATJ,GPC3,DMD,ADCY2,KCNQ3,ATRNL1,CHRM3,ARHGAP24,SLIT2,FAM20C,BRAF,BABAM2,ERBB4,SH2D1A,SH3RF3,MAP3K5,HTR2C,RAP1GAP2,PIK3C3,ITGA1,ENSG00000289694,MYO16,ESRRG,PCLO,FRMD6,ANK2,ROBO1,DGKB,CDH6,DOK6,FGF14,INPP4B,DSCAM,DGKI,DENND1B,COLEC10,LARGE1,EPHA6,IGF1R,NLGN1,IQSEC1,ANK1,PPM1L,TRIM71

			,NOS1AP,RBMS3,TBCK,SHC4,DDR2,GPC6,CELF4,CNTN6,MITF,GPC5,GHR,ULK4,PPP1R9A,TMEM117,SMAD9,LANCL2,EDAR,FHIT,ACE2,PDE4D,ITGB8,PDE3A,RIMS1,WWOX,NEK10,DIAPH3,PLCB4,TNFSF11,WNT2,OR8B8,SGCD,GRM7,PTPRM,PRKCH,NRXN3,DLC1,DENND2B,AUTS2,MCTP1,DOCK2,CDH13,FBN2,DAB1,RFTN1,ALK,SEMA3A,SLC8A3,DCLK1,KIF16B,CDH2,TENM2,USP9X,CDK6,ADGRL4,DDX60,RGS7,SRGAP2,PTPRT,SCEL,RAPGEF4,SORCS1,CAMKMT,RNF220,PTPRD,RORA,PLCB1,PRKG1,BRIP1,MAGI2,PLCL1,SCN2A,ASIC2,DOCK10,RYR2,PTPRA,MSH2,KLHDC10,RGS9,ROBO2,KSR2,BMPR1B,PEX5L,DNER,NRG1
GO:0099560	synaptic membrane adhesion	0.002273598647578808	*NRXN1,CDH6,NLGN1,LRFN5,LRRC4C,NTNG1,PTPRD*
GO:0016358	dendrite development	0.002382270306825594	*DIP2A,KALRN,DSCAM,SDK1,NLGN1,IQSEC1,PPFIA2,DAB1,ALK,SEMA3A,DCLK1,SRGAP2,PTPRD,PRKG1,DOCK10,FAT3,DCC,CTNNA2*
GO:0051716	cellular response to stimulus	0.0023905362625089607	*PTPRR,EPN2,NFIA,SLC8A1,ZNF385B,GRIK2,FGF1,ZBTB20,ARHGAP15,AHR,FSTL5,TLK1,KALRN,MKLN1,FGF12,PARP4,MAST4,NRXN1,MAGI1,GNGT1,FAM13A,DAAM2,STK24,GRM3,NTRK2,RASGEF1B,NRG3,PTPRG,CALCR,ELMO1,NCOA1,IRAG1,ATP2B4,PHEX,TMEM64,RAPGEF5,FGF13,PATJ,GPC3,DMD,ADCY2,KCNQ3,ATRNL1,CHRM3,ARHGAP24,SLIT2,FAM20C,BRAF,BABAM2,ERBB4,RAD51B,SH2D1A,SH3RF3,MAP3K5,HTR2C,RAP1GAP2,PIK3C3,ITGA1,ENSG00000289694,MYO16,ESRRG,PCLO,FRMD6,ANK2,SUPT3H,ROBO1,DGKB,CDH6,DOK6,FGF14,PTPRN2,INPP4B,DSCAM,DGKI,DENND1B,COLEC10,LARGE1,EPHA6,IGF1R,NLGN1,IQSEC1,ANK1,PPM1L,TRIM71,CFTR,NOS1AP,RBMS3,TBCK,SHC4,DDR2,GPC6,CELF4,CNTN6,FBLN5,MITF,CACNA2D1,NSMCE2,HERC2,GPC5,GHR,ULK4,PPP1R9A,TMEM117,SMAD9,LANCL2,EDAR,BRINP3,FHIT,WIPI2,DNMT3A,ACE2,PDE4D,ITGB8,PDE3A,RIMS1,WWOX,NEK10,DIAPH3,PLCB4,FTO,TNFSF11,WNT2,OR8B8,SGCD,GRM7,PTPRM,PRKCH,NRXN3,DLC1,DENND2B,LPIN1,MACROD2,AUTS2,BCAS3,COMT,MCTP1,DOCK2,CDH13,FBN2,DAB1,RFTN1,ALK,SEMA3A,SLC8A3,DCLK1,KIF16B,CDH2,TENM2,VPS41,HDAC9,USP9X,USP13,CDK6,ADGRL4,LYST,DDX60,RGS7,SRGAP2,PTPRT,SCEL,RAPGEF4,SORCS1,CAMKMT,RNF220,DLG2,PTPRD,RORA,PLCB1,NPEPPS,PRKG1,BRIP1,MAGI2,PLCL1,PXDNL,SCN2A,ASIC2,DOCK10,RYR2,PTPRA,MSH2,KLHDC10,RGS9,SLF1,ROBO2,KSR2,BMPR1B,PEX5L,DNER,NRG1,FANCB*
GO:0050794	regulation of cellular process	0.0033449140684675685	*PAX7,PTPRR,EPB41L4B,EPN2,MED13L,NFIA,DIP2A,SLC8A1,RNF103-CHMP3,ZNF385B,GRIK2,KDM4B,FGF1,ZBTB20,ARHGAP15,AHR,FSTL5,CADPS2,TLK1,ASTN2,KALRN,MKLN1,USH2A,NEGR1,FGF12,BTBD9,MAST4,NRXN1,MAGI1,GNGT1,DPP6,FAM13A,DAAM2,STK24,LMO7,GRM3,EXOC4,NTRK2,CHD7,NBEA,RASGEF1B,MIR99AHG,NRG3,PTPRG,CALCR,ELMO1,NCOA1,IRAG1,ATP2B4,TOX2,SOX6,PDE4DIP,TMEM64,RAPGEF5,FGF13,PATJ,GPC3,DMD,ADCY2,KCNQ3,ATRNL1,CHRM3,ARHGAP24,SLIT2,ZNF804A,FAM20C,BRAF,BABAM2,KMT2C,ERBB4,KIF26B,RAD51B,SH2D1A,MYO3B,SH3RF3,MAP3K5,HTR2C,TBC1D5,CNTN4,RAP1GAP2,PIK3C3,ITGA1,TCF12,SGIP1,ENSG00000289694,RUNX1,MYO16,ESRRG,PCLO,FRMD6,ANK2,SUPT3H*

			,ROBO1,DGKB,EBF1,CDH6,DOK6,JAZF1,CHODL,ZNF267,FGF14,EDIL3,INPP4B,SOX5,DSCAM,DGKI,LINC01151,DENND1B,COLEC10,LARGE1,PUS7,EPHA6,IGF1R,SPTB,NLGN1,IQSEC1,ANK1,PPM1L,CASZ1,CNTNAP2,TRIM71,KCNIP4,CFTR,NOS1AP,RBMS3,ATP6V0D2,ZNF800,TBCK,SHC4,DDR2,ADAM22,HULC,GPC6,TRPS1,CDH4,DPP10,CELF4,CNTN6,FBLN5,ZNF638,MITF,CACNA2D1,NSMCE2,PCSK1,LRFN5,UTRN,GPC5,AGBL4,GHR,ULK4,RBFOX1,LRRC4C,PPP1R9A,TMEM117,SMAD9,DYNC1H1,LANCL2,ZFPM2,FRMPD4,MEIS1,EDAR,BRINP3,FHIT,DNMT3A,TOX,ACE2,CHMP3,KCNAB1,PDE4D,ITGB8,PDE3A,RIMS1,WWOX,NEK10,BEND5,ANAPC10,DIAPH3,PLCB4,RNF216,FTO,TNFSF11,PPFIA2,WNT2,OR8B8,POU6F2,NTNG1,SGCD,GRM7,NAV3,PTPRM,PRKCH,NRXN3,DLC1,DENND2B,MIR663AHG,ATP8A2,LPIN1,AUTS2,BCAS3,MCTP1,NBAS,KMT2E,SYNE2,DOCK2,SDCCAG8,SSH2,CDH13,FBN2,DAB1,TTLL6,RFTN1,ALK,SEMA3A,YEATS2,SLC8A3,MALRD1,TENT4B,DCLK1,KIF16B,CDH2,TENM2,VPS41,HDAC9,USP9X,USP13,CDK6,ADGRL4,CADPS,DDX60,RGS7,SRGAP2,TFAP2D,PTPRT,GABPB1,SCEL,CTNNA3,RAPGEF4,CLTC,DACH2,SORCS1,CAMKMT,RNF220,SACS,AFF2,PTPRD,RORA,PLCB1,NPEPPS,PRKG1,BRIP1,MAGI2,NELL1,PLCL1,SCN2A,ASIC2,DOCK10,TMEM164,GTDC1,RYR2,LDLRAD3,PTPRA,FAT3,MSH2,KLHDC10,RGS9,MPDZ,SLF1,ROBO2,PAX3,KSR2,BMPR1B,DCC,CTNNA2,PEX5L,DNER,ETV6,NRG1,NPAS3,FANCB
GO:0051239	regulation of multicellular organismal process	0.0042891912768151744	PTPRR,EPB41L4B,EPN2,SLC8A1,GRIK2,FGF1,ZBTB20,AHR,KALRN,FGF12,NRXN1,DAAM2,NTRK2,CHD7,PTPRG,CALCR,NCOA1,ATP2B4,SOX6,TMEM64,FGF13,DMD,CHRM3,SLIT2,FAM20C,BRAF,ERBB4,HTR2C,SGIP1,RUNX1,ESRRG,ANK2,ROBO1,CHODL,SOX5,DSCAM,DENND1B,IGF1R,NLGN1,IQSEC1,CFTR,NOS1AP,DDR2,TRPS1,CDH4,CELF4,MITF,CACNA2D1,LRFN5,GHR,DYNC1H1,ZFPM2,MEIS1,TOX,ACE2,PDE4D,ITGB8,PDE3A,RIMS1,RNF216,FTO,TNFSF11,WNT2,NAV3,PTPRM,PRKCH,ATP8A2,LPIN1,FBN2,DAB1,RFTN1,SEMA3A,YEATS2,SLC8A3,HDAC9,KCNB2,CDK6,CTNNA3,PTPRD,RORA,PLCB1,PRKG1,NELL1,PLCL1,ASIC2,RYR2,MSH2,ROBO2,KSR2,BMPR1B,DCC,NRG1
GO:0065008	regulation of biological quality	0.004450919232336791	SLC8A1,GRIK2,ARHGAP15,KALRN,MKLN1,NEGR1,FGF12,NRXN1,DAAM2,NTRK2,CHD7,NBEA,CALCR,NCOA1,ATP2B4,FGF13,DMD,KCNQ3,CHRM3,SLIT2,ZNF804A,BRAF,ERBB4,GPHN,MYO3B,EXT2,HTR2C,CNTN4,RAP1GAP2,ITGA1,PCLO,ANK2,DGKB,PTPRN2,DSCAM,DGKI,LARGE1,IGF1R,SPTB,NLGN1,TRIM71,CFTR,NOS1AP,ATP6V0D2,ADAM22,LARS2,GPC6,CDH4,CELF4,ATP9B,CACNA2D1,PCSK1,LRFN5,GHR,FRMPD4,ACE2,CHMP3,PDE4D,PDE3A,RIMS1,FTO,TNFSF11,PPFIA2,NRXN3,DLC1,ATP8A2,COMT,MCTP1,SSH2,TTLL6,SEMA3A,SLC8A3,MYO5B,TENT4B,CDH2,USP9X,USP13,PTPRT,CTNNA3,RAPGEF4,PTPRD,PLCB1,PRKG1,SCN2A,ASIC2,RYR2,ROBO2,DCC,CTNNA2,NRG1
GO:0021953	central nervous system neuron differentiation	0.005467586618395771	NRXN1,NTRK2,SLIT2,ERBB4,ROBO1,AGBL4,OGDH,BRINP3,TOX,WNT2,MDGA2,DCLK1,RORA,ROBO2,BMPR1B,DCC
GO:0050	regulatio	0.005879611	NTRK2,FGF13,SLIT2,BRAF,ROBO1,CHODL,DSCAM,

770	n of axonogenesis	4529779285	*CDH4,LRRC4C,DAB1,SEMA3A,CDH2,ROBO2,DCC*
GO:0009888	tissue development	0.006007243405245102	*NFIA,SLC8A1,FGF1,ASTN2,USH2A,EXOC4,CHD7,NCOA1,PHEX,SOX6,GPC3,DMD,ARHGAP24,SLIT2,FAM20C,KAZN,ERBB4,KIF26B,RAD51B,EXT2,RUNX1,FRMD6,ROBO1,SOX5,LARGE1,PUS7,WDR72,TRIM71,CFTR,ZNF800,DDR2,TRPS1,GHR,RBFOX1,NEBL,ZFPM2,MEIS1,EDAR,TOX,PCDH15,SGCZ,PDE4D,ITGB8,CPLANE1,FTO,TNFSF11,WNT2,SGCD,PRKCH,DLC1,FBN2,SEMA3A,KIF16B,CDH2,HDAC9,CDK6,CSMD1,SCEL,FREM2,RNF220,PLCB1,PRKG1,MAGI2,NELL1,RYR2,ROBO2,BMPR1B,DNER,NRG1*
GO:0006941	striated muscle contraction	0.00603283635348787	*SLC8A1,FGF12,FGF13,DMD,ANK2,LARGE1,NOS1AP,CACNA2D1,ACE2,PDE4D,SGCD,ATP8A2,SLC8A3,CTNNA3,RYR2*
GO:0051179	localization	0.006205842026681439	*ABCC6,EPN2,TRPM6,SLC8A1,RNF103-CHMP3,ANO5,RSRC1,GRIK2,SLC22A14,CADPS2,TLK1,ASTN2,LRP1B,KALRN,MKLN1,USH2A,FGF12,BTBD9,NRXN1,GNGT1,DPP6,EXOC4,BBS9,NTRK2,CHD7,NBEA,ELMO1,NCOA1,ATP2B4,TRPM3,FGF13,GPC3,DMD,KCNQ3,CHRM3,NALF1,BRAF,ERBB4,GPHN,DOP1B,EXT2,SPAG16,HTR2C,TBC1D5,PIK3C3,SGIP1,PCLO,FRMD6,ANK2,ANO2,CACNA2D3,PTPRN2,DGKI,DENND1B,COLEC10,LARGE1,IGF1R,WDR72,NLGN1,ANK1,CNTNAP2,KCNIP4,CFTR,DYNC1I1,NOS1AP,DOP1A,NKAIN2,ATP6V0D2,ADAM22,GPC6,DPP10,FBLN5,SPEF2,ATP9B,CACNA2D1,PCSK1,HERC2,UTRN,GPC5,AGBL4,GHR,RBFOX1,NKAIN3,DYNC1H1,OSBPL10,SLC35F1,WIPI2,ACE2,CHMP3,KCNAB1,PDE4D,RIMS1,NEK10,MIPEP,DIAPH3,CPLANE1,PLCB4,FTO,TNFSF11,PPFIA2,SGCD,GRM7,PRKCH,NRXN3,ATP8A2,COMT,MCTP1,NBAS,SYNE2,DOCK2,CDH13,FBN2,DAB1,RFTN1,CACHD1,SLC8A3,MYO5B,DCLK1,KIF16B,CDH2,VPS41,USP9X,KCNB2,LYST,CADPS,RGS7,RAPGEF4,CLTC,SORCS1,DLG2,PLCB1,NPEPPS,PRKG1,MAGI2,SCN2A,ASIC2,MICAL3,RANBP17,RYR2,LDLRAD3,MSH2,RGS9,SLF1,SLC39A11,PEX5L,DNER,CEP112,NRG1,SLC25A48,DNAH5*
GO:0048589	developmental growth	0.007165982774192881	*FGF1,CHD7,FGF13,DMD,SLIT2,ERBB4,KIF26B,RAD51B,RUNX1,DSCAM,LARGE1,DDR2,CDH4,GHR,ZFPM2,MEIS1,PCDH15,RIMS1,FTO,WNT2,ATP8A2,AUTS2,COMT,SEMA3A,DCLK1,USP9X,PLCB1,PRKG1,EYS,BMPR1B,DCC,NRG1*
GO:0035418	protein localization to synapse	0.008170088807735008	*NRXN1,NBEA,ERBB4,PCLO,NLGN1,ADAM22,GPC6,NRXN3,DLG2,ASIC2*
GO:0036211	protein modification process	0.01021838403086131	*PTPRR,DIP2A,TRPM6,SLC8A1,RNF103-CHMP3,FGF1,TTC3,TLK1,KALRN,SUMF1,XXYLT1,PARP4,MAST4,NRXN1,AGBL1,ST6GALNAC3,STK24,LMO7,NTRK2,PTPRG,ATP2B4,PHEX,DMD,CHRM3,SLIT2,FAM20C,BRAF,BABAM2,B3GNT5,ERBB4,MYO3B,SH3RF3,EXT2,MAP3K5,GALNT13,PIK3C3,ROBO1,NEK1,PTPRN2,LARGE1,EPHA6,IGF1R,TMEM260,PPM1L,TRIM71,NOS1AP,TBCK,DDR2,ST8SIA6,WDSUB1,NSMCE2,HERC2,AGBL4,GHR,ULK4,CRPPA,GALNT17,PDE4D,NEK10,ANAPC10,RNF216,UGGT2,TNFSF11,MGAT4C,PTPRM,PRKCH,MACROD2,SSH2,TTLL6,ALK,YEATS2,SLC8A3,DCLK1,HDAC9,USP9X,USP13,CDK6,PTPRT,RNF220,PTPRD,NPEPPS,PRKG1,GALNTL6,PTPRA,MGAT4A,KLHDC10,SLF1,KSR2,BMPR1B*

			,NRG1,PGM3
GO:0051130	positive regulation of cellular component organization	0.010280787036164151	*KALRN,NEGR1,NRXN1,STK24,NTRK2,PDE4DIP,GPC3,DMD,SLIT2,ZNF804A,BRAF,MYO3B,TBC1D5,SGIP1,RUNX1,ROBO1,CHODL,DSCAM,COLEC10,IGF1R,NLGN1,IQSEC1,ANK1,CNTNAP2,DDR2,CDH4,NSMCE2,DYNC1H1,FRMPD4,TOX,ACE2,NAV3,ATP8A2,AUTS2,DOCK2,ALK,TENM2,PTPRD,PLCB1,MAGI2,ASIC2,SLF1,ROBO2,NRG1*
GO:0032879	regulation of localization	0.010470011345001777	*SLC8A1,CADPS2,ASTN2,KALRN,MKLN1,FGF12,BTBD9,NRXN1,DPP6,CHD7,NBEA,ATP2B4,FGF13,GPC3,DMD,CHRM3,BRAF,ERBB4,HTR2C,TBC1D5,SGIP1,PCLO,ANK2,PTPRN2,COLEC10,NLGN1,KCNIP4,CFTR,NOS1AP,NKAIN2,ADAM22,GPC6,DPP10,CACNA2D1,PCSK1,UTRN,GPC5,NKAIN3,DYNC1H1,ACE2,CHMP3,KCNAB1,PDE4D,RIMS1,PLCB4,FTO,TNFSF11,PPFIA2,GRM7,PRKCH,ATP8A2,MCTP1,DOCK2,CDH13,DCLK1,CDH2,CADPS,RGS7,RAPGEF4,CLTC,PLCB1,NPEPPS,PRKG1,MAGI2,ASIC2,RYR2,RGS9,NRG1*
GO:0001508	action potential	0.010794567193695061	*SLC8A1,GRIK2,FGF12,NTRK2,FGF13,DMD,KCNQ3,ANK2,NOS1AP,CACNA2D1,CTNNA3,SCN2A,RYR2*
GO:0021772	olfactory bulb development	0.010802836757500989	*CHD7,SLIT2,ERBB4,ROBO1,OGDH,SEMA3A,ROBO2*
GO:0034765	regulation of monoatomic ion transmembrane transport	0.012181407709833262	*SLC8A1,FGF12,DPP6,CHD7,ATP2B4,FGF13,DMD,ANK2,KCNIP4,CFTR,NOS1AP,DPP10,CACNA2D1,UTRN,KCNAB1,PDE4D,RGS7,ASIC2,RYR2,RGS9*
GO:1903779	regulation of cardiac conduction	0.01413242318030118	*SLC8A1,ATP2B4,ANK2,ACE2,SLC8A3,RYR2*
GO:0051094	positive regulation of developmental process	0.015355116802305896	*SLC8A1,FGF1,KALRN,NRXN1,STK24,NTRK2,CHD7,NCOA1,SOX6,TMEM64,DMD,SLIT2,FAM20C,BRAF,ERBB4,MAP3K5,HTR2C,TCF12,RUNX1,ROBO1,CHODL,SOX5,DSCAM,IGF1R,NLGN1,CFTR,DDR2,CDH4,GHR,ZFPM2,BRINP3,TOX,ITGB8,PDE3A,RIMS1,TNFSF11,WNT2,PRKCH,ATP8A2,FBN2,DAB1,ALK,PTPRD,PLCB1,NELL1,ASIC2,MSH2,ROBO2,BMPR1B,NRG1*
GO:0021988	olfactory lobe development	0.01578961185080916	*CHD7,SLIT2,ERBB4,ROBO1,OGDH,SEMA3A,ROBO2*
GO:0030048	actin filament-based movement	0.016813350336085536	*FGF12,FGF13,FRMD6,ANK2,NOS1AP,CACNA2D1,PDE4D,SGCD,SYNE2,MYO5B,CTNNA3,RYR2*
GO:0010842	retina layer formation	0.018069706966388037	*DSCAM,LARGE1,SDK1,PTPRM,ATP8A2,FAT3*
GO:0099173	postsynapse organization	0.018074278885614744	*DIP2A,KALRN,NRXN1,ZNF804A,GPHN,DGKB,IGF1R,NLGN1,NOS1AP,FRMPD4,PPFIA2,NRXN3,CDH2,PTPRD,DOCK10*
GO:0021987	cerebral cortex developme	0.019717791532523173	*NTRK2,NCOA1,FGF13,DMD,SLIT2,ROBO1,CNTNAP2,SYNE2,DAB1,CDH2,SRGAP2,PLCB1*

	nt		
GO:0086003	cardiac muscle cell contraction	0.019936793362334483	*FGF12,FGF13,ANK2,NOS1AP,CACNA2D1,PDE4D,SGCD,CTNNA3,RYR2*
GO:0007158	neuron cell-cell adhesion	0.020653822160069155	*ASTN2,NRXN1,CNTN4,NLGN1,NRXN3*
GO:0043412	macromolecule modification	0.02131201177683513	*PTPRR,DIP2A,TRPM6,SLC8A1,RNF103-CHMP3,FGF1,TTC3,TLK1,KALRN,SUMF1,XXYLT1,PARP4,MAST4,NRXN1,AGBL1,ST6GALNAC3,STK24,LMO7,NTRK2,PTPRG,ATP2B4,PHEX,DMD,CHRM3,SLIT2,FAM20C,BRAF,BABAM2,B3GNT5,ERBB4,MYO3B,SH3RF3,EXT2,MAP3K5,GALNT13,PIK3C3,ROBO1,NEK1,PTPRN2,LARGE1,PUS7,EPHA6,IGF1R,TMEM260,PPM1L,TRIM71,NOS1AP,TBCK,DDR2,ST8SIA6,WDSUB1,NSMCE2,HERC2,AGBL4,GHR,ULK4,CRPPA,GALNT17,PDE4D,NEK10,ANAPC10,RNF216,UGGT2,TNFSF11,MGAT4C,PTPRM,PRKCH,MACROD2,ADARB2,SSH2,TTLL6,ALK,YEATS2,SLC8A3,DCLK1,HDAC9,USP9X,USP13,CDK6,PTPRT,RNF220,PTPRD,NPEPPS,PRKG1,GALNTL6,GTDC1,PTPRA,MGAT4A,KLHDC10,SLF1,KSR2,BMPR1B,NRG1,PGM3*
GO:0098900	regulation of action potential	0.022161804650298277	*FGF12,FGF13,KCNQ3,ANK2,NOS1AP,CACNA2D1,CTNNA3,RYR2*
GO:0086001	cardiac muscle cell action potential	0.022395199988682918	*SLC8A1,FGF12,FGF13,DMD,ANK2,NOS1AP,CACNA2D1,CTNNA3,RYR2*
GO:1904062	regulation of monoatomic cation transmembrane transport	0.025559622799333857	*SLC8A1,FGF12,DPP6,CHD7,ATP2B4,FGF13,DMD,ANK2,KCNIP4,NOS1AP,DPP10,CACNA2D1,UTRN,KCNAB1,PDE4D,RGS7,RYR2,RGS9*
GO:0007612	learning	0.025843426031664393	*KALRN,NRXN1,NTRK2,FGF13,BRAF,DGKI,CNTNAP2,NRXN3,COMT,SLC8A3,SPECC1,CSMD1,PLCB1*
GO:0060048	cardiac muscle contraction	0.026870349418196198	*SLC8A1,FGF12,FGF13,DMD,ANK2,NOS1AP,CACNA2D1,ACE2,PDE4D,SGCD,CTNNA3,RYR2*
GO:0040013	negative regulation of locomotion	0.027162332379080993	*PTPRR,STK24,NRG3,PTPRG,ATP2B4,SLIT2,BRAF,ROBO1,MITF,NAV3,PTPRM,DLC1,MCTP1,SEMA3A,SRGAP2,PTPRT,PLCB1,PRKG1,MAGI2,ROBO2*
GO:0070588	calcium ion transmembrane transport	0.027162332379080993	*TRPM6,SLC8A1,CHD7,ATP2B4,TRPM3,DMD,NALF1,HTR2C,ANK2,CACNA2D3,NOS1AP,CACNA2D1,PDE4D,PLCB4,CACHD1,SLC8A3,PLCB1,SCN2A,RYR2,RGS9*
GO:0051049	regulation of transport	0.027199243478440407	*SLC8A1,CADPS2,KALRN,MKLN1,FGF12,BTBD9,NRXN1,DPP6,CHD7,ATP2B4,FGF13,GPC3,DMD,CHRM3,BRAF,HTR2C,TBC1D5,SGIP1,PCLO,ANK2,PTPRN2,COLEC10,NLGN1,KCNIP4,CFTR,NOS1AP,NKAIN2,DPP10,CACNA2D1,PCSK1,UTRN,NKAIN3,DYNC1H1,A*

			CE2,CHMP3,KCNAB1,PDE4D,RIMS1,TNFSF11,PPFIA2,GRM7,ATP8A2,MCTP1,DOCK2,CDH13,CDH2,CADPS,RGS7,RAPGEF4,PLCB1,PRKG1,MAGI2,ASIC2,RYR2,RGS9,NRG1
GO:0000165	MAPK cascade	0.027869804357908375	*PTPRR,GRIK2,FGF1,FGF12,NRXN1,NTRK2,CALCR,FGF13,DMD,BRAF,ERBB4,SH3RF3,MAP3K5,HTR2C,ITGA1,ROBO1,DOK6,FGF14,IGF1R,PPM1L,GHR,ULK4,EDAR,ACE2,NEK10,TNFSF11,DENND2B,SEMA3A,CDH2,PLCB1,KLHDC10,KSR2,NRG1*
GO:0007043	cell-cell junction assembly	0.03171407949273173	*PATJ,CDH19,CDH18,ANK2,CDH6,CNTNAP2,CDH12,CDH4,ACE2,PRKCH,CDH13,CDH2,MPDZ*
GO:0006936	muscle contraction	0.03219155739994129	*SLC8A1,FGF12,ATP2B4,FGF13,DMD,CHRM3,ANK2,LARGE1,NOS1AP,CACNA2D1,UTRN,ACE2,PDE4D,SGCD,ATP8A2,SLC8A3,KCNB2,CTNNA3,PRKG1,RYR2*
GO:0060047	heart contraction	0.03323277653367715	*SLC8A1,FGF12,ATP2B4,FGF13,DMD,EXT2,ANK2,NOS1AP,CACNA2D1,SGCZ,ACE2,PDE4D,SGCD,SLC8A3,CTNNA3,RYR2*
GO:0030336	negative regulation of cell migration	0.03394490826801871	*PTPRR,STK24,NRG3,PTPRG,ATP2B4,SLIT2,BRAF,ROBO1,MITF,NAV3,PTPRM,DLC1,MCTP1,SRGAP2,PTPRT,PLCB1,PRKG1,MAGI2*
GO:0021766	hippocampus development	0.03445657635766696	*NCOA1,ATP2B4,FGF13,LARGE1,IGF1R,OGDH,DAB1,ALK,CDK6,SCN2A*
GO:0010880	regulation of release of sequestered calcium ion into cytosol by sarcoplasmic reticulum	0.0354722775313389	*SLC8A1,CHD7,DMD,ANK2,PDE4D,RYR2*
GO:0043410	positive regulation of MAPK cascade	0.03582502181764471	*FGF1,NRXN1,NTRK2,CALCR,BRAF,ERBB4,SH3RF3,MAP3K5,HTR2C,ITGA1,ROBO1,DOK6,IGF1R,GHR,EDAR,NEK10,TNFSF11,DENND2B,SEMA3A,CDH2,PLCB1,KLHDC10,KSR2,NRG1*
GO:0098660	inorganic ion transmembrane transport	0.04124887507476212	*TRPM6,SLC8A1,ANO5,FGF12,DPP6,CHD7,ATP2B4,TRPM3,FGF13,DMD,KCNQ3,NALF1,HTR2C,ANK2,ANO2,CACNA2D3,LARGE1,KCNIP4,CFTR,NOS1AP,ATP6V0D2,DPP10,CACNA2D1,UTRN,KCNAB1,PDE4D,PLCB4,CACHD1,SLC8A3,KCNB2,RGS7,PLCB1,SCN2A,ASIC2,RYR2,RGS9,SLC39A11*
GO:0001964	startle response	0.04361139522699298	*NRXN1,CNTNAP2,PCDH15,COMT,CSMD1,CTNNA2*
GO:0070085	glycosylation	0.045395561083532036	*XXYLT1,ST6GALNAC3,B3GNT5,EXT2,GALNT13,LARGE1,TMEM260,ST8SIA6,CRPPA,GALNT17,UGGT2,MGAT4C,GRM7,GALNTL6,MGAT4A,PGM3*
GO:0050890	cognition	0.04835863976014211	*KALRN,BTBD9,NRXN1,NTRK2,CHD7,FGF13,BRAF,DOP1B,DGKI,LARGE1,CNTNAP2,NRXN3,COMT,SLC8A3,SPECC1,CSMD1,AFF2,PLCB1,SCN2A*
GO:0097553	calcium ion transmembrane	0.04889236977160157	*SLC8A1,CHD7,ATP2B4,DMD,NALF1,HTR2C,ANK2,CACNA2D1,PDE4D,PLCB4,SLC8A3,PLCB1,SCN2A,RYR2*

	import into cytosol		
GO:0030001	metal ion transport	0.04912650465612783	*TRPM6,SLC8A1,FGF12,DPP6,CHD7,ATP2B4,TRPM3,FGF13,DMD,KCNQ3,NALF1,HTR2C,ANK2,CACNA2D3,LARGE1,KCNIP4,NOS1AP,NKAIN2,DPP10,CACNA2D1,UTRN,NKAIN3,KCNAB1,PDE4D,PLCB4,CACHD1,SLC8A3,KCNB2,RGS7,CLTC,PLCB1,SCN2A,ASIC2,RYR2,RGS9,SLC39A11*
			CC
GO:0030054	cell junction	3.856665337749302e-24	*PTPRR,EPB41L4B,NFIA,DIP2A,SLC8A1,GRIK2,CADPS2,KALRN,MKLN1,USH2A,FGF12,BTBD9,NRXN1,MAGI1,LMO7,GRM3,EXOC4,NTRK2,NBEA,NRG3,ATP2B4,FBLN7,FGF13,PATJ,DMD,KCNQ3,CDH19,CHRM3,ARHGAP24,ZNF804A,KAZN,BRAF,ERBB4,GPHN,DSCAML1,HTR2C,HMCN1,ITGA1,PCLO,CDH18,FRMD6,ANK2,ANO2,DGKB,CDH6,LRRTM4,PTPRN2,DSCAM,DGKI,LARGE1,SDK1,SPTB,NLGN1,IQSEC1,ANK1,CNTNAP2,NOS1AP,PCDH9,CDH12,SHC4,DDR2,ADAM22,GPC6,CDH4,CELF4,CNTN6,PCSK1,LRFN5,UTRN,LRRC4C,PPP1R9A,SLC35F1,FRMPD4,RIMBP2,PCDH15,CNTN5,ITGB8,RIMS1,DIAPH3,PLCB4,PPFIA2,NTNG1,GRM7,PTPRM,PRKCH,NRXN3,DLC1,PLEKHA5,COMT,MCTP1,SYNE2,SDCCAG8,SSH2,CDH13,SEMA3A,SLC8A3,CADM2,DCLK1,CDH2,TENM2,CADPS,SRGAP2,PTPRT,CTNNA3,RAPGEF4,CLTC,DLG2,PTPRD,PLCB1,MAGI2,SCN2A,ASIC2,DOCK10,PTPRA,RGS9,MPDZ,DLGAP2,DCC,CTNNA2,CEP112,NRG1*
GO:0071944	cell periphery	9.429879863723904e-23	*ABCC6,PTPRR,EPB41L4B,EPN2,DIP2A,TRPM6,SLC8A1,ANO5,PCDHA13,GRIK2,SLC22A14,FGF1,ARHGAP15,ASTN2,LRP1B,MKLN1,USH2A,NEGR1,PCDHA12,NRXN1,MAGI1,GNGT1,DPP6,LMO7,GRM3,EXOC4,BBS9,NTRK2,NBEA,RASGEF1B,TMX3,NRG3,PTPRG,CALCR,ELMO1,NCOA1,ATP2B4,PHEX,PDE4DIP,TRPM3,FBLN7,RAPGEF5,FGF13,PATJ,GPC3,DMD,ADCY2,KCNQ3,CDH19,CHRM3,ZNF804A,NALF1,KAZN,BRAF,ERBB4,GPHN,MAP3K5,DSCAML1,HTR2C,TBC1D5,CNTN4,RAP1GAP2,HMCN1,ITGA1,SGIP1,MYO16,PCDHA2,PCDHA11,PCLO,CDH18,FRMD6,ANK2,ANO2,PCDHA9,ROBO1,DGKB,PCDHAC2,CDH6,CACNA2D3,PCDHA10,EDIL3,LRRTM4,PTPRN2,DSCAM,DGKI,COL28A1,COLEC10,LARGE1,SDK1,EPHA6,IGF1R,SPTB,NLGN1,ANK1,CNTNAP2,KCNIP4,CFTR,NOS1AP,OTOGL,PCDH9,NKAIN2,ATP6V0D2,PCDHA1,PCDHA8,CDH12,SHC4,DDR2,ADAM22,GPC6,CDH4,DPP10,CNTN6,PARM1,FBLN5,ATP9B,CACNA2D1,PCDHAC1,HERC2,LRFN5,UTRN,GPC5,GHR,PCDH7,LRRC4C,PPP1R9A,TMEM117,NKAIN3,DYNC1H1,LANCL2,EDAR,RIMBP2,FHIT,PCDH15,SGCZ,ACE2,CHMP3,KCNAB1,PDE4D,CNTN5,ITGB8,RIMS1,WWOX,PCDHA7,LSAMP,DIAPH3,PCDHA5,PLCB4,FTO,TNFSF11,PPFIA2,ENOX1,WNT2,OR8B8,STEAP1B,CALN1,NTNG1,SGCD,GRM7,PTPRM,PRKCH,NRXN3,DLC1,DENND2B,ATP8A2,COMT,PCDHA6,KMT2E,SYNE2,DOCK2,CDH13,MDGA2,FBN2,RFTN1,PCDHA4,SNTG1,ALK,CACHD1,SLC8A3,MYO5B,CADM2,TENT4B,DCLK1,CDH2,TENM2,KCNB2,ADGRL4,RGS7,SRGAP2,PTPRT,SCEL,FREM2,RAPGEF4,CLTC,THSD7B,DLG2,PTPRD,PRKG1,MAGI2,PXDNL,SCN2A,ASIC2,MICAL3,ADK,RYR2,LDLRAD3,PTPRA,FAT3,PCDHA3,RGS9,MPDZ,EYS,ROBO2,SLC39A11,KSR2,DLGAP2,BMPR1B,DCC,CTNNA2,DNER,ETV6,CEP112,NRG1*
GO:0045	synapse	1.203836612	*DIP2A,SLC8A1,GRIK2,CADPS2,KALRN,MKLN1,USH*

202		1603144e-21	*2A,FGF12,BTBD9,NRXN1,GRM3,EXOC4,NTRK2,NBEA,NRG3,ATP2B4,DMD,KCNQ3,CHRM3,ZNF804A,BRAF,ERBB4,GPHN,DSCAML1,HTR2C,PCLO,ANK2,ANO2,DGKB,CDH6,LRRTM4,PTPRN2,DSCAM,DGKI,LARGE1,SDK1,SPTB,NLGN1,IQSEC1,ANK1,CNTNAP2,NOS1AP,SHC4,ADAM22,GPC6,CELF4,CNTN6,PCSK1,LRFN5,UTRN,LRRC4C,PPP1R9A,SLC35F1,FRMPD4,RIMBP2,PCDH15,CNTN5,RIMS1,DIAPH3,PLCB4,PPFIA2,NTNG1,GRM7,NRXN3,PLEKHA5,COMT,MCTP1,CDH13,SEMA3A,SLC8A3,CADM2,DCLK1,CDH2,TENM2,CADPS,SRGAP2,PTPRT,RAPGEF4,DLG2,PTPRD,PLCB1,MAGI2,SCN2A,ASIC2,DOCK10,PTPRA,RGS9,MPDZ,DLGAP2,DCC,CEP112,NRG1*
GO:0005886	plasma membrane	1.5183216565054413e-20	*ABCC6,PTPRR,EPB41L4B,EPN2,DIP2A,TRPM6,SLC8A1,ANO5,PCDHA13,GRIK2,SLC22A14,ARHGAP15,LRP1B,USH2A,NEGR1,PCDHA12,NRXN1,MAGI1,GNGT1,DPP6,LMO7,GRM3,EXOC4,BBS9,NTRK2,NBEA,RASGEF1B,TMX3,NRG3,PTPRG,CALCR,ELMO1,NCOA1,ATP2B4,PHEX,TRPM3,RAPGEF5,FGF13,PATJ,GPC3,DMD,ADCY2,KCNQ3,CDH19,CHRM3,ZNF804A,NALF1,KAZN,BRAF,ERBB4,GPHN,MAP3K5,DSCAML1,HTR2C,TBC1D5,CNTN4,RAP1GAP2,HMCN1,ITGA1,SGIP1,MYO16,PCDHA2,PCDHA11,CDH18,FRMD6,ANK2,ANO2,PCDHA9,ROBO1,DGKB,PCDHAC2,CDH6,CACNA2D3,PCDHA10,LRRTM4,PTPRN2,DSCAM,DGKI,COLEC10,LARGE1,SDK1,EPHA6,IGF1R,SPTB,NLGN1,ANK1,CNTNAP2,KCNIP4,CFTR,NOS1AP,PCDH9,NKAIN2,ATP6V0D2,PCDHA1,PCDHA8,CDH12,SHC4,DDR2,ADAM22,GPC6,CDH4,DPP10,CNTN6,PARM1,ATP9B,CACNA2D1,PCDHAC1,HERC2,LRFN5,UTRN,GPC5,GHR,PCDH7,LRRC4C,TMEM117,NKAIN3,LANCL2,EDAR,RIMBP2,FHIT,PCDH15,SGCZ,ACE2,CHMP3,KCNAB1,PDE4D,CNTN5,ITGB8,RIMS1,WWOX,PCDHA7,LSAMP,DIAPH3,PCDHA5,PLCB4,FTO,TNFSF11,PPFIA2,ENOX1,OR8B8,STEAP1B,CALN1,NTNG1,SGCD,GRM7,PTPRM,PRKCH,NRXN3,DLC1,DENND2B,ATP8A2,COMT,PCDHA6,KMT2E,SYNE2,DOCK2,CDH13,MDGA2,RFTN1,PCDHA4,SNTG1,ALK,CACHD1,SLC8A3,CADM2,TENT4B,DCLK1,CDH2,TENM2,KCNB2,ADGRL4,RGS7,SRGAP2,PTPRT,SCEL,FREM2,RAPGEF4,CLTC,THSD7B,DLG2,PTPRD,PRKG1,MAGI2,PXDNL,SCN2A,ASIC2,MICAL3,ADK,RYR2,LDLRAD3,PTPRA,FAT3,PCDHA3,RGS9,MPDZ,ROBO2,SLC39A11,KSR2,DLGAP2,BMPR1B,DCC,CTNNA2,DNER,ETV6,CEP112,NRG1*
GO:0043005	neuron projection	1.0000005502916313e-16	*DIP2A,SLC8A1,GRIK2,USH2A,NEGR1,NRXN1,GNGT1,GRM3,EXOC4,NTRK2,CALCR,FGF13,DMD,ADCY2,KCNQ3,CHRM3,ZNF804A,BRAF,GPHN,MYO3B,DSCAML1,HTR2C,CNTN4,PCLO,ANK2,ANO2,ROBO1,DSCAM,DGKI,EPHA6,IGF1R,NLGN1,ANK1,CNTNAP2,KCNIP4,PCDH9,ADAM22,CNTN6,PCSK1,AGBL4,PPP1R9A,DYNC1H1,FRMPD4,BRINP3,PCDH15,KCNAB1,CNTN5,PLCB4,PPFIA2,GRM7,AUTS2,COMT,SDCCAG8,CDH13,SEMA3A,SLC8A3,CADM2,DCLK1,CDH2,TENM2,USP9X,KCNB2,RGS7,SRGAP2,DLG2,SACS,MAGI2,SCN2A,ASIC2,DOCK10,FAT3,RGS9,MPDZ,EYS,ROBO2,BMPR1B,DCC,CTNNA2,DNER*
GO:0016020	membrane	1.3465034013429077e-16	*ABCC6,PTPRR,EPB41L4B,EPN2,DIP2A,TRPM6,SLC8A1,RNF103-CHMP3,ANO5,PCDHA13,PREP,GRIK2,SLC22A14,ARHGAP15,TTC3,CADPS2,ASTN2,LRP1B,KALRN,USH2A,NEGR1,XXYLT1,PARP4,PCDHA12,NRXN1,MAGI1,GNGT1,ST6GALNAC3,DPP6,STK24,LMO7,GRM3,EXOC4,BBS9,NTRK2,NBEA,RASGEF1B,TMX3,NRG3,PTP*

			RG,CALCR,ELMO1,NCOA1,IRAG1,ATP2B4,PHEX,TRPM3,TMEM64,CNTNAP5,RAPGEF5,FGF13,PATJ,GPC3,DMD,ADCY2,KCNQ3,CDH19,ATRNL1,CHRM3,SLIT2,ZNF804A,FAM20C,NALF1,KAZN,BRAF,B3GNT5,ERBB4,GPHN,DOP1B,EXT2,MAP3K5,STAG2,DSCAML1,HTR2C,GALNT13,TBC1D5,CNTN4,RAP1GAP2,PIK3C3,HMCN1,ITGA1,SGIP1,MYO16,PCDHA2,PCDHA11,PCLO,CDH18,FRMD6,ANK2,ANO2,PCDHA9,ROBO1,DGKB,PRRC2C,PCDHAC2,CDH6,CHODL,CACNA2D3,PCDHA10,LRRTM4,PTPRN2,INPP4B,DSCAM,DGKI,COLEC10,LARGE1,SDK1,EPHA6,IGF1R,SPTB,NLGN1,TMEM260,IQSEC1,ANK1,PPM1L,CNTNAP2,KCNIP4,CFTR,NOS1AP,DOP1A,PCDH9,NKAIN2,ATP6V0D2,PCDHA1,PCDHA8,CDH12,SHC4,DDR2,ADAM22,GPC6,CDH4,DPP10,ST8SIA6,CNTN6,PARM1,ATP9B,MITF,CACNA2D1,PCSK1,PCDHAC1,HERC2,LRFN5,UTRN,GPC5,OGDH,GHR,PCDH7,LRRC4C,TMEM117,NKAIN3,DYNC1H1,NAALADL2,OSBPL10,LANCL2,MRPS28,SLC35F1,GALNT17,EDAR,RIMBP2,FHIT,WIPI2,PCDH15,SGCZ,ACE2,CHMP3,KCNAB1,PDE4D,CNTN5,ITGB8,PDE3A,RIMS1,WWOX,PCDHA7,LSAMP,DIAPH3,TMEM178B,CPLANE1,PCDHA5,PLCB4,FTO,TNFSF11,PPFIA2,ENOX1,OR8B8,STEAP1B,MGAT4C,CALN1,UXS1,NTNG1,SGCD,GRM7,STS,NAV3,PTPRM,PRKCH,NRXN3,DLC1,DENND2B,ATP8A2,CERS6,LPIN1,PLEKHA5,SYT16,COMT,PCDHA6,MCTP1,NBAS,ZPLD1,KMT2E,SYNE2,DOCK2,CDH13,MDGA2,RFTN1,PCDHA4,SNTG1,ALK,CACHD1,SLC8A3,MYO5B,CADM2,MALRD1,TENT4B,DCLK1,KIF16B,CDH2,TENM2,VPS41,KIAA1549L,USP9X,KCNB2,ADGRL4,LYST,CADPS,RGS7,SRGAP2,SPECC1,PTPRT,CSMD1,SCEL,FREM2,RAPGEF4,CLTC,THSD7B,SORCS1,DLG2,PTPRD,PLCB1,PRKG1,BRIP1,MAGI2,GALNTL6,PXDNL,SCN2A,ASIC2,DOCK10,MICAL3,ADK,TMEM164,RYR2,LDLRAD3,PTPRA,FAT3,MSH2,PCDHA3,MGAT4A,RGS9,MPDZ,EYS,ROBO2,SLC39A11,KSR2,DLGAP2,BMPR1B,DCC,CTNNA2,PEX5L,DNER,ETV6,CEP112,NRG1,SLC25A48
GO:0042995	cell projection	6.517552356592586e-16	*DIP2A,TRPM6,SLC8A1,GRIK2,SLC22A14,MKLN1,USH2A,NEGR1,NRXN1,MAGI1,GNGT1,GRM3,EXOC4,DNAH7,BBS9,NTRK2,CALCR,ATP2B4,DNAH8,FGF13,DMD,ADCY2,KCNQ3,CHRM3,ARHGAP24,ZNF804A,BRAF,GPHN,MYO3B,SPAG16,DSCAML1,HTR2C,CNTN4,PIK3C3,PCLO,ANK2,ANO2,ROBO1,DSCAM,DGKI,EPHA6,IGF1R,SPTB,NLGN1,ANK1,CNTNAP2,KCNIP4,NOS1AP,PCDH9,ADAM22,CNTN6,SPEF2,PCSK1,UTRN,AGBL4,PPP1R9A,DYNC1H1,FRMPD4,BRINP3,PCDH15,ACE2,KCNAB1,CNTN5,RIMS1,WWOX,DIAPH3,CPLANE1,PLCB4,PPFIA2,GRM7,PTPRM,NRXN3,DLC1,ATP8A2,AUTS2,COMT,SYNE2,SDCCAG8,CDH13,TTLL6,SNTG1,SEMA3A,SLC8A3,CADM2,DCLK1,CDH2,TENM2,USP9X,KCNB2,CDK6,RGS7,SRGAP2,CTNNA3,DLG2,SACS,MAGI2,SCN2A,ASIC2,DOCK10,MICAL3,FAT3,RGS9,MPDZ,EYS,ROBO2,BMPR1B,DCC,CTNNA2,DNER,DNAH5*
GO:0120025	plasma membrane bounded cell projection	1.0750903132859037e-13	*DIP2A,TRPM6,SLC8A1,GRIK2,SLC22A14,MKLN1,USH2A,NEGR1,NRXN1,GNGT1,GRM3,EXOC4,DNAH7,BBS9,NTRK2,CALCR,ATP2B4,DNAH8,FGF13,DMD,ADCY2,KCNQ3,CHRM3,ZNF804A,BRAF,GPHN,MYO3B,SPAG16,DSCAML1,HTR2C,CNTN4,PIK3C3,PCLO,ANK2,ANO2,ROBO1,DSCAM,DGKI,EPHA6,IGF1R,NLGN1,ANK1,CNTNAP2,KCNIP4,NOS1AP,PCDH9,ADAM22,CNTN6,SPEF2,PCSK1,UTRN,AGBL4,PPP1R9A,DYNC1H1,FRMPD4,BRINP3,PCDH15,ACE2,KCNAB1,CNTN*

			5,WWOX,CPLANE1,PLCB4,PPFIA2,GRM7,PTPRM,DLC1,AUTS2,COMT,SYNE2,SDCCAG8,CDH13,TTLL6,SNTG1,SEMA3A,SLC8A3,CADM2,DCLK1,CDH2,TENM2,USP9X,KCNB2,CDK6,RGS7,SRGAP2,CTNNA3,DLG2,SACS,MAGI2,SCN2A,ASIC2,DOCK10,FAT3,RGS9,MPDZ,EYS,ROBO2,BMPR1B,DCC,CTNNA2,DNER,DNAH5
GO:0098794	postsynapse	1.3498846590357754e-13	*DIP2A,SLC8A1,GRIK2,KALRN,MKLN1,GRM3,NTRK2,NBEA,DMD,CHRM3,ZNF804A,ERBB4,GPHN,PCLO,ANK2,DGKB,LRRTM4,DGKI,SPTB,NLGN1,IQSEC1,ANK1,SHC4,ADAM22,CELF4,LRFN5,UTRN,LRRC4C,PPP1R9A,FRMPD4,PLCB4,PPFIA2,GRM7,PLEKHA5,SLC8A3,DCLK1,CDH2,TENM2,SRGAP2,PTPRT,DLG2,PLCB1,MAGI2,ASIC2,DOCK10,RGS9,MPDZ,DLGAP2,DCC*
GO:0097060	synaptic membrane	1.9555039021298487e-13	*GRIK2,NRXN1,GRM3,NBEA,ATP2B4,DMD,CHRM3,ERBB4,GPHN,ANK2,ANO2,DGKB,LRRTM4,DGKI,NLGN1,ANK1,CNTNAP2,SHC4,ADAM22,CNTN6,LRFN5,UTRN,LRRC4C,CNTN5,RIMS1,PPFIA2,NTNG1,GRM7,NRXN3,TENM2,SRGAP2,PTPRT,DLG2,PTPRD,SCN2A,PTPRA,RGS9,DCC*
GO:0098978	glutamatergic synapse	7.597451629207898e-12	*GRIK2,CADPS2,BTBD9,NRXN1,GRM3,NBEA,NRG3,ATP2B4,ERBB4,PCLO,ANO2,DGKB,CDH6,DGKI,SPTB,NLGN1,NOS1AP,ADAM22,GPC6,LRFN5,LRRC4C,FRMPD4,PLCB4,PPFIA2,NTNG1,NRXN3,PLEKHA5,SEMA3A,CADPS,PTPRT,PTPRD,PLCB1,SCN2A,DOCK10,RGS9,DLGAP2,NRG1*
GO:0098984	neuron to neuron synapse	1.609840500346437e-11	*SLC8A1,GRIK2,KALRN,NRXN1,GRM3,NTRK2,DMD,ERBB4,GPHN,PCLO,DGKI,NLGN1,IQSEC1,ADAM22,LRFN5,LRRC4C,PPP1R9A,FRMPD4,PLCB4,GRM7,PLEKHA5,SLC8A3,DCLK1,CDH2,SRGAP2,PTPRT,RAPGEF4,DLG2,PTPRD,MAGI2,RGS9,MPDZ,DLGAP2,DCC*
GO:0032279	asymmetric synapse	2.2742711249938524e-10	*SLC8A1,GRIK2,KALRN,GRM3,NTRK2,DMD,ERBB4,GPHN,PCLO,DGKI,NLGN1,IQSEC1,ADAM22,LRFN5,LRRC4C,PPP1R9A,FRMPD4,PLCB4,GRM7,PLEKHA5,SLC8A3,DCLK1,CDH2,SRGAP2,PTPRT,DLG2,MAGI2,RGS9,MPDZ,DLGAP2,DCC*
GO:0030424	axon	2.3803016960489515e-10	*SLC8A1,GRIK2,USH2A,NRXN1,GRM3,EXOC4,NTRK2,CALCR,FGF13,DMD,KCNQ3,ZNF804A,DSCAML1,CNTN4,PCLO,ROBO1,DSCAM,DGKI,IGF1R,ANK1,CNTNAP2,PCDH9,ADAM22,CNTN6,PCSK1,AGBL4,DYNC1H1,KCNAB1,CNTN5,PPFIA2,GRM7,AUTS2,COMT,SEMA3A,SLC8A3,CADM2,TENM2,USP9X,DLG2,SACS,SCN2A,ROBO2,DCC,CTNNA2*
GO:0014069	postsynaptic density	3.5842389763254404e-10	*SLC8A1,GRIK2,KALRN,GRM3,NTRK2,DMD,ERBB4,GPHN,PCLO,DGKI,NLGN1,IQSEC1,ADAM22,LRFN5,LRRC4C,PPP1R9A,FRMPD4,PLCB4,PLEKHA5,SLC8A3,DCLK1,CDH2,SRGAP2,PTPRT,DLG2,MAGI2,RGS9,MPDZ,DLGAP2,DCC*
GO:0099572	postsynaptic specialization	5.745642053061406e-10	*SLC8A1,GRIK2,KALRN,GRM3,NTRK2,DMD,ERBB4,GPHN,PCLO,DGKI,NLGN1,IQSEC1,ADAM22,LRFN5,LRRC4C,PPP1R9A,FRMPD4,PLCB4,PPFIA2,PLEKHA5,SLC8A3,DCLK1,CDH2,SRGAP2,PTPRT,DLG2,MAGI2,RGS9,MPDZ,DLGAP2,DCC*
GO:0098590	plasma membrane region	5.2557215694396425e-9	*ABCC6,TRPM6,GRIK2,USH2A,NRXN1,LMO7,GRM3,BBS9,NBEA,ATP2B4,PATJ,DMD,CHRM3,ERBB4,GPHN,HMCN1,FRMD6,ANK2,ANO2,DGKB,LRRTM4,DGKI,IGF1R,NLGN1,ANK1,CNTNAP2,CFTR,NOS1AP,ATP6V0D2,SHC4,DDR2,ADAM22,CNTN6,LRFN5,UTRN,LRRC4C,ACE2,PDE4D,CNTN5,RIMS1,DIAPH3,PPFIA2,NTNG1,GRM7,NRXN3,DLC1,SYNE2,CDH13,SNTG1,CDH2,TENM2,SRGAP2,PTPRT,DLG2,PTPRD,SCN2A,PTPRA,RGS9,MPDZ,ROBO2,DCC,NRG1*

GO:0036477	somatodendritic compartment	1.0921186600740296e-8	*DIP2A,SLC8A1,GRIK2,ASTN2,USH2A,NEGR1,NRXN1,GRM3,NTRK2,FGF13,DMD,ADCY2,CHRM3,ZNF804A,GPHN,HTR2C,ITGA1,DSCAM,DGKI,EPHA6,IGF1R,NLGN1,CNTNAP2,KCNIP4,PCSK1,GHR,PPP1R9A,FRMPD4,BRINP3,PLCB4,PPFIA2,GRM7,COMT,SEMA3A,SLC8A3,CADM2,TENM2,KCNB2,SRGAP2,DLG2,SACS,MAGI2,ASIC2,DOCK10,FAT3,MPDZ,BMPR1B,DNER*
GO:0030425	dendrite	5.116333802518192e-8	*DIP2A,SLC8A1,GRIK2,NEGR1,GRM3,NTRK2,FGF13,ADCY2,CHRM3,ZNF804A,GPHN,HTR2C,DSCAM,DGKI,EPHA6,NLGN1,CNTNAP2,KCNIP4,PCSK1,PPP1R9A,FRMPD4,BRINP3,PLCB4,PPFIA2,GRM7,COMT,SEMA3A,SLC8A3,TENM2,KCNB2,SRGAP2,SACS,MAGI2,ASIC2,DOCK10,FAT3,MPDZ,BMPR1B,DNER*
GO:0097447	dendritic tree	5.6146451283796616e-8	*DIP2A,SLC8A1,GRIK2,NEGR1,GRM3,NTRK2,FGF13,ADCY2,CHRM3,ZNF804A,GPHN,HTR2C,DSCAM,DGKI,EPHA6,NLGN1,CNTNAP2,KCNIP4,PCSK1,PPP1R9A,FRMPD4,BRINP3,PLCB4,PPFIA2,GRM7,COMT,SEMA3A,SLC8A3,TENM2,KCNB2,SRGAP2,SACS,MAGI2,ASIC2,DOCK10,FAT3,MPDZ,BMPR1B,DNER*
GO:0045211	postsynaptic membrane	3.70531396455598e-7	*GRIK2,GRM3,NBEA,DMD,CHRM3,ERBB4,GPHN,ANK2,DGKB,LRRTM4,NLGN1,ANK1,SHC4,ADAM22,LRFN5,UTRN,LRRC4C,GRM7,TENM2,SRGAP2,PTPRT,DLG2,RGS9,DCC*
GO:0042383	sarcolemma	7.746347246152229e-7	*SLC8A1,ATP2B4,FGF13,DMD,ANK2,IGF1R,ANK1,NOS1AP,CACNA2D1,UTRN,SGCZ,SGCD,SLC8A3,CDH2,PRKG1,SCN2A,RYR2*
GO:0098793	presynapse	0.00000213475323968141 02	*SLC8A1,GRIK2,CADPS2,USH2A,NRXN1,GRM3,NTRK2,ATP2B4,DMD,ZNF804A,BRAF,ERBB4,PCLO,ANO2,PTPRN2,DGKI,NLGN1,IQSEC1,CNTN6,PCSK1,SLC35F1,CNTN5,RIMS1,PPFIA2,NTNG1,GRM7,NRXN3,MCTP1,SLC8A3,CDH2,CADPS,PTPRD,SCN2A,RGS9*
GO:0005856	cytoskeleton	0.0000147983325326058 28	*EPB41L4B,RNF103-CHMP3,KALRN,USH2A,PARP4,EXOC4,DNAH7,BBS9,PDE4DIP,DNAH8,FGF13,PATJ,DMD,ARHGAP24,ZNF804A,KAZN,KIF26B,GPHN,MYO3B,DIAPH2,STAG2,SPAG16,RAP1GAP2,PIK3C3,MYO16,PCLO,FRMD6,ANK2,NEK1,CHODL,SPTB,ANK1,DYNC1I1,NOS1AP,TBCK,DDR2,SPEF2,HERC2,UTRN,AGBL4,PPP1R9A,DYNC1H1,NEBL,OSBPL10,LANCL2,FRMPD4,SGCZ,CHMP3,PDE4D,RIMS1,DIAPH3,SFI1,SGCD,NAV3,DLC1,AUTS2,BCAS3,KMT2E,SYNE2,DOCK2,SDCCAG8,SSH2,LMNTD1,TTLL6,SNTG1,YEATS2,MYO5B,KIF16B,CDH2,VPS41,USP9X,CDK6,LYST,DDX60,SPECC1,CTNNA3,CLTC,MAGI2,MICAL3,EYS,SLF1,DLGAP2,CTNNA2,CEP112,DNAH5*
GO:0098797	plasma membrane protein complex	0.00002709128121252555	*GRIK2,GNGT1,DPP6,CALCR,DMD,KCNQ3,CDH19,TBC1D5,ITGA1,SGIP1,CDH18,CDH6,CACNA2D3,IGF1R,CNTNAP2,KCNIP4,ATP6V0D2,CDH12,CDH4,DPP10,CACNA2D1,UTRN,SGCZ,KCNAB1,PDE4D,ITGB8,SGCD,CDH13,SNTG1,CACHD1,CDH2,KCNB2,CLTC,DLG2,SCN2A,BMPR1B,CTNNA2*
GO:0005737	cytoplasm	0.00003056532561144941	*ABCC6,PTPRR,EPB41L4B,EPN2,PIR,SUGCT,DIP2A,SLC8A1,RNF103-CHMP3,ANO5,PREP,RSRC1,GRIK2,DPYD,SLC22A14,KDM4B,FGF1,ZBTB20,ARHGAP15,AHR,TTC3,CADPS2,ASTN2,KALRN,MKLN1,SUMF1,USH2A,XXYLT1,FGF12,BTBD9,PARP4,MAST4,NRXN1,AGBL1,MAGI1,FARS2,ST6GALNAC3,FAM13A,STK24,LMO7,EXOC4,DNAH7,BBS9,NTRK2,ANXA10,NBEA,RASGEF1B,TMX3,CALCR,ELMO1,NCOA1,IRAG1,ATP2B4,PHEX,SOX6,PDE4DIP,TMEM64,DNAH8,FGF13,PATJ,GPC3,DMD,ADCY2,KCNQ3,CHRM3,ARHGAP24,SLIT2,ZNF804*

			A,FAM20C,KAZN,BRAF,BABAM2,KMT2C,B3GNT5,ERBB4,KIF26B,GPHN,DOP1B,SH2D1A,MYO3B,EXT2,DIAPH2,MAP3K5,STAG2,SPAG16,GALNT13,TBC1D5,RAP1GAP2,PIK3C3,HMCN1,ITGA1,TCF12,SGIP1,ARMC8,MYO16,PCDHA2,PCLO,FRMD6,ANK2,ROBO1,NEK1,DGKB,PRRC2C,DOK6,JAZF1,CHODL,CPED1,FGF14,PTPRN2,INPP4B,DGKI,COL28A1,DENND1B,COLEC10,LARGE1,CCDC33,WDR72,SPTB,NLGN1,TMEM260,IQSEC1,ANK1,PPM1L,CASZ1,CNTNAP2,TRIM71,KCNIP4,CFTR,DYNC1I1,NOS1AP,DOP1A,RBMS3,ATP6V0D2,PCDHA1,TBCK,LARS2,GPC6,MCF2L2,CELF4,ST8SIA6,PARM1,SPEF2,ZNF638,ATP9B,MITF,CACNA2D1,PCSK1,HERC2,UTRN,GPC5,AGBL4,OGDH,GHR,RBFOX1,PCDH7,PPP1R9A,TMEM117,SMAD9,DYNC1H1,CRPPA,NEBL,OSBPL10,LANCL2,ZFPM2,MRPS28,SLC35F1,GALNT17,BRINP3,FHIT,WIPI2,DNMT3A,PCDH15,SGCZ,ACE2,CHMP3,KCNAB1,PDE4D,PDE3A,RIMS1,WWOX,BEND5,ANAPC10,LSAMP,MIPEP,DIAPH3,PLCB4,RNF216,FTO,UGGT2,TNFSF11,PPFIA2,WNT2,STEAP1B,SFI1,MGAT4C,CALN1,UXS1,SGCD,GRM7,PTPRM,PRKCH,DLC1,DENND2B,ATP8A2,CERS6,LPIN1,PLEKHA5,AUTS2,BCAS3,ADARB2,COMT,MCTP1,NBAS,ZPLD1,KMT2E,SYNE2,DOCK2,SDCCAG8,SSH2,LMNTD1,CDH13,DAB1,TTLL6,RFTN1,SNTG1,SLC8A3,MYO5B,MALRD1,TENT4B,DCLK1,KIF16B,CDH2,TENM2,VPS41,HDAC9,USP9X,USP13,NBPF1,CDK6,ADGRL4,LYST,CADPS,DDX60,RGS7,SRGAP2,PPP2R2C,SPECC1,GABPB1,SCEL,CTNNA3,RAPGEF4,CLTC,SORCS1,CAMKMT,RNF220,ARHGEF37,DLG2,SACS,PLCB1,NPEPPS,PRKG1,BRIP1,MAGI2,NELL1,PLCL1,GALNTL6,PXDNL,DOCK10,MICAL3,ADK,RANBP17,GTDC1,RYR2,MGAT4A,KLHDC10,RGS9,MPDZ,EYS,SLF1,SLC39A11,KSR2,DLGAP2,DCC,CTNNA2,PEX5L,DNER,ETV6,CEP112,PGM3,SLC25A48,DNAH5,FANCB
GO:0016342	catenin complex	0.0000322354339872956 44	*CDH19,CDH18,CDH6,CDH12,CDH4,CDH13,CDH2,CTNNA2*
GO:0005911	cell-cell junction	0.00012148704595400182	*EPB41L4B,SLC8A1,MAGI1,LMO7,FGF13,PATJ,CDH19,ARHGAP24,KAZN,HMCN1,CDH18,FRMD6,ANK2,CDH6,CNTNAP2,PCDH9,CDH12,CDH4,PTPRM,PRKCH,SDCCAG8,CDH13,CDH2,CTNNA3,DLG2,MAGI2,SCN2A,MPDZ,CTNNA2*
GO:0070161	anchoring junction	0.00013869982644558836	*EPB41L4B,SLC8A1,MAGI1,LMO7,FBLN7,FGF13,PATJ,DMD,CDH19,ARHGAP24,KAZN,HMCN1,ITGA1,CDH18,FRMD6,ANK2,CDH6,CNTNAP2,NOS1AP,PCDH9,CDH12,DDR2,CDH4,ITGB8,PTPRM,PRKCH,DLC1,SYNE2,SDCCAG8,SSH2,CDH13,SLC8A3,CDH2,CTNNA3,CLTC,DLG2,MAGI2,SCN2A,PTPRA,MPDZ,CTNNA2*
GO:0042734	presynaptic membrane	0.00030558592040026667	*GRIK2,NRXN1,GRM3,ATP2B4,ERBB4,ANO2,CNTN6,CNTN5,RIMS1,PPFIA2,NTNG1,NRXN3,PTPRD,SCN2A,RGS9*
GO:0005912	adherens junction	0.0013467565391743971	*MAGI1,LMO7,CDH19,ARHGAP24,HMCN1,CDH18,CDH6,CDH12,CDH4,PTPRM,CDH13,CDH2,CTNNA3,DLG2,CTNNA2*
GO:0098982	GABA-ergic synapse	0.001669272899438719	*NRXN1,NBEA,ERBB4,PCLO,NLGN1,LRFN5,CNTN5,NRXN3,CDH13,PLCB1*
GO:0043197	dendritic spine	0.002236508284909288	*DIP2A,GRM3,NTRK2,ZNF804A,DGKI,NLGN1,PPP1R9A,FRMPD4,PPFIA2,SLC8A3,TENM2,SRGAP2,ASIC2,DOCK10*
GO:0044309	neuron spine	0.0029144101117369757	*DIP2A,GRM3,NTRK2,ZNF804A,DGKI,NLGN1,PPP1R9A,FRMPD4,PPFIA2,SLC8A3,TENM2,SRGAP2,ASIC*

			2,DOCK10
GO:0098796	membrane protein complex	0.003329470022913015	*EPN2,GRIK2,GNGT1,DPP6,CALCR,DMD,KCNQ3,CDH19,EXT2,MAP3K5,TBC1D5,PIK3C3,ITGA1,SGIP1,CDH18,ANO2,CDH6,CACNA2D3,IGF1R,ANK1,CNTNAP2,KCNIP4,CFTR,ATP6V0D2,CDH12,CDH4,DPP10,CACNA2D1,UTRN,SGCZ,CHMP3,KCNAB1,PDE4D,ITGB8,DIAPH3,SGCD,SYNE2,CDH13,SNTG1,CACHD1,CDH2,VPS41,KCNB2,CLTC,DLG2,SCN2A,RYR2,BMPR1B,CTNNA2*
GO:0016010	dystrophin-associated glycoprotein complex	0.006083686686905698	*DMD,UTRN,SGCZ,SGCD,SNTG1*
GO:0099634	postsynaptic specialization membrane	0.008380541915048325	*GRIK2,ERBB4,GPHN,NLGN1,ADAM22,LRFN5,LRRC4C,PTPRT,DLG2,RGS9,DCC*
GO:0034703	cation channel complex	0.011496748110647386	*GRIK2,DPP6,KCNQ3,CACNA2D3,CNTNAP2,KCNIP4,DPP10,CACNA2D1,KCNAB1,PDE4D,CACHD1,KCNB2,DLG2,SCN2A,RYR2*
GO:0019897	extrinsic component of plasma membrane	0.014085242164790858	*GNGT1,NBEA,CDH19,CDH18,CDH6,CDH12,CDH4,KCNAB1,CDH13,CDH2,CTNNA2*
GO:0005938	cell cortex	0.015265310558251541	*FGF1,ASTN2,MKLN1,EXOC4,PDE4DIP,HMCN1,PCLO,SPTB,PPP1R9A,DYNC1H1,LANCL2,RIMS1,GRM7,DLC1,DENND2B,MYO5B,CDH2,MICAL3*
GO:0030175	filopodium	0.015524395008529592	*FGF13,DMD,MYO3B,NLGN1,NOS1AP,UTRN,PPP1R9A,DYNC1H1,SYNE2,TENM2*
GO:0044304	main axon	0.018005670199847928	*KCNQ3,ANK1,CNTNAP2,ADAM22,KCNAB1,DLG2,SCN2A,ROBO2*
GO:0030315	T-tubule	0.020070906122352825	*SLC8A1,ATP2B4,ANK2,IGF1R,NOS1AP,CACNA2D1,SCN2A*
GO:0019898	extrinsic component of membrane	0.02195905944788505	*KALRN,GNGT1,NBEA,CDH19,PIK3C3,CDH18,CDH6,CDH12,CDH4,WIPI2,KCNAB1,CDH13,CDH2,CTNNA2*
GO:0090665	glycoprotein complex	0.022063410747230665	*DMD,UTRN,SGCZ,SGCD,SNTG1*
GO:0150034	distal axon	0.03833972726708947	*SLC8A1,GRIK2,USH2A,NRXN1,EXOC4,NTRK2,FGF13,ZNF804A,DSCAM,PCDH9,PCSK1,AUTS2,SLC8A3,TENM2,USP9X,DCC*
GO:0043025	neuronal cell body	0.041044959007708826	*SLC8A1,GRIK2,ASTN2,USH2A,NEGR1,NRXN1,DMD,ZNF804A,ITGA1,DSCAM,IGF1R,CNTNAP2,KCNIP4,PCSK1,GHR,BRINP3,SLC8A3,CADM2,KCNB2,DLG2,ASIC2,BMPR1B,DNER*
GO:0034705	potassium channel complex	0.042686186386168445	*GRIK2,DPP6,KCNQ3,CNTNAP2,KCNIP4,DPP10,KCNAB1,KCNB2,DLG2*
GO:0012505	endomembrane system	0.0447737434016821	*ABCC6,EPN2,RNF103-CHMP3,ANO5,TTC3,ASTN2,SUMF1,XXYLT1,NRXN1,ST6GALNAC3,STK24,LMO7,NTRK2,NBEA,RASGEF1B,TMX3,CALCR,IRAG1,PHEX,PDE4DIP,TMEM64,GPC3,DMD,CHRM3,FAM20C,B3GNT5,DOP1B,EXT2,DIAPH2,MAP3K5,GALNT13,TBC1D5,RAP1GAP2,PIK3C3,ITGA1,SGIP1,ARMC8,PCDHA2,ANK2,CHODL,CPED1*

			,PTPRN2,DGKI,COL28A1,COLEC10,LARGE1,WDR72,NLGN1,TMEM260,IQSEC1,ANK1,PPM1L,CNTNAP2,KCNIP4,CFTR,DYNC1I1,NOS1AP,DOP1A,ATP6V0D2,PCDHA1,GPC6,ST8SIA6,PARM1,SPEF2,ATP9B,CACNA2D1,PCSK1,GPC5,AGBL4,RBFOX1,PCDH7,TMEM117,DYNC1H1,SLC35F1,GALNT17,ACE2,CHMP3,WWOX,BEND5,DIAPH3,PLCB4,UGGT2,PPFIA2,STEAP1B,MGAT4C,CALN1,UXS1,SGCD,NAV3,DLC1,DENND2B,ATP8A2,CERS6,LPIN1,MCTP1,NBAS,SYNE2,DOCK2,SSH2,LMNTD1,RFTN1,SLC8A3,MYO5B,MALRD1,KIF16B,CDH2,TENM2,VPS41,CADPS,RGS7,CLTC,SORCS1,CAMKMT,PLCB1,PRKG1,BRIP1,MAGI2,NELL1,GALNTL6,PXDNL,RANBP17,RYR2,MGAT4A,SLC39A11,DNER
GO:0098858	actin-based cell projection	0.04559173125180398	*USH2A,EXOC4,FGF13,DMD,MYO3B,NLGN1,NOS1AP,UTRN,PPP1R9A,DYNC1H1,PCDH15,WWOX,SYNE2,TENM2*
GO:0098839	postsynaptic density membrane	0.049783467396344046	*GRIK2,ERBB4,ADAM22,LRFN5,LRRC4C,PTPRT,DLG2,RGS9,DCC*

Table S5. GO associations with biological processes (BP), molecular functions (MF), and cellular components (CC) of 1797 rDNA-contacting genes detected only in Mel Z cells grown on Matrigel. Related to Figure 2A and D.

GO.ID	Description	padj	Genes
		MF	
GO:0005515	protein binding	1.5718313208976555e-7	*SLC18A1,KLHL13,ABCB7,PBX4,ACOT12,NHLRC3,MYO9B,COL4A5,ERG,PARN,STK16,XK,SEMA4D,MET,MAX,TNS3,SCYL3,TEAD1,EIF4G3,ACSM3,SNX16,IFTAP,SIAH3,DUS3L,CETN3,GAS2L1,CHMP1B,ELAPOR2,PRORP,ZNF534,TOX3,DNAH11,SLC9A9,CDC42BPA,POLR2J,POLG,EXOC2,TACR1,SDC2,CIITA,FANCC,ARAP1,ANKLE2,CEP85,RPRD1B,CIP2A,AHNAK,ACTR5,EIF4A3,DLEC1,NFE2L2,SCHIP1,TFEC,NFYC,H2BC18,UPP2,UBTD1,DTNB,VRK2,ITSN1,R3HDM2,ATP2B2,PREX2,ESPNL,SCRN1,FAM177A1,ELAVL2,TANC2,PSKH2,FBF1,NTN4,SERP1,POLR1A,ENKUR,RAB11FIP4,SNTA1,PPCDC,ASZ1,NUB1,SRGAP2C,WIF1,FLI1,SPATS2L,CLIC5,ESRRB,ARHGAP26,ZFHX2,RBP7,PTPN4,SETBP1,ZBTB7C,ITGA2,IL1RAPL1,KIF5C,BLK,GPM6A,SPATA16,WNT16,KRT86,SETD3,ADCY1,GRID1,RPS6KA2,EFR3B,TRIM24,ZDHHC17,HLCS,FIG4,AP1S3,CFAP47,FOXP2,FBXO34,KMT2D,LPAR3,SHE,ANO4,GIPC2,GFOD1,TXLNG,SP110,SH3BP2,SH3TC2,CEP290,TACC1,SH3PXD2B,OCLN,CFAP91,FASTKD5,NETO1,EGFR,PTPRQ,GBE1,TNPO1,SNX32,DNPEP,NEO1,SRGAP2B,STAG1,CLDN14,TIMM23,PLEKHB2,UBOX5,TCERG1,F10,FAM184A,CABIN1,PSMD10,FBXO47,FBXL17,PDZD2,EOLA1,DHX29,GPAM,IRF2,SPATA6L,LRP12,TBC1D22A,ESRP1,UBE2A,BRMS1L,RBM41,RGS12,CBX3,ACAP2,SLC39A10,ZNF496,CHCHD6,ZNF550,MPHOSPH9,KIZ,RNF10,FAT1,PIK3R1,IL17B,AGMO,NOVA1,SARNP,SHTN1,ATG12,FNDC3B,MASP1,CD200R1,CALD1,SH3GL2,CLBA1,HIBADH,CRTC1,VCF*

			1,FHIP1A,PITPNC1,SLC30A3,DOCK7,PDK3,FBXL4,ROR1,PAX5,AAK1,RTL8B,CRB1,UTP15,ARID1A,THEMIS,SMAD6,RYBP,NYAP2,PSD3,MEF2C,FZR1,ST8SIA5,DBNL,TBC1D19,ELP4,RYK,MACF1,PXT1,TUBGCP3,SLC15A5,TFDP2,PTPRE,MFSD6,SERINC3,TBC1D31,PTPN12,PTPRZ1,ACACA,PLN,EML1,CTDSPL2,SLC16A7,NEDD4,FLYWCH1,LRRC38,VWA8,CYSLTR2,PARVA,SECISBP2,URI1,SCML2,ANKRD28,CHD6,PRDX1,NDUFAF6,PACS2,ATG4B,AEBP2,SLC7A14,DSC2,SERPINA1,OLFM1,MID1,TSHZ2,POGK,SLC12A2,EFNB3,OTX1,TRIM37,DLG3,PROSER1,PVRIG,FLOT2,SRP68,SV2B,ILK,LEF1,EYA1,MAPK14,MYBPC3,WDR17,RAPGEF1,ALG13,UNC79,ZNF516,SLC1A2,ARHGEF3,GRIN2B,UBXN10,SVOP,IFNGR2,DENND4A,TBC1D20,OXR1,SFMBT2,SNX2,GRHPR,SCAMP5,IL17RB,PARP2,MINDY3,KLHL4,EIF3F,RDH16,FCHSD2,SLC4A7,DNAJB6,NPIPB3,SPCS1,MTUS1,LEMD3,PLEKHF2,PIEZO1,NEB,KANSL1L,KLHL32,TMEM100,NUP210,PDIA5,VDR,C16ORF74,NEK11,RTN4,GNL3L,CACNA1D,PRKAA2,ARHGEF11,IVNS1ABP,CD163,DPT,BBS2,CLSTN1,ZNF155,SERGEF,SCYL2,SKAP1,CHM,SKIC3,MYO3A,SERPINE2,NUBPL,PLAAT3,IL12RB2,PIK3CG,ANOS1,TPP1,STIL,ANKFN1,ATP6V1E2,NUP160,IQGAP1,MYCBP2,WIPI1,LATS1,PHYHIPL,KLHL12,SNX30,FANCI,ACAA1,PRSS23,WNT2B,LRRC37A3,EXD3,TNFRSF10B,MAP2,FBXW11,MTCL1,HSPH1,SUGP2,AZIN2,IYD,SMG6,HLA-DMA,PEMT,ARHGAP6,PPP2R2B,CHD5,JAK1,ITGA9,RADIL,KCNB1,HSD17B12,ANKRD27,AIM2,ANK3,ZNF19,AMT,KITLG,ARID4B,GMDS,PLEKHM2,NR1I2,ZNF266,RFX7,RELN,PDHB,ADAMTSL3,NXPH1,CRKL,TENT5A,ARHGEF10,SYTL3,SNAP91,CDKAL1,VAV3,RPS6KC1,CD40LG,VRK1,URM1,DPYSL3,ZNF420,SCN9A,SPRING1,SLC16A10,ATRN,CCDC88A,NDUFB9,SLC35B4,ARNT2,SPOCK1,HPSE2,PLCE1,TNIP3,TACC2,FRAS1,DCAF5,GNAL,CSTF1,STK32C,MSH3,BAK1,LAX1,STK26,ENOX2,TENM4,PPP3CA,CHRNA5,MKNK1,DYSF,NKD1,RBPJ,RIN2,PDE6C,PLEKHG7,ASCC1,CDH10,DENND1A,MZT1,PDZRN3,TASP1,MICU1,TAF10,MBD5,BACE2,KDM5A,ZNF549,NOMO1,ARL6IP6,CNOT6,PKIB,TBC1D9B,PXMP2,ASH1L,RSPO2,ACSF2,DISC1,PROX1,MAP3K7CL,FMN1,RALGPS1,CCDC6,VSIG1,LRRC42,ITGB1,RBM26,CYB561,DRC7,ZNF827,CORO1C,SPIDR,C17ORF80,NEFL,NAV2,STK3,CHN2,RNF150,C1GALT1,HNF4G,SMCO4,MSR1,EXOSC3,SH2D3C,APTX,TRPC4,RAMP3,GNG2,FRS3,CPB1,LIMS1,DOCK4,ACKR2,ARPC1A,BCL11B,CERS3,GLCE,PLS3,TSPAN2,PCSK2,KCNIP1,PWWP2A,TBC1D10A,SSBP2,REEP1,RPH3A,CTTNBP2,CYTH4,NAP1L1,SLC38A6,VPS29,PLPP4,CCDC102B,COPB2,UBR1,CNN2,COL4A2,ARHGAP32,DYRK3,IP6K2,CHRNA6,TLN2,HDAC4,FYB1,RBM45,SEM1,IQCE,LVRN,RPGRIP1L,MYLK,UNC5C,TLR7,PABIR3,P3H2,CFL1,WDR7,SLAIN2,HMG20A,CEP85L,PLEKHG4B,RBBP8,MED13,PHF2,RAI2,AFDN,LRP2,CYTIP,MTDH,LIG1,ZDHHC13,WWC3,CAMK4,CLPX,VWC2,REPS1,FBXL7,RPA3,TMEM108,MB21D2,SEZ6L,AFG2B,UPRT,ATXN1,ADAM17,SEMA4F,ELAVL1,TRAK1,STXBP1,IDH1,LMOD2,HDGFL2,MUC13,N

			UP205,PLEKHG1,NHLH1,ELP2,SLCO5A1,SNTB1,ORC4,APC,TTLL5,INO80,NAA11,MOCS2,ERGIC2,PEAR1,CDA,PPARGC1B,ESR2,NOL10,LNX1,ERC1,ZNF440,RUBCNL,AGO3,C9,PVR,CKAP4,RPL39L,ACER1,IQGAP2,CDYL,RLF,ANKRA2,TAF5L,JPT1,CTNND2,DNAH14,RASSF3,UGGT1,PACSIN2,DPYSL2,RAB8B,NSD2,PHACTR3,KCND1,TMEM267,JADE3,CARD18,PSMC2,MAPKAP1,EXOC6B,DNAH6,ACOX1,USP24,NUP153,HSD17B4,TRMT10B,RAI14,GPATCH2L,NRP2,SIPA1L2,CAVIN1,C1ORF21,METTL15,FOXJ2,CEP162,ELOVL7,NR3C1,BORCS5,FSTL4,ZC3HAV1,MTOR,KSR1,FAF1,SNAP25,DEFB124,SGSM1,GOLGA8A,ZNF221,CLDND1,PIP5K1A,GH2,BTBD2,MMACHC,RAB6B,KYNU,ADAL,ASCC2,SPRED1,GNG4,CNTNAP4,CDH26,PDS5B,CRIM1,MEI4,RBM46,RNF115,TRIM61,ESD,CDHR3,PER2,ANKRD24,GLI3,LHPP,KAT6A,BCOR,NTRK3,RXFP1,GAB2,DIS3L2,SNTB2,PDCD10,PDE1A,LMTK2,HHAT,CREB5,CCDC68,ADGRG6,GOLGA7,FERMT2,GCNA,GLUD1,TSPOAP1,ST3GAL2,DNM3,SREK1IP1,PHIP,EPB41,DENND5A,SS18,SFRP5,DOCK9,ZYG11B,PPARA,CD244,SHISA6,NFASC,HDX,ZNF229,PHF6,HMGB4,FGF7,TENT5D,MARCHF10,TBCA,STXBP6,MGA,RTTN,CACNA1A,EDA,LIMCH1,TNFSF13B,MTMR7,PTH2R,IAH1,COL14A1,RBM47,EIF2A,JARID2,FIRRM,HDAC2,TGOLN2,FGD1,TRIM39,TRRAP,SEMA3D,EIF4E3,USP48,KCNH5,RRM1,MAGEA1,CADM1,KLHL29,INPP5D,CFAP161,THUMPD1,ATF6,PRKAR1B,GBF1,IPO11,SERPINE1,CCDC88C,BACH1,CUL9,EWSR1,SLC26A5,AKAP13,PKM,GCG,CCDC150,TKFC,LINC02218,PTPN3,STAC,SCN8A,RTL4,SSX2IP,TTC17,ELP1,IRF8,HIVEP3,HEPACAM2,RFX4,CCDC198,REM1,AGAP1,FREM1,RIPOR2,CD69,FUNDC2,EPS15L1,CYB561A3,PARD3B,XIRP2,KDM3A,MZT2A,CAST,ADGRA3,GPR39,CASK,HSPG2,SLCO4C1,GCNT1,BPIFB3,GGA2,USH1C,NLRP14,DNAJC13,PSME3IP1,GOLGA8B
GO:0005085	guanyl-nucleotide exchange factor activity	0.000039408326215270196	*ITSN1,PREX2,DOCK7,PSD3,DENND11,RAPGEF1,ARHGEF3,DENND4A,ARHGEF11,SERGEF,MYCBP2,ANKRD27,ARHGEF10,VAV3,CCDC88A,PLCE1,RIN2,PLEKHG7,DENND1A,RALGPS1,SH2D3C,DOCK4,TBC1D10A,CYTH4,PLEKHG4B,PLEKHG1,DENND5A,DOCK9,FGD1,GBF1,CCDC88C,AKAP13*
GO:0060589	nucleoside-triphosphatase regulator activity	0.0000596597428406343	*MYO9B,RASA4B,ARAP1,ITSN1,PREX2,ARHGAP26,TBC1D22A,RGS12,ACAP2,DOCK7,PSD3,TBC1D19,DENND11,RAPGEF1,ARHGEF3,DENND4A,TBC1D20,ARHGEF11,SERGEF,CHM,IQGAP1,MYCBP2,ARHGAP6,ANKRD27,ARHGEF10,VAV3,CCDC88A,PLCE1,RIN2,PLEKHG7,DENND1A,TBC1D9B,RALGPS1,CHN2,SH2D3C,DOCK4,TBC1D10A,CYTH4,ARHGAP32,PLEKHG4B,PLEKHG1,IQGAP2,SIPA1L2,SGSM1,DENND5A,DOCK9,CPEB2,FGD1,GBF1,CCDC88C,AKAP13,AGAP1*
GO:0030695	GTPase regulator activity	0.0000596597428406343	*MYO9B,RASA4B,ARAP1,ITSN1,PREX2,ARHGAP26,TBC1D22A,RGS12,ACAP2,DOCK7,PSD3,TBC1D19,DENND11,RAPGEF1,ARHGEF3,DENND4A,TBC1D20,ARHGEF11,SERGEF,CHM,IQGAP1,MYCBP2,ARHGAP6,ANKRD27,ARHGEF10,VAV3,CCDC88A,PLCE1,RIN2,PLEKHG7,DENND1A,TBC1D9B,RALGPS1,CHN2,SH2D3C,DOCK4,TBC1D10A,CYTH4,ARHGAP32,PLEKHG4B,PLEKHG1,IQGAP2,SIP*

			A1L2,SGSM1,DENND5A,DOCK9,CPEB2,FGD1,GBF1,CCDC88C,AKAP13,AGAP1
GO:0045296	cadherin binding	0.0031989345922409066	*CIP2A,AHNAK,EGFR,NEO1,DHX29,SHTN1,CALD1,DBNL,MACF1,PARVA,PRDX1,SNX2,RTN4,IQGAP1,ANK3,CRKL,CDH10,ITGB1,TRPC4,TBC1D10A,CNN2,AFDN,MB21D2,IDH1,ERC1,CTNND2,PACSIN2,ZC3HAV1,CDH26,CDHR3,DOCK9,STXBP6,EIF2A,PKM,EPS15L1,CAST*
GO:0030165	PDZ domain binding	0.006335743792425932	*SDC2,ATP2B2,SNTA1,NKD1,DOCK4,TBC1D10A,ADAM17,SNTB1,LNX1,ERC1,ACOX1,CRIM1,SHISA6,CADM1,CCDC88C*
GO:0005547	phosphatidylinositol-3,4,5-trisphosphate binding	0.007753586156264446	*ARAP1,PLEKHB2,FCHSD2,IQGAP1,IQGAP2,MAPKAP1,GAB2,FERMT2,GBF1,FUNDC2*
GO:0019904	protein domain specific binding	0.012385986071037266	*CHMP1B,POLR2J,SDC2,NFE2L2,VRK2,ATP2B2,ENKUR,SNTA1,SH3BP2,SH3PXD2B,OCLN,CBX3,DBNL,TFDP2,PTPN12,NEDD4,SRP68,LEF1,RAPGEF1,SKAP1,IQGAP1,ARHGAP6,DPYSL3,CCDC88A,BAK1,LAX1,NKD1,DENND1A,PROX1,FMN1,CDC6,NEFL,DOCK4,TBC1D10A,CTTNBP2,LRP2,REPS1,ADAM17,TRAK1,STXBP1,SNTB1,PPARGC1B,LNX1,ERC1,RAB8B,CARD18,ACOX1,FAF1,SNAP25,CRIM1,PPARA,SHISA6,RRM1,CADM1,INPP5D,CCDC88C*
GO:0005516	calmodulin binding	0.03104342992138271	*MYO9B,ATP2B2,ENKUR,SNTA1,ADCY1,EGFR,CALD1,MYO3A,IQGAP1,MAP2,PPP3CA,MKNK1,CNN2,MYLK,CAMK4,SNTB1,IQGAP2,SNTB2,PDE1A,EPB41,KCNH5,EWSR1,REM1,CASK*
GO:0008092	cytoskeletal protein binding	0.036551222615142875	*MYO9B,CETN3,GAS2L1,DLEC1,ESPNL,SNTA1,PTPN4,KIF5C,SETD3,EGFR,PSMD10,SPATA6L,SHTN1,CALD1,DBNL,MACF1,TUBGCP3,EML1,PARVA,MID1,MYBPC3,KLHL4,MTUS1,NEB,CACNA1D,CLSTN1,MYO3A,IQGAP1,MAP2,FBXW11,MTCL1,HSPH1,ANK3,PLEKHM2,ARHGEF10,DPYSL3,CCDC88A,DISC1,FMN1,ITGB1,CORO1C,ARPC1A,PLS3,REEP1,CNN2,TLN2,MYLK,UNC5C,CFL1,AFDN,TRAK1,LMOD2,SNTB1,APC,TTLL5,INO80,IQGAP2,PACSIN2,PHACTR3,RAI14,SNAP25,RAB6B,ANKRD24,SNTB2,LMTK2,FERMT2,DNM3,EPB41,PHF6,TBCA,LIMCH1,CCDC88C,SLC26A5,PTPN3,XIRP2,USH1C*
GO:0035091	phosphatidylinositol binding	0.04282659998856954	*SNX16,SNX25,ARAP1,SH3PXD2B,SNX32,PLEKHB2,PITPNC1,SNX2,FCHSD2,PLEKHF2,IQGAP1,WIPI1,SNX30,SNAP91,RPS6KC1,CCDC88A,DENND1A,RPH3A,ARHGAP32,RUBCNL,IQGAP2,MAPKAP1,GAB2,FERMT2,EPB41,GBF1,FUNDC2,PARD3B,GGA2*
GO:0050839	cell adhesion molecule binding	0.04605862952036891	*CIP2A,AHNAK,ITGA2,EGFR,NEO1,DHX29,SHTN1,CALD1,DBNL,MACF1,PTPRZ1,PARVA,PRDX1,DSC2,SNX2,RTN4,IQGAP1,ITGA9,ANK3,CRKL,CD40LG,TENM4,CDH10,ITGB1,TRPC4,TBC1D10A,CNN2,TLN2,AFDN,MB21D2,ADAM17,IDH1,ERC1,PVR,CTNND2,PACSIN2,ZC3HAV1,CDH26,CDHR3,FERMT2,DOCK9,NFASC,STXBP6,EIF2A,CADM1,PKM,EPS15L1,CAST*
			BP
GO:0000902	cell morphogenesis	6.312676787632114e-7	*MYO9B,XK,SEMA4D,MET,SDC2,ARAP1,PREX2,TANC2,IL1RAPL1,KIF5C,GPM6A,ADCY1,ZDHHC17,LPAR3,EGFR,PTPRQ,NEO1,FAT1,SHTN1,SH3GL2,DOCK7,CRB1,NYAP2,MEF2C,DBNL,RYK,MA*

			CF1,PTPRZ1,NEDD4,PARVA,OLFM1,EFNB3,ILK,LEF1,MAPK14,TBC1D20,SNX2,VDR,RTN4,ANOS1,IQGAP1,MYCBP2,LATS1,MAP2,ANKRD27,ANK3,RELN,PPP3CA,CDH10,FRYL,DISC1,PROX1,VSIG1,ITGB1,CORO1C,NEFL,ENPP2,BCL11B,ARHGAP32,UNC5C,CFL1,PLEKHG4B,LRP2,TMEM108,ADAM17,SEMA4F,TRAK1,STXBP1,COL27A1,CTNND2,PACSIN2,NRP2,FSTL4,SNAP25,CDH26,CDHR3,ANKRD24,GLI3,FERMT2,DNM3,PHIP,SS18,NFASC,FGD1,SEMA3D,CCDC88C,SLC26A5,ST8SIA2,RIPOR2
GO:0048731	system development	0.00000110071811850126 94	*PBX4,XK,SEMA4D,MET,SCYL3,MMP16,ELAPOR2,DNAH11,SDC2,FANCC,ANKLE2,EIF4A3,NFE2L2,CYP19A1,SLC46A2,DTNB,GPR158,ATP2B2,PREX2,TANC2,CSMD3,NTN4,SERP1,SRGAP2C,WIF1,ARHGAP26,ZFHX2,ITGA2,IL1RAPL1,KIF5C,GPM6A,WNT16,ADCY1,RPS6KA2,PLPPR5,ZDHHC17,FIG4,FOXP2,LPAR3,SH3TC2,CEP290,TACC1,SH3PXD2B,EGFR,PTPRQ,NEO1,MYT1L,SRGAP2B,FBXL17,LRP12,ESRP1,RNF10,FAT1,NOVA1,SHTN1,BTD,CALD1,SH3GL2,DOCK7,ROR1,PAX5,CRB1,ARID1A,SMAD6,NYAP2,MEF2C,FZR1,DBNL,RYK,MACF1,TFDP2,PTPRZ1,PLN,EML1,NEDD4,PARVA,SECISBP2,ZNF609,PRDX1,OLFM1,SLC12A2,EFNB3,OTX1,ILK,LEF1,EYA1,MAPK14,MYBPC3,RAPGEF1,SLC1A2,GABRB2,GRIN2B,TBC1D20,PARP2,ZSWIM6,SLC4A7,NEB,TMEM100,VDR,CREB3L2,RTN4,BBS2,CLSTN1,SCYL2,SERPINE2,PLAAT3,PIK3CG,ANOS1,COL4A4,TPP1,STIL,NUP160,IQGAP1,MYCBP2,WNT2B,MAP2,FBXW11,CHD5,JAK1,KCNB1,GNPAT,ANKRD27,AIM2,ANK3,KITLG,ARID4B,RELN,CRKL,ARHGEF10,VAV3,CD40LG,DPYSL3,ATRN,CCDC88A,ARNT2,SPOCK1,PLCE1,TACC2,FRAS1,MSH3,BAK1,TENM4,PPP3CA,NKD1,RBPJ,RIN2,PDE6C,DLX6-AS1,ADAMTS16,TAF10,MBD5,FRYL,ASH1L,RSPO2,DISC1,PROX1,FMN1,ITGB1,CORO1C,NEFL,NAV2,STK3,C1GALT1,EXOSC3,TRPC4,BCL11B,PLS3,TSPAN2,PCSK2,NAP1L1,COL4A2,ARHGAP32,HDAC4,RBM45,RPGRIP1L,MYLK,UNC5C,CFL1,HMG20A,CEP85L,PLEKHG4B,PHF2,LRP2,MTDH,VWC2,TMEM108,SEZ6L,ATXN1,TMEM59L,ADAM17,SEMA4F,TRAK1,STXBP1,IDH1,NHLH1,APC,TTLL5,PPARGC1B,COL27A1,CTNND2,DPYSL2,NSD2,AARS1,MAPKAP1,HSD17B4,EFHD1,NRP2,FOXJ2,NR3C1,DLX6,FSTL4,MTOR,SNAP25,BTBD2,RAB6B,SPRED1,GALC,CRIM1,ABCB5,PER2,ANKRD24,GLI3,BCOR,NTRK3,RXFP1,PDCD10,ADGRG6,GLUD1,DNM3,DENND5A,SFRP5,PPARA,NFASC,FGF7,EDA,COL14A1,JARID2,HDAC2,SEMA3D,RRM1,CADM1,ATF6,SERPINE1,AKAP13,PKM,PTPN3,SCN8A,ST8SIA2,RFX4,FREM1,RIPOR2,XIRP2,HSPG2,GCNT1,USH1C*
GO:0032502	developmental process	0.00000146252913679605 57	*PBX4,MYO9B,XK,SEMA4D,MET,SCYL3,TEAD1,EIF4G3,IFTAP,SIAH3,MMP16,ELAPOR2,DNAH11,SDC2,FANCC,ARAP1,ANKLE2,ACTR5,EIF4A3,DLEC1,NFE2L2,CYP19A1,SLC46A2,DTNB,GPR158,ATP2B2,PREX2,TANC2,CSMD3,NTN4,SERP1,PLEKHM3,ENKUR,ASZ1,SRGAP2C,WIF1,FLI1,ESRRB,ARHGAP26,ZFHX2,ZBTB7C,ITGA2,IL1RAPL1,KIF5C,BLK,GPM6A,SPATA16,WNT16,KRT86,SETD3,ADCY1,RPS6KA2,PLPPR5,ZDHHC17,FIG4,CFAP47,FOXP2,KMT2D,LPAR3,TXLNG,SH*

			3TC2,CEP290,TACC1,SH3PXD2B,CFAP91,EGFR,PTPRQ,NEO1,MYT1L,SRGAP2B,PLEKHB2,FBXL17,SLC9A4,GPAM,SPATA6L,LRP12,ESRP1,BRMS1L,SLC39A10,RNF10,FAT1,PIK3R1,NOVA1,SHTN1,TMEM232,BTD,CALD1,SH3GL2,DOCK7,XKR4,ROR1,PAX5,CRB1,ARID1A,THEMIS,SMAD6,NYAP2,MEF2C,FZR1,DBNL,RYK,MACF1,WEE2,TFDP2,PTPN12,PTPRZ1,PLN,EML1,NEDD4,PARVA,SECISBP2,SLC9C1,SCML2,ZNF609,PRDX1,ATG4B,OLFM1,MID1,SLC12A2,EFNB3,OTX1,DLG3,FLOT2,ILK,LEF1,EYA1,MAPK14,MYBPC3,RAPGEF1,ZNF516,SLC1A2,GABRB2,GRIN2B,CRISPLD1,TBC1D20,SNX2,PARP2,ZSWIM6,SLC4A7,DNAJB6,PIEZO1,NEB,TMEM100,VDR,CREB3L2,RTN4,BBS2,CLSTN1,SCYL2,MYO3A,SERPINE2,PLAAT3,PIK3CG,ANOS1,COL4A4,TPP1,STIL,NUP160,IQGAP1,MYCBP2,LATS1,KLHL12,WNT2B,MAP2,FBXW11,AZIN2,NHSL1,PEMT,CHD5,JAK1,RADIL,KCNB1,GNPAT,ANKRD27,AIM2,ANK3,KITLG,ARID4B,NR1I2,RELN,CRKL,TENT5A,ARHGEF10,NDC1,VAV3,CD40LG,DPYSL3,SCN9A,ATRN,CCDC88A,ARNT2,SPOCK1,PLCE1,TACC2,FRAS1,MSH3,BAK1,TENM4,PPP3CA,NKD1,RBPJ,RIN2,PDE6C,DLX6-AS1,CDH10,ADAMTS16,TAF10,MBD5,FRYL,ASH1L,RSPO2,DISC1,PROX1,FMN1,VSIG1,ITGB1,DRC7,CORO1C,NEFL,NAV2,STK3,C1GALT1,HNF4G,MSR1,EXOSC3,TRPC4,ENPP2,BCL11B,CERS3,PLS3,TSPAN2,PCSK2,NAP1L1,PLPP4,CNN2,COL4A2,ARHGAP32,DYRK3,HDAC4,RBM45,IQCE,RPGRIP1L,MYLK,UNC5C,CFAP69,FLG2,CFL1,DTYMK,WDR7,HMG20A,CEP85L,PLEKHG4B,RBBP8,RNASEH2B,PHF2,RAI2,AFDN,LRP2,MTDH,LIG1,WWC3,CAMK4,VWC2,TMEM108,SEZ6L,UPRT,ATXN1,TMEM59L,ADAM17,SEMA4F,ELAVL1,TRAK1,STXBP1,IDH1,LMOD2,HDGFL2,NHLH1,APC,TTLL5,INO80,PPARGC1B,COL27A1,RPL39L,ACER1,CDYL,TAF5L,CTNND2,PACSIN2,DPYSL2,METTL8,NSD2,AARS1,PSMC2,MAPKAP1,ACOX1,HSD17B4,EFHD1,RAI14,NRP2,FOXJ2,NR3C1,DLX6,FSTL4,MTOR,SNAP25,PIP5K1A,GH2,BTBD2,RAB6B,SPRED1,GALC,CDH26,CRIM1,MEI4,RBM46,ABCB5,CDHR3,PER2,ANKRD24,GLI3,KAT6A,BCOR,NTRK3,RXFP1,GAB2,DIS3L2,PDCD10,PRAMEF11,ADGRG6,FERMT2,GLUD1,DNM3,PHIP,DENND5A,SS18,SFRP5,PPARA,SHISA6,NFASC,PHF6,FGF7,MGA,RTTN,EDA,TNFSF13B,COL14A1,RBM47,JARID2,HDAC2,TPO,FGD1,SEMA3D,RRM1,CADM1,INPP5D,ATF6,SERPINE1,CCDC88C,PRAMEF4,SLC26A5,AKAP13,PKM,PTPN3,SCN8A,ST8SIA2,IRF8,HIVEP3,RFX4,FREM1,RIPOR2,XIRP2,KDM3A,HSPG2,SLCO4C1,GCNT1,USH1C,NLRP14,DNAJC13
GO:0007399	nervous system development	0.00000277747510857087 95	*PBX4,XK,SEMA4D,MET,SCYL3,ELAPOR2,DNAH11,SDC2,FANCC,ANKLE2,NFE2L2,DTNB,GPR158,ATP2B2,PREX2,TANC2,CSMD3,NTN4,SRGAP2C,ARHGAP26,ZFHX2,IL1RAPL1,KIF5C,GPM6A,WNT16,ADCY1,PLPPR5,ZDHHC17,FIG4,FOXP2,LPAR3,SH3TC2,CEP290,TACC1,EGFR,PTPRQ,NEO1,MYT1L,SRGAP2B,FBXL17,LRP12,ESRP1,RNF10,NOVA1,SHTN1,BTD,SH3GL2,DOCK7,ROR1,PAX5,CRB1,ARID1A,NYAP2,MEF2C,DBNL,RYK,MACF1,PTPRZ1,EML1,NEDD4,SECISBP2,ZNF609,OLFM1,EFNB3,OTX1,ILK,LEF1,EYA1,RAPGE*

			F1,SLC1A2,GABRB2,GRIN2B,ZSWIM6,SLC4A7,RTN4,BBS2,CLSTN1,SCYL2,SERPINE2,ANOS1,TPP1,STIL,IQGAP1,MYCBP2,WNT2B,MAP2,FBXW11,CHD5,KCNB1,GNPAT,ANKRD27,AIM2,ANK3,RELN,CRKL,ARHGEF10,DPYSL3,ATRN,CCDC88A,ARNT2,SPOCK1,TACC2,TENM4,PPP3CA,NKD1,RBPJ,PDE6C,DLX6-AS1,MBD5,FRYL,RSPO2,DISC1,PROX1,ITGB1,CORO1C,NEFL,NAV2,STK3,TRPC4,BCL11B,TSPAN2,PCSK2,NAP1L1,ARHGAP32,HDAC4,RBM45,RPGRIP1L,UNC5C,CFL1,HMG20A,CEP85L,PLEKHG4B,LRP2,VWC2,TMEM108,SEZ6L,ATXN1,ADAM17,SEMA4F,TRAK1,STXBP1,NHLH1,APC,CTNND2,DPYSL2,AARS1,MAPKAP1,EFHD1,NRP2,NR3C1,DLX6,FSTL4,MTOR,SNAP25,BTBD2,RAB6B,GALC,CRIM1,PER2,ANKRD24,GLI3,NTRK3,ADGRG6,GLUD1,DNM3,DENND5A,NFASC,JARID2,HDAC2,SEMA3D,RRM1,SCN8A,ST8SIA2,RFX4,RIPOR2,HSPG2,USH1C
GO:0030030	cell projection organization	0.000005514885633727258	*MYO9B,XK,SEMA4D,SDC2,ARAP1,NFE2L2,PREX2,TANC2,CSMD3,FBF1,SRGAP2C,ITGA2,IL1RAPL1,KIF5C,GPM6A,ADCY1,PLPPR5,ZDHHC17,CFAP47,LPAR3,CEP290,OCLN,CFAP91,EGFR,NEO1,LRP12,MPHOSPH9,PIK3R1,SHTN1,TMEM232,SH3GL2,DOCK7,ROR1,NYAP2,MEF2C,DBNL,RYK,MACF1,TBC1D31,PTPRZ1,NEDD4,PARVA,OLFM1,EFNB3,ILK,RAPGEF1,GRIN2B,UBXN10,SNX2,RTN4,BBS2,MYO3A,ANOS1,IQGAP1,MYCBP2,MAP2,ANKRD27,ANK3,RELN,CRKL,VAV3,DPYSL3,CCDC88A,SPOCK1,PLCE1,STK26,PPP3CA,ADAMTS16,FRYL,STON1,DISC1,ITGB1,DRC7,CORO1C,NEFL,ENPP2,BCL11B,GLCE,TSPAN2,ARHGAP32,RPGRIP1L,MYLK,UNC5C,CFAP69,CFL1,PLEKHG4B,LRP2,TMEM108,CC2D2B,ADAM17,SEMA4F,TRAK1,STXBP1,APC,CTNND2,PACSIN2,DPYSL2,RAB8B,EFHD1,NRP2,CEP162,FSTL4,MTOR,SNAP25,PIP5K1A,RAB6B,ANKRD24,GLI3,NTRK3,DNM3,DENND5A,NFASC,RTTN,HDAC2,FGD1,SEMA3D,CFAP161,GBF1,SSX2IP,ST8SIA2,TTC17,RFX4,RIPOR2,USH1C*
GO:0048856	anatomical structure development	0.0000135735798411875443	*PBX4,MYO9B,XK,SEMA4D,MET,SCYL3,TEAD1,EIF4G3,SIAH3,MMP16,ELAPOR2,DNAH11,SDC2,FANCC,ARAP1,ANKLE2,ACTR5,EIF4A3,NFE2L2,CYP19A1,SLC46A2,DTNB,GPR158,ATP2B2,PREX2,TANC2,CSMD3,NTN4,SERP1,PLEKHM3,ENKUR,SRGAP2C,WIF1,FLI1,ESRRB,ARHGAP26,ZFHX2,ITGA2,IL1RAPL1,KIF5C,GPM6A,SPATA16,WNT16,KRT86,SETD3,ADCY1,RPS6KA2,PLPPR5,ZDHHC17,FIG4,CFAP47,FOXP2,KMT2D,LPAR3,TXLNG,SH3TC2,CEP290,TACC1,SH3PXD2B,EGFR,PTPRQ,NEO1,MYT1L,SRGAP2B,FBXL17,SLC9A4,LRP12,ESRP1,SLC39A10,RNF10,FAT1,PIK3R1,NOVA1,SHTN1,BTD,CALD1,SH3GL2,DOCK7,XKR4,ROR1,PAX5,CRB1,ARID1A,THEMIS,SMAD6,NYAP2,MEF2C,FZR1,DBNL,RYK,MACF1,WEE2,TFDP2,PTPN12,PTPRZ1,PLN,EML1,NEDD4,PARVA,SECISBP2,SCML2,ZNF609,PRDX1,ATG4B,OLFM1,MID1,SLC12A2,EFNB3,OTX1,DLG3,FLOT2,ILK,LEF1,EYA1,MAPK14,MYBPC3,RAPGEF1,ZNF516,SLC1A2,GABRB2,GRIN2B,CRISPLD1,TBC1D20,SNX2,PARP2,ZSWIM6,SLC4A7,DNAJB6,PIEZO1,NEB,TMEM100,VDR,CREB3L2,RTN4,BBS2,CLSTN1,SCYL2,MYO3A,SERPINE2,PLAAT3,PIK3CG,ANOS1,COL4A4,TPP1,STIL,NUP*

			160,IQGAP1,MYCBP2,LATS1,KLHL12,WNT2B,MAP2,FBXW11,PEMT,CHD5,JAK1,RADIL,KCNB1,GNPAT,ANKRD27,AIM2,ANK3,KITLG,ARID4B,RELN,CRKL,TENT5A,ARHGEF10,VAV3,CD40LG,DPYSL3,SCN9A,ATRN,CCDC88A,ARNT2,SPOCK1,PLCE1,TACC2,FRAS1,MSH3,BAK1,TENM4,PPP3CA,NKD1,RBPJ,RIN2,PDE6C,DLX6-AS1,CDH10,ADAMTS16,TAF10,MBD5,FRYL,ASH1L,RSPO2,DISC1,PROX1,FMN1,VSIG1,ITGB1,DRC7,CORO1C,NEFL,NAV2,STK3,C1GALT1,HNF4G,EXOSC3,TRPC4,ENPP2,BCL11B,CERS3,PLS3,TSPAN2,PCSK2,NAP1L1,PLPP4,CNN2,COL4A2,ARHGAP32,DYRK3,HDAC4,RBM45,IQCE,RPGRIP1L,MYLK,UNC5C,CFAP69,FLG2,CFL1,DTYMK,WDR7,HMG20A,CEP85L,PLEKHG4B,RBBP8,RNASEH2B,PHF2,RAI2,AFDN,LRP2,MTDH,LIG1,CAMK4,VWC2,TMEM108,SEZ6L,UPRT,ATXN1,TMEM59L,ADAM17,SEMA4F,TRAK1,STXBP1,IDH1,LMOD2,HDGFL2,NHLH1,APC,TTLL5,INO80,PPARGC1B,COL27A1,ACER1,CDYL,CTNND2,PACSIN2,DPYSL2,METTL8,NSD2,AARS1,MAPKAP1,HSD17B4,EFHD1,NRP2,FOXJ2,NR3C1,DLX6,FSTL4,MTOR,SNAP25,PIP5K1A,GH2,BTBD2,RAB6B,SPRED1,GALC,CDH26,CRIM1,MEI4,RBM46,ABCB5,CDHR3,PER2,ANKRD24,GLI3,KAT6A,BCOR,NTRK3,RXFP1,GAB2,PDCD10,ADGRG6,FERMT2,GLUD1,DNM3,PHIP,DENND5A,SS18,SFRP5,PPARA,NFASC,PHF6,FGF7,RTTN,EDA,TNFSF13B,COL14A1,RBM47,JARID2,HDAC2,TPO,FGD1,SEMA3D,RRM1,CADM1,INPP5D,ATF6,SERPINE1,CCDC88C,SLC26A5,AKAP13,PKM,PTPN3,SCN8A,ST8SIA2,IRF8,HIVEP3,RFX4,FREM1,RIPOR2,XIRP2,KDM3A,HSPG2,GCNT1,USH1C
GO:0007275	multicellular organism development	0.000018523192560297455	*PBX4,XK,SEMA4D,MET,SCYL3,TEAD1,SIAH3,MMP16,ELAPOR2,DNAH11,SDC2,FANCC,ANKLE2,ACTR5,EIF4A3,NFE2L2,CYP19A1,SLC46A2,DTNB,GPR158,ATP2B2,PREX2,TANC2,CSMD3,NTN4,SERP1,ENKUR,SRGAP2C,WIF1,ARHGAP26,ZFHX2,ITGA2,IL1RAPL1,KIF5C,GPM6A,WNT16,ADCY1,RPS6KA2,PLPPR5,ZDHHC17,FIG4,FOXP2,LPAR3,TXLNG,SH3TC2,CEP290,TACC1,SH3PXD2B,EGFR,PTPRQ,NEO1,MYT1L,SRGAP2B,FBXL17,LRP12,ESRP1,RNF10,FAT1,PIK3R1,NOVA1,SHTN1,BTD,CALD1,SH3GL2,DOCK7,ROR1,PAX5,CRB1,ARID1A,SMAD6,NYAP2,MEF2C,FZR1,DBNL,RYK,MACF1,TFDP2,PTPRZ1,PLN,EML1,NEDD4,PARVA,SECISBP2,ZNF609,PRDX1,OLFM1,MID1,SLC12A2,EFNB3,OTX1,ILK,LEF1,EYA1,MAPK14,MYBPC3,RAPGEF1,SLC1A2,GABRB2,GRIN2B,TBC1D20,PARP2,ZSWIM6,SLC4A7,DNAJB6,NEB,TMEM100,VDR,CREB3L2,RTN4,BBS2,CLSTN1,SCYL2,MYO3A,SERPINE2,PLAAT3,PIK3CG,ANOS1,COL4A4,TPP1,STIL,NUP160,IQGAP1,MYCBP2,LATS1,KLHL12,WNT2B,MAP2,FBXW11,PEMT,CHD5,JAK1,KCNB1,GNPAT,ANKRD27,AIM2,ANK3,KITLG,ARID4B,RELN,CRKL,TENT5A,ARHGEF10,VAV3,CD40LG,DPYSL3,SCN9A,ATRN,CCDC88A,ARNT2,SPOCK1,PLCE1,TACC2,FRAS1,MSH3,BAK1,TENM4,PPP3CA,NKD1,RBPJ,RIN2,PDE6C,DLX6-AS1,ADAMTS16,TAF10,MBD5,FRYL,ASH1L,RSPO2,DISC1,PROX1,FMN1,ITGB1,CORO1C,NEFL,NAV2,STK3,C1GALT1,EXOSC3,TRPC4,BCL11B,PLS3,TSPAN2,PCSK2,NAP1L1,PLPP4,COL4A2,*

			ARHGAP32,HDAC4,RBM45,IQCE,RPGRIP1L,MYLK,UNC5C,CFL1,HMG20A,CEP85L,PLEKHG4B,RBBP8,RNASEH2B,PHF2,RAI2,LRP2,MTDH,CAMK4,VWC2,TMEM108,SEZ6L,ATXN1,TMEM59L,ADAM17,SEMA4F,TRAK1,STXBP1,IDH1,NHLH1,APC,TTLL5,INO80,PPARGC1B,COL27A1,CTNND2,DPYSL2,NSD2,AARS1,MAPKAP1,HSD17B4,EFHD1,NRP2,FOXJ2,NR3C1,DLX6,FSTL4,MTOR,SNAP25,BTBD2,RAB6B,SPRED1,GALC,CRIM1,RBM46,ABCB5,PER2,ANKRD24,GLI3,KAT6A,BCOR,NTRK3,RXFP1,PDCD10,ADGRG6,FERMT2,GLUD1,DNM3,DENND5A,SFRP5,PPARA,NFASC,PHF6,FGF7,RTTN,EDA,COL14A1,JARID2,HDAC2,TPO,SEMA3D,RRM1,CADM1,INPP5D,ATF6,SERPINE1,AKAP13,PKM,PTPN3,SCN8A,ST8SIA2,RFX4,FREM1,RIPOR2,XIRP2,HSPG2,GCNT1,USH1C
GO:0030182	neuron differentiation	0.000027583005296182516	*PBX4,XK,SEMA4D,MET,SCYL3,SDC2,NFE2L2,DTNB,ATP2B2,PREX2,TANC2,CSMD3,NTN4,SRGAP2C,ZFHX2,IL1RAPL1,KIF5C,GPM6A,WNT16,ADCY1,PLPPR5,ZDHHC17,FIG4,LPAR3,CEP290,EGFR,PTPRQ,NEO1,MYT1L,LRP12,ESRP1,SHTN1,SH3GL2,DOCK7,ROR1,CRB1,NYAP2,MEF2C,DBNL,RYK,MACF1,PTPRZ1,NEDD4,SECISBP2,OLFM1,EFNB3,EYA1,RAPGEF1,GABRB2,ZSWIM6,SLC4A7,RTN4,SCYL2,ANOS1,IQGAP1,MYCBP2,WNT2B,MAP2,CHD5,KCNB1,ANKRD27,ANK3,RELN,CRKL,DPYSL3,CCDC88A,SPOCK1,TENM4,PPP3CA,NKD1,RBPJ,PDE6C,FRYL,RSPO2,DISC1,PROX1,ITGB1,NEFL,BCL11B,TSPAN2,ARHGAP32,RPGRIP1L,UNC5C,CFL1,HMG20A,PLEKHG4B,LRP2,VWC2,TMEM108,ADAM17,SEMA4F,TRAK1,STXBP1,CTNND2,DPYSL2,EFHD1,NRP2,FSTL4,SNAP25,RAB6B,ANKRD24,GLI3,NTRK3,DNM3,DENND5A,NFASC,HDAC2,SEMA3D,ST8SIA2,RIPOR2,USH1C*
GO:0048666	neuron development	0.000035909788485631135	*PBX4,XK,SEMA4D,SCYL3,SDC2,NFE2L2,PREX2,TANC2,CSMD3,NTN4,SRGAP2C,IL1RAPL1,KIF5C,GPM6A,ADCY1,PLPPR5,ZDHHC17,FIG4,LPAR3,CEP290,EGFR,PTPRQ,NEO1,MYT1L,LRP12,SHTN1,SH3GL2,DOCK7,ROR1,CRB1,NYAP2,MEF2C,DBNL,RYK,MACF1,PTPRZ1,NEDD4,SECISBP2,OLFM1,EFNB3,RAPGEF1,GABRB2,SLC4A7,RTN4,SCYL2,ANOS1,IQGAP1,MYCBP2,MAP2,KCNB1,ANKRD27,ANK3,RELN,CRKL,DPYSL3,CCDC88A,SPOCK1,TENM4,PPP3CA,PDE6C,FRYL,DISC1,ITGB1,NEFL,BCL11B,TSPAN2,ARHGAP32,RPGRIP1L,UNC5C,CFL1,PLEKHG4B,LRP2,TMEM108,ADAM17,SEMA4F,TRAK1,STXBP1,CTNND2,DPYSL2,EFHD1,NRP2,FSTL4,SNAP25,RAB6B,ANKRD24,GLI3,NTRK3,DNM3,DENND5A,NFASC,HDAC2,SEMA3D,ST8SIA2,RIPOR2,USH1C*
GO:0048699	generation of neurons	0.00004627863136320627	*PBX4,XK,SEMA4D,MET,SCYL3,SDC2,NFE2L2,DTNB,ATP2B2,PREX2,TANC2,CSMD3,NTN4,SRGAP2C,ZFHX2,IL1RAPL1,KIF5C,GPM6A,WNT16,ADCY1,PLPPR5,ZDHHC17,FIG4,LPAR3,CEP290,EGFR,PTPRQ,NEO1,MYT1L,LRP12,ESRP1,SHTN1,SH3GL2,DOCK7,ROR1,CRB1,NYAP2,MEF2C,DBNL,RYK,MACF1,PTPRZ1,EML1,NEDD4,SECISBP2,ZNF609,OLFM1,EFNB3,LEF1,EYA1,RAPGEF1,GABRB2,ZSWIM6,SLC4A7,RTN4,SCYL2,ANOS1,IQGAP1,MYCBP2,WNT2B,MAP2,CHD5,KCNB1,ANKRD27,ANK3,RELN,CRKL,DPYSL3,CCDC88A,SPOCK1,TENM4,PPP3CA,NKD1,RBPJ,PDE6C,FRYL,RSPO2,DISC1,PROX1,ITGB1,NEFL,BCL11B*

			,TSPAN2,ARHGAP32,RPGRIP1L,UNC5C,CFL1,HMG20A,CEP85L,PLEKHG4B,LRP2,VWC2,TMEM108,ADAM17,SEMA4F,TRAK1,STXBP1,CTNND2,DPYSL2,EFHD1,NRP2,FSTL4,SNAP25,RAB6B,ANKRD24,GLI3,NTRK3,DNM3,DENND5A,NFASC,HDAC2,SEMA3D,ST8SIA2,RIPOR2,USH1C
GO:0022008	neurogenesis	0.00004799168314449987	*PBX4,XK,SEMA4D,MET,SCYL3,SDC2,NFE2L2,DTNB,ATP2B2,PREX2,TANC2,CSMD3,NTN4,SRGAP2C,ZFHX2,IL1RAPL1,KIF5C,GPM6A,WNT16,ADCY1,PLPPR5,ZDHHC17,FIG4,LPAR3,SH3TC2,CEP290,EGFR,PTPRQ,NEO1,MYT1L,LRP12,ESRP1,RNF10,SHTN1,SH3GL2,DOCK7,ROR1,CRB1,NYAP2,MEF2C,DBNL,RYK,MACF1,PTPRZ1,EML1,NEDD4,SECISBP2,ZNF609,OLFM1,EFNB3,ILK,LEF1,EYA1,RAPGEF1,GABRB2,ZSWIM6,SLC4A7,RTN4,SCYL2,SERPINE2,ANOS1,IQGAP1,MYCBP2,WNT2B,MAP2,CHD5,KCNB1,ANKRD27,ANK3,RELN,CRKL,ARHGEF10,DPYSL3,CCDC88A,SPOCK1,TENM4,PPP3CA,NKD1,RBPJ,PDE6C,FRYL,RSPO2,DISC1,PROX1,ITGB1,NEFL,NAV2,TRPC4,BCL11B,TSPAN2,NAP1L1,ARHGAP32,RPGRIP1L,UNC5C,CFL1,HMG20A,CEP85L,PLEKHG4B,LRP2,VWC2,TMEM108,ADAM17,SEMA4F,TRAK1,STXBP1,CTNND2,DPYSL2,EFHD1,NRP2,NR3C1,FSTL4,MTOR,SNAP25,BTBD2,RAB6B,PER2,ANKRD24,GLI3,NTRK3,ADGRG6,DNM3,DENND5A,NFASC,HDAC2,SEMA3D,ST8SIA2,RIPOR2,USH1C*
GO:0120036	plasma membrane bounded cell projection organization	0.0000511251891553275 67	*MYO9B,XK,SEMA4D,SDC2,ARAP1,NFE2L2,PREX2,TANC2,CSMD3,FBF1,SRGAP2C,IL1RAPL1,KIF5C,GPM6A,ADCY1,PLPPR5,ZDHHC17,CFAP47,LPAR3,CEP290,OCLN,CFAP91,EGFR,NEO1,LRP12,MPHOSPH9,PIK3R1,SHTN1,SH3GL2,DOCK7,ROR1,NYAP2,MEF2C,DBNL,RYK,MACF1,TBC1D31,PTPRZ1,NEDD4,PARVA,OLFM1,EFNB3,RAPGEF1,GRIN2B,UBXN10,SNX2,RTN4,BBS2,MYO3A,ANOS1,IQGAP1,MYCBP2,MAP2,ANKRD27,ANK3,RELN,CRKL,VAV3,DPYSL3,CCDC88A,SPOCK1,PLCE1,STK26,PPP3CA,ADAMTS16,FRYL,STON1,DISC1,ITGB1,DRC7,CORO1C,NEFL,ENPP2,BCL11B,TSPAN2,ARHGAP32,RPGRIP1L,MYLK,UNC5C,CFAP69,CFL1,PLEKHG4B,LRP2,TMEM108,CC2D2B,ADAM17,SEMA4F,TRAK1,STXBP1,APC,CTNND2,DPYSL2,EFHD1,NRP2,CEP162,FSTL4,MTOR,SNAP25,PIP5K1A,RAB6B,ANKRD24,GLI3,NTRK3,DNM3,DENND5A,NFASC,RTTN,HDAC2,FGD1,SEMA3D,CFAP161,GBF1,SSX2IP,ST8SIA2,TTC17,RFX4,RIPOR2,USH1C*
GO:0009653	anatomical structure morphogenesis	0.00007147931129561045	*PBX4,MYO9B,XK,SEMA4D,MET,MMP16,DNAH11,SDC2,FANCC,ARAP1,EIF4A3,NFE2L2,CYP19A1,PREX2,TANC2,NTN4,SERP1,FLI1,ITGA2,IL1RAPL1,KIF5C,GPM6A,WNT16,ADCY1,ZDHHC17,FIG4,LPAR3,CEP290,EGFR,PTPRQ,NEO1,FAT1,SHTN1,CALD1,SH3GL2,DOCK7,PAX5,CRB1,SMAD6,NYAP2,MEF2C,DBNL,RYK,MACF1,PTPRZ1,PLN,NEDD4,PARVA,SCML2,OLFM1,SLC12A2,EFNB3,OTX1,DLG3,ILK,LEF1,EYA1,MAPK14,MYBPC3,CRISPLD1,TBC1D20,SNX2,DNAJB6,NEB,TMEM100,VDR,RTN4,BBS2,MYO3A,SERPINE2,PIK3CG,ANOS1,STIL,IQGAP1,MYCBP2,LATS1,KLHL12,WNT2B,MAP2,FBXW11,JAK1,GNPAT,ANKRD27,ANK3,RELN,CRKL,VAV3,FRAS1,BAK1,TENM4,PPP3CA,NKD1,RBPJ,RIN2,PDE6C,CDH10,ADAMTS16,TAF10,FRYL,ASH1L,RSPO2,DISC1,PROX1,FMN1,VSIG1,ITGB1,CORO1C,NEFL,STK3*

			,C1GALT1,ENPP2,BCL11B,COL4A2,ARHGAP32,IQCE,RPGRIP1L,MYLK,UNC5C,FLG2,CFL1,PLEKHG4B,LRP2,MTDH,LIG1,TMEM108,TMEM59L,ADAM17,SEMA4F,TRAK1,STXBP1,LMOD2,APC,PPARGC1B,COL27A1,CTNND2,PACSIN2,NSD2,NRP2,FOXJ2,NR3C1,DLX6,FSTL4,MTOR,SNAP25,SPRED1,CDH26,RBM46,CDHR3,ANKRD24,GLI3,BCOR,RXFP1,PDCD10,ADGRG6,FERMT2,DNM3,PHIP,SS18,SFRP5,PPARA,NFASC,FGF7,EDA,TNFSF13B,HDAC2,FGD1,SEMA3D,SERPINE1,CCDC88C,SLC26A5,AKAP13,PKM,ST8SIA2,FREM1,RIPOR2,XIRP2,HSPG2,GCNT1,USH1C
GO:0051056	regulation of small GTPase mediated signal transduction	0.0003559034795048139	MYO9B,SEMA4D,MET,RASA4B,ARAP1,ITSN1,PREX2,ARHGAP26,PSD3,RAPGEF1,ARHGEF3,DENND4A,RTN4,ARHGEF11,PIK3CG,ARHGAP6,KITLG,RELN,CRKL,ARHGEF10,VAV3,DENND1A,RALGPS1,ITGB1,CHN2,CYTH4,ARHGAP32,PLEKHG4B,PLEKHG1,MAPKAP1,SIPA1L2,FGD1,GBF1,AKAP13,SSX2IP,RIPOR2
GO:0032501	multicellular organismal process	0.00036538085943359526	PBX4,XK,SEMA4D,MET,SCYL3,TEAD1,ACSM3,SIAH3,CETN3,MMP16,ELAPOR2,DNAH11,TACR1,SDC2,ABHD12,FANCC,ANKLE2,ACTR5,EIF4A3,NFE2L2,CYP19A1,SLC46A2,DTNB,GPR158,ATP2B2,PREX2,ESPNL,TANC2,CSMD3,NTN4,SERP1,ENKUR,SNTA1,SRGAP2C,WIF1,FLI1,CLIC5,ESRRB,ARHGAP26,ZFHX2,RAB44,ITGA2,IL1RAPL1,KIF5C,GPM6A,WNT16,KRT86,SETD3,ADCY1,GRID1,RPS6KA2,PLPPR5,ZDHHC17,FIG4,FOXP2,LPAR3,TXLNG,SH3TC2,CEP290,TACC1,SH3PXD2B,OCLN,NETO1,EGFR,PTPRQ,NEO1,MYT1L,SRGAP2B,F10,FBXL17,EOLA1,SLC9A4,GPAM,LRP12,ESRP1,BRMS1L,SLC39A10,RNF10,FAT1,PIK3R1,IL17B,NOVA1,SHTN1,ATG12,CD200R1,BTD,CALD1,SH3GL2,CRTC1,DOCK7,ROR1,PAX5,CRB1,ARID1A,THEMIS,SMAD6,NYAP2,MEF2C,FZR1,DBNL,RYK,MACF1,TFDP2,PTPRZ1,ACACA,PLN,EML1,SLC16A7,NEDD4,PARVA,SECISBP2,ZNF609,PRDX1,DSC2,SERPINA1,OLFM1,MID1,SLC12A2,EFNB3,OTX1,FLOT2,ILK,LEF1,EYA1,MAPK14,MYBPC3,RAPGEF1,ZNF516,SLC1A2,GABRB2,GRIN2B,CRISPLD1,IFNGR2,TBC1D20,OXR1,SCAMP5,PARP2,MYL1,ZSWIM6,SLC4A7,DNAJB6,OR2T11,NEB,TMEM100,VDR,CREB3L2,RTN4,CACNA1D,PRKAA2,ARHGEF11,BBS2,CLSTN1,TMEM150C,SCYL2,CHM,MYO3A,SERPINE2,PLAAT3,IL12RB2,PIK3CG,ANOS1,COL4A4,TPP1,STIL,ANKFN1,NUP160,IQGAP1,MYCBP2,LATS1,KLHL12,OR9Q1,WNT2B,MAP2,FBXW11,HSPH1,HLA-DMA,PEMT,CHD5,JAK1,ITGA9,KCNB1,GNPAT,ANKRD27,AIM2,ANK3,KITLG,ARID4B,RELN,CRKL,TENT5A,ARHGEF10,VAV3,CD40LG,DPYSL3,SCN9A,ATRN,CCDC88A,NDUFB9,ARNT2,SPOCK1,PLCE1,TACC2,CYP39A1,FRAS1,MSH3,BAK1,LAX1,TENM4,PPP3CA,CHRNA5,DYSF,NKD1,RBPJ,RIN2,PDE6C,DLX6-AS1,ADAMTS16,TAF10,MBD5,ASAH2,FRYL,ASH1L,RSPO2,OR5K1,OR13C9,DISC1,PROX1,FMN1,PIEZO2,VSIG1,ITGB1,CORO1C,NEFL,NAV2,STK3,FCAR,C1GALT1,MSR1,EXOSC3,TRPC4,RAMP3,ENPP2,DOCK4,BCL11B,CERS3,PLS3,TSPAN2,PCSK2,NAP1L1,PLPP4,COL4A2,ARHGAP32,D

			YRK3,CHRNA6,HDAC4,RBM45,IQCE,RPGRIP1L,MYLK,UNC5C,CFAP69,TLR7,CFL1,HMG20A,CEP85L,TUSC3,PLEKHG4B,RBBP8,RNASEH2B,PHF2,RAI2,LRP2,MTDH,WWC3,CAMK4,VWC2,TMEM108,SEZ6L,SLC39A8,UPRT,ATXN1,TMEM59L,ADAM17,SEMA4F,ELAVL1,TRAK1,STXBP1,OR6N1,IDH1,LMOD2,MUC13,NHLH1,SNTB1,CYP8B1,APC,TTLL5,INO80,PEAR1,PPARGC1B,CTRB1,COL27A1,TAF5L,CTNND2,DPYSL2,RAB8B,NSD2,GRM8,AARS1,CARD18,PSMC2,MAPKAP1,HSD17B4,EFHD1,PIGR,NRP2,FOXJ2,NR3C1,DLX6,FSTL4,ZC3HAV1,MTOR,SNAP25,DEFB124,OR11H6,BTBD2,RAB6B,TBXAS1,SPRED1,GALC,CNTNAP4,CDH26,CRIM1,RBM46,ABCB5,PER2,ANKRD24,GLI3,KAT6A,BCOR,NTRK3,RXFP1,GAB2,DIS3L2,PDCD10,ADGRG6,FERMT2,GLUD1,DNM3,EPB41,DENND5A,SS18,SFRP5,PPARA,CD244,NFASC,PHF6,FGF7,RTTN,EDA,TNFSF13B,PTH2R,COL14A1,RBM47,JARID2,HDAC2,TPO,INSYN2B,SEMA3D,NPSR1,RRM1,CADM1,INPP5D,OR4C6,ATF6,PRKAR1B,GBF1,SERPINE1,SLC26A5,AKAP13,PKM,GCG,PTPN3,STAC,SCN8A,RTL4,ST8SIA2,IRF8,RFX4,CCDC198,OR6K6,REM1,FREM1,RIPOR2,FUNDC2,XIRP2,KDM3A,HSPG2,GCNT1,USH1C,DNAJC13
GO:0007264	small GTPase-mediated signal transduction	0.00036651382372321665	*MYO9B,SEMA4D,MET,RASA4B,ARAP1,ITSN1,PREX2,ARHGAP26,SHTN1,DOCK7,PSD3,DBNL,RAPGEF1,ARHGEF3,DENND4A,RTN4,ARHGEF11,CHM,PIK3CG,ARHGAP6,KITLG,RELN,CRKL,ARHGEF10,VAV3,CCDC88A,PLCE1,RIN2,PLEKHG7,DENND1A,RALGPS1,ITGB1,CHN2,SH2D3C,DOCK4,CYTH4,ARHGAP32,CFL1,PLEKHG4B,PLEKHG1,MAPKAP1,SIPA1L2,KSR1,DOCK9,FGD1,GBF1,CCDC88C,AKAP13,SSX2IP,RIPOR2*
GO:0048858	cell projection morphogenesis	0.0004519620651356858	*MYO9B,XK,SEMA4D,SDC2,PREX2,TANC2,IL1RAPL1,KIF5C,GPM6A,ADCY1,ZDHHC17,LPAR3,EGFR,NEO1,SHTN1,SH3GL2,DOCK7,NYAP2,DBNL,RYK,MACF1,PTPRZ1,NEDD4,OLFM1,EFNB3,SNX2,RTN4,ANOS1,IQGAP1,MYCBP2,MAP2,ANKRD27,ANK3,RELN,PPP3CA,DISC1,ITGB1,CORO1C,NEFL,ENPP2,BCL11B,ARHGAP32,UNC5C,CFL1,PLEKHG4B,LRP2,TMEM108,ADAM17,SEMA4F,TRAK1,STXBP1,CTNND2,PACSIN2,NRP2,FSTL4,SNAP25,GLI3,DNM3,NFASC,SEMA3D,ST8SIA2*
GO:0120039	plasma membrane bounded cell projection morphogenesis	0.0007781705159255558	*MYO9B,XK,SEMA4D,SDC2,PREX2,TANC2,IL1RAPL1,KIF5C,GPM6A,ADCY1,ZDHHC17,LPAR3,EGFR,NEO1,SHTN1,SH3GL2,DOCK7,NYAP2,DBNL,RYK,MACF1,PTPRZ1,NEDD4,OLFM1,EFNB3,SNX2,RTN4,ANOS1,IQGAP1,MYCBP2,MAP2,ANKRD27,ANK3,RELN,PPP3CA,DISC1,ITGB1,CORO1C,NEFL,ENPP2,BCL11B,ARHGAP32,UNC5C,CFL1,PLEKHG4B,LRP2,TMEM108,ADAM17,SEMA4F,TRAK1,STXBP1,CTNND2,NRP2,FSTL4,SNAP25,GLI3,DNM3,NFASC,SEMA3D,ST8SIA2*
GO:0031344	regulation of cell projection organization	0.0008185129420642737	*XK,SEMA4D,SDC2,ARAP1,NFE2L2,TANC2,CSMD3,SRGAP2C,ITGA2,IL1RAPL1,GPM6A,PLPPR5,LPAR3,OCLN,NEO1,MPHOSPH9,PIK3R1,SHTN1,ROR1,RYK,MACF1,NEDD4,EFNB3,RAPGEF1,GRIN2B,RTN4,MYO3A,MYCBP2,MAP2,ANKRD27,RELN,CRKL,DPYSL3,CCDC88A,SPOCK1,PLCE1,PPP3CA,ADAMTS16,DISC1,CORO1C,NEFL,ENPP2,GLCE,ARHGAP32,CFL1,SEMA4F,TRAK1,APC,DPYSL2,RAB8B,FSTL4,MTOR,SNAP25,NTRK3,DNM3,DENND5A,HDAC2,SEMA3D,RIPOR2,USH1C*

GO:0065007	biological regulation	0.0009671386906864191	*KLHL13,ABCB7,PBX4,NHLRC3,MYO9B,COL4A5,ERG,PARN,STK16,XK,SEMA4D,MET,MAX,TEAD1,EIF4G3,ACSM3,SIAH3,DUS3L,RASA4B,GAS2L1,CHMP1B,ELAPOR2,ZNF534,TOX3,DNAH11,SLC9A9,EXOC2,TACR1,SNX25,SDC2,ABHD12,CIITA,ARAP1,ANKLE2,CEP85,RPRD1B,AHNAK,ACTR5,EIF4A3,DLEC1,NFE2L2,SCHIP1,TFEC,NFYC,CYP19A1,SLC46A2,DTNB,GPR158,VRK2,ITSN1,ATP2B2,PREX2,SCRN1,ELAVL2,TANC2,CSMD3,NTN4,SERP1,POLR1A,RAB11FIP4,HSFX4,SNTA1,ASZ1,NUB1,SRGAP2C,WIF1,FLI1,ESRRB,ARHGAP26,ZFHX2,LINC-PINT,TRHDE,RAB44,SETBP1,ZBTB7C,ITGA2,IL1RAPL1,BLK,GPM6A,WNT16,SETD3,ADCY1,GRID1,RPS6KA2,PLPPR5,TRIM24,ZDHHC17,FIG4,FOXP2,KMT2D,LPAR3,ANO4,TXLNG,SP110,SH3BP2,SH3TC2,CEP290,TACC1,OCLN,FASTKD5,NETO1,EGFR,GBE1,SNX32,PCGEM1,NEO1,MYT1L,PLEKHB2,TCERG1,F10,CABIN1,PSMD10,FBXL17,EOLA1,SLC9A4,DHX29,GPAM,IRF2,LRP12,ESRP1,HSFX3,BRMS1L,RGS12,CBX3,SLC39A10,ZNF496,ZNF550,MPHOSPH9,RNF10,FAT1,SLC2A2,PIK3R1,IL17B,NOVA1,SARNP,SHTN1,ATG12,MASP1,CD200R1,SH3GL2,CRTC1,PITPNC1,SLC30A3,DOCK7,PDK3,XKR4,ROR1,PAX5,AAK1,CRB1,UTP15,ARID1A,THEMIS,SMAD6,RYBP,NYAP2,PSD3,ABCA13,ZNF519,MEF2C,FZR1,DBNL,ELP4,RYK,MACF1,PXT1,WEE2,TFDP2,PTPRE,SERINC3,ZNF347,PTPN12,PTPRZ1,PLN,CTDSPL2,NEDD4,NEK4,CACNA1E,FLYWCH1,LRRC38,CYSLTR2,PARVA,SECISBP2,URI1,SLC9C1,SCML2,CHD6,ZNF609,PRDX1,AEBP2,DSC2,SERPINA1,OLFM1,MID1,TSHZ2,POGK,SLC12A2,EFNB3,OTX1,TRIM37,DLG3,PVRIG,FLOT2,SV2B,ILK,LEF1,EYA1,MAPK14,ZNF816-ZNF321P,MYBPC3,ZFHX4,RAPGEF1,GPRC6A,ZNF516,SLC1A2,ARHGEF3,GABRB2,GRIN2B,IFNGR2,DENND4A,TBC1D20,OXR1,SFMBT2,SCAMP5,IL17RB,PARP2,RDH16,FCHSD2,SLC4A7,DNAJB6,MTUS1,LEMD3,PIEZO1,OR2T11,NEB,CSRNP3,MRGPRX1,TMEM100,VDR,NEK11,CREB3L2,RTN4,GNL3L,CACNA1D,PRKAA2,ARHGEF11,IVNS1ABP,SGPP2,DPT,BBS2,CLSTN1,ZNF155,SERGEF,SCYL2,SKAP1,CHM,SKIC3,MYO3A,SERPINE2,PLAAT3,IL12RB2,PIK3CG,STIL,ANKFN1,ATP6V1E2,IQGAP1,MYCBP2,WIPI1,LATS1,ZSCAN25,KLHL12,LOC349160,SNX30,FANCI,OR9Q1,WNT2B,TNFRSF10B,MAP2,FBXW11,MTCL1,HSPH1,AZIN2,IYD,SMG6,HLA-DMA,PEMT,ARHGAP6,CHD5,JAK1,ITGA9,RADIL,KCNB1,HSD17B12,GRM4,ANKRD27,AIM2,ANK3,ZNF19,KITLG,ARID4B,GMDS,PLEKHM2,NR1I2,ZNF266,RFX7,RELN,CRKL,TENT5A,ARHGEF10,SNAP91,ZNF816,VAV3,RPS6KC1,CD40LG,VRK1,GPAT3,DPYSL3,ZNF420,SPRING1,SLC16A10,ATRN,CCDC88A,SLC35B4,ARNT2,SPOCK1,HPSE2,PLCE1,TNIP3,DCAF5,GNAL,STK32C,MSH3,BAK1,LAX1,STK26,TENM4,PPP3CA,CHRNA5,MKNK1,DYSF,NKD1,RBPJ,RIN2,PDE6C,PLEKHG7,DLX6-AS1,ASCC1,DENND1A,ENSG00000290317,ADAMTS16,TASP1,MICU1,TAF10,MBD5,BACE2,KDM5A,ZNF549,CNOT6,PKIB,ASAH2,STON1,ASH1L,RSPO2,ZNF805,OR5K1,OR13C9,DISC1,PROX1,*

			FMN1,ZNF891,RALGPS1,PIEZO2,ITGB1,ZNF827,CORO1C,SPIDR,NEFL,NAV2,STK3,CHN2,FCAR,HNF4G,MSR1,EXOSC3,GABRG3,SH2D3C,APTX,RAMP3,GNG2,ZNF705A,FRS3,LIMS1,ENPP2,DOCK4,ACKR2,MCTP2,BCL11B,GLCE,PCSK2,KCNIP1,PWWP2A,LOC105373170,TBC1D10A,SSBP2,RPH3A,CTTNBP2,CYTH4,NAP1L1,PLPP4,UBR1,CNN2,COL4A2,SLC1A6,ARHGAP32,DYRK3,IP6K2,CHRNA6,HDAC4,FYB1,RPGRIP1L,MYLK,UNC5C,CFAP69,TLR7,P3H2,CFL1,SLAIN2,HMG20A,PLEKHG4B,RBBP8,MED13,RNASEH2B,PHF2,TAAR2,AFDN,LRP2,CYTIP,MTDH,ZDHHC13,WWC3,CAMK4,VWC2,RPA3,TMEM108,SEZ6L,SLC39A8,UPRT,ATXN1,ADAM17,SEMA4F,ELAVL1,TRAK1,STXBP1,OR6N1,IDH1,LMOD2,HDGFL2,PLEKHG1,NHLH1,ELP2,CYP8B1,APC,INO80,PEAR1,CDA,PPARGC1B,ESR2,ERC1,ZNF440,RUBCNL,AGO3,C9,PVR,ACER1,IQGAP2,CDYL,RLF,ANKRA2,TAF5L,CTNND2,RASSF3,PACSIN2,DPYSL2,GIMAP8,RAB8B,METTL8,NSD2,GRM8,AARS1,JADE3,CARD18,PSMC2,MAPKAP1,EVC2,GON4L,MGAT5,NUP153,HSD17B4,EFHD1,PIGR,NRP2,SIPA1L2,CAVIN1,TMEM165,FOXJ2,NR3C1,DLX6,BORCS5,FSTL4,ZC3HAV1,MTOR,KSR1,FAF1,SNAP25,DEFB124,CHST4,OR11H6,ZNF221,PIP5K1A,GH2,TBXAS1,ASCC2,SPRED1,GNG4,CNTNAP4,PDS5B,CRIM1,RBM46,ABCB5,RNF115,CDHR3,PER2,GLI3,KAT6A,BCOR,KCNK12,NTRK3,RXFP1,TADA1,GAB2,DIS3L2,PDCD10,PDE1A,PRAMEF11,HHAT,CREB5,CCDC68,ADGRG6,GOLGA7,FERMT2,GPR82,GLUD1,TSPOAP1,DNM3,PHIP,EPB41,DENND5A,SS18,SFRP5,DOCK9,ZYG11B,PPARA,CD244,SHISA6,HDX,CPEB2,ZNF229,PHF6,HMGB4,FGF7,TENT5D,STXBP6,MGA,CACNA1A,EDA,LIMCH1,TNFSF13B,PTH2R,COL14A1,RBM47,EIF2A,JARID2,FIRRM,HDAC2,TPO,FGD1,TRIM39,INSYN2B,TRRAP,SEMA3D,NPSR1,EIF4E3,KCNH5,RRM1,MAGEA1,CADM1,INPP5D,CLYBL,OR4C6,ATF6,PRKAR1B,GBF1,SERPINE1,CCDC88C,BACH1,CUL9,PRAMEF4,EWSR1,TM7SF3,SLC26A5,AKAP13,PKM,GCG,ATP12A,TKFC,LINC02218,PTPN3,STAC,SCN8A,NNT,SSX2IP,ST8SIA2,ELP1,IRF8,HIVEP3,RFX4,OR6K6,ZNF30,REM1,RIPOR2,FUNDC2,KDM3A,CAST,ADGRA3,GPR39,CASK,HSPG2,GCNT1,USH1C,NLRP14,DNAJC13,PSME3IP1
GO:0030154	cell differentiation	0.002761156909061044	*PBX4,XK,SEMA4D,MET,SCYL3,EIF4G3,SDC2,FANCC,DLEC1,NFE2L2,SLC46A2,DTNB,ATP2B2,PREX2,TANC2,CSMD3,NTN4,PLEKHM3,ASZ1,SRGAP2C,WIF1,FLI1,ESRRB,ZFHX2,ZBTB7C,ITGA2,IL1RAPL1,KIF5C,BLK,GPM6A,SPATA16,WNT16,KRT86,SETD3,ADCY1,RPS6KA2,PLPPR5,ZDHHC17,FIG4,CFAP47,KMT2D,LPAR3,SH3TC2,CEP290,SH3PXD2B,CFAP91,EGFR,PTPRQ,NEO1,MYT1L,PLEKHB2,FBXL17,SLC9A4,LRP12,ESRP1,SLC39A10,RNF10,FAT1,PIK3R1,SHTN1,SH3GL2,DOCK7,ROR1,PAX5,CRB1,ARID1A,THEMIS,SMAD6,NYAP2,MEF2C,FZR1,DBNL,RYK,MACF1,WEE2,PTPRZ1,PLN,EML1,NEDD4,SECISBP2,SLC9C1,ZNF609,OLFM1,EFNB3,FLOT2,ILK,LEF1,EYA1,MAPK14,RAPGEF1,ZNF516,GABRB2,TBC1D20,PARP2,ZSWIM6,SLC4A7,DNAJB6,PIEZO1,NEB,TMEM100,VDR,CREB3L2,RTN4,BBS2,SCYL2,SERPINE2,PLAAT3,ANOS1,TPP1,IQGAP1*

			,MYCBP2,LATS1,KLHL12,WNT2B,MAP2,FBXW11,NHSL1,CHD5,JAK1,RADIL,KCNB1,ANKRD27,ANK3,KITLG,ARID4B,NR1I2,RELN,CRKL,TENT5A,ARHGEF10,CD40LG,DPYSL3,CCDC88A,SPOCK1,BAK1,TENM4,PPP3CA,NKD1,RBPJ,RIN2,PDE6C,TAF10,FRYL,RSPO2,DISC1,PROX1,VSIG1,ITGB1,DRC7,CORO1C,NEFL,NAV2,STK3,C1GALT1,HNF4G,MSR1,TRPC4,BCL11B,CERS3,TSPAN2,NAP1L1,CNN2,COL4A2,ARHGAP32,DYRK3,HDAC4,RBM45,RPGRIP1L,UNC5C,CFAP69,CFL1,DTYMK,WDR7,HMG20A,CEP85L,PLEKHG4B,AFDN,LRP2,CAMK4,VWC2,TMEM108,ADAM17,SEMA4F,TRAK1,STXBP1,LMOD2,HDGFL2,NHLH1,APC,PPARGC1B,COL27A1,ACER1,CDYL,CTNND2,DPYSL2,METTL8,PSMC2,HSD17B4,EFHD1,RAI14,NRP2,FOXJ2,NR3C1,DLX6,FSTL4,MTOR,SNAP25,PIP5K1A,BTBD2,RAB6B,SPRED1,CRIM1,MEI4,RBM46,ABCB5,PER2,ANKRD24,GLI3,KAT6A,NTRK3,RXFP1,GAB2,PRAMEF11,ADGRG6,FERMT2,DNM3,DENND5A,SS18,SFRP5,PPARA,NFASC,FGF7,MGA,EDA,TNFSF13B,COL14A1,RBM47,JARID2,HDAC2,TPO,SEMA3D,CADM1,INPP5D,SERPINE1,CCDC88C,PRAMEF4,AKAP13,ST8SIA2,IRF8,HIVEP3,RIPOR2,KDM3A,HSPG2,SLCO4C1,USH1C,NLRP14,DNAJC13
GO:0048869	cellular developmental process	0.002813658963660346	PBX4,XK,SEMA4D,MET,SCYL3,EIF4G3,SDC2,FANCC,DLEC1,NFE2L2,SLC46A2,DTNB,ATP2B2,PREX2,TANC2,CSMD3,NTN4,PLEKHM3,ASZ1,SRGAP2C,WIF1,FLI1,ESRRB,ZFHX2,ZBTB7C,ITGA2,IL1RAPL1,KIF5C,BLK,GPM6A,SPATA16,WNT16,KRT86,SETD3,ADCY1,RPS6KA2,PLPPR5,ZDHHC17,FIG4,CFAP47,KMT2D,LPAR3,SH3TC2,CEP290,SH3PXD2B,CFAP91,EGFR,PTPRQ,NEO1,MYT1L,PLEKHB2,FBXL17,SLC9A4,LRP12,ESRP1,SLC39A10,RNF10,FAT1,PIK3R1,SHTN1,SH3GL2,DOCK7,ROR1,PAX5,CRB1,ARID1A,THEMIS,SMAD6,NYAP2,MEF2C,FZR1,DBNL,RYK,MACF1,WEE2,PTPRZ1,PLN,EML1,NEDD4,SECISBP2,SLC9C1,ZNF609,OLFM1,EFNB3,FLOT2,ILK,LEF1,EYA1,MAPK14,RAPGEF1,ZNF516,GABRB2,TBC1D20,PARP2,ZSWIM6,SLC4A7,DNAJB6,PIEZO1,NEB,TMEM100,VDR,CREB3L2,RTN4,BBS2,SCYL2,SERPINE2,PLAAT3,ANOS1,TPP1,IQGAP1,MYCBP2,LATS1,KLHL12,WNT2B,MAP2,FBXW11,NHSL1,CHD5,JAK1,RADIL,KCNB1,ANKRD27,ANK3,KITLG,ARID4B,NR1I2,RELN,CRKL,TENT5A,ARHGEF10,CD40LG,DPYSL3,CCDC88A,SPOCK1,BAK1,TENM4,PPP3CA,NKD1,RBPJ,RIN2,PDE6C,TAF10,FRYL,RSPO2,DISC1,PROX1,VSIG1,ITGB1,DRC7,CORO1C,NEFL,NAV2,STK3,C1GALT1,HNF4G,MSR1,TRPC4,BCL11B,CERS3,TSPAN2,NAP1L1,CNN2,COL4A2,ARHGAP32,DYRK3,HDAC4,RBM45,RPGRIP1L,UNC5C,CFAP69,CFL1,DTYMK,WDR7,HMG20A,CEP85L,PLEKHG4B,AFDN,LRP2,CAMK4,VWC2,TMEM108,ADAM17,SEMA4F,TRAK1,STXBP1,LMOD2,HDGFL2,NHLH1,APC,PPARGC1B,COL27A1,ACER1,CDYL,CTNND2,DPYSL2,METTL8,PSMC2,HSD17B4,EFHD1,RAI14,NRP2,FOXJ2,NR3C1,DLX6,FSTL4,MTOR,SNAP25,PIP5K1A,BTBD2,RAB6B,SPRED1,CRIM1,MEI4,RBM46,ABCB5,PER2,ANKRD24,GLI3,KAT6A,NTRK3,RXFP1,GAB2,PRAMEF11,ADGRG6,FERMT2,DNM3,DENND5A,SS18,SFRP5,PPARA,NFASC,FGF7,MGA,EDA,TNFSF13B,COL14A1,RBM47,JARID

			2,HDAC2,TPO,SEMA3D,CADM1,INPP5D,SERPINE1,CCDC88C,PRAMEF4,AKAP13,ST8SIA2,IRF8,HIVEP3,RIPOR2,KDM3A,HSPG2,SLCO4C1,USH1C,NLRP14,DNAJC13
GO:0031175	neuron projection development	0.0032776764327834213	*XK,SEMA4D,SDC2,NFE2L2,PREX2,TANC2,CSMD3,SRGAP2C,IL1RAPL1,KIF5C,GPM6A,ADCY1,PLPPR5,ZDHHC17,LPAR3,EGFR,NEO1,LRP12,SHTN1,SH3GL2,DOCK7,ROR1,NYAP2,MEF2C,DBNL,RYK,MACF1,PTPRZ1,NEDD4,OLFM1,EFNB3,RAPGEF1,RTN4,ANOS1,IQGAP1,MYCBP2,MAP2,ANKRD27,ANK3,RELN,CRKL,DPYSL3,CCDC88A,SPOCK1,PPP3CA,FRYL,DISC1,ITGB1,NEFL,BCL11B,TSPAN2,ARHGAP32,UNC5C,CFL1,PLEKHG4B,LRP2,TMEM108,ADAM17,SEMA4F,TRAK1,STXBP1,CTNND2,DPYSL2,EFHD1,NRP2,FSTL4,SNAP25,RAB6B,ANKRD24,GLI3,NTRK3,DNM3,DENND5A,NFASC,HDAC2,SEMA3D,ST8SIA2,RIPOR2,USH1C*
GO:0120035	regulation of plasma membrane bounded cell projection organization	0.0038323978142119353	*XK,SEMA4D,SDC2,ARAP1,NFE2L2,TANC2,CSMD3,SRGAP2C,IL1RAPL1,GPM6A,PLPPR5,LPAR3,OCLN,NEO1,MPHOSPH9,PIK3R1,SHTN1,ROR1,RYK,MACF1,NEDD4,EFNB3,RAPGEF1,GRIN2B,RTN4,MYO3A,MYCBP2,MAP2,ANKRD27,RELN,CRKL,DPYSL3,CCDC88A,SPOCK1,PLCE1,PPP3CA,ADAMTS16,DISC1,CORO1C,NEFL,ENPP2,ARHGAP32,CFL1,SEMA4F,TRAK1,APC,DPYSL2,FSTL4,MTOR,SNAP25,NTRK3,DNM3,DENND5A,HDAC2,SEMA3D,RIPOR2,USH1C*
GO:0048513	animal organ development	0.005710450682558214	*PBX4,XK,SEMA4D,MET,TEAD1,MMP16,DNAH11,FANCC,EIF4A3,CYP19A1,SLC46A2,GPR158,ATP2B2,NTN4,SERP1,SRGAP2C,WIF1,FLI1,ESRRB,ITGA2,GPM6A,WNT16,KRT86,RPS6KA2,FOXP2,TXLNG,CEP290,TACC1,SH3PXD2B,EGFR,PTPRQ,FBXL17,ESRP1,SLC39A10,FAT1,DOCK7,ROR1,PAX5,CRB1,SMAD6,MEF2C,FZR1,RYK,TFDP2,PLN,EML1,PARVA,SECISBP2,ZNF609,ATG4B,OLFM1,OTX1,ILK,LEF1,EYA1,MAPK14,MYBPC3,ZNF516,SLC1A2,GABRB2,GRIN2B,TBC1D20,PARP2,ZSWIM6,SLC4A7,DNAJB6,NEB,TMEM100,VDR,CREB3L2,RTN4,BBS2,SCYL2,MYO3A,SERPINE2,PLAAT3,COL4A4,STIL,NUP160,IQGAP1,LATS1,KLHL12,WNT2B,FBXW11,CHD5,RADIL,GNPAT,AIM2,KITLG,ARID4B,RELN,CRKL,TENT5A,ATRN,ARNT2,PLCE1,TACC2,FRAS1,BAK1,TENM4,PPP3CA,NKD1,RBPJ,PDE6C,DLX6-AS1,ADAMTS16,TAF10,ASH1L,RSPO2,DISC1,PROX1,FMN1,ITGB1,CORO1C,NEFL,STK3,C1GALT1,BCL11B,CERS3,PLS3,ARHGAP32,HDAC4,RPGRIP1L,MYLK,UNC5C,FLG2,PHF2,RAI2,LRP2,TMEM108,UPRT,ATXN1,TMEM59L,ADAM17,SEMA4F,IDH1,HDGFL2,APC,TTLL5,PPARGC1B,COL27A1,ACER1,METTL8,NSD2,AARS1,MAPKAP1,HSD17B4,NRP2,NR3C1,DLX6,MTOR,PIP5K1A,GH2,SPRED1,ABCB5,ANKRD24,GLI3,BCOR,NTRK3,RXFP1,ADGRG6,FERMT2,GLUD1,PPARA,NFASC,FGF7,EDA,COL14A1,JARID2,HDAC2,TPO,FGD1,SEMA3D,RRM1,CADM1,ATF6,SERPINE1,SLC26A5,AKAP13,PTPN3,HIVEP3,RFX4,FREM1,RIPOR2,XIRP2,HSPG2,GCNT1,USH1C*
GO:0050789	regulation of biological process	0.007439655352113255	*KLHL13,ABCB7,PBX4,NHLRC3,MYO9B,COL4A5,ERG,PARN,STK16,XK,SEMA4D,MET,MAX,TEAD1,EIF4G3,SIAH3,DUS3L,RASA4B,GAS2L1,CHMP1B,ELAPOR2,ZNF534,TOX3,DNAH11,EXOC2,TA*

			CR1,SNX25,SDC2,ABHD12,CIITA,ARAP1,ANKLE2,CEP85,RPRD1B,AHNAK,ACTR5,EIF4A3,DLEC1,NFE2L2,SCHIP1,TFEC,NFYC,CYP19A1,SLC46A2,DTNB,GPR158,VRK2,ITSN1,ATP2B2,PREX2,SCRN1,ELAVL2,TANC2,CSMD3,NTN4,SERP1,POLR1A,RAB11FIP4,HSFX4,SNTA1,ASZ1,NUB1,SRGAP2C,WIF1,FLI1,ESRRB,ARHGAP26,ZFHX2,LINC-PINT,TRHDE,RAB44,SETBP1,ZBTB7C,ITGA2,IL1RAPL1,BLK,GPM6A,WNT16,SETD3,ADCY1,GRID1,RPS6KA2,PLPPR5,TRIM24,ZDHHC17,FIG4,FOXP2,KMT2D,LPAR3,TXLNG,SP110,SH3BP2,SH3TC2,CEP290,TACC1,OCLN,FASTKD5,NETO1,EGFR,GBE1,SNX32,PCGEM1,NEO1,MYT1L,PLEKHB2,TCERG1,F10,CABIN1,PSMD10,FBXL17,EOLA1,DHX29,GPAM,IRF2,LRP12,ESRP1,HSFX3,BRMS1L,RGS12,CBX3,SLC39A10,ZNF496,ZNF550,MPHOSPH9,RNF10,FAT1,SLC2A2,PIK3R1,IL17B,NOVA1,SARNP,SHTN1,ATG12,MASP1,CD200R1,SH3GL2,CRTC1,PITPNC1,SLC30A3,DOCK7,PDK3,ROR1,PAX5,AAK1,CRB1,UTP15,ARID1A,THEMIS,SMAD6,RYBP,NYAP2,PSD3,ABCA13,ZNF519,MEF2C,FZR1,DBNL,ELP4,RYK,MACF1,PXT1,WEE2,TFDP2,PTPRE,SERINC3,ZNF347,PTPN12,PTPRZ1,PLN,CTDSPL2,NEDD4,NEK4,CACNA1E,FLYWCH1,LRRC38,CYSLTR2,PARVA,SECISBP2,URI1,SCML2,CHD6,ZNF609,PRDX1,AEBP2,DSC2,OLFM1,MID1,TSHZ2,POGK,SLC12A2,EFNB3,OTX1,TRIM37,DLG3,PVRIG,FLOT2,SV2B,ILK,LEF1,EYA1,MAPK14,ZNF816-ZNF321P,MYBPC3,ZFHX4,RAPGEF1,GPRC6A,ZNF516,SLC1A2,ARHGEF3,GABRB2,GRIN2B,IFNGR2,DENND4A,TBC1D20,OXR1,SFMBT2,SCAMP5,IL17RB,PARP2,FCHSD2,DNAJB6,MTUS1,LEMD3,PIEZO1,OR2T11,NEB,CSRNP3,MRGPRX1,TMEM100,VDR,NEK11,CREB3L2,RTN4,GNL3L,CACNA1D,PRKAA2,ARHGEF11,IVNS1ABP,SGPP2,DPT,BBS2,CLSTN1,ZNF155,SERGEF,SCYL2,SKAP1,CHM,SKIC3,MYO3A,SERPINE2,PLAAT3,IL12RB2,PIK3CG,STIL,ANKFN1,ATP6V1E2,IQGAP1,MYCBP2,WIPI1,LATS1,ZSCAN25,KLHL12,LOC349160,SNX30,FANCI,OR9Q1,WNT2B,TNFRSF10B,MAP2,FBXW11,MTCL1,HSPH1,AZIN2,SMG6,HLA-DMA,PEMT,ARHGAP6,CHD5,JAK1,ITGA9,RADIL,KCNB1,HSD17B12,GRM4,ANKRD27,AIM2,ANK3,ZNF19,KITLG,ARID4B,GMDS,PLEKHM2,NR1I2,ZNF266,RFX7,RELN,CRKL,TENT5A,ARHGEF10,SNAP91,ZNF816,VAV3,RPS6KC1,CD40LG,VRK1,GPAT3,DPYSL3,ZNF420,SPRING1,SLC16A10,ATRN,CCDC88A,SLC35B4,ARNT2,SPOCK1,HPSE2,PLCE1,TNIP3,DCAF5,GNAL,STK32C,MSH3,BAK1,LAX1,STK26,TENM4,PPP3CA,CHRNA5,MKNK1,DYSF,NKD1,RBPJ,RIN2,PDE6C,PLEKHG7,DLX6-AS1,ASCC1,DENND1A,ENSG00000290317,ADAMTS16,TASP1,MICU1,TAF10,MBD5,BACE2,KDM5A,ZNF549,CNOT6,PKIB,ASAH2,STON1,ASH1L,RSPO2,ZNF805,OR5K1,OR13C9,DISC1,PROX1,FMN1,ZNF891,RALGPS1,ITGB1,ZNF827,CORO1C,SPIDR,NEFL,STK3,CHN2,FCAR,HNF4G,MSR1,EXOSC3,GABRG3,SH2D3C,RAMP3,GNG2,ZNF705A,FRS3,LIMS1,ENPP2,DOCK4,ACKR2,MCTP2,BCL11B,GLCE,KCNIP1,PWWP2A,LOC105373170

			,*TBC1D10A,SSBP2,RPH3A,CTTNBP2,CYTH4,NAP1L1,PLPP4,UBR1,CNN2,COL4A2,SLC1A6,ARHGAP32,DYRK3,IP6K2,CHRNA6,HDAC4,FYB1,RPGRIP1L,MYLK,UNC5C,CFAP69,TLR7,P3H2,CFL1,SLAIN2,HMG20A,PLEKHG4B,RBBP8,MED13,RNASEH2B,PHF2,TAAR2,AFDN,LRP2,CYTIP,MTDH,ZDHHC13,WWC3,CAMK4,VWC2,RPA3,TMEM108,SEZ6L,SLC39A8,ATXN1,ADAM17,SEMA4F,ELAVL1,TRAK1,STXBP1,OR6N1,IDH1,LMOD2,HDGFL2,PLEKHG1,NHLH1,ELP2,CYP8B1,APC,INO80,PEAR1,CDA,PPARGC1B,ESR2,ERC1,ZNF440,RUBCNL,AGO3,C9,PVR,ACER1,IQGAP2,CDYL,RLF,ANKRA2,TAF5L,CTNND2,RASSF3,PACSIN2,DPYSL2,GIMAP8,RAB8B,METTL8,NSD2,GRM8,AARS1,JADE3,CARD18,PSMC2,MAPKAP1,EVC2,GON4L,MGAT5,NUP153,EFHD1,PIGR,NRP2,SIPA1L2,CAVIN1,FOXJ2,NR3C1,DLX6,BORCS5,FSTL4,ZC3HAV1,MTOR,KSR1,FAF1,SNAP25,DEFB124,CHST4,OR11H6,ZNF221,PIP5K1A,GH2,TBXAS1,ASCC2,SPRED1,GNG4,CNTNAP4,PDS5B,CRIM1,RBM46,RNF115,CDHR3,PER2,GLI3,KAT6A,BCOR,NTRK3,RXFP1,TADA1,GAB2,DIS3L2,PDCD10,PDE1A,PRAMEF11,HHAT,CREB5,CCDC68,ADGRG6,FERMT2,GPR82,GLUD1,TSPOAP1,DNM3,PHIP,EPB41,DENND5A,SS18,SFRP5,DOCK9,ZYG11B,PPARA,CD244,SHISA6,HDX,CPEB2,ZNF229,PHF6,HMGB4,FGF7,TENT5D,STXBP6,MGA,CACNA1A,EDA,LIMCH1,TNFSF13B,PTH2R,COL14A1,RBM47,EIF2A,JARID2,FIRRM,HDAC2,FGD1,TRIM39,INSYN2B,TRRAP,SEMA3D,NPSR1,EIF4E3,KCNH5,RRM1,MAGEA1,CADM1,INPP5D,CLYBL,OR4C6,ATF6,PRKAR1B,GBF1,SERPINE1,CCDC88C,BACH1,CUL9,PRAMEF4,EWSR1,TM7SF3,SLC26A5,AKAP13,PKM,GCG,TKFC,LINC02218,PTPN3,STAC,NNT,SSX2IP,ST8SIA2,ELP1,IRF8,HIVEP3,RFX4,OR6K6,ZNF30,REM1,RIPOR2,FUNDC2,KDM3A,CAST,ADGRA3,GPR39,CASK,HSPG2,GCNT1,USH1C,NLRP14,DNAJC13,PSME3IP1*
GO:0048518	positive regulation of biological process	0.007632922233070338	*ABCB7,PBX4,ERG,PARN,STK16,SEMA4D,MET,MAX,TEAD1,EIF4G3,ELAPOR2,TOX3,TACR1,CIITA,ARAP1,RPRD1B,AHNAK,ACTR5,EIF4A3,NFE2L2,SCHIP1,TFEC,NFYC,CYP19A1,SLC46A2,GPR158,SERP1,NUB1,SRGAP2C,WIF1,FLI1,ESRRB,ZBTB7C,ITGA2,IL1RAPL1,BLK,GPM6A,WNT16,SETD3,ADCY1,RPS6KA2,PLPPR5,TRIM24,ZDHHC17,KMT2D,LPAR3,CEP290,TACC1,OCLN,NETO1,EGFR,NEO1,TCERG1,F10,PSMD10,DHX29,GPAM,IRF2,BRMS1L,SLC39A10,ZNF496,RNF10,FAT1,SLC2A2,PIK3R1,IL17B,SHTN1,ATG12,MASP1,CRTC1,SLC30A3,DOCK7,ROR1,PAX5,AAK1,UTP15,ARID1A,THEMIS,SMAD6,RYBP,ABCA13,MEF2C,FZR1,RYK,MACF1,PXT1,WEE2,TFDP2,SERINC3,PTPRZ1,CTDSPL2,NEDD4,NEK4,LRRC38,SECISBP2,CHD6,ZNF609,OLFM1,MID1,SLC12A2,EFNB3,OTX1,TRIM37,DLG3,PVRIG,FLOT2,ILK,LEF1,EYA1,MAPK14,RAPGEF1,ZNF516,SLC1A2,ARHGEF3,GRIN2B,TBC1D20,SCAMP5,PARP2,FCHSD2,PIEZO1,CSRNP3,TMEM100,VDR,CREB3L2,RTN4,GNL3L,CACNA1D,PRKAA2,ARHGEF11,BBS2,CLSTN1,SKAP1,MYO3A,SERPINE2,IL12RB2,PIK3CG,STIL,IQGAP1,MYCBP2,WIPI1,LATS1,SNX30,FANCI,WNT2B,TNFRSF10B,MAP2,FBXW11,MTCL1,HSPH1,AZIN2,HLA-*

			DMA,PEMT,CHD5,JAK1,KCNB1,HSD17B12,GRM4,ANKRD27,AIM2,ANK3,KITLG,ARID4B,NR1I2,RFX7,RELN,CRKL,TENT5A,ARHGEF10,VAV3,CD40LG,VRK1,DPYSL3,SPRING1,CCDC88A,ARNT2,HPSE2,PLCE1,TNIP3,BAK1,LAX1,TENM4,PPP3CA,NKD1,RBPJ,RIN2,DLX6-AS1,TASP1,TAF10,MBD5,KDM5A,CNOT6,PKIB,ASH1L,RSPO2,DISC1,PROX1,FMN1,ITGB1,ZNF827,CORO1C,SPIDR,NEFL,STK3,FCAR,HNF4G,MSR1,EXOSC3,SH2D3C,RAMP3,LIMS1,ENPP2,DOCK4,BCL11B,GLCE,PWWP2A,TBC1D10A,SSBP2,NAP1L1,PLPP4,CNN2,ARHGAP32,DYRK3,IP6K2,HDAC4,FYB1,MYLK,UNC5C,CFAP69,TLR7,CFL1,SLAIN2,MED13,RNASEH2B,PHF2,AFDN,LRP2,MTDH,ZDHHC13,CAMK4,VWC2,TMEM108,ADAM17,SEMA4F,ELAVL1,TRAK1,STXBP1,LMOD2,HDGFL2,NHLH1,CYP8B1,APC,INO80,PEAR1,PPARGC1B,ESR2,AGO3,C9,PVR,RLF,TAF5L,RAB8B,METTL8,NSD2,PSMC2,MAPKAP1,MGAT5,NRP2,CAVIN1,FOXJ2,NR3C1,DLX6,BORCS5,ZC3HAV1,MTOR,KSR1,FAF1,SNAP25,DEFB124,CHST4,GH2,TBXAS1,SPRED1,RBM46,PER2,GLI3,KAT6A,NTRK3,TADA1,GAB2,DIS3L2,PDCD10,PRAMEF11,CREB5,FERMT2,GLUD1,DNM3,PHIP,EPB41,SS18,ZYG11B,PPARA,CD244,ZNF229,FGF7,TENT5D,MGA,EDA,LIMCH1,TNFSF13B,PTH2R,RBM47,EIF2A,HDAC2,TRIM39,TRRAP,SEMA3D,NPSR1,RRM1,CADM1,INPP5D,CLYBL,ATF6,PRKAR1B,SERPINE1,BACH1,PRAMEF4,TM7SF3,SLC26A5,AKAP13,PKM,GCG,TKFC,LINC02218,STAC,NNT,ST8SIA2,IRF8,HIVEP3,RFX4,RIPOR2,KDM3A,CASK,HSPG2,GCNT1
GO:0048812	neuron projection morphogenesis	0.0077588066528453715	XK,SEMA4D,SDC2,PREX2,TANC2,IL1RAPL1,KIF5C,GPM6A,ADCY1,ZDHHC17,LPAR3,EGFR,NEO1,SHTN1,SH3GL2,DOCK7,NYAP2,DBNL,RYK,MACF1,PTPRZ1,NEDD4,OLFM1,EFNB3,RTN4,ANOS1,IQGAP1,MYCBP2,MAP2,ANKRD27,ANK3,RELN,PPP3CA,DISC1,ITGB1,NEFL,BCL11B,ARHGAP32,UNC5C,CFL1,PLEKHG4B,LRP2,TMEM108,ADAM17,SEMA4F,TRAK1,STXBP1,CTNND2,NRP2,FSTL4,SNAP25,GLI3,DNM3,NFASC,SEMA3D,ST8SIA2
GO:0034330	cell junction organization	0.011166396150327102	COL4A5,SEMA4D,GPR158,TANC2,FBF1,SNTA1,SRGAP2C,ITGA2,IL1RAPL1,GPM6A,GRID1,OCLN,CLDN14,PIK3R1,MEF2C,DBNL,RYK,MACF1,NEDD4,EFNB3,MAPK14,RAPGEF1,GABRB2,GRIN2B,CLSTN1,MYCBP2,ARHGAP6,GNPAT,ANK3,RELN,CRKL,CDH10,PDZRN3,STON1,DISC1,FMN1,ITGB1,CORO1C,NEFL,LIMS1,CTTNBP2,TLN2,CFL1,AFDN,TMEM108,SEZ6L,APC,ERC1,CTNND2,RAB8B,NRP2,SNAP25,PIP5K1A,CDH26,CDHR3,FERMT2,DNM3,SHISA6,LIMCH1,ST8SIA2,XIRP2,CAST
GO:0048522	positive regulation of cellular process	0.011981489015041676	ABCB7,PBX4,ERG,PARN,STK16,SEMA4D,MET,MAX,TEAD1,EIF4G3,ELAPOR2,TOX3,TACR1,CIITA,ARAP1,RPRD1B,AHNAK,ACTR5,EIF4A3,NFE2L2,SCHIP1,TFEC,NFYC,CYP19A1,SLC46A2,GPR158,SERP1,NUB1,SRGAP2C,WIF1,FLI1,ESRRB,ZBTB7C,ITGA2,IL1RAPL1,BLK,GPM6A,WNT16,SETD3,ADCY1,RPS6KA2,PLPPR5,TRIM24,ZDHHC17,KMT2D,LPAR3,CEP290,TACC1,OCLN,NETO1,EGFR,NEO1,TCERG1,F10,PSMD10,DHX29,GPAM,IRF2,SLC39A10,ZNF496,RNF10,FAT1,SLC2A2,PIK3R1,IL17B,SHTN1,CRTC1,DOCK7,

			ROR1,PAX5,AAK1,UTP15,ARID1A,SMAD6,RYBP,ABCA13,MEF2C,FZR1,RYK,MACF1,PXT1,WEE2,TFDP2,SERINC3,PTPRZ1,CTDSPL2,NEDD4,NEK4,LRRC38,SECISBP2,CHD6,ZNF609,OLFM1,MID1,SLC12A2,EFNB3,OTX1,TRIM37,DLG3,FLOT2,ILK,LEF1,EYA1,MAPK14,RAPGEF1,ZNF516,SLC1A2,ARHGEF3,GRIN2B,TBC1D20,SCAMP5,PARP2,FCHSD2,PIEZO1,CSRNP3,TMEM100,VDR,CREB3L2,RTN4,GNL3L,PRKAA2,ARHGEF11,CLSTN1,SKAP1,MYO3A,SERPINE2,IL12RB2,PIK3CG,STIL,IQGAP1,MYCBP2,WIPI1,LATS1,SNX30,FANCI,TNFRSF10B,MAP2,FBXW11,MTCL1,HSPH1,AZIN2,HLA-DMA,PEMT,CHD5,JAK1,KCNB1,HSD17B12,GRM4,ANKRD27,AIM2,ANK3,KITLG,ARID4B,NR1I2,RFX7,RELN,CRKL,TENT5A,ARHGEF10,VAV3,CD40LG,VRK1,DPYSL3,SPRING1,CCDC88A,ARNT2,HPSE2,PLCE1,BAK1,TENM4,PPP3CA,NKD1,RBPJ,RIN2,DLX6-AS1,TASP1,TAF10,MBD5,KDM5A,CNOT6,PKIB,ASH1L,RSPO2,DISC1,PROX1,FMN1,ITGB1,ZNF827,CORO1C,SPIDR,NEFL,STK3,FCAR,HNF4G,MSR1,EXOSC3,SH2D3C,RAMP3,LIMS1,ENPP2,DOCK4,BCL11B,GLCE,PWWP2A,TBC1D10A,SSBP2,NAP1L1,CNN2,ARHGAP32,DYRK3,IP6K2,HDAC4,MYLK,UNC5C,CFAP69,TLR7,SLAIN2,MED13,RNASEH2B,PHF2,AFDN,LRP2,MTDH,ZDHHC13,CAMK4,VWC2,TMEM108,ADAM17,SEMA4F,ELAVL1,TRAK1,STXBP1,LMOD2,HDGFL2,NHLH1,APC,INO80,PEAR1,PPARGC1B,ESR2,AGO3,PVR,RLF,TAF5L,RAB8B,METTL8,NSD2,PSMC2,MAPKAP1,MGAT5,NRP2,CAVIN1,FOXJ2,NR3C1,DLX6,BORCS5,ZC3HAV1,MTOR,KSR1,FAF1,SNAP25,DEFB124,CHST4,GH2,SPRED1,RBM46,PER2,GLI3,KAT6A,NTRK3,TADA1,GAB2,DIS3L2,PDCD10,PRAMEF11,CREB5,FERMT2,GLUD1,DNM3,PHIP,EPB41,SS18,ZYG11B,PPARA,CD244,ZNF229,FGF7,TENT5D,MGA,EDA,LIMCH1,TNFSF13B,PTH2R,RBM47,EIF2A,HDAC2,TRIM39,TRRAP,SEMA3D,NPSR1,RRM1,CADM1,INPP5D,CLYBL,ATF6,PRKAR1B,SERPINE1,BACH1,PRAMEF4,TM7SF3,SLC26A5,AKAP13,PKM,GCG,LINC02218,STAC,NNT,ST8SIA2,IRF8,HIVEP3,RFX4,RIPOR2,KDM3A,CASK,HSPG2,GCNT1
GO:0009887	animal organ morphogenesis	0.014188094879174412	PBX4,MMP16,DNAH11,FANCC,EIF4A3,NTN4,SERP1,FLI1,ITGA2,WNT16,CEP290,EGFR,PTPRQ,FAT1,PAX5,CRB1,SMAD6,MEF2C,RYK,PARVA,OLFM1,OTX1,ILK,LEF1,EYA1,MAPK14,MYBPC3,TBC1D20,TMEM100,VDR,RTN4,BBS2,MYO3A,STIL,WNT2B,FBXW11,CRKL,FRAS1,BAK1,PPP3CA,NKD1,RBPJ,PDE6C,ADAMTS16,TAF10,ASH1L,RSPO2,PROX1,FMN1,BCL11B,RPGRIP1L,MYLK,LRP2,TMEM59L,APC,PPARGC1B,COL27A1,NSD2,NRP2,NR3C1,DLX6,MTOR,ANKRD24,GLI3,BCOR,RXFP1,ADGRG6,PPARA,FGF7,EDA,HDAC2,FGD1,SERPINE1,FREM1,RIPOR2,XIRP2,GCNT1,USH1C
GO:0040007	growth	0.018374672478579105	SEMA4D,TEAD1,CYP19A1,SERP1,KMT2D,LPAR3,EGFR,PSMD10,GPAM,LRP12,SHTN1,SH3GL2,MEF2C,RYK,MACF1,PTPN12,URI1,OLFM1,MAPK14,SLC1A2,IL17RB,PARP2,RTN4,ARHGEF11,BBS2,CLSTN1,SERPINE2,STIL,IQGAP1,MYCBP2,LATS1,MAP2,CRKL,ATRN,SPOCK1,TENM4,PPP3CA,NKD1,RBPJ,TAF10,MBD5,RSPO2,DISC1,PR

			OX1,FMN1,ITGB1,STK3,ARHGAP32,IP6K2,WWC3,TMEM108,ADAM17,SEMA4F,HDGFL2,INO80,CDA,COL27A1,ESR2,JADE3,MAPKAP1,NRP2,FSTL4,MTOR,GH2,GNG4,GLI3,PPARA,FGF7,COL14A1,JARID2,SEMA3D,ST8SIA2
GO:0048667	cell morphogenesis involved in neuron differentiation	0.020754896771636402	*XK,SEMA4D,SDC2,PREX2,TANC2,IL1RAPL1,KIF5C,ADCY1,ZDHHC17,LPAR3,PTPRQ,NEO1,SHTN1,DOCK7,MEF2C,RYK,MACF1,PTPRZ1,NEDD4,OLFM1,EFNB3,RTN4,ANOS1,MYCBP2,MAP2,ANKRD27,ANK3,RELN,PPP3CA,DISC1,ITGB1,NEFL,BCL11B,ARHGAP32,UNC5C,CFL1,PLEKHG4B,ADAM17,SEMA4F,TRAK1,STXBP1,CTNND2,NRP2,FSTL4,SNAP25,ANKRD24,GLI3,DNM3,NFASC,SEMA3D,RIPOR2*
GO:0050794	regulation of cellular process	0.021952003767518376	*KLHL13,ABCB7,PBX4,NHLRC3,MYO9B,COL4A5,ERG,PARN,STK16,XK,SEMA4D,MET,MAX,TEAD1,EIF4G3,SIAH3,DUS3L,RASA4B,GAS2L1,CHMP1B,ELAPOR2,ZNF534,TOX3,DNAH11,TACR1,SNX25,SDC2,CIITA,ARAP1,ANKLE2,CEP85,RPRD1B,AHNAK,ACTR5,EIF4A3,DLEC1,NFE2L2,SCHIP1,TFEC,NFYC,CYP19A1,SLC46A2,GPR158,VRK2,ITSN1,PREX2,SCRN1,ELAVL2,TANC2,CSMD3,SERP1,POLR1A,RAB11FIP4,HSFX4,SNTA1,ASZ1,NUB1,SRGAP2C,WIF1,FLI1,ESRRB,ARHGAP26,ZFHX2,LINC-PINT,TRHDE,SETBP1,ZBTB7C,ITGA2,IL1RAPL1,BLK,GPM6A,WNT16,SETD3,ADCY1,GRID1,RPS6KA2,PLPPR5,TRIM24,ZDHHC17,FIG4,FOXP2,KMT2D,LPAR3,TXLNG,SP110,SH3BP2,SH3TC2,CEP290,TACC1,OCLN,FASTKD5,NETO1,EGFR,GBE1,SNX32,PCGEM1,NEO1,MYT1L,PLEKHB2,TCERG1,F10,CABIN1,PSMD10,FBXL17,EOLA1,DHX29,GPAM,IRF2,LRP12,ESRP1,HSFX3,BRMS1L,RGS12,CBX3,SLC39A10,ZNF496,ZNF550,MPHOSPH9,RNF10,FAT1,SLC2A2,PIK3R1,IL17B,NOVA1,SARNP,SHTN1,ATG12,CD200R1,SH3GL2,CRTC1,PITPNC1,DOCK7,PDK3,ROR1,PAX5,AAK1,UTP15,ARID1A,THEMIS,SMAD6,RYBP,NYAP2,PSD3,ABCA13,ZNF519,MEF2C,FZR1,DBNL,ELP4,RYK,MACF1,PXT1,WEE2,TFDP2,PTPRE,SERINC3,ZNF347,PTPN12,PTPRZ1,PLN,CTDSPL2,NEDD4,NEK4,FLYWCH1,LRRC38,CYSLTR2,SECISBP2,URI1,SCML2,CHD6,ZNF609,PRDX1,AEBP2,DSC2,OLFM1,MID1,TSHZ2,POGK,SLC12A2,EFNB3,OTX1,TRIM37,DLG3,PVRIG,FLOT2,SV2B,ILK,LEF1,EYA1,MAPK14,ZNF816-ZNF321P,MYBPC3,ZFHX4,RAPGEF1,GPRC6A,ZNF516,SLC1A2,ARHGEF3,GRIN2B,IFNGR2,DENND4A,TBC1D20,OXR1,SFMBT2,SCAMP5,IL17RB,PARP2,FCHSD2,DNAJB6,MTUS1,LEMD3,PIEZO1,OR2T11,NEB,CSRNP3,MRGPRX1,TMEM100,VDR,NEK11,CREB3L2,RTN4,GNL3L,CACNA1D,PRKAA2,ARHGEF11,IVNS1ABP,SGPP2,DPT,BBS2,CLSTN1,ZNF155,SERGEF,SCYL2,SKAP1,CHM,SKIC3,MYO3A,SERPINE2,IL12RB2,PIK3CG,STIL,ANKFN1,ATP6V1E2,IQGAP1,MYCBP2,WIPI1,LATS1,ZSCAN25,KLHL12,LOC349160,SNX30,FANCI,OR9Q1,WNT2B,TNFRSF10B,MAP2,FBXW11,MTCL1,HSPH1,AZIN2,SMG6,HLA-DMA,PEMT,ARHGAP6,CHD5,JAK1,ITGA9,RADIL,KCNB1,HSD17B12,GRM4,ANKRD27,AIM2,ANK3,ZNF19,KITLG,ARID4B,GMDS,PLEKHM2,NR1I2,ZNF266,RFX7,RELN,CRKL,TENT5A,ARHGEF10,SNAP91,ZNF816,VAV3,RPS6KC1,CD40LG,VRK*

			1,GPAT3,DPYSL3,ZNF420,SPRING1,CCDC88A,SLC35B4,ARNT2,SPOCK1,HPSE2,PLCE1,TNIP3,DCAF5,GNAL,STK32C,MSH3,BAK1,LAX1,STK26,TENM4,PPP3CA,CHRNA5,MKNK1,DYSF,NKD1,RBPJ,RIN2,PDE6C,PLEKHG7,DLX6-AS1,ASCC1,DENND1A,ENSG00000290317,ADAMTS16,TASP1,MICU1,TAF10,MBD5,BACE2,KDM5A,ZNF549,CNOT6,PKIB,ASAH2,STON1,ASH1L,RSPO2,ZNF805,OR5K1,OR13C9,DISC1,PROX1,FMN1,ZNF891,RALGPS1,ITGB1,ZNF827,CORO1C,SPIDR,NEFL,STK3,CHN2,FCAR,HNF4G,MSR1,EXOSC3,SH2D3C,RAMP3,GNG2,ZNF705A,FRS3,LIMS1,ENPP2,DOCK4,ACKR2,MCTP2,BCL11B,GLCE,KCNIP1,PWWP2A,LOC105373170,TBC1D10A,SSBP2,RPH3A,CTTNBP2,CYTH4,NAP1L1,PLPP4,UBR1,CNN2,COL4A2,ARHGAP32,DYRK3,IP6K2,CHRNA6,HDAC4,FYB1,RPGRIP1L,MYLK,UNC5C,CFAP69,TLR7,P3H2,CFL1,SLAIN2,HMG20A,PLEKHG4B,RBBP8,MED13,RNASEH2B,PHF2,TAAR2,AFDN,LRP2,CYTIP,MTDH,ZDHHC13,WWC3,CAMK4,VWC2,RPA3,TMEM108,SEZ6L,SLC39A8,ATXN1,ADAM17,SEMA4F,ELAVL1,TRAK1,STXBP1,OR6N1,IDH1,LMOD2,HDGFL2,PLEKHG1,NHLH1,ELP2,CYP8B1,APC,INO80,PEAR1,CDA,PPARGC1B,ESR2,ERC1,ZNF440,RUBCNL,AGO3,PVR,ACER1,IQGAP2,CDYL,RLF,ANKRA2,TAF5L,CTNND2,RASSF3,PACSIN2,DPYSL2,GIMAP8,RAB8B,METTL8,NSD2,GRM8,AARS1,JADE3,CARD18,PSMC2,MAPKAP1,EVC2,GON4L,MGAT5,NUP153,EFHD1,PIGR,NRP2,SIPA1L2,CAVIN1,FOXJ2,NR3C1,DLX6,BORCS5,FSTL4,ZC3HAV1,MTOR,KSR1,FAF1,SNAP25,DEFB124,CHST4,OR11H6,ZNF221,PIP5K1A,GH2,ASCC2,SPRED1,GNG4,CNTNAP4,PDS5B,CRIM1,RBM46,RNF115,PER2,GLI3,KAT6A,BCOR,NTRK3,RXFP1,TADA1,GAB2,DIS3L2,PDCD10,PDE1A,PRAMEF11,HHAT,CREB5,CCDC68,ADGRG6,FERMT2,GPR82,GLUD1,TSPOAP1,DNM3,PHIP,EPB41,DENND5A,SS18,SFRP5,DOCK9,ZYG11B,PPARA,CD244,SHISA6,HDX,CPEB2,ZNF229,PHF6,HMGB4,FGF7,TENT5D,STXBP6,MGA,CACNA1A,EDA,LIMCH1,TNFSF13B,PTH2R,COL14A1,RBM47,EIF2A,JARID2,FIRRM,HDAC2,FGD1,TRIM39,INSYN2B,TRRAP,SEMA3D,NPSR1,EIF4E3,KCNH5,RRM1,MAGEA1,CADM1,INPP5D,CLYBL,OR4C6,ATF6,PRKAR1B,GBF1,SERPINE1,CCDC88C,BACH1,CUL9,PRAMEF4,EWSR1,TM7SF3,SLC26A5,AKAP13,PKM,GCG,TKFC,LINC02218,PTPN3,STAC,NNT,SSX2IP,ST8SIA2,ELP1,IRF8,HIVEP3,RFX4,OR6K6,ZNF30,REM1,RIPOR2,FUNDC2,KDM3A,CAST,ADGRA3,GPR39,CASK,HSPG2,GCNT1,USH1C,DNAJC13,PSME3IP1
GO:0051179	localization	0.027317909373898823	*SLC18A1,ABCB7,PARN,XK,MET,SCYL3,SNX16,SIAH3,CETN3,GAS2L1,CHMP1B,DNAH11,SLC9A9,EXOC2,TACR1,SNX25,HEPHL1,AHNAK,EIF4A3,NFE2L2,CYP19A1,SLC46A2,GPR158,ITSN1,ATP2B2,SCRN1,TANC2,SERP1,POLR1A,TPTE2,RAB11FIP4,SNTA1,CLIC5,RBP7,RAB44,SLC16A11,ITGA2,IL1RAPL1,KIF5C,BLK,GPM6A,GRID1,EFR3B,ZDHHC17,AP1S3,ANO4,SH3TC2,CEP290,SH3PXD2B,OCLN,NETO1,EGFR,TNPO1,SNX32,NEO1,STAG1,TIMM23,PSMD10,SLC9A4,LRP12,ACAP2,SLC39A10,SLC2A2,PIK3R1,SARNP,TMEM232,SH3GL2,CLBA1,FHIP1A,PITPNC1,SL*

			C30A3,DOCK7,XKR4,AAK1,CRB1,ABCA13,MEF2C,DBNL,MACF1,SLC15A5,MFSD6,SERINC3,PLN,CTDSPL2,SLC16A7,NEDD4,CACNA1E,LRRC38,SLC9C1,SNAP25-AS1,PACS2,ATG4B,SLC7A14,MID1,SLC12A2,TRIM37,DLG3,FLOT2,SRP68,SV2B,LEF1,MAPK14,MYBPC3,RAPGEF1,SLC1A2,GABRB2,GRIN2B,SVOP,TBC1D20,SNX2,SCAMP5,FCHSD2,SLC4A7,DNAJB6,SPCS1,PLEKHF2,PIEZO1,NUP210,VDR,CREB3L2,RTN4,GNL3L,CACNA1D,PRKAA2,CD163,BBS2,CLSTN1,TMEM150C,SERGEF,SCYL2,SKAP1,CHM,SERPINE2,PLAAT3,PIK3CG,TPP1,STIL,ANKFN1,ATP6V1E2,NUP160,MYCBP2,WIPI1,LATS1,KLHL12,SNX30,SPNS3,MAP2,FBXW11,MTCL1,AZIN2,SMG6,JAK1,KCNB1,GRM4,ANKRD27,ANK3,PLEKHM2,NR1I2,RELN,CRKL,SYTL3,NDC1,SNAP91,VAV3,VRK1,SCN9A,SLC16A10,CCDC88A,SLC35B4,PLCE1,FRAS1,BAK1,TTC9-DT,PPP3CA,CHRNA5,DYSF,RIN2,DENND1A,MZT1,MICU1,NOMO1,STON1,DISC1,PIEZO2,ITGB1,ZNF827,RHCE,CORO1C,SPIDR,NEFL,STK3,MSR1,GABRG3,TRPC4,RAMP3,LIMS1,ENPP2,ACKR2,MCTP2,KCNIP1,TBC1D10A,REEP1,RPH3A,SLC38A6,VPS29,PLPP4,COPB2,CNN2,SLC1A6,CHRNA6,FYB1,SEM1,MYLK,CFL1,TUSC3,COPG2,AFDN,LRP2,ZDHHC13,REPS1,TMEM108,CC2D2B,SLC39A8,UPRT,ATXN1,ELAVL1,TRAK1,STXBP1,NUP205,SLCO5A1,APC,ERGIC2,PEAR1,STON1-GTF2A1L,ERC1,RUBCNL,PACSIN2,DPYSL2,RAB8B,NSD2,KCND1,EXOC6B,NUP153,TRMT10B,PIGR,CAVIN1,TMEM165,NR3C1,BORCS5,MTOR,FAF1,SNAP25,PIP5K1A,RAB6B,NALF2,ABCB5,PER2,GLI3,KCNK12,GAB2,PDCD10,LMTK2,CCDC68,GOLGA7,FERMT2,GLUD1,TSPOAP1,DNM3,EPB41,DENND5A,PPARA,SHISA6,FGF7,STXBP6,CACNA1A,JARID2,NPSR1,KCNH5,PRKAR1B,GBF1,IPO11,SERPINE1,CCDC88C,TM7SF3,SLC26A5,GCG,ATP12A,PTPN3,STAC,SCN8A,NNT,SSX2IP,TTC17,IRF8,REM1,AGAP1,RIPOR2,EPS15L1,CYB561A3,PARD3B,CASK,HSPG2,SLCO4C1,GGA2,USH1C,DNAJC13
GO:0040008	regulation of growth	0.032442827240334066	*SEMA4D,TEAD1,SERP1,LPAR3,EGFR,PSMD10,GPAM,LRP12,SHTN1,MEF2C,RYK,MACF1,URI1,OLFM1,MAPK14,IL17RB,PARP2,RTN4,ARHGEF11,BBS2,CLSTN1,SERPINE2,MYCBP2,LATS1,MAP2,CRKL,ATRN,SPOCK1,RBPJ,MBD5,DISC1,PROX1,STK3,ARHGAP32,IP6K2,WWC3,ADAM17,SEMA4F,HDGFL2,INO80,CDA,ESR2,JADE3,MAPKAP1,FSTL4,MTOR,GH2,GNG4,PPARA,COL14A1,JARID2,SEMA3D*
GO:0010646	regulation of cell communication	0.03465855442236649	*MYO9B,SEMA4D,MET,RASA4B,ELAPOR2,TACR1,SNX25,ARAP1,EIF4A3,NFE2L2,SCHIP1,CYP19A1,SLC46A2,GPR158,VRK2,ITSN1,PREX2,SERP1,WIF1,ARHGAP26,ITGA2,BLK,WNT16,ADCY1,GRID1,TRIM24,ZDHHC17,KMT2D,LPAR3,SH3TC2,NETO1,EGFR,NEO1,F10,PSMD10,FBXL17,BRMS1L,RGS12,SLC39A10,SLC2A2,PIK3R1,ROR1,AAK1,SMAD6,PSD3,MEF2C,RYK,MACF1,PTPRE,SERINC3,PTPN12,CTDSPL2,NEDD4,URI1,PRDX1,MID1,SLC12A2,EFNB3,PVRIG,FLOT2,SV2B,ILK,LEF1,EYA1,MAPK14,RAPGEF1,ARHGEF3,GRIN2B,DENND4A,LEMD3,TMEM100,VDR,RTN4*

			,PRKAA2,ARHGEF11,IVNS1ABP,CLSTN1,SCYL2,SERPINE2,PIK3CG,IQGAP1,LATS1,TNFRSF10B,FBXW11,ARHGAP6,CHD5,KCNB1,GRM4,AIM2,ANK3,KITLG,ARID4B,RELN,CRKL,ARHGEF10,VAV3,GPAT3,SPRING1,CCDC88A,TNIP3,BAK1,LAX1,PPP3CA,CHRNA5,DYSF,NKD1,RBPJ,DENND1A,MBD5,ASAH2,ASH1L,RSPO2,DISC1,PROX1,RALGPS1,ITGB1,NEFL,STK3,CHN2,RAMP3,LIMS1,MCTP2,GLCE,CYTH4,UBR1,ARHGAP32,DYRK3,CHRNA6,RPGRIP1L,TLR7,PLEKHG4B,RNASEH2B,LRP2,MTDH,ZDHHC13,WWC3,VWC2,TMEM108,SEZ6L,SLC39A8,ADAM17,STXBP1,PLEKHG1,ELP2,APC,ERC1,AGO3,CTNND2,PACSIN2,RAB8B,GRM8,MAPKAP1,MGAT5,SIPA1L2,FSTL4,ZC3HAV1,MTOR,KSR1,FAF1,SNAP25,PIP5K1A,GH2,SPRED1,GNG4,CNTNAP4,CRIM1,RNF115,PER2,GLI3,KAT6A,NTRK3,PDCD10,FERMT2,GLUD1,TSPOAP1,PHIP,SFRP5,PPARA,SHISA6,CACNA1A,EDA,RBM47,EIF2A,HDAC2,FGD1,TRIM39,NPSR1,MAGEA1,CADM1,INPP5D,ATF6,PRKAR1B,GBF1,SERPINE1,CCDC88C,TM7SF3,AKAP13,GCG,TKFC,PTPN3,SSX2IP,RFX4,RIPOR2,CASK
GO:0043491	phosphatidylinositol 3-kinase/protein kinase B signal transduction	0.03801762825476113	SEMA4D,PREX2,WNT16,EGFR,PIK3R1,ROR1,NYAP2,NEDD4,ILK,RAPGEF1,TMEM100,RTN4,SERPINE2,PIK3CG,AIM2,RELN,VAV3,CCDC88A,ITGB1,STK3,RAMP3,LRP2,MTDH,PEAR1,MTOR,PIP5K1A,NTRK3,GAB2,FERMT2,SFRP5,PPARA
GO:0007010	cytoskeleton organization	0.04037719767272316	MET,CETN3,GAS2L1,CHMP1B,CDC42BPA,TACR1,ARAP1,CEP85,ESPNL,SRGAP2C,ARHGAP26,KRT86,SETD3,CFAP47,TACC1,OCLN,CFAP91,STAG1,KIZ,FAT1,PIK3R1,SHTN1,CALD1,DOCK7,DBNL,MACF1,TUBGCP3,EML1,PARVA,MID1,TRIM37,FCHSD2,DNAJB6,NEB,PRKAA2,ARHGEF11,BBS2,MYO3A,STIL,ANKFN1,IQGAP1,MYCBP2,LATS1,MAP2,FBXW11,MTCL1,ARHGAP6,ANK3,ARHGEF10,DPYSL3,CCDC88A,TACC2,MZT1,DISC1,PROX1,FMN1,CCDC6,ITGB1,DRC7,CORO1C,NEFL,ARPC1A,PLS3,CCDC102B,CNN2,TLN2,CFAP69,CFL1,SLAIN2,LMOD2,APC,TTLL5,INO80,PPARGC1B,IQGAP2,PACSIN2,DPYSL2,PHACTR3,MAPKAP1,MTOR,PIP5K1A,TUBA3D,CCDC68,FERMT2,PHIP,EPB41,SS18,NCKAP5,FGF7,RTTN,LIMCH1,FGD1,CCDC88C,CUL9,AKAP13,SSX2IP,TTC17,HEPACAM2,RIPOR2,PARD3B,XIRP2,USH1C,GOLGA8B
GO:0048468	cell development	0.044042063619344395	PBX4,XK,SEMA4D,MET,SCYL3,EIF4G3,SDC2,FANCC,NFE2L2,SLC46A2,PREX2,TANC2,CSMD3,NTN4,SRGAP2C,FLI1,IL1RAPL1,KIF5C,GPM6A,SPATA16,ADCY1,RPS6KA2,PLPPR5,ZDHHC17,FIG4,CFAP47,KMT2D,LPAR3,SH3TC2,CEP290,EGFR,PTPRQ,NEO1,MYT1L,SLC9A4,LRP12,RNF10,FAT1,PIK3R1,SHTN1,SH3GL2,DOCK7,ROR1,PAX5,CRB1,ARID1A,THEMIS,NYAP2,MEF2C,DBNL,RYK,MACF1,WEE2,PTPRZ1,PLN,EML1,NEDD4,SECISBP2,OLFM1,EFNB3,ILK,LEF1,MAPK14,RAPGEF1,GABRB2,TBC1D20,PARP2,SLC4A7,NEB,RTN4,BBS2,SCYL2,SERPINE2,ANOS1,IQGAP1,MYCBP2,KLHL12,WNT2B,MAP2,FBXW11,CHD5,JAK1,RADIL,KCNB1,ANKRD27,ANK3,KITLG

			,ARID4B,RELN,CRKL,ARHGEF10,CD40LG,DPYSL3,CCDC88A,SPOCK1,BAK1,TENM4,PPP3CA,RBPJ,PDE6C,FRYL,DISC1,PROX1,VSIG1,ITGB1,DRC7,CORO1C,NEFL,STK3,C1GALT1,BCL11B,TSPAN2,NAP1L1,CNN2,ARHGAP32,DYRK3,HDAC4,RPGRIP1L,UNC5C,CFAP69,CFL1,WDR7,PLEKHG4B,AFDN,LRP2,CAMK4,TMEM108,ADAM17,SEMA4F,TRAK1,STXBP1,LMOD2,PPARGC1B,COL27A1,CDYL,CTNND2,DPYSL2,HSD17B4,EFHD1,NRP2,NR3C1,FSTL4,MTOR,SNAP25,RAB6B,MEI4,RBM46,PER2,ANKRD24,GLI3,KAT6A,NTRK3,GAB2,ADGRG6,DNM3,DENND5A,PPARA,NFASC,TNFSF13B,COL14A1,RBM47,HDAC2,TPO,SEMA3D,INPP5D,CCDC88C,AKAP13,ST8SIA2,IRF8,RIPOR2,KDM3A,USH1C
GO:0007417	central nervous system development	0.047877834138521855	*PBX4,SCYL3,FANCC,ANKLE2,GPR158,SRGAP2C,FOXP2,CEP290,TACC1,EGFR,BTD,SH3GL2,DOCK7,ROR1,PAX5,RYK,PTPRZ1,EML1,SECISBP2,OTX1,LEF1,SLC1A2,GRIN2B,ZSWIM6,RTN4,BBS2,SCYL2,SERPINE2,TPP1,STIL,MYCBP2,WNT2B,MAP2,FBXW11,CHD5,GNPAT,AIM2,RELN,CRKL,ATRN,ARNT2,SPOCK1,TACC2,TENM4,RBPJ,DLX6-AS1,DISC1,PROX1,ITGB1,CORO1C,NEFL,NAV2,STK3,TRPC4,BCL11B,TSPAN2,ARHGAP32,RPGRIP1L,UNC5C,LRP2,TMEM108,ATXN1,NHLH1,AARS1,MAPKAP1,NRP2,NR3C1,MTOR,GLI3,GLUD1,NFASC,JARID2,HDAC2,RRM1,RFX4,HSPG2*
GO:0006810	transport	0.04937643785279441	*SLC18A1,ABCB7,XK,MET,SNX16,CETN3,CHMP1B,DNAH11,SLC9A9,EXOC2,TACR1,SNX25,HEPHL1,AHNAK,EIF4A3,NFE2L2,CYP19A1,SLC46A2,GPR158,ITSN1,ATP2B2,SCRN1,TANC2,SERP1,TPTE2,RAB11FIP4,SNTA1,CLIC5,RBP7,RAB44,SLC16A11,ITGA2,IL1RAPL1,KIF5C,BLK,GPM6A,GRID1,ZDHHC17,AP1S3,ANO4,SH3TC2,CEP290,OCLN,NETO1,EGFR,TNPO1,SNX32,NEO1,TIMM23,SLC9A4,LRP12,ACAP2,SLC39A10,SLC2A2,PIK3R1,SARNP,SH3GL2,CLBA1,PITPNC1,SLC30A3,DOCK7,XKR4,AAK1,ABCA13,MEF2C,DBNL,MACF1,SLC15A5,MFSD6,SERINC3,PLN,CTDSPL2,SLC16A7,NEDD4,CACNA1E,LRRC38,SLC9C1,ATG4B,SLC7A14,SLC12A2,TRIM37,FLOT2,SV2B,MAPK14,MYBPC3,RAPGEF1,SLC1A2,GABRB2,GRIN2B,SVOP,TBC1D20,SNX2,SCAMP5,FCHSD2,SLC4A7,PLEKHF2,PIEZO1,NUP210,VDR,CREB3L2,CACNA1D,CD163,BBS2,CLSTN1,TMEM150C,SERGEF,SCYL2,CHM,SERPINE2,PIK3CG,ATP6V1E2,NUP160,WIPI1,KLHL12,SNX30,SPNS3,MAP2,FBXW11,AZIN2,SMG6,KCNB1,GRM4,ANKRD27,ANK3,NR1I2,RELN,SYTL3,NDC1,SNAP91,VAV3,SCN9A,SLC16A10,CCDC88A,SLC35B4,PLCE1,FRAS1,BAK1,PPP3CA,CHRNA5,DYSF,RIN2,DENND1A,MICU1,STON1,PIEZO2,ITGB1,RHCE,CORO1C,NEFL,STK3,MSR1,GABRG3,TRPC4,RAMP3,ENPP2,ACKR2,MCTP2,KCNIP1,TBC1D10A,RPH3A,SLC38A6,VPS29,PLPP4,COPB2,CNN2,SLC1A6,CHRNA6,SEM1,MYLK,TUSC3,COPG2,LRP2,ZDHHC13,REPS1,TMEM108,SLC39A8,UPRT,ATXN1,ELAVL1,TRAK1,STXBP1,NUP205,SLCO5A1,ERGIC2,PEAR1,STON1-GTF2A1L,ERC1,RUBCNL,PACSIN2,DPYSL2,RAB8B,KCND1,EXOC6B,NUP153,TRMT10B,PIGR,CAVIN1,TMEM165,BORCS5,SNAP25,PIP5K1A,RAB6B,NALF2,ABCB5,PER2,GLI3,KCNK12,GAB2,P*

			DCD10,LMTK2,GOLGA7,GLUD1,TSPOAP1,DNM3,EPB41,DENND5A,PPARA,FGF7,STXBP6,CACNA1A,NPSR1,KCNH5,PRKAR1B,GBF1,IPO11,SERPINE1,CCDC88C,TM7SF3,SLC26A5,GCG,ATP12A,PTPN3,STAC,SCN8A,NNT,SSX2IP,TTC17,IRF8,REM1,AGAP1,EPS15L1,CYB561A3,CASK,HSPG2,SLCO4C1,GGA2,DNAJC13
		CC	
GO:0005737	cytoplasm	3.6129804408450525e-19	*SLC18A1,KLHL13,ABCB7,ACOT12,NHLRC3,MYO9B,COL4A5,ERG,PARN,STK16,XK,TNS3,SCYL3,EIF4G3,ACSM3,SNX16,IFTAP,SIAH3,CETN3,RASA4B,GAS2L1,CHMP1B,MMP16,FAXC,PRORP,TOX3,DNAH11,SLC9A9,CDC42BPA,POLG,EXOC2,SNX25,SDC2,ABHD12,COX10,CIITA,FANCC,ARAP1,UQCRHL,ANKLE2,CEP85,CIP2A,AHNAK,ACTR5,EIF4A3,DLEC1,NFE2L2,SCHIP1,TFEC,BLOC1S5-TXNDC5,H2BC18,UPP2,CYP19A1,SLC46A2,DTNB,VRK2,ITSN1,ATP2B2,PREX2,ESPNL,SCRN1,FBF1,SERP1,PLEKHM3,ENKUR,TPTE2,RAB11FIP4,SNTA1,PPCDC,ASZ1,NUB1,MISFA,FLI1,SPATS2L,CLIC5,ESRRB,ARHGAP26,RBP7,TRHDE,RAB44,PTPN4,SETBP1,ITGA2,IL1RAPL1,KIF5C,BLK,SPATA16,WNT16,KRT86,SETD3,ACOX3,ADCY1,RPS6KA2,EFR3B,TRIM24,ZDHHC17,HLCS,FIG4,AP1S3,CFAP47,LPAR3,GIPC2,TXLNG,SH3TC2,CEP290,TACC1,SH3PXD2B,OCLN,CFAP91,FASTKD5,NETO1,EGFR,GBE1,TNPO1,SNX32,DNPEP,NEO1,GLT8D1,STAG1,CLDN14,TIMM23,PLEKHB2,CAPN11,F10,FAM184A,CABIN1,PSMD10,FBXL17,PDZD2,SLC9A4,DHX29,GPAM,IRF2,UBE2A,RGS12,ACOXL,ACAP2,CHCHD6,MPHOSPH9,KIZ,RNF10,FAT1,SLC2A2,PIK3R1,AGMO,NOVA1,SARNP,SHTN1,ATG12,MASP1,BTD,CALD1,SH3GL2,CLBA1,HIBADH,CRTC1,PITPNC1,SLC30A3,PDK3,FBXL4,ROR1,ADAMTSL1,AAK1,UTP15,THEMIS,SMAD6,RYBP,ABCA13,MEF2C,FZR1,ST8SIA5,DBNL,ELP4,RYK,MACF1,PXT1,DENND11,TUBGCP3,WEE2,PTPRE,SERINC3,TBC1D31,PTPN12,ACACA,PLN,EML1,SLC16A7,NEDD4,NEK4,FLYWCH1,VWA8,PARVA,SECISBP2,URI1,ANKRD28,SNAP25-AS1,PRDX1,NDUFAF6,PACS2,ATG4B,SLC7A14,DSC2,GLB1L,SERPINA1,OLFM1,MID1,SLC12A2,TRIM37,DLG3,FLOT2,SRP68,SV2B,ILK,LEF1,EYA1,MAPK14,MYBPC3,RAPGEF1,ALG13,EIF2S3B,ARHGEF3,GABRB2,GRIN2B,UBXN10,SVOP,IFNGR2,DENND4A,TBC1D20,OXR1,SFMBT2,SNX2,GRHPR,SCAMP5,MYL1,KLHL4,EIF3F,RDH16,FCHSD2,DNAJB6,SPCS1,MTUS1,PLEKHF2,B3GLCT,PIEZO1,NEB,TMEM100,NUP210,PDIA5,VDR,CREB3L2,RTN4,GNL3L,CACNA1D,PRKAA2,ARHGEF11,IVNS1ABP,SGPP2,CD163,BBS2,CLSTN1,TMEM150C,SERGEF,SCYL2,AOPEP,SKAP1,CHM,SKIC3,MRPL3,MYO3A,SERPINE2,NUBPL,PLAAT3,PIK3CG,COL4A4,TPP1,STIL,ATP6V1E2,NBPF10,NUP160,IQGAP1,MYCBP2,WIPI1,LATS1,PHYHIPL,KLHL12,SNX30,FANCI,THNSL1,ACAA1,PRSS23,MAP2,FBXW11,MTCL1,HSPH1,AZIN2,IYD,SMG6,HLA-DMA,PEMT,ARHGAP6,PPP2R2B,CHD5,JAK1,GPD2,KCNB1,GNPAT,HSD17B12,HS6ST3,GRM4,ANKRD27,AIM2,ANK3,AMT,KITLG,ARID4B,GMDS,PLEKHM2,RELN,PDHB,CRKL,TENT5A,ARHGEF10,*

			SYTL3,NDC1,SULT1C4,SNAP91,CDKAL1,VAV3,RPS6KC1,CD40LG,VRK1,PAPOLB,URM1,GPAT3,DPYSL3,SPRING1,ATRN,CCDC88A,NDUFB9,SLC35B4,UGT3A2,ARNT2,SPOCK1,PLCE1,TNIP3,TACC2,CYP39A1,DCAF5,BAK1,MRPL45,LAX1,STK26,ENOX2,TENM4,TTC9-DT,PPP3CA,CES2,MKNK1,DYSF,NKD1,RBPJ,RIN2,DENND1A,MZT1,PDZRN3,TASP1,NBPF19,MICU1,TAF10,BACE2,NOMO1,CNOT6,PKIB,ASAH2,FRYL,STON1,PXMP2,ASH1L,ACSF2,DISC1,PROX1,MAP3K7CL,FMN1,RALGPS1,CCDC6,ITGB1,CYB561,DRC7,ZNF827,DSE,CORO1C,C17ORF80,NEFL,STK3,CHN2,RNF150,FCAR,C1GALT1,HNF4G,MSR1,EXOSC3,SH2D3C,APTX,TRPC4,RAMP3,FRS3,CPB1,LIMS1,DOCK4,ACKR2,ARPC1A,MCTP2,CERS3,GLCE,PLS3,PCSK2,KCNIP1,TBC1D10A,SSBP2,REEP1,RPH3A,CTTNBP2,NME9,CYTH4,NAP1L1,VPS29,CCDC102B,COPB2,UBR1,CNN2,COL4A2,SLC1A6,ARHGAP32,DYRK3,IP6K2,TLN2,HDAC4,FYB1,RBM45,SEM1,GYS2,LVRN,RPGRIP1L,MYLK,CFAP69,TLR7,FLG2,P3H2,CFL1,DTYMK,WDR7,SLAIN2,CEP85L,TUSC3,COPG2,PLEKHG4B,AFDN,LRP2,CYTIP,MTDH,LIG1,ZDHHC13,WWC3,CAMK4,SERPINA9,GALNT14,CLPX,REPS1,FBXL7,TMEM108,SEZ6L,PCYOX1L,SLC39A8,AFG2B,UPRT,ATXN1,TMEM59L,ADAM17,SEMA4F,ELAVL1,TRAK1,STXBP1,IDH1,LMOD2,TSNAX-DISC1,HDGFL2,MUC13,NUP205,PLEKHG1,ELP2,SNTB1,ORC4,CYP8B1,APC,TTLL5,INO80,NAA11,MOCS2,ERGIC2,CDA,PPARGC1B,STON1-GTF2A1L,COL27A1,ESR2,LNX1,ERC1,RUBCNL,AGO3,PVR,CKAP4,RPL39L,ACER1,IQGAP2,CDYL,ANKRA2,TAF5L,JPT1,CTNND2,DNAH14,RASSF3,UGGT1,PACSIN2,DPYSL2,GIMAP8,RAB8B,METTL8,NSD2,AARS1,PSMC2,MAPKAP1,EXOC6B,EVC2,DNAH6,MGAT5,ACOX1,HAO1,USP24,NUP153,HSD17B4,EFHD1,TRMT10B,COL21A1,RAI14,PIGR,SIPA1L2,CAVIN1,METTL15,TMEM165,CEP162,ELOVL7,NR3C1,BORCS5,FSTL4,ZC3HAV1,MTOR,KSR1,FAF1,SNAP25,SGSM1,CHST4,GOLGA8A,PIP5K1A,GH2,BTBD2,MMACHC,RAB6B,HS6ST2,KYNU,ADAL,TBXAS1,ASCC2,SPRED1,GALC,TUBA3D,PDS5B,RBM46,RNF115,TRIM61,ESD,PER2,GLI3,LHPP,KAT6A,TADA1,GAB2,DIS3L2,SNTB2,PDCD10,PDE1A,PRAMEF11,LMTK2,HHAT,CCDC68,ADGRG6,GOLGA7,FERMT2,GLUD1,TSPOAP1,ST3GAL2,DNM3,EPB41,DENND5A,DOCK9,AGBL3,ZYG11B,NFASC,CPEB2,FGF7,TBCA,STXBP6,RTTN,CACNA1A,EDA,LIMCH1,TNFSF13B,MTMR7,COL14A1,RBM47,EIF2A,JARID2,FIRRM,HDAC2,TGOLN2,FGD1,TRIM39,TRRAP,NPSR1,EIF4E3,USP48,RRM1,MAGEA1,INPP5D,CFAP161,CLYBL,ATF6,PRKAR1B,GBF1,IPO11,SERPINE1,CCDC88C,BACH1,CUL9,PRAMEF4,EWSR1,AKAP13,PKM,GCG,ATP12A,TKFC,PTPN3,STAC,SCN8A,NNT,SSX2IP,ST8SIA2,TTC17,ELP1,IRF8,HIVEP3,HEPACAM2,CCDC198,REM1,AGAP1,RIPOR2,NDUFA10,FUNDC2,EPS15L1,CYB561A3,PARD3B,XIRP2,KDM3A,MZT2A,CAST,CASK,HSPG2,SLCO4C1,GCNT1,BPIFB3,GGA2,USH1C,NLRP14,DNAJC13,FRMD4B,GOLGA8B
GO:0030054	cell junction	2.394152852268186e-10	*SLC18A1,COL4A5,PARN,TNS3,CDC42BPA,AHNAK,EIF4A3,SCHIP1,DTNB,GPR158,ITSN1,ATP2*

			B2,SCRN1,TANC2,FBF1,SNTA1,ARHGAP26,ITGA2,IL1RAPL1,KIF5C,GPM6A,ADCY1,GRID1,RPS6KA2,ZDHHC17,LPAR3,SH3PXD2B,OCLN,NETO1,EGFR,CLDN14,UBOX5,PDZD2,IRF2,RGS12,RNF10,FAT1,SLC2A2,PIK3R1,SH3GL2,SLC30A3,DOCK7,ROR1,AAK1,CRB1,THEMIS,PSD3,MEF2C,DBNL,MACF1,PTPN12,PTPRZ1,SLC16A7,NEDD4,CACNA1E,PARVA,DSC2,OLFM1,EFNB3,DLG3,FLOT2,SRP68,SV2B,ILK,MAPK14,SLC1A2,GABRB2,GRIN2B,SVOP,SCAMP5,EIF3F,FCHSD2,RTN4,CLSTN1,SKAP1,SERPINE2,IQGAP1,JAK1,KCNB1,GRM4,ANK3,CRKL,SNAP91,DPYSL3,SLC16A10,SPOCK1,PPP3CA,CHRNA5,DYSF,ASCC1,CDH10,DENND1A,PDZRN3,STON1,ASH1L,DISC1,FMN1,ITGB1,CORO1C,NEFL,CHN2,GABRG3,TRPC4,LIMS1,MCTP2,RPH3A,CTTNBP2,SLC38A6,CNN2,SLC1A6,ARHGAP32,IP6K2,CHRNA6,TLN2,HDAC4,FYB1,RPGRIP1L,UNC5C,CFL1,AFDN,MTDH,VWC2,TMEM108,ADAM17,SEMA4F,ELAVL1,STXBP1,SNTB1,APC,ERC1,PVR,CTNND2,PACSIN2,RAB8B,KCND1,RAI14,NRP2,NR3C1,BORCS5,SNAP25,PIP5K1A,RAB6B,CNTNAP4,CDH26,CDHR3,TADA1,SNTB2,FERMT2,TSPOAP1,DNM3,EPB41,SHISA6,NFASC,CPEB2,STXBP6,CACNA1A,TNFSF13B,INSYN2B,CADM1,PRKAR1B,CCDC88C,SCN8A,SSX2IP,PARD3B,XIRP2,CASK,HSPG2,USH1C,FRMD4B
GO:0042995	cell projection	1.795007658835178e-9	*SLC18A1,MYO9B,MAX,TNS3,SCYL3,IFTAP,CETN3,DNAH11,CDC42BPA,TACR1,ABHD12,EIF4A3,DLEC1,DTNB,GPR158,ITSN1,ATP2B2,ESPNL,TANC2,FBF1,ENKUR,ITGA2,IL1RAPL1,KIF5C,GPM6A,ZDHHC17,CFAP47,LPAR3,CEP290,SH3PXD2B,CFAP91,NETO1,EGFR,TNPO1,NEO1,PSMD10,RGS12,ACAP2,KIZ,FAT1,SHTN1,TMEM232,SH3GL2,SLC30A3,DOCK7,ROR1,CCDC178,AAK1,CRB1,PSD3,DBNL,MACF1,TBC1D31,PTPN12,NEDD4,NEK4,PARVA,URI1,SLC9C1,OLFM1,SLC12A2,DLG3,FLOT2,ILK,SLC1A2,GABRB2,GRIN2B,UBXN10,SNX2,FCHSD2,SLC4A7,PIEZO1,RTN4,PRKAA2,BBS2,CLSTN1,MYO3A,IQGAP1,MYCBP2,MAP2,FBXW11,AZIN2,KCNB1,ANKRD27,ANK3,KITLG,RELN,DPYSL3,SCN9A,CCDC88A,SPOCK1,PLCE1,TENM4,PPP3CA,CHRNA5,DENND1A,STON1,DISC1,ITGB1,DRC7,CORO1C,NEFL,GABRG3,SH2D3C,DOCK4,ARPC1A,BCL11B,PCSK2,KCNIP1,TBC1D10A,RPH3A,CTTNBP2,NME9,ARHGAP32,CHRNA6,TLN2,IQCE,RPGRIP1L,MYLK,UNC5C,CFAP69,CFL1,COPG2,LRP2,TMEM108,CC2D2B,ADAM17,SEMA4F,TRAK1,STXBP1,APC,TTLL5,PEAR1,ERC1,IQGAP2,CTNND2,DNAH14,PACSIN2,DPYSL2,KCND1,EVC2,DNAH6,NRP2,CEP162,MTOR,KSR1,SNAP25,PIP5K1A,CNTNAP4,CDHR3,ANKRD24,GLI3,NTRK3,LMTK2,CCDC68,FERMT2,TSPOAP1,DNM3,SHISA6,NFASC,CPEB2,RTTN,CACNA1A,FGD1,RRM1,CADM1,CFAP161,PRKAR1B,PKM,SCN8A,SSX2IP,RIPOR2,CASK,USH1C,FRMD4B*
GO:0016020	membrane	6.797675385431983e-9	*SLC18A1,ABCB7,MYO9B,COL4A5,STK16,XK,SEMA4D,MET,SCYL3,SNX16,RASA4B,CHMP1B,MMP16,ELAPOR2,FAXC,SLC9A9,EXOC2,TACR1,SNX25,SDC2,ABHD12,COX10,ARAP1,UQCRHL,HEPHL1,ANKLE2,CIP2A,AHNAK,EIF4A3,NFE2L2,SCHIP1,CYP19A1,SLC46A2,DTNB,GPR158,VRK2,ITSN1,ATP2B2,PREX2,SCRN1,CSMD3,NTN4,SE*

			RP1,PLEKHM3,TPTE2,RAB11FIP4,NPIPB5,SNTA1,MISFA,CLIC5,ARHGAP26,TRHDE,RAB44,PTPN4,SLC16A11,ITGA2,IL1RAPL1,BLK,GPM6A,ACOX3,ADCY1,GRID1,EFR3B,PLPPR5,ZDHHC17,FIG4,AP1S3,CFAP47,LPAR3,ANO4,TXLNG,SH3TC2,CEP290,FAM171B,TACC1,OCLN,CFAP91,NETO1,EGFR,PTPRQ,NEO1,GLT8D1,CLDN14,TIMM23,PLEKHB2,F10,PSMD10,FBXL17,PDZD2,SLC9A4,GPAM,LRP12,RGS12,CBX3,ACAP2,SLC39A10,CHCHD6,MPHOSPH9,KIZ,RNF10,FAT1,SLC2A2,PIK3R1,AGMO,ATG12,FNDC3B,TMEM232,CD200R1,CALD1,SH3GL2,CLBA1,CRTC1,SLC30A3,XKR4,ROR1,AAK1,CRB1,PSD3,ABCA13,FZR1,ST8SIA5,DBNL,RYK,MACF1,TUBGCP3,WEE2,SLC15A5,PTPRE,MFSD6,SERINC3,PTPRZ1,PLN,SLC66A3,SLC16A7,NEDD4,CACNA1E,LRRC38,CYSLTR2,PARVA,SLC9C1,NDUFAF6,ATG4B,SLC7A14,DSC2,SERPINA1,MID1,SLC12A2,EFNB3,TRIM37,DLG3,PVRIG,FLOT2,SV2B,ILK,WDR17,RAPGEF1,ALG13,EFCAB14,GPRC6A,UNC79,SLC1A2,GABRB2,GRIN2B,SVOP,IFNGR2,DENND4A,TBC1D20,PLD5,SNX2,SCAMP5,IL17RB,MINDY3,EIF3F,RDH16,FCHSD2,SLC4A7,DNAJB6,NPIPB3,SPCS1,MTUS1,LEMD3,PLEKHF2,B3GLCT,PIEZO1,OR2T11,MRGPRX1,TMEM100,NUP210,PDIA5,CREB3L2,RTN4,GNL3L,CACNA1D,ARHGEF11,SGPP2,CD163,BBS2,CLSTN1,TMEM150C,SCYL2,SKAP1,MRPL3,NUBPL,PLAAT3,IL12RB2,PIK3CG,ANOS1,COL4A4,TPP1,TRGV5,ATP6V1E2,NBPF10,IQGAP1,MYCBP2,WIPI1,KLHL12,SNX30,FANCI,OR9Q1,ACAA1,LRRC37A3,SPNS3,TNFRSF10B,MTCL1,AZIN2,IYD,HLA-DMA,PEMT,PPP2R2B,CHD5,JAK1,GPD2,ITGA9,KCNB1,GNPAT,HSD17B12,HS6ST3,GRM4,ANKRD27,ANK3,TMEM132B,KITLG,PLEKHM2,RELN,CRKL,SYTL3,NDC1,SNAP91,ZNF816,CDKAL1,VAV3,RPS6KC1,CD40LG,VRK1,GPAT3,SCN9A,SPRING1,SLC16A10,ATRN,CCDC88A,NDUFB9,SLC35B4,UGT3A2,HPSE2,PLCE1,TACC2,CYP39A1,FRAS1,GNAL,MSH3,BAK1,LAX1,STK26,ENOX2,TENM4,PPP3CA,CHRNA5,DYSF,NKD1,PDE6C,CDH10,DENND1A,MICU1,BACE2,NOMO1,ARL6IP6,CNOT6,ASAH2,TBC1D9B,STON1,PXMP2,OR5K1,OR13C9,FMN1,RALGPS1,PIEZO2,VSIG1,ITGB1,CYB561,DSE,RHCE,CORO1C,LHFPL6,C17ORF80,TMEM185B,CHN2,RNF150,FCAR,C1GALT1,SMCO4,MSR1,GABRG3,SH2D3C,TRPC4,RAMP3,GNG2,FRS3,LIMS1,ENPP2,DOCK4,ACKR2,ARPC1A,MCTP2,CERS3,GLCE,PLS3,TSPAN2,PCSK2,KCNIP1,TBC1D10A,REEP1,RPH3A,CYTH4,NAP1L1,SLC38A6,VPS29,PLPP4,COPB2,CNN2,COL4A2,SLC1A6,ARHGAP32,CHRNA6,TLN2,FYB1,IQCE,LVRN,RPGRIP1L,MYLK,UNC5C,CSMD2,TLR7,FLG2,CFL1,TUSC3,COPG2,PLEKHG4B,MED13,TAAR2,AFDN,LRP2,MTDH,ZDHHC13,SERPINA9,GALNT14,CLPX,VWC2,REPS1,TMEM108,SEZ6L,PCYOX1L,SLC39A8,TMEM59L,ADAM17,SEMA4F,ELAVL1,TRAK1,STXBP1,OR6N1,MUC13,NUP205,SLCO5A1,SNTB1,CYP8B1,APC,TTLL5,ERGIC2,PEAR1,ERC1,ENSG00000287694,SMIM13,RUBCNL,AGO3,C9,PVR,CKAP4,ACER1,IQGAP2,CDYL,ANKRA2,CTNND2,RASSF3,PACSIN2,DPYSL2,NUP210L,CNTNAP3C,RAB8B,GRM8,AARS1,KCND1,TMEM267,PSMC2,MAPKAP1,EVC2,MGAT5,ACOX1,NU

			P153,HSD17B4,EFHD1,TRMT10B,PIGR,NRP2,CAVIN1,GYPE,METTL15,TMEM165,ELOVL7,NR3C1,BORCS5,MTOR,TRAV5,KSR1,FAF1,SNAP25,SGSM1,CHST4,OR11H6,GOLGA8A,CLDND1,PIP5K1A,RAB6B,HS6ST2,TBXAS1,SPRED1,GNG4,CNTNAP4,CDH26,CRIM1,NALF2,ABCB5,CYYR1,CDHR3,ANKRD24,KCNK12,NTRK3,RXFP1,GAB2,SNTB2,PDCD10,LMTK2,HHAT,ADGRG6,GOLGA7,FERMT2,GPR82,ST3GAL2,DNM3,EPB41,DENND5A,DOCK9,CD244,SHISA6,IGLV3-1,NFASC,STXBP6,RTTN,CACNA1A,EDA,TNFSF13B,MTMR7,PTH2R,HDAC2,TPO,TGOLN2,SEMA3D,NPSR1,KCNH5,RRM1,MAGEA1,CADM1,INPP5D,CLYBL,OR4C6,ATF6,PRKAR1B,GBF1,SERPINE1,EWSR1,TM7SF3,SLC26A5,AKAP13,GCG,ATP12A,PTPN3,STAC,SCN8A,NNT,ST8SIA2,TTC17,HEPACAM2,CCDC198,OR6K6,REM1,FREM1,RIPOR2,NDUFA10,CD69,FUNDC2,EPS15L1,CYB561A3,PARD3B,KDM3A,CAST,ADGRA3,GPR39,CASK,HSPG2,SLCO4C1,GCNT1,SLC35F4,GGA2,USH1C,DNAJC13,GOLGA8B,TRDV3
GO:0005829	cytosol	1.0876575873925633e-8	*KLHL13,ACOT12,MYO9B,ERG,PARN,STK16,TNS3,SCYL3,EIF4G3,SNX16,IFTAP,RASA4B,CHMP1B,TOX3,CDC42BPA,EXOC2,COX10,CIITA,FANCC,ARAP1,CEP85,CIP2A,AHNAK,EIF4A3,DLEC1,NFE2L2,SCHIP1,TFEC,H2BC18,UPP2,ITSN1,PREX2,FBF1,SERP1,TPTE2,PPCDC,NUB1,FLI1,SPATS2L,ESRRB,ARHGAP26,RBP7,PTPN4,SETBP1,KIF5C,BLK,KRT86,ACOX3,RPS6KA2,EFR3B,TRIM24,HLCS,AP1S3,TXLNG,CEP290,TACC1,GBE1,TNPO1,SNX32,DNPEP,STAG1,CABIN1,PSMD10,FBXL17,PDZD2,DHX29,IRF2,UBE2A,RGS12,CHCHD6,RNF10,FAT1,PIK3R1,ATG12,MASP1,CALD1,SH3GL2,CRTC1,PITPNC1,FBXL4,AAK1,SMAD6,MEF2C,FZR1,DBNL,TUBGCP3,WEE2,PTPN12,ACACA,EML1,SLC16A7,NEDD4,NEK4,FLYWCH1,PARVA,URI1,ANKRD28,PRDX1,ATG4B,SLC7A14,MID1,TRIM37,DLG3,SRP68,ILK,MAPK14,MYBPC3,RAPGEF1,ARHGEF3,DENND4A,SFMBT2,SNX2,GRHPR,MYL1,EIF3F,DNAJB6,NEB,VDR,GNL3L,PRKAA2,ARHGEF11,IVNS1ABP,CD163,BBS2,SERGEF,AOPEP,SKAP1,CHM,SKIC3,SERPINE2,PLAAT3,PIK3CG,STIL,ATP6V1E2,NUP160,IQGAP1,MYCBP2,WIPI1,LATS1,KLHL12,FANCI,ACAA1,MAP2,FBXW11,HSPH1,AZIN2,SMG6,PEMT,ARHGAP6,PPP2R2B,CHD5,JAK1,GNPAT,ANKRD27,AIM2,ANK3,ARID4B,GMDS,CRKL,ARHGEF10,SULT1C4,VAV3,VRK1,URM1,DPYSL3,CCDC88A,PLCE1,TNIP3,TACC2,BAK1,LAX1,STK26,ENOX2,PPP3CA,MKNK1,RIN2,DENND1A,MZT1,TASP1,NOMO1,CNOT6,PXMP2,DISC1,PROX1,MAP3K7CL,CCDC6,NEFL,STK3,CHN2,HNF4G,EXOSC3,SH2D3C,LIMS1,DOCK4,ACKR2,ARPC1A,MCTP2,PLS3,TBC1D10A,RPH3A,CYTH4,VPS29,COPB2,UBR1,ARHGAP32,DYRK3,HDAC4,FYB1,SEM1,GYS2,RPGRIP1L,MYLK,P3H2,CFL1,DTYMK,SLAIN2,COPG2,PLEKHG4B,AFDN,CYTIP,WWC3,CLPX,REPS1,FBXL7,ATXN1,ADAM17,ELAVL1,TRAK1,STXBP1,IDH1,MUC13,NUP205,PLEKHG1,ELP2,ORC4,APC,TTLL5,INO80,NAA11,MOCS2,CDA,PPARGC1B,ERC1,AGO3,RPL39L,IQGAP2,ANKRA2,JPT1,RASSF3,PACSIN2,DPYSL2,GIMAP8,AARS1,PSMC2,MAPKAP1,ACOX1,HAO1,USP24,NUP153,HSD17B4,TRMT10B,COL21A1,RAI14*

			,CAVIN1,CEP162,NR3C1,ZC3HAV1,MTOR,KSR1,FAF1,SNAP25,SGSM1,GOLGA8A,PIP5K1A,BTBD2,MMACHC,RAB6B,KYNU,ADAL,TBXAS1,ASCC2,SPRED1,PDS5B,RNF115,TRIM61,ESD,PER2,GLI3,LHPP,KAT6A,TADA1,GAB2,PDCD10,PDE1A,LMTK2,FERMT2,TSPOAP1,EPB41,DENND5A,DOCK9,AGBL3,MTMR7,EIF2A,FGD1,TRIM39,EIF4E3,USP48,RRM1,INPP5D,ATF6,PRKAR1B,GBF1,IPO11,BACH1,CUL9,AKAP13,PKM,ATP12A,TKFC,PTPN3,STAC,TTC17,ELP1,IRF8,EPS15L1,MZT2A,CAST,CASK,USH1C,DNAJC13,GOLGA8B
GO:0005856	cytoskeleton	1.3692573883054368e-8	MYO9B,TNS3,CETN3,GAS2L1,CHMP1B,DNAH11,CDC42BPA,CEP85,AHNAK,NFE2L2,UPP2,FBF1,SERP1,ENKUR,RAB11FIP4,SNTA1,CLIC5,ARHGAP26,PTPN4,KIF5C,KRT86,RPS6KA2,EFR3B,CFAP47,CEP290,TACC1,SH3PXD2B,CFAP91,STAG1,FAM184A,PSMD10,PDZD2,CBX3,MPHOSPH9,KIZ,SHTN1,TMEM232,CALD1,ROR1,CCDC178,DBNL,MACF1,TUBGCP3,TFDP2,TBC1D31,PTPN12,ACACA,EML1,NEK4,PARVA,MID1,FLOT2,ILK,MAPK14,GRIN2B,MYL1,KLHL4,MTUS1,NEB,IVNS1ABP,BBS2,MYO3A,STIL,ANKFN1,IQGAP1,MYCBP2,WIPI1,LATS1,KLHL12,MAP2,FBXW11,MTCL1,HSPH1,ARHGAP6,PPP2R2B,JAK1,RADIL,ANK3,KITLG,NR1I2,ARHGEF10,NDC1,DPYSL3,CCDC88A,TACC2,DYSF,MZT1,DISC1,FMN1,CCDC6,DRC7,CORO1C,NEFL,STK3,HNF4G,GABRG3,TRPC4,ACKR2,ARPC1A,PLS3,REEP1,CTTNBP2,NME9,CYTH4,CCDC102B,CNN2,SLC1A6,ARHGAP32,DYRK3,TLN2,HDAC4,FYB1,GYS2,RPGRIP1L,MYLK,CFL1,SLAIN2,CEP85L,FBXL7,AFG2B,ADAM17,LMOD2,SNTB1,APC,TTLL5,INO80,NAA11,ERC1,CKAP4,IQGAP2,ANKRA2,JPT1,DNAH14,RASSF3,PACSIN2,DPYSL2,EVC2,DNAH6,RAI14,CEP162,NR3C1,BORCS5,SNAP25,TUBA3D,CDH26,GLI3,SNTB2,CCDC68,FERMT2,DNM3,EPB41,SS18,NCKAP5,TBCA,RTTN,EDA,LIMCH1,FIRRM,FGD1,INPP5D,CFAP161,CCDC88C,AKAP13,ATP12A,PTPN3,SSX2IP,TTC17,HEPACAM2,RIPOR2,XIRP2,MZT2A,CASK,USH1C,FRMD4B
GO:0120025	plasma membrane bounded cell projection	6.721627423230303e-8	SLC18A1,MYO9B,MAX,SCYL3,IFTAP,CETN3,DNAH11,CDC42BPA,TACR1,ABHD12,EIF4A3,DLEC1,DTNB,ITSN1,ATP2B2,ESPNL,TANC2,FBF1,ENKUR,ITGA2,IL1RAPL1,KIF5C,GPM6A,CFAP47,LPAR3,CEP290,CFAP91,NETO1,EGFR,TNPO1,NEO1,PSMD10,RGS12,ACAP2,FAT1,SHTN1,TMEM232,SLC30A3,DOCK7,ROR1,CCDC178,AAK1,CRB1,PSD3,DBNL,MACF1,TBC1D31,NEDD4,NEK4,PARVA,URI1,SLC9C1,OLFM1,SLC12A2,DLG3,FLOT2,ILK,SLC1A2,GABRB2,GRIN2B,UBXN10,SNX2,FCHSD2,SLC4A7,PIEZO1,RTN4,PRKAA2,BBS2,CLSTN1,MYO3A,IQGAP1,MYCBP2,MAP2,FBXW11,AZIN2,KCNB1,ANKRD27,ANK3,KITLG,RELN,DPYSL3,SCN9A,CCDC88A,SPOCK1,PLCE1,TENM4,PPP3CA,CHRNA5,DENND1A,DISC1,ITGB1,DRC7,CORO1C,NEFL,GABRG3,SH2D3C,DOCK4,ARPC1A,BCL11B,PCSK2,KCNIP1,TBC1D10A,RPH3A,CTTNBP2,ARHGAP32,CHRNA6,TLN2,IQCE,RPGRIP1L,MYLK,UNC5C,CFAP69,CFL1,COPG2,LRP2,TMEM108,CC2D2B,ADAM17,SEMA4F,TRAK1,STXBP1,APC,TTLL5,PEAR1,ERC1,IQGAP2,CTNND2,DNAH14,PACSIN2,DPYSL2,KCND1,EVC2,DNAH6,NRP2,CEP162,MTOR,KSR1,SNAP25,PIP5K1A,CDHR3,ANKRD24,GLI3,NTRK3,LMTK2,

			CCDC68,FERMT2,TSPOAP1,DNM3,SHISA6,NFASC,CPEB2,RTTN,FGD1,CADM1,CFAP161,PRKAR1B,PKM,SCN8A,SSX2IP,RIPOR2,CASK,USH1C,FRMD4B
GO:0031252	cell leading edge	1.2390280809304584e-7	*MYO9B,SCYL3,CDC42BPA,ITSN1,ATP2B2,OCLN,EGFR,ACAP2,FAT1,SHTN1,AAK1,PSD3,DBNL,MACF1,PARVA,FLOT2,ILK,SLC1A2,SNX2,PIEZO1,IQGAP1,KCNB1,KITLG,DPYSL3,CCDC88A,PLCE1,STON1,ITGB1,CORO1C,GABRG3,SH2D3C,TLN2,MYLK,UNC5C,CFL1,ADAM17,APC,PEAR1,IQGAP2,PACSIN2,KSR1,SNAP25,PIP5K1A,FERMT2,SHISA6,FGD1,GBF1,SSX2IP,RIPOR2,FRMD4B*
GO:0045202	synapse	0.0000050614008339845 04	*SLC18A1,COL4A5,PARN,EIF4A3,DTNB,GPR158,ITSN1,ATP2B2,SCRN1,TANC2,SNTA1,ITGA2,IL1RAPL1,KIF5C,GPM6A,ADCY1,GRID1,RPS6KA2,ZDHHC17,LPAR3,NETO1,RGS12,RNF10,SH3GL2,SLC30A3,ROR1,AAK1,PSD3,MEF2C,DBNL,PTPRZ1,SLC16A7,NEDD4,CACNA1E,OLFM1,EFNB3,DLG3,FLOT2,SV2B,MAPK14,SLC1A2,GABRB2,GRIN2B,SVOP,SCAMP5,EIF3F,FCHSD2,RTN4,CLSTN1,SERPINE2,KCNB1,GRM4,ANK3,CRKL,SNAP91,DPYSL3,SPOCK1,PPP3CA,CHRNA5,DYSF,ASCC1,CDH10,DENND1A,PDZRN3,STON1,DISC1,ITGB1,CORO1C,NEFL,CHN2,GABRG3,MCTP2,RPH3A,CTTNBP2,SLC38A6,SLC1A6,ARHGAP32,CHRNA6,TLN2,HDAC4,UNC5C,VWC2,TMEM108,SEMA4F,ELAVL1,STXBP1,SNTB1,ERC1,CTNND2,PACSIN2,RAB8B,KCND1,NRP2,NR3C1,BORCS5,SNAP25,RAB6B,CNTNAP4,CDHR3,SNTB2,TSPOAP1,DNM3,SHISA6,CPEB2,CACNA1A,INSYN2B,CADM1,PRKAR1B,SCN8A,CASK,USH1C*
GO:0071944	cell periphery	0.0000077816506861952 79	*MYO9B,COL4A5,STK16,XK,SEMA4D,MET,RASA4B,CHMP1B,MMP16,ELAPOR2,SLC9A9,EXOC2,TACR1,SDC2,ABHD12,ARAP1,HEPHL1,CEP85,CIP2A,AHNAK,NFE2L2,SCHIP1,SLC46A2,DTNB,GPR158,ITSN1,ATP2B2,PREX2,CSMD3,NTN4,PLEKHM3,TPTE2,RAB11FIP4,SNTA1,CLIC5,TRHDE,RAB44,PTPN4,ITGA2,IL1RAPL1,BLK,GPM6A,ADCY1,GRID1,EFR3B,PLPPR5,ZDHHC17,LPAR3,ANO4,SH3TC2,OCLN,NETO1,EGFR,NEO1,CLDN14,F10,PSMD10,PDZD2,SLC9A4,GPAM,LRP12,RGS12,SLC39A10,RNF10,FAT1,SLC2A2,PIK3R1,CD200R1,CALD1,SH3GL2,CRTC1,SLC30A3,XKR4,ROR1,AAK1,CRB1,PSD3,ABCA13,DBNL,RYK,MACF1,WEE2,PTPRE,MFSD6,SERINC3,PTPRZ1,SLC16A7,NEDD4,CACNA1E,LRRC38,CYSLTR2,PARVA,SLC9C1,SLC7A14,DSC2,SERPINA1,SLC12A2,EFNB3,DLG3,PVRIG,FLOT2,SV2B,ILK,RAPGEF1,GPRC6A,UNC79,SLC1A2,GABRB2,GRIN2B,SVOP,IFNGR2,SCAMP5,IL17RB,FCHSD2,SLC4A7,MTUS1,PIEZO1,OR2T11,MRGPRX1,TMEM100,RTN4,CACNA1D,ARHGEF11,CD163,DPT,BBS2,CLSTN1,TMEM150C,SKAP1,SERPINE2,NUBPL,PLAAT3,IL12RB2,PIK3CG,ANOS1,COL4A4,STIL,TRGV5,IQGAP1,MYCBP2,OR9Q1,WNT2B,TNFRSF10B,MTCL1,IYD,HLA-DMA,JAK1,ITGA9,KCNB1,HSD17B12,GRM4,ANKRD27,ANK3,KITLG,THSD4,RELN,ADAMTSL3,CRKL,SYTL3,NDC1,SNAP91,VAV3,CD40LG,SCN9A,SLC16A10,ATRN,CCDC88A,HPSE2,PLCE1,TACC2,FRAS1,GNAL,LAX1,STK26,ENOX2,TENM4,PPP3CA,CHRNA5,DYSF,NKD1,PDE6C,CDH10,DENND1A,ADAMTS16,BACE2,ASAH2,FRYL,STON1,O*

			R5K1,OR13C9,FMN1,RALGPS1,PIEZO2,VSIG1,ITGB1,RHCE,CORO1C,NAV2,FCAR,MSR1,GABRG3,SH2D3C,TRPC4,RAMP3,GNG2,FRS3,LIMS1,ENPP2,DOCK4,ACKR2,ARPC1A,PLS3,TSPAN2,KCNIP1,TBC1D10A,RPH3A,CTTNBP2,CYTH4,SLC38A6,PLPP4,COL4A2,SLC1A6,ARHGAP32,CHRNA6,TLN2,FYB1,GYS2,IQCE,RPGRIP1L,MYLK,UNC5C,CSMD2,TLR7,FLG2,P3H2,CFL1,TUSC3,PLEKHG4B,TAAR2,AFDN,LRP2,CYTIP,MTDH,VWC2,REPS1,SLC39A8,ADAM17,SEMA4F,TRAK1,STXBP1,OR6N1,MUC13,SLCO5A1,SNTB1,APC,TTLL5,PEAR1,COL27A1,ERC1,C9,PVR,CKAP4,IQGAP2,CTNND2,RASSF3,PACSIN2,DPYSL2,RAB8B,GRM8,KCND1,MAPKAP1,EXOC6B,EVC2,COL21A1,RAI14,PIGR,NRP2,CAVIN1,GYPE,BORCS5,TRAV5,KSR1,FAF1,SNAP25,OR11H6,CLDND1,PIP5K1A,SPRED1,GNG4,CNTNAP4,CDH26,CRIM1,NALF2,ABCB5,CDHR3,ANKRD24,NTRK3,RXFP1,GAB2,SNTB2,PDCD10,ADGRG6,FERMT2,GPR82,DNM3,EPB41,CD244,SHISA6,IGLV3-1,NFASC,CACNA1A,EDA,TNFSF13B,PTH2R,COL14A1,TPO,TGOLN2,NPSR1,KCNH5,MAGEA1,CADM1,INPP5D,OR4C6,PRKAR1B,SERPINE1,EWSR1,TM7SF3,SLC26A5,AKAP13,PKM,GCG,ATP12A,PTPN3,STAC,SCN8A,TTC17,CCDC198,OR6K6,REM1,FREM1,RIPOR2,CD69,EPS15L1,PARD3B,ADGRA3,GPR39,CASK,HSPG2,SLCO4C1,USH1C,DNAJC13,TRDV3
GO:0012505	endomembrane system	0.00001182764501651629	SLC18A1,NHLRC3,COL4A5,STK16,XK,SCYL3,SNX16,CETN3,CHMP1B,MMP16,SLC9A9,SNX25,SDC2,ABHD12,ARAP1,ANKLE2,CEP85,NFE2L2,BLOC1S5-TXNDC5,CYP19A1,SLC46A2,VRK2,ITSN1,SCRN1,SERP1,PLEKHM3,ENKUR,TPTE2,RAB11FIP4,CLIC5,ARHGAP26,RAB44,SPATA16,ZDHHC17,FIG4,AP1S3,TXLNG,CEP290,EGFR,SNX32,NEO1,GLT8D1,CLDN14,PLEKHB2,CAPN11,F10,PDZD2,SLC9A4,LRP12,CBX3,ACAP2,MPHOSPH9,PIK3R1,AGMO,SH3GL2,CLBA1,SLC30A3,ADAMTSL1,AAK1,UTP15,SMAD6,ABCA13,FZR1,ST8SIA5,DBNL,MACF1,SERINC3,PLN,NEDD4,PACS2,ATG4B,DSC2,SERPINA1,OLFM1,MID1,FLOT2,SRP68,SV2B,MAPK14,RAPGEF1,ALG13,GRIN2B,UBXN10,SVOP,IFNGR2,DENND4A,TBC1D20,SNX2,SCAMP5,MINDY3,RDH16,FCHSD2,NPIPB3,SPCS1,MTUS1,LEMD3,PLEKHF2,B3GLCT,PIEZO1,TMEM100,NUP210,PDIA5,CREB3L2,RTN4,PRKAA2,SGPP2,CLSTN1,SCYL2,SERPINE2,PLAAT3,COL4A4,TPP1,ATP6V1E2,NUP160,IQGAP1,WIPI1,KLHL12,SNX30,ACAA1,PRSS23,FBXW11,AZIN2,HLA-DMA,PEMT,JAK1,HSD17B12,HS6ST3,ANKRD27,ANK3,PLEKHM2,SYTL3,NDC1,SNAP91,CDKAL1,RPS6KC1,CD40LG,VRK1,GPAT3,DPYSL3,SPRING1,ATRN,CCDC88A,SLC35B4,UGT3A2,PLCE1,CYP39A1,BAK1,LAX1,STK26,CES2,DYSF,BACE2,NOMO1,ARL6IP6,ASAH2,STON1,ASH1L,DISC1,FMN1,ITGB1,CYB561,DSE,CORO1C,FCAR,C1GALT1,DOCK4,ACKR2,MCTP2,CERS3,GLCE,PCSK2,REEP1,RPH3A,CTTNBP2,CYTH4,VPS29,COPB2,CNN2,COL4A2,SLC1A6,ARHGAP32,TLR7,FLG2,P3H2,TUSC3,COPG2,LRP2,CYTIP,MTDH,ZDHHC13,GALNT14,REPS1,TMEM108,SEZ6L,PCYOX1L,TMEM59L,ADAM17,SEMA4F,ELAVL1,TRAK1,

			STXBP1,IDH1,MUC13,NUP205,CYP8B1,APC,NAA11,ERGIC2,CDA,COL27A1,ERC1,CKAP4,ACER1,IQGAP2,UGGT1,PACSIN2,NUP210L,GIMAP8,RAB8B,PSMC2,MAPKAP1,MGAT5,NUP153,COL21A1,PIGR,CAVIN1,TMEM165,ELOVL7,BORCS5,FSTL4,MTOR,KSR1,FAF1,SNAP25,SGSM1,CHST4,GOLGA8A,GH2,RAB6B,HS6ST2,TBXAS1,ESD,SNTB2,PDCD10,LMTK2,HHAT,ADGRG6,GOLGA7,GLUD1,ST3GAL2,DENND5A,DOCK9,NFASC,FGF7,EDA,MTMR7,COL14A1,TGOLN2,FGD1,TRRAP,RRM1,ATF6,PRKAR1B,GBF1,IPO11,SERPINE1,PKM,GCG,ST8SIA2,TTC17,HEPACAM2,EPS15L1,CYB561A3,PARD3B,CAST,HSPG2,SLCO4C1,GCNT1,GGA2,DNAJC13,GOLGA8B
GO:0043005	neuron projection	0.0000210725960022959 38	*SLC18A1,MAX,CETN3,TACR1,ABHD12,EIF4A3,DTNB,ITSN1,ATP2B2,ESPNL,TANC2,ITGA2,IL1RAPL1,KIF5C,GPM6A,LPAR3,CEP290,NETO1,NEO1,PSMD10,RGS12,SHTN1,SLC30A3,DOCK7,ROR1,AAK1,CRB1,DBNL,NEDD4,URI1,OLFM1,SLC12A2,DLG3,FLOT2,SLC1A2,GABRB2,GRIN2B,FCHSD2,SLC4A7,RTN4,PRKAA2,BBS2,CLSTN1,MYO3A,IQGAP1,MYCBP2,MAP2,FBXW11,AZIN2,KCNB1,ANKRD27,ANK3,RELN,DPYSL3,SCN9A,SPOCK1,TENM4,PPP3CA,CHRNA5,DENND1A,DISC1,ITGB1,NEFL,GABRG3,SH2D3C,DOCK4,BCL11B,PCSK2,KCNIP1,RPH3A,CTTNBP2,ARHGAP32,CHRNA6,RPGRIP1L,UNC5C,CFL1,COPG2,LRP2,TMEM108,SEMA4F,TRAK1,STXBP1,CTNND2,DPYSL2,KCND1,NRP2,MTOR,SNAP25,CDHR3,ANKRD24,NTRK3,LMTK2,TSPOAP1,DNM3,SHISA6,NFASC,CPEB2,CADM1,SCN8A,RIPOR2,USH1C*
GO:0098590	plasma membrane region	0.00002354724549095132	*MET,DTNB,GPR158,ITSN1,ATP2B2,RAB11FIP4,SNTA1,CLIC5,IL1RAPL1,GPM6A,ADCY1,GRID1,ZDHHC17,OCLN,NETO1,EGFR,SLC9A4,SLC39A10,RNF10,FAT1,SLC2A2,CRB1,PSD3,MACF1,SLC16A7,NEDD4,SLC12A2,EFNB3,DLG3,FLOT2,SLC1A2,GABRB2,GRIN2B,SVOP,SLC4A7,PIEZO1,BBS2,CLSTN1,SKAP1,IQGAP1,MTCL1,ITGA9,KCNB1,ANK3,CRKL,SNAP91,SLC16A10,STK26,CHRNA5,CDH10,DENND1A,ASAH2,VSIG1,ITGB1,CORO1C,GABRG3,SH2D3C,TRPC4,ARPC1A,RPH3A,SLC1A6,CHRNA6,IQCE,MYLK,CFL1,LRP2,MTDH,SLC39A8,ADAM17,SEMA4F,STXBP1,MUC13,APC,ERC1,PACSIN2,KCND1,EVC2,NRP2,CAVIN1,KSR1,SNAP25,CLDND1,PIP5K1A,SPRED1,CNTNAP4,FERMT2,DNM3,EPB41,SHISA6,CADM1,SLC26A5,ATP12A,SCN8A,RIPOR2,PARD3B,CASK,SLCO4C1*
GO:0030027	lamellipodium	0.00003081910530709897	*MYO9B,SCYL3,CDC42BPA,ITSN1,FAT1,SHTN1,DBNL,PARVA,FLOT2,ILK,SNX2,PIEZO1,KITLG,DPYSL3,CCDC88A,PLCE1,ITGB1,CORO1C,MYLK,UNC5C,CFL1,APC,PEAR1,IQGAP2,SNAP25,PIP5K1A,FERMT2,FGD1*
GO:0015629	actin cytoskeleton	0.00005510855199614563	*MYO9B,TNS3,GAS2L1,CDC42BPA,AHNAK,CLIC5,EFR3B,SH3PXD2B,CALD1,ROR1,DBNL,MACF1,PTPN12,ACACA,PARVA,FLOT2,ILK,MYL1,NEB,MYO3A,IQGAP1,ARHGAP6,NDC1,DPYSL3,FMN1,CORO1C,ACKR2,ARPC1A,PLS3,CTTNBP2,CNN2,ARHGAP32,TLN2,HDAC4,FYB1,GYS2,MYLK,CFL1,ADAM17,LMOD2,IQGAP2,RAI14,SNAP25,FERMT2,LIMCH1,AKAP13,ATP12A,TTC17,XIRP2,CASK*
GO:0005886	plasma	0.000118431	*STK16,XK,SEMA4D,MET,RASA4B,CHMP1B,MMP16,ELAPOR2,SLC9A9,EXOC2,TACR1,SDC2,ABHD*

	membrane	86144918905	12,ARAP1,HEPHL1,CIP2A,AHNAK,NFE2L2,SCHIP1,SLC46A2,DTNB,GPR158,ITSN1,ATP2B2,PREX2,CSMD3,NTN4,PLEKHM3,TPTE2,RAB11FIP4,SNTA1,CLIC5,TRHDE,RAB44,PTPN4,ITGA2,IL1RAPL1,BLK,GPM6A,ADCY1,GRID1,EFR3B,PLPPR5,ZDHHC17,LPAR3,ANO4,SH3TC2,OCLN,NETO1,EGFR,NEO1,CLDN14,F10,PSMD10,PDZD2,SLC9A4,GPAM,LRP12,RGS12,SLC39A10,RNF10,FAT1,SLC2A2,PIK3R1,CD200R1,CALD1,SH3GL2,CRTC1,SLC30A3,XKR4,ROR1,AAK1,CRB1,PSD3,ABCA13,DBNL,RYK,MACF1,WEE2,PTPRE,MFSD6,SERINC3,PTPRZ1,SLC16A7,NEDD4,CACNA1E,LRRC38,CYSLTR2,PARVA,SLC9C1,SLC7A14,DSC2,SLC12A2,EFNB3,DLG3,PVRIG,FLOT2,SV2B,ILK,RAPGEF1,GPRC6A,UNC79,SLC1A2,GABRB2,GRIN2B,SVOP,IFNGR2,SCAMP5,IL17RB,FCHSD2,SLC4A7,MTUS1,PIEZO1,OR2T11,MRGPRX1,TMEM100,RTN4,CACNA1D,ARHGEF11,CD163,BBS2,CLSTN1,TMEM150C,SKAP1,NUBPL,PLAAT3,IL12RB2,PIK3CG,ANOS1,TRGV5,IQGAP1,MYCBP2,OR9Q1,TNFRSF10B,MTCL1,IYD,HLA-DMA,JAK1,ITGA9,KCNB1,GRM4,ANKRD27,ANK3,KITLG,RELN,CRKL,SYTL3,NDC1,SNAP91,VAV3,CD40LG,SCN9A,SLC16A10,ATRN,CCDC88A,HPSE2,PLCE1,TACC2,FRAS1,GNAL,LAX1,STK26,ENOX2,TENM4,PPP3CA,CHRNA5,DYSF,NKD1,PDE6C,CDH10,DENND1A,BACE2,ASAH2,STON1,OR5K1,OR13C9,FMN1,RALGPS1,PIEZO2,VSIG1,ITGB1,RHCE,CORO1C,FCAR,MSR1,GABRG3,SH2D3C,TRPC4,RAMP3,GNG2,FRS3,LIMS1,ENPP2,DOCK4,ACKR2,ARPC1A,PLS3,TSPAN2,KCNIP1,TBC1D10A,RPH3A,CYTH4,SLC38A6,PLPP4,SLC1A6,CHRNA6,TLN2,FYB1,IQCE,RPGRIP1L,MYLK,UNC5C,CSMD2,TLR7,FLG2,CFL1,TUSC3,PLEKHG4B,TAAR2,AFDN,LRP2,MTDH,VWC2,REPS1,SLC39A8,ADAM17,SEMA4F,STXBP1,OR6N1,MUC13,SLCO5A1,SNTB1,APC,TTLL5,PEAR1,ERC1,C9,PVR,CKAP4,IQGAP2,CTNND2,RASSF3,PACSIN2,DPYSL2,RAB8B,GRM8,KCND1,MAPKAP1,EVC2,PIGR,NRP2,CAVIN1,GYPE,BORCS5,TRAV5,KSR1,FAF1,SNAP25,OR11H6,CLDND1,PIP5K1A,SPRED1,GNG4,CNTNAP4,CDH26,CRIM1,NALF2,ABCB5,CDHR3,ANKRD24,NTRK3,RXFP1,GAB2,SNTB2,PDCD10,ADGRG6,FERMT2,GPR82,DNM3,EPB41,CD244,SHISA6,IGLV3-1,NFASC,CACNA1A,EDA,TNFSF13B,PTH2R,TPO,TGOLN2,NPSR1,KCNH5,MAGEA1,CADM1,INPP5D,OR4C6,PRKAR1B,SERPINE1,EWSR1,TM7SF3,SLC26A5,GCG,ATP12A,PTPN3,STAC,SCN8A,TTC17,CCDC198,OR6K6,REM1,RIPOR2,CD69,EPS15L1,PARD3B,ADGRA3,GPR39,CASK,HSPG2,SLCO4C1,USH1C,DNAJC13,TRDV3
GO:0070161	anchoring junction	0.000144650 18393237497	TNS3,CDC42BPA,AHNAK,FBF1,SNTA1,ARHGAP26,ITGA2,SH3PXD2B,OCLN,EGFR,CLDN14,UBOX5,PDZD2,IRF2,FAT1,SLC2A2,PIK3R1,DOCK7,CRB1,THEMIS,DBNL,PTPN12,PARVA,DSC2,DLG3,FLOT2,SRP68,ILK,FCHSD2,RTN4,SKAP1,IQGAP1,JAK1,ANK3,PPP3CA,CDH10,ASH1L,FMN1,ITGB1,CORO1C,TRPC4,LIMS1,CNN2,TLN2,FYB1,RPGRIP1L,CFL1,AFDN,MTDH,ADAM17,SNTB1,APC,PVR,CTNND2,PACSIN2,RAI14,PIP5K1A,CDH26,CDHR3,TADA1,SNTB2,FERMT2,NFASC,STXBP6,TNFSF13B,CADM1,CCDC88C,SSX2IP,P

			ARD3B,XIRP2,CASK,HSPG2,FRMD4B
GO:0097060	synaptic membrane	0.000171620 25957261198	GPR158,ITSN1,ATP2B2,SNTA1,IL1RAPL1,GPM 6A,ADCY1,GRID1,ZDHHC17,NETO1,RNF10,SLC 16A7,EFNB3,DLG3,SLC1A2,GABRB2,GRIN2B,C LSTN1,KCNB1,ANK3,CRKL,SNAP91,CHRNA5,CD H10,DENND1A,ITGB1,GABRG3,RPH3A,SLC1A6, CHRNA6,SEMA4F,STXBP1,ERC1,KCND1,NRP2,S NAP25,CNTNAP4,DNM3,SHISA6,SCN8A,CASK
GO:0110165	cellular anatomica l structure	0.000856828 9883229681	SLC18A1,KLHL13,ABCB7,PBX4,ACOT12,NHLRC 3,MYO9B,COL4A5,ERG,PARN,STK16,XK,SEMA4 D,MET,MAX,TNS3,SCYL3,TEAD1,EIF4G3,ACSM 3,SNX16,IFTAP,SIAH3,CETN3,RASA4B,GAS2L 1,CHMP1B,MMP16,ELAPOR2,FAXC,PRORP,ZNF5 34,TOX3,DNAH11,SLC9A9,CDC42BPA,POLR2J, POLG,EXOC2,TACR1,SNX25,SDC2,ABHD12,COX 10,CIITA,FANCC,ARAP1,UQCRHL,HEPHL1,ANK LE2,CEP85,RPRD1B,CIP2A,AHNAK,ACTR5,EIF 4A3,DLEC1,NFE2L2,SCHIP1,TFEC,NFYC,BLOC 1S5- TXNDC5,H2BC18,UPP2,CYP19A1,SLC46A2,DTN B,GPR158,VRK2,ITSN1,R3HDM2,ATP2B2,PREX 2,ESPNL,SCRN1,TANC2,CSMD3,FBF1,NTN4,SE RP1,POLR1A,PLEKHM3,ENKUR,TPTE2,RAB11FI P4,HSFX4,NPIPB5,SNTA1,PPCDC,ASZ1,NUB1, MISFA,WIF1,FLI1,SPATS2L,CLIC5,ESRRB,AR HGAP26,ZFHX2,RBP7,TRHDE,RAB44,PTPN4,SL C16A11,SETBP1,ZBTB7C,ITGA2,IL1RAPL1,KI F5C,BLK,GPM6A,SPATA16,WNT16,KRT86,SETD 3,ACOX3,ADCY1,GRID1,RPS6KA2,EFR3B,PLPP R5,TRIM24,ZDHHC17,HLCS,FIG4,AP1S3,CFAP 47,FOXP2,KMT2D,LPAR3,ANO4,GIPC2,GFOD1, TXLNG,SP110,SH3TC2,CEP290,FAM171B,TACC 1,SH3PXD2B,OCLN,CFAP91,FASTKD5,NETO1,E GFR,PTPRQ,GBE1,TNPO1,SNX32,DNPEP,NEO1, GLT8D1,MYT1L,STAG1,CLDN14,TIMM23,PLEKH B2,CAPN11,UBOX5,TCERG1,F10,FAM184A,CAB IN1,PSMD10,FBXL17,PDZD2,SLC9A4,DHX29,G PAM,IRF2,LRP12,WEE2- AS1,ESRP1,HSFX3,UBE2A,BRMS1L,RBM41,RGS 12,ACOXL,CBX3,ACAP2,SLC39A10,ZNF496,CH CHD6,ZNF550,MPHOSPH9,KIZ,RNF10,FAT1,SL C2A2,PIK3R1,IL17B,AGMO,NOVA1,SARNP,SHT N1,ATG12,FNDC3B,MASP1,TMEM232,CD200R1, BTD,CALD1,SH3GL2,CLBA1,HIBADH,CRTC1,PI TPNC1,SLC30A3,DOCK7,PDK3,XKR4,FBXL4,RO R1,CCDC178,ADAMTSL1,PAX5,AAK1,CRB1,UTP 15,ARID1A,THEMIS,SMAD6,RYBP,PSD3,ABCA1 3,ZNF519,MEF2C,FZR1,ST8SIA5,DBNL,ELP4, RYK,MACF1,PXT1,DENND11,TUBGCP3,ITIH5,W EE2,SLC15A5,TFDP2,PTPRE,MFSD6,SERINC3, TBC1D31,ZNF347,PTPN12,PTPRZ1,ACACA,PLN ,EML1,CTDSPL2,SLC66A3,SLC16A7,NEDD4,NE K4,CACNA1E,FLYWCH1,LRRC38,OVAAL,VWA8,C YSLTR2,PARVA,SECISBP2,URI1,SLC9C1,SCML 2,ANKRD28,SNAP25- AS1,CHD6,ZNF609,PRDX1,NDUFAF6,PACS2,AT G4B,AEBP2,SLC7A14,DSC2,GLB1L,SERPINA1, OLFM1,MID1,TSHZ2,POGK,SLC12A2,EFNB3,OT X1,TRIM37,DLG3,PVRIG,FLOT2,SRP68,SV2B, ILK,LEF1,EYA1,MAPK14,MYBPC3,WDR17,ZFHX 4,RAPGEF1,ALG13,EFCAB14,GPRC6A,UNC79,Z NF516,SLC1A2,EIF2S3B,ARHGEF3,GABRB2,GR IN2B,UBXN10,SVOP,CRISPLD1,NOP16,IFNGR2 ,DENND4A,TBC1D20,PLD5,OXR1,SFMBT2,SNX2

			,GRHPR,SCAMP5,IL17RB,PARP2,MINDY3,MYL1,KLHL4,EIF3F,RDH16,FCHSD2,SLC4A7,RNU6-1117P,DNAJB6,NPIPB3,SPCS1,MTUS1,LEMD3,PLEKHF2,B3GLCT,PIEZO1,OR2T11,NEB,CSRNP3,KANSL1L,MRGPRX1,TMEM100,NUP210,PDIA5,VDR,NEK11,CREB3L2,RTN4,GNL3L,CACNA1D,PRKAA2,ARHGEF11,IVNS1ABP,SGPP2,CD163,DPT,BBS2,CLSTN1,TMEM150C,ZNF155,SERGEF,SCYL2,AOPEP,SKAP1,CHM,SKIC3,MRPL3,MYO3A,SERPINE2,NUBPL,PLAAT3,IL12RB2,PIK3CG,ANOS1,COL4A4,TPP1,STIL,ANKFN1,TRGV5,ATP6V1E2,NBPF10,NUP160,IQGAP1,MYCBP2,WIPI1,LATS1,PHYHIPL,ZSCAN25,LOC124903099,KLHL12,SNX30,FANCI,THNSL1,OR9Q1,ACAA1,PRSS23,WNT2B,LRRC37A3,SPNS3,TNFRSF10B,MAP2,RNU6-973P,FBXW11,MTCL1,HSPH1,SUGP2,AZIN2,IYD,SMG6,HLA-DMA,PEMT,ARHGAP6,PPP2R2B,CHD5,JAK1,GPD2,ITGA9,RADIL,KCNB1,GNPAT,HSD17B12,HS6ST3,GRM4,ANKRD27,AIM2,ANK3,ZNF19,TMEM132B,AMT,KITLG,ARID4B,GMDS,PLEKHM2,NR1I2,THSD4,ZNF266,RFX7,RELN,PDHB,ADAMTSL3,NXPH1,CRKL,TENT5A,ARHGEF10,SYTL3,NDC1,SULT1C4,SNAP91,ZNF816,CDKAL1,VAV3,RPS6KC1,CD40LG,VRK1,PAPOLB,URM1,GPAT3,DPYSL3,ZNF420,SCN9A,C1ORF146,SPRING1,SLC16A10,ATRN,CCDC88A,NDUFB9,SNORA36C,SLC35B4,UGT3A2,ARNT2,SPOCK1,HPSE2,PLCE1,TNIP3,TACC2,CYP39A1,FRAS1,DCAF5,GNAL,CSTF1,RNU6-10P,MSH3,BAK1,MRPL45,LAX1,STK26,ENOX2,TENM4,TTC9-DT,PPP3CA,CHRNA5,CES2,MKNK1,DYSF,NKD1,ENSG00000286856,RBPJ,RIN2,PDE6C,ASCC1,CDH10,DENND1A,MZT1,PDZRN3,ADAMTS16,TASP1,NBPF19,MICU1,TAF10,MBD5,BACE2,KDM5A,ZNF549,NOMO1,ARL6IP6,CNOT6,PKIB,ASAH2,FRYL,TBC1D9B,STON1,PXMP2,ASH1L,RSPO2,ZNF805,ACSF2,OR5K1,OR13C9,DISC1,PROX1,MAP3K7CL,FMN1,ZNF891,RALGPS1,CCDC6,PIEZO2,VSIG1,ITGB1,RBM26,CYB561,DRC7,ZNF827,DSE,RHCE,CORO1C,LHFPL6,SPIDR,C17ORF80,NEFL,NAV2,TMEM185B,STK3,CHN2,RNF150,FCAR,C1GALT1,HNF4G,SMCO4,MSR1,EXOSC3,GABRG3,SH2D3C,APTX,TRPC4,RAMP3,GNG2,ZNF705A,FRS3,CPB1,LIMS1,ENPP2,DOCK4,ACKR2,ARPC1A,MCTP2,BCL11B,CERS3,GLCE,PLS3,TSPAN2,PCSK2,KCNIP1,PWWP2A,TBC1D10A,SSBP2,REEP1,RPH3A,CTTNBP2,NME9,CYTH4,NAP1L1,SLC38A6,VPS29,PLPP4,CCDC102B,COPB2,UBR1,CNN2,COL4A2,SLC1A6,ARHGAP32,DYRK3,IP6K2,CHRNA6,TLN2,HDAC4,FYB1,RBM45,SEM1,GYS2,IQCE,LVRN,RPGRIP1L,MYLK,UNC5C,CFAP69,CSMD2,TLR7,FLG2,P3H2,CFL1,DTYMK,WDR7,SLAIN2,HMG20A,CEP85L,TUSC3,COPG2,PLEKHG4B,RBBP8,MED13,RNASEH2B,PHF2,TAAR2,RAI2,AFDN,LRP2,CYTIP,MTDH,LIG1,ZDHHC13,WWC3,CAMK4,SERPINA9,GALNT14,CLPX,VWC2,REPS1,FBXL7,RPA3,RNU6-1229P,TMEM108,CC2D2B,SEZ6L,PCYOX1L,SLC39A8,AFG2B,UPRT,ATXN1,TMEM59L,ADAM17,SEMA4F,ELAVL1,LIPJ,TRAK1,STXBP1,OR6N1,IDH1,LMOD2,TSNAX-

			DISC1,HDGFL2,MUC13,NUP205,PLEKHG1,NHLH1,ELP2,SLCO5A1,SNTB1,ORC4,CYP8B1,APC,TTLL5,INO80,NAA11,MOCS2,ERGIC2,PEAR1,CDA,PPARGC1B,STON1-GTF2A1L,CTRB1,COL27A1,ESR2,NOL10,LNX1,ERC1,ENSG00000287694,SMIM13,ZNF440,RUBCNL,AGO3,C9,PVR,CKAP4,RPL39L,ACER1,IQGAP2,CDYL,RLF,ANKRA2,TAF5L,JPT1,CTNND2,DNAH14,RASSF3,UGGT1,PACSIN2,DPYSL2,NUP210L,CNTNAP3C,GIMAP8,RAB8B,METTL8,NSD2,GRM8,PHACTR3,AARS1,KCND1,TMEM267,JADE3,PHF3,PSMC2,MAPKAP1,EXOC6B,EVC2,GON4L,DNAH6,MGAT5,ACOX1,HAO1,USP24,NUP153,HSD17B4,EFHD1,LCN12,TRMT10B,COL21A1,RAI14,GPATCH2L,PIGR,NRP2,GTF3A,SIPA1L2,CAVIN1,GYPE,METTL15,TMEM165,FOXJ2,RNU4-69P,CEP162,ELOVL7,NR3C1,DLX6,BORCS5,FSTL4,ZC3HAV1,MTOR,XACT,TRAV5,KSR1,FAF1,SNAP25,DEFB124,SGSM1,CHST4,OR11H6,GOLGA8A,ZNF221,CLDND1,PIP5K1A,GH2,BTBD2,MMACHC,RAB6B,HS6ST2,KYNU,ADAL,TBXAS1,ASCC2,SPRED1,GALC,GNG4,CNTNAP4,TUBA3D,CDH26,PDS5B,CRIM1,NALF2,MEI4,RBM46,ABCB5,RNF115,TRIM61,CYYR1,ESD,CDHR3,PER2,ANKRD24,GLI3,LHPP,KAT6A,BCOR,KCNK12,NTRK3,RXFP1,TADA1,GAB2,DIS3L2,SNTB2,PDCD10,PDE1A,RSBN1L,PRAMEF11,LMTK2,HHAT,CREB5,CCDC68,ADGRG6,GOLGA7,FERMT2,GCNA,GPR82,GLUD1,TSPOAP1,ST3GAL2,DNM3,PHIP,EPB41,DENND5A,SS18,SFRP5,DOCK9,AGBL3,ZYG11B,PPARA,NCKAP5,CD244,SHISA6,IGLV3-1,NFASC,HDX,CPEB2,ZNF229,NXPE4,PHF6,HMGB4,FGF7,TBCA,STXBP6,MGA,RTTN,CACNA1A,EDA,LIMCH1,TNFSF13B,MTMR7,PTH2R,COL14A1,RBM47,EIF2A,JARID2,CYP1B1-AS1,FIRRM,HDAC2,TPO,TGOLN2,FGD1,TRIM39,INSYN2B,TRRAP,SEMA3D,NPSR1,EIF4E3,USP48,KCNH5,RRM1,MAGEA1,CADM1,INPP5D,CFAP161,THUMPD1,CLYBL,OR4C6,ATF6,PRKAR1B,GBF1,IPO11,SERPINE1,CCDC88C,BACH1,CUL9,PRAMEF4,EWSR1,TM7SF3,SLC26A5,AKAP13,PKM,GCG,ATP12A,TKFC,LINC02218,PTPN3,STAC,SCN8A,NNT,SSX2IP,ST8SIA2,TTC17,ELP1,IRF8,HIVEP3,HEPACAM2,RFX4,CCDC198,OR6K6,ZNF30,REM1,AGAP1,FREM1,RIPOR2,NDUFA10,CD69,DBR1,FUNDC2,EPS15L1,CYB561A3,PARD3B,XIRP2,KDM3A,MZT2A,CAST,ADGRA3,GPR39,CASK,HSPG2,SLCO4C1,GCNT1,BPIFB3,SLC35F4,GGA2,USH1C,NLRP14,DNAJC13,FRMD4B,PSME3IP1,GOLGA8B,TRDV3
GO:0098794	postsynapse	0.0023195128690132044	*PARN,EIF4A3,DTNB,GPR158,ATP2B2,TANC2,SNTA1,IL1RAPL1,KIF5C,GPM6A,ADCY1,GRID1,NETO1,RNF10,PSD3,MEF2C,DBNL,SLC16A7,NEDD4,EFNB3,DLG3,GABRB2,GRIN2B,RTN4,CLSTN1,KCNB1,ANK3,CRKL,SPOCK1,PPP3CA,CHRNA5,CDH10,DISC1,ITGB1,NEFL,GABRG3,RPH3A,CTTNBP2,ARHGAP32,CHRNA6,TMEM108,SEMA4F,ELAVL1,STXBP1,CTNND2,KCND1,NRP2,SNAP25,DNM3,SHISA6,INSYN2B,CADM1,PRKAR1B,SCN8A*
GO:0005794	Golgi apparatus	0.007892049658271085	*STK16,SCYL3,MMP16,SDC2,ARAP1,CEP85,NFE2L2,PLEKHM3,TPTE2,CLIC5,SPATA16,ZDHHC17,FIG4,AP1S3,EGFR,NEO1,GLT8D1,F10,MPHOSPH9,PIK3R1,SH3GL2,CLBA1,SMAD6,ST8SIA5*

			,DBNL,MACF1,SERINC3,NEDD4,SERPINA1,MID1,IFNGR2,TBC1D20,SCAMP5,MTUS1,PRKAA2,CLSTN1,SCYL2,TPP1,WIPI1,AZIN2,HS6ST3,ANK3,CD40LG,VRK1,SPRING1,ATRN,CCDC88A,SLC35B4,PLCE1,LAX1,STK26,BACE2,ASAH2,ASH1L,DSE,C1GALT1,DOCK4,GLCE,RPH3A,CYTH4,COPB2,SLC1A6,ARHGAP32,TLR7,P3H2,COPG2,LRP2,ZDHHC13,GALNT14,TMEM59L,ADAM17,MUC13,APC,NAA11,ERGIC2,ERC1,GIMAP8,MAPKAP1,MGAT5,TMEM165,MTOR,SNAP25,SGSM1,CHST4,GOLGA8A,RAB6B,HS6ST2,SNTB2,PDCD10,LMTK2,HHAT,GOLGA7,ST3GAL2,DENND5A,FGF7,TGOLN2,FGD1,TRRAP,ATF6,GBF1,ST8SIA2,TTC17,HEPACAM2,HSPG2,GCNT1,GGA2,GOLGA8B
GO:0015630	microtubule cytoskeleton	0.012942086640642543	CETN3,GAS2L1,CHMP1B,DNAH11,CEP85,NFE2L2,FBF1,SERP1,ENKUR,RAB11FIP4,CLIC5,KIF5C,RPS6KA2,CEP290,TACC1,STAG1,FAM184A,PDZD2,CBX3,MPHOSPH9,KIZ,SHTN1,CCDC178,MACF1,TUBGCP3,TFDP2,TBC1D31,EML1,NEK4,MID1,MAPK14,KLHL4,MTUS1,BBS2,STIL,ANKFN1,IQGAP1,MYCBP2,LATS1,KLHL12,MAP2,FBXW11,MTCL1,HSPH1,RADIL,ARHGEF10,CCDC88A,TACC2,DYSF,MZT1,DISC1,STK3,HNF4G,GABRG3,REEP1,CYTH4,CCDC102B,DYRK3,RPGRIP1L,SLAIN2,CEP85L,FBXL7,AFG2B,APC,TTLL5,INO80,NAA11,ERC1,IQGAP2,JPT1,DNAH14,RASSF3,PACSIN2,DPYSL2,DNAH6,CEP162,NR3C1,BORCS5,TUBA3D,CDH26,SNTB2,CCDC68,DNM3,EPB41,SS18,NCKAP5,TBCA,RTTN,FIRRM,CFAP161,CCDC88C,SSX2IP,HEPACAM2,MZT2A
GO:0045211	postsynaptic membrane	0.015517889070892474	GPR158,ATP2B2,SNTA1,IL1RAPL1,ADCY1,GRID1,NETO1,RNF10,SLC16A7,EFNB3,DLG3,GABRB2,GRIN2B,CLSTN1,KCNB1,ANK3,CRKL,CHRNA5,CDH10,GABRG3,RPH3A,CHRNA6,SEMA4F,KCND1,NRP2,DNM3,SHISA6,SCN8A
GO:0031594	neuromuscular junction	0.016683865824993403	COL4A5,SNTA1,DLG3,FCHSD2,SERPINE2,ANK3,CRKL,SPOCK1,ASCC1,PDZRN3,ITGB1,NEFL,HDAC4
GO:0098588	bounding membrane of organelle	0.017888209208310062	SLC18A1,SCYL3,SNX16,CHMP1B,SLC9A9,SNX25,AHNAK,SLC46A2,TPTE2,RAB11FIP4,MISFA,ARHGAP26,RAB44,ZDHHC17,FIG4,AP1S3,OCLN,EGFR,PLEKHB2,SLC9A4,GPAM,ACAP2,CHCHD6,MPHOSPH9,ATG12,SH3GL2,CLBA1,SLC30A3,ABCA13,ST8SIA5,DBNL,SERINC3,PLN,ATG4B,SLC7A14,SERPINA1,TRIM37,FLOT2,SV2B,RAPGEF1,SVOP,IFNGR2,DENND4A,TBC1D20,SNX2,SCAMP5,PLEKHF2,PIEZO1,CD163,BBS2,CLSTN1,TMEM150C,SCYL2,PLAAT3,IQGAP1,WIPI1,KLHL12,SNX30,AZIN2,HLA-DMA,PPP2R2B,GNPAT,PLEKHM2,SPRING1,SLC35B4,PLCE1,BAK1,DYSF,DENND1A,BACE2,ASAH2,STON1,PXMP2,ITGB1,CYB561,DSE,CORO1C,FCAR,C1GALT1,MSR1,MCTP2,GLCE,RPH3A,CYTH4,VPS29,COPB2,ARHGAP32,IQCE,TLR7,COPG2,LRP2,ZDHHC13,GALNT14,TMEM108,SLC39A8,TMEM59L,ADAM17,ERGIC2,ERC1,RUBCNL,CKAP4,IQGAP2,PACSIN2,RAB8B,EVC2,MGAT5,ACOX1,HSD17B4,PIGR,TMEM165,BORCS5,MTOR,SNAP25,CHST4,RAB6B,HS6ST2,SNTB2,PDCD10,HHAT,GOLGA7,ST3GAL2,DENND5A,NFASC,TGOLN2,ATF6,GBF1,ST8SIA2,HEPACAM2,FUNDC2,CYB561A3,CASK,SLCO4C1,GCNT1,GGA2,DNAJC13
GO:0019897	extrinsic	0.019579129	BLK,RNF10,JAK1,CRKL,SNAP91,GNAL,PPP3CA

	component of plasma membrane	048371814	*,CDH10,GNG2,STXBP1,APC,SNAP25,GNG4,CDH26,CDHR3,FERMT2,STAC*
GO:0005911	cell-cell junction	0.020275106152738625	*CDC42BPA,AHNAK,FBF1,OCLN,CLDN14,PDZD2,FAT1,SLC2A2,PIK3R1,CRB1,THEMIS,DSC2,DLG3,FLOT2,SKAP1,IQGAP1,ANK3,PPP3CA,CDH10,ASH1L,FMN1,ITGB1,TRPC4,LIMS1,CNN2,RPGRIP1L,AFDN,MTDH,ADAM17,APC,PVR,CTNND2,PACSIN2,CDH26,CDHR3,FERMT2,NFASC,STXBP6,CADM1,SSX2IP,PARD3B,CASK,FRMD4B*
GO:0019898	extrinsic component of membrane	0.022286956877727297	*SNX16,BLK,RNF10,PIK3R1,PIK3CG,WIPI1,JAK1,CRKL,SNAP91,GNAL,PPP3CA,CDH10,GNG2,RPH3A,PLEKHG4B,STXBP1,APC,SNAP25,GNG4,CDH26,CDHR3,FERMT2,STAC*
GO:0097708	intracellular vesicle	0.023854304132580655	*SLC18A1,NHLRC3,STK16,SNX16,CHMP1B,SLC9A9,SNX25,BLOC1S5-TXNDC5,SLC46A2,ITSN1,ENKUR,RAB11FIP4,ARHGAP26,RAB44,SPATA16,ZDHHC17,FIG4,AP1S3,SH3TC2,CEP290,OCLN,EGFR,SNX32,NEO1,PLEKHB2,CAPN11,SLC9A4,ACAP2,ATG12,SH3GL2,CLBA1,SLC30A3,AAK1,ABCA13,DBNL,PRDX1,ATG4B,DSC2,SERPINA1,SLC12A2,FLOT2,SV2B,MAPK14,RAPGEF1,GABRB2,GRIN2B,SVOP,IFNGR2,DENND4A,SNX2,SCAMP5,FCHSD2,PLEKHF2,CD163,CLSTN1,SCYL2,SERPINE2,TPP1,ATP6V1E2,IQGAP1,WIPI1,KLHL12,SNX30,ACAA1,HSPH1,AZIN2,IYD,HLA-DMA,JAK1,GRM4,ANKRD27,PLEKHM2,SYTL3,SNAP91,RPS6KC1,DPYSL3,CCDC88A,STK26,DYSF,RIN2,DENND1A,BACE2,STON1,DISC1,ITGB1,CYB561,DSE,CORO1C,FCAR,MSR1,CPB1,ACKR2,MCTP2,PCSK2,RPH3A,CTTNBP2,NAP1L1,VPS29,COPB2,CNN2,ARHGAP32,TLR7,FLG2,COPG2,LRP2,CYTIP,ZDHHC13,TMEM108,PCYOX1L,ELAVL1,TRAK1,STXBP1,IDH1,ERGIC2,CDA,RUBCNL,CKAP4,IQGAP2,PACSIN2,RAB8B,PSMC2,MAPKAP1,PIGR,TMEM165,BORCS5,FSTL4,MTOR,SNAP25,SGSM1,GH2,RAB6B,SPRED1,ESD,SNTB2,LMTK2,GOLGA7,NFASC,TGOLN2,PRKAR1B,SERPINE1,PKM,GCG,SCN8A,ST8SIA2,TTC17,CCDC198,CYB561A3,SLCO4C1,GGA2,DNAJC13*
GO:0098978	glutamatergic synapse	0.024151271153441387	*PARN,EIF4A3,ATP2B2,IL1RAPL1,GPM6A,ADCY1,GRID1,NETO1,RNF10,SH3GL2,SLC30A3,DBNL,SLC16A7,EFNB3,DLG3,FLOT2,MAPK14,SLC1A2,RTN4,CLSTN1,PPP3CA,CDH10,DISC1,ITGB1,CTTNBP2,SLC1A6,SEMA4F,ELAVL1,STXBP1,PACSIN2,NRP2,SNAP25,TSPOAP1,DNM3,SHISA6,PRKAR1B,SCN8A*
GO:0005622	intracellular anatomical structure	0.026920827012060656	*SLC18A1,KLHL13,ABCB7,PBX4,ACOT12,NHLRC3,MYO9B,COL4A5,ERG,PARN,STK16,XK,MAX,TNS3,SCYL3,TEAD1,EIF4G3,ACSM3,SNX16,IFTAP,SIAH3,CETN3,RASA4B,GAS2L1,CHMP1B,MMP16,FAXC,PRORP,ZNF534,TOX3,DNAH11,SLC9A9,CDC42BPA,POLR2J,POLG,EXOC2,SNX25,SDC2,ABHD12,COX10,CIITA,FANCC,ARAP1,UQCRHL,ANKLE2,CEP85,RPRD1B,CIP2A,AHNAK,ACTR5,EIF4A3,DLEC1,NFE2L2,SCHIP1,TFEC,NFYC,BLOC1S5-TXNDC5,H2BC18,UPP2,CYP19A1,SLC46A2,DTNB,GPR158,VRK2,ITSN1,R3HDM2,ATP2B2,PREX2,ESPNL,SCRN1,FBF1,SERP1,POLR1A,PLEKHM3,ENKUR,TPTE2,RAB11FIP4,HSFX4,SNTA1,PPCDC,ASZ1,NUB1,MISFA,FLI1,SPATS2L,CLIC5*

			,ESRRB,ARHGAP26,ZFHX2,RBP7,TRHDE,RAB44,PTPN4,SETBP1,ZBTB7C,ITGA2,IL1RAPL1,KIF5C,BLK,SPATA16,WNT16,KRT86,SETD3,ACOX3,ADCY1,RPS6KA2,EFR3B,TRIM24,ZDHHC17,HLCS,FIG4,AP1S3,CFAP47,FOXP2,KMT2D,LPAR3,GIPC2,TXLNG,SP110,SH3TC2,CEP290,TACC1,SH3PXD2B,OCLN,CFAP91,FASTKD5,NETO1,EGFR,GBE1,TNPO1,SNX32,DNPEP,NEO1,GLT8D1,MYT1L,STAG1,CLDN14,TIMM23,PLEKHB2,CAPN11,UBOX5,TCERG1,F10,FAM184A,CABIN1,PSMD10,FBXL17,PDZD2,SLC9A4,DHX29,GPAM,IRF2,WEE2-AS1,ESRP1,HSFX3,UBE2A,BRMS1L,RBM41,RGS12,ACOXL,CBX3,ACAP2,ZNF496,CHCHD6,ZNF550,MPHOSPH9,KIZ,RNF10,FAT1,SLC2A2,PIK3R1,AGMO,NOVA1,SARNP,SHTN1,ATG12,MASP1,TMEM232,BTD,CALD1,SH3GL2,CLBA1,HIBADH,CRTC1,PITPNC1,SLC30A3,DOCK7,PDK3,FBXL4,ROR1,CCDC178,ADAMTSL1,PAX5,AAK1,UTP15,ARID1A,THEMIS,SMAD6,RYBP,ABCA13,ZNF519,MEF2C,FZR1,ST8SIA5,DBNL,ELP4,RYK,MACF1,PXT1,DENND11,TUBGCP3,WEE2,TFDP2,PTPRE,SERINC3,TBC1D31,ZNF347,PTPN12,ACACA,PLN,EML1,CTDSPL2,SLC16A7,NEDD4,NEK4,FLYWCH1,OVAAL,VWA8,PARVA,SECISBP2,URI1,SCML2,ANKRD28,SNAP25-AS1,CHD6,ZNF609,PRDX1,NDUFAF6,PACS2,ATG4B,AEBP2,SLC7A14,DSC2,GLB1L,SERPINA1,OLFM1,MID1,TSHZ2,POGK,SLC12A2,OTX1,TRIM37,DLG3,FLOT2,SRP68,SV2B,ILK,LEF1,EYA1,MAPK14,MYBPC3,ZFHX4,RAPGEF1,ALG13,ZNF516,EIF2S3B,ARHGEF3,GABRB2,GRIN2B,UBXN10,SVOP,NOP16,IFNGR2,DENND4A,TBC1D20,OXR1,SFMBT2,SNX2,GRHPR,SCAMP5,IL17RB,PARP2,MINDY3,MYL1,KLHL4,EIF3F,RDH16,FCHSD2,RNU6-1117P,DNAJB6,NPIPB3,SPCS1,MTUS1,LEMD3,PLEKHF2,B3GLCT,PIEZO1,NEB,CSRNP3,KANSL1L,TMEM100,NUP210,PDIA5,VDR,NEK11,CREB3L2,RTN4,GNL3L,CACNA1D,PRKAA2,ARHGEF11,IVNS1ABP,SGPP2,CD163,BBS2,CLSTN1,TMEM150C,ZNF155,SERGEF,SCYL2,AOPEP,SKAP1,CHM,SKIC3,MRPL3,MYO3A,SERPINE2,NUBPL,PLAAT3,PIK3CG,COL4A4,TPP1,STIL,ANKFN1,ATP6V1E2,NBPF10,NUP160,IQGAP1,MYCBP2,WIPI1,LATS1,PHYHIPL,ZSCAN25,LOC124903099,KLHL12,SNX30,FANCI,THNSL1,ACAA1,PRSS23,WNT2B,MAP2,RNU6-973P,FBXW11,MTCL1,HSPH1,SUGP2,AZIN2,IYD,SMG6,HLA-DMA,PEMT,ARHGAP6,PPP2R2B,CHD5,JAK1,GPD2,RADIL,KCNB1,GNPAT,HSD17B12,HS6ST3,GRM4,ANKRD27,AIM2,ANK3,ZNF19,AMT,KITLG,ARID4B,GMDS,PLEKHM2,NR1I2,ZNF266,RFX7,RELN,PDHB,ADAMTSL3,CRKL,TENT5A,ARHGEF10,SYTL3,NDC1,SULT1C4,SNAP91,ZNF816,CDKAL1,VAV3,RPS6KC1,CD40LG,VRK1,PAPOLB,URM1,GPAT3,DPYSL3,ZNF420,C1ORF146,SPRING1,SLC16A10,ATRN,CCDC88A,NDUFB9,SNORA36C,SLC35B4,UGT3A2,ARNT2,SPOCK1,PLCE1,TNIP3,TACC2,CYP39A1,DCAF5,CSTF1,RNU6-10P,MSH3,BAK1,MRPL45,LAX1,STK26,ENOX2,TENM4,TTC9-DT,PPP3CA,CES2,MKNK1,DYSF,NKD1,ENSG000

			00286856,RBPJ,RIN2,ASCC1,DENND1A,MZT1,PDZRN3,TASP1,NBPF19,MICU1,TAF10,MBD5,BACE2,KDM5A,ZNF549,NOMO1,ARL6IP6,CNOT6,PKIB,ASAH2,FRYL,STON1,PXMP2,ASH1L,ZNF805,ACSF2,OR13C9,DISC1,PROX1,MAP3K7CL,FMN1,ZNF891,RALGPS1,CCDC6,ITGB1,RBM26,CYB561,DRC7,ZNF827,DSE,CORO1C,SPIDR,C17ORF80,NEFL,NAV2,STK3,CHN2,RNF150,FCAR,C1GALT1,HNF4G,MSR1,EXOSC3,GABRG3,SH2D3C,APTX,TRPC4,RAMP3,ZNF705A,FRS3,CPB1,LIMS1,DOCK4,ACKR2,ARPC1A,MCTP2,BCL11B,CERS3,GLCE,PLS3,TSPAN2,PCSK2,KCNIP1,PWWP2A,TBC1D10A,SSBP2,REEP1,RPH3A,CTTNBP2,NME9,CYTH4,NAP1L1,VPS29,CCDC102B,COPB2,UBR1,CNN2,COL4A2,SLC1A6,ARHGAP32,DYRK3,IP6K2,TLN2,HDAC4,FYB1,RBM45,SEM1,GYS2,LVRN,RPGRIP1L,MYLK,CFAP69,TLR7,FLG2,P3H2,CFL1,DTYMK,WDR7,SLAIN2,HMG20A,CEP85L,TUSC3,COPG2,PLEKHG4B,RBBP8,MED13,RNASEH2B,PHF2,RAI2,AFDN,LRP2,CYTIP,MTDH,LIG1,ZDHHC13,WWC3,CAMK4,SERPINA9,GALNT14,CLPX,REPS1,FBXL7,RPA3,RNU6-1229P,TMEM108,SEZ6L,PCYOX1L,SLC39A8,AFG2B,UPRT,ATXN1,TMEM59L,ADAM17,SEMA4F,ELAVL1,LIPJ,TRAK1,STXBP1,IDH1,LMOD2,TSNAX-DISC1,HDGFL2,MUC13,NUP205,PLEKHG1,NHLH1,ELP2,SLCO5A1,SNTB1,ORC4,CYP8B1,APC,TTLL5,INO80,NAA11,MOCS2,ERGIC2,CDA,PPARGC1B,STON1-GTF2A1L,COL27A1,ESR2,NOL10,LNX1,ERC1,ZNF440,RUBCNL,AGO3,PVR,CKAP4,RPL39L,ACER1,IQGAP2,CDYL,RLF,ANKRA2,TAF5L,JPT1,CTNND2,DNAH14,RASSF3,UGGT1,PACSIN2,DPYSL2,NUP210L,GIMAP8,RAB8B,METTL8,NSD2,PHACTR3,AARS1,JADE3,PHF3,PSMC2,MAPKAP1,EXOC6B,EVC2,GON4L,DNAH6,MGAT5,ACOX1,HAO1,USP24,NUP153,HSD17B4,EFHD1,TRMT10B,COL21A1,RAI14,GPATCH2L,PIGR,GTF3A,SIPA1L2,CAVIN1,METTL15,TMEM165,FOXJ2,RNU4-69P,CEP162,ELOVL7,NR3C1,DLX6,BORCS5,FSTL4,ZC3HAV1,MTOR,XACT,KSR1,FAF1,SNAP25,SGSM1,CHST4,GOLGA8A,ZNF221,PIP5K1A,GH2,BTBD2,MMACHC,RAB6B,HS6ST2,KYNU,ADAL,TBXAS1,ASCC2,SPRED1,GALC,TUBA3D,CDH26,PDS5B,MEI4,RBM46,RNF115,TRIM61,ESD,PER2,GLI3,LHPP,KAT6A,BCOR,TADA1,GAB2,DIS3L2,SNTB2,PDCD10,PDE1A,RSBN1L,PRAMEF11,LMTK2,HHAT,CREB5,CCDC68,ADGRG6,GOLGA7,FERMT2,GCNA,GLUD1,TSPOAP1,ST3GAL2,DNM3,PHIP,EPB41,DENND5A,SS18,DOCK9,AGBL3,ZYG11B,PPARA,NCKAP5,NFASC,HDX,CPEB2,ZNF229,PHF6,HMGB4,FGF7,TBCA,STXBP6,MGA,RTTN,CACNA1A,EDA,LIMCH1,TNFSF13B,MTMR7,COL14A1,RBM47,EIF2A,JARID2,CYP1B1-AS1,FIRRM,HDAC2,TGOLN2,FGD1,TRIM39,TRRAP,NPSR1,EIF4E3,USP48,RRM1,MAGEA1,INPP5D,CFAP161,THUMPD1,CLYBL,ATF6,PRKAR1B,GBF1,IPO11,SERPINE1,CCDC88C,BACH1,CUL9,PRAMEF4,EWSR1,AKAP13,PKM,GCG,ATP12A,TKFC,LINC02218,PTPN3,STAC,SCN8A,NNT,SSX2IP,ST8SIA2,TTC17,ELP1,IRF8,HIVEP3,HEPACAM2,RFX4,CCDC198,ZNF30,REM1,AGAP1,RIPOR2,NDUFA10,DBR1,FUNDC2,EPS15L1,CYB56

			1A3,PARD3B,XIRP2,KDM3A,MZT2A,CAST,CASK,HSPG2,SLCO4C1,GCNT1,BPIFB3,GGA2,USH1C,NLRP14,DNAJC13,FRMD4B,PSME3IP1,GOLGA8B
GO:0031410	cytoplasmic vesicle	0.031248274833414073	*SLC18A1,NHLRC3,STK16,SNX16,CHMP1B,SLC9A9,SNX25,BLOC1S5-TXNDC5,SLC46A2,ITSN1,ENKUR,RAB11FIP4,ARHGAP26,RAB44,SPATA16,ZDHHC17,FIG4,AP1S3,SH3TC2,CEP290,OCLN,EGFR,SNX32,PLEKHB2,CAPN11,SLC9A4,ACAP2,ATG12,SH3GL2,CLBA1,SLC30A3,AAK1,ABCA13,DBNL,PRDX1,ATG4B,DSC2,SERPINA1,SLC12A2,FLOT2,SV2B,MAPK14,RAPGEF1,GABRB2,GRIN2B,SVOP,IFNGR2,DENND4A,SNX2,SCAMP5,FCHSD2,PLEKHF2,CD163,CLSTN1,SCYL2,SERPINE2,TPP1,ATP6V1E2,IQGAP1,WIPI1,KLHL12,SNX30,ACAA1,HSPH1,AZIN2,IYD,HLA-DMA,JAK1,GRM4,ANKRD27,PLEKHM2,SYTL3,SNAP91,RPS6KC1,DPYSL3,CCDC88A,STK26,DYSF,RIN2,DENND1A,BACE2,STON1,DISC1,ITGB1,CYB561,DSE,CORO1C,FCAR,MSR1,CPB1,ACKR2,MCTP2,PCSK2,RPH3A,CTTNBP2,NAP1L1,VPS29,COPB2,CNN2,ARHGAP32,TLR7,FLG2,COPG2,LRP2,CYTIP,ZDHHC13,TMEM108,PCYOX1L,ELAVL1,TRAK1,STXBP1,IDH1,ERGIC2,CDA,RUBCNL,CKAP4,IQGAP2,PACSIN2,RAB8B,PSMC2,MAPKAP1,PIGR,TMEM165,BORCS5,FSTL4,MTOR,SNAP25,SGSM1,GH2,RAB6B,SPRED1,ESD,SNTB2,LMTK2,GOLGA7,NFASC,TGOLN2,PRKAR1B,SERPINE1,PKM,GCG,SCN8A,ST8SIA2,TTC17,CCDC198,CYB561A3,SLCO4C1,GGA2,DNAJC13*
GO:0032421	stereocilium bundle	0.03810631756795042	*ESPNL,CLIC5,PTPRQ,FCHSD2,SLC4A7,BBS2,MYO3A,DOCK4,ANKRD24,RIPOR2,USH1C*
GO:0098793	presynapse	0.039250510492924814	*SLC18A1,GPR158,ITSN1,ATP2B2,SCRN1,ITGA2,GPM6A,ADCY1,ZDHHC17,SH3GL2,SLC30A3,ROR1,AAK1,DBNL,EFNB3,SV2B,SLC1A2,SVOP,SCAMP5,GRM4,SNAP91,CHRNA5,DYSF,CDH10,DENND1A,STON1,DISC1,NEFL,MCTP2,RPH3A,CTTNBP2,SLC1A6,CHRNA6,STXBP1,ERC1,RAB8B,BORCS5,SNAP25,RAB6B,CNTNAP4,CDHR3,TSPOAP1,DNM3,SCN8A,CASK*

Table S6. Differential analysis of the genomic interactions of rDNA clusters in Mel Z cells grown on plastic or on Matrigel. Differential 4C analysis was performed as described in the Materials and Methods section. The Excel file is attached separately. Related to Figures 3 and 4.

Table S7. Venn diagram showing the intersections of rDNA-contacting genes detected in Mel Z cells grown on plastic (≥ 30 contacts) with genes that either decreased or increased the number of contacts when grown for 15 h on Matrigel. Related to Figure 4.

Names	total	elements
4C decreased Plastic	2240	*AL157886.1 FSTL1 CDH12P2 CASC20 C10orf90 APBB2 AC243829.5 PFKP ABCC6 MIR3171HG AC012501.1 ESYT1 STYK1 RPL19 RTN1 HS3ST3A1 RNU7-188P AC100775.1 AC012409.2 RGN TRGC2 CDK14 ZSWIM1 EPN2 MSRB3 PDE1C ENSG00000293331 MREG HNRNPCL4 NOSTRIN ING3 ENSG00000290149 MAP3K3 NINL WSCD1 ENSG00000287515 SLC12A8 OSBPL8 LRRN1 MED13L HERC2P2 NFIA ENSG00000286309 ENSG00000291144 LINC00698 SUGCT POTED STX8 AC032019.1 PHF20 FOXK2 CPNE4 TXNDC8 RBM17 AC021517.1 SMYD3 ARHGEF26-AS1 GARS1*

		RAPGEF6 ARHGEF9 TAFA2 ABR KRT8P32 TLR8-AS1 ISM1 MAOA BLTP3B LINC01163 AC012355.1 LINC02438 HYDIN2 ENSG00000287308 HCCAT5 TRAPPC9 PCDHB1-AS1 SLC16A5 LINC01545 TMEM255A FNDC1 PREP MAP3K13 EIF1AX PPM1A ARL13B RPL15P21 SAMSN1 RN7SL556P AC010291.1 C12orf75 THADA LIMD1 IMMP1L TF SNX10-AS1 CHST11 THRB MYBPC1 USP42 IPP RCSD1 SPTBN1 UBTFL3 AF228730.2 CDC42EP3 AC013472.2 ENSG00000286111 AC021269.2 MIR3117 MYO5C LINC00841 MAPK10 CACNB1 ITPRID1 USP32 AHCYL2 DAPP1 ATF7 CD101-AS1 GNG12 FGF1 CHRNB2 CDC14C SEZ6 CLDN6 LINC01967 KCNQ5 CDH8 ARHGAP15 GNB4 AGTPBP1 AL356490.1 LOC101929710 ATF7-NPFF FHL1 TRMT6 PPP1R37 RBPMS ATP10A LINC02457 SH3PXD2A SPINK5 MRPS24 OPA3 SKAP2 PRND PIGL ATP7B GPBP1L1 PPM1H RPS6P19 KCNJ6 PCAT1 RPL21P82 BRD4 DOK4 ZAN LINC00517 AC007405.2 AC092378.1 TNRC18 MIR9-3HG LINC02828 CAMK2B GRIP1 ELMO1-AS1 CASC19 ZNF962P OR4N3BP ARHGAP11A-SCG5 TLK1 LOC124901321 URGCP-MRPS24 GPATCH8 AC008764.4 DDI2 PGM1 DCT AC073071.1 DLGAP1 FAM193A PECR LNCATV LRP5L SLC38A9 LINC01982 CDRT15P9 LINC00348 ZNF264 QRSL1P2 LINC02133 HBP1 SDR16C5 ABCG8 RGPD2 XPO4 OTUD7A KALRN AL583785.1 AC060234.3 AC107909.2 SUMF1 USH2A OPN3 MAP4K3 AC092078.2 XXYLT1 FAM157A LINC01194 FPGT-TNNI3K GALNT9 SNORD114-10 NOTCH2 LOC442028 CD93 LOC105379362 FKBP5 TMOD2 SLC44A5 SDK2 HMBOX1 SIM1-AS1 AP003900.1 ZNF551 LINC00882 RNU2-49P NPNT AC019211.1 DSC1 EPB41L3 MAP6 AC012363.2 MORC1 PARVB CDH11 RN7SL266P SHANK3 WDR26 NR4A3 CYP2U1-AS1 SPTLC3 GAS1RR AGAP12P LINC00494 AC005154.4 ARID1B LOC101927948 DPY19L2P2 LOC105375387 ENSG00000289752 AMYP1 E2F7 ACVR2B-AS1 L3MBTL4-AS1 SLC14A2 ENSG00000291166 FAM169BP AC091304.2 FOXO1 LINC02311 ZNF611 STYXL1 AC244517.9 LINC02156 AC098588.1 STK39 PDE11A-AS1 SMURF2 SRL ADAMTS12 DAAM2 FER1L6 TBC1D16 PLXNA4 AL592078.1 LINC02542 LINC00504 CDK18 LOC124900810 URB2 YBX1 STK24 DIPK2A ARMT1 CFAP70 DCDC1 CACNA1C FAM20B AC046195.1 ENSG00000287410 FAM3C ASB4 SNORA5B IDO1 AC026786.1 AC015922.1 ARB2A LOC105370489 PRKN ENSG00000286937 CNN3-DT NLGN4X EXOC4 RMND5A HSD17B2-AS1 LINC02044 PNPLA3 PRDX3P3 LINC00320 AC092447.10 F13A1 ENSG00000288041 PIK3C2B XPO6 AC103876.1 KDM7A SLC44A3 SLC5A7 GNA14-AS1 MARCHF9 FAM83B IFT172 AL136317.2 RNU6-258P CHD7 GPSM1 AL121900.2 CBLL2 AL136372.2 PTBP3 ZBTB44-DT ARL17B FAM117A CDON SERPINI1 SLC17A6 NLK SDF4 THSD7A OVCH1-AS1 GNAI1 LINC02405 SPATA31D2P MRPL13 AC138932.2 MAPKAPK3 NCAM2 PAPOLG CHML H3P38 TIAM2 CFAP54 INTS6-AS1 ZNF10 RORA-AS1 AC015804.1 TRAK2 AL139383.1 ENSG00000293038 CNMD ENSG00000286229 HERC2P3 LRP8 TAGLN3 AC018767.3 CNTN1 LINC00992 AC002451.2 AGL MEOX2 MAPRE3-AS1 STAT5B ENPEP LINC01242 NUDT19 ARHGEF4 LOC102724428 UBE2O MUC17 DDIAS AC025884.1 MTUS1-DT DUXAP9 CPEB4 AC092100.1 EIF4ENIF1 HEATR9 LOC124900848 CALCR CHRFAM7A ELMO1 ERBIN CR383658.2 RN7SL430P ZNF121 HERC2P9 ENSG00000287881 MBTPS2 RGS22 PLS3-AS1 LINC00598 CD247 ENSG00000290948 ENSG00000287621 MCC CEP192 TRIM52-AS1 PTH GEMIN8P1 ENSG00000287008 ROCR ADAR DNAH10 PDE10A TM9SF2 ENSG00000286250 TOX2 PDHX CLCN3 RHOJ CDC37 FGF10 ZFYVE16 JAZF1-AS1 LINC03104 MECOM AC009878.1 RSPH10B COL4A6 CATSPERB TSPAN18 SAMD15 NLRP8 ENSG00000286800 FRG1JP STK32B PLG NEURL4 POLR2J3 HERC5 CNTNAP5 LINC02505 LOC124900957 NUDT13 AC008109.1 CDH7 LOC101929457 ENSG00000290585 PGPEP1L UBE2E2 ILDR2 SCEL-AS1 AC034229.1 MGC27382 NCK1 ENSG00000287744 CD84 FAM168A NONO RARRES1 AC138512.1 KLHL42 NREP AF064860.1 EXO1 CCDC71L GABRA5 AL591684.2 ARHGAP23P1 EBNA1BP2 AL512380.2 SNORD114-9 DYNLRB2-AS1 NEXMIF KCNQ3 ENSG00000286274 LOC401913 ENSG00000288755 MTCP1 SH3BGR MROH6 TRAJ17 AC104248.1 AC004492.1 AC231532.1 CHRM3 KDM2B PREX1 HEY2-AS1 RGPD3 AL133500.1 PRIM2BP AIG1 ERICH1 IGLV1-41 ZNF404 PMS1 WRNIP1 ABCC13 COL24A1 ANO3 TYW1 GOLGA8R AC009511.1 COL11A1 AC119677.1 AC112721.2 AC113391.2 RSF1 AC022509.1 AC063949.2 KCTD3 NCOA7 C2orf88 RNU2-33P SERTM2 ZNF804A SEPTIN10 CIDEB MARCHF1 OR4N3P ENSG00000286376 ENSG00000286922 SLC45A1 TTC33 AL773545.1 MCPH1 ENSG00000289143 MYO1D ENSG00000287045 SESTD1 ENSG00000291338 LINC01661 ENSG00000288553 DNAAF9 BRAF ZNF214 COL1A2 DTNA TMOD3 ENSG00000286020 LOC340512 GEMIN5 EEF2KMT MRTFB ENSG00000286147 XPO7 KCNS3 AC011447.3 VWA3B ENSG00000288902 RPL34P11 ENSG00000287608 FREM2-AS1 ENSG00000290114 COL12A1 STEAP2-AS1 ABLIM1 ENSG00000287763 AC009226.1 RBMS1 ALPK2 CHRNA7 HECW1 HMGB1 ZNF304 DYNLT2B AC021733.1 CDIP1 AFF3 RFLNA AC062039.1 AL158198.1 LPP HIVEP1 ENSG00000293389 ATRX STK24-AS1 UBTFL5 LOC105371956 TGFB2 MDFIC EPB41L4A LAMA2 ADD3 TRIO ZNF385D ATXN7L1 FBXO16 ZNF85 ENSG00000291054 LINC00269 LINC00366 HOXC13 AL049875.1 SMARCC1 RAD51B NDST1 LINC00355 MUSK ENSG00000287722 KRT223P ZNF73P KCNJ18 JAM3 ENSG00000287741 KCNIP3 SFPQ AC027338.2 PNPLA4 AC004485.1 MAP3K5 ALKBH1 ARPP21 SNORA5A

		AF130359.1 RGS3 ZNF299P DUXAP8 NUP93 LDHAL6DP AC108673.3 ZCCHC4 MRPS9-AS2 RERG ZNF407-AS1 G2E3-AS1 ESRRAP2 OR5AU1 PRKAR2A FILNC1 RBM33 AL604028.1 TBC1D5 CTIF CFAP57 ENSG00000286902 FADS2B CALCRL-AS1 ATP11A AC023389.1 MRPL33 RBM23 AC008695.1 ZNF154 CSTF3 ATP4A SLC15A4 HIGD1AP9 ENSG00000288087 AL024474.2 LOC105369165 AC244517.11 AC013652.1 CDH12P4 CERS6-AS1 PCDHB8 GOLPH3 AC015908.7 PRKG2 MBTPS1 NCF2 PIK3C3 PIGV RNU6-1216P USP10 SND1 EPIC1 ENSG00000288692 HMCN1 RN7SKP141 IGFBP7 RSKR EPHA5 MACROD2-IT1 RABGAP1L-DT LINC03076 PTPN20 ENSG00000290849 AC087501.3 LRRK2-DT SKOR2 AC130650.1 CRLF3P2 CBR4 ENSG00000289694 ATP13A4 RUNX1 AL162718.1 AC009055.2 ENAH SNX10 ATP2C1 ALDH1A2 TMEM230 AL354718.2 PPTC7 STON2 MIR3681HG PTGFR IGHV1OR21-1 AL138895.2 FAM171A1 STRADA PCLO LINC01725 HUWE1 ZNF595 ASPH RRAS2 SH3BGRL ZDHHC4 UBR5 AP000894.1 AC114781.3 AC096558.1 GTF2F2 AC105031.2 SIM1 KIAA1217 ABTB2 SOX30 AC244131.2 TMEM132C ANKS1B CHL1 TRAJ16 SPOCK3 GSN PLS1 AC007991.3 TCP11L1 SPON1 MAPRE3 MICB-DT ANO2 TLR8 HPS3 AL391361.1 AC011447.2 KIF21A LOC124900600 SLIT3 ARMH3 FMO8P CACUL1 NLRP7 ARHGEF39 PHC2 MITA1 ROBO1 AC142086.6 ZNF578 ENSG00000290721 ENSG00000287021 ANKRD11 ITPR1 AL162254.1 PKP4 AC105052.4 CPM AL160286.3 DNAAF11 LINC01924 ENSG00000288069 PAK3 LINC01033 PWRN1 CDC42 NDUFAF7 AC007846.2 ABI3BP RNU1-83P KLF12 LOC124900504 AXDND1 AC011824.3 IPMK LINC03042 AC073575.2 LINC01376 CYP2A13 RPL23AP51 TMEM183A AC126763.1 ENSG00000289956 CAMK1G PRUNE2 DOK6 CASC2 SERPINB9P1 LINC01122 RNA5SP96 AC073130.2 ENSG00000288694 PRKAG2 ENSG00000290490 LINC02965 LINC00158 MLLT3 CCNT2 BTG4 HAVCR1 DRG1 RSPH10B2 TRAPPC10 LINC02942 ENSG00000288714 AC034206.1 CPQ CNOT10 CPEB1-AS1 MR1 KRTAP21-2 LOC102724710 F8 TNFRSF12A OASL GDPD1 LINC00237 CPT2 MIR325HG TAS2R14 AP000320.1 VPS8 AC010329.5 MTX3 LINC00703 AC092121.1 ENSG00000286878 C4orf50 NXT1-AS1 AC020743.2 LINC01732 ENSG00000287211 ISY1 CACNA2D3 ZNF831 ZNF429 AC242426.2 CXCR5 VN1R31P NCKAP1 PRPF40A CLDN12 KRTAP13-6P DTD1 CNTN3 MGAM LINC02427 ENSG00000289085 LINC01060 CILK1 HOXC13-AS FOSL2 PACRG IL1RAPL2 ADGRD1 HNRNPCL3 AC020912.1 EPHA3 MUC19 CHCHD3 ZNF337-AS1 ENSG00000289131 LATS2 LINC02141 TYW3 MYH14 AC008581.2 APCDD1L-DT SPDYE2B MRPL32 TPRG1 ENSG00000286168 KLHL33 ERP29 CDYL2 LRRTM4 ARHGAP18 AC013652.2 UVRAG PTPRN2 CACNB4 MYOM2 SMIM10L2B-AS1 AC113386.1 LYN ADGRB3 USP6 LOC100131779 KLF7 LINC00390 MIR4636 SUMO2 LOC102723883 GRIK1 MTA3 CTSLP4 HSD11B1-AS1 LINC02203 UBL3 DIP2B LOC107985126 NME7 SEC24C AC068234.1 AC073869.5 MIR9-1HG LARGE1 AC109583.3 PRAMEF9 ENSG00000286686 ATP6V1H AC009135.1 NUP58 FAM177B FIGNL1 SLC12A1 LINC01538 PUS7 AC068547.1 PDZD8 PAMR1 CCDC33 AC003006.1 CA10 LINC01878 RRM2 CRYBB1 SIL1 LDB2 MYRFL AC024610.2 SPTB UNC93A C21orf62 MIR3159 LOC84214 ALDH1A1 NMNAT3 FMO5 RNASEL AC010082.1 NAV1 LINC02008 CMKLR2-AS DBF4B PAQR5 DSCAS CORIN TMCC1 LRRC7 PCDHB16 TEX41 LINC00458 AC060765.1 AC027288.1 CDH12P1 DCP2 RBPMSLP PPM1L PDZRN4 SGMS2 FAM151B-DT TMED7-TICAM2 STX17-DT ENSG00000288055 SLC41A2 FER1L6-AS2 NOS3 AL355306.2 LIPE-AS1 HMGB3P30 LOC105378402 KCNIP4 VSX1 ZNF280B EIF3L NOMO2 NALCN-AS1 TNRC6C ZNF90P3 PRAMEF27 LINC01208 PGAM1P5 MLLT10 BPHL ERCC6 NOS1AP EIF2AK4 LINC00840 RN7SL738P IGKV2OR2-2 KIAA1614 AC027031.1 NIHCOLE AC016027.4 MSRA AL031599.1 LOC100419786 SYNPR RAB12 SIMC1P1 LINC02235 LINC01505 IMPG1 HMGCLL1 LINC01911 DSCR4 XKR6 PKN2-AS1 LOC101928335 OFCC1 ENSG00000287682 MAGEL2 AF165147.1 MATN2 TGFBR2 SGO1-AS1 AC141257.2 CMKLR2 ITGA11 CFAP77 MARK4 DDX60L MDN1 AC092131.1 LINC01901 SYNJ1 TTC6 TCF7L2 LOC102724421 LINC02192 ZNF800 AC114781.2 CHAF1B ENSG00000290067 KIAA1328 ENSG00000286326 ZNF462 MTRF1 ECHDC2 AC090386.2 OVCH1 CRTAC1 E2F6 ZFHX3 ENSG00000291336 CUX1 POLR2C UNC119 SHISA2 GPM6B RRAGD C2orf50 ULK3 AC083902.1 LINC02484 LOC339166 SPDYE2 GPR156 ZMAT4 CNOT10-AS1 ENSG00000289949 AP000529.1 AL445224.1 TUBBP3 GKAP1 CACNA2D4 LHFPL3 RGPD1 PPP2R5E AL161751.1 ENSG00000288954 RPL39P33 KPNB1 INHBA-AS1 ENSG00000290385 AC010332.2 TTN-AS1 GSDMD ADGRV1 RASGRF2 ST7 SERBP1 DNM1P33 AC013401.1 QTRT2 EVA1C AL161716.1 EPB41L5 GAD1 ENSG00000288187 SCG5 AL035078.2 NUDCD1 AC068254.1 MIR603 AL050327.1 WAC WHRN OCA2 AC093668.1 DAPK1 TDP2 ULK4P2 ALDH9A1 LOC100419045 RAB6C-AS1 ZNF678 LINC01362 ACSM2B PRUNE1 SAR1B TUBB8P6 TTC7B INSYN2A BTG3 APP GATA4 ATF7IP2 SMAD5 IGF2BP1 PPIL6 AC116634.1 MAMLD1 LINC02610 RPL9P14 RNLS AC009093.4 ENSG00000291100 ANKRD20A7P AL163932.1 GSKIP AL136985.3 NEPRO-AS1 RNF38 ADAM12 LOC107986837 UBE2G1 AC138969.2 NBPF12 PELI2 ATP9B MIR1273F BTG3-AS1 CACNA2D1 DHX35 ENSG00000287347 SP140L CRACDL HCN1 ENSG00000286288 CHRM5 SLC13A1 PI4KB AC129926.2 FRY LINC00670 RHOT1 ELOVL5 HERC2 AC010809.1 AL354810.1 HOOK3 USE1 AL513323.1 PIK3R5 LYZL1 PALLD ENSG00000290523 TLR1 LINC02767 CECR2 TAS2R30 TMCO3

		CASC16 RFESDP1 BMT2 AC233699.1 OR52P2P RIPK4 LINC01876 AVPR1B LMAN1L ENSG00000286406 AC091073.1 GOLGA6L10 QDPR FRRS1 IQCM AP002373.1 PTK2 AC006482.1 ENSG00000293467 AC079414.1 NOP9 IGLV3-2 USP7 TICAM2 ENSG00000293472 SCP2 NPEPPSP1 SH2B2 LINC02518 ATP1B4 AL031963.1 ZNF41 AC006153.1 AC019270.1 AVEN LINC02250 AC046185.1 GATD3 CELF1 HYCC1 DNAH12 POTEG POGZ PIP4K2B PRRC2B SYNM-AS1 UQCC1 ENSG00000288996 LRRK2 NKAIN3 INO80D ZNF395 AF279873.3 IKZF3 GET1-SH3BGR SLC38A4 AC006043.1 AC244517.4 FRY-AS1 MIR548XHG CLSTN2 APRG1 ARIH1 SAMD12-AS1 CFAP298 PLD1 AC015908.2 ITFG2 VTCN1 PKHD1 ENSG00000287039 MEG8 TENT5C KIF23-AS1 UBXN7 RARB GDNF-AS1 LINC02435 AC104169.1 ENSG00000290429 SNORD114-7 PJA2 TCF4 AC079742.1 ENSG00000287334 MGST1 RBM44 ARHGEF38 PAEP COP1 TIAM1 CSRP3 PCA3 PHACTR1 MLIP IQSEC2 ACVR2A GNAO1 AC243830.3 AC034232.2 AL033530.1 ASAP2 LINC02309 TIPIN VIT AC091078.1 LINC01324 BAALC-AS1 AL606760.2 RASSF4 C6orf58 LOC124902439 ENSG00000288635 MIR6882 SNTG2 SNORA5C AC092979.1 AC060834.2 AC091230.1 NTM AC091133.4 MEIS1 AMMECR1 NEDD4L AC004805.1 FP236315.2 CYP1D1P LINC00639 TMED11P ENSG00000289368 BRCC3 CDH12P3 ENSG00000288683 AC093010.2 LINC00636 FECHP1 DUSP14 LINC01237 PRMT8 SNX13 AC090365.1 AC093893.1 RABGEF1 AC068724.3 CYB5R4 KIT ENSG00000273937 TBC1D8 EPHA7 ENSG00000291067 SPAG9 OSBPL3 AC104574.2 ANTXR1 ENSG00000293304 RMEL3 LOC100652967 AC131254.2 AC069257.3 LINC02256 AC068299.1 C19orf47 AC007326.2 SPDL1 WNT5A TRABD2B WIPI2 PEX26 ARFGEF2 TEX2 AL671862.1 CTNNB1 ANKRD17 EFCAB5 POTEKP ENSG00000286811 AL096701.3 COL19A1 RUNX1T1 CTSK LOC102723341 PRKDC AGAP4 AC097626.1 LINC00507 IPO5 ESR1 AC026124.1 LOC124900584 TNFRSF19 ZNF479 AC091045.1 LUZP2 RPL3P1 PTPN14 LINC02240 SCFD1 ITGB8 ERC2 POFUT2 LINC02973 DMGDH TRANK1 BCAR3 TPD52L1 L3MBTL4 PPM1E ZXDC AD000090.1 VPS53 LOC100129404 FRMD4A AC099788.1 CTSLP6 LMX1A DCAF8L1 MIR3670-3 WWOX EGFEM1P LOC128462377 TPST2 FIGN CIMAP2 BTBD8 PARS2 AC103740.2 BNIPL HUNK TRAJ19 NEK10 LINC00824 LINC01448 SNX29 FILIP1 LERFS TMEM62 ANAPC10 SLC44A3-AS1 COPB2-DT FBXO15 EEDP1 PTGER4 AC018467.1 GREB1L ZNF215 AC015574.1 BBX PDE7B AC096711.2 USP40 RASSF8-AS1 PPP1R15B-AS1 VWC2L BEST3 LINC01681 LYPD6B DOCK1 HDDC2 ENSG00000287180 AC131902.1 DIAPH3 AC027644.4 CCDC107 TARDBPP2 AC005580.1 GPC4 BPGM PKIG ENSG00000286614 LINC00276 RPL21P10 EFNA5 LINC00536 IGF2BP2 AC073316.1 AL035420.1 AL391869.1 ACTR3B AC018638.8 ZNF722 CDC14B SYNE3 ENSG00000287684 LINC02808 PLCH1 OR1M1 HECTD1 VKORC1L1 PSG11-AS1 NGEF TRPC5 NEUROD2 FNIP2 ZIM2-AS1 AC097478.1 AASS JAML KAT14 UGGT2 CCNJ UBE3B ADORA2A-AS1 TNFSF11 PTPRF CASC15 LINC01473 AC010280.1 IKBKB DUX4L51 FXR2 SNHG33 AC010809.2 SH3BP5 MIR3670-4 AC068413.1 AKAP6 LOC101927690 ENSG00000293257 TRBV10-2 AC103409.1 USF3 AC002074.1 LYVE1 AC004870.4 APOH ARHGEF28 OR8B8 CLCN1 RAB3GAP2 TASOR STEAP1B CCPG1 AC104781.2 GPRIN1 EML6 POU6F2 LINGO2 OPCML CEMIP XIST AC074286.1 ATF2 LINC00877 AC091151.1 AC140481.3 RNU6-986P ZNF426 KCNJ12 HDAC8 CHRNB3 ACVR1 HLA-DQA2 PID1 COL6A5 HYAL4 EBF2 CCDC91 GRID2 ZNF423 ZCCHC17 KCNJ3 AC008632.1 AP000487.2 SNORA63D SLC17A6-DT RB1 LINC00466 NSMCE1 AC078923.1 MPV17 LOC100131635 IFNLR1 SMIM2-AS1 RNU6-826P NTNG1 GOLGA6L4 AL365214.3 PDE4B DDX10 MYHAS LINC02994 PKD1L3 KIF13A NLRP4 ADIPOR1 ENSG00000286655 SLC23A4P LOC124900205 ZNF143 GABRB3 GOSR2 PET117 SHROOM4 AC093865.1 ZSCAN23 SMG7 AC091489.1 PLPP3 UNC80 HNF1B RNU6-1311P IMMP2L ZNF615 AP001599.1 LAMP2 CCDC54 SSPN SPSB1 KIRREL3 GABRG2 F5 CFAP95 SCG5-AS1 AK5 CPS1 NCOA2 TMEM120A BNIP3L AC104984.2 AC023300.1 ENSG00000287614 PRKCH ACACB AC021088.1 AGPAT5 RABGAP1L DLC1 LINC01948 ENSG00000286476 LOC101927141 DENND2B LINC00603 XRCC5 TBRG4 LOC107984536 RGS17 LINC01500 LINC01965 HMGB3P2 GFRA1 SHLD2 SPHKAP KCND2 INPP4A BMX OR5W1P MIR663AHG TRAJ18 ZNF418 SBF2-AS1 AC012593.1 SLC24A3 RN7SL563P RAD54L2 MPRIP LINC02351 TAF15 TPST1 GRIA4 RNA5SP489 POLD3 RAB18 AP000526.1 ZNF860 TPH2 AL079305.1 POU2F1 AL445648.1 LINC00508 PRPF40B AC004870.3 AC244517.6 TMTC1 AL592490.1 ETV1 NRK ENSG00000287635 RGS7BP MGRN1 LOC339298 AC010745.2 TEX11 LINC02384 MID2 AC087683.2 RBM28 MIR198 LRTM2 LOC105375972 SIK1 FBXL13 ZCWPW2 GLOD5 OR10J4 ERCC6L2 ENSG00000291293 ENSG00000286779 PIPOX AP002336.2 RPS2P1 TP53BP2 ZNF850 LINC00592 KAT2B ESPNP LSINCT5 GOLPH3L TMTC2 WLS ACTR3C ANKS1A IGKV1-6 RYR3 TMPRSS11B PRCP SUPV3L1 AL035446.1 MEF2D MYO1B ZPLD1 MGAM2 MYL4 RPL39P31 AC091588.2 GTF2A1 SPTLC1 AC015922.2 AL137076.1 AC008758.4 AGR3 CDK5RAP2 FLVCR1 AC023830.3 UBE2E3 FSIP2LP RPL15P18 SAMD3 EPN2-AS1 RFC3 ERICH6B FARSB ARHGEF7 CCDC54-AS1 AL445430.1 ANKRD26P1 SAMD12 CU634019.3 ENSG00000293462 DACH1 RPL21P5 ATF3 LINC03082 TRDN LINC01117 LOC105377043 LINC01934 GASK1A ENSG00000290597 NPHP1 FBN2 AL136146.2 LINC00499 AC111152.2 TEKT4P2 ENSG00000290412 IKZF1 DAOA-AS1 AC005154.5

		SLC26A8 CAPZA2 SNTG1 AC004147.4 GGT8P AC011405.1 TRAJ20 AC002066.1 CACHD1 SRI ATP1B1 GREB1 AL158154.2 ADAT2 ARNT BDNF LDLRAD4 TFCP2L1 ACBD6 SEMA3E BCLAF3 HAS2-AS1 SCIN AL163195.3 ENSG00000293265 AP004609.1 MALRD1 TENT4B MACO1 AC010601.1 ZNF665 TLR6 ENSG00000293512 ENSG00000287443 CTBP2P10 NDST4 ADAMTS9 AC004917.1 ADGRF2P ITPR2-AS1 STIM1 TSEN15 TMEM237 TMEM161B ENSG00000288563 HAUS6 KIF16B RAB11FIP2 CDH2 EEFSEC COX7B2 LAG3 DPP10-AS1 ENSG00000291189 Z95331.1 PPARG LOC124902888 TMEM72-AS1 AC016573.1 TANC1 PAPPA LOC101927605 ENSG00000290217 IRAG1-AS1 ASB3 AC239585.2 MTTP GPR84-AS1 CHRNB4 LOC102724934 AC019322.1 AGAP9 CTBP2P9 AP000282.1 USP9X TGM5 NEK7 INTS15 ZNF80 PDXDC2P TENM1 IL13RA2 ENSG00000293489 LINC01322 RORB AL136441.1 CDK6 GABRB1 LINC02903 MYOF AP001116.1 LINC01754 AC142384.1 LTB4R2 ATG12P1 JAKMIP1 SH3KBP1 NALCN AC116903.2 SMOX AC020718.1 AVIL LPCAT2 ENSG00000286163 GDAP1 AMY2A DDX60 AC023509.6 AC126755.4 ENSG00000287801 ENSG00000289870 TFAP2D ISY1-RAB43 LINC00398 EIF2AP4 TTC39C MIR3670-1 MYLK4 BEND7 DIO2-AS1 Z98043.1 TPRXL USP25 KIF2C AA06 MYO5A SIPA1L3 CTSLP1 LINC00393 SLC6A13 ZNF286A-TBC1D26 LRFN2 WDFY4 SPTBN5 AC068138.1 AC092862.1 EIF1B-AS1 SFRP4 BLTP1 SPDYE16 LINC01239 OR4Q2 PNRC1 IL1RAP ULK4P3 SEMA6A-AS1 AC015922.3 ENSG00000286662 AL772307.1 JCAD VAV2 TFRC DANT1 VPS13A TES OSBPL6 SH3TC1 GRIA3 PNPLA8 SPDYE6 AC087564.1 FBN1 AC108474.1 HAPSTR1 SERPINA7P1 RAB31 ENSG00000291283 EXO5-DT DIPK2B MAP3K1 ZFP82 DDX4 IQCB1 COA1 CLEC6A ZNF292 RAPGEF4 BMPER FUT10 AC109779.1 LINC00922 RNU6-984P CLASP2 CLTC ZNF521 RPRD2 ZNF761 PDXDC2P-NPIPB14P AC024230.1 ANGPT1 DUXAP10 SEMA6A VIPR2 ZNF567-DT EIF4G1 PARAIL TMPRSS15 AL133372.2 EEF1A1P11 B3GAT2 ATP6V0A4 ACSM5 PHLPP1 ZNF618 ITGA4 DNAJC6 CXXC4-AS1 SCIRT ROCK1 AL390860.1 NCAPH M1AP AC093515.1 ENSG00000286072 TAX1BP1 AC008739.5 OR7A15P AL445430.2 TBC1D4 AC093843.1 APIP AC012456.1 MMP28 FAM13C NECTIN1-DT LINC02971 ZNF701 KCNT2 CRYBB2P1 NUP98 CLEC3A IKZF2 PML BX571673.1 FOXO3B SACS AC012616.1 AFF2 LAYN MGLL SCFD2 SCARA5 LONRF3 SLC5A12 PLEK2 TNFSF4 ENSG00000293021 AFAP1L2 DGKG SPATA25 LINC00923 SP100 ENSG00000287616 RNA5SP260 PRKG1 PLXDC2 ELAVL4 SBF2 CDH9 RF01880 FOXRED2 AL731556.2 AL359736.1 NDUFAF2 CFAP298-TCP10L LINC01524 UMODL1 AC022335.1 RFPL4B TTC28 TSEN2 MAGI2 GALK2 PCDH19 CYP4F62P ENSG00000287783 NIN LINC02060 SPG21 ZNF286A GARS1-DT ST7-OT4 ANKFY1 ZNF69 CCDC144CP PRSS50 ASIC2 MIR4645 ENSA GMDS-DT ENSG00000286259 PPP4R1L MIR9-2HG FNDC3A KHDRBS3 AIMP1 SLC30A10 AP4S1 MYO5BP1 TPK1 FOXG1-AS1 HSPBP1 PRKD1 CALCRL CASC17 SLC20A2 USP34 AC091053.1 STK4 AC138305.1 PCDH10 AC023300.3 ENSG00000289174 TNNI3K AL646090.2 ENSG00000287184 DNAAF4-CCPG1 RAB6D TMEM131 SPC25 AC112493.1 ENSG00000287378 TMEM161B-DT ST6GAL2 RNASE11 AC018742.1 AC244205.1 SCAMP1 PTCHD4 TXNL4AP1 RPL27A LINC01002 ANPEP AC021979.1 LINC01821 PRLR ZNF569 GOLGA8O LINC01697 PTPRA AC011287.1 ARL2BPP10 ENSG00000289397 AP005436.1 ZNF180 AC008758.3 PDE11A NMI RAB6C PCCA AF241726.2 CLVS1 YLPM1 EYS IL16 LINC00972 ENSG00000291276 AC012485.1 DIPK1A LINC02814 SULT1C2P2 ZSWIM7 ZNF470 ENSG00000286932 AC107373.1 LINC02339 POTEH EDARADD LINC02511 LINC00558 GNA14 OXNAD1 CR2 CR392039.3 LPAR6 ENSG00000286152 ZNF56P CTBP2 TTN LINC01721 NCALD RPS10P13 HHIP DYRK1A LOC105377209 PPFIA1 TMEM30A LINC01705 MYRIP ERICH2-DT LINC00343 PPP1R1C ZNF286B AL355838.1 KCNE2 UNC13C CUL4A LINC02882 NDUFAF4P1 AC105052.3 AC015687.1 SERINC1 LINC00265 KCTD16 CHSY3 EFCAB13 TXNRD3 TRAM2-AS1 AC026358.1 EYA4 ZNF888 AL391361.2 LINC01748 AC069287.3 DLGAP2 AL163953.1 ARL15 SPATA13 LINC01288 ZNF736 AL031432.1 MAP7D3 SEMA5A ENSG00000288035 5S_rRNA AL354861.2 LINC02762 ENSG00000290805 EIF3FP1 ELL2 SAMMSON LINC00886 LAMA4 PMEPA1 LINC01811 UBE3C ATE1 ENSG00000293330 ETNK1 AC110772.1 TTPA MYO5BP2 PEX5L CYP4F30P SLC2A1-DT PVT1 GCNT1P3 ARL17A CHAC2 GATAD2B METTL3 FMNL2 KRT85 UAP1 ZKSCAN7-AS1 PARD3 LINC00691 KCNN4 LINC01104 TRDC ZNF33A AL353132.1 SLC25A48 CP ENSG00000293385 FUNDC1 RNU6-849P NPAS3 COL6A6 BMAL2 ATP8B1 PGPEP1 AC026462.4 SATB1-AS1 BZW1-AS1 RNF6 SEMA3C VAV1 SMYD1 FANCB PEPD DPY19L1 ENSG00000289871 GRM3-AS1 GRIA2 VDAC2 KIAA0825 AC002127.2 MIR3670-2 ENSG00000276197 VPS13B SPART AC018697.1 LOC102723684 IGLV4-3 LINC02315
4C increased Plastic	139	*LINC01201 PTPRR ENSG00000286540 DIP2A SLC8A1 AL356108.1 RSRC1 DPYD SLC22A14 AHR AC022523.1 ASTN2 LRP1B FGF12 PARP4 GNGT1 RNF217-AS1 AC078828.1 IRAG1 RAPGEF5 CDH19 TPTE2P6 BABAM2 MEF2C-AS1 DOP1B EXT2 DIAPH2 LOC101927609 LINC01830 DMBT1L1 CCSER1 ENSG00000286033 ARMC8 MYO16 AC058822.1 FRMD6 LINC01091 ENSG00000289002 EBF1 C8orf34 CPED1 LINC03116 INPP4B DGKI SDK1 EPHA6 IGF1R WDR72 NLGN1 ENSG00000287916 SOX2-OT CFTR PCDH9 NKAIN2 RBMS3 AL603840.1 CFAP20DC AC108010.1 SHC4*

		LINC02112 FRMD6-AS2 TESHL LARS2 HULC AC005670.2 FBLN5 SPEF2 WDSUB1 OGDH GHR LRRC4C AP001341.1 NEBL NAALADL2 OSBPL10 LANCL2 ZFPM2 SLC35F1 GALNT17 CFAP20DC-AS1 SGCZ KCNAB1 AL121718.1 BEND5 LSAMP MIPEP PWRN4 FTO LINC02326 LINC00299 SFI1 CALN1 AC007848.1 STS ATP8A2 AL591463.1 PLEKHA5 AP005328.1 SYT16 BCAS3 MCTP1 KMT2E LINC03060 SYNE2 TTLL6 ALK SEMA3A MAD2L1-DT DCLK1 FAM135B VPS41 LINC00879 USP13 ADGRL4 ENSG00000293037 LINC01470 SRGAP2 HEATR4 SPECC1 PTPRT GABPB1 THSD7B SORCS1 CAMKMT AL355499.2 PLCB1 MICAL3 KMT2CP4 RANBP17 ENSG00000286648 MSH2 MGAT4A SLF1 SLC39A11 KSR2 PDE8B DLEU1 LINC02208 NRXN1-DT
4C decreased 2.0	1055	*MSRB1 AF240627.1 BBOX1 MEFV LINC01889 FLJ40194 AC025423.2 LOC101928894 SMIM17 LOC105375623 AC008667.3 BANK1 SRPK1 PCMTD1P7 AC125634.1 SYN3 LINC00210 AL133216.1 AL353626.3 FP671120.1 HCG24 AC243562.2 LOC102724642 Metazoa_SRP TMEM266 ENSG00000290700 PAK6-AS1 AC090696.1 PABPC5 CDK8 MRGPRX11P AC011477.7 FAM182B ENSG00000287879 C8orf44-SGK3 CBLL1 AC092138.2 BCL2 ZNF239 AL133257.1 XRN2 CNOT1 RICTOR SAMM50 AC244035.2 UNC13B CROCCP3 HNRNPA1P42 Z68871.1 IL6R AC104455.1 MIR153-2 ARMC6 HK3 REXO1L5P TTC36-AS1 SORBS2 DYRK4 ABCC6P1 ENSG00000291259 PRCPP1 RIC3 AC018442.2 AC016550.2 KCNK10 EXOC1 ETFA NPFFR2 GOLGA8CP ANKRD26P3 AL137802.1 ENSG00000288005 SLCO1B3-SLCO1B7 AKIRIN1 AC084211.1 ZNF568 ADCY3 ARHGEF26 CRAT37 ARHGAP11A-DT CPD TANK AC073834.1 GOLGA8EP ENSG00000286515 UBE2E2-DT GPR135 CBR4-DT PLA2G4C AC090772.2 AC007221.2 MIR6129 LINC01378 UBE2Q2P2 NUP133 ENSG00000287286 LOC101929200 CASC9 AC099499.1 SLC16A2 FMO9P SULF2 LOC105374069 SMS TAF1D GCH1 LINC03062 ALG9 AC092423.1 MEGF11 AC093423.3 MELK ENSG00000290766 ZNF536 NUTM2B-AS1 SRRD DEFB130A AC066613.1 AC108066.1 PYDC2-AS1 PCNX2 KANK4 ENSG00000286329 OR7E5P RLIG1P2 OR1B1 AP001605.1 AC127024.8 PCYT1B KIR2DL1 LINC01111 CBLN2 PPP1R17 HNRNPA1P30 RPL10P1 MRO AL591501.1 ZFYVE9 ZNG1A RN7SL678P AC008667.4 ACSS3 SCTR AMPH HNRNPH1 MSC-AS1 AC015849.3 ALOX12 AL160272.2 TMBIM1 BPIFB1 EMB SLC35F5 AC015845.2 SLC31A1 AC011477.6 TMEM182 RNPC3-DT MUC12-AS1 GOLGA8Q LIFR-AS1 LOC121725015 EFL1 AP000561.1 RN7SL113P AC112128.1 DEFB130B MDM2 TDRD3 AL133370.1 ITPRID2-DT AL137918.1 IZUMO1 LINC00334 ITPRID2 LOC107986178 CFAP210 RMDN1 SYF2P1 AP003100.2 SMARCA4 HS6ST1 SGTA ADGRG2 AKAP3 SLC22A13 DEFB115 TBL1XR1 TNFSF8 LOC105375421 FBLN2 LINC02448 OR4G6P GOLGA8K C5AR2 PPP1R36 AC103681.2 SCLT1 LITAFD TXNRD1 TNFRSF21 ENSG00000286479 MBTD1 RPL23AP27 PPP1CB TRIOBP MTHFD1L AC092535.1 AC006511.5 RNF111 IST1 RAB22A ATXN1L ENSG00000287725 TBC1D32 ESYT2 GNG7 PGR POTEM FAM182A L3HYPDH LEKR1 AC008914.1 AC018618.2 LOC122455341 CHIC1 C13orf42 AC145543.1 MTERF3 AC074132.1 ENSG00000287058 OR4G4P MBP AP000820.2 TANGO6 RPL9P12 DOK5 TET1 NDUFA12 ZNF737 ELMOD3 AC093227.2 BLZF1 SUZ12P1 AC112242.1 TMEM135 MED26 IGBP1P1 RIMKLA RFFL LINC02177 AC006330.1 MIR2052HG SCOC-AS1 ST3GAL5 ZNF821 RPS13P2 RPL5P31 TMEM132D SUCLG2 AC026150.3 SNORA72 C2CD5-AS1 RPL9P8 ZSWIM3 AC097532.2 BPI ZNF734P AL353612.1 LBH LINC01707 SETD4 TWIST1 HORMAD1 ENSG00000290095 UBR4 KTN1 GULP1 GSTA10P JHY RN7SL418P AC110792.1 RPL26P26 UGT2B28 ARL6 AC125613.1 ENSG00000286328 RAP1GDS1 PALS1 IGHVII-22-1 LINC02388 CHPT1 AC091965.3 ACTR3 MPPED2 ENSG00000290674 LINC02226 C8A ATP5PBP5 AC245060.6 IGHV3-72 AC245427.1 AMBRA1 REXO1L10P DSP PTK2B UBE2FP3 DERA ECPAS LINC01755 AL132656.3 ABCG1 SCOC AP001464.1 CDK12 LINC02858 LINC02445 CAMSAP3 ENSG00000286622 NOVA1-DT MYO6 KIFAP3 ENSG00000289748 LINC02303 LINC01340 BORA PRTFDC1 ENSG00000288031 Z73965.1 ENSG00000286218 GSTA9P AC109361.2 ATP6V1G1P2 ENSG00000293012 AC090679.1 SLCO3A1 SLC26A5-AS1 RN7SKP94 SNORA40 GRK3 TRAF3 PTPN2P2 CHD3 AC073575.1 GLUD1P4 CD40 EFCAB7 RALBP1P1 ZMYND8 GSAP SPICE1 PRMT2 ATP6V1G3 NMNAT1P1 TMCC2 AL353626.1 POLR2CP1 GRIK4 ZNF765 RNU6-785P RASEF GRHL2 AHI1-DT RNA5SP114 AC009403.2 POLA1 AC087477.2 AC012462.2 ST8SIA5-DT SLCO1B7 AL035706.1 ADAMTS19 LINC02227 LOC338694 SCHLAP1 EEF1B2P5 AL669831.1 AC005832.4 SMARCA5 AC095032.2 ITGB6 SMIM7 ENSG00000290427 BAGE2 NR1H2 AC068726.1 ADAM9 MAD1L1 ENSG00000291002 FAR2P1 IDI1P1 FAR2P3 RN7SL495P GARNL3 TMED5 RNFT2 AC025423.4 DCAF8L2 MTREX CASP7 SLC26A11 AC015819.4 AC105924.1 PSMA2P1 RPL5P5 TTC7A GOLGA8S ATP6V0E2-AS1 AC114316.2 UBE2B LINC01912 ENSG00000286272 MGMT POLQ LOC101927026 ARHGAP23 ENSG00000289723 FLJ43315 AC007326.4 SHMT1 FGF7P5 POU5F1P3 PRICKLE2 RN7SL759P ENSG00000286206 RN7SL536P GAPT SF1 ITGB3BP WDR47 PKN2 OR4M2-OT1 LINC02211 LINC00433 ITGAM AL137230.2 AC019183.1 CPA1 PRDM11 GOLGA8M AC016382.1 ELOVL2-AS1 LINC01497 IGHVIII-22-2 AC087477.5 FUT1 CC2D2A AC093908.1 TCP10L DKKL1P1 MYRF ORC2 LOC100506869 OTX2 LINC01189 LINC02146 LOC102724849 LINC02335 TGM6 SETP1 AL590399.2 COX18 TMCO4 CCDC80 NEMF PRODH MYO3B-AS1 ENSG00000286414 DIAPH1 U6 ADGRL3*

		LINC02160 DEFB126 AP002075.1 PCM1 HELLS SHROOM3 ZNF355P TM2D2 IL6ST AC092326.2 TMEM177 ENSG00000286806 PAK5 KIF18A LOC101927745 SEC61GP1 ANAPC5 KMT2A ARHGAP44 MLF2 RAD51L3-RFFL PAUPAR GFAP DISP2 LINC00501 ARHGEF33 AL139022.2 CAP2 PSMD1 CSNK1G3 SRFBP1 FAR2P2 WWTR1 COBL ZNF607 RASAL2 AL359232.1 AC022017.1 NRCAM HIPK2 ANKIB1 RPGR NUTM2A-AS1 PCSK5 EGLN3 AL133319.1 CLEC4A MTCO3P12 ZNF28 LOC124904710 AC124290.1 RGPD8 ENSG00000291239 GOLGA8T MIR497HG WNT4 ACTBP1 AC120036.1 AC011632.1 AC134980.2 FGF7P6 ENSG00000293191 URAHP LPAR1 ANKRD55 AL450423.1 YTHDC1 ZKSCAN7 AOAH PHF2P2 RASA3 LINC00581 ENSG00000288565 LANCL1-AS1 UBBP4 IL7 C2CD4A PNKD ENSG00000287476 SLC25A53 OR4F6 RNU6-387P TFIP11 GRAP2 RTKN2 ENSG00000289862 ENSG00000291260 DMP1 HSD17B2 LINC02745 HMGCS2 TLN1 SLC4A8 ZNF181 MTMR1 EPS8 ARHGAP21 LOC105378976 ENSG00000289253 CAND1 ENSG00000287231 GZMAP1 IPO7P2 ENSG00000286132 AL132656.2 RPL36P14 ZNF529 GSTA11P DMTN RASGRF1 KATNIP FRMD5 AC092944.1 ADGRE4P LOC101928107 MCU RNA5SP404 ALCAM MPP4 AC006019.3 ENSG00000289444 ABRAXAS2 EDNRA EFHC1 PTMS HHLA2 RYR1 SLAMF1 HIGD1AP15 ZNF728 SLC25A21 AC012038.1 AC018731.1 RGPD5 AL359757.2 ABCC11 MYH2 AL356585.3 KLF2P2 ENSG00000290410 AAMDC GOLGA8N GAPDHP70 DDX24 EML5 PRIM2 MEGF10 GGTA2P ENSG00000288044 LINC01445 GOLGA8DP GABBR1 TMEM25 AC066613.2 PPL ENSG00000286192 C1QTNF7-AS1 ANKRD20A1 ENSG00000293363 TMEM212 AC006963.1 RPL13P7 POC1A LMCD1-AS1 ATP8B4 ERCC1 FAM3B CFAP44 ZNF254 BUB1B-PAK6 LINC02888 ENSG00000290416 COL5A2 P2RY8 LINC02294 FNTB MIR548AG1 TEX9 RAD54B MCFD2 GRIA1 IL13RA1 FAM81B AC069277.1 REPS2 ZNF468 UQCRB-AS1 ARMH4 ATXN2 AC008517.1 H2AC1 DENND2A TRDN-AS1 NFATC3 KCNMB3 GOLGA6L5P GXYLT2 SLC7A11 LINC01038 ALG11 KCNMB3P1 MEP1A OR4D2 ABCA12 LINC00391 RPL7P52 CD86 MIATNB AC023813.4 AC126335.1 WWP1 POLR2M COL8A1 MIR4527HG MIS18A-AS1 SMAP1 STK32A-AS1 RERE ALS2 ECT2 ENSG00000286481 MTARC2 GSG1L BBOX1-AS1 HERC1 NR3C2 CRADD LOC105372316 OR4X2 AL390195.1 ZC4H2 TTC19 AC034213.1 SLC27A6 REXO1L9P GAREM1 ANKUB1 NXN TSPAN12 NEK5 IL17RD NAMPT LINC01182 ELOA FGF7P4 AC025278.1 ENSG00000291261 TTC36 KLF2P3 TNRC6A LOC613266 LINC00290 DMXL2 CR383656.6 STIM2 ENSG00000288843 AC021351.1 ENSG00000290928 LINC02547 LINC00571 TTC8 GCOM1 ENSG00000290596 WDR77 LOC101927950 ZNF589 CYP3A43 XPNPEP3 ENSG00000293062 ZNF616 PLA2G7 LINC01926 RPS29P27 FKBP15 TENT5B PPIAP56 AC092447.8 OSBPL9 RAB11FIP1 DPEP1 UGT2B24P DSCR9 ZNF106 COL4A1 MYZAP LOC100419851 MX1 ENSG00000290421 AL512444.1 AL160004.1 CHURC1-FNTB AC024075.3 UBE2D4 AP001207.2 FAM66B OR2A14 AC015923.1 FSBP HSBP1L1 CD47 GRAP CLDN1 AC009055.1 RNU6-705P LARP4 EGLN3-AS1 AC023813.3 ENSG00000286833 SGK3 SPINK8 TSHR ENSG00000287534 LDOC1 ATP6V0E2 MTCH2 LOC101930420 EPHB2 AC091965.1 ENSG00000286080 MEP1B SLC17A5 TEX15 LINC01644 RGPD6 PDLIM5 LINC01753 EFCAB11 EYA2 LINC02293 ZNF652 LINC02832 PSMA3-AS1 PDE6A RN7SKP86 AL512310.1 OR2T7 PTEN MTX1LP LOC107984132 MAST2 TC2N SETD2 CA13 UTP4 REXO1L4P AGA-DT PDC-AS1 ANAPC1P4 LUCAT1 VEPH1 MAB21L3 AC093423.2 NAPSB CROCC SOX21 MIR1243 ENSG00000286487 LIN54 LRRC8C ZNF197 ZNF397 MIS18A AL592546.1 CHODL-AS1 LINC00906 EEF1A1P2 TRPV6 ENSG00000293320 MARK1 IQCH PAK6 PLSCR2 ENSG00000288768 MBOAT1 C2orf27A ATP13A3 LOC105376244 OR4G3P CAMK2D RN7SL719P SWT1 PTCD2P1 ENSG00000287505 AC099753.1 SERPINB7 IL31RA PPIG ZNF407 SUN1 CUL4AP1 AL137802.3 LINC00351 COP1-DT AC245128.1 RNA5SP472 PTPN20CP OR2T27 AC138123.1 SLC9A7 MUC12 ABCD1P5 AP005212.4 ST8SIA1 DLGAP1-AS5 LOC101928622 ENSG00000291215 ASPA IQCH-AS1 ATG5 ENSG00000287083 MIR922 AC007611.1 STK32A ENSG00000289972 SPATA22 ZNF234 KCNC3 ZC2HC1C ARMC7 KBTBD11 NAP1L4 AL731684.2 LINC01667 VSTM2B-DT AC090241.3 PAPPA2 ENSG00000291036 SLC12A9 AL109840.2 MTERF4 CRYBG3 AC008591.1 GOLGA8IP TRERF1 ENSG00000286003 NELFB COL25A1 LRRC9 Z83844.1 NIPAL2 GARIN2 AC135983.1 MX1-AS1 CPPED1 TBX15 AC010653.2 AC109454.4 AC008897.2 MYO5BP3 EFHC2 LINC01931 SMYD2 ENSG00000293497 ENSG00000286207 FAM186A RN7SL106P PLUT LINC00251 AC091885.2 ENSG00000287516 LIX1-AS1 SEC23A NSUN2 ENSG00000290620 PTPRK ZC3H13 PHACTR4 ACYP1 LETM2 RGPD4 OR2M5 SYNDIG1 ASXL3 LINC02699 MIR3663HG MYH15 AC011891.2 CYP4X1 TRAPPC12 REXO1L2P EBF4 ENSG00000286740 SMURF1 AP001021.2 CSTF2 TRIM51JP LAMB1 BPTF ENSG00000289040 GRAPL H2BC1 RPS27P6 NFIB UQCC2 LIFR LNCBRM LINC02360 KCNAB2 AC048382.2 GOLGA8H CLCN5 LOC101929174 ZNF844 SRPK2 AL691482.2 AC008764.1 LOC124900778 LOC100506274 MOB1AP2 E2F6P2 AC092902.5 TNFAIP8 ENSG00000287998 ANKRD50 AL049833.3 TNN OXR1-AS1 SOX21-AS1 BANP PADI2 LNX2 AC007656.1 SYCP2 TMEM163 RAVER2 POU2F3 AC120036.4 CSN1S1 RGS6 PCNX4-DT SRGAP3 AC093895.1 ZNF525 LINC00367 GXYLT1 BRPF3 LDHBP3 RIMS2 TMEM258 PLD3 ENSG00000286085 SLC5A1 AL670729.1 LINC02241 LOC105370802

		CDCP1 AC023590.1 SIK3 IGHV3OR16-12 CHRNA9 TOPAZ1 LRRC28 MORN2 BCL11A PDZD4 NAA25 FKBP9 PLCH1-AS1 CCDC92 TEX30 DUX4L33 NECTIN3-AS1 RANBP2 AC019117.2 AC068112.1 CCDC73 FAM110B IGLVV-66 CLDN18 RNF128 AL049869.2 LRRC3B LINC02885 NDRG3 AC019226.2 RUBCN C12orf75-AS1 GALNTL5 HCCS-DT AL731661.2 MIR1304 AL138759.1 ELOA-AS1 RN7SL545P
4C increased	2527	*SLC18A1 RIT2 SLMAP OR2AF1P KLHL13 ABCB7 AL355499.1 PBX4 ACOT12 NHLRC3 LOC124903770 ENO1P2 OR11P1P MYO9B GPSM2 LINC00683 COL4A5 LINC01708 ERG ENSG00000293384 ENSG00000291325 PARN CYTH1 ENSG00000286717 AL390816.1 CHD9 STK16 XK SEMA4D MMS22L MET MAX TNS3 PDE4DIPP4 SCYL3 TEAD1 ENSG00000286637 LOC112268276 ATXN10 KIAA1210 BRCA1 LOC105370954 EIF4G3 ACSM3 AC231532.2 CNBD2 BAZ1B SNX16 AC116035.1 WWC3-AS1 LINC02476 LINC02487 FKBP14-AS1 FGGY LINC01915 SPAG11B IFTAP EOLA2 CEP350 LINC00261 SUMO1P2 ZNG1F LINC01944 LINC02997 STAU2 RN7SL275P SIAH3 LINC01121 AC034154.1 DUS3L PIWIL3 CETN3 AC116424.1 ARL14EP-DT RPL4P1 AC108025.1 CHMP1B AC015987.1 SLC9B1P5 AC004584.3 MMP16 AL136984.1 MIR654 DRD3 OR7E25P ENSG00000286104 METTL25 AC091231.1 ELAPOR2 FAXC ATP11C PRORP ZNF534 TOX3 POLR2J4 LINC02334 DNAH11 RALY-AS1 CDC42BPA POLR2J POLG AC092957.1 EXOC2 DBIP2 PTPN5 RPS3AP26 AL358934.1 MYLK-AS1 TACR1 FSIP1 ENSG00000289332 AC131025.3 AC068672.2 SNX25 SDC2 LINC02064 AC131571.1 PDYN-AS1 ABHD12 GPHB5 AP002954.1 AC005999.1 UBN2 COX10 VN1R12P CIITA AL160153.1 FANCC AP006219.1 ARAP1 UQCRHL LINC00305 AC073488.11 HEPHL1 KLHL7 RN7SL354P CCR3 ANKLE2 GACAT3 FAM218A HBG2 CEP85 LOC101928565 RPRD1B ENSG00000287618 CR381653.1 LINC01828 CIP2A LOC105371855 AHNAK ACTR5 PPP1R3B-DT YBX1P5 ZSCAN5A-AS1 ENSG00000285996 LRRC53 EIF4A3 DLEC1 COX10-DT AC004943.3 NFE2L2 GRPEL2-AS1 AC069335.1 SCHIP1 MIR376A2 AC004594.1 SLC46A2-AS1 PARG OLFML2A UIMC1 TFEC ENSG00000286830 NFYC H2BC18 BLOC1S5-TXNDC5 OTOA UPP2 CYP19A1 LOC105374367 MLIP-AS1 SLC46A2 UBTD1 AC073488.10 LINC00313 BX640514.1 DTNB GPR158 VRK2 ITSN1 OAS1 AC128707.1 LINC02934 DEUP1 AC092552.1 R3HDM2 ATP2B2 AC073488.2 CPVL NBPF13P ESPNL PREX2 SCRN1 LINC01374 AC007314.1 FAM177A1 ELAVL2 AC073488.5 MAP9-AS1 PSKH2 ENSG00000286332 AC135507.1 NAV2-AS4 CSMD3 LOC124903568 FBF1 FBXL20 NTN4 SERP1 CNTNAP3B GTF2IP1 AC090888.1 POLR1A TBX3-AS1 AC025031.1 PLEKHM3 ENKUR LOC105375146 CENPBD2P TPTE2 AC017002.6 RAB11FIP4 AC010307.2 CLMAT3 AC116353.4 C10orf53 HSFX4 NPIPB5 GRPEL2 SNTA1 PPCDC CHD1-DT RPL7AP83 MICOS10-NBL1 ENSG00000289178 ENSG00000293110 LINC02578 TTC28-AS1 CLDN10 CBX3P10 CCDC144A ASZ1 CU638689.3 RF00425 ENSG00000289699 NUB1 SRGAP2C ENSG00000293482 WIF1 MISFA FLI1 PPIEL CSNK2A2 DYM-AS1 LINC02197 SPATS2L PABPC5-AS1 CLIC5 ERICH3-AS1 ESRRB ZFHX2 ARHGAP26 TDRD9 LINC-PINT RBP7 ENSG00000290548 NR2F2-AS1 TRHDE RAB44 PTPN4 AC105180.1 LEMD1-AS1 LOC652276 LOC107985211 SLC16A11 SETBP1 ZBTB7C ITGA2 GRIN2A ENSG00000288643 AL442647.1 ENSG00000290070 KARS1P2 MIR5702 LOC285638 AC093459.1 NELL2 AC026316.5 IL1RAPL1 WDPCP AC119751.4 ENSG00000287108 AC091564.3 KIF5C BLK GPM6A SPATA16 AC068205.2 AC034195.1 AL359706.1 WNT16 RERGL KRT86 SETD3 MRPS35P2 PMS2P13 AC245517.1 CCZ1B AC011444.2 ADCY1 GRID1 RPS6KA2 RN7SL271P EFR3B PLPPR5 AVL9 LINC01790 TRIM24 RPS4XP20 ZDHHC17 AC005682.1 SMG1P5 HLCS LINC02831 CNBD1 FIG4 AC106864.2 LINC01621 EIF2AK2 AP1S3 CFAP47 AC026398.1 GSK3B-DT AP000844.2 FOXP2 LOC102724452 RNU6-389P FP325331.1 ENSG00000288016 FBXO34 ENSG00000286062 ENSG00000286069 AC025031.4 F2RL2 KMT2D GUSBP5 LPAR3 C3orf49 CKMT1B AC104116.1 SHE ANO4 GIPC2 FMR1 C5orf64 AC008825.1 LINC01393 GFOD1 WASH7P ENSG00000288106 TXLNG FOXN3 SP110 SH3BP2 SH3TC2 CEP290 FAM171B CNTNAP3P2 WDFY3 AC239859.1 KC6 LRIG3-DT SHQ1 TACC1 RAD9B SH3PXD2B OCLN AC092484.1 CFAP91 TUT4 AK8 AC024598.1 ENSG00000293339 FASTKD5 ITGBL1 NETO1 AL109935.2 PTPRQ TNPO1 GBE1 ENSG00000288620 SNX5 CDK13 AC004965.1 AC097634.4 DPY19L2P1 AL096711.2 SNX32 PCGEM1 DNPEP LOC100506321 LINC00589 AF130417.1 FAM230H NEO1 CELF2-AS2 GLT8D1 LINC02894 MYT1L RASA2 SRGAP2B STAG1 LINC02428 CLDN14 IQCJ-SCHIP1 AC004702.1 CARM1P1 GUCY1B2 LINC01968 ITPKB PDE3A-AS1 LINC01829 PLEKHB2 TIMM23 CAPN11 UBOX5 TCERG1 AC092807.3 HBE1 AC024257.1 SLC66A1 F10 SLC7A14-AS1 CKMT1A APOO LINC01323 CFAP20DC-DT LINC00970 DNPEP-AS1 SRGAP1 AC024581.1 LINC00370 SCN1A-AS1 LINC03099 DIO2 FAM184A ARHGEF35-AS1 AC073325.1 CABIN1 SYNE1 PSMD10 FBXO47 KRT89P CCNO-DT FBXL17 PDZD2 SOX1-OT AC106798.1 EOLA1 AC104389.4 RPS3AP5 SLC9A4 DHX29 GAS2 STPG2 AP004833.1 LINC01678 GPAM NABP1 MSRB3-AS1 IRF2 HEATR6 GET1P1 RUNX2 SPATA6L LRP12 TCF12-DT ENSG00000289842 WEE2-AS1 AC093766.1 LOC100287944 ENSG00000291120 AL353133.2 TSPEAR TBC1D22A FILIP1L LINC01492 LRRC1 ESRP1 HSFX3 AL589666.1 HAND2-AS1 NPIPB4 UBE2A BRMS1L RBM41 GOLGA6L1 AC099329.2 AC011477.4 RGS12 LNCOG PPP6R3 AC245102.2 AC008133.1 LINC02963*

		CCDC157 ACOXL AC007529.2 OSBP2 CBX3 LINC02955 ACAP2 SLC39A10 CDCA2 KCNV1 PRKG1-AS1 AC020687.1 NBL1 ENSG00000289788 ZNF496 KCTD8 ZNF550 CHCHD6 KIZ MPHOSPH9 LINC00607 LOC102724289 RNF10 LINC02181 MGC4859 AC084357.2 ENSG00000287051 FAT1 AL137220.1 SLC2A2 ENSG00000291047 PIK3R1 AL078621.3 STAMBPL1 AC139143.1 IL17B LOC101928253 AGMO ABCB11 SARNP CFDP1 PGM5 COL26A1 LINC01259 SHTN1 ATG12 FNDC3B AC073488.4 MASP1 AC034114.2 LOC100419716 PMS2P2 TMEM232 AC104365.1 USP50 CD200R1 LINC00587 BTD CALD1 STPG2-AS1 POLR3F CCDC144NL-AS1 AC068725.1 SH3GL2 ZNF847P CLBA1 HIBADH ENSG00000288577 LINC02233 CRTC1 VCF1 GSE1 FHIP1A LINC01611 NBPF14 N4BP2L1 PITPNC1 DARS1-AS1 MYLK3 ENSG00000291178 SLC30A3 LINC00663 NXPE3 AC091046.1 AL137009.1 DOCK7 ENSG00000286353 AC106706.1 PDK3 PTCSC2 MTX2 XKR4 THUMPD3-AS1 FBXL4 ROR1 AC079362.1 CAP2P1 SMAD3-DT RAPGEF4-AS1 TMEM183BP SCAP ADAMTSL1 VENTXP1 AC024382.1 PAX5 RTL8B AC090023.2 AAK1 LINC02755 CRB1 LINC02436 CTTN UTP15 RNU6ATAC36P PASK USP8 AC026826.2 GPR75 AC116353.5 ARID1A PARD6B THEMIS LINC00404 PLPPR5-AS1 LOC124900945 SMAD6 OR2AJ1 FAM230B RYBP NYAP2 PSD3 CLDN10-AS1 ABCA13 AL355922.4 RFX2 AC012368.1 ACY3 MEF2C ZNF519 AP002856.2 FZR1 BAIAP2L1 ST8SIA5 TBC1D19 DBNL PPP1R9A-AS1 SRGAP2-AS1 AC037486.1 AC235565.2 MRPS36P3 ENSG00000287877 ELP4 RMDN2-AS1 RYK MACF1 AP2B1P1 PXT1 TAF4B AC087639.2 MALT1 STARD4-AS1 DENND11 AC079466.2 TUBGCP3 ITIH5 WEE2 IQCK MTM1 SLC15A5 TFDP2 ABCB10P4 CHMP4BP1 LINC00384 MRPS10P2 AC110296.1 KCNE1 NDUFAF4P3 LINC02930 PTPRE UNC5B ZSCAN5A ENSG00000289205 MFSD6 SERINC3 AL035401.1 TBC1D31 ZNF347 PPIAP67 PTPN12 CRYZL1 PTPRZ1 RNA5SP232 LINC01749 KAZN-AS1 AC105450.1 ACACA AC026786.2 CD55 PLN EML1 DDX39BP1 CTDSPL2 SHISA3 RPS3AP6 SLC66A3 SLC16A7 ALDH7A1P2 NEDD4 OCLNP1 NEK4 RBBP9 CACNA1E ENSG00000289849 AC073488.9 FAM230G ENSG00000287526 NF1P2 ENSG00000293315 LOC105375297 AC024559.1 LINC01203 FLYWCH1 LRRC38 DOCK8 HMGB1P5 OVAAL AC078777.1 CYSLTR2 VWA8 DDAH1 PARVA LINC02154 PPP1R8 LRRC37A2 AC106729.1 AC002064.1 SECISBP2 LINC02237 URI1 LOC127903862 MARK3P1 SLC9C1 SCML2 ANKRD28 AL031847.2 LINC01809 CHD6 FGD4 SSX11P PDXK PDE4DIPP2 ZNF609 PRDX1 NDUFAF6 PMS2P6 COX17 PACS2 HACE1 SNRPF NUDT16-DT AL121782.1 ATG4B SCD5 KTN1-AS1 AEBP2 SLC7A14 AL133255.1 DSC2 RBMS2 TRG-AS1 NPSR1-AS1 SERPINA1 GLB1L LOC100132172 MYO10 KRT127P LINC02693 LINC00376 OLFM1 CRHR2 RN7SL77P MID1 AC020897.1 POGK TSHZ2 ENSG00000289376 LINC00862 SLC12A2 OTX1 EFNB3 TRIM37 LOC101926964 ENSG00000286432 LINC02775 LINC02458 DLG3 RNU6-687P FAR2 SSR3 AC119751.1 TRAT1 LOC105376219 ENSG00000291284 ERHP2 PROSER1 FLOT2 PVRIG SRP68 SV2B FAM230A LINC01879 AC068987.2 AL445070.1 ILK LEF1 LINC02552 EYA1 DAAM2-AS1 ZNF816-ZNF321P MAPK14 PRR14L MYBPC3 WDR17 AC011499.1 ENSG00000290921 ZFHX4 RNF216P1 SLC25A13 ENSG00000290578 RAPGEF1 ALG13 ENSG00000287776 EFCAB14 AC068633.1 GPRC6A AC009139.2 EOLA2-DT UNC79 LOC100129616 SH3GL3 AKR7A2 RNU1-55P ZNF516 CERKL ENSG00000286728 SLC1A2 HOMER2P1 ZEB1-AS1 AC027228.2 AL450442.1 EIF2S3B ARHGEF3 GABRB2 BTBD9-AS1 EDNRB LOC101927293 AC010196.1 LINC01938 GRIN2B UBXN10 SPDYE3 SVOP ARSB GPR141 CRISPLD1 AC106895.2 NOP16 IFNGR2 ROR1-AS1 DENND4A TBC1D20 AC083939.1 AL445218.1 GOLGA6L2 PLD5 RABGAP1 OXR1 AC245748.2 LINC00700 SFMBT2 SOWAHCP2 SETD5 SNX2 LOC729732 GRHPR AL929601.1 SCAMP5 IL17RB DCAF7 PARP2 MINDY3 ZSWIM6 MYL1 KLHL4 AC006041.1 TTC3-AS1 LINC03096 OVOL2 SMPX EIF3F RDH16 MIR376B ENSG00000286980 FCHSD2 SLC4A7 CFAP418-AS1 MTND3P22 RNU6-1117P ENSG00000290808 LNCTSI DNAJB6 F10-AS1 LINC02612 SNRPGP9 MTCL3 LINC02279 AC023442.3 MIGA1 RPL23AP7 NPIPB3 RNU6-581P ENSG00000289530 OMA1 ENSG00000290967 LINC02683 ZNF724 SPCS1 PRR4 MTUS1 GABRA6 LEMD3 PLEKHF2 KHDRBS2 B3GLCT PIEZO1 SOAT1 BRD10 OR2T11 GGT4P SPTLC1P2 SLC9B2 ENSG00000287042 NEB AC103719.1 AL157359.2 PTAR1 AC090888.3 CSRNP3 AC087762.1 KANSL1L AL157944.1 MRGPRX1 KLHL32 AC018618.1 TMEM100 LINC00861 LINC02253 FAM120C LINC01588 LYPD6 PDIA5 LOC105370409 AL606517.2 PMS2P8 XPR1 VDR LOC127814297 ENSG00000287603 SPESP1 AC097625.1 CCDC149 AC087636.1 NEK11 PDPN CREB3L2 RTN4 ENSG00000286962 AC096887.1 GNL3L CACNA1D ARHGEF11 PRKAA2 IVNS1ABP CD163 SGPP2 DPT BBS2 CTNNA2-AS1 CLSTN1 SPAG17 RPL17P38 TMEM150C ZNF155 SERGEF SCYL2 AC090912.1 AOPEP SKAP1 AC190387.1 NT5C3A CU634019.5 AC108749.1 KRT18P35 LINC02375 MAOB AC022816.1 AC026415.1 LINC00395 SKIC3 MRPL3 SERPINE2 MYO3A ZNG1E LOC101928866 MICOS10 NUBPL LOC105377146 PLAAT3 GGNBP1 POGLUT2 AC129915.3 AC132153.1 IL12RB2 LINC01221 PSG2 LINC00363 LOC100507336 PIK3CG RPS3AP25 ANOS1 COL4A4 TPP1 LOC100130691 TBC1D30 ANKFN1 STIL TFDP1 TRGV5 IL1R2 ATP6V1E2 TNPO3 NBPF10 HTT AL935212.2 NUP160 FGF14-IT1 MCCD1P1 AC132803.1 AC137810.1 CDHR17P NECTIN3 AC016152.1 FREM3 AL136084.3 AC121757.1 ABCB10P3 IQGAP1 MYCBP2 WIPI1 MIR6841 LATS1

		ENSG00000286875 AC098850.3 NUP153-AS1 ZSCAN25 ANKRD20A21P LINC01442 AC007881.2 LOC124903099 ENSG00000293441 KLHL12 ENSG00000286215 LOC349160 ENSG00000286097 SNX30 AC007092.1 AC007277.1 GNPDA2 TSNARE1 LINC00942 FANCI LINC00342 THNSL1 OR9Q1 ENSG00000290589 ACAA1 OR4K17 SPARCL1 CPEB2-DT ITGA9-AS1 PRSS23 WNT2B CPHL1P LRRC37A3 TMDD1 ENSG00000287299 SPNS3 EXD3 MTCO3P42 H2AZ1-DT TNFRSF10B IUR1 MAP2 RNU6-973P AC023078.5 GRM5P1 ENSG00000286982 NPIPB13 ENSG00000287771 FBXW11 MTCL1 PRDX6-AS1 LINC01392 PTPRO ZFAND3 HSPH1 ENSG00000286248 AC008268.1 SUGP2 AZIN2 LINC01029 IYD AC108517.1 AC026992.1 SPATA21 MIR518C SLC29A4P1 HNRNPA1P36 MIR646HG NHSL1 SMG6 ENSG00000287523 HLA-DMA AC093802.1 CEACAM16-AS1 AC010343.3 TCERG1P2 SNORD17 LOC105370500 ENSG00000286041 RPS4XP23 PEMT ARHGAP6 ENSG00000289503 LINC01845 LOC100420057 LINC00927 POLR2J2 PPP2R2B AL356010.2 CHD5 LINC02196 JAK1 GPD2 IL12A-AS1 ITGA9 ACSL6 AC130448.2 TOR1AIP2 ULK2 KCNB1 RADIL AC015909.5 CANX ZMIZ1 UGP2 LINC00298 TRIM39-RPP21 CES5A AC034228.3 RP1L1 AC084200.1 AL109763.1 AC105919.1 GNPAT HSD17B12 HS6ST3 GRM4 ANKRD27 LOC100887080 AIM2 ANK3 LINC01283 LINC00911 RF02271 GTF2IRD1 LINC00578 ZNF19 TEX21P TMEM132B PPP1R14C MARK2P11 AL391095.1 AMT ENPP7P10 KITLG SV2C ARID4B SATL1 GMDS CSAG2 ZNF326 NDUFS5P5 MAP4K4 CNIH3 PLEKHM2 NR1I2 THSD4 ZNF266 NXPE1 AC106745.1 PENK-AS1 ENSG00000286512 LINC01471 ENSG00000286371 LINC01414 AC073488.8 HIP1 RAET1E AC004852.2 AC068313.1 RFX7 RELN MFHAS1 OR51B5 SRP14P2 HS3ST4 ENSG00000286556 AF107885.2 LRRC37A ENSG00000286239 STAG3L4 PDHB ADD3-AS1 ADAMTSL3 FBXO32 PMS2P7 NXPH1 KMT2CP1 MTMR3 AC012467.2 CRKL CSAG3 LINC00871 MYO18B TENT5A ARHGEF10 FGD6 SYTL3 NDC1 AC009093.2 SULT1C4 PTPN13 LINC02742 LINC02074 SNAP91 ZNF816 AC104452.1 VAV3 RPS6KC1 INPP5A CD40LG VRK1 AC100802.1 ENSG00000287950 PAPOLB URM1 LINC03041 GPAT3 ENSG00000287862 SCN9A ZNF420 DPYSL3 AC073488.3 C1orf146 ATP2B1 AC009498.1 SPRING1 AL807742.1 SLC16A10 PLCE1-AS2 LINC01252 SLC28A2-AS1 ATRN NDUFB9 CCDC88A SNORA36C SLC35B4 ARNT2 UGT3A2 RNGTT RPS20P32 APBA1 PITX1-AS1 HPSE2 PLCE1 AC007262.2 TNIP3 FAM106A TRMT2B-AS1 ENSG00000288799 TACC2 AC073488.6 AC009950.1 NAALAD2 NT5DC1 CYP39A1 AC099511.1 AC006927.5 AC022568.1 DCAF5 LINC01423 GNAL LINC01202 AP004607.6 CSTF1 PEBP4 STK32C AC020611.2 AC090114.3 MIR7110 OR7A1P MSH3 RNU6-10P UBE2F-SCLY MINDY1 LINC02664 BAK1 MRPL45 CASTOR2 LRRC37A4P AC034268.2 DIS3L2P1 LAX1 AC106894.1 AC013644.1 LINC00705 PTPRG-AS1 CACTIN STK26 CLCC1 AC005008.2 AC002428.1 MIR520C LINC03020 ENOX2 TENM4 AC068152.1 GOLGA6L25 TTC9-DT LOC102723446 DCP1A RPS12P20 ZCRB1 PPP3CA CHRNA5 CES2 MIR526A1 AC133065.1 AC093799.1 DUX4 MKNK1 DYSF NKD1 AL157762.1 ENSG00000286856 ENSG00000287849 RBPJ KMT2CP3 AL356807.1 GTF2IP4 CYRIB SPDYE17 ENSG00000287320 C3 RIN2 PDE6C AL137247.1 AC016629.1 PLEKHG7 DLX6-AS1 KBTBD3 ASCC1 CDH10 DENND1A MZT1 RPL13AP17 ENSG00000290317 PDZRN3 LINC02068 OR52D1 ADAMTS16 TASP1 LINC00381 NBPF19 AC008700.1 MICU1 EBPL TAF10 MMP24OS ENSG00000287329 TRIP12 C3orf52 MBD5 BACE2 KDM5A AC009154.1 ZNF549 AC010328.3 AP002761.2 ZNF747-DT AC004875.1 XAGE1A LINC02026 LOC102724354 LOC101927855 FCGBP NOMO1 AL133375.1 ARL6IP6 LINC01579 ZNF320 CNOT6 SDK1-AS1 PKIB AC007091.1 SPANXA2-OT1 ENSG00000293024 GALNT18 LINC02284 ASAH2 TBX2-AS1 FRYL LINC01145 TBC1D9B LINC03019 STON1 RPL21P3 PXMP2 ASH1L RSPO2 LINC02266 TPMT AC010636.2 AC016598.2 ZNF747 ZNF805 ACSF2 ENSG00000288632 OR5K1 OR13C9 DOCK8-AS2 DISC1 PROX1 BMP6 MAP3K7CL FMN1 ZNF891 RALGPS1 CCDC6 PAQR5-DT GUSBP1 MUC5B CTSB AC026474.1 AL133353.1 LINC00613 DNAJC11 ASAP1 Z96074.1 LRRC2 VSIG1 CU633906.3 LRRC42 AQP8 CREBBP RPS10-NUDT3 ITGB1 AP000317.1 BVES RBM26 AC134698.4 AP003108.3 AC022031.2 RNA5SP29 CYB561 AC010632.3 DRC7 SLC25A36P1 ZNF827 AL109837.2 UBA5P1 AC093274.1 LMAN2L LINC00240 DSE LINC02346 NFKBIZ LINC01823 ENSG00000291175 RHCE CORO1C KANK3 ENSG00000287247 NSG2 GPATCH11 LHFPL6 SPIDR CCDST AP001180.5 C17orf80 NEFL NAV2 ATP6V1A TMEM185B IMPA1P1 STK3 CHN2 RNF150 NBEAP3 FCAR AC119751.7 FSIP2-AS1 C1GALT1 HNF4G SMCO4 MSR1 EXOSC3 AP2B1 MED15P7 GABRG3 AC011453.1 ENSG00000290790 PSMD7-DT AL138752.2 ENSG00000293093 LINC03095 DPY19L1P1 AC245060.5 CR936218.1 SH2D3C APTX AC007000.3 TRPC4 RAMP3 MTCO3P39 GNG2 ZNF705A FRS3 ENSG00000289336 PCDH11X CPB1 XYLT2 MIR222HG LIMS1 LINC01456 ENPP2 DOCK4 ACKR2 LINC01900 ARPC1A UBL4B LINC00382 MCTP2 ENSG00000286458 BCL11B CERS3 GLCE PLS3 AC062021.1 PCSK2 TSPAN2 KCNIP1 PWWP2A LOC105373170 SLC29A4P2 LRRTM4-AS1 AC073283.3 ZFPM2-AS1 MIR7851 GFRA2 TBC1D10A ENSG00000287188 EPHB1 SSBP2 CASP8AP2 AC007422.2 REEP1 RPH3A ENSG00000287014 CTTNBP2 SLCO6A1 LINC01088 TULP2 NME9 CYTH4 NAP1L1 SLC38A6 LINC03109 AL121594.1 HDAC2-AS2 VPS29 PLPP4 CCDC102B AC132219.1 LINC01985 SNHG26 COPB2 UBR1 RTCA-AS1 AC005972.3 CNN2 COL4A2

		SLC1A6 TCF7L1 KMT2CP2 ARHGAP32 PAGE1 MRPS10 DYRK3 IP6K2 AC114501.2 KRTAP10-12 AC069444.2 LNC-LBCS CNTLN CHRNA6 AP000688.3 CMSS1 AP002765.1 ENSG00000288960 LINC02929 AC002428.2 ENSG00000287478 TLN2 HDAC4 ZNF582 MIR376A1 GDNF KLHL1 F11-AS1 LINC01276 FYB1 TSKS RIOK3P1 AL935212.1 CALCB MARCHF5 RBM45 SEM1 AC073488.1 GYS2 IQCE LVRN MYLK SPRY4-AS1 UNC5C CFAP69 ACSBG1 MINDY4 LINC03051 CSGALNACT1 CSMD2 TLR7 FLG2 IGHV3-62 PABIR3 SLC37A2 P3H2 AC064859.1 CFL1 BAZ2B LARS2-AS1 PPIAP76 ENSG00000288067 LINC01793 AC006441.4 DTYMK KRT20 WDR7 ENSG00000290821 DIAPH2-AS1 SLAIN2 HMG20A CEP85L TUSC3 COPG2 SNX18P5 PLEKHG4B ZFHX2-AS1 SYNCRIP CCDC38 RBBP8 AC090517.4 MED13 RNASEH2B PHF2 TAAR2 RAI2 AFDN ENSG00000288591 CAPS2 DEPDC1-AS1 CYTIP LRP2 MTDH ENSG00000290606 ENSG00000287918 ENSG00000288804 SEMA6D GOLGA6L6 LIG1 EHMT1 ZDHHC13 WWC3 LINC02715 IL17RA LINC02069 SERPINA9 CAMK4 GALNT14 CLPX AC026410.1 RASA4EP AC009084.1 VWC2 LINC01170 REPS1 FBXL7 RN7SL25P RPA3 TOMM40P2 RNU6-1229P TMEM108 RPL29P26 AC069209.1 CC2D2B CLUHP10 TPTE BFSP1 CHMP1B-AS1 DOCK7-DT LCP2 MB21D2 GAB3 SEZ6L PCYOX1L AC005828.4 SLC39A8 TMEM123 AC002350.1 AC092675.1 GLI2 AFG2B MANCR XAGE1B AL122018.1 AC009242.1 TAS2R13 AC233702.7 U2SURPP1 LINC00412 MAP4K3-DT TGFBR3 MAP3K7 UPRT GTF2I ATXN1 TMEM59L ADAM17 LINC00968 FAM78B ENSG00000286185 MEI1 SEMA4F AL133353.2 LIPJ ELAVL1 S100PBP ZFHX3-AS1 TRAK1 AC004922.1 STXBP1 OR6N1 AL353072.2 ENSG00000287923 AC008696.2 IDH1 NUAK2 AL109914.1 TIMM23B FP236315.1 LMOD2 LINC00424 AC092329.1 ENSG00000289846 LOC112268173 TSNAX-DISC1 MIR4713HG ABCC1 LINC01958 HDGFL2 MUC13 NUP205 AC074131.1 PLEKHG1 RBAK-RBAKDN NHLH1 CLDN14-AS1 AP005203.1 ELP2 SLCO5A1 NHSL2 LINC02401 RPSAP37 BX323845.2 AC021443.1 SNRNP200 AL354676.1 LINC01723 ORC4 AC135586.2 AC022031.1 AC021231.1 LINC01204 CYP8B1 ENSG00000286500 ZNF350-AS1 APC PEX5L-AS1 GNA12 LINC01680 LINC00540 AC090371.2 TTLL5 IL18R1 AL513325.1 INO80 NAA11 C8orf74 LOC728554 MOCS2 ERGIC2 SULT2B1 SPTSSB PEAR1 CDA LINC01591 AC096711.3 BANCR PPARGC1B STON1-GTF2A1L CTRB1 COL27A1 AC093523.1 PHLDB2 BMS1P17 ESR2 AC078962.2 AC239727.1 AC005225.3 AL359237.1 AC124254.1 AC091551.1 AC108081.1 NOL10 AC092650.1 AC068759.1 ENSG00000286208 LNX1 MIR376C AC087386.1 LOC105374191 ENSG00000287694 ERC1 SMIM13 AF228730.5 MIR8058 PARM1-AS1 ZNF440 ROS1 RUBCNL AGO3 UBOX5-AS1 C9 PVR CYYR1-AS1 CASTOR3P CKAP4 TMPRSS11A LINC01837 ENSG00000293001 MOB3B RPL39L ACER1 IQGAP2 CDYL RLF AC087289.4 CLPB ENSG00000290507 FAM230E AC092645.1 CACTIN-AS1 LINC00326 SPINK1 TNPO1-DT ANKRA2 C12orf42 AC026719.1 TAF5L AP000265.1 JPT1 LINC02841 CTNND2 CU633904.2 DNAH14 FRMD3 LARRPM AC010197.2 ERI1 DELEC1 RNF216-IT1 RASSF3 UGGT1 PACSIN2 RDH13 TIA1 AC127029.3 ENSG00000293401 RPL36AP47 MIR181A1HG DPYSL2 NEK2P4 ENSG00000288700 ENSG00000287169 SYNPO2 NUP210L AC008574.1 CNTNAP3C AL049775.2 GIMAP8 RAB8B AC017091.1 METTL8 AL023574.1 NSD2 ZNF516-DT ASCC3 ACYP2 GRM8 SLC23A2 PHACTR3 RMDN2 AL139352.1 LOC124900206 AC107023.1 EIF3J-DT AC005261.3 PAIP2 ENSG00000291209 AARS1 KCND1 PTPN11 ENSG00000286499 TMEM45A TMEM267 KIAA1958 JADE3 PPIAP73 CARD18 PHF3 ENSG00000287352 PSMC2 ENSG00000287831 MAPKAP1 LINC01853 COL15A1 EXOC6B LINC01950 EVC2 GON4L LINC03017 RIC1 ENSG00000293214 AC007179.2 AC007780.1 ENSG00000291262 ENSG00000286812 AC104031.1 LINC02109 ENSG00000287362 MGAT5 AC104304.1 ACOX1 IGHV3-42 USP24 HAO1 MAEL NUP153 CLPTM1 LINC01581 FBXO38-DT HSD17B4 EFHD1 LCN12 RNF215 AL500522.1 TRMT10B TRAV32 COL21A1 UFL1-AS1 RAI14 AL691403.1 ENSG00000292277 LOC285626 GPATCH2L PIGR ENSG00000286286 NRP2 GTF3A LOC101928386 SIPA1L2 CAVIN1 MIR548AD GYPE MSL3-DT AC006946.3 SLC25A24P1 SNORD30 PLXNC1 KCTD5 C1orf21 METTL15 TMEM165 AC008537.1 AC016745.2 LINC01229 FOXJ2 RNU4-69P CEP162 HECTD4 ELOVL7 AC092111.3 NR3C1 DLX6 MIR300 AC068944.1 KMT2CP5 BORCS5 AC011466.2 FSTL4 ZC3HAV1 ABCB10P1 ARHGAP28 PGM5-AS1 MTOR LINC01797 AF212831.1 FAM230J ENSG00000287907 TRAV5 NBPF20 KSR1 CBLB LINC02217 FAF1 AC006116.9 SNAP25 TUFT1 AC004691.1 RP1 PGM5P4-AS1 DEFB124 SGSM1 ARL10 ADH7 TIMM23B-AGAP6 MFSD4B-DT AC011476.2 CHST4 CCDC144BP AC005050.1 OR11H6 ENSG00000287258 AC083795.2 ENSG00000290565 GOLGA8A ZNF221 CLDND1 PIP5K1A BTBD2 GH2 MMACHC AC034234.1 AC131025.1 DISP1 RAB6B HS6ST2 AC008539.1 KYNU AC015468.3 LOC105370906 SMN2 ENSG00000288894 ADAL AC090193.2 TBXAS1 ASCC2 AC046136.1 SPRED1 ENSG00000289744 ADAM10 GALC GNG4 CNTNAP4 NUDT19P4 WASHC1 TUBA3D AL445928.2 CDH26 CBY2 SZRD1 FAM53C ENSG00000293242 RNF32-DT SNX19P4 PDS5B CRIM1 ENSG00000289487 ENSG00000291233 NALF2 MEI4 RBM46 SUGP1 PFKFB3 SPANXD ABCB5 POTEA LOC105369292 RNF115 TRIM61 DPY19L2 ENSG00000287544 CYYR1 ESD CYP2G1P CDHR3 LINC00689 PER2 AL139300.1 ERICH3 LINC01076 PLEKHH2 ANKRD24 GLI3 IPO5P1 LHPP KAT6A BCOR KCNK12 NTRK3 RXFP1 LINC02932 SLC35F3 LINC01493

		ENSG00000287729 LINC01807 TADA1 GAB2 STAG3L3 LINC02821 DIS3L2 ENSG00000290702 RAET1E-AS1 CTCF SNTB2 ENSG00000287291 ENSG00000286954 AC073263.1 PDE1A RSBN1L PDCD10 ENSG00000286584 LOC107985643 PRAMEF11 TFG EPHX2 RPS3AP21 LMTK2 AC023055.1 AL133268.3 HHAT UBE4A LINC00632 NWD2 IDH3A CREB5 PSMA3P1 FAM227B CCDC68 ADGRG6 SLC4A10 COPG2IT1 GOLGA7 AL357153.3 FERMT2 AC245519.1 GCNA GPR82 AC068587.3 AC130710.1 LOC124906209 AC008056.2 TSPOAP1 GLUD1 DNM3 AC016885.1 ST3GAL2 SYT1 SREK1IP1 PHIP EPB41 DENND5A LINC00309 SS18 LOC124900792 SFRP5 DOCK9 RPL23AP38 ADAM6 GOLGA6L24 AGBL3 ZYG11B RFC2 PPARA NCKAP5 CD244 AP000311.1 LINC01643 LINC01933 RN7SKP173 SHISA6 IGLV3-1 NFASC AC122134.1 OR2A1-AS1 HDX SCPEP1 WEE1P2 SLC28A2 AC094108.1 MAP2K5 RAI1 AC104339.1 ZNF229 LINC00513 GORAB-AS1 NXPE4 PHF6 HMGB4 EPB41L2 CADM3 ENSG00000288075 ENSG00000287533 FGF7 TENT5D MARCHF10 TBCA PACSIN1 ENSG00000287290 STXBP6 MGA LINC02549 DENND2B-AS1 AL137782.1 FAM230D FOXP4-AS1 AC021660.2 RTTN CACNA1A AC091544.5 AL596087.2 RN7SKP284 AC141586.1 EDA CNTRL LOC100533679 LIMCH1 TNFSF13B Z93403.1 GNPNAT1 AC006288.1 ENSG00000291170 MTMR7 CNTNAP3 PTH2R AC005909.1 ELOVL6 LOC100420587 AC125603.4 IAH1 MICAL2 COL14A1 AC073488.7 IGHV7-40 RNU2-38P SLC22A25 RBM47 AC005828.3 ENSG00000286209 EIF2A JARID2 CYP1B1-AS1 CELSR1 CYTOR HDAC2 FIRRM TPO AP003385.1 TGOLN2 FGD1 GSTA3 AC008415.1 TRIM44 GNAO1-DT INSYN2B TRIM39 AC009090.6 SEMA3D AC105213.1 NBPF26 ENSG00000286134 PROM1 ENSG00000287368 LINC01422 OR2T8 NPSR1 RPL7P15 GLIDR EIF4E3 ACP3 USP48 KCNH5 RRM1 MAGEA1 CADM1 KLHL29 INPP5D CFAP161 THUMPD1 AL356309.1 CLYBL OR4C6 SPDYE9 RN7SL797P ATF6 PRKAR1B GBF1 IPO11 IQCJ LINC02805 SERPINE1 CCDC88C ABHD15-AS1 SMN1 BACH1 CUL9 ENSG00000289228 PRAMEF4 INMT-MINDY4 EWSR1 DTX2P1-UPK3BP1-PMS2P11 TM7SF3 CYTH3 KLC1 SLC26A5 AKAP13 AC091047.1 HBS1L MLANA PKM GPAT4-AS1 GCG ARFGEF1 MRPL45P2 ATP12A AC090709.1 LINC01735 CCDC150 TKFC ENSG00000289293 ENSG00000290832 LINC02218 SMARCAD1-DT PTPN3 ENSG00000292979 SEPHS1P1 FOXP1 PMS2P10 STAC SCN8A AC010547.2 AF241728.1 AL139042.1 RALGAPA1 RTL4 NNT SSX2IP ST8SIA2 TTC17 ELP1 IRF8 RPL7L1P16 HIVEP3 LOC124904332 AC074386.1 PHF5AP1 C2orf42 AC005999.2 AC018629.1 AL357052.1 AL008638.3 HEPACAM2 KAT7P1 AC023824.1 RFX4 CCDC198 AC118282.1 OR6K6 ZNF30 ZRANB2-DT PHB1P13 REM1 ACTA2 LINC01482 AGAP1 FREM1 RIPOR2 AC091564.7 CEP192P1 LINC02899 NDUFA10 CBR1-AS1 KCNK5 ENSG00000287579 CD69 B3GALNT2 AL132671.2 FUNDC2 DBR1 EPS15L1 CYB561A3 PARD3B AC021660.3 ENSG00000291188 XIRP2 Z99571.1 AC079950.1 AL078601.2 LINC01248 KDM3A AL356272.1 ENSG00000293483 MZT2A CAST OR2L3 AL354984.2 AC005394.1 TAOK1 UNC45A KBTBD11-OT1 GPR39 ADGRA3 CASK HSPG2 MIR6744 SLCO4C1 RPS29P9 GCNT1 LINC00973 BPIFB3 AC139493.2 SLC35F4 AF121898.1 PMS2P14 GGA2 AC007731.3 AC008277.1 PRDM7 USH1C FP236315.3 FMN2 AC118758.3 LOC101927468 LINC02307 UBXN2A LINC02269 AC136428.4 LINC01762 AC021188.1 NLRP14 PCNT DNAJC13 FRMD4B LOC345471 AFG2A PSME3IP1 GOLGA8B TRDV3 ENSG00000286780 ENSG00000287469 LINC01478
Plastic	398	*AC092634.2 PAX7 RALYL FP236241.1 DLEU7 EPB41L4B LRMDA PIR PRH1-PRR4 LINC03007 TRPM6 RNF103-CHMP3 ANO5 PCDHA13 ZNF385B LOC102724843 AF127577.4 GRIK2 OTX2-AS1 LINC03000 KDM4B AC090809.1 ZBTB20 LINC00486 LOC107984685 FSTL5 LOC102724019 TTC3 CADPS2 FAM9B MKLN1 AC009262.1 NEGR1 BTBD9 FRG1-DT PCDHA12 AC069228.1 MAST4 NRXN1 AC079801.1 AGBL1 MAGI1 AC036214.3 FARS2 ST6GALNAC3 DPP6 FAM13A AL365295.1 LMO7 GRM3 LINC02822 DNAH7 TAFA1 BBS9 ENSG00000290711 NTRK2 ANXA10 ANKRD34C-AS1 NBEA AL512598.2 RASGEF1B TMX3 LINC00273 MIR99AHG ENSG00000293415 NRG3 PTPRG NCOA1 LINC01684 AL390957.1 ATP2B4 PHEX SOX6 PDE4DIP ENSG00000287474 TRPM3 FBLN7 TMEM64 DNAH8 PRANCR FGF13 DRAIC PUDP PATJ LINC01798 GPC3 DMD ADCY2 LINC02566 LINC02966 ASTN1 ADAMTS9-AS2 GNG12-AS1 ATRNL1 CDIN1 ARHGAP24 LINC00907 SLIT2 FAM20C ENSG00000291181 AC010235.1 LINC00347 AC098588.2 NALF1 KAZN ENSG00000289084 KMT2C FKBP9P1 CU638689.2 LINC01320 ASMER1 B3GNT5 ERBB4 KIF26B GPHN TNIK SH2D1A MYO3B SH3RF3 AL138720.1 STAG2 SPAG16 DSCAML1 HTR2C GALNT13 CNTN4 RAP1GAP2 LINC01695 LOC105373436 ITGA1 TCF12 SGIP1 ESRRG AL161757.2 PCDHA2 FRG1HP PCDHA11 PRH1 ENSG00000290357 SLC38A4-AS1 CDH18 ANK2 LOC101927314 MIR100HG PCDHA9 SUPT3H CD226 NEK1 DGKB OBI1-AS1 PRRC2C PCDHAC2 CDH6 AC109830.1 ENSG00000290397 RERG-AS1 WDR64 JAZF1 BNIP3P41 CHODL ZNF267 LINC01692 FGF14 SLC8A1-AS1 PCDHA10 EDIL3 SOX5 DSCAM LINC01151 COL28A1 DENND1B COLEC10 LINC02694 LINC02328 AC008035.1 TMEM260 IQSEC1 ANK1 CASZ1 CNTNAP2 TRIM71 ENSG00000286523 DYNC1I1 OTOGL DOP1A C21orf62-AS1 FP700111.1 CIBAR1-DT ATP6V0D2 PCDHA1 PCDHA8 CU638689.4 ENSG00000290551 TBCK CDH12 AC098650.1 LINC01596 CBR3-AS1 RPS3AP27 DDR2 ADAM22 GPC6 MCF2L2 TRPS1 ENSG00000293394 CDH4 DPP10 CELF4 ST8SIA6*

		CNTN6 PARM1 CU633906.2 ENSG00000286745 ZNF638 MITF NSMCE2 PCSK1 MIR4435-2HG PCDHAC1 LRFN5 UTRN GPC5 AGBL4 ULK4 RBFOX1 PCDH7 AC084149.1 PPP1R9A TMEM117 RNA5-8SN5 LINC01090 SMAD9 DYNC1H1 CRPPA AC006148.1 AC007161.3 MRPS28 TRD-AS1 LINC02306 FRMPD4 EDAR PMS2P4 BRINP3 RIMBP2 FHIT AC009093.10 DNMT3A TOX PCDH15 ACE2 CHMP3 PDE4D CNTN5 PDE3A RIMS1 PCDHA7 ENSG00000287392 AC007100.1 LINC01440 TMEM178B CPLANE1 NOL4 PCDHA5 PLCB4 RNF216 PPFIA2 ENTPD1-AS1 ENOX1 WNT2 ENSG00000292991 UMAD1 MGAT4C DIRC3 ANKRD44 UXS1 GLDC PLPPR1 LINC01584 ZNF804B SGCD GRM7 NAV3 AC092343.1 AL110292.1 AC079943.2 LOC124903324 PTPRM ENSG00000286225 NRXN3 CU633906.4 CERS6 LPIN1 MACROD2 ADAM7-AS1 BACH2 AUTS2 AC092042.3 LOC102724701 ADARB2 ENSG00000288723 COMT PCDHA6 ENSG00000286110 NBAS DOCK2 SDCCAG8 SSH2 AL035078.4 LMNTD1 CDH13 LINC02343 MDGA2 AC103796.1 BDNF-AS 5_8S_rRNA DANT2 DAB1 RFTN1 PCDHA4 AC068051.1 YEATS2 CCDC26 SLC8A3 ENSG00000290983 MYO5B CADM2 TENM2 TEX13D HDAC9 KIAA1549L ZBED3-AS1 KCNB2 NBPF1 AC090376.1 LYST CADPS LINC03105 RGS7 AC067956.1 PPP2R2C CSMD1 ENSG00000286746 SCEL CTNNA3 FREM2 ENSG00000287404 RBKS DACH2 RNF220 ARHGEF37 DLG2 RORA PTPRD NPEPPS BRIP1 MIR924HG LOC107986400 NELL1 MIR4300HG PLCL1 LINC02215 GALNTL6 PXDNL SCN2A DOCK10 POT1-AS1 ADK TMEM164 GTDC1 RYR2 SLX4IP LDLRAD3 CU633904.3 FAT3 PCDHA3 AC078845.1 KLHDC10 RGS9 MPDZ AC016766.1 ROBO2 ENSG00000288891 LINC02055 RGS10 LINC00378 AC026167.1 PAX3 LOC105379109 BMPR1B DCC CTNNA2 DNER ETV6 CEP112 NRG1 PGM3 AC092167.1 DNAH5

Table S8. GO associations with biological processes (BP), molecular functions (MF), and cellular components (CC) of 398 genes that maintained stable contact with rDNA clusters. Related to Figures 4 and 5.

GO.ID	Description	padj	Genes
		MF	
GO:0005509	calcium ion binding	3.2201658032407466e-11	*PCDHA13,FSTL5,PCDHA12,NRXN1,DNAH7,ANXA10,FBLN7,SLIT2,FAM20C,PCDHA2,PCDHA11,CDH18,PCDHA9,DGKB,PCDHAC2,CDH6,PCDHA10,EDIL3,PCDHA1,PCDHA8,CDH12,CDH4,PCDHAC1,PCDH7,PCDH15,PCDHA7,PCDHA5,PLCB4,GRM7,PCDHA6,CDH13,PCDHA4,TENM2,NELL1,RYR2,FAT3,PCDHA3,DNER*
GO:0043169	cation binding	2.2197006214256457e-8	*PIR,TRPM6,PCDHA13,ZNF385B,KDM4B,ZBTB20,FSTL5,TTC3,CADPS2,PCDHA12,MAST4,NRXN1,AGBL1,LMO7,DNAH7,NTRK2,ANXA10,ATP2B4,PHEX,PDE4DIP,FBLN7,PUDP,DMD,ADCY2,SLIT2,FAM20C,KMT2C,GPHN,SH3RF3,HTR2C,GALNT13,ITGA1,ESRRG,PCDHA2,PCDHA11,CDH18,PCDHA9,NEK1,DGKB,PCDHAC2,CDH6,JAZF1,ZNF267,PCDHA10,EDIL3,CASZ1,TRIM71,PCDHA1,PCDHA8,CDH12,TRPS1,CDH4,ZNF638,NSMCE2,PCDHAC1,UTRN,AGBL4,PCDH7,SMAD9,DNMT3A,PCDH15,ACE2,CHMP3,PDE4D,PDE3A,RIMS1,PCDHA7,PCDHA5,PLCB4,RNF216,MGAT4C,GLDC,ZNF804B,GRM7,NRXN3,ADARB2,COMT,PCDHA6,CDH13,PCDHA4,SLC8A3,TENM2,TEX13D,HDAC9,CADPS,SCEL,FREM2,RBKS,RNF220,RORA,NPEPPS,BRIP1,NELL1,GALNTL6,PXDNL,ADK,RYR2,FAT3,PCDHA3,BMPR1B,DNER,PGM3*
GO:0046872	metal ion binding	8.21416450466005e-8	*PIR,TRPM6,PCDHA13,ZNF385B,KDM4B,ZBTB20,FSTL5,TTC3,CADPS2,PCDHA12,MAST4,NRXN1,AGBL1,LMO7,DNAH7,NTRK2,ANXA10,ATP2B4,PHEX,PDE4DIP,FBLN7,PUDP,DMD,ADCY2,SLIT2,FAM20C,KMT2C,GPHN,SH3RF3,GALNT13,ITGA1,ESRRG,PCDHA2,PCDHA11,CDH18,PCDHA9,NEK1,DGKB,PCDHAC2,CDH6,JAZF1,ZNF267,PCDHA10,EDIL3,CASZ1,TRIM71,PCDHA1*

			,PCDHA8,CDH12,TRPS1,CDH4,ZNF638,NSMCE2,PCDHAC1,UTRN,AGBL4,PCDH7,SMAD9,DNMT3A,PCDH15,ACE2,PDE4D,PDE3A,RIMS1,PCDHA7,PCDHA5,PLCB4,RNF216,MGAT4C,ZNF804B,GRM7,NRXN3,ADARB2,COMT,PCDHA6,CDH13,PCDHA4,SLC8A3,TENM2,TEX13D,HDAC9,CADPS,SCEL,FREM2,RBKS,RNF220,RORA,NPEPPS,BRIP1,NELL1,GALNTL6,PXDNL,ADK,RYR2,FAT3,PCDHA3,BMPR1B,DNER,PGM3
GO:0043167	ion binding	0.0000177119224463348	PIR,TRPM6,PCDHA13,ZNF385B,KDM4B,ZBTB20,FSTL5,TTC3,CADPS2,PCDHA12,MAST4,NRXN1,AGBL1,MAGI1,FARS2,LMO7,DNAH7,NTRK2,ANXA10,ATP2B4,PHEX,PDE4DIP,FBLN7,DNAH8,PUDP,DMD,ADCY2,SLIT2,FAM20C,KMT2C,ERBB4,KIF26B,GPHN,TNIK,MYO3B,SH3RF3,HTR2C,GALNT13,ITGA1,ESRRG,PCDHA2,PCDHA11,CDH18,PCDHA9,NEK1,DGKB,PCDHAC2,CDH6,JAZF1,ZNF267,PCDHA10,EDIL3,CASZ1,TRIM71,PCDHA1,PCDHA8,TBCK,CDH12,DDR2,TRPS1,CDH4,ZNF638,NSMCE2,PCDHAC1,UTRN,AGBL4,ULK4,PCDH7,SMAD9,DYNC1H1,FRMPD4,DNMT3A,PCDH15,ACE2,CHMP3,PDE4D,PDE3A,RIMS1,PCDHA7,PCDHA5,PLCB4,RNF216,MGAT4C,UXS1,GLDC,ZNF804B,GRM7,NAV3,NRXN3,ADARB2,COMT,PCDHA6,CDH13,PCDHA4,SLC8A3,MYO5B,TENM2,TEX13D,HDAC9,CADPS,SCEL,FREM2,RBKS,RNF220,RORA,NPEPPS,BRIP1,NELL1,GALNTL6,PXDNL,ADK,RYR2,FAT3,PCDHA3,BMPR1B,DNER,PGM3,DNAH5
GO:0036094	small molecule binding	0.0000315536069663333	PIR,TRPM6,PCDHA13,ZNF385B,KDM4B,ZBTB20,FSTL5,TTC3,CADPS2,PCDHA12,MAST4,NRXN1,AGBL1,MAGI1,FARS2,LMO7,DNAH7,NTRK2,ANXA10,ATP2B4,PHEX,PDE4DIP,FBLN7,DNAH8,PUDP,DMD,ADCY2,SLIT2,FAM20C,KMT2C,ERBB4,KIF26B,GPHN,TNIK,MYO3B,SH3RF3,HTR2C,GALNT13,ITGA1,ESRRG,PCDHA2,PCDHA11,CDH18,PCDHA9,NEK1,DGKB,PCDHAC2,CDH6,JAZF1,ZNF267,PCDHA10,EDIL3,COLEC10,CASZ1,TRIM71,PCDHA1,PCDHA8,TBCK,CDH12,DDR2,TRPS1,CDH4,ZNF638,NSMCE2,PCDHAC1,UTRN,AGBL4,ULK4,PCDH7,SMAD9,DYNC1H1,FRMPD4,FHIT,DNMT3A,PCDH15,ACE2,CHMP3,PDE4D,PDE3A,RIMS1,PCDHA7,PCDHA5,PLCB4,RNF216,MGAT4C,UXS1,GLDC,ZNF804B,GRM7,NAV3,NRXN3,ADARB2,COMT,PCDHA6,CDH13,PCDHA4,SLC8A3,MYO5B,TENM2,TEX13D,HDAC9,CADPS,SCEL,FREM2,RBKS,RNF220,RORA,NPEPPS,BRIP1,NELL1,GALNTL6,PXDNL,ADK,RYR2,FAT3,PCDHA3,BMPR1B,DNER,PGM3,DNAH5
GO:0050839	cell adhesion molecule binding	0.0000792467992606342	NRXN1,DMD,DSCAML1,CNTN4,ITGA1,CDH18,CD226,CDH6,EDIL3,DSCAM,CDH12,ADAM22,CDH4,CNTN6,UTRN,CNTN5,PTPRM,NRXN3,CDH13,TENM2,CTNNA3,PTPRD,CTNNA2,NRG1
GO:0008569	minus-end-directed microtubule motor activity	0.022079925384229548	DNAH7,DNAH8,DYNC1H1,DNAH5
GO:0099106	ion channel regulator	0.03782245957437657	NRXN1,DPP6,GRM3,ATP2B4,FGF13,FGF14,DPP10,PDE4D,NRXN3

	activity		
GO:0003774	cytoskeletal motor activity	0.044424597090940404	*DNAH7,DNAH8,KIF26B,MYO3B,DYNC1I1,DYNC1H1,MYO5B,DNAH5*
GO:0016247	channel regulator activity	0.04914389645405964	*NRXN1,DPP6,GRM3,ATP2B4,FGF13,FGF14,DPP10,PDE4D,NRXN3*
GO:0005509	calcium ion binding	3.2201658032407466e-11	*PCDHA13,FSTL5,PCDHA12,NRXN1,DNAH7,ANXA10,FBLN7,SLIT2,FAM20C,PCDHA2,PCDHA11,CDH18,PCDHA9,DGKB,PCDHAC2,CDH6,PCDHA10,EDIL3,PCDHA1,PCDHA8,CDH12,CDH4,PCDHAC1,PCDH7,PCDH15,PCDHA7,PCDHA5,PLCB4,GRM7,PCDHA6,CDH13,PCDHA4,TENM2,NELL1,RYR2,FAT3,PCDHA3,DNER*
GO:0043169	cation binding	2.2197006214256457e-8	*PIR,TRPM6,PCDHA13,ZNF385B,KDM4B,ZBTB20,FSTL5,TTC3,CADPS2,PCDHA12,MAST4,NRXN1,AGBL1,LMO7,DNAH7,NTRK2,ANXA10,ATP2B4,PHEX,PDE4DIP,FBLN7,PUDP,DMD,ADCY2,SLIT2,FAM20C,KMT2C,GPHN,SH3RF3,HTR2C,GALNT13,ITGA1,ESRRG,PCDHA2,PCDHA11,CDH18,PCDHA9,NEK1,DGKB,PCDHAC2,CDH6,JAZF1,ZNF267,PCDHA10,EDIL3,CASZ1,TRIM71,PCDHA1,PCDHA8,CDH12,TRPS1,CDH4,ZNF638,NSMCE2,PCDHAC1,UTRN,AGBL4,PCDH7,SMAD9,DNMT3A,PCDH15,ACE2,CHMP3,PDE4D,PDE3A,RIMS1,PCDHA7,PCDHA5,PLCB4,RNF216,MGAT4C,GLDC,ZNF804B,GRM7,NRXN3,ADARB2,COMT,PCDHA6,CDH13,PCDHA4,SLC8A3,TENM2,TEX13D,HDAC9,CADPS,SCEL,FREM2,RBKS,RNF220,RORA,NPEPPS,BRIP1,NELL1,GALNTL6,PXDNL,ADK,RYR2,FAT3,PCDHA3,BMPR1B,DNER,PGM3*
GO:0046872	metal ion binding	8.21416450466005e-8	*PIR,TRPM6,PCDHA13,ZNF385B,KDM4B,ZBTB20,FSTL5,TTC3,CADPS2,PCDHA12,MAST4,NRXN1,AGBL1,LMO7,DNAH7,NTRK2,ANXA10,ATP2B4,PHEX,PDE4DIP,FBLN7,PUDP,DMD,ADCY2,SLIT2,FAM20C,KMT2C,GPHN,SH3RF3,GALNT13,ITGA1,ESRRG,PCDHA2,PCDHA11,CDH18,PCDHA9,NEK1,DGKB,PCDHAC2,CDH6,JAZF1,ZNF267,PCDHA10,EDIL3,CASZ1,TRIM71,PCDHA1,PCDHA8,CDH12,TRPS1,CDH4,ZNF638,NSMCE2,PCDHAC1,UTRN,AGBL4,PCDH7,SMAD9,DNMT3A,PCDH15,ACE2,PDE4D,PDE3A,RIMS1,PCDHA7,PCDHA5,PLCB4,RNF216,MGAT4C,ZNF804B,GRM7,NRXN3,ADARB2,COMT,PCDHA6,CDH13,PCDHA4,SLC8A3,TENM2,TEX13D,HDAC9,CADPS,SCEL,FREM2,RBKS,RNF220,RORA,NPEPPS,BRIP1,NELL1,GALNTL6,PXDNL,ADK,RYR2,FAT3,PCDHA3,BMPR1B,DNER,PGM3*
GO:0043167	ion binding	0.0000177119224463348	*PIR,TRPM6,PCDHA13,ZNF385B,KDM4B,ZBTB20,FSTL5,TTC3,CADPS2,PCDHA12,MAST4,NRXN1,AGBL1,MAGI1,FARS2,LMO7,DNAH7,NTRK2,ANXA10,ATP2B4,PHEX,PDE4DIP,FBLN7,DNAH8,PUDP,DMD,ADCY2,SLIT2,FAM20C,KMT2C,ERBB4,KIF26B,GPHN,TNIK,MYO3B,SH3RF3,HTR2C,GALNT13,ITGA1,ESRRG,PCDHA2,PCDHA11,CDH18,PCDHA9,NEK1,DGKB,PCDHAC2,CDH6,JAZF1,ZNF267,PCDHA10,EDIL3,CASZ1,TRIM71,PCDHA1,PCDHA8,TBCK,CDH12,DDR2,TRPS1,CDH4,ZNF638,NSMCE2,PCDHAC1,UTRN,AGBL4,ULK4,PCDH7,SMAD9,DYNC1H1,FRMPD4,DNMT3A,PCDH15,ACE2,CHMP3,PDE4D,PDE3A,RIMS1,PCDHA7,PCDHA5,PLCB4,RNF216,MGAT*

			4C,UXS1,GLDC,ZNF804B,GRM7,NAV3,NRXN3,ADARB2,COMT,PCDHA6,CDH13,PCDHA4,SLC8A3,MYO5B,TENM2,TEX13D,HDAC9,CADPS,SCEL,FREM2,RBKS,RNF220,RORA,NPEPPS,BRIP1,NELL1,GALNTL6,PXDNL,ADK,RYR2,FAT3,PCDHA3,BMPR1B,DNER,PGM3,DNAH5
GO:0036094	small molecule binding	0.0000315536069663333	*PIR,TRPM6,PCDHA13,ZNF385B,KDM4B,ZBTB20,FSTL5,TTC3,CADPS2,PCDHA12,MAST4,NRXN1,AGBL1,MAGI1,FARS2,LMO7,DNAH7,NTRK2,ANXA10,ATP2B4,PHEX,PDE4DIP,FBLN7,DNAH8,PUDP,DMD,ADCY2,SLIT2,FAM20C,KMT2C,ERBB4,KIF26B,GPHN,TNIK,MYO3B,SH3RF3,HTR2C,GALNT13,ITGA1,ESRRG,PCDHA2,PCDHA11,CDH18,PCDHA9,NEK1,DGKB,PCDHAC2,CDH6,JAZF1,ZNF267,PCDHA10,EDIL3,COLEC10,CASZ1,TRIM71,PCDHA1,PCDHA8,TBCK,CDH12,DDR2,TRPS1,CDH4,ZNF638,NSMCE2,PCDHAC1,UTRN,AGBL4,ULK4,PCDH7,SMAD9,DYNC1H1,FRMPD4,FHIT,DNMT3A,PCDH15,ACE2,CHMP3,PDE4D,PDE3A,RIMS1,PCDHA7,PCDHA5,PLCB4,RNF216,MGAT4C,UXS1,GLDC,ZNF804B,GRM7,NAV3,NRXN3,ADARB2,COMT,PCDHA6,CDH13,PCDHA4,SLC8A3,MYO5B,TENM2,TEX13D,HDAC9,CADPS,SCEL,FREM2,RBKS,RNF220,RORA,NPEPPS,BRIP1,NELL1,GALNTL6,PXDNL,ADK,RYR2,FAT3,PCDHA3,BMPR1B,DNER,PGM3,DNAH5*
GO:0050839	cell adhesion molecule binding	0.0000792467992606342	*NRXN1,DMD,DSCAML1,CNTN4,ITGA1,CDH18,CD226,CDH6,EDIL3,DSCAM,CDH12,ADAM22,CDH4,CNTN6,UTRN,CNTN5,PTPRM,NRXN3,CDH13,TENM2,CTNNA3,PTPRD,CTNNA2,NRG1*
GO:0008569	minus-end-directed microtubule motor activity	0.022079925384229548	*DNAH7,DNAH8,DYNC1H1,DNAH5*
GO:0099106	ion channel regulator activity	0.03782245957437657	*NRXN1,DPP6,GRM3,ATP2B4,FGF13,FGF14,DPP10,PDE4D,NRXN3*
GO:0003774	cytoskeletal motor activity	0.044424597090940404	*DNAH7,DNAH8,KIF26B,MYO3B,DYNC1I1,DYNC1H1,MYO5B,DNAH5*
GO:0016247	channel regulator activity	0.04914389645405964	*NRXN1,DPP6,GRM3,ATP2B4,FGF13,FGF14,DPP10,PDE4D,NRXN3*
		BP	
GO:0007399	nervous system development	2.0183874061463716e-24	*PCDHA13,KDM4B,NEGR1,PCDHA12,NRXN1,TAFA1,NTRK2,NRG3,PTPRG,NCOA1,ATP2B4,SOX6,FGF13,DMD,ASTN1,SLIT2,B3GNT5,ERBB4,TNIK,DSCAML1,CNTN4,ITGA1,TCF12,PCDHA2,PCDHA11,ANK2,PCDHA9,PCDHAC2,CHODL,FGF14,PCDHA10,SOX5,DSCAM,IQSEC1,CASZ1,CNTNAP2,TRIM71,PCDHA1,PCDHA8,DDR2,ADAM22,CDH4,CNTN6,PCSK1,PCDHAC1,LRFN5,AGBL4,ULK4,RBFOX1,PPP1R9A,CRPPA,BRINP3,DNMT3A,TOX,PCDH15,CNTN5,RIMS1,PCDHA7,CPLANE1,PCDHA5,PPFIA2,WNT2,PLPPR1,GRM7,NAV3,PTPRM,NRXN3,LPIN1,MACROD2,AUTS2,COMT,PCDHA6,SDCCAG8,MDGA2,DAB1,PCDHA4,SLC8A3,TENM2,HDAC9,RNF220,RORA,PTPRD*

			,NELL1,SCN2A,DOCK10,FAT3,PCDHA3,RGS9,ROBO2,PAX3,BMPR1B,DCC,CTNNA2,DNER,ETV6,NRG1,DNAH5
GO:0007156	homophilic cell adhesion via plasma membrane adhesion molecules	1.600965725513845e-23	PCDHA13,PCDHA12,DSCAML1,CNTN4,PCDHA2,PCDHA11,CDH18,PCDHA9,PCDHAC2,CDH6,PCDHA10,DSCAM,PCDHA1,PCDHA8,CDH12,CDH4,CNTN6,PCDHAC1,PCDH7,PCDH15,PCDHA7,PCDHA5,PTPRM,PCDHA6,CDH13,PCDHA4,CADM2,FAT3,PCDHA3,ROBO2
GO:0098742	cell-cell adhesion via plasma-membrane adhesion molecules	2.2278053397801544e-22	PCDHA13,PCDHA12,NRXN1,DSCAML1,CNTN4,PCDHA2,PCDHA11,CDH18,PCDHA9,PCDHAC2,CDH6,PCDHA10,DSCAM,PCDHA1,PCDHA8,CDH12,CDH4,CNTN6,PCDHAC1,LRFN5,PCDH7,PCDH15,PCDHA7,PCDHA5,PTPRM,PCDHA6,CDH13,DAB1,PCDHA4,CADM2,TENM2,PTPRD,FAT3,PCDHA3,ROBO2
GO:0048731	system development	5.2027990005181e-18	PCDHA13,KDM4B,NEGR1,PCDHA12,NRXN1,TAFA1,NTRK2,NRG3,PTPRG,NCOA1,ATP2B4,PHEX,SOX6,FGF13,GPC3,DMD,ASTN1,ARHGAP24,SLIT2,FAM20C,B3GNT5,ERBB4,KIF26B,TNIK,DSCAML1,CNTN4,ITGA1,TCF12,PCDHA2,PCDHA11,ANK2,PCDHA9,PCDHAC2,CHODL,FGF14,PCDHA10,SOX5,DSCAM,IQSEC1,CASZ1,CNTNAP2,TRIM71,PCDHA1,PCDHA8,DDR2,ADAM22,TRPS1,CDH4,CELF4,CNTN6,MITF,PCSK1,PCDHAC1,LRFN5,AGBL4,ULK4,RBFOX1,PPP1R9A,CRPPA,EDAR,BRINP3,DNMT3A,TOX,PCDH15,CNTN5,RIMS1,PCDHA7,CPLANE1,PCDHA5,PPFIA2,WNT2,PLPPR1,SGCD,GRM7,NAV3,PTPRM,NRXN3,LPIN1,MACROD2,AUTS2,COMT,PCDHA6,SDCCAG8,CDH13,MDGA2,DAB1,PCDHA4,SLC8A3,TENM2,HDAC9,CSMD1,FREM2,RNF220,RORA,PTPRD,BRIP1,NELL1,SCN2A,DOCK10,RYR2,FAT3,PCDHA3,RGS9,ROBO2,PAX3,BMPR1B,DCC,CTNNA2,DNER,ETV6,NRG1,DNAH5
GO:0007275	multicellular organism development	1.240683744738341e-15	PCDHA13,KDM4B,NEGR1,PCDHA12,NRXN1,TAFA1,NTRK2,NRG3,PTPRG,NCOA1,ATP2B4,PHEX,SOX6,TMEM64,FGF13,GPC3,DMD,ASTN1,ARHGAP24,SLIT2,FAM20C,B3GNT5,ERBB4,KIF26B,TNIK,MYO3B,DSCAML1,CNTN4,ITGA1,TCF12,PCDHA2,PCDHA11,ANK2,PCDHA9,PCDHAC2,CHODL,FGF14,PCDHA10,SOX5,DSCAM,IQSEC1,CASZ1,CNTNAP2,TRIM71,PCDHA1,PCDHA8,DDR2,ADAM22,TRPS1,CDH4,CELF4,ST8SIA6,CNTN6,MITF,PCSK1,PCDHAC1,LRFN5,AGBL4,ULK4,RBFOX1,PPP1R9A,CRPPA,EDAR,BRINP3,DNMT3A,TOX,PCDH15,CNTN5,RIMS1,PCDHA7,CPLANE1,PCDHA5,PPFIA2,WNT2,PLPPR1,SGCD,GRM7,NAV3,PTPRM,NRXN3,LPIN1,MACROD2,AUTS2,COMT,PCDHA6,SDCCAG8,CDH13,MDGA2,DAB1,PCDHA4,YEATS2,SLC8A3,TENM2,HDAC9,CSMD1,SCEL,FREM2,DACH2,RNF220,RORA,PTPRD,BRIP1,NELL1,SCN2A,DOCK10,RYR2,FAT3,PCDHA3,RGS9,ROBO2,PAX3,BMPR1B,DCC,CTNNA2,DNER,ETV6,NRG1,DNAH5
GO:0048856	anatomical structure development	1.5845087219922929e-15	PAX7,PIR,PCDHA13,KDM4B,FAM9B,MKLN1,NEGR1,PCDHA12,NRXN1,TAFA1,NTRK2,NRG3,PTPRG,NCOA1,ATP2B4,PHEX,SOX6,TMEM64,FGF13,GPC3,DMD,ASTN1,CDIN1,ARHGAP24,SLIT2,FAM20C,KAZN,B3GNT5,ERBB4,KIF26B,TNIK,MYO3B,SPAG16,DSCAML1,CNTN4,ITGA1,TC

			F12,PCDHA2,PCDHA11,CDH18,ANK2,PCDHA9,PRRC2C,PCDHAC2,CDH6,CHODL,FGF14,PCDHA10,SOX5,DSCAM,COLEC10,IQSEC1,CASZ1,CNTNAP2,TRIM71,PCDHA1,PCDHA8,CDH12,DDR2,ADAM22,TRPS1,CDH4,CELF4,ST8SIA6,CNTN6,MITF,PCSK1,PCDHAC1,LRFN5,UTRN,AGBL4,ULK4,RBFOX1,PPP1R9A,SMAD9,CRPPA,EDAR,BRINP3,DNMT3A,TOX,PCDH15,PDE4D,CNTN5,PDE3A,RIMS1,PCDHA7,CPLANE1,PCDHA5,PPFIA2,WNT2,PLPPR1,SGCD,GRM7,NAV3,PTPRM,NRXN3,LPIN1,MACROD2,AUTS2,COMT,PCDHA6,DOCK2,SDCCAG8,CDH13,MDGA2,DAB1,PCDHA4,YEATS2,SLC8A3,TENM2,HDAC9,CSMD1,SCEL,FREM2,DACH2,RNF220,RORA,PTPRD,BRIP1,NELL1,SCN2A,DOCK10,RYR2,FAT3,PCDHA3,RGS9,ROBO2,PAX3,BMPR1B,DCC,CTNNA2,DNER,ETV6,NRG1,PGM3,DNAH5
GO:0007155	cell adhesion	1.5065437692442665e-14	*EPB41L4B,PCDHA13,MKLN1,NEGR1,PCDHA12,NRXN1,MAGI1,LMO7,FBLN7,DMD,ASTN1,KIF26B,DSCAML1,CNTN4,ITGA1,PCDHA2,PCDHA11,CDH18,PCDHA9,CD226,PCDHAC2,CDH6,PCDHA10,EDIL3,DSCAM,COL28A1,CNTNAP2,PCDHA1,PCDHA8,CDH12,DDR2,ADAM22,CDH4,CNTN6,PCDHAC1,LRFN5,UTRN,PCDH7,PCDH15,CNTN5,PCDHA7,PCDHA5,PPFIA2,PTPRM,NRXN3,PCDHA6,CDH13,DAB1,PCDHA4,CADM2,TENM2,CTNNA3,FREM2,DLG2,PTPRD,FAT3,PCDHA3,ROBO2,DCC,CTNNA2,NRG1*
GO:0032502	developmental process	2.679932771630597e-14	*PAX7,LRMDA,PIR,PCDHA13,KDM4B,FSTL5,FAM9B,MKLN1,NEGR1,PCDHA12,NRXN1,TAFA1,BBS9,NTRK2,NRG3,PTPRG,NCOA1,ATP2B4,PHEX,SOX6,TMEM64,FGF13,GPC3,DMD,ASTN1,CDIN1,ARHGAP24,SLIT2,FAM20C,KAZN,B3GNT5,ERBB4,KIF26B,TNIK,MYO3B,SPAG16,DSCAML1,HTR2C,CNTN4,ITGA1,TCF12,PCDHA2,PCDHA11,CDH18,ANK2,PCDHA9,PRRC2C,PCDHAC2,CDH6,CHODL,FGF14,PCDHA10,SOX5,DSCAM,COLEC10,IQSEC1,CASZ1,CNTNAP2,TRIM71,PCDHA1,PCDHA8,CDH12,DDR2,ADAM22,TRPS1,CDH4,CELF4,ST8SIA6,CNTN6,MITF,PCSK1,PCDHAC1,LRFN5,UTRN,AGBL4,ULK4,RBFOX1,PPP1R9A,SMAD9,CRPPA,EDAR,BRINP3,DNMT3A,TOX,PCDH15,PDE4D,CNTN5,PDE3A,RIMS1,PCDHA7,CPLANE1,PCDHA5,PPFIA2,WNT2,PLPPR1,SGCD,GRM7,NAV3,PTPRM,NRXN3,LPIN1,MACROD2,AUTS2,COMT,PCDHA6,DOCK2,SDCCAG8,SSH2,CDH13,MDGA2,DAB1,PCDHA4,YEATS2,SLC8A3,TENM2,HDAC9,CSMD1,SCEL,FREM2,DACH2,RNF220,RORA,PTPRD,BRIP1,NELL1,SCN2A,DOCK10,RYR2,FAT3,PCDHA3,RGS9,ROBO2,PAX3,BMPR1B,DCC,CTNNA2,DNER,ETV6,NRG1,PGM3,DNAH5*
GO:0022008	neurogenesis	2.2464397692217434e-13	*NEGR1,NRXN1,TAFA1,NTRK2,NRG3,PTPRG,NCOA1,SOX6,FGF13,DMD,ASTN1,SLIT2,ERBB4,TNIK,DSCAML1,CNTN4,ITGA1,TCF12,PCDHAC2,CHODL,SOX5,DSCAM,IQSEC1,CASZ1,CNTNAP2,DDR2,ADAM22,CDH4,CNTN6,PCSK1,AGBL4,ULK4,PPP1R9A,CRPPA,BRINP3,DNMT3A,TOX,PCDH15,CNTN5,RIMS1,PPFIA2,WNT2,GRM7,NAV3,PTPRM,NRXN3,AUTS2,SDCCAG8,MDGA2,DAB1,SLC8A3,TENM2,HDAC9,RNF220,RORA,PTPRD,DOCK10,FAT3,ROBO2,BMPR1B,DCC,CTNNA2,DNER,ETV6*

GO:0098609	cell-cell adhesion	4.193673945420706e-13	*PCDHA13,NEGR1,PCDHA12,NRXN1,MAGI1,ASTN1,KIF26B,DSCAML1,CNTN4,ITGA1,PCDHA2,PCDHA11,CDH18,PCDHA9,PCDHAC2,CDH6,PCDHA10,DSCAM,PCDHA1,PCDHA8,CDH12,CDH4,CNTN6,PCDHAC1,LRFN5,PCDH7,PCDH15,CNTN5,PCDHA7,PCDHA5,PTPRM,NRXN3,PCDHA6,CDH13,DAB1,PCDHA4,CADM2,TENM2,CTNNA3,DLG2,PTPRD,FAT3,PCDHA3,ROBO2,DCC,CTNNA2*
GO:0048699	generation of neurons	1.3822791218409217e-12	*NEGR1,NRXN1,TAFA1,NTRK2,NRG3,PTPRG,NCOA1,FGF13,DMD,ASTN1,SLIT2,ERBB4,TNIK,DSCAML1,CNTN4,ITGA1,TCF12,PCDHAC2,CHODL,SOX5,DSCAM,IQSEC1,CASZ1,CNTNAP2,DDR2,CDH4,CNTN6,AGBL4,ULK4,PPP1R9A,CRPPA,BRINP3,DNMT3A,TOX,PCDH15,CNTN5,RIMS1,PPFIA2,WNT2,GRM7,PTPRM,NRXN3,AUTS2,SDCCAG8,MDGA2,DAB1,TENM2,HDAC9,RNF220,RORA,PTPRD,DOCK10,FAT3,ROBO2,BMPR1B,DCC,CTNNA2,DNER*
GO:0032501	multicellular organismal process	4.704035730240899e-12	*EPB41L4B,PIR,PCDHA13,GRIK2,KDM4B,ZBTB20,NEGR1,BTBD9,PCDHA12,NRXN1,TAFA1,BBS9,NTRK2,NBEA,NRG3,PTPRG,NCOA1,ATP2B4,PHEX,SOX6,TMEM64,FGF13,GPC3,DMD,ASTN1,CDIN1,ARHGAP24,SLIT2,FAM20C,KAZN,B3GNT5,ERBB4,KIF26B,TNIK,MYO3B,SPAG16,DSCAML1,HTR2C,CNTN4,ITGA1,TCF12,SGIP1,ESRRG,PCDHA2,PCDHA11,ANK2,PCDHA9,CD226,DGKB,PCDHAC2,CHODL,FGF14,PCDHA10,SOX5,DSCAM,DENND1B,IQSEC1,CASZ1,CNTNAP2,TRIM71,PCDHA1,PCDHA8,DDR2,ADAM22,TRPS1,CDH4,CELF4,ST8SIA6,CNTN6,MITF,PCSK1,PCDHAC1,LRFN5,UTRN,AGBL4,ULK4,RBFOX1,PPP1R9A,DYNC1H1,CRPPA,EDAR,BRINP3,DNMT3A,TOX,PCDH15,ACE2,PDE4D,CNTN5,PDE3A,RIMS1,PCDHA7,CPLANE1,PCDHA5,RNF216,PPFIA2,WNT2,PLPPR1,SGCD,GRM7,NAV3,PTPRM,NRXN3,LPIN1,MACROD2,AUTS2,COMT,PCDHA6,DOCK2,SDCCAG8,CDH13,MDGA2,DAB1,RFTN1,PCDHA4,YEATS2,SLC8A3,MYO5B,TENM2,HDAC9,KCNB2,CSMD1,SCEL,CTNNA3,FREM2,DACH2,RNF220,RORA,PTPRD,BRIP1,NELL1,PLCL1,SCN2A,DOCK10,RYR2,FAT3,PCDHA3,RGS9,ROBO2,PAX3,BMPR1B,DCC,CTNNA2,DNER,ETV6,NRG1,DNAH5*
GO:0030182	neuron differentiation	4.904289548107135e-10	*NEGR1,NRXN1,NTRK2,PTPRG,NCOA1,FGF13,DMD,SLIT2,ERBB4,TNIK,DSCAML1,CNTN4,ITGA1,TCF12,PCDHAC2,CHODL,DSCAM,IQSEC1,CASZ1,CNTNAP2,DDR2,CDH4,CNTN6,AGBL4,ULK4,PPP1R9A,CRPPA,BRINP3,DNMT3A,TOX,PCDH15,CNTN5,RIMS1,PPFIA2,WNT2,GRM7,PTPRM,NRXN3,AUTS2,MDGA2,DAB1,TENM2,HDAC9,RNF220,RORA,PTPRD,DOCK10,FAT3,ROBO2,BMPR1B,DCC,CTNNA2*
GO:0120036	plasma membrane bounded cell projection organization	5.5145106569113704e-8	*NEGR1,NRXN1,DNAH7,BBS9,NTRK2,PTPRG,DNAH8,FGF13,DMD,ARHGAP24,SLIT2,TNIK,MYO3B,SPAG16,DSCAML1,CNTN4,ITGA1,NEK1,PCDHAC2,CHODL,DSCAM,IQSEC1,CNTNAP2,DDR2,CDH4,CNTN6,ULK4,PPP1R9A,CRPPA,TOX,PCDH15,CNTN5,RIMS1,CPLANE1,PPFIA2,GRM7,PTPRM,NRXN3,AUTS2,SDCCAG8,CDH13,DAB1,TENM2,PTPRD,DOCK10,FAT3,ROBO2,BMPR1B,DCC,CTNNA2,DNAH5*
GO:0031175	neuron projectio	1.3785782531268244e-7	*NEGR1,NRXN1,NTRK2,PTPRG,FGF13,DMD,SLIT2,TNIK,DSCAML1,CNTN4,ITGA1,PCDHAC2,CHODL,DSCAM,IQSEC1,CNTNAP2,DDR2,CDH4,C*

	n developme nt		NTN6,ULK4,PPP1R9A,CRPPA,TOX,PCDH15,CNTN5,RIMS1,PPFIA2,GRM7,PTPRM,NRXN3,AUTS2,DAB1,PTPRD,DOCK10,FAT3,ROBO2,BMPR1B,DCC,CTNNA2
GO:0030030	cell projection organization	1.4587770905265904e-7	NEGR1,NRXN1,DNAH7,BBS9,NTRK2,PTPRG,DNAH8,FGF13,DMD,ARHGAP24,SLIT2,TNIK,MYO3B,SPAG16,DSCAML1,CNTN4,ITGA1,NEK1,PCDHAC2,CHODL,DSCAM,IQSEC1,CNTNAP2,DDR2,CDH4,CNTN6,ULK4,PPP1R9A,CRPPA,TOX,PCDH15,CNTN5,RIMS1,CPLANE1,PPFIA2,GRM7,PTPRM,NRXN3,AUTS2,SDCCAG8,CDH13,DAB1,TENM2,PTPRD,DOCK10,FAT3,ROBO2,BMPR1B,DCC,CTNNA2,DNAH5
GO:0048666	neuron development	1.98778754996964e-7	NEGR1,NRXN1,NTRK2,PTPRG,FGF13,DMD,SLIT2,TNIK,DSCAML1,CNTN4,ITGA1,PCDHAC2,CHODL,DSCAM,IQSEC1,CNTNAP2,DDR2,CDH4,CNTN6,AGBL4,ULK4,PPP1R9A,CRPPA,TOX,PCDH15,CNTN5,RIMS1,PPFIA2,GRM7,PTPRM,NRXN3,AUTS2,DAB1,TENM2,RNF220,PTPRD,DOCK10,FAT3,ROBO2,BMPR1B,DCC,CTNNA2
GO:0030154	cell differentiation	3.8576584311972467e-7	LRMDA,PIR,FSTL5,FAM9B,NEGR1,NRXN1,TAFA1,BBS9,NTRK2,NRG3,PTPRG,NCOA1,SOX6,TMEM64,FGF13,GPC3,DMD,ASTN1,CDIN1,ARHGAP24,SLIT2,FAM20C,KAZN,ERBB4,TNIK,SPAG16,DSCAML1,HTR2C,CNTN4,ITGA1,TCF12,ANK2,PRRC2C,PCDHAC2,CHODL,SOX5,DSCAM,IQSEC1,CASZ1,CNTNAP2,DDR2,ADAM22,TRPS1,CDH4,CELF4,CNTN6,MITF,PCSK1,AGBL4,ULK4,RBFOX1,PPP1R9A,SMAD9,CRPPA,EDAR,BRINP3,DNMT3A,TOX,PCDH15,PDE4D,CNTN5,PDE3A,RIMS1,PPFIA2,WNT2,SGCD,GRM7,NAV3,PTPRM,NRXN3,AUTS2,DOCK2,SDCCAG8,SSH2,MDGA2,DAB1,SLC8A3,TENM2,HDAC9,SCEL,RNF220,RORA,PTPRD,BRIP1,NELL1,DOCK10,FAT3,ROBO2,BMPR1B,DCC,CTNNA2,DNER,ETV6,NRG1,PGM3
GO:0048869	cellular developmental process	3.9076618623866194e-7	LRMDA,PIR,FSTL5,FAM9B,NEGR1,NRXN1,TAFA1,BBS9,NTRK2,NRG3,PTPRG,NCOA1,SOX6,TMEM64,FGF13,GPC3,DMD,ASTN1,CDIN1,ARHGAP24,SLIT2,FAM20C,KAZN,ERBB4,TNIK,SPAG16,DSCAML1,HTR2C,CNTN4,ITGA1,TCF12,ANK2,PRRC2C,PCDHAC2,CHODL,SOX5,DSCAM,IQSEC1,CASZ1,CNTNAP2,DDR2,ADAM22,TRPS1,CDH4,CELF4,CNTN6,MITF,PCSK1,AGBL4,ULK4,RBFOX1,PPP1R9A,SMAD9,CRPPA,EDAR,BRINP3,DNMT3A,TOX,PCDH15,PDE4D,CNTN5,PDE3A,RIMS1,PPFIA2,WNT2,SGCD,GRM7,NAV3,PTPRM,NRXN3,AUTS2,DOCK2,SDCCAG8,SSH2,MDGA2,DAB1,SLC8A3,TENM2,HDAC9,SCEL,RNF220,RORA,PTPRD,BRIP1,NELL1,DOCK10,FAT3,ROBO2,BMPR1B,DCC,CTNNA2,DNER,ETV6,NRG1,PGM3
GO:0034330	cell junction organization	9.310871740617454e-7	NEGR1,NRXN1,NTRK2,NBEA,NRG3,FGF13,PATJ,ERBB4,GPHN,CDH18,ANK2,DGKB,CDH6,DSCAM,IQSEC1,CNTNAP2,CDH12,CDH4,LRFN5,FRMPD4,ACE2,CNTN5,PPFIA2,NRXN3,CDH13,SLC8A3,PTPRD,DOCK10,MPDZ,ROBO2,CTNNA2,DNER
GO:0048812	neuron projection morphogenesis	0.0000020195821357739596	NRXN1,NTRK2,FGF13,DMD,SLIT2,TNIK,DSCAML1,CNTN4,ITGA1,PCDHAC2,CHODL,DSCAM,CNTNAP2,CDH4,CNTN6,CRPPA,CNTN5,RIMS1,PPFIA2,PTPRM,NRXN3,AUTS2,DAB1,PTPRD,DOCK10,ROBO2,BMPR1B,DCC,CTNNA2

GO:0000902	cell morphogenesis	0.0000033048859766993835	*MKLN1,NRXN1,NTRK2,FGF13,DMD,SLIT2,TNIK,DSCAML1,CNTN4,ITGA1,CDH18,PCDHAC2,CDH6,CHODL,DSCAM,CNTNAP2,CDH12,CDH4,CNTN6,CRPPA,PCDH15,CNTN5,RIMS1,PPFIA2,PTPRM,NRXN3,AUTS2,CDH13,DAB1,PTPRD,DOCK10,FAT3,ROBO2,BMPR1B,DCC,CTNNA2*
GO:0048468	cell development	0.0000034001493733019144	*PIR,FAM9B,NEGR1,NRXN1,NTRK2,PTPRG,TMEM64,FGF13,GPC3,DMD,CDIN1,SLIT2,FAM20C,ERBB4,TNIK,SPAG16,DSCAML1,CNTN4,ITGA1,ANK2,PRRC2C,PCDHAC2,CHODL,DSCAM,IQSEC1,CNTNAP2,DDR2,ADAM22,CDH4,CELF4,CNTN6,MITF,AGBL4,ULK4,PPP1R9A,CRPPA,DNMT3A,TOX,PCDH15,PDE4D,CNTN5,PDE3A,RIMS1,PPFIA2,WNT2,SGCD,GRM7,PTPRM,NRXN3,AUTS2,DOCK2,DAB1,SLC8A3,TENM2,HDAC9,RNF220,RORA,PTPRD,BRIP1,DOCK10,FAT3,ROBO2,BMPR1B,DCC,CTNNA2,DNER,ETV6,NRG1,PGM3*
GO:0120039	plasma membrane bounded cell projection morphogenesis	0.0000035028498634993 78	*NRXN1,NTRK2,FGF13,DMD,SLIT2,TNIK,DSCAML1,CNTN4,ITGA1,PCDHAC2,CHODL,DSCAM,CNTNAP2,CDH4,CNTN6,CRPPA,CNTN5,RIMS1,PPFIA2,PTPRM,NRXN3,AUTS2,DAB1,PTPRD,DOCK10,ROBO2,BMPR1B,DCC,CTNNA2*
GO:0007417	central nervous system development	0.0000036577496769847 93	*KDM4B,NRXN1,NTRK2,NRG3,NCOA1,ATP2B4,SOX6,FGF13,DMD,SLIT2,B3GNT5,ERBB4,DSCAML1,CNTN4,CNTNAP2,ADAM22,CNTN6,PCSK1,AGBL4,BRINP3,TOX,CNTN5,CPLANE1,WNT2,MACROD2,COMT,MDGA2,DAB1,SLC8A3,RORA,SCN2A,ROBO2,BMPR1B,DCC,CTNNA2,DNER,DNAH5*
GO:0048858	cell projection morphogenesis	0.0000041456975770935 35	*NRXN1,NTRK2,FGF13,DMD,SLIT2,TNIK,DSCAML1,CNTN4,ITGA1,PCDHAC2,CHODL,DSCAM,CNTNAP2,CDH4,CNTN6,CRPPA,CNTN5,RIMS1,PPFIA2,PTPRM,NRXN3,AUTS2,DAB1,PTPRD,DOCK10,ROBO2,BMPR1B,DCC,CTNNA2*
GO:0048667	cell morphogenesis involved in neuron differentiation	0.0000048599714453920 92	*NRXN1,NTRK2,FGF13,SLIT2,TNIK,DSCAML1,CNTN4,PCDHAC2,CHODL,DSCAM,CDH4,CNTN6,CRPPA,PCDH15,CNTN5,PPFIA2,PTPRM,NRXN3,AUTS2,DAB1,PTPRD,DOCK10,FAT3,ROBO2,BMPR1B,DCC,CTNNA2*
GO:0009653	anatomical structure morphogenesis	0.0000067538651886958 41	*PAX7,MKLN1,NRXN1,NTRK2,NRG3,NCOA1,ATP2B4,PHEX,SOX6,FGF13,GPC3,DMD,ARHGAP24,SLIT2,FAM20C,ERBB4,KIF26B,TNIK,MYO3B,DSCAML1,CNTN4,ITGA1,CDH18,ANK2,PCDHAC2,CDH6,CHODL,SOX5,DSCAM,CNTNAP2,TRIM71,CDH12,CDH4,CNTN6,SMAD9,CRPPA,EDAR,PCDH15,CNTN5,RIMS1,CPLANE1,PPFIA2,WNT2,SGCD,PTPRM,NRXN3,AUTS2,COMT,DOCK2,SDCCAG8,CDH13,DAB1,HDAC9,CSMD1,FREM2,RORA,PTPRD,DOCK10,RYR2,FAT3,ROBO2,PAX3,BMPR1B,DCC,CTNNA2,NRG1*
GO:0034329	cell junction assembly	0.0000083632201442750 64	*NEGR1,NRXN1,NTRK2,NRG3,FGF13,PATJ,ERBB4,CDH18,ANK2,CDH6,DSCAM,CNTNAP2,CDH12,CDH4,LRFN5,ACE2,CNTN5,NRXN3,CDH13,PTPRD,MPDZ,ROBO2,DNER*
GO:0007409	axonogenesis	0.0000735489307625682 2	*NRXN1,NTRK2,FGF13,SLIT2,DSCAML1,CNTN4,PCDHAC2,CHODL,DSCAM,CDH4,CNTN6,CRPPA*

			,CNTN5,PTPRM,NRXN3,AUTS2,DAB1,PTPRD,ROBO2,BMPR1B,DCC,CTNNA2
GO:0097485	neuron projection guidance	0.00008645769701686746	*NRXN1,SLIT2,DSCAML1,CNTN4,PCDHAC2,DSCAM,CDH4,CNTN6,CRPPA,CNTN5,PTPRM,NRXN3,PTPRD,ROBO2,BMPR1B,DCC*
GO:0007411	axon guidance	0.00008645769701686746	*NRXN1,SLIT2,DSCAML1,CNTN4,PCDHAC2,DSCAM,CDH4,CNTN6,CRPPA,CNTN5,PTPRM,NRXN3,PTPRD,ROBO2,BMPR1B,DCC*
GO:0120035	regulation of plasma membrane bounded cell projection organization	0.00014557724825100052	*NEGR1,NRXN1,NTRK2,PTPRG,FGF13,DMD,ARHGAP24,SLIT2,TNIK,MYO3B,CHODL,DSCAM,DDR2,CDH4,ULK4,TOX,PPFIA2,AUTS2,SDCCAG8,DAB1,TENM2,PTPRD,FAT3,ROBO2,DCC,CTNNA2*
GO:0061564	axon development	0.00015536443628009247	*NRXN1,NTRK2,FGF13,SLIT2,DSCAML1,CNTN4,PCDHAC2,CHODL,DSCAM,CDH4,CNTN6,CRPPA,CNTN5,GRM7,PTPRM,NRXN3,AUTS2,DAB1,PTPRD,ROBO2,BMPR1B,DCC,CTNNA2*
GO:0097120	receptor localization to synapse	0.00015812068988094546	*NRXN1,NBEA,ERBB4,GPHN,ADAM22,GPC6,NRXN3,DLG2,CEP112*
GO:0050808	synapse organization	0.0001599027679364596	*NEGR1,NRXN1,NTRK2,NBEA,NRG3,FGF13,ERBB4,GPHN,DGKB,CDH6,DSCAM,LRFN5,FRMPD4,CNTN5,PPFIA2,NRXN3,SLC8A3,PTPRD,DOCK10,ROBO2,CTNNA2,DNER*
GO:0031344	regulation of cell projection organization	0.0002336103628397868	*NEGR1,NRXN1,NTRK2,PTPRG,FGF13,DMD,ARHGAP24,SLIT2,TNIK,MYO3B,CHODL,DSCAM,DDR2,CDH4,ULK4,TOX,PPFIA2,AUTS2,SDCCAG8,DAB1,TENM2,PTPRD,FAT3,ROBO2,DCC,CTNNA2*
GO:0010975	regulation of neuron projection development	0.00026700734078554284	*NEGR1,NRXN1,NTRK2,PTPRG,FGF13,DMD,SLIT2,TNIK,CHODL,DSCAM,DDR2,CDH4,ULK4,TOX,PPFIA2,DAB1,PTPRD,FAT3,ROBO2,DCC,CTNNA2*
GO:0099536	synaptic signaling	0.0003868924669279174	*GRIK2,CADPS2,BTBD9,NRXN1,GRM3,NTRK2,NRG3,DMD,HTR2C,CNTN4,DGKB,CELF4,UTRN,PPP1R9A,RIMBP2,RIMS1,PLCB4,PPFIA2,GRM7,NRXN3,COMT,SLC8A3,CADPS,DLG2,PTPRD,PLCL1,RGS10,DCC*
GO:0007420	brain development	0.0004257943610349892	*KDM4B,NRXN1,NTRK2,NRG3,NCOA1,ATP2B4,SOX6,FGF13,DMD,SLIT2,ERBB4,DSCAML1,CNTN4,CNTNAP2,PCSK1,TOX,CNTN5,CPLANE1,WNT2,MACROD2,COMT,DAB1,RORA,SCN2A,ROBO2,CTNNA2,DNAH5*
GO:0060322	head development	0.0015275721953906989	*KDM4B,NRXN1,NTRK2,NRG3,NCOA1,ATP2B4,SOX6,FGF13,DMD,SLIT2,ERBB4,DSCAML1,CNTN4,CNTNAP2,PCSK1,TOX,CNTN5,CPLANE1,WNT2,MACROD2,COMT,DAB1,RORA,SCN2A,ROBO2,CTNNA2,DNAH5*
GO:0007416	synapse assembly	0.0016662954348922118	*NEGR1,NRXN1,NTRK2,NRG3,FGF13,ERBB4,DSCAM,LRFN5,CNTN5,NRXN3,PTPRD,ROBO2,DNER*

GO:0021953	central nervous system neuron differentiation	0.001863556687991644	*NRXN1,NTRK2,SLIT2,ERBB4,AGBL4,BRINP3,TOX,WNT2,MDGA2,RORA,ROBO2,BMPR1B,DCC*
GO:0007268	chemical synaptic transmission	0.002101278535865587	*GRIK2,CADPS2,BTBD9,NRXN1,GRM3,NTRK2,NRG3,DMD,HTR2C,CNTN4,DGKB,CELF4,PPP1R9A,RIMBP2,RIMS1,PLCB4,PPFIA2,GRM7,NRXN3,COMT,SLC8A3,CADPS,DLG2,PTPRD,PLCL1,DCC*
GO:0098916	anterograde trans-synaptic signaling	0.002101278535865587	*GRIK2,CADPS2,BTBD9,NRXN1,GRM3,NTRK2,NRG3,DMD,HTR2C,CNTN4,DGKB,CELF4,PPP1R9A,RIMBP2,RIMS1,PLCB4,PPFIA2,GRM7,NRXN3,COMT,SLC8A3,CADPS,DLG2,PTPRD,PLCL1,DCC*
GO:0001964	startle response	0.00237998392494136	*NRXN1,CNTNAP2,PCDH15,COMT,CSMD1,CTNNA2*
GO:0099537	trans-synaptic signaling	0.0024345553127522956	*GRIK2,CADPS2,BTBD9,NRXN1,GRM3,NTRK2,NRG3,DMD,HTR2C,CNTN4,DGKB,CELF4,PPP1R9A,RIMBP2,RIMS1,PLCB4,PPFIA2,GRM7,NRXN3,COMT,SLC8A3,CADPS,DLG2,PTPRD,PLCL1,DCC*
GO:0001764	neuron migration	0.003622529441139881	*NTRK2,NRG3,FGF13,ASTN1,ULK4,AUTS2,SDCCAG8,DAB1,FAT3,DCC,CTNNA2,DNER*
GO:0007267	cell-cell signaling	0.004257260570279139	*GRIK2,CADPS2,BTBD9,NRXN1,GRM3,NTRK2,NRG3,PHEX,FGF13,DMD,SH2D1A,HTR2C,CNTN4,ANK2,DGKB,FGF14,CELF4,PCSK1,UTRN,PPP1R9A,RIMBP2,RIMS1,PLCB4,PPFIA2,WNT2,GRM7,NRXN3,COMT,SLC8A3,CADPS,DLG2,PTPRD,PLCL1,RYR2,RGS10,DCC*
GO:0051128	regulation of cellular component organization	0.008905946712873912	*RNF103-CHMP3,MKLN1,NEGR1,BTBD9,NRXN1,NTRK2,NRG3,PTPRG,PDE4DIP,FGF13,PATJ,GPC3,DMD,ARHGAP24,SLIT2,TNIK,MYO3B,SGIP1,DGKB,CHODL,DSCAM,COLEC10,IQSEC1,ANK1,CNTNAP2,DDR2,CDH4,NSMCE2,LRFN5,ULK4,DYNC1H1,FRMPD4,TOX,ACE2,CHMP3,PDE3A,RIMS1,PPFIA2,NAV3,AUTS2,DOCK2,SDCCAG8,SSH2,CDH13,DAB1,TENM2,PTPRD,FAT3,MPDZ,ROBO2,DCC,CTNNA2,NRG1*
GO:0050804	modulation of chemical synaptic transmission	0.01261343242231277	*GRIK2,BTBD9,NRXN1,GRM3,NTRK2,NRG3,CNTN4,DGKB,CELF4,PPP1R9A,RIMS1,PLCB4,PPFIA2,GRM7,NRXN3,SLC8A3,PTPRD,PLCL1,DCC*
GO:0099177	regulation of trans-synaptic signaling	0.012984529484148754	*GRIK2,BTBD9,NRXN1,GRM3,NTRK2,NRG3,CNTN4,DGKB,CELF4,PPP1R9A,RIMS1,PLCB4,PPFIA2,GRM7,NRXN3,SLC8A3,PTPRD,PLCL1,DCC*
GO:0048513	animal organ development	0.01653221861694307	*PAX7,KDM4B,NRXN1,NTRK2,NRG3,NCOA1,ATP2B4,PHEX,SOX6,FGF13,GPC3,DMD,SLIT2,FAM20C,KAZN,ERBB4,KIF26B,MYO3B,DSCAML1,CNTN4,TCF12,ANK2,CHODL,SOX5,DSCAM,CNTNAP2,DDR2,TRPS1,CELF4,MITF,PCSK1,UTRN,RBFOX1,EDAR,TOX,PCDH15,CNTN5,CPLANE1,WNT2,SGCD,PTPRM,LPIN1,MACROD2,COMT,DAB1,HDAC9,CSMD1,SCEL,FREM2,RORA,BRIP1,NELL1,SCN2A,RYR2,FAT3,ROBO2,PAX3,BMPR1B,CTNNA2,DNER,NRG1,DNAH5*

GO:1903779	regulation of cardiac conduction	0.019250455551654333	*ATP2B4,ANK2,ACE2,SLC8A3,RYR2*
GO:0008038	neuron recognition	0.028155416526814872	*DSCAML1,CNTN4,DSCAM,CNTNAP2,CNTN6,ROBO2*
GO:0007610	behavior	0.02878714770417524	*GRIK2,NEGR1,BTBD9,NRXN1,NTRK2,NCOA1,FGF13,DMD,ASTN1,HTR2C,SGIP1,DSCAM,CNTNAP2,ADAM22,PCDH15,ACE2,NRXN3,COMT,DAB1,SLC8A3,CSMD1,SCN2A*
GO:0007154	cell communication	0.03341245162946985	*ZNF385B,GRIK2,FSTL5,CADPS2,MKLN1,BTBD9,MAST4,NRXN1,MAGI1,FAM13A,GRM3,TAFA1,NTRK2,RASGEF1B,NRG3,PTPRG,NCOA1,ATP2B4,PHEX,TMEM64,FGF13,PATJ,GPC3,DMD,ADCY2,ATRNL1,ARHGAP24,SLIT2,FAM20C,ERBB4,TNIK,SH2D1A,SH3RF3,HTR2C,CNTN4,RAP1GAP2,ITGA1,ESRRG,ANK2,CD226,DGKB,CDH6,FGF14,DSCAM,DENND1B,COLEC10,IQSEC1,ANK1,CNTNAP2,TRIM71,TBCK,DDR2,GPC6,CELF4,CNTN6,MITF,PCSK1,UTRN,GPC5,ULK4,PPP1R9A,TMEM117,SMAD9,EDAR,RIMBP2,FHIT,ACE2,PDE4D,PDE3A,RIMS1,PLCB4,PPFIA2,WNT2,PLPPR1,SGCD,GRM7,PTPRM,NRXN3,AUTS2,COMT,DOCK2,CDH13,DAB1,RFTN1,SLC8A3,TENM2,CADPS,RGS7,SCEL,CTNNA3,FREM2,RNF220,DLG2,RORA,PTPRD,BRIP1,PLCL1,SCN2A,DOCK10,RYR2,KLHDC10,RGS9,ROBO2,RGS10,BMPR1B,DCC,DNER,NRG1*
GO:0006942	regulation of striated muscle contraction	0.03371236296015852	*FGF13,DMD,ANK2,ACE2,PDE4D,SLC8A3,CTNNA3,RYR2*
GO:0070593	dendrite self-avoidance	0.03452132602975487	*DSCAML1,CNTN4,DSCAM,CNTN6*
GO:0099633	protein localization to postsynaptic specialization membrane	0.03459504460220107	*NBEA,ERBB4,ADAM22,GPC6,NRXN3*
GO:0099645	neurotransmitter receptor localization to postsynaptic specialization membrane	0.03459504460220107	*NBEA,ERBB4,ADAM22,GPC6,NRXN3*
GO:0031503	protein-containing complex localizat	0.03531183965462704	*NRXN1,NBEA,ERBB4,GPHN,ADAM22,GPC6,SGCD,NRXN3,DLG2,CEP112,NRG1*

	ion		
GO:0008037	cell recognition	0.03585959430188735	*DSCAML1,CNTN4,CD226,DSCAM,COLEC10,CNTNAP2,CNTN6,PCDHA7,DOCK2,ROBO2*
GO:0007043	cell-cell junction assembly	0.04002136295754816	*PATJ,CDH18,ANK2,CDH6,CNTNAP2,CDH12,CDH4,ACE2,CDH13,MPDZ*
GO:0016339	calcium-dependent cell-cell adhesion via plasma membrane cell adhesion molecules	0.04118414260052329	*NRXN1,CDH18,CDH6,CDH12,CDH4,CDH13*
GO:0031346	positive regulation of cell projection organization	0.048508864385594166	*NEGR1,NRXN1,NTRK2,DMD,SLIT2,MYO3B,CHODL,DSCAM,DDR2,CDH4,TOX,AUTS2,TENM2,PTPRD,ROBO2*
BP			
GO:0007399	nervous system development	2.0183874061463716e-24	*PCDHA13,KDM4B,NEGR1,PCDHA12,NRXN1,TAFA1,NTRK2,NRG3,PTPRG,NCOA1,ATP2B4,SOX6,FGF13,DMD,ASTN1,SLIT2,B3GNT5,ERBB4,TNIK,DSCAML1,CNTN4,ITGA1,TCF12,PCDHA2,PCDHA11,ANK2,PCDHA9,PCDHAC2,CHODL,FGF14,PCDHA10,SOX5,DSCAM,IQSEC1,CASZ1,CNTNAP2,TRIM71,PCDHA1,PCDHA8,DDR2,ADAM22,CDH4,CNTN6,PCSK1,PCDHAC1,LRFN5,AGBL4,ULK4,RBFOX1,PPP1R9A,CRPPA,BRINP3,DNMT3A,TOX,PCDH15,CNTN5,RIMS1,PCDHA7,CPLANE1,PCDHA5,PPFIA2,WNT2,PLPPR1,GRM7,NAV3,PTPRM,NRXN3,LPIN1,MACROD2,AUTS2,COMT,PCDHA6,SDCCAG8,MDGA2,DAB1,PCDHA4,SLC8A3,TENM2,HDAC9,RNF220,RORA,PTPRD,NELL1,SCN2A,DOCK10,FAT3,PCDHA3,RGS9,ROBO2,PAX3,BMPR1B,DCC,CTNNA2,DNER,ETV6,NRG1,DNAH5*
GO:0007156	homophilic cell adhesion via plasma membrane adhesion molecules	1.600965725513845e-23	*PCDHA13,PCDHA12,DSCAML1,CNTN4,PCDHA2,PCDHA11,CDH18,PCDHA9,PCDHAC2,CDH6,PCDHA10,DSCAM,PCDHA1,PCDHA8,CDH12,CDH4,CNTN6,PCDHAC1,PCDH7,PCDH15,PCDHA7,PCDHA5,PTPRM,PCDHA6,CDH13,PCDHA4,CADM2,FAT3,PCDHA3,ROBO2*
GO:0098742	cell-cell adhesion via plasma-membrane adhesion molecules	2.2278053397801544e-22	*PCDHA13,PCDHA12,NRXN1,DSCAML1,CNTN4,PCDHA2,PCDHA11,CDH18,PCDHA9,PCDHAC2,CDH6,PCDHA10,DSCAM,PCDHA1,PCDHA8,CDH12,CDH4,CNTN6,PCDHAC1,LRFN5,PCDH7,PCDH15,PCDHA7,PCDHA5,PTPRM,PCDHA6,CDH13,DAB1,PCDHA4,CADM2,TENM2,PTPRD,FAT3,PCDHA3,ROBO2*
GO:0048731	system development	5.2027990005181e-18	*PCDHA13,KDM4B,NEGR1,PCDHA12,NRXN1,TAFA1,NTRK2,NRG3,PTPRG,NCOA1,ATP2B4,PHEX,SOX6,FGF13,GPC3,DMD,ASTN1,ARHGAP24,SLIT2,FAM20C,B3GNT5,ERBB4,KIF26B,TNIK,*

			DSCAML1,CNTN4,ITGA1,TCF12,PCDHA2,PCDHA11,ANK2,PCDHA9,PCDHAC2,CHODL,FGF14,PCDHA10,SOX5,DSCAM,IQSEC1,CASZ1,CNTNAP2,TRIM71,PCDHA1,PCDHA8,DDR2,ADAM22,TRPS1,CDH4,CELF4,CNTN6,MITF,PCSK1,PCDHAC1,LRFN5,AGBL4,ULK4,RBFOX1,PPP1R9A,CRPPA,EDAR,BRINP3,DNMT3A,TOX,PCDH15,CNTN5,RIMS1,PCDHA7,CPLANE1,PCDHA5,PPFIA2,WNT2,PLPPR1,SGCD,GRM7,NAV3,PTPRM,NRXN3,LPIN1,MACROD2,AUTS2,COMT,PCDHA6,SDCCAG8,CDH13,MDGA2,DAB1,PCDHA4,SLC8A3,TENM2,HDAC9,CSMD1,FREM2,RNF220,RORA,PTPRD,BRIP1,NELL1,SCN2A,DOCK10,RYR2,FAT3,PCDHA3,RGS9,ROBO2,PAX3,BMPR1B,DCC,CTNNA2,DNER,ETV6,NRG1,DNAH5
GO:0007275	multicellular organism development	1.240683744738341e-15	*PCDHA13,KDM4B,NEGR1,PCDHA12,NRXN1,TAFA1,NTRK2,NRG3,PTPRG,NCOA1,ATP2B4,PHEX,SOX6,TMEM64,FGF13,GPC3,DMD,ASTN1,ARHGAP24,SLIT2,FAM20C,B3GNT5,ERBB4,KIF26B,TNIK,MYO3B,DSCAML1,CNTN4,ITGA1,TCF12,PCDHA2,PCDHA11,ANK2,PCDHA9,PCDHAC2,CHODL,FGF14,PCDHA10,SOX5,DSCAM,IQSEC1,CASZ1,CNTNAP2,TRIM71,PCDHA1,PCDHA8,DDR2,ADAM22,TRPS1,CDH4,CELF4,ST8SIA6,CNTN6,MITF,PCSK1,PCDHAC1,LRFN5,AGBL4,ULK4,RBFOX1,PPP1R9A,CRPPA,EDAR,BRINP3,DNMT3A,TOX,PCDH15,CNTN5,RIMS1,PCDHA7,CPLANE1,PCDHA5,PPFIA2,WNT2,PLPPR1,SGCD,GRM7,NAV3,PTPRM,NRXN3,LPIN1,MACROD2,AUTS2,COMT,PCDHA6,SDCCAG8,CDH13,MDGA2,DAB1,PCDHA4,YEATS2,SLC8A3,TENM2,HDAC9,CSMD1,SCEL,FREM2,DACH2,RNF220,RORA,PTPRD,BRIP1,NELL1,SCN2A,DOCK10,RYR2,FAT3,PCDHA3,RGS9,ROBO2,PAX3,BMPR1B,DCC,CTNNA2,DNER,ETV6,NRG1,DNAH5*
GO:0048856	anatomical structure development	1.5845087219922929e-15	*PAX7,PIR,PCDHA13,KDM4B,FAM9B,MKLN1,NEGR1,PCDHA12,NRXN1,TAFA1,NTRK2,NRG3,PTPRG,NCOA1,ATP2B4,PHEX,SOX6,TMEM64,FGF13,GPC3,DMD,ASTN1,CDIN1,ARHGAP24,SLIT2,FAM20C,KAZN,B3GNT5,ERBB4,KIF26B,TNIK,MYO3B,SPAG16,DSCAML1,CNTN4,ITGA1,TCF12,PCDHA2,PCDHA11,CDH18,ANK2,PCDHA9,PRRC2C,PCDHAC2,CDH6,CHODL,FGF14,PCDHA10,SOX5,DSCAM,COLEC10,IQSEC1,CASZ1,CNTNAP2,TRIM71,PCDHA1,PCDHA8,CDH12,DDR2,ADAM22,TRPS1,CDH4,CELF4,ST8SIA6,CNTN6,MITF,PCSK1,PCDHAC1,LRFN5,UTRN,AGBL4,ULK4,RBFOX1,PPP1R9A,SMAD9,CRPPA,EDAR,BRINP3,DNMT3A,TOX,PCDH15,PDE4D,CNTN5,PDE3A,RIMS1,PCDHA7,CPLANE1,PCDHA5,PPFIA2,WNT2,PLPPR1,SGCD,GRM7,NAV3,PTPRM,NRXN3,LPIN1,MACROD2,AUTS2,COMT,PCDHA6,DOCK2,SDCCAG8,CDH13,MDGA2,DAB1,PCDHA4,YEATS2,SLC8A3,TENM2,HDAC9,CSMD1,SCEL,FREM2,DACH2,RNF220,RORA,PTPRD,BRIP1,NELL1,SCN2A,DOCK10,RYR2,FAT3,PCDHA3,RGS9,ROBO2,PAX3,BMPR1B,DCC,CTNNA2,DNER,ETV6,NRG1,PGM3,DNAH5*
GO:0007155	cell adhesion	1.5065437692442665e-14	*EPB41L4B,PCDHA13,MKLN1,NEGR1,PCDHA12,NRXN1,MAGI1,LMO7,FBLN7,DMD,ASTN1,KIF26B,DSCAML1,CNTN4,ITGA1,PCDHA2,PCDHA11,CDH18,PCDHA9,CD226,PCDHAC2,CDH6,PCDHA10,EDIL3,DSCAM,COL28A1,CNTNAP2,PCDHA1,PCDHA8,CDH12,DDR2,ADAM22,CDH4,CNTN6*

			,PCDHAC1,LRFN5,UTRN,PCDH7,PCDH15,CNTN5,PCDHA7,PCDHA5,PPFIA2,PTPRM,NRXN3,PCDHA6,CDH13,DAB1,PCDHA4,CADM2,TENM2,CTNNA3,FREM2,DLG2,PTPRD,FAT3,PCDHA3,ROBO2,DCC,CTNNA2,NRG1
GO:0032502	developmental process	2.679932771630597e-14	*PAX7,LRMDA,PIR,PCDHA13,KDM4B,FSTL5,FAM9B,MKLN1,NEGR1,PCDHA12,NRXN1,TAFA1,BBS9,NTRK2,NRG3,PTPRG,NCOA1,ATP2B4,PHEX,SOX6,TMEM64,FGF13,GPC3,DMD,ASTN1,CDIN1,ARHGAP24,SLIT2,FAM20C,KAZN,B3GNT5,ERBB4,KIF26B,TNIK,MYO3B,SPAG16,DSCAML1,HTR2C,CNTN4,ITGA1,TCF12,PCDHA2,PCDHA11,CDH18,ANK2,PCDHA9,PRRC2C,PCDHAC2,CDH6,CHODL,FGF14,PCDHA10,SOX5,DSCAM,COLEC10,IQSEC1,CASZ1,CNTNAP2,TRIM71,PCDHA1,PCDHA8,CDH12,DDR2,ADAM22,TRPS1,CDH4,CELF4,ST8SIA6,CNTN6,MITF,PCSK1,PCDHAC1,LRFN5,UTRN,AGBL4,ULK4,RBFOX1,PPP1R9A,SMAD9,CRPPA,EDAR,BRINP3,DNMT3A,TOX,PCDH15,PDE4D,CNTN5,PDE3A,RIMS1,PCDHA7,CPLANE1,PCDHA5,PPFIA2,WNT2,PLPPR1,SGCD,GRM7,NAV3,PTPRM,NRXN3,LPIN1,MACROD2,AUTS2,COMT,PCDHA6,DOCK2,SDCCAG8,SSH2,CDH13,MDGA2,DAB1,PCDHA4,YEATS2,SLC8A3,TENM2,HDAC9,CSMD1,SCEL,FREM2,DACH2,RNF220,RORA,PTPRD,BRIP1,NELL1,SCN2A,DOCK10,RYR2,FAT3,PCDHA3,RGS9,ROBO2,PAX3,BMPR1B,DCC,CTNNA2,DNER,ETV6,NRG1,PGM3,DNAH5*
GO:0022008	neurogenesis	2.2464397692217434e-13	*NEGR1,NRXN1,TAFA1,NTRK2,NRG3,PTPRG,NCOA1,SOX6,FGF13,DMD,ASTN1,SLIT2,ERBB4,TNIK,DSCAML1,CNTN4,ITGA1,TCF12,PCDHAC2,CHODL,SOX5,DSCAM,IQSEC1,CASZ1,CNTNAP2,DDR2,ADAM22,CDH4,CNTN6,PCSK1,AGBL4,ULK4,PPP1R9A,CRPPA,BRINP3,DNMT3A,TOX,PCDH15,CNTN5,RIMS1,PPFIA2,WNT2,GRM7,NAV3,PTPRM,NRXN3,AUTS2,SDCCAG8,MDGA2,DAB1,SLC8A3,TENM2,HDAC9,RNF220,RORA,PTPRD,DOCK10,FAT3,ROBO2,BMPR1B,DCC,CTNNA2,DNER,ETV6*
GO:0098609	cell-cell adhesion	4.193673945420706e-13	*PCDHA13,NEGR1,PCDHA12,NRXN1,MAGI1,ASTN1,KIF26B,DSCAML1,CNTN4,ITGA1,PCDHA2,PCDHA11,CDH18,PCDHA9,PCDHAC2,CDH6,PCDHA10,DSCAM,PCDHA1,PCDHA8,CDH12,CDH4,CNTN6,PCDHAC1,LRFN5,PCDH7,PCDH15,CNTN5,PCDHA7,PCDHA5,PTPRM,NRXN3,PCDHA6,CDH13,DAB1,PCDHA4,CADM2,TENM2,CTNNA3,DLG2,PTPRD,FAT3,PCDHA3,ROBO2,DCC,CTNNA2*
GO:0048699	generation of neurons	1.3822791218409217e-12	*NEGR1,NRXN1,TAFA1,NTRK2,NRG3,PTPRG,NCOA1,FGF13,DMD,ASTN1,SLIT2,ERBB4,TNIK,DSCAML1,CNTN4,ITGA1,TCF12,PCDHAC2,CHODL,SOX5,DSCAM,IQSEC1,CASZ1,CNTNAP2,DDR2,CDH4,CNTN6,AGBL4,ULK4,PPP1R9A,CRPPA,BRINP3,DNMT3A,TOX,PCDH15,CNTN5,RIMS1,PPFIA2,WNT2,GRM7,PTPRM,NRXN3,AUTS2,SDCCAG8,MDGA2,DAB1,TENM2,HDAC9,RNF220,RORA,PTPRD,DOCK10,FAT3,ROBO2,BMPR1B,DCC,CTNNA2,DNER*
GO:0032501	multicellular organismal process	4.704035730240899e-12	*EPB41L4B,PIR,PCDHA13,GRIK2,KDM4B,ZBTB20,NEGR1,BTBD9,PCDHA12,NRXN1,TAFA1,BBS9,NTRK2,NBEA,NRG3,PTPRG,NCOA1,ATP2B4,PHEX,SOX6,TMEM64,FGF13,GPC3,DMD,ASTN1,CDIN1,ARHGAP24,SLIT2,FAM20C,KAZN,B3GNT5,ERBB4,KIF26B,TNIK,MYO3B,SPAG16,D*

			SCAML1,HTR2C,CNTN4,ITGA1,TCF12,SGIP1,ESRRG,PCDHA2,PCDHA11,ANK2,PCDHA9,CD226,DGKB,PCDHAC2,CHODL,FGF14,PCDHA10,SOX5,DSCAM,DENND1B,IQSEC1,CASZ1,CNTNAP2,TRIM71,PCDHA1,PCDHA8,DDR2,ADAM22,TRPS1,CDH4,CELF4,ST8SIA6,CNTN6,MITF,PCSK1,PCDHAC1,LRFN5,UTRN,AGBL4,ULK4,RBFOX1,PPP1R9A,DYNC1H1,CRPPA,EDAR,BRINP3,DNMT3A,TOX,PCDH15,ACE2,PDE4D,CNTN5,PDE3A,RIMS1,PCDHA7,CPLANE1,PCDHA5,RNF216,PPFIA2,WNT2,PLPPR1,SGCD,GRM7,NAV3,PTPRM,NRXN3,LPIN1,MACROD2,AUTS2,COMT,PCDHA6,DOCK2,SDCCAG8,CDH13,MDGA2,DAB1,RFTN1,PCDHA4,YEATS2,SLC8A3,MYO5B,TENM2,HDAC9,KCNB2,CSMD1,SCEL,CTNNA3,FREM2,DACH2,RNF220,RORA,PTPRD,BRIP1,NELL1,PLCL1,SCN2A,DOCK10,RYR2,FAT3,PCDHA3,RGS9,ROBO2,PAX3,BMPR1B,DCC,CTNNA2,DNER,ETV6,NRG1,DNAH5
GO:0030182	neuron differentiation	4.904289548107135e-10	*NEGR1,NRXN1,NTRK2,PTPRG,NCOA1,FGF13,DMD,SLIT2,ERBB4,TNIK,DSCAML1,CNTN4,ITGA1,TCF12,PCDHAC2,CHODL,DSCAM,IQSEC1,CASZ1,CNTNAP2,DDR2,CDH4,CNTN6,AGBL4,ULK4,PPP1R9A,CRPPA,BRINP3,DNMT3A,TOX,PCDH15,CNTN5,RIMS1,PPFIA2,WNT2,GRM7,PTPRM,NRXN3,AUTS2,MDGA2,DAB1,TENM2,HDAC9,RNF220,RORA,PTPRD,DOCK10,FAT3,ROBO2,BMPR1B,DCC,CTNNA2*
GO:0120036	plasma membrane bounded cell projection organization	5.5145106569113704e-8	*NEGR1,NRXN1,DNAH7,BBS9,NTRK2,PTPRG,DNAH8,FGF13,DMD,ARHGAP24,SLIT2,TNIK,MYO3B,SPAG16,DSCAML1,CNTN4,ITGA1,NEK1,PCDHAC2,CHODL,DSCAM,IQSEC1,CNTNAP2,DDR2,CDH4,CNTN6,ULK4,PPP1R9A,CRPPA,TOX,PCDH15,CNTN5,RIMS1,CPLANE1,PPFIA2,GRM7,PTPRM,NRXN3,AUTS2,SDCCAG8,CDH13,DAB1,TENM2,PTPRD,DOCK10,FAT3,ROBO2,BMPR1B,DCC,CTNNA2,DNAH5*
GO:0031175	neuron projection development	1.3785782531268244e-7	*NEGR1,NRXN1,NTRK2,PTPRG,FGF13,DMD,SLIT2,TNIK,DSCAML1,CNTN4,ITGA1,PCDHAC2,CHODL,DSCAM,IQSEC1,CNTNAP2,DDR2,CDH4,CNTN6,ULK4,PPP1R9A,CRPPA,TOX,PCDH15,CNTN5,RIMS1,PPFIA2,GRM7,PTPRM,NRXN3,AUTS2,DAB1,PTPRD,DOCK10,FAT3,ROBO2,BMPR1B,DCC,CTNNA2*
GO:0030030	cell projection organization	1.4587770905265904e-7	*NEGR1,NRXN1,DNAH7,BBS9,NTRK2,PTPRG,DNAH8,FGF13,DMD,ARHGAP24,SLIT2,TNIK,MYO3B,SPAG16,DSCAML1,CNTN4,ITGA1,NEK1,PCDHAC2,CHODL,DSCAM,IQSEC1,CNTNAP2,DDR2,CDH4,CNTN6,ULK4,PPP1R9A,CRPPA,TOX,PCDH15,CNTN5,RIMS1,CPLANE1,PPFIA2,GRM7,PTPRM,NRXN3,AUTS2,SDCCAG8,CDH13,DAB1,TENM2,PTPRD,DOCK10,FAT3,ROBO2,BMPR1B,DCC,CTNNA2,DNAH5*
GO:0048666	neuron development	1.98778754996964e-7	*NEGR1,NRXN1,NTRK2,PTPRG,FGF13,DMD,SLIT2,TNIK,DSCAML1,CNTN4,ITGA1,PCDHAC2,CHODL,DSCAM,IQSEC1,CNTNAP2,DDR2,CDH4,CNTN6,AGBL4,ULK4,PPP1R9A,CRPPA,TOX,PCDH15,CNTN5,RIMS1,PPFIA2,GRM7,PTPRM,NRXN3,AUTS2,DAB1,TENM2,RNF220,PTPRD,DOCK10,FAT3,ROBO2,BMPR1B,DCC,CTNNA2*
GO:0030154	cell differentiation	3.8576584311972467e-7	*LRMDA,PIR,FSTL5,FAM9B,NEGR1,NRXN1,TAFA1,BBS9,NTRK2,NRG3,PTPRG,NCOA1,SOX6,TMEM64,FGF13,GPC3,DMD,ASTN1,CDIN1,ARHG*

			AP24,SLIT2,FAM20C,KAZN,ERBB4,TNIK,SPAG16,DSCAML1,HTR2C,CNTN4,ITGA1,TCF12,ANK2,PRRC2C,PCDHAC2,CHODL,SOX5,DSCAM,IQSEC1,CASZ1,CNTNAP2,DDR2,ADAM22,TRPS1,CDH4,CELF4,CNTN6,MITF,PCSK1,AGBL4,ULK4,RBFOX1,PPP1R9A,SMAD9,CRPPA,EDAR,BRINP3,DNMT3A,TOX,PCDH15,PDE4D,CNTN5,PDE3A,RIMS1,PPFIA2,WNT2,SGCD,GRM7,NAV3,PTPRM,NRXN3,AUTS2,DOCK2,SDCCAG8,SSH2,MDGA2,DAB1,SLC8A3,TENM2,HDAC9,SCEL,RNF220,RORA,PTPRD,BRIP1,NELL1,DOCK10,FAT3,ROBO2,BMPR1B,DCC,CTNNA2,DNER,ETV6,NRG1,PGM3
GO:0048869	cellular developmental process	3.9076618623866194e-7	*LRMDA,PIR,FSTL5,FAM9B,NEGR1,NRXN1,TAFA1,BBS9,NTRK2,NRG3,PTPRG,NCOA1,SOX6,TMEM64,FGF13,GPC3,DMD,ASTN1,CDIN1,ARHGAP24,SLIT2,FAM20C,KAZN,ERBB4,TNIK,SPAG16,DSCAML1,HTR2C,CNTN4,ITGA1,TCF12,ANK2,PRRC2C,PCDHAC2,CHODL,SOX5,DSCAM,IQSEC1,CASZ1,CNTNAP2,DDR2,ADAM22,TRPS1,CDH4,CELF4,CNTN6,MITF,PCSK1,AGBL4,ULK4,RBFOX1,PPP1R9A,SMAD9,CRPPA,EDAR,BRINP3,DNMT3A,TOX,PCDH15,PDE4D,CNTN5,PDE3A,RIMS1,PPFIA2,WNT2,SGCD,GRM7,NAV3,PTPRM,NRXN3,AUTS2,DOCK2,SDCCAG8,SSH2,MDGA2,DAB1,SLC8A3,TENM2,HDAC9,SCEL,RNF220,RORA,PTPRD,BRIP1,NELL1,DOCK10,FAT3,ROBO2,BMPR1B,DCC,CTNNA2,DNER,ETV6,NRG1,PGM3*
GO:0034330	cell junction organization	9.310871740617454e-7	*NEGR1,NRXN1,NTRK2,NBEA,NRG3,FGF13,PATJ,ERBB4,GPHN,CDH18,ANK2,DGKB,CDH6,DSCAM,IQSEC1,CNTNAP2,CDH12,CDH4,LRFN5,FRMPD4,ACE2,CNTN5,PPFIA2,NRXN3,CDH13,SLC8A3,PTPRD,DOCK10,MPDZ,ROBO2,CTNNA2,DNER*
GO:0048812	neuron projection morphogenesis	0.0000020195821357739596	*NRXN1,NTRK2,FGF13,DMD,SLIT2,TNIK,DSCAML1,CNTN4,ITGA1,PCDHAC2,CHODL,DSCAM,CNTNAP2,CDH4,CNTN6,CRPPA,CNTN5,RIMS1,PPFIA2,PTPRM,NRXN3,AUTS2,DAB1,PTPRD,DOCK10,ROBO2,BMPR1B,DCC,CTNNA2*
GO:0000902	cell morphogenesis	0.0000033048859766993835	*MKLN1,NRXN1,NTRK2,FGF13,DMD,SLIT2,TNIK,DSCAML1,CNTN4,ITGA1,CDH18,PCDHAC2,CDH6,CHODL,DSCAM,CNTNAP2,CDH12,CDH4,CNTN6,CRPPA,PCDH15,CNTN5,RIMS1,PPFIA2,PTPRM,NRXN3,AUTS2,CDH13,DAB1,PTPRD,DOCK10,FAT3,ROBO2,BMPR1B,DCC,CTNNA2*
GO:0048468	cell development	0.0000034001493733019144	*PIR,FAM9B,NEGR1,NRXN1,NTRK2,PTPRG,TMEM64,FGF13,GPC3,DMD,CDIN1,SLIT2,FAM20C,ERBB4,TNIK,SPAG16,DSCAML1,CNTN4,ITGA1,ANK2,PRRC2C,PCDHAC2,CHODL,DSCAM,IQSEC1,CNTNAP2,DDR2,ADAM22,CDH4,CELF4,CNTN6,MITF,AGBL4,ULK4,PPP1R9A,CRPPA,DNMT3A,TOX,PCDH15,PDE4D,CNTN5,PDE3A,RIMS1,PPFIA2,WNT2,SGCD,GRM7,PTPRM,NRXN3,AUTS2,DOCK2,DAB1,SLC8A3,TENM2,HDAC9,RNF220,RORA,PTPRD,BRIP1,DOCK10,FAT3,ROBO2,BMPR1B,DCC,CTNNA2,DNER,ETV6,NRG1,PGM3*
GO:0120039	plasma membrane bounded cell projectio	0.000003502849863499378	*NRXN1,NTRK2,FGF13,DMD,SLIT2,TNIK,DSCAML1,CNTN4,ITGA1,PCDHAC2,CHODL,DSCAM,CNTNAP2,CDH4,CNTN6,CRPPA,CNTN5,RIMS1,PPFIA2,PTPRM,NRXN3,AUTS2,DAB1,PTPRD,DOCK10,ROBO2,BMPR1B,DCC,CTNNA2*

	n morphogenesis		
GO:0007417	central nervous system development	0.000003657749676984793	*KDM4B,NRXN1,NTRK2,NRG3,NCOA1,ATP2B4,SOX6,FGF13,DMD,SLIT2,B3GNT5,ERBB4,DSCAML1,CNTN4,CNTNAP2,ADAM22,CNTN6,PCSK1,AGBL4,BRINP3,TOX,CNTN5,CPLANE1,WNT2,MACROD2,COMT,MDGA2,DAB1,SLC8A3,RORA,SCN2A,ROBO2,BMPR1B,DCC,CTNNA2,DNER,DNAH5*
GO:0048858	cell projection morphogenesis	0.000004145697577093535	*NRXN1,NTRK2,FGF13,DMD,SLIT2,TNIK,DSCAML1,CNTN4,ITGA1,PCDHAC2,CHODL,DSCAM,CNTNAP2,CDH4,CNTN6,CRPPA,CNTN5,RIMS1,PPFIA2,PTPRM,NRXN3,AUTS2,DAB1,PTPRD,DOCK10,ROBO2,BMPR1B,DCC,CTNNA2*
GO:0048667	cell morphogenesis involved in neuron differentiation	0.000004859971445392092	*NRXN1,NTRK2,FGF13,SLIT2,TNIK,DSCAML1,CNTN4,PCDHAC2,CHODL,DSCAM,CDH4,CNTN6,CRPPA,PCDH15,CNTN5,PPFIA2,PTPRM,NRXN3,AUTS2,DAB1,PTPRD,DOCK10,FAT3,ROBO2,BMPR1B,DCC,CTNNA2*
GO:0009653	anatomical structure morphogenesis	0.000006753865188695841	*PAX7,MKLN1,NRXN1,NTRK2,NRG3,NCOA1,ATP2B4,PHEX,SOX6,FGF13,GPC3,DMD,ARHGAP24,SLIT2,FAM20C,ERBB4,KIF26B,TNIK,MYO3B,DSCAML1,CNTN4,ITGA1,CDH18,ANK2,PCDHAC2,CDH6,CHODL,SOX5,DSCAM,CNTNAP2,TRIM71,CDH12,CDH4,CNTN6,SMAD9,CRPPA,EDAR,PCDH15,CNTN5,RIMS1,CPLANE1,PPFIA2,WNT2,SGCD,PTPRM,NRXN3,AUTS2,COMT,DOCK2,SDCCAG8,CDH13,DAB1,HDAC9,CSMD1,FREM2,RORA,PTPRD,DOCK10,RYR2,FAT3,ROBO2,PAX3,BMPR1B,DCC,CTNNA2,NRG1*
GO:0034329	cell junction assembly	0.000008363220144275064	*NEGR1,NRXN1,NTRK2,NRG3,FGF13,PATJ,ERBB4,CDH18,ANK2,CDH6,DSCAM,CNTNAP2,CDH12,CDH4,LRFN5,ACE2,CNTN5,NRXN3,CDH13,PTPRD,MPDZ,ROBO2,DNER*
GO:0007409	axonogenesis	0.00007354893076256822	*NRXN1,NTRK2,FGF13,SLIT2,DSCAML1,CNTN4,PCDHAC2,CHODL,DSCAM,CDH4,CNTN6,CRPPA,CNTN5,PTPRM,NRXN3,AUTS2,DAB1,PTPRD,ROBO2,BMPR1B,DCC,CTNNA2*
GO:0097485	neuron projection guidance	0.00008645769701686746	*NRXN1,SLIT2,DSCAML1,CNTN4,PCDHAC2,DSCAM,CDH4,CNTN6,CRPPA,CNTN5,PTPRM,NRXN3,PTPRD,ROBO2,BMPR1B,DCC*
GO:0007411	axon guidance	0.00008645769701686746	*NRXN1,SLIT2,DSCAML1,CNTN4,PCDHAC2,DSCAM,CDH4,CNTN6,CRPPA,CNTN5,PTPRM,NRXN3,PTPRD,ROBO2,BMPR1B,DCC*
GO:0120035	regulation of plasma membrane bounded cell projection organization	0.00014557724825100052	*NEGR1,NRXN1,NTRK2,PTPRG,FGF13,DMD,ARHGAP24,SLIT2,TNIK,MYO3B,CHODL,DSCAM,DDR2,CDH4,ULK4,TOX,PPFIA2,AUTS2,SDCCAG8,DAB1,TENM2,PTPRD,FAT3,ROBO2,DCC,CTNNA2*
GO:0061564	axon development	0.00015536443628009247	*NRXN1,NTRK2,FGF13,SLIT2,DSCAML1,CNTN4,PCDHAC2,CHODL,DSCAM,CDH4,CNTN6,CRPPA,CNTN5,GRM7,PTPRM,NRXN3,AUTS2,DAB1,PTPRD,ROBO2,BMPR1B,DCC,CTNNA2*

GO:0097120	receptor localization to synapse	0.00015812068988094546	*NRXN1,NBEA,ERBB4,GPHN,ADAM22,GPC6,NRXN3,DLG2,CEP112*
GO:0050808	synapse organization	0.0001599027679364596	*NEGR1,NRXN1,NTRK2,NBEA,NRG3,FGF13,ERBB4,GPHN,DGKB,CDH6,DSCAM,LRFN5,FRMPD4,CNTN5,PPFIA2,NRXN3,SLC8A3,PTPRD,DOCK10,ROBO2,CTNNA2,DNER*
GO:0031344	regulation of cell projection organization	0.0002336103628397868	*NEGR1,NRXN1,NTRK2,PTPRG,FGF13,DMD,ARHGAP24,SLIT2,TNIK,MYO3B,CHODL,DSCAM,DDR2,CDH4,ULK4,TOX,PPFIA2,AUTS2,SDCCAG8,DAB1,TENM2,PTPRD,FAT3,ROBO2,DCC,CTNNA2*
GO:0010975	regulation of neuron projection development	0.00026700734078554284	*NEGR1,NRXN1,NTRK2,PTPRG,FGF13,DMD,SLIT2,TNIK,CHODL,DSCAM,DDR2,CDH4,ULK4,TOX,PPFIA2,DAB1,PTPRD,FAT3,ROBO2,DCC,CTNNA2*
GO:0099536	synaptic signaling	0.0003868924669279174	*GRIK2,CADPS2,BTBD9,NRXN1,GRM3,NTRK2,NRG3,DMD,HTR2C,CNTN4,DGKB,CELF4,UTRN,PPP1R9A,RIMBP2,RIMS1,PLCB4,PPFIA2,GRM7,NRXN3,COMT,SLC8A3,CADPS,DLG2,PTPRD,PLCL1,RGS10,DCC*
GO:0007420	brain development	0.0004257943610349892	*KDM4B,NRXN1,NTRK2,NRG3,NCOA1,ATP2B4,SOX6,FGF13,DMD,SLIT2,ERBB4,DSCAML1,CNTN4,CNTNAP2,PCSK1,TOX,CNTN5,CPLANE1,WNT2,MACROD2,COMT,DAB1,RORA,SCN2A,ROBO2,CTNNA2,DNAH5*
GO:0060322	head development	0.0015275721953906989	*KDM4B,NRXN1,NTRK2,NRG3,NCOA1,ATP2B4,SOX6,FGF13,DMD,SLIT2,ERBB4,DSCAML1,CNTN4,CNTNAP2,PCSK1,TOX,CNTN5,CPLANE1,WNT2,MACROD2,COMT,DAB1,RORA,SCN2A,ROBO2,CTNNA2,DNAH5*
GO:0007416	synapse assembly	0.0016662954348922118	*NEGR1,NRXN1,NTRK2,NRG3,FGF13,ERBB4,DSCAM,LRFN5,CNTN5,NRXN3,PTPRD,ROBO2,DNER*
GO:0021953	central nervous system neuron differentiation	0.0018635566877991644	*NRXN1,NTRK2,SLIT2,ERBB4,AGBL4,BRINP3,TOX,WNT2,MDGA2,RORA,ROBO2,BMPR1B,DCC*
GO:0007268	chemical synaptic transmission	0.002101278535865587	*GRIK2,CADPS2,BTBD9,NRXN1,GRM3,NTRK2,NRG3,DMD,HTR2C,CNTN4,DGKB,CELF4,PPP1R9A,RIMBP2,RIMS1,PLCB4,PPFIA2,GRM7,NRXN3,COMT,SLC8A3,CADPS,DLG2,PTPRD,PLCL1,DCC*
GO:0098916	anterograde trans-synaptic signaling	0.002101278535865587	*GRIK2,CADPS2,BTBD9,NRXN1,GRM3,NTRK2,NRG3,DMD,HTR2C,CNTN4,DGKB,CELF4,PPP1R9A,RIMBP2,RIMS1,PLCB4,PPFIA2,GRM7,NRXN3,COMT,SLC8A3,CADPS,DLG2,PTPRD,PLCL1,DCC*
GO:0001964	startle response	0.00237998392494136	*NRXN1,CNTNAP2,PCDH15,COMT,CSMD1,CTNNA2*
GO:0099537	trans-synaptic signaling	0.0024345553127522956	*GRIK2,CADPS2,BTBD9,NRXN1,GRM3,NTRK2,NRG3,DMD,HTR2C,CNTN4,DGKB,CELF4,PPP1R9A,RIMBP2,RIMS1,PLCB4,PPFIA2,GRM7,NRXN3,COMT,SLC8A3,CADPS,DLG2,PTPRD,PLCL1,DCC*

GO:0001764	neuron migration	0.003622529441139881	*NTRK2,NRG3,FGF13,ASTN1,ULK4,AUTS2,SDCCAG8,DAB1,FAT3,DCC,CTNNA2,DNER*
GO:0007267	cell-cell signaling	0.004257260570279139	*GRIK2,CADPS2,BTBD9,NRXN1,GRM3,NTRK2,NRG3,PHEX,FGF13,DMD,SH2D1A,HTR2C,CNTN4,ANK2,DGKB,FGF14,CELF4,PCSK1,UTRN,PPP1R9A,RIMBP2,RIMS1,PLCB4,PPFIA2,WNT2,GRM7,NRXN3,COMT,SLC8A3,CADPS,DLG2,PTPRD,PLCL1,RYR2,RGS10,DCC*
GO:0051128	regulation of cellular component organization	0.008905946712873912	*RNF103-CHMP3,MKLN1,NEGR1,BTBD9,NRXN1,NTRK2,NRG3,PTPRG,PDE4DIP,FGF13,PATJ,GPC3,DMD,ARHGAP24,SLIT2,TNIK,MYO3B,SGIP1,DGKB,CHODL,DSCAM,COLEC10,IQSEC1,ANK1,CNTNAP2,DDR2,CDH4,NSMCE2,LRFN5,ULK4,DYNC1H1,FRMPD4,TOX,ACE2,CHMP3,PDE3A,RIMS1,PPFIA2,NAV3,AUTS2,DOCK2,SDCCAG8,SSH2,CDH13,DAB1,TENM2,PTPRD,FAT3,MPDZ,ROBO2,DCC,CTNNA2,NRG1*
GO:0050804	modulation of chemical synaptic transmission	0.01261343242231277	*GRIK2,BTBD9,NRXN1,GRM3,NTRK2,NRG3,CNTN4,DGKB,CELF4,PPP1R9A,RIMS1,PLCB4,PPFIA2,GRM7,NRXN3,SLC8A3,PTPRD,PLCL1,DCC*
GO:0099177	regulation of trans-synaptic signaling	0.012984529484148754	*GRIK2,BTBD9,NRXN1,GRM3,NTRK2,NRG3,CNTN4,DGKB,CELF4,PPP1R9A,RIMS1,PLCB4,PPFIA2,GRM7,NRXN3,SLC8A3,PTPRD,PLCL1,DCC*
GO:0048513	animal organ development	0.01653221861694307	*PAX7,KDM4B,NRXN1,NTRK2,NRG3,NCOA1,ATP2B4,PHEX,SOX6,FGF13,GPC3,DMD,SLIT2,FAM20C,KAZN,ERBB4,KIF26B,MYO3B,DSCAML1,CNTN4,TCF12,ANK2,CHODL,SOX5,DSCAM,CNTNAP2,DDR2,TRPS1,CELF4,MITF,PCSK1,UTRN,RBFOX1,EDAR,TOX,PCDH15,CNTN5,CPLANE1,WNT2,SGCD,PTPRM,LPIN1,MACROD2,COMT,DAB1,HDAC9,CSMD1,SCEL,FREM2,RORA,BRIP1,NELL1,SCN2A,RYR2,FAT3,ROBO2,PAX3,BMPR1B,CTNNA2,DNER,NRG1,DNAH5*
GO:1903779	regulation of cardiac conduction	0.019250455551654333	*ATP2B4,ANK2,ACE2,SLC8A3,RYR2*
GO:0008038	neuron recognition	0.028155416526814872	*DSCAML1,CNTN4,DSCAM,CNTNAP2,CNTN6,ROBO2*
GO:0007610	behavior	0.02878714770417524	*GRIK2,NEGR1,BTBD9,NRXN1,NTRK2,NCOA1,FGF13,DMD,ASTN1,HTR2C,SGIP1,DSCAM,CNTNAP2,ADAM22,PCDH15,ACE2,NRXN3,COMT,DAB1,SLC8A3,CSMD1,SCN2A*
GO:0007154	cell communication	0.03341245162946985	*ZNF385B,GRIK2,FSTL5,CADPS2,MKLN1,BTBD9,MAST4,NRXN1,MAGI1,FAM13A,GRM3,TAFA1,NTRK2,RASGEF1B,NRG3,PTPRG,NCOA1,ATP2B4,PHEX,TMEM64,FGF13,PATJ,GPC3,DMD,ADCY2,ATRNL1,ARHGAP24,SLIT2,FAM20C,ERBB4,TNIK,SH2D1A,SH3RF3,HTR2C,CNTN4,RAP1GAP2,ITGA1,ESRRG,ANK2,CD226,DGKB,CDH6,FGF14,DSCAM,DENND1B,COLEC10,IQSEC1,ANK1,CNTNAP2,TRIM71,TBCK,DDR2,GPC6,CELF4,CNTN6,MITF,PCSK1,UTRN,GPC5,ULK4,PPP1R9A,TMEM117,SMAD9,EDAR,RIMBP2,FHIT,*

			ACE2,PDE4D,PDE3A,RIMS1,PLCB4,PPFIA2,WNT2,PLPPR1,SGCD,GRM7,PTPRM,NRXN3,AUTS2,COMT,DOCK2,CDH13,DAB1,RFTN1,SLC8A3,TENM2,CADPS,RGS7,SCEL,CTNNA3,FREM2,RNF220,DLG2,RORA,PTPRD,BRIP1,PLCL1,SCN2A,DOCK10,RYR2,KLHDC10,RGS9,ROBO2,RGS10,BMPR1B,DCC,DNER,NRG1
GO:0006942	regulation of striated muscle contraction	0.03371236296015852	*FGF13,DMD,ANK2,ACE2,PDE4D,SLC8A3,CTNNA3,RYR2*
GO:0070593	dendrite self-avoidance	0.03452132602975487	*DSCAML1,CNTN4,DSCAM,CNTN6*
GO:0099633	protein localization to postsynaptic specialization membrane	0.03459504460220107	*NBEA,ERBB4,ADAM22,GPC6,NRXN3*
GO:0099645	neurotransmitter receptor localization to postsynaptic specialization membrane	0.03459504460220107	*NBEA,ERBB4,ADAM22,GPC6,NRXN3*
GO:0031503	protein-containing complex localization	0.03531183965462704	*NRXN1,NBEA,ERBB4,GPHN,ADAM22,GPC6,SGCD,NRXN3,DLG2,CEP112,NRG1*
GO:0008037	cell recognition	0.03585959430188735	*DSCAML1,CNTN4,CD226,DSCAM,COLEC10,CNTNAP2,CNTN6,PCDHA7,DOCK2,ROBO2*
GO:0007043	cell-cell junction assembly	0.04002136295754816	*PATJ,CDH18,ANK2,CDH6,CNTNAP2,CDH12,CDH4,ACE2,CDH13,MPDZ*
GO:0016339	calcium-dependent cell-cell adhesion via plasma membrane cell adhesion molecules	0.04118414260052329	*NRXN1,CDH18,CDH6,CDH12,CDH4,CDH13*
GO:0031346	positive regulation of cell projection organizat	0.048508864385594166	*NEGR1,NRXN1,NTRK2,DMD,SLIT2,MYO3B,CHODL,DSCAM,DDR2,CDH4,TOX,AUTS2,TENM2,PTPRD,ROBO2*

	ion		
		CC	
GO:0030054	cell junction	9.536013542833894e-15	*EPB41L4B,GRIK2,CADPS2,MKLN1,BTBD9,NRXN1,MAGI1,LMO7,GRM3,NTRK2,NBEA,NRG3,ATP2B4,FBLN7,FGF13,PATJ,DMD,ARHGAP24,KAZN,ERBB4,GPHN,TNIK,DSCAML1,HTR2C,ITGA1,CDH18,ANK2,DGKB,CDH6,DSCAM,IQSEC1,ANK1,CNTNAP2,CDH12,DDR2,ADAM22,GPC6,CDH4,CELF4,CNTN6,PCSK1,LRFN5,UTRN,PPP1R9A,FRMPD4,RIMBP2,PCDH15,CNTN5,RIMS1,PLCB4,PPFIA2,GRM7,PTPRM,NRXN3,COMT,SDCCAG8,SSH2,CDH13,SLC8A3,CADM2,TENM2,CADPS,CTNNA3,DLG2,PTPRD,SCN2A,DOCK10,RGS9,MPDZ,RGS10,DCC,CTNNA2,CEP112,NRG1*
GO:0071944	cell periphery	5.439168659210053e-14	*EPB41L4B,TRPM6,ANO5,PCDHA13,GRIK2,MKLN1,NEGR1,PCDHA12,NRXN1,MAGI1,DPP6,LMO7,GRM3,BBS9,NTRK2,NBEA,RASGEF1B,TMX3,NRG3,PTPRG,NCOA1,ATP2B4,PHEX,PDE4DIP,TRPM3,FBLN7,FGF13,PATJ,GPC3,DMD,ADCY2,ASTN1,NALF1,KAZN,ERBB4,GPHN,TNIK,DSCAML1,HTR2C,CNTN4,RAP1GAP2,ITGA1,SGIP1,PCDHA2,PCDHA11,CDH18,ANK2,PCDHA9,CD226,DGKB,PCDHAC2,CDH6,PCDHA10,EDIL3,DSCAM,COL28A1,COLEC10,ANK1,CNTNAP2,OTOGL,ATP6V0D2,PCDHA1,PCDHA8,CDH12,DDR2,ADAM22,GPC6,CDH4,DPP10,CNTN6,PARM1,PCDHAC1,LRFN5,UTRN,GPC5,PCDH7,PPP1R9A,TMEM117,DYNC1H1,EDAR,RIMBP2,FHIT,PCDH15,ACE2,CHMP3,PDE4D,CNTN5,RIMS1,PCDHA7,PCDHA5,PLCB4,PPFIA2,ENOX1,WNT2,GLDC,PLPPR1,SGCD,GRM7,PTPRM,NRXN3,COMT,PCDHA6,DOCK2,CDH13,MDGA2,RFTN1,PCDHA4,SLC8A3,MYO5B,CADM2,TENM2,KCNB2,RGS7,SCEL,FREM2,DLG2,PTPRD,PXDNL,SCN2A,ADK,RYR2,LDLRAD3,FAT3,PCDHA3,RGS9,MPDZ,ROBO2,RGS10,BMPR1B,DCC,CTNNA2,DNER,ETV6,CEP112,NRG1*
GO:0045202	synapse	6.242092062634343e-13	*GRIK2,CADPS2,MKLN1,BTBD9,NRXN1,GRM3,NTRK2,NBEA,NRG3,ATP2B4,DMD,ERBB4,GPHN,TNIK,DSCAML1,HTR2C,ANK2,DGKB,CDH6,DSCAM,IQSEC1,ANK1,CNTNAP2,ADAM22,GPC6,CELF4,CNTN6,PCSK1,LRFN5,UTRN,PPP1R9A,FRMPD4,RIMBP2,PCDH15,CNTN5,RIMS1,PLCB4,PPFIA2,GRM7,NRXN3,COMT,CDH13,SLC8A3,CADM2,TENM2,CADPS,DLG2,PTPRD,SCN2A,DOCK10,RGS9,MPDZ,RGS10,DCC,CEP112,NRG1*
GO:0005886	plasma membrane	1.8363155768200323e-12	*EPB41L4B,TRPM6,ANO5,PCDHA13,GRIK2,NEGR1,PCDHA12,NRXN1,MAGI1,DPP6,LMO7,GRM3,BBS9,NTRK2,NBEA,RASGEF1B,TMX3,NRG3,PTPRG,NCOA1,ATP2B4,PHEX,TRPM3,FGF13,PATJ,GPC3,DMD,ADCY2,ASTN1,NALF1,KAZN,ERBB4,GPHN,TNIK,DSCAML1,HTR2C,CNTN4,RAP1GAP2,ITGA1,SGIP1,PCDHA2,PCDHA11,CDH18,ANK2,PCDHA9,CD226,DGKB,PCDHAC2,CDH6,PCDHA10,DSCAM,COLEC10,ANK1,CNTNAP2,ATP6V0D2,PCDHA1,PCDHA8,CDH12,DDR2,ADAM22,GPC6,CDH4,DPP10,CNTN6,PARM1,PCDHAC1,LRFN5,UTRN,GPC5,PCDH7,TMEM117,EDAR,RIMBP2,FHIT,PCDH15,ACE2,CHMP3,PDE4D,CNTN5,RIMS1,PCDHA7,PCDHA5,PLCB4,PPFIA2,ENOX1,GLDC,PLPPR1,SGCD,GRM7,PTPRM,NRXN3,COMT,PCDHA6,DOCK2,CDH13,MDGA2,RFTN1,PCDHA4,SLC8A3,CADM2,TENM2,KCNB2,RG*

			S7,SCEL,FREM2,DLG2,PTPRD,PXDNL,SCN2A,ADK,RYR2,LDLRAD3,FAT3,PCDHA3,RGS9,MPDZ,ROBO2,RGS10,BMPR1B,DCC,CTNNA2,DNER,ETV6,CEP112,NRG1
GO:0043005	neuron projection	1.2643577100402034e-11	*GRIK2,NEGR1,NRXN1,GRM3,NTRK2,FGF13,DMD,ADCY2,GPHN,MYO3B,DSCAML1,HTR2C,CNTN4,ANK2,DSCAM,ANK1,CNTNAP2,ADAM22,CNTN6,PCSK1,AGBL4,PPP1R9A,DYNC1H1,FRMPD4,BRINP3,PCDH15,CNTN5,PLCB4,PPFIA2,PLPPR1,GRM7,AUTS2,COMT,SDCCAG8,CDH13,SLC8A3,CADM2,TENM2,KCNB2,RGS7,DLG2,SCN2A,DOCK10,FAT3,RGS9,MPDZ,ROBO2,BMPR1B,DCC,CTNNA2,DNER*
GO:0097060	synaptic membrane	7.408348523353843e-11	*GRIK2,NRXN1,GRM3,NBEA,ATP2B4,DMD,ERBB4,GPHN,ANK2,DGKB,ANK1,CNTNAP2,ADAM22,CNTN6,LRFN5,UTRN,CNTN5,RIMS1,PPFIA2,GRM7,NRXN3,TENM2,DLG2,PTPRD,SCN2A,RGS9,DCC*
GO:0042995	cell projection	5.198584599300502e-10	*TRPM6,GRIK2,MKLN1,NEGR1,NRXN1,MAGI1,GRM3,DNAH7,BBS9,NTRK2,ATP2B4,DNAH8,FGF13,DMD,ADCY2,ARHGAP24,GPHN,MYO3B,SPAG16,DSCAML1,HTR2C,CNTN4,ANK2,DSCAM,ANK1,CNTNAP2,ADAM22,CNTN6,PCSK1,UTRN,AGBL4,PPP1R9A,DYNC1H1,FRMPD4,BRINP3,PCDH15,ACE2,CNTN5,RIMS1,CPLANE1,PLCB4,PPFIA2,PLPPR1,GRM7,PTPRM,NRXN3,AUTS2,COMT,SDCCAG8,CDH13,SLC8A3,CADM2,TENM2,KCNB2,RGS7,CTNNA3,DLG2,SCN2A,DOCK10,FAT3,RGS9,MPDZ,ROBO2,BMPR1B,DCC,CTNNA2,DNER,DNAH5*
GO:0120025	plasma membrane bounded cell projection	5.045126357065853e-9	*TRPM6,GRIK2,MKLN1,NEGR1,NRXN1,GRM3,DNAH7,BBS9,NTRK2,ATP2B4,DNAH8,FGF13,DMD,ADCY2,GPHN,MYO3B,SPAG16,DSCAML1,HTR2C,CNTN4,ANK2,DSCAM,ANK1,CNTNAP2,ADAM22,CNTN6,PCSK1,UTRN,AGBL4,PPP1R9A,DYNC1H1,FRMPD4,BRINP3,PCDH15,ACE2,CNTN5,CPLANE1,PLCB4,PPFIA2,PLPPR1,GRM7,PTPRM,AUTS2,COMT,SDCCAG8,CDH13,SLC8A3,CADM2,TENM2,KCNB2,RGS7,CTNNA3,DLG2,SCN2A,DOCK10,FAT3,RGS9,MPDZ,ROBO2,BMPR1B,DCC,CTNNA2,DNER,DNAH5*
GO:0098978	glutamatergic synapse	1.20777951691049e-8	*GRIK2,CADPS2,BTBD9,NRXN1,GRM3,NBEA,NRG3,ATP2B4,ERBB4,TNIK,DGKB,CDH6,ADAM22,GPC6,LRFN5,FRMPD4,PLCB4,PPFIA2,NRXN3,CADPS,PTPRD,SCN2A,DOCK10,RGS9,NRG1*
GO:0016020	membrane	1.3755354697876318e-7	*EPB41L4B,TRPM6,RNF103-CHMP3,ANO5,PCDHA13,GRIK2,TTC3,CADPS2,NEGR1,PCDHA12,NRXN1,MAGI1,ST6GALNAC3,DPP6,LMO7,GRM3,BBS9,NTRK2,NBEA,RASGEF1B,TMX3,NRG3,PTPRG,NCOA1,ATP2B4,PHEX,TRPM3,TMEM64,FGF13,PATJ,GPC3,DMD,ADCY2,ASTN1,ATRNL1,SLIT2,FAM20C,NALF1,KAZN,B3GNT5,ERBB4,GPHN,TNIK,STAG2,DSCAML1,HTR2C,GALNT13,CNTN4,RAP1GAP2,ITGA1,SGIP1,PCDHA2,PCDHA11,CDH18,ANK2,PCDHA9,CD226,DGKB,PRRC2C,PCDHAC2,CDH6,CHODL,PCDHA10,DSCAM,COLEC10,TMEM260,IQSEC1,ANK1,CNTNAP2,DOP1A,ATP6V0D2,PCDHA1,PCDHA8,CDH12,DDR2,ADAM22,GPC6,CDH4,DPP10,ST8SIA6,CNTN6,PARM1,MITF,PCSK1,PCDHAC1,LRFN5,UTRN,GPC5,PCDH7,TMEM117,DYNC1H1,MRPS28,EDAR,RIMBP2,FHIT,PCDH15,ACE2,CHMP3,PDE4D,CNTN5,PDE3A,RIMS1,P*

			CDHA7,TMEM178B,CPLANE1,PCDHA5,PLCB4,PPFIA2,ENOX1,MGAT4C,UXS1,GLDC,PLPPR1,SGCD,GRM7,NAV3,PTPRM,NRXN3,CERS6,LPIN1,COMT,PCDHA6,NBAS,DOCK2,CDH13,MDGA2,RFTN1,PCDHA4,SLC8A3,MYO5B,CADM2,TENM2,KIAA1549L,KCNB2,LYST,CADPS,RGS7,CSMD1,SCEL,FREM2,DLG2,PTPRD,BRIP1,GALNTL6,PXDNL,SCN2A,DOCK10,ADK,TMEM164,RYR2,LDLRAD3,FAT3,PCDHA3,RGS9,MPDZ,ROBO2,RGS10,BMPR1B,DCC,CTNNA2,DNER,ETV6,CEP112,NRG1
GO:0098794	postsynapse	2.3514380037835368e-7	*GRIK2,MKLN1,GRM3,NTRK2,NBEA,DMD,ERBB4,GPHN,TNIK,ANK2,DGKB,IQSEC1,ANK1,ADAM22,CELF4,LRFN5,UTRN,PPP1R9A,FRMPD4,PLCB4,PPFIA2,GRM7,SLC8A3,TENM2,DLG2,DOCK10,RGS9,MPDZ,DCC*
GO:0030424	axon	2.6131953339412577e-7	*GRIK2,NRXN1,GRM3,NTRK2,FGF13,DMD,DSCAML1,CNTN4,DSCAM,ANK1,CNTNAP2,ADAM22,CNTN6,PCSK1,AGBL4,DYNC1H1,CNTN5,PPFIA2,GRM7,AUTS2,COMT,SLC8A3,CADM2,TENM2,DLG2,SCN2A,ROBO2,DCC,CTNNA2*
GO:0098984	neuron to neuron synapse	8.379879415444104e-7	*GRIK2,NRXN1,GRM3,NTRK2,DMD,ERBB4,GPHN,TNIK,IQSEC1,ADAM22,LRFN5,PPP1R9A,FRMPD4,PLCB4,GRM7,SLC8A3,DLG2,PTPRD,RGS9,MPDZ,DCC*
GO:0036477	somatodendritic compartment	0.0000018464719326729756	*GRIK2,NEGR1,NRXN1,GRM3,NTRK2,FGF13,DMD,ADCY2,ASTN1,GPHN,HTR2C,ITGA1,DSCAM,CNTNAP2,PCSK1,PPP1R9A,FRMPD4,BRINP3,PLCB4,PPFIA2,GRM7,COMT,SLC8A3,CADM2,TENM2,KCNB2,DLG2,DOCK10,FAT3,MPDZ,BMPR1B,DNER*
GO:0098590	plasma membrane region	0.000002942184836103807	*TRPM6,GRIK2,NRXN1,LMO7,GRM3,BBS9,NBEA,ATP2B4,PATJ,DMD,ERBB4,GPHN,TNIK,ANK2,DGKB,ANK1,CNTNAP2,ATP6V0D2,DDR2,ADAM22,CNTN6,LRFN5,UTRN,ACE2,PDE4D,CNTN5,RIMS1,PPFIA2,GRM7,NRXN3,CDH13,TENM2,DLG2,PTPRD,SCN2A,RGS9,MPDZ,ROBO2,DCC,NRG1*
GO:0032279	asymmetric synapse	0.000005785430205666026	*GRIK2,GRM3,NTRK2,DMD,ERBB4,GPHN,TNIK,IQSEC1,ADAM22,LRFN5,PPP1R9A,FRMPD4,PLCB4,GRM7,SLC8A3,DLG2,RGS9,MPDZ,DCC*
GO:0030425	dendrite	0.00000799923976122755	*GRIK2,NEGR1,GRM3,NTRK2,FGF13,ADCY2,GPHN,HTR2C,DSCAM,CNTNAP2,PCSK1,PPP1R9A,FRMPD4,BRINP3,PLCB4,PPFIA2,GRM7,COMT,SLC8A3,TENM2,KCNB2,DOCK10,FAT3,MPDZ,BMPR1B,DNER*
GO:0097447	dendritic tree	0.000008528146000266637	*GRIK2,NEGR1,GRM3,NTRK2,FGF13,ADCY2,GPHN,HTR2C,DSCAM,CNTNAP2,PCSK1,PPP1R9A,FRMPD4,BRINP3,PLCB4,PPFIA2,GRM7,COMT,SLC8A3,TENM2,KCNB2,DOCK10,FAT3,MPDZ,BMPR1B,DNER*
GO:0099572	postsynaptic specialization	0.000010144078835148047	*GRIK2,GRM3,NTRK2,DMD,ERBB4,GPHN,TNIK,IQSEC1,ADAM22,LRFN5,PPP1R9A,FRMPD4,PLCB4,PPFIA2,SLC8A3,DLG2,RGS9,MPDZ,DCC*
GO:0045211	postsynaptic membrane	0.000010840894282507175	*GRIK2,GRM3,NBEA,DMD,ERBB4,GPHN,ANK2,DGKB,ANK1,ADAM22,LRFN5,UTRN,GRM7,TENM2,DLG2,RGS9,DCC*
GO:0014069	postsynaptic density	0.000015154983520955791	*GRIK2,GRM3,NTRK2,DMD,ERBB4,GPHN,TNIK,IQSEC1,ADAM22,LRFN5,PPP1R9A,FRMPD4,PLCB4,SLC8A3,DLG2,RGS9,MPDZ,DCC*
GO:0042734	presynaptic	0.00002967864097555745	*GRIK2,NRXN1,GRM3,ATP2B4,ERBB4,CNTN6,CNTN5,RIMS1,PPFIA2,NRXN3,PTPRD,SCN2A,R*

	membrane		*GS9*
GO:0005911	cell-cell junction	0.0002879959464039303	*EPB41L4B,MAGI1,LMO7,FGF13,PATJ,ARHGAP24,KAZN,CDH18,ANK2,CDH6,CNTNAP2,CDH12,CDH4,PTPRM,SDCCAG8,CDH13,CTNNA3,DLG2,SCN2A,MPDZ,CTNNA2*
GO:0098793	presynapse	0.0003230067418830328	*GRIK2,CADPS2,NRXN1,GRM3,NTRK2,ATP2B4,DMD,ERBB4,TNIK,IQSEC1,CNTN6,PCSK1,CNTN5,RIMS1,PPFIA2,GRM7,NRXN3,SLC8A3,CADPS,PTPRD,SCN2A,RGS9*
GO:0016342	catenin complex	0.0004890560010498426	*CDH18,CDH6,CDH12,CDH4,CDH13,CTNNA2*
GO:0005912	adherens junction	0.0007849278067043183	*MAGI1,LMO7,ARHGAP24,CDH18,CDH6,CDH12,CDH4,PTPRM,CDH13,CTNNA3,DLG2,CTNNA2*
GO:0042383	sarcolemma	0.0023565832944179564	*ATP2B4,FGF13,DMD,ANK2,ANK1,UTRN,SGCD,SLC8A3,SCN2A,RYR2*
GO:0070161	anchoring junction	0.00264378890803761	*EPB41L4B,MAGI1,LMO7,FBLN7,FGF13,PATJ,DMD,ARHGAP24,KAZN,ITGA1,CDH18,ANK2,CDH6,CNTNAP2,CDH12,DDR2,CDH4,PTPRM,SDCCAG8,SSH2,CDH13,SLC8A3,CTNNA3,DLG2,SCN2A,MPDZ,CTNNA2*
GO:0098982	GABA-ergic synapse	0.02030721770513117	*NRXN1,NBEA,ERBB4,LRFN5,CNTN5,NRXN3,CDH13*
GO:0099634	postsynaptic specialization membrane	0.033336845502942566	*GRIK2,ERBB4,GPHN,ADAM22,LRFN5,DLG2,RGS9,DCC*
GO:0098797	plasma membrane protein complex	0.04185274699120382	*GRIK2,DPP6,DMD,ITGA1,SGIP1,CDH18,CDH6,CNTNAP2,ATP6V0D2,CDH12,CDH4,DPP10,UTRN,PDE4D,SGCD,CDH13,KCNB2,DLG2,SCN2A,BMPR1B,CTNNA2*
GO:0030054	cell junction	9.536013542833894e-15	*EPB41L4B,GRIK2,CADPS2,MKLN1,BTBD9,NRXN1,MAGI1,LMO7,GRM3,NTRK2,NBEA,NRG3,ATP2B4,FBLN7,FGF13,PATJ,DMD,ARHGAP24,KAZN,ERBB4,GPHN,TNIK,DSCAML1,HTR2C,ITGA1,CDH18,ANK2,DGKB,CDH6,DSCAM,IQSEC1,ANK1,CNTNAP2,CDH12,DDR2,ADAM22,GPC6,CDH4,CELF4,CNTN6,PCSK1,LRFN5,UTRN,PPP1R9A,FRMPD4,RIMBP2,PCDH15,CNTN5,RIMS1,PLCB4,PPFIA2,GRM7,PTPRM,NRXN3,COMT,SDCCAG8,SSH2,CDH13,SLC8A3,CADM2,TENM2,CADPS,CTNNA3,DLG2,PTPRD,SCN2A,DOCK10,RGS9,MPDZ,RGS10,DCC,CTNNA2,CEP112,NRG1*
GO:0071944	cell periphery	5.439168659210053e-14	*EPB41L4B,TRPM6,ANO5,PCDHA13,GRIK2,MKLN1,NEGR1,PCDHA12,NRXN1,MAGI1,DPP6,LMO7,GRM3,BBS9,NTRK2,NBEA,RASGEF1B,TMX3,NRG3,PTPRG,NCOA1,ATP2B4,PHEX,PDE4DIP,TRPM3,FBLN7,FGF13,PATJ,GPC3,DMD,ADCY2,ASTN1,NALF1,KAZN,ERBB4,GPHN,TNIK,DSCAML1,HTR2C,CNTN4,RAP1GAP2,ITGA1,SGIP1,PCDHA2,PCDHA11,CDH18,ANK2,PCDHA9,CD226,DGKB,PCDHAC2,CDH6,PCDHA10,EDIL3,DSCAM,COL28A1,COLEC10,ANK1,CNTNAP2,OTOGL,ATP6V0D2,PCDHA1,PCDHA8,CDH12,DDR2,ADAM22,GPC6,CDH4,DPP10,CNTN6,PARM1,PCDHAC1,LRFN5,UTRN,GPC5,PCDH7,PPP1R9A,TMEM117,DYNC1H1,EDAR,RIMBP2,FHIT,PCDH15,ACE2,CHMP3,PDE4D,CNTN5,RIMS1,PCDHA7,PCDHA5,PLCB4,PPFIA2,ENOX1,WNT2,GLDC,PLPPR1,SGCD,GRM7,PTPRM,NRXN3,COMT,PCDHA6,DOCK2,CDH13,MDGA2,RFTN1,PCDHA4,SLC*

			8A3,MYO5B,CADM2,TENM2,KCNB2,RGS7,SCEL,FREM2,DLG2,PTPRD,PXDNL,SCN2A,ADK,RYR2,LDLRAD3,FAT3,PCDHA3,RGS9,MPDZ,ROBO2,RGS10,BMPR1B,DCC,CTNNA2,DNER,ETV6,CEP112,NRG1
GO:0045202	synapse	6.242092062634343e-13	*GRIK2,CADPS2,MKLN1,BTBD9,NRXN1,GRM3,NTRK2,NBEA,NRG3,ATP2B4,DMD,ERBB4,GPHN,TNIK,DSCAML1,HTR2C,ANK2,DGKB,CDH6,DSCAM,IQSEC1,ANK1,CNTNAP2,ADAM22,GPC6,CELF4,CNTN6,PCSK1,LRFN5,UTRN,PPP1R9A,FRMPD4,RIMBP2,PCDH15,CNTN5,RIMS1,PLCB4,PPFIA2,GRM7,NRXN3,COMT,CDH13,SLC8A3,CADM2,TENM2,CADPS,DLG2,PTPRD,SCN2A,DOCK10,RGS9,MPDZ,RGS10,DCC,CEP112,NRG1*
GO:0005886	plasma membrane	1.8363155768200323e-12	*EPB41L4B,TRPM6,ANO5,PCDHA13,GRIK2,NEGR1,PCDHA12,NRXN1,MAGI1,DPP6,LMO7,GRM3,BBS9,NTRK2,NBEA,RASGEF1B,TMX3,NRG3,PTPRG,NCOA1,ATP2B4,PHEX,TRPM3,FGF13,PATJ,GPC3,DMD,ADCY2,ASTN1,NALF1,KAZN,ERBB4,GPHN,TNIK,DSCAML1,HTR2C,CNTN4,RAP1GAP2,ITGA1,SGIP1,PCDHA2,PCDHA11,CDH18,ANK2,PCDHA9,CD226,DGKB,PCDHAC2,CDH6,PCDHA10,DSCAM,COLEC10,ANK1,CNTNAP2,ATP6V0D2,PCDHA1,PCDHA8,CDH12,DDR2,ADAM22,GPC6,CDH4,DPP10,CNTN6,PARM1,PCDHAC1,LRFN5,UTRN,GPC5,PCDH7,TMEM117,EDAR,RIMBP2,FHIT,PCDH15,ACE2,CHMP3,PDE4D,CNTN5,RIMS1,PCDHA7,PCDHA5,PLCB4,PPFIA2,ENOX1,GLDC,PLPPR1,SGCD,GRM7,PTPRM,NRXN3,COMT,PCDHA6,DOCK2,CDH13,MDGA2,RFTN1,PCDHA4,SLC8A3,CADM2,TENM2,KCNB2,RGS7,SCEL,FREM2,DLG2,PTPRD,PXDNL,SCN2A,ADK,RYR2,LDLRAD3,FAT3,PCDHA3,RGS9,MPDZ,ROBO2,RGS10,BMPR1B,DCC,CTNNA2,DNER,ETV6,CEP112,NRG1*
GO:0043005	neuron projection	1.2643577100402034e-11	*GRIK2,NEGR1,NRXN1,GRM3,NTRK2,FGF13,DMD,ADCY2,GPHN,MYO3B,DSCAML1,HTR2C,CNTN4,ANK2,DSCAM,ANK1,CNTNAP2,ADAM22,CNTN6,PCSK1,AGBL4,PPP1R9A,DYNC1H1,FRMPD4,BRINP3,PCDH15,CNTN5,PLCB4,PPFIA2,PLPPR1,GRM7,AUTS2,COMT,SDCCAG8,CDH13,SLC8A3,CADM2,TENM2,KCNB2,RGS7,DLG2,SCN2A,DOCK10,FAT3,RGS9,MPDZ,ROBO2,BMPR1B,DCC,CTNNA2,DNER*
GO:0097060	synaptic membrane	7.408348523353843e-11	*GRIK2,NRXN1,GRM3,NBEA,ATP2B4,DMD,ERBB4,GPHN,ANK2,DGKB,ANK1,CNTNAP2,ADAM22,CNTN6,LRFN5,UTRN,CNTN5,RIMS1,PPFIA2,GRM7,NRXN3,TENM2,DLG2,PTPRD,SCN2A,RGS9,DCC*
GO:0042995	cell projection	5.198584599300502e-10	*TRPM6,GRIK2,MKLN1,NEGR1,NRXN1,MAGI1,GRM3,DNAH7,BBS9,NTRK2,ATP2B4,DNAH8,FGF13,DMD,ADCY2,ARHGAP24,GPHN,MYO3B,SPAG16,DSCAML1,HTR2C,CNTN4,ANK2,DSCAM,ANK1,CNTNAP2,ADAM22,CNTN6,PCSK1,UTRN,AGBL4,PPP1R9A,DYNC1H1,FRMPD4,BRINP3,PCDH15,ACE2,CNTN5,RIMS1,CPLANE1,PLCB4,PPFIA2,PLPPR1,GRM7,PTPRM,NRXN3,AUTS2,COMT,SDCCAG8,CDH13,SLC8A3,CADM2,TENM2,KCNB2,RGS7,CTNNA3,DLG2,SCN2A,DOCK10,FAT3,RGS9,MPDZ,ROBO2,BMPR1B,DCC,CTNNA2,DNER,DNAH5*
GO:0120025	plasma membrane	5.045126357065853e-9	*TRPM6,GRIK2,MKLN1,NEGR1,NRXN1,GRM3,DNAH7,BBS9,NTRK2,ATP2B4,DNAH8,FGF13,DMD*

	bounded cell projection		*,ADCY2,GPHN,MYO3B,SPAG16,DSCAML1,HTR2C,CNTN4,ANK2,DSCAM,ANK1,CNTNAP2,ADAM22,CNTN6,PCSK1,UTRN,AGBL4,PPP1R9A,DYNC1H1,FRMPD4,BRINP3,PCDH15,ACE2,CNTN5,CPLANE1,PLCB4,PPFIA2,PLPPR1,GRM7,PTPRM,AUTS2,COMT,SDCCAG8,CDH13,SLC8A3,CADM2,TENM2,KCNB2,RGS7,CTNNA3,DLG2,SCN2A,DOCK10,FAT3,RGS9,MPDZ,ROBO2,BMPR1B,DCC,CTNNA2,DNER,DNAH5*
GO:0098978	glutamatergic synapse	1.20777951691049e-8	*GRIK2,CADPS2,BTBD9,NRXN1,GRM3,NBEA,NRG3,ATP2B4,ERBB4,TNIK,DGKB,CDH6,ADAM22,GPC6,LRFN5,FRMPD4,PLCB4,PPFIA2,NRXN3,CADPS,PTPRD,SCN2A,DOCK10,RGS9,NRG1*
GO:0016020	membrane	1.3755354697876318e-7	*EPB41L4B,TRPM6,RNF103-CHMP3,ANO5,PCDHA13,GRIK2,TTC3,CADPS2,NEGR1,PCDHA12,NRXN1,MAGI1,ST6GALNAC3,DPP6,LMO7,GRM3,BBS9,NTRK2,NBEA,RASGEF1B,TMX3,NRG3,PTPRG,NCOA1,ATP2B4,PHEX,TRPM3,TMEM64,FGF13,PATJ,GPC3,DMD,ADCY2,ASTN1,ATRNL1,SLIT2,FAM20C,NALF1,KAZN,B3GNT5,ERBB4,GPHN,TNIK,STAG2,DSCAML1,HTR2C,GALNT13,CNTN4,RAP1GAP2,ITGA1,SGIP1,PCDHA2,PCDHA11,CDH18,ANK2,PCDHA9,CD226,DGKB,PRRC2C,PCDHAC2,CDH6,CHODL,PCDHA10,DSCAM,COLEC10,TMEM260,IQSEC1,ANK1,CNTNAP2,DOP1A,ATP6V0D2,PCDHA1,PCDHA8,CDH12,DDR2,ADAM22,GPC6,CDH4,DPP10,ST8SIA6,CNTN6,PARM1,MITF,PCSK1,PCDHAC1,LRFN5,UTRN,GPC5,PCDH7,TMEM117,DYNC1H1,MRPS28,EDAR,RIMBP2,FHIT,PCDH15,ACE2,CHMP3,PDE4D,CNTN5,PDE3A,RIMS1,PCDHA7,TMEM178B,CPLANE1,PCDHA5,PLCB4,PPFIA2,ENOX1,MGAT4C,UXS1,GLDC,PLPPR1,SGCD,GRM7,NAV3,PTPRM,NRXN3,CERS6,LPIN1,COMT,PCDHA6,NBAS,DOCK2,CDH13,MDGA2,RFTN1,PCDHA4,SLC8A3,MYO5B,CADM2,TENM2,KIAA1549L,KCNB2,LYST,CADPS,RGS7,CSMD1,SCEL,FREM2,DLG2,PTPRD,BRIP1,GALNTL6,PXDNL,SCN2A,DOCK10,ADK,TMEM164,RYR2,LDLRAD3,FAT3,PCDHA3,RGS9,MPDZ,ROBO2,RGS10,BMPR1B,DCC,CTNNA2,DNER,ETV6,CEP112,NRG1*
GO:0098794	postsynapse	2.3514380037835368e-7	*GRIK2,MKLN1,GRM3,NTRK2,NBEA,DMD,ERBB4,GPHN,TNIK,ANK2,DGKB,IQSEC1,ANK1,ADAM22,CELF4,LRFN5,UTRN,PPP1R9A,FRMPD4,PLCB4,PPFIA2,GRM7,SLC8A3,TENM2,DLG2,DOCK10,RGS9,MPDZ,DCC*
GO:0030424	axon	2.6131953339412577e-7	*GRIK2,NRXN1,GRM3,NTRK2,FGF13,DMD,DSCAML1,CNTN4,DSCAM,ANK1,CNTNAP2,ADAM22,CNTN6,PCSK1,AGBL4,DYNC1H1,CNTN5,PPFIA2,GRM7,AUTS2,COMT,SLC8A3,CADM2,TENM2,DLG2,SCN2A,ROBO2,DCC,CTNNA2*
GO:0098984	neuron to neuron synapse	8.379879415444104e-7	*GRIK2,NRXN1,GRM3,NTRK2,DMD,ERBB4,GPHN,TNIK,IQSEC1,ADAM22,LRFN5,PPP1R9A,FRMPD4,PLCB4,GRM7,SLC8A3,DLG2,PTPRD,RGS9,MPDZ,DCC*
GO:0036477	somatodendritic compartment	0.0000018464719326729756	*GRIK2,NEGR1,NRXN1,GRM3,NTRK2,FGF13,DMD,ADCY2,ASTN1,GPHN,HTR2C,ITGA1,DSCAM,CNTNAP2,PCSK1,PPP1R9A,FRMPD4,BRINP3,PLCB4,PPFIA2,GRM7,COMT,SLC8A3,CADM2,TENM2,KCNB2,DLG2,DOCK10,FAT3,MPDZ,BMPR1B,DNER*
GO:009859	plasma	0.00000294218	*TRPM6,GRIK2,NRXN1,LMO7,GRM3,BBS9,NBEA*

0	membrane region	4836103807	*,ATP2B4,PATJ,DMD,ERBB4,GPHN,TNIK,ANK2,DGKB,ANK1,CNTNAP2,ATP6V0D2,DDR2,ADAM22,CNTN6,LRFN5,UTRN,ACE2,PDE4D,CNTN5,RIMS1,PPFIA2,GRM7,NRXN3,CDH13,TENM2,DLG2,PTPRD,SCN2A,RGS9,MPDZ,ROBO2,DCC,NRG1*
GO:0032279	asymmetric synapse	0.000005785430205666026	*GRIK2,GRM3,NTRK2,DMD,ERBB4,GPHN,TNIK,IQSEC1,ADAM22,LRFN5,PPP1R9A,FRMPD4,PLCB4,GRM7,SLC8A3,DLG2,RGS9,MPDZ,DCC*
GO:0030425	dendrite	0.00000799923976122755	*GRIK2,NEGR1,GRM3,NTRK2,FGF13,ADCY2,GPHN,HTR2C,DSCAM,CNTNAP2,PCSK1,PPP1R9A,FRMPD4,BRINP3,PLCB4,PPFIA2,GRM7,COMT,SLC8A3,TENM2,KCNB2,DOCK10,FAT3,MPDZ,BMPR1B,DNER*
GO:0097447	dendritic tree	0.000008528146000266637	*GRIK2,NEGR1,GRM3,NTRK2,FGF13,ADCY2,GPHN,HTR2C,DSCAM,CNTNAP2,PCSK1,PPP1R9A,FRMPD4,BRINP3,PLCB4,PPFIA2,GRM7,COMT,SLC8A3,TENM2,KCNB2,DOCK10,FAT3,MPDZ,BMPR1B,DNER*
GO:0099572	postsynaptic specialization	0.000010144078835148047	*GRIK2,GRM3,NTRK2,DMD,ERBB4,GPHN,TNIK,IQSEC1,ADAM22,LRFN5,PPP1R9A,FRMPD4,PLCB4,PPFIA2,SLC8A3,DLG2,RGS9,MPDZ,DCC*
GO:0045211	postsynaptic membrane	0.000010840894282507175	*GRIK2,GRM3,NBEA,DMD,ERBB4,GPHN,ANK2,DGKB,ANK1,ADAM22,LRFN5,UTRN,GRM7,TENM2,DLG2,RGS9,DCC*
GO:0014069	postsynaptic density	0.000015154983520955791	*GRIK2,GRM3,NTRK2,DMD,ERBB4,GPHN,TNIK,IQSEC1,ADAM22,LRFN5,PPP1R9A,FRMPD4,PLCB4,SLC8A3,DLG2,RGS9,MPDZ,DCC*
GO:0042734	presynaptic membrane	0.000029678640975557 45	*GRIK2,NRXN1,GRM3,ATP2B4,ERBB4,CNTN6,CNTN5,RIMS1,PPFIA2,NRXN3,PTPRD,SCN2A,RGS9*
GO:0005911	cell-cell junction	0.0002879959464039303	*EPB41L4B,MAGI1,LMO7,FGF13,PATJ,ARHGAP24,KAZN,CDH18,ANK2,CDH6,CNTNAP2,CDH12,CDH4,PTPRM,SDCCAG8,CDH13,CTNNA3,DLG2,SCN2A,MPDZ,CTNNA2*
GO:0098793	presynapse	0.0003230067418830328	*GRIK2,CADPS2,NRXN1,GRM3,NTRK2,ATP2B4,DMD,ERBB4,TNIK,IQSEC1,CNTN6,PCSK1,CNTN5,RIMS1,PPFIA2,GRM7,NRXN3,SLC8A3,CADPS,PTPRD,SCN2A,RGS9*
GO:0016342	catenin complex	0.0004890560010498426	*CDH18,CDH6,CDH12,CDH4,CDH13,CTNNA2*
GO:0005912	adherens junction	0.0007849278067043183	*MAGI1,LMO7,ARHGAP24,CDH18,CDH6,CDH12,CDH4,PTPRM,CDH13,CTNNA3,DLG2,CTNNA2*
GO:0042383	sarcolemma	0.0023565832944179564	*ATP2B4,FGF13,DMD,ANK2,ANK1,UTRN,SGCD,SLC8A3,SCN2A,RYR2*
GO:0070161	anchoring junction	0.00264378890803761	*EPB41L4B,MAGI1,LMO7,FBLN7,FGF13,PATJ,DMD,ARHGAP24,KAZN,ITGA1,CDH18,ANK2,CDH6,CNTNAP2,CDH12,DDR2,CDH4,PTPRM,SDCCAG8,SSH2,CDH13,SLC8A3,CTNNA3,DLG2,SCN2A,MPDZ,CTNNA2*
GO:0098982	GABA-ergic synapse	0.02030721770513117	*NRXN1,NBEA,ERBB4,LRFN5,CNTN5,NRXN3,CDH13*
GO:0099634	postsynaptic specialization membrane	0.033336845502942566	*GRIK2,ERBB4,GPHN,ADAM22,LRFN5,DLG2,RGS9,DCC*
GO:0098797	plasma membrane protein	0.04185274699120382	*GRIK2,DPP6,DMD,ITGA1,SGIP1,CDH18,CDH6,CNTNAP2,ATP6V0D2,CDH12,CDH4,DPP10,UTRN,PDE4D,SGCD,CDH13,KCNB2,DLG2,SCN2A,*

	complex		*BMPR1B,CTNNA2*

Table S9. The 398 genes that maintained stable contact with rDNA clusters are co-expressed with 55 different lincRNAs. The search was performed in ttps://maayanlab.cloud/Enrichr/enrich# for lncHUB lncRNA Co-Expression. Related to Figures 4 and 5.

**Term**	**Overlap**	**Adjusted P-value**	**Genes**
FGF12-AS3	15/100	2.915126820682806E-6	*PCDHA13;PCDHA12;PCDHA11;PCDHA10;PCDHA1;PCDHA5;PCDHA4;PCDHA3;PCDHA2;PCDHA9;PCDHA8;PCDHAC2;PCDHA7;PCDHAC1;PCDHA6*
LINC01651	13/100	7.170215834704184E-5	*GALNT13;PLPPR1;DSCAM;CADM2;NRXN1;TCF12;PCDH15;ADAM22;GRIK2;DNER;SOX6;DSCAML1;ASTN1*
CXXC4-AS1	13/100	7.170215834704184E-5	*GALNT13;DSCAM;DGKB;CADM2;NRXN1;TCF12;PCDH15;ADAM22;GRIK2;DPP6;DNER;DSCAML1;ASTN1*
LINC00689	11/100	0.0014031601763771964	*GALNT13;DSCAM;DGKB;CADM2;DNER;TCF12;PCDH15;ADAM22;GRIK2;SOX6;DSCAML1*
LINC01572	11/100	0.0014031601763771964	*GALNT13;PLPPR1;DSCAM;CADM2;NRXN1;DNER;TCF12;ADAM22;GRIK2;DSCAML1;ASTN1*
FRMPD3-AS1	11/100	0.0014031601763771964	*NTRK2;GALNT13;DSCAM;CADM2;NRXN1;DNER;TCF12;ADAM22;ADCY2;GRIK2;ASTN1*
SLC6A1-AS1	11/100	0.0014031601763771964	*GALNT13;PLPPR1;DSCAM;CADM2;NRXN1;DNER;TCF12;ADAM22;GRIK2;DSCAML1;ASTN1*
MAPT-AS1	11/100	0.0014031601763771964	*GALNT13;DSCAM;CADM2;NRXN1;DNER;TCF12;ADAM22;ADCY2;GRIK2;DSCAML1;ASTN1*
LINC02520	10/100	0.004389066882134769	*GALNT13;PLPPR1;DSCAM;CADM2;DNER;TCF12;ADAM22;GPC3;GRIK2;DSCAML1*
LINC01602	10/100	0.004389066882134769	*GALNT13;DSCAM;DGKB;DNER;TCF12;GRIK2;SOX6;TOX;MDGA2;DSCAML1*
LINC01102	10/100	0.004389066882134769	*DPP6;GALNT13;PLPPR1;DSCAM;CADM2;DNER;ADAM22;GRIK2;DSCAML1;ASTN1*
LINC02151	10/100	0.004389066882134769	*SLC8A3;DPP6;GALNT13;PLPPR1;DSCAM;DNER;ADAM22;GRIK2;DSCAML1;ASTN1*
LINC01741	10/100	0.004389066882134769	*GALNT13;PLPPR1;DSCAM;DGKB;DNER;TCF12;GRIK2;SOX6;TOX;DSCAML1*
LINC02008	10/100	0.004389066882134769	*GALNT13;DSCAM;CADM2;NRXN1;DNER;TCF12;ADAM22;GRIK2;DSCAML1;ASTN1*
LINC02598	10/100	0.004389066882134769	*NTRK2;GALNT13;PLPPR1;DSCAM;TCF12;PCDH15;ADAM22;ADCY2;GRIK2;DSCAML1*
DSCAM-IT1	10/100	0.004389066882134769	*GALNT13;PLPPR1;DSCAM;CADM2;DNER;TCF12;PCDH15;GRIK2;SOX6;DSCAML1*

LINC02731	9/100	0.011298235283639104	*DPP6;GALNT13;DSCAM;CADM2;DNER;ADAM22;GRIK2;DSCAML1;ASTN1*
LINC00237	9/100	0.011298235283639104	*GALNT13;PLPPR1;DSCAM;CADM2;DNER;TCF12;ADAM22;GRIK2;DSCAML1*
LINC00368	9/100	0.011298235283639104	*DPP6;GALNT13;DSCAM;CADM2;NRXN1;PCDH15;ADAM22;DSCAML1;ASTN1*
LINC01323	9/100	0.011298235283639104	*GALNT13;DLG2;DSCAM;CADM2;PCDH15;ADAM22;CTNNA3;GRIK2;DSCAML1*
LINC02283	9/100	0.011298235283639104	*GALNT13;PLPPR1;DSCAM;CADM2;DNER;TCF12;PCDH15;GRIK2;DSCAML1*
LINC02110	9/100	0.011298235283639104	*GALNT13;DSCAM;CADM2;DNER;ADAM22;GRIK2;SOX6;DSCAML1;ASTN1*
LINC01415	9/100	0.011298235283639104	*GALNT13;DSCAM;CADM2;NRXN1;DNER;TCF12;ADAM22;GRIK2;ASTN1*
LINC01785	9/100	0.011298235283639104	*GRM3;AGBL4;RBFOX1;DLG2;FRMPD4;GPC5;SCN2A;NOL4;RGS7*
LINC02339	9/100	0.011298235283639104	*GALNT13;PLPPR1;DSCAM;CADM2;NRXN1;DNER;ADAM22;GRIK2;DSCAML1*
ANKRD62P1-PARP4P3	9/100	0.011298235283639104	*NTRK2;GALNT13;DSCAM;CADM2;TRPS1;TCF12;TTC3;PCDH15;DSCAML1*
ZNF32-AS3	9/100	0.011298235283639104	*GALNT13;DSCAM;CADM2;NRXN1;TCF12;TTC3;ADAM22;DSCAML1;ASTN1*
ARHGEF7-AS1	9/100	0.011298235283639104	*NCOA1;DPP6;NBEA;CADM2;NRXN1;TCF12;ADAM22;MPDZ;NOL4*
ASIC4-AS1	9/100	0.011298235283639104	*GALNT13;PLPPR1;DSCAM;CADM2;DNER;TCF12;ADAM22;GRIK2;DSCAML1*
NCAM1-AS1	9/100	0.011298235283639104	*DPP6;GALNT13;DSCAM;CADM2;NRXN1;DNER;ADAM22;GRIK2;ASTN1*
ARHGEF7-IT1	9/100	0.011298235283639104	*NCOA1;MKLN1;NBEA;TCF12;TTC3;ADAM22;ZBTB20;FAT3;MPDZ*
ZBTB20-AS4	9/100	0.011298235283639104	*NCOA1;MKLN1;GALNT13;KMT2C;DOP1A;TCF12;TTC3;ADAM22;ZBTB20*
LRP4-AS1	9/100	0.011298235283639104	*GALNT13;DSCAM;CADM2;NRXN1;TCF12;ADAM22;GRIK2;DSCAML1;ASTN1*
GDNF-AS1	9/100	0.011298235283639104	*GALNT13;DSCAM;PCDH15;ADAM22;GRIK2;SOX6;MDGA2;DSCAML1;ASTN1*
ADGRA1-AS1	9/100	0.011298235283639104	*DPP6;GALNT13;DSCAM;CADM2;NRXN1;PCDH15;ADAM22;DSCAML1;ASTN1*
LINC01546	8/100	0.0361285740711499 1	*GALNT13;PLPPR1;DSCAM;DNER;ADAM22;GRIK2;DSCAML1;ASTN1*

LINC01677	8/100	0.0361285740711499 1	*NTRK2;GALNT13;DSCAM;CADM2;DNER;ADAM22;GRIK2;DSCAML1*
LINC01069	8/100	0.0361285740711499 1	*SGCD;NRG3;PIR;PAX3;MITF;SOX6;MDGA2;SOX5*
LINC02827	8/100	0.0361285740711499 1	*EDAR;DENND1B;PATJ;CERS6;AUTS2;TRPS1;ZNF638;CPLANE1*
LINC02117	8/100	0.0361285740711499 1	*DPP6;GALNT13;CADM2;ADAM22;RIMBP2;GRIK2;SOX6;ASTN1*
LINC00599	8/100	0.0361285740711499 1	*GALNT13;DSCAM;CADM2;NRXN1;DNER;ADAM22;GRIK2;DSCAML1*
LINC02552	8/100	0.0361285740711499 1	*NELL1;SGCD;NRG3;PCDH7;PAX3;MITF;MDGA2;SOX5*
KIRREL3-AS3	8/100	0.0361285740711499 1	*RBFOX1;DLG2;FRMPD4;TAFA1;CELF4;SCN2A;DYNC1I1;RGS7*
RORB-AS1	8/100	0.0361285740711499 1	*RBFOX1;DLG2;FRMPD4;CTNNA2;SCN2A;PPFIA2;RGS7;ASTN1*
GPR158-AS1	8/100	0.0361285740711499 1	*SLC8A3;DPP6;GALNT13;CADM2;DNER;ADAM22;GRIK2;ASTN1*
OBI1-AS1	8/100	0.0361285740711499 1	*NTRK2;GALNT13;CADM2;NRXN1;TCF12;PCDH15;ADAM22;ADCY2*
ARHGEF7-AS2	8/100	0.0361285740711499 1	*DPP6;CADM2;NRXN1;DNER;ADAM22;DSCAML1;NOL4;ASTN1*
CPB2-AS1	8/100	0.0361285740711499 1	*GALNT13;PLPPR1;DSCAM;CADM2;NRXN1;DNER;GRIK2;ASTN1*
NRXN2-AS1	8/100	0.0361285740711499 1	*GALNT13;DSCAM;CADM2;NRXN1;DNER;ADAM22;GRIK2;ASTN1*
ADAMTS9-AS2	8/100	0.0361285740711499 1	*CEP112;FAM13A;ITGA1;ZBTB20;PTPRM;RORA;UTRN;PTPRG*
FGF10-AS1	8/100	0.0361285740711499 1	*PCSK1;FGF14;MAST4;ERBB4;TRPS1;ST8SIA6;BMPR1B;EDIL3*
OPCML-IT1	8/100	0.0361285740711499 1	*PLPPR1;DSCAM;CADM2;DNER;TCF12;PCDH15;GRIK2;DSCAML1*
MIR9-3HG	8/100	0.0361285740711499 1	*GALNT13;DSCAM;CADM2;TCF12;PCDH15;ADAM22;GRIK2;DSCAML1*
CCND2-AS1	8/100	0.0361285740711499 1	*DPP6;GALNT13;DSCAM;CADM2;DNER;ADAM22;DSCAML1;ASTN1*
JAKMIP2-AS1	8/100	0.0361285740711499 1	*DPP6;GALNT13;DSCAM;CADM2;NRXN1;DNER;ADAM22;ASTN1*

Table S10. The 398 genes that maintained stable contact with rDNA clusters are simultaneously regulated in different combinations by 391 different transcription factors. The search was performed in TF-Gene Co-occurrence (Enrichr Submissions TF-Gene Co-occurrence, https://maayanlab.cloud/Enrichr/). Related to Figure 5.

Term	Overlap	Adjusted P-value	Genes
POU6F2	79/299	2.9215419541599807E-63	*ROBO2;DPP10;PCSK1;RYR2;CNTNAP2;TENM2;DGKB;CELF4;PTPRM;ZBTB20;RORA;GRIK2;ADARB2;BACH2;RIMS1;CDH4;GRM7;DNER;MACROD2;SOX6;SOX5;PPFIA2;MAGI1;CADPS2;RBFOX1;DSCAM;DCC;PDE4D;NRG1;ANK1;NAV3;NRG3;CDH12;CDH13;TOX;CDH18;NRXN1;PCDH15;NRXN3;ADCY2;NOL4;HDAC9;FSTL5;DPP6;ERBB4;DMD;CTNNA3;GPC5;CTNNA2;SLIT2;CSMD1;GPC6;TRPM3;DYNC1I1;RAP1GAP2;NTRK2;CNTN5;AUTS2;NEGR1;CADM2;ATRNL1;KCNB2;PCDH7;CADPS;ESRRG;PTPRD;MGAT4C;AGBL4;NELL1;RALYL;DLG2;FGF14;DAB1;NBEA;KIF26B;RIMBP2;FAT3;CNTN4;MDGA2*
ZNF804B	76/299	1.4002958224459074E-59	*ROBO2;DPP10;RYR2;CNTNAP2;TENM2;DGKB;PTPRM;RORA;GRIK2;PTPRG;GRM3;RIMS1;CDH4;GRM7;ZNF385B;MACROD2;EDIL3;SOX5;PPFIA2;RGS7;CADPS2;RBFOX1;DSCAM;DCC;PDE4D;ANK2;NRG1;NAV3;NRG3;CDH12;CDH13;FREM2;CDH18;NRXN1;PCDH15;NRXN3;NOL4;FHIT;HDAC9;FSTL5;DPP6;ERBB4;DMD;CTNNA3;GPC5;CTNNA2;SLIT2;CSMD1;GPC6;BRINP3;TRPM3;CNTN5;AUTS2;NEGR1;CADM2;ATRNL1;CNTN6;KCNB2;PCDH7;CADPS;ESRRG;PTPRD;MGAT4C;AGBL4;NELL1;LRFN5;RALYL;DLG2;FGF14;DAB1;NBEA;KIF26B;FAT3;GALNTL6;CNTN4;MDGA2*
SLC4A10	63/299	1.8453745364388836E-43	*ROBO2;DPP10;RYR2;CNTNAP2;FRMPD4;DGKB;CELF4;RORA;GRIK2;DOCK10;RIMS1;GRM7;DNER;EDIL3;SOX5;PPFIA2;RGS7;CADPS2;RBFOX1;DSCAM;SGIP1;DCC;PDE4D;ANK2;ANK1;PLCB4;NAV3;CDH18;ASTN1;DDR2;NRXN1;ADAM22;NRXN3;ADCY2;NOL4;HDAC9;FSTL5;DPP6;ERBB4;DMD;CTNNA2;CSMD1;BRINP3;TRPM3;DYNC1I1;RAP1GAP2;NTRK2;CNTN5;NEGR1;CADM2;ATRNL1;PCDH7;CADPS;ESRRG;PTPRD;NELL1;RALYL;DLG2;FGF14;NBEA;RIMBP2;CNTN4;SCN2A*
DACH2	60/299	3.734332862842493E-40	*ROBO2;DPP10;PCSK1;RYR2;CNTNAP2;TENM2;GALNT13;FRMPD4;DGKB;CELF4;HTR2C;GRIK2;DNER;SOX6;EDIL3;PPFIA2;RGS7;CADPS2;RBFOX1;DCC;ANK2;ANK1;NAV3;CDH12;TOX;CDH18;DDR2;NRXN1;PCDH15;NRXN3;NOL4;HDAC9;FSTL5;DPP6;ERBB4;GPC3;DMD;CTNNA3;CTNNA2;SLIT2;TRPM3;DYNC1I1;NTRK2;CNTN5;NEGR1;CADM2;CADPS;ESRRG;PTPRD;MGAT4C;NELL1;RALYL;DLG2;FGF14;NBEA;FAT3;CNTN4;SCN2A;FGF13;BMPR1B*
ZNF385D	60/299	3.734332862842493E-40	*ROBO2;RYR2;CNTNAP2;TENM2;DGKB;PTPRM;ZBTB20;RORA;GRIK2;RIMS1;SGCD;TRPS1;DNER;ZNF385B;SOX6;EDIL3;SOX5;MAGI1;CADPS2;RBFOX1;SGIP1;DCC;PDE4D;ANK2;NRG1;PLCB4;NAV3;CDH12;CDH13;TOX;CDH18;ASTN1;DDR2;NRXN1;NRXN3;HDAC9;DPP6;CTNNA3;CTNNA2;SLIT2;CSMD1;GPC6;TRPM3;NTRK2;CNTN5;NEGR1;CADM2;ATRNL1;PCDH7;CADPS;ESRRG;ENOX1;PTPRD;NELL1;LRFN5;DLG2;FGF14;NBEA;PDE3A;CNTN4*
RGS6	59/299	3.81650034949096E-39	*ROBO2;DPP10;RYR2;CNTNAP2;TENM2;PTPRM;ZBTB20;RORA;GRIK2;ADARB2;CDH6;RIMS1;CDH4;GRM7;SOX6;EDIL3;SOX5;RGS7;MAGI1;CADPS2;RBF*

			OX1;DCC;PDE4D;ANK2;NRG1;ANK1;PLCB4;CDH13;DDR2;NRXN1;NRXN3;ADCY2;HDAC9;DPP6;ERBB4;DMD;CTNNA3;SLIT2;CSMD1;GPC6;TRPM3;RAP1GAP2;NTRK2;AUTS2;NEGR1;CADM2;ATRNL1;PCDH7;CADPS;ESRRG;PTPRD;DLG2;FGF14;DAB1;NBEA;KIF26B;PDE3A;FAT3;CNTN4
RGS7	59/299	3.81650034949096E-39	*ROBO2;DPP10;PCSK1;RYR2;CNTNAP2;TENM2;CELF4;RORA;GRIK2;RIMS1;CDH4;GRM7;DNER;SOX5;PPFIA2;CADPS2;RBFOX1;ANK2;NRG1;ANK1;PLCB4;NAV3;CDH13;CDH18;DDR2;NRXN1;NRXN3;ADCY2;NOL4;HDAC9;DPP6;ERBB4;CTNNA2;SLIT2;CSMD1;DYNC1I1;RAP1GAP2;NTRK2;NEGR1;CADM2;ATRNL1;KCNB2;PCDH7;CADPS;ESRRG;PTPRD;NELL1;RALYL;DLG2;FGF14;DAB1;NBEA;PPP2R2C;KIF26B;RIMBP2;FAT3;CNTN4;SCN2A;FGF13*
ESRRG	58/299	4.1468524523700324E-38	*ROBO2;DPP10;RYR2;CNTNAP2;TENM2;CELF4;PTPRM;ZBTB20;RORA;GRIK2;BACH2;CDH6;CDH4;TRPS1;DNER;SOX6;SOX5;MAGI1;CADPS2;RBFOX1;PDE4D;ANK2;LMO7;NRG1;ANK1;PLCB4;CDH13;TOX;DDR2;CASZ1;NRXN1;NRXN3;HDAC9;DPP6;ERBB4;DMD;CTNNA3;CTNNA2;SLIT2;CSMD1;GPC6;TRPM3;RAP1GAP2;NTRK2;AUTS2;NEGR1;CADM2;ATRNL1;PCDH7;CADPS;PTPRD;DLG2;DAB1;NBEA;KIF26B;FAT3;CNTN4;BMPR1B*
MYT1L	58/299	4.1468524523700324E-38	*ROBO2;DPP10;RYR2;CNTNAP2;TENM2;DGKB;CELF4;PTPRM;ZBTB20;RORA;GRIK2;ADARB2;RIMS1;CDH4;GRM7;SOX5;PPFIA2;RGS7;CADPS2;RBFOX1;DSCAM;SGIP1;DCC;PDE4D;DNMT3A;ANK2;ANK1;NAV3;CDH13;ASTN1;KMT2C;NRXN1;NRXN3;NOL4;DPP6;CTNNA2;CSMD1;TRPM3;RAP1GAP2;NTRK2;CNTN5;AUTS2;NEGR1;CADM2;ATRNL1;CADPS;ESRRG;PTPRD;RALYL;DLG2;FGF14;DAB1;NBEA;PPP2R2C;KIF26B;RIMBP2;FAT3;SCN2A*
NPAS3	57/299	5.089373344251292E-37	*ROBO2;CNTNAP2;TENM2;DGKB;PTPRM;ZBTB20;RORA;GRIK2;ADARB2;BACH2;PTPRG;CDH6;CDH4;TRPS1;MACROD2;SOX6;SOX5;MAGI1;CADPS2;RBFOX1;DCC;PDE4D;ANK2;NRG1;ANK1;NAV3;NRG3;CDH13;TNIK;TOX;DDR2;NRXN1;PCDH15;NRXN3;HDAC9;DPP6;ERBB4;CTNNA2;SLIT2;CSMD1;GPC6;TRPM3;NTRK2;AUTS2;NEGR1;CADM2;PCDH7;CADPS;ESRRG;PTPRD;DLG2;FGF14;DAB1;NBEA;KIF26B;FAT3;BMPR1B*
ZIM2	53/299	1.2495234769937264E-32	*ROBO2;DPP10;RYR2;CNTNAP2;TENM2;GALNT13;ZBTB20;RORA;GRIK2;ADARB2;RIMS1;SOX6;SOX5;PPFIA2;CADPS2;RBFOX1;DSCAM;DCC;PDE4D;ANK2;NRG1;ANK1;NAV3;CDH12;CDH13;CDH18;ASTN1;DDR2;NRXN1;PCDH15;NRXN3;FSTL5;DPP6;ERBB4;DMD;CTNNA3;CTNNA2;CSMD1;BRINP3;NTRK2;CNTN5;AUTS2;ATRNL1;PCDH7;PDE4DIP;ESRRG;PTPRD;AGBL4;DLG2;DAB1;NBEA;KIF26B;RIMBP2*
FOXP2	52/299	1.10265112770121E-31	*ROBO2;CNTNAP2;TENM2;PTPRM;ZBTB20;RORA;GRIK2;BACH2;PTPRG;RIMS1;TRPS1;SOX6;EDIL3;SOX5;CADPS2;RBFOX1;DCC;PDE4D;ANK2;MITF;LMO7;NRG1;ANK1;PLCB4;CDH13;TOX;DDR2;NRXN1;NRXN3;HDAC9;ERBB4;DMD;SLIT2;CSMD1;GPC6;NTRK2;AUTS2;NEGR1;CADM2;ATRNL1;PCDH7;ESRRG;ARHGAP24;PTPRD;DLG2;NBEA;KIF26B;PDE3A;FAT3;CNTN4;SCN2A;BMPR1B*
PKHD1	52/299	1.10265112770121E-31	*ROBO2;DPP10;RYR2;CNTNAP2;TENM2;ZBTB20;RORA;GRIK2;ADARB2;CDH6;DOCK10;RIMS1;CDH4;MACROD2;SOX6;SOX5;CADPS2;RBFOX1;DSCAM;DCC;PDE4D;LMO7;ANK1;NRG3;CDH13;FREM2;DDR2;DNAH8;DNAH5;NRXN1;PCDH15;NRXN3;FHIT;HDAC9*

			;DPP6;ERBB4;DMD;CTNNA3;CTNNA2;SLIT2;CSMD1;GPC6;NTRK2;CNTN5;ESRRG;PTPRD;DLG2;DAB1;NBEA;KIF26B;FAT3;BMPR1B
ZMAT4	52/299	1.10265112770121E-31	*ROBO2;DPP10;PCSK1;RYR2;CNTNAP2;TENM2;DGKB;CELF4;GRIK2;CDH6;RIMS1;CDH4;DNER;EDIL3;SOX5;PPFIA2;RGS7;CADPS2;RBFOX1;PDE4D;NRG1;ANK1;NRG3;CDH13;TOX;FREM2;CDH18;NRXN1;NRXN3;ADCY2;HDAC9;DPP6;CTNNA2;SLIT2;CSMD1;NTRK2;CNTN5;AUTS2;NEGR1;CADM2;PCDH7;CADPS;ESRRG;ENOX1;PTPRD;NELL1;DLG2;FGF14;DAB1;FAT3;CNTN4;FGF13*
ZNF536	52/299	1.10265112770121E-31	*ROBO2;RYR2;CNTNAP2;TENM2;CELF4;RORA;GRIK2;ADARB2;BACH2;RIMS1;CDH4;DNER;SOX6;EDIL3;SOX5;PPFIA2;CADPS2;RBFOX1;DCC;PDE4D;ANK2;NRG1;ANK1;NAV3;NRG3;CDH13;TOX;FREM2;ASTN1;DDR2;NRXN1;NRXN3;HDAC9;DPP6;ERBB4;CTNNA2;SLIT2;CSMD1;NTRK2;AUTS2;NEGR1;ATRNL1;PLCL1;PCDH7;CADPS;ESRRG;PTPRD;NELL1;DLG2;DAB1;KIF26B;CNTN4*
SOX5	51/299	1.2120948695435296E-30	*ROBO2;CNTNAP2;TENM2;MAST4;PTPRM;ZBTB20;RORA;GRIK2;BACH2;PTPRG;CDH6;DOCK10;TRPS1;SOX6;EDIL3;MAGI1;CADPS2;RBFOX1;DCC;PDE4D;ANK2;MITF;NRG1;ANK1;PLCB4;NAV3;CDH13;TNIK;UTRN;TOX;DDR2;NRXN1;NRXN3;HDAC9;ERBB4;DMD;SLIT2;CSMD1;GPC6;NTRK2;AUTS2;NEGR1;ATRNL1;PCDH7;ESRRG;ARHGAP24;PTPRD;DLG2;DAB1;NBEA;FAT3*
PLXNA4	49/299	1.4490098263171776E-28	*ROBO2;RYR2;CNTNAP2;TENM2;MAST4;PTPRM;ZBTB20;RORA;GRIK2;BACH2;PTPRG;CDH4;EDIL3;SOX5;MAGI1;CADPS2;RBFOX1;PDE4D;ANK2;LMO7;NRG1;ANK1;NAV3;CDH13;TNIK;DDR2;NRXN1;NRXN3;HDAC9;DPP6;ERBB4;DMD;SLIT2;CSMD1;GPC6;RAP1GAP2;NTRK2;AUTS2;NEGR1;ATRNL1;PCDH7;CADPS;ATP2B4;PTPRD;DLG2;DAB1;NBEA;KIF26B;FAT3*
ST18	48/299	1.4034256175131093E-27	*ROBO2;DPP10;PCSK1;CNTNAP2;TENM2;DGKB;NRXN1;CELF4;NRXN3;ZBTB20;RORA;GRIK2;NOL4;HDAC9;BACH2;DOCK10;FSTL5;DPP6;ERBB4;TRPS1;DNER;CTNNA2;SOX6;EDIL3;TRPM3;SOX5;PPFIA2;CADPS2;NTRK2;RBFOX1;AUTS2;NEGR1;DCC;KCNB2;PLCL1;CADPS;ANK2;ESRRG;ANK1;PTPRD;RALYL;DLG2;NAV3;RIMBP2;FAT3;CNTN4;TOX;DDR2*
ZNF385B	48/299	1.4034256175131093E-27	*ROBO2;RYR2;CNTNAP2;TENM2;PARM1;NRXN1;NRXN3;PTPRM;ZBTB20;RORA;GRIK2;HDAC9;RIMS1;CDH4;DPP6;ERBB4;DNER;DMD;CTNNA3;CTNNA2;MACROD2;SLIT2;CSMD1;GPC6;CADPS2;NTRK2;RBFOX1;AUTS2;NEGR1;CADM2;ATRNL1;PDE4D;PCDH7;CADPS;ANK2;ESRRG;NRG1;ANK1;PTPRD;NELL1;DLG2;NBEA;NAV3;CDH13;FAT3;CNTN4;FGF13;DDR2*
ZNF804A	46/299	1.438481725149118E-25	*ROBO2;RYR2;CNTNAP2;TENM2;PARM1;NRXN1;NRXN3;RORA;GRIK2;HDAC9;DOCK10;FSTL5;RIMS1;ERBB4;TRPS1;DNER;DMD;SLIT2;CSMD1;GPC6;EDIL3;CADPS2;CNTN5;AUTS2;NEGR1;ATRNL1;DCC;PDE4D;PLCL1;PCDH7;ANK2;NRG1;ARHGAP24;PTPRD;NELL1;LRFN5;DLG2;NBEA;NAV3;KIF26B;CDH13;FAT3;CNTN4;SCN2A;TOX;DDR2*
ADAMTS17	45/299	1.3643530685113195E-24	*ROBO2;CASZ1;TENM2;PARM1;MAST4;NRXN1;NRXN3;PTPRM;ZBTB20;ADCY2;RORA;ADARB2;HDAC9;BACH2;PTPRG;CDH4;DPP6;SLIT2;SOX6;CSMD1;GPC6;SOX5;MAGI1;CADPS2;NTRK2;RBFOX1;AUTS2;DCC;PDE4D;PCDH7;KAZN;ANK2;LMO7;ANK1;SH3RF3;PTPRD;DLG2;DAB1;NAV3;MYO5B;KIF26B;CDH*

			13;FAT3;FREM2;DDR2
ADAMTS19	42/299	1.0341910257909115E-21	*ROBO2;RYR2;CNTNAP2;TENM2;PARM1;NRXN1;PCDH15;NRXN3;ADCY2;GRIK2;CDH6;CDH4;ERBB4;GPC3;CTNNA2;SLIT2;CSMD1;EDIL3;CADPS2;NTRK2;RBFOX1;DSCAM;AUTS2;NEGR1;ATRNL1;PDE4D;PCDH7;CADPS;ESRRG;ANK1;ANO5;PTPRD;LRFN5;DLG2;MYO5B;KIF26B;PDE3A;FAT3;CNTN4;FGF13;FREM2;DDR2*
ZNF98	42/299	1.0341910257909115E-21	*ROBO2;DPP10;RYR2;CNTNAP2;TENM2;DNAH7;KMT2C;NRXN1;NRXN3;RORA;GRIK2;CDH4;DPP6;GRM7;TRPS1;PCDHA3;DMD;GPC5;CTNNA2;SLIT2;SOX6;CSMD1;CADPS2;NTRK2;RBFOX1;CNTN5;AUTS2;DCC;PDE4D;PCDH7;PDE4DIP;NRG1;PTPRD;MGAT4C;DAB1;NBEA;PDE3A;CDH13;CNTN4;BMPR1B;FREM2;DDR2*
CAMTA1	41/299	8.425603990529185E-21	*ROBO2;RYR2;CNTNAP2;CASZ1;TENM2;MAST4;NRXN1;CELF4;NRXN3;ZBTB20;RORA;BACH2;PTPRG;CDH4;DPP6;TRPS1;MACROD2;CSMD1;SOX5;MAGI1;RAP1GAP2;CADPS2;NTRK2;RBFOX1;AUTS2;NEGR1;ATRNL1;PDE4D;PCDH7;PDE4DIP;ANK2;LMO7;ESRRG;ANK1;PTPRD;DLG2;DAB1;NBEA;KIF26B;CDH13;DDR2*
PRDM16	41/299	8.425603990529185E-21	*RYR2;CNTNAP2;CASZ1;TENM2;NRXN1;NRXN3;PTPRM;ZBTB20;RORA;BACH2;PTPRG;CDH4;DPP6;ERBB4;TRPS1;DMD;SLIT2;SOX6;CSMD1;SOX5;MAGI1;RAP1GAP2;CADPS2;NTRK2;RBFOX1;AUTS2;PDE4D;PCDH7;DNMT3A;KAZN;ANK2;ESRRG;ANK1;PTPRD;DLG2;DAB1;KIF26B;CDH13;TOX;BMPR1B;DDR2*
ZNF717	40/299	6.957954091157082E-20	*ROBO2;CNTNAP2;PATJ;KMT2C;NRXN1;NRXN3;PTPRM;ZBTB20;RORA;GRIK2;FHIT;HDAC9;BACH2;DOCK10;CDH4;DPP6;ERBB4;DMD;CTNNA2;MACROD2;CSMD1;SOX5;CADPS2;RBFOX1;CNTN5;AUTS2;ATRNL1;PDE4D;PDE4DIP;MITF;NBPF1;PTPRD;LRFN5;PLCB4;NBEA;KIF26B;TNIK;CNTN4;FREM2;DDR2*
ZNF716	39/299	5.577824845411585E-19	*ROBO2;DPP10;RYR2;CNTNAP2;TENM2;NBAS;DGKB;KMT2C;DNAH5;NRXN1;PCDH15;NRXN3;ZBTB20;GRIK2;FSTL5;CDH4;DPP6;SGCD;MACROD2;CSMD1;RBFOX1;DSCAM;CNTN5;AUTS2;CNTN6;DCC;PDE4D;PCDH7;PXDNL;ESRRG;NRG1;PTPRD;DLG2;NRG3;CDH13;FAT3;MDGA2;CDH18;DDR2*
AFF3	38/299	3.917839375560439E-18	*ROBO2;CNTNAP2;MAST4;NRXN3;PTPRM;ZBTB20;RORA;HDAC9;BACH2;DOCK10;TRPS1;SLIT2;SOX6;SOX5;RAP1GAP2;CADPS2;NTRK2;AUTS2;NEGR1;ANKRD44;PDE4D;PCDH7;ATP2B4;ANK2;LMO7;NRG1;ANK1;PTPRD;PLCB4;NBEA;NAV3;KIF26B;CDH13;FAT3;TNIK;UTRN;TOX;DDR2*
CHD6	38/299	3.917839375560439E-18	*ROBO2;RYR2;CNTNAP2;CASZ1;MAST4;KMT2C;NRXN1;CELF4;NRXN3;ZBTB20;RORA;PPP1R9A;BACH2;CDH4;TRPS1;MAGI1;RAP1GAP2;DYNC1H1;CADPS2;NTRK2;AUTS2;PDE4D;PCDH7;DNMT3A;CADPS;ATP2B4;PRRC2C;PDE4DIP;ANK2;LMO7;ANK1;PTPRD;DLG2;NBEA;KIF26B;TNIK;UTRN;DDR2*
CPXCR1	38/299	3.917839375560439E-18	*ROBO2;DPP10;CNTNAP2;TENM2;DGKB;DNAH5;NRXN1;PCDH15;HTR2C;GRIK2;ADARB2;DACH2;NOL4;HDAC9;PHEX;CDH6;FSTL5;DMD;CTNNA3;SLIT2;CSMD1;GPC6;BRINP3;CADPS2;RBFOX1;CNTN5;CADM2;CNTN6;DCC;PCDH7;NRG1;OTOGL;PTPRD;MGAT4C;NAV3;CDH12;CDH18;DDR2*
SOX6	38/299	3.917839375560439E-18	*ROBO2;TENM2;NRXN1;PTPRM;ZBTB20;RORA;PPP1R9A;HDAC9;BACH2;CDH6;ERBB4;TRPS1;DMD;SLIT2;GPC6;SOX5;MAGI1;CADPS2;NTRK2;AUTS2;PDE4D;PCDH7;ATP2B4;ANK2;MITF;LMO7;ESRRG;ANK1;PTPRD;DLG2;NBEA;KIF26B;CDH13;FAT3;TNI*

			K;UTRN;TOX;DDR2
TFAP2D	37/299	2.7093099464421133E-17	ROBO2;DPP10;CNTNAP2;TENM2;DGKB;NRXN1;CELF4;NRXN3;RORA;GRIK2;ADARB2;NOL4;GRM3;RIMS1;GRM7;CSMD1;BRINP3;SOX5;CADPS2;RBFOX1;CNTN5;NEGR1;CADM2;CNTN6;DCC;PCDH7;PAX3;ESRRG;ANK1;NELL1;LRFN5;RALYL;DLG2;PAX7;CDH12;CNTN4;CDH18
THRB	37/299	2.7093099464421133E-17	ROBO2;PARM1;NRXN1;NRXN3;PTPRM;ZBTB20;RORA;HDAC9;BACH2;ERBB4;TRPS1;DMD;SLIT2;SOX6;GPC6;EDIL3;SOX5;MAGI1;CADPS2;NTRK2;NEGR1;PDE4D;PCDH7;ANK2;MITF;LMO7;ESRRG;ANK1;PTPRD;PLCB4;DLG2;NBEA;CDH13;TNIK;UTRN;TOX;DDR2
ZNF479	37/299	2.7093099464421133E-17	ROBO2;DPP10;CNTNAP2;TENM2;DGKB;KMT2C;DNAH5;NRXN3;DPP6;ERBB4;ZNF385B;DMD;CTNNA2;MACROD2;CSMD1;ZNF267;CADPS2;RBFOX1;CNTN5;AUTS2;CNTN6;DCC;PDE4D;PLCL1;ZNF804B;PDE4DIP;NRG1;PTPRD;MGAT4C;DLG2;DAB1;AGBL1;NBEA;NRG3;FAT3;CNTN4;CDH18
ZNF99	37/299	2.7093099464421133E-17	ROBO2;CHODL;DPP10;RYR2;CNTNAP2;TENM2;DNAH8;KMT2C;DNAH5;NRXN1;NRXN3;NOL4;CDH6;DPP6;PCDHA3;DMD;CTNNA3;SOX6;TRPM6;CSMD1;BRINP3;CADPS2;CNTN5;NEGR1;DCC;PDE4DIP;PXDNL;NRG1;ANO5;PTPRD;RALYL;NAV3;NRG3;FAT3;CNTN4;MDGA2;FREM2
LMX1A	36/299	1.9359789494956158E-16	DPP10;CNTNAP2;CASZ1;TENM2;NRXN1;CELF4;NRXN3;ADCY2;CDH6;CDH4;DPP6;ERBB4;DNER;CTNNA2;SLIT2;SOX6;CSMD1;BRINP3;SOX5;RGS7;CADPS2;NTRK2;RBFOX1;DCC;CADPS;PAX3;ESRRG;NRG1;ANK1;NELL1;DAB1;PAX7;KIF26B;CDH13;ASTN1;DDR2
RFX3	36/299	1.9359789494956158E-16	ROBO2;MAST4;KMT2C;NRXN1;ZBTB20;RORA;PPP1R9A;LYST;HDAC9;BACH2;DOCK10;TRPS1;SOX6;SOX5;MAGI1;CADPS2;NTRK2;CERS6;AUTS2;NEGR1;ANKRD44;PDE4D;TCF12;DNMT3A;ATP2B4;ANK2;LMO7;ESRRG;ANK1;PTPRD;PLCB4;NBEA;TNIK;UTRN;TOX;DDR2
CUX2	35/299	1.3414473605662394E-15	RYR2;CNTNAP2;CASZ1;NRXN1;CELF4;NRXN3;RORA;HDAC9;BACH2;CDH4;CSMD1;SOX5;MAGI1;RAP1GAP2;CADPS2;NTRK2;RBFOX1;AUTS2;NEGR1;PDE4D;CADPS;KAZN;ANK2;ESRRG;ANK1;PTPRD;DLG2;DAB1;NBEA;KIF26B;RIMBP2;CDH13;FAT3;TOX;DDR2
ZNF454	35/299	1.3414473605662394E-15	ROBO2;DPP10;PCSK1;RYR2;CNTNAP2;GALNT13;NRXN1;NRXN3;GRIK2;CDH4;DPP6;GRM7;ERBB4;CTNNA2;SLIT2;CSMD1;SOX5;CADPS2;RBFOX1;NEGR1;DCC;PCDH7;CADPS;ANK2;ESRRG;NRG1;PCDHA12;PTPRD;NELL1;LRFN5;DAB1;NBEA;KIF26B;CNTN4;DDR2
KLF12	34/299	8.390119777829284E-15	ROBO2;PTPRM;ZBTB20;RORA;HDAC9;BACH2;PTPRG;DOCK10;TRPS1;SLIT2;SOX6;GPC6;SOX5;MAGI1;CADPS2;NTRK2;AUTS2;NEGR1;PDE4D;PCDH7;TCF12;ATP2B4;PDE4DIP;LMO7;ANK1;ARHGAP24;PTPRD;PLCB4;NBEA;NAV3;TNIK;UTRN;TOX;DDR2
RORB	34/299	8.390119777829284E-15	ROBO2;CNTNAP2;TENM2;NRXN1;NRXN3;ZBTB20;ADCY2;RORA;GRIK2;HDAC9;CDH6;CDH4;TRPS1;SLIT2;SOX6;GPC6;EDIL3;SOX5;CADPS2;NTRK2;RBFOX1;NEGR1;CADM2;PDE4D;PCDH7;ANK2;ESRRG;ANK1;PTPRD;DLG2;CDH13;FAT3;TOX;DDR2
ZFPM2	34/299	8.390119777829284E-15	ROBO2;RYR2;TENM2;NRXN3;PTPRM;ZBTB20;RORA;HDAC9;CDH6;ERBB4;TRPS1;DMD;SLIT2;GPC6;EDIL3;SOX5;CADPS2;NTRK2;RBFOX1;AUTS2;NEGR1;PDE4D;PCDH7;ANK2;MITF;NRG1;PTPRD;PLCB4

			;NBEA;NAV3;KIF26B;CDH13;TOX;DDR2
ZNF407	34/299	8.3901197778 29284E-15	*ROBO2;DENND1B;MAST4;KMT2C;NRXN1;NRXN3;PTPRM;ZBTB20;RORA;HDAC9;BACH2;PTPRG;DOCK10;TRPS1;DMD;MACROD2;CSMD1;MAGI1;DYNC1H1;CADPS2;AUTS2;PDE4D;TCF12;PRRC2C;PDE4DIP;ANK2;LMO7;NRG1;ARHGAP24;PTPRD;NBEA;KIF26B;UTRN;DDR2*
ZSCAN1	34/299	8.3901197778 29284E-15	*FBLN7;DPP10;RYR2;CNTNAP2;CASZ1;NRXN1;CELF4;NRXN3;ADARB2;CDH4;DPP6;GRM7;TRPS1;CTNNA2;CSMD1;PCDHA6;CADPS2;RBFOX1;PCDH7;CADPS;NRG1;PCDHA12;ANK1;PCDHA10;SH3RF3;NELL1;DAB1;NRG3;PAX7;KIF26B;CDH13;FAT3;FREM2;DDR2*
FAM170A	33/299	5.2393495112 97019E-14	*ROBO2;DPP10;PCSK1;CNTNAP2;TENM2;GALNT13;DGKB;PCDH15;NRXN3;GRIK2;CDH6;FSTL5;CDH4;GRM7;ERBB4;GPC5;CSMD1;BRINP3;CADPS2;RBFOX1;CNTN5;CNTN6;DCC;ESRRG;LRFN5;RALYL;DLG2;MYO3B;KIF26B;CDH12;MDGA2;CDH18;DDR2*
NR3C2	33/299	5.2393495112 97019E-14	*PARM1;NRXN1;PTPRM;ZBTB20;RORA;PPP1R9A;HDAC9;BACH2;ERBB4;DMD;SLIT2;SOX6;SOX5;CADPS2;NTRK2;AUTS2;NEGR1;PDE4D;PLCL1;PCDH7;ANK2;MITF;LMO7;ANK1;ARHGAP24;PTPRD;PLCB4;NBEA;KIF26B;TNIK;UTRN;TOX;DDR2*
TSHZ2	33/299	5.2393495112 97019E-14	*ROBO2;CNTNAP2;TENM2;PARM1;MAST4;PTPRM;ZBTB20;RORA;BACH2;TRPS1;GPC3;DMD;SLIT2;SOX6;GPC6;SOX5;CADPS2;NTRK2;AUTS2;PDE4D;PCDH7;ATP2B4;ANK2;ESRRG;ANK1;ARHGAP24;PTPRD;DLG2;NBEA;KIF26B;CDH13;TOX;DDR2*
ZIM3	33/299	5.2393495112 97019E-14	*ROBO2;DPP10;RYR2;CNTNAP2;TENM2;DNAH5;NRXN1;PCDH15;NRXN3;FSTL5;RIMS1;ERBB4;CSMD1;BRINP3;SOX5;CADPS2;RBFOX1;CNTN5;AUTS2;CNTN6;DCC;ANK2;ESRRG;ANK1;PTPRD;MGAT4C;NELL1;NAV3;CDH12;FAT3;CNTN4;CDH18;DDR2*
FEZF2	32/299	3.2404877505 41081E-13	*ROBO2;PCSK1;TENM2;NRXN1;CELF4;NRXN3;ADCY2;GRIK2;NOL4;RIMS1;CDH4;DPP6;ERBB4;SLIT2;CSMD1;SOX5;RGS7;CADPS2;NTRK2;RBFOX1;DCC;PCDH7;CADPS;PAX3;NRG1;ANK1;NELL1;DAB1;PAX7;CDH13;TOX;DDR2*
SIM1	32/299	3.2404877505 41081E-13	*ROBO2;PCSK1;CASZ1;NRXN1;NRXN3;ZBTB20;RORA;GRIK2;HDAC9;CDH6;DPP6;ERBB4;TRPS1;DNER;SLIT2;SOX6;CSMD1;GPC6;EDIL3;SOX5;CADPS2;NTRK2;RBFOX1;DCC;PCDH7;PAX3;MITF;ESRRG;NRG1;ANK1;KIF26B;DDR2*
TGIF2LX	32/299	3.2404877505 41081E-13	*DPP10;NRXN1;PCDH15;CELF4;NRXN3;HTR2C;RORA;GRIK2;DACH2;FSTL5;CDH4;DPP6;DMD;CTNNA2;CSMD1;GPC6;SOX5;RBFOX1;DCC;SH2D1A;PDE4DIP;MITF;ESRRG;ETV6;OTOGL;PTPRD;NRG3;MYO3B;CDH12;FGF13;CDH18;DDR2*
PKNOX2	31/299	1.9031450622 981405E-12	*ROBO2;CNTNAP2;PARM1;NRXN1;NRXN3;PTPRM;ADCY2;RORA;BACH2;PTPRG;CDH6;CDH4;DPP6;SLIT2;SOX6;GPC6;SOX5;CADPS2;NTRK2;RBFOX1;AUTS2;PDE4D;PCDH7;ANK2;ESRRG;ANK1;PTPRD;DLG2;KIF26B;CDH13;DDR2*
RARB	31/299	1.9031450622 981405E-12	*ROBO2;NRXN3;PTPRM;ZBTB20;RORA;HDAC9;BACH2;CDH6;ERBB4;TRPS1;SLIT2;SOX6;SOX5;CADPS2;NTRK2;NEGR1;PDE4D;PCDH7;MITF;LMO7;ESRRG;NRG1;ANK1;ARHGAP24;PTPRD;PLCB4;KIF26B;CDH13;FAT3;TOX;DDR2*
SETBP1	31/299	1.9031450622 981405E-12	*ROBO2;MAST4;KMT2C;NRXN3;PTPRM;ZBTB20;RORA;HDAC9;BACH2;TRPS1;DMD;SLIT2;GPC6;SOX5;CADPS2;NTRK2;AUTS2;ATRNL1;PDE4D;PCDH7;ATP2B4;ANK2;ANK1;ARHGAP24;PTPRD;NBEA;KIF26B;TNIK;UTRN;TOX;DDR2*

ZFAT	31/299	1.9031450622981405E-12	*MAST4;NRXN3;ZBTB20;RORA;HDAC9;BACH2;TRPS1;SOX5;JAZF1;MAGI1;RAP1GAP2;CADPS2;RBFOX1;AUTS2;IQSEC1;PDE4D;DNMT3A;ATP2B4;PDE4DIP;LMO7;NRG1;ANK1;ETV6;PTPRD;KIF26B;FAT3;TNIK;UTRN;TOX;LPIN1;DDR2*
DPF3	30/299	1.1006712158505583E-11	*CASZ1;PTPRM;ZBTB20;RORA;HDAC9;BACH2;CDH4;SLIT2;SOX6;SOX5;RAP1GAP2;CADPS2;NTRK2;RBFOX1;AUTS2;NEGR1;PDE4D;ANK2;LMO7;ESRRG;NRG1;ANK1;ARHGAP24;DAB1;KIF26B;CDH13;TNIK;TOX;LPIN1;DDR2*
ZNF208	30/299	1.1006712158505583E-11	*RYR2;CNTNAP2;KMT2C;NRXN1;NRXN3;PPP1R9A;HDAC9;CDH6;DPP6;ERBB4;GPC3;DMD;CSMD1;EDIL3;CADPS2;NTRK2;NEGR1;ATRNL1;PCDH7;PDE4DIP;ANO5;PTPRD;DLG2;NBEA;NAV3;FAT3;CNTN4;FGF13;FREM2;DDR2*
ZNF718	30/299	1.1006712158505583E-11	*RYR2;CNTNAP2;CASZ1;NRXN1;NRXN3;RORA;GRIK2;PPP1R9A;DOCK10;BRIP1;DPP6;TRPS1;MACROD2;SLIT2;CSMD1;EDIL3;MAGI1;RAP1GAP2;CADPS2;AUTS2;PDE4D;PCDH7;DNMT3A;PDE4DIP;ANK1;NBPF1;PLCB4;NBEA;CDH13;DDR2*
MYT1	29/299	5.945289272349029E-11	*ROBO2;PCSK1;CNTNAP2;NRXN1;CELF4;NRXN3;RORA;GRIK2;NOL4;BACH2;CDH4;DPP6;ERBB4;DNER;CTNNA2;SOX5;CADPS2;NTRK2;DCC;CADPS;ANK2;ESRRG;ANK1;PTPRD;KIF26B;RIMBP2;TOX;DSCAML1;ASTN1*
POU3F2	29/299	5.945289272349029E-11	*ROBO2;CNTNAP2;NRXN1;NRXN3;RORA;GRIK2;NOL4;CDH6;CDH4;ERBB4;DNER;SLIT2;SOX6;SOX5;CADPS2;NTRK2;RBFOX1;NEGR1;DCC;PCDH7;PAX3;ESRRG;NRG1;PTPRD;FAT3;TOX;BMPR1B;MDGA2;DDR2*
RAPGEF4	29/299	5.945289272349029E-11	*ROBO2;RYR2;TENM2;NRXN1;PTPRM;ZBTB20;RORA;GRIK2;HDAC9;RIMS1;CTNNA2;DYNC1I1;RAP1GAP2;CADPS2;NTRK2;AUTS2;PDE4D;CADPS;ANK2;LMO7;ESRRG;ANK1;PTPRD;PLCB4;DLG2;CDH13;TNIK;SCN2A;DDR2*
ZFHX4	29/299	5.945289272349029E-11	*ROBO2;TENM2;ZBTB20;RORA;HDAC9;BACH2;CDH6;TRPS1;DMD;SLIT2;SOX6;GPC6;EDIL3;SOX5;CADPS2;NTRK2;AUTS2;NEGR1;PDE4D;PCDH7;ANK2;NRG1;PTPRD;NBEA;NAV3;KIF26B;FAT3;TOX;DDR2*
ZNF648	29/299	5.945289272349029E-11	*RYR2;CASZ1;TENM2;DNAH5;NRXN1;NRXN3;GRIK2;CDH4;DPP6;TRPS1;SLIT2;CSMD1;SOX5;RGS7;CADPS2;NTRK2;NEGR1;PDE4D;PCDH7;ATP2B4;PDE4DIP;ESRRG;PAX7;KIF26B;CDH13;FAT3;TOX;DSCAML1;DDR2*
DBX2	28/299	3.105718525790822E-10	*ROBO2;TENM2;NRXN1;RORA;ADARB2;CDH4;DPP6;TRPS1;GPC5;SOX6;CSMD1;SOX5;CADPS2;NTRK2;RBFOX1;CADM2;DCC;PAX3;ESRRG;ANK1;FGF14;DAB1;PPP2R2C;PAX7;KIF26B;BMPR1B;FREM2;DDR2*
TBX5	28/299	3.105718525790822E-10	*ROBO2;RYR2;CASZ1;NRXN1;GRIK2;DPP6;SGCD;SLIT2;SOX6;CSMD1;WNT2;SOX5;CADPS2;NTRK2;RBFOX1;PCDH7;CADPS;PAX3;MITF;ESRRG;NRG1;ANK1;PTPRD;NELL1;PAX7;KIF26B;CDH13;DDR2*
ZIC4	28/299	3.105718525790822E-10	*ROBO2;RYR2;TENM2;NRXN1;CELF4;NRXN3;GRIK2;CDH6;CDH4;DPP6;ERBB4;SLIT2;SOX6;SOX5;CADPS2;NTRK2;RBFOX1;DCC;PCDH7;CADPS;PAX3;ESRRG;NRG1;PAX7;KIF26B;CDH13;BMPR1B;DDR2*
ZNF578	28/299	3.105718525790822E-10	*ROBO2;RYR2;CNTNAP2;TENM2;NRXN1;NRXN3;GRIK2;HDAC9;CDH4;DPP6;ERBB4;CSMD1;RAP1GAP2;CADPS2;AUTS2;NEGR1;CADM2;DCC;PDE4D;PCDH7;LMO7;ESRRG;NRG1;PTPRD;NBEA;PDE3A;CNTN4;SCN2A*

ZSCAN23	28/299	3.1057185257 90822E-10	*ROBO2;RYR2;GALNT13;NRXN1;ZBTB20;RORA;BACH2;PTPRG;CDH6;TRPS1;SLIT2;SOX6;CSMD1;SOX5;CADPS2;AUTS2;NEGR1;ATRNL1;SGIP1;PCDH7;ANK2;ESRRG;NBEA;KIF26B;FAT3;TOX;BMPR1B;DDR2*
ARX	27/299	1.4655010591 468382E-9	*ROBO2;PCSK1;CNTNAP2;NRXN1;CELF4;NRXN3;ADCY2;RORA;GRIK2;CDH4;DPP6;DNER;GPC3;DMD;SLIT2;SOX6;CADPS2;NTRK2;RBFOX1;AUTS2;PCDH7;CADPS;ESRRG;ANK1;NELL1;FGF13;DDR2*
FOXD4L5	27/299	1.4655010591 468382E-9	*ROBO2;CNTNAP2;KMT2C;DNAH5;NRXN1;NRXN3;GRIK2;CDH4;DPP6;GRM7;TRPS1;DMD;CADPS2;NTRK2;RBFOX1;CNTN5;NEGR1;PDE4D;PCDH7;PDE4DIP;ESRRG;NRG1;NELL1;RALYL;DAB1;KIF26B;CDH18*
NEUROD6	27/299	1.4655010591 468382E-9	*ROBO2;PCSK1;TENM2;NRXN1;CELF4;NRXN3;HTR2C;GRIK2;NOL4;GRM3;CDH4;DPP6;SOX5;PPFIA2;RGS7;CADPS2;RBFOX1;NEGR1;DCC;CADPS;NELL1;RALYL;DLG2;DAB1;RIMBP2;CDH13;SCN2A*
POU3F4	27/299	1.4655010591 468382E-9	*ROBO2;DGKB;NRXN1;CELF4;HTR2C;RORA;GRIK2;CDH6;GRM3;CDH4;DPP6;DMD;SOX6;RGS9;EDIL3;SOX5;CADPS2;NTRK2;RBFOX1;NEGR1;DCC;PCDH7;PAX3;RALYL;DLG2;FAT3;DDR2*
PROX1	27/299	1.4655010591 468382E-9	*ROBO2;CASZ1;PARM1;NRXN1;ZBTB20;RORA;HDAC9;BACH2;ERBB4;TRPS1;SLIT2;SOX6;EDIL3;SOX5;CADPS2;NTRK2;AUTS2;PCDH7;ANK2;LMO7;ESRRG;ANK1;PTPRD;NBEA;KIF26B;TNIK;DDR2*
RFX4	27/299	1.4655010591 468382E-9	*RYR2;CNTNAP2;DGKB;NRXN1;CELF4;NRXN3;RORA;BACH2;CDH4;TRPS1;CTNNA2;SOX6;SOX5;CADPS2;NTRK2;RBFOX1;DCC;PAX3;ESRRG;NRG1;ANK1;DLG2;DAB1;PAX7;TOX;BMPR1B;DDR2*
SCAPER	27/299	1.4655010591 468382E-9	*MAST4;KMT2C;FAM13A;ZBTB20;RORA;PPP1R9A;LYST;HDAC9;BACH2;TRPS1;NEK1;NCOA1;MAGI1;CADPS2;AUTS2;PDE4D;TCF12;PDE4DIP;ANK2;LMO7;NBEA;NAV3;ZNF638;TTC3;TNIK;UTRN;DDR2*
ZNF483	27/299	1.4655010591 468382E-9	*ROBO2;RYR2;NRXN1;ADAM22;NRXN3;RORA;PPP1R9A;ERBB4;DNER;SLIT2;EDIL3;RAP1GAP2;CADPS2;NTRK2;AUTS2;NEGR1;CADM2;PCDH7;CADPS;ANK2;PTPRD;PLCB4;NBEA;TNIK;TOX;FGF13;DDR2*
ZNF540	27/299	1.4655010591 468382E-9	*CNTNAP2;PARM1;NRXN1;NRXN3;ZBTB20;RORA;GRIK2;BACH2;DPP6;DMD;PPFIA2;CADPS2;NTRK2;NEGR1;CADM2;ATRNL1;PDE4D;PLCL1;PDE4DIP;LMO7;ESRRG;DLG2;NBEA;NAV3;CDH12;SCN2A;DDR2*
ZNF835	27/299	1.4655010591 468382E-9	*GLDC;NRXN1;NRXN3;ADCY2;GRIK2;ADARB2;PPP1R9A;DPP6;DNER;CTNNA2;CSMD1;CADPS2;NTRK2;RBFOX1;DSCAM;CADM2;DCC;PCDH7;CADPS;NRG1;ANK1;SH3RF3;CNTN4;TOX;DSCAML1;FREM2;DDR2*
DACH1	26/299	6.8188867626 72163E-9	*ROBO2;CNTNAP2;PARM1;NRXN1;RORA;HDAC9;BACH2;CDH6;ERBB4;TRPS1;SLIT2;SOX6;GPC6;EDIL3;SOX5;CADPS2;NTRK2;AUTS2;NEGR1;PCDH7;ESRRG;PTPRD;NBEA;CDH13;TOX;DDR2*
GRM6	26/299	6.8188867626 72163E-9	*RYR2;CASZ1;NRXN1;NRXN3;ADCY2;GRIK2;DOCK10;DPP6;GRM7;DMD;CTNNA2;CSMD1;CADPS2;NTRK2;RBFOX1;DSCAM;PDE4D;CADPS;ESRRG;NRG1;ANK1;KIF26B;CDH13;CNTN4;DSCAML1;DDR2*
NEUROD4	26/299	6.8188867626 72163E-9	*ROBO2;PCSK1;TENM2;NRXN1;PCDH15;CELF4;NRXN3;GRIK2;CDH6;FSTL5;ERBB4;CTNNA2;SOX5;CADPS2;NTRK2;RBFOX1;DCC;CADPS;PAX3;ESRRG;NRG1;FGF14;PAX7;BMPR1B;ASTN1;DDR2*
NHLH2	26/299	6.8188867626 72163E-9	*ROBO2;CASZ1;NRXN1;NRXN3;GRIK2;NOL4;DPP6;ERBB4;DNER;SLIT2;SOX6;CADPS2;NTRK2;RBFOX1;AUTS2;DCC;PCDH7;CADPS;PAX3;ESRRG;NRG1;ANK1;PAX7;KIF26B;CDH13;DDR2*
PRDM5	26/299	6.8188867626	*ROBO2;PTPRM;ZBTB20;RORA;HDAC9;PTPRG;TRPS*

		72163E-9	*1;DMD;SLIT2;GPC6;EDIL3;SOX5;CADPS2;AUTS2;PCDH7;ANK2;MITF;NRG1;ANK1;PTPRD;PLCB4;NBEA;CDH13;UTRN;BMPR1B;DDR2*
PRDM9	26/299	6.8188867626 72163E-9	*KMT2C;DNAH5;NRXN1;PCDH15;RORA;GRIK2;HDAC9;DOCK10;DPP6;TRPS1;DMD;CSMD1;BRINP3;CADPS2;CNTN5;PCDH7;PDE4DIP;ESRRG;ANK1;DLG2;CDH12;FAT3;UTRN;LPIN1;CDH18;DDR2*
SALL3	26/299	6.8188867626 72163E-9	*ROBO2;TRIM71;CNTNAP2;CASZ1;GLDC;NRXN1;CELF4;NRXN3;RORA;CDH4;DPP6;SOX6;CSMD1;SOX5;CADPS2;NTRK2;RBFOX1;AUTS2;CADPS;ESRRG;ANK1;PTPRD;DAB1;KIF26B;CDH13;DDR2*
SATB2	26/299	6.8188867626 72163E-9	*ROBO2;ZBTB20;RORA;PTPRG;TRPS1;SLIT2;SOX5;CADPS2;NTRK2;AUTS2;PDE4D;PCDH7;ATP2B4;ANK2;LMO7;ESRRG;ANK1;PTPRD;PLCB4;NBEA;NAV3;CDH13;TNIK;UTRN;TOX;DDR2*
ZNF568	26/299	6.8188867626 72163E-9	*RYR2;CNTNAP2;KMT2C;NRXN1;NRXN3;ZBTB20;RORA;GRIK2;HDAC9;PTPRG;DPP6;DMD;SLIT2;CSMD1;CADPS2;AUTS2;PDE4D;PCDH7;PDE4DIP;ANK2;LMO7;ESRRG;PTPRD;PLCB4;NBEA;DDR2*
DLX6	25/299	3.1365566247 76081E-8	*ROBO2;CNTNAP2;NRXN1;CELF4;NRXN3;RORA;GRIK2;DPP6;ERBB4;SLIT2;SOX6;DYNC1I1;SOX5;CADPS2;NTRK2;RBFOX1;PCDH7;PAX3;LMO7;ESRRG;NRG1;ANK1;PAX7;KIF26B;DDR2*
PLXNA2	25/299	3.1365566247 76081E-8	*TENM2;PTPRM;ZBTB20;RORA;BACH2;PTPRG;SLIT2;CADPS2;NTRK2;AUTS2;PDE4D;PCDH7;ATP2B4;PDE4DIP;ANK2;LMO7;NRG1;ANK1;PTPRD;NBEA;KIF26B;CDH13;FAT3;TNIK;DDR2*
PRDM10	25/299	3.1365566247 76081E-8	*MAST4;KMT2C;ZBTB20;RORA;LYST;HDAC9;BACH2;TRPS1;MAGI1;DYNC1H1;CERS6;AUTS2;PDE4D;TCF12;DNMT3A;ATP2B4;PDE4DIP;LMO7;ANK1;ETV6;PLCB4;NBEA;TNIK;UTRN;DDR2*
RORA	25/299	3.1365566247 76081E-8	*NRXN3;PTPRM;ZBTB20;HDAC9;BACH2;TRPS1;SOX6;GPC6;SOX5;CADPS2;NTRK2;AUTS2;PDE4D;PDE4DIP;ANK2;ESRRG;ANK1;PTPRD;PLCB4;DLG2;CDH13;TNIK;UTRN;TOX;DDR2*
SORBS2	25/299	3.1365566247 76081E-8	*NRXN1;PTPRM;ZBTB20;RORA;HDAC9;DMD;SLIT2;MAGI1;CADPS2;NTRK2;AUTS2;PDE4D;PCDH7;ATP2B4;ANK2;LMO7;ANK1;PTPRD;PLCB4;NBEA;NAV3;CDH13;TNIK;UTRN;DDR2*
TUB	25/299	3.1365566247 76081E-8	*CNTNAP2;NRXN1;NRXN3;RORA;GRIK2;DPP6;SLIT2;EDIL3;RAP1GAP2;CADPS2;NTRK2;NEGR1;PDE4D;PCDH7;ATP2B4;ANK2;LMO7;ANK1;PTPRD;DLG2;DAB1;NBEA;NAV3;PPP2R2C;DDR2*
ZNF418	25/299	3.1365566247 76081E-8	*FBLN7;MAST4;KMT2C;NRXN1;NRXN3;PTPRM;ZBTB20;RORA;GRIK2;HDAC9;BACH2;DPP6;DMD;CADPS2;AUTS2;PDE4D;PCDH7;DNMT3A;PDE4DIP;ANK2;NRG1;NBEA;TNIK;FREM2;DDR2*
BCL11B	24/299	1.3220078202 860265E-7	*ROBO2;RAP1GAP2;CADPS2;CNTNAP2;NTRK2;RBFOX1;CASZ1;AUTS2;PDE4D;PCDH7;ATP2B4;ZBTB20;RORA;ANK1;BACH2;DOCK10;CDH4;NBEA;KIF26B;TRPS1;TNIK;TOX;SOX5;DDR2*
ESRRB	24/299	1.3220078202 860265E-7	*ROBO2;CADPS2;CNTNAP2;NTRK2;RBFOX1;CASZ1;GLDC;PDE4D;CELF4;NRXN3;RORA;MITF;ESRRG;NRG1;ANK1;HDAC9;ENOX1;CDH4;DAB1;ERBB4;KIF26B;CDH13;SOX5;DDR2*
INSM2	24/299	1.3220078202 860265E-7	*RAP1GAP2;PCSK1;RYR2;CADPS2;NTRK2;RBFOX1;CASZ1;DCC;NRXN1;CADPS;CELF4;NRXN3;RORA;ESRRG;ANK1;NOL4;DPP6;RALYL;KIF26B;DNER;RIMBP2;SLIT2;TOX;RGS7*
PAX7	24/299	1.3220078202 860265E-7	*RYR2;CADPS2;NTRK2;RBFOX1;CASZ1;DCC;CELF4;NRXN3;PAX3;ESRRG;NRG1;ADARB2;ANK1;NELL1;CDH4;DPP6;DAB1;ERBB4;KIF26B;CDH13;SLIT2;SOX6;SOX5;DDR2*

SOHLH2	24/299	1.3220078202860265E-7	*RYR2;CADPS2;CNTNAP2;RBFOX1;TENM2;PARM1;GLDC;PDE4D;FAM13A;NRXN3;MITF;LMO7;NRG1;ANK1;ENOX1;PPP2R2C;KIF26B;CDH13;TNIK;SLIT2;FGF13;CSMD1;GPC6;DDR2*
TBR1	24/299	1.3220078202860265E-7	*ROBO2;CADPS2;CNTNAP2;RBFOX1;KDM4B;DSCAM;KMT2C;NRXN1;DNMT3A;CELF4;NRXN3;PTPRM;ANK2;RORA;ESRRG;ANK1;RIMS1;CDH4;NBEA;GRM7;SCN2A;SOX5;DDR2;RGS7*
TFAP2B	24/299	1.3220078202860265E-7	*ROBO2;CADPS2;NTRK2;RBFOX1;TENM2;DCC;PCDH7;CELF4;PAX3;RORA;MITF;ESRRG;NRG1;GRIK2;ANK1;PAX7;ERBB4;KIF26B;SLIT2;SOX6;CSMD1;EDIL3;SOX5;DDR2*
TOX	24/299	1.3220078202860265E-7	*ROBO2;CADPS2;NTRK2;AUTS2;NEGR1;PDE4D;NRXN1;PCDH7;PTPRM;ZBTB20;RORA;NRG1;ANK1;HDAC9;BACH2;DOCK10;PTPRD;PLCB4;KIF26B;TRPS1;TNIK;SLIT2;SOX5;DDR2*
ZKSCAN2	24/299	1.3220078202860265E-7	*ROBO2;CADPS2;CNTNAP2;CERS6;AUTS2;NEGR1;DCC;KMT2C;PDE4D;PLCL1;DNMT3A;ANK2;RORA;ANK1;BACH2;PTPRD;DLG2;NBEA;KIF26B;TRPS1;TNIK;SLIT2;TOX;DDR2*
ZNF135	24/299	1.3220078202860265E-7	*MAGI1;CADPS2;CNTNAP2;AUTS2;NEGR1;PDE4D;NRXN1;PCDH7;NRXN3;PTPRM;ANK2;RORA;HDAC9;BACH2;PTPRD;DPP6;PLCB4;NBEA;DMD;CNTN4;SLIT2;SOX6;TOX;DDR2*
ZNF354C	24/299	1.3220078202860265E-7	*ROBO2;RYR2;CADPS2;NTRK2;TENM2;AUTS2;PCDH7;ZBTB20;SMAD9;RORA;NRG1;HDAC9;BACH2;PLCB4;DLG2;NBEA;TRPS1;PDE3A;DMD;SLIT2;SOX6;EDIL3;SOX5;DDR2*
ZNF660	24/299	1.3220078202860265E-7	*ROBO2;CADPS2;AUTS2;NEGR1;DCC;KMT2C;PLCL1;NRXN3;ZBTB20;RORA;PPP1R9A;HDAC9;PTPRG;CDH6;DOCK10;PTPRD;FGF14;NBEA;TNIK;SLIT2;TOX;GPC6;SOX5;DDR2*
BCL11A	23/299	5.256476699850769E-7	*CADPS2;CNTNAP2;NTRK2;DSCAM;AUTS2;KMT2C;PDE4D;NRXN1;PCDH7;DNMT3A;ZBTB20;ANK2;RORA;ANK1;HDAC9;BACH2;PTPRD;RIMS1;TRPS1;TNIK;SCN2A;TOX;DDR2*
DEPDC5	23/299	5.256476699850769E-7	*MAGI1;DYNC1H1;CADPS2;AUTS2;MAST4;IQSEC1;KMT2C;PDE4D;DNMT3A;ATP2B4;PRRC2C;ZBTB20;PDE4DIP;ANK2;ANK1;LYST;HDAC9;PTPRD;PLCB4;TNIK;UTRN;LPIN1;DDR2*
FOXG1	23/299	5.256476699850769E-7	*ROBO2;CADPS2;CNTNAP2;NTRK2;RBFOX1;AUTS2;NRXN1;PCDH7;CELF4;PAX3;ZBTB20;LMO7;GRIK2;ANK1;CDH6;CDH4;DPP6;NBEA;ERBB4;CDH13;SLIT2;SOX5;DDR2*
HDX	23/299	5.256476699850769E-7	*ROBO2;CADPS2;NEGR1;PDE4D;PLCL1;PCDH7;ZBTB20;ANK2;NRG1;HDAC9;BACH2;CDH6;DOCK10;PTPRD;PLCB4;NBEA;TRPS1;DMD;TNIK;SLIT2;TOX;GPC6;DDR2*
KIAA1549	23/299	5.256476699850769E-7	*MAGI1;NTRK2;AUTS2;KMT2C;PDE4D;DNMT3A;NRXN3;ATP2B4;PDE4DIP;ANK2;LMO7;PPP1R9A;BACH2;PTPRD;PLCB4;NBEA;KIF26B;TRPS1;FAT3;TNIK;FREM2;SOX5;DDR2*
PAX3	23/299	5.256476699850769E-7	*ROBO2;CADPS2;NTRK2;RBFOX1;DCC;PCDH7;RORA;MITF;ESRRG;NRG1;GRIK2;ANK1;CDH6;PTPRD;CDH4;PAX7;ERBB4;KIF26B;SLIT2;SOX6;BMPR1B;SOX5;DDR2*
POU4F1	23/299	5.256476699850769E-7	*CADPS2;CNTNAP2;NTRK2;RBFOX1;CASZ1;AUTS2;CELF4;NRXN3;PAX3;RORA;ESRRG;NRG1;ANK1;HDAC9;BACH2;NELL1;CDH4;PAX7;KIF26B;SLIT2;TOX;SOX5;DDR2*
TBX22	23/299	5.256476699850769E-7	*ROBO2;CHODL;DPP10;CADPS2;COLEC10;CNTN5;CNTN6;PCDH15;HTR2C;DACH2;ANK1;MGAT4C;DPP6;LRFN5;ERBB4;CDH12;DMD;CNTN4;FGF13;MDGA2*

			;BRINP3;CDH18;DDR2
TOX3	23/299	5.2564766998 50769E-7	*ROBO2;CADPS2;CNTNAP2;NTRK2;PARM1;AUTS2;NRXN1;PCDH7;CADPS;RORA;LMO7;ESRRG;BACH2;PTPRD;CDH4;DAB1;NBEA;KIF26B;CDH13;SLIT2;TOX;GPC6;DDR2*
ZBTB20	23/299	5.2564766998 50769E-7	*CADPS2;NTRK2;AUTS2;MAST4;KMT2C;PDE4D;PTPRM;PDE4DIP;ANK2;RORA;ANK1;HDAC9;BACH2;PTPRD;NBEA;TRPS1;DMD;TNIK;SOX6;UTRN;LPIN1;SOX5;DDR2*
ZNF229	23/299	5.2564766998 50769E-7	*ROBO2;RYR2;CADPS2;CNTNAP2;AUTS2;NEGR1;KMT2C;PDE4D;NRXN1;PCDH7;NRXN3;KIAA1549L;PTPRM;LMO7;NRG1;NBEA;NAV3;TRPS1;FAT3;DMD;CSMD1;GPC6;DDR2*
ZNF462	23/299	5.2564766998 50769E-7	*MAGI1;CADPS2;AUTS2;MAST4;KMT2C;PDE4D;PCDH7;ATP2B4;ZBTB20;ANK2;RORA;LMO7;PPP1R9A;PTPRG;PTPRD;NBEA;NAV3;KIF26B;TRPS1;TNIK;UTRN;SOX5;DDR2*
ZNF471	23/299	5.2564766998 50769E-7	*RYR2;CADPS2;NTRK2;NEGR1;PLCL1;PCDH7;ZBTB20;RORA;MITF;GRIK2;LYST;PLCB4;NBEA;CDH13;FAT3;DMD;CNTN4;SLIT2;SOX6;MPDZ;EDIL3;SOX5;DDR2*
ZNF676	23/299	5.2564766998 50769E-7	*RYR2;CADPS2;CNTNAP2;NTRK2;TENM2;NEGR1;ATRNL1;NRXN1;PCDH7;GRIK2;ANO5;DPP6;DLG2;NBEA;NAV3;PDE3A;GPC3;CTNNA2;CNTN4;SOX6;CSMD1;EDIL3;DDR2*
ZNF705D	23/299	5.2564766998 50769E-7	*PCSK1;CADPS2;CNTNAP2;RBFOX1;CNTN5;NEGR1;DCC;KMT2C;PLCL1;PDE4DIP;RORA;ESRRG;NBPF1;FSTL5;NELL1;DPP6;KIF26B;DMD;MACROD2;TOX;CSMD1;WNT2;SOX5*
ASCL1	22/299	2.0540887906 19987E-6	*CADPS2;CNTNAP2;NTRK2;DCC;NRXN1;CADPS;CELF4;RORA;ESRRG;NRG1;NOL4;CDH6;NELL1;DPP6;FGF14;ERBB4;DNER;SOX6;TOX;SOX5;ASTN1;DDR2*
DUXA	22/299	2.0540887906 19987E-6	*ROBO2;RYR2;RBFOX1;CNTN5;DGKB;KMT2C;TCF12;PDE4DIP;RORA;MITF;ESRRG;ENOX1;CDH6;BRIP1;CDH4;PAX7;DMD;CNTN4;SOX6;CDH18;SOX5;DDR2*
FBXO41	22/299	2.0540887906 19987E-6	*RAP1GAP2;CADPS2;CNTNAP2;NTRK2;NEGR1;IQSEC1;PDE4D;NRXN1;DNMT3A;CADPS;CELF4;ANK2;RORA;GRIK2;ANK1;DLG2;NBEA;PPP2R2C;RIMBP2;CTNNA2;SCN2A;DDR2*
MLLT3	22/299	2.0540887906 19987E-6	*CADPS2;CERS6;AUTS2;MAST4;PDE4D;ZBTB20;PDE4DIP;ANK2;RORA;LMO7;ANK1;HDAC9;BACH2;PTPRD;PLCB4;NBEA;TRPS1;TNIK;SOX6;UTRN;TOX;DDR2*
NFIA	22/299	2.0540887906 19987E-6	*CADPS2;NTRK2;AUTS2;PDE4D;PCDH7;ATP2B4;PTPRM;ZBTB20;PDE4DIP;ANK2;RORA;LMO7;ANK1;BACH2;PTPRD;NBEA;TRPS1;DMD;SOX6;UTRN;SOX5;DDR2*
ONECUT2	22/299	2.0540887906 19987E-6	*ROBO2;CADPS2;CNTNAP2;NTRK2;RBFOX1;CASZ1;DCC;NRXN1;PCDH7;CELF4;NRXN3;ZBTB20;RORA;ESRRG;ANK1;BACH2;PTPRD;NBEA;PPP2R2C;KIF26B;SOX6;DDR2*
PBX1	22/299	2.0540887906 19987E-6	*MAGI1;CADPS2;NTRK2;AUTS2;PDE4D;PCDH7;ATP2B4;PTPRM;ZBTB20;ANK2;RORA;ANK1;HDAC9;PTPRD;NBEA;TRPS1;TNIK;SLIT2;SOX6;UTRN;SOX5;DDR2*
PRDM2	22/299	2.0540887906 19987E-6	*NCOA1;MAGI1;AUTS2;MAST4;IQSEC1;KMT2C;PDE4D;TCF12;DNMT3A;ATP2B4;PRRC2C;ZBTB20;PDE4DIP;RORA;SSH2;ANK1;LYST;BACH2;ETV6;TRPS1;UTRN;DDR2*
ZNF214	22/299	2.0540887906 19987E-6	*CADPS2;NTRK2;DNAH7;PDE4D;DNAH5;NRXN1;FAM13A;NRXN3;RORA;LMO7;NRG1;DLG2;NBEA;ERBB4*

			;KIF26B;TRPS1;FAT3;TNIK;SLIT2;TOX;BMPR1B;DDR2
ZNF334	22/299	2.054088790619987E-6	*CADPS2;NTRK2;AUTS2;PDE4D;NRXN1;PCDH7;CADPS;NRXN3;ANK2;RORA;ESRRG;PPP1R9A;PTPRD;DPP6;NBEA;KIF26B;DNER;DMD;SLIT2;TOX;FREM2;DDR2*
ZNF391	22/299	2.054088790619987E-6	*CADPS2;PARM1;AUTS2;NEGR1;PDE4D;PCDH7;ADAM22;ANK2;RORA;LMO7;PPP1R9A;HDAC9;ARHGAP24;PTPRD;DLG2;NBEA;TRPS1;TNIK;SLIT2;SCN2A;SOX5;DDR2*
ZNF713	22/299	2.054088790619987E-6	*CADPS2;AUTS2;PDE4D;TCF12;ZBTB20;SMAD9;ANK2;RORA;ESRRG;GRIK2;HDAC9;BACH2;BRIP1;NBEA;DMD;CNTN4;SOX6;TOX;GPC6;FREM2;SOX5;DDR2*
ZNF827	22/299	2.054088790619987E-6	*MAGI1;CADPS2;AUTS2;MAST4;KMT2C;PDE4D;DNMT3A;ATP2B4;ZBTB20;RORA;PPP1R9A;HDAC9;BACH2;ETV6;DOCK10;NBEA;KIF26B;TRPS1;TNIK;UTRN;TOX;DDR2*
FAM171B	21/299	7.765824510322848E-6	*ROBO2;CADPS2;NTRK2;NEGR1;NRXN1;PCDH7;ATP2B4;ANK2;RORA;ANK1;HDAC9;PTPRG;PTPRD;NBEA;NAV3;TRPS1;TNIK;SLIT2;GPC6;EDIL3;DDR2*
FEZF1	21/299	7.765824510322848E-6	*TRIM71;CADPS2;CNTNAP2;NTRK2;RBFOX1;CASZ1;TENM2;NRXN1;PCDH7;CADPS;PAX3;ESRRG;GRIK2;ANK1;DAB1;PAX7;ERBB4;CTNNA2;SLIT2;SOX5;DDR2*
FOXD4L6	21/299	7.765824510322848E-6	*PCSK1;NTRK2;RBFOX1;NEGR1;PDE4D;NRXN1;PCDH7;PDE4DIP;ESRRG;NRG1;GRIK2;PTPRD;GRM3;NELL1;DPP6;RALYL;PAX7;TRPS1;EDIL3;CDH18;SOX5*
GLIS1	21/299	7.765824510322848E-6	*CADPS2;CASZ1;AUTS2;NEGR1;PCDH7;RORA;ESRRG;NRG1;ANK1;BACH2;ENOX1;PTPRD;CDH4;DAB1;KIF26B;CDH13;SLIT2;SOX6;GPC6;SOX5;DDR2*
GLIS3	21/299	7.765824510322848E-6	*CADPS2;NTRK2;AUTS2;PDE4D;PCDH7;PTPRM;ZBTB20;RORA;LMO7;NRG1;ANK1;HDAC9;BACH2;PTPRD;KIF26B;TRPS1;CDH13;TNIK;SLIT2;GPC6;DDR2*
HMBOX1	21/299	7.765824510322848E-6	*NCOA1;MAST4;KMT2C;PDE4D;TCF12;ATP2B4;PRRC2C;ZBTB20;PDE4DIP;ANK2;RORA;LMO7;SSH2;LYST;BACH2;NBEA;TRPS1;TNIK;SOX6;UTRN;DDR2*
LMX1B	21/299	7.765824510322848E-6	*CADPS2;CNTNAP2;NTRK2;RBFOX1;CASZ1;AUTS2;CELF4;PAX3;RORA;ESRRG;NRG1;ANK1;BACH2;CDH4;DPP6;PAX7;ERBB4;KIF26B;CDH13;SOX5;DDR2*
PAX6	21/299	7.765824510322848E-6	*CADPS2;NTRK2;NRXN1;PAX3;ZBTB20;ANK2;RORA;MITF;ESRRG;NRG1;ANK1;HDAC9;PAX7;ERBB4;TRPS1;DNER;SLIT2;SOX6;BMPR1B;SOX5;DDR2*
PRDM13	21/299	7.765824510322848E-6	*RYR2;NTRK2;RBFOX1;CASZ1;DCC;PDE4D;NRXN1;CELF4;NRXN3;PAX3;RORA;ESRRG;NRG1;GRIK2;ANK1;CDH4;DPP6;PPP2R2C;PAX7;SLIT2;RNF220*
ZBTB7C	21/299	7.765824510322848E-6	*CADPS2;NTRK2;CASZ1;TENM2;PARM1;AUTS2;PDE4D;RORA;LMO7;NRG1;ANK1;PTPRD;CDH4;PLCB4;MYO5B;KIF26B;CDH13;SLIT2;SOX6;SOX5;DDR2*
ZNF233	21/299	7.765824510322848E-6	*ROBO2;CADPS2;CNTNAP2;ATRNL1;KMT2C;PDE4D;DNAH5;PCDH7;FAM13A;NRXN3;ZBTB20;PDE4DIP;RORA;LMO7;ESRRG;GRIK2;ARHGAP24;PTPRD;PLCB4;NBEA;DMD*
ZNF287	21/299	7.765824510322848E-6	*CADPS2;AUTS2;NEGR1;PDE4D;PCDH7;ATP2B4;ZBTB20;PDE4DIP;ANK2;RORA;LMO7;GRIK2;LYST;BACH2;PLCB4;SGCD;NBEA;TRPS1;TOX;SOX5;DDR2*
ASCL4	20/299	2.6571888532941092E-5	*CADPS2;NTRK2;RBFOX1;CASZ1;AUTS2;DNAH8;PDE4D;NRXN1;PCDH7;NRXN3;PAX3;ESRRG;ANK1;NELL1;DPP6;DLG2;DAB1;PAX7;KIF26B;DDR2*
BAZ2B	20/299	2.6571888532	*NCOA1;CADPS2;AUTS2;ANKRD44;KMT2C;PDE4D;T*

		941092E-5	*CF12;PRRC2C;ZBTB20;RORA;SSH2;PPP1R9A;LYST;PTPRD;STAG2;NBEA;TRPS1;ZNF638;UTRN;DDR2*
BNC2	20/299	2.6571888532941092E-5	*AUTS2;NEGR1;PDE4D;PCDH7;ZBTB20;ANK2;RORA;MITF;ARHGAP24;PTPRD;SGCD;NAV3;KIF26B;TRPS1;CDH13;SLIT2;GPC6;EDIL3;SOX5;DDR2*
CUX1	20/299	2.6571888532941092E-5	*MAGI1;DYNC1H1;AUTS2;IQSEC1;KMT2C;PDE4D;TCF12;DNMT3A;ATP2B4;PTPRM;ZBTB20;PDE4DIP;RORA;ANK1;BACH2;ETV6;PTPRD;TRPS1;UTRN;DDR2*
FOXP1	20/299	2.6571888532941092E-5	*MAGI1;AUTS2;MAST4;KMT2C;PDE4D;TCF12;DNMT3A;PTPRM;ZBTB20;ANK2;RORA;LMO7;ANK1;HDAC9;BACH2;ETV6;TRPS1;UTRN;SOX5;DDR2*
HIVEP3	20/299	2.6571888532941092E-5	*RAP1GAP2;CASZ1;AUTS2;MAST4;PDE4D;PCDH7;DNMT3A;ATP2B4;ZBTB20;PDE4DIP;RORA;ANK1;HDAC9;BACH2;ETV6;KIF26B;TRPS1;TNIK;SOX5;DDR2*
IRX6	20/299	2.6571888532941092E-5	*RYR2;CADPS2;NTRK2;RBFOX1;PARM1;PCDH7;RORA;ESRRG;ANK1;CDH4;DAB1;PAX7;KIF26B;DNER;GPC3;CDH13;SLIT2;SOX6;CSMD1;DDR2*
LCORL	20/299	2.6571888532941092E-5	*CADPS2;CERS6;AUTS2;NEGR1;KMT2C;PDE4D;NRXN1;TCF12;ZBTB20;RORA;LMO7;HDAC9;BACH2;PTPRD;DLG2;NBEA;TRPS1;SOX6;UTRN;DDR2*
NEUROD1	20/299	2.6571888532941092E-5	*ROBO2;PCSK1;CADPS2;CNTNAP2;NTRK2;RBFOX1;NEGR1;NRXN1;CADPS;CELF4;ESRRG;GRIK2;NOL4;DPP6;RALYL;FGF14;DAB1;NBEA;DNER;RIMBP2*
NR2E1	20/299	2.6571888532941092E-5	*CADPS2;NTRK2;RBFOX1;DCC;NRXN3;PAX3;RORA;ESRRG;GRIK2;ANK1;NOL4;CDH4;DAB1;PAX7;CTNNA2;SLIT2;SOX6;BMPR1B;SOX5;DDR2*
PAX2	20/299	2.6571888532941092E-5	*CADPS2;CNTNAP2;NTRK2;RBFOX1;CASZ1;PCDH7;CELF4;NRXN3;PAX3;RORA;ESRRG;NRG1;ANK1;BACH2;CDH4;DPP6;PAX7;KIF26B;SOX6;DDR2*
PGR	20/299	2.6571888532941092E-5	*ROBO2;RYR2;CADPS2;CNTNAP2;NTRK2;NEGR1;NRXN1;PCDH7;RORA;MITF;ESRRG;HDAC9;NAV3;ERBB4;TRPS1;DMD;CNTN4;SLIT2;SOX6;DDR2*
PRDM6	20/299	2.6571888532941092E-5	*ROBO2;RYR2;CADPS2;NTRK2;CASZ1;PARM1;PCDH7;RORA;ESRRG;ANK1;PTPRD;KIF26B;PDE3A;FAT3;SLIT2;SOX6;GPC6;EDIL3;SOX5;DDR2*
RBM20	20/299	2.6571888532941092E-5	*RYR2;CADPS2;NTRK2;CASZ1;PARM1;PDE4D;PCDH7;RFTN1;PDE4DIP;ANK2;RORA;LMO7;ESRRG;ANK1;BACH2;KIF26B;CDH13;FAT3;SOX6;DDR2*
RERE	20/299	2.6571888532941092E-5	*MAGI1;DYNC1H1;AUTS2;MAST4;IQSEC1;KMT2C;PDE4D;TCF12;DNMT3A;ATP2B4;ZBTB20;PDE4DIP;ANK2;RORA;LMO7;ANK1;BACH2;UTRN;LPIN1;DDR2*
RFX6	20/299	2.6571888532941092E-5	*ROBO2;PCSK1;CADPS2;CNTNAP2;NTRK2;TENM2;PARM1;KCNB2;NRXN1;PCDH7;CADPS;ESRRG;NOL4;CDH6;PTPRD;KIF26B;GPC3;RIMBP2;BMPR1B;FREM2*
ZNF236	20/299	2.6571888532941092E-5	*NCOA1;MAGI1;DENND1B;DYNC1H1;CADPS2;KDM4B;AUTS2;KMT2C;TCF12;PRRC2C;PDE4DIP;RORA;SSH2;BACH2;PTPRG;MKLN1;NBEA;TRPS1;UTRN;DDR2*
ZNF608	20/299	2.6571888532941092E-5	*MAGI1;CADPS2;AUTS2;MAST4;PDE4D;PCDH7;ZBTB20;PDE4DIP;RORA;HDAC9;BACH2;ARHGAP24;NBEA;KIF26B;TRPS1;TNIK;SLIT2;UTRN;TOX;DDR2*
ZNF711	20/299	2.6571888532941092E-5	*ROBO2;CADPS2;CNTNAP2;NTRK2;AUTS2;PCDH7;RORA;BACH2;PTPRD;PLCB4;NBEA;TRPS1;GPC3;DMD;SLIT2;SOX6;TOX;FGF13;EDIL3;DDR2*
ZNF781	20/299	2.6571888532941092E-5	*CADPS2;NTRK2;NEGR1;PDE4D;NRXN1;PCDH7;NRXN3;ANK2;RORA;ESRRG;HDAC9;BACH2;NBEA;DMD;CNTN4;SLIT2;SOX6;ST6GALNAC3;SOX5;DDR2*
ZSCAN20	20/299	2.6571888532	*DENND1B;CADPS2;CASZ1;AUTS2;KMT2C;PDE4D;A*

		941092E-5	*TP2B4;PDE4DIP;LMO7;ANK1;LYST;HDAC9;BACH2;ARHGAP24;DOCK10;KIF26B;TRPS1;LPIN1;SOX5;DDR2*
BACH2	19/299	8.622431470174563E-5	*CADPS2;AUTS2;PDE4D;PLCL1;ZBTB20;ANK2;RORA;ANK1;HDAC9;PTPRD;KIF26B;TRPS1;TNIK;SLIT2;SOX6;UTRN;TOX;SOX5;DDR2*
DMRTB1	19/299	8.622431470174563E-5	*RYR2;CADPS2;NTRK2;CASZ1;CADPS;ESRRG;ANK1;CDH4;DPP6;DAB1;PAX7;ERBB4;KIF26B;GPC3;SOX6;BMPR1B;SOX5;RNF220;DDR2*
FERD3L	19/299	8.622431470174563E-5	*ROBO2;RYR2;CADPS2;RBFOX1;PCDH7;CELF4;PAX3;ESRRG;NRG1;GRIK2;ADARB2;ANK1;NELL1;DPP6;LRFN5;NRG3;PAX7;SLIT2;CSMD1*
FOXN3	19/299	8.622431470174563E-5	*MAGI1;AUTS2;MAST4;KMT2C;PDE4D;TCF12;ATP2B4;ZBTB20;ANK2;RORA;ANK1;BACH2;PTPRG;ETV6;PTPRD;TRPS1;UTRN;LPIN1;DDR2*
HNF4G	19/299	8.622431470174563E-5	*ROBO2;CADPS2;PCDH7;ZBTB20;RORA;MITF;LMO7;ESRRG;HDAC9;CDH6;PTPRD;TRPS1;SLIT2;SOX6;TOX;BMPR1B;EDIL3;SOX5;DDR2*
IKZF2	19/299	8.622431470174563E-5	*CADPS2;MAST4;ANKRD44;PDE4D;PCDH7;ATP2B4;ZBTB20;RORA;LMO7;ANK1;HDAC9;BACH2;DOCK10;TRPS1;TNIK;SOX6;UTRN;TOX;DDR2*
ISX	19/299	8.622431470174563E-5	*DPP10;PCSK1;CADPS2;CNTNAP2;AUTS2;DNAH5;NRXN1;RORA;LMO7;ESRRG;PTPRD;MGAT4C;ACE2;DLG2;MYO5B;KIF26B;SOX6;EDIL3;TRPM3*
LHX4	19/299	8.622431470174563E-5	*RAP1GAP2;DPP10;CADPS2;CASZ1;NEGR1;NRXN1;ATP2B4;PAX3;RORA;ESRRG;ANK1;BACH2;CDH4;DLG2;PAX7;ERBB4;KIF26B;DNER;DDR2*
LHX8	19/299	8.622431470174563E-5	*CADPS2;NTRK2;NEGR1;DCC;PAX3;ESRRG;GRIK2;ANK1;CDH6;CDH4;DAB1;PAX7;KIF26B;CDH13;CNTN4;SLIT2;SOX6;BRINP3;DDR2*
LHX9	19/299	8.622431470174563E-5	*ROBO2;CADPS2;NTRK2;AUTS2;NEGR1;PCDH7;PAX3;RORA;ESRRG;NRG1;GRIK2;ANK1;PAX7;KIF26B;CDH13;SLIT2;SOX6;SOX5;DDR2*
POU3F3	19/299	8.622431470174563E-5	*CADPS2;NTRK2;AUTS2;DCC;NRXN1;PCDH7;PAX3;ESRRG;GRIK2;CDH4;DPP6;DAB1;PAX7;ERBB4;DNER;SLIT2;SOX6;SOX5;DDR2*
RAPGEF5	19/299	8.622431470174563E-5	*MAGI1;CADPS2;NTRK2;PARM1;AUTS2;PDE4D;PCDH7;PTPRM;ZBTB20;RORA;LMO7;ANK1;HDAC9;DOCK10;PTPRD;PLCB4;TNIK;TOX;DDR2*
SHOX	19/299	8.622431470174563E-5	*RYR2;NTRK2;RBFOX1;NRXN1;PCDH7;NRXN3;PAX3;ADCY2;MITF;NRG1;GRIK2;CDH4;DPP6;GRM7;PAX7;CDH13;CNTN4;CSMD1;DDR2*
TBX20	19/299	8.622431470174563E-5	*ROBO2;RYR2;CADPS2;NTRK2;RBFOX1;CASZ1;PCDH7;RORA;ESRRG;NRG1;ANK1;PTPRD;KIF26B;PDE3A;CDH13;SLIT2;SOX6;SOX5;DDR2*
TRERF1	19/299	8.622431470174563E-5	*RAP1GAP2;AUTS2;MAST4;PDE4D;PCDH7;DNMT3A;ATP2B4;RORA;LMO7;ANK1;HDAC9;ETV6;DOCK10;TRPS1;TNIK;UTRN;TOX;LPIN1;DDR2*
TSHZ3	19/299	8.622431470174563E-5	*AUTS2;PCDH7;ATP2B4;PTPRM;RORA;NRG1;ANK1;ARHGAP24;PTPRD;NAV3;KIF26B;TRPS1;CDH13;SLIT2;TOX;GPC6;EDIL3;SOX5;DDR2*
ZMAT1	19/299	8.622431470174563E-5	*CADPS2;PARM1;NEGR1;PDE4D;ZBTB20;RORA;PPP1R9A;ANK1;HDAC9;BACH2;DOCK10;STAG2;NBEA;TRPS1;GPC3;DMD;TNIK;TOX;DDR2*
ZNF154	19/299	8.622431470174563E-5	*CADPS2;NTRK2;PARM1;NEGR1;SGIP1;PDE4D;PCDH7;ZBTB20;RORA;LMO7;NRG1;PPP1R9A;BACH2;DOCK10;TRPS1;FAT3;DMD;TOX;DDR2*
ZNF365	19/299	8.622431470174563E-5	*CADPS2;CNTNAP2;NTRK2;NEGR1;PDE4D;PCDH7;NRXN3;RORA;NRG1;HDAC9;BACH2;DOCK10;DLG2;NBEA;NAV3;CDH13;SCN2A;DDR2;JAZF1*
ZNF423	19/299	8.622431470174563E-5	*ROBO2;CADPS2;NTRK2;AUTS2;PDE4D;NRXN1;DNMT3A;PTPRM;ANK2;RORA;BACH2;PTPRD;CDH4;NBEA;KIF26B;CDH13;SLIT2;TOX;DDR2*

ZNF618	19/299	8.622431470174563E-5	*MAGI1;CADPS2;AUTS2;PDE4D;PCDH7;DNMT3A;ATP2B4;ZBTB20;PPP1R9A;HDAC9;BACH2;DOCK10;NBEA;KIF26B;TRPS1;TNIK;SLIT2;UTRN;DDR2*
ZNF677	19/299	8.622431470174563E-5	*RYR2;CADPS2;NEGR1;NRXN1;PCDH7;NRXN3;ZBTB20;ANK2;ADCY2;RORA;NRG1;BACH2;CDH4;NBEA;DMD;SLIT2;UTRN;SOX5;DDR2*
ZNF704	19/299	8.622431470174563E-5	*MAGI1;CADPS2;NTRK2;AUTS2;PDE4D;PCDH7;ZBTB20;RORA;MITF;PPP1R9A;HDAC9;BACH2;PTPRD;NBEA;TRPS1;DMD;SLIT2;SOX6;DDR2*
ZNF774	19/299	8.622431470174563E-5	*CADPS2;PATJ;KDM4B;MAST4;FAM13A;DNMT3A;ATP2B4;PTPRM;ZBTB20;PDE4DIP;ANK2;RORA;LYST;NBEA;DMD;TNIK;UTRN;SOX5;DDR2*
ARNT2	18/299	2.7804844539018115E-4	*RAP1GAP2;CADPS2;CNTNAP2;NTRK2;AUTS2;PCDH7;ANK2;RORA;NRG1;ANK1;PTPRD;NBEA;PPP2R2C;TNIK;SLIT2;TOX;SOX5;DDR2*
ATOH1	18/299	2.7804844539018115E-4	*ROBO2;DPP10;PCSK1;CADPS2;NTRK2;RBFOX1;CASZ1;PCDH7;NRXN3;ESRRG;HDAC9;CDH4;PAX7;ERBB4;SLIT2;TOX;BRINP3;DDR2*
BARHL2	18/299	2.7804844539018115E-4	*CADPS2;NTRK2;RBFOX1;CASZ1;CELF4;PAX3;RORA;ESRRG;ANK1;NELL1;CDH4;DPP6;DAB1;PAX7;ERBB4;KIF26B;SOX6;SOX5*
CHD7	18/299	2.7804844539018115E-4	*DYNC1H1;CASZ1;AUTS2;MAST4;KMT2C;PDE4D;TCF12;DNMT3A;PRRC2C;ZBTB20;RORA;ANK1;BACH2;ETV6;DOCK10;TRPS1;UTRN;DDR2*
CHD9	18/299	2.7804844539018115E-4	*NCOA1;DYNC1H1;CERS6;KMT2C;PDE4D;TCF12;ATP2B4;PRRC2C;ZBTB20;RORA;LYST;MKLN1;STAG2;TRPS1;ZNF638;TTC3;UTRN;DDR2*
DRGX	18/299	2.7804844539018115E-4	*RYR2;NTRK2;RBFOX1;CASZ1;NRXN1;CELF4;NRXN3;PAX3;RORA;ESRRG;ANK1;NELL1;CDH4;DPP6;PAX7;CDH13;SOX6;SOX5*
NR2E3	18/299	2.7804844539018115E-4	*CADPS2;NTRK2;CASZ1;AUTS2;NRXN3;PAX3;RORA;MITF;ESRRG;NRG1;GRIK2;BACH2;KIF26B;TRPS1;SOX6;TOX;SOX5;DDR2*
PKHD1L1	18/299	2.7804844539018115E-4	*RYR2;CADPS2;NRXN1;PCDH15;ATP2B4;ANK2;ANK1;DOCK10;PTPRD;DLG2;ERBB4;KIF26B;DMD;SLIT2;SOX6;CSMD1;FREM2;DDR2*
RGS9	18/299	2.7804844539018115E-4	*RAP1GAP2;PCSK1;CADPS2;NTRK2;NEGR1;DGKB;PCDH7;ATP2B4;RORA;GRIK2;ANK1;CDH4;DLG2;MYO5B;CDH13;TOX;SOX5;DDR2*
RREB1	18/299	2.7804844539018115E-4	*CASZ1;AUTS2;IQSEC1;KMT2C;PDE4D;TCF12;DNMT3A;ATP2B4;ZBTB20;PDE4DIP;RORA;LMO7;ANK1;BACH2;ETV6;TRPS1;UTRN;DDR2*
SALL1	18/299	2.7804844539018115E-4	*CADPS2;NTRK2;AUTS2;GLDC;PCDH7;ANK2;ESRRG;NRG1;ANK1;PTPRD;KIF26B;TRPS1;SLIT2;SOX6;TOX;GPC6;SOX5;DDR2*
SOX1	18/299	2.7804844539018115E-4	*NTRK2;RBFOX1;CASZ1;DCC;NRXN1;PCDH7;CELF4;NRXN3;PAX3;RORA;ESRRG;CDH4;DPP6;DAB1;PAX7;SLIT2;SOX6;SOX5*
TIGD4	18/299	2.7804844539018115E-4	*CHODL;CADPS2;CNTNAP2;PDE4D;PLCL1;ULK4;ADAM22;ANK2;ESRRG;PPP1R9A;ANK1;HDAC9;RASGEF1B;DMD;CTNNA3;SOX6;BMPR1B;DDR2*
ZNF382	18/299	2.7804844539018115E-4	*ROBO2;CADPS2;CNTNAP2;PARM1;PLCL1;NRXN3;KIAA1549L;ZBTB20;RORA;GRIK2;HDAC9;CDH6;NBEA;NAV3;DMD;TNIK;TOX;DDR2*
ZNF516	18/299	2.7804844539018115E-4	*RAP1GAP2;KDM4B;AUTS2;IQSEC1;KMT2C;PDE4D;ATP2B4;ZBTB20;PDE4DIP;RORA;HDAC9;BACH2;NBEA;TRPS1;TNIK;UTRN;LPIN1;DDR2*
ZNF626	18/299	2.7804844539018115E-4	*MAGI1;CADPS2;CNTNAP2;NEGR1;PDE4D;PTPRM;ZBTB20;PDE4DIP;RORA;PPP1R9A;HDAC9;NBEA;TRPS1;DMD;SLIT2;UTRN;EDIL3;DDR2*
ZNF793	18/299	2.7804844539018115E-4	*RAP1GAP2;RYR2;CADPS2;PATJ;AUTS2;NEGR1;PDE4D;PCDH7;RORA;PPP1R9A;ANO5;HDAC9;BACH2;PTPRD;NBEA;DMD;CNTN4;DDR2*

DBX1	17/299	8.1936032366 00101E-4	*NTRK2;RBFOX1;CASZ1;DCC;PCDH7;CELF4;PAX3;ESRRG;ANK1;NELL1;CDH4;DPP6;PAX7;ERBB4;KIF26B;SLIT2;SOX6*
DMRT3	17/299	8.1936032366 00101E-4	*CADPS2;CNTNAP2;NTRK2;PCDH7;PAX3;RORA;ESRRG;NRG1;ANK1;CDH6;NELL1;CDH4;PAX7;SLIT2;SOX6;SOX5;DDR2*
DMRTA2	17/299	8.1936032366 00101E-4	*CADPS2;NTRK2;RBFOX1;CASZ1;PCDH7;CELF4;PAX3;RORA;ESRRG;NRG1;ANK1;CDH4;DPP6;PAX7;KIF26B;SLIT2;SOX6*
EBF3	17/299	8.1936032366 00101E-4	*CADPS2;NTRK2;CASZ1;AUTS2;PCDH7;PTPRM;ESRRG;ANK1;BACH2;PTPRD;KIF26B;GPC3;CDH13;SLIT2;SOX6;GPC6;DDR2*
FOXI2	17/299	8.1936032366 00101E-4	*RYR2;CNTNAP2;RBFOX1;NRXN1;PCDH7;CELF4;NRXN3;ESRRG;NRG1;CDH4;DPP6;DAB1;NBEA;GRM7;PAX7;CDH13;CTNNA2*
GLI3	17/299	8.1936032366 00101E-4	*NTRK2;AUTS2;PCDH7;PTPRM;ANK2;RORA;MITF;BACH2;PTPRG;KIF26B;TRPS1;SLIT2;SOX6;BMPR1B;GPC6;SOX5;DDR2*
GTF2IRD 2B	17/299	8.1936032366 00101E-4	*MAGI1;CADPS2;CASZ1;ARHGEF37;AUTS2;MAST4;KMT2C;PDE4D;PLCL1;PTPRM;ZBTB20;PDE4DIP;RORA;LMO7;LYST;NBEA;DDR2*
HSF5	17/299	8.1936032366 00101E-4	*CADPS2;DCC;NRXN3;RORA;LMO7;ESRRG;ANK1;HDAC9;BACH2;SH3RF3;CDH13;GPC5;TOX;DOCK2;FREM2;DDR2;JAZF1*
INSM1	17/299	8.1936032366 00101E-4	*ROBO2;PCSK1;CADPS2;NTRK2;AUTS2;NRXN1;CADPS;CELF4;ESRRG;ANK1;NOL4;BACH2;CDH4;DNER;RIMBP2;TOX;DDR2*
JAZF1	17/299	8.1936032366 00101E-4	*RAP1GAP2;CADPS2;NEGR1;PDE4D;PLCL1;PCDH7;ATP2B4;ZBTB20;ANK2;RORA;ANK1;HDAC9;BACH2;DOCK10;TOX;GPC6;DDR2*
MKRN3	17/299	8.1936032366 00101E-4	*CADPS2;CNTNAP2;NRXN1;CADPS;RORA;GRIK2;HDAC9;BACH2;NAV3;ERBB4;DNER;SLIT2;TOX;FGF13;EDIL3;ASTN1;DDR2*
NFIB	17/299	8.1936032366 00101E-4	*MAGI1;CADPS2;NTRK2;AUTS2;PDE4D;PTPRM;ZBTB20;ANK2;RORA;LMO7;PTPRD;NBEA;TRPS1;SLIT2;UTRN;SOX5;DDR2*
ONECUT1	17/299	8.1936032366 00101E-4	*CADPS2;NTRK2;CASZ1;NRXN1;PAX3;RORA;ESRRG;NRG1;ANK1;CDH6;CDH4;PAX7;ERBB4;KIF26B;SOX6;SOX5;DDR2*
PHF21A	17/299	8.1936032366 00101E-4	*NCOA1;KDM4B;AUTS2;KMT2C;PDE4D;TCF12;DNMT3A;ATP2B4;PRRC2C;PTPRM;ZBTB20;ANK2;RORA;ETV6;TRPS1;UTRN;DDR2*
PRDM15	17/299	8.1936032366 00101E-4	*RAP1GAP2;CNTNAP2;KDM4B;AUTS2;MAST4;IQSEC1;KMT2C;PDE4D;DNMT3A;PDE4DIP;SSH2;ANK1;BACH2;ETV6;TRPS1;LPIN1;DDR2*
SCRT2	17/299	8.1936032366 00101E-4	*ROBO2;RBFOX1;CASZ1;AUTS2;DCC;NRXN1;CADPS;CELF4;NRXN3;ADARB2;ANK1;CDH4;DPP6;DAB1;KIF26B;DNER;DSCAML1*
SIX3	17/299	8.1936032366 00101E-4	*CADPS2;NTRK2;CASZ1;NRXN1;PCDH7;CELF4;PAX3;ZBTB20;RORA;ESRRG;ANK1;CDH6;CDH4;DPP6;PAX7;RGS9;DDR2*
TRPS1	17/299	8.1936032366 00101E-4	*CADPS2;AUTS2;PDE4D;PCDH7;ATP2B4;ZBTB20;RORA;HDAC9;DOCK10;PTPRD;NBEA;TNIK;SLIT2;UTRN;TOX;SOX5;DDR2*
ZNF343	17/299	8.1936032366 00101E-4	*MAGI1;RAP1GAP2;CADPS2;NBAS;PDE4D;TCF12;DNMT3A;ATP2B4;PDE4DIP;HDAC9;BACH2;DOCK10;PLCB4;TTC3;MACROD2;TNIK;DDR2*
ZNF415	17/299	8.1936032366 00101E-4	*CADPS2;PARM1;PDE4D;ZBTB20;PDE4DIP;ANK2;RORA;PPP1R9A;HDAC9;PLCB4;NBEA;DNER;DMD;SLIT2;TOX;EDIL3;DDR2*
ZNF43	17/299	8.1936032366 00101E-4	*CADPS2;NEGR1;DNMT3A;PTPRM;ZBTB20;PDE4DIP;RORA;PPP1R9A;HDAC9;PLCB4;NBEA;TRPS1;DMD;SLIT2;TOX;EDIL3;DDR2*

ZNF546	17/299	8.193603236600101E-4	*ROBO2;DENND1B;CADPS2;NEGR1;PDE4D;FAM13A;ZBTB20;HDAC9;BACH2;DOCK10;PLCB4;NBEA;TMEM117;TRPS1;SOX6;SOX5;DDR2*
ZNF596	17/299	8.193603236600101E-4	*RYR2;CADPS2;MAST4;NEGR1;PDE4D;ZBTB20;PDE4DIP;RORA;LMO7;NRG1;HDAC9;BACH2;ARHGAP24;NBEA;TRPS1;DMD;DDR2*
ZNF599	17/299	8.193603236600101E-4	*ROBO2;CADPS2;CASZ1;AUTS2;ANKRD44;PDE4D;NRXN3;ZBTB20;PDE4DIP;NRG1;ANK1;NBEA;KIF26B;DNER;FAM20C;TOX;DDR2*
ZNF645	17/299	8.193603236600101E-4	*NCOA1;DPP10;NTRK2;RBFOX1;ZBTB20;DACH2;PHEX;TMEM164;LRMDA;SCEL;STAG2;PAX7;ERBB4;CDH13;DMD;MACROD2;DDR2*
ZNF681	17/299	8.193603236600101E-4	*RYR2;CADPS2;CNTNAP2;AUTS2;GLDC;KMT2C;PDE4D;PDE4DIP;RORA;LMO7;HDAC9;BRIP1;NBEA;FAT3;TOX;FREM2;DDR2*
ATOH7	16/299	0.002275772749748585	*PCSK1;CADPS2;NTRK2;NEGR1;DGKB;SGIP1;ATP2B4;ESRRG;ANK1;DLG2;DAB1;KIF26B;CTNNA3;SOX6;RGS9;DDR2*
ATXN7	16/299	0.002275772749748585	*NCOA1;AUTS2;KMT2C;PDE4D;TCF12;PRRC2C;ZBTB20;RORA;SSH2;LYST;BACH2;ETV6;TRPS1;TNIK;UTRN;DDR2*
CASZ1	16/299	0.002275772749748585	*RAP1GAP2;CADPS2;AUTS2;MAST4;PDE4D;ATP2B4;ZBTB20;KAZN;PDE4DIP;RORA;LMO7;ESRRG;ANK1;BACH2;KIF26B;DDR2*
DMRTA1	16/299	0.002275772749748585	*CADPS2;CNTNAP2;NTRK2;NEGR1;PCDH7;ESRRG;HDAC9;CDH6;CDH4;SLIT2;SOX6;TOX;BMPR1B;GPC6;EDIL3;DDR2*
DZIP1	16/299	0.002275772749748585	*CADPS2;NTRK2;NEGR1;PCDH7;ATP2B4;ANK2;ANK1;PTPRD;NBEA;TRPS1;DMD;SLIT2;GPC6;MPDZ;EDIL3;DDR2*
EBF1	16/299	0.002275772749748585	*NTRK2;AUTS2;RFTN1;PTPRM;ZBTB20;ANK2;RORA;ANK1;HDAC9;BACH2;ARHGAP24;CDH13;SLIT2;TOX;GPC6;DDR2*
FOXD4L3	16/299	0.002275772749748585	*RYR2;RBFOX1;CASZ1;DSCAM;PCDH7;PAX3;PDE4DIP;ESRRG;NRG1;DPP6;PAX7;ERBB4;KIF26B;SLIT2;TOX;CSMD1*
FOXR2	16/299	0.002275772749748585	*CADPS2;TENM2;CNTN5;GLDC;DCC;NRXN1;DACH2;NOL4;PHEX;FSTL5;DAB1;DNER;GPC3;SLIT2;CDH18;DDR2*
GCM2	16/299	0.002275772749748585	*PCSK1;RYR2;NTRK2;RBFOX1;DCC;NRXN1;CELF4;PAX3;RORA;ESRRG;GRIK2;ANK1;FGF14;PAX7;CSMD1;DDR2*
IRX4	16/299	0.002275772749748585	*RAP1GAP2;CADPS2;CNTNAP2;NTRK2;RBFOX1;CASZ1;TENM2;NRXN1;PCDH7;ESRRG;ANK1;PPP2R2C;PAX7;KIF26B;CDH13;SLIT2*
LHX5	16/299	0.002275772749748585	*CADPS2;NTRK2;RBFOX1;CASZ1;NRXN1;CELF4;NRXN3;PAX3;RORA;ESRRG;ANK1;CDH6;CDH4;DPP6;PAX7;KIF26B*
LIN28B	16/299	0.002275772749748585	*ROBO2;TRIM71;CADPS2;CNTNAP2;GLDC;DNMT3A;NRXN3;RORA;ESRRG;HDAC9;PTPRD;GPC3;TNIK;SLIT2;GPC6;DDR2*
NR5A2	16/299	0.002275772749748585	*CADPS2;NTRK2;CASZ1;PDE4D;RORA;ESRRG;NRG1;ANK1;BACH2;CDH6;ERBB4;KIF26B;TRPS1;SLIT2;SOX6;DDR2*
OLIG3	16/299	0.002275772749748585	*PCSK1;CADPS2;NTRK2;RBFOX1;CASZ1;NRXN1;CELF4;PAX3;ESRRG;BACH2;CDH6;PAX7;ERBB4;KIF26B;SLIT2;DDR2*
PAX4	16/299	0.002275772749748585	*PCSK1;CADPS2;NEGR1;DGKB;DNAH5;CADPS;ADAM22;PAX3;ESRRG;MGAT4C;DLG2;PDE3A;SOX6;RGS9;TRPM3;SOX5*
PAX5	16/299	0.002275772749748585	*CADPS2;CNTNAP2;CASZ1;AUTS2;PAX3;RORA;ESRRG;ANK1;HDAC9;BACH2;PAX7;ERBB4;TOX;DOCK2;SOX5;DDR2*

POU2F1	16/299	0.002275772749748585	*NCOA1;AUTS2;KMT2C;PDE4D;TCF12;ATP2B4;PRRC2C;ZBTB20;RORA;BACH2;ETV6;TRPS1;SOX6;UTRN;SOX5;DDR2*
PTF1A	16/299	0.002275772749748585	*RYR2;CADPS2;RBFOX1;CASZ1;CELF4;PAX3;ESRRG;ANK1;NOL4;NELL1;CDH4;DPP6;FGF14;NBEA;PAX7;ERBB4*
SATB1	16/299	0.002275772749748585	*CADPS2;AUTS2;PDE4D;ATP2B4;ZBTB20;RORA;ANK1;HDAC9;BACH2;DOCK10;NBEA;TRPS1;TNIK;UTRN;TOX;DDR2*
SP8	16/299	0.002275772749748585	*ROBO2;CADPS2;CNTNAP2;PCDH7;CELF4;NRXN3;PAX3;BACH2;CDH6;CDH4;PAX7;KIF26B;SLIT2;SOX6;TOX;DDR2*
TFDP3	16/299	0.002275772749748585	*PCSK1;TENM2;NRXN1;CADPS;ZBTB20;HTR2C;RORA;HDAC9;FSTL5;PLCB4;GPC3;CDH12;DMD;FGF13;CDH18;DDR2*
ZFHX3	16/299	0.002275772749748585	*CASZ1;AUTS2;KMT2C;PDE4D;ATP2B4;ZBTB20;PDE4DIP;RORA;LMO7;ANK1;BACH2;ETV6;KIF26B;TRPS1;UTRN;DDR2*
ZFR2	16/299	0.002275772749748585	*RAP1GAP2;RYR2;CADPS2;CNTNAP2;RBFOX1;NRXN1;DNMT3A;CELF4;ADCY2;RORA;ANK1;CDH4;DPP6;CTNNA2;ASTN1;DDR2*
ZIC1	16/299	0.002275772749748585	*CADPS2;NTRK2;RBFOX1;TENM2;PCDH7;PAX3;ESRRG;GRIK2;CDH6;PTPRD;TRPS1;DNER;CDH13;SLIT2;SOX6;DDR2*
ZNF221	16/299	0.002275772749748585	*RYR2;CADPS2;MAST4;KMT2C;PDE4D;PCDH7;PDE4DIP;RORA;DOCK10;BRIP1;NBEA;NAV3;TRPS1;FREM2;SOX5;DDR2*
ZNF506	16/299	0.002275772749748585	*CADPS2;AUTS2;ANKRD44;PDE4D;ZBTB20;PDE4DIP;RORA;LMO7;LYST;BACH2;NBEA;TRPS1;DMD;TNIK;UTRN;DDR2*
ZNF532	16/299	0.002275772749748585	*AUTS2;KMT2C;PDE4D;TCF12;ATP2B4;PTPRM;ZBTB20;ANK2;RORA;NRG1;HDAC9;BACH2;NBEA;TRPS1;UTRN;DDR2*
ZNF667	16/299	0.002275772749748585	*CADPS2;CNTNAP2;AUTS2;NEGR1;NRXN1;PTPRM;ANK2;RORA;GRIK2;PPP1R9A;HDAC9;BACH2;NBEA;TOX;SOX5;DDR2*
ZNF81	16/299	0.002275772749748585	*NCOA1;DENND1B;CADPS2;KMT2C;TCF12;ATP2B4;PRRC2C;PDE4DIP;ANK2;LYST;MKLN1;STAG2;TRPS1;ZNF638;DMD;UTRN*
ASH1L	15/299	0.005967889783620459	*NCOA1;DYNC1H1;KMT2C;DNMT3A;PRRC2C;ZBTB20;ANK2;LYST;STAG2;NBEA;ZNF638;TTC3;SCN2A;UTRN;DDR2*
ETV1	15/299	0.005967889783620459	*CADPS2;NTRK2;PCDH7;ANK2;RORA;ANK1;HDAC9;PTPRD;PLCB4;NAV3;TRPS1;SLIT2;EDIL3;SOX5;DDR2*
FOXO6	15/299	0.005967889783620459	*RAP1GAP2;CADPS2;CASZ1;AUTS2;RORA;ESRRG;ANK1;BACH2;CDH4;PPP2R2C;MYO5B;KIF26B;CDH13;TOX;DDR2*
GATA4	15/299	0.005967889783620459	*RYR2;CASZ1;PCDH7;PAX3;RORA;MITF;ESRRG;NRG1;ANK1;PTPRD;PAX7;KIF26B;GPC3;SOX6;DDR2*
HELZ	15/299	0.005967889783620459	*NCOA1;DYNC1H1;KMT2C;ATP2B4;PRRC2C;ZBTB20;RORA;SSH2;LYST;MKLN1;STAG2;TRPS1;ZNF638;UTRN;DDR2*
ISL1	15/299	0.005967889783620459	*ROBO2;PCSK1;CADPS2;NTRK2;PARM1;AUTS2;NRXN1;PCDH7;CADPS;CELF4;ESRRG;PTPRD;DNER;SLIT2;DDR2*
KLF8	15/299	0.005967889783620459	*ATP2B4;ANK2;RORA;MITF;ANK1;HDAC9;BACH2;TRPS1;GPC3;DMD;TNIK;SOX6;FGF13;SOX5;DDR2*
MEIS1	15/299	0.005967889783620459	*CADPS2;NTRK2;AUTS2;PDE4D;PCDH7;ATP2B4;ZBTB20;RORA;MITF;PTPRD;TRPS1;SLIT2;SOX6;GPC6;DDR2*
NEUROG2	15/299	0.0059678897	*CADPS2;NTRK2;CASZ1;PAX3;ESRRG;ANK1;BACH2;CDH4;PAX7;ERBB4;KIF26B;SLIT2;SOX6;TOX;S*

		83620459	*OX5*
NRK	15/299	0.0059678897 83620459	*ROBO2;CADPS2;NTRK2;PARM1;PCDH7;ANK1;PTPRD;GPC3;DMD;SLIT2;FGF13;GPC6;EDIL3;FREM2;DDR2*
OTX2	15/299	0.0059678897 83620459	*TRIM71;CADPS2;CNTNAP2;NTRK2;RBFOX1;CASZ1;NRXN1;PCDH7;PAX3;ESRRG;DAB1;PAX7;GPC3;SLIT2;TRPM3*
PAX1	15/299	0.0059678897 83620459	*NTRK2;RBFOX1;CASZ1;PCDH7;CELF4;PAX3;ESRRG;ANK1;CDH4;DPP6;PAX7;ERBB4;KIF26B;SLIT2;DDR2*
PHOX2B	15/299	0.0059678897 83620459	*NTRK2;RBFOX1;NRXN1;CELF4;PAX3;RORA;ESRRG;GRIK2;DPP6;PAX7;ERBB4;SLIT2;SOX6;SOX5;DDR2*
PRB4	15/299	0.0059678897 83620459	*MAGI1;PCSK1;RYR2;NTRK2;TENM2;CNTN5;PDE4D;PDE4DIP;ANK2;NRG1;PTPRD;CTNNA3;UTRN;DYNC1I1;DDR2*
SHPRH	15/299	0.0059678897 83620459	*KMT2C;TCF12;PRRC2C;ZBTB20;LYST;MKLN1;STAG2;NBEA;ZNF638;TTC3;NEK1;DMD;TNIK;UTRN;DDR2*
SSH2	15/299	0.0059678897 83620459	*IQSEC1;ANKRD44;KMT2C;PDE4D;ATP2B4;PRRC2C;ZBTB20;RORA;LYST;BACH2;ETV6;DOCK10;UTRN;DOCK2;DDR2*
TCF20	15/299	0.0059678897 83620459	*DYNC1H1;CERS6;AUTS2;MAST4;IQSEC1;KMT2C;DNMT3A;ATP2B4;PRRC2C;ZBTB20;RORA;LMO7;TRPS1;UTRN;DDR2*
TEAD1	15/299	0.0059678897 83620459	*MAGI1;AUTS2;PDE4D;ATP2B4;ZBTB20;RORA;MITF;LMO7;BACH2;PTPRG;TRPS1;SOX6;UTRN;SOX5;DDR2*
TFDP2	15/299	0.0059678897 83620459	*PDE4D;TCF12;DNMT3A;ATP2B4;ZBTB20;PDE4DIP;RORA;ANK1;HDAC9;TRPS1;TNIK;SOX6;UTRN;LPIN1;DDR2*
VSX1	15/299	0.0059678897 83620459	*RYR2;CADPS2;NTRK2;RBFOX1;CASZ1;CELF4;NRXN3;PAX3;ESRRG;GRIK2;ANK1;CDH4;DPP6;PAX7;CDH13*
WNT8B	15/299	0.0059678897 83620459	*CADPS2;NTRK2;RBFOX1;HTR2C;ESRRG;GRIK2;PPP1R9A;CDH6;CDH4;MYO3B;PAX7;ERBB4;BMPR1B;RGS9;DDR2*
ZFP28	15/299	0.0059678897 83620459	*CADPS2;NEGR1;PDE4D;PTPRM;PDE4DIP;RORA;LMO7;ANK1;HDAC9;BACH2;ARHGAP24;NBEA;DMD;SLIT2;DDR2*
ZHX3	15/299	0.0059678897 83620459	*NTRK2;MAST4;KMT2C;ATP2B4;ZBTB20;PDE4DIP;ANK2;RORA;LMO7;SSH2;LYST;TRPS1;UTRN;LPIN1;DDR2*
ZNF254	15/299	0.0059678897 83620459	*DENND1B;CADPS2;PDE4D;TCF12;ATP2B4;ZBTB20;PDE4DIP;RORA;PPP1R9A;HDAC9;PLCB4;NBEA;TRPS1;EDIL3;DDR2*
ZNF280D	15/299	0.0059678897 83620459	*CADPS2;KMT2C;TCF12;FAM13A;ATP2B4;ZBTB20;PDE4DIP;RORA;LYST;DOCK10;STAG2;TRPS1;ZNF638;UTRN;DDR2*
ZNF311	15/299	0.0059678897 83620459	*CADPS2;CNTNAP2;CASZ1;TENM2;NRXN3;NRG1;GRIK2;ANK1;CDH4;KIF26B;DNER;CDH13;SLIT2;TOX;DDR2*
ZNF396	15/299	0.0059678897 83620459	*CADPS2;MAST4;DNAH7;NEGR1;PDE4D;DNAH5;PLCL1;PDE4DIP;RORA;MITF;NBEA;TRPS1;DMD;SOX5;DDR2*
ZNF605	15/299	0.0059678897 83620459	*CADPS2;CNTNAP2;NEGR1;DNMT3A;ZBTB20;LMO7;PPP1R9A;DOCK10;PLCB4;NBEA;TRPS1;ZNF638;TTC3;SOX5;DDR2*
ZNF678	15/299	0.0059678897 83620459	*DENND1B;CADPS2;KMT2C;PDE4D;TCF12;ATP2B4;PRRC2C;PDE4DIP;RORA;HDAC9;BRIP1;TRPS1;TNIK;UTRN;DDR2*
ZNF720	15/299	0.0059678897 83620459	*MAGI1;DENND1B;CADPS2;PATJ;ATP2B4;PRRC2C;PDE4DIP;RORA;HDAC9;MKLN1;ZNF638;UTRN;TOX*

			;ZNF267;DDR2
ZXDC	15/299	0.0059678897 83620459	*DYNC1H1;KDM4B;CERS6;IQSEC1;KMT2C;PDE4D;TCF12;DNMT3A;ZBTB20;PDE4DIP;RORA;ANK1;UTRN;LPIN1;DDR2*
AFF1	14/299	0.0143745180 45711224	*CERS6;MAST4;IQSEC1;KMT2C;ATP2B4;ZBTB20;PDE4DIP;RORA;ANK1;ETV6;TRPS1;UTRN;LPIN1;DDR2*
CREB5	14/299	0.0143745180 45711224	*PDE4D;PCDH7;ZBTB20;ANK2;RORA;MITF;NRG1;HDAC9;BACH2;DOCK10;PTPRD;TRPS1;SOX5;DDR2*
ESR1	14/299	0.0143745180 45711224	*CADPS2;NTRK2;PDE4D;ZBTB20;RORA;MITF;ESRRG;NRG1;ANK1;HDAC9;DLG2;ERBB4;TRPS1;DDR2*
FMNL2	14/299	0.0143745180 45711224	*AUTS2;PDE4D;ATP2B4;PTPRM;ZBTB20;ANK2;RORA;HDAC9;PTPRG;DOCK10;TRPS1;TNIK;UTRN;DDR2*
FOXK1	14/299	0.0143745180 45711224	*DYNC1H1;CASZ1;AUTS2;IQSEC1;PDE4D;DNMT3A;PRRC2C;RORA;LMO7;ANK1;BACH2;ETV6;UTRN;DDR2*
HELT	14/299	0.0143745180 45711224	*CADPS2;NTRK2;CASZ1;DCC;NRXN1;CELF4;PAX3;ESRRG;ANK1;NELL1;DPP6;PAX7;ERBB4;SOX6*
HKR1	14/299	0.0143745180 45711224	*MAGI1;CADPS2;PATJ;CASZ1;KDM4B;KMT2C;NRXN3;ZBTB20;PDE4DIP;RORA;LMO7;NBEA;TNIK;DDR2*
HOXD3	14/299	0.0143745180 45711224	*ROBO2;CADPS2;NTRK2;CASZ1;PCDH7;PAX3;ESRRG;ANK1;DPP6;PAX7;KIF26B;SLIT2;SOX6;DDR2*
LCOR	14/299	0.0143745180 45711224	*DENND1B;KMT2C;PDE4D;ATP2B4;PRRC2C;ZBTB20;RORA;LYST;BACH2;MKLN1;STAG2;TRPS1;UTRN;DDR2*
LEUTX	14/299	0.0143745180 45711224	*DPP10;TENM2;DCC;PDE4D;DNAH5;PAX3;BACH2;DPP6;DAB1;KIF26B;FAT3;CTNNA3;DYNC1I1;DDR2*
MEF2C	14/299	0.0143745180 45711224	*CADPS2;PDE4D;PCDH7;RFTN1;ATP2B4;ZBTB20;ANK2;RORA;ANK1;HDAC9;DOCK10;TRPS1;SLIT2;DDR2*
MEIS2	14/299	0.0143745180 45711224	*ROBO2;CADPS2;NTRK2;AUTS2;PCDH7;ATP2B4;ZBTB20;ANK2;BACH2;PTPRD;TRPS1;CDH13;SLIT2;DDR2*
MLXIP	14/299	0.0143745180 45711224	*AUTS2;IQSEC1;KMT2C;DNMT3A;ATP2B4;ZBTB20;RORA;SSH2;ANK1;BACH2;ETV6;UTRN;LPIN1;DDR2*
NCOA1	14/299	0.0143745180 45711224	*AUTS2;KMT2C;TCF12;DNMT3A;ATP2B4;ZBTB20;PDE4DIP;ANK2;RORA;BACH2;NBEA;TRPS1;UTRN;DDR2*
PLXNC1	14/299	0.0143745180 45711224	*NTRK2;ANKRD44;RFTN1;ATP2B4;RORA;ANK1;LYST;HDAC9;DOCK10;RGS10;SLIT2;TOX;DOCK2;DDR2*
POU1F1	14/299	0.0143745180 45711224	*CADPS2;NRXN1;PCDH15;PAX3;HTR2C;RORA;MITF;GRIK2;CDH6;DLG2;ERBB4;SOX6;SOX5;DDR2*
POU3F1	14/299	0.0143745180 45711224	*NTRK2;RBFOX1;CASZ1;PCDH7;RORA;NRG1;ANK1;CDH4;DAB1;PPP2R2C;SLIT2;SOX6;SOX5;DDR2*
PRDM7	14/299	0.0143745180 45711224	*CADPS2;CNTNAP2;NTRK2;NEGR1;PCDH7;PDE4DIP;MITF;HDAC9;NAV3;NRG3;KIF26B;SOX6;ATP6V0D2;DDR2*
PRDM8	14/299	0.0143745180 45711224	*CADPS2;TENM2;AUTS2;NEGR1;PDE4D;PCDH7;RORA;LMO7;NRG1;ANK1;BACH2;SLIT2;TOX;DDR2*
PROX2	14/299	0.0143745180 45711224	*MAGI1;PCSK1;CADPS2;NRXN1;ZBTB20;RORA;LMO7;ANK1;BACH2;PTPRD;DLG2;KIF26B;SOX6;DDR2*
SMAD9	14/299	0.0143745180 45711224	*ROBO2;CADPS2;NTRK2;PARM1;PCDH7;ANK2;RORA;LMO7;PLCB4;PDE3A;SLIT2;UTRN;EDIL3;DDR2*
SOX11	14/299	0.0143745180 45711224	*ROBO2;CNTNAP2;NTRK2;AUTS2;PCDH7;BACH2;CDH6;PTPRD;CDH4;KIF26B;FAT3;SLIT2;SOX5;DDR2*
SOX14	14/299	0.0143745180 45711224	*RBFOX1;CASZ1;CELF4;PAX3;ESRRG;ADARB2;ANK1;NELL1;CDH4;DPP6;NRG3;PAX7;ERBB4;CSMD1*

SOX30	14/299	0.0143745180 45711224	*CADPS2;CASZ1;NRXN1;PAX3;RORA;ESRRG;NRG1; GRIK2;ANK1;DPP6;PAX7;DOCK2;CDH18;DDR2*
TOX2	14/299	0.0143745180 45711224	*CADPS2;AUTS2;ATP2B4;NRG1;ANK1;HDAC9;BACH 2;CDH4;DNER;FAM20C;CDH13;SLIT2;TOX;DDR2*
VSX2	14/299	0.0143745180 45711224	*NTRK2;RBFOX1;CASZ1;CELF4;PAX3;RORA;ESRRG ;ANK1;CDH4;NRG3;PAX7;ERBB4;KIF26B;SOX5*
ZBTB38	14/299	0.0143745180 45711224	*MAST4;KMT2C;PDE4D;ATP2B4;ZBTB20;PDE4DIP; RORA;LMO7;ANK1;LYST;TRPS1;UTRN;LPIN1;DDR 2*
ZC3H6	14/299	0.0143745180 45711224	*CADPS2;KMT2C;FAM13A;ZBTB20;PDE4DIP;RORA; LYST;BACH2;NBEA;DMD;TNIK;UTRN;LPIN1;DDR2*
ZFP37	14/299	0.0143745180 45711224	*CADPS2;PDE4D;ZBTB20;PDE4DIP;ANK2;RORA;PP P1R9A;PTPRD;PLCB4;NBEA;NAV3;TRPS1;SOX5;D DR2*
ZHX1	14/299	0.0143745180 45711224	*NCOA1;DENND1B;KMT2C;PDE4D;TCF12;ZBTB20;P DE4DIP;RORA;LYST;STAG2;TRPS1;ZNF638;TTC3 ;DDR2*
ZNF141	14/299	0.0143745180 45711224	*CADPS2;CNTNAP2;AUTS2;GLDC;PDE4D;DNMT3A;P DE4DIP;RORA;PPP1R9A;HDAC9;NBEA;TRPS1;ZNF 267;DDR2*
ZNF157	14/299	0.0143745180 45711224	*MAGI1;CADPS2;CNTNAP2;NRXN1;PCDH7;ADAM22; DACH2;DNER;DMD;CTNNA2;TOX;MCF2L2;ASTN1;D DR2*
ZNF182	14/299	0.0143745180 45711224	*CADPS2;KMT2C;ZBTB20;RORA;BACH2;STAG2;PLC B4;TRPS1;ZNF638;NEK1;DMD;TNIK;UTRN;DDR2*
ZNF438	14/299	0.0143745180 45711224	*CADPS2;PDE4D;ATP2B4;ZBTB20;PDE4DIP;RORA; SSH2;HDAC9;ARHGAP24;ETV6;LRMDA;TRPS1;LPI N1;DDR2*
ZNF518A	14/299	0.0143745180 45711224	*DENND1B;CADPS2;KMT2C;ATP2B4;ZBTB20;PDE4D IP;RORA;LYST;MKLN1;STAG2;TRPS1;ZNF638;NE K1;DDR2*
ZNF518B	14/299	0.0143745180 45711224	*CADPS2;PARM1;PDE4D;PCDH7;ZBTB20;PDE4DIP; HDAC9;BACH2;NBEA;TTC3;SLIT2;UTRN;LPIN1;D DR2*
ZNF521	14/299	0.0143745180 45711224	*ROBO2;CADPS2;NTRK2;TENM2;NEGR1;PCDH7;ZBT B20;PTPRD;TRPS1;CDH13;SLIT2;TOX;GPC6;DDR 2*
ZNF573	14/299	0.0143745180 45711224	*MAGI1;CADPS2;PATJ;AUTS2;MAST4;KMT2C;PDE4 D;FAM13A;ZBTB20;PDE4DIP;RORA;NBEA;TRPS1; DDR2*
ZNF595	14/299	0.0143745180 45711224	*CADPS2;IQSEC1;KMT2C;PDE4D;ATP2B4;PDE4DIP ;RORA;NBPF1;HDAC9;BACH2;TRPS1;TOX;LPIN1; DDR2*
ZNF84	14/299	0.0143745180 45711224	*MAGI1;KMT2C;PDE4D;TCF12;ZBTB20;PDE4DIP;R ORA;BACH2;MKLN1;STAG2;NBEA;ZNF638;TTC3;D DR2*
ZXDA	14/299	0.0143745180 45711224	*CADPS2;PDE4D;ZBTB20;RORA;PPP1R9A;HDAC9;B ACH2;PTPRD;NBEA;TRPS1;DMD;UTRN;SOX5;DDR2*
ALX1	13/299	0.0315429653 690396	*CADPS2;PCDH7;PAX3;ESRRG;CDH6;PAX7;ERBB4; KIF26B;SLIT2;SOX6;GPC6;EDIL3;DDR2*
ALX4	13/299	0.0315429653 690396	*NTRK2;CASZ1;PAX3;RORA;ESRRG;ANK1;CDH4;PA X7;KIF26B;TRPS1;SOX6;CSMD1;DDR2*
ARID2	13/299	0.0315429653 690396	*NCOA1;KMT2C;TCF12;DNMT3A;PRRC2C;ZBTB20;R ORA;ETV6;MKLN1;STAG2;TRPS1;UTRN;DDR2*
BSX	13/299	0.0315429653 690396	*RBFOX1;CASZ1;CELF4;PAX3;RORA;ESRRG;CDH4; DPP6;PAX7;DSCAML1;ASTN1;RNF220;DDR2*
DMRT1	13/299	0.0315429653 690396	*RYR2;CADPS2;NTRK2;RBFOX1;GLDC;PCDH7;PAX3 ;ANK1;DPP6;PAX7;KIF26B;CSMD1;DDR2*
EMX1	13/299	0.0315429653 690396	*CADPS2;CNTNAP2;CASZ1;PCDH7;PAX3;RORA;ESR RG;NRG1;ANK1;CDH4;PAX7;TOX;DDR2*
ERG	13/299	0.0315429653	*NTRK2;PDE4D;ITGA1;PTPRM;ZBTB20;RORA;HDAC*

		690396	*9;ETV6;CDH13;DMD;SLIT2;UTRN;DDR2*
ESX1	13/299	0.0315429653 690396	*PAX3;HTR2C;ESRRG;ANK1;PPP2R2C;PAX7;ERBB4 ;KIF26B;GPC3;CDH13;SLIT2;SOX6;FGF13*
FOXD4L1	13/299	0.0315429653 690396	*CADPS2;CNTNAP2;CASZ1;NRXN3;PAX3;NRG1;ANK 1;ARHGAP24;PAX7;KIF26B;ZNF385B;TOX;DDR2*
GTF2IRD 2	13/299	0.0315429653 690396	*CADPS2;MAST4;KMT2C;PDE4D;PTPRM;ZBTB20;PD E4DIP;ANK2;PPP1R9A;ANK1;BACH2;UTRN;DDR2*
HIVEP2	13/299	0.0315429653 690396	*PDE4D;ATP2B4;ZBTB20;PDE4DIP;RORA;LMO7;AN K1;HDAC9;BACH2;ETV6;TRPS1;UTRN;DDR2*
HLF	13/299	0.0315429653 690396	*CADPS2;NTRK2;PARM1;PCDH7;ATP2B4;ZBTB20;R ORA;ANK1;PTPRD;PLCB4;SLIT2;SOX5;DDR2*
IKZF4	13/299	0.0315429653 690396	*CADPS2;NTRK2;AUTS2;DNMT3A;ATP2B4;ZBTB20; RORA;PPP1R9A;BACH2;KIF26B;TRPS1;TOX;DDR2*
IRX1	13/299	0.0315429653 690396	*CADPS2;NTRK2;PCDH7;ESRRG;NRG1;ANK1;CDH4; DPP6;KIF26B;CDH13;SLIT2;GPC6;DDR2*
IRX5	13/299	0.0315429653 690396	*CADPS2;NTRK2;CASZ1;AUTS2;PCDH7;RORA;NRG1 ;ANK1;KIF26B;TRPS1;CDH13;SLIT2;DDR2*
KLF14	13/299	0.0315429653 690396	*CADPS2;NTRK2;DGKB;PAX3;ESRRG;NRG1;ANK1;H DAC9;CDH4;DPP6;PAX7;KIF26B;SLIT2*
LHX1	13/299	0.0315429653 690396	*CADPS2;CNTNAP2;CASZ1;PCDH7;CELF4;PAX3;ES RRG;ANK1;CDH6;PAX7;KIF26B;SLIT2;DDR2*
MAEL	13/299	0.0315429653 690396	*RYR2;CADPS2;CNTNAP2;ESRRG;ANK1;RIMS1;ERB B4;KIF26B;DNER;BRINP3;CDH18;DDR2;RGS7*
MEF2A	13/299	0.0315429653 690396	*KMT2C;PDE4D;TCF12;ATP2B4;ZBTB20;PDE4DIP; RORA;MITF;LMO7;HDAC9;TRPS1;UTRN;DDR2*
MGA	13/299	0.0315429653 690396	*DYNC1H1;KMT2C;TCF12;PRRC2C;ZBTB20;LYST;M KLN1;STAG2;TRPS1;ZNF638;TTC3;UTRN;DDR2*
NCOA2	13/299	0.0315429653 690396	*NCOA1;DYNC1H1;ANKRD44;KMT2C;TCF12;PRRC2C ;ZBTB20;PDE4DIP;RORA;LYST;TRPS1;UTRN;DDR 2*
NCOR2	13/299	0.0315429653 690396	*DYNC1H1;KDM4B;AUTS2;IQSEC1;KMT2C;DNMT3A; PRRC2C;ZBTB20;PDE4DIP;ANK1;ETV6;UTRN;DDR 2*
NEUROD2	13/299	0.0315429653 690396	*RAP1GAP2;CADPS2;NTRK2;RBFOX1;NRXN1;CELF4 ;RORA;ANK1;BACH2;CDH4;DPP6;DAB1;DDR2*
NFATC2	13/299	0.0315429653 690396	*NTRK2;AUTS2;PDE4D;ATP2B4;ZBTB20;RORA;ANK 1;HDAC9;BACH2;DOCK10;TNIK;SOX5;DDR2*
NOTO	13/299	0.0315429653 690396	*RYR2;CADPS2;NTRK2;CASZ1;CELF4;PAX3;RORA; ESRRG;ANK1;NBEA;PAX7;KIF26B;SLIT2*
NR6A1	13/299	0.0315429653 690396	*TRIM71;AUTS2;GLDC;PDE4D;DNMT3A;ZBTB20;RO RA;MITF;ESRRG;BACH2;SOX6;SOX5;DDR2*
PDX1	13/299	0.0315429653 690396	*PCSK1;CADPS2;NTRK2;PCDH7;CADPS;PAX3;ESRR G;ANK1;NBEA;PAX7;PDE3A;SOX6;SOX5*
POGZ	13/299	0.0315429653 690396	*DYNC1H1;KDM4B;DSCAM;KMT2C;DNMT3A;PRRC2C; ZBTB20;PDE4DIP;ANK2;STAG2;ZNF638;SCN2A;D DR2*
POU4F2	13/299	0.0315429653 690396	*CADPS2;RBFOX1;DCC;CELF4;NRXN3;PAX3;ESRRG ;NELL1;CDH4;DPP6;PAX7;DNER;SLIT2*
POU4F3	13/299	0.0315429653 690396	*PCSK1;CNTNAP2;RBFOX1;CASZ1;NRXN1;CELF4;P AX3;ESRRG;GRIK2;ANK1;CDH4;DPP6;PAX7*
RFX7	13/299	0.0315429653 690396	*KDM4B;DSCAM;KMT2C;NRXN1;TCF12;DNMT3A;PRR C2C;ZBTB20;ANK2;TRPS1;SCN2A;UTRN;DDR2*
RHOXF2B	13/299	0.0315429653 690396	*PCSK1;CADPS2;FAM9B;ATRNL1;NRXN3;KIAA1549 L;ESRRG;CDH6;STAG2;FGF14;CDH12;ZNF385B;B RINP3*
SCML4	13/299	0.0315429653 690396	*CADPS2;ANKRD44;PDE4D;NRXN1;ZBTB20;RORA;A NK1;BACH2;DOCK10;TNIK;UTRN;TOX;DDR2*
SCRT1	13/299	0.0315429653 690396	*CADPS2;NTRK2;RBFOX1;CASZ1;NRXN1;CADPS;CE LF4;ESRRG;ANK1;DPP6;PPP2R2C;DNER;RIMBP2*
T	13/299	0.0315429653	*CADPS2;RBFOX1;PCDH7;PAX3;RORA;ESRRG;ANK1*

		690396	*;CDH4;DAB1;PAX7;KIF26B;CDH13;DDR2*
TGIF2LY	13/299	0.0315429653 690396	*DPP10;CADPS2;PCDH7;HTR2C;PXDNL;ESRRG;FSTL5;LRFN5;NRG3;TRPS1;CDH12;CDH18;DDR2*
TP63	13/299	0.0315429653 690396	*NTRK2;TENM2;PDE4D;PCDH7;ZBTB20;RORA;MITF;NRG1;HDAC9;BACH2;ERBB4;DMD;DDR2*
VAX1	13/299	0.0315429653 690396	*CADPS2;RBFOX1;CASZ1;CELF4;PAX3;ESRRG;ANK1;DPP6;PAX7;KIF26B;SLIT2;SOX6;SOX5*
ZBTB37	13/299	0.0315429653 690396	*DENND1B;CADPS2;ANKRD44;KMT2C;ATP2B4;PRRC2C;ZBTB20;RORA;LYST;BACH2;TRPS1;UTRN;DDR2*
ZFHX2	13/299	0.0315429653 690396	*RAP1GAP2;CADPS2;CASZ1;AUTS2;DNMT3A;ATP2B4;ZBTB20;ANK2;ANK1;BACH2;KIF26B;TRPS1;SOX5*
ZFP14	13/299	0.0315429653 690396	*CADPS2;CNTNAP2;ZBTB20;PDE4DIP;RORA;PPP1R9A;BACH2;PLCB4;NBEA;TRPS1;UTRN;SOX5;DDR2*
ZFP2	13/299	0.0315429653 690396	*CADPS2;NTRK2;AUTS2;PDE4D;ZBTB20;RORA;PPP1R9A;ANK1;BACH2;NBEA;DMD;SOX6;DDR2*
ZFP92	13/299	0.0315429653 690396	*ROBO2;CADPS2;NTRK2;ZBTB20;RORA;ANK1;PTPRD;DAB1;MYO5B;SLIT2;RGS9;SOX5;DDR2*
ZIC3	13/299	0.0315429653 690396	*TRIM71;CADPS2;CNTNAP2;GLDC;NRXN1;PCDH7;CADPS;HTR2C;ESRRG;ANK1;GPC3;SLIT2;FGF13*
ZNF148	13/299	0.0315429653 690396	*NCOA1;KMT2C;TCF12;PRRC2C;ZBTB20;RORA;MKLN1;STAG2;TRPS1;ZNF638;TTC3;UTRN;DDR2*
ZNF19	13/299	0.0315429653 690396	*FBLN7;CADPS2;PATJ;AUTS2;KMT2C;PDE4D;PLCL1;PDE4DIP;RORA;LMO7;BACH2;SOX6;DDR2*
ZNF248	13/299	0.0315429653 690396	*DENND1B;CADPS2;AUTS2;PDE4D;TCF12;ATP2B4;PDE4DIP;RORA;LYST;NBEA;TRPS1;TTC3;DDR2*
ZNF37A	13/299	0.0315429653 690396	*DENND1B;CADPS2;PDE4D;PRRC2C;RORA;TMEM64;PPP1R9A;LYST;HDAC9;MKLN1;TRPS1;TTC3;DDR2*
ZNF493	13/299	0.0315429653 690396	*MAGI1;CADPS2;KMT2C;ATP2B4;ZBTB20;PDE4DIP;RORA;PPP1R9A;LYST;NBEA;TRPS1;ZNF638;DDR2*
ZNF559	13/299	0.0315429653 690396	*KDM4B;DSCAM;AUTS2;KMT2C;NRXN1;DNMT3A;PTPRM;ZBTB20;ANK2;RORA;RIMS1;SCN2A;DDR2*
ZNF560	13/299	0.0315429653 690396	*CHODL;PCSK1;RYR2;CADPS2;CNTNAP2;GLDC;NRXN1;PCDH7;DAB1;FGF13;MDGA2;CSMD1;FREM2*
ZNF610	13/299	0.0315429653 690396	*CADPS2;AUTS2;NEGR1;PDE4D;PCDH7;ZBTB20;RORA;BACH2;PLCB4;NBEA;SLIT2;TOX;DDR2*
ZNF621	13/299	0.0315429653 690396	*KMT2C;TCF12;ATP2B4;PRRC2C;ZBTB20;PDE4DIP;RORA;LYST;MKLN1;STAG2;TRPS1;UTRN;DDR2*
ZNF652	13/299	0.0315429653 690396	*NCOA1;DYNC1H1;KMT2C;ATP2B4;PRRC2C;ZBTB20;RORA;MKLN1;STAG2;TRPS1;TTC3;UTRN;DDR2*
ZNF662	13/299	0.0315429653 690396	*CADPS2;NTRK2;AUTS2;NEGR1;PDE4D;SMAD9;RORA;BACH2;PLCB4;DMD;CNTN4;SOX6;DDR2*
ZNF709	13/299	0.0315429653 690396	*DENND1B;CADPS2;KDM4B;AUTS2;PDE4D;DNMT3A;ZBTB20;RORA;LMO7;BACH2;PTPRG;UTRN;DDR2*

Table S11. The 398 genes that maintained stable contact with rDNA clusters are highly associated with the H3K27me3 mark in normal and cultivated human cells. The search was performed in https://maayanlab.cloud/Enrichr/enrich# ENCODE Histone Modifications 2015. Related to Figures 4 and 5.

Term	Overlap	Adjusted P-value	Genes
H3K27me3 bronchial epithelial	78/2082	1.0782932224832745E-5	*TRIM71;ROBO2;PCSK1;RYR2;GALNT13;MAST4;GLDC;CELF4;HTR2C;RORA;GRIK2;ADARB2;DACH2;PPP1R9A;DOCK10;GRM3;RIMS1;*

cell hg19			*GRM7;DNER;ZNF385B;EDIL3;MCF2L2;RGS9;PCDHAC1;SOX5;PPFIA2;RGS7;DCC;PAX3;KAZN;NRG1;ANO5;NBPF1;NRG3;DOCK2;DSCAML1;FREM2;ASTN1;PARM1;NRXN1;ADAM22;ADCY2;HDAC9;NOL4;ERBB4;GPC3;GPC5;SLIT2;CSMD1;GPC6;WNT2;LINC00273;CNTN5;AUTS2;NEGR1;CADM2;ATRNL1;IQSEC1;KCNB2;PLCL1;ZNF804B;CADPS;SMAD9;PDE4DIP;ESRRG;PTPRD;SH3RF3;NELL1;LRFN5;RALYL;DAB1;FGF14;NBEA;PPP2R2C;PDE3A;GALNTL6;CNTN4;MDGA2*
H3K27me3 megakaryocyte mm9	91/2638	1.4400229514202476E-5	*DPP10;ROBO2;CNTNAP2;PCSK1;RYR2;GALNT13;GLDC;DGKB;FAM13A;HTR2C;PTPRM;RORA;GRIK2;ADARB2;DACH2;PTPRG;CDH6;DOCK10;GRM3;RIMS1;SGCD;GRM7;TRPS1;EDIL3;PCDHAC2;PCDHAC1;SOX5;PPFIA2;RGS7;MAGI1;CADPS2;RBFOX1;CERS6;SGIP1;NRG1;ANK2;LMO7;PCDHA12;ANK1;ANO5;PCDHA10;EDAR;ACE2;NRG3;PAX7;B3GNT5;DSCAML1;CDH18;ASTN1;CHODL;FBLN7;ADAM22;ADCY2;FHIT;DPP6;GPC3;GPC5;PCDHA2;CTNNA2;CSMD1;MPDZ;TRPM6;GPC6;WNT2;TRPM3;DYNC1I1;NTRK2;AUTS2;NEGR1;ATRNL1;PLCL1;SH2D1A;CADPS;PDE4DIP;ARHGAP24;ENOX1;PTPRD;SH3RF3;NELL1;LRFN5;RALYL;DAB1;FGF14;DLG2;NBEA;MYO5B;TMEM117;PIR;GALNTL6;CNTN4;MDGA2*
H3K27me3 SK-N-SH hg19	74/2149	1.6125907331176128E-4	*TRIM71;ROBO2;DENND1B;CNTNAP2;PCSK1;RYR2;MAST4;FRMPD4;HTR2C;ADARB2;DACH2;PPP1R9A;PTPRG;CDH6;GRM3;RIMS1;CDH4;RASGEF1B;DNER;ZNF385B;EDIL3;PPFIA2;RGS7;CADPS2;RBFOX1;DSCAM;DCC;PAX3;KAZN;NRG3;CDH12;FAM20C;CDH13;DSCAML1;FREM2;CDH18;ASTN1;PARM1;ADCY2;HDAC9;DPP6;ERBB4;ST8SIA6;GPC5;SLIT2;CSMD1;TRPM6;WNT2;TRPM3;DYNC1I1;LINC00273;NTRK2;CNTN5;CADM2;IQSEC1;KCNB2;PCDH7;SH2D1A;ZNF804B;CADPS;PDE4DIP;ESRRG;PTPRD;SH3RF3;NELL1;LRFN5;RALYL;PPP2R2C;MYO5B;GALNTL6;CNTN4;MDGA2;FGF13;RNF220*
H3K27me3 MCF-7 hg19	42/989	1.912798430448817E-4	*ROBO2;PCSK1;PARM1;MAST4;NRXN3;ADAMTS9-AS2;ADARB2;DACH2;PPP1R9A;HDAC9;NOL4;GRM3;RIMS1;ERBB4;ZNF385B;GPC5;EDIL3;RGS9;SOX5;PPFIA2;LINC00273;NTRK2;CNTN5;AUTS2;NEGR1;CADM2;KCNB2;PLCL1;ITGA1;ZNF804B;SMAD9;PDE4DIP;EDAR;PTPRD;SH3RF3;LRFN5;RALYL;KIF26B;CDH13;TNIK;CNTN4;BMPR1B*
H3K27me3 mammary epithelial cell hg19	83/2586	2.9518479290722104E-4	*TRIM71;ROBO2;CNTNAP2;PCSK1;RYR2;GALNT13;FRMPD4;GLDC;CELF4;HTR2C;ADARB2;DACH2;PPP1R9A;CDH6;DOCK10;GRM3;EPB41L4B;GRM7;RASGEF1B;DNER;ZNF385B;MCF2L2;DLEU7;SOX5;PPFIA2;RGS7;CADPS2;RBFOX1;DCC;PAX3;KAZN;ANO5;NBPF1;NRG3;PAX7;CDH12;TOX;FREM2;CDH18;FBLN7;PARM1;DNAH8;NRXN1;ADAM22;NRXN3;ADCY2;HDAC9;NOL4;DPP6;ERBB4;ST8SIA6;GPC5;CTNNA3;SLIT2;CSMD1;WNT2;LINC00273;RAP1GAP2;NTRK2;CNTN5;AUTS2;CADM2;ATRNL1;IQSEC1;KCNB2;ZNF804B;CADPS;PDE4DIP;ESRRG;PTPRD;AGBL4;NELL1;LRFN5;RALYL;FGF14;NBEA;PPP2R2C;PDE3A;GAL*

			NTL6;CNTN4;MDGA2;FGF13;RNF220
H3K27me3 keratinocyte hg19	71/2221	0.0013929963611168223	*TRIM71;CNTNAP2;PCSK1;RYR2;GALNT13;FRMPD4;GLDC;ADARB2;DACH2;PPP1R9A;DOCK10;GRM3;RIMS1;DNER;ZNF385B;EDIL3;DLEU7;PCDHAC2;PCDHAC1;SOX5;PPFIA2;RGS7;CADPS2;RBFOX1;DCC;PAX3;KAZN;NRG1;ANO5;PLCB4;NRG3;CDH12;TOX;DOCK2;FREM2;ASTN1;PARM1;NRXN1;NRXN3;ADCY2;HDAC9;NOL4;FSTL5;DPP6;ERBB4;GPC3;GPC6;WNT2;DYNC1I1;LINC00273;CNTN5;AUTS2;CNTN6;KCNB2;PLCL1;ZNF804B;CADPS;SMAD9;NELL1;LRFN5;RALYL;FGF14;NBEA;MYO5B;KIF26B;PDE3A;GALNTL6;CNTN4;MDGA2;FGF13;BMPR1B*
H3K27me3 CD14-positive monocyte hg19	100/3445	0.0013929963611168223	*GALNT13;FRMPD4;GLDC;RORA;ADAMTS9-AS2;ADARB2;BACH2;CDH6;RIMS1;CDH4;MACROD2;DLEU7;PCDHAC2;PCDHAC1;PPFIA2;RGS7;MAGI1;CADPS2;RBFOX1;KAZN;ANK2;PCDHA12;MIR100HG;ANK1;PCDHA11;PCDHA10;EDAR;FREM2;ASTN1;FBLN7;CASZ1;DPP6;PCDHA5;PCDHA3;ST8SIA6;PCDHA2;PCDHA9;TRPM6;WNT2;PCDHA7;TRPM3;PCDHA6;CEP112;CNTN5;ARHGEF37;CADM2;IQSEC1;PTPRD;LRFN5;DAB1;GALNTL6;CNTN4;FGF13;BMPR1B;TRIM71;ROBO2;PCSK1;MAST4;CELF4;HTR2C;GRIK2;PPP1R9A;PHEX;GRM3;PGM3;ZNF385B;MCF2L2;DIRC3;SGIP1;DNMT3A;PAX3;NBPF1;PLCB4;NRG3;PAX7;CDH12;CDH13;DSCAML1;CDH18;NRXN1;ADAM22;HDAC9;NOL4;FSTL5;GPC5;CTNNA3;CTNNA2;SLIT2;CSMD1;GPC6;NTRK2;KCNB2;PCDH7;ZNF804B;CADPS;ENOX1;AGBL4;NELL1;KIF26B;MDGA2*
H3K27me3 GM12878 hg19	91/3221	0.0078122371573310905	*TRIM71;ROBO2;DENND1B;CNTNAP2;RYR2;MAST4;ULK4;PTPRM;ADAMTS9-AS2;GRIK2;PPP1R9A;BACH2;CDH6;GRM3;RIMS1;SGCD;GRM7;RASGEF1B;TRPS1;DNER;ZNF385B;MACROD2;EDIL3;MCF2L2;DLEU7;SOX5;PPFIA2;RGS7;MAGI1;CADPS2;DCC;ITGA1;PAX3;MITF;NRG1;NBPF1;PLCB4;NRG3;B3GNT5;CDH12;CDH13;TOX;FREM2;ASTN1;FBLN7;PARM1;WDR64;HDAC9;NOL4;FSTL5;SCEL;ERBB4;ST8SIA6;GPC5;CTNNA3;SLIT2;WNT2;DYNC1I1;LINC00273;RAP1GAP2;CEP112;CNTN5;ARHGEF37;NEGR1;CADM2;IQSEC1;KCNB2;PCDH7;PLCL1;ZNF804B;CADPS;SMAD9;PDE4DIP;ESRRG;ENOX1;SLC8A3;PTPRD;SH3RF3;AGBL4;NELL1;LRFN5;RALYL;DAB1;FGF14;NBEA;KIF26B;TMEM117;PDE3A;GALNTL6;CNTN4;BMPR1B*
H3K27me3 Caco-2 hg19	18/380	0.01918853845097927	*ROBO2;LINC00273;CADPS2;PARM1;MAST4;ANKRD44;PLCL1;PDE4DIP;HDAC9;MKLN1;NBEA;ERBB4;KIF26B;PDE3A;ZNF385B;TOX;WNT2;DYNC1I1*
H3K27me3 kidney epithelial cell hg19	65/2217	0.0228917577611 02742	*TRIM71;ROBO2;PCSK1;RYR2;GALNT13;FRMPD4;HTR2C;RORA;GRIK2;ADARB2;GRM3;RIMS1;GRM7;DNER;ZNF385B;MCF2L2;RGS9;DLEU7;PCDHAC2;PCDHAC1;PPFIA2;DSCAM;PAX3;KAZN;NRG1;ANK1;NBPF1;PAX7;CDH12;FREM2;CDH18;ASTN1;FBLN7;DNAH8;NRXN1;ADCY2;HDAC9;NOL4;DPP6;ERBB4;GPC3;ST8SIA6;GPC5;SLIT2;WNT2;TRPM3;IQSEC1;PLCL1;ZNF804B;CADPS;ESRRG;SLC8A3;PTPRD;SH3RF3;AGBL4;NELL1;RALYL;DAB1*

			;FGF14;PPP2R2C;PDE3A;GALNTL6;CNTN4;MDGA2;RNF220
H3K9me3 CD14-positive monocyte hg19	59/2000	0.03301576454214255	*TRIM71;PCSK1;RYR2;GALNT13;FRMPD4;GLDC;RORA;ADAMTS9-AS2;GRIK2;DACH2;PTPRG;CDH6;GRM3;ZNF385B;MACROD2;EDIL3;MCF2L2;PPFIA2;MAGI1;CADPS2;DSCAM;DCC;KAZN;NRG1;ANK1;CDH13;DSCAML1;FREM2;FBLN7;PARM1;NRXN1;HDAC9;NOL4;GPC3;SLIT2;WNT2;NTRK2;CADM2;ATRNL1;IQSEC1;KCNB2;PCDH7;ZNF804B;CADPS;PDE4DIP;ESRRG;ENOX1;SLC8A3;PTPRD;SH3RF3;NELL1;LRFN5;RALYL;DAB1;FGF14;PDE3A;GALNTL6;BMPR1B;RNF220*
H3K27me3 BJ hg19	61/2090	0.03321799994196486	*TRIM71;ROBO2;PCSK1;RYR2;MAST4;GLDC;CELF4;RORA;DACH2;PPP1R9A;BACH2;CDH6;RIMS1;CDH4;RASGEF1B;DNER;ZNF385B;EDIL3;MCF2L2;PCDHAC1;SOX5;RGS7;CADPS2;NRG1;ANK1;NRG3;B3GNT5;FREM2;CDH18;ASTN1;PARM1;DNAH8;ADAM22;ADCY2;HDAC9;NOL4;ERBB4;GPC5;WNT2;DYNC1I1;LINC00273;NTRK2;ATRNL1;IQSEC1;KCNB2;PCDH7;PLCL1;ZNF804B;CADPS;ESRRG;SLC8A3;PTPRD;NELL1;LRFN5;RALYL;FGF14;PPP2R2C;MYO5B;GALNTL6;FGF13;BMPR1B*
H3K27me3 cardiac mesoderm hg19	92/3485	0.04527826759268389	*ROBO2;PCSK1;MAST4;FRMPD4;GLDC;HTR2C;GRIK2;ADARB2;CDH6;DOCK10;GRM3;CDH4;DNER;ZNF385B;MCF2L2;RGS9;DLEU7;PCDHAC2;PCDHAC1;PPFIA2;RGS7;CADPS2;RBFOX1;DSCAM;SGIP1;DCC;ANKRD44;PAX3;NRG1;ANK1;EDAR;NRG3;PAX7;B3GNT5;CDH12;CDH13;TNIK;DOCK2;DSCAML1;CDH18;ASTN1;FBLN7;CASZ1;PARM1;DNAH8;NRXN1;HDAC9;NOL4;DPP6;ERBB4;GPC3;GPC5;SLIT2;CSMD1;TRPM6;WNT2;TRPM3;DYNC1I1;NTRK2;CNTN5;ARHGEF37;CADM2;IQSEC1;KCNB2;PCDH7;PLCL1;RFTN1;ZNF804B;CADPS;PDE4DIP;ESRRG;ENOX1;SLC8A3;PTPRD;SH3RF3;MKLN1;NELL1;LRFN5;RALYL;FGF14;PPP2R2C;MYO5B;KIF26B;TMEM117;PDE3A;RGS10;GALNTL6;CNTN4;MDGA2;FGF13;BMPR1B;RNF220*

Table S12. The 398 genes with stable contacts with rDNA clusters are simultaneously regulated in different combinations by 391 different transcription factors. The search was performed in TF-Gene Co-occurrence (Enrichr Submissions TF-Gene Co-occurrence, https://maayanlab.cloud/Enrichr/). Related to Figure 5B.

Term	Overlap	Adjusted P-value	Genes
POU6F2	79/299	2.9215419541599807E-63	*ROBO2;DPP10;PCSK1;RYR2;CNTNAP2;TENM2;DGKB;CELF4;PTPRM;ZBTB20;RORA;GRIK2;ADARB2;BACH2;RIMS1;CDH4;GRM7;DNER;MACROD2;SOX6;SOX5;PPFIA2;MAGI1;CADPS2;RBFOX1;DSCAM;DCC;PDE4D;NRG1;ANK1;NAV3;NRG3;CDH12;CDH13;TOX;CDH18;NRXN1;PCDH15;NRXN3;ADCY2;NOL4;HDAC9;FSTL5;DPP6;ERBB4;DMD;CTNNA3;GPC5;CTNNA2;SLIT2;CSMD1;GPC6;TRPM3;DYNC1I1;RAP1GAP2;NTRK2;CNTN5;AUTS2*

			;NEGR1;CADM2;ATRNL1;KCNB2;PCDH7;CADPS;ESRRG;PTPRD;MGAT4C;AGBL4;NELL1;RALYL;DLG2;FGF14;DAB1;NBEA;KIF26B;RIMBP2;FAT3;CNTN4;MDGA2
ZNF804B	76/299	1.4002958224459074E-59	*ROBO2;DPP10;RYR2;CNTNAP2;TENM2;DGKB;PTPRM;RORA;GRIK2;PTPRG;GRM3;RIMS1;CDH4;GRM7;ZNF385B;MACROD2;EDIL3;SOX5;PPFIA2;RGS7;CADPS2;RBFOX1;DSCAM;DCC;PDE4D;ANK2;NRG1;NAV3;NRG3;CDH12;CDH13;FREM2;CDH18;NRXN1;PCDH15;NRXN3;NOL4;FHIT;HDAC9;FSTL5;DPP6;ERBB4;DMD;CTNNA3;GPC5;CTNNA2;SLIT2;CSMD1;GPC6;BRINP3;TRPM3;CNTN5;AUTS2;NEGR1;CADM2;ATRNL1;CNTN6;KCNB2;PCDH7;CADPS;ESRRG;PTPRD;MGAT4C;AGBL4;NELL1;LRFN5;RALYL;DLG2;FGF14;DAB1;NBEA;KIF26B;FAT3;GALNTL6;CNTN4;MDGA2*
SLC4A10	63/299	1.8453745364388836E-43	*ROBO2;DPP10;RYR2;CNTNAP2;FRMPD4;DGKB;CELF4;RORA;GRIK2;DOCK10;RIMS1;GRM7;DNER;EDIL3;SOX5;PPFIA2;RGS7;CADPS2;RBFOX1;DSCAM;SGIP1;DCC;PDE4D;ANK2;ANK1;PLCB4;NAV3;CDH18;ASTN1;DDR2;NRXN1;ADAM22;NRXN3;ADCY2;NOL4;HDAC9;FSTL5;DPP6;ERBB4;DMD;CTNNA2;CSMD1;BRINP3;TRPM3;DYNC1I1;RAP1GAP2;NTRK2;CNTN5;NEGR1;CADM2;ATRNL1;PCDH7;CADPS;ESRRG;PTPRD;NELL1;RALYL;DLG2;FGF14;NBEA;RIMBP2;CNTN4;SCN2A*
DACH2	60/299	3.734332862842493E-40	*ROBO2;DPP10;PCSK1;RYR2;CNTNAP2;TENM2;GALNT13;FRMPD4;DGKB;CELF4;HTR2C;GRIK2;DNER;SOX6;EDIL3;PPFIA2;RGS7;CADPS2;RBFOX1;DCC;ANK2;ANK1;NAV3;CDH12;TOX;CDH18;DDR2;NRXN1;PCDH15;NRXN3;NOL4;HDAC9;FSTL5;DPP6;ERBB4;GPC3;DMD;CTNNA3;CTNNA2;SLIT2;TRPM3;DYNC1I1;NTRK2;CNTN5;NEGR1;CADM2;CADPS;ESRRG;PTPRD;MGAT4C;NELL1;RALYL;DLG2;FGF14;NBEA;FAT3;CNTN4;SCN2A;FGF13;BMPR1B*
ZNF385D	60/299	3.734332862842493E-40	*ROBO2;RYR2;CNTNAP2;TENM2;DGKB;PTPRM;ZBTB20;RORA;GRIK2;RIMS1;SGCD;TRPS1;DNER;ZNF385B;SOX6;EDIL3;SOX5;MAGI1;CADPS2;RBFOX1;SGIP1;DCC;PDE4D;ANK2;NRG1;PLCB4;NAV3;CDH12;CDH13;TOX;CDH18;ASTN1;DDR2;NRXN1;NRXN3;HDAC9;DPP6;CTNNA3;CTNNA2;SLIT2;CSMD1;GPC6;TRPM3;NTRK2;CNTN5;NEGR1;CADM2;ATRNL1;PCDH7;CADPS;ESRRG;ENOX1;PTPRD;NELL1;LRFN5;DLG2;FGF14;NBEA;PDE3A;CNTN4*
RGS6	59/299	3.81650034949096E-39	*ROBO2;DPP10;RYR2;CNTNAP2;TENM2;PTPRM;ZBTB20;RORA;GRIK2;ADARB2;CDH6;RIMS1;CDH4;GRM7;SOX6;EDIL3;SOX5;RGS7;MAGI1;CADPS2;RBFOX1;DCC;PDE4D;ANK2;NRG1;ANK1;PLCB4;CDH13;DDR2;NRXN1;NRXN3;ADCY2;HDAC9;DPP6;ERBB4;DMD;CTNNA3;SLIT2;CSMD1;GPC6;TRPM3;RAP1GAP2;NTRK2;AUTS2;NEGR1;CADM2;ATRNL1;PCDH7;CADPS;ESRRG;PTPRD;DLG2;FGF14;DAB1;NBEA;KIF26B;PDE3A;FAT3;CNTN4*
RGS7	59/299	3.81650034949096E-39	*ROBO2;DPP10;PCSK1;RYR2;CNTNAP2;TENM2;CELF4;RORA;GRIK2;RIMS1;CDH4;GRM7;DNER;SOX5;PPFIA2;CADPS2;RBFOX1;ANK2;NRG1;ANK1;PLCB4;NAV3;CDH13;CDH18;DDR2;NRXN1;NR*

			XN3;ADCY2;NOL4;HDAC9;DPP6;ERBB4;CTNNA2;SLIT2;CSMD1;DYNC1I1;RAP1GAP2;NTRK2;NEGR1;CADM2;ATRNL1;KCNB2;PCDH7;CADPS;ESRRG;PTPRD;NELL1;RALYL;DLG2;FGF14;DAB1;NBEA;PPP2R2C;KIF26B;RIMBP2;FAT3;CNTN4;SCN2A;FGF13
ESRRG	58/299	4.1468524523700324E-38	*ROBO2;DPP10;RYR2;CNTNAP2;TENM2;CELF4;PTPRM;ZBTB20;RORA;GRIK2;BACH2;CDH6;CDH4;TRPS1;DNER;SOX6;SOX5;MAGI1;CADPS2;RBFOX1;PDE4D;ANK2;LMO7;NRG1;ANK1;PLCB4;CDH13;TOX;DDR2;CASZ1;NRXN1;NRXN3;HDAC9;DPP6;ERBB4;DMD;CTNNA3;CTNNA2;SLIT2;CSMD1;GPC6;TRPM3;RAP1GAP2;NTRK2;AUTS2;NEGR1;CADM2;ATRNL1;PCDH7;CADPS;PTPRD;DLG2;DAB1;NBEA;KIF26B;FAT3;CNTN4;BMPR1B*
MYT1L	58/299	4.1468524523700324E-38	*ROBO2;DPP10;RYR2;CNTNAP2;TENM2;DGKB;CELF4;PTPRM;ZBTB20;RORA;GRIK2;ADARB2;RIMS1;CDH4;GRM7;SOX5;PPFIA2;RGS7;CADPS2;RBFOX1;DSCAM;SGIP1;DCC;PDE4D;DNMT3A;ANK2;ANK1;NAV3;CDH13;ASTN1;KMT2C;NRXN1;NRXN3;NOL4;DPP6;CTNNA2;CSMD1;TRPM3;RAP1GAP2;NTRK2;CNTN5;AUTS2;NEGR1;CADM2;ATRNL1;CADPS;ESRRG;PTPRD;RALYL;DLG2;FGF14;DAB1;NBEA;PPP2R2C;KIF26B;RIMBP2;FAT3;SCN2A*
NPAS3	57/299	5.089373344251292E-37	*ROBO2;CNTNAP2;TENM2;DGKB;PTPRM;ZBTB20;RORA;GRIK2;ADARB2;BACH2;PTPRG;CDH6;CDH4;TRPS1;MACROD2;SOX6;SOX5;MAGI1;CADPS2;RBFOX1;DCC;PDE4D;ANK2;NRG1;ANK1;NAV3;NRG3;CDH13;TNIK;TOX;DDR2;NRXN1;PCDH15;NRXN3;HDAC9;DPP6;ERBB4;CTNNA2;SLIT2;CSMD1;GPC6;TRPM3;NTRK2;AUTS2;NEGR1;CADM2;PCDH7;CADPS;ESRRG;PTPRD;DLG2;FGF14;DAB1;NBEA;KIF26B;FAT3;BMPR1B*
ZIM2	53/299	1.2495234769937264E-32	*ROBO2;DPP10;RYR2;CNTNAP2;TENM2;GALNT13;ZBTB20;RORA;GRIK2;ADARB2;RIMS1;SOX6;SOX5;PPFIA2;CADPS2;RBFOX1;DSCAM;DCC;PDE4D;ANK2;NRG1;ANK1;NAV3;CDH12;CDH13;CDH18;ASTN1;DDR2;NRXN1;PCDH15;NRXN3;FSTL5;DPP6;ERBB4;DMD;CTNNA3;CTNNA2;CSMD1;BRINP3;NTRK2;CNTN5;AUTS2;ATRNL1;PCDH7;PDE4DIP;ESRRG;PTPRD;AGBL4;DLG2;DAB1;NBEA;KIF26B;RIMBP2*
FOXP2	52/299	1.10265112770121E-31	*ROBO2;CNTNAP2;TENM2;PTPRM;ZBTB20;RORA;GRIK2;BACH2;PTPRG;RIMS1;TRPS1;SOX6;EDIL3;SOX5;CADPS2;RBFOX1;DCC;PDE4D;ANK2;MITF;LMO7;NRG1;ANK1;PLCB4;CDH13;TOX;DDR2;NRXN1;NRXN3;HDAC9;ERBB4;DMD;SLIT2;CSMD1;GPC6;NTRK2;AUTS2;NEGR1;CADM2;ATRNL1;PCDH7;ESRRG;ARHGAP24;PTPRD;DLG2;NBEA;KIF26B;PDE3A;FAT3;CNTN4;SCN2A;BMPR1B*
PKHD1	52/299	1.10265112770121E-31	*ROBO2;DPP10;RYR2;CNTNAP2;TENM2;ZBTB20;RORA;GRIK2;ADARB2;CDH6;DOCK10;RIMS1;CDH4;MACROD2;SOX6;SOX5;CADPS2;RBFOX1;DSCAM;DCC;PDE4D;LMO7;ANK1;NRG3;CDH13;FREM2;DDR2;DNAH8;DNAH5;NRXN1;PCDH15;NRXN3;FHIT;HDAC9;DPP6;ERBB4;DMD;CTNNA3;CTNNA2;SLIT2;CSMD1;GPC6;NTRK2;CNTN5;ESRRG;PTPRD;DLG2;DAB1;NBEA;KIF26B;FAT3;BMPR1B*
ZMAT4	52/299	1.1026511277	*ROBO2;DPP10;PCSK1;RYR2;CNTNAP2;TENM2;D*

		0121E-31	*GKB;CELF4;GRIK2;CDH6;RIMS1;CDH4;DNER;EDIL3;SOX5;PPFIA2;RGS7;CADPS2;RBFOX1;PDE4D;NRG1;ANK1;NRG3;CDH13;TOX;FREM2;CDH18;NRXN1;NRXN3;ADCY2;HDAC9;DPP6;CTNNA2;SLIT2;CSMD1;NTRK2;CNTN5;AUTS2;NEGR1;CADM2;PCDH7;CADPS;ESRRG;ENOX1;PTPRD;NELL1;DLG2;FGF14;DAB1;FAT3;CNTN4;FGF13*
ZNF536	52/299	1.10265112770121E-31	*ROBO2;RYR2;CNTNAP2;TENM2;CELF4;RORA;GRIK2;ADARB2;BACH2;RIMS1;CDH4;DNER;SOX6;EDIL3;SOX5;PPFIA2;CADPS2;RBFOX1;DCC;PDE4D;ANK2;NRG1;ANK1;NAV3;NRG3;CDH13;TOX;FREM2;ASTN1;DDR2;NRXN1;NRXN3;HDAC9;DPP6;ERBB4;CTNNA2;SLIT2;CSMD1;NTRK2;AUTS2;NEGR1;ATRNL1;PLCL1;PCDH7;CADPS;ESRRG;PTPRD;NELL1;DLG2;DAB1;KIF26B;CNTN4*
SOX5	51/299	1.2120948695435296E-30	*ROBO2;CNTNAP2;TENM2;MAST4;PTPRM;ZBTB20;RORA;GRIK2;BACH2;PTPRG;CDH6;DOCK10;TRPS1;SOX6;EDIL3;MAGI1;CADPS2;RBFOX1;DCC;PDE4D;ANK2;MITF;NRG1;ANK1;PLCB4;NAV3;CDH13;TNIK;UTRN;TOX;DDR2;NRXN1;NRXN3;HDAC9;ERBB4;DMD;SLIT2;CSMD1;GPC6;NTRK2;AUTS2;NEGR1;ATRNL1;PCDH7;ESRRG;ARHGAP24;PTPRD;DLG2;DAB1;NBEA;FAT3*
PLXNA4	49/299	1.4490098263171776E-28	*ROBO2;RYR2;CNTNAP2;TENM2;MAST4;PTPRM;ZBTB20;RORA;GRIK2;BACH2;PTPRG;CDH4;EDIL3;SOX5;MAGI1;CADPS2;RBFOX1;PDE4D;ANK2;LMO7;NRG1;ANK1;NAV3;CDH13;TNIK;DDR2;NRXN1;NRXN3;HDAC9;DPP6;ERBB4;DMD;SLIT2;CSMD1;GPC6;RAP1GAP2;NTRK2;AUTS2;NEGR1;ATRNL1;PCDH7;CADPS;ATP2B4;PTPRD;DLG2;DAB1;NBEA;KIF26B;FAT3*
ST18	48/299	1.4034256175131093E-27	*ROBO2;DPP10;PCSK1;CNTNAP2;TENM2;DGKB;NRXN1;CELF4;NRXN3;ZBTB20;RORA;GRIK2;NOL4;HDAC9;BACH2;DOCK10;FSTL5;DPP6;ERBB4;TRPS1;DNER;CTNNA2;SOX6;EDIL3;TRPM3;SOX5;PPFIA2;CADPS2;NTRK2;RBFOX1;AUTS2;NEGR1;DCC;KCNB2;PLCL1;CADPS;ANK2;ESRRG;ANK1;PTPRD;RALYL;DLG2;NAV3;RIMBP2;FAT3;CNTN4;TOX;DDR2*
ZNF385B	48/299	1.4034256175131093E-27	*ROBO2;RYR2;CNTNAP2;TENM2;PARM1;NRXN1;NRXN3;PTPRM;ZBTB20;RORA;GRIK2;HDAC9;RIMS1;CDH4;DPP6;ERBB4;DNER;DMD;CTNNA3;CTNNA2;MACROD2;SLIT2;CSMD1;GPC6;CADPS2;NTRK2;RBFOX1;AUTS2;NEGR1;CADM2;ATRNL1;PDE4D;PCDH7;CADPS;ANK2;ESRRG;NRG1;ANK1;PTPRD;NELL1;DLG2;NBEA;NAV3;CDH13;FAT3;CNTN4;FGF13;DDR2*
ZNF804A	46/299	1.438481725149118E-25	*ROBO2;RYR2;CNTNAP2;TENM2;PARM1;NRXN1;NRXN3;RORA;GRIK2;HDAC9;DOCK10;FSTL5;RIMS1;ERBB4;TRPS1;DNER;DMD;SLIT2;CSMD1;GPC6;EDIL3;CADPS2;CNTN5;AUTS2;NEGR1;ATRNL1;DCC;PDE4D;PLCL1;PCDH7;ANK2;NRG1;ARHGAP24;PTPRD;NELL1;LRFN5;DLG2;NBEA;NAV3;KIF26B;CDH13;FAT3;CNTN4;SCN2A;TOX;DDR2*
ADAMTS17	45/299	1.3643530685113195E-24	*ROBO2;CASZ1;TENM2;PARM1;MAST4;NRXN1;NRXN3;PTPRM;ZBTB20;ADCY2;RORA;ADARB2;HDAC9;BACH2;PTPRG;CDH4;DPP6;SLIT2;SOX6;CSMD1;GPC6;SOX5;MAGI1;CADPS2;NTRK2;RBFOX1;AUTS2;DCC;PDE4D;PCDH7;KAZN;ANK2;LMO7*

			;ANK1;SH3RF3;PTPRD;DLG2;DAB1;NAV3;MYO5B;KIF26B;CDH13;FAT3;FREM2;DDR2
ADAMTS19	42/299	1.0341910257909115E-21	ROBO2;RYR2;CNTNAP2;TENM2;PARM1;NRXN1;PCDH15;NRXN3;ADCY2;GRIK2;CDH6;CDH4;ERBB4;GPC3;CTNNA2;SLIT2;CSMD1;EDIL3;CADPS2;NTRK2;RBFOX1;DSCAM;AUTS2;NEGR1;ATRNL1;PDE4D;PCDH7;CADPS;ESRRG;ANK1;ANO5;PTPRD;LRFN5;DLG2;MYO5B;KIF26B;PDE3A;FAT3;CNTN4;FGF13;FREM2;DDR2
ZNF98	42/299	1.0341910257909115E-21	ROBO2;DPP10;RYR2;CNTNAP2;TENM2;DNAH7;KMT2C;NRXN1;NRXN3;RORA;GRIK2;CDH4;DPP6;GRM7;TRPS1;PCDHA3;DMD;GPC5;CTNNA2;SLIT2;SOX6;CSMD1;CADPS2;NTRK2;RBFOX1;CNTN5;AUTS2;DCC;PDE4D;PCDH7;PDE4DIP;NRG1;PTPRD;MGAT4C;DAB1;NBEA;PDE3A;CDH13;CNTN4;BMPR1B;FREM2;DDR2
CAMTA1	41/299	8.425603990529185E-21	ROBO2;RYR2;CNTNAP2;CASZ1;TENM2;MAST4;NRXN1;CELF4;NRXN3;ZBTB20;RORA;BACH2;PTPRG;CDH4;DPP6;TRPS1;MACROD2;CSMD1;SOX5;MAGI1;RAP1GAP2;CADPS2;NTRK2;RBFOX1;AUTS2;NEGR1;ATRNL1;PDE4D;PCDH7;PDE4DIP;ANK2;LMO7;ESRRG;ANK1;PTPRD;DLG2;DAB1;NBEA;KIF26B;CDH13;DDR2
PRDM16	41/299	8.425603990529185E-21	RYR2;CNTNAP2;CASZ1;TENM2;NRXN1;NRXN3;PTPRM;ZBTB20;RORA;BACH2;PTPRG;CDH4;DPP6;ERBB4;TRPS1;DMD;SLIT2;SOX6;CSMD1;SOX5;MAGI1;RAP1GAP2;CADPS2;NTRK2;RBFOX1;AUTS2;PDE4D;PCDH7;DNMT3A;KAZN;ANK2;ESRRG;ANK1;PTPRD;DLG2;DAB1;KIF26B;CDH13;TOX;BMPR1B;DDR2
ZNF717	40/299	6.957954091157082E-20	ROBO2;CNTNAP2;PATJ;KMT2C;NRXN1;NRXN3;PTPRM;ZBTB20;RORA;GRIK2;FHIT;HDAC9;BACH2;DOCK10;CDH4;DPP6;ERBB4;DMD;CTNNA2;MACROD2;CSMD1;SOX5;CADPS2;RBFOX1;CNTN5;AUTS2;ATRNL1;PDE4D;PDE4DIP;MITF;NBPF1;PTPRD;LRFN5;PLCB4;NBEA;KIF26B;TNIK;CNTN4;FREM2;DDR2
ZNF716	39/299	5.577824845411585E-19	ROBO2;DPP10;RYR2;CNTNAP2;TENM2;NBAS;DGKB;KMT2C;DNAH5;NRXN1;PCDH15;NRXN3;ZBTB20;GRIK2;FSTL5;CDH4;DPP6;SGCD;MACROD2;CSMD1;RBFOX1;DSCAM;CNTN5;AUTS2;CNTN6;DCC;PDE4D;PCDH7;PXDNL;ESRRG;NRG1;PTPRD;DLG2;NRG3;CDH13;FAT3;MDGA2;CDH18;DDR2
AFF3	38/299	3.917839375560439E-18	ROBO2;CNTNAP2;MAST4;NRXN3;PTPRM;ZBTB20;RORA;HDAC9;BACH2;DOCK10;TRPS1;SLIT2;SOX6;SOX5;RAP1GAP2;CADPS2;NTRK2;AUTS2;NEGR1;ANKRD44;PDE4D;PCDH7;ATP2B4;ANK2;LMO7;NRG1;ANK1;PTPRD;PLCB4;NBEA;NAV3;KIF26B;CDH13;FAT3;TNIK;UTRN;TOX;DDR2
CHD6	38/299	3.917839375560439E-18	ROBO2;RYR2;CNTNAP2;CASZ1;MAST4;KMT2C;NRXN1;CELF4;NRXN3;ZBTB20;RORA;PPP1R9A;BACH2;CDH4;TRPS1;MAGI1;RAP1GAP2;DYNC1H1;CADPS2;NTRK2;AUTS2;PDE4D;PCDH7;DNMT3A;CADPS;ATP2B4;PRRC2C;PDE4DIP;ANK2;LMO7;ANK1;PTPRD;DLG2;NBEA;KIF26B;TNIK;UTRN;DDR2
CPXCR1	38/299	3.917839375560439E-18	ROBO2;DPP10;CNTNAP2;TENM2;DGKB;DNAH5;NRXN1;PCDH15;HTR2C;GRIK2;ADARB2;DACH2;NOL4;HDAC9;PHEX;CDH6;FSTL5;DMD;CTNNA3;SLIT2;CSMD1;GPC6;BRINP3;CADPS2;RBFOX1;C

			NTN5;CADM2;CNTN6;DCC;PCDH7;NRG1;OTOGL;PTPRD;MGAT4C;NAV3;CDH12;CDH18;DDR2
SOX6	38/299	3.917839375560439E-18	*ROBO2;TENM2;NRXN1;PTPRM;ZBTB20;RORA;PPP1R9A;HDAC9;BACH2;CDH6;ERBB4;TRPS1;DMD;SLIT2;GPC6;SOX5;MAGI1;CADPS2;NTRK2;AUTS2;PDE4D;PCDH7;ATP2B4;ANK2;MITF;LMO7;ESRRG;ANK1;PTPRD;DLG2;NBEA;KIF26B;CDH13;FAT3;TNIK;UTRN;TOX;DDR2*
TFAP2D	37/299	2.7093099464421133E-17	*ROBO2;DPP10;CNTNAP2;TENM2;DGKB;NRXN1;CELF4;NRXN3;RORA;GRIK2;ADARB2;NOL4;GRM3;RIMS1;GRM7;CSMD1;BRINP3;SOX5;CADPS2;RBFOX1;CNTN5;NEGR1;CADM2;CNTN6;DCC;PCDH7;PAX3;ESRRG;ANK1;NELL1;LRFN5;RALYL;DLG2;PAX7;CDH12;CNTN4;CDH18*
THRB	37/299	2.7093099464421133E-17	*ROBO2;PARM1;NRXN1;NRXN3;PTPRM;ZBTB20;RORA;HDAC9;BACH2;ERBB4;TRPS1;DMD;SLIT2;SOX6;GPC6;EDIL3;SOX5;MAGI1;CADPS2;NTRK2;NEGR1;PDE4D;PCDH7;ANK2;MITF;LMO7;ESRRG;ANK1;PTPRD;PLCB4;DLG2;NBEA;CDH13;TNIK;UTRN;TOX;DDR2*
ZNF479	37/299	2.7093099464421133E-17	*ROBO2;DPP10;CNTNAP2;TENM2;DGKB;KMT2C;DNAH5;NRXN3;DPP6;ERBB4;ZNF385B;DMD;CTNNA2;MACROD2;CSMD1;ZNF267;CADPS2;RBFOX1;CNTN5;AUTS2;CNTN6;DCC;PDE4D;PLCL1;ZNF804B;PDE4DIP;NRG1;PTPRD;MGAT4C;DLG2;DAB1;AGBL1;NBEA;NRG3;FAT3;CNTN4;CDH18*
ZNF99	37/299	2.7093099464421133E-17	*ROBO2;CHODL;DPP10;RYR2;CNTNAP2;TENM2;DNAH8;KMT2C;DNAH5;NRXN1;NRXN3;NOL4;CDH6;DPP6;PCDHA3;DMD;CTNNA3;SOX6;TRPM6;CSMD1;BRINP3;CADPS2;CNTN5;NEGR1;DCC;PDE4DIP;PXDNL;NRG1;ANO5;PTPRD;RALYL;NAV3;NRG3;FAT3;CNTN4;MDGA2;FREM2*
LMX1A	36/299	1.9359789494956158E-16	*DPP10;CNTNAP2;CASZ1;TENM2;NRXN1;CELF4;NRXN3;ADCY2;CDH6;CDH4;DPP6;ERBB4;DNER;CTNNA2;SLIT2;SOX6;CSMD1;BRINP3;SOX5;RGS7;CADPS2;NTRK2;RBFOX1;DCC;CADPS;PAX3;ESRRG;NRG1;ANK1;NELL1;DAB1;PAX7;KIF26B;CDH13;ASTN1;DDR2*
RFX3	36/299	1.9359789494956158E-16	*ROBO2;MAST4;KMT2C;NRXN1;ZBTB20;RORA;PPP1R9A;LYST;HDAC9;BACH2;DOCK10;TRPS1;SOX6;SOX5;MAGI1;CADPS2;NTRK2;CERS6;AUTS2;NEGR1;ANKRD44;PDE4D;TCF12;DNMT3A;ATP2B4;ANK2;LMO7;ESRRG;ANK1;PTPRD;PLCB4;NBEA;TNIK;UTRN;TOX;DDR2*
CUX2	35/299	1.3414473605662394E-15	*RYR2;CNTNAP2;CASZ1;NRXN1;CELF4;NRXN3;RORA;HDAC9;BACH2;CDH4;CSMD1;SOX5;MAGI1;RAP1GAP2;CADPS2;NTRK2;RBFOX1;AUTS2;NEGR1;PDE4D;CADPS;KAZN;ANK2;ESRRG;ANK1;PTPRD;DLG2;DAB1;NBEA;KIF26B;RIMBP2;CDH13;FAT3;TOX;DDR2*
ZNF454	35/299	1.3414473605662394E-15	*ROBO2;DPP10;PCSK1;RYR2;CNTNAP2;GALNT13;NRXN1;NRXN3;GRIK2;CDH4;DPP6;GRM7;ERBB4;CTNNA2;SLIT2;CSMD1;SOX5;CADPS2;RBFOX1;NEGR1;DCC;PCDH7;CADPS;ANK2;ESRRG;NRG1;PCDHA12;PTPRD;NELL1;LRFN5;DAB1;NBEA;KIF26B;CNTN4;DDR2*
KLF12	34/299	8.390119777829284E-15	*ROBO2;PTPRM;ZBTB20;RORA;HDAC9;BACH2;PTPRG;DOCK10;TRPS1;SLIT2;SOX6;GPC6;SOX5;MAGI1;CADPS2;NTRK2;AUTS2;NEGR1;PDE4D;PCDH7;TCF12;ATP2B4;PDE4DIP;LMO7;ANK1;AR*

			HGAP24;PTPRD;PLCB4;NBEA;NAV3;TNIK;UTRN;TOX;DDR2
RORB	34/299	8.390119777829284E-15	*ROBO2;CNTNAP2;TENM2;NRXN1;NRXN3;ZBTB20;ADCY2;RORA;GRIK2;HDAC9;CDH6;CDH4;TRPS1;SLIT2;SOX6;GPC6;EDIL3;SOX5;CADPS2;NTRK2;RBFOX1;NEGR1;CADM2;PDE4D;PCDH7;ANK2;ESRRG;ANK1;PTPRD;DLG2;CDH13;FAT3;TOX;DDR2*
ZFPM2	34/299	8.390119777829284E-15	*ROBO2;RYR2;TENM2;NRXN3;PTPRM;ZBTB20;RORA;HDAC9;CDH6;ERBB4;TRPS1;DMD;SLIT2;GPC6;EDIL3;SOX5;CADPS2;NTRK2;RBFOX1;AUTS2;NEGR1;PDE4D;PCDH7;ANK2;MITF;NRG1;PTPRD;PLCB4;NBEA;NAV3;KIF26B;CDH13;TOX;DDR2*
ZNF407	34/299	8.390119777829284E-15	*ROBO2;DENND1B;MAST4;KMT2C;NRXN1;NRXN3;PTPRM;ZBTB20;RORA;HDAC9;BACH2;PTPRG;DOCK10;TRPS1;DMD;MACROD2;CSMD1;MAGI1;DYNC1H1;CADPS2;AUTS2;PDE4D;TCF12;PRRC2C;PDE4DIP;ANK2;LMO7;NRG1;ARHGAP24;PTPRD;NBEA;KIF26B;UTRN;DDR2*
ZSCAN1	34/299	8.390119777829284E-15	*FBLN7;DPP10;RYR2;CNTNAP2;CASZ1;NRXN1;CELF4;NRXN3;ADARB2;CDH4;DPP6;GRM7;TRPS1;CTNNA2;CSMD1;PCDHA6;CADPS2;RBFOX1;PCDH7;CADPS;NRG1;PCDHA12;ANK1;PCDHA10;SH3RF3;NELL1;DAB1;NRG3;PAX7;KIF26B;CDH13;FAT3;FREM2;DDR2*
FAM170A	33/299	5.239349511297019E-14	*ROBO2;DPP10;PCSK1;CNTNAP2;TENM2;GALNT13;DGKB;PCDH15;NRXN3;GRIK2;CDH6;FSTL5;CDH4;GRM7;ERBB4;GPC5;CSMD1;BRINP3;CADPS2;RBFOX1;CNTN5;CNTN6;DCC;ESRRG;LRFN5;RALYL;DLG2;MYO3B;KIF26B;CDH12;MDGA2;CDH18;DDR2*
NR3C2	33/299	5.239349511297019E-14	*PARM1;NRXN1;PTPRM;ZBTB20;RORA;PPP1R9A;HDAC9;BACH2;ERBB4;DMD;SLIT2;SOX6;SOX5;CADPS2;NTRK2;AUTS2;NEGR1;PDE4D;PLCL1;PCDH7;ANK2;MITF;LMO7;ANK1;ARHGAP24;PTPRD;PLCB4;NBEA;KIF26B;TNIK;UTRN;TOX;DDR2*
TSHZ2	33/299	5.239349511297019E-14	*ROBO2;CNTNAP2;TENM2;PARM1;MAST4;PTPRM;ZBTB20;RORA;BACH2;TRPS1;GPC3;DMD;SLIT2;SOX6;GPC6;SOX5;CADPS2;NTRK2;AUTS2;PDE4D;PCDH7;ATP2B4;ANK2;ESRRG;ANK1;ARHGAP24;PTPRD;DLG2;NBEA;KIF26B;CDH13;TOX;DDR2*
ZIM3	33/299	5.239349511297019E-14	*ROBO2;DPP10;RYR2;CNTNAP2;TENM2;DNAH5;NRXN1;PCDH15;NRXN3;FSTL5;RIMS1;ERBB4;CSMD1;BRINP3;SOX5;CADPS2;RBFOX1;CNTN5;AUTS2;CNTN6;DCC;ANK2;ESRRG;ANK1;PTPRD;MGAT4C;NELL1;NAV3;CDH12;FAT3;CNTN4;CDH18;DDR2*
FEZF2	32/299	3.240487750541081E-13	*ROBO2;PCSK1;TENM2;NRXN1;CELF4;NRXN3;ADCY2;GRIK2;NOL4;RIMS1;CDH4;DPP6;ERBB4;SLIT2;CSMD1;SOX5;RGS7;CADPS2;NTRK2;RBFOX1;DCC;PCDH7;CADPS;PAX3;NRG1;ANK1;NELL1;DAB1;PAX7;CDH13;TOX;DDR2*
SIM1	32/299	3.240487750541081E-13	*ROBO2;PCSK1;CASZ1;NRXN1;NRXN3;ZBTB20;RORA;GRIK2;HDAC9;CDH6;DPP6;ERBB4;TRPS1;DNER;SLIT2;SOX6;CSMD1;GPC6;EDIL3;SOX5;CADPS2;NTRK2;RBFOX1;DCC;PCDH7;PAX3;MITF;ESRRG;NRG1;ANK1;KIF26B;DDR2*
TGIF2LX	32/299	3.2404877505	*DPP10;NRXN1;PCDH15;CELF4;NRXN3;HTR2C;R*

		41081E-13	*ORA;GRIK2;DACH2;FSTL5;CDH4;DPP6;DMD;CTNNA2;CSMD1;GPC6;SOX5;RBFOX1;DCC;SH2D1A;PDE4DIP;MITF;ESRRG;ETV6;OTOGL;PTPRD;NRG3;MYO3B;CDH12;FGF13;CDH18;DDR2*
PKNOX2	31/299	1.9031450622981405E-12	*ROBO2;CNTNAP2;PARM1;NRXN1;NRXN3;PTPRM;ADCY2;RORA;BACH2;PTPRG;CDH6;CDH4;DPP6;SLIT2;SOX6;GPC6;SOX5;CADPS2;NTRK2;RBFOX1;AUTS2;PDE4D;PCDH7;ANK2;ESRRG;ANK1;PTPRD;DLG2;KIF26B;CDH13;DDR2*
RARB	31/299	1.9031450622981405E-12	*ROBO2;NRXN3;PTPRM;ZBTB20;RORA;HDAC9;BACH2;CDH6;ERBB4;TRPS1;SLIT2;SOX6;SOX5;CADPS2;NTRK2;NEGR1;PDE4D;PCDH7;MITF;LMO7;ESRRG;NRG1;ANK1;ARHGAP24;PTPRD;PLCB4;KIF26B;CDH13;FAT3;TOX;DDR2*
SETBP1	31/299	1.9031450622981405E-12	*ROBO2;MAST4;KMT2C;NRXN3;PTPRM;ZBTB20;RORA;HDAC9;BACH2;TRPS1;DMD;SLIT2;GPC6;SOX5;CADPS2;NTRK2;AUTS2;ATRNL1;PDE4D;PCDH7;ATP2B4;ANK2;ANK1;ARHGAP24;PTPRD;NBEA;KIF26B;TNIK;UTRN;TOX;DDR2*
ZFAT	31/299	1.9031450622981405E-12	*MAST4;NRXN3;ZBTB20;RORA;HDAC9;BACH2;TRPS1;SOX5;JAZF1;MAGI1;RAP1GAP2;CADPS2;RBFOX1;AUTS2;IQSEC1;PDE4D;DNMT3A;ATP2B4;PDE4DIP;LMO7;NRG1;ANK1;ETV6;PTPRD;KIF26B;FAT3;TNIK;UTRN;TOX;LPIN1;DDR2*
DPF3	30/299	1.1006712158505583E-11	*CASZ1;PTPRM;ZBTB20;RORA;HDAC9;BACH2;CDH4;SLIT2;SOX6;SOX5;RAP1GAP2;CADPS2;NTRK2;RBFOX1;AUTS2;NEGR1;PDE4D;ANK2;LMO7;ESRRG;NRG1;ANK1;ARHGAP24;DAB1;KIF26B;CDH13;TNIK;TOX;LPIN1;DDR2*
ZNF208	30/299	1.1006712158505583E-11	*RYR2;CNTNAP2;KMT2C;NRXN1;NRXN3;PPP1R9A;HDAC9;CDH6;DPP6;ERBB4;GPC3;DMD;CSMD1;EDIL3;CADPS2;NTRK2;NEGR1;ATRNL1;PCDH7;PDE4DIP;ANO5;PTPRD;DLG2;NBEA;NAV3;FAT3;CNTN4;FGF13;FREM2;DDR2*
ZNF718	30/299	1.1006712158505583E-11	*RYR2;CNTNAP2;CASZ1;NRXN1;NRXN3;RORA;GRIK2;PPP1R9A;DOCK10;BRIP1;DPP6;TRPS1;MACROD2;SLIT2;CSMD1;EDIL3;MAGI1;RAP1GAP2;CADPS2;AUTS2;PDE4D;PCDH7;DNMT3A;PDE4DIP;ANK1;NBPF1;PLCB4;NBEA;CDH13;DDR2*
MYT1	29/299	5.945289272349029E-11	*ROBO2;PCSK1;CNTNAP2;NRXN1;CELF4;NRXN3;RORA;GRIK2;NOL4;BACH2;CDH4;DPP6;ERBB4;DNER;CTNNA2;SOX5;CADPS2;NTRK2;DCC;CADPS;ANK2;ESRRG;ANK1;PTPRD;KIF26B;RIMBP2;TOX;DSCAML1;ASTN1*
POU3F2	29/299	5.945289272349029E-11	*ROBO2;CNTNAP2;NRXN1;NRXN3;RORA;GRIK2;NOL4;CDH6;CDH4;ERBB4;DNER;SLIT2;SOX6;SOX5;CADPS2;NTRK2;RBFOX1;NEGR1;DCC;PCDH7;PAX3;ESRRG;NRG1;PTPRD;FAT3;TOX;BMPR1B;MDGA2;DDR2*
RAPGEF4	29/299	5.945289272349029E-11	*ROBO2;RYR2;TENM2;NRXN1;PTPRM;ZBTB20;RORA;GRIK2;HDAC9;RIMS1;CTNNA2;DYNC1I1;RAP1GAP2;CADPS2;NTRK2;AUTS2;PDE4D;CADPS;ANK2;LMO7;ESRRG;ANK1;PTPRD;PLCB4;DLG2;CDH13;TNIK;SCN2A;DDR2*
ZFHX4	29/299	5.945289272349029E-11	*ROBO2;TENM2;ZBTB20;RORA;HDAC9;BACH2;CDH6;TRPS1;DMD;SLIT2;SOX6;GPC6;EDIL3;SOX5;CADPS2;NTRK2;AUTS2;NEGR1;PDE4D;PCDH7;ANK2;NRG1;PTPRD;NBEA;NAV3;KIF26B;FAT3;TOX;DDR2*
ZNF648	29/299	5.9452892723	*RYR2;CASZ1;TENM2;DNAH5;NRXN1;NRXN3;GRI*

		49029E-11	*K2;CDH4;DPP6;TRPS1;SLIT2;CSMD1;SOX5;RGS7;CADPS2;NTRK2;NEGR1;PDE4D;PCDH7;ATP2B4;PDE4DIP;ESRRG;PAX7;KIF26B;CDH13;FAT3;TOX;DSCAML1;DDR2*
DBX2	28/299	3.1057185257 90822E-10	*ROBO2;TENM2;NRXN1;RORA;ADARB2;CDH4;DPP6;TRPS1;GPC5;SOX6;CSMD1;SOX5;CADPS2;NTRK2;RBFOX1;CADM2;DCC;PAX3;ESRRG;ANK1;FGF14;DAB1;PPP2R2C;PAX7;KIF26B;BMPR1B;FREM2;DDR2*
TBX5	28/299	3.1057185257 90822E-10	*ROBO2;RYR2;CASZ1;NRXN1;GRIK2;DPP6;SGCD;SLIT2;SOX6;CSMD1;WNT2;SOX5;CADPS2;NTRK2;RBFOX1;PCDH7;CADPS;PAX3;MITF;ESRRG;NRG1;ANK1;PTPRD;NELL1;PAX7;KIF26B;CDH13;DDR2*
ZIC4	28/299	3.1057185257 90822E-10	*ROBO2;RYR2;TENM2;NRXN1;CELF4;NRXN3;GRIK2;CDH6;CDH4;DPP6;ERBB4;SLIT2;SOX6;SOX5;CADPS2;NTRK2;RBFOX1;DCC;PCDH7;CADPS;PAX3;ESRRG;NRG1;PAX7;KIF26B;CDH13;BMPR1B;DDR2*
ZNF578	28/299	3.1057185257 90822E-10	*ROBO2;RYR2;CNTNAP2;TENM2;NRXN1;NRXN3;GRIK2;HDAC9;CDH4;DPP6;ERBB4;CSMD1;RAP1GAP2;CADPS2;AUTS2;NEGR1;CADM2;DCC;PDE4D;PCDH7;LMO7;ESRRG;NRG1;PTPRD;NBEA;PDE3A;CNTN4;SCN2A*
ZSCAN23	28/299	3.1057185257 90822E-10	*ROBO2;RYR2;GALNT13;NRXN1;ZBTB20;RORA;BACH2;PTPRG;CDH6;TRPS1;SLIT2;SOX6;CSMD1;SOX5;CADPS2;AUTS2;NEGR1;ATRNL1;SGIP1;PCDH7;ANK2;ESRRG;NBEA;KIF26B;FAT3;TOX;BMPR1B;DDR2*
ARX	27/299	1.4655010591 468382E-9	*ROBO2;PCSK1;CNTNAP2;NRXN1;CELF4;NRXN3;ADCY2;RORA;GRIK2;CDH4;DPP6;DNER;GPC3;DMD;SLIT2;SOX6;CADPS2;NTRK2;RBFOX1;AUTS2;PCDH7;CADPS;ESRRG;ANK1;NELL1;FGF13;DDR2*
FOXD4L5	27/299	1.4655010591 468382E-9	*ROBO2;CNTNAP2;KMT2C;DNAH5;NRXN1;NRXN3;GRIK2;CDH4;DPP6;GRM7;TRPS1;DMD;CADPS2;NTRK2;RBFOX1;CNTN5;NEGR1;PDE4D;PCDH7;PDE4DIP;ESRRG;NRG1;NELL1;RALYL;DAB1;KIF26B;CDH18*
NEUROD6	27/299	1.4655010591 468382E-9	*ROBO2;PCSK1;TENM2;NRXN1;CELF4;NRXN3;HTR2C;GRIK2;NOL4;GRM3;CDH4;DPP6;SOX5;PPFIA2;RGS7;CADPS2;RBFOX1;NEGR1;DCC;CADPS;NELL1;RALYL;DLG2;DAB1;RIMBP2;CDH13;SCN2A*
POU3F4	27/299	1.4655010591 468382E-9	*ROBO2;DGKB;NRXN1;CELF4;HTR2C;RORA;GRIK2;CDH6;GRM3;CDH4;DPP6;DMD;SOX6;RGS9;EDIL3;SOX5;CADPS2;NTRK2;RBFOX1;NEGR1;DCC;PCDH7;PAX3;RALYL;DLG2;FAT3;DDR2*
PROX1	27/299	1.4655010591 468382E-9	*ROBO2;CASZ1;PARM1;NRXN1;ZBTB20;RORA;HDAC9;BACH2;ERBB4;TRPS1;SLIT2;SOX6;EDIL3;SOX5;CADPS2;NTRK2;AUTS2;PCDH7;ANK2;LMO7;ESRRG;ANK1;PTPRD;NBEA;KIF26B;TNIK;DDR2*
RFX4	27/299	1.4655010591 468382E-9	*RYR2;CNTNAP2;DGKB;NRXN1;CELF4;NRXN3;RORA;BACH2;CDH4;TRPS1;CTNNA2;SOX6;SOX5;CADPS2;NTRK2;RBFOX1;DCC;PAX3;ESRRG;NRG1;ANK1;DLG2;DAB1;PAX7;TOX;BMPR1B;DDR2*
SCAPER	27/299	1.4655010591 468382E-9	*MAST4;KMT2C;FAM13A;ZBTB20;RORA;PPP1R9A;LYST;HDAC9;BACH2;TRPS1;NEK1;NCOA1;MAGI1;CADPS2;AUTS2;PDE4D;TCF12;PDE4DIP;AN*

			K2;LMO7;NBEA;NAV3;ZNF638;TTC3;TNIK;UTRN;DDR2
ZNF483	27/299	1.4655010591468382E-9	*ROBO2;RYR2;NRXN1;ADAM22;NRXN3;RORA;PPP1R9A;ERBB4;DNER;SLIT2;EDIL3;RAP1GAP2;CADPS2;NTRK2;AUTS2;NEGR1;CADM2;PCDH7;CADPS;ANK2;PTPRD;PLCB4;NBEA;TNIK;TOX;FGF13;DDR2*
ZNF540	27/299	1.4655010591468382E-9	*CNTNAP2;PARM1;NRXN1;NRXN3;ZBTB20;RORA;GRIK2;BACH2;DPP6;DMD;PPFIA2;CADPS2;NTRK2;NEGR1;CADM2;ATRNL1;PDE4D;PLCL1;PDE4DIP;LMO7;ESRRG;DLG2;NBEA;NAV3;CDH12;SCN2A;DDR2*
ZNF835	27/299	1.4655010591468382E-9	*GLDC;NRXN1;NRXN3;ADCY2;GRIK2;ADARB2;PPP1R9A;DPP6;DNER;CTNNA2;CSMD1;CADPS2;NTRK2;RBFOX1;DSCAM;CADM2;DCC;PCDH7;CADPS;NRG1;ANK1;SH3RF3;CNTN4;TOX;DSCAML1;FREM2;DDR2*
DACH1	26/299	6.818886762672163E-9	*ROBO2;CNTNAP2;PARM1;NRXN1;RORA;HDAC9;BACH2;CDH6;ERBB4;TRPS1;SLIT2;SOX6;GPC6;EDIL3;SOX5;CADPS2;NTRK2;AUTS2;NEGR1;PCDH7;ESRRG;PTPRD;NBEA;CDH13;TOX;DDR2*
GRM6	26/299	6.818886762672163E-9	*RYR2;CASZ1;NRXN1;NRXN3;ADCY2;GRIK2;DOCK10;DPP6;GRM7;DMD;CTNNA2;CSMD1;CADPS2;NTRK2;RBFOX1;DSCAM;PDE4D;CADPS;ESRRG;NRG1;ANK1;KIF26B;CDH13;CNTN4;DSCAML1;DDR2*
NEUROD4	26/299	6.818886762672163E-9	*ROBO2;PCSK1;TENM2;NRXN1;PCDH15;CELF4;NRXN3;GRIK2;CDH6;FSTL5;ERBB4;CTNNA2;SOX5;CADPS2;NTRK2;RBFOX1;DCC;CADPS;PAX3;ESRRG;NRG1;FGF14;PAX7;BMPR1B;ASTN1;DDR2*
NHLH2	26/299	6.818886762672163E-9	*ROBO2;CASZ1;NRXN1;NRXN3;GRIK2;NOL4;DPP6;ERBB4;DNER;SLIT2;SOX6;CADPS2;NTRK2;RBFOX1;AUTS2;DCC;PCDH7;CADPS;PAX3;ESRRG;NRG1;ANK1;PAX7;KIF26B;CDH13;DDR2*
PRDM5	26/299	6.818886762672163E-9	*ROBO2;PTPRM;ZBTB20;RORA;HDAC9;PTPRG;TRPS1;DMD;SLIT2;GPC6;EDIL3;SOX5;CADPS2;AUTS2;PCDH7;ANK2;MITF;NRG1;ANK1;PTPRD;PLCB4;NBEA;CDH13;UTRN;BMPR1B;DDR2*
PRDM9	26/299	6.818886762672163E-9	*KMT2C;DNAH5;NRXN1;PCDH15;RORA;GRIK2;HDAC9;DOCK10;DPP6;TRPS1;DMD;CSMD1;BRINP3;CADPS2;CNTN5;PCDH7;PDE4DIP;ESRRG;ANK1;DLG2;CDH12;FAT3;UTRN;LPIN1;CDH18;DDR2*
SALL3	26/299	6.818886762672163E-9	*ROBO2;TRIM71;CNTNAP2;CASZ1;GLDC;NRXN1;CELF4;NRXN3;RORA;CDH4;DPP6;SOX6;CSMD1;SOX5;CADPS2;NTRK2;RBFOX1;AUTS2;CADPS;ESRRG;ANK1;PTPRD;DAB1;KIF26B;CDH13;DDR2*
SATB2	26/299	6.818886762672163E-9	*ROBO2;ZBTB20;RORA;PTPRG;TRPS1;SLIT2;SOX5;CADPS2;NTRK2;AUTS2;PDE4D;PCDH7;ATP2B4;ANK2;LMO7;ESRRG;ANK1;PTPRD;PLCB4;NBEA;NAV3;CDH13;TNIK;UTRN;TOX;DDR2*
ZNF568	26/299	6.818886762672163E-9	*RYR2;CNTNAP2;KMT2C;NRXN1;NRXN3;ZBTB20;RORA;GRIK2;HDAC9;PTPRG;DPP6;DMD;SLIT2;CSMD1;CADPS2;AUTS2;PDE4D;PCDH7;PDE4DIP;ANK2;LMO7;ESRRG;PTPRD;PLCB4;NBEA;DDR2*
DLX6	25/299	3.136556624776081E-8	*ROBO2;CNTNAP2;NRXN1;CELF4;NRXN3;RORA;GRIK2;DPP6;ERBB4;SLIT2;SOX6;DYNC1I1;SOX5;CADPS2;NTRK2;RBFOX1;PCDH7;PAX3;LMO7;ESRRG;NRG1;ANK1;PAX7;KIF26B;DDR2*
PLXNA2	25/299	3.136556624776081E-8	*TENM2;PTPRM;ZBTB20;RORA;BACH2;PTPRG;SLIT2;CADPS2;NTRK2;AUTS2;PDE4D;PCDH7;ATP*

			2B4;PDE4DIP;ANK2;LMO7;NRG1;ANK1;PTPRD;NBEA;KIF26B;CDH13;FAT3;TNIK;DDR2
PRDM10	25/299	3.1365566247 76081E-8	*MAST4;KMT2C;ZBTB20;RORA;LYST;HDAC9;BACH2;TRPS1;MAGI1;DYNC1H1;CERS6;AUTS2;PDE4D;TCF12;DNMT3A;ATP2B4;PDE4DIP;LMO7;ANK1;ETV6;PLCB4;NBEA;TNIK;UTRN;DDR2*
RORA	25/299	3.1365566247 76081E-8	*NRXN3;PTPRM;ZBTB20;HDAC9;BACH2;TRPS1;SOX6;GPC6;SOX5;CADPS2;NTRK2;AUTS2;PDE4D;PDE4DIP;ANK2;ESRRG;ANK1;PTPRD;PLCB4;DLG2;CDH13;TNIK;UTRN;TOX;DDR2*
SORBS2	25/299	3.1365566247 76081E-8	*NRXN1;PTPRM;ZBTB20;RORA;HDAC9;DMD;SLIT2;MAGI1;CADPS2;NTRK2;AUTS2;PDE4D;PCDH7;ATP2B4;ANK2;LMO7;ANK1;PTPRD;PLCB4;NBEA;NAV3;CDH13;TNIK;UTRN;DDR2*
TUB	25/299	3.1365566247 76081E-8	*CNTNAP2;NRXN1;NRXN3;RORA;GRIK2;DPP6;SLIT2;EDIL3;RAP1GAP2;CADPS2;NTRK2;NEGR1;PDE4D;PCDH7;ATP2B4;ANK2;LMO7;ANK1;PTPRD;DLG2;DAB1;NBEA;NAV3;PPP2R2C;DDR2*
ZNF418	25/299	3.1365566247 76081E-8	*FBLN7;MAST4;KMT2C;NRXN1;NRXN3;PTPRM;ZBTB20;RORA;GRIK2;HDAC9;BACH2;DPP6;DMD;CADPS2;AUTS2;PDE4D;PCDH7;DNMT3A;PDE4DIP;ANK2;NRG1;NBEA;TNIK;FREM2;DDR2*
BCL11B	24/299	1.3220078202 860265E-7	*ROBO2;RAP1GAP2;CADPS2;CNTNAP2;NTRK2;RBFOX1;CASZ1;AUTS2;PDE4D;PCDH7;ATP2B4;ZBTB20;RORA;ANK1;BACH2;DOCK10;CDH4;NBEA;KIF26B;TRPS1;TNIK;TOX;SOX5;DDR2*
ESRRB	24/299	1.3220078202 860265E-7	*ROBO2;CADPS2;CNTNAP2;NTRK2;RBFOX1;CASZ1;GLDC;PDE4D;CELF4;NRXN3;RORA;MITF;ESRRG;NRG1;ANK1;HDAC9;ENOX1;CDH4;DAB1;ERBB4;KIF26B;CDH13;SOX5;DDR2*
INSM2	24/299	1.3220078202 860265E-7	*RAP1GAP2;PCSK1;RYR2;CADPS2;NTRK2;RBFOX1;CASZ1;DCC;NRXN1;CADPS;CELF4;NRXN3;RORA;ESRRG;ANK1;NOL4;DPP6;RALYL;KIF26B;DNER;RIMBP2;SLIT2;TOX;RGS7*
PAX7	24/299	1.3220078202 860265E-7	*RYR2;CADPS2;NTRK2;RBFOX1;CASZ1;DCC;CELF4;NRXN3;PAX3;ESRRG;NRG1;ADARB2;ANK1;NELL1;CDH4;DPP6;DAB1;ERBB4;KIF26B;CDH13;SLIT2;SOX6;SOX5;DDR2*
SOHLH2	24/299	1.3220078202 860265E-7	*RYR2;CADPS2;CNTNAP2;RBFOX1;TENM2;PARM1;GLDC;PDE4D;FAM13A;NRXN3;MITF;LMO7;NRG1;ANK1;ENOX1;PPP2R2C;KIF26B;CDH13;TNIK;SLIT2;FGF13;CSMD1;GPC6;DDR2*
TBR1	24/299	1.3220078202 860265E-7	*ROBO2;CADPS2;CNTNAP2;RBFOX1;KDM4B;DSCAM;KMT2C;NRXN1;DNMT3A;CELF4;NRXN3;PTPRM;ANK2;RORA;ESRRG;ANK1;RIMS1;CDH4;NBEA;GRM7;SCN2A;SOX5;DDR2;RGS7*
TFAP2B	24/299	1.3220078202 860265E-7	*ROBO2;CADPS2;NTRK2;RBFOX1;TENM2;DCC;PCDH7;CELF4;PAX3;RORA;MITF;ESRRG;NRG1;GRIK2;ANK1;PAX7;ERBB4;KIF26B;SLIT2;SOX6;CSMD1;EDIL3;SOX5;DDR2*
TOX	24/299	1.3220078202 860265E-7	*ROBO2;CADPS2;NTRK2;AUTS2;NEGR1;PDE4D;NRXN1;PCDH7;PTPRM;ZBTB20;RORA;NRG1;ANK1;HDAC9;BACH2;DOCK10;PTPRD;PLCB4;KIF26B;TRPS1;TNIK;SLIT2;SOX5;DDR2*
ZKSCAN2	24/299	1.3220078202 860265E-7	*ROBO2;CADPS2;CNTNAP2;CERS6;AUTS2;NEGR1;DCC;KMT2C;PDE4D;PLCL1;DNMT3A;ANK2;RORA;ANK1;BACH2;PTPRD;DLG2;NBEA;KIF26B;TRPS1;TNIK;SLIT2;TOX;DDR2*
ZNF135	24/299	1.3220078202 860265E-7	*MAGI1;CADPS2;CNTNAP2;AUTS2;NEGR1;PDE4D;NRXN1;PCDH7;NRXN3;PTPRM;ANK2;RORA;HDA*

			C9;BACH2;PTPRD;DPP6;PLCB4;NBEA;DMD;CNTN4;SLIT2;SOX6;TOX;DDR2
ZNF354C	24/299	1.3220078202860265E-7	ROBO2;RYR2;CADPS2;NTRK2;TENM2;AUTS2;PCDH7;ZBTB20;SMAD9;RORA;NRG1;HDAC9;BACH2;PLCB4;DLG2;NBEA;TRPS1;PDE3A;DMD;SLIT2;SOX6;EDIL3;SOX5;DDR2
ZNF660	24/299	1.3220078202860265E-7	ROBO2;CADPS2;AUTS2;NEGR1;DCC;KMT2C;PLCL1;NRXN3;ZBTB20;RORA;PPP1R9A;HDAC9;PTPRG;CDH6;DOCK10;PTPRD;FGF14;NBEA;TNIK;SLIT2;TOX;GPC6;SOX5;DDR2
BCL11A	23/299	5.256476699850769E-7	CADPS2;CNTNAP2;NTRK2;DSCAM;AUTS2;KMT2C;PDE4D;NRXN1;PCDH7;DNMT3A;ZBTB20;ANK2;RORA;ANK1;HDAC9;BACH2;PTPRD;RIMS1;TRPS1;TNIK;SCN2A;TOX;DDR2
DEPDC5	23/299	5.256476699850769E-7	MAGI1;DYNC1H1;CADPS2;AUTS2;MAST4;IQSEC1;KMT2C;PDE4D;DNMT3A;ATP2B4;PRRC2C;ZBTB20;PDE4DIP;ANK2;ANK1;LYST;HDAC9;PTPRD;PLCB4;TNIK;UTRN;LPIN1;DDR2
FOXG1	23/299	5.256476699850769E-7	ROBO2;CADPS2;CNTNAP2;NTRK2;RBFOX1;AUTS2;NRXN1;PCDH7;CELF4;PAX3;ZBTB20;LMO7;GRIK2;ANK1;CDH6;CDH4;DPP6;NBEA;ERBB4;CDH13;SLIT2;SOX5;DDR2
HDX	23/299	5.256476699850769E-7	ROBO2;CADPS2;NEGR1;PDE4D;PLCL1;PCDH7;ZBTB20;ANK2;NRG1;HDAC9;BACH2;CDH6;DOCK10;PTPRD;PLCB4;NBEA;TRPS1;DMD;TNIK;SLIT2;TOX;GPC6;DDR2
KIAA1549	23/299	5.256476699850769E-7	MAGI1;NTRK2;AUTS2;KMT2C;PDE4D;DNMT3A;NRXN3;ATP2B4;PDE4DIP;ANK2;LMO7;PPP1R9A;BACH2;PTPRD;PLCB4;NBEA;KIF26B;TRPS1;FAT3;TNIK;FREM2;SOX5;DDR2
PAX3	23/299	5.256476699850769E-7	ROBO2;CADPS2;NTRK2;RBFOX1;DCC;PCDH7;RORA;MITF;ESRRG;NRG1;GRIK2;ANK1;CDH6;PTPRD;CDH4;PAX7;ERBB4;KIF26B;SLIT2;SOX6;BMPR1B;SOX5;DDR2
POU4F1	23/299	5.256476699850769E-7	CADPS2;CNTNAP2;NTRK2;RBFOX1;CASZ1;AUTS2;CELF4;NRXN3;PAX3;RORA;ESRRG;NRG1;ANK1;HDAC9;BACH2;NELL1;CDH4;PAX7;KIF26B;SLIT2;TOX;SOX5;DDR2
TBX22	23/299	5.256476699850769E-7	ROBO2;CHODL;DPP10;CADPS2;COLEC10;CNTN5;CNTN6;PCDH15;HTR2C;DACH2;ANK1;MGAT4C;DPP6;LRFN5;ERBB4;CDH12;DMD;CNTN4;FGF13;MDGA2;BRINP3;CDH18;DDR2
TOX3	23/299	5.256476699850769E-7	ROBO2;CADPS2;CNTNAP2;NTRK2;PARM1;AUTS2;NRXN1;PCDH7;CADPS;RORA;LMO7;ESRRG;BACH2;PTPRD;CDH4;DAB1;NBEA;KIF26B;CDH13;SLIT2;TOX;GPC6;DDR2
ZBTB20	23/299	5.256476699850769E-7	CADPS2;NTRK2;AUTS2;MAST4;KMT2C;PDE4D;PTPRM;PDE4DIP;ANK2;RORA;ANK1;HDAC9;BACH2;PTPRD;NBEA;TRPS1;DMD;TNIK;SOX6;UTRN;LPIN1;SOX5;DDR2
ZNF229	23/299	5.256476699850769E-7	ROBO2;RYR2;CADPS2;CNTNAP2;AUTS2;NEGR1;KMT2C;PDE4D;NRXN1;PCDH7;NRXN3;KIAA1549L;PTPRM;LMO7;NRG1;NBEA;NAV3;TRPS1;FAT3;DMD;CSMD1;GPC6;DDR2
ZNF462	23/299	5.256476699850769E-7	MAGI1;CADPS2;AUTS2;MAST4;KMT2C;PDE4D;PCDH7;ATP2B4;ZBTB20;ANK2;RORA;LMO7;PPP1R9A;PTPRG;PTPRD;NBEA;NAV3;KIF26B;TRPS1;TNIK;UTRN;SOX5;DDR2
ZNF471	23/299	5.256476699850769E-7	RYR2;CADPS2;NTRK2;NEGR1;PLCL1;PCDH7;ZBTB20;RORA;MITF;GRIK2;LYST;PLCB4;NBEA;C

			DH13;FAT3;DMD;CNTN4;SLIT2;SOX6;MPDZ;EDIL3;SOX5;DDR2
ZNF676	23/299	5.256476699850769E-7	*RYR2;CADPS2;CNTNAP2;NTRK2;TENM2;NEGR1;ATRNL1;NRXN1;PCDH7;GRIK2;ANO5;DPP6;DLG2;NBEA;NAV3;PDE3A;GPC3;CTNNA2;CNTN4;SOX6;CSMD1;EDIL3;DDR2*
ZNF705D	23/299	5.256476699850769E-7	*PCSK1;CADPS2;CNTNAP2;RBFOX1;CNTN5;NEGR1;DCC;KMT2C;PLCL1;PDE4DIP;RORA;ESRRG;NBPF1;FSTL5;NELL1;DPP6;KIF26B;DMD;MACROD2;TOX;CSMD1;WNT2;SOX5*
ASCL1	22/299	2.054088790619987E-6	*CADPS2;CNTNAP2;NTRK2;DCC;NRXN1;CADPS;CELF4;RORA;ESRRG;NRG1;NOL4;CDH6;NELL1;DPP6;FGF14;ERBB4;DNER;SOX6;TOX;SOX5;ASTN1;DDR2*
DUXA	22/299	2.054088790619987E-6	*ROBO2;RYR2;RBFOX1;CNTN5;DGKB;KMT2C;TCF12;PDE4DIP;RORA;MITF;ESRRG;ENOX1;CDH6;BRIP1;CDH4;PAX7;DMD;CNTN4;SOX6;CDH18;SOX5;DDR2*
FBXO41	22/299	2.054088790619987E-6	*RAP1GAP2;CADPS2;CNTNAP2;NTRK2;NEGR1;IQSEC1;PDE4D;NRXN1;DNMT3A;CADPS;CELF4;ANK2;RORA;GRIK2;ANK1;DLG2;NBEA;PPP2R2C;RIMBP2;CTNNA2;SCN2A;DDR2*
MLLT3	22/299	2.054088790619987E-6	*CADPS2;CERS6;AUTS2;MAST4;PDE4D;ZBTB20;PDE4DIP;ANK2;RORA;LMO7;ANK1;HDAC9;BACH2;PTPRD;PLCB4;NBEA;TRPS1;TNIK;SOX6;UTRN;TOX;DDR2*
NFIA	22/299	2.054088790619987E-6	*CADPS2;NTRK2;AUTS2;PDE4D;PCDH7;ATP2B4;PTPRM;ZBTB20;PDE4DIP;ANK2;RORA;LMO7;ANK1;BACH2;PTPRD;NBEA;TRPS1;DMD;SOX6;UTRN;SOX5;DDR2*
ONECUT2	22/299	2.054088790619987E-6	*ROBO2;CADPS2;CNTNAP2;NTRK2;RBFOX1;CASZ1;DCC;NRXN1;PCDH7;CELF4;NRXN3;ZBTB20;RORA;ESRRG;ANK1;BACH2;PTPRD;NBEA;PPP2R2C;KIF26B;SOX6;DDR2*
PBX1	22/299	2.054088790619987E-6	*MAGI1;CADPS2;NTRK2;AUTS2;PDE4D;PCDH7;ATP2B4;PTPRM;ZBTB20;ANK2;RORA;ANK1;HDAC9;PTPRD;NBEA;TRPS1;TNIK;SLIT2;SOX6;UTRN;SOX5;DDR2*
PRDM2	22/299	2.054088790619987E-6	*NCOA1;MAGI1;AUTS2;MAST4;IQSEC1;KMT2C;PDE4D;TCF12;DNMT3A;ATP2B4;PRRC2C;ZBTB20;PDE4DIP;RORA;SSH2;ANK1;LYST;BACH2;ETV6;TRPS1;UTRN;DDR2*
ZNF214	22/299	2.054088790619987E-6	*CADPS2;NTRK2;DNAH7;PDE4D;DNAH5;NRXN1;FAM13A;NRXN3;RORA;LMO7;NRG1;DLG2;NBEA;ERBB4;KIF26B;TRPS1;FAT3;TNIK;SLIT2;TOX;BMPR1B;DDR2*
ZNF334	22/299	2.054088790619987E-6	*CADPS2;NTRK2;AUTS2;PDE4D;NRXN1;PCDH7;CADPS;NRXN3;ANK2;RORA;ESRRG;PPP1R9A;PTPRD;DPP6;NBEA;KIF26B;DNER;DMD;SLIT2;TOX;FREM2;DDR2*
ZNF391	22/299	2.054088790619987E-6	*CADPS2;PARM1;AUTS2;NEGR1;PDE4D;PCDH7;ADAM22;ANK2;RORA;LMO7;PPP1R9A;HDAC9;ARHGAP24;PTPRD;DLG2;NBEA;TRPS1;TNIK;SLIT2;SCN2A;SOX5;DDR2*
ZNF713	22/299	2.054088790619987E-6	*CADPS2;AUTS2;PDE4D;TCF12;ZBTB20;SMAD9;ANK2;RORA;ESRRG;GRIK2;HDAC9;BACH2;BRIP1;NBEA;DMD;CNTN4;SOX6;TOX;GPC6;FREM2;SOX5;DDR2*
ZNF827	22/299	2.054088790619987E-6	*MAGI1;CADPS2;AUTS2;MAST4;KMT2C;PDE4D;DNMT3A;ATP2B4;ZBTB20;RORA;PPP1R9A;HDAC9*

			;BACH2;ETV6;DOCK10;NBEA;KIF26B;TRPS1;TNIK;UTRN;TOX;DDR2
FAM171B	21/299	7.765824510322848E-6	*ROBO2;CADPS2;NTRK2;NEGR1;NRXN1;PCDH7;ATP2B4;ANK2;RORA;ANK1;HDAC9;PTPRG;PTPRD;NBEA;NAV3;TRPS1;TNIK;SLIT2;GPC6;EDIL3;DDR2*
FEZF1	21/299	7.765824510322848E-6	*TRIM71;CADPS2;CNTNAP2;NTRK2;RBFOX1;CASZ1;TENM2;NRXN1;PCDH7;CADPS;PAX3;ESRRG;GRIK2;ANK1;DAB1;PAX7;ERBB4;CTNNA2;SLIT2;SOX5;DDR2*
FOXD4L6	21/299	7.765824510322848E-6	*PCSK1;NTRK2;RBFOX1;NEGR1;PDE4D;NRXN1;PCDH7;PDE4DIP;ESRRG;NRG1;GRIK2;PTPRD;GRM3;NELL1;DPP6;RALYL;PAX7;TRPS1;EDIL3;CDH18;SOX5*
GLIS1	21/299	7.765824510322848E-6	*CADPS2;CASZ1;AUTS2;NEGR1;PCDH7;RORA;ESRRG;NRG1;ANK1;BACH2;ENOX1;PTPRD;CDH4;DAB1;KIF26B;CDH13;SLIT2;SOX6;GPC6;SOX5;DDR2*
GLIS3	21/299	7.765824510322848E-6	*CADPS2;NTRK2;AUTS2;PDE4D;PCDH7;PTPRM;ZBTB20;RORA;LMO7;NRG1;ANK1;HDAC9;BACH2;PTPRD;KIF26B;TRPS1;CDH13;TNIK;SLIT2;GPC6;DDR2*
HMBOX1	21/299	7.765824510322848E-6	*NCOA1;MAST4;KMT2C;PDE4D;TCF12;ATP2B4;PRRC2C;ZBTB20;PDE4DIP;ANK2;RORA;LMO7;SSH2;LYST;BACH2;NBEA;TRPS1;TNIK;SOX6;UTRN;DDR2*
LMX1B	21/299	7.765824510322848E-6	*CADPS2;CNTNAP2;NTRK2;RBFOX1;CASZ1;AUTS2;CELF4;PAX3;RORA;ESRRG;NRG1;ANK1;BACH2;CDH4;DPP6;PAX7;ERBB4;KIF26B;CDH13;SOX5;DDR2*
PAX6	21/299	7.765824510322848E-6	*CADPS2;NTRK2;NRXN1;PAX3;ZBTB20;ANK2;RORA;MITF;ESRRG;NRG1;ANK1;HDAC9;PAX7;ERBB4;TRPS1;DNER;SLIT2;SOX6;BMPR1B;SOX5;DDR2*
PRDM13	21/299	7.765824510322848E-6	*RYR2;NTRK2;RBFOX1;CASZ1;DCC;PDE4D;NRXN1;CELF4;NRXN3;PAX3;RORA;ESRRG;NRG1;GRIK2;ANK1;CDH4;DPP6;PPP2R2C;PAX7;SLIT2;RNF220*
ZBTB7C	21/299	7.765824510322848E-6	*CADPS2;NTRK2;CASZ1;TENM2;PARM1;AUTS2;PDE4D;RORA;LMO7;NRG1;ANK1;PTPRD;CDH4;PLCB4;MYO5B;KIF26B;CDH13;SLIT2;SOX6;SOX5;DDR2*
ZNF233	21/299	7.765824510322848E-6	*ROBO2;CADPS2;CNTNAP2;ATRNL1;KMT2C;PDE4D;DNAH5;PCDH7;FAM13A;NRXN3;ZBTB20;PDE4DIP;RORA;LMO7;ESRRG;GRIK2;ARHGAP24;PTPRD;PLCB4;NBEA;DMD*
ZNF287	21/299	7.765824510322848E-6	*CADPS2;AUTS2;NEGR1;PDE4D;PCDH7;ATP2B4;ZBTB20;PDE4DIP;ANK2;RORA;LMO7;GRIK2;LYST;BACH2;PLCB4;SGCD;NBEA;TRPS1;TOX;SOX5;DDR2*
ASCL4	20/299	2.6571888532941092E-5	*CADPS2;NTRK2;RBFOX1;CASZ1;AUTS2;DNAH8;PDE4D;NRXN1;PCDH7;NRXN3;PAX3;ESRRG;ANK1;NELL1;DPP6;DLG2;DAB1;PAX7;KIF26B;DDR2*
BAZ2B	20/299	2.6571888532941092E-5	*NCOA1;CADPS2;AUTS2;ANKRD44;KMT2C;PDE4D;TCF12;PRRC2C;ZBTB20;RORA;SSH2;PPP1R9A;LYST;PTPRD;STAG2;NBEA;TRPS1;ZNF638;UTRN;DDR2*
BNC2	20/299	2.6571888532941092E-5	*AUTS2;NEGR1;PDE4D;PCDH7;ZBTB20;ANK2;RORA;MITF;ARHGAP24;PTPRD;SGCD;NAV3;KIF26*

			B;TRPS1;CDH13;SLIT2;GPC6;EDIL3;SOX5;DDR2
CUX1	20/299	2.6571888532941092E-5	*MAGI1;DYNC1H1;AUTS2;IQSEC1;KMT2C;PDE4D;TCF12;DNMT3A;ATP2B4;PTPRM;ZBTB20;PDE4DIP;RORA;ANK1;BACH2;ETV6;PTPRD;TRPS1;UTRN;DDR2*
FOXP1	20/299	2.6571888532941092E-5	*MAGI1;AUTS2;MAST4;KMT2C;PDE4D;TCF12;DNMT3A;PTPRM;ZBTB20;ANK2;RORA;LMO7;ANK1;HDAC9;BACH2;ETV6;TRPS1;UTRN;SOX5;DDR2*
HIVEP3	20/299	2.6571888532941092E-5	*RAP1GAP2;CASZ1;AUTS2;MAST4;PDE4D;PCDH7;DNMT3A;ATP2B4;ZBTB20;PDE4DIP;RORA;ANK1;HDAC9;BACH2;ETV6;KIF26B;TRPS1;TNIK;SOX5;DDR2*
IRX6	20/299	2.6571888532941092E-5	*RYR2;CADPS2;NTRK2;RBFOX1;PARM1;PCDH7;RORA;ESRRG;ANK1;CDH4;DAB1;PAX7;KIF26B;DNER;GPC3;CDH13;SLIT2;SOX6;CSMD1;DDR2*
LCORL	20/299	2.6571888532941092E-5	*CADPS2;CERS6;AUTS2;NEGR1;KMT2C;PDE4D;NRXN1;TCF12;ZBTB20;RORA;LMO7;HDAC9;BACH2;PTPRD;DLG2;NBEA;TRPS1;SOX6;UTRN;DDR2*
NEUROD1	20/299	2.6571888532941092E-5	*ROBO2;PCSK1;CADPS2;CNTNAP2;NTRK2;RBFOX1;NEGR1;NRXN1;CADPS;CELF4;ESRRG;GRIK2;NOL4;DPP6;RALYL;FGF14;DAB1;NBEA;DNER;RIMBP2*
NR2E1	20/299	2.6571888532941092E-5	*CADPS2;NTRK2;RBFOX1;DCC;NRXN3;PAX3;RORA;ESRRG;GRIK2;ANK1;NOL4;CDH4;DAB1;PAX7;CTNNA2;SLIT2;SOX6;BMPR1B;SOX5;DDR2*
PAX2	20/299	2.6571888532941092E-5	*CADPS2;CNTNAP2;NTRK2;RBFOX1;CASZ1;PCDH7;CELF4;NRXN3;PAX3;RORA;ESRRG;NRG1;ANK1;BACH2;CDH4;DPP6;PAX7;KIF26B;SOX6;DDR2*
PGR	20/299	2.6571888532941092E-5	*ROBO2;RYR2;CADPS2;CNTNAP2;NTRK2;NEGR1;NRXN1;PCDH7;RORA;MITF;ESRRG;HDAC9;NAV3;ERBB4;TRPS1;DMD;CNTN4;SLIT2;SOX6;DDR2*
PRDM6	20/299	2.6571888532941092E-5	*ROBO2;RYR2;CADPS2;NTRK2;CASZ1;PARM1;PCDH7;RORA;ESRRG;ANK1;PTPRD;KIF26B;PDE3A;FAT3;SLIT2;SOX6;GPC6;EDIL3;SOX5;DDR2*
RBM20	20/299	2.6571888532941092E-5	*RYR2;CADPS2;NTRK2;CASZ1;PARM1;PDE4D;PCDH7;RFTN1;PDE4DIP;ANK2;RORA;LMO7;ESRRG;ANK1;BACH2;KIF26B;CDH13;FAT3;SOX6;DDR2*
RERE	20/299	2.6571888532941092E-5	*MAGI1;DYNC1H1;AUTS2;MAST4;IQSEC1;KMT2C;PDE4D;TCF12;DNMT3A;ATP2B4;ZBTB20;PDE4DIP;ANK2;RORA;LMO7;ANK1;BACH2;UTRN;LPIN1;DDR2*
RFX6	20/299	2.6571888532941092E-5	*ROBO2;PCSK1;CADPS2;CNTNAP2;NTRK2;TENM2;PARM1;KCNB2;NRXN1;PCDH7;CADPS;ESRRG;NOL4;CDH6;PTPRD;KIF26B;GPC3;RIMBP2;BMPR1B;FREM2*
ZNF236	20/299	2.6571888532941092E-5	*NCOA1;MAGI1;DENND1B;DYNC1H1;CADPS2;KDM4B;AUTS2;KMT2C;TCF12;PRRC2C;PDE4DIP;RORA;SSH2;BACH2;PTPRG;MKLN1;NBEA;TRPS1;UTRN;DDR2*
ZNF608	20/299	2.6571888532941092E-5	*MAGI1;CADPS2;AUTS2;MAST4;PDE4D;PCDH7;ZBTB20;PDE4DIP;RORA;HDAC9;BACH2;ARHGAP24;NBEA;KIF26B;TRPS1;TNIK;SLIT2;UTRN;TOX;DDR2*
ZNF711	20/299	2.6571888532941092E-5	*ROBO2;CADPS2;CNTNAP2;NTRK2;AUTS2;PCDH7;RORA;BACH2;PTPRD;PLCB4;NBEA;TRPS1;GPC3;DMD;SLIT2;SOX6;TOX;FGF13;EDIL3;DDR2*
ZNF781	20/299	2.6571888532	*CADPS2;NTRK2;NEGR1;PDE4D;NRXN1;PCDH7;N*

		941092E-5	*RXN3;ANK2;RORA;ESRRG;HDAC9;BACH2;NBEA;DMD;CNTN4;SLIT2;SOX6;ST6GALNAC3;SOX5;DDR2*
ZSCAN20	20/299	2.6571888532941092E-5	*DENND1B;CADPS2;CASZ1;AUTS2;KMT2C;PDE4D;ATP2B4;PDE4DIP;LMO7;ANK1;LYST;HDAC9;BACH2;ARHGAP24;DOCK10;KIF26B;TRPS1;LPIN1;SOX5;DDR2*
BACH2	19/299	8.622431470174563E-5	*CADPS2;AUTS2;PDE4D;PLCL1;ZBTB20;ANK2;RORA;ANK1;HDAC9;PTPRD;KIF26B;TRPS1;TNIK;SLIT2;SOX6;UTRN;TOX;SOX5;DDR2*
DMRTB1	19/299	8.622431470174563E-5	*RYR2;CADPS2;NTRK2;CASZ1;CADPS;ESRRG;ANK1;CDH4;DPP6;DAB1;PAX7;ERBB4;KIF26B;GPC3;SOX6;BMPR1B;SOX5;RNF220;DDR2*
FERD3L	19/299	8.622431470174563E-5	*ROBO2;RYR2;CADPS2;RBFOX1;PCDH7;CELF4;PAX3;ESRRG;NRG1;GRIK2;ADARB2;ANK1;NELL1;DPP6;LRFN5;NRG3;PAX7;SLIT2;CSMD1*
FOXN3	19/299	8.622431470174563E-5	*MAGI1;AUTS2;MAST4;KMT2C;PDE4D;TCF12;ATP2B4;ZBTB20;ANK2;RORA;ANK1;BACH2;PTPRG;ETV6;PTPRD;TRPS1;UTRN;LPIN1;DDR2*
HNF4G	19/299	8.622431470174563E-5	*ROBO2;CADPS2;PCDH7;ZBTB20;RORA;MITF;LMO7;ESRRG;HDAC9;CDH6;PTPRD;TRPS1;SLIT2;SOX6;TOX;BMPR1B;EDIL3;SOX5;DDR2*
IKZF2	19/299	8.622431470174563E-5	*CADPS2;MAST4;ANKRD44;PDE4D;PCDH7;ATP2B4;ZBTB20;RORA;LMO7;ANK1;HDAC9;BACH2;DOCK10;TRPS1;TNIK;SOX6;UTRN;TOX;DDR2*
ISX	19/299	8.622431470174563E-5	*DPP10;PCSK1;CADPS2;CNTNAP2;AUTS2;DNAH5;NRXN1;RORA;LMO7;ESRRG;PTPRD;MGAT4C;ACE2;DLG2;MYO5B;KIF26B;SOX6;EDIL3;TRPM3*
LHX4	19/299	8.622431470174563E-5	*RAP1GAP2;DPP10;CADPS2;CASZ1;NEGR1;NRXN1;ATP2B4;PAX3;RORA;ESRRG;ANK1;BACH2;CDH4;DLG2;PAX7;ERBB4;KIF26B;DNER;DDR2*
LHX8	19/299	8.622431470174563E-5	*CADPS2;NTRK2;NEGR1;DCC;PAX3;ESRRG;GRIK2;ANK1;CDH6;CDH4;DAB1;PAX7;KIF26B;CDH13;CNTN4;SLIT2;SOX6;BRINP3;DDR2*
LHX9	19/299	8.622431470174563E-5	*ROBO2;CADPS2;NTRK2;AUTS2;NEGR1;PCDH7;PAX3;RORA;ESRRG;NRG1;GRIK2;ANK1;PAX7;KIF26B;CDH13;SLIT2;SOX6;SOX5;DDR2*
POU3F3	19/299	8.622431470174563E-5	*CADPS2;NTRK2;AUTS2;DCC;NRXN1;PCDH7;PAX3;ESRRG;GRIK2;CDH4;DPP6;DAB1;PAX7;ERBB4;DNER;SLIT2;SOX6;SOX5;DDR2*
RAPGEF5	19/299	8.622431470174563E-5	*MAGI1;CADPS2;NTRK2;PARM1;AUTS2;PDE4D;PCDH7;PTPRM;ZBTB20;RORA;LMO7;ANK1;HDAC9;DOCK10;PTPRD;PLCB4;TNIK;TOX;DDR2*
SHOX	19/299	8.622431470174563E-5	*RYR2;NTRK2;RBFOX1;NRXN1;PCDH7;NRXN3;PAX3;ADCY2;MITF;NRG1;GRIK2;CDH4;DPP6;GRM7;PAX7;CDH13;CNTN4;CSMD1;DDR2*
TBX20	19/299	8.622431470174563E-5	*ROBO2;RYR2;CADPS2;NTRK2;RBFOX1;CASZ1;PCDH7;RORA;ESRRG;NRG1;ANK1;PTPRD;KIF26B;PDE3A;CDH13;SLIT2;SOX6;SOX5;DDR2*
TRERF1	19/299	8.622431470174563E-5	*RAP1GAP2;AUTS2;MAST4;PDE4D;PCDH7;DNMT3A;ATP2B4;RORA;LMO7;ANK1;HDAC9;ETV6;DOCK10;TRPS1;TNIK;UTRN;TOX;LPIN1;DDR2*
TSHZ3	19/299	8.622431470174563E-5	*AUTS2;PCDH7;ATP2B4;PTPRM;RORA;NRG1;ANK1;ARHGAP24;PTPRD;NAV3;KIF26B;TRPS1;CDH13;SLIT2;TOX;GPC6;EDIL3;SOX5;DDR2*
ZMAT1	19/299	8.622431470174563E-5	*CADPS2;PARM1;NEGR1;PDE4D;ZBTB20;RORA;PPP1R9A;ANK1;HDAC9;BACH2;DOCK10;STAG2;NBEA;TRPS1;GPC3;DMD;TNIK;TOX;DDR2*
ZNF154	19/299	8.622431470174563E-5	*CADPS2;NTRK2;PARM1;NEGR1;SGIP1;PDE4D;PCDH7;ZBTB20;RORA;LMO7;NRG1;PPP1R9A;BAC*

			H2;DOCK10;TRPS1;FAT3;DMD;TOX;DDR2
ZNF365	19/299	8.6224314701 74563E-5	*CADPS2;CNTNAP2;NTRK2;NEGR1;PDE4D;PCDH7 ;NRXN3;RORA;NRG1;HDAC9;BACH2;DOCK10;DL G2;NBEA;NAV3;CDH13;SCN2A;DDR2;JAZF1*
ZNF423	19/299	8.6224314701 74563E-5	*ROBO2;CADPS2;NTRK2;AUTS2;PDE4D;NRXN1;D NMT3A;PTPRM;ANK2;RORA;BACH2;PTPRD;CDH4 ;NBEA;KIF26B;CDH13;SLIT2;TOX;DDR2*
ZNF618	19/299	8.6224314701 74563E-5	*MAGI1;CADPS2;AUTS2;PDE4D;PCDH7;DNMT3A; ATP2B4;ZBTB20;PPP1R9A;HDAC9;BACH2;DOCK 10;NBEA;KIF26B;TRPS1;TNIK;SLIT2;UTRN;D DR2*
ZNF677	19/299	8.6224314701 74563E-5	*RYR2;CADPS2;NEGR1;NRXN1;PCDH7;NRXN3;ZB TB20;ANK2;ADCY2;RORA;NRG1;BACH2;CDH4;N BEA;DMD;SLIT2;UTRN;SOX5;DDR2*
ZNF704	19/299	8.6224314701 74563E-5	*MAGI1;CADPS2;NTRK2;AUTS2;PDE4D;PCDH7;Z BTB20;RORA;MITF;PPP1R9A;HDAC9;BACH2;PT PRD;NBEA;TRPS1;DMD;SLIT2;SOX6;DDR2*
ZNF774	19/299	8.6224314701 74563E-5	*CADPS2;PATJ;KDM4B;MAST4;FAM13A;DNMT3A; ATP2B4;PTPRM;ZBTB20;PDE4DIP;ANK2;RORA; LYST;NBEA;DMD;TNIK;UTRN;SOX5;DDR2*
ARNT2	18/299	2.7804844539 018115E-4	*RAP1GAP2;CADPS2;CNTNAP2;NTRK2;AUTS2;PC DH7;ANK2;RORA;NRG1;ANK1;PTPRD;NBEA;PPP 2R2C;TNIK;SLIT2;TOX;SOX5;DDR2*
ATOH1	18/299	2.7804844539 018115E-4	*ROBO2;DPP10;PCSK1;CADPS2;NTRK2;RBFOX1; CASZ1;PCDH7;NRXN3;ESRRG;HDAC9;CDH4;PAX 7;ERBB4;SLIT2;TOX;BRINP3;DDR2*
BARHL2	18/299	2.7804844539 018115E-4	*CADPS2;NTRK2;RBFOX1;CASZ1;CELF4;PAX3;R ORA;ESRRG;ANK1;NELL1;CDH4;DPP6;DAB1;PA X7;ERBB4;KIF26B;SOX6;SOX5*
CHD7	18/299	2.7804844539 018115E-4	*DYNC1H1;CASZ1;AUTS2;MAST4;KMT2C;PDE4D; TCF12;DNMT3A;PRRC2C;ZBTB20;RORA;ANK1;B ACH2;ETV6;DOCK10;TRPS1;UTRN;DDR2*
CHD9	18/299	2.7804844539 018115E-4	*NCOA1;DYNC1H1;CERS6;KMT2C;PDE4D;TCF12; ATP2B4;PRRC2C;ZBTB20;RORA;LYST;MKLN1;S TAG2;TRPS1;ZNF638;TTC3;UTRN;DDR2*
DRGX	18/299	2.7804844539 018115E-4	*RYR2;NTRK2;RBFOX1;CASZ1;NRXN1;CELF4;NR XN3;PAX3;RORA;ESRRG;ANK1;NELL1;CDH4;DP P6;PAX7;CDH13;SOX6;SOX5*
NR2E3	18/299	2.7804844539 018115E-4	*CADPS2;NTRK2;CASZ1;AUTS2;NRXN3;PAX3;RO RA;MITF;ESRRG;NRG1;GRIK2;BACH2;KIF26B; TRPS1;SOX6;TOX;SOX5;DDR2*
PKHD1L1	18/299	2.7804844539 018115E-4	*RYR2;CADPS2;NRXN1;PCDH15;ATP2B4;ANK2;A NK1;DOCK10;PTPRD;DLG2;ERBB4;KIF26B;DMD ;SLIT2;SOX6;CSMD1;FREM2;DDR2*
RGS9	18/299	2.7804844539 018115E-4	*RAP1GAP2;PCSK1;CADPS2;NTRK2;NEGR1;DGKB ;PCDH7;ATP2B4;RORA;GRIK2;ANK1;CDH4;DLG 2;MYO5B;CDH13;TOX;SOX5;DDR2*
RREB1	18/299	2.7804844539 018115E-4	*CASZ1;AUTS2;IQSEC1;KMT2C;PDE4D;TCF12;D NMT3A;ATP2B4;ZBTB20;PDE4DIP;RORA;LMO7; ANK1;BACH2;ETV6;TRPS1;UTRN;DDR2*
SALL1	18/299	2.7804844539 018115E-4	*CADPS2;NTRK2;AUTS2;GLDC;PCDH7;ANK2;ESR RG;NRG1;ANK1;PTPRD;KIF26B;TRPS1;SLIT2; SOX6;TOX;GPC6;SOX5;DDR2*
SOX1	18/299	2.7804844539 018115E-4	*NTRK2;RBFOX1;CASZ1;DCC;NRXN1;PCDH7;CEL F4;NRXN3;PAX3;RORA;ESRRG;CDH4;DPP6;DAB 1;PAX7;SLIT2;SOX6;SOX5*
TIGD4	18/299	2.7804844539 018115E-4	*CHODL;CADPS2;CNTNAP2;PDE4D;PLCL1;ULK4; ADAM22;ANK2;ESRRG;PPP1R9A;ANK1;HDAC9;R ASGEF1B;DMD;CTNNA3;SOX6;BMPR1B;DDR2*
ZNF382	18/299	2.7804844539	*ROBO2;CADPS2;CNTNAP2;PARM1;PLCL1;NRXN3*

		018115E-4	*;KIAA1549L;ZBTB20;RORA;GRIK2;HDAC9;CDH6;NBEA;NAV3;DMD;TNIK;TOX;DDR2*
ZNF516	18/299	2.7804844539018115E-4	*RAP1GAP2;KDM4B;AUTS2;IQSEC1;KMT2C;PDE4D;ATP2B4;ZBTB20;PDE4DIP;RORA;HDAC9;BACH2;NBEA;TRPS1;TNIK;UTRN;LPIN1;DDR2*
ZNF626	18/299	2.7804844539018115E-4	*MAGI1;CADPS2;CNTNAP2;NEGR1;PDE4D;PTPRM;ZBTB20;PDE4DIP;RORA;PPP1R9A;HDAC9;NBEA;TRPS1;DMD;SLIT2;UTRN;EDIL3;DDR2*
ZNF793	18/299	2.7804844539018115E-4	*RAP1GAP2;RYR2;CADPS2;PATJ;AUTS2;NEGR1;PDE4D;PCDH7;RORA;PPP1R9A;ANO5;HDAC9;BACH2;PTPRD;NBEA;DMD;CNTN4;DDR2*
DBX1	17/299	8.193603236600101E-4	*NTRK2;RBFOX1;CASZ1;DCC;PCDH7;CELF4;PAX3;ESRRG;ANK1;NELL1;CDH4;DPP6;PAX7;ERBB4;KIF26B;SLIT2;SOX6*
DMRT3	17/299	8.193603236600101E-4	*CADPS2;CNTNAP2;NTRK2;PCDH7;PAX3;RORA;ESRRG;NRG1;ANK1;CDH6;NELL1;CDH4;PAX7;SLIT2;SOX6;SOX5;DDR2*
DMRTA2	17/299	8.193603236600101E-4	*CADPS2;NTRK2;RBFOX1;CASZ1;PCDH7;CELF4;PAX3;RORA;ESRRG;NRG1;ANK1;CDH4;DPP6;PAX7;KIF26B;SLIT2;SOX6*
EBF3	17/299	8.193603236600101E-4	*CADPS2;NTRK2;CASZ1;AUTS2;PCDH7;PTPRM;ESRRG;ANK1;BACH2;PTPRD;KIF26B;GPC3;CDH13;SLIT2;SOX6;GPC6;DDR2*
FOXI2	17/299	8.193603236600101E-4	*RYR2;CNTNAP2;RBFOX1;NRXN1;PCDH7;CELF4;NRXN3;ESRRG;NRG1;CDH4;DPP6;DAB1;NBEA;GRM7;PAX7;CDH13;CTNNA2*
GLI3	17/299	8.193603236600101E-4	*NTRK2;AUTS2;PCDH7;PTPRM;ANK2;RORA;MITF;BACH2;PTPRG;KIF26B;TRPS1;SLIT2;SOX6;BMPR1B;GPC6;SOX5;DDR2*
GTF2IRD2B	17/299	8.193603236600101E-4	*MAGI1;CADPS2;CASZ1;ARHGEF37;AUTS2;MAST4;KMT2C;PDE4D;PLCL1;PTPRM;ZBTB20;PDE4DIP;RORA;LMO7;LYST;NBEA;DDR2*
HSF5	17/299	8.193603236600101E-4	*CADPS2;DCC;NRXN3;RORA;LMO7;ESRRG;ANK1;HDAC9;BACH2;SH3RF3;CDH13;GPC5;TOX;DOCK2;FREM2;DDR2;JAZF1*
INSM1	17/299	8.193603236600101E-4	*ROBO2;PCSK1;CADPS2;NTRK2;AUTS2;NRXN1;CADPS;CELF4;ESRRG;ANK1;NOL4;BACH2;CDH4;DNER;RIMBP2;TOX;DDR2*
JAZF1	17/299	8.193603236600101E-4	*RAP1GAP2;CADPS2;NEGR1;PDE4D;PLCL1;PCDH7;ATP2B4;ZBTB20;ANK2;RORA;ANK1;HDAC9;BACH2;DOCK10;TOX;GPC6;DDR2*
MKRN3	17/299	8.193603236600101E-4	*CADPS2;CNTNAP2;NRXN1;CADPS;RORA;GRIK2;HDAC9;BACH2;NAV3;ERBB4;DNER;SLIT2;TOX;FGF13;EDIL3;ASTN1;DDR2*
NFIB	17/299	8.193603236600101E-4	*MAGI1;CADPS2;NTRK2;AUTS2;PDE4D;PTPRM;ZBTB20;ANK2;RORA;LMO7;PTPRD;NBEA;TRPS1;SLIT2;UTRN;SOX5;DDR2*
ONECUT1	17/299	8.193603236600101E-4	*CADPS2;NTRK2;CASZ1;NRXN1;PAX3;RORA;ESRRG;NRG1;ANK1;CDH6;CDH4;PAX7;ERBB4;KIF26B;SOX6;SOX5;DDR2*
PHF21A	17/299	8.193603236600101E-4	*NCOA1;KDM4B;AUTS2;KMT2C;PDE4D;TCF12;DNMT3A;ATP2B4;PRRC2C;PTPRM;ZBTB20;ANK2;RORA;ETV6;TRPS1;UTRN;DDR2*
PRDM15	17/299	8.193603236600101E-4	*RAP1GAP2;CNTNAP2;KDM4B;AUTS2;MAST4;IQSEC1;KMT2C;PDE4D;DNMT3A;PDE4DIP;SSH2;ANK1;BACH2;ETV6;TRPS1;LPIN1;DDR2*
SCRT2	17/299	8.193603236600101E-4	*ROBO2;RBFOX1;CASZ1;AUTS2;DCC;NRXN1;CADPS;CELF4;NRXN3;ADARB2;ANK1;CDH4;DPP6;DAB1;KIF26B;DNER;DSCAML1*
SIX3	17/299	8.1936032366	*CADPS2;NTRK2;CASZ1;NRXN1;PCDH7;CELF4;P*

		00101E-4	*AX3;ZBTB20;RORA;ESRRG;ANK1;CDH6;CDH4;DPP6;PAX7;RGS9;DDR2*
TRPS1	17/299	8.193603236600101E-4	*CADPS2;AUTS2;PDE4D;PCDH7;ATP2B4;ZBTB20;RORA;HDAC9;DOCK10;PTPRD;NBEA;TNIK;SLIT2;UTRN;TOX;SOX5;DDR2*
ZNF343	17/299	8.193603236600101E-4	*MAGI1;RAP1GAP2;CADPS2;NBAS;PDE4D;TCF12;DNMT3A;ATP2B4;PDE4DIP;HDAC9;BACH2;DOCK10;PLCB4;TTC3;MACROD2;TNIK;DDR2*
ZNF415	17/299	8.193603236600101E-4	*CADPS2;PARM1;PDE4D;ZBTB20;PDE4DIP;ANK2;RORA;PPP1R9A;HDAC9;PLCB4;NBEA;DNER;DMD;SLIT2;TOX;EDIL3;DDR2*
ZNF43	17/299	8.193603236600101E-4	*CADPS2;NEGR1;DNMT3A;PTPRM;ZBTB20;PDE4DIP;RORA;PPP1R9A;HDAC9;PLCB4;NBEA;TRPS1;DMD;SLIT2;TOX;EDIL3;DDR2*
ZNF546	17/299	8.193603236600101E-4	*ROBO2;DENND1B;CADPS2;NEGR1;PDE4D;FAM13A;ZBTB20;HDAC9;BACH2;DOCK10;PLCB4;NBEA;TMEM117;TRPS1;SOX6;SOX5;DDR2*
ZNF596	17/299	8.193603236600101E-4	*RYR2;CADPS2;MAST4;NEGR1;PDE4D;ZBTB20;PDE4DIP;RORA;LMO7;NRG1;HDAC9;BACH2;ARHGAP24;NBEA;TRPS1;DMD;DDR2*
ZNF599	17/299	8.193603236600101E-4	*ROBO2;CADPS2;CASZ1;AUTS2;ANKRD44;PDE4D;NRXN3;ZBTB20;PDE4DIP;NRG1;ANK1;NBEA;KIF26B;DNER;FAM20C;TOX;DDR2*
ZNF645	17/299	8.193603236600101E-4	*NCOA1;DPP10;NTRK2;RBFOX1;ZBTB20;DACH2;PHEX;TMEM164;LRMDA;SCEL;STAG2;PAX7;ERBB4;CDH13;DMD;MACROD2;DDR2*
ZNF681	17/299	8.193603236600101E-4	*RYR2;CADPS2;CNTNAP2;AUTS2;GLDC;KMT2C;PDE4D;PDE4DIP;RORA;LMO7;HDAC9;BRIP1;NBEA;FAT3;TOX;FREM2;DDR2*
ATOH7	16/299	0.002275772749748585	*PCSK1;CADPS2;NTRK2;NEGR1;DGKB;SGIP1;ATP2B4;ESRRG;ANK1;DLG2;DAB1;KIF26B;CTNNA3;SOX6;RGS9;DDR2*
ATXN7	16/299	0.002275772749748585	*NCOA1;AUTS2;KMT2C;PDE4D;TCF12;PRRC2C;ZBTB20;RORA;SSH2;LYST;BACH2;ETV6;TRPS1;TNIK;UTRN;DDR2*
CASZ1	16/299	0.002275772749748585	*RAP1GAP2;CADPS2;AUTS2;MAST4;PDE4D;ATP2B4;ZBTB20;KAZN;PDE4DIP;RORA;LMO7;ESRRG;ANK1;BACH2;KIF26B;DDR2*
DMRTA1	16/299	0.002275772749748585	*CADPS2;CNTNAP2;NTRK2;NEGR1;PCDH7;ESRRG;HDAC9;CDH6;CDH4;SLIT2;SOX6;TOX;BMPR1B;GPC6;EDIL3;DDR2*
DZIP1	16/299	0.002275772749748585	*CADPS2;NTRK2;NEGR1;PCDH7;ATP2B4;ANK2;ANK1;PTPRD;NBEA;TRPS1;DMD;SLIT2;GPC6;MPDZ;EDIL3;DDR2*
EBF1	16/299	0.002275772749748585	*NTRK2;AUTS2;RFTN1;PTPRM;ZBTB20;ANK2;RORA;ANK1;HDAC9;BACH2;ARHGAP24;CDH13;SLIT2;TOX;GPC6;DDR2*
FOXD4L3	16/299	0.002275772749748585	*RYR2;RBFOX1;CASZ1;DSCAM;PCDH7;PAX3;PDE4DIP;ESRRG;NRG1;DPP6;PAX7;ERBB4;KIF26B;SLIT2;TOX;CSMD1*
FOXR2	16/299	0.002275772749748585	*CADPS2;TENM2;CNTN5;GLDC;DCC;NRXN1;DACH2;NOL4;PHEX;FSTL5;DAB1;DNER;GPC3;SLIT2;CDH18;DDR2*
GCM2	16/299	0.002275772749748585	*PCSK1;RYR2;NTRK2;RBFOX1;DCC;NRXN1;CELF4;PAX3;RORA;ESRRG;GRIK2;ANK1;FGF14;PAX7;CSMD1;DDR2*
IRX4	16/299	0.002275772749748585	*RAP1GAP2;CADPS2;CNTNAP2;NTRK2;RBFOX1;CASZ1;TENM2;NRXN1;PCDH7;ESRRG;ANK1;PPP2R2C;PAX7;KIF26B;CDH13;SLIT2*
LHX5	16/299	0.0022757727	*CADPS2;NTRK2;RBFOX1;CASZ1;NRXN1;CELF4;*

		49748585	*NRXN3;PAX3;RORA;ESRRG;ANK1;CDH6;CDH4;DPP6;PAX7;KIF26B*
LIN28B	16/299	0.002275772749748585	*ROBO2;TRIM71;CADPS2;CNTNAP2;GLDC;DNMT3A;NRXN3;RORA;ESRRG;HDAC9;PTPRD;GPC3;TNIK;SLIT2;GPC6;DDR2*
NR5A2	16/299	0.002275772749748585	*CADPS2;NTRK2;CASZ1;PDE4D;RORA;ESRRG;NRG1;ANK1;BACH2;CDH6;ERBB4;KIF26B;TRPS1;SLIT2;SOX6;DDR2*
OLIG3	16/299	0.002275772749748585	*PCSK1;CADPS2;NTRK2;RBFOX1;CASZ1;NRXN1;CELF4;PAX3;ESRRG;BACH2;CDH6;PAX7;ERBB4;KIF26B;SLIT2;DDR2*
PAX4	16/299	0.002275772749748585	*PCSK1;CADPS2;NEGR1;DGKB;DNAH5;CADPS;ADAM22;PAX3;ESRRG;MGAT4C;DLG2;PDE3A;SOX6;RGS9;TRPM3;SOX5*
PAX5	16/299	0.002275772749748585	*CADPS2;CNTNAP2;CASZ1;AUTS2;PAX3;RORA;ESRRG;ANK1;HDAC9;BACH2;PAX7;ERBB4;TOX;DOCK2;SOX5;DDR2*
POU2F1	16/299	0.002275772749748585	*NCOA1;AUTS2;KMT2C;PDE4D;TCF12;ATP2B4;PRRC2C;ZBTB20;RORA;BACH2;ETV6;TRPS1;SOX6;UTRN;SOX5;DDR2*
PTF1A	16/299	0.002275772749748585	*RYR2;CADPS2;RBFOX1;CASZ1;CELF4;PAX3;ESRRG;ANK1;NOL4;NELL1;CDH4;DPP6;FGF14;NBEA;PAX7;ERBB4*
SATB1	16/299	0.002275772749748585	*CADPS2;AUTS2;PDE4D;ATP2B4;ZBTB20;RORA;ANK1;HDAC9;BACH2;DOCK10;NBEA;TRPS1;TNIK;UTRN;TOX;DDR2*
SP8	16/299	0.002275772749748585	*ROBO2;CADPS2;CNTNAP2;PCDH7;CELF4;NRXN3;PAX3;BACH2;CDH6;CDH4;PAX7;KIF26B;SLIT2;SOX6;TOX;DDR2*
TFDP3	16/299	0.002275772749748585	*PCSK1;TENM2;NRXN1;CADPS;ZBTB20;HTR2C;RORA;HDAC9;FSTL5;PLCB4;GPC3;CDH12;DMD;FGF13;CDH18;DDR2*
ZFHX3	16/299	0.002275772749748585	*CASZ1;AUTS2;KMT2C;PDE4D;ATP2B4;ZBTB20;PDE4DIP;RORA;LMO7;ANK1;BACH2;ETV6;KIF26B;TRPS1;UTRN;DDR2*
ZFR2	16/299	0.002275772749748585	*RAP1GAP2;RYR2;CADPS2;CNTNAP2;RBFOX1;NRXN1;DNMT3A;CELF4;ADCY2;RORA;ANK1;CDH4;DPP6;CTNNA2;ASTN1;DDR2*
ZIC1	16/299	0.002275772749748585	*CADPS2;NTRK2;RBFOX1;TENM2;PCDH7;PAX3;ESRRG;GRIK2;CDH6;PTPRD;TRPS1;DNER;CDH13;SLIT2;SOX6;DDR2*
ZNF221	16/299	0.002275772749748585	*RYR2;CADPS2;MAST4;KMT2C;PDE4D;PCDH7;PDE4DIP;RORA;DOCK10;BRIP1;NBEA;NAV3;TRPS1;FREM2;SOX5;DDR2*
ZNF506	16/299	0.002275772749748585	*CADPS2;AUTS2;ANKRD44;PDE4D;ZBTB20;PDE4DIP;RORA;LMO7;LYST;BACH2;NBEA;TRPS1;DMD;TNIK;UTRN;DDR2*
ZNF532	16/299	0.002275772749748585	*AUTS2;KMT2C;PDE4D;TCF12;ATP2B4;PTPRM;ZBTB20;ANK2;RORA;NRG1;HDAC9;BACH2;NBEA;TRPS1;UTRN;DDR2*
ZNF667	16/299	0.002275772749748585	*CADPS2;CNTNAP2;AUTS2;NEGR1;NRXN1;PTPRM;ANK2;RORA;GRIK2;PPP1R9A;HDAC9;BACH2;NBEA;TOX;SOX5;DDR2*
ZNF81	16/299	0.002275772749748585	*NCOA1;DENND1B;CADPS2;KMT2C;TCF12;ATP2B4;PRRC2C;PDE4DIP;ANK2;LYST;MKLN1;STAG2;TRPS1;ZNF638;DMD;UTRN*
ASH1L	15/299	0.005967889783620459	*NCOA1;DYNC1H1;KMT2C;DNMT3A;PRRC2C;ZBTB20;ANK2;LYST;STAG2;NBEA;ZNF638;TTC3;SCN2A;UTRN;DDR2*
ETV1	15/299	0.0059678897	*CADPS2;NTRK2;PCDH7;ANK2;RORA;ANK1;HDAC*

		83620459	*9;PTPRD;PLCB4;NAV3;TRPS1;SLIT2;EDIL3;SOX5;DDR2*
FOXO6	15/299	0.005967889783620459	*RAP1GAP2;CADPS2;CASZ1;AUTS2;RORA;ESRRG;ANK1;BACH2;CDH4;PPP2R2C;MYO5B;KIF26B;CDH13;TOX;DDR2*
GATA4	15/299	0.005967889783620459	*RYR2;CASZ1;PCDH7;PAX3;RORA;MITF;ESRRG;NRG1;ANK1;PTPRD;PAX7;KIF26B;GPC3;SOX6;DDR2*
HELZ	15/299	0.005967889783620459	*NCOA1;DYNC1H1;KMT2C;ATP2B4;PRRC2C;ZBTB20;RORA;SSH2;LYST;MKLN1;STAG2;TRPS1;ZNF638;UTRN;DDR2*
ISL1	15/299	0.005967889783620459	*ROBO2;PCSK1;CADPS2;NTRK2;PARM1;AUTS2;NRXN1;PCDH7;CADPS;CELF4;ESRRG;PTPRD;DNER;SLIT2;DDR2*
KLF8	15/299	0.005967889783620459	*ATP2B4;ANK2;RORA;MITF;ANK1;HDAC9;BACH2;TRPS1;GPC3;DMD;TNIK;SOX6;FGF13;SOX5;DDR2*
MEIS1	15/299	0.005967889783620459	*CADPS2;NTRK2;AUTS2;PDE4D;PCDH7;ATP2B4;ZBTB20;RORA;MITF;PTPRD;TRPS1;SLIT2;SOX6;GPC6;DDR2*
NEUROG2	15/299	0.005967889783620459	*CADPS2;NTRK2;CASZ1;PAX3;ESRRG;ANK1;BACH2;CDH4;PAX7;ERBB4;KIF26B;SLIT2;SOX6;TOX;SOX5*
NRK	15/299	0.005967889783620459	*ROBO2;CADPS2;NTRK2;PARM1;PCDH7;ANK1;PTPRD;GPC3;DMD;SLIT2;FGF13;GPC6;EDIL3;FREM2;DDR2*
OTX2	15/299	0.005967889783620459	*TRIM71;CADPS2;CNTNAP2;NTRK2;RBFOX1;CASZ1;NRXN1;PCDH7;PAX3;ESRRG;DAB1;PAX7;GPC3;SLIT2;TRPM3*
PAX1	15/299	0.005967889783620459	*NTRK2;RBFOX1;CASZ1;PCDH7;CELF4;PAX3;ESRRG;ANK1;CDH4;DPP6;PAX7;ERBB4;KIF26B;SLIT2;DDR2*
PHOX2B	15/299	0.005967889783620459	*NTRK2;RBFOX1;NRXN1;CELF4;PAX3;RORA;ESRRG;GRIK2;DPP6;PAX7;ERBB4;SLIT2;SOX6;SOX5;DDR2*
PRB4	15/299	0.005967889783620459	*MAGI1;PCSK1;RYR2;NTRK2;TENM2;CNTN5;PDE4D;PDE4DIP;ANK2;NRG1;PTPRD;CTNNA3;UTRN;DYNC1I1;DDR2*
SHPRH	15/299	0.005967889783620459	*KMT2C;TCF12;PRRC2C;ZBTB20;LYST;MKLN1;STAG2;NBEA;ZNF638;TTC3;NEK1;DMD;TNIK;UTRN;DDR2*
SSH2	15/299	0.005967889783620459	*IQSEC1;ANKRD44;KMT2C;PDE4D;ATP2B4;PRRC2C;ZBTB20;RORA;LYST;BACH2;ETV6;DOCK10;UTRN;DOCK2;DDR2*
TCF20	15/299	0.005967889783620459	*DYNC1H1;CERS6;AUTS2;MAST4;IQSEC1;KMT2C;DNMT3A;ATP2B4;PRRC2C;ZBTB20;RORA;LMO7;TRPS1;UTRN;DDR2*
TEAD1	15/299	0.005967889783620459	*MAGI1;AUTS2;PDE4D;ATP2B4;ZBTB20;RORA;MITF;LMO7;BACH2;PTPRG;TRPS1;SOX6;UTRN;SOX5;DDR2*
TFDP2	15/299	0.005967889783620459	*PDE4D;TCF12;DNMT3A;ATP2B4;ZBTB20;PDE4DIP;RORA;ANK1;HDAC9;TRPS1;TNIK;SOX6;UTRN;LPIN1;DDR2*
VSX1	15/299	0.005967889783620459	*RYR2;CADPS2;NTRK2;RBFOX1;CASZ1;CELF4;NRXN3;PAX3;ESRRG;GRIK2;ANK1;CDH4;DPP6;PAX7;CDH13*
WNT8B	15/299	0.005967889783620459	*CADPS2;NTRK2;RBFOX1;HTR2C;ESRRG;GRIK2;PPP1R9A;CDH6;CDH4;MYO3B;PAX7;ERBB4;BMPR1B;RGS9;DDR2*
ZFP28	15/299	0.0059678897	*CADPS2;NEGR1;PDE4D;PTPRM;PDE4DIP;RORA;*

		83620459	*LMO7;ANK1;HDAC9;BACH2;ARHGAP24;NBEA;DMD;SLIT2;DDR2*
ZHX3	15/299	0.0059678897 83620459	*NTRK2;MAST4;KMT2C;ATP2B4;ZBTB20;PDE4DIP;ANK2;RORA;LMO7;SSH2;LYST;TRPS1;UTRN;LPIN1;DDR2*
ZNF254	15/299	0.0059678897 83620459	*DENND1B;CADPS2;PDE4D;TCF12;ATP2B4;ZBTB20;PDE4DIP;RORA;PPP1R9A;HDAC9;PLCB4;NBEA;TRPS1;EDIL3;DDR2*
ZNF280D	15/299	0.0059678897 83620459	*CADPS2;KMT2C;TCF12;FAM13A;ATP2B4;ZBTB20;PDE4DIP;RORA;LYST;DOCK10;STAG2;TRPS1;ZNF638;UTRN;DDR2*
ZNF311	15/299	0.0059678897 83620459	*CADPS2;CNTNAP2;CASZ1;TENM2;NRXN3;NRG1;GRIK2;ANK1;CDH4;KIF26B;DNER;CDH13;SLIT2;TOX;DDR2*
ZNF396	15/299	0.0059678897 83620459	*CADPS2;MAST4;DNAH7;NEGR1;PDE4D;DNAH5;PLCL1;PDE4DIP;RORA;MITF;NBEA;TRPS1;DMD;SOX5;DDR2*
ZNF605	15/299	0.0059678897 83620459	*CADPS2;CNTNAP2;NEGR1;DNMT3A;ZBTB20;LMO7;PPP1R9A;DOCK10;PLCB4;NBEA;TRPS1;ZNF638;TTC3;SOX5;DDR2*
ZNF678	15/299	0.0059678897 83620459	*DENND1B;CADPS2;KMT2C;PDE4D;TCF12;ATP2B4;PRRC2C;PDE4DIP;RORA;HDAC9;BRIP1;TRPS1;TNIK;UTRN;DDR2*
ZNF720	15/299	0.0059678897 83620459	*MAGI1;DENND1B;CADPS2;PATJ;ATP2B4;PRRC2C;PDE4DIP;RORA;HDAC9;MKLN1;ZNF638;UTRN;TOX;ZNF267;DDR2*
ZXDC	15/299	0.0059678897 83620459	*DYNC1H1;KDM4B;CERS6;IQSEC1;KMT2C;PDE4D;TCF12;DNMT3A;ZBTB20;PDE4DIP;RORA;ANK1;UTRN;LPIN1;DDR2*
AFF1	14/299	0.0143745180 45711224	*CERS6;MAST4;IQSEC1;KMT2C;ATP2B4;ZBTB20;PDE4DIP;RORA;ANK1;ETV6;TRPS1;UTRN;LPIN1;DDR2*
CREB5	14/299	0.0143745180 45711224	*PDE4D;PCDH7;ZBTB20;ANK2;RORA;MITF;NRG1;HDAC9;BACH2;DOCK10;PTPRD;TRPS1;SOX5;DDR2*
ESR1	14/299	0.0143745180 45711224	*CADPS2;NTRK2;PDE4D;ZBTB20;RORA;MITF;ESRRG;NRG1;ANK1;HDAC9;DLG2;ERBB4;TRPS1;DDR2*
FMNL2	14/299	0.0143745180 45711224	*AUTS2;PDE4D;ATP2B4;PTPRM;ZBTB20;ANK2;RORA;HDAC9;PTPRG;DOCK10;TRPS1;TNIK;UTRN;DDR2*
FOXK1	14/299	0.0143745180 45711224	*DYNC1H1;CASZ1;AUTS2;IQSEC1;PDE4D;DNMT3A;PRRC2C;RORA;LMO7;ANK1;BACH2;ETV6;UTRN;DDR2*
HELT	14/299	0.0143745180 45711224	*CADPS2;NTRK2;CASZ1;DCC;NRXN1;CELF4;PAX3;ESRRG;ANK1;NELL1;DPP6;PAX7;ERBB4;SOX6*
HKR1	14/299	0.0143745180 45711224	*MAGI1;CADPS2;PATJ;CASZ1;KDM4B;KMT2C;NRXN3;ZBTB20;PDE4DIP;RORA;LMO7;NBEA;TNIK;DDR2*
HOXD3	14/299	0.0143745180 45711224	*ROBO2;CADPS2;NTRK2;CASZ1;PCDH7;PAX3;ESRRG;ANK1;DPP6;PAX7;KIF26B;SLIT2;SOX6;DDR2*
LCOR	14/299	0.0143745180 45711224	*DENND1B;KMT2C;PDE4D;ATP2B4;PRRC2C;ZBTB20;RORA;LYST;BACH2;MKLN1;STAG2;TRPS1;UTRN;DDR2*
LEUTX	14/299	0.0143745180 45711224	*DPP10;TENM2;DCC;PDE4D;DNAH5;PAX3;BACH2;DPP6;DAB1;KIF26B;FAT3;CTNNA3;DYNC1I1;DDR2*
MEF2C	14/299	0.0143745180	*CADPS2;PDE4D;PCDH7;RFTN1;ATP2B4;ZBTB20*

		45711224	*;ANK2;RORA;ANK1;HDAC9;DOCK10;TRPS1;SLIT2;DDR2*
MEIS2	14/299	0.0143745180 45711224	*ROBO2;CADPS2;NTRK2;AUTS2;PCDH7;ATP2B4;ZBTB20;ANK2;BACH2;PTPRD;TRPS1;CDH13;SLIT2;DDR2*
MLXIP	14/299	0.0143745180 45711224	*AUTS2;IQSEC1;KMT2C;DNMT3A;ATP2B4;ZBTB20;RORA;SSH2;ANK1;BACH2;ETV6;UTRN;LPIN1;DDR2*
NCOA1	14/299	0.0143745180 45711224	*AUTS2;KMT2C;TCF12;DNMT3A;ATP2B4;ZBTB20;PDE4DIP;ANK2;RORA;BACH2;NBEA;TRPS1;UTRN;DDR2*
PLXNC1	14/299	0.0143745180 45711224	*NTRK2;ANKRD44;RFTN1;ATP2B4;RORA;ANK1;LYST;HDAC9;DOCK10;RGS10;SLIT2;TOX;DOCK2;DDR2*
POU1F1	14/299	0.0143745180 45711224	*CADPS2;NRXN1;PCDH15;PAX3;HTR2C;RORA;MITF;GRIK2;CDH6;DLG2;ERBB4;SOX6;SOX5;DDR2*
POU3F1	14/299	0.0143745180 45711224	*NTRK2;RBFOX1;CASZ1;PCDH7;RORA;NRG1;ANK1;CDH4;DAB1;PPP2R2C;SLIT2;SOX6;SOX5;DDR2*
PRDM7	14/299	0.0143745180 45711224	*CADPS2;CNTNAP2;NTRK2;NEGR1;PCDH7;PDE4DIP;MITF;HDAC9;NAV3;NRG3;KIF26B;SOX6;ATP6V0D2;DDR2*
PRDM8	14/299	0.0143745180 45711224	*CADPS2;TENM2;AUTS2;NEGR1;PDE4D;PCDH7;RORA;LMO7;NRG1;ANK1;BACH2;SLIT2;TOX;DDR2*
PROX2	14/299	0.0143745180 45711224	*MAGI1;PCSK1;CADPS2;NRXN1;ZBTB20;RORA;LMO7;ANK1;BACH2;PTPRD;DLG2;KIF26B;SOX6;DDR2*
SMAD9	14/299	0.0143745180 45711224	*ROBO2;CADPS2;NTRK2;PARM1;PCDH7;ANK2;RORA;LMO7;PLCB4;PDE3A;SLIT2;UTRN;EDIL3;DDR2*
SOX11	14/299	0.0143745180 45711224	*ROBO2;CNTNAP2;NTRK2;AUTS2;PCDH7;BACH2;CDH6;PTPRD;CDH4;KIF26B;FAT3;SLIT2;SOX5;DDR2*
SOX14	14/299	0.0143745180 45711224	*RBFOX1;CASZ1;CELF4;PAX3;ESRRG;ADARB2;ANK1;NELL1;CDH4;DPP6;NRG3;PAX7;ERBB4;CSMD1*
SOX30	14/299	0.0143745180 45711224	*CADPS2;CASZ1;NRXN1;PAX3;RORA;ESRRG;NRG1;GRIK2;ANK1;DPP6;PAX7;DOCK2;CDH18;DDR2*
TOX2	14/299	0.0143745180 45711224	*CADPS2;AUTS2;ATP2B4;NRG1;ANK1;HDAC9;BACH2;CDH4;DNER;FAM20C;CDH13;SLIT2;TOX;DDR2*
VSX2	14/299	0.0143745180 45711224	*NTRK2;RBFOX1;CASZ1;CELF4;PAX3;RORA;ESRRG;ANK1;CDH4;NRG3;PAX7;ERBB4;KIF26B;SOX5*
ZBTB38	14/299	0.0143745180 45711224	*MAST4;KMT2C;PDE4D;ATP2B4;ZBTB20;PDE4DIP;RORA;LMO7;ANK1;LYST;TRPS1;UTRN;LPIN1;DDR2*
ZC3H6	14/299	0.0143745180 45711224	*CADPS2;KMT2C;FAM13A;ZBTB20;PDE4DIP;RORA;LYST;BACH2;NBEA;DMD;TNIK;UTRN;LPIN1;DDR2*
ZFP37	14/299	0.0143745180 45711224	*CADPS2;PDE4D;ZBTB20;PDE4DIP;ANK2;RORA;PPP1R9A;PTPRD;PLCB4;NBEA;NAV3;TRPS1;SOX5;DDR2*
ZHX1	14/299	0.0143745180 45711224	*NCOA1;DENND1B;KMT2C;PDE4D;TCF12;ZBTB20;PDE4DIP;RORA;LYST;STAG2;TRPS1;ZNF638;TTC3;DDR2*
ZNF141	14/299	0.0143745180	*CADPS2;CNTNAP2;AUTS2;GLDC;PDE4D;DNMT3A*

		45711224	*;PDE4DIP;RORA;PPP1R9A;HDAC9;NBEA;TRPS1;ZNF267;DDR2*
ZNF157	14/299	0.0143745180 45711224	*MAGI1;CADPS2;CNTNAP2;NRXN1;PCDH7;ADAM22;DACH2;DNER;DMD;CTNNA2;TOX;MCF2L2;ASTN1;DDR2*
ZNF182	14/299	0.0143745180 45711224	*CADPS2;KMT2C;ZBTB20;RORA;BACH2;STAG2;PLCB4;TRPS1;ZNF638;NEK1;DMD;TNIK;UTRN;DDR2*
ZNF438	14/299	0.0143745180 45711224	*CADPS2;PDE4D;ATP2B4;ZBTB20;PDE4DIP;RORA;SSH2;HDAC9;ARHGAP24;ETV6;LRMDA;TRPS1;LPIN1;DDR2*
ZNF518A	14/299	0.0143745180 45711224	*DENND1B;CADPS2;KMT2C;ATP2B4;ZBTB20;PDE4DIP;RORA;LYST;MKLN1;STAG2;TRPS1;ZNF638;NEK1;DDR2*
ZNF518B	14/299	0.0143745180 45711224	*CADPS2;PARM1;PDE4D;PCDH7;ZBTB20;PDE4DIP;HDAC9;BACH2;NBEA;TTC3;SLIT2;UTRN;LPIN1;DDR2*
ZNF521	14/299	0.0143745180 45711224	*ROBO2;CADPS2;NTRK2;TENM2;NEGR1;PCDH7;ZBTB20;PTPRD;TRPS1;CDH13;SLIT2;TOX;GPC6;DDR2*
ZNF573	14/299	0.0143745180 45711224	*MAGI1;CADPS2;PATJ;AUTS2;MAST4;KMT2C;PDE4D;FAM13A;ZBTB20;PDE4DIP;RORA;NBEA;TRPS1;DDR2*
ZNF595	14/299	0.0143745180 45711224	*CADPS2;IQSEC1;KMT2C;PDE4D;ATP2B4;PDE4DIP;RORA;NBPF1;HDAC9;BACH2;TRPS1;TOX;LPIN1;DDR2*
ZNF84	14/299	0.0143745180 45711224	*MAGI1;KMT2C;PDE4D;TCF12;ZBTB20;PDE4DIP;RORA;BACH2;MKLN1;STAG2;NBEA;ZNF638;TTC3;DDR2*
ZXDA	14/299	0.0143745180 45711224	*CADPS2;PDE4D;ZBTB20;RORA;PPP1R9A;HDAC9;BACH2;PTPRD;NBEA;TRPS1;DMD;UTRN;SOX5;DDR2*
ALX1	13/299	0.0315429653 690396	*CADPS2;PCDH7;PAX3;ESRRG;CDH6;PAX7;ERBB4;KIF26B;SLIT2;SOX6;GPC6;EDIL3;DDR2*
ALX4	13/299	0.0315429653 690396	*NTRK2;CASZ1;PAX3;RORA;ESRRG;ANK1;CDH4;PAX7;KIF26B;TRPS1;SOX6;CSMD1;DDR2*
ARID2	13/299	0.0315429653 690396	*NCOA1;KMT2C;TCF12;DNMT3A;PRRC2C;ZBTB20;RORA;ETV6;MKLN1;STAG2;TRPS1;UTRN;DDR2*
BSX	13/299	0.0315429653 690396	*RBFOX1;CASZ1;CELF4;PAX3;RORA;ESRRG;CDH4;DPP6;PAX7;DSCAML1;ASTN1;RNF220;DDR2*
DMRT1	13/299	0.0315429653 690396	*RYR2;CADPS2;NTRK2;RBFOX1;GLDC;PCDH7;PAX3;ANK1;DPP6;PAX7;KIF26B;CSMD1;DDR2*
EMX1	13/299	0.0315429653 690396	*CADPS2;CNTNAP2;CASZ1;PCDH7;PAX3;RORA;ESRRG;NRG1;ANK1;CDH4;PAX7;TOX;DDR2*
ERG	13/299	0.0315429653 690396	*NTRK2;PDE4D;ITGA1;PTPRM;ZBTB20;RORA;HDAC9;ETV6;CDH13;DMD;SLIT2;UTRN;DDR2*
ESX1	13/299	0.0315429653 690396	*PAX3;HTR2C;ESRRG;ANK1;PPP2R2C;PAX7;ERBB4;KIF26B;GPC3;CDH13;SLIT2;SOX6;FGF13*
FOXD4L1	13/299	0.0315429653 690396	*CADPS2;CNTNAP2;CASZ1;NRXN3;PAX3;NRG1;ANK1;ARHGAP24;PAX7;KIF26B;ZNF385B;TOX;DDR2*
GTF2IRD2	13/299	0.0315429653 690396	*CADPS2;MAST4;KMT2C;PDE4D;PTPRM;ZBTB20;PDE4DIP;ANK2;PPP1R9A;ANK1;BACH2;UTRN;DDR2*
HIVEP2	13/299	0.0315429653 690396	*PDE4D;ATP2B4;ZBTB20;PDE4DIP;RORA;LMO7;ANK1;HDAC9;BACH2;ETV6;TRPS1;UTRN;DDR2*
HLF	13/299	0.0315429653 690396	*CADPS2;NTRK2;PARM1;PCDH7;ATP2B4;ZBTB20;RORA;ANK1;PTPRD;PLCB4;SLIT2;SOX5;DDR2*
IKZF4	13/299	0.0315429653 690396	*CADPS2;NTRK2;AUTS2;DNMT3A;ATP2B4;ZBTB20;RORA;PPP1R9A;BACH2;KIF26B;TRPS1;TOX;*

			DDR2
IRX1	13/299	0.0315429653690396	*CADPS2;NTRK2;PCDH7;ESRRG;NRG1;ANK1;CDH4;DPP6;KIF26B;CDH13;SLIT2;GPC6;DDR2*
IRX5	13/299	0.0315429653690396	*CADPS2;NTRK2;CASZ1;AUTS2;PCDH7;RORA;NRG1;ANK1;KIF26B;TRPS1;CDH13;SLIT2;DDR2*
KLF14	13/299	0.0315429653690396	*CADPS2;NTRK2;DGKB;PAX3;ESRRG;NRG1;ANK1;HDAC9;CDH4;DPP6;PAX7;KIF26B;SLIT2*
LHX1	13/299	0.0315429653690396	*CADPS2;CNTNAP2;CASZ1;PCDH7;CELF4;PAX3;ESRRG;ANK1;CDH6;PAX7;KIF26B;SLIT2;DDR2*
MAEL	13/299	0.0315429653690396	*RYR2;CADPS2;CNTNAP2;ESRRG;ANK1;RIMS1;ERBB4;KIF26B;DNER;BRINP3;CDH18;DDR2;RGS7*
MEF2A	13/299	0.0315429653690396	*KMT2C;PDE4D;TCF12;ATP2B4;ZBTB20;PDE4DIP;RORA;MITF;LMO7;HDAC9;TRPS1;UTRN;DDR2*
MGA	13/299	0.0315429653690396	*DYNC1H1;KMT2C;TCF12;PRRC2C;ZBTB20;LYST;MKLN1;STAG2;TRPS1;ZNF638;TTC3;UTRN;DDR2*
NCOA2	13/299	0.0315429653690396	*NCOA1;DYNC1H1;ANKRD44;KMT2C;TCF12;PRRC2C;ZBTB20;PDE4DIP;RORA;LYST;TRPS1;UTRN;DDR2*
NCOR2	13/299	0.0315429653690396	*DYNC1H1;KDM4B;AUTS2;IQSEC1;KMT2C;DNMT3A;PRRC2C;ZBTB20;PDE4DIP;ANK1;ETV6;UTRN;DDR2*
NEUROD2	13/299	0.0315429653690396	*RAP1GAP2;CADPS2;NTRK2;RBFOX1;NRXN1;CELF4;RORA;ANK1;BACH2;CDH4;DPP6;DAB1;DDR2*
NFATC2	13/299	0.0315429653690396	*NTRK2;AUTS2;PDE4D;ATP2B4;ZBTB20;RORA;ANK1;HDAC9;BACH2;DOCK10;TNIK;SOX5;DDR2*
NOTO	13/299	0.0315429653690396	*RYR2;CADPS2;NTRK2;CASZ1;CELF4;PAX3;RORA;ESRRG;ANK1;NBEA;PAX7;KIF26B;SLIT2*
NR6A1	13/299	0.0315429653690396	*TRIM71;AUTS2;GLDC;PDE4D;DNMT3A;ZBTB20;RORA;MITF;ESRRG;BACH2;SOX6;SOX5;DDR2*
PDX1	13/299	0.0315429653690396	*PCSK1;CADPS2;NTRK2;PCDH7;CADPS;PAX3;ESRRG;ANK1;NBEA;PAX7;PDE3A;SOX6;SOX5*
POGZ	13/299	0.0315429653690396	*DYNC1H1;KDM4B;DSCAM;KMT2C;DNMT3A;PRRC2C;ZBTB20;PDE4DIP;ANK2;STAG2;ZNF638;SCN2A;DDR2*
POU4F2	13/299	0.0315429653690396	*CADPS2;RBFOX1;DCC;CELF4;NRXN3;PAX3;ESRRG;NELL1;CDH4;DPP6;PAX7;DNER;SLIT2*
POU4F3	13/299	0.0315429653690396	*PCSK1;CNTNAP2;RBFOX1;CASZ1;NRXN1;CELF4;PAX3;ESRRG;GRIK2;ANK1;CDH4;DPP6;PAX7*
RFX7	13/299	0.0315429653690396	*KDM4B;DSCAM;KMT2C;NRXN1;TCF12;DNMT3A;PRRC2C;ZBTB20;ANK2;TRPS1;SCN2A;UTRN;DDR2*
RHOXF2B	13/299	0.0315429653690396	*PCSK1;CADPS2;FAM9B;ATRNL1;NRXN3;KIAA1549L;ESRRG;CDH6;STAG2;FGF14;CDH12;ZNF385B;BRINP3*
SCML4	13/299	0.0315429653690396	*CADPS2;ANKRD44;PDE4D;NRXN1;ZBTB20;RORA;ANK1;BACH2;DOCK10;TNIK;UTRN;TOX;DDR2*
SCRT1	13/299	0.0315429653690396	*CADPS2;NTRK2;RBFOX1;CASZ1;NRXN1;CADPS;CELF4;ESRRG;ANK1;DPP6;PPP2R2C;DNER;RIMBP2*
T	13/299	0.0315429653690396	*CADPS2;RBFOX1;PCDH7;PAX3;RORA;ESRRG;ANK1;CDH4;DAB1;PAX7;KIF26B;CDH13;DDR2*
TGIF2LY	13/299	0.0315429653690396	*DPP10;CADPS2;PCDH7;HTR2C;PXDNL;ESRRG;FSTL5;LRFN5;NRG3;TRPS1;CDH12;CDH18;DDR2*
TP63	13/299	0.0315429653690396	*NTRK2;TENM2;PDE4D;PCDH7;ZBTB20;RORA;MITF;NRG1;HDAC9;BACH2;ERBB4;DMD;DDR2*
VAX1	13/299	0.0315429653690396	*CADPS2;RBFOX1;CASZ1;CELF4;PAX3;ESRRG;ANK1;DPP6;PAX7;KIF26B;SLIT2;SOX6;SOX5*
ZBTB37	13/299	0.0315429653	*DENND1B;CADPS2;ANKRD44;KMT2C;ATP2B4;PR*

		690396	*RC2C;ZBTB20;RORA;LYST;BACH2;TRPS1;UTRN;DDR2*
ZFHX2	13/299	0.0315429653690396	*RAP1GAP2;CADPS2;CASZ1;AUTS2;DNMT3A;ATP2B4;ZBTB20;ANK2;ANK1;BACH2;KIF26B;TRPS1;SOX5*
ZFP14	13/299	0.0315429653690396	*CADPS2;CNTNAP2;ZBTB20;PDE4DIP;RORA;PPP1R9A;BACH2;PLCB4;NBEA;TRPS1;UTRN;SOX5;DDR2*
ZFP2	13/299	0.0315429653690396	*CADPS2;NTRK2;AUTS2;PDE4D;ZBTB20;RORA;PPP1R9A;ANK1;BACH2;NBEA;DMD;SOX6;DDR2*
ZFP92	13/299	0.0315429653690396	*ROBO2;CADPS2;NTRK2;ZBTB20;RORA;ANK1;PTPRD;DAB1;MYO5B;SLIT2;RGS9;SOX5;DDR2*
ZIC3	13/299	0.0315429653690396	*TRIM71;CADPS2;CNTNAP2;GLDC;NRXN1;PCDH7;CADPS;HTR2C;ESRRG;ANK1;GPC3;SLIT2;FGF13*
ZNF148	13/299	0.0315429653690396	*NCOA1;KMT2C;TCF12;PRRC2C;ZBTB20;RORA;MKLN1;STAG2;TRPS1;ZNF638;TTC3;UTRN;DDR2*
ZNF19	13/299	0.0315429653690396	*FBLN7;CADPS2;PATJ;AUTS2;KMT2C;PDE4D;PLCL1;PDE4DIP;RORA;LMO7;BACH2;SOX6;DDR2*
ZNF248	13/299	0.0315429653690396	*DENND1B;CADPS2;AUTS2;PDE4D;TCF12;ATP2B4;PDE4DIP;RORA;LYST;NBEA;TRPS1;TTC3;DDR2*
ZNF37A	13/299	0.0315429653690396	*DENND1B;CADPS2;PDE4D;PRRC2C;RORA;TMEM64;PPP1R9A;LYST;HDAC9;MKLN1;TRPS1;TTC3;DDR2*
ZNF493	13/299	0.0315429653690396	*MAGI1;CADPS2;KMT2C;ATP2B4;ZBTB20;PDE4DIP;RORA;PPP1R9A;LYST;NBEA;TRPS1;ZNF638;DDR2*
ZNF559	13/299	0.0315429653690396	*KDM4B;DSCAM;AUTS2;KMT2C;NRXN1;DNMT3A;PTPRM;ZBTB20;ANK2;RORA;RIMS1;SCN2A;DDR2*
ZNF560	13/299	0.0315429653690396	*CHODL;PCSK1;RYR2;CADPS2;CNTNAP2;GLDC;NRXN1;PCDH7;DAB1;FGF13;MDGA2;CSMD1;FREM2*
ZNF610	13/299	0.0315429653690396	*CADPS2;AUTS2;NEGR1;PDE4D;PCDH7;ZBTB20;RORA;BACH2;PLCB4;NBEA;SLIT2;TOX;DDR2*
ZNF621	13/299	0.0315429653690396	*KMT2C;TCF12;ATP2B4;PRRC2C;ZBTB20;PDE4DIP;RORA;LYST;MKLN1;STAG2;TRPS1;UTRN;DDR2*
ZNF652	13/299	0.0315429653690396	*NCOA1;DYNC1H1;KMT2C;ATP2B4;PRRC2C;ZBTB20;RORA;MKLN1;STAG2;TRPS1;TTC3;UTRN;DDR2*
ZNF662	13/299	0.0315429653690396	*CADPS2;NTRK2;AUTS2;NEGR1;PDE4D;SMAD9;RORA;BACH2;PLCB4;DMD;CNTN4;SOX6;DDR2*
ZNF709	13/299	0.0315429653690396	*DENND1B;CADPS2;KDM4B;AUTS2;PDE4D;DNMT3A;ZBTB20;RORA;LMO7;BACH2;PTPRG;UTRN;DDR2*

Table S13. GO associations with biological processes (BP), molecular functions (MF), and cellular components (CC) of 2527 rDNA-contacting genes detected only in Mel Z cells grown on Matrigel. The number of contacts with nucleoli increased in these genes. Related to Figure 6A.

GO.ID	Description	padj	Genes
MF			
GO:0005	protein	2.0561217592103	*SLC18A1,RIT2,SLMAP,KLHL13,ABCB7,PBX4,A*

515	binding	18e-11	*COT12,NHLRC3,MYO9B,GPSM2,COL4A5,ERG,PARN,CYTH1,CHD9,STK16,XK,SEMA4D,MMS22L,MET,MAX,TNS3,SCYL3,TEAD1,ATXN10,BRCA1,EIF4G3,ACSM3,BAZ1B,SNX16,IFTAP,CEP350,STAU2,SIAH3,DUS3L,PIWIL3,CETN3,CHMP1B,DRD3,ELAPOR2,ATP11C,PRORP,ZNF534,TOX3,DNAH11,CDC42BPA,POLR2J,POLG,EXOC2,PTPN5,TACR1,SDC2,GPHB5,CIITA,FANCC,ARAP1,KLHL7,CCR3,ANKLE2,HBG2,CEP85,RPRD1B,CIP2A,AHNAK,ACTR5,LRRC53,EIF4A3,DLEC1,NFE2L2,SCHIP1,PARG,OLFML2A,UIMC1,TFEC,NFYC,H2BC18,UPP2,UBTD1,DTNB,VRK2,ITSN1,OAS1,DEUP1,R3HDM2,ATP2B2,ESPNL,PREX2,SCRN1,FAM177A1,ELAVL2,PSKH2,FBF1,FBXL20,NTN4,SERP1,POLR1A,ENKUR,RAB11FIP4,GRPEL2,SNTA1,PPCDC,CLDN10,ASZ1,NUB1,SRGAP2C,WIF1,FLI1,CSNK2A2,SPATS2L,CLIC5,ESRRB,ZFHX2,ARHGAP26,RBP7,PTPN4,SETBP1,ZBTB7C,ITGA2,GRIN2A,NELL2,IL1RAPL1,WDPCP,KIF5C,BLK,GPM6A,SPATA16,WNT16,KRT86,SETD3,ADCY1,GRID1,RPS6KA2,EFR3B,TRIM24,ZDHHC17,HLCS,FIG4,EIF2AK2,AP1S3,CFAP47,FOXP2,FBXO34,F2RL2,KMT2D,LPAR3,SHE,ANO4,GIPC2,FMR1,GFOD1,TXLNG,FOXN3,SP110,SH3BP2,SH3TC2,CEP290,WDFY3,SHQ1,TACC1,RAD9B,SH3PXD2B,OCLN,CFAP91,TUT4,AK8,FASTKD5,ITGBL1,NETO1,PTPRQ,TNPO1,GBE1,SNX5,CDK13,SNX32,DNPEP,NEO1,SRGAP2B,STAG1,CLDN14,ITPKB,PLEKHB2,TIMM23,UBOX5,TCERG1,HBE1,SLC66A1,F10,APOO,SRGAP1,DIO2,FAM184A,CABIN1,SYNE1,PSMD10,FBXO47,FBXL17,PDZD2,EOLA1,DHX29,GAS2,GPAM,NABP1,IRF2,RUNX2,SPATA6L,LRP12,TBC1D22A,FILIP1L,LRRC1,ESRP1,UBE2A,BRMS1L,RBM41,RGS12,PPP6R3,CCDC157,OSBP2,CBX3,ACAP2,SLC39A10,KCNV1,ZNF496,KCTD8,ZNF550,CHCHD6,KIZ,MPHOSPH9,RNF10,FAT1,PIK3R1,STAMBPL1,IL17B,AGMO,ABCB11,SARNP,COL26A1,SHTN1,ATG12,FNDC3B,MASP1,CD200R1,CALD1,POLR3F,SH3GL2,CLBA1,HIBADH,CRTC1,VCF1,GSE1,FHIP1A,PITPNC1,MYLK3,SLC30A3,NXPE3,DOCK7,PDK3,MTX2,FBXL4,ROR1,SCAP,PAX5,RTL8B,AAK1,CRB1,CTTN,UTP15,PASK,USP8,ARID1A,PARD6B,THEMIS,SMAD6,RYBP,NYAP2,PSD3,RFX2,ACY3,MEF2C,FZR1,BAIAP2L1,ST8SIA5,TBC1D19,DBNL,ELP4,RYK,MACF1,PXT1,TAF4B,MALT1,TUBGCP3,MTM1,SLC15A5,TFDP2,KCNE1,PTPRE,UNC5B,ZSCAN5A,MFSD6,SERINC3,TBC1D31,PTPN12,CRYZL1,PTPRZ1,ACACA,CD55,PLN,EML1,CTDSPL2,SHISA3,SLC16A7,NEDD4,RBBP9,FLYWCH1,LRRC38,DOCK8,CYSLTR2,VWA8,PARVA,PPP1R8,SECISBP2,URI1,SCML2,ANKRD28,CHD6,FGD4,PDXK,PRDX1,NDUFAF6,COX17,PACS2,HACE1,SNRPF,ATG4B,AEBP2,SLC7A14,DSC2,SERPINA1,MYO10,OLFM1,CRHR2,MID1,POGK,TSHZ2,SLC12A2,OTX1,EFNB3,TRIM37,DLG3,SSR3,TRAT1,PROSER1,FLOT2,PVRIG,SRP68,SV2B,ILK,LEF1,EYA1,MAPK14,MYBPC3,WDR17,SLC25A13,RAPGEF1,ALG13,UNC79,SH3GL3,AKR7A2,ZNF516,SLC1A2,ARHGEF3,EDNRB,GRIN2B,UBXN10,SPDYE3,SVOP,GPR141,IFNG*

			R2,DENND4A,TBC1D20,OXR1,SFMBT2,SETD5,SNX2,GRHPR,SCAMP5,IL17RB,DCAF7,PARP2,MINDY3,KLHL4,OVOL2,EIF3F,RDH16,FCHSD2,SLC4A7,DNAJB6,MIGA1,NPIPB3,SPCS1,MTUS1,LEMD3,PLEKHF2,KHDRBS2,PIEZO1,SOAT1,BRD10,SLC9B2,NEB,KANSL1L,KLHL32,TMEM100,LYPD6,PDIA5,XPR1,VDR,NEK11,PDPN,RTN4,GNL3L,CACNA1D,ARHGEF11,PRKAA2,IVNS1ABP,CD163,DPT,BBS2,CLSTN1,ZNF155,SERGEF,SCYL2,SKAP1,NT5C3A,MAOB,SKIC3,SERPINE2,MYO3A,MICOS10,NUBPL,PLAAT3,POGLUT2,IL12RB2,PIK3CG,ANOS1,TPP1,ANKFN1,STIL,TFDP1,IL1R2,ATP6V1E2,TNPO3,HTT,NUP160,NECTIN3,IQGAP1,MYCBP2,WIPI1,LATS1,KLHL12,SNX30,GNPDA2,TSNARE1,FANCI,ACAA1,SPARCL1,PRSS23,WNT2B,LRRC37A3,TMDD1,EXD3,TNFRSF10B,MAP2,FBXW11,MTCL1,PTPRO,ZFAND3,HSPH1,SUGP2,AZIN2,IYD,SMG6,HLA-DMA,PEMT,ARHGAP6,POLR2J2,PPP2R2B,CHD5,JAK1,ITGA9,ACSL6,TOR1AIP2,ULK2,KCNB1,RADIL,CANX,ZMIZ1,UGP2,HSD17B12,ANKRD27,AIM2,ANK3,GTF2IRD1,ZNF19,AMT,KITLG,SV2C,ARID4B,GMDS,ZNF326,MAP4K4,CNIH3,PLEKHM2,NR1I2,ZNF266,NXPE1,HIP1,RAET1E,RFX7,RELN,MFHAS1,PDHB,ADAMTSL3,FBXO32,NXPH1,MTMR3,CRKL,MYO18B,TENT5A,ARHGEF10,FGD6,SYTL3,PTPN13,SNAP91,VAV3,RPS6KC1,INPP5A,CD40LG,VRK1,URM1,SCN9A,ZNF420,DPYSL3,ATP2B1,SPRING1,SLC16A10,ATRN,NDUFB9,CCDC88A,SLC35B4,ARNT2,RNGTT,APBA1,HPSE2,PLCE1,TNIP3,TACC2,NAALAD2,DCAF5,GNAL,CSTF1,PEBP4,STK32C,MSH3,MINDY1,BAK1,CASTOR2,LAX1,CACTIN,STK26,CLCC1,ENOX2,TENM4,DCP1A,ZCRB1,PPP3CA,CHRNA5,MKNK1,DYSF,NKD1,RBPJ,CYRIB,SPDYE17,C3,RIN2,PDE6C,PLEKHG7,KBTBD3,ASCC1,CDH10,DENND1A,MZT1,PDZRN3,TASP1,MICU1,TAF10,TRIP12,C3ORF52,MBD5,BACE2,KDM5A,ZNF549,FCGBP,NOMO1,ARL6IP6,ZNF320,CNOT6,PKIB,TBC1D9B,PXMP2,ASH1L,RSPO2,TPMT,ZNF747,ACSF2,DISC1,PROX1,BMP6,MAP3K7CL,FMN1,RALGPS1,CCDC6,MUC5B,CTSB,DNAJC11,ASAP1,LRRC2,VSIG1,LRRC42,AQP8,CREBBP,ITGB1,BVES,RBM26,CYB561,DRC7,ZNF827,LMAN2L,NFKBIZ,CORO1C,KANK3,NSG2,SPIDR,C17ORF80,NEFL,NAV2,ATP6V1A,STK3,CHN2,RNF150,C1GALT1,HNF4G,SMCO4,MSR1,EXOSC3,AP2B1,SH2D3C,APTX,TRPC4,RAMP3,GNG2,FRS3,CPB1,LIMS1,DOCK4,ACKR2,ARPC1A,UBL4B,BCL11B,CERS3,GLCE,PLS3,PCSK2,TSPAN2,KCNIP1,PWWP2A,TBC1D10A,EPHB1,SSBP2,CASP8AP2,REEP1,RPH3A,CTTNBP2,SLCO6A1,CYTH4,NAP1L1,SLC38A6,VPS29,PLPP4,CCDC102B,COPB2,UBR1,CNN2,COL4A2,TCF7L1,ARHGAP32,PAGE1,DYRK3,IP6K2,CNTLN,CHRNA6,CMSS1,TLN2,HDAC4,GDNF,KLHL1,FYB1,TSKS,CALCB,MARCHF5,RBM45,SEM1,IQCE,LVRN,MYLK,UNC5C,CSGALNACT1,TLR7,PABIR3,P3H2,CFL1,KRT20,WDR7,SLAIN2,HMG20A,CEP85L,PLEKHG4B,RBBP8,MED13,PHF2,RAI2,AFDN,CYTIP,LRP2,MTDH,SEMA6D,LIG1,EHMT1,ZDHHC13,WWC3,IL17RA,CAMK4,CLP

			X,VWC2,REPS1,FBXL7,RPA3,TMEM108,TPTE,BFSP1,LCP2,MB21D2,SEZ6L,TMEM123,GLI2,AFG2B,TGFBR3,MAP3K7,UPRT,GTF2I,ATXN1,ADAM17,SEMA4F,ELAVL1,S100PBP,TRAK1,STXBP1,IDH1,NUAK2,LMOD2,HDGFL2,MUC13,NUP205,PLEKHG1,NHLH1,ELP2,SLCO5A1,NHSL2,SNRNP200,ORC4,APC,GNA12,TTLL5,IL18R1,INO80,NAA11,C8ORF74,MOCS2,ERGIC2,SULT2B1,SPTSSB,PEAR1,CDA,PPARGC1B,PHLDB2,ESR2,NOL10,LNX1,ERC1,ZNF440,ROS1,RUBCNL,AGO3,C9,PVR,CKAP4,MOB3B,RPL39L,ACER1,IQGAP2,CDYL,RLF,CLPB,SPINK1,ANKRA2,TAF5L,JPT1,CTNND2,DNAH14,FRMD3,ERI1,RASSF3,UGGT1,PACSIN2,TIA1,DPYSL2,SYNPO2,RAB8B,NSD2,ASCC3,ACYP2,SLC23A2,PHACTR3,RMDN2,PAIP2,KCND1,PTPN11,TMEM45A,TMEM267,KIAA1958,JADE3,CARD18,PSMC2,MAPKAP1,EXOC6B,RIC1,ACOX1,USP24,NUP153,CLPTM1,HSD17B4,TRMT10B,RAI14,GPATCH2L,NRP2,SIPA1L2,CAVIN1,PLXNC1,KCTD5,C1ORF21,METTL15,FOXJ2,CEP162,ELOVL7,NR3C1,BORCS5,FSTL4,ZC3HAV1,MTOR,KSR1,CBLB,FAF1,SNAP25,TUFT1,RP1,DEFB124,SGSM1,ADH7,GOLGA8A,ZNF221,CLDND1,PIP5K1A,BTBD2,GH2,MMACHC,DISP1,RAB6B,KYNU,ADAL,ASCC2,SPRED1,ADAM10,GNG4,CNTNAP4,WASHC1,CDH26,CBY2,SZRD1,FAM53C,PDS5B,CRIM1,MEI4,RBM46,SUGP1,PFKFB3,SPANXD,RNF115,TRIM61,ESD,CDHR3,PER2,ERICH3,PLEKHH2,ANKRD24,GLI3,LHPP,KAT6A,BCOR,NTRK3,RXFP1,GAB2,DIS3L2,CTCF,SNTB2,PDE1A,PDCD10,TFG,EPHX2,LMTK2,HHAT,UBE4A,NWD2,IDH3A,CREB5,CCDC68,ADGRG6,GOLGA7,FERMT2,GCNA,TSPOAP1,GLUD1,DNM3,ST3GAL2,SYT1,SREK1IP1,PHIP,EPB41,DENND5A,SS18,SFRP5,DOCK9,ZYG11B,RFC2,PPARA,CD244,SHISA6,NFASC,HDX,MAP2K5,ZNF229,PHF6,HMGB4,EPB41L2,CADM3,FGF7,TENT5D,MARCHF10,TBCA,PACSIN1,STXBP6,MGA,RTTN,CACNA1A,EDA,CNTRL,LIMCH1,TNFSF13B,GNPNAT1,MTMR7,PTH2R,ELOVL6,IAH1,MICAL2,COL14A1,RBM47,EIF2A,JARID2,HDAC2,FIRRM,TGOLN2,FGD1,TRIM44,TRIM39,SEMA3D,PROM1,EIF4E3,ACP3,USP48,KCNH5,RRM1,MAGEA1,CADM1,KLHL29,INPP5D,CFAP161,THUMPD1,SPDYE9,ATF6,PRKAR1B,GBF1,IPO11,SERPINE1,CCDC88C,BACH1,CUL9,EWSR1,CYTH3,KLC1,SLC26A5,AKAP13,HBS1L,MLANA,PKM,GCG,ARFGEF1,CCDC150,TKFC,LINC02218,PTPN3,STAC,SCN8A,RALGAPA1,RTL4,SSX2IP,TTC17,ELP1,IRF8,HIVEP3,C2ORF42,HEPACAM2,RFX4,CCDC198,REM1,ACTA2,AGAP1,FREM1,RIPOR2,KCNK5,CD69,B3GALNT2,FUNDC2,EPS15L1,CYB561A3,PARD3B,XIRP2,KDM3A,MZT2A,CAST,TAOK1,UNC45A,GPR39,ADGRA3,CASK,HSPG2,SLCO4C1,GCNT1,BPIFB3,GGA2,PRDM7,USH1C,FMN2,UBXN2A,NLRP14,PCNT,DNAJC13,AFG2A,PSME3IP1,GOLGA8B
GO:0005085	guanyl-nucleotide exchang	0.000020670639041584468	*CYTH1,ITSN1,PREX2,DOCK7,PSD3,DENND11,DOCK8,FGD4,RAPGEF1,ARHGEF3,DENND4A,ARHGEF11,SERGEF,MYCBP2,ANKRD27,ARHGEF10,FGD6,VAV3,CCDC88A,PLCE1,RIN2,PLEKHG7,DEN*

	e factor activity		*ND1A,RALGPS1,SH2D3C,DOCK4,TBC1D10A,CYTH4,PLEKHG4B,PLEKHG1,RIC1,DENND5A,DOCK9,FGD1,GBF1,CCDC88C,CYTH3,AKAP13,ARFGEF1*
GO:0030695	GTPase regulator activity	0.0002853917475571833	*MYO9B,GPSM2,CYTH1,ARAP1,ITSN1,PREX2,ARHGAP26,RASA2,SRGAP1,TBC1D22A,RGS12,ACAP2,DOCK7,PSD3,TBC1D19,DENND11,DOCK8,FGD4,RAPGEF1,ARHGEF3,DENND4A,TBC1D20,ARHGEF11,SERGEF,IQGAP1,MYCBP2,ARHGAP6,ANKRD27,ARHGEF10,FGD6,VAV3,CCDC88A,PLCE1,RIN2,PLEKHG7,DENND1A,TBC1D9B,RALGPS1,ASAP1,CHN2,SH2D3C,DOCK4,TBC1D10A,CYTH4,ARHGAP32,PLEKHG4B,PLEKHG1,IQGAP2,RIC1,SIPA1L2,ARHGAP28,SGSM1,DENND5A,DOCK9,FGD1,GBF1,CCDC88C,CYTH3,AKAP13,ARFGEF1,RALGAPA1,AGAP1*
GO:0060589	nucleoside-triphosphatase regulator activity	0.0002853917475571833	*MYO9B,GPSM2,CYTH1,ARAP1,ITSN1,PREX2,ARHGAP26,RASA2,SRGAP1,TBC1D22A,RGS12,ACAP2,DOCK7,PSD3,TBC1D19,DENND11,DOCK8,FGD4,RAPGEF1,ARHGEF3,DENND4A,TBC1D20,ARHGEF11,SERGEF,IQGAP1,MYCBP2,ARHGAP6,ANKRD27,ARHGEF10,FGD6,VAV3,CCDC88A,PLCE1,RIN2,PLEKHG7,DENND1A,TBC1D9B,RALGPS1,ASAP1,CHN2,SH2D3C,DOCK4,TBC1D10A,CYTH4,ARHGAP32,PLEKHG4B,PLEKHG1,IQGAP2,RIC1,SIPA1L2,ARHGAP28,SGSM1,DENND5A,DOCK9,FGD1,GBF1,CCDC88C,CYTH3,AKAP13,ARFGEF1,RALGAPA1,AGAP1*
GO:0005547	phosphatidylinositol-3,4,5-trisphosphate binding	0.0004659534548220729	*ARAP1,PLEKHB2,MYO10,FCHSD2,IQGAP1,ASAP1,IQGAP2,MAPKAP1,GAB2,FERMT2,GBF1,CYTH3,FUNDC2*
GO:0008092	cytoskeletal protein binding	0.0008960905340805718	*MYO9B,BRCA1,CEP350,STAU2,CETN3,DLEC1,ESPNL,SNTA1,PTPN4,KIF5C,SETD3,FMR1,SNX5,SYNE1,PSMD10,GAS2,SPATA6L,SHTN1,CALD1,BAIAP2L1,DBNL,MACF1,TUBGCP3,KCNE1,EML1,PARVA,FGD4,MYO10,MID1,MYBPC3,KLHL4,MTUS1,NEB,CACNA1D,CLSTN1,MYO3A,HTT,IQGAP1,MAP2,FBXW11,MTCL1,HSPH1,ANK3,MAP4K4,PLEKHM2,HIP1,MYO18B,ARHGEF10,DPYSL3,CCDC88A,DCP1A,DISC1,FMN1,ITGB1,CORO1C,ARPC1A,PLS3,REEP1,CNN2,TLN2,KLHL1,MYLK,UNC5C,CFL1,AFDN,TRAK1,LMOD2,APC,TTLL5,INO80,IQGAP2,FRMD3,PACSIN2,SYNPO2,PHACTR3,RMDN2,RAI14,SNAP25,RP1,RAB6B,WASHC1,PLEKHH2,ANKRD24,SNTB2,LMTK2,FERMT2,DNM3,EPB41,PHF6,EPB41L2,TBCA,PACSIN1,LIMCH1,MICAL2,PROM1,CCDC88C,KLC1,SLC26A5,ARFGEF1,PTPN3,XIRP2,TAOK1,USH1C,FMN2*
GO:0045296	cadherin binding	0.001660139889278025	*CIP2A,AHNAK,SNX5,NEO1,DHX29,SHTN1,CALD1,CTTN,USP8,BAIAP2L1,DBNL,MACF1,PARVA,PRDX1,SNX2,RTN4,IQGAP1,PTPRO,ANK3,CRKL,CDH10,ASAP1,ITGB1,TRPC4,TBC1D10A,CNN2,AFDN,MB21D2,IDH1,PHLDB2,ERC1,CTNND2,PACSIN2,PTPN11,ZC3HAV1,CDH26,CDHR3,DOCK9,STXBP6,EIF2A,PROM1,PKM,EPS15L1,CAST,UNC45A*

GO:0035091	phosphatidylinositol binding	0.006444600418048456	*SNX16,SNX25,ARAP1,WDFY3,SH3PXD2B,SNX5,SNX32,PLEKHB2,PITPNC1,PASK,MTM1,MYO10,SNX2,FCHSD2,PLEKHF2,IQGAP1,WIPI1,SNX30,HIP1,SNAP91,RPS6KC1,CCDC88A,DENND1A,ASAP1,RPH3A,ARHGAP32,RUBCNL,IQGAP2,MAPKAP1,GAB2,FERMT2,SYT1,EPB41,GBF1,CYTH3,FUNDC2,PARD3B,GGA2*
GO:0019904	protein domain specific binding	0.027878044112019565	*GPSM2,CHMP1B,POLR2J,SDC2,NFE2L2,VRK2,ATP2B2,ENKUR,SNTA1,SH3BP2,SH3PXD2B,OCLN,RUNX2,CBX3,USP8,DBNL,TFDP2,PTPN12,NEDD4,SRP68,LEF1,RAPGEF1,KHDRBS2,SKAP1,TFDP1,IQGAP1,ARHGAP6,INPP5A,DPYSL3,ATP2B1,CCDC88A,BAK1,LAX1,NKD1,DENND1A,PROX1,FMN1,CCDC6,NFKBIZ,NEFL,DOCK4,TBC1D10A,CTTNBP2,CNTLN,LRP2,EHMT1,REPS1,TGFBR3,ADAM17,TRAK1,STXBP1,PPARGC1B,LNX1,ERC1,RAB8B,CARD18,ACOX1,CBLB,FAF1,SNAP25,ADAM10,CRIM1,PPARA,SHISA6,EPB41L2,RRM1,CADM1,INPP5D,CCDC88C*
BP			
GO:0032502	developmental process	1.4957037825862664e-9	*RIT2,PBX4,MYO9B,XK,SEMA4D,MET,SCYL3,TEAD1,ATXN10,BRCA1,EIF4G3,CNBD2,IFTAP,STAU2,SIAH3,PIWIL3,MMP16,DRD3,ELAPOR2,ATP11C,DNAH11,SDC2,FANCC,ARAP1,CCR3,ANKLE2,ACTR5,EIF4A3,DLEC1,NFE2L2,CYP19A1,SLC46A2,DTNB,GPR158,DEUP1,ATP2B2,PREX2,CSMD3,NTN4,SERP1,PLEKHM3,ENKUR,ASZ1,SRGAP2C,WIF1,FLI1,CSNK2A2,ESRRB,ZFHX2,ARHGAP26,TDRD9,ZBTB7C,ITGA2,GRIN2A,NELL2,IL1RAPL1,WDPCP,KIF5C,BLK,GPM6A,SPATA16,WNT16,KRT86,SETD3,ADCY1,RPS6KA2,PLPPR5,ZDHHC17,FIG4,EIF2AK2,CFAP47,FOXP2,KMT2D,LPAR3,FMR1,TXLNG,FOXN3,SH3TC2,CEP290,TACC1,SH3PXD2B,CFAP91,TUT4,AK8,PTPRQ,CDK13,NEO1,MYT1L,SRGAP2B,ITPKB,PLEKHB2,SYNE1,FBXL17,SLC9A4,GAS2,GPAM,RUNX2,SPATA6L,LRP12,TSPEAR,ESRP1,BRMS1L,OSBP2,SLC39A10,RNF10,FAT1,PIK3R1,CFDP1,PGM5,SHTN1,TMEM232,BTD,CALD1,SH3GL2,MYLK3,DOCK7,XKR4,ROR1,PAX5,CRB1,CTTN,ARID1A,PARD6B,THEMIS,SMAD6,NYAP2,RFX2,MEF2C,FZR1,DBNL,RYK,MACF1,TAF4B,MALT1,WEE2,MTM1,TFDP2,KCNE1,UNC5B,PTPN12,PTPRZ1,PLN,EML1,NEDD4,RBBP9,DDAH1,PARVA,SECISBP2,SLC9C1,SCML2,FGD4,ZNF609,PRDX1,ATG4B,MYO10,OLFM1,MID1,SLC12A2,OTX1,EFNB3,DLG3,FLOT2,ILK,LEF1,EYA1,MAPK14,MYBPC3,RAPGEF1,SH3GL3,ZNF516,SLC1A2,GABRB2,EDNRB,GRIN2B,ARSB,CRISPLD1,TBC1D20,SETD5,SNX2,PARP2,ZSWIM6,OVOL2,SLC4A7,DNAJB6,OMA1,PIEZO1,SOAT1,SLC9B2,NEB,TMEM100,VDR,PDPN,CREB3L2,RTN4,BBS2,CLSTN1,SPAG17,SCYL2,MAOB,SERPINE2,MYO3A,PLAAT3,PIK3CG,ANOS1,COL4A4,TPP1,STIL,TFDP1,NUP160,NECTIN3,FREM3,IQGAP1,MYCBP2,LATS1,KLHL12,SPARCL1,WNT2B,MAP2,FBXW11,PTPRO,AZIN2,NHSL1,PEMT,CHD5,JAK1,ACSL6,ULK2,KCNB1,RADIL,ZMIZ1,UGP2,RP1L1,GNPAT,ANKRD27,AIM2,ANK3,KITLG,ARID4B,MAP4K4,NR1I2,HIP1,RELN,MFHAS1,CRKL,MYO18B,TENT5A,ARHGEF10,FGD6,NDC1,PTPN13,VAV3,CD40*

			LG,SCN9A,DPYSL3,ATP2B1,ATRN,CCDC88A,ARNT2,APBA1,PLCE1,TACC2,MSH3,BAK1,TENM4,PPP3CA,NKD1,RBPJ,CYRIB,C3,RIN2,PDE6C,DLX6-AS1,CDH10,ADAMTS16,TAF10,TRIP12,MBD5,FRYL,ASH1L,RSPO2,DISC1,PROX1,BMP6,FMN1,CTSB,ASAP1,VSIG1,AQP8,CREBBP,ITGB1,BVES,DRC7,NFKBIZ,CORO1C,NEFL,NAV2,ATP6V1A,STK3,C1GALT1,HNF4G,MSR1,EXOSC3,AP2B1,TRPC4,ENPP2,BCL11B,CERS3,PLS3,PCSK2,TSPAN2,GFRA2,EPHB1,NAP1L1,PLPP4,CNN2,COL4A2,ARHGAP32,DYRK3,HDAC4,GDNF,KLHL1,MARCHF5,RBM45,IQCE,MYLK,UNC5C,CFAP69,ACSBG1,CSGALNACT1,FLG2,CFL1,DTYMK,KRT20,WDR7,HMG20A,CEP85L,PLEKHG4B,CCDC38,RBBP8,RNASEH2B,PHF2,RAI2,AFDN,LRP2,MTDH,SEMA6D,LIG1,EHMT1,WWC3,CAMK4,VWC2,TMEM108,BFSP1,GAB3,SEZ6L,GLI2,TGFBR3,UPRT,GTF2I,ATXN1,TMEM59L,ADAM17,SEMA4F,ELAVL1,TRAK1,STXBP1,IDH1,LMOD2,HDGFL2,NHLH1,NHSL2,SNRNP200,APC,GNA12,TTLL5,IL18R1,INO80,SULT2B1,PPARGC1B,COL27A1,PHLDB2,ROS1,RPL39L,ACER1,CDYL,CLPB,SPINK1,TAF5L,CTNND2,PACSIN2,RDH13,DPYSL2,METTL8,NSD2,SLC23A2,PAIP2,AARS1,PTPN11,PSMC2,MAPKAP1,COL15A1,RIC1,ACOX1,MAEL,CLPTM1,HSD17B4,EFHD1,RAI14,NRP2,PLXNC1,FOXJ2,NR3C1,DLX6,FSTL4,MTOR,CBLB,SNAP25,TUFT1,RP1,PIP5K1A,BTBD2,GH2,DISP1,RAB6B,SPRED1,ADAM10,GALC,CDH26,CRIM1,MEI4,RBM46,ABCB5,DPY19L2,CDHR3,PER2,ANKRD24,GLI3,KAT6A,BCOR,NTRK3,RXFP1,GAB2,DIS3L2,CTCF,PDCD10,PRAMEF11,ADGRG6,SLC4A10,FERMT2,GLUD1,DNM3,SYT1,PHIP,DENND5A,SS18,SFRP5,PPARA,SHISA6,NFASC,MAP2K5,PHF6,FGF7,PACSIN1,MGA,RTTN,EDA,CNTRL,TNFSF13B,MICAL2,COL14A1,RBM47,JARID2,CELSR1,HDAC2,TPO,FGD1,SEMA3D,PROM1,RRM1,CADM1,INPP5D,ATF6,SERPINE1,CCDC88C,PRAMEF4,SLC26A5,AKAP13,PKM,PTPN3,SCN8A,ST8SIA2,IRF8,HIVEP3,RFX4,ACTA2,FREM1,RIPOR2,XIRP2,KDM3A,TAOK1,UNC45A,HSPG2,SLCO4C1,GCNT1,USH1C,FMN2,NLRP14,DNAJC13,AFG2A
GO:0048856	anatomical structure development	1.2691656649631689e-8	RIT2,PBX4,MYO9B,XK,SEMA4D,MET,SCYL3,TEAD1,ATXN10,BRCA1,EIF4G3,STAU2,SIAH3,MMP16,DRD3,ELAPOR2,ATP11C,DNAH11,SDC2,FANCC,ARAP1,CCR3,ANKLE2,ACTR5,EIF4A3,NFE2L2,CYP19A1,SLC46A2,DTNB,GPR158,DEUP1,ATP2B2,PREX2,CSMD3,NTN4,SERP1,PLEKHM3,ENKUR,SRGAP2C,WIF1,FLI1,CSNK2A2,ESRRB,ZFHX2,ARHGAP26,ITGA2,GRIN2A,NELL2,IL1RAPL1,WDPCP,KIF5C,GPM6A,SPATA16,WNT16,KRT86,SETD3,ADCY1,RPS6KA2,PLPPR5,ZDHHC17,FIG4,EIF2AK2,CFAP47,FOXP2,KMT2D,LPAR3,FMR1,TXLNG,FOXN3,SH3TC2,CEP290,TACC1,SH3PXD2B,TUT4,AK8,PTPRQ,CDK13,NEO1,MYT1L,SRGAP2B,ITPKB,SYNE1,FBXL17,SLC9A4,GAS2,RUNX2,LRP12,TSPEAR,ESRP1,OSBP2,SLC39A10,RNF10,FAT1,PIK3R1,CFDP1,PGM5,SHTN1,BTD,CALD1,SH3GL2,MYLK3,DOCK7,XKR4,ROR1,PAX5,CRB1,CTTN,ARID1A,PARD6B,THEM

			IS,SMAD6,NYAP2,RFX2,MEF2C,FZR1,DBNL,RYK,MACF1,TAF4B,MALT1,WEE2,MTM1,TFDP2,KCNE1,UNC5B,PTPN12,PTPRZ1,PLN,EML1,NEDD4,RBBP9,DDAH1,PARVA,SECISBP2,SCML2,FGD4,ZNF609,PRDX1,ATG4B,MYO10,OLFM1,MID1,SLC12A2,OTX1,EFNB3,DLG3,FLOT2,ILK,LEF1,EYA1,MAPK14,MYBPC3,RAPGEF1,SH3GL3,ZNF516,SLC1A2,GABRB2,EDNRB,GRIN2B,ARSB,CRISPLD1,TBC1D20,SETD5,SNX2,PARP2,ZSWIM6,OVOL2,SLC4A7,DNAJB6,OMA1,PIEZO1,SLC9B2,NEB,TMEM100,VDR,PDPN,CREB3L2,RTN4,BBS2,CLSTN1,SPAG17,SCYL2,MAOB,SERPINE2,MYO3A,PLAAT3,PIK3CG,ANOS1,COL4A4,TPP1,STIL,TFDP1,NUP160,NECTIN3,FREM3,IQGAP1,MYCBP2,LATS1,KLHL12,SPARCL1,WNT2B,MAP2,FBXW11,PTPRO,PEMT,CHD5,JAK1,ACSL6,ULK2,KCNB1,RADIL,ZMIZ1,UGP2,RP1L1,GNPAT,ANKRD27,AIM2,ANK3,KITLG,ARID4B,MAP4K4,RELN,MFHAS1,CRKL,MYO18B,TENT5A,ARHGEF10,FGD6,PTPN13,VAV3,CD40LG,SCN9A,DPYSL3,ATP2B1,ATRN,CCDC88A,ARNT2,APBA1,PLCE1,TACC2,MSH3,BAK1,TENM4,PPP3CA,NKD1,RBPJ,CYRIB,C3,RIN2,PDE6C,DLX6-AS1,CDH10,ADAMTS16,TAF10,TRIP12,MBD5,FRYL,ASH1L,RSPO2,DISC1,PROX1,BMP6,FMN1,CTSB,ASAP1,VSIG1,AQP8,CREBBP,ITGB1,BVES,DRC7,NFKBIZ,CORO1C,NEFL,NAV2,STK3,C1GALT1,HNF4G,EXOSC3,AP2B1,TRPC4,ENPP2,BCL11B,CERS3,PLS3,PCSK2,TSPAN2,GFRA2,EPHB1,NAP1L1,PLPP4,CNN2,COL4A2,ARHGAP32,DYRK3,HDAC4,GDNF,KLHL1,MARCHF5,RBM45,IQCE,MYLK,UNC5C,CFAP69,ACSBG1,CSGALNACT1,FLG2,CFL1,DTYMK,KRT20,WDR7,HMG20A,CEP85L,PLEKHG4B,CCDC38,RBBP8,RNASEH2B,PHF2,RAI2,AFDN,LRP2,MTDH,SEMA6D,LIG1,EHMT1,CAMK4,VWC2,TMEM108,BFSP1,GAB3,SEZ6L,GLI2,TGFBR3,UPRT,GTF2I,ATXN1,TMEM59L,ADAM17,SEMA4F,TRAK1,STXBP1,IDH1,LMOD2,HDGFL2,NHLH1,APC,GNA12,TTLL5,IL18R1,INO80,SULT2B1,PPARGC1B,COL27A1,PHLDB2,ROS1,ACER1,CDYL,CLPB,SPINK1,CTNND2,PACSIN2,RDH13,DPYSL2,METTL8,NSD2,SLC23A2,AARS1,PTPN11,MAPKAP1,COL15A1,RIC1,MAEL,CLPTM1,HSD17B4,EFHD1,NRP2,PLXNC1,FOXJ2,NR3C1,DLX6,FSTL4,MTOR,CBLB,SNAP25,TUFT1,RP1,PIP5K1A,BTBD2,GH2,DISP1,RAB6B,SPRED1,ADAM10,GALC,CDH26,CRIM1,MEI4,RBM46,ABCB5,DPY19L2,CDHR3,PER2,ANKRD24,GLI3,KAT6A,BCOR,NTRK3,RXFP1,GAB2,CTCF,PDCD10,ADGRG6,SLC4A10,FERMT2,GLUD1,DNM3,SYT1,PHIP,DENND5A,SS18,SFRP5,PPARA,NFASC,MAP2K5,PHF6,FGF7,PACSIN1,RTTN,EDA,CNTRL,TNFSF13B,MICAL2,COL14A1,RBM47,JARID2,CELSR1,HDAC2,TPO,FGD1,SEMA3D,PROM1,RRM1,CADM1,INPP5D,ATF6,SERPINE1,CCDC88C,SLC26A5,AKAP13,PKM,PTPN3,SCN8A,ST8SIA2,IRF8,HIVEP3,RFX4,ACTA2,FREM1,RIPOR2,XIRP2,KDM3A,TAOK1,UNC45A,HSPG2,GCNT1,USH1C,FMN2,AFG2A
GO:0000902	cell morphog	1.933082034110866e-8	*MYO9B,XK,SEMA4D,MET,STAU2,SDC2,ARAP1,PREX2,NELL2,IL1RAPL1,WDPCP,KIF5C,GPM6A,*

	enesis		ADCY1,ZDHHC17,LPAR3,PTPRQ,NEO1,SYNE1,GAS2,FAT1,CFDP1,SHTN1,SH3GL2,DOCK7,CRB1,CTTN,PARD6B,NYAP2,MEF2C,DBNL,RYK,MACF1,UNC5B,PTPRZ1,NEDD4,PARVA,FGD4,MYO10,OLFM1,EFNB3,ILK,LEF1,MAPK14,TBC1D20,SNX2,VDR,PDPN,RTN4,ANOS1,IQGAP1,MYCBP2,LATS1,MAP2,PTPRO,ULK2,ANKRD27,ANK3,MAP4K4,RELN,FGD6,PPP3CA,CDH10,FRYL,DISC1,PROX1,VSIG1,ITGB1,BVES,CORO1C,NEFL,ENPP2,BCL11B,EPHB1,ARHGAP32,GDNF,UNC5C,CFL1,PLEKHG4B,LRP2,SEMA6D,TMEM108,GLI2,ADAM17,SEMA4F,TRAK1,STXBP1,GNA12,COL27A1,CTNND2,PACSIN2,SLC23A2,PTPN11,MAEL,NRP2,PLXNC1,FSTL4,SNAP25,CDH26,CDHR3,ANKRD24,GLI3,FERMT2,DNM3,SYT1,PHIP,SS18,NFASC,PACSIN1,FGD1,SEMA3D,CCDC88C,SLC26A5,ST8SIA2,RIPOR2
GO:0030030	cell projection organization	6.527351272990074e-7	RIT2,MYO9B,XK,SEMA4D,ATXN10,CEP350,STAU2,SDC2,ARAP1,NFE2L2,DEUP1,PREX2,CSMD3,FBF1,SRGAP2C,ITGA2,NELL2,IL1RAPL1,WDPCP,KIF5C,GPM6A,ADCY1,PLPPR5,ZDHHC17,CFAP47,LPAR3,FMR1,CEP290,OCLN,CFAP91,NEO1,SYNE1,LRP12,MPHOSPH9,PIK3R1,SHTN1,TMEM232,SH3GL2,DOCK7,ROR1,CTTN,PARD6B,NYAP2,RFX2,MEF2C,DBNL,RYK,MACF1,UNC5B,TBC1D31,PTPRZ1,NEDD4,PARVA,FGD4,MYO10,OLFM1,EFNB3,ILK,RAPGEF1,GRIN2B,UBXN10,ARSB,SNX2,PDPN,RTN4,BBS2,SPAG17,MYO3A,ANOS1,HTT,IQGAP1,MYCBP2,MAP2,PTPRO,ULK2,RP1L1,ANKRD27,ANK3,MAP4K4,RELN,CRKL,FGD6,VAV3,DPYSL3,CCDC88A,PLCE1,STK26,PPP3CA,ADAMTS16,FRYL,STON1,DISC1,ASAP1,ITGB1,DRC7,CORO1C,NEFL,ENPP2,BCL11B,GLCE,TSPAN2,EPHB1,ARHGAP32,GDNF,KLHL1,MYLK,UNC5C,CFAP69,CFL1,PLEKHG4B,CCDC38,LRP2,SEMA6D,TMEM108,CC2D2B,GLI2,ADAM17,SEMA4F,TRAK1,STXBP1,APC,CTNND2,PACSIN2,DPYSL2,RAB8B,SLC23A2,PTPN11,EFHD1,NRP2,PLXNC1,CEP162,FSTL4,MTOR,SNAP25,RP1,PIP5K1A,RAB6B,WASHC1,ERICH3,ANKRD24,GLI3,NTRK3,DNM3,SYT1,DENND5A,NFASC,PACSIN1,RTTN,HDAC2,FGD1,SEMA3D,CFAP161,GBF1,SSX2IP,ST8SIA2,TTC17,RFX4,RIPOR2,USH1C,PCNT
GO:0048731	system development	0.0000012371769154669782	RIT2,PBX4,XK,SEMA4D,MET,SCYL3,ATXN10,BRCA1,STAU2,MMP16,DRD3,ELAPOR2,DNAH11,SDC2,FANCC,CCR3,ANKLE2,EIF4A3,NFE2L2,CYP19A1,SLC46A2,DTNB,GPR158,ATP2B2,PREX2,CSMD3,NTN4,SERP1,SRGAP2C,WIF1,CSNK2A2,ZFHX2,ARHGAP26,ITGA2,GRIN2A,NELL2,IL1RAPL1,WDPCP,KIF5C,GPM6A,WNT16,ADCY1,RPS6KA2,PLPPR5,ZDHHC17,FIG4,FOXP2,LPAR3,FMR1,FOXN3,SH3TC2,CEP290,TACC1,SH3PXD2B,AK8,PTPRQ,NEO1,MYT1L,SRGAP2B,SYNE1,FBXL17,GAS2,RUNX2,LRP12,ESRP1,RNF10,FAT1,SHTN1,BTD,CALD1,SH3GL2,MYLK3,DOCK7,ROR1,PAX5,CRB1,CTTN,ARID1A,PARD6B,SMAD6,NYAP2,MEF2C,FZR1,DBNL,RYK,MACF1,TFDP2,KCNE1,UNC5B,PTPRZ1,PLN,EML1,NEDD4,RBBP9,DDAH1,PARVA,SECISBP2,ZNF609,PRDX1,OLFM1,SLC12A2,OTX1,EFNB3,ILK,LEF1,EYA1,

			MAPK14,MYBPC3,RAPGEF1,SH3GL3,SLC1A2,GABRB2,EDNRB,GRIN2B,ARSB,TBC1D20,SETD5,PARP2,ZSWIM6,OVOL2,SLC4A7,SLC9B2,NEB,TMEM100,VDR,PDPN,CREB3L2,RTN4,BBS2,CLSTN1,SCYL2,MAOB,SERPINE2,PLAAT3,PIK3CG,ANOS1,COL4A4,TPP1,STIL,NUP160,NECTIN3,IQGAP1,MYCBP2,WNT2B,MAP2,FBXW11,PTPRO,CHD5,JAK1,ACSL6,ULK2,KCNB1,ZMIZ1,UGP2,RP1L1,GNPAT,ANKRD27,AIM2,ANK3,KITLG,ARID4B,MAP4K4,RELN,CRKL,MYO18B,ARHGEF10,PTPN13,VAV3,CD40LG,DPYSL3,ATRN,CCDC88A,ARNT2,APBA1,PLCE1,TACC2,MSH3,BAK1,TENM4,PPP3CA,NKD1,RBPJ,C3,RIN2,PDE6C,DLX6-AS1,ADAMTS16,TAF10,MBD5,FRYL,ASH1L,RSPO2,DISC1,PROX1,BMP6,FMN1,ASAP1,ITGB1,BVES,NFKBIZ,CORO1C,NEFL,NAV2,STK3,C1GALT1,EXOSC3,AP2B1,TRPC4,BCL11B,PLS3,PCSK2,TSPAN2,GFRA2,EPHB1,NAP1L1,COL4A2,ARHGAP32,HDAC4,GDNF,KLHL1,RBM45,MYLK,UNC5C,ACSBG1,CSGALNACT1,CFL1,HMG20A,CEP85L,PLEKHG4B,PHF2,LRP2,MTDH,SEMA6D,VWC2,TMEM108,BFSP1,SEZ6L,GLI2,TGFBR3,GTF2I,ATXN1,TMEM59L,ADAM17,SEMA4F,TRAK1,STXBP1,IDH1,NHLH1,APC,TTLL5,PPARGC1B,COL27A1,CTNND2,RDH13,DPYSL2,NSD2,SLC23A2,AARS1,PTPN11,MAPKAP1,COL15A1,HSD17B4,EFHD1,NRP2,PLXNC1,FOXJ2,NR3C1,DLX6,FSTL4,MTOR,CBLB,SNAP25,RP1,BTBD2,DISP1,RAB6B,SPRED1,ADAM10,GALC,CRIM1,ABCB5,PER2,ANKRD24,GLI3,BCOR,NTRK3,RXFP1,PDCD10,ADGRG6,SLC4A10,GLUD1,DNM3,SYT1,DENND5A,SFRP5,PPARA,NFASC,MAP2K5,FGF7,PACSIN1,EDA,CNTRL,MICAL2,COL14A1,JARID2,CELSR1,HDAC2,SEMA3D,PROM1,RRM1,CADM1,ATF6,SERPINE1,AKAP13,PKM,PTPN3,SCN8A,ST8SIA2,RFX4,ACTA2,FREM1,RIPOR2,XIRP2,TAOK1,HSPG2,GCNT1,USH1C,AFG2A
GO:0048666	neuron development	0.0000015180219996070717	*RIT2,PBX4,XK,SEMA4D,SCYL3,ATXN10,STAU2,SDC2,NFE2L2,PREX2,CSMD3,NTN4,SRGAP2C,NELL2,IL1RAPL1,WDPCP,KIF5C,GPM6A,ADCY1,PLPPR5,ZDHHC17,FIG4,LPAR3,FMR1,CEP290,PTPRQ,NEO1,MYT1L,SYNE1,LRP12,SHTN1,SH3GL2,DOCK7,ROR1,CRB1,CTTN,PARD6B,NYAP2,MEF2C,DBNL,RYK,MACF1,UNC5B,PTPRZ1,NEDD4,SECISBP2,OLFM1,EFNB3,RAPGEF1,GABRB2,EDNRB,ARSB,SLC4A7,RTN4,SCYL2,ANOS1,IQGAP1,MYCBP2,MAP2,PTPRO,ULK2,KCNB1,ZMIZ1,RP1L1,ANKRD27,ANK3,MAP4K4,RELN,CRKL,DPYSL3,CCDC88A,TENM4,PPP3CA,C3,PDE6C,FRYL,DISC1,ASAP1,ITGB1,NEFL,BCL11B,TSPAN2,EPHB1,ARHGAP32,GDNF,KLHL1,UNC5C,CFL1,PLEKHG4B,LRP2,SEMA6D,TMEM108,GLI2,ADAM17,SEMA4F,TRAK1,STXBP1,CTNND2,RDH13,DPYSL2,SLC23A2,PTPN11,EFHD1,NRP2,PLXNC1,FSTL4,SNAP25,RP1,RAB6B,ANKRD24,GLI3,NTRK3,SLC4A10,DNM3,SYT1,DENND5A,NFASC,PACSIN1,HDAC2,SEMA3D,ST8SIA2,RIPOR2,TAOK1,USH1C*
GO:0009653	anatomical structu	0.0000020759378561674546	*PBX4,MYO9B,XK,SEMA4D,MET,BRCA1,STAU2,MMP16,DNAH11,SDC2,FANCC,ARAP1,CCR3,EIF4A3,NFE2L2,CYP19A1,PREX2,NTN4,SERP1,FLI*

	re morphogenesis		*1,ITGA2,NELL2,IL1RAPL1,WDPCP,KIF5C,GPM6A,WNT16,ADCY1,ZDHHC17,FIG4,LPAR3,FOXN3,CEP290,PTPRQ,NEO1,SYNE1,GAS2,RUNX2,TSPEAR,FAT1,CFDP1,PGM5,SHTN1,CALD1,SH3GL2,MYLK3,DOCK7,PAX5,CRB1,CTTN,PARD6B,SMAD6,NYAP2,RFX2,MEF2C,DBNL,RYK,MACF1,UNC5B,PTPRZ1,PLN,NEDD4,DDAH1,PARVA,SCML2,FGD4,MYO10,OLFM1,SLC12A2,OTX1,EFNB3,DLG3,ILK,LEF1,EYA1,MAPK14,MYBPC3,CRISPLD1,TBC1D20,SNX2,OVOL2,DNAJB6,OMA1,NEB,TMEM100,VDR,PDPN,RTN4,BBS2,SERPINE2,MYO3A,PIK3CG,ANOS1,STIL,NECTIN3,FREM3,IQGAP1,MYCBP2,LATS1,KLHL12,WNT2B,MAP2,FBXW11,PTPRO,JAK1,ULK2,ZMIZ1,GNPAT,ANKRD27,ANK3,MAP4K4,RELN,CRKL,MYO18B,FGD6,VAV3,BAK1,TENM4,PPP3CA,NKD1,RBPJ,CYRIB,C3,RIN2,PDE6C,CDH10,ADAMTS16,TAF10,FRYL,ASH1L,RSPO2,DISC1,PROX1,BMP6,FMN1,VSIG1,CREBBP,ITGB1,BVES,CORO1C,NEFL,STK3,C1GALT1,ENPP2,BCL11B,EPHB1,COL4A2,ARHGAP32,GDNF,MARCHF5,IQCE,MYLK,UNC5C,CSGALNACT1,FLG2,CFL1,PLEKHG4B,LRP2,MTDH,SEMA6D,LIG1,TMEM108,GLI2,TGFBR3,GTF2I,TMEM59L,ADAM17,SEMA4F,TRAK1,STXBP1,LMOD2,APC,GNA12,PPARGC1B,COL27A1,PHLDB2,CTNND2,PACSIN2,RDH13,NSD2,SLC23A2,PTPN11,COL15A1,MAEL,NRP2,PLXNC1,FOXJ2,NR3C1,DLX6,FSTL4,MTOR,SNAP25,TUFT1,RP1,SPRED1,CDH26,RBM46,CDHR3,ANKRD24,GLI3,BCOR,RXFP1,PDCD10,ADGRG6,SLC4A10,FERMT2,DNM3,SYT1,PHIP,SS18,SFRP5,PPARA,NFASC,MAP2K5,FGF7,PACSIN1,EDA,TNFSF13B,MICAL2,CELSR1,HDAC2,FGD1,SEMA3D,PROM1,SERPINE1,CCDC88C,SLC26A5,AKAP13,PKM,ST8SIA2,ACTA2,FREM1,RIPOR2,XIRP2,HSPG2,GCNT1,USH1C*
GO:0030182	neuron differentiation	0.0000023468755989614727	*RIT2,PBX4,XK,SEMA4D,MET,SCYL3,ATXN10,STAU2,SDC2,NFE2L2,DTNB,ATP2B2,PREX2,CSMD3,NTN4,SRGAP2C,ZFHX2,NELL2,IL1RAPL1,WDPCP,KIF5C,GPM6A,WNT16,ADCY1,PLPPR5,ZDHHC17,FIG4,LPAR3,FMR1,CEP290,PTPRQ,NEO1,MYT1L,SYNE1,RUNX2,LRP12,ESRP1,SHTN1,SH3GL2,DOCK7,ROR1,CRB1,CTTN,PARD6B,NYAP2,MEF2C,DBNL,RYK,MACF1,UNC5B,PTPRZ1,NEDD4,SECISBP2,OLFM1,EFNB3,EYA1,RAPGEF1,SH3GL3,GABRB2,EDNRB,ARSB,ZSWIM6,SLC4A7,RTN4,SCYL2,ANOS1,IQGAP1,MYCBP2,WNT2B,MAP2,PTPRO,CHD5,ULK2,KCNB1,ZMIZ1,RP1L1,ANKRD27,ANK3,MAP4K4,RELN,CRKL,DPYSL3,CCDC88A,TENM4,PPP3CA,NKD1,RBPJ,C3,PDE6C,FRYL,RSPO2,DISC1,PROX1,BMP6,ASAP1,ITGB1,NEFL,BCL11B,TSPAN2,EPHB1,ARHGAP32,GDNF,KLHL1,UNC5C,CFL1,HMG20A,PLEKHG4B,LRP2,SEMA6D,VWC2,TMEM108,GLI2,ADAM17,SEMA4F,TRAK1,STXBP1,CTNND2,RDH13,DPYSL2,SLC23A2,PTPN11,EFHD1,NRP2,PLXNC1,FSTL4,SNAP25,RP1,RAB6B,ANKRD24,GLI3,NTRK3,SLC4A10,DNM3,SYT1,DENND5A,NFASC,PACSIN1,HDAC2,SEMA3D,PROM1,ST8SIA2,RIPOR2,TAOK1,USH1C*
GO:0048	generat	0.0000029444610	*RIT2,PBX4,XK,SEMA4D,MET,SCYL3,ATXN10,S*

699	ion of neurons	506913685	*TAU2,SDC2,NFE2L2,DTNB,ATP2B2,PREX2,CSMD3,NTN4,SRGAP2C,ZFHX2,NELL2,IL1RAPL1,WDPCP,KIF5C,GPM6A,WNT16,ADCY1,PLPPR5,ZDHHC17,FIG4,LPAR3,FMR1,CEP290,PTPRQ,NEO1,MYT1L,SYNE1,RUNX2,LRP12,ESRP1,SHTN1,SH3GL2,DOCK7,ROR1,CRB1,CTTN,PARD6B,NYAP2,MEF2C,DBNL,RYK,MACF1,UNC5B,PTPRZ1,EML1,NEDD4,SECISBP2,ZNF609,OLFM1,EFNB3,LEF1,EYA1,RAPGEF1,SH3GL3,GABRB2,EDNRB,ARSB,ZSWIM6,SLC4A7,RTN4,SCYL2,ANOS1,IQGAP1,MYCBP2,WNT2B,MAP2,PTPRO,CHD5,ACSL6,ULK2,KCNB1,ZMIZ1,RP1L1,ANKRD27,ANK3,MAP4K4,RELN,CRKL,DPYSL3,CCDC88A,TENM4,PPP3CA,NKD1,RBPJ,C3,PDE6C,FRYL,RSPO2,DISC1,PROX1,BMP6,ASAP1,ITGB1,NEFL,BCL11B,TSPAN2,EPHB1,ARHGAP32,GDNF,KLHL1,UNC5C,CFL1,HMG20A,CEP85L,PLEKHG4B,LRP2,SEMA6D,VWC2,TMEM108,GLI2,ADAM17,SEMA4F,TRAK1,STXBP1,CTNND2,RDH13,DPYSL2,SLC23A2,PTPN11,EFHD1,NRP2,PLXNC1,FSTL4,SNAP25,RP1,RAB6B,ANKRD24,GLI3,NTRK3,SLC4A10,DNM3,SYT1,DENND5A,NFASC,PACSIN1,CELSR1,HDAC2,SEMA3D,PROM1,ST8SIA2,RIPOR2,TAOK1,USH1C*
GO:0007264	small GTPase-mediated signal transduction	0.0000030462751871644348	*RIT2,MYO9B,CYTH1,SEMA4D,MET,ARAP1,ITSN1,PREX2,ARHGAP26,F2RL2,RASA2,ITPKB,SHTN1,USP50,DOCK7,USP8,PSD3,DBNL,DOCK8,FGD4,HACE1,RAPGEF1,ARHGEF3,DENND4A,PDPN,RTN4,ARHGEF11,PIK3CG,ARHGAP6,KITLG,MAP4K4,RELN,CRKL,ARHGEF10,VAV3,CCDC88A,PLCE1,CYRIB,RIN2,PLEKHG7,DENND1A,RALGPS1,ITGB1,CHN2,SH2D3C,DOCK4,CYTH4,ARHGAP32,CFL1,PLEKHG4B,PLEKHG1,GNA12,MAPKAP1,SIPA1L2,ARHGAP28,KSR1,DOCK9,CELSR1,FGD1,GBF1,CCDC88C,CYTH3,AKAP13,ARFGEF1,RALGAPA1,SSX2IP,RIPOR2*
GO:0051056	regulation of small GTPase mediated signal transduction	0.0000034664270499214386	*RIT2,MYO9B,CYTH1,SEMA4D,MET,ARAP1,ITSN1,PREX2,ARHGAP26,F2RL2,RASA2,ITPKB,PSD3,DOCK8,FGD4,RAPGEF1,ARHGEF3,DENND4A,RTN4,ARHGEF11,PIK3CG,ARHGAP6,KITLG,MAP4K4,RELN,CRKL,ARHGEF10,VAV3,CYRIB,DENND1A,RALGPS1,ITGB1,CHN2,CYTH4,ARHGAP32,PLEKHG4B,PLEKHG1,MAPKAP1,SIPA1L2,ARHGAP28,FGD1,GBF1,CYTH3,AKAP13,ARFGEF1,RALGAPA1,SSX2IP,RIPOR2*
GO:0007399	nervous system development	0.000003780788872518793	*RIT2,PBX4,XK,SEMA4D,MET,SCYL3,ATXN10,STAU2,DRD3,ELAPOR2,DNAH11,SDC2,FANCC,ANKLE2,NFE2L2,DTNB,GPR158,ATP2B2,PREX2,CSMD3,NTN4,SRGAP2C,CSNK2A2,ZFHX2,ARHGAP26,GRIN2A,NELL2,IL1RAPL1,WDPCP,KIF5C,GPM6A,WNT16,ADCY1,PLPPR5,ZDHHC17,FIG4,FOXP2,LPAR3,FMR1,SH3TC2,CEP290,TACC1,AK8,PTPRQ,NEO1,MYT1L,SRGAP2B,SYNE1,FBXL17,RUNX2,LRP12,ESRP1,RNF10,SHTN1,BTD,SH3GL2,DOCK7,ROR1,PAX5,CRB1,CTTN,ARID1A,PARD6B,NYAP2,MEF2C,DBNL,RYK,MACF1,KCNE1,UNC5B,PTPRZ1,EML1,NEDD4,SECISBP2,ZNF609,OLFM1,OTX1,EFNB3,ILK,LEF1,EYA1,RAPGEF1,SH3GL3,SLC1A2,GABRB2,EDNRB,GRIN2B,ARSB,SETD5,ZSWIM6,OVOL2,SLC4A7,RTN4,BBS2,CLSTN1,SCYL2,MAOB,SERPINE2,ANOS1,T*

			PP1,STIL,IQGAP1,MYCBP2,WNT2B,MAP2,FBXW11,PTPRO,CHD5,ACSL6,ULK2,KCNB1,ZMIZ1,UGP2,RP1L1,GNPAT,ANKRD27,AIM2,ANK3,MAP4K4,RELN,CRKL,ARHGEF10,PTPN13,DPYSL3,ATRN,CCDC88A,ARNT2,APBA1,TACC2,TENM4,PPP3CA,NKD1,RBPJ,C3,PDE6C,DLX6-AS1,MBD5,FRYL,RSPO2,DISC1,PROX1,BMP6,ASAP1,ITGB1,CORO1C,NEFL,NAV2,STK3,TRPC4,BCL11B,PCSK2,TSPAN2,GFRA2,EPHB1,NAP1L1,ARHGAP32,HDAC4,GDNF,KLHL1,RBM45,UNC5C,ACSBG1,CFL1,HMG20A,CEP85L,PLEKHG4B,LRP2,SEMA6D,VWC2,TMEM108,SEZ6L,GLI2,ATXN1,ADAM17,SEMA4F,TRAK1,STXBP1,NHLH1,APC,CTNND2,RDH13,DPYSL2,SLC23A2,AARS1,PTPN11,MAPKAP1,EFHD1,NRP2,PLXNC1,NR3C1,DLX6,FSTL4,MTOR,SNAP25,RP1,BTBD2,RAB6B,GALC,CRIM1,PER2,ANKRD24,GLI3,NTRK3,ADGRG6,SLC4A10,GLUD1,DNM3,SYT1,DENND5A,NFASC,PACSIN1,JARID2,CELSR1,HDAC2,SEMA3D,PROM1,RRM1,SCN8A,ST8SIA2,RFX4,RIPOR2,TAOK1,HSPG2,USH1C,AFG2A
GO:0065007	biological regulation	0.000004336843252103668	RIT2,SLMAP,KLHL13,ABCB7,PBX4,NHLRC3,MYO9B,GPSM2,COL4A5,ERG,PARN,CYTH1,STK16,XK,SEMA4D,MET,MAX,TEAD1,BRCA1,EIF4G3,ACSM3,BAZ1B,CEP350,STAU2,SIAH3,DUS3L,PIWIL3,CHMP1B,MIR654,DRD3,ELAPOR2,ATP11C,ZNF534,TOX3,DNAH11,EXOC2,PTPN5,TACR1,SNX25,SDC2,ABHD12,GPHB5,CIITA,ARAP1,CCR3,ANKLE2,CEP85,RPRD1B,AHNAK,ACTR5,EIF4A3,DLEC1,NFE2L2,SCHIP1,MIR376A2,PARG,OLFML2A,UIMC1,TFEC,NFYC,CYP19A1,SLC46A2,DTNB,GPR158,VRK2,ITSN1,OAS1,ATP2B2,PREX2,SCRN1,ELAVL2,CSMD3,FBXL20,NTN4,SERP1,POLR1A,RAB11FIP4,HSFX4,SNTA1,CLDN10,ASZ1,NUB1,SRGAP2C,WIF1,FLI1,CSNK2A2,ESRRB,ZFHX2,ARHGAP26,TDRD9,LINC-PINT,TRHDE,RAB44,SETBP1,ZBTB7C,ITGA2,GRIN2A,IL1RAPL1,WDPCP,BLK,GPM6A,WNT16,SETD3,ADCY1,GRID1,RPS6KA2,PLPPR5,TRIM24,ZDHHC17,FIG4,EIF2AK2,FOXP2,F2RL2,KMT2D,LPAR3,ANO4,FMR1,TXLNG,FOXN3,SP110,SH3BP2,SH3TC2,CEP290,SHQ1,TACC1,RAD9B,OCLN,TUT4,FASTKD5,ITGBL1,NETO1,GBE1,SNX5,CDK13,SNX32,PCGEM1,NEO1,MYT1L,RASA2,ITPKB,PLEKHB2,TCERG1,F10,SRGAP1,DIO2,CABIN1,SYNE1,PSMD10,FBXL17,EOLA1,SLC9A4,DHX29,GAS2,GPAM,NABP1,IRF2,RUNX2,LRP12,TSPEAR,ESRP1,HSFX3,BRMS1L,RGS12,CBX3,SLC39A10,CDCA2,ZNF496,KCTD8,ZNF550,MPHOSPH9,RNF10,FAT1,SLC2A2,PIK3R1,STAMBPL1,IL17B,ABCB11,SARNP,CFDP1,COL26A1,SHTN1,ATG12,MASP1,USP50,CD200R1,POLR3F,SH3GL2,CRTC1,PITPNC1,MYLK3,SLC30A3,DOCK7,PDK3,XKR4,ROR1,SCAP,PAX5,AAK1,CRB1,CTTN,UTP15,PASK,USP8,GPR75,ARID1A,PARD6B,THEMIS,SMAD6,OR2AJ1,RYBP,NYAP2,PSD3,ABCA13,RFX2,MEF2C,ZNF519,FZR1,BAIAP2L1,DBNL,ELP4,RYK,MACF1,PXT1,TAF4B,MALT1,WEE2,MTM1,TFDP2,KCNE1,PTPRE,UNC5B,ZSCAN5A,SERINC3,ZNF347,PTPN12,PTPRZ1,CD55,PLN,CTDSPL2,SHISA3,NEDD4,NEK4,RBBP9,CAC

			NA1E,FLYWCH1,LRRC38,DOCK8,CYSLTR2,DDAH1,PARVA,SECISBP2,URI1,SLC9C1,SCML2,CHD6,FGD4,ZNF609,PRDX1,COX17,HACE1,AEBP2,DSC2,SERPINA1,MYO10,OLFM1,CRHR2,MID1,POGK,TSHZ2,SLC12A2,OTX1,EFNB3,TRIM37,DLG3,TRAT1,FLOT2,PVRIG,SV2B,ILK,LEF1,EYA1,ZNF816-ZNF321P,MAPK14,MYBPC3,ZFHX4,RAPGEF1,GPRC6A,SH3GL3,ZNF516,CERKL,SLC1A2,ARHGEF3,GABRB2,EDNRB,GRIN2B,SPDYE3,ARSB,GPR141,IFNGR2,DENND4A,TBC1D20,OXR1,SFMBT2,SETD5,SCAMP5,IL17RB,PARP2,OVOL2,RDH16,MIR376B,FCHSD2,SLC4A7,DNAJB6,MTCL3,OMA1,MTUS1,GABRA6,LEMD3,KHDRBS2,PIEZO1,SOAT1,OR2T11,SLC9B2,NEB,CSRNP3,MRGPRX1,TMEM100,LYPD6,VDR,NEK11,PDPN,CREB3L2,RTN4,GNL3L,CACNA1D,ARHGEF11,PRKAA2,IVNS1ABP,SGPP2,DPT,BBS2,CLSTN1,ZNF155,SERGEF,SCYL2,SKAP1,MAOB,SKIC3,SERPINE2,MYO3A,PLAAT3,IL12RB2,PIK3CG,ANKFN1,STIL,TFDP1,IL1R2,ATP6V1E2,HTT,IQGAP1,MYCBP2,WIPI1,LATS1,ZSCAN25,KLHL12,LOC349160,SNX30,FANCI,OR9Q1,OR4K17,SPARCL1,WNT2B,TMDD1,TNFRSF10B,MAP2,FBXW11,MTCL1,PTPRO,HSPH1,AZIN2,IYD,MIR518C,SMG6,HLA-DMA,PEMT,ARHGAP6,CHD5,JAK1,ITGA9,TOR1AIP2,ULK2,KCNB1,RADIL,ZMIZ1,RP1L1,HSD17B12,GRM4,ANKRD27,AIM2,ANK3,GTF2IRD1,ZNF19,PPP1R14C,KITLG,SV2C,ARID4B,GMDS,ZNF326,MAP4K4,CNIH3,PLEKHM2,NR1I2,ZNF266,HIP1,RAET1E,RFX7,RELN,MFHAS1,OR51B5,FBXO32,MTMR3,CRKL,TENT5A,ARHGEF10,FGD6,PTPN13,SNAP91,ZNF816,VAV3,RPS6KC1,INPP5A,CD40LG,VRK1,GPAT3,ZNF420,DPYSL3,ATP2B1,SPRING1,SLC16A10,ATRN,CCDC88A,SLC35B4,ARNT2,APBA1,HPSE2,PLCE1,TNIP3,DCAF5,GNAL,STK32C,MSH3,BAK1,CASTOR2,LAX1,CACTIN,STK26,MIR520C,TENM4,DCP1A,PPP3CA,CHRNA5,MIR526A1,MKNK1,DYSF,NKD1,RBPJ,CYRIB,SPDYE17,C3,RIN2,PDE6C,PLEKHG7,DLX6-AS1,ASCC1,DENND1A,ENSG00000290317,OR52D1,ADAMTS16,TASP1,MICU1,TAF10,TRIP12,MBD5,BACE2,KDM5A,ZNF549,ZNF320,CNOT6,PKIB,ASAH2,STON1,ASH1L,RSPO2,ZNF747,ZNF805,OR5K1,OR13C9,DISC1,PROX1,BMP6,FMN1,ZNF891,RALGPS1,CTSB,ASAP1,LRRC2,AQP8,CREBBP,ITGB1,BVES,ZNF827,NFKBIZ,CORO1C,KANK3,NSG2,SPIDR,NEFL,NAV2,ATP6V1A,STK3,CHN2,FCAR,HNF4G,MSR1,EXOSC3,AP2B1,GABRG3,SH2D3C,APTX,RAMP3,GNG2,ZNF705A,FRS3,LIMS1,ENPP2,DOCK4,ACKR2,UBL4B,MCTP2,BCL11B,GLCE,PCSK2,KCNIP1,PWWP2A,LOC105373170,GFRA2,TBC1D10A,EPHB1,SSBP2,CASP8AP2,RPH3A,CTTNBP2,CYTH4,NAP1L1,PLPP4,UBR1,CNN2,COL4A2,SLC1A6,TCF7L1,ARHGAP32,DYRK3,IP6K2,CHRNA6,HDAC4,ZNF582,GDNF,FYB1,CALCB,MARCHF5,MYLK,UNC5C,CFAP69,TLR7,P3H2,CFL1,KRT20,SLAIN2,HMG20A,PLEKHG4B,RBBP8,MED13,RNASEH2B,PHF2,TAAR2,AFDN,CYTIP,LRP2,MTDH,SEMA6D,EHMT1,ZDH

			HC13,WWC3,IL17RA,CAMK4,VWC2,RPA3,TMEM108,TPTE,LCP2,GAB3,SEZ6L,SLC39A8,GLI2,TAS2R13,TGFBR3,MAP3K7,UPRT,GTF2I,ATXN1,ADAM17,SEMA4F,ELAVL1,TRAK1,STXBP1,OR6N1,IDH1,NUAK2,LMOD2,ABCC1,HDGFL2,PLEKHG1,RBAK-RBAKDN,NHLH1,ELP2,CYP8B1,APC,GNA12,IL18R1,INO80,SULT2B1,SPTSSB,PEAR1,CDA,PPARGC1B,PHLDB2,ESR2,MIR376C,ERC1,ZNF440,ROS1,RUBCNL,AGO3,C9,PVR,MOB3B,ACER1,IQGAP2,CDYL,RLF,CLPB,SPINK1,ANKRA2,TAF5L,CTNND2,ERI1,RASSF3,PACSIN2,RDH13,TIA1,DPYSL2,SYNPO2,GIMAP8,RAB8B,METTL8,NSD2,GRM8,SLC23A2,PAIP2,AARS1,PTPN11,JADE3,CARD18,PSMC2,MAPKAP1,COL15A1,EVC2,GON4L,RIC1,MGAT5,MAEL,NUP153,CLPTM1,HSD17B4,EFHD1,PIGR,NRP2,SIPA1L2,CAVIN1,PLXNC1,TMEM165,FOXJ2,NR3C1,DLX6,MIR300,BORCS5,FSTL4,ZC3HAV1,ARHGAP28,MTOR,KSR1,CBLB,FAF1,SNAP25,TUFT1,RP1,DEFB124,ADH7,CHST4,OR11H6,ZNF221,PIP5K1A,GH2,DISP1,TBXAS1,ASCC2,SPRED1,ADAM10,GNG4,CNTNAP4,WASHC1,PDS5B,CRIM1,RBM46,ABCB5,RNF115,CDHR3,PER2,PLEKHH2,GLI3,KAT6A,BCOR,KCNK12,NTRK3,RXFP1,TADA1,GAB2,DIS3L2,CTCF,PDE1A,PDCD10,PRAMEF11,TFG,EPHX2,HHAT,CREB5,CCDC68,ADGRG6,SLC4A10,GOLGA7,FERMT2,GPR82,TSPOAP1,GLUD1,DNM3,SYT1,PHIP,EPB41,DENND5A,SS18,SFRP5,DOCK9,ZYG11B,RFC2,PPARA,CD244,SHISA6,HDX,SCPEP1,MAP2K5,ZNF229,PHF6,HMGB4,EPB41L2,FGF7,TENT5D,PACSIN1,STXBP6,MGA,CACNA1A,EDA,LIMCH1,TNFSF13B,PTH2R,ELOVL6,MICAL2,COL14A1,RBM47,EIF2A,JARID2,CELSR1,HDAC2,FIRRM,TPO,FGD1,TRIM44,INSYN2B,TRIM39,SEMA3D,PROM1,OR2T8,NPSR1,EIF4E3,ACP3,KCNH5,RRM1,MAGEA1,CADM1,INPP5D,CLYBL,OR4C6,SPDYE9,ATF6,PRKAR1B,GBF1,SERPINE1,CCDC88C,BACH1,CUL9,PRAMEF4,EWSR1,TM7SF3,CYTH3,SLC26A5,AKAP13,HBS1L,PKM,GCG,ARFGEF1,ATP12A,TKFC,LINC02218,PTPN3,STAC,SCN8A,RALGAPA1,NNT,SSX2IP,ST8SIA2,ELP1,IRF8,HIVEP3,RFX4,OR6K6,ZNF30,REM1,ACTA2,RIPOR2,KCNK5,FUNDC2,KDM3A,CAST,OR2L3,TAOK1,GPR39,ADGRA3,CASK,HSPG2,GCNT1,PRDM7,USH1C,FMN2,UBXN2A,NLRP14,PCNT,DNAJC13,PSME3IP1
GO:0120036	plasma membrane bounded cell projection organization	0.000005923776579943129	*RIT2,MYO9B,XK,SEMA4D,ATXN10,CEP350,STAU2,SDC2,ARAP1,NFE2L2,PREX2,CSMD3,FBF1,SRGAP2C,NELL2,IL1RAPL1,WDPCP,KIF5C,GPM6A,ADCY1,PLPPR5,ZDHHC17,CFAP47,LPAR3,FMR1,CEP290,OCLN,CFAP91,NEO1,SYNE1,LRP12,MPHOSPH9,PIK3R1,SHTN1,SH3GL2,DOCK7,ROR1,CTTN,PARD6B,NYAP2,RFX2,MEF2C,DBNL,RYK,MACF1,UNC5B,TBC1D31,PTPRZ1,NEDD4,PARVA,FGD4,MYO10,OLFM1,EFNB3,RAPGEF1,GRIN2B,UBXN10,ARSB,SNX2,PDPN,RTN4,BBS2,SPAG17,MYO3A,ANOS1,HTT,IQGAP1,MYCBP2,MAP2,PTPRO,ULK2,RP1L1,ANKRD27,ANK3,MAP4K4,RELN,CRKL,FGD6,VAV3,DPYSL3,CCDC88A,PLCE1,STK26,PPP3CA,ADAMTS16,FRYL,STON1,*

			DISC1,ASAP1,ITGB1,DRC7,CORO1C,NEFL,ENPP2,BCL11B,TSPAN2,EPHB1,ARHGAP32,GDNF,KLHL1,MYLK,UNC5C,CFAP69,CFL1,PLEKHG4B,CCDC38,LRP2,SEMA6D,TMEM108,CC2D2B,GLI2,ADAM17,SEMA4F,TRAK1,STXBP1,APC,CTNND2,DPYSL2,SLC23A2,PTPN11,EFHD1,NRP2,PLXNC1,CEP162,FSTL4,MTOR,SNAP25,RP1,PIP5K1A,RAB6B,WASHC1,ERICH3,ANKRD24,GLI3,NTRK3,DNM3,SYT1,DENND5A,NFASC,PACSIN1,RTTN,HDAC2,FGD1,SEMA3D,CFAP161,GBF1,SSX2IP,ST8SIA2,TTC17,RFX4,RIPOR2,USH1C,PCNT
GO:0048518	positive regulation of biological process	0.000007729543649520333	*RIT2,ABCB7,PBX4,GPSM2,ERG,PARN,STK16,SEMA4D,MET,MAX,TEAD1,BRCA1,EIF4G3,BAZ1B,STAU2,DRD3,ELAPOR2,ATP11C,TOX3,TACR1,CIITA,ARAP1,CCR3,RPRD1B,AHNAK,ACTR5,EIF4A3,NFE2L2,SCHIP1,UIMC1,TFEC,NFYC,CYP19A1,SLC46A2,GPR158,OAS1,SERP1,NUB1,SRGAP2C,WIF1,FLI1,CSNK2A2,ESRRB,ZBTB7C,ITGA2,GRIN2A,IL1RAPL1,BLK,GPM6A,WNT16,SETD3,ADCY1,RPS6KA2,PLPPR5,TRIM24,ZDHHC17,EIF2AK2,F2RL2,KMT2D,LPAR3,FMR1,CEP290,SHQ1,TACC1,OCLN,TUT4,NETO1,SNX5,CDK13,NEO1,ITPKB,TCERG1,F10,DIO2,SYNE1,PSMD10,DHX29,GPAM,IRF2,RUNX2,BRMS1L,SLC39A10,ZNF496,RNF10,FAT1,SLC2A2,PIK3R1,STAMBPL1,IL17B,ABCB11,COL26A1,SHTN1,ATG12,MASP1,USP50,POLR3F,CRTC1,MYLK3,SLC30A3,DOCK7,ROR1,SCAP,PAX5,AAK1,CTTN,UTP15,PASK,USP8,ARID1A,THEMIS,SMAD6,RYBP,ABCA13,RFX2,MEF2C,FZR1,BAIAP2L1,RYK,MACF1,PXT1,TAF4B,MALT1,WEE2,MTM1,TFDP2,KCNE1,UNC5B,SERINC3,PTPRZ1,CD55,CTDSPL2,NEDD4,NEK4,RBBP9,LRRC38,DOCK8,DDAH1,SECISBP2,CHD6,ZNF609,COX17,MYO10,OLFM1,CRHR2,MID1,SLC12A2,OTX1,EFNB3,TRIM37,DLG3,TRAT1,FLOT2,PVRIG,ILK,LEF1,EYA1,MAPK14,RAPGEF1,SH3GL3,ZNF516,SLC1A2,ARHGEF3,EDNRB,GRIN2B,SPDYE3,ARSB,TBC1D20,SCAMP5,PARP2,OVOL2,FCHSD2,OMA1,PIEZO1,SOAT1,SLC9B2,CSRNP3,TMEM100,LYPD6,VDR,PDPN,CREB3L2,RTN4,GNL3L,CACNA1D,ARHGEF11,PRKAA2,BBS2,CLSTN1,SKAP1,MAOB,SERPINE2,MYO3A,IL12RB2,PIK3CG,STIL,TFDP1,HTT,IQGAP1,MYCBP2,WIPI1,LATS1,SNX30,FANCI,WNT2B,TNFRSF10B,MAP2,FBXW11,MTCL1,HSPH1,AZIN2,HLA-DMA,PEMT,CHD5,JAK1,ULK2,KCNB1,ZMIZ1,HSD17B12,GRM4,ANKRD27,AIM2,ANK3,KITLG,ARID4B,MAP4K4,NR1I2,HIP1,RAET1E,RFX7,RELN,MFHAS1,CRKL,TENT5A,ARHGEF10,VAV3,CD40LG,VRK1,DPYSL3,ATP2B1,SPRING1,CCDC88A,ARNT2,HPSE2,PLCE1,TNIP3,BAK1,LAX1,CACTIN,TENM4,DCP1A,PPP3CA,NKD1,RBPJ,CYRIB,SPDYE17,C3,RIN2,DLX6-AS1,TASP1,TAF10,MBD5,KDM5A,CNOT6,PKIB,ASH1L,RSPO2,DISC1,PROX1,BMP6,FMN1,ASAP1,CREBBP,ITGB1,BVES,ZNF827,NFKBIZ,CORO1C,SPIDR,NEFL,STK3,FCAR,HNF4G,MSR1,EXOSC3,SH2D3C,RAMP3,LIMS1,ENPP2,DOCK4,UBL4B,BCL11B,GLCE,PWWP2A,TBC1D10A,EPHB1,SSBP2,NAP1L1,PLPP4,CNN2,ARHGAP32,DYRK3,*

			IP6K2,HDAC4,GDNF,FYB1,MARCHF5,MYLK,UNC5C,CFAP69,TLR7,CFL1,SLAIN2,MED13,RNASEH2B,PHF2,AFDN,LRP2,MTDH,SEMA6D,EHMT1,ZDHHC13,IL17RA,CAMK4,VWC2,TMEM108,LCP2,GLI2,TAS2R13,TGFBR3,MAP3K7,GTF2I,ADAM17,SEMA4F,ELAVL1,TRAK1,STXBP1,LMOD2,ABCC1,HDGFL2,NHLH1,CYP8B1,APC,IL18R1,INO80,SULT2B1,PEAR1,PPARGC1B,PHLDB2,ESR2,AGO3,C9,PVR,MOB3B,RLF,CLPB,SPINK1,TAF5L,TIA1,SYNPO2,RAB8B,METTL8,NSD2,SLC23A2,PAIP2,PTPN11,PSMC2,MAPKAP1,MGAT5,NRP2,CAVIN1,PLXNC1,FOXJ2,NR3C1,DLX6,BORCS5,ZC3HAV1,MTOR,KSR1,CBLB,FAF1,SNAP25,RP1,DEFB124,CHST4,GH2,TBXAS1,SPRED1,ADAM10,WASHC1,RBM46,PER2,GLI3,KAT6A,NTRK3,TADA1,GAB2,DIS3L2,CTCF,PDCD10,PRAMEF11,TFG,EPHX2,CREB5,FERMT2,GLUD1,DNM3,SYT1,PHIP,EPB41,SS18,ZYG11B,RFC2,PPARA,CD244,MAP2K5,ZNF229,EPB41L2,FGF7,TENT5D,PACSIN1,MGA,EDA,LIMCH1,TNFSF13B,PTH2R,ELOVL6,MICAL2,RBM47,EIF2A,HDAC2,TRIM44,TRIM39,SEMA3D,PROM1,NPSR1,ACP3,RRM1,CADM1,INPP5D,CLYBL,SPDYE9,ATF6,PRKAR1B,SERPINE1,BACH1,PRAMEF4,TM7SF3,CYTH3,SLC26A5,AKAP13,PKM,GCG,ARFGEF1,TKFC,LINC02218,STAC,NNT,ST8SIA2,IRF8,HIVEP3,RFX4,ACTA2,RIPOR2,KDM3A,TAOK1,CASK,HSPG2,GCNT1,PRDM7,FMN2,UBXN2A,PCNT
GO:0010646	regulation of cell communication	0.00000899978294194776	*RIT2,MYO9B,CYTH1,SEMA4D,MET,BRCA1,STAU2,DRD3,ELAPOR2,TACR1,SNX25,GPHB5,ARAP1,EIF4A3,NFE2L2,SCHIP1,CYP19A1,SLC46A2,GPR158,VRK2,ITSN1,OAS1,PREX2,FBXL20,SERP1,WIF1,CSNK2A2,ARHGAP26,ITGA2,GRIN2A,BLK,WNT16,ADCY1,GRID1,TRIM24,ZDHHC17,EIF2AK2,F2RL2,KMT2D,LPAR3,FMR1,SH3TC2,NETO1,SNX5,CDK13,NEO1,RASA2,ITPKB,F10,SYNE1,PSMD10,FBXL17,GAS2,RUNX2,TSPEAR,BRMS1L,RGS12,SLC39A10,KCTD8,SLC2A2,PIK3R1,STAMBPL1,USP50,ROR1,AAK1,CTTN,PASK,USP8,SMAD6,PSD3,MEF2C,RYK,MACF1,MALT1,MTM1,PTPRE,UNC5B,SERINC3,PTPN12,CD55,CTDSPL2,SHISA3,NEDD4,DOCK8,URI1,FGD4,PRDX1,CRHR2,MID1,SLC12A2,EFNB3,TRAT1,FLOT2,PVRIG,SV2B,ILK,LEF1,EYA1,MAPK14,RAPGEF1,ARHGEF3,EDNRB,GRIN2B,DENND4A,OVOL2,LEMD3,SLC9B2,TMEM100,LYPD6,VDR,RTN4,ARHGEF11,PRKAA2,IVNS1ABP,CLSTN1,SCYL2,SERPINE2,PIK3CG,IL1R2,HTT,IQGAP1,LATS1,TNFRSF10B,FBXW11,PTPRO,ARHGAP6,CHD5,KCNB1,ZMIZ1,GRM4,AIM2,ANK3,KITLG,SV2C,ARID4B,MAP4K4,CNIH3,HIP1,RELN,MFHAS1,CRKL,ARHGEF10,PTPN13,VAV3,INPP5A,GPAT3,SPRING1,CCDC88A,APBA1,TNIP3,BAK1,CASTOR2,LAX1,CACTIN,PPP3CA,CHRNA5,DYSF,NKD1,RBPJ,CYRIB,C3,DENND1A,MBD5,ASAH2,ASH1L,RSPO2,DISC1,PROX1,BMP6,RALGPS1,CREBBP,ITGB1,NEFL,STK3,CHN2,RAMP3,LIMS1,MCTP2,GLCE,EPHB1,CYTH4,UBR1,TCF7L1,ARHGAP32,DYRK3,CHRNA6,GDNF,TLR7,PLEKHG4B,RNASEH2B,LRP2,MTDH,ZDHHC13,WWC3,VWC2,TMEM108,SEZ6L,SLC39A8,GLI2,TGFBR3,MAP3K7,A*

			DAM17,STXBP1,NUAK2,PLEKHG1,ELP2,APC,GNA12,IL18R1,ERC1,ROS1,AGO3,MOB3B,SPINK1,CTNND2,PACSIN2,RAB8B,GRM8,PAIP2,PTPN11,MAPKAP1,MGAT5,SIPA1L2,FSTL4,ZC3HAV1,ARHGAP28,MTOR,KSR1,CBLB,FAF1,SNAP25,PIP5K1A,GH2,SPRED1,ADAM10,GNG4,CNTNAP4,CRIM1,RNF115,PER2,GLI3,KAT6A,NTRK3,PDCD10,TFG,SLC4A10,FERMT2,TSPOAP1,GLUD1,SYT1,PHIP,SFRP5,PPARA,SHISA6,MAP2K5,CACNA1A,EDA,RBM47,EIF2A,HDAC2,FGD1,TRIM44,TRIM39,NPSR1,ACP3,MAGEA1,CADM1,INPP5D,ATF6,PRKAR1B,GBF1,SERPINE1,CCDC88C,TM7SF3,CYTH3,AKAP13,GCG,ARFGEF1,TKFC,PTPN3,RALGAPA1,SSX2IP,RFX4,ACTA2,RIPOR2,TAOK1,CASK
GO:0022008	neurogenesis	0.000010873927381154835	*RIT2,PBX4,XK,SEMA4D,MET,SCYL3,ATXN10,STAU2,DRD3,SDC2,NFE2L2,DTNB,ATP2B2,PREX2,CSMD3,NTN4,SRGAP2C,ZFHX2,GRIN2A,NELL2,IL1RAPL1,WDPCP,KIF5C,GPM6A,WNT16,ADCY1,PLPPR5,ZDHHC17,FIG4,LPAR3,FMR1,SH3TC2,CEP290,PTPRQ,NEO1,MYT1L,SYNE1,RUNX2,LRP12,ESRP1,RNF10,SHTN1,SH3GL2,DOCK7,ROR1,CRB1,CTTN,PARD6B,NYAP2,MEF2C,DBNL,RYK,MACF1,UNC5B,PTPRZ1,EML1,NEDD4,SECISBP2,ZNF609,OLFM1,EFNB3,ILK,LEF1,EYA1,RAPGEF1,SH3GL3,GABRB2,EDNRB,ARSB,ZSWIM6,SLC4A7,RTN4,SCYL2,SERPINE2,ANOS1,IQGAP1,MYCBP2,WNT2B,MAP2,PTPRO,CHD5,ACSL6,ULK2,KCNB1,ZMIZ1,RP1L1,ANKRD27,ANK3,MAP4K4,RELN,CRKL,ARHGEF10,DPYSL3,CCDC88A,TENM4,PPP3CA,NKD1,RBPJ,C3,PDE6C,FRYL,RSPO2,DISC1,PROX1,BMP6,ASAP1,ITGB1,NEFL,NAV2,TRPC4,BCL11B,TSPAN2,EPHB1,NAP1L1,ARHGAP32,GDNF,KLHL1,UNC5C,CFL1,HMG20A,CEP85L,PLEKHG4B,LRP2,SEMA6D,VWC2,TMEM108,GLI2,ADAM17,SEMA4F,TRAK1,STXBP1,CTNND2,RDH13,DPYSL2,SLC23A2,PTPN11,EFHD1,NRP2,PLXNC1,NR3C1,FSTL4,MTOR,SNAP25,RP1,BTBD2,RAB6B,PER2,ANKRD24,GLI3,NTRK3,ADGRG6,SLC4A10,DNM3,SYT1,DENND5A,NFASC,PACSIN1,CELSR1,HDAC2,SEMA3D,PROM1,ST8SIA2,RIPOR2,TAOK1,USH1C*
GO:0023051	regulation of signaling	0.000011249122435588183	*RIT2,MYO9B,CYTH1,SEMA4D,MET,BRCA1,STAU2,DRD3,ELAPOR2,TACR1,SNX25,GPHB5,ARAP1,EIF4A3,NFE2L2,SCHIP1,CYP19A1,SLC46A2,GPR158,VRK2,ITSN1,OAS1,PREX2,FBXL20,SERP1,WIF1,CSNK2A2,ARHGAP26,GRIN2A,BLK,WNT16,ADCY1,GRID1,TRIM24,ZDHHC17,EIF2AK2,F2RL2,KMT2D,LPAR3,FMR1,SH3TC2,NETO1,SNX5,CDK13,NEO1,RASA2,ITPKB,F10,SYNE1,PSMD10,FBXL17,GAS2,RUNX2,TSPEAR,BRMS1L,RGS12,SLC39A10,KCTD8,SLC2A2,PIK3R1,STAMBPL1,USP50,ROR1,AAK1,CTTN,PASK,USP8,SMAD6,PSD3,MEF2C,RYK,MACF1,MALT1,MTM1,PTPRE,UNC5B,SERINC3,PTPN12,CD55,CTDSPL2,SHISA3,NEDD4,DOCK8,URI1,FGD4,PRDX1,CRHR2,MID1,SLC12A2,EFNB3,TRAT1,FLOT2,PVRIG,SV2B,ILK,LEF1,EYA1,MAPK14,RAPGEF1,ARHGEF3,EDNRB,GRIN2B,DENND4A,OVOL2,LEMD3,SLC9B2,TMEM100,LYPD6,VDR,RTN4,ARHGEF11,PRKAA2,IVNS1ABP,CLSTN1,SCYL2,SERPI*

			NE2,PIK3CG,IL1R2,HTT,IQGAP1,LATS1,TNFRSF10B,FBXW11,PTPRO,ARHGAP6,CHD5,KCNB1,ZMIZ1,GRM4,AIM2,KITLG,SV2C,ARID4B,MAP4K4,CNIH3,HIP1,RELN,MFHAS1,CRKL,ARHGEF10,PTPN13,VAV3,INPP5A,GPAT3,SPRING1,CCDC88A,APBA1,TNIP3,BAK1,CASTOR2,LAX1,CACTIN,PPP3CA,CHRNA5,DYSF,NKD1,RBPJ,CYRIB,C3,DENND1A,MBD5,ASAH2,ASH1L,RSPO2,DISC1,PROX1,BMP6,RALGPS1,CREBBP,ITGB1,BVES,NEFL,STK3,CHN2,RAMP3,LIMS1,MCTP2,GLCE,EPHB1,CYTH4,UBR1,TCF7L1,ARHGAP32,DYRK3,CHRNA6,GDNF,TLR7,PLEKHG4B,RNASEH2B,LRP2,MTDH,ZDHHC13,WWC3,VWC2,TMEM108,SEZ6L,SLC39A8,GLI2,TGFBR3,MAP3K7,ADAM17,STXBP1,NUAK2,PLEKHG1,ELP2,APC,GNA12,IL18R1,ERC1,ROS1,AGO3,MOB3B,SPINK1,CTNND2,PACSIN2,RAB8B,GRM8,PAIP2,PTPN11,MAPKAP1,MGAT5,SIPA1L2,FSTL4,ZC3HAV1,ARHGAP28,MTOR,KSR1,CBLB,FAF1,SNAP25,PIP5K1A,GH2,SPRED1,ADAM10,GNG4,CNTNAP4,CRIM1,RNF115,PER2,GLI3,KAT6A,NTRK3,PDCD10,TFG,SLC4A10,FERMT2,TSPOAP1,GLUD1,SYT1,PHIP,SFRP5,PPARA,SHISA6,MAP2K5,CACNA1A,EDA,RBM47,EIF2A,HDAC2,FGD1,TRIM44,TRIM39,NPSR1,ACP3,MAGEA1,CADM1,INPP5D,ATF6,PRKAR1B,GBF1,SERPINE1,CCDC88C,TM7SF3,CYTH3,AKAP13,GCG,ARFGEF1,TKFC,PTPN3,RALGAPA1,SSX2IP,RFX4,ACTA2,RIPOR2,TAOK1,CASK
GO:0007275	multicellular organism development	0.000015834139076826015	*RIT2,PBX4,XK,SEMA4D,MET,SCYL3,TEAD1,ATXN10,BRCA1,STAU2,SIAH3,MMP16,DRD3,ELAPOR2,ATP11C,DNAH11,SDC2,FANCC,CCR3,ANKLE2,ACTR5,EIF4A3,NFE2L2,CYP19A1,SLC46A2,DTNB,GPR158,ATP2B2,PREX2,CSMD3,NTN4,SERP1,ENKUR,SRGAP2C,WIF1,CSNK2A2,ZFHX2,ARHGAP26,ITGA2,GRIN2A,NELL2,IL1RAPL1,WDPCP,KIF5C,GPM6A,WNT16,ADCY1,RPS6KA2,PLPPR5,ZDHHC17,FIG4,FOXP2,LPAR3,FMR1,TXLNG,FOXN3,SH3TC2,CEP290,TACC1,SH3PXD2B,AK8,PTPRQ,NEO1,MYT1L,SRGAP2B,ITPKB,SYNE1,FBXL17,GAS2,RUNX2,LRP12,ESRP1,RNF10,FAT1,PIK3R1,SHTN1,BTD,CALD1,SH3GL2,MYLK3,DOCK7,ROR1,PAX5,CRB1,CTTN,ARID1A,PARD6B,SMAD6,NYAP2,MEF2C,FZR1,DBNL,RYK,MACF1,MALT1,TFDP2,KCNE1,UNC5B,PTPRZ1,PLN,EML1,NEDD4,RBBP9,DDAH1,PARVA,SECISBP2,ZNF609,PRDX1,OLFM1,MID1,SLC12A2,OTX1,EFNB3,ILK,LEF1,EYA1,MAPK14,MYBPC3,RAPGEF1,SH3GL3,SLC1A2,GABRB2,EDNRB,GRIN2B,ARSB,TBC1D20,SETD5,PARP2,ZSWIM6,OVOL2,SLC4A7,DNAJB6,SLC9B2,NEB,TMEM100,VDR,PDPN,CREB3L2,RTN4,BBS2,CLSTN1,SCYL2,MAOB,SERPINE2,MYO3A,PLAAT3,PIK3CG,ANOS1,COL4A4,TPP1,STIL,NUP160,NECTIN3,IQGAP1,MYCBP2,LATS1,KLHL12,WNT2B,MAP2,FBXW11,PTPRO,PEMT,CHD5,JAK1,ACSL6,ULK2,KCNB1,ZMIZ1,UGP2,RP1L1,GNPAT,ANKRD27,AIM2,ANK3,KITLG,ARID4B,MAP4K4,RELN,CRKL,MYO18B,TENT5A,ARHGEF10,PTPN13,VAV3,CD40LG,SCN9A,DPYSL3,ATP2B1,ATRN,CCDC88A,ARNT2,APBA1,PLCE1,TACC2,MSH3,BAK1,TENM4,P*

			PP3CA,NKD1,RBPJ,C3,RIN2,PDE6C,DLX6-AS1,ADAMTS16,TAF10,TRIP12,MBD5,FRYL,ASH1L,RSPO2,DISC1,PROX1,BMP6,FMN1,ASAP1,CREBBP,ITGB1,BVES,NFKBIZ,CORO1C,NEFL,NAV2,STK3,C1GALT1,EXOSC3,AP2B1,TRPC4,BCL11B,PLS3,PCSK2,TSPAN2,GFRA2,EPHB1,NAP1L1,PLPP4,COL4A2,ARHGAP32,HDAC4,GDNF,KLHL1,RBM45,IQCE,MYLK,UNC5C,ACSBG1,CSGALNACT1,CFL1,HMG20A,CEP85L,PLEKHG4B,RBBP8,RNASEH2B,PHF2,RAI2,LRP2,MTDH,SEMA6D,EHMT1,CAMK4,VWC2,TMEM108,BFSP1,SEZ6L,GLI2,TGFBR3,GTF2I,ATXN1,TMEM59L,ADAM17,SEMA4F,TRAK1,STXBP1,IDH1,NHLH1,APC,GNA12,TTLL5,INO80,SULT2B1,PPARGC1B,COL27A1,PHLDB2,CTNND2,RDH13,DPYSL2,NSD2,SLC23A2,AARS1,PTPN11,MAPKAP1,COL15A1,CLPTM1,HSD17B4,EFHD1,NRP2,PLXNC1,FOXJ2,NR3C1,DLX6,FSTL4,MTOR,CBLB,SNAP25,RP1,BTBD2,DISP1,RAB6B,SPRED1,ADAM10,GALC,CRIM1,RBM46,ABCB5,PER2,ANKRD24,GLI3,KAT6A,BCOR,NTRK3,RXFP1,PDCD10,ADGRG6,SLC4A10,FERMT2,GLUD1,DNM3,SYT1,DENND5A,SFRP5,PPARA,NFASC,MAP2K5,PHF6,FGF7,PACSIN1,RTTN,EDA,CNTRL,MICAL2,COL14A1,JARID2,CELSR1,HDAC2,TPO,SEMA3D,PROM1,RRM1,CADM1,INPP5D,ATF6,SERPINE1,AKAP13,PKM,PTPN3,SCN8A,ST8SIA2,RFX4,ACTA2,FREM1,RIPOR2,XIRP2,TAOK1,HSPG2,GCNT1,USH1C,AFG2A
GO:0048522	positive regulation of cellular process	0.000018984791176581792	*RIT2,ABCB7,PBX4,GPSM2,ERG,PARN,STK16,SEMA4D,MET,MAX,TEAD1,BRCA1,EIF4G3,BAZ1B,STAU2,DRD3,ELAPOR2,ATP11C,TOX3,TACR1,CIITA,ARAP1,CCR3,RPRD1B,AHNAK,ACTR5,EIF4A3,NFE2L2,SCHIP1,UIMC1,TFEC,NFYC,CYP19A1,SLC46A2,GPR158,OAS1,SERP1,NUB1,SRGAP2C,WIF1,FLI1,CSNK2A2,ESRRB,ZBTB7C,ITGA2,GRIN2A,IL1RAPL1,BLK,GPM6A,WNT16,SETD3,ADCY1,RPS6KA2,PLPPR5,TRIM24,ZDHHC17,EIF2AK2,F2RL2,KMT2D,LPAR3,FMR1,CEP290,SHQ1,TACC1,OCLN,TUT4,NETO1,SNX5,CDK13,NEO1,ITPKB,TCERG1,F10,DIO2,SYNE1,PSMD10,DHX29,GPAM,IRF2,RUNX2,SLC39A10,ZNF496,RNF10,FAT1,SLC2A2,PIK3R1,STAMBPL1,IL17B,COL26A1,SHTN1,USP50,POLR3F,CRTC1,MYLK3,DOCK7,ROR1,SCAP,PAX5,AAK1,CTTN,UTP15,PASK,USP8,ARID1A,SMAD6,RYBP,ABCA13,RFX2,MEF2C,FZR1,BAIAP2L1,RYK,MACF1,PXT1,TAF4B,MALT1,WEE2,TFDP2,KCNE1,UNC5B,SERINC3,PTPRZ1,CD55,CTDSPL2,NEDD4,NEK4,RBBP9,LRRC38,DOCK8,DDAH1,SECISBP2,CHD6,ZNF609,COX17,MYO10,OLFM1,CRHR2,MID1,SLC12A2,OTX1,EFNB3,TRIM37,DLG3,TRAT1,FLOT2,ILK,LEF1,EYA1,MAPK14,RAPGEF1,SH3GL3,ZNF516,SLC1A2,ARHGEF3,EDNRB,GRIN2B,SPDYE3,ARSB,TBC1D20,SCAMP5,PARP2,OVOL2,FCHSD2,OMA1,PIEZO1,SOAT1,SLC9B2,CSRNP3,TMEM100,LYPD6,VDR,PDPN,CREB3L2,RTN4,GNL3L,ARHGEF11,PRKAA2,CLSTN1,SKAP1,MAOB,SERPINE2,MYO3A,IL12RB2,PIK3CG,STIL,TFDP1,HTT,IQGAP1,MYCBP2,WIPI1,LATS1,SNX30,FANCI,TNFRSF10B,MAP2,FBXW11,MTCL1,HSPH1,AZIN2,HLA-*

			DMA,PEMT,CHD5,JAK1,ULK2,KCNB1,ZMIZ1,HSD17B12,GRM4,ANKRD27,AIM2,ANK3,KITLG,ARID4B,MAP4K4,NR1I2,HIP1,RAET1E,RFX7,RELN,MFHAS1,CRKL,TENT5A,ARHGEF10,VAV3,CD40LG,VRK1,DPYSL3,SPRING1,CCDC88A,ARNT2,HPSE2,PLCE1,BAK1,TENM4,DCP1A,PPP3CA,NKD1,RBPJ,CYRIB,SPDYE17,C3,RIN2,DLX6-AS1,TASP1,TAF10,MBD5,KDM5A,CNOT6,PKIB,ASH1L,RSPO2,DISC1,PROX1,BMP6,FMN1,ASAP1,CREBBP,ITGB1,BVES,ZNF827,NFKBIZ,CORO1C,SPIDR,NEFL,STK3,FCAR,HNF4G,MSR1,EXOSC3,SH2D3C,RAMP3,LIMS1,ENPP2,DOCK4,UBL4B,BCL11B,GLCE,PWWP2A,TBC1D10A,EPHB1,SSBP2,NAP1L1,CNN2,ARHGAP32,DYRK3,IP6K2,HDAC4,GDNF,MARCHF5,MYLK,UNC5C,CFAP69,TLR7,SLAIN2,MED13,RNASEH2B,PHF2,AFDN,LRP2,MTDH,SEMA6D,EHMT1,ZDHHC13,IL17RA,CAMK4,VWC2,TMEM108,LCP2,GLI2,TAS2R13,TGFBR3,MAP3K7,GTF2I,ADAM17,SEMA4F,ELAVL1,TRAK1,STXBP1,LMOD2,HDGFL2,NHLH1,APC,IL18R1,INO80,SULT2B1,PEAR1,PPARGC1B,PHLDB2,ESR2,AGO3,PVR,MOB3B,RLF,SPINK1,TAF5L,TIA1,SYNPO2,RAB8B,METTL8,NSD2,SLC23A2,PAIP2,PTPN11,PSMC2,MAPKAP1,MGAT5,NRP2,CAVIN1,PLXNC1,FOXJ2,NR3C1,DLX6,BORCS5,ZC3HAV1,MTOR,KSR1,CBLB,FAF1,SNAP25,RP1,DEFB124,CHST4,GH2,SPRED1,ADAM10,WASHC1,RBM46,PER2,GLI3,KAT6A,NTRK3,TADA1,GAB2,DIS3L2,CTCF,PDCD10,PRAMEF11,TFG,EPHX2,CREB5,FERMT2,GLUD1,DNM3,SYT1,PHIP,EPB41,SS18,ZYG11B,RFC2,PPARA,CD244,MAP2K5,ZNF229,EPB41L2,FGF7,TENT5D,PACSIN1,MGA,EDA,LIMCH1,TNFSF13B,PTH2R,ELOVL6,MICAL2,RBM47,EIF2A,HDAC2,TRIM44,TRIM39,SEMA3D,PROM1,NPSR1,ACP3,RRM1,CADM1,INPP5D,CLYBL,SPDYE9,ATF6,PRKAR1B,SERPINE1,BACH1,PRAMEF4,TM7SF3,CYTH3,SLC26A5,AKAP13,PKM,GCG,LINC02218,STAC,NNT,ST8SIA2,IRF8,HIVEP3,RFX4,ACTA2,RIPOR2,KDM3A,TAOK1,CASK,HSPG2,GCNT1,PRDM7,FMN2,UBXN2A,PCNT
GO:0050789	regulation of biological process	0.000026837999251222695	*RIT2,SLMAP,KLHL13,ABCB7,PBX4,NHLRC3,MYO9B,GPSM2,COL4A5,ERG,PARN,CYTH1,STK16,XK,SEMA4D,MET,MAX,TEAD1,BRCA1,EIF4G3,BAZ1B,CEP350,STAU2,SIAH3,DUS3L,PIWIL3,CHMP1B,MIR654,DRD3,ELAPOR2,ATP11C,ZNF534,TOX3,DNAH11,EXOC2,PTPN5,TACR1,SNX25,SDC2,ABHD12,GPHB5,CIITA,ARAP1,CCR3,ANKLE2,CEP85,RPRD1B,AHNAK,ACTR5,EIF4A3,DLEC1,NFE2L2,SCHIP1,MIR376A2,PARG,OLFML2A,UIMC1,TFEC,NFYC,CYP19A1,SLC46A2,DTNB,GPR158,VRK2,ITSN1,OAS1,ATP2B2,PREX2,SCRN1,ELAVL2,CSMD3,FBXL20,NTN4,SERP1,POLR1A,RAB11FIP4,HSFX4,SNTA1,CLDN10,ASZ1,NUB1,SRGAP2C,WIF1,FLI1,CSNK2A2,ESRRB,ZFHX2,ARHGAP26,TDRD9,LINC-PINT,TRHDE,RAB44,SETBP1,ZBTB7C,ITGA2,GRIN2A,IL1RAPL1,WDPCP,BLK,GPM6A,WNT16,SETD3,ADCY1,GRID1,RPS6KA2,PLPPR5,TRIM24,ZDHHC17,FIG4,EIF2AK2,FOXP2,F2RL2,KMT2D,LPAR3,FMR1,TXLNG,FOXN3,SP110,SH3BP2,*

			SH3TC2,CEP290,SHQ1,TACC1,RAD9B,OCLN,TUT4,FASTKD5,ITGBL1,NETO1,GBE1,SNX5,CDK13,SNX32,PCGEM1,NEO1,MYT1L,RASA2,ITPKB,PLEKHB2,TCERG1,F10,SRGAP1,DIO2,CABIN1,SYNE1,PSMD10,FBXL17,EOLA1,DHX29,GAS2,GPAM,NABP1,IRF2,RUNX2,LRP12,TSPEAR,ESRP1,HSFX3,BRMS1L,RGS12,CBX3,SLC39A10,CDCA2,ZNF496,KCTD8,ZNF550,MPHOSPH9,RNF10,FAT1,SLC2A2,PIK3R1,STAMBPL1,IL17B,ABCB11,SARNP,CFDP1,COL26A1,SHTN1,ATG12,MASP1,USP50,CD200R1,POLR3F,SH3GL2,CRTC1,PITPNC1,MYLK3,SLC30A3,DOCK7,PDK3,ROR1,SCAP,PAX5,AAK1,CRB1,CTTN,UTP15,PASK,USP8,GPR75,ARID1A,PARD6B,THEMIS,SMAD6,OR2AJ1,RYBP,NYAP2,PSD3,ABCA13,RFX2,MEF2C,ZNF519,FZR1,BAIAP2L1,DBNL,ELP4,RYK,MACF1,PXT1,TAF4B,MALT1,WEE2,MTM1,TFDP2,KCNE1,PTPRE,UNC5B,ZSCAN5A,SERINC3,ZNF347,PTPN12,PTPRZ1,CD55,PLN,CTDSPL2,SHISA3,NEDD4,NEK4,RBBP9,CACNA1E,FLYWCH1,LRRC38,DOCK8,CYSLTR2,DDAH1,PARVA,SECISBP2,URI1,SCML2,CHD6,FGD4,ZNF609,PRDX1,COX17,HACE1,AEBP2,DSC2,MYO10,OLFM1,CRHR2,MID1,POGK,TSHZ2,SLC12A2,OTX1,EFNB3,TRIM37,DLG3,TRAT1,FLOT2,PVRIG,SV2B,ILK,LEF1,EYA1,ZNF816-ZNF321P,MAPK14,MYBPC3,ZFHX4,RAPGEF1,GPRC6A,SH3GL3,ZNF516,CERKL,SLC1A2,ARHGEF3,GABRB2,EDNRB,GRIN2B,SPDYE3,ARSB,GPR141,IFNGR2,DENND4A,TBC1D20,OXR1,SFMBT2,SETD5,SCAMP5,IL17RB,PARP2,OVOL2,MIR376B,FCHSD2,DNAJB6,MTCL3,OMA1,MTUS1,GABRA6,LEMD3,KHDRBS2,PIEZO1,SOAT1,OR2T11,SLC9B2,NEB,CSRNP3,MRGPRX1,TMEM100,LYPD6,VDR,NEK11,PDPN,CREB3L2,RTN4,GNL3L,CACNA1D,ARHGEF11,PRKAA2,IVNS1ABP,SGPP2,DPT,BBS2,CLSTN1,ZNF155,SERGEF,SCYL2,SKAP1,MAOB,SKIC3,SERPINE2,MYO3A,PLAAT3,IL12RB2,PIK3CG,ANKFN1,STIL,TFDP1,IL1R2,ATP6V1E2,HTT,IQGAP1,MYCBP2,WIPI1,LATS1,ZSCAN25,KLHL12,LOC349160,SNX30,FANCI,OR9Q1,OR4K17,SPARCL1,WNT2B,TMDD1,TNFRSF10B,MAP2,FBXW11,MTCL1,PTPRO,HSPH1,AZIN2,MIR518C,SMG6,HLA-DMA,PEMT,ARHGAP6,CHD5,JAK1,ITGA9,ULK2,KCNB1,RADIL,ZMIZ1,RP1L1,HSD17B12,GRM4,ANKRD27,AIM2,ANK3,GTF2IRD1,ZNF19,PPP1R14C,KITLG,SV2C,ARID4B,GMDS,ZNF326,MAP4K4,CNIH3,PLEKHM2,NR1I2,ZNF266,HIP1,RAET1E,RFX7,RELN,MFHAS1,OR51B5,FBXO32,MTMR3,CRKL,TENT5A,ARHGEF10,FGD6,PTPN13,SNAP91,ZNF816,VAV3,RPS6KC1,INPP5A,CD40LG,VRK1,GPAT3,ZNF420,DPYSL3,ATP2B1,SPRING1,SLC16A10,ATRN,CCDC88A,SLC35B4,ARNT2,APBA1,HPSE2,PLCE1,TNIP3,DCAF5,GNAL,STK32C,MSH3,BAK1,CASTOR2,LAX1,CACTIN,STK26,MIR520C,TENM4,DCP1A,PPP3CA,CHRNA5,MIR526A1,MKNK1,DYSF,NKD1,RBPJ,CYRIB,SPDYE17,C3,RIN2,PDE6C,PLEKHG7,DLX6-AS1,ASCC1,DENND1A,ENSG00000290317,OR52D1,ADAMTS16,TASP1,MICU1,TAF10,TRIP12,M

			BD5,BACE2,KDM5A,ZNF549,ZNF320,CNOT6,PKIB,ASAH2,STON1,ASH1L,RSPO2,ZNF747,ZNF805,OR5K1,OR13C9,DISC1,PROX1,BMP6,FMN1,ZNF891,RALGPS1,CTSB,ASAP1,LRRC2,AQP8,CREBBP,ITGB1,BVES,ZNF827,NFKBIZ,CORO1C,KANK3,NSG2,SPIDR,NEFL,ATP6V1A,STK3,CHN2,FCAR,HNF4G,MSR1,EXOSC3,GABRG3,SH2D3C,RAMP3,GNG2,ZNF705A,FRS3,LIMS1,ENPP2,DOCK4,ACKR2,UBL4B,MCTP2,BCL11B,GLCE,KCNIP1,PWWP2A,LOC105373170,GFRA2,TBC1D10A,EPHB1,SSBP2,CASP8AP2,RPH3A,CTTNBP2,CYTH4,NAP1L1,PLPP4,UBR1,CNN2,COL4A2,SLC1A6,TCF7L1,ARHGAP32,DYRK3,IP6K2,CHRNA6,HDAC4,ZNF582,GDNF,FYB1,CALCB,MARCHF5,MYLK,UNC5C,CFAP69,TLR7,P3H2,CFL1,KRT20,SLAIN2,HMG20A,PLEKHG4B,RBBP8,MED13,RNASEH2B,PHF2,TAAR2,AFDN,CYTIP,LRP2,MTDH,SEMA6D,EHMT1,ZDHHC13,WWC3,IL17RA,CAMK4,VWC2,RPA3,TMEM108,TPTE,LCP2,GAB3,SEZ6L,SLC39A8,GLI2,TAS2R13,TGFBR3,MAP3K7,GTF2I,ATXN1,ADAM17,SEMA4F,ELAVL1,TRAK1,STXBP1,OR6N1,IDH1,NUAK2,LMOD2,ABCC1,HDGFL2,PLEKHG1,RBAK-RBAKDN,NHLH1,ELP2,CYP8B1,APC,GNA12,IL18R1,INO80,SULT2B1,PEAR1,CDA,PPARGC1B,PHLDB2,ESR2,MIR376C,ERC1,ZNF440,ROS1,RUBCNL,AGO3,C9,PVR,MOB3B,ACER1,IQGAP2,CDYL,RLF,CLPB,SPINK1,ANKRA2,TAF5L,CTNND2,ERI1,RASSF3,PACSIN2,TIA1,DPYSL2,SYNPO2,GIMAP8,RAB8B,METTL8,NSD2,GRM8,SLC23A2,PAIP2,AARS1,PTPN11,JADE3,CARD18,PSMC2,MAPKAP1,COL15A1,EVC2,GON4L,RIC1,MGAT5,MAEL,NUP153,CLPTM1,EFHD1,PIGR,NRP2,SIPA1L2,CAVIN1,PLXNC1,FOXJ2,NR3C1,DLX6,MIR300,BORCS5,FSTL4,ZC3HAV1,ARHGAP28,MTOR,KSR1,CBLB,FAF1,SNAP25,TUFT1,RP1,DEFB124,CHST4,OR11H6,ZNF221,PIP5K1A,GH2,DISP1,TBXAS1,ASCC2,SPRED1,ADAM10,GNG4,CNTNAP4,WASHC1,PDS5B,CRIM1,RBM46,RNF115,CDHR3,PER2,PLEKHH2,GLI3,KAT6A,BCOR,NTRK3,RXFP1,TADA1,GAB2,DIS3L2,CTCF,PDE1A,PDCD10,PRAMEF11,TFG,EPHX2,HHAT,CREB5,CCDC68,ADGRG6,SLC4A10,FERMT2,GPR82,TSPOAP1,GLUD1,DNM3,SYT1,PHIP,EPB41,DENND5A,SS18,SFRP5,DOCK9,ZYG11B,RFC2,PPARA,CD244,SHISA6,HDX,MAP2K5,ZNF229,PHF6,HMGB4,EPB41L2,FGF7,TENT5D,PACSIN1,STXBP6,MGA,CACNA1A,EDA,LIMCH1,TNFSF13B,PTH2R,ELOVL6,MICAL2,COL14A1,RBM47,EIF2A,JARID2,CELSR1,HDAC2,FIRRM,FGD1,TRIM44,INSYN2B,TRIM39,SEMA3D,PROM1,OR2T8,NPSR1,EIF4E3,ACP3,KCNH5,RRM1,MAGEA1,CADM1,INPP5D,CLYBL,OR4C6,SPDYE9,ATF6,PRKAR1B,GBF1,SERPINE1,CCDC88C,BACH1,CUL9,PRAMEF4,EWSR1,TM7SF3,CYTH3,SLC26A5,AKAP13,HBS1L,PKM,GCG,ARFGEF1,TKFC,LINC02218,PTPN3,STAC,RALGAPA1,NNT,SSX2IP,ST8SIA2,ELP1,IRF8,HIVEP3,RFX4,OR6K6,ZNF30,REM1,ACTA2,RIPOR2,FUNDC2,KDM3A,CAST,OR2L3,TAOK1,GPR39,ADGRA3,CASK,HSPG2,GCNT1,PRDM7,USH1C,FMN2,UBXN2A,NLRP14,PCNT,DNAJC13

			,PSME3IP1
GO:0032501	multicellular organismal process	0.00002860449448444557	RIT2,SLMAP,PBX4,XK,SEMA4D,MET,SCYL3,TEAD1,ATXN10,BRCA1,ACSM3,STAU2,SIAH3,CETN3,MMP16,DRD3,ELAPOR2,ATP11C,DNAH11,TACR1,SDC2,ABHD12,FANCC,CCR3,ANKLE2,ACTR5,EIF4A3,NFE2L2,OTOA,CYP19A1,SLC46A2,DTNB,GPR158,OAS1,ATP2B2,ESPNL,PREX2,CSMD3,FBXL20,NTN4,SERP1,ENKUR,SNTA1,SRGAP2C,WIF1,FLI1,CSNK2A2,CLIC5,ESRRB,ZFHX2,ARHGAP26,RAB44,ITGA2,GRIN2A,NELL2,IL1RAPL1,WDPCP,KIF5C,GPM6A,WNT16,KRT86,SETD3,ADCY1,GRID1,RPS6KA2,PLPPR5,ZDHHC17,FIG4,EIF2AK2,FOXP2,F2RL2,LPAR3,FMR1,TXLNG,FOXN3,SH3TC2,CEP290,TACC1,SH3PXD2B,OCLN,TUT4,AK8,NETO1,PTPRQ,NEO1,MYT1L,SRGAP2B,ITPKB,F10,DIO2,SYNE1,FBXL17,EOLA1,SLC9A4,GAS2,GPAM,RUNX2,LRP12,TSPEAR,ESRP1,BRMS1L,SLC39A10,RNF10,FAT1,PIK3R1,IL17B,SHTN1,ATG12,USP50,CD200R1,BTD,CALD1,POLR3F,SH3GL2,CRTC1,MYLK3,DOCK7,ROR1,PAX5,CRB1,CTTN,PASK,ARID1A,PARD6B,THEMIS,SMAD6,OR2AJ1,NYAP2,MEF2C,FZR1,DBNL,RYK,MACF1,MALT1,MTM1,TFDP2,KCNE1,UNC5B,PTPRZ1,ACACA,CD55,PLN,EML1,SLC16A7,NEDD4,RBBP9,DOCK8,DDAH1,PARVA,SECISBP2,ZNF609,PRDX1,DSC2,SERPINA1,OLFM1,MID1,SLC12A2,OTX1,EFNB3,FLOT2,ILK,LEF1,EYA1,MAPK14,MYBPC3,RAPGEF1,SH3GL3,ZNF516,SLC1A2,GABRB2,EDNRB,GRIN2B,ARSB,CRISPLD1,IFNGR2,TBC1D20,OXR1,SETD5,SCAMP5,PARP2,ZSWIM6,MYL1,OVOL2,SMPX,SLC4A7,DNAJB6,OMA1,SOAT1,OR2T11,SLC9B2,NEB,TMEM100,VDR,PDPN,CREB3L2,RTN4,CACNA1D,ARHGEF11,PRKAA2,BBS2,CLSTN1,TMEM150C,SCYL2,MAOB,SERPINE2,MYO3A,PLAAT3,IL12RB2,PSG2,PIK3CG,ANOS1,COL4A4,TPP1,ANKFN1,STIL,IL1R2,HTT,NUP160,NECTIN3,IQGAP1,MYCBP2,LATS1,KLHL12,OR9Q1,OR4K17,WNT2B,MAP2,FBXW11,PTPRO,HSPH1,HLA-DMA,PEMT,CHD5,JAK1,ITGA9,ACSL6,ULK2,KCNB1,ZMIZ1,UGP2,RP1L1,GNPAT,ANKRD27,AIM2,ANK3,GTF2IRD1,KITLG,ARID4B,MAP4K4,RELN,MFHAS1,OR51B5,FBXO32,CRKL,MYO18B,TENT5A,ARHGEF10,PTPN13,VAV3,CD40LG,SCN9A,DPYSL3,ATP2B1,ATRN,NDUFB9,CCDC88A,ARNT2,APBA1,PLCE1,TACC2,CYP39A1,MSH3,BAK1,LAX1,CACTIN,TENM4,PPP3CA,CHRNA5,DYSF,NKD1,RBPJ,CYRIB,C3,RIN2,PDE6C,DLX6-AS1,OR52D1,ADAMTS16,TAF10,TRIP12,MBD5,ASAH2,FRYL,ASH1L,RSPO2,OR5K1,OR13C9,DISC1,PROX1,BMP6,FMN1,CTSB,ASAP1,VSIG1,AQP8,CREBBP,ITGB1,BVES,NFKBIZ,CORO1C,NEFL,NAV2,STK3,FCAR,C1GALT1,MSR1,EXOSC3,AP2B1,TRPC4,RAMP3,ENPP2,DOCK4,BCL11B,CERS3,PLS3,PCSK2,TSPAN2,GFRA2,EPHB1,TULP2,NAP1L1,PLPP4,COL4A2,ARHGAP32,DYRK3,CHRNA6,HDAC4,GDNF,KLHL1,RBM45,IQCE,MYLK,UNC5C,CFAP69,ACSBG1,CSGALNACT1,TLR7,CFL1,HMG20A,CEP85L,TUSC3,PLEKHG4B,RBBP8,RNASEH2B,PHF2,RAI2,LRP2,MTDH,SEMA6D,EHMT1,WWC3,IL17RA,CAMK4,VWC2,TMEM108,B

			FSP1,LCP2,SEZ6L,SLC39A8,GLI2,TAS2R13,TGFBR3,MAP3K7,UPRT,GTF2I,ATXN1,TMEM59L,ADAM17,SEMA4F,ELAVL1,TRAK1,STXBP1,OR6N1,IDH1,LMOD2,ABCC1,MUC13,NHLH1,SNRNP200,CYP8B1,APC,GNA12,TTLL5,IL18R1,INO80,SULT2B1,PEAR1,PPARGC1B,CTRB1,COL27A1,PHLDB2,SPINK1,TAF5L,CTNND2,RDH13,TIA1,DPYSL2,RAB8B,NSD2,GRM8,SLC23A2,PAIP2,AARS1,PTPN11,CARD18,PSMC2,MAPKAP1,COL15A1,MAEL,CLPTM1,HSD17B4,EFHD1,PIGR,NRP2,PLXNC1,FOXJ2,NR3C1,DLX6,FSTL4,ZC3HAV1,MTOR,CBLB,SNAP25,TUFT1,RP1,DEFB124,OR11H6,BTBD2,DISP1,RAB6B,TBXAS1,SPRED1,ADAM10,GALC,CNTNAP4,CDH26,CRIM1,RBM46,ABCB5,PER2,ANKRD24,GLI3,KAT6A,BCOR,NTRK3,RXFP1,GAB2,DIS3L2,PDCD10,ADGRG6,SLC4A10,FERMT2,GLUD1,DNM3,SYT1,EPB41,DENND5A,SS18,SFRP5,PPARA,CD244,NFASC,SCPEP1,SLC28A2,MAP2K5,PHF6,FGF7,PACSIN1,RTTN,EDA,CNTRL,TNFSF13B,PTH2R,ELOVL6,MICAL2,COL14A1,RBM47,JARID2,CELSR1,HDAC2,TPO,INSYN2B,SEMA3D,PROM1,OR2T8,NPSR1,ACP3,RRM1,CADM1,INPP5D,OR4C6,ATF6,PRKAR1B,GBF1,SERPINE1,SLC26A5,AKAP13,PKM,GCG,PTPN3,STAC,SCN8A,RTL4,ST8SIA2,IRF8,RFX4,CCDC198,OR6K6,REM1,ACTA2,FREM1,RIPOR2,FUNDC2,XIRP2,KDM3A,OR2L3,TAOK1,HSPG2,GCNT1,USH1C,DNAJC13,AFG2A
GO:0007154	cell communication	0.000041798563330543985	*RIT2,MYO9B,GPSM2,COL4A5,ERG,CYTH1,SEMA4D,MET,TEAD1,BRCA1,STAU2,DRD3,ELAPOR2,PTPN5,TACR1,SNX25,GPHB5,ARAP1,CCR3,EIF4A3,NFE2L2,SCHIP1,OLFML2A,UIMC1,OTOA,CYP19A1,SLC46A2,DTNB,GPR158,VRK2,ITSN1,OAS1,PREX2,FBXL20,NTN4,SERP1,WIF1,CSNK2A2,ESRRB,ARHGAP26,TRHDE,RAB44,ITGA2,GRIN2A,IL1RAPL1,WDPCP,BLK,WNT16,ADCY1,GRID1,RPS6KA2,PLPPR5,TRIM24,ZDHHC17,EIF2AK2,F2RL2,KMT2D,LPAR3,FMR1,FOXN3,SH3BP2,SH3TC2,RAD9B,ITGBL1,NETO1,SNX5,CDK13,NEO1,RASA2,ITPKB,F10,SRGAP1,CABIN1,SYNE1,PSMD10,FBXL17,GAS2,NABP1,RUNX2,LRP12,TSPEAR,BRMS1L,RGS12,SLC39A10,KCTD8,RNF10,FAT1,SLC2A2,PIK3R1,STAMBPL1,IL17B,ABCB11,SHTN1,USP50,CD200R1,SH3GL2,PITPNC1,DOCK7,PDK3,ROR1,SCAP,AAK1,CRB1,CTTN,PASK,USP8,GPR75,ARID1A,THEMIS,SMAD6,OR2AJ1,NYAP2,PSD3,MEF2C,FZR1,DBNL,RYK,MACF1,MALT1,MTM1,PTPRE,UNC5B,SERINC3,PTPN12,CD55,PLN,CTDSPL2,SHISA3,NEDD4,CACNA1E,DOCK8,CYSLTR2,DDAH1,URI1,FGD4,PRDX1,HACE1,DSC2,MYO10,OLFM1,CRHR2,MID1,SLC12A2,EFNB3,DLG3,TRAT1,FLOT2,PVRIG,SV2B,ILK,LEF1,EYA1,MAPK14,RAPGEF1,GPRC6A,SH3GL3,SLC1A2,ARHGEF3,GABRB2,EDNRB,GRIN2B,GPR141,IFNGR2,DENND4A,IL17RB,PARP2,OVOL2,FCHSD2,OMA1,GABRA6,LEMD3,OR2T11,SLC9B2,MRGPRX1,TMEM100,LYPD6,VDR,NEK11,PDPN,RTN4,CACNA1D,ARHGEF11,PRKAA2,IVNS1ABP,BBS2,CLSTN1,SERGEF,SCYL2,SKAP1,MAOB,SERPINE2,IL12RB2,PIK3CG,STIL,IL1R2,HTT,FREM3,IQGAP1,LATS1,KLHL12,O*

			R9Q1,OR4K17,SPARCL1,WNT2B,TMDD1,TNFRSF10B,FBXW11,PTPRO,ARHGAP6,CHD5,JAK1,ITGA9,ULK2,KCNB1,RADIL,ZMIZ1,RP1L1,GRM4,AIM2,ANK3,KITLG,SV2C,ARID4B,GMDS,MAP4K4,CNIH3,NR1I2,HIP1,RELN,MFHAS1,OR51B5,CRKL,ARHGEF10,PTPN13,VAV3,RPS6KC1,INPP5A,CD40LG,VRK1,GPAT3,SPRING1,SLC16A10,CCDC88A,APBA1,PLCE1,TNIP3,GNAL,STK32C,BAK1,CASTOR2,LAX1,CACTIN,STK26,TENM4,PPP3CA,CHRNA5,MKNK1,DYSF,NKD1,RBPJ,CYRIB,C3,RIN2,PDE6C,PLEKHG7,DENND1A,OR52D1,MBD5,ASAH2,STON1,ASH1L,RSPO2,OR5K1,OR13C9,DISC1,PROX1,BMP6,RALGPS1,LRRC2,CREBBP,ITGB1,NFKBIZ,CORO1C,NSG2,NEFL,STK3,CHN2,FCAR,HNF4G,GABRG3,SH2D3C,RAMP3,GNG2,FRS3,LIMS1,DOCK4,ACKR2,MCTP2,GLCE,GFRA2,EPHB1,CASP8AP2,RPH3A,CYTH4,PLPP4,UBR1,COL4A2,SLC1A6,TCF7L1,ARHGAP32,DYRK3,CHRNA6,HDAC4,GDNF,FYB1,CALCB,UNC5C,TLR7,CFL1,PLEKHG4B,RBBP8,RNASEH2B,TAAR2,AFDN,LRP2,MTDH,SEMA6D,ZDHHC13,WWC3,IL17RA,CAMK4,VWC2,TMEM108,TPTE,LCP2,GAB3,SEZ6L,SLC39A8,GLI2,TAS2R13,TGFBR3,MAP3K7,ADAM17,SEMA4F,STXBP1,OR6N1,NUAK2,PLEKHG1,ELP2,CYP8B1,APC,GNA12,IL18R1,PEAR1,CDA,PPARGC1B,ESR2,ERC1,ROS1,AGO3,MOB3B,IQGAP2,CLPB,SPINK1,CTNND2,RASSF3,PACSIN2,DPYSL2,RAB8B,GRM8,PAIP2,PTPN11,MAPKAP1,COL15A1,EVC2,MGAT5,MAEL,PIGR,NRP2,SIPA1L2,PLXNC1,NR3C1,FSTL4,ZC3HAV1,ARHGAP28,MTOR,KSR1,CBLB,FAF1,SNAP25,TUFT1,RP1,DEFB124,CHST4,OR11H6,PIP5K1A,GH2,DISP1,SPRED1,ADAM10,GNG4,CNTNAP4,CRIM1,RNF115,CDHR3,PER2,GLI3,KAT6A,NTRK3,RXFP1,GAB2,PDE1A,PDCD10,TFG,HHAT,CCDC68,ADGRG6,SLC4A10,FERMT2,GPR82,TSPOAP1,GLUD1,SYT1,PHIP,SS18,SFRP5,DOCK9,PPARA,CD244,SHISA6,MAP2K5,FGF7,CACNA1A,EDA,TNFSF13B,PTH2R,RBM47,EIF2A,CELSR1,HDAC2,FGD1,TRIM44,INSYN2B,TRIM39,SEMA3D,OR2T8,NPSR1,ACP3,MAGEA1,CADM1,INPP5D,OR4C6,ATF6,PRKAR1B,GBF1,SERPINE1,CCDC88C,TM7SF3,CYTH3,AKAP13,HBS1L,GCG,ARFGEF1,TKFC,PTPN3,STAC,SCN8A,RALGAPA1,SSX2IP,RFX4,OR6K6,REM1,ACTA2,FREM1,RIPOR2,KDM3A,OR2L3,TAOK1,GPR39,ADGRA3,CASK,HSPG2,FMN2,PCNT
GO:0050794	regulation of cellular process	0.000043051778582433895	*RIT2,SLMAP,KLHL13,ABCB7,PBX4,NHLRC3,MYO9B,GPSM2,COL4A5,ERG,PARN,CYTH1,STK16,XK,SEMA4D,MET,MAX,TEAD1,BRCA1,EIF4G3,BAZ1B,CEP350,STAU2,SIAH3,DUS3L,PIWIL3,CHMP1B,MIR654,DRD3,ELAPOR2,ATP11C,ZNF534,TOX3,DNAH11,PTPN5,TACR1,SNX25,SDC2,GPHB5,CIITA,ARAP1,CCR3,ANKLE2,CEP85,RPRD1B,AHNAK,ACTR5,EIF4A3,DLEC1,NFE2L2,SCHIP1,MIR376A2,PARG,OLFML2A,UIMC1,TFEC,NFYC,CYP19A1,SLC46A2,GPR158,VRK2,ITSN1,OAS1,PREX2,SCRN1,ELAVL2,CSMD3,FBXL20,SERP1,POLR1A,RAB11FIP4,HSFX4,SNTA1,ASZ1,NUB1,SRGAP2C,WIF1,FLI1,CSNK2A2,ESRRB,ZFHX2,ARHGAP26,TDRD9,LINC-*

			PINT,TRHDE,SETBP1,ZBTB7C,ITGA2,GRIN2A,IL1RAPL1,WDPCP,BLK,GPM6A,WNT16,SETD3,ADCY1,GRID1,RPS6KA2,PLPPR5,TRIM24,ZDHHC17,FIG4,EIF2AK2,FOXP2,F2RL2,KMT2D,LPAR3,FMR1,TXLNG,FOXN3,SP110,SH3BP2,SH3TC2,CEP290,SHQ1,TACC1,RAD9B,OCLN,TUT4,FASTKD5,ITGBL1,NETO1,GBE1,SNX5,CDK13,SNX32,PCGEM1,NEO1,MYT1L,RASA2,ITPKB,PLEKHB2,TCERG1,F10,SRGAP1,DIO2,CABIN1,SYNE1,PSMD10,FBXL17,EOLA1,DHX29,GAS2,GPAM,NABP1,IRF2,RUNX2,LRP12,TSPEAR,ESRP1,HSFX3,BRMS1L,RGS12,CBX3,SLC39A10,CDCA2,ZNF496,KCTD8,ZNF550,MPHOSPH9,RNF10,FAT1,SLC2A2,PIK3R1,STAMBPL1,IL17B,ABCB11,SARNP,CFDP1,COL26A1,SHTN1,ATG12,USP50,CD200R1,POLR3F,SH3GL2,CRTC1,PITPNC1,MYLK3,DOCK7,PDK3,ROR1,SCAP,PAX5,AAK1,CTTN,UTP15,PASK,USP8,GPR75,ARID1A,PARD6B,THEMIS,SMAD6,OR2AJ1,RYBP,NYAP2,PSD3,ABCA13,RFX2,MEF2C,ZNF519,FZR1,BAIAP2L1,DBNL,ELP4,RYK,MACF1,PXT1,TAF4B,MALT1,WEE2,MTM1,TFDP2,KCNE1,PTPRE,UNC5B,ZSCAN5A,SERINC3,ZNF347,PTPN12,PTPRZ1,CD55,PLN,CTDSPL2,SHISA3,NEDD4,NEK4,RBBP9,FLYWCH1,LRRC38,DOCK8,CYSLTR2,DDAH1,SECISBP2,URI1,SCML2,CHD6,FGD4,ZNF609,PRDX1,COX17,HACE1,AEBP2,DSC2,MYO10,OLFM1,CRHR2,MID1,POGK,TSHZ2,SLC12A2,OTX1,EFNB3,TRIM37,DLG3,TRAT1,FLOT2,PVRIG,SV2B,ILK,LEF1,EYA1,ZNF816-ZNF321P,MAPK14,MYBPC3,ZFHX4,RAPGEF1,GPRC6A,SH3GL3,ZNF516,CERKL,SLC1A2,ARHGEF3,EDNRB,GRIN2B,SPDYE3,ARSB,GPR141,IFNGR2,DENND4A,TBC1D20,OXR1,SFMBT2,SETD5,SCAMP5,IL17RB,PARP2,OVOL2,MIR376B,FCHSD2,DNAJB6,MTCL3,OMA1,MTUS1,GABRA6,LEMD3,KHDRBS2,PIEZO1,SOAT1,OR2T11,SLC9B2,NEB,CSRNP3,MRGPRX1,TMEM100,LYPD6,VDR,NEK11,PDPN,CREB3L2,RTN4,GNL3L,CACNA1D,ARHGEF11,PRKAA2,IVNS1ABP,SGPP2,DPT,BBS2,CLSTN1,ZNF155,SERGEF,SCYL2,SKAP1,MAOB,SKIC3,SERPINE2,MYO3A,IL12RB2,PIK3CG,ANKFN1,STIL,TFDP1,IL1R2,ATP6V1E2,HTT,IQGAP1,MYCBP2,WIPI1,LATS1,ZSCAN25,KLHL12,LOC349160,SNX30,FANCI,OR9Q1,OR4K17,SPARCL1,WNT2B,TMDD1,TNFRSF10B,MAP2,FBXW11,MTCL1,PTPRO,HSPH1,AZIN2,MIR518C,SMG6,HLA-DMA,PEMT,ARHGAP6,CHD5,JAK1,ITGA9,ULK2,KCNB1,RADIL,ZMIZ1,RP1L1,HSD17B12,GRM4,ANKRD27,AIM2,ANK3,GTF2IRD1,ZNF19,PPP1R14C,KITLG,SV2C,ARID4B,GMDS,ZNF326,MAP4K4,CNIH3,PLEKHM2,NR1I2,ZNF266,HIP1,RAET1E,RFX7,RELN,MFHAS1,OR51B5,MTMR3,CRKL,TENT5A,ARHGEF10,PTPN13,SNAP91,ZNF816,VAV3,RPS6KC1,INPP5A,CD40LG,VRK1,GPAT3,ZNF420,DPYSL3,ATP2B1,SPRING1,CCDC88A,SLC35B4,ARNT2,APBA1,HPSE2,PLCE1,TNIP3,DCAF5,GNAL,STK32C,MSH3,BAK1,CASTOR2,LAX1,CACTIN,STK26,MIR520C,TENM4,DCP1A,PPP3CA,CHRNA5,MIR526A1,MKNK1,DYSF,NKD1,RB

			PJ,CYRIB,SPDYE17,C3,RIN2,PDE6C,PLEKHG7,DLX6-AS1,ASCC1,DENND1A,ENSG00000290317,OR52D1,ADAMTS16,TASP1,MICU1,TAF10,TRIP12,MBD5,BACE2,KDM5A,ZNF549,ZNF320,CNOT6,PKIB,ASAH2,STON1,ASH1L,RSPO2,ZNF747,ZNF805,OR5K1,OR13C9,DISC1,PROX1,BMP6,FMN1,ZNF891,RALGPS1,CTSB,ASAP1,LRRC2,AQP8,CREBBP,ITGB1,BVES,ZNF827,NFKBIZ,CORO1C,KANK3,NSG2,SPIDR,NEFL,ATP6V1A,STK3,CHN2,FCAR,HNF4G,MSR1,EXOSC3,SH2D3C,RAMP3,GNG2,ZNF705A,FRS3,LIMS1,ENPP2,DOCK4,ACKR2,UBL4B,MCTP2,BCL11B,GLCE,KCNIP1,PWWP2A,LOC105373170,GFRA2,TBC1D10A,EPHB1,SSBP2,CASP8AP2,RPH3A,CTTNBP2,CYTH4,NAP1L1,PLPP4,UBR1,CNN2,COL4A2,TCF7L1,ARHGAP32,DYRK3,IP6K2,CHRNA6,HDAC4,ZNF582,GDNF,FYB1,CALCB,MARCHF5,MYLK,UNC5C,CFAP69,TLR7,P3H2,CFL1,KRT20,SLAIN2,HMG20A,PLEKHG4B,RBBP8,MED13,RNASEH2B,PHF2,TAAR2,AFDN,CYTIP,LRP2,MTDH,SEMA6D,EHMT1,ZDHHC13,WWC3,IL17RA,CAMK4,VWC2,RPA3,TMEM108,TPTE,LCP2,GAB3,SEZ6L,SLC39A8,GLI2,TAS2R13,TGFBR3,MAP3K7,GTF2I,ATXN1,ADAM17,SEMA4F,ELAVL1,TRAK1,STXBP1,OR6N1,IDH1,NUAK2,LMOD2,HDGFL2,PLEKHG1,RBAK-RBAKDN,NHLH1,ELP2,CYP8B1,APC,GNA12,IL18R1,INO80,SULT2B1,PEAR1,CDA,PPARGC1B,PHLDB2,ESR2,MIR376C,ERC1,ZNF440,ROS1,RUBCNL,AGO3,PVR,MOB3B,ACER1,IQGAP2,CDYL,RLF,CLPB,SPINK1,ANKRA2,TAF5L,CTNND2,ERI1,RASSF3,PACSIN2,TIA1,DPYSL2,SYNPO2,GIMAP8,RAB8B,METTL8,NSD2,GRM8,SLC23A2,PAIP2,AARS1,PTPN11,JADE3,CARD18,PSMC2,MAPKAP1,COL15A1,EVC2,GON4L,RIC1,MGAT5,MAEL,NUP153,CLPTM1,EFHD1,PIGR,NRP2,SIPA1L2,CAVIN1,PLXNC1,FOXJ2,NR3C1,DLX6,MIR300,BORCS5,FSTL4,ZC3HAV1,ARHGAP28,MTOR,KSR1,CBLB,FAF1,SNAP25,TUFT1,RP1,DEFB124,CHST4,OR11H6,ZNF221,PIP5K1A,GH2,DISP1,ASCC2,SPRED1,ADAM10,GNG4,CNTNAP4,WASHC1,PDS5B,CRIM1,RBM46,RNF115,PER2,PLEKHH2,GLI3,KAT6A,BCOR,NTRK3,RXFP1,TADA1,GAB2,DIS3L2,CTCF,PDE1A,PDCD10,PRAMEF11,TFG,EPHX2,HHAT,CREB5,CCDC68,ADGRG6,SLC4A10,FERMT2,GPR82,TSPOAP1,GLUD1,DNM3,SYT1,PHIP,EPB41,DENND5A,SS18,SFRP5,DOCK9,ZYG11B,RFC2,PPARA,CD244,SHISA6,HDX,MAP2K5,ZNF229,PHF6,HMGB4,EPB41L2,FGF7,TENT5D,PACSIN1,STXBP6,MGA,CACNA1A,EDA,LIMCH1,TNFSF13B,PTH2R,ELOVL6,MICAL2,COL14A1,RBM47,EIF2A,JARID2,CELSR1,HDAC2,FIRRM,FGD1,TRIM44,INSYN2B,TRIM39,SEMA3D,PROM1,OR2T8,NPSR1,EIF4E3,ACP3,KCNH5,RRM1,MAGEA1,CADM1,INPP5D,CLYBL,OR4C6,SPDYE9,ATF6,PRKAR1B,GBF1,SERPINE1,CCDC88C,BACH1,CUL9,PRAMEF4,EWSR1,TM7SF3,CYTH3,SLC26A5,AKAP13,HBS1L,PKM,GCG,ARFGEF1,TKFC,LINC02218,PTPN3,STAC,RALGAPA1,NNT,SSX2IP,ST8SIA2,ELP1,IRF8,HIVEP3,RFX4,OR6K6,ZNF30,REM1,ACTA2,RIPOR2,FUNDC

			2,KDM3A,CAST,OR2L3,TAOK1,GPR39,ADGRA3,CASK,HSPG2,GCNT1,PRDM7,USH1C,FMN2,UBXN2A,PCNT,DNAJC13,PSME3IP1
GO:0030154	cell differentiation	0.00006210253967357704	*RIT2,PBX4,XK,SEMA4D,MET,SCYL3,ATXN10,EIF4G3,STAU2,PIWIL3,DRD3,ATP11C,SDC2,FANCC,DLEC1,NFE2L2,SLC46A2,DTNB,DEUP1,ATP2B2,PREX2,CSMD3,NTN4,PLEKHM3,ASZ1,SRGAP2C,WIF1,FLI1,ESRRB,ZFHX2,TDRD9,ZBTB7C,ITGA2,GRIN2A,NELL2,IL1RAPL1,WDPCP,KIF5C,BLK,GPM6A,SPATA16,WNT16,KRT86,SETD3,ADCY1,RPS6KA2,PLPPR5,ZDHHC17,FIG4,EIF2AK2,CFAP47,KMT2D,LPAR3,FMR1,SH3TC2,CEP290,SH3PXD2B,CFAP91,TUT4,PTPRQ,CDK13,NEO1,MYT1L,ITPKB,PLEKHB2,SYNE1,FBXL17,SLC9A4,RUNX2,LRP12,ESRP1,OSBP2,SLC39A10,RNF10,FAT1,PIK3R1,PGM5,SHTN1,SH3GL2,MYLK3,DOCK7,ROR1,PAX5,CRB1,CTTN,ARID1A,PARD6B,THEMIS,SMAD6,NYAP2,RFX2,MEF2C,FZR1,DBNL,RYK,MACF1,TAF4B,MALT1,WEE2,KCNE1,UNC5B,PTPRZ1,PLN,EML1,NEDD4,RBBP9,SECISBP2,SLC9C1,ZNF609,OLFM1,EFNB3,FLOT2,ILK,LEF1,EYA1,MAPK14,RAPGEF1,SH3GL3,ZNF516,GABRB2,EDNRB,ARSB,TBC1D20,PARP2,ZSWIM6,OVOL2,SLC4A7,DNAJB6,PIEZO1,SOAT1,SLC9B2,NEB,TMEM100,VDR,PDPN,CREB3L2,RTN4,BBS2,SPAG17,SCYL2,SERPINE2,PLAAT3,ANOS1,TPP1,IQGAP1,MYCBP2,LATS1,KLHL12,WNT2B,MAP2,FBXW11,PTPRO,NHSL1,CHD5,JAK1,ACSL6,ULK2,KCNB1,RADIL,ZMIZ1,RP1L1,ANKRD27,ANK3,KITLG,ARID4B,MAP4K4,NR1I2,HIP1,RELN,MFHAS1,CRKL,MYO18B,TENT5A,ARHGEF10,CD40LG,DPYSL3,CCDC88A,BAK1,TENM4,PPP3CA,NKD1,RBPJ,C3,RIN2,PDE6C,TAF10,FRYL,RSPO2,DISC1,PROX1,BMP6,CTSB,ASAP1,VSIG1,AQP8,ITGB1,BVES,DRC7,NFKBIZ,CORO1C,NEFL,NAV2,STK3,C1GALT1,HNF4G,MSR1,TRPC4,BCL11B,CERS3,TSPAN2,EPHB1,NAP1L1,CNN2,COL4A2,ARHGAP32,DYRK3,HDAC4,GDNF,KLHL1,RBM45,UNC5C,CFAP69,CFL1,DTYMK,KRT20,WDR7,HMG20A,CEP85L,PLEKHG4B,CCDC38,AFDN,LRP2,SEMA6D,CAMK4,VWC2,TMEM108,BFSP1,GAB3,GLI2,TGFBR3,ADAM17,SEMA4F,TRAK1,STXBP1,LMOD2,HDGFL2,NHLH1,NHSL2,SNRNP200,APC,GNA12,IL18R1,SULT2B1,PPARGC1B,COL27A1,PHLDB2,ROS1,ACER1,CDYL,CLPB,SPINK1,CTNND2,RDH13,DPYSL2,METTL8,SLC23A2,PTPN11,PSMC2,COL15A1,MAEL,CLPTM1,HSD17B4,EFHD1,RAI14,NRP2,PLXNC1,FOXJ2,NR3C1,DLX6,FSTL4,MTOR,SNAP25,RP1,PIP5K1A,BTBD2,RAB6B,SPRED1,CRIM1,MEI4,RBM46,ABCB5,DPY19L2,PER2,ANKRD24,GLI3,KAT6A,NTRK3,RXFP1,GAB2,CTCF,PRAMEF11,ADGRG6,SLC4A10,FERMT2,DNM3,SYT1,DENND5A,SS18,SFRP5,PPARA,NFASC,FGF7,PACSIN1,MGA,EDA,TNFSF13B,COL14A1,RBM47,JARID2,CELSR1,HDAC2,TPO,SEMA3D,PROM1,CADM1,INPP5D,SERPINE1,CCDC88C,PRAMEF4,AKAP13,ST8SIA2,IRF8,HIVEP3,ACTA2,RIPOR2,KDM3A,TAOK1,UNC45A,HSPG2,SLCO4C1,USH1C,FMN2,NLRP14,DNAJC13,AFG2A*
GO:0048	cellula	0.0000636911355	*RIT2,PBX4,XK,SEMA4D,MET,SCYL3,ATXN10,E*

869	r developmental process	474864	*IF4G3,STAU2,PIWIL3,DRD3,ATP11C,SDC2,FANCC,DLEC1,NFE2L2,SLC46A2,DTNB,DEUP1,ATP2B2,PREX2,CSMD3,NTN4,PLEKHM3,ASZ1,SRGAP2C,WIF1,FLI1,ESRRB,ZFHX2,TDRD9,ZBTB7C,ITGA2,GRIN2A,NELL2,IL1RAPL1,WDPCP,KIF5C,BLK,GPM6A,SPATA16,WNT16,KRT86,SETD3,ADCY1,RPS6KA2,PLPPR5,ZDHHC17,FIG4,EIF2AK2,CFAP47,KMT2D,LPAR3,FMR1,SH3TC2,CEP290,SH3PXD2B,CFAP91,TUT4,PTPRQ,CDK13,NEO1,MYT1L,ITPKB,PLEKHB2,SYNE1,FBXL17,SLC9A4,RUNX2,LRP12,ESRP1,OSBP2,SLC39A10,RNF10,FAT1,PIK3R1,PGM5,SHTN1,SH3GL2,MYLK3,DOCK7,ROR1,PAX5,CRB1,CTTN,ARID1A,PARD6B,THEMIS,SMAD6,NYAP2,RFX2,MEF2C,FZR1,DBNL,RYK,MACF1,TAF4B,MALT1,WEE2,KCNE1,UNC5B,PTPRZ1,PLN,EML1,NEDD4,RBBP9,SECISBP2,SLC9C1,ZNF609,OLFM1,EFNB3,FLOT2,ILK,LEF1,EYA1,MAPK14,RAPGEF1,SH3GL3,ZNF516,GABRB2,EDNRB,ARSB,TBC1D20,PARP2,ZSWIM6,OVOL2,SLC4A7,DNAJB6,PIEZO1,SOAT1,SLC9B2,NEB,TMEM100,VDR,PDPN,CREB3L2,RTN4,BBS2,SPAG17,SCYL2,SERPINE2,PLAAT3,ANOS1,TPP1,IQGAP1,MYCBP2,LATS1,KLHL12,WNT2B,MAP2,FBXW11,PTPRO,NHSL1,CHD5,JAK1,ACSL6,ULK2,KCNB1,RADIL,ZMIZ1,RP1L1,ANKRD27,ANK3,KITLG,ARID4B,MAP4K4,NR1I2,HIP1,RELN,MFHAS1,CRKL,MYO18B,TENT5A,ARHGEF10,CD40LG,DPYSL3,CCDC88A,BAK1,TENM4,PPP3CA,NKD1,RBPJ,C3,RIN2,PDE6C,TAF10,FRYL,RSPO2,DISC1,PROX1,BMP6,CTSB,ASAP1,VSIG1,AQP8,ITGB1,BVES,DRC7,NFKBIZ,CORO1C,NEFL,NAV2,STK3,C1GALT1,HNF4G,MSR1,TRPC4,BCL11B,CERS3,TSPAN2,EPHB1,NAP1L1,CNN2,COL4A2,ARHGAP32,DYRK3,HDAC4,GDNF,KLHL1,RBM45,UNC5C,CFAP69,CFL1,DTYMK,KRT20,WDR7,HMG20A,CEP85L,PLEKHG4B,CCDC38,AFDN,LRP2,SEMA6D,CAMK4,VWC2,TMEM108,BFSP1,GAB3,GLI2,TGFBR3,ADAM17,SEMA4F,TRAK1,STXBP1,LMOD2,HDGFL2,NHLH1,NHSL2,SNRNP200,APC,GNA12,IL18R1,SULT2B1,PPARGC1B,COL27A1,PHLDB2,ROS1,ACER1,CDYL,CLPB,SPINK1,CTNND2,RDH13,DPYSL2,METTL8,SLC23A2,PTPN11,PSMC2,COL15A1,MAEL,CLPTM1,HSD17B4,EFHD1,RAI14,NRP2,PLXNC1,FOXJ2,NR3C1,DLX6,FSTL4,MTOR,SNAP25,RP1,PIP5K1A,BTBD2,RAB6B,SPRED1,CRIM1,MEI4,RBM46,ABCB5,DPY19L2,PER2,ANKRD24,GLI3,KAT6A,NTRK3,RXFP1,GAB2,CTCF,PRAMEF11,ADGRG6,SLC4A10,FERMT2,DNM3,SYT1,DENND5A,SS18,SFRP5,PPARA,NFASC,FGF7,PACSIN1,MGA,EDA,TNFSF13B,COL14A1,RBM47,JARID2,CELSR1,HDAC2,TPO,SEMA3D,PROM1,CADM1,INPP5D,SERPINE1,CCDC88C,PRAMEF4,AKAP13,ST8SIA2,IRF8,HIVEP3,ACTA2,RIPOR2,KDM3A,TAOK1,UNC45A,HSPG2,SLCO4C1,USH1C,FMN2,NLRP14,DNAJC13,AFG2A*
GO:0048858	cell projection morphogenesis	0.00008045012579374711	*MYO9B,XK,SEMA4D,STAU2,SDC2,PREX2,NELL2,IL1RAPL1,KIF5C,GPM6A,ADCY1,ZDHHC17,LPAR3,NEO1,SYNE1,SHTN1,SH3GL2,DOCK7,CTTN,PARD6B,NYAP2,DBNL,RYK,MACF1,UNC5B,PTPRZ1,NEDD4,OLFM1,EFNB3,SNX2,PDPN,RTN4,A*

			NOS1,IQGAP1,MYCBP2,MAP2,PTPRO,ULK2,ANKRD27,ANK3,MAP4K4,RELN,PPP3CA,DISC1,ITGB1,CORO1C,NEFL,ENPP2,BCL11B,EPHB1,ARHGAP32,GDNF,UNC5C,CFL1,PLEKHG4B,LRP2,SEMA6D,TMEM108,GLI2,ADAM17,SEMA4F,TRAK1,STXBP1,CTNND2,PACSIN2,SLC23A2,PTPN11,NRP2,PLXNC1,FSTL4,SNAP25,GLI3,DNM3,SYT1,NFASC,PACSIN1,SEMA3D,ST8SIA2
GO:0007010	cytoskeleton organization	0.00009174335030696469	*GPSM2,MET,BRCA1,CEP350,STAU2,CETN3,CHMP1B,CDC42BPA,TACR1,ARAP1,CEP85,DEUP1,ESPNL,SRGAP2C,ARHGAP26,WDPCP,KRT86,SETD3,CFAP47,TACC1,OCLN,CFAP91,STAG1,GAS2,KIZ,FAT1,PIK3R1,PGM5,SHTN1,CALD1,MYLK3,DOCK7,CTTN,PARD6B,BAIAP2L1,DBNL,MACF1,TUBGCP3,MTM1,EML1,PARVA,FGD4,MID1,TRIM37,FCHSD2,DNAJB6,NEB,ARHGEF11,PRKAA2,BBS2,SPAG17,MYO3A,ANKFN1,STIL,HTT,IQGAP1,MYCBP2,LATS1,MAP2,FBXW11,MTCL1,ARHGAP6,RP1L1,ANK3,HIP1,MYO18B,ARHGEF10,FGD6,DPYSL3,CCDC88A,TACC2,CYRIB,MZT1,DISC1,PROX1,FMN1,CCDC6,ITGB1,DRC7,CORO1C,KANK3,NEFL,ARPC1A,PLS3,CCDC102B,CNN2,CNTLN,TLN2,KLHL1,CFAP69,CFL1,KRT20,SLAIN2,BFSP1,NUAK2,LMOD2,APC,TTLL5,INO80,PPARGC1B,PHLDB2,IQGAP2,FRMD3,PACSIN2,DPYSL2,SYNPO2,PHACTR3,MAPKAP1,ARHGAP28,MTOR,RP1,PIP5K1A,WASHC1,TUBA3D,PLEKHH2,CCDC68,FERMT2,PHIP,EPB41,SS18,NCKAP5,EPB41L2,FGF7,PACSIN1,RTTN,LIMCH1,MICAL2,CELSR1,FGD1,CCDC88C,CUL9,AKAP13,ARFGEF1,SSX2IP,TTC17,HEPACAM2,RIPOR2,PARD3B,XIRP2,TAOK1,USH1C,FMN2,PCNT,GOLGA8B*
GO:0023052	signaling	0.00009504780690989234	*RIT2,MYO9B,GPSM2,COL4A5,ERG,CYTH1,SEMA4D,MET,TEAD1,BRCA1,STAU2,DRD3,ELAPOR2,PTPN5,TACR1,SNX25,GPHB5,ARAP1,CCR3,EIF4A3,NFE2L2,SCHIP1,OLFML2A,UIMC1,CYP19A1,SLC46A2,DTNB,GPR158,VRK2,ITSN1,OAS1,PREX2,FBXL20,NTN4,SERP1,WIF1,CSNK2A2,ESRRB,ARHGAP26,TRHDE,RAB44,ITGA2,GRIN2A,IL1RAPL1,WDPCP,BLK,WNT16,ADCY1,GRID1,RPS6KA2,PLPPR5,TRIM24,ZDHHC17,EIF2AK2,F2RL2,KMT2D,LPAR3,FMR1,FOXN3,SH3BP2,SH3TC2,RAD9B,ITGBL1,NETO1,SNX5,CDK13,NEO1,RASA2,ITPKB,F10,SRGAP1,CABIN1,SYNE1,PSMD10,FBXL17,GAS2,NABP1,RUNX2,LRP12,TSPEAR,BRMS1L,RGS12,SLC39A10,KCTD8,RNF10,FAT1,SLC2A2,PIK3R1,STAMBPL1,IL17B,ABCB11,SHTN1,USP50,CD200R1,SH3GL2,PITPNC1,DOCK7,PDK3,ROR1,SCAP,AAK1,CRB1,CTTN,PASK,USP8,GPR75,ARID1A,THEMIS,SMAD6,OR2AJ1,NYAP2,PSD3,MEF2C,FZR1,DBNL,RYK,MACF1,MALT1,MTM1,PTPRE,UNC5B,SERINC3,PTPN12,CD55,PLN,CTDSPL2,SHISA3,NEDD4,CACNA1E,DOCK8,CYSLTR2,DDAH1,URI1,FGD4,PRDX1,HACE1,MYO10,OLFM1,CRHR2,MID1,SLC12A2,EFNB3,DLG3,TRAT1,FLOT2,PVRIG,SV2B,ILK,LEF1,EYA1,MAPK14,RAPGEF1,GPRC6A,SH3GL3,SLC1A2,ARHGEF3,GABRB2,EDNRB,GRIN2B,GPR141,IFNGR2,DENND4A,IL17RB,PARP2,OVOL2,FCHSD2,OMA1,GABRA6,LEMD3,OR2T11,SLC9B2,MRGPRX1,TMEM100,LYPD6,VDR,NEK11,PDP*

			N,RTN4,CACNA1D,ARHGEF11,PRKAA2,IVNS1ABP,BBS2,CLSTN1,SERGEF,SCYL2,SKAP1,MAOB,SERPINE2,IL12RB2,PIK3CG,STIL,IL1R2,HTT,IQGAP1,LATS1,KLHL12,OR9Q1,OR4K17,SPARCL1,WNT2B,TMDD1,TNFRSF10B,FBXW11,PTPRO,ARHGAP6,CHD5,JAK1,ITGA9,ULK2,KCNB1,RADIL,ZMIZ1,RP1L1,GRM4,AIM2,ANK3,KITLG,SV2C,ARID4B,GMDS,MAP4K4,CNIH3,NR1I2,HIP1,RELN,MFHAS1,OR51B5,CRKL,ARHGEF10,PTPN13,VAV3,RPS6KC1,INPP5A,CD40LG,VRK1,GPAT3,SPRING1,SLC16A10,CCDC88A,APBA1,PLCE1,TNIP3,GNAL,STK32C,BAK1,CASTOR2,LAX1,CACTIN,STK26,TENM4,PPP3CA,CHRNA5,MKNK1,DYSF,NKD1,RBPJ,CYRIB,C3,RIN2,PDE6C,PLEKHG7,DENND1A,OR52D1,MBD5,ASAH2,STON1,ASH1L,RSPO2,OR5K1,OR13C9,DISC1,PROX1,BMP6,RALGPS1,LRRC2,CREBBP,ITGB1,BVES,NFKBIZ,CORO1C,NSG2,NEFL,STK3,CHN2,FCAR,HNF4G,GABRG3,SH2D3C,RAMP3,GNG2,FRS3,LIMS1,DOCK4,ACKR2,MCTP2,GLCE,GFRA2,EPHB1,CASP8AP2,RPH3A,CYTH4,PLPP4,UBR1,COL4A2,SLC1A6,TCF7L1,ARHGAP32,DYRK3,CHRNA6,HDAC4,GDNF,FYB1,CALCB,UNC5C,TLR7,CFL1,PLEKHG4B,RBBP8,RNASEH2B,TAAR2,AFDN,LRP2,MTDH,SEMA6D,ZDHHC13,WWC3,IL17RA,CAMK4,VWC2,TMEM108,TPTE,LCP2,GAB3,SEZ6L,SLC39A8,GLI2,TAS2R13,TGFBR3,MAP3K7,ADAM17,SEMA4F,STXBP1,OR6N1,NUAK2,PLEKHG1,ELP2,CYP8B1,APC,GNA12,IL18R1,PEAR1,CDA,PPARGC1B,ESR2,ERC1,ROS1,AGO3,MOB3B,IQGAP2,CLPB,SPINK1,CTNND2,RASSF3,PACSIN2,DPYSL2,RAB8B,GRM8,PAIP2,PTPN11,MAPKAP1,COL15A1,EVC2,MGAT5,MAEL,PIGR,NRP2,SIPA1L2,PLXNC1,NR3C1,FSTL4,ZC3HAV1,ARHGAP28,MTOR,KSR1,CBLB,FAF1,SNAP25,TUFT1,RP1,DEFB124,CHST4,OR11H6,PIP5K1A,GH2,DISP1,SPRED1,ADAM10,GNG4,CNTNAP4,CRIM1,RNF115,CDHR3,PER2,GLI3,KAT6A,NTRK3,RXFP1,GAB2,PDE1A,PDCD10,TFG,HHAT,CCDC68,ADGRG6,SLC4A10,FERMT2,GPR82,TSPOAP1,GLUD1,SYT1,PHIP,SS18,SFRP5,DOCK9,PPARA,CD244,SHISA6,MAP2K5,FGF7,CACNA1A,EDA,TNFSF13B,PTH2R,RBM47,EIF2A,CELSR1,HDAC2,FGD1,TRIM44,INSYN2B,TRIM39,SEMA3D,OR2T8,NPSR1,ACP3,MAGEA1,CADM1,INPP5D,OR4C6,ATF6,PRKAR1B,GBF1,SERPINE1,CCDC88C,TM7SF3,CYTH3,AKAP13,HBS1L,GCG,ARFGEF1,TKFC,PTPN3,STAC,RALGAPA1,SSX2IP,RFX4,OR6K6,REM1,ACTA2,RIPOR2,KDM3A,OR2L3,TAOK1,GPR39,ADGRA3,CASK,HSPG2,FMN2,PCNT*
GO:0007155	cell adhesion	0.0001239631409866396	*CYTH1,SEMA4D,CCR3,OTOA,NTN4,CNTNAP3B,CLDN10,ITGA2,IL1RAPL1,WDPCP,ITGBL1,NEO1,CLDN14,ITPKB,PDZD2,GPAM,FAT1,PIK3R1,CFDP1,PGM5,COL26A1,CD200R1,CRB1,CTTN,ARID1A,SMAD6,BAIAP2L1,MACF1,MALT1,CD55,DOCK8,PARVA,DSC2,MYO10,EFNB3,DLG3,FLOT2,ILK,LEF1,MAPK14,MYBPC3,RAPGEF1,DNAJB6,PIEZO1,PDPN,RTN4,DPT,CLSTN1,SKAP1,SERPINE2,PSG2,PIK3CG,ANOS1,NECTIN3,FREM3,SPARCL1,PTPRO,HSPH1,HLA-DMA,ARHGAP6,JAK1,ITGA9,RADIL,ZMIZ1,HSD*

			17B12,ANK3,KITLG,MAP4K4,RELN,CRKL,VAV3,CD40LG,APBA1,LAX1,TENM4,PPP3CA,RBPJ,CYRIB,RIN2,CDH10,STON1,DISC1,BMP6,FMN1,ITGB1,BVES,NFKBIZ,CORO1C,LIMS1,EPHB1,TLN2,UNC5C,FLG2,AFDN,CYTIP,VWC2,SLC39A8,GLI2,ADAM17,STXBP1,APC,PEAR1,PHLDB2,ENSG00000287694,PVR,CTNND2,SLC23A2,PTPN11,COL15A1,NRP2,PLXNC1,MTOR,CBLB,FAF1,CHST4,PIP5K1A,ADAM10,CNTNAP4,CDH26,CDHR3,GLI3,FERMT2,PPARA,NFASC,MAP2K5,CADM3,STXBP6,EDA,LIMCH1,TNFSF13B,COL14A1,CELSR1,CADM1,SERPINE1,CYTH3,KLC1,SSX2IP,FREM1,RIPOR2,PARD3B,CASK,HSPG2,GCNT1
GO:0048468	cell development	0.00012406117910389327	*RIT2,PBX4,XK,SEMA4D,MET,SCYL3,ATXN10,EIF4G3,STAU2,DRD3,ATP11C,SDC2,FANCC,NFE2L2,SLC46A2,PREX2,CSMD3,NTN4,SRGAP2C,FLI1,NELL2,IL1RAPL1,WDPCP,KIF5C,GPM6A,SPATA16,ADCY1,RPS6KA2,PLPPR5,ZDHHC17,FIG4,EIF2AK2,CFAP47,KMT2D,LPAR3,FMR1,SH3TC2,CEP290,TUT4,PTPRQ,CDK13,NEO1,MYT1L,ITPKB,SYNE1,SLC9A4,RUNX2,LRP12,OSBP2,RNF10,FAT1,PIK3R1,PGM5,SHTN1,SH3GL2,MYLK3,DOCK7,ROR1,PAX5,CRB1,CTTN,ARID1A,PARD6B,THEMIS,NYAP2,RFX2,MEF2C,DBNL,RYK,MACF1,TAF4B,MALT1,WEE2,KCNE1,UNC5B,PTPRZ1,PLN,EML1,NEDD4,SECISBP2,OLFM1,EFNB3,ILK,LEF1,MAPK14,RAPGEF1,GABRB2,EDNRB,ARSB,TBC1D20,PARP2,OVOL2,SLC4A7,SLC9B2,NEB,RTN4,BBS2,SPAG17,SCYL2,SERPINE2,ANOS1,IQGAP1,MYCBP2,KLHL12,WNT2B,MAP2,FBXW11,PTPRO,CHD5,JAK1,ULK2,KCNB1,RADIL,ZMIZ1,RP1L1,ANKRD27,ANK3,KITLG,ARID4B,MAP4K4,RELN,MFHAS1,CRKL,MYO18B,ARHGEF10,CD40LG,DPYSL3,CCDC88A,BAK1,TENM4,PPP3CA,RBPJ,C3,PDE6C,FRYL,DISC1,PROX1,BMP6,ASAP1,VSIG1,AQP8,ITGB1,BVES,DRC7,NFKBIZ,CORO1C,NEFL,STK3,C1GALT1,BCL11B,TSPAN2,EPHB1,NAP1L1,CNN2,ARHGAP32,DYRK3,HDAC4,GDNF,KLHL1,UNC5C,CFAP69,CFL1,WDR7,PLEKHG4B,CCDC38,AFDN,LRP2,SEMA6D,CAMK4,TMEM108,BFSP1,GAB3,GLI2,TGFBR3,ADAM17,SEMA4F,TRAK1,STXBP1,LMOD2,IL18R1,PPARGC1B,COL27A1,ROS1,CDYL,CLPB,SPINK1,CTNND2,RDH13,DPYSL2,SLC23A2,PTPN11,MAEL,CLPTM1,HSD17B4,EFHD1,NRP2,PLXNC1,NR3C1,FSTL4,MTOR,SNAP25,RP1,RAB6B,MEI4,RBM46,DPY19L2,PER2,ANKRD24,GLI3,KAT6A,NTRK3,GAB2,CTCF,ADGRG6,SLC4A10,DNM3,SYT1,DENND5A,PPARA,NFASC,PACSIN1,TNFSF13B,COL14A1,RBM47,HDAC2,TPO,SEMA3D,INPP5D,CCDC88C,AKAP13,ST8SIA2,IRF8,ACTA2,RIPOR2,KDM3A,TAOK1,USH1C,FMN2*
GO:0120039	plasma membrane bounded cell projection morphogenesis	0.0001245731516317083	*MYO9B,XK,SEMA4D,STAU2,SDC2,PREX2,NELL2,IL1RAPL1,KIF5C,GPM6A,ADCY1,ZDHHC17,LPAR3,NEO1,SYNE1,SHTN1,SH3GL2,DOCK7,CTTN,PARD6B,NYAP2,DBNL,RYK,MACF1,UNC5B,PTPRZ1,NEDD4,OLFM1,EFNB3,SNX2,PDPN,RTN4,ANOS1,IQGAP1,MYCBP2,MAP2,PTPRO,ULK2,ANKRD27,ANK3,MAP4K4,RELN,PPP3CA,DISC1,ITGB1,CORO1C,NEFL,ENPP2,BCL11B,EPHB1,ARHGAP32,GDNF,UNC5C,CFL1,PLEKHG4B,LRP2,SEM*

			A6D,TMEM108,GLI2,ADAM17,SEMA4F,TRAK1,STXBP1,CTNND2,SLC23A2,PTPN11,NRP2,PLXNC1,FSTL4,SNAP25,GLI3,DNM3,SYT1,NFASC,PACSIN1,SEMA3D,ST8SIA2
GO:0051716	cellular response to stimulus	0.0001744833530857437	*RIT2,MYO9B,GPSM2,COL4A5,ERG,CYTH1,SEMA4D,MMS22L,MET,TEAD1,BRCA1,BAZ1B,STAU2,DRD3,ELAPOR2,POLG,PTPN5,TACR1,SNX25,GPHB5,CIITA,FANCC,ARAP1,CCR3,HBG2,CIP2A,ACTR5,EIF4A3,NFE2L2,SCHIP1,PARG,OLFML2A,UIMC1,TFEC,CYP19A1,SLC46A2,GPR158,VRK2,ITSN1,OAS1,PREX2,SERP1,WIF1,CSNK2A2,ESRRB,ARHGAP26,TRHDE,ITGA2,GRIN2A,IL1RAPL1,WDPCP,BLK,WNT16,ADCY1,GRID1,RPS6KA2,PLPPR5,TRIM24,ZDHHC17,EIF2AK2,F2RL2,KMT2D,LPAR3,FMR1,FOXN3,SH3BP2,SH3TC2,RAD9B,ITGBL1,NETO1,SNX5,CDK13,NEO1,RASA2,ITPKB,HBE1,F10,SRGAP1,CABIN1,SYNE1,PSMD10,FBXL17,GAS2,NABP1,RUNX2,LRP12,TSPEAR,UBE2A,BRMS1L,RGS12,CBX3,SLC39A10,KCTD8,CHCHD6,RNF10,FAT1,SLC2A2,PIK3R1,STAMBPL1,IL17B,ABCB11,SHTN1,USP50,CD200R1,SH3GL2,PITPNC1,DOCK7,PDK3,ROR1,SCAP,AAK1,CRB1,CTTN,PASK,USP8,GPR75,ARID1A,THEMIS,SMAD6,OR2AJ1,NYAP2,PSD3,MEF2C,FZR1,DBNL,RYK,MACF1,MALT1,MTM1,KCNE1,PTPRE,UNC5B,SERINC3,PTPN12,ACACA,CD55,PLN,CTDSPL2,SHISA3,NEDD4,NEK4,FLYWCH1,DOCK8,CYSLTR2,DDAH1,PARVA,URI1,FGD4,PRDX1,HACE1,DSC2,MYO10,OLFM1,CRHR2,MID1,SLC12A2,EFNB3,TRAT1,FLOT2,PVRIG,ILK,LEF1,EYA1,MAPK14,RAPGEF1,GPRC6A,SH3GL3,SLC1A2,ARHGEF3,GABRB2,EDNRB,GRIN2B,GPR141,IFNGR2,DENND4A,OXR1,SCAMP5,IL17RB,PARP2,OVOL2,DNAJB6,OMA1,MTUS1,GABRA6,LEMD3,PIEZO1,OR2T11,SLC9B2,MRGPRX1,TMEM100,LYPD6,XPR1,VDR,NEK11,PDPN,CREB3L2,RTN4,CACNA1D,ARHGEF11,PRKAA2,IVNS1ABP,BBS2,TMEM150C,SERGEF,SCYL2,SKAP1,SERPINE2,IL12RB2,PIK3CG,STIL,IL1R2,HTT,IQGAP1,WIPI1,LATS1,KLHL12,FANCI,OR9Q1,ACAA1,OR4K17,SPARCL1,WNT2B,TMDD1,TNFRSF10B,FBXW11,PTPRO,ARHGAP6,CHD5,JAK1,ITGA9,ULK2,KCNB1,RADIL,ZMIZ1,RP1L1,GRM4,ANKRD27,AIM2,ANK3,KITLG,ARID4B,GMDS,MAP4K4,CNIH3,NR1I2,HIP1,RELN,MFHAS1,OR51B5,FBXO32,MTMR3,CRKL,ARHGEF10,SULT1C4,PTPN13,VAV3,RPS6KC1,INPP5A,CD40LG,VRK1,GPAT3,ATP2B1,SPRING1,CCDC88A,UGT3A2,PLCE1,TNIP3,GNAL,STK32C,MSH3,BAK1,CASTOR2,LAX1,CACTIN,STK26,TENM4,PPP3CA,CHRNA5,CES2,MKNK1,NKD1,RBPJ,CYRIB,C3,RIN2,PDE6C,PLEKHG7,ASCC1,DENND1A,OR52D1,MICU1,TAF10,TRIP12,MBD5,ASAH2,STON1,ASH1L,RSPO2,TPMT,OR5K1,OR13C9,DISC1,PROX1,BMP6,RALGPS1,CTSB,LRRC2,AQP8,CREBBP,ITGB1,NFKBIZ,CORO1C,NSG2,SPIDR,NEFL,ATP6V1A,STK3,CHN2,FCAR,HNF4G,MSR1,SH2D3C,APTX,RAMP3,GNG2,FRS3,LIMS1,DOCK4,ACKR2,MCTP2,GLCE,GFRA2,EPHB1,CASP8AP2,CYTH4,PLPP4,UBR1,CNN2,COL4A2,TCF7L1,ARHGAP32,DYRK3,IP6K2,CHRNA6,HDAC4,GDNF,FYB1,CALCB,*

			SEM1,MYLK,UNC5C,TLR7,CFL1,KRT20,PLEKHG4B,RBBP8,RNASEH2B,TAAR2,AFDN,LRP2,MTDH,SEMA6D,LIG1,ZDHHC13,WWC3,IL17RA,CAMK4,VWC2,RPA3,TMEM108,TPTE,LCP2,GAB3,SEZ6L,SLC39A8,GLI2,TAS2R13,TGFBR3,MAP3K7,ADAM17,SEMA4F,OR6N1,NUAK2,ABCC1,HDGFL2,PLEKHG1,ELP2,CYP8B1,APC,GNA12,IL18R1,INO80,PEAR1,CDA,PPARGC1B,ESR2,ERC1,ROS1,AGO3,MOB3B,ACER1,IQGAP2,CLPB,SPINK1,TAF5L,CTNND2,RASSF3,DPYSL2,NSD2,ASCC3,GRM8,SLC23A2,PTPN11,MAPKAP1,COL15A1,EVC2,MGAT5,MAEL,EFHD1,PIGR,NRP2,SIPA1L2,PLXNC1,NR3C1,FSTL4,ZC3HAV1,ARHGAP28,MTOR,KSR1,CBLB,FAF1,TUFT1,RP1,DEFB124,OR11H6,PIP5K1A,GH2,DISP1,ASCC2,SPRED1,ADAM10,GNG4,CRIM1,RNF115,ESD,GLI3,KAT6A,NTRK3,RXFP1,TADA1,GAB2,PDE1A,PDCD10,TFG,HHAT,CCDC68,ADGRG6,FERMT2,GCNA,GPR82,SYT1,PHIP,SS18,SFRP5,DOCK9,RFC2,PPARA,CD244,SHISA6,SLC28A2,MAP2K5,FGF7,CACNA1A,EDA,TNFSF13B,PTH2R,RBM47,EIF2A,CELSR1,HDAC2,FIRRM,TPO,FGD1,GSTA3,TRIM44,INSYN2B,TRIM39,SEMA3D,OR2T8,NPSR1,ACP3,RRM1,MAGEA1,CADM1,INPP5D,OR4C6,ATF6,PRKAR1B,GBF1,SERPINE1,CCDC88C,BACH1,TM7SF3,CYTH3,AKAP13,HBS1L,PKM,GCG,ARFGEF1,TKFC,PTPN3,STAC,RALGAPA1,NNT,SSX2IP,IRF8,RFX4,OR6K6,REM1,ACTA2,RIPOR2,CD69,KDM3A,OR2L3,TAOK1,GPR39,ADGRA3,CASK,HSPG2,FMN2,UBXN2A,PCNT
GO:0006996	organelle organization	0.00023816538156099315	*GPSM2,PARN,MET,ATXN10,BRCA1,BAZ1B,CEP350,STAU2,CETN3,CHMP1B,DRD3,CDC42BPA,POLG,TACR1,COX10,ARAP1,ANKLE2,CEP85,CIP2A,ACTR5,DEUP1,ESPNL,FBF1,ASZ1,SRGAP2C,ARHGAP26,TDRD9,WDPCP,KIF5C,KRT86,SETD3,RPS6KA2,FIG4,AP1S3,CFAP47,FMR1,CEP290,TACC1,SH3PXD2B,OCLN,CFAP91,STAG1,APOO,SYNE1,PSMD10,DHX29,GAS2,CDCA2,CHCHD6,KIZ,MPHOSPH9,FAT1,PIK3R1,PGM5,SHTN1,ATG12,USP50,CALD1,MYLK3,DOCK7,MTX2,SCAP,CTTN,USP8,ARID1A,PARD6B,RFX2,FZR1,BAIAP2L1,DBNL,MACF1,TUBGCP3,WEE2,MTM1,KCNE1,TBC1D31,PLN,EML1,PARVA,FGD4,NDUFAF6,COX17,PACS2,HACE1,ATG4B,MID1,TRIM37,UBXN10,ARSB,TBC1D20,FCHSD2,SLC4A7,DNAJB6,MIGA1,OMA1,PLEKHF2,NEB,RTN4,GNL3L,ARHGEF11,PRKAA2,BBS2,SPAG17,SKIC3,SERPINE2,MYO3A,MICOS10,NUBPL,PLAAT3,TPP1,ANKFN1,STIL,HTT,IQGAP1,MYCBP2,WIPI1,LATS1,KLHL12,SNX30,TSNARE1,MAP2,FBXW11,MTCL1,SMG6,ARHGAP6,CHD5,TOR1AIP2,ULK2,RP1L1,ANKRD27,ANK3,PLEKHM2,HIP1,RELN,MTMR3,CRKL,MYO18B,ARHGEF10,FGD6,NDC1,SNAP91,VAV3,VRK1,DPYSL3,C1ORF146,NDUFB9,CCDC88A,TACC2,MSH3,BAK1,CYRIB,ASCC1,MZT1,ADAMTS16,BACE2,NOMO1,CNOT6,PKIB,DISC1,PROX1,FMN1,CCDC6,DNAJC11,ASAP1,ITGB1,DRC7,ZNF827,CORO1C,KANK3,NEFL,NAV2,ATP6V1A,ARPC1A,PLS3,REEP1,CCDC102B,CNN2,DYRK3,CNTLN,TLN2,KLHL1,MARCHF5,CFAP69,CFL1,KRT20,SLAIN2,CCDC38,PHF2,RPA3,TMEM108*

			,CC2D2B,BFSP1,MEI1,STXBP1,NUAK2,LMOD2,NUP205,ORC4,APC,TTLL5,INO80,SPTSSB,PPARGC1B,PHLDB2,RUBCNL,IQGAP2,FRMD3,PACSIN2,TIA1,DPYSL2,SYNPO2,RAB8B,ASCC3,PHACTR3,MAPKAP1,ACOX1,MAEL,NUP153,TRMT10B,TMEM165,FOXJ2,CEP162,ARHGAP28,MTOR,SNAP25,RP1,GOLGA8A,PIP5K1A,RAB6B,ASCC2,WASHC1,TUBA3D,PDS5B,MEI4,RBM46,ERICH3,PLEKHH2,KAT6A,DIS3L2,CTCF,PDCD10,TFG,CCDC68,FERMT2,SYT1,PHIP,EPB41,SS18,RFC2,NCKAP5,EPB41L2,FGF7,PACSIN1,STXBP6,RTTN,LIMCH1,MICAL2,EIF2A,CELSR1,FGD1,ACP3,RRM1,CFAP161,GBF1,CCDC88C,CUL9,KLC1,AKAP13,HBS1L,ARFGEF1,SSX2IP,TTC17,HEPACAM2,RFX4,RIPOR2,NDUFA10,PARD3B,XIRP2,KDM3A,TAOK1,PRDM7,USH1C,FMN2,UBXN2A,PCNT,DNAJC13,GOLGA8B
GO:0030029	actin filament-based process	0.0002919319081577124	MYO9B,MET,STAU2,CDC42BPA,TACR1,ARAP1,ESPNL,SNTA1,ARHGAP26,SETD3,GAS2,ACAP2,FAT1,PIK3R1,PGM5,SHTN1,CALD1,MYLK3,CTTN,BAIAP2L1,DBNL,KCNE1,PLN,PARVA,FGD4,DSC2,MYBPC3,MYL1,FCHSD2,DNAJB6,NEB,PDPN,CACNA1D,ARHGEF11,MYO3A,IQGAP1,LATS1,ARHGAP6,HIP1,MYO18B,ARHGEF10,FGD6,DPYSL3,CCDC88A,CYRIB,PROX1,FMN1,ITGB1,CORO1C,KANK3,ARPC1A,PLS3,CNN2,KLHL1,CFL1,NUAK2,LMOD2,PPARGC1B,PHLDB2,IQGAP2,FRMD3,PACSIN2,SYNPO2,PHACTR3,ARHGAP28,MTOR,PIP5K1A,WASHC1,PLEKHH2,FERMT2,EPB41,EPB41L2,FGF7,PACSIN1,LIMCH1,MICAL2,CELSR1,FGD1,CCDC88C,AKAP13,ARFGEF1,TTC17,ACTA2,XIRP2,TAOK1,USH1C,FMN2
GO:0051179	localization	0.0003519554971991509	SLC18A1,RIT2,SLMAP,ABCB7,GPSM2,PARN,CYTH1,XK,MMS22L,MET,SCYL3,BRCA1,SNX16,CEP350,STAU2,SIAH3,CETN3,CHMP1B,DRD3,ATP11C,DNAH11,EXOC2,TACR1,SNX25,HEPHL1,HBG2,AHNAK,EIF4A3,NFE2L2,CYP19A1,SLC46A2,GPR158,ITSN1,ATP2B2,SCRN1,FBXL20,SERP1,POLR1A,TPTE2,RAB11FIP4,GRPEL2,SNTA1,CLDN10,CSNK2A2,CLIC5,RBP7,RAB44,SLC16A11,ITGA2,GRIN2A,IL1RAPL1,WDPCP,KIF5C,BLK,GPM6A,CCZ1B,GRID1,EFR3B,ZDHHC17,AP1S3,F2RL2,ANO4,FMR1,SH3TC2,CEP290,SHQ1,SH3PXD2B,OCLN,NETO1,TNPO1,SNX5,SNX32,NEO1,STAG1,TIMM23,HBE1,SLC66A1,APOO,SYNE1,PSMD10,SLC9A4,LRP12,OSBP2,ACAP2,SLC39A10,KCNV1,SLC2A2,PIK3R1,ABCB11,SARNP,TMEM232,SH3GL2,CLBA1,FHIP1A,PITPNC1,SLC30A3,DOCK7,MTX2,XKR4,SCAP,AAK1,CRB1,CTTN,PASK,USP8,PARD6B,ABCA13,MEF2C,DBNL,MACF1,MALT1,MTM1,SLC15A5,KCNE1,MFSD6,SERINC3,PLN,CTDSPL2,SLC16A7,NEDD4,CACNA1E,LRRC38,SLC9C1,COX17,PACS2,ATG4B,SLC7A14,MYO10,MID1,SLC12A2,TRIM37,DLG3,SSR3,TRAT1,FLOT2,SRP68,SV2B,LEF1,MAPK14,MYBPC3,SLC25A13,RAPGEF1,SH3GL3,SLC1A2,GABRB2,EDNRB,GRIN2B,SVOP,ARSB,TBC1D20,SNX2,SCAMP5,FCHSD2,SLC4A7,DNAJB6,SPCS1,GABRA6,PLEKHF2,PIEZO1,SOAT1,SLC9B2,XPR1,VDR,CREB3L2,RTN4,GNL3L,CACNA1D,PRKAA2,CD163,BBS2,CLSTN1,SPAG17,TMEM150C

			,SERGEF,SCYL2,SKAP1,MAOB,SERPINE2,PLAAT3,PIK3CG,TPP1,ANKFN1,STIL,ATP6V1E2,TNPO3,HTT,NUP160,NECTIN3,MYCBP2,WIPI1,LATS1,KLHL12,SNX30,TSNARE1,SPNS3,MAP2,FBXW11,MTCL1,ENSG00000286248,AZIN2,SMG6,JAK1,TOR1AIP2,KCNB1,CANX,GRM4,ANKRD27,ANK3,SV2C,MAP4K4,CNIH3,PLEKHM2,NR1I2,HIP1,RELN,MFHAS1,CRKL,SYTL3,NDC1,SNAP91,VAV3,VRK1,SCN9A,ATP2B1,SLC16A10,CCDC88A,SLC35B4,APBA1,PLCE1,BAK1,CLCC1,TTC9-DT,DCP1A,PPP3CA,CHRNA5,DYSF,C3,RIN2,DENND1A,MZT1,MICU1,NOMO1,STON1,DISC1,BMP6,ASAP1,AQP8,CREBBP,ITGB1,BVES,ZNF827,LMAN2L,RHCE,CORO1C,NSG2,SPIDR,NEFL,ATP6V1A,STK3,MSR1,AP2B1,GABRG3,TRPC4,RAMP3,LIMS1,ENPP2,ACKR2,UBL4B,MCTP2,KCNIP1,TBC1D10A,REEP1,RPH3A,SLCO6A1,TULP2,SLC38A6,VPS29,PLPP4,COPB2,CNN2,SLC1A6,CNTLN,CHRNA6,GDNF,FYB1,MARCHF5,SEM1,MYLK,SLC37A2,CFL1,KRT20,TUSC3,COPG2,CCDC38,AFDN,LRP2,ZDHHC13,REPS1,TMEM108,CC2D2B,TPTE,SLC39A8,UPRT,ATXN1,ELAVL1,TRAK1,STXBP1,NUAK2,TIMM23B,ABCC1,NUP205,SLCO5A1,APC,ERGIC2,PEAR1,STON1-GTF2A1L,PHLDB2,ERC1,ROS1,RUBCNL,SPINK1,PACSIN2,TIA1,DPYSL2,RAB8B,NSD2,SLC23A2,KCND1,PTPN11,EXOC6B,RIC1,NUP153,TRMT10B,PIGR,CAVIN1,TMEM165,NR3C1,BORCS5,MTOR,CBLB,FAF1,SNAP25,PIP5K1A,DISP1,RAB6B,ADAM10,WASHC1,FAM53C,NALF2,ABCB5,PER2,GLI3,KCNK12,SLC35F3,GAB2,CTCF,PDCD10,TFG,LMTK2,CCDC68,SLC4A10,GOLGA7,FERMT2,TSPOAP1,GLUD1,DNM3,SYT1,EPB41,DENND5A,PPARA,SHISA6,SLC28A2,EPB41L2,FGF7,PACSIN1,STXBP6,CACNA1A,SLC22A25,JARID2,CELSR1,NPSR1,KCNH5,PRKAR1B,GBF1,IPO11,SERPINE1,CCDC88C,TM7SF3,CYTH3,SLC26A5,GCG,ARFGEF1,ATP12A,PTPN3,STAC,SCN8A,NNT,SSX2IP,TTC17,IRF8,REM1,AGAP1,RIPOR2,KCNK5,EPS15L1,CYB561A3,PARD3B,CASK,HSPG2,SLCO4C1,GGA2,USH1C,FMN2,PCNT,DNAJC13*
GO:0031344	regulation of cell projection organization	0.0005753276243843233	*RIT2,XK,SEMA4D,STAU2,SDC2,ARAP1,NFE2L2,CSMD3,SRGAP2C,ITGA2,IL1RAPL1,WDPCP,GPM6A,PLPPR5,LPAR3,FMR1,OCLN,NEO1,SYNE1,MPHOSPH9,PIK3R1,SHTN1,ROR1,RYK,MACF1,NEDD4,MYO10,EFNB3,RAPGEF1,GRIN2B,ARSB,PDPN,RTN4,MYO3A,HTT,MYCBP2,MAP2,PTPRO,ULK2,ANKRD27,MAP4K4,RELN,CRKL,DPYSL3,CCDC88A,PLCE1,PPP3CA,ADAMTS16,DISC1,CORO1C,NEFL,ENPP2,GLCE,ARHGAP32,CFL1,SEMA6D,SEMA4F,TRAK1,APC,DPYSL2,RAB8B,PLXNC1,FSTL4,MTOR,SNAP25,RP1,WASHC1,NTRK3,DNM3,DENND5A,PACSIN1,HDAC2,SEMA3D,RIPOR2,USH1C*
GO:0051128	regulation of cellular compone	0.0005780660233691449	*RIT2,ABCB7,GPSM2,PARN,XK,SEMA4D,MET,TEAD1,BRCA1,BAZ1B,STAU2,CHMP1B,DRD3,TACR1,SDC2,ARAP1,AHNAK,ACTR5,NFE2L2,GPR158,CSMD3,SRGAP2C,ITGA2,IL1RAPL1,WDPCP,GPM6A,GRID1,RPS6KA2,PLPPR5,EIF2AK2,LPAR3*

	nt organization		,FMR1,OCLN,NEO1,SYNE1,PSMD10,CDCA2,MPHOSPH9,PIK3R1,SHTN1,ATG12,USP50,MYLK3,ROR1,AAK1,CTTN,USP8,ARID1A,ABCA13,MEF2C,FZR1,BAIAP2L1,RYK,MACF1,WEE2,MTM1,NEDD4,URI1,MYO10,OLFM1,MID1,EFNB3,TRIM37,MAPK14,RAPGEF1,SH3GL3,GRIN2B,ARSB,SETD5,SCAMP5,IL17RB,PARP2,FCHSD2,DNAJB6,OMA1,PIEZO1,NEB,PDPN,RTN4,GNL3L,ARHGEF11,PRKAA2,CLSTN1,SKAP1,SERPINE2,MYO3A,STIL,HTT,MYCBP2,WIPI1,LATS1,SNX30,MAP2,PTPRO,SMG6,ARHGAP6,ULK2,ANKRD27,MAP4K4,HIP1,RELN,MTMR3,CRKL,ARHGEF10,PTPN13,SNAP91,DPYSL3,CCDC88A,PLCE1,BAK1,PPP3CA,DYSF,CYRIB,C3,ADAMTS16,CNOT6,PKIB,STON1,DISC1,PROX1,BMP6,FMN1,ASAP1,ITGB1,ZNF827,CORO1C,KANK3,SPIDR,NEFL,LIMS1,ENPP2,GLCE,EPHB1,CTTNBP2,CNN2,ARHGAP32,IP6K2,MARCHF5,CFL1,SLAIN2,PHF2,SEMA6D,TGFBR3,ADAM17,SEMA4F,TRAK1,STXBP1,LMOD2,HDGFL2,APC,INO80,CDA,PHLDB2,ESR2,ROS1,IQGAP2,ANKRA2,PACSIN2,DPYSL2,SYNPO2,RAB8B,SLC23A2,PTPN11,JADE3,MAPKAP1,RIC1,NRP2,PLXNC1,FSTL4,ARHGAP28,MTOR,FAF1,SNAP25,RP1,ADAM10,GNG4,WASHC1,PLEKHH2,NTRK3,CTCF,PDCD10,FERMT2,DNM3,SYT1,PHIP,DENND5A,PPARA,MAP2K5,PACSIN1,STXBP6,LIMCH1,COL14A1,CELSR1,HDAC2,SEMA3D,SERPINE1,CUL9,AKAP13,ARFGEF1,ST8SIA2,RIPOR2,TAOK1,PRDM7,USH1C
GO:0065008	regulation of biological quality	0.0009809452072906406	SLMAP,PARN,XK,SEMA4D,ACSM3,STAU2,SIAH3,DRD3,ATP11C,TACR1,ARAP1,CCR3,EIF4A3,NFE2L2,CYP19A1,GPR158,SERP1,SNTA1,FLI1,RAB44,ITGA2,GRIN2A,IL1RAPL1,WDPCP,BLK,GPM6A,ADCY1,GRID1,RPS6KA2,TRIM24,F2RL2,LPAR3,ANO4,FMR1,OCLN,TUT4,FASTKD5,NETO1,F10,DIO2,SYNE1,SLC9A4,GAS2,SLC2A2,PIK3R1,CFDP1,SHTN1,SH3GL2,CRTC1,MYLK3,XKR4,AAK1,CTTN,PASK,USP8,MEF2C,BAIAP2L1,RYK,MACF1,KCNE1,CD55,PLN,NEDD4,DDAH1,PARVA,URI1,SLC9C1,FGD4,DSC2,SERPINA1,MYO10,OLFM1,CRHR2,SLC12A2,EFNB3,TRIM37,DLG3,FLOT2,ILK,MAPK14,GABRB2,EDNRB,GRIN2B,TBC1D20,SETD5,RDH16,FCHSD2,SLC4A7,GABRA6,PIEZO1,SLC9B2,NEB,VDR,PDPN,RTN4,GNL3L,CACNA1D,BBS2,CLSTN1,SERPINE2,MYO3A,PIK3CG,HTT,MYCBP2,LATS1,MAP2,FBXW11,PTPRO,IYD,ITGA9,ULK2,KCNB1,HSD17B12,ANK3,MAP4K4,HIP1,RELN,TENT5A,FGD6,PTPN13,VAV3,CD40LG,ATP2B1,SLC16A10,BAK1,DCP1A,PPP3CA,CHRNA5,CYRIB,ADAMTS16,BACE2,CNOT6,DISC1,BMP6,FMN1,CTSB,ASAP1,CREBBP,ITGB1,BVES,KANK3,NEFL,NAV2,ATP6V1A,STK3,AP2B1,GABRG3,APTX,DOCK4,ACKR2,PCSK2,EPHB1,CTTNBP2,SLC1A6,ARHGAP32,IP6K2,CHRNA6,GDNF,CFL1,SEMA6D,TMEM108,SLC39A8,MAP3K7,UPRT,SEMA4F,ELAVL1,STXBP1,LMOD2,ABCC1,GNA12,PEAR1,ERC1,AGO3,SPINK1,RDH13,RAB8B,PAIP2,AARS1,PTPN11,HSD17B4,NRP2,PLXNC1,TMEM165,NR3C1,BORCS5,FSTL4,ARHGAP28,MTOR,SNAP25,ADH7,DISP1,TBXAS1,ADAM10,RBM46,ABCB5,PER2,PLEKHH2,KC

			NK12,DIS3L2,PDCD10,SLC4A10,GOLGA7,FERMT2,GLUD1,DNM3,PHIP,PPARA,SHISA6,SCPEP1,TENT5D,CACNA1A,RBM47,TPO,FGD1,TRIM44,INSYN2B,TRIM39,SEMA3D,KCNH5,PRKAR1B,SERPINE1,CCDC88C,TM7SF3,SLC26A5,GCG,ARFGEF1,PTPN3,SCN8A,NNT,ST8SIA2,ACTA2,KCNK5,FUNDC2,USH1C
GO:0035556	intracellular signal transduction	0.0013533671321706315	*RIT2,MYO9B,CYTH1,SEMA4D,MET,TEAD1,BRCA1,DRD3,GPHB5,ARAP1,CCR3,NFE2L2,SCHIP1,UIMC1,SLC46A2,VRK2,ITSN1,OAS1,PREX2,SERP1,ESRRB,ARHGAP26,GRIN2A,BLK,WNT16,ADCY1,RPS6KA2,TRIM24,ZDHHC17,EIF2AK2,F2RL2,KMT2D,LPAR3,FOXN3,RAD9B,RASA2,ITPKB,F10,PSMD10,NABP1,RUNX2,PIK3R1,STAMBPL1,SHTN1,USP50,CD200R1,DOCK7,PDK3,ROR1,SCAP,PASK,USP8,ARID1A,SMAD6,NYAP2,PSD3,MEF2C,FZR1,DBNL,RYK,MALT1,MTM1,UNC5B,SERINC3,PLN,NEDD4,DOCK8,DDAH1,URI1,FGD4,PRDX1,HACE1,MID1,TRAT1,FLOT2,ILK,MAPK14,RAPGEF1,ARHGEF3,EDNRB,GRIN2B,DENND4A,OVOL2,OMA1,TMEM100,VDR,NEK11,PDPN,RTN4,ARHGEF11,PRKAA2,IVNS1ABP,SERPINE2,PIK3CG,HTT,IQGAP1,LATS1,TNFRSF10B,FBXW11,ARHGAP6,CHD5,JAK1,ZMIZ1,RP1L1,GRM4,AIM2,KITLG,MAP4K4,NR1I2,HIP1,RELN,MFHAS1,CRKL,ARHGEF10,PTPN13,VAV3,GPAT3,SPRING1,CCDC88A,PLCE1,TNIP3,STK32C,BAK1,CASTOR2,LAX1,CACTIN,STK26,PPP3CA,MKNK1,NKD1,CYRIB,RIN2,PLEKHG7,DENND1A,ASH1L,DISC1,PROX1,BMP6,RALGPS1,CREBBP,ITGB1,STK3,CHN2,HNF4G,SH2D3C,RAMP3,LIMS1,DOCK4,ACKR2,MCTP2,GLCE,EPHB1,CYTH4,UBR1,ARHGAP32,DYRK3,HDAC4,TLR7,CFL1,PLEKHG4B,RBBP8,RNASEH2B,LRP2,MTDH,ZDHHC13,WWC3,CAMK4,LCP2,SEZ6L,SLC39A8,TGFBR3,MAP3K7,NUAK2,PLEKHG1,APC,GNA12,IL18R1,PEAR1,PPARGC1B,ESR2,ERC1,ROS1,AGO3,MOB3B,CLPB,SPINK1,PTPN11,MAPKAP1,MAEL,SIPA1L2,NR3C1,ZC3HAV1,ARHGAP28,MTOR,KSR1,CBLB,TUFT1,RP1,DEFB124,PIP5K1A,SPRED1,KAT6A,NTRK3,GAB2,PDCD10,TFG,CCDC68,ADGRG6,FERMT2,SS18,SFRP5,DOCK9,PPARA,MAP2K5,EDA,EIF2A,CELSR1,FGD1,TRIM44,TRIM39,NPSR1,INPP5D,ATF6,GBF1,CCDC88C,CYTH3,AKAP13,GCG,ARFGEF1,TKFC,PTPN3,RALGAPA1,SSX2IP,REM1,ACTA2,RIPOR2,KDM3A,TAOK1,CASK,FMN2*
GO:0141124	intracellular signaling cassette	0.0016009153178307767	*RIT2,MYO9B,CYTH1,SEMA4D,MET,DRD3,ARAP1,CCR3,NFE2L2,VRK2,ITSN1,PREX2,ARHGAP26,GRIN2A,WNT16,ADCY1,ZDHHC17,EIF2AK2,F2RL2,LPAR3,RASA2,ITPKB,PSMD10,RUNX2,PIK3R1,SHTN1,USP50,DOCK7,ROR1,USP8,SMAD6,NYAP2,PSD3,MEF2C,DBNL,RYK,MALT1,MTM1,UNC5B,PLN,NEDD4,DOCK8,DDAH1,FGD4,PRDX1,HACE1,MID1,TRAT1,FLOT2,ILK,MAPK14,RAPGEF1,ARHGEF3,EDNRB,GRIN2B,DENND4A,OVOL2,OMA1,TMEM100,PDPN,RTN4,ARHGEF11,SERPINE2,PIK3CG,HTT,IQGAP1,TNFRSF10B,FBXW11,ARHGAP6,ZMIZ1,GRM4,AIM2,KITLG,MAP4K4,HIP1,RELN,MFHAS1,CRKL,ARHGEF10,PTPN13,VAV3,CCDC88A,PLCE1,TNIP3,LAX1,CACTIN,P*

			PP3CA,NKD1,CYRIB,RIN2,PLEKHG7,DENND1A,ASH1L,BMP6,RALGPS1,CREBBP,ITGB1,STK3,CHN2,SH2D3C,RAMP3,LIMS1,DOCK4,ACKR2,MCTP2,GLCE,EPHB1,CYTH4,ARHGAP32,HDAC4,TLR7,CFL1,PLEKHG4B,LRP2,MTDH,ZDHHC13,SLC39A8,TGFBR3,MAP3K7,PLEKHG1,GNA12,IL18R1,PEAR1,ERC1,ROS1,AGO3,SPINK1,PTPN11,MAPKAP1,SIPA1L2,ZC3HAV1,ARHGAP28,MTOR,KSR1,DEFB124,PIP5K1A,SPRED1,NTRK3,GAB2,PDCD10,TFG,ADGRG6,FERMT2,SFRP5,DOCK9,PPARA,MAP2K5,EDA,CELSR1,FGD1,TRIM44,TRIM39,NPSR1,GBF1,CCDC88C,CYTH3,AKAP13,GCG,ARFGEF1,PTPN3,RALGAPA1,SSX2IP,REM1,ACTA2,RIPOR2,TAOK1
GO:0048812	neuron projection morphogenesis	0.001792911625267679	XK,SEMA4D,STAU2,SDC2,PREX2,NELL2,IL1RAPL1,KIF5C,GPM6A,ADCY1,ZDHHC17,LPAR3,NEO1,SYNE1,SHTN1,SH3GL2,DOCK7,CTTN,PARD6B,NYAP2,DBNL,RYK,MACF1,UNC5B,PTPRZ1,NEDD4,OLFM1,EFNB3,RTN4,ANOS1,IQGAP1,MYCBP2,MAP2,PTPRO,ULK2,ANKRD27,ANK3,MAP4K4,RELN,PPP3CA,DISC1,ITGB1,NEFL,BCL11B,EPHB1,ARHGAP32,GDNF,UNC5C,CFL1,PLEKHG4B,LRP2,SEMA6D,TMEM108,GLI2,ADAM17,SEMA4F,TRAK1,STXBP1,CTNND2,SLC23A2,PTPN11,NRP2,PLXNC1,FSTL4,SNAP25,GLI3,DNM3,SYT1,NFASC,PACSIN1,SEMA3D,ST8SIA2
GO:0120035	regulation of plasma membrane bounded cell projection organization	0.0018987054408400276	RIT2,XK,SEMA4D,STAU2,SDC2,ARAP1,NFE2L2,CSMD3,SRGAP2C,IL1RAPL1,WDPCP,GPM6A,PLPPR5,LPAR3,FMR1,OCLN,NEO1,SYNE1,MPHOSPH9,PIK3R1,SHTN1,ROR1,RYK,MACF1,NEDD4,MYO10,EFNB3,RAPGEF1,GRIN2B,ARSB,PDPN,RTN4,MYO3A,HTT,MYCBP2,MAP2,PTPRO,ULK2,ANKRD27,MAP4K4,RELN,CRKL,DPYSL3,CCDC88A,PLCE1,PPP3CA,ADAMTS16,DISC1,CORO1C,NEFL,ENPP2,ARHGAP32,CFL1,SEMA6D,SEMA4F,TRAK1,APC,DPYSL2,PLXNC1,FSTL4,MTOR,SNAP25,RP1,WASHC1,NTRK3,DNM3,DENND5A,PACSIN1,HDAC2,SEMA3D,RIPOR2,USH1C
GO:0009966	regulation of signal transduction	0.0020479133127888054	RIT2,MYO9B,CYTH1,SEMA4D,MET,BRCA1,DRD3,ELAPOR2,SNX25,GPHB5,ARAP1,EIF4A3,NFE2L2,SCHIP1,SLC46A2,GPR158,VRK2,ITSN1,OAS1,PREX2,WIF1,CSNK2A2,ARHGAP26,GRIN2A,WNT16,TRIM24,ZDHHC17,EIF2AK2,F2RL2,KMT2D,LPAR3,SH3TC2,NETO1,SNX5,CDK13,NEO1,RASA2,ITPKB,F10,SYNE1,PSMD10,FBXL17,GAS2,RUNX2,TSPEAR,BRMS1L,RGS12,SLC39A10,KCTD8,PIK3R1,STAMBPL1,USP50,ROR1,AAK1,CTTN,USP8,SMAD6,PSD3,MEF2C,RYK,MACF1,MALT1,MTM1,PTPRE,UNC5B,SERINC3,PTPN12,CD55,CTDSPL2,SHISA3,NEDD4,DOCK8,URI1,FGD4,PRDX1,MID1,TRAT1,FLOT2,PVRIG,ILK,LEF1,EYA1,MAPK14,RAPGEF1,ARHGEF3,EDNRB,GRIN2B,DENND4A,OVOL2,LEMD3,TMEM100,LYPD6,VDR,RTN4,ARHGEF11,PRKAA2,IVNS1ABP,SCYL2,SERPINE2,PIK3CG,IL1R2,HTT,IQGAP1,LATS1,TNFRSF10B,FBXW11,PTPRO,ARHGAP6,CHD5,ZMIZ1,GRM4,AIM2,KITLG,ARID4B,MAP4K4,CNIH3,HIP1,RELN,MFHAS1,CRKL,ARHGEF10,PTPN13,VAV3,INPP5A,GPAT3,SPRING1,CCDC88A,TNIP3,BAK1,CASTOR2,LAX1,CACTIN,PPP3CA,NKD1,RBPJ,CYRIB,C3,DENND1A,MBD5,ASA

			H2,ASH1L,RSPO2,DISC1,PROX1,BMP6,RALGPS1,CREBBP,ITGB1,STK3,CHN2,RAMP3,LIMS1,GLCE,EPHB1,CYTH4,UBR1,TCF7L1,ARHGAP32,DYRK3,GDNF,TLR7,PLEKHG4B,RNASEH2B,LRP2,MTDH,ZDHHC13,WWC3,VWC2,TMEM108,SEZ6L,SLC39A8,GLI2,TGFBR3,MAP3K7,ADAM17,NUAK2,PLEKHG1,ELP2,APC,GNA12,IL18R1,ROS1,AGO3,MOB3B,SPINK1,CTNND2,PTPN11,MAPKAP1,MGAT5,SIPA1L2,FSTL4,ZC3HAV1,ARHGAP28,MTOR,KSR1,CBLB,FAF1,PIP5K1A,GH2,SPRED1,ADAM10,GNG4,CRIM1,RNF115,GLI3,KAT6A,NTRK3,PDCD10,TFG,FERMT2,PHIP,SFRP5,PPARA,SHISA6,MAP2K5,EDA,RBM47,EIF2A,HDAC2,FGD1,TRIM44,TRIM39,NPSR1,ACP3,MAGEA1,CADM1,INPP5D,ATF6,PRKAR1B,GBF1,SERPINE1,CCDC88C,CYTH3,AKAP13,GCG,ARFGEF1,TKFC,PTPN3,RALGAPA1,SSX2IP,RFX4,ACTA2,RIPOR2,TAOK1
GO:0031175	neuron projection development	0.0024614274164631317	*RIT2,XK,SEMA4D,ATXN10,STAU2,SDC2,NFE2L2,PREX2,CSMD3,SRGAP2C,NELL2,IL1RAPL1,KIF5C,GPM6A,ADCY1,PLPPR5,ZDHHC17,LPAR3,FMR1,NEO1,SYNE1,LRP12,SHTN1,SH3GL2,DOCK7,ROR1,CTTN,PARD6B,NYAP2,MEF2C,DBNL,RYK,MACF1,UNC5B,PTPRZ1,NEDD4,OLFM1,EFNB3,RAPGEF1,ARSB,RTN4,ANOS1,IQGAP1,MYCBP2,MAP2,PTPRO,ULK2,ANKRD27,ANK3,MAP4K4,RELN,CRKL,DPYSL3,CCDC88A,PPP3CA,FRYL,DISC1,ASAP1,ITGB1,NEFL,BCL11B,TSPAN2,EPHB1,ARHGAP32,GDNF,KLHL1,UNC5C,CFL1,PLEKHG4B,LRP2,SEMA6D,TMEM108,GLI2,ADAM17,SEMA4F,TRAK1,STXBP1,CTNND2,DPYSL2,SLC23A2,PTPN11,EFHD1,NRP2,PLXNC1,FSTL4,SNAP25,RAB6B,ANKRD24,GLI3,NTRK3,DNM3,SYT1,DENND5A,NFASC,PACSIN1,HDAC2,SEMA3D,ST8SIA2,RIPOR2,USH1C*
GO:0048583	regulation of response to stimulus	0.0031370312355620085	*RIT2,MYO9B,CYTH1,SEMA4D,MET,BRCA1,DRD3,ELAPOR2,SNX25,ABHD12,GPHB5,ARAP1,ACTR5,EIF4A3,NFE2L2,SCHIP1,PARG,UIMC1,CYP19A1,SLC46A2,GPR158,VRK2,ITSN1,OAS1,PREX2,WIF1,CSNK2A2,ARHGAP26,ITGA2,GRIN2A,BLK,WNT16,TRIM24,ZDHHC17,EIF2AK2,F2RL2,KMT2D,LPAR3,SH3TC2,OCLN,NETO1,SNX5,CDK13,NEO1,RASA2,ITPKB,F10,SYNE1,PSMD10,FBXL17,GAS2,RUNX2,TSPEAR,BRMS1L,RGS12,SLC39A10,KCTD8,PIK3R1,STAMBPL1,ATG12,MASP1,USP50,CD200R1,POLR3F,ROR1,AAK1,CTTN,USP8,ARID1A,THEMIS,SMAD6,PSD3,MEF2C,RYK,MACF1,MALT1,MTM1,PTPRE,UNC5B,SERINC3,PTPN12,CD55,CTDSPL2,SHISA3,NEDD4,DOCK8,DDAH1,URI1,FGD4,PRDX1,MID1,SLC12A2,TRAT1,FLOT2,PVRIG,ILK,LEF1,EYA1,MAPK14,RAPGEF1,ARHGEF3,EDNRB,GRIN2B,DENND4A,OXR1,PARP2,OVOL2,DNAJB6,MTUS1,LEMD3,TMEM100,LYPD6,VDR,RTN4,ARHGEF11,PRKAA2,IVNS1ABP,BBS2,SCYL2,SKAP1,SERPINE2,PIK3CG,IL1R2,HTT,IQGAP1,MYCBP2,LATS1,TNFRSF10B,FBXW11,PTPRO,HLA-DMA,ARHGAP6,CHD5,ZMIZ1,GRM4,AIM2,GTF2IRD1,KITLG,ARID4B,MAP4K4,CNIH3,HIP1,RAET1E,RELN,MFHAS1,FBXO32,CRKL,ARHGEF10,PTPN13,VAV3,INPP5A,GPAT3,ATP2B1,SPRING1*

			,CCDC88A,TNIP3,BAK1,CASTOR2,LAX1,CACTIN,PPP3CA,NKD1,RBPJ,CYRIB,C3,DENND1A,MICU1,TAF10,MBD5,ASAH2,ASH1L,RSPO2,DISC1,PROX1,BMP6,RALGPS1,CREBBP,ITGB1,NFKBIZ,SPIDR,STK3,CHN2,FCAR,EXOSC3,RAMP3,LIMS1,GLCE,EPHB1,CYTH4,PLPP4,UBR1,TCF7L1,ARHGAP32,DYRK3,HDAC4,GDNF,FYB1,MYLK,TLR7,PLEKHG4B,RNASEH2B,LRP2,MTDH,SEMA6D,ZDHHC13,WWC3,IL17RA,VWC2,TMEM108,LCP2,SEZ6L,SLC39A8,GLI2,TGFBR3,MAP3K7,ADAM17,SEMA4F,NUAK2,ABCC1,HDGFL2,PLEKHG1,ELP2,APC,GNA12,IL18R1,INO80,PHLDB2,ROS1,AGO3,C9,PVR,MOB3B,CLPB,SPINK1,TAF5L,CTNND2,NSD2,PTPN11,MAPKAP1,MGAT5,EFHD1,PIGR,SIPA1L2,FSTL4,ZC3HAV1,ARHGAP28,MTOR,KSR1,CBLB,FAF1,DEFB124,PIP5K1A,GH2,SPRED1,ADAM10,GNG4,CRIM1,RNF115,GLI3,KAT6A,NTRK3,TADA1,PDCD10,TFG,FERMT2,PHIP,SFRP5,PPARA,SHISA6,MAP2K5,EDA,TNFSF13B,RBM47,EIF2A,JARID2,HDAC2,FGD1,TRIM44,TRIM39,SEMA3D,NPSR1,ACP3,MAGEA1,CADM1,INPP5D,ATF6,PRKAR1B,GBF1,SERPINE1,CCDC88C,CYTH3,AKAP13,GCG,ARFGEF1,TKFC,PTPN3,RALGAPA1,SSX2IP,RFX4,ACTA2,RIPOR2,TAOK1,CASK,FMN2,UBXN2A,NLRP14
GO:0006810	transport	0.003529883085462714	SLC18A1,RIT2,SLMAP,ABCB7,CYTH1,XK,MET,SNX16,STAU2,CETN3,CHMP1B,DRD3,ATP11C,DNAH11,EXOC2,TACR1,SNX25,HEPHL1,HBG2,AHNAK,EIF4A3,NFE2L2,CYP19A1,SLC46A2,GPR158,ITSN1,ATP2B2,SCRN1,FBXL20,SERP1,TPTE2,RAB11FIP4,GRPEL2,SNTA1,CLDN10,CLIC5,RBP7,RAB44,SLC16A11,ITGA2,GRIN2A,IL1RAPL1,WDPCP,KIF5C,BLK,GPM6A,CCZ1B,GRID1,ZDHHC17,AP1S3,F2RL2,ANO4,FMR1,SH3TC2,CEP290,OCLN,NETO1,TNPO1,SNX5,SNX32,NEO1,TIMM23,HBE1,SLC66A1,APOO,SYNE1,SLC9A4,LRP12,OSBP2,ACAP2,SLC39A10,KCNV1,SLC2A2,PIK3R1,ABCB11,SARNP,SH3GL2,CLBA1,PITPNC1,SLC30A3,DOCK7,MTX2,XKR4,SCAP,AAK1,CTTN,PASK,ABCA13,MEF2C,DBNL,MACF1,MALT1,MTM1,SLC15A5,KCNE1,MFSD6,SERINC3,PLN,CTDSPL2,SLC16A7,NEDD4,CACNA1E,LRRC38,SLC9C1,COX17,ATG4B,SLC7A14,MYO10,SLC12A2,TRIM37,TRAT1,FLOT2,SV2B,MAPK14,MYBPC3,SLC25A13,RAPGEF1,SH3GL3,SLC1A2,GABRB2,EDNRB,GRIN2B,SVOP,ARSB,TBC1D20,SNX2,SCAMP5,FCHSD2,SLC4A7,GABRA6,PLEKHF2,PIEZO1,SOAT1,SLC9B2,XPR1,VDR,CREB3L2,CACNA1D,CD163,BBS2,CLSTN1,SPAG17,TMEM150C,SERGEF,SCYL2,MAOB,SERPINE2,PIK3CG,ATP6V1E2,TNPO3,HTT,NUP160,WIPI1,KLHL12,SNX30,TSNARE1,SPNS3,MAP2,FBXW11,AZIN2,SMG6,KCNB1,CANX,GRM4,ANKRD27,ANK3,SV2C,MAP4K4,CNIH3,NR1I2,HIP1,RELN,SYTL3,NDC1,SNAP91,VAV3,SCN9A,ATP2B1,SLC16A10,CCDC88A,SLC35B4,APBA1,PLCE1,BAK1,CLCC1,PPP3CA,CHRNA5,DYSF,C3,RIN2,DENND1A,MICU1,STON1,BMP6,AQP8,ITGB1,BVES,LMAN2L,RHCE,CORO1C,NSG2,NEFL,ATP6V1A,STK3,MSR1,AP2B1,GABRG3,TRPC4,RAMP3,ENPP2,ACKR2,MCTP2,KCNIP1,TBC1D10A,RPH3A,SLCO6A1,

			SLC38A6,VPS29,PLPP4,COPB2,CNN2,SLC1A6,CHRNA6,GDNF,SEM1,MYLK,SLC37A2,KRT20,TUSC3,COPG2,CCDC38,LRP2,ZDHHC13,REPS1,TMEM108,TPTE,SLC39A8,UPRT,ATXN1,ELAVL1,TRAK1,STXBP1,TIMM23B,ABCC1,NUP205,SLCO5A1,ERGIC2,PEAR1,STON1-GTF2A1L,ERC1,ROS1,RUBCNL,SPINK1,PACSIN2,DPYSL2,RAB8B,SLC23A2,KCND1,PTPN11,EXOC6B,RIC1,NUP153,TRMT10B,PIGR,CAVIN1,TMEM165,BORCS5,CBLB,SNAP25,PIP5K1A,DISP1,RAB6B,WASHC1,FAM53C,NALF2,ABCB5,PER2,GLI3,KCNK12,SLC35F3,GAB2,PDCD10,TFG,LMTK2,SLC4A10,GOLGA7,TSPOAP1,GLUD1,DNM3,SYT1,EPB41,DENND5A,PPARA,SLC28A2,FGF7,PACSIN1,STXBP6,CACNA1A,SLC22A25,NPSR1,KCNH5,PRKAR1B,GBF1,IPO11,SERPINE1,CCDC88C,TM7SF3,CYTH3,SLC26A5,GCG,ARFGEF1,ATP12A,PTPN3,STAC,SCN8A,NNT,SSX2IP,TTC17,IRF8,REM1,AGAP1,KCNK5,EPS15L1,CYB561A3,CASK,HSPG2,SLCO4C1,GGA2,FMN2,PCNT,DNAJC13
GO:0007165	signal transduction	0.003843773912505997	*RIT2,MYO9B,GPSM2,COL4A5,ERG,CYTH1,SEMA4D,MET,TEAD1,BRCA1,DRD3,ELAPOR2,PTPN5,TACR1,SNX25,GPHB5,ARAP1,CCR3,EIF4A3,NFE2L2,SCHIP1,OLFML2A,UIMC1,SLC46A2,GPR158,VRK2,ITSN1,OAS1,PREX2,SERP1,WIF1,CSNK2A2,ESRRB,ARHGAP26,TRHDE,ITGA2,GRIN2A,IL1RAPL1,WDPCP,BLK,WNT16,ADCY1,GRID1,RPS6KA2,PLPPR5,TRIM24,ZDHHC17,EIF2AK2,F2RL2,KMT2D,LPAR3,FMR1,FOXN3,SH3BP2,SH3TC2,RAD9B,ITGBL1,NETO1,SNX5,CDK13,NEO1,RASA2,ITPKB,F10,SRGAP1,CABIN1,SYNE1,PSMD10,FBXL17,GAS2,NABP1,RUNX2,LRP12,TSPEAR,BRMS1L,RGS12,SLC39A10,KCTD8,RNF10,PIK3R1,STAMBPL1,IL17B,ABCB11,SHTN1,USP50,CD200R1,SH3GL2,PITPNC1,DOCK7,PDK3,ROR1,SCAP,AAK1,CTTN,PASK,USP8,GPR75,ARID1A,THEMIS,SMAD6,OR2AJ1,NYAP2,PSD3,MEF2C,FZR1,DBNL,RYK,MACF1,MALT1,MTM1,PTPRE,UNC5B,SERINC3,PTPN12,CD55,PLN,CTDSPL2,SHISA3,NEDD4,DOCK8,CYSLTR2,DDAH1,URI1,FGD4,PRDX1,HACE1,MYO10,OLFM1,CRHR2,MID1,EFNB3,TRAT1,FLOT2,PVRIG,ILK,LEF1,EYA1,MAPK14,RAPGEF1,GPRC6A,SH3GL3,ARHGEF3,EDNRB,GRIN2B,GPR141,IFNGR2,DENND4A,IL17RB,PARP2,OVOL2,OMA1,GABRA6,LEMD3,OR2T11,MRGPRX1,TMEM100,LYPD6,VDR,NEK11,PDPN,RTN4,CACNA1D,ARHGEF11,PRKAA2,IVNS1ABP,BBS2,SERGEF,SCYL2,SKAP1,SERPINE2,IL12RB2,PIK3CG,STIL,IL1R2,HTT,IQGAP1,LATS1,KLHL12,OR9Q1,OR4K17,SPARCL1,WNT2B,TMDD1,TNFRSF10B,FBXW11,PTPRO,ARHGAP6,CHD5,JAK1,ITGA9,ULK2,KCNB1,RADIL,ZMIZ1,RP1L1,GRM4,AIM2,ANK3,KITLG,ARID4B,GMDS,MAP4K4,CNIH3,NR1I2,HIP1,RELN,MFHAS1,OR51B5,CRKL,ARHGEF10,PTPN13,VAV3,RPS6KC1,INPP5A,CD40LG,VRK1,GPAT3,SPRING1,CCDC88A,PLCE1,TNIP3,GNAL,STK32C,BAK1,CASTOR2,LAX1,CACTIN,STK26,TENM4,PPP3CA,CHRNA5,MKNK1,NKD1,RBPJ,CYRIB,C3,RIN2,PDE6C,PLEKHG7,DENND1A,OR52D1,MBD5,ASAH*

			2,STON1,ASH1L,RSPO2,OR5K1,OR13C9,DISC1,PROX1,BMP6,RALGPS1,LRRC2,CREBBP,ITGB1,NFKBIZ,CORO1C,NSG2,STK3,CHN2,FCAR,HNF4G,SH2D3C,RAMP3,GNG2,FRS3,LIMS1,DOCK4,ACKR2,MCTP2,GLCE,GFRA2,EPHB1,CASP8AP2,CYTH4,PLPP4,UBR1,COL4A2,TCF7L1,ARHGAP32,DYRK3,CHRNA6,HDAC4,GDNF,FYB1,CALCB,UNC5C,TLR7,CFL1,PLEKHG4B,RBBP8,RNASEH2B,TAAR2,AFDN,LRP2,MTDH,SEMA6D,ZDHHC13,WWC3,IL17RA,CAMK4,VWC2,TMEM108,TPTE,LCP2,GAB3,SEZ6L,SLC39A8,GLI2,TAS2R13,TGFBR3,MAP3K7,ADAM17,SEMA4F,OR6N1,NUAK2,PLEKHG1,ELP2,CYP8B1,APC,GNA12,IL18R1,PEAR1,CDA,PPARGC1B,ESR2,ERC1,ROS1,AGO3,MOB3B,IQGAP2,CLPB,SPINK1,CTNND2,RASSF3,DPYSL2,GRM8,PTPN11,MAPKAP1,COL15A1,EVC2,MGAT5,MAEL,PIGR,NRP2,SIPA1L2,PLXNC1,NR3C1,FSTL4,ZC3HAV1,ARHGAP28,MTOR,KSR1,CBLB,FAF1,TUFT1,RP1,DEFB124,OR11H6,PIP5K1A,GH2,DISP1,SPRED1,ADAM10,GNG4,CRIM1,RNF115,GLI3,KAT6A,NTRK3,RXFP1,GAB2,PDE1A,PDCD10,TFG,HHAT,CCDC68,ADGRG6,FERMT2,GPR82,PHIP,SS18,SFRP5,DOCK9,PPARA,CD244,SHISA6,MAP2K5,FGF7,EDA,TNFSF13B,PTH2R,RBM47,EIF2A,CELSR1,HDAC2,FGD1,TRIM44,INSYN2B,TRIM39,SEMA3D,OR2T8,NPSR1,ACP3,MAGEA1,CADM1,INPP5D,OR4C6,ATF6,PRKAR1B,GBF1,SERPINE1,CCDC88C,CYTH3,AKAP13,HBS1L,GCG,ARFGEF1,TKFC,PTPN3,STAC,RALGAPA1,SSX2IP,RFX4,OR6K6,REM1,ACTA2,RIPOR2,KDM3A,OR2L3,TAOK1,GPR39,ADGRA3,CASK,HSPG2,FMN2,PCNT*
GO:0034330	cell junction organization	0.00502480464086004	*COL4A5,SEMA4D,STAU2,GPR158,FBF1,SNTA1,CLDN10,SRGAP2C,ITGA2,IL1RAPL1,WDPCP,GPM6A,GRID1,OCLN,CLDN14,PIK3R1,CTTN,PARD6B,MEF2C,DBNL,RYK,MACF1,NEDD4,EFNB3,MAPK14,RAPGEF1,GABRB2,GRIN2B,SETD5,CLSTN1,MYCBP2,SPARCL1,PTPRO,ARHGAP6,GNPAT,ANK3,MAP4K4,RELN,CRKL,PTPN13,C3,CDH10,PDZRN3,STON1,DISC1,BMP6,FMN1,ASAP1,ITGB1,CORO1C,NEFL,LIMS1,EPHB1,CTTNBP2,TLN2,GDNF,CFL1,AFDN,TMEM108,SEZ6L,APC,PHLDB2,ERC1,CTNND2,RAB8B,NRP2,PLXNC1,SNAP25,PIP5K1A,ADAM10,CDH26,CDHR3,FERMT2,DNM3,SHISA6,LIMCH1,ST8SIA2,XIRP2,CAST*
GO:0030036	actin cytoskeleton organization	0.0080785031109417	*MET,STAU2,CDC42BPA,TACR1,ARAP1,ESPNL,ARHGAP26,SETD3,GAS2,FAT1,PIK3R1,PGM5,SHTN1,CALD1,MYLK3,CTTN,BAIAP2L1,DBNL,PARVA,FGD4,FCHSD2,DNAJB6,NEB,ARHGEF11,MYO3A,IQGAP1,LATS1,ARHGAP6,HIP1,MYO18B,ARHGEF10,FGD6,DPYSL3,CCDC88A,CYRIB,PROX1,FMN1,ITGB1,CORO1C,KANK3,ARPC1A,PLS3,CNN2,KLHL1,CFL1,NUAK2,LMOD2,PPARGC1B,PHLDB2,IQGAP2,FRMD3,PACSIN2,SYNPO2,PHACTR3,ARHGAP28,MTOR,PIP5K1A,WASHC1,PLEKHH2,FERMT2,EPB41,EPB41L2,FGF7,PACSIN1,LIMCH1,MICAL2,CELSR1,FGD1,AKAP13,ARFGEF1,TTC17,XIRP2,TAOK1,USH1C,FMN2*
GO:0048667	cell morphogenesis	0.008143526434447837	*XK,SEMA4D,STAU2,SDC2,PREX2,NELL2,IL1RAPL1,WDPCP,KIF5C,ADCY1,ZDHHC17,LPAR3,PTPRQ,NEO1,SYNE1,SHTN1,DOCK7,CTTN,PARD6B*

	involved in neuron differentiation		,MEF2C,RYK,MACF1,UNC5B,PTPRZ1,NEDD4,OLFM1,EFNB3,RTN4,ANOS1,MYCBP2,MAP2,PTPRO,ULK2,ANKRD27,ANK3,RELN,PPP3CA,DISC1,ITGB1,NEFL,BCL11B,EPHB1,ARHGAP32,GDNF,UNC5C,CFL1,PLEKHG4B,SEMA6D,GLI2,ADAM17,SEMA4F,TRAK1,STXBP1,CTNND2,PTPN11,NRP2,PLXNC1,FSTL4,SNAP25,ANKRD24,GLI3,DNM3,NFASC,SEMA3D,RIPOR2
GO:0006796	phosphate-containing compound metabolic process	0.008658607510285368	RIT2,ACOT12,ERG,STK16,SEMA4D,MET,TNS3,SCYL3,ACSM3,BAZ1B,FGGY,CDC42BPA,PTPN5,ABHD12,CIITA,ANKLE2,CEP85,PARG,UPP2,VRK2,PSKH2,TPTE2,PPCDC,CSNK2A2,PTPN4,BLK,ADCY1,RPS6KA2,EFR3B,PLPPR5,FIG4,EIF2AK2,FMR1,OCLN,AK8,PTPRQ,CDK13,ITPKB,PSMD10,GPAM,PIK3R1,SH3GL2,MYLK3,DOCK7,PDK3,ROR1,AAK1,PASK,MEF2C,RYK,MALT1,WEE2,MTM1,PTPRE,PTPN12,PTPRZ1,ACACA,NEK4,URI1,PDXK,DLG3,FAR2,ILK,MAPK14,SLC25A13,CERKL,EDNRB,SPDYE3,SLC4A7,NEK11,PRKAA2,SGPP2,SCYL2,NT5C3A,MYO3A,PLAAT3,PIK3CG,HTT,IQGAP1,LATS1,GNPDA2,TNFRSF10B,FBXW11,PTPRO,SMG6,PEMT,JAK1,GPD2,ACSL6,ULK2,UGP2,GNPAT,HSD17B12,PPP1R14C,KITLG,GMDS,MAP4K4,RELN,PDHB,MTMR3,CRKL,SULT1C4,PTPN13,RPS6KC1,INPP5A,VRK1,GPAT3,NDUFB9,CCDC88A,RNGTT,STK32C,BAK1,LAX1,CACTIN,STK26,PPP3CA,MKNK1,SPDYE17,C3,PKIB,ACSF2,PROX1,BMP6,CORO1C,ATP6V1A,STK3,SH2D3C,ENPP2,EPHB1,NME9,PLPP4,DYRK3,IP6K2,HDAC4,MYLK,ACSBG1,CSGALNACT1,TLR7,DTYMK,RNASEH2B,CAMK4,CLPX,TPTE,LCP2,MAP3K7,UPRT,ADAM17,IDH1,NUAK2,APC,MOCS2,SULT2B1,CDA,PPARGC1B,ERC1,ROS1,MOB3B,SPINK1,UGGT1,SYNPO2,ACYP2,PTPN11,MAPKAP1,HSD17B4,ELOVL7,MTOR,KSR1,PIP5K1A,GH2,KYNU,ADAL,SPRED1,ADAM10,PFKFB3,LHPP,NTRK3,PDE1A,PDCD10,EPHX2,LMTK2,FERMT2,PHIP,PPARA,CD244,MAP2K5,FGF7,PACSIN1,LIMCH1,GNPNAT1,MTMR7,ELOVL6,HDAC2,FIRRM,ACP3,RRM1,CADM1,INPP5D,SPDYE9,PRKAR1B,CCDC88C,PKM,GCG,TKFC,PTPN3,NNT,NDUFA10,TAOK1,CASK
GO:0006793	phosphorus metabolic process	0.009377847883110265	RIT2,ACOT12,ERG,STK16,SEMA4D,MET,TNS3,SCYL3,ACSM3,BAZ1B,FGGY,CDC42BPA,PTPN5,ABHD12,CIITA,ANKLE2,CEP85,PARG,UPP2,VRK2,PSKH2,TPTE2,PPCDC,CSNK2A2,PTPN4,BLK,ADCY1,RPS6KA2,EFR3B,PLPPR5,FIG4,EIF2AK2,FMR1,OCLN,AK8,PTPRQ,CDK13,ITPKB,PSMD10,GPAM,PIK3R1,SH3GL2,MYLK3,DOCK7,PDK3,ROR1,AAK1,PASK,MEF2C,RYK,MALT1,WEE2,MTM1,PTPRE,PTPN12,PTPRZ1,ACACA,NEK4,URI1,PDXK,DLG3,FAR2,ILK,MAPK14,SLC25A13,CERKL,EDNRB,SPDYE3,SLC4A7,NEK11,PRKAA2,SGPP2,SCYL2,NT5C3A,MYO3A,PLAAT3,PIK3CG,HTT,IQGAP1,LATS1,GNPDA2,TNFRSF10B,FBXW11,PTPRO,SMG6,PEMT,JAK1,GPD2,ACSL6,ULK2,UGP2,GNPAT,HSD17B12,PPP1R14C,KITLG,GMDS,MAP4K4,RELN,PDHB,MTMR3,CRKL,SULT1C4,PTPN13,RPS6KC1,INPP5A,VRK1,GPAT3,NDUFB9,CCDC88A,RNGTT,STK32C,BAK1,LAX1,CACTIN,STK26,PPP3CA,MKNK1,SPDYE17,C3,PK

			IB,ACSF2,PROX1,BMP6,CORO1C,ATP6V1A,STK3,SH2D3C,ENPP2,EPHB1,NME9,PLPP4,DYRK3,IP6K2,HDAC4,MYLK,ACSBG1,CSGALNACT1,TLR7,DTYMK,RNASEH2B,CAMK4,CLPX,TPTE,LCP2,MAP3K7,UPRT,ADAM17,IDH1,NUAK2,APC,MOCS2,SULT2B1,CDA,PPARGC1B,ERC1,ROS1,MOB3B,SPINK1,UGGT1,SYNPO2,ACYP2,PTPN11,MAPKAP1,HSD17B4,ELOVL7,MTOR,KSR1,PIP5K1A,GH2,KYNU,ADAL,SPRED1,ADAM10,PFKFB3,LHPP,NTRK3,PDE1A,PDCD10,EPHX2,LMTK2,FERMT2,PHIP,PPARA,CD244,MAP2K5,FGF7,PACSIN1,LIMCH1,GNPNAT1,MTMR7,ELOVL6,HDAC2,FIRRM,ACP3,RRM1,CADM1,INPP5D,SPDYE9,PRKAR1B,CCDC88C,PKM,GCG,TKFC,PTPN3,NNT,NDUFA10,TAOK1,CASK
GO:0050896	response to stimulus	0.014241762548954366	*RIT2,MYO9B,GPSM2,COL4A5,ERG,CYTH1,STK16,SEMA4D,MMS22L,MET,SCYL3,TEAD1,BRCA1,BAZ1B,STAU2,CHMP1B,DRD3,ELAPOR2,POLG,PTPN5,TACR1,SNX25,ABHD12,GPHB5,CIITA,FANCC,ARAP1,CCR3,HBG2,CIP2A,AHNAK,ACTR5,EIF4A3,DLEC1,NFE2L2,SCHIP1,PARG,OLFML2A,UIMC1,TFEC,CYP19A1,SLC46A2,GPR158,VRK2,ITSN1,OAS1,PREX2,FBXL20,SERP1,NUB1,WIF1,CSNK2A2,CLIC5,ESRRB,ARHGAP26,TRHDE,RAB44,ITGA2,GRIN2A,IL1RAPL1,WDPCP,BLK,GPM6A,WNT16,ADCY1,GRID1,RPS6KA2,PLPPR5,TRIM24,ZDHHC17,HLCS,EIF2AK2,F2RL2,KMT2D,LPAR3,FMR1,FOXN3,SH3BP2,SH3TC2,RAD9B,OCLN,ITGBL1,NETO1,PTPRQ,SNX5,CDK13,NEO1,RASA2,ITPKB,HBE1,F10,SRGAP1,DIO2,CABIN1,SYNE1,PSMD10,FBXL17,GAS2,GPAM,NABP1,IRF2,RUNX2,LRP12,TSPEAR,UBE2A,BRMS1L,RGS12,CBX3,ACAP2,SLC39A10,KCTD8,CHCHD6,RNF10,FAT1,SLC2A2,PIK3R1,STAMBPL1,IL17B,ABCB11,SHTN1,ATG12,MASP1,USP50,CD200R1,POLR3F,SH3GL2,CRTC1,PITPNC1,MYLK3,SLC30A3,DOCK7,PDK3,ROR1,SCAP,AAK1,CRB1,CTTN,PASK,USP8,GPR75,ARID1A,THEMIS,SMAD6,OR2AJ1,NYAP2,PSD3,RFX2,MEF2C,FZR1,DBNL,RYK,MACF1,MALT1,MTM1,KCNE1,PTPRE,UNC5B,SERINC3,PTPN12,ACACA,CD55,PLN,CTDSPL2,SHISA3,NEDD4,NEK4,RBBP9,FLYWCH1,DOCK8,CYSLTR2,DDAH1,PARVA,URI1,FGD4,PRDX1,HACE1,DSC2,SERPINA1,MYO10,OLFM1,CRHR2,MID1,SLC12A2,EFNB3,TRAT1,FLOT2,PVRIG,SRP68,ILK,LEF1,EYA1,MAPK14,SLC25A13,RAPGEF1,GPRC6A,SH3GL3,ZNF516,SLC1A2,ARHGEF3,GABRB2,EDNRB,GRIN2B,ARSB,GPR141,IFNGR2,DENND4A,OXR1,SCAMP5,IL17RB,PARP2,OVOL2,SLC4A7,DNAJB6,OMA1,MTUS1,GABRA6,LEMD3,PIEZO1,OR2T11,SLC9B2,MRGPRX1,TMEM100,LYPD6,XPR1,VDR,NEK11,PDPN,CREB3L2,RTN4,CACNA1D,ARHGEF11,PRKAA2,IVNS1ABP,CD163,BBS2,TMEM150C,SERGEF,SCYL2,SKAP1,NT5C3A,MAOB,SERPINE2,MYO3A,PLAAT3,IL12RB2,PIK3CG,ANOS1,ANKFN1,STIL,TRGV5,IL1R2,HTT,IQGAP1,MYCBP2,WIPI1,LATS1,KLHL12,FANCI,OR9Q1,ACAA1,OR4K17,SPARCL1,WNT2B,TMDD1,TNFRSF10B,FBXW11,PTPRO,HSPH1,HLA-DMA,ARHGAP6,CHD5,JAK1,ITGA9,ULK2,KCNB1*

			,RADIL,ZMIZ1,RP1L1,GNPAT,GRM4,ANKRD27,AIM2,ANK3,GTF2IRD1,KITLG,ARID4B,GMDS,MAP4K4,CNIH3,PLEKHM2,NR1I2,HIP1,RAET1E,RELN,MFHAS1,OR51B5,FBXO32,MTMR3,CRKL,TENT5A,ARHGEF10,SULT1C4,PTPN13,VAV3,RPS6KC1,INPP5A,CD40LG,VRK1,GPAT3,SCN9A,DPYSL3,ATP2B1,SPRING1,ATRN,CCDC88A,ARNT2,UGT3A2,PLCE1,TNIP3,GNAL,STK32C,MSH3,BAK1,CASTOR2,LAX1,CACTIN,STK26,TENM4,PPP3CA,CHRNA5,CES2,MKNK1,DYSF,NKD1,RBPJ,CYRIB,C3,RIN2,PDE6C,PLEKHG7,ASCC1,DENND1A,OR52D1,MICU1,TAF10,TRIP12,MBD5,ASAH2,STON1,ASH1L,RSPO2,TPMT,OR5K1,OR13C9,DISC1,PROX1,BMP6,RALGPS1,CTSB,LRRC2,AQP8,CREBBP,ITGB1,BVES,NFKBIZ,CORO1C,NSG2,SPIDR,NEFL,ATP6V1A,STK3,CHN2,FCAR,HNF4G,MSR1,EXOSC3,GABRG3,SH2D3C,APTX,RAMP3,GNG2,FRS3,LIMS1,ENPP2,DOCK4,ACKR2,MCTP2,GLCE,TSPAN2,GFRA2,EPHB1,CASP8AP2,CYTH4,PLPP4,UBR1,CNN2,COL4A2,TCF7L1,ARHGAP32,DYRK3,IP6K2,CHRNA6,HDAC4,GDNF,FYB1,CALCB,SEM1,GYS2,MYLK,UNC5C,CFAP69,ACSBG1,TLR7,CFL1,DTYMK,KRT20,PLEKHG4B,RBBP8,RNASEH2B,TAAR2,AFDN,LRP2,MTDH,SEMA6D,LIG1,EHMT1,ZDHHC13,WWC3,IL17RA,CAMK4,VWC2,RPA3,TMEM108,TPTE,LCP2,GAB3,SEZ6L,SLC39A8,GLI2,TAS2R13,TGFBR3,MAP3K7,UPRT,ADAM17,SEMA4F,STXBP1,OR6N1,IDH1,NUAK2,ABCC1,HDGFL2,PLEKHG1,ELP2,CYP8B1,APC,GNA12,IL18R1,INO80,PEAR1,CDA,PPARGC1B,PHLDB2,ESR2,ERC1,ROS1,AGO3,C9,PVR,MOB3B,ACER1,IQGAP2,CLPB,SPINK1,TAF5L,CTNND2,RASSF3,RDH13,DPYSL2,NSD2,ASCC3,GRM8,SLC23A2,PTPN11,CARD18,MAPKAP1,COL15A1,EVC2,MGAT5,HAO1,MAEL,EFHD1,PIGR,NRP2,SIPA1L2,PLXNC1,NR3C1,FSTL4,ZC3HAV1,ARHGAP28,MTOR,TRAV5,KSR1,CBLB,FAF1,TUFT1,RP1,DEFB124,ADH7,CHST4,OR11H6,PIP5K1A,GH2,DISP1,KYNU,TBXAS1,ASCC2,SPRED1,ADAM10,GNG4,CRIM1,RNF115,ESD,PER2,GLI3,KAT6A,NTRK3,RXFP1,TADA1,GAB2,PDE1A,PDCD10,TFG,EPHX2,HHAT,CCDC68,ADGRG6,SLC4A10,FERMT2,GCNA,GPR82,SYT1,PHIP,SS18,SFRP5,DOCK9,RFC2,PPARA,CD244,SHISA6,IGLV3-1,SLC28A2,MAP2K5,FGF7,CACNA1A,EDA,TNFSF13B,PTH2R,RBM47,EIF2A,JARID2,CELSR1,HDAC2,FIRRM,TPO,FGD1,GSTA3,TRIM44,INSYN2B,TRIM39,SEMA3D,OR2T8,NPSR1,ACP3,RRM1,MAGEA1,CADM1,INPP5D,OR4C6,ATF6,PRKAR1B,GBF1,SERPINE1,CCDC88C,BACH1,TM7SF3,CYTH3,SLC26A5,AKAP13,HBS1L,PKM,GCG,ARFGEF1,TKFC,PTPN3,STAC,RALGAPA1,NNT,SSX2IP,ST8SIA2,IRF8,RFX4,OR6K6,REM1,ACTA2,RIPOR2,CD69,FUNDC2,KDM3A,OR2L3,TAOK1,GPR39,ADGRA3,CASK,HSPG2,GCNT1,BPIFB3,FMN2,UBXN2A,NLRP14,PCNT,TRDV3
GO:0048513	animal organ development	0.017502310164864105	*PBX4,XK,SEMA4D,MET,TEAD1,STAU2,MMP16,DNAH11,FANCC,EIF4A3,CYP19A1,SLC46A2,GPR158,ATP2B2,NTN4,SERP1,SRGAP2C,WIF1,FLI1,CSNK2A2,ESRRB,ITGA2,GRIN2A,WDPCP,GPM*

			6A,WNT16,KRT86,RPS6KA2,FOXP2,TXLNG,FOXN3,CEP290,TACC1,SH3PXD2B,AK8,PTPRQ,SYNE1,FBXL17,GAS2,RUNX2,TSPEAR,ESRP1,SLC39A10,FAT1,MYLK3,DOCK7,ROR1,PAX5,CRB1,SMAD6,MEF2C,FZR1,RYK,MTM1,TFDP2,KCNE1,PLN,EML1,RBBP9,PARVA,SECISBP2,ZNF609,ATG4B,OLFM1,OTX1,ILK,LEF1,EYA1,MAPK14,MYBPC3,ZNF516,SLC1A2,GABRB2,EDNRB,GRIN2B,TBC1D20,PARP2,ZSWIM6,OVOL2,SLC4A7,DNAJB6,SLC9B2,NEB,TMEM100,VDR,PDPN,CREB3L2,RTN4,BBS2,SCYL2,MAOB,SERPINE2,MYO3A,PLAAT3,COL4A4,STIL,NUP160,NECTIN3,IQGAP1,LATS1,KLHL12,WNT2B,FBXW11,PTPRO,CHD5,RADIL,ZMIZ1,UGP2,RP1L1,GNPAT,AIM2,KITLG,ARID4B,RELN,CRKL,MYO18B,TENT5A,ATP2B1,ATRN,ARNT2,PLCE1,TACC2,BAK1,TENM4,PPP3CA,NKD1,RBPJ,PDE6C,DLX6-AS1,ADAMTS16,TAF10,ASH1L,RSPO2,DISC1,PROX1,BMP6,FMN1,CTSB,ITGB1,BVES,NFKBIZ,CORO1C,NEFL,STK3,C1GALT1,AP2B1,BCL11B,CERS3,PLS3,EPHB1,ARHGAP32,HDAC4,GDNF,KLHL1,MYLK,UNC5C,CSGALNACT1,FLG2,PHF2,RAI2,LRP2,SEMA6D,TMEM108,BFSP1,GLI2,TGFBR3,UPRT,ATXN1,TMEM59L,ADAM17,SEMA4F,IDH1,HDGFL2,APC,TTLL5,PPARGC1B,COL27A1,PHLDB2,ACER1,RDH13,METTL8,NSD2,AARS1,PTPN11,MAPKAP1,HSD17B4,NRP2,NR3C1,DLX6,MTOR,TUFT1,RP1,PIP5K1A,GH2,DISP1,SPRED1,ADAM10,ABCB5,ANKRD24,GLI3,BCOR,NTRK3,RXFP1,ADGRG6,SLC4A10,FERMT2,GLUD1,PPARA,NFASC,MAP2K5,FGF7,EDA,CNTRL,MICAL2,COL14A1,JARID2,CELSR1,HDAC2,TPO,FGD1,SEMA3D,PROM1,RRM1,CADM1,ATF6,SERPINE1,SLC26A5,AKAP13,PTPN3,HIVEP3,RFX4,ACTA2,FREM1,RIPOR2,XIRP2,UNC45A,HSPG2,GCNT1,USH1C,AFG2A
GO:0030010	establishment of cell polarity	0.02107378817056724	*GPSM2,CYTH1,FBF1,WDPCP,FAT1,SHTN1,DOCK7,CRB1,DOCK8,FLOT2,ARHGEF11,ANKFN1,HTT,FBXW11,CRKL,CYRIB,ITGB1,EPHB1,PHLDB2,GBF1,CYTH3,ARFGEF1,RIPOR2,PARD3B,FRMD4B*
GO:0016311	dephosphorylation	0.02209570506677249	*TNS3,PTPN5,TPTE2,PTPN4,PLPPR5,FIG4,PTPRQ,MEF2C,MTM1,PTPRE,PTPN12,PTPRZ1,URI1,SGPP2,IQGAP1,FBXW11,PTPRO,SMG6,MTMR3,PTPN13,INPP5A,RNGTT,PPP3CA,PLPP4,TPTE,PTPN11,LHPP,EPHX2,MTMR7,ACP3,INPP5D,PTPN3*
GO:0051641	cellular localization	0.022430956336438313	*SLC18A1,RIT2,SLMAP,GPSM2,MMS22L,SCYL3,SNX16,CEP350,STAU2,SIAH3,CETN3,CHMP1B,DRD3,DNAH11,EXOC2,SNX25,EIF4A3,GPR158,ITSN1,SCRN1,FBXL20,SERP1,POLR1A,RAB11FIP4,GRPEL2,CSNK2A2,RAB44,GRIN2A,WDPCP,KIF5C,BLK,EFR3B,ZDHHC17,AP1S3,F2RL2,ANO4,FMR1,SH3TC2,CEP290,SH3PXD2B,OCLN,NETO1,TNPO1,SNX5,SNX32,NEO1,TIMM23,SYNE1,PSMD10,ACAP2,SLC2A2,PIK3R1,SARNP,SH3GL2,CLBA1,FHIP1A,SLC30A3,DOCK7,MTX2,SCAP,AAK1,CRB1,CTTN,USP8,PARD6B,ABCA13,MEF2C,MACF1,MALT1,MTM1,SLC15A5,KCNE1,PLN,CTDSPL2,NEDD4,PACS2,ATG4B,MYO10,MID1,SLC12A2,TRIM37,DLG3,SSR3,FLOT2,SRP68,S*

			V2B,LEF1,MAPK14,MYBPC3,SH3GL3,SLC1A2,EDNRB,ARSB,TBC1D20,SNX2,SCAMP5,FCHSD2,DNAJB6,SPCS1,PLEKHF2,SLC9B2,CREB3L2,RTN4,GNL3L,PRKAA2,BBS2,CLSTN1,SPAG17,SERGEF,SCYL2,SKAP1,PIK3CG,TPP1,ANKFN1,STIL,TNPO3,HTT,NUP160,NECTIN3,MYCBP2,WIPI1,LATS1,KLHL12,SNX30,TSNARE1,MAP2,FBXW11,MTCL1,ENSG00000286248,SMG6,JAK1,TOR1AIP2,KCNB1,CANX,GRM4,ANKRD27,ANK3,SV2C,MAP4K4,PLEKHM2,HIP1,RELN,MFHAS1,CRKL,SYTL3,NDC1,SNAP91,VRK1,CCDC88A,APBA1,PLCE1,BAK1,TTC9-DT,DCP1A,PPP3CA,DYSF,DENND1A,NOMO1,STON1,DISC1,BMP6,ASAP1,CREBBP,ITGB1,BVES,ZNF827,LMAN2L,CORO1C,NSG2,SPIDR,NEFL,ATP6V1A,STK3,MSR1,AP2B1,RAMP3,LIMS1,UBL4B,MCTP2,TBC1D10A,REEP1,RPH3A,TULP2,VPS29,COPB2,CNN2,SLC1A6,CNTLN,GDNF,FYB1,MARCHF5,SEM1,CFL1,KRT20,COPG2,CCDC38,AFDN,LRP2,REPS1,TMEM108,CC2D2B,SLC39A8,ATXN1,ELAVL1,TRAK1,STXBP1,NUAK2,TIMM23B,NUP205,APC,ERGIC2,PHLDB2,ERC1,PACSIN2,TIA1,RAB8B,NSD2,PTPN11,EXOC6B,RIC1,NUP153,TRMT10B,PIGR,CAVIN1,BORCS5,MTOR,CBLB,FAF1,SNAP25,PIP5K1A,DISP1,RAB6B,ADAM10,WASHC1,FAM53C,PER2,GLI3,GAB2,CTCF,PDCD10,TFG,LMTK2,CCDC68,GOLGA7,FERMT2,TSPOAP1,GLUD1,DNM3,SYT1,EPB41,DENND5A,SHISA6,EPB41L2,FGF7,PACSIN1,JARID2,CELSR1,NPSR1,PRKAR1B,GBF1,IPO11,CCDC88C,TM7SF3,GCG,ARFGEF1,STAC,SSX2IP,TTC17,AGAP1,RIPOR2,EPS15L1,PARD3B,CASK,GGA2,USH1C,FMN2,PCNT,DNAJC13
GO:0120031	plasma membrane bounded cell projection assembly	0.024029675241776505	*ATXN10,CEP350,STAU2,ARAP1,FBF1,SRGAP2C,WDPCP,GPM6A,PLPPR5,CFAP47,LPAR3,FMR1,CEP290,OCLN,CFAP91,MPHOSPH9,PIK3R1,SHTN1,CTTN,RFX2,TBC1D31,PARVA,FGD4,MYO10,UBXN10,BBS2,SPAG17,MYO3A,HTT,PTPRO,RP1L1,FGD6,VAV3,DPYSL3,CCDC88A,PLCE1,STK26,ADAMTS16,STON1,DISC1,ASAP1,ITGB1,DRC7,CORO1C,MYLK,CFAP69,CCDC38,CC2D2B,APC,CEP162,MTOR,RP1,PIP5K1A,WASHC1,ERICH3,DNM3,FGD1,CFAP161,GBF1,SSX2IP,RFX4,RIPOR2,PCNT*
GO:0043087	regulation of GTPase activity	0.029499297057425263	*SEMA4D,MET,ARAP1,RASA2,DOCK7,DOCK8,FGD4,RAPGEF1,TBC1D20,IQGAP1,MAP4K4,ARHGEF10,FGD6,VAV3,ASAP1,ITGB1,BVES,CORO1C,CHN2,LIMS1,IQGAP2,RIC1,PIP5K1A,NTRK3,FERMT2,DOCK9,FGD1,ARFGEF1,RALGAPA1,RIPOR2*
GO:0050793	regulation of developmental process	0.03056823404891307	*XK,SEMA4D,BRCA1,STAU2,DRD3,ELAPOR2,ATP11C,SDC2,ARAP1,CCR3,ACTR5,NFE2L2,SLC46A2,CSMD3,NTN4,SERP1,SRGAP2C,WIF1,ESRRB,ZFHX2,ZBTB7C,IL1RAPL1,WDPCP,SETD3,FIG4,EIF2AK2,LPAR3,FMR1,TXLNG,CDK13,NEO1,ITPKB,PLEKHB2,SYNE1,GAS2,GPAM,RUNX2,ESRP1,BRMS1L,RNF10,PIK3R1,CFDP1,SHTN1,MYLK3,DOCK7,CTTN,ARID1A,SMAD6,MEF2C,RYK,MACF1,MALT1,WEE2,MTM1,PTPRZ1,NEDD4,DDAH1,PARVA,FGD4,MYO10,OLFM1,SLC12A2,EFNB3,FLOT2,ILK,LEF1,EYA1,MAPK14,SH3GL3,ED*

			NRB,PARP2,OVOL2,OMA1,PIEZO1,SLC9B2,TMEM100,VDR,PDPN,RTN4,BBS2,CLSTN1,SERPINE2,PLAAT3,PIK3CG,MYCBP2,LATS1,WNT2B,MAP2,JAK1,ULK2,ZMIZ1,ANKRD27,KITLG,ARID4B,MAP4K4,RELN,CRKL,TENT5A,FGD6,PTPN13,ATP2B1,ATRN,TENM4,PPP3CA,RBPJ,CYRIB,C3,RIN2,TRIP12,MBD5,RSPO2,DISC1,PROX1,BMP6,ASAP1,ITGB1,BVES,NFKBIZ,CORO1C,NEFL,STK3,MSR1,EXOSC3,ENPP2,BCL11B,EPHB1,NAP1L1,COL4A2,ARHGAP32,HDAC4,GDNF,MARCHF5,UNC5C,CFL1,HMG20A,LRP2,MTDH,SEMA6D,EHMT1,WWC3,CAMK4,VWC2,GLI2,TGFBR3,GTF2I,SEMA4F,ELAVL1,TRAK1,APC,GNA12,INO80,SULT2B1,PPARGC1B,PHLDB2,TAF5L,NSD2,SLC23A2,PTPN11,MAEL,CLPTM1,PLXNC1,FOXJ2,FSTL4,MTOR,SPRED1,ADAM10,CRIM1,PER2,GLI3,KAT6A,BCOR,PDCD10,PRAMEF11,FERMT2,DNM3,SYT1,PHIP,SS18,SFRP5,PPARA,FGF7,PACSIN1,TNFSF13B,COL14A1,JARID2,HDAC2,FGD1,SEMA3D,PROM1,INPP5D,SERPINE1,PRAMEF4,SLC26A5,PKM,ST8SIA2,RIPOR2,KDM3A,HSPG2
GO:0009887	animal organ morphogenesis	0.03373624967810573	PBX4,STAU2,MMP16,DNAH11,FANCC,EIF4A3,NTN4,SERP1,FLI1,ITGA2,WDPCP,WNT16,FOXN3,CEP290,PTPRQ,RUNX2,TSPEAR,FAT1,PAX5,CRB1,SMAD6,MEF2C,RYK,PARVA,OLFM1,OTX1,ILK,LEF1,EYA1,MAPK14,MYBPC3,TBC1D20,OVOL2,TMEM100,VDR,RTN4,BBS2,MYO3A,STIL,NECTIN3,WNT2B,FBXW11,ZMIZ1,CRKL,BAK1,PPP3CA,NKD1,RBPJ,PDE6C,ADAMTS16,TAF10,ASH1L,RSPO2,PROX1,BMP6,FMN1,BCL11B,EPHB1,GDNF,MYLK,CSGALNACT1,LRP2,GLI2,TGFBR3,TMEM59L,APC,PPARGC1B,COL27A1,RDH13,NSD2,NRP2,NR3C1,DLX6,MTOR,TUFT1,RP1,ANKRD24,GLI3,BCOR,RXFP1,ADGRG6,SLC4A10,PPARA,FGF7,EDA,MICAL2,CELSR1,HDAC2,FGD1,PROM1,SERPINE1,ACTA2,FREM1,RIPOR2,XIRP2,GCNT1,USH1C
GO:0030031	cell projection assembly	0.04750662984514967	ATXN10,CEP350,STAU2,ARAP1,FBF1,SRGAP2C,WDPCP,GPM6A,PLPPR5,CFAP47,LPAR3,FMR1,CEP290,OCLN,CFAP91,MPHOSPH9,PIK3R1,SHTN1,CTTN,RFX2,TBC1D31,PARVA,FGD4,MYO10,UBXN10,BBS2,SPAG17,MYO3A,HTT,PTPRO,RP1L1,FGD6,VAV3,DPYSL3,CCDC88A,PLCE1,STK26,ADAMTS16,STON1,DISC1,ASAP1,ITGB1,DRC7,CORO1C,MYLK,CFAP69,CCDC38,CC2D2B,APC,CEP162,MTOR,RP1,PIP5K1A,WASHC1,ERICH3,DNM3,FGD1,CFAP161,GBF1,SSX2IP,RFX4,RIPOR2,PCNT
GO:0051239	regulation of multicellular organismal process	0.048876923004999275	SEMA4D,BRCA1,STAU2,DRD3,ELAPOR2,ATP11C,TACR1,CCR3,ACTR5,EIF4A3,NFE2L2,CYP19A1,SLC46A2,OAS1,ATP2B2,NTN4,SERP1,SNTA1,ESRRB,ZFHX2,ITGA2,GRIN2A,IL1RAPL1,WDPCP,SETD3,FIG4,EIF2AK2,LPAR3,FMR1,TXLNG,NETO1,ITPKB,DIO2,SYNE1,EOLA1,GPAM,RUNX2,ESRP1,BRMS1L,SLC39A10,RNF10,PIK3R1,IL17B,SHTN1,ATG12,USP50,CD200R1,POLR3F,DOCK7,CTTN,PASK,ARID1A,SMAD6,MEF2C,RYK,MACF1,MALT1,MTM1,KCNE1,PTPRZ1,CD55,PLN,DOCK8,DDAH1,DSC2,OLFM1,SLC12A2,EFNB3,FLOT2,LEF1,MAPK14,MYBPC3,ZNF516,EDNRB,GRIN2B,ARSB,SCAMP5,PARP2,OVOL2,OMA1,

			SLC9B2,TMEM100,VDR,PDPN,RTN4,CACNA1D,BBS2,CLSTN1,SERPINE2,IL12RB2,PIK3CG,IL1R2,IQGAP1,LATS1,WNT2B,MAP2,PTPRO,HSPH1,HLA-DMA,PEMT,JAK1,ITGA9,ULK2,ZMIZ1,ANKRD27,AIM2,GTF2IRD1,KITLG,ARID4B,MAP4K4,RELN,MFHAS1,FBXO32,TENT5A,PTPN13,VAV3,CD40LG,ATP2B1,ATRN,LAX1,CACTIN,TENM4,PPP3CA,RBPJ,CYRIB,C3,TRIP12,MBD5,RSPO2,DISC1,PROX1,BMP6,ITGB1,BVES,NFKBIZ,CORO1C,NEFL,STK3,EXOSC3,ENPP2,DOCK4,EPHB1,NAP1L1,COL4A2,ARHGAP32,HDAC4,GDNF,TLR7,CFL1,LRP2,MTDH,SEMA6D,EHMT1,WWC3,IL17RA,CAMK4,TMEM108,GLI2,TGFBR3,MAP3K7,GTF2I,ADAM17,SEMA4F,ELAVL1,TRAK1,CYP8B1,APC,IL18R1,INO80,SULT2B1,PEAR1,PPARGC1B,PHLDB2,SPINK1,TAF5L,TIA1,RAB8B,NSD2,PTPN11,CARD18,MAEL,CLPTM1,PLXNC1,FOXJ2,FSTL4,ZC3HAV1,MTOR,CBLB,DEFB124,TBXAS1,SPRED1,ADAM10,CNTNAP4,PER2,GLI3,KAT6A,BCOR,PDCD10,FERMT2,EPB41,SS18,PPARA,CD244,MAP2K5,FGF7,TNFSF13B,PTH2R,ELOVL6,COL14A1,RBM47,JARID2,HDAC2,SEMA3D,PROM1,NPSR1,ACP3,CADM1,INPP5D,PRKAR1B,SERPINE1,PKM,ST8SIA2,IRF8,REM1,ACTA2,RIPOR2,FUNDC2,KDM3A,HSPG2
CC			
GO:0005737	cytoplasm	1.808954315274598e-28	*SLC18A1,RIT2,SLMAP,KLHL13,ABCB7,ACOT12,NHLRC3,MYO9B,GPSM2,COL4A5,ERG,PARN,CYTH1,CHD9,STK16,XK,MMS22L,TNS3,SCYL3,ATXN10,KIAA1210,BRCA1,EIF4G3,ACSM3,CNBD2,SNX16,FGGY,IFTAP,CEP350,STAU2,SIAH3,PIWIL3,CETN3,CHMP1B,MMP16,FAXC,ATP11C,PRORP,TOX3,DNAH11,CDC42BPA,POLG,EXOC2,PTPN5,SNX25,SDC2,ABHD12,GPHB5,COX10,CIITA,FANCC,ARAP1,UQCRHL,KLHL7,CCR3,ANKLE2,HBG2,CEP85,CIP2A,AHNAK,ACTR5,EIF4A3,DLEC1,NFE2L2,SCHIP1,PARG,TFEC,H2BC18,BLOC1S5-TXNDC5,UPP2,CYP19A1,SLC46A2,DTNB,VRK2,ITSN1,OAS1,DEUP1,ATP2B2,ESPNL,PREX2,SCRN1,FBF1,FBXL20,SERP1,PLEKHM3,ENKUR,TPTE2,RAB11FIP4,GRPEL2,SNTA1,PPCDC,CLDN10,ASZ1,NUB1,MISFA,FLI1,CSNK2A2,SPATS2L,CLIC5,ESRRB,ARHGAP26,TDRD9,RBP7,TRHDE,RAB44,PTPN4,SETBP1,ITGA2,GRIN2A,NELL2,IL1RAPL1,WDPCP,KIF5C,BLK,SPATA16,WNT16,KRT86,SETD3,CCZ1B,ADCY1,RPS6KA2,EFR3B,AVL9,TRIM24,ZDHHC17,HLCS,FIG4,EIF2AK2,AP1S3,CFAP47,LPAR3,GIPC2,FMR1,TXLNG,SH3TC2,CEP290,WDFY3,SHQ1,TACC1,SH3PXD2B,OCLN,CFAP91,TUT4,AK8,FASTKD5,NETO1,TNPO1,GBE1,SNX5,CDK13,SNX32,DNPEP,NEO1,GLT8D1,RASA2,STAG1,CLDN14,ITPKB,PLEKHB2,TIMM23,CAPN11,HBE1,SLC66A1,F10,APOO,SRGAP1,FAM184A,CABIN1,SYNE1,PSMD10,FBXL17,PDZD2,SLC9A4,DHX29,GAS2,GPAM,NABP1,IRF2,RUNX2,FILIP1L,LRRC1,UBE2A,RGS12,PPP6R3,ACOXL,OSBP2,ACAP2,CDCA2,CHCHD6,KIZ,MPHOSPH9,RNF10,FAT1,SLC2A2,PIK3R1,STAMBPL1,AGMO,ABCB11,SARNP,PGM5,COL26A*

			1,SHTN1,ATG12,MASP1,USP50,BTD,CALD1,POLR3F,SH3GL2,CLBA1,HIBADH,CRTC1,PITPNC1,MYLK3,SLC30A3,PDK3,MTX2,FBXL4,ROR1,SCAP,ADAMTSL1,AAK1,CTTN,UTP15,PASK,USP8,PARD6B,THEMIS,SMAD6,RYBP,ABCA13,RFX2,ACY3,MEF2C,FZR1,BAIAP2L1,ST8SIA5,DBNL,ELP4,RYK,MACF1,PXT1,TAF4B,MALT1,DENND11,TUBGCP3,WEE2,MTM1,KCNE1,PTPRE,SERINC3,TBC1D31,PTPN12,CRYZL1,ACACA,CD55,PLN,EML1,SHISA3,SLC16A7,NEDD4,NEK4,FLYWCH1,DOCK8,VWA8,DDAH1,PARVA,PPP1R8,SECISBP2,URI1,ANKRD28,FGD4,PDXK,PRDX1,NDUFAF6,COX17,PACS2,HACE1,SNRPF,ATG4B,SCD5,SLC7A14,DSC2,RBMS2,SERPINA1,GLB1L,MYO10,OLFM1,MID1,SLC12A2,TRIM37,DLG3,FAR2,SSR3,FLOT2,SRP68,SV2B,ILK,LEF1,EYA1,MAPK14,MYBPC3,SLC25A13,RAPGEF1,ALG13,SH3GL3,AKR7A2,CERKL,EIF2S3B,ARHGEF3,GABRB2,GRIN2B,UBXN10,SVOP,ARSB,IFNGR2,DENND4A,TBC1D20,OXR1,SFMBT2,SNX2,GRHPR,SCAMP5,DCAF7,MYL1,KLHL4,SMPX,EIF3F,RDH16,FCHSD2,DNAJB6,MIGA1,OMA1,SPCS1,MTUS1,PLEKHF2,B3GLCT,PIEZO1,SOAT1,SLC9B2,NEB,PTAR1,TMEM100,LYPD6,PDIA5,XPR1,VDR,SPESP1,PDPN,CREB3L2,RTN4,GNL3L,CACNA1D,ARHGEF11,PRKAA2,IVNS1ABP,CD163,SGPP2,BBS2,CLSTN1,SPAG17,TMEM150C,SERGEF,SCYL2,AOPEP,SKAP1,NT5C3A,MAOB,SKIC3,MRPL3,SERPINE2,MYO3A,MICOS10,NUBPL,PLAAT3,POGLUT2,PIK3CG,COL4A4,TPP1,STIL,TFDP1,IL1R2,ATP6V1E2,TNPO3,NBPF10,HTT,NUP160,IQGAP1,MYCBP2,WIPI1,LATS1,KLHL12,SNX30,GNPDA2,TSNARE1,FANCI,THNSL1,ACAA1,SPARCL1,PRSS23,MAP2,FBXW11,MTCL1,HSPH1,ENSG00000286248,AZIN2,IYD,SMG6,HLA-DMA,PEMT,ARHGAP6,PPP2R2B,CHD5,JAK1,GPD2,ACSL6,TOR1AIP2,ULK2,KCNB1,CANX,ZMIZ1,UGP2,RP1L1,GNPAT,HSD17B12,HS6ST3,GRM4,ANKRD27,AIM2,ANK3,GTF2IRD1,PPP1R14C,AMT,KITLG,SV2C,ARID4B,GMDS,MAP4K4,CNIH3,PLEKHM2,HIP1,RELN,MFHAS1,HS3ST4,PDHB,FBXO32,MTMR3,CRKL,MYO18B,TENT5A,ARHGEF10,FGD6,SYTL3,NDC1,SULT1C4,PTPN13,SNAP91,VAV3,RPS6KC1,CD40LG,VRK1,PAPOLB,URM1,GPAT3,DPYSL3,ATP2B1,SPRING1,ATRN,NDUFB9,CCDC88A,SLC35B4,ARNT2,UGT3A2,APBA1,PLCE1,TNIP3,TACC2,CYP39A1,DCAF5,BAK1,MRPL45,CASTOR2,LAX1,CACTIN,STK26,CLCC1,ENOX2,TENM4,TTC9-DT,DCP1A,ZCRB1,PPP3CA,CES2,MKNK1,DYSF,NKD1,RBPJ,CYRIB,C3,RIN2,DENND1A,MZT1,PDZRN3,TASP1,NBPF19,MICU1,EBPL,TAF10,TRIP12,C3ORF52,BACE2,NOMO1,CNOT6,PKIB,GALNT18,ASAH2,FRYL,STON1,PXMP2,ASH1L,TPMT,ACSF2,DISC1,PROX1,MAP3K7CL,FMN1,RALGPS1,CCDC6,MUC5B,CTSB,DNAJC11,ASAP1,AQP8,CREBBP,RPS10-NUDT3,ITGB1,CYB561,DRC7,ZNF827,LMAN2L,DSE,NFKBIZ,CORO1C,KANK3,NSG2,C17ORF80,NEFL,ATP6V1A,STK3,CHN2,RNF150,FCAR,C1GALT1,HNF4G,MSR1,EXOSC3,AP2B1,SH2D3C,AP

			TX,TRPC4,RAMP3,FRS3,CPB1,XYLT2,LIMS1,DOCK4,ACKR2,ARPC1A,UBL4B,MCTP2,CERS3,GLCE,PLS3,PCSK2,KCNIP1,TBC1D10A,EPHB1,SSBP2,REEP1,RPH3A,CTTNBP2,TULP2,NME9,CYTH4,NAP1L1,VPS29,CCDC102B,COPB2,UBR1,CNN2,COL4A2,SLC1A6,TCF7L1,ARHGAP32,MRPS10,DYRK3,IP6K2,KRTAP10-12,CNTLN,TLN2,HDAC4,GDNF,KLHL1,FYB1,TSKS,MARCHF5,RBM45,SEM1,GYS2,LVRN,MYLK,CFAP69,ACSBG1,CSGALNACT1,TLR7,FLG2,SLC37A2,P3H2,CFL1,DTYMK,KRT20,WDR7,SLAIN2,CEP85L,TUSC3,COPG2,PLEKHG4B,CCDC38,AFDN,CYTIP,LRP2,MTDH,SEMA6D,LIG1,ZDHHC13,WWC3,SERPINA9,CAMK4,GALNT14,CLPX,REPS1,FBXL7,TMEM108,TPTE,BFSP1,LCP2,GAB3,SEZ6L,PCYOX1L,SLC39A8,TMEM123,GLI2,AFG2B,MAP3K7,UPRT,GTF2I,ATXN1,TMEM59L,ADAM17,SEMA4F,ELAVL1,S100PBP,TRAK1,STXBP1,IDH1,TIMM23B,LMOD2,TSNAX-DISC1,HDGFL2,MUC13,NUP205,PLEKHG1,ELP2,ORC4,CYP8B1,APC,GNA12,TTLL5,INO80,NAA11,MOCS2,ERGIC2,SULT2B1,SPTSSB,CDA,PPARGC1B,STON1-GTF2A1L,COL27A1,PHLDB2,ESR2,LNX1,ERC1,ROS1,RUBCNL,AGO3,PVR,CKAP4,MOB3B,RPL39L,ACER1,IQGAP2,CDYL,CLPB,ANKRA2,TAF5L,JPT1,CTNND2,DNAH14,ERI1,RASSF3,UGGT1,PACSIN2,RDH13,TIA1,DPYSL2,SYNPO2,GIMAP8,RAB8B,METTL8,NSD2,ASCC3,SLC23A2,RMDN2,PAIP2,AARS1,PTPN11,PSMC2,MAPKAP1,COL15A1,EXOC6B,EVC2,RIC1,MGAT5,ACOX1,USP24,HAO1,MAEL,NUP153,HSD17B4,EFHD1,RNF215,TRMT10B,COL21A1,RAI14,PIGR,SIPA1L2,CAVIN1,KCTD5,METTL15,TMEM165,CEP162,ELOVL7,NR3C1,BORCS5,FSTL4,ZC3HAV1,ARHGAP28,MTOR,KSR1,CBLB,FAF1,SNAP25,TUFT1,RP1,SGSM1,ADH7,CHST4,GOLGA8A,PIP5K1A,BTBD2,GH2,MMACHC,RAB6B,HS6ST2,KYNU,ADAL,TBXAS1,ASCC2,SPRED1,ADAM10,GALC,WASHC1,TUBA3D,CBY2,PDS5B,RBM46,PFKFB3,SPANXD,RNF115,TRIM61,ESD,PER2,ERICH3,PLEKHH2,GLI3,LHPP,KAT6A,TADA1,GAB2,DIS3L2,SNTB2,PDE1A,PDCD10,PRAMEF11,TFG,EPHX2,LMTK2,HHAT,UBE4A,IDH3A,CCDC68,ADGRG6,GOLGA7,FERMT2,TSPOAP1,GLUD1,DNM3,ST3GAL2,SYT1,EPB41,DENND5A,DOCK9,AGBL3,ZYG11B,NFASC,SCPEP1,SLC28A2,EPB41L2,FGF7,TBCA,PACSIN1,STXBP6,RTTN,CACNA1A,EDA,CNTRL,LIMCH1,TNFSF13B,GNPNAT1,MTMR7,ELOVL6,MICAL2,COL14A1,RBM47,EIF2A,JARID2,HDAC2,FIRRM,TGOLN2,FGD1,GSTA3,TRIM44,TRIM39,PROM1,NPSR1,EIF4E3,ACP3,USP48,RRM1,MAGEA1,INPP5D,CFAP161,CLYBL,ATF6,PRKAR1B,GBF1,IPO11,SERPINE1,CCDC88C,BACH1,CUL9,PRAMEF4,EWSR1,CYTH3,KLC1,AKAP13,HBS1L,MLANA,PKM,GCG,ARFGEF1,ATP12A,TKFC,PTPN3,STAC,SCN8A,RALGAPA1,NNT,SSX2IP,ST8SIA2,TTC17,ELP1,IRF8,HIVEP3,HEPACAM2,CCDC198,REM1,ACTA2,AGAP1,RIPOR2,NDUFA10,B3GALNT2,FUNDC2,EPS15L1,CYB561A3,PARD3B,XIRP2,KDM3A,MZT2A,CAST,TAOK1,UNC45A,CA

			SK,HSPG2,SLCO4C1,GCNT1,BPIFB3,GGA2,USH 1C,FMN2,UBXN2A,NLRP14,PCNT,DNAJC13,FRM D4B,AFG2A,GOLGA8B
GO:0005 829	cytosol	7.7140766465320 91e-15	*RIT2,KLHL13,ACOT12,MYO9B,GPSM2,ERG,PAR N,CYTH1,CHD9,STK16,MMS22L,TNS3,SCYL3,A TXN10,EIF4G3,CNBD2,SNX16,IFTAP,CHMP1B, TOX3,CDC42BPA,EXOC2,PTPN5,COX10,CIITA, FANCC,ARAP1,KLHL7,HBG2,CEP85,CIP2A,AHN AK,EIF4A3,DLEC1,NFE2L2,SCHIP1,PARG,TFE C,H2BC18,UPP2,ITSN1,OAS1,PREX2,FBF1,FB XL20,SERP1,TPTE2,PPCDC,NUB1,FLI1,CSNK2 A2,SPATS2L,ESRRB,ARHGAP26,RBP7,PTPN4,S ETBP1,KIF5C,BLK,KRT86,RPS6KA2,EFR3B,TR IM24,HLCS,EIF2AK2,AP1S3,FMR1,TXLNG,CEP 290,WDFY3,SHQ1,TACC1,TUT4,AK8,TNPO1,GB E1,SNX5,CDK13,SNX32,DNPEP,RASA2,STAG1, ITPKB,HBE1,APOO,SRGAP1,CABIN1,PSMD10,F BXL17,PDZD2,DHX29,GAS2,NABP1,IRF2,RUNX 2,LRRC1,UBE2A,RGS12,PPP6R3,OSBP2,CDCA2 ,CHCHD6,RNF10,FAT1,PIK3R1,STAMBPL1,PGM 5,ATG12,MASP1,USP50,CALD1,POLR3F,SH3GL 2,CRTC1,PITPNC1,MYLK3,FBXL4,AAK1,CTTN, PASK,USP8,PARD6B,SMAD6,ACY3,MEF2C,FZR1 ,BAIAP2L1,DBNL,MALT1,TUBGCP3,WEE2,MTM1 ,PTPN12,CRYZL1,ACACA,EML1,SLC16A7,NEDD 4,NEK4,FLYWCH1,DOCK8,DDAH1,PARVA,URI1, ANKRD28,FGD4,PDXK,PRDX1,SNRPF,ATG4B,SL C7A14,RBMS2,MYO10,MID1,TRIM37,DLG3,SRP 68,ILK,MAPK14,MYBPC3,RAPGEF1,AKR7A2,CE RKL,ARHGEF3,DENND4A,SFMBT2,SNX2,GRHPR, DCAF7,MYL1,EIF3F,DNAJB6,NEB,VDR,SPESP1 ,PDPN,GNL3L,ARHGEF11,PRKAA2,IVNS1ABP,C D163,BBS2,SERGEF,AOPEP,SKAP1,NT5C3A,SK IC3,SERPINE2,PLAAT3,POGLUT2,PIK3CG,STI L,TFDP1,ATP6V1E2,HTT,NUP160,IQGAP1,MYC BP2,WIPI1,LATS1,KLHL12,GNPDA2,FANCI,AC AA1,MAP2,FBXW11,HSPH1,AZIN2,SMG6,PEMT, ARHGAP6,PPP2R2B,CHD5,JAK1,ULK2,ZMIZ1,U GP2,GNPAT,ANKRD27,AIM2,ANK3,GTF2IRD1,A RID4B,GMDS,HIP1,FBXO32,MTMR3,CRKL,ARHG EF10,SULT1C4,PTPN13,VAV3,VRK1,URM1,DPY SL3,CCDC88A,APBA1,PLCE1,TNIP3,TACC2,BA K1,CASTOR2,LAX1,CACTIN,STK26,ENOX2,DCP 1A,ZCRB1,PPP3CA,MKNK1,RIN2,DENND1A,MZT 1,TASP1,TRIP12,NOMO1,CNOT6,PXMP2,TPMT, DISC1,PROX1,MAP3K7CL,CCDC6,ASAP1,CREBB P,RPS10- NUDT3,NEFL,ATP6V1A,STK3,CHN2,HNF4G,EXO SC3,AP2B1,SH2D3C,LIMS1,DOCK4,ACKR2,ARP C1A,MCTP2,PLS3,TBC1D10A,EPHB1,RPH3A,CY TH4,VPS29,COPB2,UBR1,TCF7L1,ARHGAP32,D YRK3,KRTAP10- 12,CNTLN,HDAC4,FYB1,SEM1,GYS2,MYLK,ACS BG1,P3H2,CFL1,DTYMK,KRT20,SLAIN2,COPG2 ,PLEKHG4B,AFDN,CYTIP,WWC3,CLPX,REPS1,F BXL7,TPTE,LCP2,GLI2,MAP3K7,ATXN1,ADAM1 7,ELAVL1,S100PBP,TRAK1,STXBP1,IDH1,MUC 13,NUP205,PLEKHG1,ELP2,ORC4,APC,TTLL5, INO80,NAA11,MOCS2,SULT2B1,CDA,PPARGC1B ,PHLDB2,ERC1,AGO3,RPL39L,IQGAP2,ANKRA2 ,JPT1,RASSF3,PACSIN2,TIA1,DPYSL2,SYNPO*

			2,GIMAP8,ASCC3,RMDN2,AARS1,PTPN11,PSMC2,MAPKAP1,RIC1,ACOX1,USP24,HAO1,NUP153,HSD17B4,TRMT10B,COL21A1,RAI14,CAVIN1,KCTD5,CEP162,NR3C1,ZC3HAV1,ARHGAP28,MTOR,KSR1,CBLB,FAF1,SNAP25,SGSM1,ADH7,GOLGA8A,PIP5K1A,BTBD2,MMACHC,RAB6B,KYNU,ADAL,TBXAS1,ASCC2,SPRED1,WASHC1,PDS5B,PFKFB3,RNF115,TRIM61,ESD,PER2,PLEKHH2,GLI3,LHPP,KAT6A,TADA1,GAB2,PDE1A,PDCD10,TFG,EPHX2,LMTK2,FERMT2,TSPOAP1,EPB41,DENND5A,DOCK9,AGBL3,SCPEP1,EPB41L2,PACSIN1,CNTRL,GNPNAT1,MTMR7,EIF2A,FGD1,GSTA3,TRIM39,EIF4E3,ACP3,USP48,RRM1,INPP5D,ATF6,PRKAR1B,GBF1,IPO11,BACH1,CUL9,CYTH3,KLC1,AKAP13,HBS1L,PKM,ARFGEF1,ATP12A,TKFC,PTPN3,STAC,TTC17,ELP1,IRF8,ACTA2,EPS15L1,MZT2A,CAST,TAOK1,UNC45A,CASK,USH1C,FMN2,UBXN2A,PCNT,DNAJC13,GOLGA8B
GO:0016020	membrane	2.2189188447112186e-14	*SLC18A1,RIT2,SLMAP,ABCB7,MYO9B,GPSM2,COL4A5,CYTH1,STK16,XK,SEMA4D,MET,SCYL3,ATXN10,BRCA1,SNX16,CEP350,STAU2,CHMP1B,MMP16,DRD3,ELAPOR2,FAXC,ATP11C,EXOC2,PTPN5,TACR1,SNX25,SDC2,ABHD12,COX10,ARAP1,UQCRHL,HEPHL1,KLHL7,CCR3,ANKLE2,CIP2A,AHNAK,LRRC53,EIF4A3,NFE2L2,SCHIP1,OTOA,CYP19A1,SLC46A2,DTNB,GPR158,VRK2,ITSN1,OAS1,DEUP1,ATP2B2,PREX2,SCRN1,CSMD3,NTN4,SERP1,CNTNAP3B,PLEKHM3,TPTE2,RAB11FIP4,NPIPB5,GRPEL2,SNTA1,CLDN10,MISFA,CLIC5,ARHGAP26,TRHDE,RAB44,PTPN4,SLC16A11,ITGA2,GRIN2A,IL1RAPL1,WDPCP,BLK,GPM6A,CCZ1B,ADCY1,GRID1,EFR3B,PLPPR5,AVL9,ZDHHC17,FIG4,EIF2AK2,AP1S3,CFAP47,F2RL2,LPAR3,ANO4,FMR1,TXLNG,SH3TC2,CEP290,FAM171B,WDFY3,TACC1,OCLN,CFAP91,ITGBL1,NETO1,PTPRQ,SNX5,NEO1,GLT8D1,CLDN14,ITPKB,PLEKHB2,TIMM23,SLC66A1,F10,APOO,DIO2,SYNE1,PSMD10,FBXL17,PDZD2,SLC9A4,GAS2,GPAM,LRP12,TSPEAR,FILIP1L,LRRC1,RGS12,PPP6R3,OSBP2,CBX3,ACAP2,SLC39A10,KCNV1,KCTD8,CHCHD6,KIZ,MPHOSPH9,RNF10,FAT1,SLC2A2,PIK3R1,STAMBPL1,AGMO,ABCB11,PGM5,COL26A1,ATG12,FNDC3B,TMEM232,USP50,CD200R1,CALD1,SH3GL2,CLBA1,CRTC1,SLC30A3,MTX2,XKR4,ROR1,SCAP,AAK1,CRB1,CTTN,USP8,GPR75,PARD6B,OR2AJ1,PSD3,ABCA13,ACY3,FZR1,BAIAP2L1,ST8SIA5,DBNL,RYK,MACF1,TUBGCP3,WEE2,MTM1,SLC15A5,KCNE1,PTPRE,UNC5B,MFSD6,SERINC3,PTPRZ1,CD55,PLN,SHISA3,SLC66A3,SLC16A7,NEDD4,CACNA1E,LRRC38,DOCK8,CYSLTR2,PARVA,SLC9C1,NDUFAF6,HACE1,ATG4B,SCD5,SLC7A14,DSC2,SERPINA1,MYO10,CRHR2,MID1,SLC12A2,EFNB3,TRIM37,DLG3,FAR2,SSR3,TRAT1,FLOT2,PVRIG,SV2B,ILK,WDR17,SLC25A13,RAPGEF1,ALG13,EFCAB14,GPRC6A,UNC79,SH3GL3,SLC1A2,GABRB2,EDNRB,GRIN2B,SVOP,GPR141,IFNGR2,DENND4A,TBC1D20,PLD5,SNX2,SCAMP5,IL17RB,MINDY3,EIF3F,RDH16,FCHSD2,SLC4A7,DNAJB6,MTCL3,MIGA1,NPIPB3,OMA1,SP*

			CS1,MTUS1,GABRA6,LEMD3,PLEKHF2,B3GLCT,PIEZO1,SOAT1,OR2T11,SLC9B2,MRGPRX1,TMEM100,LYPD6,PDIA5,XPR1,PDPN,CREB3L2,RTN4,GNL3L,CACNA1D,ARHGEF11,CD163,SGPP2,BBS2,CLSTN1,TMEM150C,SCYL2,SKAP1,MAOB,MRPL3,MICOS10,NUBPL,PLAAT3,IL12RB2,PIK3CG,ANOS1,COL4A4,TPP1,TRGV5,IL1R2,ATP6V1E2,TNPO3,NBPF10,HTT,NECTIN3,FREM3,IQGAP1,MYCBP2,WIPI1,KLHL12,SNX30,TSNARE1,FANCI,OR9Q1,ACAA1,OR4K17,LRRC37A3,TMDD1,SPNS3,TNFRSF10B,MTCL1,PTPRO,AZIN2,IYD,HLA-DMA,PEMT,PPP2R2B,CHD5,JAK1,GPD2,ITGA9,ACSL6,TOR1AIP2,ULK2,KCNB1,CANX,GNPAT,HSD17B12,HS6ST3,GRM4,ANKRD27,ANK3,TMEM132B,PPP1R14C,KITLG,SV2C,CNIH3,PLEKHM2,NXPE1,HIP1,RAET1E,RELN,OR51B5,HS3ST4,MTMR3,CRKL,SYTL3,NDC1,PTPN13,SNAP91,ZNF816,VAV3,RPS6KC1,INPP5A,CD40LG,VRK1,GPAT3,SCN9A,ATP2B1,SPRING1,SLC16A10,ATRN,NDUFB9,CCDC88A,SLC35B4,UGT3A2,APBA1,HPSE2,PLCE1,TACC2,NAALAD2,CYP39A1,GNAL,PEBP4,MSH3,BAK1,LAX1,STK26,CLCC1,ENOX2,TENM4,DCP1A,PPP3CA,CHRNA5,DYSF,NKD1,CYRIB,C3,PDE6C,CDH10,DENND1A,OR52D1,MICU1,EBPL,C3ORF52,BACE2,NOMO1,ARL6IP6,CNOT6,GALNT18,ASAH2,TBC1D9B,STON1,PXMP2,OR5K1,OR13C9,FMN1,RALGPS1,MUC5B,CTSB,DNAJC11,ASAP1,VSIG1,AQP8,ITGB1,BVES,CYB561,LMAN2L,DSE,RHCE,CORO1C,NSG2,LHFPL6,C17ORF80,ATP6V1A,TMEM185B,CHN2,RNF150,FCAR,C1GALT1,SMCO4,MSR1,AP2B1,GABRG3,SH2D3C,TRPC4,RAMP3,GNG2,FRS3,XYLT2,LIMS1,ENPP2,DOCK4,ACKR2,ARPC1A,MCTP2,CERS3,GLCE,PLS3,PCSK2,TSPAN2,KCNIP1,GFRA2,TBC1D10A,EPHB1,REEP1,RPH3A,SLCO6A1,CYTH4,NAP1L1,SLC38A6,VPS29,PLPP4,COPB2,CNN2,COL4A2,SLC1A6,ARHGAP32,MRPS10,CHRNA6,TLN2,FYB1,MARCHF5,IQCE,LVRN,MYLK,UNC5C,ACSBG1,CSGALNACT1,CSMD2,TLR7,FLG2,SLC37A2,CFL1,TUSC3,COPG2,PLEKHG4B,MED13,TAAR2,AFDN,LRP2,MTDH,SEMA6D,EHMT1,ZDHHC13,IL17RA,SERPINA9,GALNT14,CLPX,VWC2,REPS1,TMEM108,TPTE,BFSP1,LCP2,SEZ6L,PCYOX1L,SLC39A8,TMEM123,GLI2,TAS2R13,TGFBR3,MAP3K7,GTF2I,TMEM59L,ADAM17,SEMA4F,ELAVL1,TRAK1,STXBP1,OR6N1,TIMM23B,ABCC1,MUC13,NUP205,SLCO5A1,SNRNP200,CYP8B1,APC,GNA12,TTLL5,IL18R1,ERGIC2,SPTSSB,PEAR1,PHLDB2,ENSG00000287694,ERC1,SMIM13,ROS1,RUBCNL,AGO3,C9,PVR,CKAP4,TMPRSS11A,ACER1,IQGAP2,CDYL,ANKRA2,CTNND2,FRMD3,RASSF3,PACSIN2,RDH13,DPYSL2,NUP210L,CNTNAP3C,RAB8B,ASCC3,GRM8,SLC23A2,RMDN2,AARS1,KCND1,TMEM45A,TMEM267,PSMC2,MAPKAP1,COL15A1,EVC2,RIC1,MGAT5,ACOX1,NUP153,CLPTM1,HSD17B4,EFHD1,RNF215,TRMT10B,PIGR,NRP2,CAVIN1,GYPE,PLXNC1,METTL15,TMEM165,ELOVL7,NR3C1,BORCS5,MTOR,TRAV5,KSR1,CBLB,FAF1,SNAP25,SGSM1,ADH7,CHST4,OR11H6,GOLGA8A,CLDND1,PIP5K1A

			,DISP1,RAB6B,HS6ST2,TBXAS1,SPRED1,ADAM10,GNG4,CNTNAP4,WASHC1,CDH26,CRIM1,NALF2,ABCB5,DPY19L2,CYYR1,CDHR3,PLEKHH2,ANKRD24,KCNK12,NTRK3,RXFP1,SLC35F3,GAB2,SNTB2,PDCD10,LMTK2,HHAT,ADGRG6,SLC4A10,GOLGA7,FERMT2,GPR82,DNM3,ST3GAL2,SYT1,EPB41,DENND5A,DOCK9,CD244,SHISA6,IGLV3-1,NFASC,SLC28A2,EPB41L2,CADM3,PACSIN1,STXBP6,RTTN,CACNA1A,EDA,CNTRL,TNFSF13B,GNPNAT1,MTMR7,PTH2R,ELOVL6,SLC22A25,CELSR1,HDAC2,TPO,TGOLN2,SEMA3D,PROM1,OR2T8,NPSR1,ACP3,KCNH5,RRM1,MAGEA1,CADM1,INPP5D,CLYBL,OR4C6,ATF6,PRKAR1B,GBF1,SERPINE1,EWSR1,TM7SF3,CYTH3,KLC1,SLC26A5,AKAP13,HBS1L,MLANA,GCG,ARFGEF1,ATP12A,PTPN3,STAC,SCN8A,NNT,ST8SIA2,TTC17,HEPACAM2,CCDC198,OR6K6,REM1,FREM1,RIPOR2,NDUFA10,KCNK5,CD69,B3GALNT2,FUNDC2,EPS15L1,CYB561A3,PARD3B,KDM3A,CAST,OR2L3,GPR39,ADGRA3,CASK,HSPG2,SLCO4C1,GCNT1,SLC35F4,GGA2,USH1C,FMN2,PCNT,DNAJC13,GOLGA8B,TRDV3
GO:0042995	cell projection	2.925875621265091e-13	*SLC18A1,RIT2,MYO9B,MAX,TNS3,SCYL3,ATXN10,IFTAP,CEP350,STAU2,CETN3,DNAH11,CDC42BPA,TACR1,ABHD12,EIF4A3,DLEC1,DTNB,GPR158,ITSN1,ATP2B2,ESPNL,FBF1,ENKUR,ITGA2,GRIN2A,IL1RAPL1,WDPCP,KIF5C,GPM6A,ZDHHC17,CFAP47,LPAR3,FMR1,CEP290,WDFY3,SH3PXD2B,CFAP91,AK8,NETO1,TNPO1,SNX5,NEO1,SYNE1,PSMD10,TSPEAR,RGS12,OSBP2,ACAP2,KCTD8,KIZ,FAT1,SHTN1,TMEM232,USP50,SH3GL2,SLC30A3,DOCK7,ROR1,AAK1,CRB1,CTTN,USP8,PSD3,DBNL,MACF1,MTM1,TBC1D31,PTPN12,NEDD4,NEK4,DOCK8,PARVA,URI1,SLC9C1,FGD4,MYO10,OLFM1,CRHR2,SLC12A2,DLG3,FLOT2,ILK,CERKL,SLC1A2,GABRB2,GRIN2B,UBXN10,SNX2,FCHSD2,SLC4A7,GABRA6,PIEZO1,SLC9B2,LYPD6,PDPN,RTN4,PRKAA2,BBS2,CLSTN1,SPAG17,MAOB,MYO3A,HTT,NECTIN3,IQGAP1,MYCBP2,MAP2,FBXW11,PTPRO,AZIN2,KCNB1,RP1L1,ANKRD27,ANK3,KITLG,CNIH3,RELN,FGD6,PTPN13,INPP5A,SCN9A,DPYSL3,ATP2B1,CCDC88A,APBA1,PLCE1,TENM4,PPP3CA,CHRNA5,CYRIB,DENND1A,STON1,DISC1,ASAP1,AQP8,ITGB1,BVES,DRC7,CORO1C,NSG2,NEFL,ATP6V1A,GABRG3,SH2D3C,DOCK4,ARPC1A,BCL11B,PCSK2,KCNIP1,TBC1D10A,EPHB1,RPH3A,CTTNBP2,TULP2,NME9,ARHGAP32,CHRNA6,TLN2,KLHL1,IQCE,MYLK,UNC5C,CFAP69,CFL1,COPG2,CCDC38,LRP2,TMEM108,CC2D2B,GLI2,ADAM17,SEMA4F,TRAK1,STXBP1,APC,GNA12,TTLL5,PEAR1,ERC1,IQGAP2,CTNND2,DNAH14,PACSIN2,DPYSL2,KCND1,EVC2,NRP2,CEP162,MTOR,KSR1,SNAP25,RP1,PIP5K1A,ADAM10,CNTNAP4,CDHR3,ERICH3,PLEKHH2,ANKRD24,GLI3,NTRK3,LMTK2,CCDC68,SLC4A10,FERMT2,TSPOAP1,DNM3,SYT1,SHISA6,NFASC,SLC28A2,PACSIN1,RTTN,CACNA1A,CNTRL,FGD1,PROM1,ACP3,RRM1,CADM1,CFAP161,PRKAR1B,CYTH3,KLC1,PKM,SCN8A,SSX2IP,ACTA2,RIPOR2,CASK,U*

			SH1C,FMN2,UBXN2A,FRMD4B
GO:0030054	cell junction	6.884991611659271e-12	SLC18A1,RIT2,COL4A5,PARN,CYTH1,TNS3,KIAA1210,DRD3,CDC42BPA,PTPN5,AHNAK,EIF4A3,SCHIP1,DTNB,GPR158,ITSN1,ATP2B2,SCRN1,FBF1,FBXL20,SNTA1,CLDN10,ARHGAP26,ITGA2,GRIN2A,IL1RAPL1,KIF5C,GPM6A,ADCY1,GRID1,RPS6KA2,ZDHHC17,LPAR3,FMR1,SH3PXD2B,OCLN,ITGBL1,NETO1,CLDN14,UBOX5,SYNE1,PDZD2,IRF2,RGS12,KCTD8,RNF10,FAT1,SLC2A2,PIK3R1,ABCB11,PGM5,USP50,SH3GL2,SLC30A3,DOCK7,ROR1,AAK1,CRB1,CTTN,USP8,PARD6B,THEMIS,PSD3,MEF2C,BAIAP2L1,DBNL,MACF1,PTPN12,PTPRZ1,SLC16A7,NEDD4,CACNA1E,PARVA,DSC2,OLFM1,CRHR2,EFNB3,DLG3,FLOT2,SRP68,SV2B,ILK,MAPK14,SH3GL3,SLC1A2,GABRB2,GRIN2B,SVOP,SCAMP5,SMPX,EIF3F,FCHSD2,GABRA6,SLC9B2,LYPD6,PDPN,RTN4,CLSTN1,SKAP1,SERPINE2,HTT,NECTIN3,IQGAP1,SPARCL1,PTPRO,JAK1,KCNB1,CANX,GRM4,ANK3,SV2C,MAP4K4,CNIH3,HIP1,CRKL,SNAP91,DPYSL3,ATP2B1,SLC16A10,APBA1,PPP3CA,CHRNA5,DYSF,ASCC1,CDH10,DENND1A,PDZRN3,STON1,ASH1L,DISC1,FMN1,ASAP1,ITGB1,BVES,CORO1C,NEFL,ATP6V1A,CHN2,AP2B1,GABRG3,TRPC4,LIMS1,MCTP2,EPHB1,RPH3A,CTTNBP2,SLC38A6,CNN2,SLC1A6,ARHGAP32,IP6K2,CHRNA6,TLN2,HDAC4,FYB1,UNC5C,CFL1,AFDN,MTDH,VWC2,TMEM108,LCP2,ADAM17,SEMA4F,ELAVL1,STXBP1,APC,GNA12,PHLDB2,ERC1,PVR,CTNND2,PACSIN2,SYNPO2,RAB8B,KCND1,RAI14,NRP2,PLXNC1,NR3C1,BORCS5,ARHGAP28,SNAP25,PIP5K1A,RAB6B,ADAM10,CNTNAP4,CDH26,CDHR3,TADA1,SNTB2,SLC4A10,FERMT2,TSPOAP1,DNM3,SYT1,EPB41,SHISA6,NFASC,EPB41L2,CADM3,PACSIN1,STXBP6,CACNA1A,TNFSF13B,INSYN2B,CADM1,PRKAR1B,CCDC88C,CYTH3,SCN8A,SSX2IP,PARD3B,XIRP2,CASK,HSPG2,USH1C,FRMD4B
GO:0120025	plasma membrane bounded cell projection	1.990133896640545e-11	SLC18A1,RIT2,MYO9B,MAX,SCYL3,ATXN10,IFTAP,STAU2,CETN3,DNAH11,CDC42BPA,TACR1,ABHD12,EIF4A3,DLEC1,DTNB,ITSN1,ATP2B2,ESPNL,FBF1,ENKUR,ITGA2,GRIN2A,IL1RAPL1,WDPCP,KIF5C,GPM6A,CFAP47,LPAR3,FMR1,CEP290,WDFY3,CFAP91,AK8,NETO1,TNPO1,SNX5,NEO1,SYNE1,PSMD10,TSPEAR,RGS12,OSBP2,ACAP2,FAT1,SHTN1,TMEM232,USP50,SLC30A3,DOCK7,ROR1,AAK1,CRB1,CTTN,USP8,PSD3,DBNL,MACF1,MTM1,TBC1D31,NEDD4,NEK4,DOCK8,PARVA,URI1,SLC9C1,FGD4,MYO10,OLFM1,CRHR2,SLC12A2,DLG3,FLOT2,ILK,CERKL,SLC1A2,GABRB2,GRIN2B,UBXN10,SNX2,FCHSD2,SLC4A7,GABRA6,PIEZO1,SLC9B2,LYPD6,PDPN,RTN4,PRKAA2,BBS2,CLSTN1,SPAG17,MAOB,MYO3A,HTT,NECTIN3,IQGAP1,MYCBP2,MAP2,FBXW11,PTPRO,AZIN2,KCNB1,RP1L1,ANKRD27,ANK3,KITLG,CNIH3,RELN,FGD6,PTPN13,INPP5A,SCN9A,DPYSL3,CCDC88A,APBA1,PLCE1,TENM4,PPP3CA,CHRNA5,CYRIB,DENND1A,DISC1,ASAP1,AQP8,ITGB1,BVES,DRC7,CORO1C,NSG2,NEFL,ATP6V1A,GABRG3,SH2D3C,DOCK4,ARPC1A,BCL11B,PCSK2,KCNIP1,TBC1D10A,EPHB1,RP

			H3A,CTTNBP2,TULP2,ARHGAP32,CHRNA6,TLN2,KLHL1,IQCE,MYLK,UNC5C,CFAP69,CFL1,COPG2,CCDC38,LRP2,TMEM108,CC2D2B,GLI2,ADAM17,SEMA4F,TRAK1,STXBP1,APC,GNA12,TTLL5,PEAR1,ERC1,IQGAP2,CTNND2,DNAH14,PACSIN2,DPYSL2,KCND1,EVC2,NRP2,CEP162,MTOR,KSR1,SNAP25,RP1,PIP5K1A,ADAM10,CDHR3,ERICH3,PLEKHH2,ANKRD24,GLI3,NTRK3,LMTK2,CCDC68,SLC4A10,FERMT2,TSPOAP1,DNM3,SYT1,SHISA6,NFASC,SLC28A2,PACSIN1,RTTN,CNTRL,FGD1,PROM1,ACP3,CADM1,CFAP161,PRKAR1B,CYTH3,KLC1,PKM,SCN8A,SSX2IP,ACTA2,RIPOR2,CASK,USH1C,FMN2,UBXN2A,FRMD4B
GO:0031252	cell leading edge	7.207967227490132e-10	MYO9B,SCYL3,CDC42BPA,ITSN1,ATP2B2,OCLN,SNX5,ACAP2,FAT1,SHTN1,AAK1,CTTN,PSD3,DBNL,MACF1,MTM1,DOCK8,PARVA,FGD4,MYO10,FLOT2,ILK,SLC1A2,SNX2,GABRA6,PIEZO1,PDPN,IQGAP1,PTPRO,KCNB1,KITLG,FGD6,PTPN13,DPYSL3,CCDC88A,PLCE1,STON1,ITGB1,CORO1C,GABRG3,SH2D3C,TLN2,MYLK,UNC5C,CFL1,ADAM17,APC,PEAR1,PHLDB2,IQGAP2,PACSIN2,KSR1,SNAP25,PIP5K1A,PLEKHH2,FERMT2,SHISA6,PACSIN1,FGD1,GBF1,CYTH3,SSX2IP,ACTA2,RIPOR2,FRMD4B
GO:0098590	plasma membrane region	3.946115895382666e-9	MET,OTOA,DTNB,GPR158,ITSN1,ATP2B2,RAB11FIP4,SNTA1,CLIC5,GRIN2A,IL1RAPL1,WDPCP,GPM6A,ADCY1,GRID1,ZDHHC17,F2RL2,FMR1,OCLN,NETO1,SYNE1,SLC9A4,TSPEAR,SLC39A10,KCTD8,RNF10,FAT1,SLC2A2,ABCB11,CRB1,PARD6B,PSD3,ACY3,MACF1,KCNE1,SLC16A7,NEDD4,DOCK8,MYO10,SLC12A2,EFNB3,DLG3,FLOT2,SLC1A2,GABRB2,GRIN2B,SVOP,SLC4A7,GABRA6,PIEZO1,SLC9B2,PDPN,BBS2,CLSTN1,SKAP1,NECTIN3,IQGAP1,MTCL1,PTPRO,ITGA9,KCNB1,ANK3,CNIH3,HIP1,CRKL,SNAP91,ATP2B1,SLC16A10,APBA1,STK26,CHRNA5,CDH10,DENND1A,ASAH2,CTSB,VSIG1,AQP8,ITGB1,BVES,CORO1C,ATP6V1A,GABRG3,SH2D3C,TRPC4,ARPC1A,RPH3A,SLC1A6,CHRNA6,IQCE,MYLK,CFL1,LRP2,MTDH,LCP2,SLC39A8,ADAM17,SEMA4F,STXBP1,ABCC1,MUC13,APC,GNA12,ERC1,PACSIN2,SLC23A2,KCND1,EVC2,NRP2,CAVIN1,KSR1,SNAP25,CLDND1,PIP5K1A,DISP1,SPRED1,ADAM10,CNTNAP4,SLC4A10,FERMT2,DNM3,SYT1,EPB41,SHISA6,SLC28A2,PACSIN1,SLC22A25,PROM1,CADM1,SLC26A5,ATP12A,SCN8A,RIPOR2,PARD3B,CASK,SLCO4C1
GO:0005856	cytoskeleton	4.295063478615406e-9	SLMAP,MYO9B,GPSM2,TNS3,BRCA1,CEP350,STAU2,CETN3,CHMP1B,DNAH11,CDC42BPA,CEP85,AHNAK,NFE2L2,UPP2,DEUP1,FBF1,SERP1,ENKUR,RAB11FIP4,SNTA1,CLIC5,ARHGAP26,PTPN4,WDPCP,KIF5C,KRT86,RPS6KA2,EFR3B,CFAP47,CEP290,TACC1,SH3PXD2B,CFAP91,AK8,STAG1,ITPKB,FAM184A,SYNE1,PSMD10,PDZD2,GAS2,CBX3,KIZ,MPHOSPH9,PGM5,SHTN1,TMEM232,USP50,CALD1,MYLK3,ROR1,CTTN,BAIAP2L1,DBNL,MACF1,TUBGCP3,TFDP2,TBC1D31,PTPN12,ACACA,EML1,NEK4,PARVA,FGD4,MYO10,MID1,TRAT1,FLOT2,ILK,MAPK14,GRIN2B,MYL1,KLHL4,MTUS1,NEB,IVNS1ABP,BBS2,SPAG17,MYO3A,ANKFN1,STIL,HTT,IQGAP1,MYCBP2,W

			IPI1,LATS1,KLHL12,MAP2,FBXW11,MTCL1,HSPH1,ARHGAP6,PPP2R2B,JAK1,RADIL,RP1L1,ANK3,KITLG,NR1I2,HIP1,MYO18B,ARHGEF10,FGD6,NDC1,PTPN13,DPYSL3,CCDC88A,TACC2,DYSF,MZT1,DISC1,FMN1,CCDC6,ASAP1,DRC7,CORO1C,KANK3,NEFL,STK3,HNF4G,GABRG3,TRPC4,ACKR2,ARPC1A,PLS3,REEP1,CTTNBP2,NME9,CYTH4,CCDC102B,CNN2,SLC1A6,ARHGAP32,DYRK3,KRTAP10-12,CNTLN,TLN2,HDAC4,KLHL1,FYB1,TSKS,GYS2,MYLK,CFL1,KRT20,SLAIN2,CEP85L,CCDC38,FBXL7,BFSP1,GLI2,AFG2B,ADAM17,LMOD2,APC,TTLL5,INO80,NAA11,PHLDB2,ERC1,CKAP4,IQGAP2,ANKRA2,JPT1,DNAH14,FRMD3,RASSF3,PACSIN2,DPYSL2,SYNPO2,RMDN2,EVC2,RAI14,CEP162,NR3C1,BORCS5,SNAP25,RP1,WASHC1,TUBA3D,CDH26,PLEKHH2,GLI3,SNTB2,CCDC68,FERMT2,DNM3,EPB41,SS18,NCKAP5,MAP2K5,EPB41L2,TBCA,RTTN,EDA,CNTRL,LIMCH1,MICAL2,FIRRM,FGD1,INPP5D,CFAP161,CCDC88C,KLC1,AKAP13,ATP12A,PTPN3,SSX2IP,TTC17,HEPACAM2,ACTA2,RIPOR2,XIRP2,MZT2A,TAOK1,CASK,USH1C,FMN2,PCNT,FRMD4B,AFG2A
GO:0012505	endomembrane system	1.0585809640398342e-8	*SLC18A1,RIT2,SLMAP,NHLRC3,COL4A5,CYTH1,STK16,XK,SCYL3,KIAA1210,SNX16,STAU2,CETN3,CHMP1B,MMP16,ATP11C,PTPN5,SNX25,SDC2,ABHD12,ARAP1,ANKLE2,CEP85,NFE2L2,BLOC1S5-TXNDC5,CYP19A1,SLC46A2,VRK2,ITSN1,OAS1,SCRN1,SERP1,PLEKHM3,ENKUR,TPTE2,RAB11FIP4,CSNK2A2,CLIC5,ARHGAP26,RAB44,GRIN2A,SPATA16,CCZ1B,AVL9,ZDHHC17,FIG4,AP1S3,TXLNG,CEP290,WDFY3,SNX5,CDK13,SNX32,NEO1,GLT8D1,CLDN14,ITPKB,PLEKHB2,CAPN11,F10,APOO,SYNE1,PDZD2,SLC9A4,LRP12,OSBP2,CBX3,ACAP2,MPHOSPH9,PIK3R1,STAMBPL1,AGMO,ABCB11,COL26A1,SH3GL2,CLBA1,SLC30A3,SCAP,ADAMTSL1,AAK1,CTTN,UTP15,USP8,SMAD6,ABCA13,FZR1,ST8SIA5,DBNL,MACF1,MTM1,SERINC3,CD55,PLN,SHISA3,NEDD4,FGD4,PDXK,PACS2,HACE1,ATG4B,SCD5,DSC2,SERPINA1,OLFM1,MID1,SSR3,FLOT2,SRP68,SV2B,MAPK14,RAPGEF1,ALG13,SH3GL3,AKR7A2,CERKL,EDNRB,GRIN2B,UBXN10,SVOP,ARSB,IFNGR2,DENND4A,TBC1D20,SNX2,SCAMP5,MINDY3,RDH16,FCHSD2,NPIPB3,SPCS1,MTUS1,LEMD3,PLEKHF2,B3GLCT,PIEZO1,SOAT1,SLC9B2,TMEM100,PDIA5,XPR1,SPESP1,CREB3L2,RTN4,PRKAA2,SGPP2,CLSTN1,SPAG17,SCYL2,NT5C3A,SERPINE2,PLAAT3,POGLUT2,COL4A4,TPP1,ATP6V1E2,TNPO3,HTT,NUP160,IQGAP1,WIPI1,KLHL12,SNX30,TSNARE1,ACAA1,SPARCL1,PRSS23,FBXW11,AZIN2,HLA-DMA,PEMT,JAK1,ACSL6,TOR1AIP2,CANX,HSD17B12,HS6ST3,ANKRD27,ANK3,SV2C,CNIH3,PLEKHM2,HIP1,HS3ST4,FGD6,SYTL3,NDC1,SNAP91,RPS6KC1,CD40LG,VRK1,GPAT3,DPYSL3,ATP2B1,SPRING1,ATRN,CCDC88A,SLC35B4,UGT3A2,APBA1,PLCE1,CYP39A1,BAK1,LAX1,STK26,CLCC1,CES2,DYSF,CYRIB,C3,EBPL,C3ORF52*

			,BACE2,NOMO1,ARL6IP6,GALNT18,ASAH2,STON1,ASH1L,DISC1,FMN1,MUC5B,CTSB,ASAP1,AQP8,ITGB1,CYB561,LMAN2L,DSE,CORO1C,NSG2,ATP6V1A,FCAR,C1GALT1,AP2B1,XYLT2,DOCK4,ACKR2,MCTP2,CERS3,GLCE,PCSK2,EPHB1,REEP1,RPH3A,CTTNBP2,CYTH4,VPS29,COPB2,CNN2,COL4A2,SLC1A6,ARHGAP32,GDNF,MARCHF5,ACSBG1,CSGALNACT1,TLR7,FLG2,SLC37A2,P3H2,TUSC3,COPG2,CYTIP,LRP2,MTDH,SEMA6D,ZDHHC13,GALNT14,REPS1,TMEM108,SEZ6L,PCYOX1L,MAP3K7,TMEM59L,ADAM17,SEMA4F,ELAVL1,TRAK1,STXBP1,IDH1,MUC13,NUP205,CYP8B1,APC,NAA11,ERGIC2,SULT2B1,SPTSSB,CDA,COL27A1,ERC1,CKAP4,ACER1,IQGAP2,UGGT1,PACSIN2,NUP210L,GIMAP8,RAB8B,RMDN2,PSMC2,MAPKAP1,COL15A1,RIC1,MGAT5,NUP153,CLPTM1,COL21A1,PIGR,CAVIN1,TMEM165,ELOVL7,BORCS5,FSTL4,MTOR,KSR1,FAF1,SNAP25,SGSM1,CHST4,GOLGA8A,GH2,RAB6B,HS6ST2,TBXAS1,ADAM10,WASHC1,DPY19L2,ESD,SNTB2,PDCD10,TFG,LMTK2,HHAT,ADGRG6,GOLGA7,GLUD1,ST3GAL2,SYT1,DENND5A,DOCK9,NFASC,FGF7,PACSIN1,EDA,GNPNAT1,MTMR7,ELOVL6,COL14A1,TGOLN2,FGD1,PROM1,ACP3,RRM1,ATF6,PRKAR1B,GBF1,IPO11,SERPINE1,CYTH3,MLANA,PKM,GCG,ARFGEF1,ST8SIA2,TTC17,HEPACAM2,B3GALNT2,EPS15L1,CYB561A3,PARD3B,CAST,UNC45A,HSPG2,SLCO4C1,GCNT1,GGA2,FMN2,UBXN2A,DNAJC13,GOLGA8B
GO:0071944	cell periphery	1.8294054299349393e-8	*RIT2,SLMAP,MYO9B,GPSM2,COL4A5,CYTH1,STK16,XK,SEMA4D,MET,ATXN10,BRCA1,STAU2,CHMP1B,MMP16,DRD3,ELAPOR2,ATP11C,EXOC2,PTPN5,TACR1,SDC2,ABHD12,ARAP1,HEPHL1,KLHL7,CCR3,CEP85,CIP2A,AHNAK,LRRC53,NFE2L2,SCHIP1,OLFML2A,OTOA,SLC46A2,DTNB,GPR158,ITSN1,ATP2B2,PREX2,CSMD3,NTN4,PLEKHM3,TPTE2,RAB11FIP4,SNTA1,CLDN10,CLIC5,TRHDE,RAB44,PTPN4,ITGA2,GRIN2A,IL1RAPL1,WDPCP,BLK,GPM6A,ADCY1,GRID1,EFR3B,PLPPR5,ZDHHC17,F2RL2,LPAR3,ANO4,FMR1,SH3TC2,WDFY3,OCLN,ITGBL1,NETO1,SNX5,NEO1,CLDN14,F10,DIO2,SYNE1,PSMD10,PDZD2,SLC9A4,GPAM,LRP12,TSPEAR,RGS12,PPP6R3,OSBP2,SLC39A10,KCNV1,KCTD8,RNF10,FAT1,SLC2A2,PIK3R1,ABCB11,PGM5,COL26A1,USP50,CD200R1,CALD1,SH3GL2,CRTC1,SLC30A3,XKR4,ROR1,AAK1,CRB1,CTTN,USP8,GPR75,PARD6B,OR2AJ1,PSD3,ABCA13,ACY3,BAIAP2L1,DBNL,RYK,MACF1,WEE2,MTM1,KCNE1,PTPRE,UNC5B,MFSD6,SERINC3,PTPRZ1,CD55,SLC16A7,NEDD4,CACNA1E,LRRC38,DOCK8,CYSLTR2,PARVA,SLC9C1,SLC7A14,DSC2,SERPINA1,MYO10,CRHR2,SLC12A2,EFNB3,DLG3,TRAT1,FLOT2,PVRIG,SV2B,ILK,SLC25A13,RAPGEF1,GPRC6A,UNC79,SLC1A2,GABRB2,EDNRB,GRIN2B,SVOP,GPR141,IFNGR2,SCAMP5,IL17RB,FCHSD2,SLC4A7,MIGA1,MTUS1,GABRA6,PIEZO1,OR2T11,SLC9B2,MRGPRX1,TMEM100,LYPD6,XPR1,PDPN,RTN4,CACNA1D,ARHGEF11,CD163,DPT,BBS2,CLSTN1,TMEM150C,SKAP1,SERPINE2,NUBPL,PLAAT3,IL12RB2,PIK3CG,ANOS1,COL4A4,STIL,*

			TRGV5,IL1R2,NECTIN3,FREM3,IQGAP1,MYCBP2,OR9Q1,OR4K17,SPARCL1,WNT2B,TNFRSF10B,MTCL1,PTPRO,IYD,HLA-DMA,JAK1,ITGA9,ACSL6,KCNB1,HSD17B12,GRM4,ANKRD27,ANK3,KITLG,SV2C,CNIH3,THSD4,HIP1,RAET1E,RELN,OR51B5,ADAMTSL3,CRKL,SYTL3,NDC1,PTPN13,SNAP91,VAV3,INPP5A,CD40LG,SCN9A,ATP2B1,SLC16A10,ATRN,CCDC88A,APBA1,HPSE2,PLCE1,TACC2,NAALAD2,GNAL,LAX1,STK26,ENOX2,TENM4,PPP3CA,CHRNA5,DYSF,NKD1,C3,PDE6C,CDH10,DENND1A,OR52D1,ADAMTS16,C3ORF52,BACE2,ASAH2,FRYL,STON1,OR5K1,OR13C9,FMN1,RALGPS1,MUC5B,CTSB,VSIG1,AQP8,ITGB1,BVES,RHCE,CORO1C,NAV2,ATP6V1A,FCAR,MSR1,AP2B1,GABRG3,SH2D3C,TRPC4,RAMP3,GNG2,FRS3,LIMS1,ENPP2,DOCK4,ACKR2,ARPC1A,PLS3,TSPAN2,KCNIP1,GFRA2,TBC1D10A,EPHB1,RPH3A,CTTNBP2,SLCO6A1,CYTH4,SLC38A6,PLPP4,COL4A2,SLC1A6,ARHGAP32,CHRNA6,TLN2,FYB1,GYS2,IQCE,MYLK,UNC5C,ACSBG1,CSMD2,TLR7,FLG2,P3H2,CFL1,TUSC3,PLEKHG4B,TAAR2,AFDN,CYTIP,LRP2,MTDH,SEMA6D,IL17RA,VWC2,REPS1,BFSP1,LCP2,SLC39A8,TMEM123,TAS2R13,TGFBR3,MAP3K7,ADAM17,SEMA4F,TRAK1,STXBP1,OR6N1,ABCC1,MUC13,SLCO5A1,APC,GNA12,TTLL5,IL18R1,PEAR1,COL27A1,PHLDB2,ERC1,ROS1,C9,PVR,CKAP4,TMPRSS11A,IQGAP2,CTNND2,RASSF3,PACSIN2,DPYSL2,RAB8B,GRM8,SLC23A2,KCND1,MAPKAP1,COL15A1,EXOC6B,EVC2,CLPTM1,COL21A1,RAI14,PIGR,NRP2,CAVIN1,GYPE,PLXNC1,BORCS5,TRAV5,KSR1,CBLB,FAF1,SNAP25,ADH7,OR11H6,CLDND1,PIP5K1A,DISP1,SPRED1,ADAM10,GNG4,CNTNAP4,WASHC1,CDH26,CRIM1,NALF2,ABCB5,CDHR3,PLEKHH2,ANKRD24,NTRK3,RXFP1,GAB2,SNTB2,PDCD10,ADGRG6,SLC4A10,FERMT2,GPR82,DNM3,SYT1,EPB41,CD244,SHISA6,IGLV3-1,NFASC,SLC28A2,EPB41L2,CADM3,PACSIN1,CACNA1A,EDA,TNFSF13B,PTH2R,COL14A1,SLC22A25,CELSR1,TPO,TGOLN2,PROM1,OR2T8,NPSR1,ACP3,KCNH5,MAGEA1,CADM1,INPP5D,OR4C6,PRKAR1B,SERPINE1,EWSR1,TM7SF3,CYTH3,SLC26A5,AKAP13,MLANA,PKM,GCG,ATP12A,PTPN3,STAC,SCN8A,TTC17,CCDC198,OR6K6,REM1,ACTA2,FREM1,RIPOR2,KCNK5,CD69,EPS15L1,PARD3B,OR2L3,GPR39,ADGRA3,CASK,HSPG2,SLCO4C1,USH1C,FMN2,DNAJC13,TRDV3
GO:0030027	lamellipodium	6.474293211273366e-8	MYO9B,SCYL3,CDC42BPA,ITSN1,FAT1,SHTN1,CTTN,DBNL,DOCK8,PARVA,FGD4,MYO10,FLOT2,ILK,SNX2,PIEZO1,PDPN,PTPRO,KITLG,FGD6,PTPN13,DPYSL3,CCDC88A,PLCE1,ITGB1,CORO1C,MYLK,UNC5C,CFL1,APC,PEAR1,IQGAP2,SNAP25,PIP5K1A,PLEKHH2,FERMT2,FGD1,ACTA2
GO:0043005	neuron projection	1.319266729897565e-7	SLC18A1,RIT2,MAX,ATXN10,STAU2,CETN3,TACR1,ABHD12,EIF4A3,DTNB,ITSN1,ATP2B2,ESPNL,ITGA2,GRIN2A,IL1RAPL1,KIF5C,GPM6A,LPAR3,FMR1,CEP290,WDFY3,NETO1,NEO1,SYNE1,PSMD10,TSPEAR,RGS12,OSBP2,SHTN1,USP50,SLC30A3,DOCK7,ROR1,AAK1,CRB1,CTTN,U

			SP8,DBNL,NEDD4,URI1,MYO10,OLFM1,CRHR2,SLC12A2,DLG3,FLOT2,CERKL,SLC1A2,GABRB2,GRIN2B,FCHSD2,SLC4A7,GABRA6,LYPD6,RTN4,PRKAA2,BBS2,CLSTN1,MAOB,MYO3A,HTT,NECTIN3,IQGAP1,MYCBP2,MAP2,FBXW11,PTPRO,AZIN2,KCNB1,RP1L1,ANKRD27,ANK3,CNIH3,RELN,INPP5A,SCN9A,DPYSL3,APBA1,TENM4,PPP3CA,CHRNA5,DENND1A,DISC1,ASAP1,ITGB1,NSG2,NEFL,GABRG3,SH2D3C,DOCK4,BCL11B,PCSK2,KCNIP1,EPHB1,RPH3A,CTTNBP2,ARHGAP32,CHRNA6,KLHL1,UNC5C,CFL1,COPG2,LRP2,TMEM108,SEMA4F,TRAK1,STXBP1,GNA12,CTNND2,DPYSL2,KCND1,NRP2,MTOR,SNAP25,RP1,ADAM10,CDHR3,ANKRD24,NTRK3,LMTK2,SLC4A10,TSPOAP1,DNM3,SYT1,SHISA6,NFASC,PACSIN1,PROM1,CADM1,KLC1,SCN8A,RIPOR2,USH1C,UBXN2A
GO:0005886	plasma membrane	1.3926545145625027e-7	*RIT2,SLMAP,GPSM2,CYTH1,STK16,XK,SEMA4D,MET,ATXN10,BRCA1,STAU2,CHMP1B,MMP16,DRD3,ELAPOR2,ATP11C,EXOC2,PTPN5,TACR1,SDC2,ABHD12,ARAP1,HEPHL1,KLHL7,CCR3,CIP2A,AHNAK,LRRC53,NFE2L2,SCHIP1,OTOA,SLC46A2,DTNB,GPR158,ITSN1,ATP2B2,PREX2,CSMD3,NTN4,PLEKHM3,TPTE2,RAB11FIP4,SNTA1,CLDN10,CLIC5,TRHDE,RAB44,PTPN4,ITGA2,GRIN2A,IL1RAPL1,WDPCP,BLK,GPM6A,ADCY1,GRID1,EFR3B,PLPPR5,ZDHHC17,F2RL2,LPAR3,ANO4,FMR1,SH3TC2,WDFY3,OCLN,ITGBL1,NETO1,SNX5,NEO1,CLDN14,F10,DIO2,SYNE1,PSMD10,PDZD2,SLC9A4,GPAM,LRP12,TSPEAR,RGS12,PPP6R3,OSBP2,SLC39A10,KCNV1,KCTD8,RNF10,FAT1,SLC2A2,PIK3R1,ABCB11,PGM5,COL26A1,USP50,CD200R1,CALD1,SH3GL2,CRTC1,SLC30A3,XKR4,ROR1,AAK1,CRB1,CTTN,USP8,GPR75,PARD6B,OR2AJ1,PSD3,ABCA13,ACY3,BAIAP2L1,DBNL,RYK,MACF1,WEE2,MTM1,KCNE1,PTPRE,UNC5B,MFSD6,SERINC3,PTPRZ1,CD55,SLC16A7,NEDD4,CACNA1E,LRRC38,DOCK8,CYSLTR2,PARVA,SLC9C1,SLC7A14,DSC2,MYO10,CRHR2,SLC12A2,EFNB3,DLG3,TRAT1,FLOT2,PVRIG,SV2B,ILK,SLC25A13,RAPGEF1,GPRC6A,UNC79,SLC1A2,GABRB2,EDNRB,GRIN2B,SVOP,GPR141,IFNGR2,SCAMP5,IL17RB,FCHSD2,SLC4A7,MIGA1,MTUS1,GABRA6,PIEZO1,OR2T11,SLC9B2,MRGPRX1,TMEM100,LYPD6,XPR1,PDPN,RTN4,CACNA1D,ARHGEF11,CD163,BBS2,CLSTN1,TMEM150C,SKAP1,NUBPL,PLAAT3,IL12RB2,PIK3CG,ANOS1,TRGV5,IL1R2,NECTIN3,IQGAP1,MYCBP2,OR9Q1,OR4K17,TNFRSF10B,MTCL1,PTPRO,IYD,HLA-DMA,JAK1,ITGA9,ACSL6,KCNB1,GRM4,ANKRD27,ANK3,KITLG,SV2C,CNIH3,HIP1,RAET1E,RELN,OR51B5,CRKL,SYTL3,NDC1,PTPN13,SNAP91,VAV3,INPP5A,CD40LG,SCN9A,ATP2B1,SLC16A10,ATRN,CCDC88A,APBA1,HPSE2,PLCE1,TACC2,NAALAD2,GNAL,LAX1,STK26,ENOX2,TENM4,PPP3CA,CHRNA5,DYSF,NKD1,C3,PDE6C,CDH10,DENND1A,OR52D1,C3ORF52,BACE2,ASAH2,STON1,OR5K1,OR13C9,FMN1,RALGPS1,MUC5B,CTSB,VSIG1,AQP8,ITGB1,BVES,RHCE,CORO1C,ATP6V1A,FCAR,MSR1,AP2B1,GABRG3,SH2D3C*

			,TRPC4,RAMP3,GNG2,FRS3,LIMS1,ENPP2,DOCK4,ACKR2,ARPC1A,PLS3,TSPAN2,KCNIP1,GFRA2,TBC1D10A,EPHB1,RPH3A,SLCO6A1,CYTH4,SLC38A6,PLPP4,SLC1A6,CHRNA6,TLN2,FYB1,IQCE,MYLK,UNC5C,ACSBG1,CSMD2,TLR7,FLG2,CFL1,TUSC3,PLEKHG4B,TAAR2,AFDN,LRP2,MTDH,SEMA6D,IL17RA,VWC2,REPS1,BFSP1,LCP2,SLC39A8,TMEM123,TAS2R13,TGFBR3,MAP3K7,ADAM17,SEMA4F,STXBP1,OR6N1,ABCC1,MUC13,SLCO5A1,APC,GNA12,TTLL5,IL18R1,PEAR1,PHLDB2,ERC1,ROS1,C9,PVR,CKAP4,TMPRSS11A,IQGAP2,CTNND2,RASSF3,PACSIN2,DPYSL2,RAB8B,GRM8,SLC23A2,KCND1,MAPKAP1,EVC2,CLPTM1,PIGR,NRP2,CAVIN1,GYPE,PLXNC1,BORCS5,TRAV5,KSR1,CBLB,FAF1,SNAP25,ADH7,OR11H6,CLDND1,PIP5K1A,DISP1,SPRED1,ADAM10,GNG4,CNTNAP4,CDH26,CRIM1,NALF2,ABCB5,CDHR3,PLEKHH2,ANKRD24,NTRK3,RXFP1,GAB2,SNTB2,PDCD10,ADGRG6,SLC4A10,FERMT2,GPR82,DNM3,SYT1,EPB41,CD244,SHISA6,IGLV3-1,NFASC,SLC28A2,EPB41L2,CADM3,PACSIN1,CACNA1A,EDA,TNFSF13B,PTH2R,SLC22A25,CELSR1,TPO,TGOLN2,PROM1,OR2T8,NPSR1,ACP3,KCNH5,MAGEA1,CADM1,INPP5D,OR4C6,PRKAR1B,SERPINE1,EWSR1,TM7SF3,CYTH3,SLC26A5,MLANA,GCG,ATP12A,PTPN3,STAC,SCN8A,TTC17,CCDC198,OR6K6,REM1,RIPOR2,KCNK5,CD69,EPS15L1,PARD3B,OR2L3,GPR39,ADGRA3,CASK,HSPG2,SLCO4C1,USH1C,FMN2,DNAJC13,TRDV3
GO:0045202	synapse	0.000001884847322780044	*SLC18A1,RIT2,COL4A5,PARN,DRD3,EIF4A3,DTNB,GPR158,ITSN1,ATP2B2,SCRN1,FBXL20,SNTA1,ITGA2,GRIN2A,IL1RAPL1,KIF5C,GPM6A,ADCY1,GRID1,RPS6KA2,ZDHHC17,LPAR3,FMR1,NETO1,SYNE1,RGS12,KCTD8,RNF10,USP50,SH3GL2,SLC30A3,ROR1,AAK1,CTTN,USP8,PSD3,MEF2C,DBNL,PTPRZ1,SLC16A7,NEDD4,CACNA1E,OLFM1,CRHR2,EFNB3,DLG3,FLOT2,SV2B,MAPK14,SH3GL3,SLC1A2,GABRB2,GRIN2B,SVOP,SCAMP5,EIF3F,FCHSD2,GABRA6,SLC9B2,LYPD6,RTN4,CLSTN1,SERPINE2,HTT,NECTIN3,SPARCL1,PTPRO,KCNB1,CANX,GRM4,ANK3,SV2C,CNIH3,HIP1,CRKL,SNAP91,DPYSL3,ATP2B1,APBA1,PPP3CA,CHRNA5,DYSF,ASCC1,CDH10,DENND1A,PDZRN3,STON1,DISC1,ASAP1,ITGB1,CORO1C,NEFL,ATP6V1A,CHN2,AP2B1,GABRG3,MCTP2,EPHB1,RPH3A,CTTNBP2,SLC38A6,SLC1A6,ARHGAP32,CHRNA6,TLN2,HDAC4,UNC5C,VWC2,TMEM108,SEMA4F,ELAVL1,STXBP1,ERC1,CTNND2,PACSIN2,RAB8B,KCND1,NRP2,PLXNC1,NR3C1,BORCS5,SNAP25,RAB6B,ADAM10,CNTNAP4,CDHR3,SNTB2,SLC4A10,TSPOAP1,DNM3,SYT1,SHISA6,PACSIN1,CACNA1A,INSYN2B,CADM1,PRKAR1B,SCN8A,CASK,USH1C*
GO:0097060	synaptic membrane	0.000003599565444718349	*GPR158,ITSN1,ATP2B2,SNTA1,GRIN2A,IL1RAPL1,GPM6A,ADCY1,GRID1,ZDHHC17,FMR1,NETO1,SYNE1,KCTD8,RNF10,SLC16A7,EFNB3,DLG3,SLC1A2,GABRB2,GRIN2B,GABRA6,CLSTN1,NECTIN3,PTPRO,KCNB1,ANK3,CNIH3,HIP1,CRKL,SNAP91,ATP2B1,APBA1,CHRNA5,CDH10,DEN*

			ND1A,ITGB1,GABRG3,RPH3A,SLC1A6,CHRNA6,SEMA4F,STXBP1,ERC1,KCND1,NRP2,SNAP25,ADAM10,CNTNAP4,DNM3,SYT1,SHISA6,SCN8A,CASK
GO:0015629	actin cytoskeleton	0.000010997207542772295	*MYO9B,TNS3,CDC42BPA,AHNAK,CLIC5,EFR3B,SH3PXD2B,GAS2,PGM5,CALD1,MYLK3,ROR1,CTTN,BAIAP2L1,DBNL,MACF1,PTPN12,ACACA,PARVA,MYO10,FLOT2,ILK,MYL1,NEB,MYO3A,IQGAP1,ARHGAP6,MYO18B,NDC1,DPYSL3,FMN1,ASAP1,CORO1C,ACKR2,ARPC1A,PLS3,CTTNBP2,CNN2,ARHGAP32,TLN2,HDAC4,FYB1,GYS2,MYLK,CFL1,ADAM17,LMOD2,IQGAP2,SYNPO2,RAI14,SNAP25,PLEKHH2,FERMT2,EPB41L2,LIMCH1,MICAL2,AKAP13,ATP12A,TTC17,ACTA2,XIRP2,CASK,FMN2*
GO:0005794	Golgi apparatus	0.000018311737230966848	*RIT2,CYTH1,STK16,SCYL3,MMP16,SDC2,ARAP1,CEP85,NFE2L2,PLEKHM3,TPTE2,CLIC5,SPATA16,ZDHHC17,FIG4,AP1S3,CDK13,NEO1,GLT8D1,F10,APOO,SYNE1,MPHOSPH9,PIK3R1,ABCB11,COL26A1,SH3GL2,CLBA1,SCAP,CTTN,SMAD6,ST8SIA5,DBNL,MACF1,SERINC3,CD55,NEDD4,FGD4,HACE1,SERPINA1,MID1,AKR7A2,CERKL,IFNGR2,TBC1D20,SCAMP5,MTUS1,XPR1,PRKAA2,CLSTN1,SPAG17,SCYL2,TPP1,HTT,WIPI1,AZIN2,HS6ST3,ANK3,HIP1,HS3ST4,FGD6,CD40LG,VRK1,SPRING1,ATRN,CCDC88A,SLC35B4,APBA1,PLCE1,LAX1,STK26,CLCC1,BACE2,GALNT18,ASAH2,ASH1L,MUC5B,ASAP1,LMAN2L,DSE,NSG2,ATP6V1A,C1GALT1,XYLT2,DOCK4,GLCE,RPH3A,CYTH4,COPB2,SLC1A6,ARHGAP32,GDNF,CSGALNACT1,TLR7,P3H2,COPG2,LRP2,SEMA6D,ZDHHC13,GALNT14,TMEM59L,ADAM17,MUC13,APC,NAA11,ERGIC2,ERC1,GIMAP8,RMDN2,MAPKAP1,RIC1,MGAT5,TMEM165,MTOR,SNAP25,SGSM1,CHST4,GOLGA8A,RAB6B,HS6ST2,ADAM10,SNTB2,PDCD10,LMTK2,HHAT,GOLGA7,ST3GAL2,SYT1,DENND5A,FGF7,GNPNAT1,TGOLN2,FGD1,ACP3,ATF6,GBF1,CYTH3,MLANA,ARFGEF1,ST8SIA2,TTC17,HEPACAM2,B3GALNT2,UNC45A,HSPG2,GCNT1,GGA2,UBXN2A,GOLGA8B*
GO:0070161	anchoring junction	0.00006862551853317899	*CYTH1,TNS3,KIAA1210,CDC42BPA,AHNAK,FBF1,SNTA1,CLDN10,ARHGAP26,ITGA2,SH3PXD2B,OCLN,ITGBL1,CLDN14,UBOX5,PDZD2,IRF2,FAT1,SLC2A2,PIK3R1,ABCB11,PGM5,DOCK7,CRB1,CTTN,PARD6B,THEMIS,BAIAP2L1,DBNL,PTPN12,PARVA,DSC2,DLG3,FLOT2,SRP68,ILK,SMPX,FCHSD2,PDPN,RTN4,SKAP1,NECTIN3,IQGAP1,JAK1,ANK3,MAP4K4,PPP3CA,CDH10,ASH1L,FMN1,ITGB1,BVES,CORO1C,TRPC4,LIMS1,CNN2,TLN2,FYB1,CFL1,AFDN,MTDH,LCP2,ADAM17,APC,GNA12,PHLDB2,PVR,CTNND2,PACSIN2,SYNPO2,RAI14,PIP5K1A,ADAM10,CDH26,CDHR3,TADA1,SNTB2,FERMT2,NFASC,EPB41L2,CADM3,STXBP6,TNFSF13B,CADM1,CCDC88C,CYTH3,SSX2IP,PARD3B,XIRP2,CASK,HSPG2,FRMD4B*
GO:0098794	postsynapse	0.0001422666886038891	*PARN,EIF4A3,DTNB,GPR158,ATP2B2,SNTA1,GRIN2A,IL1RAPL1,KIF5C,GPM6A,ADCY1,GRID1,FMR1,NETO1,SYNE1,KCTD8,RNF10,USP50,CTTN,USP8,PSD3,MEF2C,DBNL,SLC16A7,NEDD4,*

			EFNB3,DLG3,SH3GL3,GABRB2,GRIN2B,GABRA6,RTN4,CLSTN1,HTT,NECTIN3,PTPRO,KCNB1,ANK3,CNIH3,HIP1,CRKL,APBA1,PPP3CA,CHRNA5,CDH10,DISC1,ASAP1,ITGB1,NEFL,AP2B1,GABRG3,RPH3A,CTTNBP2,ARHGAP32,CHRNA6,TMEM108,SEMA4F,ELAVL1,STXBP1,CTNND2,KCND1,NRP2,SNAP25,ADAM10,SLC4A10,DNM3,SHISA6,INSYN2B,CADM1,PRKAR1B,SCN8A
GO:0098978	glutamatergic synapse	0.0001616511692220231	*PARN,EIF4A3,ATP2B2,FBXL20,GRIN2A,IL1RAPL1,GPM6A,ADCY1,GRID1,NETO1,RNF10,SH3GL2,SLC30A3,USP8,DBNL,SLC16A7,EFNB3,DLG3,FLOT2,MAPK14,SH3GL3,SLC1A2,RTN4,CLSTN1,SPARCL1,PTPRO,HIP1,ATP2B1,APBA1,PPP3CA,CDH10,DISC1,ASAP1,ITGB1,AP2B1,EPHB1,CTTNBP2,SLC1A6,SEMA4F,ELAVL1,STXBP1,PACSIN2,NRP2,PLXNC1,SNAP25,ADAM10,TSPOAP1,DNM3,SYT1,SHISA6,PRKAR1B,SCN8A*
GO:0098588	bounding membrane of organelle	0.00017273724373017535	*SLC18A1,CYTH1,SCYL3,SNX16,CHMP1B,ATP11C,SNX25,AHNAK,SLC46A2,TPTE2,RAB11FIP4,MISFA,ARHGAP26,RAB44,CCZ1B,ZDHHC17,FIG4,AP1S3,WDFY3,OCLN,SNX5,PLEKHB2,SLC66A1,APOO,SYNE1,SLC9A4,GPAM,TSPEAR,ACAP2,CHCHD6,MPHOSPH9,ABCB11,ATG12,SH3GL2,CLBA1,SLC30A3,MTX2,SCAP,USP8,ABCA13,ST8SIA5,DBNL,SERINC3,CD55,PLN,HACE1,ATG4B,SLC7A14,SERPINA1,TRIM37,FAR2,FLOT2,SV2B,RAPGEF1,SH3GL3,SVOP,IFNGR2,DENND4A,TBC1D20,SNX2,SCAMP5,MIGA1,PLEKHF2,PIEZO1,SLC9B2,CD163,BBS2,CLSTN1,TMEM150C,SCYL2,MAOB,MICOS10,PLAAT3,IQGAP1,WIPI1,KLHL12,SNX30,AZIN2,HLA-DMA,PPP2R2B,ACSL6,CANX,GNPAT,SV2C,CNIH3,PLEKHM2,HIP1,HS3ST4,ATP2B1,SPRING1,SLC35B4,PLCE1,BAK1,CLCC1,DYSF,DENND1A,BACE2,GALNT18,ASAH2,STON1,PXMP2,DNAJC11,ASAP1,AQP8,ITGB1,CYB561,LMAN2L,DSE,CORO1C,NSG2,ATP6V1A,FCAR,C1GALT1,MSR1,AP2B1,XYLT2,MCTP2,GLCE,EPHB1,RPH3A,CYTH4,VPS29,COPB2,ARHGAP32,MARCHF5,IQCE,CSGALNACT1,TLR7,COPG2,LRP2,ZDHHC13,GALNT14,TMEM108,SLC39A8,MAP3K7,TMEM59L,ADAM17,ERGIC2,ERC1,RUBCNL,CKAP4,IQGAP2,PACSIN2,RAB8B,EVC2,RIC1,MGAT5,ACOX1,HSD17B4,PIGR,TMEM165,BORCS5,MTOR,SNAP25,CHST4,RAB6B,HS6ST2,ADAM10,WASHC1,SNTB2,PDCD10,HHAT,GOLGA7,ST3GAL2,SYT1,DENND5A,NFASC,GNPNAT1,TGOLN2,PROM1,ACP3,ATF6,GBF1,CYTH3,ARFGEF1,ST8SIA2,HEPACAM2,B3GALNT2,FUNDC2,CYB561A3,CASK,SLCO4C1,GCNT1,GGA2,DNAJC13*
GO:0097447	dendritic tree	0.00032360876424577796	*RIT2,MAX,ATXN10,STAU2,TACR1,ABHD12,EIF4A3,DTNB,ATP2B2,GRIN2A,IL1RAPL1,KIF5C,GPM6A,FMR1,SYNE1,RGS12,OSBP2,USP50,CTTN,USP8,DBNL,NEDD4,URI1,CRHR2,FLOT2,GABRA6,LYPD6,PRKAA2,MAOB,HTT,NECTIN3,MAP2,PTPRO,AZIN2,KCNB1,ANK3,CNIH3,RELN,INPP5A,APBA1,PPP3CA,DENND1A,ASAP1,ITGB1,NSG2,GABRG3,PCSK2,KCNIP1,EPHB1,RPH3A,CTTNBP2,ARHGAP32,KLHL1,UNC5C,LRP2,TMEM108,SEMA4F,TRAK1,CTNND2,DPYSL2,KCND1,MTOR,ADAM10,SLC4A10,DNM3,SHISA6,CADM1,UBX*

			N2A
GO:0098793	presynapse	0.0003413761824497642	*SLC18A1,GPR158,ITSN1,ATP2B2,SCRN1,FBXL20,ITGA2,GRIN2A,GPM6A,ADCY1,ZDHHC17,FMR1,KCTD8,SH3GL2,SLC30A3,ROR1,AAK1,DBNL,CRHR2,EFNB3,SV2B,SH3GL3,SLC1A2,SVOP,SCAMP5,SLC9B2,HTT,CANX,GRM4,SV2C,HIP1,SNAP91,ATP2B1,APBA1,CHRNA5,DYSF,CDH10,DENND1A,STON1,DISC1,NEFL,ATP6V1A,AP2B1,MCTP2,RPH3A,CTTNBP2,SLC1A6,CHRNA6,STXBP1,ERC1,RAB8B,BORCS5,SNAP25,RAB6B,CNTNAP4,CDHR3,SLC4A10,TSPOAP1,DNM3,SYT1,PACSIN1,SCN8A,CASK*
GO:0030425	dendrite	0.0005837501444152523	*MAX,ATXN10,STAU2,TACR1,ABHD12,EIF4A3,DTNB,ATP2B2,GRIN2A,IL1RAPL1,KIF5C,GPM6A,FMR1,SYNE1,RGS12,OSBP2,USP50,CTTN,USP8,DBNL,NEDD4,URI1,CRHR2,FLOT2,GABRA6,LYPD6,PRKAA2,MAOB,HTT,NECTIN3,MAP2,PTPRO,AZIN2,KCNB1,ANK3,CNIH3,RELN,INPP5A,APBA1,PPP3CA,DENND1A,ASAP1,ITGB1,NSG2,GABRG3,PCSK2,KCNIP1,EPHB1,RPH3A,CTTNBP2,ARHGAP32,KLHL1,UNC5C,LRP2,TMEM108,SEMA4F,TRAK1,CTNND2,DPYSL2,KCND1,MTOR,ADAM10,SLC4A10,DNM3,SHISA6,CADM1,UBXN2A*
GO:0110165	cellular anatomical structure	0.0009062004076870665	*SLC18A1,RIT2,SLMAP,KLHL13,ABCB7,PBX4,ACOT12,NHLRC3,MYO9B,GPSM2,COL4A5,ERG,PARN,CYTH1,CHD9,STK16,XK,SEMA4D,MMS22L,MET,MAX,TNS3,SCYL3,TEAD1,ATXN10,KIAA1210,BRCA1,EIF4G3,ACSM3,CNBD2,BAZ1B,SNX16,FGGY,IFTAP,CEP350,STAU2,SIAH3,PIWIL3,CETN3,CHMP1B,MMP16,DRD3,ELAPOR2,FAXC,ATP11C,PRORP,ZNF534,TOX3,DNAH11,CDC42BPA,POLR2J,POLG,EXOC2,PTPN5,TACR1,SNX25,SDC2,ABHD12,GPHB5,UBN2,COX10,CIITA,FANCC,ARAP1,UQCRHL,HEPHL1,KLHL7,CCR3,ANKLE2,HBG2,CEP85,RPRD1B,CIP2A,AHNAK,ACTR5,LRRC53,EIF4A3,DLEC1,NFE2L2,SCHIP1,PARG,OLFML2A,UIMC1,TFEC,NFYC,H2BC18,BLOC1S5-TXNDC5,OTOA,UPP2,CYP19A1,SLC46A2,DTNB,GPR158,VRK2,ITSN1,OAS1,DEUP1,R3HDM2,ATP2B2,CPVL,ESPNL,PREX2,SCRN1,CSMD3,FBF1,FBXL20,NTN4,SERP1,CNTNAP3B,POLR1A,PLEKHM3,ENKUR,TPTE2,RAB11FIP4,HSFX4,NPIPB5,GRPEL2,SNTA1,PPCDC,CLDN10,ASZ1,NUB1,WIF1,MISFA,FLI1,CSNK2A2,SPATS2L,CLIC5,ESRRB,ZFHX2,ARHGAP26,TDRD9,RBP7,TRHDE,RAB44,PTPN4,SLC16A11,SETBP1,ZBTB7C,ITGA2,GRIN2A,NELL2,IL1RAPL1,WDPCP,KIF5C,BLK,GPM6A,SPATA16,WNT16,KRT86,SETD3,CCZ1B,ADCY1,GRID1,RPS6KA2,EFR3B,PLPPR5,AVL9,TRIM24,ZDHHC17,HLCS,FIG4,EIF2AK2,AP1S3,CFAP47,FOXP2,F2RL2,KMT2D,LPAR3,ANO4,GIPC2,FMR1,GFOD1,TXLNG,FOXN3,SP110,SH3TC2,CEP290,FAM171B,WDFY3,SHQ1,TACC1,RAD9B,SH3PXD2B,OCLN,CFAP91,TUT4,AK8,FASTKD5,ITGBL1,NETO1,PTPRQ,TNPO1,GBE1,SNX5,CDK13,SNX32,DNPEP,NEO1,GLT8D1,MYT1L,RASA2,STAG1,CLDN14,ITPKB,PLEKHB2,TIMM23,CAPN11,UBOX5,TCERG1,HBE1,SLC66A1,F10,APOO,SRGAP1,DIO2,FAM184A,CABIN1,SYNE1,PSMD10,FBXL17,PDZD2,SLC9A4,DHX29,GAS*

			2,GPAM,NABP1,IRF2,RUNX2,LRP12,WEE2-AS1,TSPEAR,FILIP1L,LRRC1,ESRP1,HSFX3,UBE2A,BRMS1L,RBM41,RGS12,PPP6R3,ACOXL,OSBP2,CBX3,ACAP2,SLC39A10,CDCA2,KCNV1,ZNF496,KCTD8,ZNF550,CHCHD6,KIZ,MPHOSPH9,RNF10,FAT1,SLC2A2,PIK3R1,STAMBPL1,IL17B,AGMO,ABCB11,SARNP,CFDP1,PGM5,COL26A1,SHTN1,ATG12,FNDC3B,MASP1,TMEM232,USP50,CD200R1,BTD,CALD1,POLR3F,SH3GL2,CLBA1,HIBADH,CRTC1,PITPNC1,MYLK3,SLC30A3,NXPE3,DOCK7,PDK3,MTX2,XKR4,FBXL4,ROR1,SCAP,ADAMTSL1,PAX5,AAK1,CRB1,CTTN,UTP15,PASK,USP8,GPR75,ARID1A,PARD6B,THEMIS,SMAD6,OR2AJ1,RYBP,PSD3,ABCA13,RFX2,ACY3,MEF2C,ZNF519,FZR1,BAIAP2L1,ST8SIA5,DBNL,ELP4,RYK,MACF1,PXT1,TAF4B,MALT1,DENND11,TUBGCP3,ITIH5,WEE2,MTM1,SLC15A5,TFDP2,KCNE1,PTPRE,UNC5B,ZSCAN5A,MFSD6,SERINC3,TBC1D31,ZNF347,PTPN12,CRYZL1,PTPRZ1,RNA5SP232,ACACA,CD55,PLN,EML1,CTDSPL2,SHISA3,SLC66A3,SLC16A7,NEDD4,NEK4,RBBP9,CACNA1E,FLYWCH1,LRRC38,DOCK8,OVAAL,CYSLTR2,VWA8,DDAH1,PARVA,PPP1R8,SECISBP2,URI1,SLC9C1,SCML2,ANKRD28,CHD6,FGD4,PDXK,ZNF609,PRDX1,NDUFAF6,COX17,PACS2,HACE1,SNRPF,ATG4B,SCD5,AEBP2,SLC7A14,DSC2,RBMS2,SERPINA1,GLB1L,MYO10,OLFM1,CRHR2,MID1,POGK,TSHZ2,SLC12A2,OTX1,EFNB3,TRIM37,DLG3,FAR2,SSR3,TRAT1,FLOT2,PVRIG,SRP68,SV2B,ILK,LEF1,EYA1,MAPK14,MYBPC3,WDR17,ZFHX4,SLC25A13,RAPGEF1,ALG13,EFCAB14,GPRC6A,UNC79,SH3GL3,AKR7A2,ZNF516,CERKL,SLC1A2,EIF2S3B,ARHGEF3,GABRB2,EDNRB,GRIN2B,UBXN10,SVOP,ARSB,GPR141,CRISPLD1,NOP16,IFNGR2,DENND4A,TBC1D20,PLD5,OXR1,SFMBT2,SETD5,SNX2,GRHPR,SCAMP5,IL17RB,DCAF7,PARP2,MINDY3,MYL1,KLHL4,OVOL2,SMPX,EIF3F,RDH16,FCHSD2,SLC4A7,RNU6-1117P,DNAJB6,MTCL3,MIGA1,NPIPB3,RNU6-581P,OMA1,SPCS1,MTUS1,GABRA6,LEMD3,PLEKHF2,KHDRBS2,B3GLCT,PIEZO1,SOAT1,OR2T11,SLC9B2,NEB,PTAR1,CSRNP3,KANSL1L,MRGPRX1,TMEM100,FAM120C,LYPD6,PDIA5,XPR1,VDR,SPESP1,NEK11,PDPN,CREB3L2,RTN4,GNL3L,CACNA1D,ARHGEF11,PRKAA2,IVNS1ABP,CD163,SGPP2,DPT,BBS2,CLSTN1,SPAG17,TMEM150C,ZNF155,SERGEF,SCYL2,AOPEP,SKAP1,NT5C3A,MAOB,SKIC3,MRPL3,SERPINE2,MYO3A,MICOS10,NUBPL,PLAAT3,POGLUT2,IL12RB2,PSG2,PIK3CG,ANOS1,COL4A4,TPP1,ANKFN1,STIL,TFDP1,TRGV5,IL1R2,ATP6V1E2,TNPO3,NBPF10,HTT,NUP160,NECTIN3,FREM3,IQGAP1,MYCBP2,WIPI1,LATS1,ZSCAN25,LOC124903099,KLHL12,SNX30,GNPDA2,TSNARE1,FANCI,THNSL1,OR9Q1,ACAA1,OR4K17,SPARCL1,PRSS23,WNT2B,LRRC37A3,TMDD1,SPNS3,TNFRSF10B,MAP2,RNU6-973P,FBXW11,MTCL1,PTPRO,HSPH1,ENSG00000286248,SUGP2,AZIN2,IYD,SMG6,HLA-DMA,SNORD17,PEMT,ARHGAP6,POLR2J2,PPP2R

			2B,CHD5,JAK1,GPD2,ITGA9,ACSL6,TOR1AIP2,ULK2,KCNB1,RADIL,CANX,ZMIZ1,UGP2,CES5A,RP1L1,GNPAT,HSD17B12,HS6ST3,GRM4,ANKRD27,AIM2,ANK3,GTF2IRD1,ZNF19,TMEM132B,PPP1R14C,AMT,KITLG,SV2C,ARID4B,GMDS,ZNF326,MAP4K4,CNIH3,PLEKHM2,NR1I2,THSD4,ZNF266,NXPE1,HIP1,RAET1E,RFX7,RELN,MFHAS1,OR51B5,HS3ST4,PDHB,ADAMTSL3,FBXO32,NXPH1,MTMR3,CRKL,MYO18B,TENT5A,ARHGEF10,FGD6,SYTL3,NDC1,SULT1C4,PTPN13,SNAP91,ZNF816,VAV3,RPS6KC1,INPP5A,CD40LG,VRK1,PAPOLB,URM1,GPAT3,SCN9A,ZNF420,DPYSL3,C1ORF146,ATP2B1,SPRING1,SLC16A10,ATRN,NDUFB9,CCDC88A,SNORA36C,SLC35B4,ARNT2,UGT3A2,RNGTT,APBA1,HPSE2,PLCE1,TNIP3,TACC2,NAALAD2,CYP39A1,DCAF5,GNAL,CSTF1,PEBP4,MSH3,RNU6-10P,MINDY1,BAK1,MRPL45,CASTOR2,LAX1,CACTIN,STK26,CLCC1,ENOX2,TENM4,TTC9-DT,DCP1A,ZCRB1,PPP3CA,CHRNA5,CES2,MKNK1,DYSF,NKD1,ENSG00000286856,RBPJ,CYRIB,C3,RIN2,PDE6C,ASCC1,CDH10,DENND1A,MZT1,PDZRN3,OR52D1,ADAMTS16,TASP1,NBPF19,MICU1,EBPL,TAF10,TRIP12,C3ORF52,MBD5,BACE2,KDM5A,ZNF549,NOMO1,ARL6IP6,ZNF320,CNOT6,PKIB,GALNT18,ASAH2,FRYL,TBC1D9B,STON1,PXMP2,ASH1L,RSPO2,TPMT,ZNF805,ACSF2,OR5K1,OR13C9,DISC1,PROX1,BMP6,MAP3K7CL,FMN1,ZNF891,RALGPS1,CCDC6,MUC5B,CTSB,DNAJC11,ASAP1,VSIG1,AQP8,CREBBP,RPS10-NUDT3,ITGB1,BVES,RBM26,CYB561,DRC7,ZNF827,LMAN2L,DSE,NFKBIZ,RHCE,CORO1C,KANK3,NSG2,LHFPL6,SPIDR,C17ORF80,NEFL,NAV2,ATP6V1A,TMEM185B,STK3,CHN2,RNF150,FCAR,C1GALT1,HNF4G,SMCO4,MSR1,EXOSC3,AP2B1,GABRG3,SH2D3C,APTX,TRPC4,RAMP3,GNG2,ZNF705A,FRS3,CPB1,XYLT2,LIMS1,ENPP2,DOCK4,ACKR2,ARPC1A,UBL4B,MCTP2,BCL11B,CERS3,GLCE,PLS3,PCSK2,TSPAN2,KCNIP1,PWWP2A,GFRA2,TBC1D10A,EPHB1,SSBP2,REEP1,RPH3A,CTTNBP2,SLCO6A1,TULP2,NME9,CYTH4,NAP1L1,SLC38A6,VPS29,PLPP4,CCDC102B,COPB2,UBR1,CNN2,COL4A2,SLC1A6,TCF7L1,ARHGAP32,MRPS10,DYRK3,IP6K2,KRTAP10-12,CNTLN,CHRNA6,TLN2,HDAC4,ZNF582,GDNF,KLHL1,FYB1,TSKS,CALCB,MARCHF5,RBM45,SEM1,GYS2,IQCE,LVRN,MYLK,UNC5C,CFAP69,ACSBG1,CSGALNACT1,CSMD2,TLR7,FLG2,SLC37A2,P3H2,CFL1,DTYMK,KRT20,WDR7,SLAIN2,HMG20A,CEP85L,TUSC3,COPG2,PLEKHG4B,CCDC38,RBBP8,MED13,RNASEH2B,PHF2,TAAR2,RAI2,AFDN,CYTIP,LRP2,MTDH,SEMA6D,LIG1,EHMT1,ZDHHC13,WWC3,IL17RA,SERPINA9,CAMK4,GALNT14,CLPX,VWC2,REPS1,FBXL7,RPA3,RNU6-1229P,TMEM108,CC2D2B,TPTE,BFSP1,LCP2,GAB3,SEZ6L,PCYOX1L,SLC39A8,TMEM123,GLI2,AFG2B,TAS2R13,TGFBR3,MAP3K7,UPRT,GTF2I,ATXN1,TMEM59L,ADAM17,SEMA4F,LIPJ,ELAVL1,S100PBP,TRAK1,STXBP1,OR6N1,IDH1,TI

			MM23B,LMOD2,TSNAX-DISC1,ABCC1,HDGFL2,MUC13,NUP205,PLEKHG1,NHLH1,ELP2,SLCO5A1,SNRNP200,ORC4,CYP8B1,APC,GNA12,TTLL5,IL18R1,INO80,NAA11,MOCS2,ERGIC2,SULT2B1,SPTSSB,PEAR1,CDA,PPARGC1B,STON1-GTF2A1L,CTRB1,COL27A1,PHLDB2,ESR2,NOL10,LNX1,ENSG00000287694,ERC1,SMIM13,ZNF440,ROS1,RUBCNL,AGO3,C9,PVR,CKAP4,TMPRSS11A,MOB3B,RPL39L,ACER1,IQGAP2,CDYL,RLF,CLPB,SPINK1,ANKRA2,TAF5L,JPT1,CTNND2,DNAH14,FRMD3,ERI1,RASSF3,UGGT1,PACSIN2,RDH13,TIA1,DPYSL2,SYNPO2,NUP210L,CNTNAP3C,GIMAP8,RAB8B,METTL8,NSD2,ASCC3,GRM8,SLC23A2,PHACTR3,RMDN2,PAIP2,AARS1,KCND1,PTPN11,TMEM45A,TMEM267,JADE3,PHF3,PSMC2,MAPKAP1,COL15A1,EXOC6B,EVC2,GON4L,RIC1,MGAT5,ACOX1,USP24,HAO1,MAEL,NUP153,CLPTM1,HSD17B4,EFHD1,LCN12,RNF215,TRMT10B,COL21A1,RAI14,GPATCH2L,PIGR,NRP2,GTF3A,SIPA1L2,CAVIN1,GYPE,PLXNC1,KCTD5,METTL15,TMEM165,FOXJ2,RNU4-69P,CEP162,ELOVL7,NR3C1,DLX6,BORCS5,FSTL4,ZC3HAV1,ARHGAP28,MTOR,TRAV5,KSR1,CBLB,FAF1,SNAP25,TUFT1,RP1,DEFB124,SGSM1,ADH7,CHST4,OR11H6,GOLGA8A,ZNF221,CLDND1,PIP5K1A,BTBD2,GH2,MMACHC,DISP1,RAB6B,HS6ST2,KYNU,ADAL,TBXAS1,ASCC2,SPRED1,ADAM10,GALC,GNG4,CNTNAP4,WASHC1,TUBA3D,CDH26,CBY2,FAM53C,PDS5B,CRIM1,NALF2,MEI4,RBM46,SUGP1,PFKFB3,SPANXD,ABCB5,RNF115,TRIM61,DPY19L2,CYYR1,ESD,CDHR3,PER2,ERICH3,PLEKHH2,ANKRD24,GLI3,LHPP,KAT6A,BCOR,KCNK12,NTRK3,RXFP1,SLC35F3,TADA1,GAB2,DIS3L2,CTCF,SNTB2,PDE1A,RSBN1L,PDCD10,PRAMEF11,TFG,EPHX2,LMTK2,HHAT,UBE4A,IDH3A,CREB5,CCDC68,ADGRG6,SLC4A10,GOLGA7,FERMT2,GCNA,GPR82,TSPOAP1,GLUD1,DNM3,ST3GAL2,SYT1,PHIP,EPB41,DENND5A,SS18,SFRP5,DOCK9,AGBL3,ZYG11B,RFC2,PPARA,NCKAP5,CD244,SHISA6,IGLV3-1,NFASC,HDX,SCPEP1,SLC28A2,MAP2K5,ZNF229,NXPE4,PHF6,HMGB4,EPB41L2,CADM3,FGF7,TBCA,PACSIN1,STXBP6,MGA,RTTN,CACNA1A,EDA,CNTRL,LIMCH1,TNFSF13B,GNPNAT1,MTMR7,PTH2R,ELOVL6,MICAL2,COL14A1,SLC22A25,RBM47,EIF2A,JARID2,CYP1B1-AS1,CELSR1,HDAC2,FIRRM,TPO,TGOLN2,FGD1,GSTA3,TRIM44,INSYN2B,TRIM39,SEMA3D,PROM1,OR2T8,NPSR1,EIF4E3,ACP3,USP48,KCNH5,RRM1,MAGEA1,CADM1,INPP5D,CFAP161,THUMPD1,CLYBL,OR4C6,ATF6,PRKAR1B,GBF1,IPO11,SERPINE1,CCDC88C,BACH1,CUL9,PRAMEF4,EWSR1,TM7SF3,CYTH3,KLC1,SLC26A5,AKAP13,HBS1L,MLANA,PKM,GCG,ARFGEF1,ATP12A,TKFC,LINC02218,PTPN3,STAC,SCN8A,RALGAPA1,NNT,SSX2IP,ST8SIA2,TTC17,ELP1,IRF8,HIVEP3,C2ORF42,HEPACAM2,RFX4,CCDC198,OR6K6,ZNF30,REM1,ACTA2,AGAP1,FREM1,RIPOR2,NDUFA10,KCNK5,CD69,B3GALNT2,FUNDC2,DBR1,EPS15L1,CYB561A3,PARD3B,XIRP2,KDM3

			A,MZT2A,CAST,OR2L3,TAOK1,UNC45A,GPR39,ADGRA3,CASK,HSPG2,SLCO4C1,GCNT1,BPIFB3,SLC35F4,GGA2,PRDM7,USH1C,FMN2,UBXN2A,NLRP14,PCNT,DNAJC13,FRMD4B,AFG2A,PSME3IP1,GOLGA8B,TRDV3
GO:0045211	postsynaptic membrane	0.0010106936242287449	*GPR158,ATP2B2,SNTA1,GRIN2A,IL1RAPL1,ADCY1,GRID1,FMR1,NETO1,SYNE1,KCTD8,RNF10,SLC16A7,EFNB3,DLG3,GABRB2,GRIN2B,GABRA6,CLSTN1,NECTIN3,PTPRO,KCNB1,ANK3,CNIH3,HIP1,CRKL,CHRNA5,CDH10,GABRG3,RPH3A,CHRNA6,SEMA4F,KCND1,NRP2,DNM3,SHISA6,SCN8A*
GO:0016328	lateral plasma membrane	0.0010136813491747549	*GPSM2,SNTA1,SLC16A7,SLC12A2,SLC4A7,IQGAP1,MTCL1,PTPRO,KCNB1,ANK3,ATP2B1,BVES,CORO1C,ABCC1,APC,GNA12,SLC4A10,SLC26A5*
GO:0098858	actin-based cell projection	0.0012465988021203355	*ESPNL,GPM6A,FMR1,TSPEAR,FAT1,SHTN1,CRB1,MTM1,FGD4,MYO10,FCHSD2,SLC4A7,PDPN,BBS2,MYO3A,MAP2,KITLG,ITGB1,ATP6V1A,DOCK4,TBC1D10A,EPHB1,UNC5C,IQGAP2,SNAP25,ANKRD24,PROM1,ACP3,ACTA2,RIPOR2,USH1C,FMN2*
GO:0036477	somatodendritic compartment	0.0014676885266181312	*RIT2,MAX,ATXN10,STAU2,TACR1,ABHD12,EIF4A3,DTNB,ATP2B2,GRIN2A,IL1RAPL1,KIF5C,GPM6A,FMR1,WDFY3,SYNE1,RGS12,OSBP2,SHTN1,USP50,CTTN,USP8,DBNL,NEDD4,CACNA1E,URI1,MYO10,OLFM1,CRHR2,SLC12A2,FLOT2,GABRA6,TMEM100,LYPD6,PRKAA2,MAOB,HTT,NECTIN3,MAP2,FBXW11,PTPRO,AZIN2,KCNB1,ANK3,CNIH3,RELN,INPP5A,APBA1,PPP3CA,DENND1A,ASAP1,ITGB1,NSG2,GABRG3,PCSK2,KCNIP1,EPHB1,RPH3A,CTTNBP2,ARHGAP32,KLHL1,UNC5C,LRP2,TMEM108,SEZ6L,SEMA4F,TRAK1,GNA12,CTNND2,DPYSL2,KCND1,MTOR,SNAP25,ADAM10,PDE1A,LMTK2,SLC4A10,DNM3,SHISA6,CACNA1A,RRM1,CADM1,UBXN2A*
GO:0097708	intracellular vesicle	0.0014831189956360674	*SLC18A1,NHLRC3,STK16,KIAA1210,SNX16,CHMP1B,ATP11C,SNX25,BLOC1S5-TXNDC5,SLC46A2,ITSN1,ENKUR,RAB11FIP4,CSNK2A2,ARHGAP26,RAB44,GRIN2A,SPATA16,CCZ1B,AVL9,ZDHHC17,FIG4,AP1S3,SH3TC2,CEP290,OCLN,SNX5,CDK13,SNX32,NEO1,PLEKHB2,CAPN11,SLC9A4,ACAP2,STAMBPL1,ABCB11,ATG12,SH3GL2,CLBA1,SLC30A3,SCAP,AAK1,CTTN,USP8,ABCA13,DBNL,MTM1,CD55,PDXK,PRDX1,ATG4B,DSC2,SERPINA1,SLC12A2,FLOT2,SV2B,MAPK14,RAPGEF1,SH3GL3,GABRB2,GRIN2B,SVOP,ARSB,IFNGR2,DENND4A,SNX2,SCAMP5,FCHSD2,PLEKHF2,SLC9B2,SPESP1,PDPN,CD163,CLSTN1,SPAG17,SCYL2,SERPINE2,TPP1,ATP6V1E2,HTT,IQGAP1,WIPI1,KLHL12,SNX30,ACAA1,HSPH1,AZIN2,IYD,HLA-DMA,JAK1,ULK2,CANX,GRM4,ANKRD27,SV2C,CNIH3,PLEKHM2,HIP1,SYTL3,SNAP91,RPS6KC1,DPYSL3,ATP2B1,CCDC88A,APBA1,STK26,DYSF,CYRIB,C3,RIN2,DENND1A,BACE2,STON1,DISC1,CTSB,AQP8,ITGB1,CYB561,LMAN2L,DSE,CORO1C,NSG2,ATP6V1A,FCAR,MSR1,AP2B1,CPB1,ACKR2,MCTP2,PCSK2,EPHB1,RPH3A,CTTNBP2,NAP1L1,VPS29,COPB2,CNN2,ARHGAP32,AC*

			SBG1,TLR7,FLG2,COPG2,CYTIP,LRP2,ZDHHC13,TMEM108,PCYOX1L,TMEM123,MAP3K7,ELAVL1,TRAK1,STXBP1,IDH1,ERGIC2,CDA,RUBCNL,CKAP4,IQGAP2,PACSIN2,RAB8B,PSMC2,MAPKAP1,PIGR,TMEM165,BORCS5,FSTL4,MTOR,SNAP25,SGSM1,GH2,RAB6B,SPRED1,ADAM10,WASHC1,CBY2,ESD,SNTB2,LMTK2,GOLGA7,SYT1,NFASC,SLC28A2,PACSIN1,GNPNAT1,TGOLN2,ACP3,PRKAR1B,SERPINE1,KLC1,MLANA,PKM,GCG,SCN8A,ST8SIA2,TTC17,CCDC198,CYB561A3,SLCO4C1,GGA2,FMN2,DNAJC13
GO:0031090	organelle membrane	0.002008044578684214	*SLC18A1,ABCB7,CYTH1,XK,SCYL3,SNX16,STAU2,CHMP1B,ATP11C,PTPN5,SNX25,ABHD12,COX10,ARAP1,UQCRHL,ANKLE2,AHNAK,CYP19A1,SLC46A2,VRK2,SCRN1,SERP1,TPTE2,RAB11FIP4,GRPEL2,MISFA,ARHGAP26,RAB44,GRIN2A,CCZ1B,ZDHHC17,FIG4,AP1S3,TXLNG,WDFY3,OCLN,SNX5,PLEKHB2,TIMM23,SLC66A1,APOO,SYNE1,SLC9A4,GPAM,TSPEAR,CBX3,ACAP2,CHCHD6,MPHOSPH9,PIK3R1,AGMO,ABCB11,ATG12,SH3GL2,CLBA1,SLC30A3,MTX2,SCAP,USP8,ABCA13,FZR1,ST8SIA5,DBNL,SERINC3,CD55,PLN,SHISA3,NDUFAF6,HACE1,ATG4B,SCD5,SLC7A14,SERPINA1,SLC12A2,TRIM37,FAR2,SSR3,FLOT2,SV2B,SLC25A13,RAPGEF1,ALG13,SH3GL3,GABRB2,EDNRB,GRIN2B,SVOP,IFNGR2,DENND4A,TBC1D20,SNX2,SCAMP5,MINDY3,RDH16,MIGA1,OMA1,SPCS1,LEMD3,PLEKHF2,B3GLCT,PIEZO1,SOAT1,SLC9B2,PDIA5,CREB3L2,RTN4,CD163,SGPP2,BBS2,CLSTN1,TMEM150C,SCYL2,MAOB,MRPL3,MICOS10,PLAAT3,HTT,IQGAP1,WIPI1,KLHL12,SNX30,AZIN2,IYD,HLA-DMA,PEMT,PPP2R2B,GPD2,ACSL6,TOR1AIP2,ULK2,CANX,GNPAT,HSD17B12,ANKRD27,SV2C,CNIH3,PLEKHM2,HIP1,HS3ST4,NDC1,GPAT3,ATP2B1,SPRING1,NDUFB9,SLC35B4,UGT3A2,PLCE1,CYP39A1,BAK1,STK26,CLCC1,DYSF,DENND1A,MICU1,EBPL,BACE2,NOMO1,ARL6IP6,GALNT18,ASAH2,STON1,PXMP2,FMN1,DNAJC11,ASAP1,AQP8,ITGB1,CYB561,LMAN2L,DSE,CORO1C,NSG2,ATP6V1A,FCAR,C1GALT1,MSR1,AP2B1,XYLT2,ACKR2,MCTP2,CERS3,GLCE,EPHB1,REEP1,RPH3A,CYTH4,VPS29,COPB2,ARHGAP32,MRPS10,MARCHF5,IQCE,CSGALNACT1,TLR7,SLC37A2,TUSC3,COPG2,LRP2,MTDH,ZDHHC13,GALNT14,CLPX,TMEM108,SEZ6L,SLC39A8,MAP3K7,TMEM59L,ADAM17,TRAK1,TIMM23B,NUP205,CYP8B1,ERGIC2,SPTSSB,ERC1,RUBCNL,CKAP4,ACER1,IQGAP2,PACSIN2,RDH13,RAB8B,EVC2,RIC1,MGAT5,ACOX1,NUP153,HSD17B4,EFHD1,TRMT10B,PIGR,TMEM165,ELOVL7,BORCS5,MTOR,KSR1,FAF1,SNAP25,SGSM1,CHST4,GOLGA8A,RAB6B,HS6ST2,TBXAS1,ADAM10,WASHC1,DPY19L2,SNTB2,PDCD10,HHAT,ADGRG6,GOLGA7,ST3GAL2,SYT1,DENND5A,NFASC,SLC28A2,PACSIN1,EDA,GNPNAT1,ELOVL6,TGOLN2,PROM1,ACP3,ATF6,GBF1,CYTH3,MLANA,ARFGEF1,NNT,ST8SIA2,HEPACAM2,NDUFA10,B3GALNT2,FUNDC2,CYB561A3,CASK,SLCO4C1,GCNT1,GGA2,FMN2,DNAJC13,GOLGA8B*
GO:0000	Golgi	0.0020215417849	*CYTH1,SCYL3,TPTE2,ZDHHC17,FIG4,AP1S3,A*

139	membrane	183187	*POO,MPHOSPH9,ABCB11,SH3GL2,SCAP,ST8SIA5,DBNL,SERINC3,CD55,HACE1,IFNGR2,TBC1D20,SCAMP5,CLSTN1,WIPI1,HS3ST4,SPRING1,SLC35B4,PLCE1,CLCC1,GALNT18,ASAH2,ASAP1,LMAN2L,DSE,ATP6V1A,C1GALT1,XYLT2,GLCE,CYTH4,COPB2,ARHGAP32,CSGALNACT1,TLR7,COPG2,ZDHHC13,GALNT14,TMEM59L,ADAM17,ERGIC2,ERC1,RIC1,MGAT5,TMEM165,MTOR,CHST4,RAB6B,HS6ST2,ADAM10,PDCD10,HHAT,GOLGA7,ST3GAL2,DENND5A,GNPNAT1,ATF6,GBF1,CYTH3,ARFGEF1,ST8SIA2,HEPACAM2,B3GALNT2,GCNT1*
GO:0031410	cytoplasmic vesicle	0.002722676665490522	*SLC18A1,NHLRC3,STK16,KIAA1210,SNX16,CHMP1B,ATP11C,SNX25,BLOC1S5-TXNDC5,SLC46A2,ITSN1,ENKUR,RAB11FIP4,CSNK2A2,ARHGAP26,RAB44,GRIN2A,SPATA16,CCZ1B,AVL9,ZDHHC17,FIG4,AP1S3,SH3TC2,CEP290,OCLN,SNX5,CDK13,SNX32,PLEKHB2,CAPN11,SLC9A4,ACAP2,STAMBPL1,ABCB11,ATG12,SH3GL2,CLBA1,SLC30A3,SCAP,AAK1,CTTN,USP8,ABCA13,DBNL,MTM1,CD55,PDXK,PRDX1,ATG4B,DSC2,SERPINA1,SLC12A2,FLOT2,SV2B,MAPK14,RAPGEF1,SH3GL3,GABRB2,GRIN2B,SVOP,ARSB,IFNGR2,DENND4A,SNX2,SCAMP5,FCHSD2,PLEKHF2,SLC9B2,SPESP1,PDPN,CD163,CLSTN1,SPAG17,SCYL2,SERPINE2,TPP1,ATP6V1E2,HTT,IQGAP1,WIPI1,KLHL12,SNX30,ACAA1,HSPH1,AZIN2,IYD,HLA-DMA,JAK1,ULK2,CANX,GRM4,ANKRD27,SV2C,CNIH3,PLEKHM2,HIP1,SYTL3,SNAP91,RPS6KC1,DPYSL3,ATP2B1,CCDC88A,APBA1,STK26,DYSF,CYRIB,C3,RIN2,DENND1A,BACE2,STON1,DISC1,CTSB,ITGB1,CYB561,LMAN2L,DSE,CORO1C,NSG2,ATP6V1A,FCAR,MSR1,AP2B1,CPB1,ACKR2,MCTP2,PCSK2,EPHB1,RPH3A,CTTNBP2,NAP1L1,VPS29,COPB2,CNN2,ARHGAP32,ACSBG1,TLR7,FLG2,COPG2,CYTIP,LRP2,ZDHHC13,TMEM108,PCYOX1L,TMEM123,MAP3K7,ELAVL1,TRAK1,STXBP1,IDH1,ERGIC2,CDA,RUBCNL,CKAP4,IQGAP2,PACSIN2,RAB8B,PSMC2,MAPKAP1,PIGR,TMEM165,BORCS5,FSTL4,MTOR,SNAP25,SGSM1,GH2,RAB6B,SPRED1,ADAM10,WASHC1,CBY2,ESD,SNTB2,LMTK2,GOLGA7,SYT1,NFASC,SLC28A2,PACSIN1,GNPNAT1,TGOLN2,ACP3,PRKAR1B,SERPINE1,KLC1,MLANA,PKM,GCG,SCN8A,ST8SIA2,TTC17,CCDC198,CYB561A3,SLCO4C1,GGA2,FMN2,DNAJC13*
GO:0001726	ruffle	0.0029293134277099423	*MYO9B,SNX5,ACAP2,CTTN,PSD3,DBNL,MACF1,MTM1,FGD4,MYO10,PDPN,IQGAP1,FGD6,ITGB1,CORO1C,SH2D3C,TLN2,CFL1,ADAM17,APC,PACSIN2,KSR1,PIP5K1A,PACSIN1,FGD1,CYTH3,FRMD4B*
GO:0005938	cell cortex	0.0031950462610332592	*MYO9B,GPSM2,EXOC2,CEP85,CLIC5,WDPCP,CALD1,CTTN,PARD6B,DBNL,NEDD4,MYO10,FLOT2,STIL,IQGAP1,FRYL,CORO1C,TRPC4,CTTNBP2,ARHGAP32,GYS2,CYTIP,BFSP1,TRAK1,STXBP1,PHLDB2,ERC1,IQGAP2,EXOC6B,RAI14,SNAP25,WASHC1,PLEKHH2,FERMT2,EPB41,EPB41L2,AKAP13,PARD3B,FMN2*
GO:0042	presyna	0.0053034043431	*GPR158,ITSN1,ATP2B2,GRIN2A,GPM6A,ZDHHC*

734	ptic membrane	01748	*17,FMR1,KCTD8,EFNB3,SLC1A2,HIP1,SNAP91,ATP2B1,APBA1,CDH10,DENND1A,SLC1A6,CHRNA6,STXBP1,ERC1,SNAP25,CNTNAP4,SYT1,SCN8A,CASK*
GO:0030175	filopodium	0.005472975587104871	*GPM6A,FMR1,FAT1,SHTN1,MTM1,FGD4,MYO10,PDPN,MYO3A,MAP2,KITLG,ITGB1,EPHB1,UNC5C,IQGAP2,SNAP25,ACP3,ACTA2,RIPOR2*
GO:0005911	cell-cell junction	0.0061514649155493505	*CYTH1,KIAA1210,CDC42BPA,AHNAK,FBF1,CLDN10,OCLN,CLDN14,PDZD2,FAT1,SLC2A2,PIK3R1,ABCB11,PGM5,CRB1,PARD6B,THEMIS,BAIAP2L1,DSC2,DLG3,FLOT2,SKAP1,NECTIN3,IQGAP1,ANK3,PPP3CA,CDH10,ASH1L,FMN1,ITGB1,BVES,TRPC4,LIMS1,CNN2,AFDN,MTDH,LCP2,ADAM17,APC,PVR,CTNND2,PACSIN2,ADAM10,CDH26,CDHR3,FERMT2,NFASC,CADM3,STXBP6,CADM1,CYTH3,SSX2IP,PARD3B,CASK,FRMD4B*
GO:0019898	extrinsic component of membrane	0.016291010092275307	*SNX16,BLK,RNF10,PIK3R1,USP50,USP8,PIK3CG,WIPI1,JAK1,CRKL,SNAP91,GNAL,PPP3CA,CDH10,ATP6V1A,GNG2,GFRA2,RPH3A,PLEKHG4B,STXBP1,APC,GNA12,SNAP25,GNG4,CDH26,CDHR3,FERMT2,STAC*
GO:0012506	vesicle membrane	0.0181669112516 1764	*SLC18A1,SNX16,CHMP1B,ATP11C,SNX25,SLC46A2,RAB11FIP4,ARHGAP26,RAB44,GRIN2A,ZDHHC17,FIG4,AP1S3,SNX5,PLEKHB2,SLC9A4,ACAP2,ABCB11,ATG12,SH3GL2,CLBA1,SLC30A3,SCAP,USP8,ABCA13,DBNL,CD55,SLC12A2,FLOT2,SV2B,RAPGEF1,SH3GL3,GABRB2,SVOP,IFNGR2,DENND4A,SNX2,SCAMP5,PLEKHF2,SLC9B2,CD163,SCYL2,HTT,IQGAP1,WIPI1,KLHL12,SNX30,IYD,HLA-DMA,ULK2,CANX,ANKRD27,SV2C,CNIH3,PLEKHM2,HIP1,ATP2B1,STK26,DYSF,DENND1A,BACE2,STON1,ITGB1,CYB561,DSE,CORO1C,NSG2,ATP6V1A,FCAR,MSR1,AP2B1,MCTP2,EPHB1,RPH3A,VPS29,COPB2,ARHGAP32,TLR7,COPG2,LRP2,ZDHHC13,TMEM108,MAP3K7,CKAP4,IQGAP2,PACSIN2,RAB8B,PIGR,TMEM165,BORCS5,SNAP25,SGSM1,ADAM10,WASHC1,SNTB2,SYT1,NFASC,SLC28A2,PACSIN1,GNPNAT1,TGOLN2,ACP3,CYB561A3,SLCO4C1,GGA2,FMN2,DNAJC13*
GO:0019897	extrinsic component of plasma membrane	0.0219306017929 8003	*BLK,RNF10,USP50,USP8,JAK1,CRKL,SNAP91,GNAL,PPP3CA,CDH10,GNG2,STXBP1,APC,GNA12,SNAP25,GNG4,CDH26,CDHR3,FERMT2,STAC*
GO:0030659	cytoplasmic vesicle membrane	0.028695383551265025	*SLC18A1,SNX16,CHMP1B,ATP11C,SNX25,SLC46A2,RAB11FIP4,ARHGAP26,RAB44,GRIN2A,ZDHHC17,FIG4,AP1S3,SNX5,PLEKHB2,SLC9A4,ACAP2,ABCB11,ATG12,SH3GL2,CLBA1,SLC30A3,SCAP,USP8,ABCA13,DBNL,CD55,SLC12A2,FLOT2,SV2B,RAPGEF1,SH3GL3,GABRB2,SVOP,IFNGR2,DENND4A,SNX2,SCAMP5,PLEKHF2,SLC9B2,CD163,SCYL2,HTT,IQGAP1,WIPI1,KLHL12,SNX30,IYD,HLA-DMA,ULK2,CANX,ANKRD27,SV2C,CNIH3,PLEKHM2,HIP1,ATP2B1,DYSF,DENND1A,BACE2,STON1,ITGB1,CYB561,DSE,CORO1C,NSG2,ATP6V1A*

			,FCAR,MSR1,AP2B1,MCTP2,EPHB1,RPH3A,VPS29,COPB2,ARHGAP32,TLR7,COPG2,LRP2,ZDHHC13,TMEM108,MAP3K7,CKAP4,IQGAP2,PACSIN2,RAB8B,PIGR,TMEM165,BORCS5,SNAP25,SGSM1,ADAM10,WASHC1,SNTB2,SYT1,NFASC,PACSIN1,GNPNAT1,TGOLN2,ACP3,CYB561A3,SLCO4C1,GGA2,FMN2,DNAJC13
GO:0005622	intracellular anatomical structure	0.03246607351242145	SLC18A1,RIT2,SLMAP,KLHL13,ABCB7,PBX4,ACOT12,NHLRC3,MYO9B,GPSM2,COL4A5,ERG,PARN,CYTH1,CHD9,STK16,XK,MMS22L,MAX,TNS3,SCYL3,TEAD1,ATXN10,KIAA1210,BRCA1,EIF4G3,ACSM3,CNBD2,BAZ1B,SNX16,FGGY,IFTAP,CEP350,STAU2,SIAH3,PIWIL3,CETN3,CHMP1B,MMP16,FAXC,ATP11C,PRORP,ZNF534,TOX3,DNAH11,CDC42BPA,POLR2J,POLG,EXOC2,PTPN5,SNX25,SDC2,ABHD12,GPHB5,UBN2,COX10,CIITA,FANCC,ARAP1,UQCRHL,KLHL7,CCR3,ANKLE2,HBG2,CEP85,RPRD1B,CIP2A,AHNAK,ACTR5,EIF4A3,DLEC1,NFE2L2,SCHIP1,PARG,UIMC1,TFEC,NFYC,H2BC18,BLOC1S5-TXNDC5,UPP2,CYP19A1,SLC46A2,DTNB,GPR158,VRK2,ITSN1,OAS1,DEUP1,R3HDM2,ATP2B2,ESPNL,PREX2,SCRN1,FBF1,FBXL20,SERP1,POLR1A,PLEKHM3,ENKUR,TPTE2,RAB11FIP4,HSFX4,GRPEL2,SNTA1,PPCDC,CLDN10,ASZ1,NUB1,MISFA,FLI1,CSNK2A2,SPATS2L,CLIC5,ESRRB,ZFHX2,ARHGAP26,TDRD9,RBP7,TRHDE,RAB44,PTPN4,SETBP1,ZBTB7C,ITGA2,GRIN2A,NELL2,IL1RAPL1,WDPCP,KIF5C,BLK,SPATA16,WNT16,KRT86,SETD3,CCZ1B,ADCY1,RPS6KA2,EFR3B,AVL9,TRIM24,ZDHHC17,HLCS,FIG4,EIF2AK2,AP1S3,CFAP47,FOXP2,KMT2D,LPAR3,GIPC2,FMR1,TXLNG,FOXN3,SP110,SH3TC2,CEP290,WDFY3,SHQ1,TACC1,RAD9B,SH3PXD2B,OCLN,CFAP91,TUT4,AK8,FASTKD5,NETO1,TNPO1,GBE1,SNX5,CDK13,SNX32,DNPEP,NEO1,GLT8D1,MYT1L,RASA2,STAG1,CLDN14,ITPKB,PLEKHB2,TIMM23,CAPN11,UBOX5,TCERG1,HBE1,SLC66A1,F10,APOO,SRGAP1,FAM184A,CABIN1,SYNE1,PSMD10,FBXL17,PDZD2,SLC9A4,DHX29,GAS2,GPAM,NABP1,IRF2,RUNX2,WEE2-AS1,FILIP1L,LRRC1,ESRP1,HSFX3,UBE2A,BRMS1L,RBM41,RGS12,PPP6R3,ACOXL,OSBP2,CBX3,ACAP2,CDCA2,ZNF496,ZNF550,CHCHD6,KIZ,MPHOSPH9,RNF10,FAT1,SLC2A2,PIK3R1,STAMBPL1,AGMO,ABCB11,SARNP,CFDP1,PGM5,COL26A1,SHTN1,ATG12,MASP1,TMEM232,USP50,BTD,CALD1,POLR3F,SH3GL2,CLBA1,HIBADH,CRTC1,PITPNC1,MYLK3,SLC30A3,DOCK7,PDK3,MTX2,FBXL4,ROR1,SCAP,ADAMTSL1,PAX5,AAK1,CTTN,UTP15,PASK,USP8,ARID1A,PARD6B,THEMIS,SMAD6,RYBP,ABCA13,RFX2,ACY3,MEF2C,ZNF519,FZR1,BAIAP2L1,ST8SIA5,DBNL,ELP4,RYK,MACF1,PXT1,TAF4B,MALT1,DENND11,TUBGCP3,WEE2,MTM1,TFDP2,KCNE1,PTPRE,ZSCAN5A,SERINC3,TBC1D31,ZNF347,PTPN12,CRYZL1,RNA5SP232,ACACA,CD55,PLN,EML1,CTDSPL2,SHISA3,SLC16A7,NEDD4,NEK4,RBBP9,FLYWCH1,DOCK8,OVAAL,VWA8,DDAH1,PARVA,PPP1R8,SECISBP2,URI1,SCML2,ANKRD28,CHD6,FGD4,PDXK,ZNF609,PRDX1,NDUFAF6,COX17,P

			ACS2,HACE1,SNRPF,ATG4B,SCD5,AEBP2,SLC7A14,DSC2,RBMS2,SERPINA1,GLB1L,MYO10,OLFM1,MID1,POGK,TSHZ2,SLC12A2,OTX1,TRIM37,DLG3,FAR2,SSR3,TRAT1,FLOT2,SRP68,SV2B,ILK,LEF1,EYA1,MAPK14,MYBPC3,ZFHX4,SLC25A13,RAPGEF1,ALG13,SH3GL3,AKR7A2,ZNF516,CERKL,EIF2S3B,ARHGEF3,GABRB2,EDNRB,GRIN2B,UBXN10,SVOP,ARSB,NOP16,IFNGR2,DENND4A,TBC1D20,OXR1,SFMBT2,SETD5,SNX2,GRHPR,SCAMP5,IL17RB,DCAF7,PARP2,MINDY3,MYL1,KLHL4,OVOL2,SMPX,EIF3F,RDH16,FCHSD2,RNU6-1117P,DNAJB6,MIGA1,NPIPB3,RNU6-581P,OMA1,SPCS1,MTUS1,LEMD3,PLEKHF2,KHDRBS2,B3GLCT,PIEZO1,SOAT1,SLC9B2,NEB,PTAR1,CSRNP3,KANSL1L,TMEM100,FAM120C,LYPD6,PDIA5,XPR1,VDR,SPESP1,NEK11,PDPN,CREB3L2,RTN4,GNL3L,CACNA1D,ARHGEF11,PRKAA2,IVNS1ABP,CD163,SGPP2,BBS2,CLSTN1,SPAG17,TMEM150C,ZNF155,SERGEF,SCYL2,AOPEP,SKAP1,NT5C3A,MAOB,SKIC3,MRPL3,SERPINE2,MYO3A,MICOS10,NUBPL,PLAAT3,POGLUT2,PIK3CG,COL4A4,TPP1,ANKFN1,STIL,TFDP1,IL1R2,ATP6V1E2,TNPO3,NBPF10,HTT,NUP160,IQGAP1,MYCBP2,WIPI1,LATS1,ZSCAN25,LOC124903099,KLHL12,SNX30,GNPDA2,TSNARE1,FANCI,THNSL1,ACAA1,SPARCL1,PRSS23,WNT2B,MAP2,RNU6-973P,FBXW11,MTCL1,HSPH1,ENSG00000286248,SUGP2,AZIN2,IYD,SMG6,HLA-DMA,SNORD17,PEMT,ARHGAP6,POLR2J2,PPP2R2B,CHD5,JAK1,GPD2,ACSL6,TOR1AIP2,ULK2,KCNB1,RADIL,CANX,ZMIZ1,UGP2,RP1L1,GNPAT,HSD17B12,HS6ST3,GRM4,ANKRD27,AIM2,ANK3,GTF2IRD1,ZNF19,PPP1R14C,AMT,KITLG,SV2C,ARID4B,GMDS,ZNF326,MAP4K4,CNIH3,PLEKHM2,NR1I2,ZNF266,HIP1,RFX7,RELN,MFHAS1,HS3ST4,PDHB,ADAMTSL3,FBXO32,MTMR3,CRKL,MYO18B,TENT5A,ARHGEF10,FGD6,SYTL3,NDC1,SULT1C4,PTPN13,SNAP91,ZNF816,VAV3,RPS6KC1,CD40LG,VRK1,PAPOLB,URM1,GPAT3,ZNF420,DPYSL3,C1ORF146,ATP2B1,SPRING1,SLC16A10,ATRN,NDUFB9,CCDC88A,SNORA36C,SLC35B4,ARNT2,UGT3A2,RNGTT,APBA1,PLCE1,TNIP3,TACC2,CYP39A1,DCAF5,CSTF1,MSH3,RNU6-10P,MINDY1,BAK1,MRPL45,CASTOR2,LAX1,CACTIN,STK26,CLCC1,ENOX2,TENM4,TTC9-DT,DCP1A,ZCRB1,PPP3CA,CES2,MKNK1,DYSF,NKD1,ENSG00000286856,RBPJ,CYRIB,C3,RIN2,ASCC1,DENND1A,MZT1,PDZRN3,TASP1,NBPF19,MICU1,EBPL,TAF10,TRIP12,C3ORF52,MBD5,BACE2,KDM5A,ZNF549,NOMO1,ARL6IP6,ZNF320,CNOT6,PKIB,GALNT18,ASAH2,FRYL,STON1,PXMP2,ASH1L,TPMT,ZNF805,ACSF2,OR13C9,DISC1,PROX1,MAP3K7CL,FMN1,ZNF891,RALGPS1,CCDC6,MUC5B,CTSB,DNAJC11,ASAP1,AQP8,CREBBP,RPS10-NUDT3,ITGB1,RBM26,CYB561,DRC7,ZNF827,LMAN2L,DSE,NFKBIZ,CORO1C,KANK3,NSG2,SPIDR,C17ORF80,NEFL,NAV2,ATP6V1A,STK3,CHN

			2,RNF150,FCAR,C1GALT1,HNF4G,MSR1,EXOSC3,AP2B1,GABRG3,SH2D3C,APTX,TRPC4,RAMP3,ZNF705A,FRS3,CPB1,XYLT2,LIMS1,DOCK4,ACKR2,ARPC1A,UBL4B,MCTP2,BCL11B,CERS3,GLCE,PLS3,PCSK2,TSPAN2,KCNIP1,PWWP2A,TBC1D10A,EPHB1,SSBP2,REEP1,RPH3A,CTTNBP2,TULP2,NME9,CYTH4,NAP1L1,VPS29,CCDC102B,COPB2,UBR1,CNN2,COL4A2,SLC1A6,TCF7L1,ARHGAP32,MRPS10,DYRK3,IP6K2,KRTAP10-12,CNTLN,TLN2,HDAC4,ZNF582,GDNF,KLHL1,FYB1,TSKS,MARCHF5,RBM45,SEM1,GYS2,LVRN,MYLK,CFAP69,ACSBG1,CSGALNACT1,TLR7,FLG2,SLC37A2,P3H2,CFL1,DTYMK,KRT20,WDR7,SLAIN2,HMG20A,CEP85L,TUSC3,COPG2,PLEKHG4B,CCDC38,RBBP8,MED13,RNASEH2B,PHF2,RAI2,AFDN,CYTIP,LRP2,MTDH,SEMA6D,LIG1,EHMT1,ZDHHC13,WWC3,SERPINA9,CAMK4,GALNT14,CLPX,REPS1,FBXL7,RPA3,RNU6-1229P,TMEM108,TPTE,BFSP1,LCP2,GAB3,SEZ6L,PCYOX1L,SLC39A8,TMEM123,GLI2,AFG2B,MAP3K7,UPRT,GTF2I,ATXN1,TMEM59L,ADAM17,SEMA4F,LIPJ,ELAVL1,S100PBP,TRAK1,STXBP1,IDH1,TIMM23B,LMOD2,TSNAX-DISC1,HDGFL2,MUC13,NUP205,PLEKHG1,NHLH1,ELP2,SLCO5A1,SNRNP200,ORC4,CYP8B1,APC,GNA12,TTLL5,INO80,NAA11,MOCS2,ERGIC2,SULT2B1,SPTSSB,CDA,PPARGC1B,STON1-GTF2A1L,COL27A1,PHLDB2,ESR2,NOL10,LNX1,ERC1,ZNF440,ROS1,RUBCNL,AGO3,PVR,CKAP4,MOB3B,RPL39L,ACER1,IQGAP2,CDYL,RLF,CLPB,ANKRA2,TAF5L,JPT1,CTNND2,DNAH14,FRMD3,ERI1,RASSF3,UGGT1,PACSIN2,RDH13,TIA1,DPYSL2,SYNPO2,NUP210L,GIMAP8,RAB8B,METTL8,NSD2,ASCC3,SLC23A2,PHACTR3,RMDN2,PAIP2,AARS1,PTPN11,JADE3,PHF3,PSMC2,MAPKAP1,COL15A1,EXOC6B,EVC2,GON4L,RIC1,MGAT5,ACOX1,USP24,HAO1,MAEL,NUP153,HSD17B4,EFHD1,RNF215,TRMT10B,COL21A1,RAI14,GPATCH2L,PIGR,GTF3A,SIPA1L2,CAVIN1,KCTD5,METTL15,TMEM165,FOXJ2,RNU4-69P,CEP162,ELOVL7,NR3C1,DLX6,BORCS5,FSTL4,ZC3HAV1,ARHGAP28,MTOR,KSR1,CBLB,FAF1,SNAP25,TUFT1,RP1,SGSM1,ADH7,CHST4,GOLGA8A,ZNF221,PIP5K1A,BTBD2,GH2,MMACHC,RAB6B,HS6ST2,KYNU,ADAL,TBXAS1,ASCC2,SPRED1,ADAM10,GALC,WASHC1,TUBA3D,CDH26,CBY2,FAM53C,PDS5B,MEI4,RBM46,SUGP1,PFKFB3,SPANXD,RNF115,TRIM61,DPY19L2,ESD,PER2,ERICH3,PLEKHH2,GLI3,LHPP,KAT6A,BCOR,TADA1,GAB2,DIS3L2,CTCF,SNTB2,PDE1A,RSBN1L,PDCD10,PRAMEF11,TFG,EPHX2,LMTK2,HHAT,UBE4A,IDH3A,CREB5,CCDC68,ADGRG6,GOLGA7,FERMT2,GCNA,TSPOAP1,GLUD1,DNM3,ST3GAL2,SYT1,PHIP,EPB41,DENND5A,SS18,DOCK9,AGBL3,ZYG11B,RFC2,PPARA,NCKAP5,NFASC,HDX,SCPEP1,SLC28A2,MAP2K5,ZNF229,PHF6,HMGB4,EPB41L2,FGF7,TBCA,PACSIN1,STXBP6,MGA,RTTN,CACNA1A,EDA,CNTRL,LIMCH1,TNFSF13B,GNPNAT1,MTMR7,ELOVL6,MICAL2,COL14A1,RBM47,EIF2A,JARID2,CYP1B1-AS1,CELSR1,HDAC2,FIRRM,TGOLN2,FGD1,GST

			A3,TRIM44,TRIM39,PROM1,NPSR1,EIF4E3,ACP3,USP48,RRM1,MAGEA1,INPP5D,CFAP161,THUMPD1,CLYBL,ATF6,PRKAR1B,GBF1,IPO11,SERPINE1,CCDC88C,BACH1,CUL9,PRAMEF4,EWSR1,CYTH3,KLC1,AKAP13,HBS1L,MLANA,PKM,GCG,ARFGEF1,ATP12A,TKFC,LINC02218,PTPN3,STAC,SCN8A,RALGAPA1,NNT,SSX2IP,ST8SIA2,TTC17,ELP1,IRF8,HIVEP3,C2ORF42,HEPACAM2,RFX4,CCDC198,ZNF30,REM1,ACTA2,AGAP1,RIPOR2,NDUFA10,B3GALNT2,FUNDC2,DBR1,EPS15L1,CYB561A3,PARD3B,XIRP2,KDM3A,MZT2A,CAST,TAOK1,UNC45A,CASK,HSPG2,SLCO4C1,GCNT1,BPIFB3,GGA2,PRDM7,USH1C,FMN2,UBXN2A,NLRP14,PCNT,DNAJC13,FRMD4B,AFG2A,PSME3IP1,GOLGA8B
GO:0005654	nucleoplasm	0.033037983258829166	RIT2,ACOT12,ERG,PARN,CHD9,STK16,MMS22L,MAX,TEAD1,BRCA1,BAZ1B,SIAH3,CHMP1B,PRORP,TOX3,POLR2J,PTPN5,UBN2,CIITA,FANCC,ARAP1,KLHL7,RPRD1B,ACTR5,EIF4A3,NFE2L2,SCHIP1,PARG,UIMC1,TFEC,NFYC,H2BC18,OAS1,POLR1A,NUB1,FLI1,CSNK2A2,SPATS2L,ESRRB,PTPN4,SETBP1,SETD3,RPS6KA2,TRIM24,EIF2AK2,KMT2D,FMR1,SP110,WDFY3,SHQ1,RAD9B,CDK13,NEO1,STAG1,UBOX5,TCERG1,CABIN1,SYNE1,PSMD10,FBXL17,NABP1,IRF2,RUNX2,ESRP1,UBE2A,BRMS1L,RGS12,PPP6R3,CBX3,CDCA2,SARNP,CFDP1,ATG12,MASP1,POLR3F,CRTC1,PITPNC1,FBXL4,PAX5,UTP15,ARID1A,SMAD6,RYBP,MEF2C,FZR1,BAIAP2L1,ELP4,TAF4B,WEE2,TFDP2,PTPN12,CTDSPL2,SLC16A7,RBBP9,FLYWCH1,PPP1R8,SECISBP2,URI1,ANKRD28,CHD6,PDXK,ZNF609,HACE1,SNRPF,AEBP2,SLC7A14,POGK,TRIM37,LEF1,EYA1,MAPK14,CERKL,NOP16,SFMBT2,SETD5,DCAF7,PARP2,MINDY3,DNAJB6,KHDRBS2,VDR,SPESP1,NEK11,CREB3L2,GNL3L,ARHGEF11,PRKAA2,IVNS1ABP,SERGEF,SKAP1,NT5C3A,SKIC3,POGLUT2,STIL,TFDP1,HTT,MYCBP2,FANCI,HSPH1,SUGP2,IYD,POLR2J2,CHD5,ZMIZ1,AIM2,GTF2IRD1,AMT,ARID4B,ZNF326,NR1I2,PDHB,FBXO32,CRKL,PTPN13,VRK1,ZNF420,ATP2B1,ARNT2,RNGTT,TACC2,DCAF5,CSTF1,MSH3,MINDY1,CACTIN,ZCRB1,PPP3CA,MKNK1,RBPJ,ASCC1,TAF10,TRIP12,MBD5,KDM5A,ASH1L,OR13C9,PROX1,DNAJC11,CREBBP,NFKBIZ,SPIDR,NAV2,ATP6V1A,HNF4G,EXOSC3,APTX,ACKR2,MCTP2,PCSK2,TSPAN2,TCF7L1,ARHGAP32,DYRK3,IP6K2,CNTLN,HDAC4,RBM45,SEM1,P3H2,RBBP8,MED13,RNASEH2B,PHF2,AFDN,CYTIP,MTDH,LIG1,EHMT1,CAMK4,CLPX,REPS1,RPA3,GLI2,UPRT,GTF2I,ATXN1,ELAVL1,S100PBP,STXBP1,PLEKHG1,ELP2,SNRNP200,ORC4,APC,INO80,NAA11,MOCS2,PPARGC1B,STON1-GTF2A1L,ESR2,AGO3,CDYL,TAF5L,PACSIN2,TIA1,SYNPO2,NSD2,ASCC3,PHACTR3,PTPN11,JADE3,PSMC2,MAPKAP1,GON4L,USP24,NUP153,TRMT10B,RAI14,GTF3A,CAVIN1,FOXJ2,CEP162,NR3C1,ARHGAP28,MTOR,CBLB,FAF1,PIP5K1A,KYNU,ASCC2,SPRED1,PDS5B,SUGP1,PFKFB3,PER2,PLEKHH2,GLI3,LHPP,KAT6A,BCOR,TADA1,CTCF,SNTB2,FERMT2,GCNA,EPB41,RFC2,P

			PARA,PHF6,EPB41L2,MGA,JARID2,CELSR1,HDAC2,TGOLN2,USP48,THUMPD1,ATF6,IPO11,BACH1,EWSR1,CYTH3,ARFGEF1,LINC02218,IRF8,C2ORF42,HEPACAM2,DBR1,KDM3A,MZT2A,UNC45A,PSME3IP1

Table S14. GO associations with biological processes (BP), molecular functions (MF), and cellular components (CC) of 1055 rDNA-contacting genes that exhibited a decrease in the number of contacts with nucleoli in Mel Z cells grown on Matrigel. Related to Figure 6B.

GO.ID	Description	padj	Genes
		MF	
GO:0046872	metal ion binding	0.026029315303996912	*MSRB1,BBOX1,MEFV,SRPK1,CBLL1,ZNF239,XRN2,UNC13B,DYRK4,ADCY3,CPD,TANK,PLA2G4C,SULF2,GCH1,MELK,ZNF536,ZFYVE9,ZNG1A,ALOX12,SLC31A1,MDM2,FBLN2,LITAFD,MBTD1,PPP1CB,RNF111,ESYT2,PGR,TET1,ZNF737,RIMKLA,RFFL,ZNF821,SUCLG2,ZSWIM3,UBR4,ARL6,CHPT1,MPPED2,PRTFDC1,TRAF3,CHD3,EFCAB7,ZMYND8,ZNF765,RASEF,POLA1,ADAMTS19,ITGB6,NR1H2,ADAM9,RNFT2,MGMT,POLQ,SHMT1,PRICKLE2,SF1,ITGAM,CPA1,TGM6,ADGRL3,KMT2A,ZNF607,ANKIB1,EGLN3,CLEC4A,ZNF28,ZKSCAN7,AOAH,RASA3,PNKD,DMP1,ZNF181,ENSG00000286132,ZNF529,EFHC1,RYR1,ZNF728,PRIM2,ATP8B4,ZNF254,COL5A2,FNTB,MCFD2,REPS2,ZNF468,MEP1A,SMAP1,RERE,MTARC2,NR3C2,ZC4H2,NEK5,STIM2,ZNF589,CYP3A43,XPNPEP3,ZNF616,DPEP1,ZNF106,MEP1B,PDLIM5,EFCAB11,EYA2,ZNF652,PDE6A,MAST2,SETD2,CA13,LIN54,ZNF197,ZNF397,MIS18A,TRPV6,MARK1,PLSCR2,ATP13A3,ZNF407,ASPA,STK32A,ZNF234,ZC2HC1C,PAPPA2,TRERF1,CPPED1,EFHC2,SMYD2,SEC23A,ZC3H13,ASXL3,CYP4X1,EBF4,BPTF,ZNF844,SRPK2,PADI2,LNX2,TMEM163,ZNF525,BRPF3,RIMS2,SIK3,BCL11A,FKBP9,RANBP2,RNF128,GALNTL5*
GO:0004897	ciliary neurotrophic factor receptor activity	0.03809205298406871	*IL6R,IL6ST,LIFR*
		BP	
GO:0065007	biological regulation	5.860363926319896e-7	*MEFV,BANK1,SRPK1,SYN3,CDK8,CBLL1,BCL2,ZNF239,XRN2,CNOT1,RICTOR,UNC13B,IL6R,SORBS2,RIC3,KCNK10,EXOC1,NPFFR2,AKIRIN1,ADCY3,TANK,GPR135,PLA2G4C,ENSG00000287286,SLC16A2,SULF2,TAF1D,GCH1,MELK,ZNF536,SRRD,KANK4,OR1B1,KIR2DL1,CBLN2,PPP1R17,ZFYVE9,SCTR,AMPH,HNRNPH1,ALOX12,TMBIM1,BPIFB1,SLC31A1,TMEM182,MDM2,TDRD3,SMARCA4,SGTA,ADGRG2,AKAP3,SLC22A13,TBL1XR1,TNFSF8,FBLN2,C5AR2,PPP1R36,LITAFD,TXNRD1,TNFRSF21,MBTD1,PPP1CB,TRIOBP,RNF111,IST1,RAB22A,ATXN1L,TBC1D32,GNG7,PGR,MTERF3,MBP,DOK5,TET1,ZNF737,TMEM135,MED26,RFFL,ZNF821,BPI,LBH,SETD4,TWIST1,HORMAD1,UBR4,JHY,UGT2B28,ARL6,RAP1GDS1,PALS1,CHPT1,ACTR3,C8A,AMBRA1,DSP,PTK2B,ABCG1,SCOC,CDK12,CAMSAP3,MYO6,KIFAP3,BORA,SLCO3A1,GRK3,TRAF3,CHD3,CD40,EFCAB7,ZMYND8,GSAP,SPICE1,PRMT2,GRIK4,ZNF765,*

			GRHL2,POLA1,SMARCA5,ITGB6,NR1H2,ADAM9,MAD1L1,GARNL3,RNFT2,CASP7,UBE2B,MGMT,POLQ,ARHGAP23,SHMT1,PRICKLE2,SF1,ITGB3BP,WDR47,PKN2,OR4M2-OT1,ITGAM,PRDM11,FUT1,CC2D2A,TCP10L,MYRF,ORC2,OTX2,CCDC80,PRODH,DIAPH1,ADGRL3,PCM1,HELLS,SHROOM3,IL6ST,PAK5,KIF18A,ANAPC5,KMT2A,ARHGAP44,MLF2,GFAP,DISP2,CAP2,PSMD1,CSNK1G3,WWTR1,COBL,ZNF607,RASAL2,NRCAM,HIPK2,ANKIB1,PCSK5,EGLN3,CLEC4A,ZNF28,WNT4,LPAR1,YTHDC1,ZKSCAN7,AOAH,RASA3,IL7,C2CD4A,PNKD,OR4F6,TFIP11,GRAP2,RTKN2,DMP1,HSD17B2,TLN1,SLC4A8,ZNF181,MTMR1,EPS8,ARHGAP21,CAND1,ZNF529,DMTN,RASGRF1,FRMD5,MCU,ALCAM,EDNRA,EFHC1,PTMS,HHLA2,RYR1,SLAMF1,ZNF728,AAMDC,PRIM2,MEGF10,GABBR1,TMEM25,ATP8B4,ERCC1,FAM3B,ZNF254,COL5A2,GRIA1,IL13RA1,REPS2,ZNF468,ARMH4,ATXN2,NFATC3,KCNMB3,SLC7A11,MEP1A,OR4D2,ABCA12,CD86,WWP1,POLR2M,COL8A1,SMAP1,RERE,ALS2,ECT2,GSG1L,HERC1,NR3C2,CRADD,OR4X2,ZC4H2,GAREM1,NXN,TSPAN12,NEK5,IL17RD,NAMPT,ELOA,TTC36,TNRC6A,DMXL2,STIM2,TTC8,WDR77,ZNF589,ZNF616,PLA2G7,TENT5B,RAB11FIP1,DPEP1,ZNF106,COL4A1,MYZAP,MX1,OR2A14,HSBP1L1,CD47,GRAP,CLDN1,LARP4,TSHR,LDOC1,ATP6V0E2,MTCH2,EPHB2,TEX15,PDLIM5,EYA2,ZNF652,PDE6A,PTEN,MAST2,SETD2,UTP4,VEPH1,CROCC,SOX21,LIN54,LRRC8C,ZNF197,ZNF397,TRPV6,MARK1,PLSCR2,MBOAT1,CAMK2D,SERPINB7,IL31RA,ZNF407,OR2T27,SLC9A7,MUC12,ST8SIA1,ASPA,ATG5,MIR922,STK32A,SPATA22,ZNF234,PAPPA2,SLC12A9,MTERF4,TRERF1,NELFB,TBX15,EFHC2,SMYD2,NSUN2,PTPRK,ZC3H13,PHACTR4,OR2M5,SYNDIG1,ASXL3,TRAPPC12,EBF4,SMURF1,LAMB1,BPTF,NFIB,UQCC2,LIFR,KCNAB2,ZNF844,SRPK2,TNFAIP8,TNN,BANP,PADI2,SYCP2,POU2F3,RGS6,SRGAP3,ZNF525,BRPF3,RIMS2,PLD3,SIK3,CHRNA9,LRRC28,BCL11A,CCDC92,RANBP2,CLDN18,RNF128,NDRG3,RUBCN
GO:0050789	regulation of biological process	8.2658274734 48487e-7	*MEFV,BANK1,SRPK1,SYN3,CDK8,CBLL1,BCL2,ZNF239,XRN2,CNOT1,RICTOR,UNC13B,IL6R,SORBS2,RIC3,KCNK10,EXOC1,NPFFR2,AKIRIN1,ADCY3,TANK,GPR135,PLA2G4C,ENSG00000287286,SLC16A2,SULF2,TAF1D,GCH1,MELK,ZNF536,SRRD,KANK4,OR1B1,KIR2DL1,CBLN2,PPP1R17,ZFYVE9,SCTR,AMPH,HNRNPH1,ALOX12,TMBIM1,BPIFB1,SLC31A1,TMEM182,MDM2,TDRD3,SMARCA4,SGTA,ADGRG2,AKAP3,SLC22A13,TBL1XR1,TNFSF8,FBLN2,C5AR2,PPP1R36,LITAFD,TXNRD1,TNFRSF21,MBTD1,PPP1CB,TRIOBP,RNF111,IST1,ATXN1L,TBC1D32,GNG7,PGR,MTERF3,MBP,DOK5,TET1,ZNF737,TMEM135,MED26,RFFL,ZNF821,BPI,LBH,SETD4,TWIST1,HORMAD1,UBR4,JHY,ARL6,RAP1GDS1,PALS1,CHPT1,ACTR3,C8A,AMBRA1,DSP,PTK2B,ABCG1,SCOC,CDK12,CAMSAP3,MYO6,KIFAP3,BORA,SLCO3A1,GRK3,TRAF3,CHD3,CD40,EFCAB7,ZMYND8,GSAP,SPICE1,PRMT2,GRIK4,ZNF765,GRHL2,POLA1,SMARCA5,ITGB6,NR1H2,ADAM9,MAD1L1,GARNL3,RNFT2,CASP7,UBE2B,MGMT,POLQ,ARHGAP23,SHMT1,PRICKLE2,SF1,ITGB3BP,WDR47,PKN2,OR4M2-OT1,ITGAM,PRDM11,FUT1,CC2D2A,TCP10L,MYRF,ORC2,OTX2,CCDC80,PRODH,DIAPH1,ADGRL3,PCM1,HELLS,SHROOM3,IL6ST,PAK5,KIF18A,ANAPC5,KMT2A,A*

			RHGAP44,MLF2,GFAP,DISP2,CAP2,PSMD1,CSNK1G3,WWTR1,COBL,ZNF607,RASAL2,NRCAM,HIPK2,ANKIB1,PCSK5,EGLN3,CLEC4A,ZNF28,WNT4,LPAR1,YTHDC1,ZKSCAN7,AOAH,RASA3,IL7,C2CD4A,PNKD,OR4F6,TFIP11,GRAP2,RTKN2,DMP1,TLN1,SLC4A8,ZNF181,MTMR1,EPS8,ARHGAP21,CAND1,ZNF529,DMTN,RASGRF1,FRMD5,MCU,ALCAM,EDNRA,EFHC1,PTMS,HHLA2,RYR1,SLAMF1,ZNF728,AAMDC,PRIM2,MEGF10,GABBR1,TMEM25,ERCC1,FAM3B,ZNF254,COL5A2,GRIA1,IL13RA1,REPS2,ZNF468,ARMH4,ATXN2,NFATC3,SLC7A11,OR4D2,ABCA12,CD86,WWP1,POLR2M,COL8A1,SMAP1,RERE,ALS2,ECT2,GSG1L,HERC1,NR3C2,CRADD,OR4X2,ZC4H2,GAREM1,NXN,TSPAN12,NEK5,IL17RD,NAMPT,ELOA,TTC36,TNRC6A,STIM2,TTC8,WDR77,ZNF589,ZNF616,PLA2G7,TENT5B,RAB11FIP1,DPEP1,ZNF106,COL4A1,MYZAP,MX1,OR2A14,HSBP1L1,CD47,GRAP,CLDN1,LARP4,TSHR,LDOC1,ATP6V0E2,MTCH2,EPHB2,TEX15,PDLIM5,EYA2,ZNF652,PDE6A,PTEN,MAST2,SETD2,UTP4,VEPH1,CROCC,SOX21,LIN54,LRRC8C,ZNF197,ZNF397,TRPV6,MARK1,MBOAT1,CAMK2D,SERPINB7,IL31RA,ZNF407,OR2T27,SLC9A7,MUC12,ST8SIA1,ASPA,ATG5,MIR922,STK32A,SPATA22,ZNF234,PAPPA2,MTERF4,TRERF1,NELFB,TBX15,EFHC2,SMYD2,NSUN2,PTPRK,ZC3H13,PHACTR4,OR2M5,SYNDIG1,ASXL3,TRAPPC12,EBF4,SMURF1,LAMB1,BPTF,NFIB,UQCC2,LIFR,KCNAB2,ZNF844,SRPK2,TNFAIP8,TNN,BANP,PADI2,SYCP2,POU2F3,RGS6,SRGAP3,ZNF525,BRPF3,RIMS2,PLD3,SIK3,CHRNA9,LRRC28,BCL11A,CCDC92,RANBP2,CLDN18,RNF128,NDRG3,RUBCN
GO:0050794	regulation of cellular process	9.6167812158 42384e-7	MEFV,BANK1,SRPK1,SYN3,CDK8,CBLL1,BCL2,ZNF239,XRN2,CNOT1,RICTOR,UNC13B,IL6R,SORBS2,RIC3,KCNK10,EXOC1,NPFFR2,AKIRIN1,ADCY3,TANK,GPR135,PLA2G4C,ENSG00000287286,SLC16A2,SULF2,TAF1D,GCH1,MELK,ZNF536,SRRD,KANK4,OR1B1,KIR2DL1,CBLN2,PPP1R17,ZFYVE9,SCTR,HNRNPH1,ALOX12,TMBIM1,BPIFB1,SLC31A1,TMEM182,MDM2,TDRD3,SMARCA4,SGTA,ADGRG2,AKAP3,SLC22A13,TBL1XR1,TNFSF8,FBLN2,C5AR2,PPP1R36,LITAFD,TXNRD1,TNFRSF21,MBTD1,PPP1CB,TRIOBP,RNF111,IST1,ATXN1L,TBC1D32,GNG7,PGR,MTERF3,MBP,DOK5,TET1,ZNF737,TMEM135,MED26,RFFL,ZNF821,BPI,LBH,SETD4,TWIST1,HORMAD1,UBR4,ARL6,RAP1GDS1,PALS1,CHPT1,ACTR3,AMBRA1,DSP,PTK2B,ABCG1,SCOC,CDK12,CAMSAP3,MYO6,KIFAP3,BORA,SLCO3A1,GRK3,TRAF3,CHD3,CD40,EFCAB7,ZMYND8,GSAP,SPICE1,PRMT2,GRIK4,ZNF765,GRHL2,POLA1,SMARCA5,ITGB6,NR1H2,ADAM9,MAD1L1,GARNL3,RNFT2,CASP7,UBE2B,MGMT,POLQ,ARHGAP23,SHMT1,PRICKLE2,SF1,ITGB3BP,WDR47,PKN2,OR4M2-OT1,ITGAM,PRDM11,FUT1,CC2D2A,TCP10L,MYRF,ORC2,OTX2,CCDC80,PRODH,DIAPH1,ADGRL3,PCM1,HELLS,IL6ST,PAK5,KIF18A,ANAPC5,KMT2A,ARHGAP44,MLF2,GFAP,DISP2,CAP2,PSMD1,CSNK1G3,WWTR1,COBL,ZNF607,RASAL2,NRCAM,HIPK2,ANKIB1,EGLN3,CLEC4A,ZNF28,WNT4,LPAR1,YTHDC1,ZKSCAN7,RASA3,IL7,C2CD4A,PNKD,OR4F6,TFIP11,GRAP2,RTKN2,DMP1,TLN1,SLC4A8,ZNF181,MTMR1,EPS8,ARHGAP21,CAND1,ZNF529,DMTN,RASGRF1,FRMD5,MCU,ALCAM,EDNRA,EFHC1,PTMS,HHLA2,RYR1,SLAMF1,ZNF728,AAMDC,PRIM2,MEGF10,GABBR1,TMEM25,ERCC1,FAM3B,ZNF254,COL5A2,GRIA1,IL13RA1,REPS2,ZNF468,AR

			MH4,ATXN2,NFATC3,SLC7A11,OR4D2,ABCA12,CD86,WWP1,POLR2M,COL8A1,SMAP1,RERE,ALS2,ECT2,GSG1L,HERC1,NR3C2,CRADD,OR4X2,ZC4H2,GAREM1,NXN,TSPAN12,NEK5,IL17RD,NAMPT,ELOA,TTC36,TNRC6A,STIM2,TTC8,WDR77,ZNF589,ZNF616,PLA2G7,TENT5B,RAB11FIP1,DPEP1,ZNF106,COL4A1,MYZAP,MX1,OR2A14,HSBP1L1,CD47,GRAP,CLDN1,LARP4,TSHR,LDOC1,ATP6V0E2,MTCH2,EPHB2,TEX15,PDLIM5,EYA2,ZNF652,PDE6A,PTEN,MAST2,SETD2,UTP4,VEPH1,CROCC,SOX21,LIN54,LRRC8C,ZNF197,ZNF397,TRPV6,MARK1,MBOAT1,CAMK2D,SERPINB7,IL31RA,ZNF407,OR2T27,MUC12,ST8SIA1,ASPA,ATG5,MIR922,STK32A,SPATA22,ZNF234,PAPPA2,MTERF4,TRERF1,NELFB,TBX15,EFHC2,SMYD2,NSUN2,PTPRK,PHACTR4,OR2M5,SYNDIG1,ASXL3,TRAPPC12,EBF4,SMURF1,LAMB1,BPTF,NFIB,UQCC2,LIFR,KCNAB2,ZNF844,SRPK2,TNFAIP8,TNN,BANP,PADI2,SYCP2,POU2F3,RGS6,SRGAP3,ZNF525,BRPF3,RIMS2,PLD3,SIK3,CHRNA9,LRRC28,BCL11A,RANBP2,CLDN18,RNF128,NDRG3,RUBCN
GO:0032501	multicellular organismal process	0.00010811006827754365	*MEFV,BANK1,BCL2,XRN2,CNOT1,RICTOR,UNC13B,IL6R,SORBS2,KCNK10,ADCY3,TANK,PLA2G4C,NUP133,SLC16A2,SULF2,GCH1,MEGF11,ZNF536,OR1B1,PCYT1B,CBLN2,PPP1R17,ZNG1A,SCTR,ALOX12,TMBIM1,EMB,SLC31A1,MDM2,SMARCA4,HS6ST1,AKAP3,TBL1XR1,TNFSF8,C5AR2,SCLT1,LITAFD,TXNRD1,TNFRSF21,MBTD1,TRIOBP,MTHFD1L,RNF111,IST1,ATXN1L,TBC1D32,GNG7,PGR,MBP,DOK5,TET1,NDUFA12,TMEM135,BPI,LBH,SETD4,TWIST1,HORMAD1,JHY,ARL6,RAP1GDS1,PALS1,AMBRA1,DSP,PTK2B,ABCG1,CAMSAP3,MYO6,KIFAP3,SLCO3A1,TRAF3,CD40,ZMYND8,GRHL2,POLA1,ITGB6,NR1H2,ADAM9,MAD1L1,CASP7,UBE2B,POLQ,GAPT,SF1,WDR47,PKN2,OR4M2-OT1,ITGAM,FUT1,CC2D2A,MYRF,OTX2,DIAPH1,ADGRL3,PCM1,HELLS,SHROOM3,IL6ST,PAK5,KIF18A,KMT2A,ARHGAP44,GFAP,WWTR1,COBL,RASAL2,NRCAM,HIPK2,RPGR,PCSK5,CLEC4A,WNT4,LPAR1,YTHDC1,IL7,C2CD4A,PNKD,OR4F6,DMP1,HSD17B2,HMGCS2,TLN1,SLC4A8,EPS8,DMTN,RASGRF1,KATNIP,ALCAM,EDNRA,EFHC1,HHLA2,RYR1,SLAMF1,MYH2,MEGF10,GABBR1,TMEM25,PPL,ERCC1,COL5A2,RAD54B,GRIA1,NFATC3,KCNMB3,SLC7A11,OR4D2,ABCA12,CD86,WWP1,COL8A1,SMAP1,RERE,ALS2,ECT2,HERC1,OR4X2,ZC4H2,NXN,TSPAN12,IL17RD,TTC36,TTC8,WDR77,XPNPEP3,PLA2G7,RAB11FIP1,COL4A1,OR2A14,CD47,GRAP,CLDN1,TSHR,LDOC1,EPHB2,PDLIM5,EYA2,PDE6A,PTEN,MAST2,SETD2,CROCC,SOX21,TRPV6,MARK1,MBOAT1,CAMK2D,SERPINB7,IL31RA,SUN1,OR2T27,ASPA,ATG5,SPATA22,PAPPA2,MTERF4,CRYBG3,TBX15,EFHC2,SMYD2,NSUN2,ZC3H13,PHACTR4,OR2M5,SYNDIG1,MYH15,SMURF1,LAMB1,BPTF,NFIB,CLCN5,SRPK2,TNN,PADI2,LNX2,SYCP2,TMEM163,BRPF3,RIMS2,PLD3,SLC5A1,SIK3,CHRNA9,BCL11A,CLDN18,RNF128*
GO:0032502	developmental process	0.00011530732729296356	*SRPK1,BCL2,XRN2,CNOT1,RICTOR,UNC13B,IL6R,ARMC6,SORBS2,AKIRIN1,ARHGEF26,NUP133,SULF2,MEGF11,MELK,ZNF536,PCYT1B,CBLN2,PPP1R17,ZNG1A,ALOX12,TMBIM1,EMB,SLC31A1,TMEM182,MDM2,SMARCA4,HS6ST1,ADGRG2,AKAP3,TBL1XR1,TNFSF8,SCLT1,TXNRD1,TNFRSF21,MBTD1,TRIOBP,MTHFD1L,RNF111,IST1,ATXN1L,TBC1D32,PGR,MBP,DOK5,TET1,TMEM135,LBH,TWIST1,HORMAD1,JHY,ARL6,PALS1,AM*

			BRA1,DSP,PTK2B,ABCG1,CDK12,CAMSAP3,MYO6,KIFAP3,CHD3,CD40,ZMYND8,PRMT2,GRHL2,ITGB6,NR1H2,ADAM9,MAD1L1,CASP7,TTC7A,UBE2B,POLQ,SF1,WDR47,ITGAM,FUT1,CC2D2A,MYRF,OTX2,DIAPH1,ADGRL3,PCM1,HELLS,SHROOM3,IL6ST,KIF18A,KMT2A,ARHGAP44,GFAP,CAP2,WWTR1,COBL,RASAL2,NRCAM,HIPK2,PCSK5,WNT4,LPAR1,YTHDC1,IL7,TFIP11,RTKN2,DMP1,HSD17B2,HMGCS2,EPS8,CAND1,DMTN,RASGRF1,MCU,ALCAM,EDNRA,EFHC1,RYR1,SLAMF1,AAMDC,MEGF10,GABBR1,PPL,ERCC1,CFAP44,COL5A2,GRIA1,NFATC3,SLC7A11,ABCA12,CD86,WWP1,COL8A1,SMAP1,RERE,ALS2,ECT2,HERC1,ZC4H2,NXN,TSPAN12,NEK5,IL17RD,TTC36,TNRC6A,TTC8,WDR77,COL4A1,CD47,CLDN1,LARP4,TSHR,LDOC1,MTCH2,EPHB2,TEX15,PDLIM5,EYA2,PDE6A,PTEN,MAST2,SETD2,SOX21,LRRC8C,MARK1,MBOAT1,SERPINB7,IL31RA,SUN1,ASPA,ATG5,SPATA22,PAPPA2,MTERF4,CRYBG3,NELFB,TBX15,EFHC2,SMYD2,NSUN2,ZC3H13,PHACTR4,SYNDIG1,ASXL3,MYH15,SMURF1,LAMB1,BPTF,H2BC1,NFIB,UQCC2,SRPK2,TNN,PADI2,LNX2,SYCP2,TMEM163,POU2F3,BRPF3,RIMS2,PLD3,SIK3,CHRNA9,TOPAZ1,MORN2,BCL11A,CLDN18,NDRG3,GALNTL5
GO:0048856	anatomical structure development	0.00031468372894005763	*SRPK1,BCL2,XRN2,CNOT1,RICTOR,IL6R,ARMC6,SORBS2,AKIRIN1,ARHGEF26,NUP133,SULF2,MEGF11,MELK,ZNF536,PCYT1B,CBLN2,PPP1R17,ZNG1A,ALOX12,TMBIM1,EMB,SLC31A1,TMEM182,MDM2,SMARCA4,HS6ST1,AKAP3,TBL1XR1,TNFSF8,SCLT1,TXNRD1,TNFRSF21,MBTD1,TRIOBP,MTHFD1L,RNF111,IST1,ATXN1L,TBC1D32,PGR,MBP,DOK5,TET1,TMEM135,LBH,TWIST1,HORMAD1,JHY,ARL6,PALS1,AMBRA1,DSP,PTK2B,CAMSAP3,MYO6,KIFAP3,CD40,ZMYND8,PRMT2,GRHL2,ITGB6,ADAM9,MAD1L1,CASP7,TTC7A,UBE2B,POLQ,SF1,WDR47,ITGAM,FUT1,CC2D2A,MYRF,OTX2,DIAPH1,ADGRL3,PCM1,HELLS,SHROOM3,IL6ST,KIF18A,KMT2A,ARHGAP44,GFAP,CAP2,WWTR1,COBL,RASAL2,NRCAM,HIPK2,PCSK5,WNT4,LPAR1,YTHDC1,IL7,TFIP11,RTKN2,DMP1,HSD17B2,HMGCS2,EPS8,DMTN,RASGRF1,MCU,ALCAM,EDNRA,EFHC1,RYR1,SLAMF1,MEGF10,PPL,ERCC1,CFAP44,COL5A2,GRIA1,NFATC3,SLC7A11,ABCA12,CD86,WWP1,COL8A1,SMAP1,RERE,ALS2,ECT2,HERC1,ZC4H2,NXN,TSPAN12,NEK5,IL17RD,TTC36,TNRC6A,TTC8,WDR77,COL4A1,CD47,CLDN1,LARP4,TSHR,LDOC1,MTCH2,EPHB2,PDLIM5,EYA2,PDE6A,PTEN,SETD2,SOX21,MARK1,MBOAT1,SERPINB7,IL31RA,SUN1,ASPA,ATG5,SPATA22,PAPPA2,MTERF4,CRYBG3,TBX15,EFHC2,SMYD2,NSUN2,PHACTR4,SYNDIG1,ASXL3,MYH15,SMURF1,LAMB1,BPTF,H2BC1,NFIB,UQCC2,SRPK2,TNN,PADI2,LNX2,SYCP2,TMEM163,POU2F3,BRPF3,RIMS2,PLD3,SIK3,CHRNA9,TOPAZ1,BCL11A,CLDN18,GALNTL5*
GO:0006996	organelle organization	0.0024108436264420993	*MSRB1,SRPK1,BCL2,CNOT1,RICTOR,SAMM50,UNC13B,SORBS2,PLA2G4C,NUP133,GCH1,KANK4,GOLGA8Q,EFL1,TDRD3,RMDN1,SMARCA4,SGTA,SCLT1,TRIOBP,IST1,RAB22A,TBC1D32,ESYT2,MTERF3,NDUFA12,BLZF1,TMEM135,HORMAD1,JHY,ARL6,RAP1GDS1,ACTR3,AMBRA1,DSP,PTK2B,CAMSAP3,MYO6,KIFAP3,BORA,CHD3,ZMYND8,SPICE1,SMARCA5,MAD1L1,TMED5,GOLGA8S,UBE2B,POLQ,SF1,ITGB3BP,WDR47,CC2D2A,COX18,NEMF,DIAPH1,PCM1,SHROOM3,PAK5,KIF18A,ANAPC5,ARHGAP44,GFAP,CAP2,WWTR1,COBL,HIPK2,RPGR,WNT4,LPAR1,TFIP11,TLN1,EPS8,ARHGAP21,DMTN*

			,FRMD5,MCU,ABRAXAS2,EDNRA,EFHC1,SLAMF1,GOLGA8N,PPL,POC1A,ATP8B4,ERCC1,CFAP44,RAD54B,ATXN2,POLR2M,ALS2,ECT2,TTC19,TTC36,TTC8,MYZAP,CD47,LARP4,MTCH2,EPHB2,TEX15,PDLIM5,EYA2,PTEN,MAST2,SETD2,CROCC,MIS18A,MARK1,SUN1,ATG5,SPATA22,MTERF4,SEC23A,PHACTR4,TRAPPC12,SMURF1,H2BC1,NFIB,UQCC2,GOLGA8H,SRPK2,SYCP2,SIK3,RUBCN
GO:0048513	animal organ development	0.005708148050758335	BCL2,XRN2,IL6R,SORBS2,AKIRIN1,NUP133,SULF2,MEGF11,PCYT1B,ZNG1A,ALOX12,TMEM182,MDM2,SMARCA4,HS6ST1,TBL1XR1,TRIOBP,MTHFD1L,ATXN1L,TBC1D32,PGR,MBP,TMEM135,LBH,TWIST1,JHY,PALS1,DSP,PTK2B,MYO6,GRHL2,ITGB6,ADAM9,MAD1L1,CASP7,SF1,WDR47,ITGAM,FUT1,CC2D2A,OTX2,PCM1,HELLS,KIF18A,KMT2A,WWTR1,COBL,RASAL2,NRCAM,HIPK2,PCSK5,WNT4,LPAR1,IL7,TFIP11,DMP1,HSD17B2,HMGCS2,EDNRA,EFHC1,RYR1,MEGF10,PPL,ERCC1,COL5A2,GRIA1,SLC7A11,ABCA12,COL8A1,RERE,HERC1,TSPAN12,IL17RD,TTC8,WDR77,COL4A1,CLDN1,TSHR,LDOC1,EPHB2,PDLIM5,PDE6A,PTEN,SETD2,SOX21,MARK1,SERPINB7,SUN1,ATG5,PAPPA2,MTERF4,CRYBG3,TBX15,SMYD2,NSUN2,PHACTR4,ASXL3,MYH15,LAMB1,BPTF,NFIB,UQCC2,TNN,PADI2,SYCP2,POU2F3,SIK3,CHRNA9,CLDN18
GO:0030154	cell differentiation	0.0061577638797422046	SRPK1,BCL2,XRN2,CNOT1,IL6R,ARMC6,SORBS2,AKIRIN1,ARHGEF26,NUP133,SULF2,MELK,ZNF536,EMB,TMEM182,MDM2,SMARCA4,HS6ST1,TBL1XR1,TNFSF8,SCLT1,TNFRSF21,TRIOBP,IST1,ATXN1L,PGR,DOK5,TET1,LBH,TWIST1,HORMAD1,JHY,PALS1,AMBRA1,DSP,PTK2B,ABCG1,CDK12,CAMSAP3,MYO6,KIFAP3,CHD3,ZMYND8,PRMT2,GRHL2,ITGB6,NR1H2,ADAM9,CASP7,TTC7A,UBE2B,SF1,WDR47,ITGAM,MYRF,OTX2,ADGRL3,PCM1,IL6ST,KMT2A,ARHGAP44,GFAP,WWTR1,COBL,NRCAM,HIPK2,WNT4,LPAR1,YTHDC1,IL7,RTKN2,CAND1,DMTN,RASGRF1,ALCAM,EDNRA,RYR1,SLAMF1,AAMDC,MEGF10,GABBR1,PPL,ERCC1,CFAP44,COL5A2,NFATC3,SLC7A11,ABCA12,CD86,COL8A1,SMAP1,RERE,ALS2,ECT2,HERC1,ZC4H2,NXN,NEK5,IL17RD,TTC36,TTC8,WDR77,COL4A1,CLDN1,TSHR,MTCH2,EPHB2,TEX15,PDLIM5,EYA2,PTEN,MAST2,SETD2,SOX21,LRRC8C,MARK1,MBOAT1,IL31RA,SUN1,ASPA,ATG5,NELFB,TBX15,EFHC2,NSUN2,PHACTR4,SMURF1,LAMB1,H2BC1,NFIB,UQCC2,SRPK2,TNN,LNX2,SYCP2,POU2F3,BRPF3,RIMS2,PLD3,TOPAZ1,MORN2,BCL11A,CLDN18,NDRG3,GALNTL5
GO:0048869	cellular developmental process	0.006239178979319561	SRPK1,BCL2,XRN2,CNOT1,IL6R,ARMC6,SORBS2,AKIRIN1,ARHGEF26,NUP133,SULF2,MELK,ZNF536,EMB,TMEM182,MDM2,SMARCA4,HS6ST1,TBL1XR1,TNFSF8,SCLT1,TNFRSF21,TRIOBP,IST1,ATXN1L,PGR,DOK5,TET1,LBH,TWIST1,HORMAD1,JHY,PALS1,AMBRA1,DSP,PTK2B,ABCG1,CDK12,CAMSAP3,MYO6,KIFAP3,CHD3,ZMYND8,PRMT2,GRHL2,ITGB6,NR1H2,ADAM9,CASP7,TTC7A,UBE2B,SF1,WDR47,ITGAM,MYRF,OTX2,ADGRL3,PCM1,IL6ST,KMT2A,ARHGAP44,GFAP,WWTR1,COBL,NRCAM,HIPK2,WNT4,LPAR1,YTHDC1,IL7,RTKN2,CAND1,DMTN,RASGRF1,ALCAM,EDNRA,RYR1,SLAMF1,AAMDC,MEGF10,GABBR1,PPL,ERCC1,CFAP44,COL5A2,NFATC3,SLC7A11,ABCA12,CD86,COL8A1,SMAP1,RERE,ALS2,ECT2,HERC1,ZC4H2,NXN,NEK5,IL17RD,TTC36,TTC8,WDR77,COL4A1,CLDN1,TSHR,MTCH2,EPHB2,TEX15,PDLIM5,EYA2,PTEN,MAST2,SETD2,SOX21,

			LRRC8C,MARK1,MBOAT1,IL31RA,SUN1,ASPA,ATG5,NELFB,TBX15,EFHC2,NSUN2,PHACTR4,SMURF1,LAMB1,H2BC1,NFIB,UQCC2,SRPK2,TNN,LNX2,SYCP2,POU2F3,BRPF3,RIMS2,PLD3,TOPAZ1,MORN2,BCL11A,CLDN18,NDRG3,GALNTL5
GO:0007275	multicellular organism development	0.01148978096681521	*BCL2,XRN2,CNOT1,RICTOR,IL6R,SORBS2,NUP133,SULF2,MEGF11,ZNF536,PCYT1B,CBLN2,PPP1R17,ZNG1A,EMB,SLC31A1,MDM2,SMARCA4,HS6ST1,AKAP3,TBL1XR1,SCLT1,TXNRD1,TNFRSF21,MBTD1,TRIOBP,MTHFD1L,RNF111,IST1,ATXN1L,TBC1D32,PGR,MBP,DOK5,TET1,TMEM135,TWIST1,HORMAD1,JHY,ARL6,PALS1,AMBRA1,DSP,PTK2B,CAMSAP3,MYO6,KIFAP3,CD40,ZMYND8,GRHL2,ITGB6,MAD1L1,CASP7,UBE2B,POLQ,SF1,WDR47,ITGAM,FUT1,CC2D2A,MYRF,OTX2,ADGRL3,PCM1,HELLS,SHROOM3,IL6ST,KIF18A,KMT2A,ARHGAP44,GFAP,WWTR1,COBL,NRCAM,HIPK2,PCSK5,WNT4,LPAR1,YTHDC1,IL7,DMP1,HSD17B2,HMGCS2,RASGRF1,ALCAM,EDNRA,EFHC1,RYR1,ERCC1,COL5A2,GRIA1,NFATC3,SLC7A11,ABCA12,CD86,WWP1,COL8A1,SMAP1,RERE,ALS2,ECT2,HERC1,ZC4H2,NXN,TSPAN12,TTC36,TTC8,WDR77,COL4A1,CD47,CLDN1,TSHR,EPHB2,PDLIM5,EYA2,PDE6A,PTEN,SETD2,MARK1,MBOAT1,SERPINB7,SUN1,ASPA,ATG5,SPATA22,PAPPA2,MTERF4,CRYBG3,TBX15,EFHC2,SMYD2,NSUN2,PHACTR4,SYNDIG1,MYH15,SMURF1,LAMB1,BPTF,NFIB,SRPK2,TNN,PADI2,LNX2,SYCP2,TMEM163,BRPF3,RIMS2,SIK3,CHRNA9,BCL11A,CLDN18*
		CC	
GO:0016020	membrane	0.00003555203507333685	*SMIM17,SRPK1,SYN3,TMEM266,BCL2,XRN2,CNOT1,SAMM50,UNC13B,IL6R,SORBS2,RIC3,KCNK10,EXOC1,NPFFR2,SLCO1B3-SLCO1B7,AKIRIN1,ADCY3,CPD,GPR135,PLA2G4C,NUP133,SLC16A2,SULF2,GCH1,ALG9,MEGF11,MELK,PCNX2,OR1B1,PCYT1B,KIR2DL1,ZFYVE9,SCTR,AMPH,HNRNPH1,ALOX12,TMBIM1,EMB,SLC35F5,SLC31A1,TMEM182,GOLGA8Q,MDM2,IZUMO1,ITPRID2,SMARCA4,HS6ST1,SGTA,ADGRG2,SLC22A13,TNFSF8,C5AR2,SCLT1,LITAFD,TNFRSF21,MTHFD1L,RAB22A,ENSG00000287725,ESYT2,GNG7,PGR,POTEM,CHIC1,MTERF3,MBP,TANGO6,NDUFA12,BLZF1,TMEM135,RFFL,ST3GAL5,TMEM132D,SUCLG2,BPI,UBR4,KTN1,UGT2B28,ARL6,PALS1,CHPT1,ACTR3,C8A,IGHV3-72,AMBRA1,DSP,PTK2B,ECPAS,ABCG1,SCOC,MYO6,KIFAP3,SLCO3A1,GRK3,TRAF3,CD40,EFCAB7,ATP6V1G3,TMCC2,GRIK4,GRHL2,ITGB6,ADAM9,TMED5,RNFT2,SLC26A11,TTC7A,GOLGA8S,UBE2B,MGMT,ENSG00000289723,PRICKLE2,GAPT,ITGB3BP,PKN2,OR4M2-OT1,ITGAM,FUT1,MYRF,ORC2,COX18,TMCO4,CCDC80,PRODH,DIAPH1,ADGRL3,PCM1,SHROOM3,TM2D2,IL6ST,TMEM177,PAK5,KIF18A,ARHGAP44,MLF2,GFAP,DISP2,CAP2,PSMD1,CSNK1G3,WWTR1,COBL,NRCAM,PCSK5,CLEC4A,WNT4,LPAR1,YTHDC1,RASA3,PNKD,SLC25A53,OR4F6,GRAP2,RTKN2,HSD17B2,TLN1,SLC4A8,MTMR1,EPS8,ARHGAP21,CAND1,DMTN,RASGRF1,FRMD5,MCU,ALCAM,MPP4,EDNRA,HHLA2,RYR1,SLAMF1,SLC25A21,ABCC11,GOLGA8N,DDX24,MEGF10,GABBR1,TMEM25,PPL,ANKRD20A1,TMEM212,ATP8B4,MCFD2,GRIA1,IL13RA1,REPS2,ARMH4,ATXN2,KCNMB3,GXYLT2,SLC7A11,ALG11,MEP1A,OR4D2,ABCA12,CD86,WWP1,SMAP1,ECT2,MTARC2,GSG1L,HERC1,NR3C2,OR4X2,ZC4H2,TTC19,SLC27A6,GAREM1,TSPAN12,IL17RD,*

			DMXL2,STIM2,TTC8,CYP3A43,FKBP15,OSBPL9,RAB11FIP1,DPEP1,ZNF106,COL4A1,MYZAP,MX1,OR2A14,CD47,GRAP,CLDN1,LARP4,TSHR,ATP6V0E2,MTCH2,EPHB2,MEP1B,SLC17A5,PDLIM5,PDE6A,PTEN,MAST2,VEPH1,CROCC,LRRC8C,ZNF397,TRPV6,MARK1,PLSCR2,MBOAT1,ATP13A3,CAMK2D,IL31RA,SUN1,OR2T27,SLC9A7,MUC12,ST8SIA1,ATG5,STK32A,KCNC3,PAPPA2,SLC12A9,COL25A1,NIPAL2,GARIN2,CPPED1,SEC23A,PTPRK,LETM2,OR2M5,SYNDIG1,CYP4X1,SMURF1,UQCC2,LIFR,KCNAB2,GOLGA8H,CLCN5,LNX2,TMEM163,POU2F3,RGS6,GXYLT1,RIMS2,TMEM258,PLD3,SLC5A1,CDCP1,IGHV3OR16-12,CHRNA9,RANBP2,CLDN18,RNF128,LRRC3B,GALNTL5
GO:0005886	plasma membrane	0.0010615793837060 22	*SRPK1,TMEM266,UNC13B,IL6R,SORBS2,KCNK10,EXOC1,NPFFR2,SLCO1B3-SLCO1B7,ADCY3,CPD,GPR135,PLA2G4C,SLC16A2,SULF2,MEGF11,MELK,OR1B1,KIR2DL1,SCTR,AMPH,ALOX12,TMBIM1,EMB,SLC31A1,TMEM182,MDM2,IZUMO1,ITPRID2,HS6ST1,ADGRG2,SLC22A13,TNFSF8,C5AR2,TNFRSF21,RAB22A,ESYT2,GNG7,PGR,CHIC1,MBP,RFFL,SUCLG2,UBR4,KTN1,ARL6,PALS1,C8A,IGHV3-72,DSP,PTK2B,ABCG1,MYO6,KIFAP3,SLCO3A1,GRK3,TRAF3,CD40,EFCAB7,ATP6V1G3,GRIK4,ITGB6,ADAM9,SLC26A11,TTC7A,UBE2B,ENSG00000289723,GAPT,PKN2,OR4M2-OT1,ITGAM,FUT1,DIAPH1,ADGRL3,SHROOM3,IL6ST,PAK5,KIF18A,ARHGAP44,DISP2,CAP2,CSNK1G3,WWTR1,COBL,NRCAM,CLEC4A,WNT4,LPAR1,YTHDC1,RASA3,OR4F6,GRAP2,RTKN2,TLN1,SLC4A8,MTMR1,EPS8,ARHGAP21,DMTN,RASGRF1,ALCAM,MPP4,EDNRA,HHLA2,RYR1,SLAMF1,ABCC11,MEGF10,GABBR1,TMEM25,PPL,ANKRD20A1,ATP8B4,GRIA1,IL13RA1,REPS2,KCNMB3,SLC7A11,MEP1A,OR4D2,ABCA12,CD86,WWP1,SMAP1,ECT2,GSG1L,OR4X2,ZC4H2,SLC27A6,GAREM1,TSPAN12,IL17RD,STIM2,TTC8,DPEP1,MYZAP,OR2A14,CD47,GRAP,CLDN1,TSHR,EPHB2,MEP1B,SLC17A5,PDE6A,PTEN,MAST2,VEPH1,CROCC,LRRC8C,ZNF397,TRPV6,MARK1,PLSCR2,CAMK2D,IL31RA,OR2T27,SLC9A7,MUC12,STK32A,KCNC3,PAPPA2,SLC12A9,COL25A1,CPPED1,PTPRK,OR2M5,SYNDIG1,SMURF1,LIFR,KCNAB2,CLCN5,LNX2,TMEM163,POU2F3,RGS6,RIMS2,SLC5A1,CDCP1,IGHV3OR16-12,CHRNA9,CLDN18*
GO:0071944	cell periphery	0.0012631398061157 78	*SRPK1,TMEM266,UNC13B,IL6R,SORBS2,KCNK10,EXOC1,NPFFR2,SLCO1B3-SLCO1B7,ADCY3,CPD,GPR135,PLA2G4C,SLC16A2,SULF2,MEGF11,MELK,OR1B1,KIR2DL1,SCTR,AMPH,ALOX12,TMBIM1,EMB,SLC31A1,TMEM182,MDM2,IZUMO1,ITPRID2,HS6ST1,ADGRG2,SLC22A13,TNFSF8,FBLN2,C5AR2,TNFRSF21,RAB22A,ESYT2,GNG7,PGR,CHIC1,MBP,RFFL,SUCLG2,UBR4,KTN1,ARL6,PALS1,C8A,IGHV3-72,DSP,PTK2B,ABCG1,MYO6,KIFAP3,SLCO3A1,GRK3,TRAF3,CD40,EFCAB7,ATP6V1G3,GRIK4,ITGB6,ADAM9,SLC26A11,TTC7A,UBE2B,ENSG00000289723,GAPT,PKN2,OR4M2-OT1,ITGAM,FUT1,CCDC80,DIAPH1,ADGRL3,SHROOM3,IL6ST,PAK5,KIF18A,ARHGAP44,DISP2,CAP2,CSNK1G3,WWTR1,COBL,NRCAM,CLEC4A,WNT4,LPAR1,YTHDC1,RASA3,IL7,OR4F6,TFIP11,GRAP2,RTKN2,DMP1,TLN1,SLC4A8,MTMR1,EPS8,ARHGAP21,DMTN,RASGRF*

			1,ALCAM,MPP4,EDNRA,HHLA2,RYR1,SLAMF1,ABCC11,MEGF10,GABBR1,TMEM25,PPL,ANKRD20A1,ATP8B4,COL5A2,GRIA1,IL13RA1,REPS2,KCNMB3,SLC7A11,MEP1A,OR4D2,ABCA12,CD86,WWP1,COL8A1,SMAP1,ECT2,GSG1L,OR4X2,ZC4H2,SLC27A6,GAREM1,TSPAN12,IL17RD,STIM2,TTC8,DPEP1,COL4A1,MYZAP,OR2A14,CD47,GRAP,CLDN1,TSHR,EPHB2,MEP1B,SLC17A5,PDE6A,PTEN,MAST2,VEPH1,CROCC,LRRC8C,ZNF397,TRPV6,MARK1,PLSCR2,CAMK2D,IL31RA,OR2T27,SLC9A7,MUC12,STK32A,KCNC3,PAPPA2,SLC12A9,COL25A1,CPPED1,PTPRK,OR2M5,SYNDIG1,SMURF1,LAMB1,LIFR,KCNAB2,CLCN5,TNN,LNX2,TMEM163,POU2F3,RGS6,RIMS2,SLC5A1,CDCP1,IGHV3OR16-12,CHRNA9,PDZD4,CLDN18,LRRC3B
GO:0005737	cytoplasm	0.00773803738238375	*MSRB1,BBOX1,MEFV,BANK1,SRPK1,SYN3,TMEM266,PABPC5,CBLL1,BCL2,CNOT1,RICTOR,SAMM50,UNC13B,ARMC6,HK3,SORBS2,DYRK4,RIC3,EXOC1,ETFA,SLCO1B3-SLCO1B7,ADCY3,ARHGEF26,TANK,GPR135,PLA2G4C,NUP133,SULF2,SMS,TAF1D,GCH1,ALG9,MELK,SRRD,KANK4,PCYT1B,ZFYVE9,ZNG1A,ACSS3,SCTR,AMPH,HNRNPH1,ALOX12,TMBIM1,SLC31A1,GOLGA8Q,EFL1,MDM2,TDRD3,IZUMO1,ITPRID2,CFAP210,RMDN1,HS6ST1,SGTA,ADGRG2,AKAP3,SLC22A13,SCLT1,LITAFD,TXNRD1,PPP1CB,TRIOBP,MTHFD1L,RNF111,IST1,RAB22A,ENSG00000287725,TBC1D32,ESYT2,PGR,CHIC1,MTERF3,MBP,DOK5,NDUFA12,ELMOD3,BLZF1,TMEM135,RIMKLA,RFFL,ST3GAL5,SUCLG2,BPI,LBH,SETD4,UBR4,KTN1,GULP1,UGT2B28,ARL6,RAP1GDS1,PALS1,CHPT1,ACTR3,AMBRA1,DSP,PTK2B,DERA,ECPAS,ABCG1,SCOC,CAMSAP3,MYO6,KIFAP3,BORA,PRTFDC1,GRK3,TRAF3,CHD3,ZMYND8,GSAP,SPICE1,PRMT2,ATP6V1G3,TMCC2,RASEF,POLA1,NR1H2,MAD1L1,GARNL3,TMED5,DCAF8L2,CASP7,SLC26A11,TTC7A,GOLGA8S,UBE2B,POLQ,ARHGAP23,SHMT1,PRICKLE2,GAPT,ITGB3BP,WDR47,PKN2,ITGAM,PRDM11,FUT1,CC2D2A,MYRF,TGM6,COX18,NEMF,PRODH,DIAPH1,PCM1,SHROOM3,TMEM177,PAK5,KIF18A,ANAPC5,KMT2A,ARHGAP44,MLF2,GFAP,CAP2,PSMD1,CSNK1G3,SRFBP1,WWTR1,COBL,RASAL2,HIPK2,ANKIB1,RPGR,PCSK5,EGLN3,WNT4,LPAR1,AOAH,RASA3,PNKD,SLC25A53,TFIP11,GRAP2,RTKN2,DMP1,HSD17B2,HMGCS2,TLN1,SLC4A8,MTMR1,EPS8,ARHGAP21,CAND1,DMTN,RASGRF1,KATNIP,MCU,MPP4,ABRAXAS2,EFHC1,RYR1,SLAMF1,SLC25A21,ABCC11,MYH2,AAMDC,GOLGA8N,EML5,GABBR1,TMEM25,PPL,POC1A,ATP8B4,ERCC1,CFAP44,COL5A2,FNTB,TEX9,MCFD2,GRIA1,REPS2,ATXN2,DENND2A,NFATC3,ALG11,ABCA12,WWP1,POLR2M,COL8A1,SMAP1,ALS2,ECT2,MTARC2,HERC1,NR3C2,CRADD,ZC4H2,TTC19,NXN,IL17RD,NAMPT,TNRC6A,DMXL2,STIM2,TTC8,WDR77,CYP3A43,XPNPEP3,FKBP15,TENT5B,OSBPL9,RAB11FIP1,ZNF106,COL4A1,MYZAP,MX1,HSBP1L1,CD47,GRAP,LARP4,ATP6V0E2,MTCH2,EPHB2,SLC17A5,TEX15,PDLIM5,EYA2,PTEN,MAST2,SETD2,CA13,CROCC,LRRC8C,ZNF397,MIS18A,MARK1,PLSCR2,MBOAT1,ATP13A3,CAMK2D,SERPINB7,PPIG,SUN1,SLC9A7,MUC12,ST8SIA1,ASPA,ATG5,KCNC3,NAP1L4,MTERF4,TRERF1,NELFB,COL25A1,LRRC9,CPPED1,EFHC2,SMYD2,SEC23A,NSUN2,PHACTR4,LETM2,RGPD4,SYNDIG1,MYH15,CYP4X1,TRAPPC12,SMURF1,LAMB1,BPTF,UQCC2,KCNAB2,GOLGA8H,CLCN5,SRPK2,TNFA*

			IP8,ANKRD50,PADI2,TMEM163,RAVER2,POU2F3,RGS6,SRGAP3,BRPF3,RIMS2,TMEM258,PLD3,SLC5A1,SIK3,TOPAZ1,MORN2,BCL11A,PDZD4,NAA25,FKBP9,CCDC92,RANBP2,FAM110B,RNF128,NDRG3,RUBCN,GALNTL5
GO:0042995	cell projection	0.008051545891977768	*MEFV,TMEM266,UNC13B,SORBS2,RIC3,ADCY3,ARHGEF26,GCH1,EMB,CFAP210,AKAP3,SCLT1,TNFRSF21,TRIOBP,RAB22A,ATXN1L,TBC1D32,MBP,ELMOD3,ARL6,PALS1,ACTR3,AMBRA1,PTK2B,CAMSAP3,MYO6,KIFAP3,GRK3,CD40,EFCAB7,ZMYND8,WDR47,PKN2,CC2D2A,OTX2,DIAPH1,ADGRL3,PCM1,IL6ST,KIF18A,ARHGAP44,GFAP,COBL,NRCAM,RPGR,LPAR1,TLN1,SLC4A8,EPS8,DMTN,RASGRF1,KATNIP,ALCAM,EFHC1,MEGF10,GABBR1,POC1A,CFAP44,GRIA1,SLC7A11,ALS2,TTC8,FKBP15,DPEP1,EPHB2,PDLIM5,PDE6A,PTEN,CROCC,MARK1,CAMK2D,IL31RA,ATG5,KCNC3,GARIN2,EFHC2,PHACTR4,SYNDIG1,SMURF1,BPTF,NFIB,KCNAB2,TNN,RGS6,RIMS2,SLC5A1,CHRNA9*
GO:0098590	plasma membrane region	0.012299406039752865	*UNC13B,IL6R,SORBS2,ADCY3,SLC16A2,MEGF11,SLC31A1,ADGRG2,SLC22A13,C5AR2,ARL6,PALS1,DSP,MYO6,KIFAP3,SLCO3A1,EFCAB7,GRIK4,ADAM9,SLC26A11,PKN2,ITGAM,DIAPH1,SHROOM3,KIF18A,NRCAM,LPAR1,TLN1,SLC4A8,EPS8,DMTN,ABCC11,GABBR1,GRIA1,SLC7A11,ECT2,GSG1L,ZC4H2,TTC8,DPEP1,CLDN1,TSHR,EPHB2,SLC17A5,PDE6A,PTEN,IL31RA,KCNC3,PAPPA2,SYNDIG1,RIMS2,SLC5A1,CHRNA9*
GO:0120025	plasma membrane bounded cell projection	0.021041008870979643	*MEFV,TMEM266,UNC13B,SORBS2,RIC3,ADCY3,ARHGEF26,GCH1,EMB,CFAP210,AKAP3,SCLT1,TNFRSF21,TRIOBP,RAB22A,ATXN1L,TBC1D32,MBP,ELMOD3,ARL6,PALS1,ACTR3,AMBRA1,PTK2B,CAMSAP3,MYO6,KIFAP3,CD40,EFCAB7,ZMYND8,WDR47,PKN2,CC2D2A,OTX2,DIAPH1,ADGRL3,PCM1,IL6ST,KIF18A,ARHGAP44,GFAP,COBL,NRCAM,RPGR,LPAR1,TLN1,SLC4A8,EPS8,DMTN,RASGRF1,ALCAM,EFHC1,GABBR1,POC1A,CFAP44,GRIA1,SLC7A11,ALS2,TTC8,FKBP15,DPEP1,EPHB2,PDE6A,PTEN,CROCC,MARK1,CAMK2D,IL31RA,ATG5,KCNC3,GARIN2,EFHC2,PHACTR4,SYNDIG1,SMURF1,BPTF,NFIB,KCNAB2,TNN,RGS6,SLC5A1,CHRNA9*

Table S15. GO associations with biological processes (BP), molecular functions (MF), and cellular components (CC) of 2240 genes that exhibited a decrease in the number of contacts with rDNA clusters while growing on Matrigel. Related to Figure 6C.

GO.ID	Description	padj	Genes
		MF	
GO:0005515	protein binding	1.4429137656352487e-12	*FSTL1,C10ORF90,APBB2,PFKP,ESYT1,STYK1,RPL19,RTN1,CDK14,EPN2,MSRB3,PDE1C,MREG,NOSTRIN,ING3,MAP3K3,NINL,SLC12A8,OSBPL8,LRRN1,NFIA,POTED,STX8,PHF20,FOXK2,CPNE4,RBM17,SMYD3,GARS1,RAPGEF6,ARHGEF9,TAFA2,ABR,MAOA,BLTP3B,TRAPPC9,SLC16A5,FNDC1,PREP,MAP3K13,EIF1AX,PPM1A,ARL13B,SAMSN1,THADA,LIMD1,TF,THRB,MYBPC1,USP42,IPP,RCSD1,SPTBN1,CDC42EP3,MYO5C,MAPK10,CACNB1,ITPRID1,USP32,AHCYL2,DAPP1,ATF7,GNG12,FGF1,CHRNB2,SEZ6,CLDN6,KCNQ5,CDH8,GNB4,AGTPBP1,FHL1,TRMT6,PPP1R3*

			7,RBPMS,ATP10A,SH3PXD2A,SPINK5,SKAP2,PRND,ATP7B,GPBP1L1,PPM1H,KCNJ6,BRD4,DOK4,ZAN,CAMK2B,GRIP1,TLK1,GPATCH8,DDI2,PGM1,DCT,DLGAP1,PECR,SLC38A9,ZNF264,HBP1,SDR16C5,ABCG8,RGPD2,XPO4,OTUD7A,KALRN,SUMF1,USH2A,MAP4K3,FPGT-TNNI3K,NOTCH2,CD93,FKBP5,TMOD2,SLC44A5,SDK2,HMBOX1,NPNT,DSC1,EPB41L3,MAP6,PARVB,CDH11,SHANK3,WDR26,NR4A3,SPTLC3,ARID1B,E2F7,SLC14A2,FOXO1,STYXL1,STK39,SMURF2,SRL,ADAMTS12,DAAM2,TBC1D16,PLXNA4,CDK18,YBX1,STK24,ARMT1,CFAP70,DCDC1,CACNA1C,FAM20B,FAM3C,ASB4,ARB2A,PRKN,NLGN4X,EXOC4,RMND5A,F13A1,PIK3C2B,XPO6,KDM7A,SLC44A3,FAM83B,IFT172,CHD7,GPSM1,CBLL2,CDON,NLK,THSD7A,GNAI1,MRPL13,MAPKAPK3,NCAM2,TIAM2,ZNF10,TRAK2,CNMD,LRP8,TAGLN3,CNTN1,AGL,MEOX2,STAT5B,ARHGEF4,UBE2O,MUC17,CPEB4,EIF4ENIF1,HEATR9,CALCR,ELMO1,ERBIN,ZNF121,MBTPS2,RGS22,CD247,MCC,CEP192,PTH,ADAR,DNAH10,PDE10A,TOX2,PDHX,CLCN3,RHOJ,CDC37,FGF10,ZFYVE16,MECOM,COL4A6,TSPAN18,SAMD15,NLRP8,PLG,NEURL4,HERC5,CDH7,UBE2E2,NCK1,CD84,FAM168A,NONO,RARRES1,KLHL42,NREP,EXO1,GABRA5,EBNA1BP2,KCNQ3,MTCP1,SH3BGR,CHRM3,KDM2B,PREX1,RGPD3,AIG1,ERICH1,ZNF404,PMS1,WRNIP1,COL24A1,ANO3,GOLGA8R,RSF1,KCTD3,NCOA7,C2ORF88,SEPTIN10,CIDEB,MARCHF1,TTC33,MCPH1,MYO1D,SESTD1,DNAAF9,BRAF,COL1A2,DTNA,TMOD3,GEMIN5,EEF2KMT,MRTFB,XPO7,KCNS3,COL12A1,ABLIM1,RBMS1,CHRNA7,HECW1,HMGB1,ZNF304,DYNLT2B,CDIP1,RFLNA,LPP,HIVEP1,ATRX,TGFB2,MDFIC,EPB41L4A,LAMA2,ADD3,TRIO,ATXN7L1,FBXO16,ZNF85,HOXC13,SMARCC1,RAD51B,NDST1,MUSK,KCNJ18,JAM3,KCNIP3,SFPQ,MAP3K5,ALKBH1,ARPP21,RGS3,NUP93,RERG,PRKAR2A,TBC1D5,CTIF,CFAP57,ATP11A,RBM23,CSTF3,SLC15A4,PCDHB8,GOLPH3,PRKG2,MBTPS1,NCF2,PIK3C3,PIGV,USP10,SND1,HMCN1,IGFBP7,RSKR,EPHA5,SKOR2,CBR4,RUNX1,ENAH,SNX10,TMEM230,PPTC7,STON2,FAM171A1,STRADA,PCLO,HUWE1,ZNF595,ASPH,RRAS2,SH3BGRL,ZDHHC4,UBR5,GTF2F2,SIM1,KIAA1217,ABTB2,SOX30,ANKS1B,CHL1,SPOCK3,GSN,PLS1,TCP11L1,SPON1,MAPRE3,ANO2,TLR8,HPS3,KIF21A,SLIT3,ARMH3,CACUL1,NLRP7,ARHGEF39,PHC2,ROBO1,ZNF578,ANKRD11,ITPR1,PKP4,DNAAF11,PAK3,NDUFAF7,ABI3BP,KLF12,IPMK,TMEM183A,CAMK1G,PRUNE2,DOK6,PRKAG2,MLLT3,CCNT2,BTG4,HAVCR1,TRAPPC10,CPQ,CNOT10,MR1,KRTAP21-2,F8,TNFRSF12A,OASL,CPT2,VPS8,ISY1,CXCR5,NCKAP1,PRPF40A,CLDN12,CNTN3,MGAM,CILK1,FOSL2,PACRG,ADGRD1,EPHA3,CHCHD3,LATS2,TYW3,MYH14,SPDYE2B,MRPL32,TPRG1,KLHL33,ERP29,CDYL2,LRRTM4,ARHGAP18,UVRAG,MYOM2,LYN,ADGRB3,USP6,SUMO2,MTA3,UBL3,DIP2B,NME7,SEC24C,LARGE1,ATP6V1H,NUP58,FIGNL1,PUS7,PDZD8,CCDC33,RRM2,CRYBB1

			,SIL1,LDB2,SPTB,UNC93A,C21ORF62,ALDH1A1,RNASEL,NAV1,DBF4B,PAQR5,CORIN,TMCC1,LRRC7,DCP2,PDZRN4,SGMS2,SLC41A2,NOS3,KCNIP4,VSX1,EIF3L,NOMO2,TNRC6C,MLLT10,BPHL,ERCC6,NOS1AP,EIF2AK4,KIAA1614,MSRA,SYNPR,MAGEL2,MATN2,TGFBR2,CMKLR2,ITGA11,CFAP77,MARK4,MDN1,SYNJ1,TTC6,TCF7L2,CHAF1B,KIAA1328,ZNF462,CRTAC1,E2F6,ZFHX3,POLR2C,UNC119,SHISA2,GPM6B,RRAGD,C2ORF50,ULK3,SPDYE2,ZMAT4,GKAP1,LHFPL3,RGPD1,PPP2R5E,KPNB1,GSDMD,ADGRV1,RASGRF2,ST7,SERBP1,QTRT2,EPB41L5,GAD1,SCG5,NUDCD1,WAC,WHRN,OCA2,DAPK1,TDP2,ACSM2B,PRUNE1,SAR1B,TTC7B,INSYN2A,BTG3,APP,GATA4,ATF7IP2,SMAD5,IGF2BP1,PPIL6,RNLS,GSKIP,RNF38,ADAM12,UBE2G1,PELI2,SP140L,HCN1,CHRM5,PI4KB,RHOT1,ELOVL5,HERC2,HOOK3,USE1,PALLD,TLR1,CECR2,RIPK4,AVPR1B,IQCM,PTK2,USP7,TICAM2,SCP2,SH2B2,ATP1B4,ZNF41,AVEN,CELF1,HYCC1,DNAH12,POTEG,POGZ,PIP4K2B,PRRC2B,UQCC1,LRRK2,IKZF3,SLC38A4,CLSTN2,ARIH1,CFAP298,PLD1,ITFG2,VTCN1,PKHD1,TENT5C,UBXN7,RARB,PJA2,TCF4,MGST1,RBM44,ARHGEF38,PAEP,COP1,TIAM1,CSRP3,PHACTR1,MLIP,IQSEC2,ACVR2A,GNAO1,ASAP2,TIPIN,RASSF4,C6ORF58,SNTG2,NTM,MEIS1,AMMECR1,NEDD4L,BRCC3,DUSP14,PRMT8,RABGEF1,KIT,TBC1D8,EPHA7,SPAG9,OSBPL3,ANTXR1,C19ORF47,SPDL1,WNT5A,TRABD2B,WIPI2,PEX26,ARFGEF2,CTNNB1,ANKRD17,RUNX1T1,CTSK,PRKDC,AGAP4,IPO5,ESR1,TNFRSF19,ZNF479,PTPN14,SCFD1,ITGB8,ERC2,DMGDH,TRANK1,BCAR3,TPD52L1,L3MBTL4,PPM1E,ZXDC,VPS53,LMX1A,DCAF8L1,WWOX,TPST2,FIGN,BTBD8,PARS2,BNIPL,HUNK,ANAPC10,FBXO15,PTGER4,BBX,VWC2L,BEST3,DOCK1,HDDC2,DIAPH3,CCDC107,GPC4,BPGM,PKIG,EFNA5,IGF2BP2,ACTR3B,CDC14B,SYNE3,HECTD1,VKORC1L1,NGEF,TRPC5,NEUROD2,FNIP2,AASS,JAML,KAT14,UGGT2,UBE3B,TNFSF11,PTPRF,IKBKB,FXR2,SH3BP5,AKAP6,USF3,LYVE1,APOH,ARHGEF28,CLCN1,RAB3GAP2,TASOR,STEAP1B,CCPG1,GPRIN1,EML6,POU6F2,LINGO2,OPCML,CEMIP,ATF2,ZNF426,KCNJ12,HDAC8,ACVR1,HLA-DQA2,PID1,COL6A5,EBF2,GRID2,ZNF423,ZCCHC17,KCNJ3,RB1,NSMCE1,MPV17,IFNLR1,NTNG1,PDE4B,PKD1L3,KIF13A,NLRP4,ADIPOR1,ZNF143,GABRB3,GOSR2,SHROOM4,ZSCAN23,SMG7,PLPP3,HNF1B,IMMP2L,LAMP2,SPSB1,KIRREL3,GABRG2,F5,CPS1,NCOA2,TMEM120A,BNIP3L,PRKCH,ACACB,AGPAT5,RABGAP1L,DLC1,DENND2B,XRCC5,TBRG4,RGS17,GFRA1,SHLD2,SPHKAP,KCND2,INPP4A,BMX,ZNF418,RAD54L2,MPRIP,TAF15,TPST1,GRIA4,POLD3,RAB18,ZNF860,POU2F1,PRPF40B,TMTC1,ETV1,MGRN1,TEX11,MID2,LRTM2,SIK1,FBXL13,ZCWPW2,ERCC6L2,PIPOX,TP53BP2,KAT2B,GOLPH3L,TMTC2,WLS,ACTR3C,ANKS1A,RYR3,PRCP,SUPV3L1,MEF2D,MYO1B,MYL4,GTF2A1,SPTLC1,AGR3,CDK5RAP2,FLVCR1,UBE2E3,SAMD3,RFC3,FARSB,ARH

			GEF7,SAMD12,DACH1,ATF3,TRDN,NPHP1,FBN2,IKZF1,SLC26A8,CAPZA2,SNTG1,SRI,ATP1B1,ADAT2,ARNT,BDNF,LDLRAD4,ACBD6,SEMA3E,SCIN,MALRD1,TENT4B,MACO1,TLR6,STIM1,TSEN15,TMEM237,HAUS6,KIF16B,RAB11FIP2,CDH2,LAG3,PPARG,TANC1,PAPPA,ASB3,MTTP,CHRNB4,USP9X,TGM5,NEK7,ZNF80,TENM1,IL13RA2,RORB,CDK6,GABRB1,MYOF,JAKMIP1,SH3KBP1,NALCN,AVIL,LPCAT2,GDAP1,DDX60,TFAP2D,TTC39C,MYLK4,BEND7,USP25,KIF2C,MYO5A,SIPA1L3,SLC6A13,LRFN2,WDFY4,SPTBN5,SFRP4,BLTP1,SPDYE16,PNRC1,IL1RAP,VAV2,TFRC,VPS13A,TES,OSBPL6,SH3TC1,SPDYE6,FBN1,HAPSTR1,RAB31,MAP3K1,IQCB1,COA1,CLEC6A,RAPGEF4,BMPER,CLASP2,CLTC,ZNF521,RPRD2,ANGPT1,SEMA6A,EIF4G1,TMPRSS15,ATP6V0A4,PHLPP1,ZNF618,ITGA4,DNAJC6,ROCK1,NCAPH,M1AP,TAX1BP1,TBC1D4,APIP,MMP28,FAM13C,NUP98,IKZF2,PML,SACS,LAYN,MGLL,SCARA5,LONRF3,TNFSF4,AFAP1L2,SPATA25,SP100,PRKG1,PLXDC2,ELAVL4,SBF2,CDH9,FOXRED2,NDUFAF2,UMODL1,RFPL4B,TTC28,TSEN2,MAGI2,NIN,SPG21,ZNF286A,ANKFY1,ZNF69,ASIC2,ENSA,FNDC3A,KHDRBS3,AIMP1,SLC30A10,TPK1,HSPBP1,PRKD1,CALCRL,SLC20A2,USP34,STK4,TNNI3K,SPC25,ST6GAL2,RNASE11,SCAMP1,RPL27A,ANPEP,PRLR,ZNF569,PTPRA,ZNF180,PDE11A,NMI,RAB6C,PCCA,CLVS1,YLPM1,IL16,DIPK1A,ZSWIM7,POTEH,EDARADD,GNA14,OXNAD1,CR2,LPAR6,CTBP2,TTN,NCALD,HHIP,DYRK1A,PPFIA1,TMEM30A,MYRIP,PPP1R1C,KCNE2,UNC13C,CUL4A,SERINC1,KCTD16,EYA4,DLGAP2,ARL15,MAP7D3,SEMA5A,ELL2,LAMA4,PMEPA1,UBE3C,ATE1,ETNK1,TTPA,PEX5L,GATAD2B,METTL3,FMNL2,KRT85,UAP1,PARD3,KCNN4,SLC25A48,CP,FUNDC1,NPAS3,BMAL2,ATP8B1,PGPEP1,RNF6,SEMA3C,VAV1,SMYD1,FANCB,PEPD,GRIA2,VDAC2,KIAA0825,SPART
GO:0043167	ion binding	3.299037005767889e-10	*FSTL1,PFKP,ABCC6,ESYT1,STYK1,RGN,CDK14,ZSWIM1,MSRB3,PDE1C,ING3,ENSG00000290149,MAP3K3,NINL,PHF20,FOXK2,CPNE4,SMYD3,GARS1,RAPGEF6,MAOA,MAP3K13,PPM1A,ARL13B,LIMD1,TF,THRB,MYO5C,MAPK10,USP32,DAPP1,ATF7,CHRNB2,CDH8,AGTPBP1,FHL1,ATP10A,SH3PXD2A,PRND,ATP7B,CAMK2B,TLK1,GPATCH8,PGM1,DCT,SLC38A9,ZNF264,ABCG8,OTUD7A,KALRN,SUMF1,MAP4K3,XXYLT1,FPGT-TNNI3K,GALNT9,NOTCH2,CD93,ZNF551,NPNT,DSC1,MORC1,CDH11,NR4A3,SPTLC3,ZNF611,STK39,SMURF2,SRL,ADAMTS12,FER1L6,CDK18,STK24,ARMT1,CACNA1C,FAM20B,IDO1,PRKN,NLGN4X,RMND5A,PNPLA3,F13A1,PIK3C2B,KDM7A,SLC5A7,MARCHF9,CHD7,CBLL2,NLK,SDF4,GNAI1,MAPKAPK3,PAPOLG,ZNF10,LRP8,ENPEP,NUDT19,UBE2O,CPEB4,CHRFAM7A,ZNF121,MBTPS2,MCC,ADAR,DNAH10,PDE10A,CLCN3,RHOJ,ZFYVE16,MECOM,NLRP8,STK32B,NUDT13,CDH7,UBE2E2,EXO1,CHRM3,KDM2B,ZNF404,PMS1,WRNIP1,TYW1,COL11A1,RSF1,ZNF804A,SEPTIN10,CIDEB,MARCHF1,MYO1D,SESTD1,BRAF,ZNF214,COL1A2,DTNA,ABLIM1,ALPK2,CHRNA7,ZN*

			F304,CDIP1,LPP,HIVEP1,ATRX,TRIO,ZNF385D,ZNF85,RAD51B,MUSK,KCNIP3,MAP3K5,ALKBH1,ZCCHC4,RERG,PRKAR2A,ATP11A,ZNF154,ATP4A,PCDHB8,PRKG2,PIK3C3,HMCN1,RSKR,EPHA5,CBR4,ENSG00000289694,ATP13A4,RUNX1,ATP2C1,PPTC7,STRADA,PCLO,ZNF595,ASPH,RRAS2,UBR5,SPOCK3,GSN,PLS1,SPON1,KIF21A,SLIT3,NLRP7,PHC2,ZNF578,ITPR1,CPM,PAK3,KLF12,IPMK,CYP2A13,CAMK1G,PRUNE2,PRKAG2,CPQ,F8,GDPD1,VPS8,CACNA2D3,ZNF831,ZNF429,DTD1,CILK1,EPHA3,LATS2,MYH14,LYN,KLF7,MTA3,NME7,SEC24C,LARGE1,FIGNL1,PDZD8,PAMR1,CA10,RRM2,NMNAT3,FMO5,RNASEL,DBF4B,PAQR5,PCDHB16,DCP2,PPM1L,PDZRN4,NOS3,KCNIP4,ZNF280B,MLLT10,ERCC6,EIF2AK4,RAB12,HMGCLL1,MATN2,TGFBR2,ITGA11,MARK4,DDX60L,MDN1,ZNF800,ZNF462,OVCH1,CRTAC1,ZFHX3,RRAGD,ULK3,ZMAT4,CACNA2D4,KPNB1,GSDMD,ADGRV1,QTRT2,GAD1,SCG5,DAPK1,TDP2,ZNF678,ACSM2B,PRUNE1,SAR1B,APP,GATA4,SMAD5,RNLS,RNF38,ADAM12,UBE2G1,ATP9B,CACNA2D1,DHX35,SP140L,HCN1,PI4KB,RHOT1,HERC2,BMT2,RIPK4,QDPR,FRRS1,PTK2,SCP2,ZNF41,DNAH12,POGZ,PIP4K2B,LRRK2,ZNF395,IKZF3,CLSTN2,ARIH1,RARB,PJA2,COP1,CSRP3,ACVR2A,GNAO1,ASAP2,BRCC3,PRMT8,RABGEF1,CYB5R4,KIT,EPHA7,ANTXR1,TRABD2B,WIPI2,EFCAB5,RUNX1T1,PRKDC,AGAP4,ESR1,ZNF479,ITGB8,L3MBTL4,PPM1E,ZXDC,LMX1A,FIGN,PARS2,HUNK,NEK10,ZNF215,PDE7B,HDDC2,ACTR3B,ZNF722,PLCH1,HECTD1,TRPC5,IKBKB,ARHGEF28,ATF2,ZNF426,HDAC8,CHRNB3,ACVR1,EBF2,ZNF423,ZCCHC17,NSMCE1,PDE4B,DDX10,KIF13A,NLRP4,ADIPOR1,ZNF143,ZSCAN23,ZNF615,F5,AK5,CPS1,PRKCH,ACACB,XRCC5,KCND2,BMX,ZNF418,RAD54L2,TAF15,RAB18,ZNF860,TPH2,NRK,MGRN1,MID2,SIK1,ZCWPW2,ERCC6L2,PIPOX,ZNF850,ACTR3C,RYR3,SUPV3L1,MYO1B,MYL4,SPTLC1,UBE2E3,FARSB,FBN2,IKZF1,SRI,ADAT2,ACBD6,SCIN,TENT4B,ZNF665,ADAMTS9,STIM1,KIF16B,CDH2,EEFSEC,PPARG,PAPPA,ENSG00000290217,AGAP9,TGM5,NEK7,ZNF80,RORB,CDK6,MYOF,AVIL,LPCAT2,AMY2A,DDX60,MYLK4,KIF2C,MYO5A,VAV2,TES,PNPLA8,FBN1,RAB31,MAP3K1,ZFP82,DDX4,CLEC6A,ZNF292,RAPGEF4,ZNF521,ZNF761,EIF4G1,B3GAT2,ACSM5,PHLPP1,ZNF618,ITGA4,ROCK1,TAX1BP1,APIP,MMP28,ZNF701,KCNT2,IKZF2,PML,LONRF3,PLEK2,DGKG,SP100,PRKG1,CDH9,FOXRED2,UMODL1,RFPL4B,GALK2,PCDH19,NIN,ZNF286A,ANKFY1,ZNF69,TPK1,PRKD1,STK4,PCDH10,TNNI3K,RAB6D,ANPEP,PRLR,ZNF569,ZNF180,PDE11A,RAB6C,PCCA,CLVS1,EYS,ZSWIM7,ZNF470,GNA14,TTN,NCALD,HHIP,DYRK1A,MYRIP,UNC13C,CHSY3,TXNRD3,EYA4,ZNF888,ARL15,ZNF736,ETNK1,TTPA,GATAD2B,METTL3,PARD3,ZNF33A,CP,ATP8B1,RNF6,VAV1,SMYD1,PEPD,VDAC2
GO:0036094	small molecule binding	1.4146460041808265e-9	*FSTL1,PFKP,ABCC6,ESYT1,STYK1,RGN,CDK14,ZSWIM1,MSRB3,PDE1C,ING3,ENSG00000290149,MAP3K3,NINL,OSBPL8,PHF20,FOXK2,CPNE*

			4,SMYD3,GARS1,RAPGEF6,MAOA,MAP3K13,PPM1A,ARL13B,LIMD1,TF,THRB,MYO5C,MAPK10,USP32,DAPP1,ATF7,CHRNB2,CDH8,AGTPBP1,FHL1,ATP10A,SH3PXD2A,PRND,ATP7B,CAMK2B,TLK1,GPATCH8,PGM1,DCT,SLC38A9,ZNF264,ABCG8,OTUD7A,KALRN,SUMF1,MAP4K3,XXYLT1,FPGT-TNNI3K,GALNT9,NOTCH2,CD93,ZNF551,NPNT,DSC1,MORC1,CDH11,NR4A3,SPTLC3,ZNF611,STK39,SMURF2,SRL,ADAMTS12,FER1L6,CDK18,STK24,ARMT1,CACNA1C,FAM20B,IDO1,PRKN,NLGN4X,RMND5A,PNPLA3,F13A1,PIK3C2B,KDM7A,SLC5A7,MARCHF9,CHD7,CBLL2,NLK,SDF4,GNAI1,MAPKAPK3,PAPOLG,ZNF10,LRP8,ENPEP,NUDT19,UBE2O,CPEB4,CHRFAM7A,ZNF121,MBTPS2,MCC,ADAR,DNAH10,PDE10A,CLCN3,RHOJ,ZFYVE16,MECOM,NLRP8,STK32B,NUDT13,CDH7,UBE2E2,EXO1,CHRM3,KDM2B,ZNF404,PMS1,WRNIP1,TYW1,COL11A1,RSF1,ZNF804A,SEPTIN10,CIDEB,MARCHF1,MYO1D,SESTD1,BRAF,ZNF214,COL1A2,DTNA,ABLIM1,ALPK2,CHRNA7,ZNF304,CDIP1,LPP,HIVEP1,ATRX,TRIO,ZNF385D,ZNF85,RAD51B,MUSK,KCNIP3,MAP3K5,ALKBH1,ZCCHC4,RERG,PRKAR2A,ATP11A,ZNF154,ATP4A,PCDHB8,PRKG2,PIK3C3,HMCN1,RSKR,EPHA5,CBR4,ENSG00000289694,ATP13A4,RUNX1,ATP2C1,ALDH1A2,PPTC7,STRADA,PCLO,ZNF595,ASPH,RRAS2,UBR5,SPOCK3,GSN,PLS1,SPON1,KIF21A,SLIT3,NLRP7,PHC2,ZNF578,ITPR1,CPM,PAK3,KLF12,IPMK,CYP2A13,CAMK1G,PRUNE2,PRKAG2,CPQ,F8,GDPD1,VPS8,CACNA2D3,ZNF831,ZNF429,DTD1,CILK1,EPHA3,LATS2,MYH14,LYN,KLF7,MTA3,NME7,SEC24C,LARGE1,FIGNL1,PDZD8,PAMR1,CA10,RRM2,ALDH1A1,NMNAT3,FMO5,RNASEL,DBF4B,PAQR5,PCDHB16,DCP2,PPM1L,PDZRN4,NOS3,KCNIP4,ZNF280B,MLLT10,ERCC6,EIF2AK4,RAB12,HMGCLL1,MATN2,TGFBR2,ITGA11,MARK4,DDX60L,MDN1,ZNF800,ZNF462,OVCH1,CRTAC1,ZFHX3,RRAGD,ULK3,ZMAT4,CACNA2D4,KPNB1,GSDMD,ADGRV1,QTRT2,GAD1,SCG5,DAPK1,TDP2,ALDH9A1,ZNF678,ACSM2B,PRUNE1,SAR1B,APP,GATA4,SMAD5,RNLS,RNF38,ADAM12,UBE2G1,ATP9B,CACNA2D1,DHX35,SP140L,HCN1,PI4KB,RHOT1,HERC2,BMT2,RIPK4,LMAN1L,QDPR,FRRS1,PTK2,SCP2,ZNF41,DNAH12,POGZ,PIP4K2B,LRRK2,ZNF395,IKZF3,CLSTN2,ARIH1,RARB,PJA2,PAEP,COP1,CSRP3,ACVR2A,GNAO1,ASAP2,BRCC3,PRMT8,RABGEF1,CYB5R4,KIT,EPHA7,OSBPL3,ANTXR1,TRABD2B,WIPI2,EFCAB5,RUNX1T1,PRKDC,AGAP4,ESR1,ZNF479,ITGB8,L3MBTL4,PPM1E,ZXDC,LMX1A,FIGN,PARS2,HUNK,NEK10,ZNF215,PDE7B,HDDC2,ACTR3B,ZNF722,PLCH1,HECTD1,VKORC1L1,TRPC5,IKBKB,ARHGEF28,ATF2,ZNF426,HDAC8,CHRNB3,ACVR1,EBF2,ZNF423,ZCCHC17,NSMCE1,PDE4B,DDX10,KIF13A,NLRP4,ADIPOR1,ZNF143,ZSCAN23,ZNF615,F5,AK5,CPS1,PRKCH,ACACB,XRCC5,KCND2,BMX,ZNF418,RAD54L2,TAF15,RAB18,ZNF860,TPH2,NRK,MGRN1,MID2,SIK1,ZCWPW2,ERCC6L2,PIPOX,ZNF850,ACTR3C,RYR3,SUPV3L1,MYO1B,MY

			L4,SPTLC1,UBE2E3,FARSB,FBN2,IKZF1,SRI,ADAT2,ACBD6,SCIN,TENT4B,ZNF665,ADAMTS9,STIM1,KIF16B,CDH2,EEFSEC,PPARG,PAPPA,ENSG00000290217,AGAP9,TGM5,NEK7,ZNF80,RORB,CDK6,MYOF,AVIL,LPCAT2,AMY2A,DDX60,MYLK4,KIF2C,MYO5A,VAV2,TES,OSBPL6,PNPLA8,FBN1,RAB31,MAP3K1,ZFP82,DDX4,CLEC6A,ZNF292,RAPGEF4,ZNF521,ZNF761,EIF4G1,B3GAT2,ACSM5,PHLPP1,ZNF618,ITGA4,ROCK1,TAX1BP1,APIP,MMP28,ZNF701,KCNT2,IKZF2,PML,LONRF3,PLEK2,DGKG,SP100,PRKG1,CDH9,FOXRED2,UMODL1,RFPL4B,GALK2,PCDH19,NIN,ZNF286A,ANKFY1,ZNF69,TPK1,PRKD1,STK4,PCDH10,TNNI3K,RAB6D,ANPEP,PRLR,ZNF569,ZNF180,PDE11A,RAB6C,PCCA,CLVS1,EYS,ZSWIM7,ZNF470,GNA14,CTBP2,TTN,NCALD,HHIP,DYRK1A,MYRIP,UNC13C,CHSY3,TXNRD3,EYA4,ZNF888,ARL15,ZNF736,ETNK1,TTPA,GATAD2B,METTL3,PARD3,ZNF33A,CP,ATP8B1,RNF6,VAV1,SMYD1,PEPD,VDAC2
GO:0043169	cation binding	7.767499317566378e-7	FSTL1,PFKP,ESYT1,RGN,ZSWIM1,MSRB3,PDE1C,ING3,ENSG00000290149,MAP3K3,NINL,PHF20,FOXK2,CPNE4,SMYD3,MAP3K13,PPM1A,LIMD1,TF,THRB,USP32,ATF7,CHRNB2,CDH8,AGTPBP1,FHL1,ATP10A,PRND,ATP7B,GPATCH8,PGM1,DCT,SLC38A9,ZNF264,ABCG8,OTUD7A,KALRN,SUMF1,XXYLT1,GALNT9,NOTCH2,CD93,ZNF551,NPNT,DSC1,MORC1,CDH11,NR4A3,ZNF611,SMURF2,ADAMTS12,FER1L6,STK24,ARMT1,CACNA1C,FAM20B,IDO1,PRKN,RMND5A,F13A1,KDM7A,SLC5A7,MARCHF9,CBLL2,NLK,SDF4,GNAI1,PAPOLG,ZNF10,LRP8,ENPEP,NUDT19,CPEB4,CHRFAM7A,ZNF121,MBTPS2,MCC,ADAR,PDE10A,ZFYVE16,MECOM,STK32B,NUDT13,CDH7,EXO1,CHRM3,KDM2B,ZNF404,WRNIP1,TYW1,COL11A1,RSF1,ZNF804A,MARCHF1,BRAF,ZNF214,COL1A2,DTNA,ABLIM1,CHRNA7,ZNF304,CDIP1,LPP,HIVEP1,ATRX,ZNF385D,ZNF85,MUSK,KCNIP3,MAP3K5,ALKBH1,ZCCHC4,ATP11A,ZNF154,ATP4A,PCDHB8,HMCN1,ATP13A4,RUNX1,ATP2C1,PPTC7,PCLO,ZNF595,ASPH,UBR5,SPOCK3,GSN,PLS1,SPON1,SLIT3,PHC2,ZNF578,ITPR1,CPM,PAK3,KLF12,IPMK,CYP2A13,PRUNE2,PRKAG2,CPQ,F8,GDPD1,VPS8,CACNA2D3,ZNF831,ZNF429,DTD1,CILK1,LATS2,KLF7,MTA3,NME7,SEC24C,LARGE1,FIGNL1,PDZD8,PAMR1,CA10,RRM2,RNASEL,DBF4B,PAQR5,PCDHB16,DCP2,PPM1L,PDZRN4,NOS3,KCNIP4,ZNF280B,MLLT10,HMGCLL1,MATN2,TGFBR2,ITGA11,ZNF800,ZNF462,OVCH1,CRTAC1,ZFHX3,ZMAT4,CACNA2D4,KPNB1,ADGRV1,QTRT2,TDP2,ZNF678,ACSM2B,PRUNE1,SAR1B,APP,GATA4,SMAD5,RNLS,RNF38,ADAM12,ATP9B,CACNA2D1,SP140L,RHOT1,HERC2,BMT2,FRRS1,ZNF41,POGZ,LRRK2,ZNF395,IKZF3,CLSTN2,ARIH1,RARB,PJA2,COP1,CSRP3,ACVR2A,GNAO1,ASAP2,BRCC3,PRMT8,RABGEF1,CYB5R4,KIT,ANTXR1,TRABD2B,EFCAB5,RUNX1T1,AGAP4,ESR1,ZNF479,ITGB8,L3MBTL4,PPM1E,ZXDC,LMX1A,NEK10,ZNF215,PDE7B,HDDC2,ZNF722,PLCH1,HECTD1,ARHGEF28,ATF2,ZNF426,HDAC8,CHRNB3,ACVR1,EBF2,ZNF4

			23,ZCCHC17,NSMCE1,PDE4B,ADIPOR1,ZNF143,ZSCAN23,ZNF615,F5,CPS1,PRKCH,ACACB,KCND2,BMX,ZNF418,TAF15,ZNF860,TPH2,MGRN1,MID2,SIK1,ZCWPW2,ZNF850,RYR3,MYL4,FARSB,FBN2,IKZF1,SRI,ADAT2,SCIN,TENT4B,ZNF665,ADAMTS9,STIM1,CDH2,PPARG,PAPPA,ENSG00000290217,AGAP9,TGM5,NEK7,ZNF80,RORB,MYOF,LPCAT2,AMY2A,VAV2,TES,FBN1,MAP3K1,ZFP82,CLEC6A,ZNF292,ZNF521,ZNF761,B3GAT2,ACSM5,PHLPP1,ZNF618,ITGA4,ROCK1,TAX1BP1,APIP,MMP28,ZNF701,IKZF2,PML,LONRF3,DGKG,SP100,CDH9,UMODL1,RFPL4B,PCDH19,NIN,ZNF286A,ANKFY1,ZNF69,TPK1,PRKD1,STK4,PCDH10,TNNI3K,ANPEP,PRLR,ZNF569,ZNF180,PDE11A,PCCA,EYS,ZSWIM7,ZNF470,GNA14,TTN,NCALD,HHIP,MYRIP,UNC13C,CHSY3,EYA4,ZNF888,ZNF736,GATAD2B,METTL3,ZNF33A,CP,ATP8B1,RNF6,VAV1,SMYD1,PEPD,VDAC2
GO:0046872	metal ion binding	0.000009506684306713266	*FSTL1,PFKP,ESYT1,RGN,ZSWIM1,MSRB3,PDE1C,ING3,ENSG00000290149,MAP3K3,NINL,PHF20,FOXK2,CPNE4,SMYD3,MAP3K13,PPM1A,LIMD1,TF,THRB,USP32,ATF7,CDH8,AGTPBP1,FHL1,ATP10A,PRND,ATP7B,GPATCH8,PGM1,DCT,SLC38A9,ZNF264,ABCG8,OTUD7A,KALRN,SUMF1,XXYLT1,GALNT9,NOTCH2,CD93,ZNF551,NPNT,DSC1,MORC1,CDH11,NR4A3,ZNF611,SMURF2,ADAMTS12,FER1L6,STK24,ARMT1,CACNA1C,FAM20B,IDO1,PRKN,RMND5A,F13A1,KDM7A,MARCHF9,CBLL2,NLK,SDF4,GNAI1,PAPOLG,ZNF10,LRP8,ENPEP,NUDT19,CPEB4,ZNF121,MBTPS2,MCC,ADAR,PDE10A,ZFYVE16,MECOM,STK32B,NUDT13,CDH7,EXO1,KDM2B,ZNF404,WRNIP1,TYW1,COL11A1,RSF1,ZNF804A,MARCHF1,BRAF,ZNF214,COL1A2,DTNA,ABLIM1,ZNF304,CDIP1,LPP,HIVEP1,ATRX,ZNF385D,ZNF85,MUSK,KCNIP3,MAP3K5,ALKBH1,ZCCHC4,ATP11A,ZNF154,ATP4A,PCDHB8,HMCN1,ATP13A4,RUNX1,ATP2C1,PPTC7,PCLO,ZNF595,ASPH,UBR5,SPOCK3,GSN,PLS1,SPON1,SLIT3,PHC2,ZNF578,ITPR1,CPM,PAK3,KLF12,IPMK,CYP2A13,PRUNE2,CPQ,F8,GDPD1,VPS8,CACNA2D3,ZNF831,ZNF429,DTD1,CILK1,LATS2,KLF7,MTA3,NME7,SEC24C,LARGE1,FIGNL1,PDZD8,PAMR1,CA10,RRM2,RNASEL,DBF4B,PAQR5,PCDHB16,DCP2,PPM1L,PDZRN4,NOS3,KCNIP4,ZNF280B,MLLT10,HMGCLL1,MATN2,TGFBR2,ITGA11,ZNF800,ZNF462,OVCH1,CRTAC1,ZFHX3,ZMAT4,CACNA2D4,KPNB1,ADGRV1,QTRT2,TDP2,ZNF678,ACSM2B,PRUNE1,SAR1B,APP,GATA4,SMAD5,RNF38,ADAM12,ATP9B,CACNA2D1,SP140L,RHOT1,HERC2,FRRS1,ZNF41,POGZ,LRRK2,ZNF395,IKZF3,CLSTN2,ARIH1,RARB,PJA2,COP1,CSRP3,ACVR2A,GNAO1,ASAP2,BRCC3,RABGEF1,CYB5R4,KIT,ANTXR1,TRABD2B,EFCAB5,RUNX1T1,AGAP4,ESR1,ZNF479,ITGB8,L3MBTL4,PPM1E,ZXDC,LMX1A,NEK10,ZNF215,PDE7B,HDDC2,ZNF722,PLCH1,HECTD1,ARHGEF28,ATF2,ZNF426,HDAC8,ACVR1,EBF2,ZNF423,ZCCHC17,NSMCE1,PDE4B,ADIPOR1,ZNF143,ZSCAN23,ZNF615,F5,CPS1,PRKCH,ACACB,KCND2,BMX,ZNF418,TAF15,ZNF860,*

			TPH2,MGRN1,MID2,SIK1,ZCWPW2,ZNF850,RYR3,MYL4,FARSB,FBN2,IKZF1,SRI,ADAT2,SCIN,TENT4B,ZNF665,ADAMTS9,STIM1,CDH2,PPARG,PAPPA,ENSG00000290217,AGAP9,TGM5,NEK7,ZNF80,RORB,MYOF,LPCAT2,AMY2A,VAV2,TES,FBN1,MAP3K1,ZFP82,CLEC6A,ZNF292,ZNF521,ZNF761,B3GAT2,ACSM5,PHLPP1,ZNF618,ITGA4,ROCK1,TAX1BP1,APIP,MMP28,ZNF701,IKZF2,PML,LONRF3,DGKG,SP100,CDH9,UMODL1,RFPL4B,PCDH19,NIN,ZNF286A,ANKFY1,ZNF69,PRKD1,STK4,PCDH10,TNNI3K,ANPEP,PRLR,ZNF569,ZNF180,PDE11A,PCCA,EYS,ZSWIM7,ZNF470,GNA14,TTN,NCALD,HHIP,MYRIP,UNC13C,CHSY3,EYA4,ZNF888,ZNF736,GATAD2B,ZNF33A,CP,ATP8B1,RNF6,VAV1,SMYD1,PEPD
GO:0005488	binding	0.000014834050758208442	*FSTL1,C10ORF90,APBB2,PFKP,ABCC6,ESYT1,STYK1,RPL19,RTN1,RGN,CDK14,ZSWIM1,EPN2,MSRB3,PDE1C,MREG,HNRNPCL4,NOSTRIN,ING3,ENSG00000290149,MAP3K3,NINL,SLC12A8,OSBPL8,LRRN1,NFIA,POTED,STX8,PHF20,FOXK2,CPNE4,RBM17,SMYD3,GARS1,RAPGEF6,ARHGEF9,TAFA2,ABR,MAOA,BLTP3B,TRAPPC9,SLC16A5,FNDC1,PREP,MAP3K13,EIF1AX,PPM1A,ARL13B,SAMSN1,THADA,LIMD1,TF,THRB,MYBPC1,USP42,IPP,RCSD1,SPTBN1,CDC42EP3,MYO5C,MAPK10,CACNB1,ITPRID1,USP32,AHCYL2,DAPP1,ATF7,GNG12,FGF1,CHRNB2,SEZ6,CLDN6,KCNQ5,CDH8,GNB4,AGTPBP1,ATF7-NPFF,FHL1,TRMT6,PPP1R37,RBPMS,ATP10A,SH3PXD2A,SPINK5,MRPS24,SKAP2,PRND,ATP7B,GPBP1L1,PPM1H,KCNJ6,BRD4,DOK4,ZAN,TNRC18,CAMK2B,GRIP1,TLK1,GPATCH8,DDI2,PGM1,DCT,DLGAP1,PECR,SLC38A9,ZNF264,HBP1,SDR16C5,ABCG8,RGPD2,XPO4,OTUD7A,KALRN,SUMF1,USH2A,MAP4K3,XXYLT1,FPGT-TNNI3K,GALNT9,NOTCH2,CD93,FKBP5,TMOD2,SLC44A5,SDK2,HMBOX1,ZNF551,RNU2-49P,NPNT,DSC1,EPB41L3,MAP6,MORC1,PARVB,CDH11,SHANK3,WDR26,NR4A3,SPTLC3,ARID1B,E2F7,SLC14A2,FOXO1,ZNF611,STYXL1,STK39,SMURF2,SRL,ADAMTS12,DAAM2,FER1L6,TBC1D16,PLXNA4,CDK18,YBX1,STK24,ARMT1,CFAP70,DCDC1,CACNA1C,FAM20B,FAM3C,ASB4,IDO1,ARB2A,PRKN,NLGN4X,EXOC4,RMND5A,PNPLA3,F13A1,PIK3C2B,XPO6,KDM7A,SLC44A3,SLC5A7,MARCHF9,FAM83B,IFT172,CHD7,GPSM1,CBLL2,PTBP3,CDON,NLK,SDF4,THSD7A,GNAI1,MRPL13,MAPKAPK3,NCAM2,PAPOLG,TIAM2,ZNF10,TRAK2,CNMD,LRP8,TAGLN3,CNTN1,AGL,MEOX2,STAT5B,ENPEP,NUDT19,ARHGEF4,UBE2O,MUC17,CPEB4,EIF4ENIF1,HEATR9,CALCR,CHRFAM7A,ELMO1,ERBIN,ZNF121,MBTPS2,RGS22,CD247,MCC,CEP192,PTH,ADAR,DNAH10,PDE10A,TOX2,PDHX,CLCN3,RHOJ,CDC37,FGF10,ZFYVE16,MECOM,COL4A6,TSPAN18,SAMD15,NLRP8,STK32B,PLG,NEURL4,HERC5,NUDT13,CDH7,UBE2E2,NCK1,CD84,FAM168A,NONO,RARRES1,KLHL42,NREP,EXO1,GABRA5,EBNA1BP2,KCNQ3,MTCP1,SH3BGR,CHRM3,KDM2B,PREX1,RGPD3,AIG1,ERICH1,ZNF404,PMS1,WRNIP1,COL24A1,ANO3,TYW1,GOLGA8R,COL11A1,RSF1,KCTD3*

			,NCOA7,C2ORF88,RNU2-33P,ZNF804A,SEPTIN10,CIDEB,MARCHF1,TTC33,MCPH1,MYO1D,SESTD1,DNAAF9,BRAF,ZNF214,COL1A2,DTNA,TMOD3,GEMIN5,EEF2KMT,MRTFB,XPO7,KCNS3,COL12A1,ABLIM1,RBMS1,ALPK2,CHRNA7,HECW1,HMGB1,ZNF304,DYNLT2B,CDIP1,AFF3,RFLNA,LPP,HIVEP1,ATRX,TGFB2,MDFIC,EPB41L4A,LAMA2,ADD3,TRIO,ZNF385D,ATXN7L1,FBXO16,ZNF85,HOXC13,SMARCC1,RAD51B,NDST1,MUSK,KCNJ18,JAM3,KCNIP3,SFPQ,MAP3K5,ALKBH1,ARPP21,RGS3,NUP93,ZCCHC4,RERG,OR5AU1,PRKAR2A,RBM33,TBC1D5,CTIF,CFAP57,ATP11A,RBM23,ZNF154,CSTF3,ATP4A,SLC15A4,PCDHB8,GOLPH3,PRKG2,MBTPS1,NCF2,PIK3C3,PIGV,RNU6-1216P,USP10,SND1,HMCN1,IGFBP7,RSKR,EPHA5,SKOR2,CBR4,ENSG00000289694,ATP13A4,RUNX1,ENAH,SNX10,ATP2C1,ALDH1A2,TMEM230,PPTC7,STON2,IGHV1OR21-1,FAM171A1,STRADA,PCLO,HUWE1,ZNF595,ASPH,RRAS2,SH3BGRL,ZDHHC4,UBR5,GTF2F2,SIM1,KIAA1217,ABTB2,SOX30,ANKS1B,CHL1,SPOCK3,GSN,PLS1,TCP11L1,SPON1,MAPRE3,ANO2,TLR8,HPS3,KIF21A,SLIT3,ARMH3,CACUL1,NLRP7,ARHGEF39,PHC2,ROBO1,ZNF578,ANKRD11,ITPR1,PKP4,CPM,DNAAF11,PAK3,NDUFAF7,ABI3BP,RNU1-83P,KLF12,IPMK,CYP2A13,TMEM183A,CAMK1G,PRUNE2,DOK6,PRKAG2,MLLT3,CCNT2,BTG4,HAVCR1,TRAPPC10,CPQ,CNOT10,MR1,KRTAP21-2,F8,TNFRSF12A,OASL,GDPD1,CPT2,VPS8,ISY1,CACNA2D3,ZNF831,ZNF429,CXCR5,NCKAP1,PRPF40A,CLDN12,DTD1,CNTN3,MGAM,CILK1,FOSL2,PACRG,ADGRD1,HNRNPCL3,EPHA3,CHCHD3,LATS2,TYW3,MYH14,SPDYE2B,MRPL32,TPRG1,KLHL33,ERP29,CDYL2,LRRTM4,ARHGAP18,UVRAG,MYOM2,LYN,ADGRB3,USP6,KLF7,SUMO2,MTA3,UBL3,DIP2B,NME7,SEC24C,LARGE1,ATP6V1H,NUP58,FIGNL1,PUS7,PDZD8,PAMR1,CCDC33,CA10,RRM2,CRYBB1,SIL1,LDB2,MYRFL,SPTB,UNC93A,C21ORF62,ALDH1A1,NMNAT3,FMO5,RNASEL,NAV1,DBF4B,PAQR5,CORIN,TMCC1,LRRC7,PCDHB16,DCP2,PPM1L,PDZRN4,SGMS2,SLC41A2,NOS3,KCNIP4,VSX1,ZNF280B,EIF3L,NOMO2,TNRC6C,MLLT10,BPHL,ERCC6,NOS1AP,EIF2AK4,KIAA1614,MSRA,SYNPR,RAB12,IMPG1,HMGCLL1,MAGEL2,MATN2,TGFBR2,CMKLR2,ITGA11,CFAP77,MARK4,DDX60L,MDN1,SYNJ1,TTC6,TCF7L2,ZNF800,CHAF1B,KIAA1328,ZNF462,MTRF1,OVCH1,CRTAC1,E2F6,ZFHX3,CUX1,POLR2C,UNC119,SHISA2,GPM6B,RRAGD,C2ORF50,ULK3,SPDYE2,ZMAT4,GKAP1,CACNA2D4,LHFPL3,RGPD1,PPP2R5E,KPNB1,GSDMD,ADGRV1,RASGRF2,ST7,SERBP1,QTRT2,EVA1C,EPB41L5,GAD1,SCG5,NUDCD1,WAC,WHRN,OCA2,DAPK1,TDP2,ALDH9A1,ZNF678,ACSM2B,PRUNE1,SAR1B,TTC7B,INSYN2A,BTG3,APP,GATA4,ATF7IP2,SMAD5,IGF2BP1,PPIL6,RNLS,GSKIP,RNF38,ADAM12,UBE2G1,PELI2,ATP9B,CACNA2D1,DHX35,SP140L,HCN1,CHRM5,PI4KB,RHOT1,ELOVL5,HERC2,HOOK3,USE1,PIK3R5,PALLD,TLR1

			,CECR2,BMT2,RIPK4,AVPR1B,LMAN1L,QDPR,FRRS1,IQCM,PTK2,NOP9,USP7,TICAM2,SCP2,SH2B2,ATP1B4,ZNF41,AVEN,CELF1,HYCC1,DNAH12,POTEG,POGZ,PIP4K2B,PRRC2B,UQCC1,LRRK2,ZNF395,IKZF3,SLC38A4,CLSTN2,ARIH1,CFAP298,PLD1,ITFG2,VTCN1,PKHD1,TENT5C,UBXN7,RARB,PJA2,TCF4,MGST1,RBM44,ARHGEF38,PAEP,COP1,TIAM1,CSRP3,PHACTR1,MLIP,IQSEC2,ACVR2A,GNAO1,ASAP2,TIPIN,VIT,RASSF4,C6ORF58,SNTG2,NTM,MEIS1,AMMECR1,NEDD4L,BRCC3,ENSG00000288683,DUSP14,PRMT8,SNX13,RABGEF1,CYB5R4,KIT,TBC1D8,EPHA7,SPAG9,OSBPL3,ANTXR1,C19ORF47,SPDL1,WNT5A,TRABD2B,WIPI2,PEX26,ARFGEF2,TEX2,CTNNB1,ANKRD17,EFCAB5,RUNX1T1,CTSK,PRKDC,AGAP4,IPO5,ESR1,TNFRSF19,ZNF479,PTPN14,SCFD1,ITGB8,ERC2,DMGDH,TRANK1,BCAR3,TPD52L1,L3MBTL4,PPM1E,ZXDC,VPS53,LMX1A,DCAF8L1,WWOX,TPST2,FIGN,BTBD8,PARS2,BNIPL,HUNK,NEK10,SNX29,ANAPC10,FBXO15,PTGER4,ZNF215,BBX,PDE7B,VWC2L,BEST3,DOCK1,HDDC2,DIAPH3,CCDC107,GPC4,BPGM,PKIG,EFNA5,IGF2BP2,ACTR3B,ZNF722,CDC14B,SYNE3,PLCH1,HECTD1,VKORC1L1,NGEF,TRPC5,NEUROD2,FNIP2,AASS,JAML,KAT14,UGGT2,UBE3B,TNFSF11,PTPRF,IKBKB,FXR2,SH3BP5,AKAP6,USF3,LYVE1,APOH,ARHGEF28,OR8B8,CLCN1,RAB3GAP2,TASOR,STEAP1B,CCPG1,GPRIN1,EML6,POU6F2,LINGO2,OPCML,CEMIP,ATF2,RNU6-986P,ZNF426,KCNJ12,HDAC8,CHRNB3,ACVR1,HLA-DQA2,PID1,COL6A5,EBF2,GRID2,ZNF423,ZCCHC17,KCNJ3,RB1,NSMCE1,MPV17,IFNLR1,RNU6-826P,NTNG1,PDE4B,DDX10,PKD1L3,KIF13A,NLRP4,ADIPOR1,ZNF143,GABRB3,GOSR2,SHROOM4,ZSCAN23,SMG7,PLPP3,HNF1B,RNU6-1311P,IMMP2L,ZNF615,LAMP2,SPSB1,KIRREL3,GABRG2,F5,AK5,CPS1,NCOA2,TMEM120A,BNIP3L,PRKCH,ACACB,AGPAT5,RABGAP1L,DLC1,DENND2B,XRCC5,TBRG4,RGS17,GFRA1,SHLD2,SPHKAP,KCND2,INPP4A,BMX,ZNF418,RAD54L2,MPRIP,TAF15,TPST1,GRIA4,POLD3,RAB18,ZNF860,TPH2,POU2F1,PRPF40B,TMTC1,ETV1,NRK,MGRN1,TEX11,MID2,RBM28,LRTM2,SIK1,FBXL13,ZCWPW2,ERCC6L2,PIPOX,TP53BP2,ZNF850,KAT2B,GOLPH3L,TMTC2,WLS,ACTR3C,ANKS1A,RYR3,PRCP,SUPV3L1,MEF2D,MYO1B,MGAM2,MYL4,GTF2A1,SPTLC1,AGR3,CDK5RAP2,FLVCR1,UBE2E3,SAMD3,RFC3,FARSB,ARHGEF7,SAMD12,DACH1,ATF3,TRDN,NPHP1,FBN2,IKZF1,SLC26A8,CAPZA2,SNTG1,SRI,ATP1B1,ADAT2,ARNT,BDNF,LDLRAD4,TFCP2L1,ACBD6,SEMA3E,BCLAF3,SCIN,MALRD1,TENT4B,MACO1,ZNF665,TLR6,ADAMTS9,STIM1,TSEN15,TMEM237,TMEM161B,HAUS6,KIF16B,RAB11FIP2,CDH2,EEFSEC,LAG3,PPARG,TANC1,PAPPA,ENSG00000290217,ASB3,MTTP,CHRNB4,AGAP9,USP9X,TGM5,NEK7,ZNF80,TENM1,IL13RA2,RORB,CDK6,GABRB1,MYOF,JAKMIP1,SH3KBP1,NALCN,AVIL,L

			PCAT2,GDAP1,AMY2A,DDX60,TFAP2D,TTC39C,MYLK4,BEND7,USP25,KIF2C,MYO5A,SIPA1L3,SLC6A13,LRFN2,WDFY4,SPTBN5,SFRP4,BLTP1,SPDYE16,PNRC1,IL1RAP,VAV2,TFRC,VPS13A,TES,OSBPL6,SH3TC1,GRIA3,PNPLA8,SPDYE6,FBN1,HAPSTR1,RAB31,MAP3K1,ZFP82,DDX4,IQCB1,COA1,CLEC6A,ZNF292,RAPGEF4,BMPER,RNU6-984P,CLASP2,CLTC,ZNF521,RPRD2,ZNF761,ANGPT1,SEMA6A,VIPR2,EIF4G1,TMPRSS15,B3GAT2,ATP6V0A4,ACSM5,PHLPP1,ZNF618,ITGA4,DNAJC6,ROCK1,NCAPH,M1AP,TAX1BP1,TBC1D4,APIP,MMP28,FAM13C,ZNF701,KCNT2,NUP98,CLEC3A,IKZF2,PML,FOXO3B,SACS,AFF2,LAYN,MGLL,SCARA5,LONRF3,PLEK2,TNFSF4,AFAP1L2,DGKG,SPATA25,SP100,PRKG1,PLXDC2,ELAVL4,SBF2,CDH9,FOXRED2,NDUFAF2,UMODL1,RFPL4B,TTC28,TSEN2,MAGI2,GALK2,PCDH19,NIN,SPG21,ZNF286A,ANKFY1,ZNF69,ASIC2,ENSA,FNDC3A,KHDRBS3,AIMP1,SLC30A10,TPK1,HSPBP1,PRKD1,CALCRL,SLC20A2,USP34,STK4,PCDH10,TNNI3K,RAB6D,SPC25,ST6GAL2,RNASE11,SCAMP1,RPL27A,ANPEP,PRLR,ZNF569,PTPRA,ZNF180,PDE11A,NMI,RAB6C,PCCA,CLVS1,YLPM1,EYS,IL16,DIPK1A,ZSWIM7,ZNF470,POTEH,EDARADD,GNA14,OXNAD1,CR2,LPAR6,CTBP2,TTN,NCALD,HHIP,DYRK1A,PPFIA1,TMEM30A,MYRIP,PPP1R1C,KCNE2,UNC13C,CUL4A,SERINC1,KCTD16,CHSY3,TXNRD3,EYA4,ZNF888,DLGAP2,ARL15,ZNF736,MAP7D3,SEMA5A,ELL2,LAMA4,PMEPA1,UBE3C,ATE1,ETNK1,TTPA,PEX5L,PVT1,GATAD2B,METTL3,FMNL2,KRT85,UAP1,PARD3,KCNN4,ZNF33A,SLC25A48,CP,FUNDC1,RNU6-849P,NPAS3,BMAL2,ATP8B1,PGPEP1,RNF6,SEMA3C,VAV1,SMYD1,FANCB,PEPD,GRIA2,VDAC2,KIAA0825,VPS13B,SPART
GO:0030554	adenyl nucleotide binding	0.0000544019184429352 6	*PFKP,ABCC6,STYK1,CDK14,MAP3K3,GARS1,MAP3K13,MYO5C,MAPK10,ATP10A,ATP7B,CAMK2B,TLK1,ABCG8,KALRN,MAP4K3,FPGT-TNNI3K,STK39,CDK18,STK24,FAM20B,PIK3C2B,CHD7,NLK,MAPKAPK3,PAPOLG,UBE2O,DNAH10,PDE10A,CLCN3,NLRP8,STK32B,UBE2E2,PMS1,WRNIP1,MYO1D,BRAF,ALPK2,ATRX,TRIO,RAD51B,MUSK,MAP3K5,PRKAR2A,ATP11A,ATP4A,PRKG2,PIK3C3,RSKR,EPHA5,CBR4,ATP13A4,RUNX1,ATP2C1,STRADA,KIF21A,NLRP7,PAK3,IPMK,CAMK1G,PRKAG2,CILK1,EPHA3,LATS2,MYH14,LYN,NME7,FIGNL1,ALDH1A1,NMNAT3,FMO5,RNASEL,NOS3,ERCC6,EIF2AK4,TGFBR2,MARK4,DDX60L,MDN1,ULK3,DAPK1,ACSM2B,RNLS,UBE2G1,ATP9B,DHX35,HCN1,PI4KB,RIPK4,QDPR,PTK2,DNAH12,PIP4K2B,LRRK2,ACVR2A,KIT,EPHA7,PRKDC,FIGN,PARS2,HUNK,NEK10,ACTR3B,IKBKB,ACVR1,PDE4B,DDX10,KIF13A,NLRP4,AK5,CPS1,PRKCH,ACACB,XRCC5,BMX,RAD54L2,NRK,SIK1,ERCC6L2,ACTR3C,SUPV3L1,MYO1B,UBE2E3,FARSB,KIF16B,NEK7,CDK6,DDX60,MYLK4,KIF2C,MYO5A,PNPLA8,MAP3K1,DDX4,RAPGEF4,EIF4G1,ACSM5,ROCK1,KCNT2,DGKG,PRKG1,GALK2,TPK1,PRKD1,STK4,TNNI3K,P*

			CCA,CTBP2,TTN,DYRK1A,ETNK1,ATP8B1
GO:0032559	adenyl ribonucleotide binding	0.0000561566872718435 6	*PFKP,ABCC6,STYK1,CDK14,MAP3K3,GARS1,MAP3K13,MYO5C,MAPK10,ATP10A,ATP7B,CAMK2B,TLK1,ABCG8,KALRN,MAP4K3,FPGT-TNNI3K,STK39,CDK18,STK24,FAM20B,PIK3C2B,CHD7,NLK,MAPKAPK3,PAPOLG,UBE2O,DNAH10,PDE10A,CLCN3,NLRP8,STK32B,UBE2E2,PMS1,WRNIP1,MYO1D,BRAF,ALPK2,ATRX,TRIO,RAD51B,MUSK,MAP3K5,PRKAR2A,ATP11A,ATP4A,PRKG2,PIK3C3,RSKR,EPHA5,ATP13A4,RUNX1,ATP2C1,STRADA,KIF21A,NLRP7,PAK3,IPMK,CAMK1G,PRKAG2,CILK1,EPHA3,LATS2,MYH14,LYN,NME7,FIGNL1,NMNAT3,RNASEL,ERCC6,EIF2AK4,TGFBR2,MARK4,DDX60L,MDN1,ULK3,DAPK1,ACSM2B,UBE2G1,ATP9B,DHX35,HCN1,PI4KB,RIPK4,PTK2,DNAH12,PIP4K2B,LRRK2,ACVR2A,KIT,EPHA7,PRKDC,FIGN,PARS2,HUNK,NEK10,ACTR3B,IKBKB,ACVR1,PDE4B,DDX10,KIF13A,NLRP4,AK5,CPS1,PRKCH,ACACB,XRCC5,BMX,RAD54L2,NRK,SIK1,ERCC6L2,ACTR3C,SUPV3L1,MYO1B,UBE2E3,FARSB,KIF16B,NEK7,CDK6,DDX60,MYLK4,KIF2C,MYO5A,PNPLA8,MAP3K1,DDX4,RAPGEF4,EIF4G1,ACSM5,ROCK1,KCNT2,DGKG,PRKG1,GALK2,TPK1,PRKD1,STK4,TNNI3K,PCCA,TTN,DYRK1A,ETNK1,ATP8B1*
GO:0016301	kinase activity	0.0000844281269699483 2	*PFKP,STYK1,CDK14,MAP3K3,ABR,MAP3K13,MAPK10,BRD4,CAMK2B,TLK1,KALRN,MAP4K3,FPGT-TNNI3K,STK39,CDK18,STK24,FAM20B,PIK3C2B,NLK,MAPKAPK3,STK32B,BRAF,ALPK2,TRIO,MUSK,MAP3K5,PRKAR2A,PRKG2,PIK3C3,RSKR,EPHA5,STRADA,PAK3,IPMK,CAMK1G,PRKAG2,CILK1,EPHA3,LATS2,LYN,NME7,RNASEL,SGMS2,EIF2AK4,TGFBR2,MARK4,ULK3,GKAP1,DAPK1,PI4KB,PIK3R5,RIPK4,PTK2,PIP4K2B,LRRK2,ACVR2A,KIT,EPHA7,PRKDC,HUNK,NEK10,IKBKB,ACVR1,AK5,PRKCH,BMX,NRK,SIK1,NEK7,CDK6,SH3KBP1,MYLK4,MAP3K1,ROCK1,DGKG,PRKG1,MAGI2,GALK2,TPK1,PRKD1,STK4,TNNI3K,TTN,DYRK1A,ETNK1*
GO:0005524	ATP binding	0.0001541316109640162 8	*PFKP,ABCC6,STYK1,CDK14,MAP3K3,GARS1,MAP3K13,MYO5C,MAPK10,ATP10A,ATP7B,CAMK2B,TLK1,ABCG8,KALRN,MAP4K3,FPGT-TNNI3K,STK39,CDK18,STK24,FAM20B,PIK3C2B,CHD7,NLK,MAPKAPK3,PAPOLG,UBE2O,DNAH10,CLCN3,NLRP8,STK32B,UBE2E2,PMS1,WRNIP1,MYO1D,BRAF,ALPK2,ATRX,TRIO,RAD51B,MUSK,MAP3K5,ATP11A,ATP4A,PRKG2,PIK3C3,RSKR,EPHA5,ATP13A4,RUNX1,ATP2C1,STRADA,KIF21A,NLRP7,PAK3,IPMK,CAMK1G,PRKAG2,CILK1,EPHA3,LATS2,MYH14,LYN,NME7,FIGNL1,NMNAT3,RNASEL,ERCC6,EIF2AK4,TGFBR2,MARK4,DDX60L,MDN1,ULK3,DAPK1,ACSM2B,UBE2G1,ATP9B,DHX35,PI4KB,RIPK4,PTK2,DNAH12,PIP4K2B,LRRK2,ACVR2A,KIT,EPHA7,PRKDC,FIGN,PARS2,HUNK,NEK10,ACTR3B,IKBKB,ACVR1,DDX10,KIF13A,NLRP4,AK5,CPS1,PRKCH,ACACB,XRCC5,BMX,RAD54L2,NRK,SIK1,ERCC6L2,ACTR3C,SUPV3L1,MYO1B,UBE2E3,FARSB,KIF16B,NEK7,CDK6,DDX60,MYLK4,KIF2C,MYO5A,PNPLA8,MAP3K1,DDX4,EIF4G1,ACSM5,ROCK1,*

			KCNT2,DGKG,PRKG1,GALK2,TPK1,PRKD1,STK4,TNNI3K,PCCA,TTN,DYRK1A,ETNK1,ATP8B1
GO:0004672	protein kinase activity	0.0001576680479374508 8	*STYK1,CDK14,MAP3K3,ABR,MAP3K13,MAPK10,BRD4,CAMK2B,TLK1,KALRN,MAP4K3,FPGT-TNNI3K,STK39,CDK18,STK24,NLK,MAPKAPK3,STK32B,BRAF,ALPK2,TRIO,MUSK,MAP3K5,PRKG2,PIK3C3,RSKR,EPHA5,STRADA,PAK3,CAMK1G,PRKAG2,CILK1,EPHA3,LATS2,LYN,NME7,RNASEL,EIF2AK4,TGFBR2,MARK4,ULK3,DAPK1,RIPK4,PTK2,LRRK2,ACVR2A,KIT,EPHA7,PRKDC,HUNK,NEK10,IKBKB,ACVR1,PRKCH,BMX,NRK,SIK1,NEK7,CDK6,MYLK4,MAP3K1,ROCK1,PRKG1,PRKD1,STK4,TNNI3K,TTN,DYRK1A*
GO:0106310	protein serine kinase activity	0.0001759861873202771	*CDK14,MAP3K3,MAP3K13,MAPK10,CAMK2B,TLK1,KALRN,MAP4K3,STK39,CDK18,STK24,NLK,MAPKAPK3,STK32B,BRAF,ALPK2,TRIO,MAP3K5,PRKG2,RSKR,PAK3,CAMK1G,CILK1,LATS2,EIF2AK4,MARK4,ULK3,DAPK1,RIPK4,LRRK2,PRKDC,HUNK,NEK10,IKBKB,PRKCH,NRK,SIK1,NEK7,CDK6,MYLK4,MAP3K1,ROCK1,PRKG1,PRKD1,STK4,TNNI3K,TTN,DYRK1A*
GO:0016773	phosphotransferase activity, alcohol group as acceptor	0.0001810292140354541 6	*PFKP,STYK1,CDK14,MAP3K3,ABR,MAP3K13,MAPK10,BRD4,CAMK2B,TLK1,KALRN,MAP4K3,FPGT-TNNI3K,STK39,CDK18,STK24,FAM20B,PIK3C2B,NLK,MAPKAPK3,STK32B,BRAF,ALPK2,TRIO,MUSK,MAP3K5,PRKG2,PIK3C3,RSKR,EPHA5,STRADA,PAK3,IPMK,CAMK1G,PRKAG2,CILK1,EPHA3,LATS2,LYN,NME7,RNASEL,EIF2AK4,TGFBR2,MARK4,ULK3,DAPK1,PI4KB,RIPK4,PTK2,PIP4K2B,LRRK2,ACVR2A,KIT,EPHA7,PRKDC,HUNK,NEK10,IKBKB,ACVR1,PRKCH,BMX,NRK,SIK1,NEK7,CDK6,MYLK4,MAP3K1,ROCK1,DGKG,PRKG1,GALK2,PRKD1,STK4,TNNI3K,TTN,DYRK1A,ETNK1*
GO:0032553	ribonucleotide binding	0.0001898076585200130 5	*PFKP,ABCC6,STYK1,CDK14,MAP3K3,GARS1,MAP3K13,ARL13B,MYO5C,MAPK10,ATP10A,ATP7B,CAMK2B,TLK1,ABCG8,KALRN,MAP4K3,FPGT-TNNI3K,STK39,SRL,CDK18,STK24,FAM20B,PIK3C2B,CHD7,NLK,GNAI1,MAPKAPK3,PAPOLG,UBE2O,DNAH10,PDE10A,CLCN3,RHOJ,NLRP8,STK32B,UBE2E2,PMS1,WRNIP1,TYW1,SEPTIN10,MYO1D,BRAF,ALPK2,ATRX,TRIO,RAD51B,MUSK,MAP3K5,RERG,PRKAR2A,ATP11A,ATP4A,PRKG2,PIK3C3,RSKR,EPHA5,ENSG00000289694,ATP13A4,RUNX1,ATP2C1,STRADA,RRAS2,KIF21A,NLRP7,PAK3,IPMK,CAMK1G,PRKAG2,CILK1,EPHA3,LATS2,MYH14,LYN,NME7,FIGNL1,NMNAT3,RNASEL,NOS3,ERCC6,EIF2AK4,RAB12,TGFBR2,MARK4,DDX60L,MDN1,RRAGD,ULK3,SCG5,DAPK1,ACSM2B,SAR1B,UBE2G1,ATP9B,DHX35,HCN1,PI4KB,RHOT1,RIPK4,PTK2,DNAH12,PIP4K2B,LRRK2,ACVR2A,GNAO1,KIT,EPHA7,PRKDC,FIGN,PARS2,HUNK,NEK10,ACTR3B,IKBKB,ACVR1,PDE4B,DDX10,KIF13A,NLRP4,AK5,CPS1,PRKCH,ACACB,XRCC5,BMX,RAD54L2,RAB18,NRK,SIK1,ERCC6L2,ACTR3C,SUPV3L1,MYO1B,UBE2E3,FARSB,KIF16B,EEFSEC,NEK7,CDK6,DDX60,MYLK4,KIF2C,MYO5A,PNPLA8,RAB31,MAP3K1,DDX4,RAPGEF4,EIF4G1,ACSM5,ROCK1,KCN*

			T2,DGKG,PRKG1,GALK2,NIN,TPK1,PRKD1,STK4,TNNI3K,RAB6D,PDE11A,RAB6C,PCCA,GNA14,TTN,DYRK1A,ARL15,ETNK1,ATP8B1
GO:0017076	purine nucleotide binding	0.000246296947446705 25	*PFKP,ABCC6,STYK1,CDK14,MAP3K3,GARS1,MAP3K13,ARL13B,MYO5C,MAPK10,ATP10A,ATP7B,CAMK2B,TLK1,ABCG8,KALRN,MAP4K3,FPGT-TNNI3K,STK39,SRL,CDK18,STK24,FAM20B,PIK3C2B,CHD7,NLK,GNAI1,MAPKAPK3,PAPOLG,UBE2O,DNAH10,PDE10A,CLCN3,RHOJ,NLRP8,STK32B,UBE2E2,PMS1,WRNIP1,SEPTIN10,MYO1D,BRAF,ALPK2,ATRX,TRIO,RAD51B,MUSK,MAP3K5,RERG,PRKAR2A,ATP11A,ATP4A,PRKG2,PIK3C3,RSKR,EPHA5,CBR4,ENSG00000289694,ATP13A4,RUNX1,ATP2C1,STRADA,RRAS2,KIF21A,NLRP7,PAK3,IPMK,CAMK1G,PRKAG2,CILK1,EPHA3,LATS2,MYH14,LYN,NME7,FIGNL1,ALDH1A1,NMNAT3,FMO5,RNASEL,NOS3,ERCC6,EIF2AK4,RAB12,TGFBR2,MARK4,DDX60L,MDN1,RRAGD,ULK3,SCG5,DAPK1,ACSM2B,SAR1B,RNLS,UBE2G1,ATP9B,DHX35,HCN1,PI4KB,RHOT1,RIPK4,QDPR,PTK2,DNAH12,PIP4K2B,LRRK2,ACVR2A,GNAO1,KIT,EPHA7,PRKDC,FIGN,PARS2,HUNK,NEK10,ACTR3B,IKBKB,ACVR1,PDE4B,DDX10,KIF13A,NLRP4,AK5,CPS1,PRKCH,ACACB,XRCC5,BMX,RAD54L2,RAB18,NRK,SIK1,ERCC6L2,ACTR3C,SUPV3L1,MYO1B,UBE2E3,FARSB,KIF16B,EEFSEC,NEK7,CDK6,DDX60,MYLK4,KIF2C,MYO5A,PNPLA8,RAB31,MAP3K1,DDX4,RAPGEF4,EIF4G1,ACSM5,ROCK1,KCNT2,DGKG,PRKG1,GALK2,NIN,TPK1,PRKD1,STK4,TNNI3K,RAB6D,PDE11A,RAB6C,PCCA,GNA14,CTBP2,TTN,DYRK1A,ARL15,ETNK1,ATP8B1*
GO:0032555	purine ribonucleotide binding	0.000271268770474406 57	*PFKP,ABCC6,STYK1,CDK14,MAP3K3,GARS1,MAP3K13,ARL13B,MYO5C,MAPK10,ATP10A,ATP7B,CAMK2B,TLK1,ABCG8,KALRN,MAP4K3,FPGT-TNNI3K,STK39,SRL,CDK18,STK24,FAM20B,PIK3C2B,CHD7,NLK,GNAI1,MAPKAPK3,PAPOLG,UBE2O,DNAH10,PDE10A,CLCN3,RHOJ,NLRP8,STK32B,UBE2E2,PMS1,WRNIP1,SEPTIN10,MYO1D,BRAF,ALPK2,ATRX,TRIO,RAD51B,MUSK,MAP3K5,RERG,PRKAR2A,ATP11A,ATP4A,PRKG2,PIK3C3,RSKR,EPHA5,ENSG00000289694,ATP13A4,RUNX1,ATP2C1,STRADA,RRAS2,KIF21A,NLRP7,PAK3,IPMK,CAMK1G,PRKAG2,CILK1,EPHA3,LATS2,MYH14,LYN,NME7,FIGNL1,NMNAT3,RNASEL,ERCC6,EIF2AK4,RAB12,TGFBR2,MARK4,DDX60L,MDN1,RRAGD,ULK3,SCG5,DAPK1,ACSM2B,SAR1B,UBE2G1,ATP9B,DHX35,HCN1,PI4KB,RHOT1,RIPK4,PTK2,DNAH12,PIP4K2B,LRRK2,ACVR2A,GNAO1,KIT,EPHA7,PRKDC,FIGN,PARS2,HUNK,NEK10,ACTR3B,IKBKB,ACVR1,PDE4B,DDX10,KIF13A,NLRP4,AK5,CPS1,PRKCH,ACACB,XRCC5,BMX,RAD54L2,RAB18,NRK,SIK1,ERCC6L2,ACTR3C,SUPV3L1,MYO1B,UBE2E3,FARSB,KIF16B,EEFSEC,NEK7,CDK6,DDX60,MYLK4,KIF2C,MYO5A,PNPLA8,RAB31,MAP3K1,DDX4,RAPGEF4,EIF4G1,ACSM5,ROCK1,KCNT2,DGKG,PRKG1,GALK2,NIN,TPK1,PRKD1,STK4,TNNI3K,RAB6D,PDE11A,RAB6C,PCCA,GNA14,TTN,DYRK1A,ARL15,ETNK1,ATP8B1*
GO:0043	anion	0.00040864	*PFKP,ABCC6,STYK1,CDK14,MAP3K3,GARS1,RA*

168	binding	244023080685	*PGEF6,MAOA,MAP3K13,ARL13B,MYO5C,MAPK10,DAPP1,ATP10A,SH3PXD2A,ATP7B,CAMK2B,TLK1,SLC38A9,ABCG8,KALRN,MAP4K3,FPGT-TNNI3K,SPTLC3,STK39,SRL,CDK18,STK24,FAM20B,NLGN4X,PNPLA3,PIK3C2B,CHD7,NLK,GNAI1,MAPKAPK3,PAPOLG,UBE2O,DNAH10,PDE10A,CLCN3,RHOJ,ZFYVE16,NLRP8,STK32B,UBE2E2,PMS1,WRNIP1,TYW1,COL11A1,SEPTIN10,CIDEB,MYO1D,SESTD1,BRAF,ALPK2,ATRX,TRIO,RAD51B,MUSK,MAP3K5,RERG,PRKAR2A,ATP11A,ATP4A,PRKG2,PIK3C3,RSKR,EPHA5,CBR4,ENSG00000289694,ATP13A4,RUNX1,ATP2C1,STRADA,RRAS2,GSN,KIF21A,NLRP7,ITPR1,PAK3,IPMK,CAMK1G,PRKAG2,CILK1,EPHA3,LATS2,MYH14,LYN,NME7,FIGNL1,NMNAT3,FMO5,RNASEL,NOS3,ERCC6,EIF2AK4,RAB12,TGFBR2,MARK4,DDX60L,MDN1,RRAGD,ULK3,GSDMD,GAD1,SCG5,DAPK1,ACSM2B,SAR1B,RNLS,UBE2G1,ATP9B,DHX35,HCN1,PI4KB,RHOT1,RIPK4,QDPR,PTK2,SCP2,DNAH12,PIP4K2B,LRRK2,ACVR2A,GNAO1,KIT,EPHA7,WIPI2,PRKDC,FIGN,PARS2,HUNK,NEK10,ACTR3B,TRPC5,IKBKB,ACVR1,PDE4B,DDX10,KIF13A,NLRP4,AK5,CPS1,PRKCH,ACACB,XRCC5,BMX,RAD54L2,RAB18,NRK,SIK1,ERCC6L2,PIPOX,ACTR3C,SUPV3L1,MYO1B,SPTLC1,UBE2E3,FARSB,ACBD6,SCIN,KIF16B,EEFSEC,PPARG,NEK7,CDK6,AVIL,AMY2A,DDX60,MYLK4,KIF2C,MYO5A,PNPLA8,RAB31,MAP3K1,DDX4,RAPGEF4,EIF4G1,ACSM5,ROCK1,KCNT2,PLEK2,DGKG,PRKG1,FOXRED2,GALK2,NIN,TPK1,PRKD1,STK4,TNNI3K,RAB6D,PDE11A,RAB6C,PCCA,CLVS1,GNA14,TTN,DYRK1A,TXNRD3,ARL15,ETNK1,TTPA,PARD3,ATP8B1*
GO:0004674	protein serine/threonine kinase activity	0.000416471221372533 44	*CDK14,MAP3K3,ABR,MAP3K13,MAPK10,BRD4,CAMK2B,TLK1,KALRN,MAP4K3,STK39,CDK18,STK24,NLK,MAPKAPK3,STK32B,BRAF,ALPK2,TRIO,MAP3K5,PRKG2,RSKR,PAK3,CAMK1G,PRKAG2,CILK1,LATS2,EIF2AK4,TGFBR2,MARK4,ULK3,DAPK1,RIPK4,LRRK2,ACVR2A,PRKDC,HUNK,NEK10,IKBKB,ACVR1,PRKCH,NRK,SIK1,NEK7,CDK6,MYLK4,MAP3K1,ROCK1,PRKG1,PRKD1,STK4,TNNI3K,TTN,DYRK1A*
GO:0097367	carbohydrate derivative binding	0.000861282442340115 3	*FSTL1,PFKP,ABCC6,STYK1,CDK14,MAP3K3,GARS1,MAP3K13,ARL13B,MYO5C,MAPK10,FGF1,ATP10A,ATP7B,CAMK2B,TLK1,ABCG8,KALRN,MAP4K3,FPGT-TNNI3K,STK39,SRL,CDK18,STK24,FAM20B,PNPLA3,PIK3C2B,CHD7,NLK,GNAI1,MAPKAPK3,PAPOLG,UBE2O,DNAH10,PDE10A,CLCN3,RHOJ,FGF10,NLRP8,STK32B,UBE2E2,PMS1,WRNIP1,TYW1,COL11A1,SEPTIN10,MYO1D,BRAF,ALPK2,HMGB1,ATRX,TRIO,RAD51B,MUSK,MAP3K5,RERG,PRKAR2A,ATP11A,ATP4A,PRKG2,PIK3C3,RSKR,EPHA5,ENSG00000289694,ATP13A4,RUNX1,ATP2C1,STRADA,RRAS2,SPOCK3,KIF21A,SLIT3,NLRP7,PAK3,ABI3BP,IPMK,CAMK1G,PRKAG2,CILK1,EPHA3,LATS2,MYH14,LYN,NME7,FIGNL1,NMNAT3,RNASEL,NOS3,ERCC6,EIF2AK4,RAB12,IMPG1,TGFBR2,MARK4,DDX60L,MDN1,RRAGD,ULK3,EVA1C,SCG5,DAPK1,ACSM2B,SAR1B,APP,UBE2G1,ATP9B,DHX35,HCN1,PI4KB,RHO*

			T1,RIPK4,PTK2,DNAH12,PIP4K2B,LRRK2,ACVR2A,GNAO1,VIT,KIT,EPHA7,CTSK,PRKDC,FIGN,PARS2,HUNK,NEK10,ACTR3B,PTPRF,IKBKB,LYVE1,APOH,CEMIP,ACVR1,PDE4B,DDX10,KIF13A,NLRP4,AK5,CPS1,PRKCH,ACACB,XRCC5,BMX,RAD54L2,RAB18,NRK,LRTM2,SIK1,ERCC6L2,ACTR3C,SUPV3L1,MYO1B,UBE2E3,FARSB,KIF16B,EEFSEC,NEK7,TENM1,CDK6,DDX60,MYLK4,KIF2C,MYO5A,PNPLA8,FBN1,RAB31,MAP3K1,DDX4,RAPGEF4,EIF4G1,ACSM5,ROCK1,KCNT2,LAYN,DGKG,PRKG1,GALK2,NIN,TPK1,PRKD1,STK4,TNNI3K,RAB6D,PDE11A,RAB6C,PCCA,GNA14,TTN,DYRK1A,ARL15,SEMA5A,ETNK1,ATP8B1
GO:0035639	purine ribonucleoside triphosphate binding	0.000888480305036404	*PFKP,ABCC6,STYK1,CDK14,MAP3K3,GARS1,MAP3K13,ARL13B,MYO5C,MAPK10,ATP10A,ATP7B,CAMK2B,TLK1,ABCG8,KALRN,MAP4K3,FPGT-TNNI3K,STK39,SRL,CDK18,STK24,FAM20B,PIK3C2B,CHD7,NLK,GNAI1,MAPKAPK3,PAPOLG,UBE2O,DNAH10,CLCN3,RHOJ,NLRP8,STK32B,UBE2E2,PMS1,WRNIP1,SEPTIN10,MYO1D,BRAF,ALPK2,ATRX,TRIO,RAD51B,MUSK,MAP3K5,RERG,ATP11A,ATP4A,PRKG2,PIK3C3,RSKR,EPHA5,ENSG00000289694,ATP13A4,RUNX1,ATP2C1,STRADA,RRAS2,KIF21A,NLRP7,PAK3,IPMK,CAMK1G,PRKAG2,CILK1,EPHA3,LATS2,MYH14,LYN,NME7,FIGNL1,NMNAT3,RNASEL,ERCC6,EIF2AK4,RAB12,TGFBR2,MARK4,DDX60L,MDN1,RRAGD,ULK3,SCG5,DAPK1,ACSM2B,SAR1B,UBE2G1,ATP9B,DHX35,PI4KB,RHOT1,RIPK4,PTK2,DNAH12,PIP4K2B,LRRK2,ACVR2A,GNAO1,KIT,EPHA7,PRKDC,FIGN,PARS2,HUNK,NEK10,ACTR3B,IKBKB,ACVR1,DDX10,KIF13A,NLRP4,AK5,CPS1,PRKCH,ACACB,XRCC5,BMX,RAD54L2,RAB18,NRK,SIK1,ERCC6L2,ACTR3C,SUPV3L1,MYO1B,UBE2E3,FARSB,KIF16B,EEFSEC,NEK7,CDK6,DDX60,MYLK4,KIF2C,MYO5A,PNPLA8,RAB31,MAP3K1,DDX4,EIF4G1,ACSM5,ROCK1,KCNT2,DGKG,PRKG1,GALK2,NIN,TPK1,PRKD1,STK4,TNNI3K,RAB6D,RAB6C,PCCA,GNA14,TTN,DYRK1A,ARL15,ETNK1,ATP8B1*
GO:1901265	nucleoside phosphate binding	0.001659435648679319 4	*PFKP,ABCC6,STYK1,CDK14,MAP3K3,GARS1,MAOA,MAP3K13,ARL13B,MYO5C,MAPK10,ATP10A,ATP7B,CAMK2B,TLK1,ABCG8,KALRN,MAP4K3,FPGT-TNNI3K,STK39,SRL,CDK18,STK24,FAM20B,PNPLA3,PIK3C2B,CHD7,NLK,GNAI1,MAPKAPK3,PAPOLG,UBE2O,DNAH10,PDE10A,CLCN3,RHOJ,NLRP8,STK32B,UBE2E2,PMS1,WRNIP1,TYW1,SEPTIN10,MYO1D,BRAF,ALPK2,ATRX,TRIO,RAD51B,MUSK,MAP3K5,RERG,PRKAR2A,ATP11A,ATP4A,PRKG2,PIK3C3,RSKR,EPHA5,CBR4,ENSG00000289694,ATP13A4,RUNX1,ATP2C1,STRADA,RRAS2,KIF21A,NLRP7,PAK3,IPMK,CAMK1G,PRKAG2,CILK1,EPHA3,LATS2,MYH14,LYN,NME7,FIGNL1,ALDH1A1,NMNAT3,FMO5,RNASEL,NOS3,ERCC6,EIF2AK4,RAB12,TGFBR2,MARK4,DDX60L,MDN1,RRAGD,ULK3,SCG5,DAPK1,ACSM2B,SAR1B,RNLS,UBE2G1,ATP9B,DHX35,HCN1,PI4KB,RHOT1,RIPK4,QDPR,PTK2,SCP2,DNAH12,PIP4K2B,LRRK2,ACVR2A,GNAO1,KIT,EPHA7,PRK*

			DC,FIGN,PARS2,HUNK,NEK10,ACTR3B,IKBKB,ACVR1,PDE4B,DDX10,KIF13A,NLRP4,AK5,CPS1,PRKCH,ACACB,XRCC5,BMX,RAD54L2,RAB18,NRK,SIK1,ERCC6L2,PIPOX,ACTR3C,SUPV3L1,MYO1B,UBE2E3,FARSB,ACBD6,KIF16B,EEFSEC,NEK7,CDK6,DDX60,MYLK4,KIF2C,MYO5A,PNPLA8,RAB31,MAP3K1,DDX4,RAPGEF4,EIF4G1,ACSM5,ROCK1,KCNT2,DGKG,PRKG1,FOXRED2,GALK2,NIN,TPK1,PRKD1,STK4,TNNI3K,RAB6D,PDE11A,RAB6C,PCCA,GNA14,CTBP2,TTN,DYRK1A,TXNRD3,ARL15,ETNK1,ATP8B1,VDAC2
GO:0140096	catalytic activity, acting on a protein	0.002936347754393824	*C10ORF90,STYK1,CDK14,ING3,MAP3K3,SMYD3,ABR,PREP,MAP3K13,PPM1A,IMMP1L,USP42,MAPK10,USP32,CDC14C,AGTPBP1,PPM1H,BRD4,CAMK2B,TLK1,DDI2,OTUD7A,KALRN,MAP4K3,FPGT-TNNI3K,GALNT9,FKBP5,STYXL1,STK39,SMURF2,ADAMTS12,CDK18,STK24,ARMT1,ASB4,PRKN,RMND5A,F13A1,KDM7A,MARCHF9,CBLL2,NLK,MAPKAPK3,ENPEP,UBE2O,MBTPS2,MECOM,STK32B,PLG,NEURL4,HERC5,PGPEP1L,UBE2E2,KLHL42,KDM2B,MARCHF1,BRAF,EEF2KMT,ALPK2,HECW1,TRIO,MUSK,MAP3K5,PRKG2,MBTPS1,PIK3C3,USP10,RSKR,EPHA5,PTPN20,PPTC7,STRADA,HUWE1,ASPH,ZDHHC4,UBR5,CPM,PAK3,NDUFAF7,CAMK1G,PRKAG2,CPQ,CILK1,EPHA3,LATS2,PTPRN2,LYN,USP6,SUMO2,NME7,FIGNL1,PAMR1,RNASEL,CORIN,PPM1L,EIF2AK4,MAGEL2,TGFBR2,MARK4,OVCH1,ULK3,DAPK1,PPIL6,RNF38,ADAM12,UBE2G1,PELI2,HERC2,RIPK4,PTK2,USP7,LRRK2,ARIH1,PJA2,COP1,ACVR2A,NEDD4L,BRCC3,DUSP14,PRMT8,RABGEF1,KIT,EPHA7,TRABD2B,CTSK,PRKDC,PTPN14,POFUT2,PPM1E,TPST2,FIGN,HUNK,NEK10,USP40,CDC14B,HECTD1,KAT14,UBE3B,PTPRF,IKBKB,ATF2,HDAC8,ACVR1,NSMCE1,IMMP2L,CPS1,PRKCH,BMX,TPST1,TMTC1,NRK,MGRN1,SIK1,KAT2B,TMTC2,TMPRSS11B,PRCP,UBE2E3,ADAMTS9,PAPPA,USP9X,TGM5,NEK7,CDK6,MYLK4,USP25,MAP3K1,TMPRSS15,PHLPP1,DNAJC6,ROCK1,MMP28,NUP98,PML,LONRF3,PRKG1,RFPL4B,PRSS50,PRKD1,USP34,STK4,TNNI3K,ANPEP,PTPRA,TTN,DYRK1A,CUL4A,TXNRD3,EYA4,UBE3C,ATE1,PGPEP1,RNF6,SMYD1,PEPD*
GO:0016772	transferase activity, transferring phosphorus-containing groups	0.002977295718647698 7	*PFKP,STYK1,CDK14,MAP3K3,ABR,MAP3K13,MAPK10,BRD4,CAMK2B,TLK1,KALRN,MAP4K3,FPGT-TNNI3K,STK39,CDK18,STK24,FAM20B,PIK3C2B,NLK,MAPKAPK3,PAPOLG,STK32B,BRAF,ALPK2,TRIO,MUSK,MAP3K5,PRKAR2A,PRKG2,PIK3C3,RSKR,EPHA5,STRADA,PAK3,IPMK,CAMK1G,PRKAG2,OASL,CILK1,EPHA3,LATS2,LYN,NME7,NMNAT3,RNASEL,SGMS2,EIF2AK4,TGFBR2,MARK4,POLR2C,ULK3,GKAP1,DAPK1,PI4KB,PIK3R5,RIPK4,PTK2,PIP4K2B,LRRK2,TENT5C,ACVR2A,KIT,EPHA7,PRKDC,HUNK,NEK10,IKBKB,ACVR1,AK5,PRKCH,BMX,POLD3,NRK,SIK1,TENT4B,NEK7,CDK6,SH3KBP1,MYLK4,MAP3K1,ROCK1,DGKG,PRKG1,MAGI2,GALK2,TPK1,PRKD1,STK4,TNNI3K,TTN,DYRK1A,ETNK1,UAP1*
GO:0000	nucleotide	0.00334991	*PFKP,ABCC6,STYK1,CDK14,MAP3K3,GARS1,MA*

166	binding	97729257996	*OA,MAP3K13,ARL13B,MYO5C,MAPK10,ATP10A,ATP7B,CAMK2B,TLK1,ABCG8,KALRN,MAP4K3,FPGT-TNNI3K,STK39,SRL,CDK18,STK24,FAM20B,PIK3C2B,CHD7,NLK,GNAI1,MAPKAPK3,PAPOLG,UBE2O,DNAH10,PDE10A,CLCN3,RHOJ,NLRP8,STK32B,UBE2E2,PMS1,WRNIP1,TYW1,SEPTIN10,MYO1D,BRAF,ALPK2,ATRX,TRIO,RAD51B,MUSK,MAP3K5,RERG,PRKAR2A,ATP11A,ATP4A,PRKG2,PIK3C3,RSKR,EPHA5,CBR4,ENSG00000289694,ATP13A4,RUNX1,ATP2C1,STRADA,RRAS2,KIF21A,NLRP7,PAK3,IPMK,CAMK1G,PRKAG2,CILK1,EPHA3,LATS2,MYH14,LYN,NME7,FIGNL1,ALDH1A1,NMNAT3,FMO5,RNASEL,NOS3,ERCC6,EIF2AK4,RAB12,TGFBR2,MARK4,DDX60L,MDN1,RRAGD,ULK3,SCG5,DAPK1,ACSM2B,SAR1B,RNLS,UBE2G1,ATP9B,DHX35,HCN1,PI4KB,RHOT1,RIPK4,QDPR,PTK2,DNAH12,PIP4K2B,LRRK2,ACVR2A,GNAO1,KIT,EPHA7,PRKDC,FIGN,PARS2,HUNK,NEK10,ACTR3B,IKBKB,ACVR1,PDE4B,DDX10,KIF13A,NLRP4,AK5,CPS1,PRKCH,ACACB,XRCC5,BMX,RAD54L2,RAB18,NRK,SIK1,ERCC6L2,PIPOX,ACTR3C,SUPV3L1,MYO1B,UBE2E3,FARSB,KIF16B,EEFSEC,NEK7,CDK6,DDX60,MYLK4,KIF2C,MYO5A,PNPLA8,RAB31,MAP3K1,DDX4,RAPGEF4,EIF4G1,ACSM5,ROCK1,KCNT2,DGKG,PRKG1,FOXRED2,GALK2,NIN,TPK1,PRKD1,STK4,TNNI3K,RAB6D,PDE11A,RAB6C,PCCA,GNA14,CTBP2,TTN,DYRK1A,TXNRD3,ARL15,ETNK1,ATP8B1,VDAC2*
GO:1901363	heterocyclic compound binding	0.004324922540369061	*PFKP,ABCC6,STYK1,CDK14,MAP3K3,GARS1,MAOA,MAP3K13,ARL13B,MYO5C,MAPK10,CHRNB2,ATP10A,ATP7B,CAMK2B,TLK1,ABCG8,KALRN,MAP4K3,FPGT-TNNI3K,SPTLC3,STK39,SRL,CDK18,STK24,FAM20B,PNPLA3,PIK3C2B,CHD7,NLK,GNAI1,MAPKAPK3,PAPOLG,UBE2O,DNAH10,PDE10A,CLCN3,RHOJ,NLRP8,STK32B,UBE2E2,PMS1,WRNIP1,TYW1,SEPTIN10,MYO1D,BRAF,ALPK2,ATRX,TRIO,RAD51B,MUSK,MAP3K5,RERG,PRKAR2A,ATP11A,ATP4A,PRKG2,PIK3C3,RSKR,EPHA5,CBR4,ENSG00000289694,ATP13A4,RUNX1,ATP2C1,STRADA,RRAS2,KIF21A,NLRP7,PAK3,IPMK,CAMK1G,PRKAG2,CILK1,EPHA3,LATS2,MYH14,LYN,NME7,FIGNL1,ALDH1A1,NMNAT3,FMO5,RNASEL,NOS3,ERCC6,EIF2AK4,RAB12,TGFBR2,MARK4,DDX60L,MDN1,RRAGD,ULK3,GAD1,SCG5,DAPK1,ACSM2B,SAR1B,RNLS,UBE2G1,ATP9B,DHX35,HCN1,PI4KB,RHOT1,RIPK4,QDPR,PTK2,SCP2,DNAH12,PIP4K2B,LRRK2,RARB,ACVR2A,GNAO1,KIT,EPHA7,PRKDC,FIGN,PARS2,HUNK,NEK10,ACTR3B,IKBKB,CHRNB3,ACVR1,PDE4B,DDX10,KIF13A,NLRP4,AK5,CPS1,PRKCH,ACACB,XRCC5,BMX,RAD54L2,RAB18,NRK,SIK1,ERCC6L2,PIPOX,ACTR3C,SUPV3L1,MYO1B,SPTLC1,UBE2E3,FARSB,ACBD6,KIF16B,EEFSEC,NEK7,CDK6,DDX60,MYLK4,KIF2C,MYO5A,PNPLA8,RAB31,MAP3K1,DDX4,RAPGEF4,EIF4G1,ACSM5,ROCK1,KCNT2,DGKG,PRKG1,FOXRED2,GALK2,NIN,TPK1,PRKD1,STK4,TNNI3K,RAB6D,PDE11A,RAB6C,PCCA,GNA14,CTBP2,TTN,DYRK1A,TXNRD*

			3,ARL15,ETNK1,TTPA,ATP8B1,VDAC2
GO:0022835	transmitter-gated channel activity	0.00814674704795571	*CHRNB2,CHRFAM7A,GABRA5,CHRNA7,GRIK1,CHRNB3,GRID2,GABRB3,GABRG2,GRIA4,CHRNB4,GABRB1,GRIA3,GRIA2*
GO:0022824	transmitter-gated monoatomic ion channel activity	0.00814674704795571	*CHRNB2,CHRFAM7A,GABRA5,CHRNA7,GRIK1,CHRNB3,GRID2,GABRB3,GABRG2,GRIA4,CHRNB4,GABRB1,GRIA3,GRIA2*
GO:0005216	monoatomic ion channel activity	0.008199803914990702	*CACNB1,CHRNB2,KCNQ5,KCNJ6,CACNA1C,SLC17A6,CHRFAM7A,CLCN3,GABRA5,KCNQ3,ANO3,KCNS3,CHRNA7,KCNJ18,KCNIP3,ANO2,ITPR1,CACNA2D3,GRIK1,KCNIP4,CACNA2D4,CACNA2D1,HCN1,BEST3,TRPC5,CLCN1,KCNJ12,CHRNB3,GRID2,KCNJ3,PKD1L3,GABRB3,UNC80,GABRG2,TMEM120A,KCND2,SLC24A3,GRIA4,RYR3,SLC26A8,CACHD1,CHRNB4,GABRB1,NALCN,GRIA3,KCNT2,ASIC2,KCNE2,KCNN4,GRIA2,VDAC2*
GO:0003779	actin binding	0.008199803914990702	*MYBPC1,IPP,RCSD1,SPTBN1,MYO5C,CAMK2B,TMOD2,EPB41L3,PARVB,DAAM2,PRKN,TAGLN3,MYO1D,TMOD3,MRTFB,ABLIM1,ADD3,ENAH,GSN,PLS1,PACRG,MYH14,SPTB,NOS3,PALLD,PTK2,LRRK2,CSRP3,PHACTR1,SNTG2,ANTXR1,DIAPH3,ACTR3B,SYNE3,TRPC5,SHROOM4,MPRIP,ACTR3C,MYO1B,MYL4,CAPZA2,SNTG1,SCIN,AVIL,MYO5A,SPTBN5,TTN,NCALD,DYRK1A,MYRIP,FMNL2*
GO:0019899	enzyme binding	0.010584955607603412	*STX8,SMYD3,RAPGEF6,MAP3K13,THRB,SPTBN1,CDC42EP3,ATF7,SH3PXD2A,BRD4,XPO4,NOTCH2,NR4A3,FOXO1,STYXL1,DAAM2,YBX1,ARMT1,ASB4,PRKN,XPO6,FAM83B,NLK,MAPKAPK3,EIF4ENIF1,CD247,CEP192,RHOJ,CDC37,PLG,NCK1,MTCP1,PREX1,PMS1,BRAF,COL1A2,XPO7,HMGB1,ADD3,SFPQ,MAP3K5,PRKAR2A,GOLPH3,PRKG2,NCF2,SKOR2,SNX10,STRADA,CHL1,GSN,MAPRE3,CACUL1,NLRP7,PAK3,NDUFAF7,PRKAG2,CCNT2,NCKAP1,PRPF40A,PACRG,CHCHD3,SPDYE2B,MYOM2,LYN,SUMO2,MTA3,PUS7,LDB2,DBF4B,NOS1AP,TCF7L2,ZFHX3,RRAGD,SPDYE2,KPNB1,WAC,APP,GATA4,SMAD5,GSKIP,UBE2G1,HERC2,AVPR1B,PTK2,TICAM2,SH2B2,LRRK2,IKZF3,ARIH1,UBXN7,PJA2,TIAM1,PRMT8,RABGEF1,KIT,SPAG9,SPDL1,PEX26,CTNNB1,PRKDC,IPO5,ESR1,PTPN14,BCAR3,WWOX,DOCK1,DIAPH3,TRPC5,IKBKB,AKAP6,RAB3GAP2,ATF2,ACVR1,RB1,ADIPOR1,SMG7,LAMP2,PRKCH,RABGAP1L,XRCC5,MID2,SIK1,ERCC6L2,KAT2B,MEF2D,CDK5RAP2,ARHGEF7,SRI,ATP1B1,STIM1,RAB11FIP2,CDH2,PPARG,JAKMIP1,SH3KBP1,USP25,MYO5A,SPDYE16,TFRC,SPDYE6,HAPSTR1,MAP3K1,IQCB1,CLEC6A,RAPGEF4,CLASP2,CLTC,RPRD2,ANGPT1,ATP6V0A4,ROCK1,TAX1BP1,PML,SP100,PRKG1,SBF2,TTC28,MAGI2,NIN,ANKFY1,ENSA,AIMP1,HSPBP1,PRKD1,PRLR,PCCA,CTBP2,TTN,MYRIP,CUL4A,PEX5L,FMNL2,KCNN4,SPART*
GO:1904315	transmitter-gated monoatomic	0.011703875248240157	*CHRNB2,CHRFAM7A,GABRA5,CHRNA7,GRIK1,CHRNB3,GRID2,GABRG2,GRIA4,CHRNB4,GABRB1,GRIA3,GRIA2*

	ion channel activity involved in regulation of postsynaptic membrane potential		
GO:0098960	postsynaptic neurotransmitter receptor activity	0.01404241905282315	*CHRNB2,CHRFAM7A,GABRA5,CHRM3,CHRNA7,GRIK1,CHRM5,CHRNB3,GRID2,GABRG2,GRIA4,CHRNB4,GABRB1,GRIA3,GRIA2*
GO:0015276	ligand-gated monoatomic ion channel activity	0.014608629452516904	*CHRNB2,KCNJ6,CHRFAM7A,GABRA5,ANO3,CHRNA7,KCNJ18,ANO2,ITPR1,GRIK1,HCN1,KCNJ12,CHRNB3,GRID2,KCNJ3,GABRB3,GABRG2,GRIA4,RYR3,CHRNB4,GABRB1,GRIA3,KCNT2,ASIC2,KCNE2,KCNN4,GRIA2*
GO:0099529	neurotransmitter receptor activity involved in regulation of postsynaptic membrane potential	0.017613795643837006	*CHRNB2,CHRFAM7A,GABRA5,CHRNA7,GRIK1,CHRNB3,GRID2,GABRG2,GRIA4,CHRNB4,GABRB1,GRIA3,GRIA2*
GO:0022836	gated channel activity	0.017630158703850724	*CACNB1,CHRNB2,KCNQ5,KCNJ6,CACNA1C,CHRFAM7A,CLCN3,GABRA5,KCNQ3,ANO3,KCNS3,CHRNA7,KCNJ18,ANO2,ITPR1,CACNA2D3,GRIK1,CACNA2D4,CACNA2D1,HCN1,CLCN1,KCNJ12,CHRNB3,GRID2,KCNJ3,GABRB3,GABRG2,KCND2,GRIA4,RYR3,CACHD1,CHRNB4,GABRB1,GRIA3,KCNT2,ASIC2,KCNE2,KCNN4,GRIA2,VDAC2*
GO:0022834	ligand-gated channel activity	0.017722082394888254	*CHRNB2,KCNJ6,CHRFAM7A,GABRA5,ANO3,CHRNA7,KCNJ18,ANO2,ITPR1,GRIK1,HCN1,KCNJ12,CHRNB3,GRID2,KCNJ3,GABRB3,GABRG2,GRIA4,RYR3,CHRNB4,GABRB1,GRIA3,KCNT2,ASIC2,KCNE2,KCNN4,GRIA2*
GO:0016740	transferase activity	0.02886171018083366	*C10ORF90,PFKP,STYK1,HS3ST3A1,CDK14,ING3,MAP3K3,WSCD1,SUGCT,SMYD3,GARS1,ABR,MAP3K13,CHST11,MAPK10,BRD4,CAMK2B,TLK1,KALRN,MAP4K3,XXYLT1,FPGT-TNNI3K,GALNT9,SPTLC3,STK39,SMURF2,CDK18,STK24,ARMT1,FAM20B,ASB4,PRKN,RMND5A,PNPLA3,F13A1,PIK3C2B,MARCHF9,CBLL2,NLK,MAPKAPK3,PAPOLG,AGL,UBE2O,PDHX,MECOM,STK32B,NEURL4,HERC5,UBE2E2,KLHL42,MARCHF1,BRAF,EEF2KMT,ALPK2,HECW1,TRIO,NDST1,MUSK,PNPLA4,MAP3K5,ZCCHC4,PRKAR2A,PRKG2,PIK3C3,PIGV,RSKR,EPHA5,STRADA,HUWE1,ZDHHC4,UBR5,PAK3,NDUFAF7,IPMK,CAMK1G,PRKAG2,OASL,CPT2,CILK1,EPHA3,LATS2,TYW3,LYN,SUMO2,NME7,LARGE1,NMNAT3,RNASEL,SGMS2,EIF2AK4,MAGEL2,TGFBR2,MARK4,POLR2C,ULK3,GKAP1,QTRT2,DAPK1,ACSM2B,RNF38,UBE2G1,PELI2,PI4KB,ELOVL5,HERC2,PIK3R5,BMT2,RIPK4,PTK2,SCP2,PIP4K2B,LRRK2,*

			ARIH1,TENT5C,PJA2,MGST1,COP1,ACVR2A,NEDD4L,PRMT8,RABGEF1,KIT,EPHA7,PRKDC,POFUT2,TPST2,HUNK,NEK10,HECTD1,KAT14,UGGT2,UBE3B,IKBKB,ATF2,ACVR1,NSMCE1,AK5,PRKCH,AGPAT5,BMX,TPST1,POLD3,TMTC1,NRK,MGRN1,MID2,SIK1,KAT2B,TMTC2,SPTLC1,UBE2E3,TENT4B,NDST4,TGM5,NEK7,CDK6,SH3KBP1,LPCAT2,MYLK4,MAP3K1,FUT10,B3GAT2,ACSM5,ROCK1,PML,LONRF3,DGKG,PRKG1,RFPL4B,MAGI2,GALK2,TPK1,PRKD1,STK4,TNNI3K,ST6GAL2,TTN,DYRK1A,CUL4A,CHSY3,UBE3C,ATE1,ETNK1,METTL3,UAP1,RNF6,SMYD1,DPY19L1
GO:0046332	SMAD binding	0.03758891791815145	*PPM1A,SMURF2,COL1A2,SKOR2,TGFBR2,GATA4,SMAD5,CTNNB1,ACVR1,LDLRAD4,PPARG,USP9X,PML,MAGI2,PMEPA1*
GO:0003824	catalytic activity	0.037781823333188275	*C10ORF90,PFKP,ABCC6,STYK1,HS3ST3A1,RGN,CDK14,MSRB3,PDE1C,ING3,MAP3K3,WSCD1,SUGCT,SMYD3,GARS1,ABR,MAOA,PREP,MAP3K13,PPM1A,ARL13B,IMMP1L,CHST11,USP42,MAPK10,USP32,AHCYL2,CDC14C,AGTPBP1,ATP10A,PIGL,ATP7B,PPM1H,BRD4,CAMK2B,TLK1,DDI2,PGM1,DCT,PECR,SDR16C5,ABCG8,OTUD7A,KALRN,SUMF1,MAP4K3,XXYLT1,FPGT-TNNI3K,GALNT9,FKBP5,SPTLC3,STYXL1,STK39,SMURF2,ADAMTS12,CDK18,STK24,ARMT1,FAM20B,ASB4,IDO1,PRKN,RMND5A,PNPLA3,F13A1,PIK3C2B,KDM7A,MARCHF9,CHD7,CBLL2,NLK,GNAI1,MAPKAPK3,PAPOLG,AGL,ENPEP,NUDT19,UBE2O,MBTPS2,ADAR,DNAH10,PDE10A,PDHX,RHOJ,MECOM,STK32B,PLG,NEURL4,HERC5,NUDT13,PGPEP1L,UBE2E2,KLHL42,EXO1,CHRM3,KDM2B,AIG1,PMS1,WRNIP1,TYW1,RSF1,SEPTIN10,MARCHF1,BRAF,EEF2KMT,ALPK2,HECW1,HMGB1,ATRX,TRIO,RAD51B,NDST1,MUSK,PNPLA4,MAP3K5,ALKBH1,RGS3,ZCCHC4,RERG,PRKAR2A,ATP11A,ATP4A,PRKG2,MBTPS1,NCF2,PIK3C3,PIGV,USP10,SND1,RSKR,EPHA5,PTPN20,CBR4,ENSG00000289694,ATP13A4,ATP2C1,ALDH1A2,PPTC7,STRADA,HUWE1,ASPH,RRAS2,ZDHHC4,UBR5,KIF21A,CPM,PAK3,NDUFAF7,IPMK,CYP2A13,CAMK1G,PRUNE2,PRKAG2,CPQ,F8,OASL,GDPD1,CPT2,DTD1,MGAM,CILK1,IL1RAPL2,EPHA3,LATS2,TYW3,PTPRN2,LYN,USP6,SUMO2,NME7,LARGE1,ATP6V1H,FIGNL1,PUS7,PAMR1,CA10,RRM2,ALDH1A1,NMNAT3,FMO5,RNASEL,NAV1,CORIN,DCP2,PPM1L,SGMS2,NOS3,BPHL,ERCC6,EIF2AK4,MSRA,RAB12,HMGCLL1,MAGEL2,TGFBR2,MARK4,DDX60L,MDN1,SYNJ1,ECHDC2,OVCH1,POLR2C,RRAGD,ULK3,GKAP1,ADGRV1,QTRT2,GAD1,DAPK1,TDP2,ALDH9A1,ACSM2B,PRUNE1,SAR1B,PPIL6,RNLS,RNF38,ADAM12,UBE2G1,PELI2,ATP9B,DHX35,CHRM5,PI4KB,RHOT1,ELOVL5,HERC2,PIK3R5,LYZL1,TLR1,CECR2,BMT2,RIPK4,QDPR,FRRS1,PTK2,USP7,SCP2,DNAH12,PIP4K2B,LRRK2,ARIH1,PLD1,TENT5C,PJA2,MGST1,COP1,ACVR2A,GNAO1,NEDD4L,BRCC3,DUSP14,PRMT8,RABGEF1,CYB5R4,KIT,EPHA7,TRABD2B,CTSK,PRKDC,AGAP4,PTPN14,POFUT2,DMGDH,PPM1E,WWOX,TPST2,FIGN,PARS2,HUNK,NEK10,TMEM62,PDE7B,USP40,HDDC2,BPGM,CDC14B,PLCH1,HECTD1,VKORC1L1,A*

			ASS,KAT14,UGGT2,UBE3B,PTPRF,IKBKB,CEMIP,ATF2,HDAC8,ACVR1,HYAL4,NSMCE1,PDE4B,DDX10,KIF13A,PLPP3,IMMP2L,AK5,CPS1,PRKCH,ACACB,AGPAT5,XRCC5,RGS17,INPP4A,BMX,RAD54L2,TPST1,POLD3,RAB18,TPH2,TMTC1,NRK,MGRN1,MID2,SIK1,ERCC6L2,PIPOX,KAT2B,TMTC2,TMPRSS11B,PRCP,SUPV3L1,MGAM2,SPTLC1,UBE2E3,RFC3,FARSB,ADAT2,TENT4B,TLR6,NDST4,ADAMTS9,TSEN15,KIF16B,EEFSEC,PAPPA,AGAP9,USP9X,TGM5,NEK7,CDK6,SH3KBP1,SMOX,LPCAT2,AMY2A,DDX60,MYLK4,USP25,KIF2C,IL1RAP,PNPLA8,RAB31,MAP3K1,DDX4,FUT10,TMPRSS15,B3GAT2,ACSM5,PHLPP1,DNAJC6,ROCK1,APIP,MMP28,NUP98,PML,MGLL,LONRF3,DGKG,PRKG1,FOXRED2,RFPL4B,TSEN2,MAGI2,GALK2,PRSS50,TPK1,PRKD1,USP34,STK4,TNNI3K,RAB6D,ST6GAL2,RNASE11,ANPEP,PTPRA,PDE11A,RAB6C,PCCA,GNA14,OXNAD1,CTBP2,TTN,DYRK1A,CUL4A,CHSY3,TXNRD3,EYA4,ARL15,UBE3C,ATE1,ETNK1,CHAC2,METTL3,UAP1,CP,ATP8B1,PGPEP1,RNF6,SMYD1,PEPD,DPY19L1
GO:0005085	guanyl-nucleotide exchange factor activity	0.04551610254587929	*RAPGEF6,ARHGEF9,ABR,SLC38A9,KALRN,TIAM2,ARHGEF4,ELMO1,PREX1,TRIO,ARHGEF39,RASGRF2,HERC2,ARHGEF38,TIAM1,IQSEC2,RABGEF1,ARFGEF2,BCAR3,DOCK1,NGEF,SH3BP5,ARHGEF28,RAB3GAP2,DENND2B,ARHGEF7,VAV2,RAPGEF4,SBF2,VAV1*
BP			
GO:0048731	system development	9.648134744717765e-13	*APBB2,RTN1,HS3ST3A1,RGN,EPN2,MAP3K3,LRRN1,NFIA,ARHGEF9,ISM1,TRAPPC9,MAP3K13,ARL13B,CHST11,THRB,SPTBN1,FGF1,CHRNB2,SEZ6,GNB4,AGTPBP1,SPINK5,DOK4,CAMK2B,GRIP1,DCT,KALRN,USH2A,NOTCH2,TMOD2,SDK2,NPNT,EPB41L3,MAP6,CDH11,SHANK3,ARID1B,E2F7,FOXO1,STYXL1,ADAMTS12,DAAM2,PLXNA4,STK24,DIPK2A,CACNA1C,ASB4,IDO1,PRKN,NLGN4X,KDM7A,IFT172,CHD7,GPSM1,CDON,SERPINI1,SLC17A6,THSD7A,NCAM2,TIAM2,TRAK2,CNMD,LRP8,TAGLN3,CNTN1,MEOX2,STAT5B,ENPEP,EIF4ENIF1,MBTPS2,PTH,ADAR,RHOJ,FGF10,TSPAN18,PLG,NCK1,NREP,EXO1,GABRA5,NEXMIF,KCNQ3,CHRM3,KDM2B,PREX1,COL11A1,ZNF804A,MCPH1,BRAF,COL1A2,ALPK2,CHRNA7,HECW1,HMGB1,ZNF304,RFLNA,ATRX,TGFB2,LAMA2,TRIO,SMARCC1,NDST1,MUSK,JAM3,ALKBH1,SLC15A4,PCDHB8,PRKG2,EPHA5,SKOR2,RUNX1,ENAH,SNX10,ALDH1A2,PCLO,RRAS2,SIM1,KIAA1217,CHL1,GSN,PLS1,SLIT3,ROBO1,ANKRD11,DNAAF11,PAK3,ABI3BP,BTG4,TNFRSF12A,CXCR5,NCKAP1,FOSL2,IL1RAPL2,EPHA3,MYOM2,LYN,ADGRB3,KLF7,GRIK1,DIP2B,LARGE1,CA10,CRYBB1,LDB2,NAV1,PCDHB16,NOS3,VSX1,ERCC6,EIF2AK4,MATN2,TGFBR2,MARK4,TCF7L2,ZNF800,CRTAC1,ZFHX3,CUX1,UNC119,GPM6B,ADGRV1,EPB41L5,WHRN,TDP2,PRUNE1,APP,GATA4,SMAD5,IGF2BP1,MAMLD1,RNF38,ADAM12,HCN1,FRY,HOOK3,PALLD,CECR2,PTK2,SH2B2,CELF1,HYCC1,LRRK2,CLSTN2,PKHD1,RARB,TCF4,TIAM1,CSRP3,PHACTR1,ACVR2A,VIT,SNTG2,NTM,MEIS1,NEDD4L,KIT,EPHA7,*

			SPAG9,ANTXR1,WNT5A,CTNNB1,ANKRD17,COL19A1,CTSK,PRKDC,ESR1,PTPN14,ITGB8,BCAR3,LMX1A,WWOX,PTGER4,GREB1L,BBX,VWC2L,DIAPH3,GPC4,BPGM,EFNA5,IGF2BP2,HECTD1,NGEF,TRPC5,NEUROD2,TNFSF11,PTPRF,FXR2,AKAP6,APOH,ARHGEF28,GPRIN1,POU6F2,LINGO2,OPCML,ATF2,ACVR1,GRID2,ZNF423,RB1,MPV17,NTNG1,PKD1L3,GABRB3,SHROOM4,PLPP3,HNF1B,IMMP2L,KIRREL3,GABRG2,CPS1,PRKCH,ACACB,DLC1,XRCC5,GFRA1,SHLD2,RAB18,ETV1,TEX11,LRTM2,SIK1,KAT2B,WLS,ANKS1A,PRCP,MEF2D,CDK5RAP2,FLVCR1,ARHGEF7,NPHP1,FBN2,SRI,BDNF,TFCP2L1,SEMA3E,SCIN,MACO1,ADAMTS9,STIM1,CDH2,PPARG,USP9X,TENM1,RORB,CDK6,GABRB1,AVIL,TFAP2D,SIPA1L3,SFRP4,IL1RAP,JCAD,VAV2,TFRC,VPS13A,FBN1,BMPER,FUT10,CLASP2,ZNF521,ANGPT1,SEMA6A,EIF4G1,PHLPP1,ITGA4,ROCK1,CLEC3A,PML,AFF2,TNFSF4,DGKG,SP100,PRKG1,ELAVL4,SBF2,CDH9,UMODL1,MAGI2,NIN,ASIC2,FNDC3A,AIMP1,PRKD1,CALCRL,STK4,PCDH10,ANPEP,PRLR,TTN,HHIP,DYRK1A,TMEM30A,SEMA5A,LAMA4,TTPA,METTL3,PARD3,ATP8B1,RNF6,SEMA3C,SMYD1,VPS13B,SPART
GO:0007275	multicellular organism development	1.949083104149e-11	*APBB2,RTN1,HS3ST3A1,RGN,EPN2,MAP3K3,LRRN1,NFIA,ARHGEF9,ISM1,TRAPPC9,MAP3K13,ARL13B,CHST11,THRB,SPTBN1,FGF1,CHRNB2,SEZ6,GNB4,AGTPBP1,SH3PXD2A,SPINK5,BRD4,DOK4,CAMK2B,GRIP1,DCT,KALRN,USH2A,NOTCH2,TMOD2,SDK2,NPNT,EPB41L3,MAP6,CDH11,SHANK3,NR4A3,ARID1B,E2F7,FOXO1,STYXL1,SMURF2,ADAMTS12,DAAM2,PLXNA4,YBX1,STK24,DIPK2A,CACNA1C,ASB4,IDO1,PRKN,NLGN4X,EXOC4,KDM7A,SLC5A7,IFT172,CHD7,GPSM1,CDON,SERPINI1,SLC17A6,THSD7A,NCAM2,TIAM2,TRAK2,CNMD,LRP8,TAGLN3,CNTN1,MEOX2,STAT5B,ENPEP,EIF4ENIF1,MBTPS2,PTH,ADAR,RHOJ,FGF10,TSPAN18,PLG,NCK1,NREP,EXO1,GABRA5,NEXMIF,KCNQ3,CHRM3,KDM2B,PREX1,COL11A1,ZNF804A,MCPH1,BRAF,COL1A2,COL12A1,ALPK2,CHRNA7,HECW1,HMGB1,ZNF304,AFF3,RFLNA,ATRX,TGFB2,LAMA2,TRIO,HOXC13,SMARCC1,RAD51B,NDST1,MUSK,JAM3,ALKBH1,ATP11A,SLC15A4,PCDHB8,PRKG2,IGFBP7,EPHA5,SKOR2,RUNX1,ENAH,SNX10,ALDH1A2,PCLO,ASPH,RRAS2,SIM1,KIAA1217,CHL1,GSN,PLS1,SLIT3,ROBO1,ANKRD11,ITPR1,DNAAF11,PAK3,ABI3BP,MLLT3,BTG4,TNFRSF12A,CPT2,CXCR5,NCKAP1,FOSL2,IL1RAPL2,EPHA3,LATS2,MYOM2,LYN,ADGRB3,KLF7,GRIK1,DIP2B,SEC24C,LARGE1,CA10,RRM2,CRYBB1,LDB2,NAV1,PCDHB16,SGMS2,NOS3,VSX1,ERCC6,EIF2AK4,MATN2,TGFBR2,MARK4,TCF7L2,ZNF800,CRTAC1,ZFHX3,CUX1,UNC119,GPM6B,ADGRV1,EPB41L5,WHRN,TDP2,PRUNE1,APP,GATA4,SMAD5,IGF2BP1,MAMLD1,RNF38,ADAM12,DHX35,HCN1,FRY,HOOK3,PALLD,CECR2,PTK2,SH2B2,CELF1,HYCC1,LRRK2,INO80D,IKZF3,CLSTN2,PKHD1,TENT5C,RARB,TCF4,TIAM1,CSRP3,PHACTR1,ACVR2A,VIT,SNTG2,NTM,MEIS1,NEDD4L,KIT,EPHA7,SPAG9,ANTXR1,WNT5A,CTNNB1,ANKRD1*

			7,COL19A1,CTSK,PRKDC,ESR1,PTPN14,ITGB8,POFUT2,BCAR3,LMX1A,WWOX,PTGER4,GREB1L,BBX,VWC2L,DIAPH3,GPC4,BPGM,EFNA5,IGF2BP2,HECTD1,NGEF,TRPC5,NEUROD2,KAT14,TNFSF11,PTPRF,IKBKB,FXR2,AKAP6,APOH,ARHGEF28,TASOR,GPRIN1,POU6F2,LINGO2,OPCML,ATF2,ACVR1,GRID2,ZNF423,RB1,MPV17,NTNG1,PKD1L3,GABRB3,SHROOM4,PLPP3,HNF1B,IMMP2L,KIRREL3,GABRG2,CPS1,PRKCH,ACACB,DLC1,XRCC5,GFRA1,SHLD2,RAB18,ETV1,TEX11,LRTM2,SIK1,KAT2B,WLS,ANKS1A,PRCP,MEF2D,CDK5RAP2,FLVCR1,ARHGEF7,DACH1,NPHP1,FBN2,SRI,ARNT,BDNF,TFCP2L1,SEMA3E,SCIN,MACO1,ADAMTS9,STIM1,KIF16B,CDH2,LAG3,PPARG,USP9X,TENM1,RORB,CDK6,GABRB1,AVIL,TFAP2D,TTC39C,SIPA1L3,SFRP4,BLTP1,IL1RAP,JCAD,VAV2,TFRC,VPS13A,FBN1,BMPER,FUT10,CLASP2,ZNF521,ANGPT1,SEMA6A,EIF4G1,PHLPP1,ITGA4,ROCK1,CLEC3A,PML,AFF2,TNFSF4,DGKG,SP100,PRKG1,ELAVL4,SBF2,CDH9,UMODL1,MAGI2,NIN,ASIC2,FNDC3A,AIMP1,PRKD1,CALCRL,SLC20A2,STK4,PCDH10,ANPEP,PRLR,LPAR6,TTN,HHIP,DYRK1A,TMEM30A,CUL4A,EYA4,SEMA5A,LAMA4,TTPA,METTL3,PARD3,ATP8B1,RNF6,SEMA3C,SMYD1,VPS13B,SPART
GO:0065007	biological regulation	1.319127359047689e-10	*FSTL1,C10ORF90,APBB2,STYK1,RTN1,RGN,CDK14,EPN2,PDE1C,NOSTRIN,ING3,MAP3K3,SLC12A8,OSBPL8,LRRN1,MED13L,NFIA,STX8,PHF20,FOXK2,SMYD3,RAPGEF6,ARHGEF9,TAFA2,ABR,ISM1,MAOA,MAP3K13,PPM1A,ARL13B,SAMSN1,LIMD1,TF,CHST11,THRB,USP42,SPTBN1,CDC42EP3,MAPK10,CACNB1,USP32,DAPP1,ATF7,GNG12,FGF1,CHRNB2,CDC14C,SEZ6,KCNQ5,CDH8,ARHGAP15,GNB4,AGTPBP1,ATF7-NPFF,FHL1,RBPMS,ATP10A,SPINK5,OPA3,SKAP2,ATP7B,GPBP1L1,KCNJ6,BRD4,DOK4,CAMK2B,GRIP1,ARHGAP11A-SCG5,TLK1,DDI2,DCT,DLGAP1,SLC38A9,ZNF264,HBP1,SDR16C5,ABCG8,XPO4,KALRN,USH2A,MAP4K3,NOTCH2,TMOD2,HMBOX1,ZNF551,NPNT,EPB41L3,MAP6,MORC1,PARVB,CDH11,SHANK3,NR4A3,ARID1B,E2F7,FOXO1,ZNF611,STYXL1,STK39,SMURF2,ADAMTS12,DAAM2,FER1L6,TBC1D16,PLXNA4,CDK18,URB2,YBX1,STK24,DIPK2A,DCDC1,CACNA1C,FAM3C,ASB4,IDO1,ARB2A,PRKN,NLGN4X,EXOC4,F13A1,PIK3C2B,KDM7A,SLC5A7,FAM83B,IFT172,CHD7,GPSM1,CBLL2,PTBP3,CDON,SERPINI1,SLC17A6,NLK,GNAI1,MRPL13,MAPKAPK3,TIAM2,ZNF10,CNMD,LRP8,TAGLN3,CNTN1,MEOX2,STAT5B,ENPEP,ARHGEF4,UBE2O,DDIAS,CPEB4,EIF4ENIF1,CALCR,CHRFAM7A,ELMO1,ERBIN,ZNF121,MBTPS2,RGS22,CD247,MCC,PTH,ADAR,PDE10A,TM9SF2,TOX2,CLCN3,RHOJ,CDC37,FGF10,ZFYVE16,MECOM,COL4A6,TSPAN18,NLRP8,STK32B,PLG,HERC5,UBE2E2,ILDR2,NCK1,CD84,FAM168A,NONO,RARRES1,KLHL42,NREP,GABRA5,NEXMIF,KCNQ3,MTCP1,CHRM3,KDM2B,PREX1,ZNF404,WRNIP1,ANO3,RSF1,NCOA7,ZNF804A,CIDEB,MCPH1,SESTD1,BRAF,ZNF214,COL1A2,DTNA,TMOD3,*

			GEMIN5,MRTFB,ALPK2,CHRNA7,HECW1,HMGB1,ZNF304,DYNLT2B,CDIP1,AFF3,RFLNA,HIVEP1,ATRX,TGFB2,MDFIC,LAMA2,ADD3,TRIO,ZNF85,HOXC13,SMARCC1,RAD51B,NDST1,MUSK,KCNJ18,JAM3,KCNIP3,SFPQ,PNPLA4,MAP3K5,ALKBH1,RGS3,NUP93,ZCCHC4,RERG,OR5AU1,PRKAR2A,RBM33,TBC1D5,CTIF,ATP11A,RBM23,ZNF154,ATP4A,SLC15A4,GOLPH3,PRKG2,MBTPS1,PIK3C3,USP10,SND1,IGFBP7,EPHA5,SKOR2,ENSG00000289694,RUNX1,ATP2C1,ALDH1A2,PPTC7,STON2,PTGFR,FAM171A1,STRADA,PCLO,HUWE1,ZNF595,ASPH,RRAS2,SH3BGRL,UBR5,GTF2F2,SIM1,SOX30,ANKS1B,CHL1,SPOCK3,GSN,PLS1,TCP11L1,SPON1,MAPRE3,TLR8,SLIT3,ARMH3,CACUL1,NLRP7,ARHGEF39,PHC2,ROBO1,ZNF578,ITPR1,PKP4,PAK3,ABI3BP,KLF12,TMEM183A,DOK6,PRKAG2,MLLT3,CCNT2,BTG4,HAVCR1,CPQ,CNOT10,MR1,F8,TNFRSF12A,OASL,CPT2,TAS2R14,VPS8,ZNF429,CXCR5,NCKAP1,PRPF40A,DTD1,CILK1,FOSL2,IL1RAPL2,ADGRD1,EPHA3,LATS2,MYH14,SPDYE2B,ERP29,CDYL2,ARHGAP18,UVRAG,PTPRN2,LYN,ADGRB3,USP6,KLF7,SUMO2,GRIK1,MTA3,DIP2B,NME7,LARGE1,ATP6V1H,NUP58,FIGNL1,SLC12A1,PUS7,PDZD8,RRM2,LDB2,MYRFL,SPTB,ALDH1A1,FMO5,RNASEL,DBF4B,CORIN,PCDHB16,DCP2,PPM1L,SGMS2,TMED7-TICAM2,NOS3,KCNIP4,VSX1,ZNF280B,TNRC6C,PRAMEF27,MLLT10,ERCC6,NOS1AP,EIF2AK4,KIAA1614,RAB12,XKR6,MAGEL2,TGFBR2,CMKLR2,ITGA11,MARK4,SYNJ1,TCF7L2,ZNF800,ZNF462,E2F6,ZFHX3,CUX1,UNC119,SHISA2,GPM6B,RRAGD,ULK3,SPDYE2,GPR156,GKAP1,PPP2R5E,GSDMD,ADGRV1,RASGRF2,ST7,SERBP1,EPB41L5,GAD1,SCG5,WAC,WHRN,OCA2,DAPK1,TDP2,ZNF678,PRUNE1,SAR1B,INSYN2A,BTG3,APP,GATA4,ATF7IP2,SMAD5,IGF2BP1,MAMLD1,RNLS,GSKIP,ADAM12,PELI2,ATP9B,CACNA2D1,SP140L,HCN1,CHRM5,PI4KB,FRY,RHOT1,ELOVL5,HOOK3,PIK3R5,TLR1,TAS2R30,BMT2,RIPK4,AVPR1B,PTK2,USP7,TICAM2,SCP2,SH2B2,ATP1B4,ZNF41,AVEN,CELF1,POGZ,PIP4K2B,LRRK2,NKAIN3,INO80D,ZNF395,IKZF3,CLSTN2,ARIH1,CFAP298,PLD1,ITFG2,VTCN1,PKHD1,TENT5C,RARB,PJA2,TCF4,PAEP,COP1,TIAM1,CSRP3,PHACTR1,MLIP,IQSEC2,ACVR2A,GNAO1,ASAP2,TIPIN,VIT,RASSF4,MEIS1,NEDD4L,BRCC3,SNX13,RABGEF1,CYB5R4,KIT,TBC1D8,EPHA7,SPAG9,ANTXR1,SPDL1,WNT5A,TRABD2B,ARFGEF2,TEX2,CTNNB1,ANKRD17,RUNX1T1,CTSK,PRKDC,IPO5,ESR1,TNFRSF19,ZNF479,PTPN14,SCFD1,ITGB8,ERC2,POFUT2,BCAR3,TPD52L1,L3MBTL4,PPM1E,ZXDC,FRMD4A,LMX1A,WWOX,BNIPL,HUNK,NEK10,ANAPC10,PTGER4,ZNF215,BBX,PDE7B,VWC2L,BEST3,DOCK1,DIAPH3,GPC4,PKIG,EFNA5,IGF2BP2,ZNF722,CDC14B,SYNE3,PLCH1,OR1M1,HECTD1,NGEF,TRPC5,NEUROD2,FNIP2,AASS,JAML,KAT14,TNFSF11,PTPRF,IKBKB,FXR2,SH3BP5,AKAP6,TRBV10-2,USF3,LYVE1,APOH,ARHGEF28,OR8B8,CLCN1,RAB3GAP2,TASOR,CCPG1,POU6F2,LINGO2,CE

			MIP,XIST,ATF2,ZNF426,KCNJ12,HDAC8,CHRNB3,ACVR1,HLA-DQA2,PID1,EBF2,GRID2,ZNF423,ZCCHC17,KCNJ3,RB1,NSMCE1,MPV17,IFNLR1,NTNG1,PDE4B,KIF13A,NLRP4,ADIPOR1,ZNF143,GABRB3,ZSCAN23,SMG7,PLPP3,HNF1B,ZNF615,LAMP2,GABRG2,F5,CPS1,NCOA2,BNIP3L,PRKCH,ACACB,RABGAP1L,DLC1,DENND2B,XRCC5,TBRG4,RGS17,GFRA1,SHLD2,KCND2,INPP4A,BMX,MIR663AHG,ZNF418,SLC24A3,TAF15,GRIA4,RAB18,ZNF860,POU2F1,ETV1,RGS7BP,MGRN1,TEX11,MID2,MIR198,LRTM2,SIK1,TP53BP2,ZNF850,KAT2B,GOLPH3L,WLS,ANKS1A,RYR3,PRCP,SUPV3L1,MEF2D,MYL4,GTF2A1,SPTLC1,CDK5RAP2,FLVCR1,RFC3,ARHGEF7,SAMD12,DACH1,ATF3,TRDN,NPHP1,FBN2,IKZF1,CAPZA2,SRI,ATP1B1,ARNT,BDNF,LDLRAD4,TFCP2L1,SEMA3E,BCLAF3,SCIN,MALRD1,TENT4B,MACO1,ZNF665,TLR6,ADAMTS9,STIM1,TMEM237,TMEM161B,HAUS6,KIF16B,RAB11FIP2,CDH2,EEFSEC,LAG3,PPARG,TANC1,PAPPA,ASB3,MTTP,CHRNB4,USP9X,NEK7,ZNF80,TENM1,IL13RA2,RORB,CDK6,GABRB1,MYOF,LTB4R2,SH3KBP1,NALCN,AVIL,DDX60,TFAP2D,USP25,KIF2C,MYO5A,SIPA1L3,ZNF286A-TBC1D26,LRFN2,SPTBN5,SFRP4,BLTP1,SPDYE16,PNRC1,IL1RAP,JCAD,VAV2,TFRC,TES,OSBPL6,GRIA3,PNPLA8,SPDYE6,FBN1,HAPSTR1,RAB31,MAP3K1,ZFP82,DDX4,IQCB1,CLEC6A,ZNF292,RAPGEF4,BMPER,CLASP2,CLTC,ZNF521,ZNF761,ANGPT1,SEMA6A,VIPR2,EIF4G1,ATP6V0A4,PHLPP1,ZNF618,ITGA4,DNAJC6,ROCK1,NCAPH,TAX1BP1,TBC1D4,APIP,MMP28,ZNF701,NUP98,IKZF2,PML,FOXO3B,SACS,AFF2,MGLL,PLEK2,TNFSF4,AFAP1L2,DGKG,SP100,PRKG1,ELAVL4,NDUFAF2,CFAP298-TCP10L,UMODL1,RFPL4B,TTC28,MAGI2,NIN,SPG21,ZNF286A,ANKFY1,ZNF69,ASIC2,ENSA,MIR9-2HG,KHDRBS3,AIMP1,SLC30A10,HSPBP1,PRKD1,CALCRL,SLC20A2,USP34,STK4,TNNI3K,SPC25,PRLR,ZNF569,PTPRA,ZNF180,PDE11A,NMI,RAB6C,YLPM1,IL16,ZSWIM7,ZNF470,EDARADD,GNA14,CR2,LPAR6,CTBP2,TTN,NCALD,HHIP,DYRK1A,PPFIA1,TMEM30A,MYRIP,PPP1R1C,KCNE2,UNC13C,CUL4A,SERINC1,KCTD16,EYA4,ZNF888,DLGAP2,ZNF736,SEMA5A,ELL2,LAMA4,PMEPA1,TTPA,PEX5L,PVT1,GATAD2B,METTL3,FMNL2,UAP1,PARD3,KCNN4,ZNF33A,CP,NPAS3,BMAL2,ATP8B1,RNF6,SEMA3C,VAV1,SMYD1,FANCB,PEPD,GRIA2,VDAC2,SPART
GO:0050789	regulation of biological process	1.651509311947583e-10	*FSTL1,C10ORF90,APBB2,STYK1,RTN1,RGN,CDK14,EPN2,PDE1C,NOSTRIN,ING3,MAP3K3,OSBPL8,LRRN1,MED13L,NFIA,STX8,PHF20,FOXK2,SMYD3,RAPGEF6,ARHGEF9,TAFA2,ABR,ISM1,MAOA,MAP3K13,PPM1A,ARL13B,SAMSN1,LIMD1,TF,CHST11,THRB,USP42,SPTBN1,CDC42EP3,MAPK10,CACNB1,USP32,DAPP1,ATF7,GNG12,FGF1,CHRNB2,CDC14C,SEZ6,CDH8,ARHGAP15,GNB4,AGTPBP1,ATF7-NPFF,FHL1,RBPMS,ATP10A,SPINK5,OPA3,SKA*

			P2,GPBP1L1,KCNJ6,BRD4,DOK4,CAMK2B,GRIP1,ARHGAP11A-SCG5,TLK1,DCT,DLGAP1,SLC38A9,ZNF264,HBP1,SDR16C5,ABCG8,XPO4,KALRN,USH2A,MAP4K3,NOTCH2,TMOD2,HMBOX1,ZNF551,NPNT,EPB41L3,MAP6,MORC1,PARVB,CDH11,SHANK3,NR4A3,ARID1B,E2F7,FOXO1,ZNF611,STYXL1,STK39,SMURF2,ADAMTS12,DAAM2,FER1L6,TBC1D16,PLXNA4,CDK18,URB2,YBX1,STK24,DIPK2A,DCDC1,CACNA1C,FAM3C,ASB4,IDO1,ARB2A,PRKN,NLGN4X,EXOC4,PIK3C2B,KDM7A,SLC5A7,FAM83B,IFT172,CHD7,GPSM1,CBLL2,PTBP3,CDON,SERPINI1,SLC17A6,NLK,GNAI1,MRPL13,MAPKAPK3,TIAM2,ZNF10,CNMD,LRP8,TAGLN3,CNTN1,MEOX2,STAT5B,ENPEP,ARHGEF4,UBE2O,DDIAS,CPEB4,EIF4ENIF1,CALCR,CHRFAM7A,ELMO1,ERBIN,ZNF121,MBTPS2,RGS22,CD247,MCC,PTH,ADAR,PDE10A,TM9SF2,TOX2,CLCN3,RHOJ,CDC37,FGF10,ZFYVE16,MECOM,COL4A6,TSPAN18,NLRP8,STK32B,PLG,HERC5,UBE2E2,ILDR2,NCK1,CD84,FAM168A,NONO,RARRES1,KLHL42,NREP,GABRA5,NEXMIF,KCNQ3,MTCP1,CHRM3,KDM2B,PREX1,ZNF404,WRNIP1,RSF1,NCOA7,ZNF804A,CIDEB,MCPH1,SESTD1,BRAF,ZNF214,COL1A2,DTNA,TMOD3,GEMIN5,MRTFB,ALPK2,CHRNA7,HECW1,HMGB1,ZNF304,DYNLT2B,CDIP1,AFF3,RFLNA,HIVEP1,ATRX,TGFB2,MDFIC,LAMA2,ADD3,TRIO,ZNF85,HOXC13,SMARCC1,RAD51B,NDST1,MUSK,KCNJ18,JAM3,KCNIP3,SFPQ,MAP3K5,ALKBH1,RGS3,NUP93,ZCCHC4,RERG,OR5AU1,PRKAR2A,RBM33,TBC1D5,CTIF,ATP11A,RBM23,ZNF154,ATP4A,SLC15A4,GOLPH3,PRKG2,MBTPS1,PIK3C3,USP10,SND1,IGFBP7,EPHA5,SKOR2,ENSG00000289694,RUNX1,ATP2C1,ALDH1A2,PPTC7,STON2,PTGFR,FAM171A1,STRADA,PCLO,HUWE1,ZNF595,ASPH,RRAS2,SH3BGRL,UBR5,GTF2F2,SIM1,SOX30,ANKS1B,CHL1,SPOCK3,GSN,PLS1,TCP11L1,SPON1,MAPRE3,TLR8,SLIT3,ARMH3,CACUL1,NLRP7,ARHGEF39,PHC2,ROBO1,ZNF578,ITPR1,PKP4,PAK3,ABI3BP,KLF12,DOK6,PRKAG2,MLLT3,CCNT2,BTG4,HAVCR1,CNOT10,MR1,TNFRSF12A,OASL,CPT2,TAS2R14,VPS8,ZNF429,CXCR5,NCKAP1,PRPF40A,CILK1,FOSL2,IL1RAPL2,ADGRD1,EPHA3,LATS2,MYH14,SPDYE2B,ERP29,CDYL2,ARHGAP18,UVRAG,PTPRN2,LYN,ADGRB3,USP6,KLF7,SUMO2,GRIK1,MTA3,DIP2B,NME7,LARGE1,ATP6V1H,NUP58,FIGNL1,PUS7,PDZD8,RRM2,LDB2,MYRFL,SPTB,ALDH1A1,FMO5,RNASEL,DBF4B,CORIN,PCDHB16,DCP2,PPM1L,SGMS2,TMED7-TICAM2,NOS3,KCNIP4,VSX1,ZNF280B,TNRC6C,PRAMEF27,MLLT10,ERCC6,NOS1AP,EIF2AK4,KIAA1614,RAB12,MAGEL2,TGFBR2,CMKLR2,ITGA11,MARK4,SYNJ1,TCF7L2,ZNF800,ZNF462,E2F6,ZFHX3,CUX1,UNC119,SHISA2,GPM6B,RRAGD,ULK3,SPDYE2,GPR156,GKAP1,PPP2R5E,GSDMD,ADGRV1,RASGRF2,ST7,SERBP1,EPB41L5,GAD1,SCG5,WAC,WHRN,DAPK1,TDP2,ZNF678,PRUNE1,SAR1B,INSYN2A,BTG3,APP,GATA4,ATF7IP2,SMAD5,IGF2BP1,MAMLD1,RNLS,GSKIP,

			ADAM12,PELI2,CACNA2D1,SP140L,HCN1,CHRM5,PI4KB,FRY,RHOT1,ELOVL5,HOOK3,PIK3R5,TLR1,TAS2R30,BMT2,RIPK4,AVPR1B,PTK2,USP7,TICAM2,SCP2,SH2B2,ATP1B4,ZNF41,AVEN,CELF1,POGZ,PIP4K2B,LRRK2,NKAIN3,INO80D,ZNF395,IKZF3,CLSTN2,ARIH1,CFAP298,PLD1,ITFG2,VTCN1,PKHD1,TENT5C,RARB,PJA2,TCF4,PAEP,COP1,TIAM1,CSRP3,PHACTR1,MLIP,IQSEC2,ACVR2A,GNAO1,TIPIN,VIT,RASSF4,MEIS1,NEDD4L,BRCC3,SNX13,RABGEF1,CYB5R4,KIT,TBC1D8,EPHA7,SPAG9,ANTXR1,SPDL1,WNT5A,TRABD2B,ARFGEF2,TEX2,CTNNB1,ANKRD17,RUNX1T1,CTSK,PRKDC,IPO5,ESR1,TNFRSF19,ZNF479,PTPN14,SCFD1,ITGB8,ERC2,POFUT2,BCAR3,TPD52L1,L3MBTL4,PPM1E,ZXDC,FRMD4A,LMX1A,WWOX,BNIPL,HUNK,NEK10,ANAPC10,PTGER4,ZNF215,BBX,PDE7B,VWC2L,BEST3,DOCK1,DIAPH3,GPC4,PKIG,EFNA5,IGF2BP2,ZNF722,CDC14B,SYNE3,PLCH1,OR1M1,HECTD1,NGEF,TRPC5,NEUROD2,FNIP2,AASS,JAML,KAT14,TNFSF11,PTPRF,IKBKB,FXR2,SH3BP5,AKAP6,TRBV10-2,USF3,LYVE1,APOH,ARHGEF28,OR8B8,RAB3GAP2,TASOR,CCPG1,POU6F2,LINGO2,CEMIP,XIST,ATF2,ZNF426,KCNJ12,HDAC8,CHRNB3,ACVR1,HLA-DQA2,PID1,EBF2,GRID2,ZNF423,ZCCHC17,KCNJ3,RB1,NSMCE1,MPV17,IFNLR1,NTNG1,PDE4B,KIF13A,NLRP4,ADIPOR1,ZNF143,GABRB3,ZSCAN23,SMG7,PLPP3,HNF1B,ZNF615,LAMP2,GABRG2,NCOA2,BNIP3L,PRKCH,ACACB,RABGAP1L,DLC1,DENND2B,XRCC5,TBRG4,RGS17,GFRA1,SHLD2,KCND2,INPP4A,BMX,MIR663AHG,ZNF418,SLC24A3,TAF15,GRIA4,RAB18,ZNF860,POU2F1,ETV1,RGS7BP,MGRN1,TEX11,MID2,MIR198,LRTM2,SIK1,TP53BP2,ZNF850,KAT2B,GOLPH3L,WLS,ANKS1A,RYR3,PRCP,SUPV3L1,MEF2D,MYL4,GTF2A1,SPTLC1,CDK5RAP2,FLVCR1,RFC3,ARHGEF7,SAMD12,DACH1,ATF3,TRDN,NPHP1,FBN2,IKZF1,CAPZA2,SRI,ATP1B1,ARNT,BDNF,LDLRAD4,TFCP2L1,SEMA3E,BCLAF3,SCIN,MALRD1,TENT4B,MACO1,ZNF665,TLR6,ADAMTS9,STIM1,TMEM237,TMEM161B,HAUS6,KIF16B,RAB11FIP2,CDH2,EEFSEC,LAG3,PPARG,TANC1,PAPPA,ASB3,MTTP,CHRNB4,USP9X,NEK7,ZNF80,TENM1,IL13RA2,RORB,CDK6,GABRB1,MYOF,LTB4R2,SH3KBP1,NALCN,AVIL,DDX60,TFAP2D,USP25,KIF2C,MYO5A,SIPA1L3,ZNF286A-TBC1D26,LRFN2,SPTBN5,SFRP4,BLTP1,SPDYE16,PNRC1,IL1RAP,JCAD,VAV2,TFRC,TES,OSBPL6,GRIA3,PNPLA8,SPDYE6,FBN1,HAPSTR1,RAB31,MAP3K1,ZFP82,DDX4,IQCB1,CLEC6A,ZNF292,RAPGEF4,BMPER,CLASP2,CLTC,ZNF521,ZNF761,ANGPT1,SEMA6A,VIPR2,EIF4G1,PHLPP1,ZNF618,ITGA4,DNAJC6,ROCK1,NCAPH,TAX1BP1,TBC1D4,APIP,MMP28,ZNF701,NUP98,IKZF2,PML,FOXO3B,SACS,AFF2,MGLL,PLEK2,TNFSF4,AFAP1L2,DGKG,SP100,PRKG1,ELAVL4,NDUFAF2,CFAP298-TCP10L,UMODL1,RFPL4B,TTC28,MAGI2,NIN,SPG21,ZNF286A,ANKFY1,ZNF69,ASIC2,ENSA,M

			IR9-2HG,KHDRBS3,AIMP1,SLC30A10,HSPBP1,PRKD1,CALCRL,SLC20A2,USP34,STK4,TNNI3K,SPC25,PRLR,ZNF569,PTPRA,ZNF180,PDE11A,NMI,RAB6C,YLPM1,IL16,ZNF470,EDARADD,GNA14,CR2,LPAR6,CTBP2,TTN,NCALD,HHIP,DYRK1A,PPFIA1,TMEM30A,MYRIP,PPP1R1C,KCNE2,UNC13C,CUL4A,KCTD16,EYA4,ZNF888,DLGAP2,ZNF736,SEMA5A,ELL2,LAMA4,PMEPA1,TTPA,PEX5L,PVT1,GATAD2B,METTL3,FMNL2,UAP1,PARD3,KCNN4,ZNF33A,CP,NPAS3,BMAL2,ATP8B1,RNF6,SEMA3C,VAV1,SMYD1,FANCB,PEPD,GRIA2,VDAC2,SPART
GO:0048856	anatomical structure development	5.82907738328216e-10	*FSTL1,APBB2,RTN1,HS3ST3A1,RGN,EPN2,MAP3K3,LRRN1,NFIA,SMYD3,ARHGEF9,ISM1,TRAPPC9,MAP3K13,ARL13B,LIMD1,TF,CHST11,THRB,SPTBN1,CDC42EP3,FGF1,CHRNB2,SEZ6,CDH8,ARHGAP15,GNB4,AGTPBP1,FHL1,ATP10A,SH3PXD2A,SPINK5,ATP7B,BRD4,DOK4,CAMK2B,GRIP1,DCT,KALRN,USH2A,NOTCH2,TMOD2,SDK2,NPNT,EPB41L3,MAP6,PARVB,CDH11,SHANK3,NR4A3,ARID1B,E2F7,FOXO1,STYXL1,SMURF2,ADAMTS12,DAAM2,PLXNA4,YBX1,STK24,DIPK2A,CACNA1C,ASB4,IDO1,ARB2A,PRKN,NLGN4X,EXOC4,KDM7A,SLC5A7,IFT172,CHD7,GPSM1,PTBP3,CDON,SERPINI1,SLC17A6,THSD7A,NCAM2,TIAM2,CFAP54,TRAK2,CNMD,LRP8,TAGLN3,CNTN1,MEOX2,STAT5B,ENPEP,EIF4ENIF1,HEATR9,CALCR,MBTPS2,PTH,ADAR,RHOJ,FGF10,MECOM,TSPAN18,PLG,CDH7,ILDR2,NCK1,NREP,EXO1,GABRA5,NEXMIF,KCNQ3,CHRM3,KDM2B,PREX1,COL11A1,ZNF804A,MCPH1,BRAF,COL1A2,TMOD3,MRTFB,COL12A1,ABLIM1,ALPK2,CHRNA7,HECW1,HMGB1,ZNF304,AFF3,RFLNA,ATRX,TGFB2,LAMA2,TRIO,HOXC13,SMARCC1,RAD51B,NDST1,MUSK,JAM3,MAP3K5,ALKBH1,ATP11A,SLC15A4,PCDHB8,PRKG2,IGFBP7,EPHA5,SKOR2,RUNX1,ENAH,SNX10,ATP2C1,ALDH1A2,FAM171A1,PCLO,HUWE1,ASPH,RRAS2,SIM1,KIAA1217,SOX30,CHL1,GSN,PLS1,SLIT3,ROBO1,ANKRD11,ITPR1,CPM,DNAAF11,PAK3,ABI3BP,AXDND1,MLLT3,CCNT2,BTG4,CPQ,MR1,TNFRSF12A,CPT2,CXCR5,NCKAP1,PRPF40A,FOSL2,PACRG,IL1RAPL2,EPHA3,LATS2,MYH14,ARHGAP18,MYOM2,LYN,ADGRB3,KLF7,GRIK1,DIP2B,SEC24C,LARGE1,PUS7,PDZD8,CA10,RRM2,CRYBB1,LDB2,NAV1,PAQR5,PCDHB16,SGMS2,NOS3,VSX1,ERCC6,EIF2AK4,XKR6,MATN2,TGFBR2,ITGA11,MARK4,TCF7L2,ZNF800,CRTAC1,ZFHX3,CUX1,UNC119,GPM6B,ADGRV1,EPB41L5,WHRN,OCA2,TDP2,PRUNE1,APP,GATA4,SMAD5,IGF2BP1,MAMLD1,RNF38,ADAM12,DHX35,HCN1,PI4KB,FRY,HOOK3,PALLD,CECR2,RIPK4,PTK2,SH2B2,CELF1,HYCC1,LRRK2,INO80D,IKZF3,CLSTN2,ITFG2,PKHD1,TENT5C,RARB,TCF4,PAEP,TIAM1,CSRP3,PHACTR1,ACVR2A,VIT,SNTG2,NTM,MEIS1,NEDD4L,CYB5R4,KIT,EPHA7,SPAG9,ANTXR1,WNT5A,CTNNB1,ANKRD17,COL19A1,CTSK,PRKDC,ESR1,TNFRSF19,PTPN14,SCFD1,ITGB8,POFUT2,BCAR3,LMX1A,WWOX,PTGER4,GREB1L,BBX,VWC2L,DOCK1,DIAPH3,GPC4,BPGM,EFNA*

			5,IGF2BP2,SYNE3,HECTD1,NGEF,TRPC5,NEUROD2,KAT14,TNFSF11,PTPRF,IKBKB,FXR2,AKAP6,USF3,LYVE1,APOH,ARHGEF28,TASOR,GPRIN1,POU6F2,LINGO2,OPCML,ATF2,ACVR1,PID1,EBF2,GRID2,ZNF423,RB1,MPV17,NTNG1,DDX10,PKD1L3,ADIPOR1,GABRB3,SHROOM4,PLPP3,HNF1B,IMMP2L,KIRREL3,GABRG2,CPS1,NCOA2,PRKCH,ACACB,AGPAT5,DLC1,XRCC5,GFRA1,SHLD2,BMX,SLC24A3,RAB18,ETV1,TEX11,LRTM2,SIK1,KAT2B,WLS,ANKS1A,PRCP,MEF2D,CDK5RAP2,FLVCR1,ARHGEF7,DACH1,ATF3,NPHP1,FBN2,IKZF1,SRI,ARNT,BDNF,LDLRAD4,TFCP2L1,SEMA3E,SCIN,MACO1,ADAMTS9,STIM1,KIF16B,CDH2,LAG3,PPARG,TANC1,USP9X,TGM5,TENM1,RORB,CDK6,GABRB1,SH3KBP1,AVIL,TFAP2D,TTC39C,SIPA1L3,SFRP4,BLTP1,IL1RAP,JCAD,VAV2,TFRC,VPS13A,FBN1,IQCB1,BMPER,FUT10,CLASP2,ZNF521,ANGPT1,SEMA6A,EIF4G1,PHLPP1,ITGA4,ROCK1,CLEC3A,PML,AFF2,TNFSF4,DGKG,SP100,PRKG1,ELAVL4,SBF2,CDH9,UMODL1,MAGI2,NIN,ASIC2,FNDC3A,AIMP1,PRKD1,CALCRL,SLC20A2,STK4,PCDH10,ANPEP,PRLR,EYS,CR2,LPAR6,TTN,HHIP,DYRK1A,TMEM30A,KCNE2,CUL4A,EYA4,SEMA5A,LAMA4,TTPA,METTL3,FMNL2,KRT85,PARD3,ATP8B1,RNF6,SEMA3C,VAV1,SMYD1,VPS13B,SPART
GO:0007399	nervous system development	7.523667426923303e-10	*APBB2,RTN1,LRRN1,NFIA,ARHGEF9,TRAPPC9,MAP3K13,ARL13B,THRB,SPTBN1,CHRNB2,SEZ6,GNB4,AGTPBP1,SPINK5,DOK4,CAMK2B,GRIP1,DCT,KALRN,USH2A,NOTCH2,TMOD2,SDK2,EPB41L3,MAP6,CDH11,SHANK3,ARID1B,STYXL1,DAAM2,PLXNA4,STK24,PRKN,NLGN4X,KDM7A,IFT172,CHD7,GPSM1,CDON,SERPINI1,NCAM2,TIAM2,TRAK2,LRP8,TAGLN3,CNTN1,EIF4ENIF1,FGF10,NCK1,NREP,GABRA5,NEXMIF,KCNQ3,CHRM3,KDM2B,PREX1,ZNF804A,MCPH1,BRAF,CHRNA7,HECW1,HMGB1,ATRX,TGFB2,LAMA2,TRIO,SMARCC1,NDST1,MUSK,JAM3,ALKBH1,PCDHB8,EPHA5,SKOR2,RUNX1,ENAH,ALDH1A2,PCLO,RRAS2,SIM1,CHL1,GSN,PLS1,SLIT3,ROBO1,PAK3,BTG4,NCKAP1,IL1RAPL2,EPHA3,LYN,ADGRB3,KLF7,GRIK1,DIP2B,LARGE1,CA10,LDB2,NAV1,PCDHB16,VSX1,ERCC6,EIF2AK4,MATN2,TGFBR2,MARK4,CRTAC1,ZFHX3,CUX1,UNC119,GPM6B,ADGRV1,WHRN,TDP2,PRUNE1,APP,IGF2BP1,HCN1,FRY,HOOK3,PALLD,CECR2,PTK2,SH2B2,CELF1,HYCC1,LRRK2,CLSTN2,RARB,TCF4,TIAM1,PHACTR1,VIT,SNTG2,NTM,MEIS1,NEDD4L,KIT,EPHA7,SPAG9,WNT5A,CTNNB1,PRKDC,LMX1A,VWC2L,DIAPH3,GPC4,BPGM,EFNA5,IGF2BP2,NGEF,TRPC5,NEUROD2,PTPRF,FXR2,ARHGEF28,GPRIN1,POU6F2,LINGO2,OPCML,ATF2,GRID2,ZNF423,RB1,NTNG1,GABRB3,SHROOM4,PLPP3,HNF1B,IMMP2L,KIRREL3,GABRG2,PRKCH,DLC1,XRCC5,GFRA1,RAB18,ETV1,LRTM2,KAT2B,WLS,ANKS1A,MEF2D,CDK5RAP2,ARHGEF7,BDNF,SEMA3E,SCIN,MACO1,CDH2,PPARG,USP9X,TENM1,RORB,CDK6,GABRB1,AVIL,TFAP2D,IL1RAP,VPS13A,FUT10,CLASP2,ZNF521,SEMA6A,EIF4G1,ITGA4,ROCK1,AFF2,DGKG,PRKG1,ELAVL4,SBF2,CDH9,MAGI2,NIN,ASIC2,PRKD1,S*

			TK4,PCDH10,HHIP,DYRK1A,TMEM30A,SEMA5A,TTPA,METTL3,PARD3,ATP8B1,RNF6,SEMA3C,VPS13B,SPART
GO:0032502	developmental process	1.1629925911382496e-9	FSTL1,APBB2,RTN1,HS3ST3A1,RGN,EPN2,MREG,MAP3K3,OSBPL8,LRRN1,NFIA,TXNDC8,SMYD3,ARHGEF9,ISM1,TRAPPC9,MAP3K13,ARL13B,LIMD1,TF,CHST11,THRB,USP42,SPTBN1,CDC42EP3,FGF1,CHRNB2,SEZ6,CDH8,ARHGAP15,GNB4,AGTPBP1,FHL1,ATP10A,SH3PXD2A,SPINK5,ATP7B,BRD4,DOK4,CAMK2B,GRIP1,DCT,KALRN,USH2A,NOTCH2,TMOD2,SDK2,NPNT,EPB41L3,MAP6,MORC1,PARVB,CDH11,SHANK3,NR4A3,ARID1B,E2F7,FOXO1,STYXL1,SMURF2,ADAMTS12,DAAM2,PLXNA4,YBX1,STK24,DIPK2A,CACNA1C,ASB4,IDO1,ARB2A,PRKN,NLGN4X,EXOC4,PNPLA3,KDM7A,SLC5A7,IFT172,CHD7,GPSM1,PTBP3,CDON,SERPINI1,SLC17A6,SDF4,THSD7A,NCAM2,TIAM2,CFAP54,TRAK2,CNMD,LRP8,TAGLN3,CNTN1,MEOX2,STAT5B,ENPEP,EIF4ENIF1,HEATR9,CALCR,MBTPS2,PTH,ADAR,CLCN3,RHOJ,FGF10,MECOM,CATSPERB,TSPAN18,PLG,CDH7,ILDR2,NCK1,NREP,EXO1,GABRA5,NEXMIF,KCNQ3,CHRM3,KDM2B,PREX1,COL11A1,ZNF804A,MCPH1,BRAF,COL1A2,TMOD3,MRTFB,COL12A1,ABLIM1,ALPK2,CHRNA7,HECW1,HMGB1,ZNF304,AFF3,RFLNA,ATRX,TGFB2,LAMA2,TRIO,HOXC13,SMARCC1,RAD51B,NDST1,MUSK,JAM3,MAP3K5,ALKBH1,ATP11A,SLC15A4,PCDHB8,PRKG2,SND1,IGFBP7,EPHA5,SKOR2,RUNX1,ENAH,SNX10,ATP2C1,ALDH1A2,FAM171A1,PCLO,HUWE1,ASPH,RRAS2,SIM1,KIAA1217,SOX30,CHL1,GSN,PLS1,SLIT3,PHC2,ROBO1,ANKRD11,ITPR1,CPM,DNAAF11,PAK3,ABI3BP,AXDND1,MLLT3,CCNT2,BTG4,CPQ,MR1,TNFRSF12A,CPT2,CXCR5,NCKAP1,PRPF40A,FOSL2,PACRG,IL1RAPL2,EPHA3,LATS2,MYH14,ARHGAP18,MYOM2,LYN,ADGRB3,KLF7,GRIK1,MTA3,DIP2B,SEC24C,LARGE1,ATP6V1H,FIGNL1,PUS7,PDZD8,CA10,RRM2,CRYBB1,LDB2,RNASEL,NAV1,PAQR5,PCDHB16,SGMS2,NOS3,VSX1,PRAMEF27,ERCC6,EIF2AK4,XKR6,MATN2,TGFBR2,ITGA11,MARK4,TCF7L2,ZNF800,CRTAC1,ZFHX3,CUX1,UNC119,GPM6B,ADGRV1,ST7,EPB41L5,WHRN,OCA2,TDP2,PRUNE1,APP,GATA4,SMAD5,IGF2BP1,MAMLD1,RNF38,ADAM12,DHX35,HCN1,PI4KB,FRY,HERC2,HOOK3,PALLD,CECR2,RIPK4,PTK2,SH2B2,CELF1,HYCC1,PRRC2B,LRRK2,INO80D,IKZF3,CLSTN2,ITFG2,PKHD1,TENT5C,RARB,TCF4,PAEP,TIAM1,CSRP3,PHACTR1,ACVR2A,VIT,SNTG2,NTM,MEIS1,NEDD4L,CYB5R4,KIT,EPHA7,SPAG9,ANTXR1,WNT5A,CTNNB1,ANKRD17,COL19A1,RUNX1T1,CTSK,PRKDC,ESR1,TNFRSF19,PTPN14,SCFD1,ITGB8,POFUT2,BCAR3,LMX1A,WWOX,PTGER4,GREB1L,BBX,VWC2L,DOCK1,DIAPH3,GPC4,BPGM,EFNA5,IGF2BP2,SYNE3,HECTD1,NGEF,TRPC5,NEUROD2,KAT14,TNFSF11,PTPRF,IKBKB,FXR2,AKAP6,USF3,LYVE1,APOH,ARHGEF28,TASOR,GPRIN1,POU6F2,LINGO2,OPCML,ATF2,ACVR1,PID1,EBF2,GRID2,ZNF423,RB1,MPV17,NTNG1,DDX10,PKD1L3,ADIPOR1,GABRB3,SHROOM4,PLPP3,HNF1B,IMMP2L,KIRREL3,G

			ABRG2,CPS1,NCOA2,TMEM120A,PRKCH,ACACB,AGPAT5,DLC1,XRCC5,GFRA1,SHLD2,BMX,SLC24A3,RAB18,ETV1,TEX11,LRTM2,SIK1,KAT2B,WLS,ANKS1A,PRCP,MEF2D,CDK5RAP2,FLVCR1,ARHGEF7,DACH1,ATF3,NPHP1,FBN2,IKZF1,SLC26A8,SRI,ARNT,BDNF,LDLRAD4,TFCP2L1,SEMA3E,SCIN,MACO1,ADAMTS9,STIM1,KIF16B,CDH2,COX7B2,LAG3,PPARG,TANC1,USP9X,TGM5,TENM1,RORB,CDK6,GABRB1,SH3KBP1,AVIL,TFAP2D,TTC39C,SIPA1L3,SFRP4,BLTP1,IL1RAP,JCAD,VAV2,TFRC,VPS13A,FBN1,DDX4,IQCB1,BMPER,FUT10,CLASP2,CLTC,ZNF521,ANGPT1,SEMA6A,EIF4G1,ATP6V0A4,PHLPP1,ITGA4,ROCK1,M1AP,CLEC3A,PML,AFF2,TNFSF4,DGKG,SPATA25,SP100,PRKG1,ELAVL4,SBF2,CDH9,UMODL1,MAGI2,NIN,ASIC2,FNDC3A,AIMP1,PRKD1,CALCRL,SLC20A2,STK4,PCDH10,ANPEP,PRLR,EYS,EDARADD,CR2,LPAR6,CTBP2,TTN,HHIP,DYRK1A,TMEM30A,KCNE2,UNC13C,CUL4A,TXNRD3,EYA4,SEMA5A,LAMA4,TTPA,GATAD2B,METTL3,FMNL2,KRT85,PARD3,ATP8B1,RNF6,SEMA3C,VAV1,SMYD1,VPS13B,SPART
GO:0035556	intracellular signal transduction	1.5530911011929646e-9	*APBB2,RGN,MAP3K3,OSBPL8,RAPGEF6,ARHGEF9,ABR,MAP3K13,PPM1A,LIMD1,TF,THRB,SPTBN1,CDC42EP3,MAPK10,USP32,FGF1,SEZ6,ARHGAP15,RBPMS,BRD4,DOK4,GRIP1,TLK1,SLC38A9,KALRN,MAP4K3,NOTCH2,NPNT,SHANK3,NR4A3,E2F7,FOXO1,STYXL1,STK39,URB2,STK24,DIPK2A,DCDC1,CACNA1C,ASB4,PRKN,PIK3C2B,CDON,NLK,GNAI1,MAPKAPK3,TIAM2,ARHGEF4,DDIAS,CALCR,ELMO1,ERBIN,MBTPS2,PTH,PDE10A,RHOJ,FGF10,MECOM,STK32B,HERC5,NCK1,NONO,MTCP1,CHRM3,PREX1,CIDEB,BRAF,COL1A2,CHRNA7,HMGB1,CDIP1,ATRX,TGFB2,MDFIC,TRIO,NDST1,MUSK,SFPQ,MAP3K5,NUP93,RERG,PRKAR2A,SLC15A4,GOLPH3,MBTPS1,PIK3C3,USP10,EPHA5,ENSG00000289694,ATP2C1,ALDH1A2,ASPH,RRAS2,UBR5,TLR8,ROBO1,ITPR1,PAK3,DOK6,PRKAG2,OASL,CXCR5,NCKAP1,CILK1,LATS2,ERP29,ARHGAP18,LYN,FIGNL1,PPM1L,NOS3,ERCC6,NOS1AP,EIF2AK4,RAB12,TGFBR2,MARK4,TCF7L2,RRAGD,ADGRV1,RASGRF2,WAC,DAPK1,APP,GATA4,SMAD5,GSKIP,PELI2,PI4KB,RHOT1,PIK3R5,BMT2,AVPR1B,PTK2,USP7,TICAM2,SH2B2,LRRK2,ARIH1,PLD1,ITFG2,PKHD1,RARB,PJA2,TIAM1,CSRP3,IQSEC2,ACVR2A,TIPIN,BRCC3,RABGEF1,KIT,EPHA7,SPAG9,SPDL1,WNT5A,ARFGEF2,CTNNB1,ANKRD17,PRKDC,IPO5,ESR1,TNFRSF19,BCAR3,TPD52L1,WWOX,HUNK,NEK10,PTGER4,PDE7B,DOCK1,CDC14B,PLCH1,NGEF,NEUROD2,FNIP2,TNFSF11,IKBKB,SH3BP5,AKAP6,ARHGEF28,ATF2,ACVR1,RB1,ADIPOR1,LAMP2,NCOA2,PRKCH,DLC1,DENND2B,BMX,RAB18,MGRN1,MID2,SIK1,TP53BP2,WLS,RYR3,PRCP,CDK5RAP2,ARHGEF7,ATF3,ATP1B1,ARNT,LDLRAD4,SEMA3E,TLR6,CDH2,PPARG,ASB3,USP9X,NEK7,TENM1,RORB,DDX60,MYO5A,SIPA1L3,JCAD,VAV2,TFRC,PNPLA8,HAPSTR1,MAP3K1,CLEC6A,RAPGEF4,BMPER,ANGPT1,SEMA6A,PHLPP1,ROCK1,TAX1BP1,APIP,PML,PLEK2,DGKG,SP100,PRKG1,MAGI2,SLC3*

			0A10,PRKD1,STK4,SPC25,PDE11A,NMI,RAB6C,LPAR6,CTBP2,TTN,NCALD,DYRK1A,PPP1R1C,CUL4A,SEMA5A,PMEPA1,PEX5L,RNF6,VAV1,VDAC2
GO:0065008	regulation of biological quality	1.690379148602376e-9	C10ORF90,SLC12A8,LRRN1,MAP3K13,LIMD1,TF,THRB,SPTBN1,CDC42EP3,CHRNB2,SEZ6,KCNQ5,CDH8,ARHGAP15,FHL1,ATP10A,ATP7B,CAMK2B,GRIP1,DDI2,SDR16C5,KALRN,TMOD2,EPB41L3,PARVB,SHANK3,FOXO1,STK39,DAAM2,PLXNA4,YBX1,CACNA1C,PRKN,NLGN4X,F13A1,CHD7,SLC17A6,NLK,LRP8,STAT5B,ENPEP,DDIAS,EIF4ENIF1,CALCR,CHRFAM7A,ERBIN,CLCN3,RHOJ,CDC37,FGF10,TSPAN18,PLG,ILDR2,NCK1,GABRA5,KCNQ3,CHRM3,PREX1,ANO3,ZNF804A,BRAF,COL1A2,TMOD3,CHRNA7,HMGB1,ADD3,MUSK,JAM3,PNPLA4,RBM33,ATP11A,EPHA5,ALDH1A2,PTGFR,FAM171A1,PCLO,ASPH,GSN,PLS1,ITPR1,PAK3,TMEM183A,CPQ,CNOT10,F8,CXCR5,NCKAP1,PRPF40A,DTD1,FOSL2,IL1RAPL2,MYH14,ARHGAP18,PTPRN2,LYN,ADGRB3,KLF7,GRIK1,DIP2B,LARGE1,ATP6V1H,SLC12A1,SPTB,ALDH1A1,RNASEL,CORIN,DCP2,NOS3,TNRC6C,NOS1AP,EIF2AK4,XKR6,TCF7L2,ADGRV1,RASGRF2,SERBP1,SCG5,OCA2,INSYN2A,APP,IGF2BP1,RNLS,ATP9B,CACNA2D1,HCN1,RHOT1,AVPR1B,PTK2,USP7,SCP2,CELF1,LRRK2,CLSTN2,TENT5C,TIAM1,CSRP3,IQSEC2,NEDD4L,CYB5R4,KIT,EPHA7,WNT5A,CTNNB1,CTSK,PRKDC,ESR1,ERC2,PTGER4,GPC4,EFNA5,IGF2BP2,SYNE3,NGEF,TRPC5,NEUROD2,TNFSF11,FXR2,AKAP6,APOH,CLCN1,LINGO2,ATF2,HDAC8,CHRNB3,PID1,GRID2,ZCCHC17,KCNJ3,PLPP3,HNF1B,LAMP2,GABRG2,F5,CPS1,BNIP3L,DLC1,TBRG4,KCND2,TAF15,GRIA4,RGS7BP,LRTM2,KAT2B,PRCP,MYL4,ARHGEF7,TRDN,CAPZA2,SRI,ATP1B1,ARNT,BDNF,SEMA3E,SCIN,TENT4B,TMEM161B,RAB11FIP2,CDH2,PPARG,TANC1,CHRNB4,USP9X,TENM1,GABRB1,SH3KBP1,NALCN,AVIL,USP25,LRFN2,SPTBN5,IL1RAP,VAV2,GRIA3,RAPGEF4,CLASP2,ANGPT1,SEMA6A,EIF4G1,ATP6V0A4,ITGA4,ROCK1,PML,DGKG,PRKG1,ELAVL4,NDUFAF2,ASIC2,ENSA,AIMP1,PRKD1,STK4,TNNI3K,PRLR,ZSWIM7,GNA14,NCALD,PPFIA1,TMEM30A,MYRIP,KCNE2,UNC13C,SEMA5A,METTL3,FMNL2,KCNN4,ATP8B1,RNF6,SEMA3C,VAV1,GRIA2,VDAC2,SPART
GO:0006810	transport	2.0628459447197618e-9	ABCC6,ESYT1,EPN2,MREG,NOSTRIN,SLC12A8,OSBPL8,STX8,BLTP3B,TRAPPC9,SLC16A5,PPM1A,TF,RCSD1,SPTBN1,MYO5C,CACNB1,CHRNB2,KCNQ5,AGTPBP1,FHL1,ATP10A,ATP7B,KCNJ6,CAMK2B,GRIP1,TLK1,SLC38A9,ABCG8,RGPD2,XPO4,KALRN,CD93,SLC44A5,MAP6,NR4A3,SLC14A2,FOXO1,STK39,YBX1,CACNA1C,PRKN,NLGN4X,EXOC4,XPO6,SLC44A3,SLC5A7,IFT172,CHD7,SLC17A6,MAPKAPK3,CFAP54,TRAK2,LRP8,CNTN1,STAT5B,UBE2O,EIF4ENIF1,CHRFAM7A,ELMO1,PTH,ADAR,CLCN3,RHOJ,FGF10,ZFYVE16,TSPAN18,ILDR2,CD84,GABRA5,KCNQ3,CHRM3,RGPD3,ANO3,CIDEB,SLC45A1,MYO1D,SESTD1,BRAF,XPO7,KCNS3,CHRNA7,HMGB1,DYNLT2B,TGFB2,MDFIC,KCNJ18,KCNIP3,NUP93,RBM

			33,TBC1D5,ATP11A,ATP4A,SLC15A4,GOLPH3,PRKG2,MBTPS1,NCF2,PIK3C3,EPHA5,ATP13A4,SNX10,ATP2C1,TMEM230,STON2,STRADA,PCLO,ASPH,UBR5,SOX30,GSN,ANO2,HPS3,ITPR1,DNAAF11,PRKAG2,HAVCR1,TRAPPC10,CPT2,VPS8,MTX3,CACNA2D3,CILK1,FOSL2,EPHA3,ERP29,UVRAG,PTPRN2,LYN,USP6,KLF7,GRIK1,SEC24C,LARGE1,ATP6V1H,NUP58,SLC12A1,PDZD8,SIL1,C21ORF62,RNASEL,CORIN,TMCC1,TMED7-TICAM2,SLC41A2,NOS3,KCNIP4,NOS1AP,XKR6,MAGEL2,TGFBR2,MDN1,SYNJ1,TCF7L2,CUX1,UNC119,GPM6B,CACNA2D4,RGPD1,KPNB1,GSDMD,SCG5,OCA2,DAPK1,SAR1B,APP,IGF2BP1,ATP9B,CACNA2D1,HCN1,CHRM5,SLC13A1,PI4KB,RHOT1,HERC2,HOOK3,USE1,CECR2,TMCO3,AVPR1B,LMAN1L,PTK2,NOP9,USP7,TICAM2,SCP2,ATP1B4,PIP4K2B,LRRK2,NKAIN3,SLC38A4,PLD1,MGST1,TIAM1,NEDD4L,ENSG00000288683,SNX13,RABGEF1,CYB5R4,KIT,SPAG9,OSBPL3,WNT5A,PEX26,ARFGEF2,TEX2,CTNNB1,IPO5,PTPN14,SCFD1,ERC2,POFUT2,VPS53,FRMD4A,NEK10,BEST3,DOCK1,DIAPH3,BPGM,PKIG,EFNA5,IGF2BP2,SYNE3,PLCH1,TRPC5,TNFSF11,AKAP6,LYVE1,APOH,CLCN1,RAB3GAP2,CEMIP,ATF2,KCNJ12,CHRNB3,PID1,CCDC91,GRID2,KCNJ3,MPV17,PDE4B,PKD1L3,KIF13A,GABRB3,GOSR2,SMG7,PLPP3,UNC80,HNF1B,LAMP2,GABRG2,TMEM120A,BNIP3L,ACACB,RABGAP1L,KCND2,SLC24A3,GRIA4,RAB18,MGRN1,SIK1,GOLPH3L,WLS,RYR3,MYO1B,FLVCR1,ARHGEF7,TRDN,SLC26A8,CACHD1,SRI,ATP1B1,SCIN,ADAMTS9,STIM1,KIF16B,RAB11FIP2,CDH2,PPARG,MTTP,CHRNB4,USP9X,TENM1,IL13RA2,GABRB1,MYOF,JAKMIP1,SH3KBP1,NALCN,MYO5A,SLC6A13,SPTBN5,SFRP4,BLTP1,VAV2,TFRC,VPS13A,OSBPL6,GRIA3,PNPLA8,RAB31,RAPGEF4,CLASP2,CLTC,ANGPT1,ATP6V0A4,ITGA4,DNAJC6,ROCK1,TBC1D4,KCNT2,NUP98,PML,SCFD2,SCARA5,SLC5A12,SP100,PRKG1,NDUFAF2,MAGI2,ANKFY1,ASIC2,ENSA,AIMP1,SLC30A10,AP4S1,PRKD1,CALCRL,SLC20A2,STK4,RAB6D,SCAMP1,PRLR,RAB6C,IL16,CTBP2,TTN,NCALD,TMEM30A,MYRIP,KCNE2,UNC13C,TTPA,PEX5L,KCNN4,SLC25A48,CP,ATP8B1,VAV1,GRIA2,VDAC2,VPS13B
GO:0051179	localization	4.794103695271617e-9	*ABCC6,ESYT1,EPN2,MREG,NOSTRIN,SLC12A8,OSBPL8,STX8,SMYD3,RAPGEF6,BLTP3B,TRAPPC9,SLC16A5,PPM1A,ARL13B,RN7SL556P,IMMP1L,TF,CHST11,RCSD1,SPTBN1,MYO5C,CACNB1,CHRNB2,KCNQ5,AGTPBP1,FHL1,ATP10A,ATP7B,KCNJ6,CAMK2B,GRIP1,TLK1,SLC38A9,ABCG8,RGPD2,XPO4,KALRN,USH2A,CD93,SLC44A5,NPNT,EPB41L3,MAP6,SHANK3,NR4A3,SLC14A2,FOXO1,STK39,YBX1,CACNA1C,PRKN,NLGN4X,EXOC4,XPO6,SLC44A3,SLC5A7,IFT172,CHD7,GPSM1,SLC17A6,GNAI1,MAPKAPK3,CFAP54,INTS6-AS1,TRAK2,LRP8,CNTN1,STAT5B,UBE2O,EIF4ENIF1,CHRFAM7A,ELMO1,ERBIN,CD247,MCC,CEP192,PTH,ADAR,TM9SF2,CLCN3,RHOJ,CDC37*

			,FGF10,ZFYVE16,TSPAN18,ILDR2,CD84,GABRA5,KCNQ3,CHRM3,RGPD3,ANO3,CIDEB,SLC45A1,MCPH1,MYO1D,SESTD1,BRAF,XPO7,KCNS3,CHRNA7,HMGB1,DYNLT2B,ATRX,TGFB2,MDFIC,MUSK,KCNJ18,JAM3,KCNIP3,NUP93,RBM33,TBC1D5,ATP11A,ATP4A,SLC15A4,GOLPH3,PRKG2,MBTPS1,NCF2,PIK3C3,EPHA5,ATP13A4,SNX10,ATP2C1,TMEM230,STON2,STRADA,PCLO,HUWE1,ASPH,ZDHHC4,UBR5,SOX30,GSN,PLS1,MAPRE3,ANO2,HPS3,ITPR1,DNAAF11,PRKAG2,HAVCR1,TRAPPC10,CPT2,VPS8,MTX3,CACNA2D3,CILK1,FOSL2,PACRG,EPHA3,LATS2,ERP29,UVRAG,PTPRN2,LYN,USP6,KLF7,GRIK1,SEC24C,LARGE1,ATP6V1H,NUP58,SLC12A1,PDZD8,SIL1,C21ORF62,RNASEL,CORIN,TMCC1,DCP2,TMED7-TICAM2,SLC41A2,NOS3,KCNIP4,NOMO2,NOS1AP,RN7SL738P,KIAA1614,XKR6,MAGEL2,TGFBR2,MARK4,MDN1,SYNJ1,TCF7L2,CUX1,UNC119,GPM6B,RRAGD,CACNA2D4,RGPD1,KPNB1,GSDMD,ADGRV1,EPB41L5,SCG5,WHRN,OCA2,DAPK1,SAR1B,TTC7B,APP,IGF2BP1,ATP9B,CACNA2D1,HCN1,CHRM5,SLC13A1,PI4KB,RHOT1,HERC2,HOOK3,USE1,CECR2,TMCO3,AVPR1B,LMAN1L,PTK2,NOP9,USP7,TICAM2,SCP2,ATP1B4,HYCC1,PIP4K2B,LRRK2,NKAIN3,GET1-SH3BGR,SLC38A4,PLD1,PKHD1,MGST1,TIAM1,CSRP3,IQSEC2,NEDD4L,ENSG00000288683,SNX13,RABGEF1,CYB5R4,KIT,SPAG9,OSBPL3,SPDL1,WNT5A,WIPI2,PEX26,ARFGEF2,TEX2,CTNNB1,IPO5,ESR1,PTPN14,SCFD1,ERC2,POFUT2,VPS53,FRMD4A,NEK10,BEST3,DOCK1,DIAPH3,GPC4,BPGM,PKIG,EFNA5,IGF2BP2,SYNE3,PLCH1,TRPC5,TNFSF11,IKBKB,AKAP6,LYVE1,APOH,CLCN1,RAB3GAP2,TASOR,CEMIP,XIST,ATF2,KCNJ12,CHRNB3,PID1,CCDC91,GRID2,ZNF423,KCNJ3,RB1,MPV17,PDE4B,PKD1L3,KIF13A,GABRB3,GOSR2,SMG7,PLPP3,UNC80,HNF1B,IMMP2L,LAMP2,GABRG2,TMEM120A,BNIP3L,PRKCH,ACACB,RABGAP1L,XRCC5,KCND2,SLC24A3,GRIA4,RAB18,MGRN1,MID2,SIK1,KAT2B,GOLPH3L,WLS,RYR3,MYO1B,CDK5RAP2,FLVCR1,ARHGEF7,TRDN,NPHP1,FBN2,SLC26A8,CACHD1,SRI,ATP1B1,BDNF,SCIN,ADAMTS9,STIM1,KIF16B,RAB11FIP2,CDH2,PPARG,ASB3,MTTP,CHRNB4,USP9X,TENM1,IL13RA2,GABRB1,MYOF,JAKMIP1,SH3KBP1,NALCN,GDAP1,KIF2C,MYO5A,SLC6A13,SPTBN5,SFRP4,BLTP1,VAV2,TFRC,VPS13A,OSBPL6,GRIA3,PNPLA8,FBN1,RAB31,RAPGEF4,BMPER,FUT10,CLASP2,CLTC,ANGPT1,EIF4G1,ATP6V0A4,ITGA4,DNAJC6,ROCK1,TAX1BP1,TBC1D4,KCNT2,NUP98,PML,SCFD2,SCARA5,SLC5A12,SP100,PRKG1,NDUFAF2,MAGI2,NIN,ANKFY1,ASIC2,ENSA,AIMP1,SLC30A10,AP4S1,PRKD1,CALCRL,SLC20A2,STK4,RAB6D,SPC25,SCAMP1,PRLR,RAB6C,IL16,CTBP2,TTN,NCALD,PPFIA1,TMEM30A,MYRIP,KCNE2,UNC13C,TTPA,PEX5L,PARD3,KCNN4,SLC25A48,CP,ATP8B1,VAV1,GRIA2,VDAC2,VPS13B
GO:0051234	establishment of	9.392704118682859e-9	ABCC6,ESYT1,EPN2,MREG,NOSTRIN,SLC12A8,OSBPL8,STX8,SMYD3,BLTP3B,TRAPPC9,SLC16

	localization		*A5,PPM1A,RN7SL556P,IMMP1L,TF,RCSD1,SPTBN1,MYO5C,CACNB1,CHRNB2,KCNQ5,AGTPBP1,FHL1,ATP10A,ATP7B,KCNJ6,CAMK2B,GRIP1,TLK1,SLC38A9,ABCG8,RGPD2,XPO4,KALRN,USH2A,CD93,SLC44A5,NPNT,MAP6,NR4A3,SLC14A2,FOXO1,STK39,YBX1,CACNA1C,PRKN,NLGN4X,EXOC4,XPO6,SLC44A3,SLC5A7,IFT172,CHD7,GPSM1,SLC17A6,MAPKAPK3,CFAP54,INTS6-AS1,TRAK2,LRP8,CNTN1,STAT5B,UBE2O,EIF4ENIF1,CHRFAM7A,ELMO1,ERBIN,MCC,PTH,ADAR,CLCN3,RHOJ,CDC37,FGF10,ZFYVE16,TSPAN18,ILDR2,CD84,GABRA5,KCNQ3,CHRM3,RGPD3,ANO3,CIDEB,SLC45A1,MCPH1,MYO1D,SESTD1,BRAF,XPO7,KCNS3,CHRNA7,HMGB1,DYNLT2B,TGFB2,MDFIC,KCNJ18,KCNIP3,NUP93,RBM33,TBC1D5,ATP11A,ATP4A,SLC15A4,GOLPH3,PRKG2,MBTPS1,NCF2,PIK3C3,EPHA5,ATP13A4,SNX10,ATP2C1,TMEM230,STON2,STRADA,PCLO,HUWE1,ASPH,ZDHHC4,UBR5,SOX30,GSN,ANO2,HPS3,ITPR1,DNAAF11,PRKAG2,HAVCR1,TRAPPC10,CPT2,VPS8,MTX3,CACNA2D3,CILK1,FOSL2,EPHA3,ERP29,UVRAG,PTPRN2,LYN,USP6,KLF7,GRIK1,SEC24C,LARGE1,ATP6V1H,NUP58,SLC12A1,PDZD8,SIL1,C21ORF62,RNASEL,CORIN,TMCC1,TMED7-TICAM2,SLC41A2,NOS3,KCNIP4,NOMO2,NOS1AP,RN7SL738P,XKR6,MAGEL2,TGFBR2,MDN1,SYNJ1,TCF7L2,CUX1,UNC119,GPM6B,CACNA2D4,RGPD1,KPNB1,GSDMD,ADGRV1,EPB41L5,SCG5,WHRN,OCA2,DAPK1,SAR1B,APP,IGF2BP1,ATP9B,CACNA2D1,HCN1,CHRM5,SLC13A1,PI4KB,RHOT1,HERC2,HOOK3,USE1,CECR2,TMCO3,AVPR1B,LMAN1L,PTK2,NOP9,USP7,TICAM2,SCP2,ATP1B4,PIP4K2B,LRRK2,NKAIN3,GET1-SH3BGR,SLC38A4,PLD1,PKHD1,MGST1,TIAM1,NEDD4L,ENSG00000288683,SNX13,RABGEF1,CYB5R4,KIT,SPAG9,OSBPL3,SPDL1,WNT5A,PEX26,ARFGEF2,TEX2,CTNNB1,IPO5,PTPN14,SCFD1,ERC2,POFUT2,VPS53,FRMD4A,NEK10,BEST3,DOCK1,DIAPH3,BPGM,PKIG,EFNA5,IGF2BP2,SYNE3,PLCH1,TRPC5,TNFSF11,AKAP6,LYVE1,APOH,CLCN1,RAB3GAP2,CEMIP,XIST,ATF2,KCNJ12,CHRNB3,PID1,CCDC91,GRID2,KCNJ3,RB1,MPV17,PDE4B,PKD1L3,KIF13A,GABRB3,GOSR2,SMG7,PLPP3,UNC80,HNF1B,IMMP2L,LAMP2,GABRG2,TMEM120A,BNIP3L,ACACB,RABGAP1L,KCND2,SLC24A3,GRIA4,RAB18,MGRN1,SIK1,KAT2B,GOLPH3L,WLS,RYR3,MYO1B,CDK5RAP2,FLVCR1,ARHGEF7,TRDN,SLC26A8,CACHD1,SRI,ATP1B1,SCIN,ADAMTS9,STIM1,KIF16B,RAB11FIP2,CDH2,PPARG,MTTP,CHRNB4,USP9X,TENM1,IL13RA2,GABRB1,MYOF,JAKMIP1,SH3KBP1,NALCN,GDAP1,KIF2C,MYO5A,SLC6A13,SPTBN5,SFRP4,BLTP1,VAV2,TFRC,VPS13A,OSBPL6,GRIA3,PNPLA8,RAB31,RAPGEF4,FUT10,CLASP2,CLTC,ANGPT1,ATP6V0A4,ITGA4,DNAJC6,ROCK1,TBC1D4,KCNT2,NUP98,PML,SCFD2,SCARA5,SLC5A12,SP100,PRKG1,NDUFAF2,MAGI2,ANKFY1,ASIC2,ENSA,AIMP1,SLC30A10,AP4S1,PRKD1,CALCRL,SLC20A2,STK4,RAB6D,SPC25,SCAMP1,PRLR,RAB6C,IL16,CTBP2,TTN,NCALD*

			,TMEM30A,MYRIP,KCNE2,UNC13C,TTPA,PEX5L,PARD3,KCNN4,SLC25A48,CP,ATP8B1,VAV1,GRIA2,VDAC2,VPS13B
GO:0000902	cell morphogenesis	9.488027979915399e-9	APBB2,NFIA,MAP3K13,ARL13B,LIMD1,CDC42EP3,CHRNB2,CDH8,ARHGAP15,ATP10A,CAMK2B,GRIP1,KALRN,NOTCH2,EPB41L3,MAP6,PARVB,CDH11,SHANK3,PLXNA4,PRKN,TIAM2,LRP8,CNTN1,RHOJ,CDH7,BRAF,CHRNA7,HECW1,TGFB2,LAMA2,TRIO,MUSK,EPHA5,SKOR2,ENAH,FAM171A1,CHL1,PLS1,SLIT3,ROBO1,ITPR1,PAK3,NCKAP1,PRPF40A,FOSL2,EPHA3,LATS2,MYH14,ARHGAP18,ADGRB3,KLF7,DIP2B,PDZD8,EIF2AK4,MATN2,CUX1,EPB41L5,WHRN,APP,IGF2BP1,FRY,PALLD,PTK2,LRRK2,PKHD1,TIAM1,PHACTR1,NEDD4L,KIT,EPHA7,SPAG9,WNT5A,CTNNB1,PRKDC,SCFD1,LMX1A,EFNA5,SYNE3,NGEF,TRPC5,ARHGEF28,RB1,NTNG1,KIRREL3,DLC1,ETV1,LRTM2,ARHGEF7,BDNF,TFCP2L1,SEMA3E,CDH2,USP9X,SH3KBP1,SIPA1L3,CLASP2,SEMA6A,ITGA4,ROCK1,PRKG1,ELAVL4,CDH9,NIN,STK4,SEMA5A,METTL3,FMNL2,PARD3,RNF6,SEMA3C,SPART
GO:0050794	regulation of cellular process	2.9332747420695064e-8	FSTL1,C10ORF90,APBB2,STYK1,RTN1,RGN,CDK14,EPN2,PDE1C,NOSTRIN,ING3,MAP3K3,OSBPL8,LRRN1,MED13L,NFIA,STX8,PHF20,FOXK2,SMYD3,RAPGEF6,ARHGEF9,TAFA2,ABR,MAOA,MAP3K13,PPM1A,ARL13B,SAMSN1,LIMD1,TF,CHST11,THRB,USP42,SPTBN1,CDC42EP3,MAPK10,CACNB1,USP32,DAPP1,ATF7,GNG12,FGF1,CHRNB2,CDC14C,SEZ6,CDH8,ARHGAP15,GNB4,AGTPBP1,ATF7-NPFF,FHL1,RBPMS,ATP10A,SPINK5,OPA3,SKAP2,GPBP1L1,KCNJ6,BRD4,DOK4,CAMK2B,GRIP1,ARHGAP11A-SCG5,TLK1,DCT,SLC38A9,ZNF264,HBP1,SDR16C5,ABCG8,XPO4,KALRN,USH2A,MAP4K3,NOTCH2,TMOD2,HMBOX1,ZNF551,NPNT,MAP6,MORC1,CDH11,SHANK3,NR4A3,ARID1B,E2F7,FOXO1,ZNF611,STYXL1,STK39,SMURF2,ADAMTS12,DAAM2,FER1L6,TBC1D16,PLXNA4,CDK18,URB2,YBX1,STK24,DIPK2A,DCDC1,CACNA1C,FAM3C,ASB4,IDO1,ARB2A,PRKN,NLGN4X,EXOC4,PIK3C2B,KDM7A,FAM83B,IFT172,CHD7,GPSM1,CBLL2,PTBP3,CDON,SERPINI1,NLK,GNAI1,MRPL13,MAPKAPK3,TIAM2,ZNF10,CNMD,LRP8,TAGLN3,CNTN1,MEOX2,STAT5B,ARHGEF4,UBE2O,DDIAS,CPEB4,EIF4ENIF1,CALCR,CHRFAM7A,ELMO1,ERBIN,ZNF121,MBTPS2,RGS22,CD247,MCC,PTH,ADAR,PDE10A,TM9SF2,TOX2,CLCN3,RHOJ,CDC37,FGF10,ZFYVE16,MECOM,COL4A6,STK32B,PLG,HERC5,UBE2E2,ILDR2,NCK1,CD84,FAM168A,NONO,RARRES1,KLHL42,NREP,GABRA5,NEXMIF,KCNQ3,MTCP1,CHRM3,KDM2B,PREX1,ZNF404,WRNIP1,RSF1,NCOA7,ZNF804A,CIDEB,MCPH1,SESTD1,BRAF,ZNF214,COL1A2,DTNA,TMOD3,GEMIN5,MRTFB,ALPK2,CHRNA7,HECW1,HMGB1,ZNF304,DYNLT2B,CDIP1,AFF3,RFLNA,HIVEP1,ATRX,TGFB2,MDFIC,LAMA2,ADD3,TRIO,ZNF85,HOXC13,SMARCC1,RAD51B,NDST1,MUSK,KCNJ18,JAM3,KCNIP3,SFPQ,MAP3K5,ALKBH1,RGS3,NUP93,ZCCHC4,RERG,OR5AU1,PRKAR2A

			,RBM33,TBC1D5,CTIF,ATP11A,RBM23,ZNF154,ATP4A,SLC15A4,GOLPH3,PRKG2,MBTPS1,PIK3C3,USP10,SND1,IGFBP7,EPHA5,SKOR2,ENSG00000289694,RUNX1,ATP2C1,ALDH1A2,PPTC7,STON2,PTGFR,STRADA,PCLO,HUWE1,ZNF595,ASPH,RRAS2,SH3BGRL,UBR5,GTF2F2,SIM1,SOX30,ANKS1B,CHL1,SPOCK3,GSN,PLS1,TCP11L1,SPON1,MAPRE3,TLR8,SLIT3,ARMH3,CACUL1,NLRP7,ARHGEF39,PHC2,ROBO1,ZNF578,ITPR1,PKP4,PAK3,ABI3BP,KLF12,DOK6,PRKAG2,MLLT3,CCNT2,BTG4,HAVCR1,CNOT10,MR1,TNFRSF12A,OASL,CPT2,TAS2R14,VPS8,ZNF429,CXCR5,NCKAP1,PRPF40A,CILK1,FOSL2,IL1RAPL2,ADGRD1,EPHA3,LATS2,SPDYE2B,ERP29,CDYL2,ARHGAP18,UVRAG,LYN,ADGRB3,USP6,KLF7,SUMO2,GRIK1,MTA3,DIP2B,NME7,LARGE1,ATP6V1H,NUP58,FIGNL1,PUS7,RRM2,LDB2,MYRFL,SPTB,ALDH1A1,FMO5,RNASEL,DBF4B,DCP2,PPM1L,TMED7-TICAM2,NOS3,KCNIP4,VSX1,ZNF280B,TNRC6C,PRAMEF27,MLLT10,ERCC6,NOS1AP,EIF2AK4,KIAA1614,RAB12,MAGEL2,TGFBR2,CMKLR2,ITGA11,MARK4,SYNJ1,TCF7L2,ZNF800,ZNF462,E2F6,ZFHX3,CUX1,UNC119,SHISA2,GPM6B,RRAGD,ULK3,SPDYE2,GPR156,GKAP1,PPP2R5E,GSDMD,ADGRV1,RASGRF2,ST7,SERBP1,EPB41L5,SCG5,WAC,WHRN,DAPK1,TDP2,ZNF678,PRUNE1,SAR1B,INSYN2A,BTG3,APP,GATA4,ATF7IP2,SMAD5,IGF2BP1,MAMLD1,GSKIP,ADAM12,PELI2,CACNA2D1,SP140L,HCN1,CHRM5,PI4KB,FRY,RHOT1,ELOVL5,HOOK3,PIK3R5,TLR1,TAS2R30,BMT2,RIPK4,AVPR1B,PTK2,USP7,TICAM2,SCP2,SH2B2,ATP1B4,ZNF41,AVEN,CELF1,POGZ,PIP4K2B,LRRK2,INO80D,ZNF395,IKZF3,CLSTN2,ARIH1,CFAP298,PLD1,ITFG2,VTCN1,PKHD1,TENT5C,RARB,PJA2,TCF4,PAEP,COP1,TIAM1,CSRP3,PHACTR1,MLIP,IQSEC2,ACVR2A,GNAO1,TIPIN,VIT,RASSF4,MEIS1,NEDD4L,BRCC3,SNX13,RABGEF1,KIT,TBC1D8,EPHA7,SPAG9,ANTXR1,SPDL1,WNT5A,TRABD2B,ARFGEF2,TEX2,CTNNB1,ANKRD17,RUNX1T1,CTSK,PRKDC,IPO5,ESR1,TNFRSF19,ZNF479,PTPN14,SCFD1,ITGB8,ERC2,POFUT2,BCAR3,TPD52L1,L3MBTL4,PPM1E,ZXDC,FRMD4A,LMX1A,WWOX,BNIPL,HUNK,NEK10,ANAPC10,PTGER4,ZNF215,BBX,PDE7B,VWC2L,DOCK1,DIAPH3,GPC4,PKIG,EFNA5,IGF2BP2,ZNF722,CDC14B,PLCH1,OR1M1,HECTD1,NGEF,TRPC5,NEUROD2,FNIP2,AASS,JAML,KAT14,TNFSF11,PTPRF,IKBKB,FXR2,SH3BP5,AKAP6,TRBV10-2,USF3,LYVE1,APOH,ARHGEF28,OR8B8,RAB3GAP2,TASOR,CCPG1,POU6F2,LINGO2,CEMIP,XIST,ATF2,ZNF426,KCNJ12,HDAC8,CHRNB3,ACVR1,HLA-DQA2,PID1,EBF2,GRID2,ZNF423,ZCCHC17,KCNJ3,RB1,NSMCE1,MPV17,IFNLR1,NTNG1,PDE4B,KIF13A,ADIPOR1,ZNF143,GABRB3,ZSCAN23,SMG7,PLPP3,HNF1B,ZNF615,LAMP2,NCOA2,BNIP3L,PRKCH,ACACB,RABGAP1L,DLC1,DENND2B,XRCC5,TBRG4,RGS17,GFRA1,SHLD2,INPP4A,BMX,MIR663AHG,ZNF418,SLC24A3,TAF15,GR

			IA4,RAB18,ZNF860,POU2F1,ETV1,RGS7BP,MGRN1,TEX11,MID2,MIR198,LRTM2,SIK1,TP53BP2,ZNF850,KAT2B,GOLPH3L,WLS,ANKS1A,RYR3,PRCP,SUPV3L1,MEF2D,GTF2A1,SPTLC1,CDK5RAP2,RFC3,ARHGEF7,SAMD12,DACH1,ATF3,TRDN,NPHP1,FBN2,IKZF1,CAPZA2,SRI,ATP1B1,ARNT,BDNF,LDLRAD4,TFCP2L1,SEMA3E,BCLAF3,SCIN,MALRD1,TENT4B,MACO1,ZNF665,TLR6,ADAMTS9,STIM1,TMEM237,HAUS6,KIF16B,RAB11FIP2,CDH2,EEFSEC,LAG3,PPARG,TANC1,PAPPA,ASB3,CHRNB4,USP9X,NEK7,ZNF80,TENM1,IL13RA2,RORB,CDK6,GABRB1,MYOF,LTB4R2,SH3KBP1,NALCN,AVIL,DDX60,TFAP2D,USP25,KIF2C,MYO5A,SIPA1L3,ZNF286A-TBC1D26,LRFN2,SPTBN5,SFRP4,BLTP1,SPDYE16,PNRC1,IL1RAP,JCAD,VAV2,TFRC,TES,GRIA3,PNPLA8,SPDYE6,FBN1,HAPSTR1,RAB31,MAP3K1,ZFP82,DDX4,IQCB1,CLEC6A,ZNF292,RAPGEF4,BMPER,CLASP2,CLTC,ZNF521,ZNF761,ANGPT1,SEMA6A,VIPR2,EIF4G1,PHLPP1,ZNF618,ITGA4,DNAJC6,ROCK1,NCAPH,TAX1BP1,TBC1D4,APIP,MMP28,ZNF701,NUP98,IKZF2,PML,FOXO3B,SACS,AFF2,MGLL,PLEK2,TNFSF4,AFAP1L2,DGKG,SP100,PRKG1,ELAVL4,NDUFAF2,CFAP298-TCP10L,UMODL1,RFPL4B,TTC28,MAGI2,NIN,SPG21,ZNF286A,ANKFY1,ZNF69,ASIC2,ENSA,MIR9-2HG,KHDRBS3,AIMP1,SLC30A10,HSPBP1,PRKD1,CALCRL,USP34,STK4,SPC25,PRLR,ZNF569,PTPRA,ZNF180,PDE11A,NMI,RAB6C,YLPM1,IL16,ZNF470,EDARADD,GNA14,CR2,LPAR6,CTBP2,TTN,NCALD,HHIP,DYRK1A,PPFIA1,TMEM30A,MYRIP,PPP1R1C,KCNE2,UNC13C,CUL4A,KCTD16,EYA4,ZNF888,ZNF736,SEMA5A,ELL2,LAMA4,PMEPA1,TTPA,PEX5L,PVT1,GATAD2B,METTL3,UAP1,PARD3,KCNN4,ZNF33A,CP,NPAS3,BMAL2,ATP8B1,RNF6,SEMA3C,VAV1,SMYD1,FANCB,PEPD,GRIA2,VDAC2,SPART
GO:0050793	regulation of developmental process	3.7366273950960134e-8	RGN,EPN2,LRRN1,ISM1,MAP3K13,LIMD1,CDC42EP3,FGF1,CHRNB2,SEZ6,ARHGAP15,ATP10A,SPINK5,BRD4,CAMK2B,GRIP1,DCT,KALRN,USH2A,NOTCH2,NPNT,EPB41L3,MAP6,PARVB,SHANK3,ARID1B,FOXO1,SMURF2,ADAMTS12,DAAM2,PLXNA4,YBX1,STK24,ASB4,PRKN,CHD7,PTBP3,CDON,TIAM2,CNMD,LRP8,STAT5B,EIF4ENIF1,PTH,RHOJ,FGF10,TSPAN18,NREP,KDM2B,PREX1,BRAF,MRTFB,ALPK2,CHRNA7,HECW1,HMGB1,ZNF304,RFLNA,TGFB2,LAMA2,TRIO,SMARCC1,MUSK,MAP3K5,ATP11A,PRKG2,SKOR2,RUNX1,FAM171A1,HUWE1,PLS1,ROBO1,PAK3,ABI3BP,CCNT2,PRPF40A,FOSL2,EPHA3,LATS2,MYH14,ARHGAP18,LYN,ADGRB3,KLF7,MTA3,DIP2B,PUS7,PDZD8,SGMS2,NOS3,PRAMEF27,EIF2AK4,TGFBR2,TCF7L2,ZFHX3,CUX1,GPM6B,ADGRV1,ST7,PRUNE1,APP,GATA4,SMAD5,ADAM12,HOOK3,PTK2,CELF1,LRRK2,INO80D,IKZF3,CLSTN2,PKHD1,TENT5C,RARB,TCF4,PAEP,TIAM1,CSRP3,ACVR2A,MEIS1,NEDD4L,KIT,EPHA7,SPAG9,WNT5A,CTNNB1,ANKRD17,RUNX1T1,CTSK,PRKDC,ESR1,ITGB8,POFUT2,LMX1A,VWC2L,EFNA5,

			SYNE3,NGEF,TRPC5,NEUROD2,KAT14,TNFSF11,PTPRF,IKBKB,AKAP6,USF3,APOH,LINGO2,ATF2,ACVR1,PID1,GRID2,RB1,NTNG1,ADIPOR1,HNF1B,NCOA2,PRKCH,ACACB,DLC1,XRCC5,SHLD2,TEX11,LRTM2,SIK1,KAT2B,CDK5RAP2,FLVCR1,ARHGEF7,FBN2,ARNT,BDNF,LDLRAD4,SEMA3E,SCIN,ADAMTS9,STIM1,CDH2,LAG3,PPARG,RORB,CDK6,SH3KBP1,SFRP4,BLTP1,IL1RAP,JCAD,TFRC,FBN1,IQCB1,BMPER,CLASP2,SEMA6A,EIF4G1,ROCK1,PML,TNFSF4,DGKG,SP100,ELAVL4,UMODL1,NIN,ASIC2,PRKD1,SLC20A2,STK4,PRLR,CUL4A,SEMA5A,LAMA4,TTPA,GATAD2B,METTL3,FMNL2,PARD3,RNF6,SEMA3C,SMYD1,SPART
GO:0048699	generation of neurons	4.7778638285217425e-8	*APBB2,RTN1,NFIA,TRAPPC9,MAP3K13,THRB,CHRNB2,SEZ6,AGTPBP1,SPINK5,CAMK2B,GRIP1,DCT,KALRN,USH2A,NOTCH2,SDK2,EPB41L3,MAP6,CDH11,SHANK3,STYXL1,PLXNA4,STK24,PRKN,NLGN4X,IFT172,CDON,SERPINI1,NCAM2,TIAM2,LRP8,CNTN1,EIF4ENIF1,NCK1,NREP,GABRA5,NEXMIF,KCNQ3,PREX1,ZNF804A,BRAF,CHRNA7,HECW1,HMGB1,TGFB2,LAMA2,TRIO,MUSK,ALKBH1,EPHA5,SKOR2,RUNX1,ENAH,ALDH1A2,CHL1,PLS1,SLIT3,ROBO1,PAK3,BTG4,NCKAP1,EPHA3,LYN,ADGRB3,KLF7,DIP2B,LARGE1,NAV1,VSX1,ERCC6,EIF2AK4,MATN2,CRTAC1,ZFHX3,CUX1,GPM6B,ADGRV1,WHRN,TDP2,APP,IGF2BP1,HCN1,FRY,PALLD,CECR2,PTK2,LRRK2,TCF4,TIAM1,PHACTR1,NTM,MEIS1,NEDD4L,KIT,EPHA7,SPAG9,WNT5A,CTNNB1,LMX1A,VWC2L,DIAPH3,EFNA5,NGEF,TRPC5,NEUROD2,PTPRF,ARHGEF28,GPRIN1,OPCML,GRID2,RB1,NTNG1,KIRREL3,GFRA1,ETV1,LRTM2,KAT2B,ANKS1A,CDK5RAP2,BDNF,SEMA3E,CDH2,USP9X,TENM1,RORB,CDK6,GABRB1,AVIL,CLASP2,ZNF521,SEMA6A,EIF4G1,ITGA4,ROCK1,DGKG,PRKG1,ELAVL4,MAGI2,NIN,PRKD1,HHIP,TMEM30A,SEMA5A,METTL3,PARD3,ATP8B1,RNF6,SEMA3C,VPS13B,SPART*
GO:0048666	neuron development	6.873001192631132e-8	*APBB2,MAP3K13,THRB,CHRNB2,SEZ6,AGTPBP1,CAMK2B,GRIP1,KALRN,NOTCH2,EPB41L3,MAP6,CDH11,SHANK3,STYXL1,PLXNA4,STK24,PRKN,SERPINI1,NCAM2,TIAM2,LRP8,CNTN1,NCK1,NREP,GABRA5,KCNQ3,PREX1,ZNF804A,BRAF,CHRNA7,HECW1,HMGB1,TGFB2,LAMA2,TRIO,MUSK,ALKBH1,EPHA5,SKOR2,RUNX1,ENAH,CHL1,PLS1,SLIT3,ROBO1,PAK3,NCKAP1,EPHA3,LYN,ADGRB3,KLF7,DIP2B,VSX1,ERCC6,EIF2AK4,MATN2,CRTAC1,CUX1,GPM6B,ADGRV1,WHRN,TDP2,APP,IGF2BP1,HCN1,FRY,PALLD,CECR2,PTK2,LRRK2,TIAM1,PHACTR1,NTM,NEDD4L,KIT,EPHA7,SPAG9,WNT5A,CTNNB1,LMX1A,EFNA5,NGEF,TRPC5,NEUROD2,PTPRF,ARHGEF28,GPRIN1,OPCML,GRID2,RB1,NTNG1,KIRREL3,GFRA1,ETV1,LRTM2,KAT2B,ANKS1A,BDNF,SEMA3E,CDH2,USP9X,TENM1,RORB,GABRB1,AVIL,CLASP2,SEMA6A,ITGA4,ROCK1,DGKG,PRKG1,ELAVL4,MAGI2,NIN,PRKD1,TMEM30A,SEMA5A,PARD3,ATP8B1,RNF6,SEMA3C,VPS13B,SPART*
GO:0030182	neuron differentia	7.937162335223619e-8	*APBB2,RTN1,NFIA,TRAPPC9,MAP3K13,THRB,CHRNB2,SEZ6,AGTPBP1,SPINK5,CAMK2B,GRIP1*

	tion		,*KALRN,USH2A,NOTCH2,SDK2,EPB41L3,MAP6,CDH11,SHANK3,STYXL1,PLXNA4,STK24,PRKN,NLGN4X,IFT172,CDON,SERPINI1,NCAM2,TIAM2,LRP8,CNTN1,EIF4ENIF1,NCK1,NREP,GABRA5,KCNQ3,PREX1,ZNF804A,BRAF,CHRNA7,HECW1,HMGB1,TGFB2,LAMA2,TRIO,MUSK,ALKBH1,EPHA5,SKOR2,RUNX1,ENAH,ALDH1A2,CHL1,PLS1,SLIT3,ROBO1,PAK3,BTG4,NCKAP1,EPHA3,LYN,ADGRB3,KLF7,DIP2B,VSX1,ERCC6,EIF2AK4,MATN2,CRTAC1,ZFHX3,CUX1,GPM6B,ADGRV1,WHRN,TDP2,APP,IGF2BP1,HCN1,FRY,PALLD,CECR2,PTK2,LRRK2,TCF4,TIAM1,PHACTR1,NTM,MEIS1,NEDD4L,KIT,EPHA7,SPAG9,WNT5A,CTNNB1,LMX1A,VWC2L,DIAPH3,EFNA5,NGEF,TRPC5,NEUROD2,PTPRF,ARHGEF28,GPRIN1,OPCML,GRID2,RB1,NTNG1,KIRREL3,GFRA1,ETV1,LRTM2,KAT2B,ANKS1A,CDK5RAP2,BDNF,SEMA3E,CDH2,USP9X,TENM1,RORB,GABRB1,AVIL,CLASP2,ZNF521,SEMA6A,EIF4G1,ITGA4,ROCK1,DGKG,PRKG1,ELAVL4,MAGI2,NIN,PRKD1,TMEM30A,SEMA5A,PARD3,ATP8B1,RNF6,SEMA3C,VPS13B,SPART*
GO:0009653	anatomical structure morphogenesis	8.940869382179129e-8	*APBB2,HS3ST3A1,EPN2,MAP3K3,NFIA,ISM1,MAP3K13,ARL13B,LIMD1,CHST11,THRB,SPTBN1,CDC42EP3,FGF1,CHRNB2,CDH8,ARHGAP15,AGTPBP1,FHL1,ATP10A,SH3PXD2A,SPINK5,CAMK2B,GRIP1,KALRN,NOTCH2,TMOD2,SDK2,NPNT,EPB41L3,MAP6,PARVB,CDH11,SHANK3,NR4A3,E2F7,ADAMTS12,PLXNA4,YBX1,CACNA1C,ASB4,PRKN,EXOC4,IFT172,CHD7,PTBP3,CDON,SLC17A6,THSD7A,TIAM2,CNMD,LRP8,CNTN1,MEOX2,ENPEP,RHOJ,FGF10,TSPAN18,CDH7,KDM2B,COL11A1,BRAF,COL1A2,TMOD3,COL12A1,ABLIM1,ALPK2,CHRNA7,HECW1,ZNF304,AFF3,RFLNA,ATRX,TGFB2,LAMA2,TRIO,HOXC13,SMARCC1,NDST1,MUSK,JAM3,EPHA5,SKOR2,RUNX1,ENAH,SNX10,ALDH1A2,FAM171A1,HUWE1,ASPH,SOX30,CHL1,PLS1,SLIT3,ROBO1,ANKRD11,ITPR1,CPM,PAK3,TNFRSF12A,NCKAP1,PRPF40A,FOSL2,EPHA3,LATS2,MYH14,ARHGAP18,MYOM2,ADGRB3,KLF7,DIP2B,LARGE1,PDZD8,NOS3,VSX1,EIF2AK4,MATN2,TGFBR2,CUX1,EPB41L5,WHRN,APP,GATA4,SMAD5,IGF2BP1,ADAM12,HCN1,FRY,PALLD,CECR2,RIPK4,PTK2,LRRK2,PKHD1,RARB,TIAM1,CSRP3,PHACTR1,ACVR2A,MEIS1,NEDD4L,KIT,EPHA7,SPAG9,WNT5A,CTNNB1,PRKDC,ESR1,PTPN14,SCFD1,ITGB8,POFUT2,BCAR3,LMX1A,WWOX,GREB1L,DOCK1,EFNA5,IGF2BP2,SYNE3,NGEF,TRPC5,TNFSF11,LYVE1,APOH,ARHGEF28,TASOR,ATF2,ACVR1,PID1,GRID2,RB1,NTNG1,PLPP3,HNF1B,KIRREL3,DLC1,ETV1,LRTM2,WLS,PRCP,MEF2D,FLVCR1,ARHGEF7,NPHP1,FBN2,BDNF,TFCP2L1,SEMA3E,ADAMTS9,STIM1,KIF16B,CDH2,PPARG,TANC1,USP9X,RORB,SH3KBP1,TTC39C,SIPA1L3,SFRP4,JCAD,VAV2,TFRC,FBN1,BMPER,CLASP2,ANGPT1,SEMA6A,ITGA4,ROCK1,PML,SP100,PRKG1,ELAVL4,CDH9,NIN,AIMP1,PRKD1,CALCRL,STK4,ANPEP,TTN,HHIP,EYA4,SEMA5A,METTL3,FMNL2,PARD3,ATP8B1,RNF6,SEMA3C,VPS13B,SPART*
GO:0048	positive	6.42806461	*APBB2,RGN,EPN2,ING3,MAP3K3,OSBPL8,LRRN*

518	regulation of biological process	8698034e-7	1,NFIA,PHF20,FOXK2,SMYD3,MAOA,MAP3K13,PPM1A,LIMD1,TF,THRB,SPTBN1,CDC42EP3,CACNB1,USP32,ATF7,FGF1,CHRNB2,CDC14C,AGTPBP1,FHL1,RBPMS,ATP10A,GPBP1L1,BRD4,DOK4,CAMK2B,GRIP1,DCT,SLC38A9,XPO4,KALRN,NOTCH2,TMOD2,HMBOX1,NPNT,MAP6,SHANK3,NR4A3,ARID1B,E2F7,FOXO1,STYXL1,STK39,SMURF2,DAAM2,FER1L6,PLXNA4,YBX1,STK24,DIPK2A,ASB4,IDO1,PRKN,KDM7A,FAM83B,IFT172,CHD7,GPSM1,CDON,SERPINI1,GNAI1,MAPKAPK3,TIAM2,LRP8,CNTN1,MEOX2,STAT5B,UBE2O,EIF4ENIF1,CALCR,ERBIN,MBTPS2,CD247,PTH,ADAR,TOX2,CLCN3,RHOJ,FGF10,MECOM,PLG,UBE2E2,NCK1,CD84,FAM168A,NONO,MTCP1,CHRM3,KDM2B,PREX1,RSF1,NCOA7,ZNF804A,CIDEB,BRAF,TMOD3,MRTFB,CHRNA7,HMGB1,ZNF304,HIVEP1,ATRX,TGFB2,MDFIC,LAMA2,ADD3,HOXC13,SMARCC1,RAD51B,NDST1,MUSK,JAM3,SFPQ,MAP3K5,NUP93,ZCCHC4,TBC1D5,CTIF,ATP11A,RBM23,SLC15A4,GOLPH3,PRKG2,SKOR2,RUNX1,ATP2C1,ALDH1A2,PTGFR,STRADA,HUWE1,ASPH,RRAS2,SH3BGRL,UBR5,GTF2F2,SOX30,GSN,PLS1,SPON1,MAPRE3,TLR8,CACUL1,ARHGEF39,ROBO1,ITPR1,PKP4,PAK3,ABI3BP,KLF12,DOK6,PRKAG2,MLLT3,CCNT2,HAVCR1,CNOT10,MR1,TNFRSF12A,OASL,CPT2,CXCR5,NCKAP1,FOSL2,EPHA3,LATS2,SPDYE2B,ERP29,UVRAG,LYN,ADGRB3,KLF7,SUMO2,MTA3,DIP2B,LARGE1,RRM2,LDB2,MYRFL,RNASEL,DBF4B,DCP2,TMED7-TICAM2,NOS3,TNRC6C,PRAMEF27,MLLT10,ERCC6,NOS1AP,EIF2AK4,MAGEL2,TGFBR2,MARK4,SYNJ1,TCF7L2,ZNF462,ZFHX3,CUX1,UNC119,GPM6B,RRAGD,ULK3,SPDYE2,GKAP1,GSDMD,ADGRV1,RASGRF2,EPB41L5,WAC,WHRN,DAPK1,SAR1B,APP,GATA4,SMAD5,IGF2BP1,GSKIP,ADAM12,PELI2,CACNA2D1,ELOVL5,PIK3R5,TLR1,BMT2,AVPR1B,PTK2,TICAM2,SCP2,SH2B2,CELF1,POGZ,PIP4K2B,LRRK2,INO80D,ZNF395,IKZF3,CLSTN2,ARIH1,PLD1,VTCN1,PKHD1,TENT5C,RARB,PJA2,TCF4,PAEP,COP1,TIAM1,CSRP3,MLIP,IQSEC2,ACVR2A,TIPIN,VIT,MEIS1,NEDD4L,BRCC3,RABGEF1,KIT,TBC1D8,EPHA7,SPAG9,WNT5A,TRABD2B,ARFGEF2,CTNNB1,ANKRD17,CTSK,PRKDC,IPO5,ESR1,TNFRSF19,ITGB8,ERC2,POFUT2,BCAR3,TPD52L1,PPM1E,ZXDC,FRMD4A,LMX1A,WWOX,NEK10,PTGER4,VWC2L,DOCK1,EFNA5,IGF2BP2,CDC14B,HECTD1,TRPC5,NEUROD2,FNIP2,JAML,TNFSF11,IKBKB,FXR2,AKAP6,USF3,LYVE1,APOH,RAB3GAP2,CCPG1,LINGO2,CEMIP,ATF2,ACVR1,HLA-DQA2,PID1,EBF2,GRID2,ZNF423,RB1,IFNLR1,PDE4B,ADIPOR1,ZNF143,PLPP3,HNF1B,ZNF615,LAMP2,NCOA2,BNIP3L,PRKCH,ACACB,DLC1,DENND2B,XRCC5,TBRG4,GFRA1,SHLD2,BMX,SLC24A3,TAF15,POU2F1,ETV1,MID2,LRTM2,SIK1,TP53BP2,ZNF850,KAT2B,GOLPH3L,WLS,SUPV3L1,MEF2D,GTF2A1,SPTLC1,CDK5RAP2,RFC3,ARHGEF7,ATF3,TRDN,NPHP1,FBN2,SRI,ATP1B1,ARNT,BDNF,TFCP2L1,SEMA3E,BCLAF3,SCIN,TENT4B,TLR6,ADAMTS9,STIM1,RAB11FIP2

			,CDH2,LAG3,PPARG,CHRNB4,USP9X,NEK7,TENM1,RORB,CDK6,SH3KBP1,NALCN,AVIL,DDX60,TFAP2D,SFRP4,SPDYE16,IL1RAP,JCAD,VAV2,TFRC,SPDYE6,RAB31,CLEC6A,ZNF292,RAPGEF4,BMPER,CLASP2,ZNF521,ANGPT1,SEMA6A,EIF4G1,ITGA4,ROCK1,NCAPH,TAX1BP1,NUP98,PML,PLEK2,TNFSF4,AFAP1L2,SP100,PRKG1,ELAVL4,MAGI2,NIN,SPG21,ANKFY1,ASIC2,AIMP1,SLC30A10,HSPBP1,PRKD1,CALCRL,SLC20A2,USP34,STK4,PRLR,NMI,IL16,CR2,LPAR6,CTBP2,TTN,DYRK1A,TMEM30A,MYRIP,KCNE2,CUL4A,EYA4,SEMA5A,ELL2,TTPA,PVT1,GATAD2B,METTL3,UAP1,PARD3,KCNN4,ZNF33A,NPAS3,BMAL2,RNF6,SEMA3C,VAV1,SMYD1,FANCB
GO:0048522	positive regulation of cellular process	7.466143717688033e-7	APBB2,RGN,EPN2,ING3,MAP3K3,OSBPL8,LRRN1,NFIA,PHF20,FOXK2,SMYD3,MAOA,MAP3K13,PPM1A,TF,THRB,SPTBN1,CDC42EP3,USP32,ATF7,FGF1,CHRNB2,CDC14C,AGTPBP1,RBPMS,ATP10A,GPBP1L1,BRD4,DOK4,CAMK2B,GRIP1,DCT,SLC38A9,XPO4,KALRN,NOTCH2,TMOD2,HMBOX1,NPNT,MAP6,SHANK3,NR4A3,ARID1B,E2F7,FOXO1,STYXL1,STK39,SMURF2,DAAM2,FER1L6,PLXNA4,YBX1,STK24,DIPK2A,ASB4,IDO1,PRKN,KDM7A,FAM83B,IFT172,CHD7,GPSM1,CDON,SERPINI1,GNAI1,TIAM2,LRP8,CNTN1,MEOX2,STAT5B,UBE2O,EIF4ENIF1,CALCR,MBTPS2,CD247,PTH,ADAR,TOX2,CLCN3,RHOJ,FGF10,MECOM,PLG,UBE2E2,NCK1,CD84,FAM168A,MTCP1,CHRM3,KDM2B,PREX1,RSF1,NCOA7,ZNF804A,CIDEB,BRAF,TMOD3,MRTFB,CHRNA7,HMGB1,ZNF304,HIVEP1,ATRX,TGFB2,MDFIC,LAMA2,ADD3,HOXC13,SMARCC1,RAD51B,NDST1,MUSK,JAM3,SFPQ,MAP3K5,NUP93,ZCCHC4,TBC1D5,CTIF,ATP11A,RBM23,SLC15A4,GOLPH3,PRKG2,SKOR2,RUNX1,ATP2C1,ALDH1A2,PTGFR,STRADA,HUWE1,ASPH,RRAS2,SH3BGRL,UBR5,GTF2F2,SOX30,GSN,PLS1,SPON1,MAPRE3,TLR8,CACUL1,ARHGEF39,ROBO1,ITPR1,PKP4,ABI3BP,KLF12,DOK6,PRKAG2,MLLT3,CCNT2,HAVCR1,CNOT10,MR1,TNFRSF12A,OASL,CPT2,CXCR5,NCKAP1,FOSL2,EPHA3,LATS2,SPDYE2B,ERP29,UVRAG,LYN,ADGRB3,KLF7,SUMO2,MTA3,DIP2B,LARGE1,RRM2,LDB2,MYRFL,RNASEL,DBF4B,DCP2,NOS3,TNRC6C,PRAMEF27,MLLT10,ERCC6,NOS1AP,EIF2AK4,MAGEL2,TGFBR2,MARK4,SYNJ1,TCF7L2,ZNF462,ZFHX3,CUX1,UNC119,RRAGD,ULK3,SPDYE2,GKAP1,GSDMD,ADGRV1,RASGRF2,EPB41L5,WAC,WHRN,DAPK1,SAR1B,APP,GATA4,SMAD5,IGF2BP1,GSKIP,PELI2,CACNA2D1,ELOVL5,PIK3R5,TLR1,BMT2,AVPR1B,PTK2,TICAM2,SCP2,CELF1,POGZ,PIP4K2B,LRRK2,INO80D,ZNF395,IKZF3,CLSTN2,ARIH1,PLD1,VTCN1,PKHD1,TENT5C,RARB,PJA2,TCF4,PAEP,COP1,TIAM1,CSRP3,MLIP,IQSEC2,ACVR2A,TIPIN,VIT,MEIS1,NEDD4L,BRCC3,KIT,TBC1D8,EPHA7,SPAG9,WNT5A,TRABD2B,ARFGEF2,CTNNB1,ANKRD17,CTSK,PRKDC,IPO5,ESR1,TNFRSF19,ITGB8,ERC2,POFUT2,BCAR3,TPD52L1,PPM1E,ZXDC,FRMD4A,LMX1A,WWOX,NEK10,PTGER4,VWC2L,DOCK1,EFNA5,IGF2BP2,CDC14B,HECTD1,TRPC5,NEUROD2,FNIP2,JAML,TNFSF11,IKBKB,FX

			R2,AKAP6,USF3,LYVE1,RAB3GAP2,CCPG1,LINGO2,CEMIP,ATF2,ACVR1,HLA-DQA2,PID1,EBF2,GRID2,ZNF423,RB1,IFNLR1,PDE4B,ADIPOR1,ZNF143,PLPP3,HNF1B,ZNF615,NCOA2,BNIP3L,PRKCH,DLC1,DENND2B,XRCC5,TBRG4,GFRA1,SHLD2,SLC24A3,TAF15,POU2F1,ETV1,MID2,LRTM2,SIK1,TP53BP2,ZNF850,KAT2B,GOLPH3L,WLS,SUPV3L1,MEF2D,GTF2A1,SPTLC1,CDK5RAP2,RFC3,ARHGEF7,ATF3,TRDN,NPHP1,FBN2,SRI,ATP1B1,ARNT,BDNF,TFCP2L1,SEMA3E,BCLAF3,SCIN,TENT4B,TLR6,ADAMTS9,STIM1,RAB11FIP2,CDH2,LAG3,PPARG,CHRNB4,USP9X,NEK7,TENM1,RORB,CDK6,SH3KBP1,NALCN,AVIL,DDX60,TFAP2D,SFRP4,SPDYE16,IL1RAP,JCAD,VAV2,TFRC,SPDYE6,RAB31,CLEC6A,ZNF292,RAPGEF4,BMPER,CLASP2,ZNF521,ANGPT1,SEMA6A,EIF4G1,ITGA4,ROCK1,NCAPH,NUP98,PML,PLEK2,TNFSF4,AFAP1L2,SP100,ELAVL4,MAGI2,NIN,ANKFY1,ASIC2,AIMP1,SLC30A10,HSPBP1,PRKD1,CALCRL,USP34,STK4,PRLR,NMI,IL16,LPAR6,CTBP2,TTN,DYRK1A,TMEM30A,MYRIP,KCNE2,CUL4A,EYA4,SEMA5A,ELL2,PVT1,GATAD2B,METTL3,UAP1,PARD3,KCNN4,ZNF33A,NPAS3,BMAL2,RNF6,SEMA3C,VAV1,SMYD1,FANCB
GO:0022008	neurogenesis	9.871855346902076e-7	*APBB2,RTN1,NFIA,TRAPPC9,MAP3K13,THRB,CHRNB2,SEZ6,AGTPBP1,SPINK5,CAMK2B,GRIP1,DCT,KALRN,USH2A,NOTCH2,SDK2,EPB41L3,MAP6,CDH11,SHANK3,STYXL1,DAAM2,PLXNA4,STK24,PRKN,NLGN4X,IFT172,CHD7,CDON,SERPINI1,NCAM2,TIAM2,TRAK2,LRP8,CNTN1,EIF4ENIF1,FGF10,NCK1,NREP,GABRA5,NEXMIF,KCNQ3,PREX1,ZNF804A,BRAF,CHRNA7,HECW1,HMGB1,TGFB2,LAMA2,TRIO,MUSK,ALKBH1,EPHA5,SKOR2,RUNX1,ENAH,ALDH1A2,RRAS2,CHL1,PLS1,SLIT3,ROBO1,PAK3,BTG4,NCKAP1,EPHA3,LYN,ADGRB3,KLF7,DIP2B,LARGE1,NAV1,VSX1,ERCC6,EIF2AK4,MATN2,CRTAC1,ZFHX3,CUX1,GPM6B,ADGRV1,WHRN,TDP2,PRUNE1,APP,IGF2BP1,HCN1,FRY,HOOK3,PALLD,CECR2,PTK2,LRRK2,RARB,TCF4,TIAM1,PHACTR1,NTM,MEIS1,NEDD4L,KIT,EPHA7,SPAG9,WNT5A,CTNNB1,LMX1A,VWC2L,DIAPH3,EFNA5,NGEF,TRPC5,NEUROD2,PTPRF,ARHGEF28,GPRIN1,OPCML,GRID2,RB1,NTNG1,PLPP3,KIRREL3,PRKCH,XRCC5,GFRA1,ETV1,LRTM2,KAT2B,ANKS1A,CDK5RAP2,BDNF,SEMA3E,CDH2,PPARG,USP9X,TENM1,RORB,CDK6,GABRB1,AVIL,CLASP2,ZNF521,SEMA6A,EIF4G1,ITGA4,ROCK1,DGKG,PRKG1,ELAVL4,MAGI2,NIN,PRKD1,HHIP,TMEM30A,SEMA5A,METTL3,PARD3,ATP8B1,RNF6,SEMA3C,VPS13B,SPART*
GO:0031175	neuron projection development	0.000001095181086302314	*APBB2,MAP3K13,CHRNB2,SEZ6,CAMK2B,GRIP1,KALRN,NOTCH2,EPB41L3,MAP6,CDH11,SHANK3,STYXL1,PLXNA4,STK24,PRKN,SERPINI1,NCAM2,TIAM2,LRP8,CNTN1,NCK1,NREP,PREX1,ZNF804A,BRAF,CHRNA7,HECW1,HMGB1,LAMA2,TRIO,MUSK,ALKBH1,EPHA5,SKOR2,ENAH,CHL1,PLS1,SLIT3,ROBO1,PAK3,NCKAP1,EPHA3,LYN,ADGRB3,KLF7,DIP2B,ERCC6,EIF2AK4,MATN2,CRTAC1,CUX1,GPM6B,ADGRV1,WHRN,APP,IGF*

			2BP1,FRY,PALLD,CECR2,PTK2,LRRK2,TIAM1,PHACTR1,NEDD4L,KIT,EPHA7,SPAG9,WNT5A,CTNNB1,LMX1A,EFNA5,NGEF,TRPC5,NEUROD2,PTPRF,ARHGEF28,GPRIN1,GRID2,RB1,NTNG1,KIRREL3,GFRA1,ETV1,LRTM2,KAT2B,BDNF,SEMA3E,CDH2,USP9X,AVIL,CLASP2,SEMA6A,ITGA4,ROCK1,DGKG,PRKG1,ELAVL4,MAGI2,NIN,PRKD1,TMEM30A,SEMA5A,PARD3,RNF6,SEMA3C,VPS13B,SPART
GO:0007167	enzyme-linked receptor protein signaling pathway	0.000001801481820783 8214	FSTL1,EPN2,OSBPL8,NFIA,PPM1A,CHST11,SPTBN1,FGF1,RBPMS,DOK4,KALRN,NOTCH2,NPNT,NR4A3,FOXO1,SMURF2,ADAMTS12,FAM83B,NLK,MAPKAPK3,STAT5B,UBE2O,ERBIN,FGF10,COL4A6,NCK1,NREP,BRAF,COL1A2,HIVEP1,TGFB2,TRIO,SMARCC1,NDST1,MUSK,NUP93,EPHA5,SKOR2,ANKS1B,ROBO1,PAK3,DOK6,EPHA3,LATS2,LYN,PPM1L,TGFBR2,SHISA2,GKAP1,GATA4,SMAD5,PTK2,SH2B2,PIP4K2B,TIAM1,CSRP3,ACVR2A,RABGEF1,KIT,EPHA7,WNT5A,CTNNB1,ITGB8,BCAR3,VWC2L,EFNA5,NGEF,PTPRF,ARHGEF28,ATF2,ACVR1,PID1,ZNF423,ADIPOR1,GFRA1,ANKS1A,ARHGEF7,SAMD12,FBN2,ARNT,BDNF,LDLRAD4,KIF16B,PPARG,USP9X,SFRP4,JCAD,VAV2,FBN1,BMPER,ANGPT1,SEMA6A,PML,AFAP1L2,MAGI2,SLC30A10,PRKD1,PRLR,PTPRA,HHIP,PMEPA1,VAV1,SPART
GO:0022603	regulation of anatomical structure morphogenesis	0.000001998827963854 2638	EPN2,ISM1,MAP3K13,LIMD1,CDC42EP3,FGF1,CHRNB2,ARHGAP15,ATP10A,SPINK5,CAMK2B,GRIP1,KALRN,EPB41L3,MAP6,PARVB,SHANK3,ADAMTS12,PLXNA4,PRKN,TIAM2,CNMD,LRP8,RHOJ,FGF10,TSPAN18,BRAF,CHRNA7,HECW1,ZNF304,TGFB2,SKOR2,RUNX1,FAM171A1,HUWE1,ROBO1,PAK3,PRPF40A,MYH14,ARHGAP18,ADGRB3,DIP2B,PDZD8,NOS3,TGFBR2,CUX1,GATA4,ADAM12,PTK2,LRRK2,PKHD1,TIAM1,NEDD4L,KIT,EPHA7,SPAG9,WNT5A,CTNNB1,PRKDC,ESR1,ITGB8,EFNA5,SYNE3,NGEF,TRPC5,APOH,ATF2,PID1,NTNG1,DLC1,ARHGEF7,BDNF,SEMA3E,ADAMTS9,STIM1,CDH2,PPARG,SH3KBP1,JCAD,TFRC,BMPER,CLASP2,SEMA6A,ROCK1,PML,SP100,NIN,PRKD1,SEMA5A,FMNL2,RNF6,SEMA3C,SPART
GO:0016310	phosphorylation	0.000002524458442321 736	PFKP,STYK1,RGN,CDK14,MAP3K3,SMYD3,ABR,MAP3K13,SAMSN1,LIMD1,TF,MAPK10,FGF1,CAMK2B,TLK1,KALRN,MAP4K3,FPGT-TNNI3K,STK39,CDK18,STK24,DIPK2A,FAM20B,PRKN,PIK3C2B,CDON,NLK,MAPKAPK3,LRP8,ADAR,CDC37,FGF10,STK32B,HERC5,NCK1,MTCP1,BRAF,ALPK2,CHRNA7,TGFB2,TRIO,MUSK,MAP3K5,PRKAR2A,PRKG2,PIK3C3,RSKR,EPHA5,STRADA,MAPRE3,TLR8,CACUL1,ROBO1,PAK3,IPMK,CAMK1G,PRKAG2,CCNT2,CILK1,EPHA3,LATS2,SPDYE2B,ERP29,UVRAG,LYN,NME7,LDB2,RNASEL,SGMS2,ERCC6,EIF2AK4,TGFBR2,MARK4,UNC119,ULK3,SPDYE2,GKAP1,DAPK1,APP,GSKIP,PELI2,PI4KB,PIK3R5,RIPK4,PTK2,PIP4K2B,LRRK2,ACVR2A,RABGEF1,KIT,EPHA7,SPAG9,WNT5A,PRKDC,IPO5,BCAR3,TPD52L1,PPM1E,HUNK,NEK10,PKIG,EFNA5,TRPC5,FNIP2,TNFSF11,IKBKB,FXR2,CEMIP,ATF2,ACVR1,PID1,RB1,PLPP3,AK5,PRKCH,XRCC5,GFRA1,BMX,N

			RK,SIK1,BDNF,TLR6,PPARG,NEK7,TENM1,CDK6,SH3KBP1,MYLK4,SPDYE16,TFRC,SPDYE6,MAP3K1,CLEC6A,ANGPT1,EIF4G1,ROCK1,DGKG,PRKG1,MAGI2,GALK2,TPK1,PRKD1,STK4,TNNI3K,PRLR,TTN,DYRK1A,ETNK1,PARD3
GO:0006796	phosphate-containing compound metabolic process	0.000003708605155160 3926	PFKP,ABCC6,STYK1,RGN,CDK14,MAP3K3,OSBPL8,FOXK2,SMYD3,GARS1,ABR,MAP3K13,PPM1A,SAMSN1,LIMD1,TF,MAPK10,DAPP1,FGF1,CDC14C,PIGL,PPM1H,CAMK2B,TLK1,PGM1,KALRN,MAP4K3,FPGT-TNNI3K,SLC44A5,STYXL1,STK39,CDK18,STK24,DIPK2A,FAM20B,IDO1,PRKN,PNPLA3,PIK3C2B,SLC44A3,CDON,NLK,MAPKAPK3,LRP8,NUDT19,PTH,ADAR,PDE10A,PDHX,CDC37,FGF10,STK32B,HERC5,NUDT13,NCK1,MTCP1,BRAF,ALPK2,CHRNA7,TGFB2,TRIO,MUSK,MAP3K5,PRKAR2A,PRKG2,PIK3C3,PIGV,RSKR,EPHA5,PTPN20,PPTC7,STRADA,MAPRE3,TLR8,CACUL1,ROBO1,PAK3,IPMK,CAMK1G,PRKAG2,CCNT2,GDPD1,CILK1,EPHA3,LATS2,SPDYE2B,ERP29,UVRAG,PTPRN2,LYN,NME7,FIGNL1,RRM2,LDB2,NMNAT3,FMO5,RNASEL,PPM1L,SGMS2,NOS3,ERCC6,EIF2AK4,TGFBR2,MARK4,SYNJ1,UNC119,ULK3,SPDYE2,GKAP1,DAPK1,ACSM2B,TTC7B,APP,GSKIP,PELI2,CHRM5,PI4KB,ELOVL5,PIK3R5,RIPK4,AVPR1B,PTK2,SCP2,HYCC1,PIP4K2B,LRRK2,PLD1,ACVR2A,DUSP14,RABGEF1,KIT,EPHA7,SPAG9,WNT5A,PRKDC,IPO5,PTPN14,BCAR3,TPD52L1,PPM1E,TPST2,HUNK,NEK10,PDE7B,BPGM,PKIG,EFNA5,CDC14B,TRPC5,FNIP2,AASS,UGGT2,TNFSF11,PTPRF,IKBKB,FXR2,CEMIP,ATF2,ACVR1,PID1,RB1,PDE4B,SMG7,PLPP3,AK5,CPS1,PRKCH,ACACB,AGPAT5,XRCC5,GFRA1,INPP4A,BMX,TPST1,NRK,SIK1,PIPOX,KAT2B,SPTLC1,ATP1B1,ARNT,BDNF,TLR6,PPARG,NEK7,TENM1,CDK6,SH3KBP1,LPCAT2,MYLK4,SPDYE16,TFRC,PNPLA8,SPDYE6,MAP3K1,CLEC6A,ANGPT1,EIF4G1,ACSM5,DNAJC6,ROCK1,DGKG,PRKG1,MAGI2,GALK2,TPK1,PRKD1,STK4,TNNI3K,PRLR,PTPRA,TTN,DYRK1A,SERINC1,ETNK1,UAP1,PARD3
GO:0006793	phosphorus metabolic process	0.000004107304519777 251	PFKP,ABCC6,STYK1,RGN,CDK14,MAP3K3,OSBPL8,FOXK2,SMYD3,GARS1,ABR,MAP3K13,PPM1A,SAMSN1,LIMD1,TF,MAPK10,DAPP1,FGF1,CDC14C,PIGL,PPM1H,CAMK2B,TLK1,PGM1,KALRN,MAP4K3,FPGT-TNNI3K,SLC44A5,STYXL1,STK39,CDK18,STK24,DIPK2A,FAM20B,IDO1,PRKN,PNPLA3,PIK3C2B,SLC44A3,CDON,NLK,MAPKAPK3,LRP8,NUDT19,PTH,ADAR,PDE10A,PDHX,CDC37,FGF10,STK32B,HERC5,NUDT13,NCK1,MTCP1,BRAF,ALPK2,CHRNA7,TGFB2,TRIO,MUSK,MAP3K5,PRKAR2A,PRKG2,PIK3C3,PIGV,RSKR,EPHA5,PTPN20,PPTC7,STRADA,MAPRE3,TLR8,CACUL1,ROBO1,PAK3,IPMK,CAMK1G,PRKAG2,CCNT2,GDPD1,CILK1,EPHA3,LATS2,SPDYE2B,ERP29,UVRAG,PTPRN2,LYN,NME7,FIGNL1,RRM2,LDB2,NMNAT3,FMO5,RNASEL,PPM1L,SGMS2,NOS3,ERCC6,EIF2AK4,TGFBR2,MARK4,SYNJ1,UNC119,ULK3,SPDYE2,GKAP1,DAPK1,ACSM2B,TTC7B,APP,GSKIP,PELI2,CHRM5,PI4KB,ELOVL5,PIK3R5,RIPK

			4,AVPR1B,PTK2,SCP2,HYCC1,PIP4K2B,LRRK2,PLD1,ACVR2A,DUSP14,RABGEF1,KIT,EPHA7,SPAG9,WNT5A,PRKDC,IPO5,PTPN14,BCAR3,TPD52L1,PPM1E,TPST2,HUNK,NEK10,PDE7B,BPGM,PKIG,EFNA5,CDC14B,TRPC5,FNIP2,AASS,UGGT2,TNFSF11,PTPRF,IKBKB,FXR2,CEMIP,ATF2,ACVR1,PID1,RB1,PDE4B,SMG7,PLPP3,AK5,CPS1,PRKCH,ACACB,AGPAT5,XRCC5,GFRA1,INPP4A,BMX,TPST1,NRK,SIK1,PIPOX,KAT2B,SPTLC1,ATP1B1,ARNT,BDNF,TLR6,PPARG,NEK7,TENM1,CDK6,SH3KBP1,LPCAT2,MYLK4,SPDYE16,TFRC,PNPLA8,SPDYE6,MAP3K1,CLEC6A,ANGPT1,EIF4G1,ACSM5,DNAJC6,ROCK1,DGKG,PRKG1,MAGI2,GALK2,TPK1,PRKD1,STK4,TNNI3K,PRLR,PTPRA,TTN,DYRK1A,SERINC1,ETNK1,UAP1,PARD3
GO:0032501	multicellular organismal process	0.000006279839767915796	*FSTL1,APBB2,ABCC6,RTN1,HS3ST3A1,RGN,EPN2,MAP3K3,LRRN1,NFIA,ARHGEF9,TAFA2,ISM1,TRAPPC9,MAP3K13,ARL13B,SAMSN1,LIMD1,TF,CHST11,THRB,RCSD1,SPTBN1,CACNB1,FGF1,CHRNB2,SEZ6,GNB4,AGTPBP1,SH3PXD2A,SPINK5,OPA3,SKAP2,BRD4,DOK4,CAMK2B,GRIP1,DCT,ABCG8,KALRN,USH2A,NOTCH2,CD93,TMOD2,SDK2,NPNT,EPB41L3,MAP6,MORC1,CDH11,SHANK3,NR4A3,ARID1B,E2F7,FOXO1,STYXL1,STK39,SMURF2,ADAMTS12,DAAM2,PLXNA4,YBX1,STK24,DIPK2A,CACNA1C,ASB4,IDO1,PRKN,NLGN4X,EXOC4,F13A1,KDM7A,SLC5A7,IFT172,CHD7,GPSM1,PTBP3,CDON,SERPINI1,SLC17A6,THSD7A,NCAM2,TIAM2,CFAP54,TRAK2,CNMD,LRP8,TAGLN3,CNTN1,MEOX2,STAT5B,ENPEP,EIF4ENIF1,CALCR,CHRFAM7A,ERBIN,MBTPS2,CD247,MCC,PTH,ADAR,CLCN3,RHOJ,FGF10,TSPAN18,PLG,ILDR2,NCK1,CD84,NREP,EXO1,GABRA5,NEXMIF,KCNQ3,CHRM3,KDM2B,PREX1,COL11A1,ZNF804A,CIDEB,MCPH1,BRAF,COL1A2,DTNA,TMOD3,MRTFB,COL12A1,ABLIM1,ALPK2,CHRNA7,HECW1,HMGB1,ZNF304,AFF3,RFLNA,ATRX,TGFB2,LAMA2,ADD3,TRIO,HOXC13,SMARCC1,RAD51B,NDST1,MUSK,JAM3,ALKBH1,OR5AU1,ATP11A,SLC15A4,PCDHB8,PRKG2,SND1,HMCN1,IGFBP7,EPHA5,SKOR2,RUNX1,ENAH,SNX10,ALDH1A2,PTGFR,PCLO,ASPH,RRAS2,SIM1,KIAA1217,CHL1,GSN,PLS1,TLR8,SLIT3,NLRP7,ROBO1,ANKRD11,ITPR1,DNAAF11,PAK3,ABI3BP,MLLT3,BTG4,HAVCR1,MR1,F8,TNFRSF12A,CPT2,TAS2R14,CXCR5,NCKAP1,CLDN12,FOSL2,IL1RAPL2,EPHA3,LATS2,MYH14,MYOM2,LYN,ADGRB3,KLF7,GRIK1,DIP2B,SEC24C,LARGE1,FIGNL1,CA10,RRM2,CRYBB1,LDB2,ALDH1A1,NAV1,CORIN,PCDHB16,SGMS2,NOS3,VSX1,ERCC6,NOS1AP,EIF2AK4,IMPG1,MATN2,TGFBR2,ITGA11,MARK4,SYNJ1,TCF7L2,ZNF800,CRTAC1,ZFHX3,CUX1,UNC119,GPM6B,ULK3,CACNA2D4,LHFPL3,GSDMD,ADGRV1,EPB41L5,GAD1,WHRN,TDP2,PRUNE1,INSYN2A,APP,GATA4,SMAD5,IGF2BP1,MAMLD1,RNLS,RNF38,ADAM12,CACNA2D1,DHX35,HCN1,CHRM5,FRY,HOOK3,PALLD,TLR1,CECR2,TAS2R30,AVPR1B,PTK2,TICAM2,SH2B2,CELF1,HYCC1,LRRK2,INO80D,IKZF3,CLSTN2,ITFG2,VTCN1,PKHD1,TENT5C,RARB,PJA2,T*

			CF4,PAEP,TIAM1,CSRP3,PHACTR1,MLIP,ACVR2A,GNAO1,VIT,SNTG2,NTM,MEIS1,NEDD4L,RABGEF1,KIT,TBC1D8,EPHA7,SPAG9,ANTXR1,WNT5A,ARFGEF2,CTNNB1,ANKRD17,COL19A1,CTSK,PRKDC,ESR1,TNFRSF19,PTPN14,ITGB8,POFUT2,BCAR3,LMX1A,WWOX,NEK10,PTGER4,GREB1L,BBX,VWC2L,DOCK1,DIAPH3,GPC4,BPGM,EFNA5,IGF2BP2,OR1M1,HECTD1,NGEF,TRPC5,NEUROD2,JAML,KAT14,TNFSF11,PTPRF,IKBKB,FXR2,AKAP6,USF3,APOH,ARHGEF28,OR8B8,CLCN1,TASOR,GPRIN1,POU6F2,LINGO2,OPCML,CEMIP,ATF2,KCNJ12,CHRNB3,ACVR1,HLA-DQA2,EBF2,GRID2,ZNF423,KCNJ3,RB1,MPV17,NTNG1,PDE4B,PKD1L3,ADIPOR1,GABRB3,SHROOM4,PLPP3,HNF1B,IMMP2L,SSPN,KIRREL3,GABRG2,F5,CPS1,NCOA2,TMEM120A,PRKCH,ACACB,DLC1,XRCC5,GFRA1,SHLD2,KCND2,SLC24A3,RAB18,ETV1,TEX11,LRTM2,SIK1,KAT2B,WLS,ANKS1A,RYR3,PRCP,MEF2D,MYL4,CDK5RAP2,FLVCR1,ARHGEF7,DACH1,TRDN,NPHP1,FBN2,IKZF1,SRI,ATP1B1,ARNT,BDNF,LDLRAD4,TFCP2L1,SEMA3E,SCIN,MACO1,TLR6,ADAMTS9,STIM1,TMEM161B,KIF16B,CDH2,LAG3,PPARG,TANC1,PAPPA,ASB3,MTTP,CHRNB4,USP9X,TENM1,IL13RA2,RORB,CDK6,GABRB1,MYOF,LTB4R2,JAKMIP1,SH3KBP1,AVIL,AMY2A,TFAP2D,TTC39C,SIPA1L3,SLC6A13,WDFY4,SFRP4,BLTP1,IL1RAP,JCAD,VAV2,TFRC,VPS13A,FBN1,IQCB1,CLEC6A,BMPER,FUT10,CLASP2,CLTC,ZNF521,ANGPT1,SEMA6A,EIF4G1,ATP6V0A4,PHLPP1,ITGA4,ROCK1,CLEC3A,PML,AFF2,MGLL,TNFSF4,AFAP1L2,DGKG,SP100,PRKG1,ELAVL4,SBF2,CDH9,UMODL1,MAGI2,NIN,ASIC2,FNDC3A,AIMP1,PRKD1,CALCRL,SLC20A2,STK4,PCDH10,TNNI3K,ANPEP,PRLR,NMI,EYS,IL16,CR2,LPAR6,TTN,NCALD,HHIP,DYRK1A,TMEM30A,KCNE2,CUL4A,EYA4,SEMA5A,LAMA4,TTPA,METTL3,KRT85,UAP1,PARD3,KCNN4,ATP8B1,RNF6,SEMA3C,VAV1,SMYD1,VPS13B,SPART
GO:0007154	cell communication	0.000006532213411200699	*FSTL1,APBB2,RGN,CDK14,EPN2,PDE1C,NOSTRIN,MAP3K3,OSBPL8,NFIA,RAPGEF6,ARHGEF9,TAFA2,ABR,MAOA,MAP3K13,PPM1A,ARL13B,LIMD1,TF,CHST11,THRB,SPTBN1,CDC42EP3,MAPK10,CACNB1,USP32,DAPP1,GNG12,FGF1,CHRNB2,SEZ6,CDH8,ARHGAP15,GNB4,RBPMS,SKAP2,BRD4,DOK4,CAMK2B,GRIP1,ARHGAP11A-SCG5,TLK1,DLGAP1,SLC38A9,HBP1,ABCG8,KALRN,MAP4K3,NOTCH2,TMOD2,NPNT,CDH11,SHANK3,NR4A3,E2F7,FOXO1,STYXL1,STK39,SMURF2,ADAMTS12,DAAM2,PLXNA4,URB2,STK24,DIPK2A,DCDC1,CACNA1C,FAM3C,ASB4,PRKN,NLGN4X,EXOC4,PIK3C2B,SLC5A7,FAM83B,IFT172,CHD7,CDON,SLC17A6,NLK,GNAI1,MAPKAPK3,TIAM2,LRP8,CNTN1,STAT5B,ENPEP,ARHGEF4,UBE2O,DDIAS,CPEB4,CALCR,CHRFAM7A,ELMO1,ERBIN,MBTPS2,RGS22,CD247,MCC,PTH,ADAR,PDE10A,CLCN3,RHOJ,CDC37,FGF10,ZFYVE16,MECOM,COL4A6,STK32B,PLG,HERC5,ILDR2,NCK1,NONO,NREP,GABRA5,KCNQ3,MTCP1,CHRM3,PREX1,CIDEB,BRAF,COL1A2,DTNA,ALPK2,CHRNA7,HECW1,HMGB1,ZNF304,CDIP1,HIVEP1,A*

			TRX,TGFB2,MDFIC,LAMA2,TRIO,SMARCC1,NDST1,MUSK,JAM3,KCNIP3,SFPQ,MAP3K5,RGS3,NUP93,RERG,OR5AU1,PRKAR2A,SLC15A4,GOLPH3,PRKG2,MBTPS1,PIK3C3,USP10,IGFBP7,EPHA5,SKOR2,ENSG00000289694,ATP2C1,ALDH1A2,PTGFR,PCLO,ASPH,RRAS2,UBR5,SOX30,ANKS1B,CHL1,TCP11L1,TLR8,SLIT3,ROBO1,ITPR1,PKP4,PAK3,DOK6,PRKAG2,MLLT3,TNFRSF12A,OASL,TAS2R14,CXCR5,NCKAP1,CILK1,FOSL2,IL1RAPL2,ADGRD1,EPHA3,LATS2,MYH14,ERP29,ARHGAP18,PTPRN2,LYN,ADGRB3,KLF7,GRIK1,LARGE1,FIGNL1,PCDHB16,PPM1L,TMED7-TICAM2,NOS3,ERCC6,NOS1AP,EIF2AK4,RAB12,TGFBR2,CMKLR2,ITGA11,MARK4,SYNJ1,TCF7L2,UNC119,SHISA2,RRAGD,ULK3,GPR156,GKAP1,PPP2R5E,ADGRV1,RASGRF2,GAD1,SCG5,WAC,DAPK1,TDP2,INSYN2A,APP,GATA4,SMAD5,GSKIP,ADAM12,PELI2,CACNA2D1,HCN1,CHRM5,PI4KB,RHOT1,PIK3R5,TLR1,TAS2R30,BMT2,AVPR1B,PTK2,USP7,TICAM2,SH2B2,PIP4K2B,LRRK2,CLSTN2,ARIH1,PLD1,ITFG2,VTCN1,PKHD1,RARB,PJA2,TIAM1,CSRP3,IQSEC2,ACVR2A,GNAO1,TIPIN,RASSF4,BRCC3,SNX13,RABGEF1,CYB5R4,KIT,EPHA7,SPAG9,SPDL1,WNT5A,TRABD2B,ARFGEF2,TEX2,CTNNB1,ANKRD17,CTSK,PRKDC,IPO5,ESR1,TNFRSF19,ITGB8,ERC2,BCAR3,TPD52L1,WWOX,HUNK,NEK10,PTGER4,PDE7B,VWC2L,DOCK1,DIAPH3,GPC4,EFNA5,CDC14B,PLCH1,OR1M1,NGEF,NEUROD2,FNIP2,TNFSF11,PTPRF,IKBKB,SH3BP5,AKAP6,TRBV10-2,ARHGEF28,OR8B8,CLCN1,ATF2,CHRNB3,ACVR1,PID1,GRID2,ZNF423,KCNJ3,RB1,IFNLR1,NTNG1,PDE4B,ADIPOR1,GABRB3,PLPP3,HNF1B,LAMP2,GABRG2,NCOA2,BNIP3L,PRKCH,DLC1,DENND2B,RGS17,GFRA1,KCND2,INPP4A,BMX,GRIA4,RAB18,RGS7BP,MGRN1,MID2,SIK1,TP53BP2,WLS,ANKS1A,RYR3,PRCP,CDK5RAP2,ARHGEF7,SAMD12,ATF3,TRDN,NPHP1,FBN2,SNTG1,SRI,ATP1B1,ARNT,BDNF,LDLRAD4,SEMA3E,MACO1,TLR6,TMEM237,KIF16B,RAB11FIP2,CDH2,LAG3,PPARG,PAPPA,ASB3,CHRNB4,USP9X,NEK7,TENM1,IL13RA2,RORB,CDK6,GABRB1,MYOF,LTB4R2,SH3KBP1,NALCN,DDX60,MYO5A,SIPA1L3,LRFN2,SFRP4,IL1RAP,JCAD,VAV2,TFRC,GRIA3,PNPLA8,FBN1,HAPSTR1,MAP3K1,CLEC6A,RAPGEF4,BMPER,ANGPT1,SEMA6A,VIPR2,PHLPP1,ITGA4,ROCK1,TAX1BP1,APIP,PML,MGLL,PLEK2,TNFSF4,AFAP1L2,DGKG,SP100,PRKG1,NDUFAF2,MAGI2,SPG21,ASIC2,ENSA,AIMP1,SLC30A10,PRKD1,CALCRL,USP34,STK4,TNNI3K,SPC25,PRLR,PTPRA,PDE11A,NMI,RAB6C,IL16,EDARADD,GNA14,CR2,LPAR6,CTBP2,TTN,NCALD,HHIP,DYRK1A,PPFIA1,MYRIP,PPP1R1C,UNC13C,CUL4A,KCTD16,EYA4,DLGAP2,SEMA5A,PMEPA1,PEX5L,METTL3,KCNN4,RNF6,SEMA3C,VAV1,GRIA2,VDAC2,SPART
GO:0051649	establishment of localization in cell	0.000011561478426935274	*MREG,STX8,BLTP3B,TRAPPC9,PPM1A,RCSD1,MYO5C,AGTPBP1,ATP7B,GRIP1,TLK1,RGPD2,XPO4,USH2A,MAP6,NR4A3,PRKN,NLGN4X,EXOC4,XPO6,IFT172,GPSM1,SLC17A6,CFAP54,TRAK2,UBE2O,EIF4ENIF1,ADAR,CLCN3,FGF10,ZFYV*

			E16,TSPAN18,CD84,KCNQ3,RGPD3,ANO3,CIDEB,MCPH1,MYO1D,BRAF,XPO7,DYNLT2B,MDFIC,NUP93,RBM33,TBC1D5,PIK3C3,EPHA5,SNX10,TMEM230,STON2,STRADA,PCLO,ASPH,UBR5,ANO2,HPS3,DNAAF11,TRAPPC10,CPT2,VPS8,CILK1,ERP29,PTPRN2,LYN,KLF7,SEC24C,LARGE1,ATP6V1H,NUP58,SIL1,TMCC1,NOS3,MAGEL2,MDN1,SYNJ1,GPM6B,RGPD1,KPNB1,SCG5,WHRN,SAR1B,APP,RHOT1,HERC2,HOOK3,USE1,LMAN1L,NOP9,USP7,SCP2,LRRK2,PLD1,PKHD1,TIAM1,ENSG00000288683,SNX13,RABGEF1,KIT,SPAG9,SPDL1,PEX26,CTNNB1,IPO5,PTPN14,SCFD1,ERC2,VPS53,DIAPH3,PKIG,EFNA5,SYNE3,RAB3GAP2,XIST,ATF2,CCDC91,RB1,KIF13A,SMG7,LAMP2,RAB18,MGRN1,KAT2B,WLS,MYO1B,CDK5RAP2,TRDN,SRI,ATP1B1,KIF16B,RAB11FIP2,CDH2,MTTP,CHRNB4,USP9X,TENM1,IL13RA2,MYOF,KIF2C,MYO5A,SLC6A13,SPTBN5,BLTP1,VPS13A,RAB31,RAPGEF4,CLASP2,CLTC,ANGPT1,ATP6V0A4,ITGA4,DNAJC6,ROCK1,NUP98,PML,SCFD2,SP100,NDUFAF2,ANKFY1,ASIC2,AP4S1,PRKD1,STK4,RAB6D,SPC25,SCAMP1,RAB6C,CTBP2,TMEM30A,MYRIP,UNC13C,PEX5L,PARD3,KCNN4,VPS13B
GO:0141124	intracellular signaling cassette	0.000013537851596050894	*RGN,MAP3K3,OSBPL8,RAPGEF6,ARHGEF9,ABR,MAP3K13,PPM1A,TF,SPTBN1,CDC42EP3,MAPK10,FGF1,ARHGAP15,RBPMS,BRD4,DOK4,KALRN,MAP4K3,NOTCH2,NPNT,SHANK3,FOXO1,STK39,DIPK2A,CACNA1C,PRKN,PIK3C2B,CDON,NLK,GNAI1,MAPKAPK3,TIAM2,ARHGEF4,CALCR,ELMO1,PTH,PDE10A,RHOJ,FGF10,MECOM,HERC5,NCK1,CHRM3,PREX1,BRAF,COL1A2,CHRNA7,HMGB1,TGFB2,MDFIC,TRIO,NDST1,MUSK,MAP3K5,NUP93,RERG,USP10,EPHA5,ENSG00000289694,ATP2C1,ASPH,RRAS2,TLR8,ROBO1,ITPR1,PAK3,DOK6,CXCR5,NCKAP1,ERP29,ARHGAP18,LYN,PPM1L,NOS3,ERCC6,NOS1AP,EIF2AK4,RAB12,TGFBR2,TCF7L2,RASGRF2,APP,SMAD5,PELI2,RHOT1,PIK3R5,AVPR1B,PTK2,TICAM2,SH2B2,LRRK2,ARIH1,PLD1,PKHD1,PJA2,TIAM1,IQSEC2,ACVR2A,RABGEF1,KIT,EPHA7,SPAG9,WNT5A,ARFGEF2,CTNNB1,ANKRD17,ESR1,TNFRSF19,BCAR3,TPD52L1,NEK10,PTGER4,PDE7B,DOCK1,NGEF,NEUROD2,TNFSF11,IKBKB,AKAP6,ARHGEF28,ATF2,ACVR1,RB1,ADIPOR1,DLC1,DENND2B,RAB18,MGRN1,MID2,WLS,RYR3,ARHGEF7,ATF3,ATP1B1,LDLRAD4,SEMA3E,TLR6,CDH2,PPARG,TENM1,SIPA1L3,JCAD,VAV2,TFRC,MAP3K1,CLEC6A,RAPGEF4,BMPER,ANGPT1,SEMA6A,PHLPP1,ROCK1,APIP,PML,PRKG1,MAGI2,SLC30A10,PRKD1,STK4,PDE11A,NMI,RAB6C,LPAR6,NCALD,SEMA5A,PMEPA1,PEX5L,VAV1*
GO:0006468	protein phosphorylation	0.000013982933128817018	*STYK1,RGN,CDK14,MAP3K3,SMYD3,ABR,MAP3K13,SAMSN1,MAPK10,FGF1,CAMK2B,TLK1,KALRN,MAP4K3,FPGT-TNNI3K,STK39,CDK18,STK24,DIPK2A,PRKN,CDON,NLK,MAPKAPK3,LRP8,ADAR,CDC37,FGF10,STK32B,HERC5,NCK1,MTCP1,BRAF,ALPK2,CHRNA7,TGFB2,TRIO,MUSK,MAP3K5,PRKAR2A,PRKG2,RSKR,EPHA5,STRADA,MAPRE3,TLR8,CACUL1,ROBO1,PAK3,CAMK1G,PRKAG2,CCNT2,CILK*

			1,EPHA3,LATS2,SPDYE2B,ERP29,UVRAG,LYN,RNASEL,ERCC6,EIF2AK4,TGFBR2,MARK4,UNC119,ULK3,SPDYE2,DAPK1,APP,GSKIP,PELI2,PIK3R5,RIPK4,PTK2,LRRK2,ACVR2A,RABGEF1,KIT,EPHA7,SPAG9,WNT5A,PRKDC,IPO5,BCAR3,TPD52L1,PPM1E,HUNK,NEK10,PKIG,EFNA5,TRPC5,FNIP2,TNFSF11,IKBKB,FXR2,CEMIP,ATF2,ACVR1,PID1,RB1,PLPP3,PRKCH,XRCC5,GFRA1,BMX,NRK,SIK1,BDNF,TLR6,PPARG,NEK7,TENM1,CDK6,MYLK4,SPDYE16,TFRC,SPDYE6,MAP3K1,CLEC6A,ANGPT1,EIF4G1,ROCK1,PRKG1,PRKD1,STK4,TNNI3K,PRLR,TTN,DYRK1A,PARD3
GO:0023051	regulation of signaling	0.0000150401251880397 3	*FSTL1,RGN,CDK14,EPN2,MAP3K3,OSBPL8,ARHGEF9,ABR,MAOA,MAP3K13,PPM1A,LIMD1,CHST11,THRB,SPTBN1,USP32,FGF1,CHRNB2,SEZ6,ARHGAP15,BRD4,DOK4,CAMK2B,SLC38A9,KALRN,NOTCH2,TMOD2,NPNT,CDH11,SHANK3,FOXO1,STYXL1,STK39,SMURF2,ADAMTS12,DAAM2,TBC1D16,URB2,DIPK2A,PRKN,NLGN4X,FAM83B,IFT172,CHD7,CDON,NLK,GNAI1,TIAM2,LRP8,ARHGEF4,UBE2O,DDIAS,CALCR,ERBIN,RGS22,MCC,PTH,ADAR,PDE10A,CDC37,FGF10,MECOM,PLG,NCK1,NONO,NREP,KCNQ3,CHRM3,PREX1,BRAF,ALPK2,CHRNA7,HECW1,HMGB1,TGFB2,MDFIC,LAMA2,TRIO,NDST1,MUSK,SFPQ,MAP3K5,RGS3,NUP93,SLC15A4,GOLPH3,USP10,IGFBP7,EPHA5,SKOR2,ATP2C1,UBR5,SOX30,SLIT3,ROBO1,ITPR1,PAK3,DOK6,MLLT3,TNFRSF12A,OASL,LATS2,ERP29,ARHGAP18,LYN,KLF7,GRIK1,LARGE1,FIGNL1,NOS3,ERCC6,NOS1AP,EIF2AK4,TGFBR2,MARK4,TCF7L2,SHISA2,RRAGD,ULK3,GKAP1,ADGRV1,RASGRF2,SCG5,WAC,DAPK1,APP,GATA4,SMAD5,GSKIP,PELI2,PIK3R5,TLR1,BMT2,AVPR1B,PTK2,USP7,TICAM2,SH2B2,PIP4K2B,LRRK2,CLSTN2,ITFG2,PKHD1,PJA2,TIAM1,IQSEC2,ACVR2A,BRCC3,SNX13,RABGEF1,KIT,EPHA7,SPAG9,WNT5A,TRABD2B,ARFGEF2,CTNNB1,ANKRD17,CTSK,PRKDC,ESR1,TNFRSF19,ERC2,BCAR3,TPD52L1,WWOX,NEK10,VWC2L,GPC4,EFNA5,NGEF,NEUROD2,FNIP2,TNFSF11,IKBKB,AKAP6,ARHGEF28,CHRNB3,ACVR1,PID1,GRID2,ZNF423,RB1,NTNG1,PDE4B,ADIPOR1,PLPP3,HNF1B,LAMP2,PRKCH,DLC1,DENND2B,RGS17,GRIA4,RGS7BP,MGRN1,MID2,WLS,PRCP,CDK5RAP2,ARHGEF7,ATF3,FBN2,SRI,ARNT,BDNF,LDLRAD4,SEMA3E,TLR6,TMEM237,KIF16B,CDH2,PPARG,CHRNB4,USP9X,NEK7,TENM1,MYOF,NALCN,DDX60,SIPA1L3,LRFN2,SFRP4,JCAD,VAV2,TFRC,GRIA3,FBN1,HAPSTR1,CLEC6A,RAPGEF4,BMPER,ANGPT1,SEMA6A,PHLPP1,ROCK1,TAX1BP1,APIP,PML,MGLL,AFAP1L2,DGKG,SP100,NDUFAF2,MAGI2,ENSA,AIMP1,SLC30A10,PRKD1,USP34,STK4,PRLR,PTPRA,PDE11A,NMI,LPAR6,CTBP2,HHIP,DYRK1A,MYRIP,UNC13C,CUL4A,KCTD16,EYA4,SEMA5A,PMEPA1,PEX5L,METTL3,KCNN4,RNF6,VAV1,GRIA2,VDAC2,SPART*
GO:0010646	regulation of cell communicati	0.0000183446493858154 82	*FSTL1,RGN,CDK14,EPN2,MAP3K3,OSBPL8,ARHGEF9,ABR,MAOA,MAP3K13,PPM1A,LIMD1,CHST11,THRB,SPTBN1,USP32,FGF1,CHRNB2,SEZ6,*

	on		*ARHGAP15,BRD4,DOK4,CAMK2B,SLC38A9,KALRN,NOTCH2,TMOD2,NPNT,CDH11,SHANK3,FOXO1,STYXL1,STK39,SMURF2,ADAMTS12,DAAM2,URB2,DIPK2A,PRKN,NLGN4X,FAM83B,IFT172,CHD7,CDON,NLK,GNAI1,TIAM2,LRP8,ARHGEF4,UBE2O,DDIAS,CALCR,ERBIN,RGS22,MCC,PTH,ADAR,PDE10A,CDC37,FGF10,MECOM,PLG,NCK1,NONO,NREP,KCNQ3,CHRM3,PREX1,BRAF,ALPK2,CHRNA7,HECW1,HMGB1,TGFB2,MDFIC,LAMA2,TRIO,NDST1,MUSK,SFPQ,MAP3K5,RGS3,NUP93,SLC15A4,GOLPH3,USP10,IGFBP7,EPHA5,SKOR2,ATP2C1,ASPH,UBR5,SOX30,SLIT3,ROBO1,ITPR1,PAK3,DOK6,MLLT3,TNFRSF12A,OASL,LATS2,ERP29,ARHGAP18,LYN,KLF7,GRIK1,LARGE1,FIGNL1,NOS3,ERCC6,NOS1AP,EIF2AK4,TGFBR2,MARK4,TCF7L2,SHISA2,RRAGD,ULK3,GKAP1,ADGRV1,RASGRF2,SCG5,WAC,DAPK1,APP,GATA4,SMAD5,GSKIP,PELI2,PIK3R5,TLR1,BMT2,AVPR1B,PTK2,USP7,TICAM2,SH2B2,PIP4K2B,LRRK2,CLSTN2,ITFG2,PKHD1,PJA2,TIAM1,IQSEC2,ACVR2A,BRCC3,SNX13,RABGEF1,KIT,EPHA7,SPAG9,WNT5A,TRABD2B,ARFGEF2,CTNNB1,ANKRD17,CTSK,PRKDC,ESR1,TNFRSF19,ERC2,BCAR3,TPD52L1,WWOX,NEK10,VWC2L,GPC4,EFNA5,NGEF,NEUROD2,FNIP2,TNFSF11,IKBKB,AKAP6,ARHGEF28,CHRNB3,ACVR1,PID1,GRID2,ZNF423,RB1,NTNG1,PDE4B,ADIPOR1,PLPP3,HNF1B,LAMP2,PRKCH,DLC1,DENND2B,RGS17,GRIA4,RGS7BP,MGRN1,MID2,WLS,PRCP,CDK5RAP2,ARHGEF7,ATF3,TRDN,FBN2,SRI,ARNT,BDNF,LDLRAD4,SEMA3E,TLR6,TMEM237,CDH2,PPARG,CHRNB4,USP9X,NEK7,TENM1,MYOF,NALCN,DDX60,SIPA1L3,LRFN2,SFRP4,JCAD,VAV2,TFRC,GRIA3,FBN1,HAPSTR1,CLEC6A,RAPGEF4,BMPER,ANGPT1,SEMA6A,PHLPP1,ROCK1,TAX1BP1,APIP,PML,MGLL,AFAP1L2,DGKG,SP100,NDUFAF2,MAGI2,ENSA,AIMP1,SLC30A10,PRKD1,USP34,STK4,PRLR,PTPRA,PDE11A,NMI,LPAR6,CTBP2,HHIP,DYRK1A,MYRIP,UNC13C,CUL4A,KCTD16,EYA4,SEMA5A,PMEPA1,PEX5L,METTL3,KCNN4,RNF6,VAV1,GRIA2,VDAC2,SPART*
GO:0051641	cellular localization	0.000020496023745138727	*MREG,OSBPL8,STX8,SMYD3,RAPGEF6,BLTP3B,TRAPPC9,PPM1A,ARL13B,RN7SL556P,IMMP1L,RCSD1,SPTBN1,MYO5C,AGTPBP1,ATP7B,GRIP1,TLK1,RGPD2,XPO4,USH2A,NPNT,EPB41L3,MAP6,SHANK3,NR4A3,FOXO1,YBX1,CACNA1C,PRKN,NLGN4X,EXOC4,XPO6,IFT172,CHD7,GPSM1,SLC17A6,GNAI1,CFAP54,INTS6-AS1,TRAK2,UBE2O,EIF4ENIF1,ERBIN,CD247,MCC,CEP192,ADAR,TM9SF2,CLCN3,CDC37,FGF10,ZFYVE16,TSPAN18,ILDR2,CD84,KCNQ3,CHRM3,RGPD3,ANO3,CIDEB,MCPH1,MYO1D,BRAF,XPO7,DYNLT2B,ATRX,TGFB2,MDFIC,MUSK,JAM3,KCNIP3,NUP93,RBM33,TBC1D5,SLC15A4,GOLPH3,PRKG2,PIK3C3,EPHA5,SNX10,ATP2C1,TMEM230,STON2,STRADA,PCLO,HUWE1,ASPH,ZDHHC4,UBR5,GSN,PLS1,MAPRE3,ANO2,HPS3,ITPR1,DNAAF11,TRAPPC10,CPT2,VPS8,MTX3,CILK1,PACRG,EPHA3,LATS2,ERP29,UVRAG,PTPRN2,LYN,KLF7,SEC24C,LARGE1,ATP6V1H,NUP58,SIL1,TMCC1,TMED7-*

			TICAM2,NOS3,KCNIP4,NOMO2,RN7SL738P,KIAA1614,MAGEL2,MARK4,MDN1,SYNJ1,TCF7L2,UNC119,GPM6B,RRAGD,RGPD1,KPNB1,GSDMD,ADGRV1,EPB41L5,SCG5,WHRN,SAR1B,TTC7B,APP,HCN1,RHOT1,HERC2,HOOK3,USE1,LMAN1L,NOP9,USP7,SCP2,HYCC1,LRRK2,GET1-SH3BGR,PLD1,PKHD1,TIAM1,CSRP3,IQSEC2,NEDD4L,ENSG00000288683,SNX13,RABGEF1,CYB5R4,KIT,SPAG9,SPDL1,WNT5A,WIPI2,PEX26,ARFGEF2,CTNNB1,IPO5,ESR1,PTPN14,SCFD1,ERC2,VPS53,FRMD4A,DIAPH3,GPC4,PKIG,EFNA5,SYNE3,PLCH1,IKBKB,AKAP6,RAB3GAP2,TASOR,CEMIP,XIST,ATF2,PID1,CCDC91,GRID2,ZNF423,RB1,KIF13A,GOSR2,SMG7,HNF1B,IMMP2L,LAMP2,BNIP3L,PRKCH,RABGAP1L,XRCC5,RAB18,MGRN1,MID2,KAT2B,GOLPH3L,WLS,RYR3,MYO1B,CDK5RAP2,TRDN,NPHP1,FBN2,SRI,ATP1B1,BDNF,SCIN,ADAMTS9,KIF16B,RAB11FIP2,CDH2,PPARG,ASB3,MTTP,CHRNB4,USP9X,TENM1,IL13RA2,MYOF,JAKMIP1,GDAP1,KIF2C,MYO5A,SLC6A13,SPTBN5,BLTP1,TFRC,VPS13A,FBN1,RAB31,RAPGEF4,BMPER,FUT10,CLASP2,CLTC,ANGPT1,EIF4G1,ATP6V0A4,ITGA4,DNAJC6,ROCK1,TAX1BP1,NUP98,PML,SCFD2,SP100,NDUFAF2,MAGI2,NIN,ANKFY1,ASIC2,ENSA,AP4S1,PRKD1,CALCRL,STK4,RAB6D,SPC25,SCAMP1,RAB6C,CTBP2,TTN,PPFIA1,TMEM30A,MYRIP,UNC13C,PEX5L,PARD3,KCNN4,ATP8B1,VPS13B
GO:0050808	synapse organization	0.000022371879714799223	*APBB2,LRRN1,NFIA,ARHGEF9,CACNB1,CHRNB2,SEZ6,CDH8,CAMK2B,KALRN,SDK2,SHANK3,PLXNA4,NLGN4X,LRP8,ZNF804A,CHRNA7,MUSK,PCLO,PAK3,IL1RAPL2,ADGRB3,LARGE1,SPTB,PCDHB16,NOS1AP,APP,LRRK2,CLSTN2,TIAM1,EPHA7,WNT5A,CTNNB1,ERC2,LMX1A,FILIP1,GPC4,EFNA5,NGEF,NEUROD2,PTPRF,LINGO2,GRID2,NTNG1,GABRB3,KIRREL3,GABRG2,LRTM2,BDNF,SEMA3E,CDH2,TANC1,LRFN2,IL1RAP,EIF4G1,ROCK1,CDH9,ASIC2,CTBP2,PPFIA1,UNC13C*
GO:0048812	neuron projection morphogenesis	0.000026777785008433025	*APBB2,MAP3K13,CHRNB2,CAMK2B,GRIP1,KALRN,NOTCH2,EPB41L3,MAP6,CDH11,SHANK3,PLXNA4,PRKN,TIAM2,LRP8,CNTN1,BRAF,CHRNA7,HECW1,LAMA2,TRIO,MUSK,EPHA5,SKOR2,ENAH,CHL1,SLIT3,ROBO1,PAK3,NCKAP1,EPHA3,ADGRB3,KLF7,DIP2B,EIF2AK4,MATN2,CUX1,APP,IGF2BP1,PALLD,PTK2,LRRK2,TIAM1,PHACTR1,NEDD4L,KIT,EPHA7,SPAG9,WNT5A,CTNNB1,LMX1A,EFNA5,NGEF,TRPC5,ARHGEF28,NTNG1,KIRREL3,ETV1,LRTM2,BDNF,SEMA3E,CDH2,USP9X,CLASP2,SEMA6A,ITGA4,ROCK1,PRKG1,ELAVL4,NIN,SEMA5A,PARD3,RNF6,SEMA3C,SPART*
GO:0023052	signaling	0.000033084470118127224	*FSTL1,APBB2,RGN,CDK14,EPN2,PDE1C,NOSTRIN,MAP3K3,OSBPL8,NFIA,RAPGEF6,ARHGEF9,TAFA2,ABR,MAOA,MAP3K13,PPM1A,ARL13B,LIMD1,TF,CHST11,THRB,SPTBN1,CDC42EP3,MAPK10,CACNB1,USP32,DAPP1,GNG12,FGF1,CHRNB2,SEZ6,CDH8,ARHGAP15,GNB4,RBPMS,SKAP2,BRD4,DOK4,CAMK2B,GRIP1,ARHGAP11A-SCG5,TLK1,DLGAP1,SLC38A9,HBP1,ABCG8,KA*

			LRN,MAP4K3,NOTCH2,TMOD2,NPNT,CDH11,SHANK3,NR4A3,E2F7,FOXO1,STYXL1,STK39,SMURF2,ADAMTS12,DAAM2,TBC1D16,PLXNA4,URB2,STK24,DIPK2A,DCDC1,CACNA1C,FAM3C,ASB4,PRKN,NLGN4X,EXOC4,PIK3C2B,SLC5A7,FAM83B,IFT172,CHD7,CDON,SLC17A6,NLK,GNAI1,MAPKAPK3,TIAM2,LRP8,CNTN1,STAT5B,ENPEP,ARHGEF4,UBE2O,DDIAS,CPEB4,CALCR,CHRFAM7A,ELMO1,ERBIN,MBTPS2,RGS22,CD247,MCC,PTH,ADAR,PDE10A,CLCN3,RHOJ,CDC37,FGF10,ZFYVE16,MECOM,COL4A6,STK32B,PLG,HERC5,ILDR2,NCK1,NONO,NREP,GABRA5,KCNQ3,MTCP1,CHRM3,PREX1,CIDEB,BRAF,COL1A2,DTNA,ALPK2,CHRNA7,HECW1,HMGB1,ZNF304,CDIP1,HIVEP1,ATRX,TGFB2,MDFIC,LAMA2,TRIO,SMARCC1,NDST1,MUSK,KCNIP3,SFPQ,MAP3K5,RGS3,NUP93,RERG,OR5AU1,PRKAR2A,SLC15A4,GOLPH3,PRKG2,MBTPS1,PIK3C3,USP10,IGFBP7,EPHA5,SKOR2,ENSG00000289694,ATP2C1,ALDH1A2,PTGFR,PCLO,ASPH,RRAS2,UBR5,SOX30,ANKS1B,CHL1,TCP11L1,TLR8,SLIT3,ROBO1,ITPR1,PKP4,PAK3,DOK6,PRKAG2,MLLT3,TNFRSF12A,OASL,TAS2R14,CXCR5,NCKAP1,CILK1,FOSL2,IL1RAPL2,ADGRD1,EPHA3,LATS2,ERP29,ARHGAP18,PTPRN2,LYN,ADGRB3,KLF7,GRIK1,LARGE1,FIGNL1,PCDHB16,PPM1L,TMED7-TICAM2,NOS3,ERCC6,NOS1AP,EIF2AK4,RAB12,TGFBR2,CMKLR2,ITGA11,MARK4,SYNJ1,TCF7L2,UNC119,SHISA2,RRAGD,ULK3,GPR156,GKAP1,PPP2R5E,ADGRV1,RASGRF2,GAD1,SCG5,WAC,DAPK1,TDP2,INSYN2A,APP,GATA4,SMAD5,GSKIP,ADAM12,PELI2,CACNA2D1,CHRM5,PI4KB,RHOT1,PIK3R5,TLR1,TAS2R30,BMT2,AVPR1B,PTK2,USP7,TICAM2,SH2B2,PIP4K2B,LRRK2,CLSTN2,ARIH1,PLD1,ITFG2,VTCN1,PKHD1,RARB,PJA2,TIAM1,CSRP3,IQSEC2,ACVR2A,GNAO1,TIPIN,RASSF4,BRCC3,SNX13,RABGEF1,CYB5R4,KIT,EPHA7,SPAG9,SPDL1,WNT5A,TRABD2B,ARFGEF2,TEX2,CTNNB1,ANKRD17,CTSK,PRKDC,IPO5,ESR1,TNFRSF19,ITGB8,ERC2,BCAR3,TPD52L1,WWOX,HUNK,NEK10,PTGER4,PDE7B,VWC2L,DOCK1,DIAPH3,GPC4,EFNA5,CDC14B,PLCH1,OR1M1,NGEF,NEUROD2,FNIP2,TNFSF11,PTPRF,IKBKB,SH3BP5,AKAP6,TRBV10-2,ARHGEF28,OR8B8,ATF2,CHRNB3,ACVR1,PID1,GRID2,ZNF423,KCNJ3,RB1,IFNLR1,NTNG1,PDE4B,ADIPOR1,GABRB3,PLPP3,HNF1B,LAMP2,GABRG2,NCOA2,BNIP3L,PRKCH,DLC1,DENND2B,RGS17,GFRA1,KCND2,INPP4A,BMX,GRIA4,RAB18,RGS7BP,MGRN1,MID2,SIK1,TP53BP2,WLS,ANKS1A,RYR3,PRCP,CDK5RAP2,ARHGEF7,SAMD12,ATF3,NPHP1,FBN2,SRI,ATP1B1,ARNT,BDNF,LDLRAD4,SEMA3E,MACO1,TLR6,TMEM237,KIF16B,RAB11FIP2,CDH2,LAG3,PPARG,PAPPA,ASB3,CHRNB4,USP9X,NEK7,TENM1,IL13RA2,RORB,CDK6,GABRB1,MYOF,LTB4R2,SH3KBP1,NALCN,DDX60,MYO5A,SIPA1L3,LRFN2,SFRP4,IL1RAP,JCAD,VAV2,TFRC,GRIA3,PNPLA8,FBN1,HAPSTR1,MAP3K1,CLEC6A,RAPGEF4,BMPER,ANGPT1,SEMA6A,VIPR2,PHLPP1,ITGA4,ROCK1,TAX1BP1,APIP,PML,MGLL,PLEK2,TNFSF4,AFA

			P1L2,DGKG,SP100,PRKG1,NDUFAF2,MAGI2,SPG21,ASIC2,ENSA,AIMP1,SLC30A10,PRKD1,CALCRL,USP34,STK4,SPC25,PRLR,PTPRA,PDE11A,NMI,RAB6C,IL16,EDARADD,GNA14,CR2,LPAR6,CTBP2,TTN,NCALD,HHIP,DYRK1A,PPFIA1,MYRIP,PPP1R1C,UNC13C,CUL4A,KCTD16,EYA4,DLGAP2,SEMA5A,PMEPA1,PEX5L,METTL3,KCNN4,RNF6,SEMA3C,VAV1,GRIA2,VDAC2,SPART
GO:0120039	plasma membrane bounded cell projection morphogenesis	0.0000341719198346496 8	*APBB2,MAP3K13,CHRNB2,CAMK2B,GRIP1,KALRN,NOTCH2,EPB41L3,MAP6,CDH11,SHANK3,PLXNA4,PRKN,TIAM2,LRP8,CNTN1,BRAF,CHRNA7,HECW1,LAMA2,TRIO,MUSK,EPHA5,SKOR2,ENAH,CHL1,SLIT3,ROBO1,PAK3,NCKAP1,EPHA3,ADGRB3,KLF7,DIP2B,EIF2AK4,MATN2,CUX1,APP,IGF2BP1,PALLD,PTK2,LRRK2,TIAM1,PHACTR1,NEDD4L,KIT,EPHA7,SPAG9,WNT5A,CTNNB1,LMX1A,EFNA5,NGEF,TRPC5,ARHGEF28,NTNG1,KIRREL3,ETV1,LRTM2,ARHGEF7,BDNF,SEMA3E,CDH2,USP9X,CLASP2,SEMA6A,ITGA4,ROCK1,PRKG1,ELAVL4,NIN,SEMA5A,PARD3,RNF6,SEMA3C,SPART*
GO:0030154	cell differentiation	0.0000419182228629158 4	*FSTL1,APBB2,RTN1,MREG,OSBPL8,NFIA,TXNDC8,SMYD3,TRAPPC9,MAP3K13,LIMD1,TF,CHST11,THRB,USP42,FGF1,CHRNB2,SEZ6,AGTPBP1,FHL1,SH3PXD2A,SPINK5,BRD4,CAMK2B,GRIP1,DCT,KALRN,USH2A,NOTCH2,TMOD2,SDK2,NPNT,EPB41L3,MAP6,MORC1,CDH11,SHANK3,NR4A3,ARID1B,E2F7,FOXO1,STYXL1,ADAMTS12,DAAM2,PLXNA4,YBX1,STK24,ASB4,ARB2A,PRKN,NLGN4X,PNPLA3,IFT172,CHD7,GPSM1,PTBP3,CDON,SERPINI1,SDF4,THSD7A,NCAM2,TIAM2,CFAP54,TRAK2,CNMD,LRP8,CNTN1,STAT5B,EIF4ENIF1,HEATR9,CALCR,PTH,ADAR,FGF10,MECOM,CATSPERB,PLG,ILDR2,NCK1,NREP,GABRA5,NEXMIF,KCNQ3,PREX1,COL11A1,ZNF804A,BRAF,TMOD3,MRTFB,COL12A1,ALPK2,CHRNA7,HECW1,HMGB1,RFLNA,ATRX,TGFB2,LAMA2,TRIO,SMARCC1,MUSK,JAM3,MAP3K5,ALKBH1,ATP11A,PRKG2,SND1,EPHA5,SKOR2,RUNX1,ENAH,SNX10,ALDH1A2,HUWE1,RRAS2,SIM1,SOX30,CHL1,PLS1,SLIT3,ROBO1,PAK3,ABI3BP,AXDND1,MLLT3,CCNT2,BTG4,MR1,TNFRSF12A,NCKAP1,FOSL2,PACRG,EPHA3,LATS2,MYOM2,LYN,ADGRB3,KLF7,MTA3,DIP2B,LARGE1,FIGNL1,PUS7,RNASEL,NAV1,PAQR5,VSX1,PRAMEF27,ERCC6,EIF2AK4,MATN2,TGFBR2,ITGA11,TCF7L2,ZNF800,CRTAC1,ZFHX3,CUX1,GPM6B,ADGRV1,ST7,EPB41L5,WHRN,OCA2,TDP2,PRUNE1,APP,GATA4,SMAD5,IGF2BP1,ADAM12,HCN1,FRY,HOOK3,PALLD,CECR2,PTK2,SH2B2,CELF1,PRRC2B,LRRK2,IKZF3,ITFG2,PKHD1,TENT5C,RARB,TCF4,PAEP,TIAM1,CSRP3,PHACTR1,ACVR2A,NTM,MEIS1,NEDD4L,CYB5R4,KIT,EPHA7,SPAG9,WNT5A,CTNNB1,ANKRD17,COL19A1,RUNX1T1,CTSK,PRKDC,ESR1,ITGB8,POFUT2,LMX1A,WWOX,PTGER4,VWC2L,DOCK1,DIAPH3,BPGM,EFNA5,NGEF,TRPC5,NEUROD2,TNFSF11,PTPRF,IKBKB,AKAP6,USF3,ARHGEF28,TASOR,GPRIN1,POU6F2,OPCML,ATF2,ACVR1,EBF2,GRID2,ZNF423,RB1,NTNG1,ADIPOR1,PLPP3,HNF1B,KIRREL3,CPS1,TMEM120A,PRKCH,AGPAT5,XRCC5,GFRA1,*

			ETV1,TEX11,LRTM2,SIK1,KAT2B,ANKS1A,MEF2D,CDK5RAP2,FLVCR1,ARHGEF7,ATF3,NPHP1,FBN2,IKZF1,SLC26A8,ARNT,BDNF,LDLRAD4,TFCP2L1,SEMA3E,SCIN,ADAMTS9,CDH2,LAG3,PPARG,TANC1,USP9X,TENM1,RORB,CDK6,GABRB1,AVIL,SIPA1L3,SFRP4,BLTP1,TFRC,FBN1,DDX4,IQCB1,FUT10,CLASP2,CLTC,ZNF521,ANGPT1,SEMA6A,EIF4G1,ITGA4,ROCK1,M1AP,PML,TNFSF4,DGKG,SPATA25,PRKG1,ELAVL4,MAGI2,NIN,FNDC3A,PRKD1,STK4,ANPEP,PRLR,EDARADD,CR2,CTBP2,TTN,HHIP,TMEM30A,CUL4A,TXNRD3,EYA4,SEMA5A,LAMA4,TTPA,GATAD2B,METTL3,KRT85,PARD3,ATP8B1,RNF6,SEMA3C,VAV1,SMYD1,VPS13B,SPART
GO:0048869	cellular developmental process	0.00004297424059506592	*FSTL1,APBB2,RTN1,MREG,OSBPL8,NFIA,TXNDC8,SMYD3,TRAPPC9,MAP3K13,LIMD1,TF,CHST11,THRB,USP42,FGF1,CHRNB2,SEZ6,AGTPBP1,FHL1,SH3PXD2A,SPINK5,BRD4,CAMK2B,GRIP1,DCT,KALRN,USH2A,NOTCH2,TMOD2,SDK2,NPNT,EPB41L3,MAP6,MORC1,CDH11,SHANK3,NR4A3,ARID1B,E2F7,FOXO1,STYXL1,ADAMTS12,DAAM2,PLXNA4,YBX1,STK24,ASB4,ARB2A,PRKN,NLGN4X,PNPLA3,IFT172,CHD7,GPSM1,PTBP3,CDON,SERPINI1,SDF4,THSD7A,NCAM2,TIAM2,CFAP54,TRAK2,CNMD,LRP8,CNTN1,STAT5B,EIF4ENIF1,HEATR9,CALCR,PTH,ADAR,FGF10,MECOM,CATSPERB,PLG,ILDR2,NCK1,NREP,GABRA5,NEXMIF,KCNQ3,PREX1,COL11A1,ZNF804A,BRAF,TMOD3,MRTFB,COL12A1,ALPK2,CHRNA7,HECW1,HMGB1,RFLNA,ATRX,TGFB2,LAMA2,TRIO,SMARCC1,MUSK,JAM3,MAP3K5,ALKBH1,ATP11A,PRKG2,SND1,EPHA5,SKOR2,RUNX1,ENAH,SNX10,ALDH1A2,HUWE1,RRAS2,SIM1,SOX30,CHL1,PLS1,SLIT3,ROBO1,PAK3,ABI3BP,AXDND1,MLLT3,CCNT2,BTG4,MR1,TNFRSF12A,NCKAP1,FOSL2,PACRG,EPHA3,LATS2,MYOM2,LYN,ADGRB3,KLF7,MTA3,DIP2B,LARGE1,FIGNL1,PUS7,RNASEL,NAV1,PAQR5,VSX1,PRAMEF27,ERCC6,EIF2AK4,MATN2,TGFBR2,ITGA11,TCF7L2,ZNF800,CRTAC1,ZFHX3,CUX1,GPM6B,ADGRV1,ST7,EPB41L5,WHRN,OCA2,TDP2,PRUNE1,APP,GATA4,SMAD5,IGF2BP1,ADAM12,HCN1,FRY,HOOK3,PALLD,CECR2,PTK2,SH2B2,CELF1,PRRC2B,LRRK2,IKZF3,ITFG2,PKHD1,TENT5C,RARB,TCF4,PAEP,TIAM1,CSRP3,PHACTR1,ACVR2A,NTM,MEIS1,NEDD4L,CYB5R4,KIT,EPHA7,SPAG9,WNT5A,CTNNB1,ANKRD17,COL19A1,RUNX1T1,CTSK,PRKDC,ESR1,ITGB8,POFUT2,LMX1A,WWOX,PTGER4,VWC2L,DOCK1,DIAPH3,BPGM,EFNA5,NGEF,TRPC5,NEUROD2,TNFSF11,PTPRF,IKBKB,AKAP6,USF3,ARHGEF28,TASOR,GPRIN1,POU6F2,OPCML,ATF2,ACVR1,EBF2,GRID2,ZNF423,RB1,NTNG1,ADIPOR1,PLPP3,HNF1B,KIRREL3,CPS1,TMEM120A,PRKCH,AGPAT5,XRCC5,GFRA1,ETV1,TEX11,LRTM2,SIK1,KAT2B,ANKS1A,MEF2D,CDK5RAP2,FLVCR1,ARHGEF7,ATF3,NPHP1,FBN2,IKZF1,SLC26A8,ARNT,BDNF,LDLRAD4,TFCP2L1,SEMA3E,SCIN,ADAMTS9,CDH2,LAG3,PPARG,TANC1,USP9X,TENM1,RORB,CDK6,GABRB1,AVIL,SIPA1L3,SFRP4,BLTP1,TFRC,FBN1,DDX4,IQCB1,FUT10,CLASP2,CLTC,ZNF521,ANG*

			PT1,SEMA6A,EIF4G1,ITGA4,ROCK1,M1AP,PML,TNFSF4,DGKG,SPATA25,PRKG1,ELAVL4,MAGI2,NIN,FNDC3A,PRKD1,STK4,ANPEP,PRLR,EDARADD,CR2,CTBP2,TTN,HHIP,TMEM30A,CUL4A,TXNRD3,EYA4,SEMA5A,LAMA4,TTPA,GATAD2B,METTL3,KRT85,PARD3,ATP8B1,RNF6,SEMA3C,VAV1,SMYD1,VPS13B,SPART
GO:0048858	cell projection morphogenesis	0.000046970612177447805	*APBB2,MAP3K13,CHRNB2,CAMK2B,GRIP1,KALRN,NOTCH2,EPB41L3,MAP6,CDH11,SHANK3,PLXNA4,PRKN,TIAM2,LRP8,CNTN1,BRAF,CHRNA7,HECW1,LAMA2,TRIO,MUSK,EPHA5,SKOR2,ENAH,CHL1,SLIT3,ROBO1,PAK3,NCKAP1,EPHA3,ADGRB3,KLF7,DIP2B,EIF2AK4,MATN2,CUX1,APP,IGF2BP1,PALLD,PTK2,LRRK2,TIAM1,PHACTR1,NEDD4L,KIT,EPHA7,SPAG9,WNT5A,CTNNB1,LMX1A,EFNA5,NGEF,TRPC5,ARHGEF28,NTNG1,KIRREL3,ETV1,LRTM2,ARHGEF7,BDNF,SEMA3E,CDH2,USP9X,CLASP2,SEMA6A,ITGA4,ROCK1,PRKG1,ELAVL4,NIN,SEMA5A,PARD3,RNF6,SEMA3C,SPART*
GO:0048667	cell morphogenesis involved in neuron differentiation	0.0000673439207433628 2	*APBB2,MAP3K13,CHRNB2,CAMK2B,KALRN,NOTCH2,MAP6,CDH11,SHANK3,PLXNA4,TIAM2,LRP8,CNTN1,BRAF,CHRNA7,HECW1,LAMA2,TRIO,MUSK,EPHA5,SKOR2,ENAH,CHL1,PLS1,SLIT3,ROBO1,PAK3,EPHA3,ADGRB3,KLF7,DIP2B,MATN2,CUX1,WHRN,APP,IGF2BP1,PALLD,PTK2,LRRK2,TIAM1,PHACTR1,NEDD4L,KIT,EPHA7,WNT5A,LMX1A,EFNA5,NGEF,TRPC5,ARHGEF28,RB1,NTNG1,ETV1,LRTM2,BDNF,SEMA3E,CDH2,USP9X,CLASP2,SEMA6A,ITGA4,PRKG1,ELAVL4,NIN,SEMA5A,PARD3,RNF6,SEMA3C,SPART*
GO:0034330	cell junction organization	0.000086547783241291 82	*APBB2,LRRN1,NFIA,ARHGEF9,CACNB1,CHRNB2,SEZ6,CLDN6,CDH8,CAMK2B,KALRN,SDK2,EPB41L3,CDH11,SHANK3,PLXNA4,NLGN4X,LRP8,CDH7,ZNF804A,CHRNA7,TGFB2,MUSK,JAM3,PCLO,PKP4,PAK3,IL1RAPL2,EPHA3,ADGRB3,LARGE1,SPTB,PCDHB16,NOS1AP,GPM6B,EPB41L5,WHRN,APP,PTK2,LRRK2,CLSTN2,PKHD1,TIAM1,EPHA7,WNT5A,CTNNB1,ERC2,LMX1A,FILIP1,GPC4,EFNA5,NGEF,NEUROD2,PTPRF,IKBKB,LINGO2,GRID2,NTNG1,GABRB3,KIRREL3,GABRG2,PRKCH,DLC1,LRTM2,ARHGEF7,NPHP1,BDNF,SEMA3E,CDH2,TANC1,LRFN2,IL1RAP,CLASP2,EIF4G1,ROCK1,CDH9,ASIC2,PTPRA,CTBP2,PPFIA1,UNC13C,PARD3*
GO:0030029	actin filament-based process	0.000134937642444835 3	*NOSTRIN,TF,SPTBN1,CDC42EP3,MYO5C,TMOD2,EPB41L3,PARVB,SHANK3,DAAM2,CACNA1C,PRKN,THSD7A,TAGLN3,ELMO1,RHOJ,FGF10,NCK1,PREX1,MYO1D,BRAF,TMOD3,RFLNA,EPB41L4A,ADD3,JAM3,HMCN1,EPHA5,ENAH,ATP2C1,FAM171A1,GSN,PLS1,PAK3,NCKAP1,EPHA3,MYH14,ARHGAP18,MYOM2,SPTB,NOS1AP,MAGEL2,GPM6B,EPB41L5,GATA4,CACNA2D1,RHOT1,PALLD,SH2B2,CSRP3,PHACTR1,IQSEC2,NEDD4L,KIT,ANTXR1,PPM1E,PTGER4,DIAPH3,EFNA5,IKBKB,KCNJ3,PDE4B,SHROOM4,DLC1,ACTR3C,MYO1B,NPHP1,CAPZA2,SRI,SCIN,TENM1,SH3KBP1,AVIL,MYO5A,SPTBN5,CLASP2,ROCK1,PLEK2,PRKG1,PRKD1,TTN,PPFIA1,KCNE2,SEMA5A,FMNL2*

GO:0051128	regulation of cellular component organization	0.0001457566149004886 6	*C10ORF90,LRRN1,ARHGEF9,MAP3K13,TF,SPTBN1,CDC42EP3,CHRNB2,SEZ6,CDH8,FHL1,ATP10A,CAMK2B,GRIP1,TLK1,KALRN,TMOD2,HMBOX1,MAP6,SHANK3,ARID1B,STYXL1,DAAM2,PLXNA4,STK24,PRKN,SERPINI1,GNAI1,TIAM2,LRP8,CNTN1,ZFYVE16,NCK1,KDM2B,PREX1,ZNF804A,MCPH1,BRAF,TMOD3,CHRNA7,HECW1,HMGB1,DYNLT2B,ATRX,TGFB2,LAMA2,ADD3,SMARCC1,MUSK,JAM3,RERG,TBC1D5,GOLPH3,USP10,IGFBP7,EPHA5,SKOR2,RUNX1,STON2,HUWE1,ASPH,GSN,PLS1,MAPRE3,SLIT3,ARMH3,ROBO1,PAK3,MLLT3,VPS8,NCKAP1,IL1RAPL2,EPHA3,ARHGAP18,UVRAG,LYN,ADGRB3,DIP2B,NME7,SPTB,DCP2,MAGEL2,MARK4,SYNJ1,CUX1,UNC119,GPM6B,EPB41L5,PRUNE1,SAR1B,APP,RHOT1,PTK2,USP7,PIP4K2B,LRRK2,INO80D,CLSTN2,PLD1,PKHD1,TIAM1,NEDD4L,BRCC3,RABGEF1,KIT,EPHA7,SPAG9,ANTXR1,SPDL1,WNT5A,TRABD2B,CTNNB1,SCFD1,ERC2,PPM1E,LMX1A,PTGER4,DIAPH3,GPC4,EFNA5,NGEF,TRPC5,NEUROD2,FNIP2,PTPRF,IKBKB,FXR2,AKAP6,RAB3GAP2,LINGO2,HDAC8,PID1,GRID2,RB1,NSMCE1,NTNG1,ADIPOR1,LAMP2,PRKCH,DLC1,XRCC5,LRTM2,KAT2B,SUPV3L1,CDK5RAP2,ARHGEF7,NPHP1,CAPZA2,BDNF,SEMA3E,SCIN,TENT4B,TLR6,HAUS6,CDH2,PPARG,TANC1,NEK7,TENM1,AVIL,LRFN2,SPTBN5,SFRP4,BLTP1,IL1RAP,TFRC,RAB31,CLASP2,CLTC,ANGPT1,SEMA6A,EIF4G1,DNAJC6,ROCK1,NCAPH,TBC1D4,PML,SACS,PLEK2,DGKG,ELAVL4,MAGI2,NIN,ANKFY1,ASIC2,PRKD1,SPC25,PTPRA,RAB6C,YLPM1,DYRK1A,PPFIA1,TMEM30A,SEMA5A,ATP8B1,RNF6,SEMA3C,VDAC2,SPART*
GO:0044087	regulation of cellular component biogenesis	0.0001596670024203581 9	*C10ORF90,LRRN1,ARHGEF9,SPTBN1,CDC42EP3,CHRNB2,TMOD2,SHANK3,STYXL1,DAAM2,PRKN,NCK1,PREX1,BRAF,TMOD3,HMGB1,DYNLT2B,ADD3,MUSK,JAM3,USP10,GSN,MAPRE3,PAK3,VPS8,NCKAP1,IL1RAPL2,EPHA3,ARHGAP18,ADGRB3,NME7,LDB2,SPTB,MARK4,GPM6B,EPB41L5,PRUNE1,SAR1B,APP,PTK2,PIP4K2B,LRRK2,CLSTN2,PLD1,BRCC3,KIT,EPHA7,ANTXR1,WNT5A,TRABD2B,CTNNB1,SCFD1,PPM1E,PTGER4,GPC4,EFNA5,FNIP2,IKBKB,FXR2,RAB3GAP2,LINGO2,GRID2,RB1,LAMP2,PRKCH,DLC1,XRCC5,LRTM2,KAT2B,CDK5RAP2,ARHGEF7,NPHP1,CAPZA2,BDNF,SCIN,TLR6,HAUS6,PPARG,NEK7,TENM1,AVIL,SPTBN5,IL1RAP,TFRC,CLASP2,EIF4G1,DNAJC6,ROCK1,SACS,PLEK2,ASIC2,PRKD1,PTPRA,DYRK1A,PPFIA1,ATP8B1,VDAC2*
GO:0007155	cell adhesion	0.0001748271431513401 3	*ENSG00000290149,MYBPC1,CLDN6,CDH8,SPINK5,BRD4,ZAN,CD93,SDK2,NPNT,DSC1,PARVB,CDH11,NR4A3,ARID1B,ADAMTS12,PLXNA4,IDO1,NLGN4X,CBLL2,CDON,NCAM2,CNTN1,STAT5B,ERBIN,COL4A6,PLG,CNTNAP5,CDH7,ILDR2,NCK1,CD84,NEXMIF,PREX1,BRAF,TMOD3,COL12A1,HMGB1,LPP,TGFB2,LAMA2,SMARCC1,MUSK,JAM3,PCDHB8,GOLPH3,HMCN1,IGFBP7,RUNX1,ATP2C1,CHL1,SPON1,ROBO1,PKP4,ABI3BP,TNFRSF12A,CLDN12,CNTN3,EPHA3,LYN,PCDHB16,TGFBR2,ITGA11,ZFHX3,GPM6B,ADGRV1,EPB41L5,APP,ADAM12,PALLD,PTK2,CLSTN2,VTCN1*

			,*PKHD1,TIAM1,VIT,NTM,KIT,EPHA7,ANTXR1,WNT5A,CTNNB1,COL19A1,ITGB8,DOCK1,GPC4,EFNA5,JAML,TNFSF11,PTPRF,LYVE1,OPCML,ACVR1,HLA-DQA2,COL6A5,GRID2,NTNG1,PLPP3,SSPN,KIRREL3,DLC1,BMX,ARHGEF7,NPHP1,ATP1B1,SEMA3E,ADAMTS9,CDH2,LAG3,TENM1,CDK6,IL1RAP,JCAD,TFRC,FBN1,CLASP2,ANGPT1,SEMA6A,ITGA4,ROCK1,PML,TNFSF4,PRKG1,CDH9,PCDH19,FNDC3A,STK4,PCDH10,PRLR,PTPRA,PPFIA1,SEMA5A,LAMA4,METTL3,PARD3,COL6A6,VAV1*
GO:0048583	regulation of response to stimulus	0.000192450820395498 2	*FSTL1,RGN,CDK14,EPN2,ING3,MAP3K3,OSBPL8,ARHGEF9,ABR,MAOA,MAP3K13,PPM1A,SAMSN1,LIMD1,CHST11,THRB,SPTBN1,MAPK10,USP32,FGF1,SEZ6,ARHGAP15,SPINK5,BRD4,DOK4,CAMK2B,SLC38A9,KALRN,NOTCH2,TMOD2,NPNT,SHANK3,NR4A3,ARID1B,FOXO1,STYXL1,STK39,SMURF2,ADAMTS12,DAAM2,PLXNA4,URB2,STK24,DIPK2A,IDO1,PRKN,NLGN4X,FAM83B,IFT172,CDON,NLK,GNAI1,MAPKAPK3,TIAM2,LRP8,STAT5B,ARHGEF4,UBE2O,DDIAS,CALCR,ERBIN,MBTPS2,RGS22,CD247,MCC,PTH,ADAR,PDE10A,CDC37,FGF10,MECOM,NLRP8,PLG,HERC5,NCK1,FAM168A,NONO,NREP,PREX1,NCOA7,MCPH1,BRAF,ALPK2,CHRNA7,HECW1,HMGB1,TGFB2,MDFIC,LAMA2,TRIO,SMARCC1,NDST1,MUSK,JAM3,SFPQ,MAP3K5,RGS3,NUP93,SLC15A4,GOLPH3,USP10,IGFBP7,SKOR2,ATP2C1,UBR5,SOX30,TLR8,SLIT3,NLRP7,ROBO1,ITPR1,PAK3,DOK6,MLLT3,MR1,TNFRSF12A,OASL,FOSL2,LATS2,ERP29,ARHGAP18,LYN,KLF7,FIGNL1,TMED7-TICAM2,NOS3,ERCC6,NOS1AP,EIF2AK4,TGFBR2,MARK4,TCF7L2,SHISA2,RRAGD,ULK3,GKAP1,GSDMD,ADGRV1,RASGRF2,WAC,DAPK1,APP,GATA4,SMAD5,GSKIP,PELI2,PIK3R5,TLR1,BMT2,AVPR1B,PTK2,USP7,TICAM2,SH2B2,CELF1,PIP4K2B,LRRK2,INO80D,ITFG2,VTCN1,PKHD1,PJA2,TIAM1,MLIP,IQSEC2,ACVR2A,BRCC3,SNX13,RABGEF1,KIT,EPHA7,SPAG9,WNT5A,TRABD2B,ARFGEF2,CTNNB1,ANKRD17,CTSK,PRKDC,IPO5,ESR1,TNFRSF19,BCAR3,TPD52L1,WWOX,NEK10,PTGER4,VWC2L,GPC4,NGEF,NEUROD2,FNIP2,TNFSF11,PTPRF,IKBKB,AKAP6,APOH,ARHGEF28,ACVR1,HLA-DQA2,PID1,GRID2,ZNF423,RB1,IFNLR1,PDE4B,NLRP4,ADIPOR1,PLPP3,HNF1B,LAMP2,NCOA2,PRKCH,ACACB,DLC1,DENND2B,XRCC5,RGS17,SHLD2,BMX,RGS7BP,MGRN1,MID2,KAT2B,WLS,PRCP,CDK5RAP2,ATF3,FBN2,ARNT,BDNF,LDLRAD4,SEMA3E,TLR6,TMEM237,RAB11FIP2,CDH2,LAG3,PPARG,USP9X,NEK7,TENM1,DDX60,USP25,SIPA1L3,SFRP4,JCAD,VAV2,TFRC,PNPLA8,FBN1,HAPSTR1,MAP3K1,CLEC6A,BMPER,CLASP2,ANGPT1,SEMA6A,EIF4G1,PHLPP1,ROCK1,TAX1BP1,APIP,MMP28,PML,MGLL,TNFSF4,AFAP1L2,DGKG,SP100,PRKG1,NDUFAF2,MAGI2,SPG21,SLC30A10,PRKD1,USP34,STK4,PRLR,PDE11A,NMI,IL16,CR2,LPAR6,CTBP2,HHIP,DYRK1A,CUL4A,KCTD16,EYA4,SEMA5A,PMEPA1,PEX*

			5L,METTL3,KCNN4,RNF6,SEMA3C,VAV1,FANCB,VDAC2,SPART
GO:0120036	plasma membrane bounded cell projection organization	0.000211457336582976 6	*APBB2,RAPGEF6,MAP3K13,ARL13B,CDC42EP3,CHRNB2,CDC14C,SEZ6,CAMK2B,GRIP1,KALRN,NOTCH2,EPB41L3,MAP6,PARVB,CDH11,SHANK3,STYXL1,DAAM2,PLXNA4,STK24,CFAP70,PRKN,IFT172,SERPINI1,NCAM2,TIAM2,CFAP54,LRP8,CNTN1,ARHGEF4,NCK1,NREP,PREX1,ZNF804A,BRAF,ABLIM1,CHRNA7,HECW1,HMGB1,DYNLT2B,LAMA2,TRIO,MUSK,ALKBH1,GOLPH3,EPHA5,SKOR2,ENAH,SNX10,CHL1,GSN,PLS1,SLIT3,ROBO1,DNAAF11,PAK3,NCKAP1,CILK1,EPHA3,LYN,ADGRB3,KLF7,DIP2B,ERCC6,EIF2AK4,MATN2,MARK4,CRTAC1,CUX1,GPM6B,ADGRV1,WHRN,APP,IGF2BP1,FRY,PALLD,CECR2,PTK2,LRRK2,CFAP298,PLD1,PKHD1,TIAM1,PHACTR1,NEDD4L,KIT,EPHA7,SPAG9,WNT5A,CTNNB1,LMX1A,EFNA5,CDC14B,NGEF,TRPC5,NEUROD2,PTPRF,FXR2,ARHGEF28,GPRIN1,GRID2,ZNF423,RB1,NTNG1,KIRREL3,GFRA1,ETV1,LRTM2,KAT2B,ARHGEF7,BDNF,SEMA3E,TMEM237,CDH2,TANC1,USP9X,TENM1,AVIL,TTC39C,VAV2,IQCB1,CLASP2,SEMA6A,ITGA4,ROCK1,PLEK2,DGKG,PRKG1,ELAVL4,MAGI2,NIN,PRKD1,TMEM30A,SEMA5A,PARD3,ATP8B1,RNF6,SEMA3C,VPS13B,SPART*
GO:0030030	cell projection organization	0.000217444882069575 1	*APBB2,RAPGEF6,MAP3K13,ARL13B,CDC42EP3,CHRNB2,CDC14C,SEZ6,CAMK2B,GRIP1,KALRN,NOTCH2,EPB41L3,MAP6,PARVB,CDH11,SHANK3,STYXL1,DAAM2,PLXNA4,STK24,CFAP70,PRKN,IFT172,SERPINI1,NCAM2,TIAM2,CFAP54,LRP8,CNTN1,ARHGEF4,NCK1,NREP,PREX1,ZNF804A,BRAF,ABLIM1,CHRNA7,HECW1,HMGB1,DYNLT2B,LAMA2,TRIO,MUSK,ALKBH1,GOLPH3,EPHA5,SKOR2,ENAH,SNX10,CHL1,GSN,PLS1,SLIT3,ROBO1,DNAAF11,PAK3,NCKAP1,CILK1,EPHA3,LYN,ADGRB3,KLF7,DIP2B,ERCC6,EIF2AK4,MATN2,MARK4,CRTAC1,CUX1,GPM6B,ADGRV1,WHRN,APP,IGF2BP1,FRY,PALLD,CECR2,PTK2,LRRK2,CFAP298,PLD1,PKHD1,TIAM1,PHACTR1,NEDD4L,KIT,EPHA7,SPAG9,WNT5A,CTNNB1,LMX1A,DIAPH3,EFNA5,CDC14B,NGEF,TRPC5,NEUROD2,PTPRF,FXR2,ARHGEF28,GPRIN1,GRID2,ZNF423,RB1,NTNG1,KIRREL3,GFRA1,ETV1,LRTM2,KAT2B,ARHGEF7,NPHP1,BDNF,SEMA3E,SCIN,TMEM237,CDH2,TANC1,USP9X,TENM1,AVIL,TTC39C,VAV2,IQCB1,CLASP2,SEMA6A,ITGA4,ROCK1,PLEK2,DGKG,PRKG1,ELAVL4,MAGI2,NIN,PRKD1,TMEM30A,SEMA5A,PARD3,ATP8B1,RNF6,SEMA3C,VPS13B,SPART*
GO:0007165	signal transduction	0.000251847187129284 54	*FSTL1,APBB2,RGN,CDK14,EPN2,PDE1C,NOSTRIN,MAP3K3,OSBPL8,NFIA,RAPGEF6,ARHGEF9,TAFA2,ABR,MAOA,MAP3K13,PPM1A,ARL13B,LIMD1,TF,CHST11,THRB,SPTBN1,CDC42EP3,MAPK10,USP32,DAPP1,GNG12,FGF1,CHRNB2,SEZ6,ARHGAP15,GNB4,RBPMS,SKAP2,BRD4,DOK4,CAMK2B,GRIP1,ARHGAP11A-SCG5,TLK1,SLC38A9,HBP1,ABCG8,KALRN,MAP4K3,NOTCH2,TMOD2,NPNT,SHANK3,NR4A3,E2F7,FOXO1,STYXL1,STK39,SMURF2,ADAMTS12,DAAM2,PLXNA4,URB2,STK24,DIPK2A,DCDC1,CA*

			CNA1C,FAM3C,ASB4,PRKN,NLGN4X,PIK3C2B,FAM83B,IFT172,CDON,NLK,GNAI1,MAPKAPK3,TIAM2,LRP8,CNTN1,STAT5B,ARHGEF4,UBE2O,DDIAS,CPEB4,CALCR,CHRFAM7A,ELMO1,ERBIN,MBTPS2,RGS22,CD247,MCC,PTH,ADAR,PDE10A,RHOJ,CDC37,FGF10,ZFYVE16,MECOM,COL4A6,STK32B,HERC5,NCK1,NONO,NREP,GABRA5,KCNQ3,MTCP1,CHRM3,PREX1,CIDEB,BRAF,COL1A2,DTNA,ALPK2,CHRNA7,HECW1,HMGB1,ZNF304,CDIP1,HIVEP1,ATRX,TGFB2,MDFIC,LAMA2,TRIO,SMARCC1,NDST1,MUSK,KCNIP3,SFPQ,MAP3K5,RGS3,NUP93,RERG,OR5AU1,PRKAR2A,SLC15A4,GOLPH3,PRKG2,MBTPS1,PIK3C3,USP10,IGFBP7,EPHA5,SKOR2,ENSG00000289694,ATP2C1,ALDH1A2,PTGFR,PCLO,ASPH,RRAS2,UBR5,SOX30,ANKS1B,CHL1,TCP11L1,TLR8,SLIT3,ROBO1,ITPR1,PAK3,DOK6,PRKAG2,MLLT3,TNFRSF12A,OASL,TAS2R14,CXCR5,NCKAP1,CILK1,FOSL2,IL1RAPL2,ADGRD1,EPHA3,LATS2,ERP29,ARHGAP18,LYN,ADGRB3,GRIK1,LARGE1,FIGNL1,PPM1L,TMED7-TICAM2,NOS3,ERCC6,NOS1AP,EIF2AK4,RAB12,TGFBR2,CMKLR2,ITGA11,MARK4,TCF7L2,UNC119,SHISA2,RRAGD,ULK3,GPR156,GKAP1,PPP2R5E,ADGRV1,RASGRF2,SCG5,WAC,DAPK1,TDP2,INSYN2A,APP,GATA4,SMAD5,GSKIP,ADAM12,PELI2,CHRM5,PI4KB,RHOT1,PIK3R5,TLR1,TAS2R30,BMT2,AVPR1B,PTK2,USP7,TICAM2,SH2B2,PIP4K2B,LRRK2,ARIH1,PLD1,ITFG2,VTCN1,PKHD1,RARB,PJA2,TIAM1,CSRP3,IQSEC2,ACVR2A,GNAO1,TIPIN,RASSF4,BRCC3,SNX13,RABGEF1,KIT,EPHA7,SPAG9,SPDL1,WNT5A,TRABD2B,ARFGEF2,TEX2,CTNNB1,ANKRD17,CTSK,PRKDC,IPO5,ESR1,TNFRSF19,ITGB8,BCAR3,TPD52L1,WWOX,HUNK,NEK10,PTGER4,PDE7B,VWC2L,DOCK1,DIAPH3,GPC4,EFNA5,CDC14B,PLCH1,OR1M1,NGEF,NEUROD2,FNIP2,TNFSF11,PTPRF,IKBKB,SH3BP5,AKAP6,TRBV10-2,ARHGEF28,OR8B8,ATF2,CHRNB3,ACVR1,PID1,GRID2,ZNF423,RB1,IFNLR1,PDE4B,ADIPOR1,GABRB3,PLPP3,HNF1B,LAMP2,NCOA2,BNIP3L,PRKCH,DLC1,DENND2B,RGS17,GFRA1,INPP4A,BMX,GRIA4,RAB18,RGS7BP,MGRN1,MID2,SIK1,TP53BP2,WLS,ANKS1A,RYR3,PRCP,CDK5RAP2,ARHGEF7,SAMD12,ATF3,NPHP1,FBN2,SRI,ATP1B1,ARNT,BDNF,LDLRAD4,SEMA3E,MACO1,TLR6,TMEM237,KIF16B,RAB11FIP2,CDH2,LAG3,PPARG,PAPPA,ASB3,CHRNB4,USP9X,NEK7,TENM1,IL13RA2,RORB,CDK6,GABRB1,LTB4R2,DDX60,MYO5A,SIPA1L3,SFRP4,IL1RAP,JCAD,VAV2,TFRC,GRIA3,PNPLA8,FBN1,HAPSTR1,MAP3K1,CLEC6A,RAPGEF4,BMPER,ANGPT1,SEMA6A,VIPR2,PHLPP1,ITGA4,ROCK1,TAX1BP1,APIP,PML,MGLL,PLEK2,TNFSF4,AFAP1L2,DGKG,SP100,PRKG1,MAGI2,SPG21,ASIC2,AIMP1,SLC30A10,PRKD1,CALCRL,USP34,STK4,SPC25,PRLR,PTPRA,PDE11A,NMI,RAB6C,IL16,EDARADD,GNA14,CR2,LPAR6,CTBP2,TTN,NCALD,HHIP,DYRK1A,PPFIA1,PPP1R1C,CUL4A,KCTD16,EYA4,SEMA5A,PMEPA1,PEX5L,METTL3,KCNN4,RNF6,SEMA3C,VAV1,GRIA2,VDAC2,SPART

GO:0048013	ephrin receptor signaling pathway	0.000274389301319124 27	*KALRN,NCK1,EPHA5,ANKS1B,PAK3,EPHA3,LYN,PTK2,TIAM1,EPHA7,EFNA5,NGEF,ARHGEF28,ANKS1A,ARHGEF7*
GO:0030036	actin cytoskeleton organization	0.000310808262179347 1	*NOSTRIN,TF,SPTBN1,CDC42EP3,MYO5C,TMOD2,EPB41L3,PARVB,SHANK3,DAAM2,PRKN,THSD7A,TAGLN3,ELMO1,RHOJ,FGF10,NCK1,PREX1,MYO1D,BRAF,TMOD3,RFLNA,EPB41L4A,ADD3,JAM3,HMCN1,EPHA5,ENAH,ATP2C1,FAM171A1,GSN,PLS1,PAK3,NCKAP1,EPHA3,MYH14,ARHGAP18,MYOM2,SPTB,NOS1AP,MAGEL2,GPM6B,EPB41L5,RHOT1,PALLD,SH2B2,CSRP3,PHACTR1,IQSEC2,KIT,ANTXR1,PPM1E,PTGER4,DIAPH3,EFNA5,IKBKB,SHROOM4,DLC1,ACTR3C,MYO1B,NPHP1,CAPZA2,SCIN,TENM1,SH3KBP1,AVIL,MYO5A,SPTBN5,CLASP2,ROCK1,PLEK2,PRKG1,PRKD1,TTN,PPFIA1,SEMA5A,FMNL2*
GO:0003012	muscle system process	0.000473464541485867 96	*APBB2,RCSD1,CACNB1,CHRNB2,CAMK2B,TMOD2,NPNT,NR4A3,FOXO1,CACNA1C,CHRM3,DTNA,TMOD3,ASPH,GSN,MYH14,MYOM2,LARGE1,NOS3,NOS1AP,GATA4,SMAD5,CACNA2D1,TIAM1,CSRP3,MLIP,GNAO1,MEIS1,NEDD4L,KIT,AKAP6,CLCN1,KCNJ12,KCNJ3,PDE4B,SSPN,ACACB,KCND2,RYR3,MYL4,TRDN,SRI,ATP1B1,PPARG,ASB3,CHRNB4,MYOF,ROCK1,PRKG1,PRKD1,TNNI3K,TTN,KCNE2*
GO:0010975	regulation of neuron projection development	0.000565840773482061 3	*MAP3K13,CHRNB2,SEZ6,CAMK2B,KALRN,MAP6,SHANK3,STYXL1,PLXNA4,STK24,SERPINI1,TIAM2,LRP8,CNTN1,NCK1,PREX1,ZNF804A,BRAF,HECW1,MUSK,SKOR2,ROBO1,PAK3,EPHA3,LYN,ADGRB3,DIP2B,CUX1,LRRK2,TIAM1,NEDD4L,EPHA7,WNT5A,EFNA5,NGEF,TRPC5,PTPRF,GRID2,NTNG1,KAT2B,BDNF,SEMA3E,CDH2,AVIL,SEMA6A,DGKG,ELAVL4,MAGI2,NIN,PRKD1,TMEM30A,SEMA5A,RNF6,SEMA3C,SPART*
GO:0036211	protein modification process	0.000713320679350009 2	*C10ORF90,STYK1,RGN,CDK14,MAP3K3,SMYD3,ABR,MAP3K13,PPM1A,SAMSN1,USP42,MAPK10,USP32,DAPP1,FGF1,CDC14C,AGTPBP1,PPM1H,CAMK2B,TLK1,OTUD7A,KALRN,SUMF1,MAP4K3,XXYLT1,FPGT-TNNI3K,GALNT9,FOXO1,STYXL1,STK39,SMURF2,CDK18,STK24,DIPK2A,ASB4,PRKN,RMND5A,F13A1,MARCHF9,CBLL2,CDON,NLK,MAPKAPK3,LRP8,UBE2O,ADAR,CDC37,FGF10,STK32B,HERC5,UBE2E2,NCK1,KLHL42,MTCP1,CHRM3,NCOA7,MARCHF1,BRAF,EEF2KMT,ALPK2,CHRNA7,HECW1,TGFB2,TRIO,MUSK,MAP3K5,PRKAR2A,PRKG2,PIK3C3,USP10,RSKR,EPHA5,PTPN20,PPTC7,STRADA,HUWE1,ASPH,UBR5,MAPRE3,TLR8,CACUL1,ROBO1,PAK3,NDUFAF7,CAMK1G,PRKAG2,CCNT2,CILK1,EPHA3,LATS2,SPDYE2B,ERP29,UVRAG,PTPRN2,LYN,USP6,SUMO2,DIP2B,LARGE1,RNASEL,PPM1L,ERCC6,NOS1AP,EIF2AK4,MSRA,MAGEL2,TGFBR2,MARK4,UNC119,ULK3,SPDYE2,DAPK1,APP,PPIL6,GSKIP,RNF38,UBE2G1,PELI2,FRY,HERC2,PIK3R5,RIPK4,PTK2,USP7,LRRK2,ARIH1,PJA2,COP1,ACVR2A,NEDD4L,BRCC3,DUSP14,PRMT8,RABGEF1,KIT,EPHA7,SPAG9,WNT5A,TRABD2B,CTNNB1,PRKDC,IPO5,PTPN14,POFUT2,BCAR3,TPD52L1,PPM1E,TPS*

			T2,HUNK,NEK10,ANAPC10,USP40,PKIG,EFNA5,CDC14B,HECTD1,VKORC1L1,TRPC5,NEUROD2,FNIP2,KAT14,UGGT2,UBE3B,TNFSF11,PTPRF,IKBKB,FXR2,RAB3GAP2,CEMIP,ATF2,HDAC8,ACVR1,PID1,RB1,NSMCE1,PLPP3,SPSB1,PRKCH,XRCC5,GFRA1,BMX,TPST1,TMTC1,NRK,MGRN1,MID2,SIK1,KAT2B,TMTC2,UBE2E3,ARNT,BDNF,TLR6,PPARG,ASB3,USP9X,TGM5,NEK7,TENM1,CDK6,MYLK4,USP25,SPDYE16,TFRC,SPDYE6,MAP3K1,CLEC6A,FUT10,ANGPT1,EIF4G1,B3GAT2,ROCK1,PML,PRKG1,RFPL4B,HSPBP1,PRKD1,USP34,STK4,TNNI3K,ST6GAL2,PRLR,PTPRA,NMI,TTN,DYRK1A,CUL4A,UBE3C,ATE1,PARD3,RNF6,DPY19L1
GO:0009966	regulation of signal transduction	0.000984125476949514 6	FSTL1,RGN,CDK14,EPN2,MAP3K3,OSBPL8,ARHGEF9,ABR,MAOA,MAP3K13,PPM1A,LIMD1,CHST11,THRB,SPTBN1,USP32,FGF1,SEZ6,ARHGAP15,BRD4,DOK4,SLC38A9,KALRN,NOTCH2,TMOD2,NPNT,SHANK3,FOXO1,STYXL1,STK39,SMURF2,ADAMTS12,DAAM2,URB2,DIPK2A,PRKN,NLGN4X,FAM83B,IFT172,CDON,NLK,GNAI1,TIAM2,ARHGEF4,UBE2O,DDIAS,CALCR,ERBIN,RGS22,MCC,PTH,ADAR,PDE10A,CDC37,FGF10,MECOM,NCK1,NONO,NREP,PREX1,BRAF,ALPK2,CHRNA7,HECW1,HMGB1,TGFB2,MDFIC,LAMA2,TRIO,NDST1,MUSK,SFPQ,MAP3K5,RGS3,NUP93,SLC15A4,GOLPH3,USP10,IGFBP7,SKOR2,ATP2C1,UBR5,SOX30,SLIT3,ROBO1,ITPR1,PAK3,DOK6,MLLT3,TNFRSF12A,OASL,LATS2,ERP29,ARHGAP18,LYN,FIGNL1,NOS3,ERCC6,NOS1AP,TGFBR2,MARK4,TCF7L2,SHISA2,RRAGD,ULK3,GKAP1,ADGRV1,RASGRF2,WAC,DAPK1,APP,GATA4,SMAD5,GSKIP,PELI2,PIK3R5,TLR1,BMT2,AVPR1B,PTK2,USP7,TICAM2,SH2B2,PIP4K2B,LRRK2,ITFG2,PKHD1,PJA2,TIAM1,IQSEC2,ACVR2A,BRCC3,SNX13,RABGEF1,KIT,EPHA7,SPAG9,WNT5A,TRABD2B,ARFGEF2,CTNNB1,ANKRD17,CTSK,PRKDC,ESR1,TNFRSF19,BCAR3,TPD52L1,WWOX,NEK10,VWC2L,GPC4,NGEF,NEUROD2,FNIP2,TNFSF11,IKBKB,AKAP6,ARHGEF28,ACVR1,PID1,ZNF423,RB1,PDE4B,ADIPOR1,PLPP3,HNF1B,LAMP2,PRKCH,DLC1,DENND2B,RGS17,RGS7BP,MGRN1,MID2,WLS,PRCP,CDK5RAP2,ATF3,FBN2,ARNT,BDNF,LDLRAD4,SEMA3E,TLR6,TMEM237,CDH2,PPARG,USP9X,NEK7,TENM1,DDX60,SIPA1L3,SFRP4,JCAD,VAV2,TFRC,FBN1,HAPSTR1,CLEC6A,BMPER,ANGPT1,SEMA6A,PHLPP1,ROCK1,TAX1BP1,APIP,PML,MGLL,AFAP1L2,DGKG,SP100,MAGI2,SLC30A10,PRKD1,USP34,STK4,PRLR,PDE11A,NMI,LPAR6,CTBP2,HHIP,DYRK1A,CUL4A,KCTD16,EYA4,SEMA5A,PMEPA1,PEX5L,METTL3,KCNN4,RNF6,VAV1,VDAC2,SPART
GO:0007416	synapse assembly	0.001397780673866404 7	LRRN1,ARHGEF9,CHRNB2,SDK2,SHANK3,NLGN4X,MUSK,PCLO,IL1RAPL2,ADGRB3,LARGE1,PCDHB16,APP,CLSTN2,EPHA7,WNT5A,CTNNB1,GPC4,EFNA5,LINGO2,GRID2,GABRB3,KIRREL3,GABRG2,LRTM2,BDNF,CDH2,IL1RAP,EIF4G1,CDH9,ASIC2
GO:0043412	macromolecule modificatio	0.001535755213783764	C10ORF90,STYK1,RGN,CDK14,MAP3K3,SMYD3,ABR,MAP3K13,PPM1A,SAMSN1,THADA,USP42,MAPK10,USP32,DAPP1,FGF1,CDC14C,AGTPBP1,

	n		*TRMT6,PPM1H,CAMK2B,TLK1,OTUD7A,KALRN,SUMF1,MAP4K3,XXYLT1,FPGT-TNNI3K,GALNT9,FOXO1,STYXL1,STK39,SMURF2,CDK18,STK24,DIPK2A,ASB4,PRKN,RMND5A,F13A1,MARCHF9,CBLL2,CDON,NLK,MAPKAPK3,LRP8,UBE2O,ADAR,CDC37,FGF10,STK32B,HERC5,UBE2E2,NCK1,KLHL42,MTCP1,CHRM3,TYW1,NCOA7,MARCHF1,BRAF,EEF2KMT,ALPK2,CHRNA7,HECW1,TGFB2,TRIO,MUSK,MAP3K5,ALKBH1,ZCCHC4,PRKAR2A,PRKG2,PIK3C3,USP10,RSKR,EPHA5,PTPN20,PPTC7,STRADA,HUWE1,ASPH,UBR5,MAPRE3,TLR8,CACUL1,ROBO1,PAK3,NDUFAF7,CAMK1G,PRKAG2,CCNT2,CILK1,EPHA3,LATS2,TYW3,SPDYE2B,ERP29,UVRAG,PTPRN2,LYN,USP6,SUMO2,DIP2B,LARGE1,PUS7,RNASEL,PPM1L,ERCC6,NOS1AP,EIF2AK4,MSRA,MAGEL2,TGFBR2,MARK4,UNC119,ULK3,SPDYE2,QTRT2,DAPK1,APP,PPIL6,GSKIP,RNF38,UBE2G1,PELI2,FRY,HERC2,PIK3R5,RIPK4,PTK2,USP7,LRRK2,ARIH1,PJA2,COP1,ACVR2A,NEDD4L,BRCC3,DUSP14,PRMT8,RABGEF1,KIT,EPHA7,SPAG9,WNT5A,TRABD2B,CTNNB1,PRKDC,IPO5,PTPN14,POFUT2,BCAR3,TPD52L1,PPM1E,TPST2,HUNK,NEK10,ANAPC10,USP40,PKIG,EFNA5,CDC14B,HECTD1,VKORC1L1,TRPC5,NEUROD2,FNIP2,KAT14,UGGT2,UBE3B,TNFSF11,PTPRF,IKBKB,FXR2,RAB3GAP2,CEMIP,ATF2,HDAC8,ACVR1,PID1,RB1,NSMCE1,PLPP3,SPSB1,PRKCH,XRCC5,GFRA1,BMX,TPST1,TMTC1,NRK,MGRN1,MID2,SIK1,KAT2B,TMTC2,UBE2E3,ADAT2,ARNT,BDNF,TLR6,PPARG,ASB3,USP9X,TGM5,NEK7,TENM1,CDK6,MYLK4,USP25,SPDYE16,TFRC,SPDYE6,MAP3K1,CLEC6A,FUT10,ANGPT1,EIF4G1,B3GAT2,ROCK1,PML,PRKG1,RFPL4B,HSPBP1,PRKD1,USP34,STK4,TNNI3K,ST6GAL2,PRLR,PTPRA,NMI,TTN,DYRK1A,CUL4A,UBE3C,ATE1,METTL3,PARD3,RNF6,DPY19L1*
GO:0010604	positive regulation of macromolecule metabolic process	0.001719704565823803	*APBB2,RGN,ING3,NFIA,PHF20,FOXK2,SMYD3,MAP3K13,PPM1A,TF,THRB,SPTBN1,ATF7,FGF1,AGTPBP1,RBPMS,GPBP1L1,BRD4,NOTCH2,HMBOX1,NPNT,NR4A3,ARID1B,E2F7,FOXO1,FER1L6,YBX1,DIPK2A,IDO1,PRKN,KDM7A,CHD7,CDON,LRP8,CNTN1,MEOX2,STAT5B,EIF4ENIF1,CALCR,MBTPS2,PTH,ADAR,TOX2,FGF10,MECOM,NCK1,CD84,FAM168A,MTCP1,RSF1,NCOA7,ZNF804A,BRAF,MRTFB,CHRNA7,HMGB1,ZNF304,HIVEP1,ATRX,TGFB2,MDFIC,HOXC13,SMARCC1,MUSK,SFPQ,MAP3K5,ZCCHC4,CTIF,RBM23,RUNX1,ALDH1A2,PTGFR,STRADA,HUWE1,ASPH,SH3BGRL,UBR5,GTF2F2,SOX30,GSN,SPON1,MAPRE3,TLR8,CACUL1,ROBO1,KLF12,PRKAG2,MLLT3,CCNT2,CNOT10,FOSL2,SPDYE2B,ERP29,LYN,KLF7,SUMO2,MTA3,DIP2B,LDB2,MYRFL,RNASEL,DBF4B,DCP2,NOS3,TNRC6C,MLLT10,ERCC6,NOS1AP,EIF2AK4,TGFBR2,TCF7L2,ZNF462,ZFHX3,UNC119,SPDYE2,GSDMD,WAC,WHRN,APP,GATA4,SMAD5,IGF2BP1,PELI2,PIK3R5,TLR1,PTK2,TICAM2,CELF1,POGZ,LRRK2,INO80D,ZNF395,IKZF3,ARIH1,PLD1,VTCN1,TENT5C,RARB,TCF4,PAEP,COP1,CSRP3,MLIP,ACVR2A,MEIS1,NEDD4L,BRCC3,KIT,EPHA7,WNT5A,TRABD2B,A*

			RFGEF2,CTNNB1,PRKDC,ESR1,ITGB8,BCAR3,TPD52L1,ZXDC,LMX1A,WWOX,NEK10,PTGER4,EFNA5,IGF2BP2,CDC14B,HECTD1,TRPC5,NEUROD2,FNIP2,TNFSF11,IKBKB,FXR2,USF3,RAB3GAP2,CCPG1,CEMIP,ATF2,ACVR1,PID1,EBF2,ZNF423,RB1,PDE4B,ZNF143,PLPP3,HNF1B,ZNF615,NCOA2,XRCC5,GFRA1,SHLD2,SLC24A3,TAF15,POU2F1,ETV1,MID2,ZNF850,KAT2B,SUPV3L1,MEF2D,GTF2A1,CDK5RAP2,RFC3,ATF3,ARNT,BDNF,TFCP2L1,BCLAF3,TENT4B,TLR6,PPARG,NEK7,TENM1,RORB,CDK6,TFAP2D,SFRP4,SPDYE16,IL1RAP,TFRC,SPDYE6,CLEC6A,ZNF292,ZNF521,ANGPT1,EIF4G1,ROCK1,NUP98,PML,TNFSF4,AFAP1L2,SP100,ELAVL4,HSPBP1,PRKD1,STK4,PRLR,NMI,IL16,CTBP2,TTN,DYRK1A,KCNE2,CUL4A,EYA4,ELL2,PVT1,GATAD2B,METTL3,UAP1,ZNF33A,NPAS3,BMAL2,RNF6,FANCB
GO:0022604	regulation of cell morphogenesis	0.001746629301042089	*MAP3K13,LIMD1,CDC42EP3,ARHGAP15,ATP10A,CAMK2B,GRIP1,KALRN,EPB41L3,PARVB,PLXNA4,PRKN,LRP8,RHOJ,FAM171A1,PRPF40A,MYH14,ARHGAP18,PDZD8,CUX1,PTK2,TIAM1,NEDD4L,KIT,SPAG9,WNT5A,PRKDC,EFNA5,SYNE3,NTNG1,DLC1,ARHGEF7,SEMA3E,SH3KBP1,FMNL2*
GO:1902531	regulation of intracellular signal transduction	0.0018078170841916814	*RGN,MAP3K3,OSBPL8,ARHGEF9,ABR,MAP3K13,PPM1A,LIMD1,THRB,SPTBN1,USP32,FGF1,SEZ6,ARHGAP15,BRD4,DOK4,SLC38A9,KALRN,NOTCH2,NPNT,FOXO1,STYXL1,STK39,URB2,DIPK2A,PRKN,CDON,NLK,GNAI1,TIAM2,ARHGEF4,DDIAS,CALCR,ERBIN,PDE10A,FGF10,MECOM,NCK1,NONO,PREX1,BRAF,CHRNA7,HMGB1,TGFB2,MDFIC,TRIO,NDST1,MUSK,SFPQ,MAP3K5,NUP93,SLC15A4,GOLPH3,USP10,ATP2C1,ROBO1,ITPR1,PAK3,DOK6,OASL,ERP29,ARHGAP18,LYN,FIGNL1,NOS1AP,TGFBR2,MARK4,TCF7L2,RRAGD,ADGRV1,RASGRF2,WAC,APP,GATA4,PELI2,PIK3R5,BMT2,AVPR1B,PTK2,USP7,TICAM2,SH2B2,LRRK2,ITFG2,PKHD1,PJA2,TIAM1,IQSEC2,ACVR2A,BRCC3,RABGEF1,KIT,EPHA7,SPAG9,WNT5A,ARFGEF2,CTNNB1,ANKRD17,PRKDC,ESR1,TNFRSF19,BCAR3,TPD52L1,NEK10,NGEF,NEUROD2,FNIP2,TNFSF11,IKBKB,AKAP6,ARHGEF28,ACVR1,ADIPOR1,LAMP2,DLC1,DENND2B,MGRN1,MID2,WLS,PRCP,CDK5RAP2,ATF3,LDLRAD4,SEMA3E,TLR6,CDH2,PPARG,USP9X,NEK7,TENM1,DDX60,SIPA1L3,JCAD,VAV2,TFRC,HAPSTR1,CLEC6A,BMPER,ANGPT1,SEMA6A,PHLPP1,ROCK1,TAX1BP1,APIP,PML,DGKG,MAGI2,SLC30A10,PRKD1,STK4,PDE11A,NMI,LPAR6,CTBP2,DYRK1A,CUL4A,SEMA5A,PMEPA1,PEX5L,RNF6,VAV1,VDAC2*
GO:0051716	cellular response to stimulus	0.001935782301161461	*FSTL1,APBB2,RGN,CDK14,EPN2,PDE1C,NOSTRIN,ING3,MAP3K3,OSBPL8,NFIA,CPNE4,SMYD3,RAPGEF6,ARHGEF9,TAFA2,ABR,MAOA,MAP3K13,PPM1A,ARL13B,LIMD1,TF,CHST11,THRB,RCSD1,SPTBN1,CDC42EP3,MAPK10,CACNB1,USP32,DAPP1,GNG12,FGF1,CHRNB2,SEZ6,ARHGAP15,GNB4,RBPMS,SKAP2,BRD4,DOK4,CAMK2B,GRIP1,ARHGAP11A-SCG5,TLK1,DDI2,SLC38A9,HBP1,ABCG8,KALRN,MAP4K3,NOTCH2,TMOD2,NPNT,SHANK3,NR4A*

			3,ARID1B,E2F7,FOXO1,STYXL1,STK39,SMURF2,ADAMTS12,DAAM2,PLXNA4,URB2,YBX1,STK24,DIPK2A,ARMT1,DCDC1,CACNA1C,FAM3C,ASB4,PRKN,NLGN4X,PNPLA3,PIK3C2B,FAM83B,IFT172,CDON,NLK,GNAI1,MAPKAPK3,TIAM2,LRP8,CNTN1,STAT5B,ARHGEF4,UBE2O,DDIAS,CPEB4,CALCR,CHRFAM7A,ELMO1,ERBIN,MBTPS2,RGS22,CD247,MCC,PTH,ADAR,PDE10A,RHOJ,CDC37,FGF10,ZFYVE16,MECOM,COL4A6,STK32B,HERC5,UBE2E2,NCK1,FAM168A,NONO,NREP,EXO1,GABRA5,KCNQ3,MTCP1,CHRM3,PREX1,PMS1,WRNIP1,NCOA7,CIDEB,BRAF,COL1A2,DTNA,ALPK2,CHRNA7,HECW1,HMGB1,ZNF304,CDIP1,HIVEP1,ATRX,TGFB2,MDFIC,LAMA2,TRIO,SMARCC1,RAD51B,NDST1,MUSK,JAM3,KCNIP3,SFPQ,MAP3K5,ALKBH1,ARPP21,RGS3,NUP93,RERG,OR5AU1,PRKAR2A,SLC15A4,GOLPH3,PRKG2,MBTPS1,PIK3C3,USP10,IGFBP7,EPHA5,SKOR2,ENSG00000289694,ATP2C1,ALDH1A2,PTGFR,PCLO,HUWE1,ASPH,RRAS2,UBR5,ABTB2,SOX30,ANKS1B,CHL1,TCP11L1,TLR8,SLIT3,NLRP7,ROBO1,ITPR1,PAK3,CYP2A13,DOK6,PRKAG2,MLLT3,TNFRSF12A,OASL,TAS2R14,CXCR5,NCKAP1,CILK1,FOSL2,PACRG,IL1RAPL2,ADGRD1,EPHA3,LATS2,ERP29,ARHGAP18,UVRAG,PTPRN2,LYN,ADGRB3,KLF7,GRIK1,LARGE1,FIGNL1,ALDH1A1,FMO5,PPM1L,TMED7-TICAM2,NOS3,BPHL,ERCC6,NOS1AP,EIF2AK4,MSRA,RAB12,MATN2,TGFBR2,CMKLR2,ITGA11,MARK4,TCF7L2,CHAF1B,UNC119,SHISA2,RRAGD,ULK3,GPR156,GKAP1,PPP2R5E,ADGRV1,RASGRF2,SCG5,WAC,DAPK1,TDP2,ACSM2B,INSYN2A,APP,GATA4,SMAD5,IGF2BP1,GSKIP,ADAM12,PELI2,CACNA2D1,HCN1,CHRM5,PI4KB,RHOT1,HERC2,PIK3R5,TLR1,TAS2R30,BMT2,AVPR1B,PTK2,USP7,TICAM2,SH2B2,POGZ,PIP4K2B,LRRK2,INO80D,ARIH1,PLD1,ITFG2,VTCN1,PKHD1,RARB,PJA2,MGST1,TIAM1,CSRP3,IQSEC2,ACVR2A,GNAO1,TIPIN,RASSF4,BRCC3,SNX13,RABGEF1,KIT,EPHA7,SPAG9,SPDL1,WNT5A,TRABD2B,WIPI2,ARFGEF2,TEX2,CTNNB1,ANKRD17,CTSK,PRKDC,IPO5,ESR1,TNFRSF19,ITGB8,BCAR3,TPD52L1,PPM1E,WWOX,HUNK,NEK10,PTGER4,PDE7B,VWC2L,DOCK1,DIAPH3,GPC4,EFNA5,CDC14B,PLCH1,OR1M1,VKORC1L1,NGEF,NEUROD2,FNIP2,JAML,TNFSF11,PTPRF,IKBKB,SH3BP5,AKAP6,TRBV10-2,ARHGEF28,OR8B8,ATF2,CHRNB3,ACVR1,PID1,GRID2,ZNF423,RB1,NSMCE1,MPV17,IFNLR1,PDE4B,PKD1L3,ADIPOR1,GABRB3,PLPP3,HNF1B,IMMP2L,LAMP2,GABRG2,CPS1,NCOA2,BNIP3L,PRKCH,DLC1,DENND2B,XRCC5,RGS17,GFRA1,SHLD2,KCND2,INPP4A,BMX,GRIA4,POLD3,RAB18,RGS7BP,MGRN1,MID2,SIK1,ERCC6L2,TP53BP2,KAT2B,WLS,ANKS1A,RYR3,PRCP,CDK5RAP2,RFC3,ARHGEF7,SAMD12,ATF3,NPHP1,FBN2,SRI,ATP1B1,ARNT,BDNF,LDLRAD4,SEMA3E,MACO1,TLR6,TMEM237,KIF16B,RAB11FIP2,CDH2,LAG3,PPARG,PAPPA,ASB3,CHRNB4,USP9X,NEK7,TENM1,IL13RA2,RORB,CDK6,GABRB1,LTB4R2,SMOX,GDAP1,DDX60,USP25,MYO5A,SIPA

			1L3,SFRP4,IL1RAP,JCAD,VAV2,TFRC,GRIA3,PNPLA8,FBN1,HAPSTR1,RAB31,MAP3K1,CLEC6A,RAPGEF4,BMPER,ANGPT1,SEMA6A,VIPR2,EIF4G1,PHLPP1,ITGA4,ROCK1,TAX1BP1,TBC1D4,APIP,MMP28,PML,MGLL,SCARA5,PLEK2,TNFSF4,AFAP1L2,DGKG,SP100,PRKG1,ELAVL4,NDUFAF2,UMODL1,MAGI2,SPG21,ASIC2,AIMP1,SLC30A10,PRKD1,CALCRL,USP34,STK4,SPC25,PRLR,PTPRA,PDE11A,NMI,RAB6C,IL16,ZSWIM7,EDARADD,GNA14,CR2,LPAR6,CTBP2,TTN,NCALD,HHIP,DYRK1A,PPFIA1,PPP1R1C,KCNE2,CUL4A,KCTD16,TXNRD3,EYA4,SEMA5A,PMEPA1,PEX5L,METTL3,KCNN4,CP,RNF6,SEMA3C,VAV1,FANCB,GRIA2,VDAC2,SPART
GO:0048468	cell development	0.001955893251562712 3	*APBB2,SMYD3,MAP3K13,LIMD1,TF,CHST11,THRB,CHRNB2,SEZ6,AGTPBP1,SH3PXD2A,SPINK5,BRD4,CAMK2B,GRIP1,DCT,KALRN,NOTCH2,TMOD2,EPB41L3,MAP6,CDH11,SHANK3,ARID1B,STYXL1,DAAM2,PLXNA4,STK24,ARB2A,PRKN,CHD7,PTBP3,CDON,SERPINI1,NCAM2,TIAM2,CFAP54,LRP8,CNTN1,STAT5B,HEATR9,CALCR,ADAR,MECOM,NCK1,NREP,GABRA5,KCNQ3,PREX1,COL11A1,ZNF804A,BRAF,TMOD3,ALPK2,CHRNA7,HECW1,HMGB1,RFLNA,ATRX,TGFB2,LAMA2,TRIO,SMARCC1,MUSK,JAM3,ALKBH1,EPHA5,SKOR2,RUNX1,ENAH,SNX10,ALDH1A2,SOX30,CHL1,PLS1,SLIT3,ROBO1,PAK3,AXDND1,MLLT3,MR1,NCKAP1,FOSL2,PACRG,EPHA3,MYOM2,LYN,ADGRB3,KLF7,DIP2B,LARGE1,PUS7,PAQR5,VSX1,ERCC6,EIF2AK4,MATN2,TGFBR2,CRTAC1,CUX1,GPM6B,ADGRV1,EPB41L5,WHRN,OCA2,TDP2,PRUNE1,APP,GATA4,SMAD5,IGF2BP1,HCN1,FRY,HOOK3,PALLD,CECR2,PTK2,CELF1,LRRK2,IKZF3,ITFG2,PKHD1,RARB,PAEP,TIAM1,CSRP3,PHACTR1,ACVR2A,NTM,MEIS1,NEDD4L,CYB5R4,KIT,EPHA7,SPAG9,WNT5A,CTNNB1,CTSK,PRKDC,ESR1,ITGB8,LMX1A,PTGER4,DOCK1,DIAPH3,BPGM,EFNA5,NGEF,TRPC5,NEUROD2,TNFSF11,PTPRF,IKBKB,AKAP6,ARHGEF28,GPRIN1,OPCML,ATF2,ACVR1,GRID2,RB1,NTNG1,KIRREL3,PRKCH,AGPAT5,XRCC5,GFRA1,ETV1,TEX11,LRTM2,KAT2B,ANKS1A,FLVCR1,ARHGEF7,IKZF1,ARNT,BDNF,TFCP2L1,SEMA3E,SCIN,CDH2,LAG3,PPARG,USP9X,TENM1,RORB,CDK6,GABRB1,AVIL,SIPA1L3,TFRC,FBN1,FUT10,CLASP2,ANGPT1,SEMA6A,ITGA4,ROCK1,PML,TNFSF4,DGKG,PRKG1,ELAVL4,MAGI2,NIN,FNDC3A,PRKD1,STK4,CR2,TTN,TMEM30A,CUL4A,SEMA5A,TTPA,METTL3,PARD3,ATP8B1,RNF6,SEMA3C,VAV1,VPS13B,SPART*
GO:0061564	axon development	0.002104271167151928 6	*APBB2,MAP3K13,CHRNB2,KALRN,NOTCH2,MAP6,CDH11,PLXNA4,STK24,NCAM2,TIAM2,CNTN1,NREP,BRAF,LAMA2,TRIO,MUSK,EPHA5,ENAH,CHL1,SLIT3,ROBO1,PAK3,EPHA3,KLF7,DIP2B,MATN2,CRTAC1,GPM6B,APP,PALLD,PTK2,TIAM1,KIT,EPHA7,WNT5A,LMX1A,EFNA5,TRPC5,NEUROD2,PTPRF,ARHGEF28,NTNG1,ETV1,LRTM2,BDNF,SEMA3E,CDH2,USP9X,CLASP2,SEMA6A,ITGA4,PRKG1,NIN,SEMA5A,PARD3,RNF6,SEMA3C,SPART*
GO:0050	regulation	0.00256925	*LRRN1,CHRNB2,CDH8,CAMK2B,KALRN,SHANK3,*

803	of synapse structure or activity	5713848084 3	*SLC17A6,LRP8,ZNF804A,MUSK,PAK3,IL1RAPL 2,ADGRB3,APP,LRRK2,CLSTN2,TIAM1,EPHA7, WNT5A,CTNNB1,GPC4,EFNA5,NGEF,NEUROD2,L INGO2,GRID2,LRTM2,BDNF,CDH2,TANC1,LRFN 2,IL1RAP,EIF4G1,ROCK1,ASIC2*
GO:0007 010	cytoskeleto n organizatio n	0.00299574 5919428202 6	*C10ORF90,NOSTRIN,NINL,LIMD1,TF,SPTBN1, CDC42EP3,MYO5C,CDC14C,TMOD2,EPB41L3,MA P6,PARVB,SHANK3,DAAM2,PRKN,IFT172,GPSM 1,THSD7A,GNAI1,TAGLN3,ELMO1,ERBIN,CEP1 92,RHOJ,FGF10,NCK1,PREX1,MCPH1,MYO1D,B RAF,TMOD3,ABLIM1,RFLNA,ATRX,EPB41L4A,A DD3,JAM3,HMCN1,EPHA5,ENAH,ATP2C1,FAM17 1A1,PCLO,GSN,PLS1,MAPRE3,DNAAF11,PAK3, NCKAP1,PRPF40A,EPHA3,MYH14,ARHGAP18,UV RAG,MYOM2,NME7,LARGE1,FIGNL1,PDZD8,SPT B,NAV1,NOS1AP,KIAA1614,MAGEL2,MARK4,GP M6B,KPNB1,EPB41L5,PRUNE1,RHOT1,HOOK3,P ALLD,CECR2,PTK2,SH2B2,PKHD1,CSRP3,PHAC TR1,IQSEC2,KIT,ANTXR1,SPDL1,CTNNB1,PPM 1E,FIGN,PTGER4,DIAPH3,EFNA5,CDC14B,SYN E3,IKBKB,ARHGEF28,ATF2,SHROOM4,DLC1,KA T2B,ACTR3C,MYO1B,CDK5RAP2,ARHGEF7,TRDN ,NPHP1,CAPZA2,SCIN,HAUS6,NEK7,TENM1,SH 3KBP1,AVIL,KIF2C,MYO5A,SIPA1L3,SPTBN5, CLASP2,CLTC,SEMA6A,ROCK1,PLEK2,PRKG1,N IN,PRKD1,SPC25,RAB6C,TTN,DYRK1A,PPFIA1 ,MAP7D3,SEMA5A,FMNL2,KRT85,PARD3*
GO:0050 807	regulation of synapse organizatio n	0.00368859 8654679515 4	*LRRN1,CHRNB2,CDH8,CAMK2B,KALRN,SHANK3, LRP8,ZNF804A,MUSK,PAK3,IL1RAPL2,ADGRB3 ,APP,LRRK2,CLSTN2,TIAM1,EPHA7,WNT5A,CT NNB1,GPC4,EFNA5,NGEF,NEUROD2,LINGO2,GR ID2,LRTM2,BDNF,CDH2,TANC1,LRFN2,IL1RAP ,EIF4G1,ROCK1,ASIC2*
GO:0006 936	muscle contraction	0.00376476 6727637869 8	*APBB2,RCSD1,CACNB1,CHRNB2,TMOD2,NPNT,C ACNA1C,CHRM3,DTNA,TMOD3,ASPH,GSN,MYH14 ,MYOM2,LARGE1,NOS1AP,GATA4,SMAD5,CACNA 2D1,CSRP3,GNAO1,NEDD4L,KIT,CLCN1,KCNJ1 2,KCNJ3,PDE4B,SSPN,KCND2,RYR3,MYL4,TRD N,SRI,ATP1B1,ASB3,CHRNB4,MYOF,ROCK1,PR KG1,PRKD1,TNNI3K,TTN,KCNE2*
GO:0120 035	regulation of plasma membrane bounded cell projection organizatio n	0.00515246 5716265877 5	*MAP3K13,CDC42EP3,CHRNB2,SEZ6,CAMK2B,KA LRN,MAP6,SHANK3,STYXL1,DAAM2,PLXNA4,ST K24,SERPINI1,TIAM2,LRP8,CNTN1,NCK1,PRE X1,ZNF804A,BRAF,HECW1,DYNLT2B,MUSK,SKO R2,PLS1,ROBO1,PAK3,NCKAP1,EPHA3,LYN,AD GRB3,DIP2B,MARK4,CUX1,LRRK2,PLD1,TIAM1 ,NEDD4L,KIT,EPHA7,WNT5A,EFNA5,NGEF,TRP C5,PTPRF,FXR2,GRID2,NTNG1,KAT2B,ARHGEF 7,BDNF,SEMA3E,CDH2,TENM1,AVIL,SEMA6A,P LEK2,DGKG,ELAVL4,MAGI2,NIN,PRKD1,TMEM3 0A,SEMA5A,ATP8B1,RNF6,SEMA3C,SPART*
GO:0031 344	regulation of cell projection organizatio n	0.00606412 6829420023 5	*MAP3K13,CDC42EP3,CHRNB2,SEZ6,CAMK2B,GR IP1,KALRN,MAP6,SHANK3,STYXL1,DAAM2,PLX NA4,STK24,SERPINI1,TIAM2,LRP8,CNTN1,NC K1,PREX1,ZNF804A,BRAF,HECW1,DYNLT2B,MU SK,SKOR2,PLS1,ROBO1,PAK3,NCKAP1,EPHA3, LYN,ADGRB3,DIP2B,MARK4,CUX1,LRRK2,PLD1 ,TIAM1,NEDD4L,KIT,EPHA7,WNT5A,EFNA5,NG EF,TRPC5,PTPRF,FXR2,GRID2,NTNG1,KAT2B, ARHGEF7,BDNF,SEMA3E,CDH2,TENM1,AVIL,SE*

			MA6A,PLEK2,DGKG,ELAVL4,MAGI2,NIN,PRKD1,TMEM30A,SEMA5A,ATP8B1,RNF6,SEMA3C,SPART
GO:0048513	animal organ development	0.006200154340177743	*RTN1,HS3ST3A1,RGN,NFIA,TRAPPC9,ARL13B,CHST11,THRB,FGF1,CHRNB2,SEZ6,GNB4,AGTPBP1,FHL1,SPINK5,ATP7B,DCT,KALRN,USH2A,NOTCH2,SDK2,NPNT,CDH11,SHANK3,NR4A3,E2F7,ADAMTS12,PLXNA4,DIPK2A,CACNA1C,ARB2A,NLGN4X,EXOC4,KDM7A,IFT172,CHD7,CDON,SLC17A6,CNMD,LRP8,CNTN1,MEOX2,STAT5B,ENPEP,MBTPS2,PTH,RHOJ,FGF10,PLG,ILDR2,GABRA5,KDM2B,COL11A1,MCPH1,BRAF,COL1A2,MRTFB,ABLIM1,ALPK2,RFLNA,ATRX,TGFB2,LAMA2,HOXC13,SMARCC1,NDST1,ALKBH1,PRKG2,EPHA5,SKOR2,RUNX1,SNX10,ALDH1A2,SIM1,PLS1,SLIT3,ROBO1,ANKRD11,DNAAF11,ABI3BP,CCNT2,CXCR5,FOSL2,EPHA3,LATS2,MYH14,MYOM2,KLF7,LARGE1,CA10,CRYBB1,LDB2,SGMS2,NOS3,VSX1,TGFBR2,ITGA11,TCF7L2,ZNF800,ZFHX3,GPM6B,ADGRV1,EPB41L5,WHRN,APP,GATA4,SMAD5,IGF2BP1,MAMLD1,RNF38,HCN1,PI4KB,HOOK3,PALLD,CECR2,PTK2,CELF1,LRRK2,PKHD1,RARB,TIAM1,CSRP3,PHACTR1,ACVR2A,MEIS1,KIT,EPHA7,WNT5A,CTNNB1,COL19A1,CTSK,PRKDC,ESR1,TNFRSF19,ITGB8,POFUT2,BCAR3,LMX1A,WWOX,PTGER4,GREB1L,BBX,DIAPH3,HECTD1,NEUROD2,TNFSF11,FXR2,AKAP6,USF3,TASOR,ATF2,ACVR1,EBF2,GRID2,ZNF423,RB1,MPV17,PKD1L3,ADIPOR1,SHROOM4,HNF1B,IMMP2L,KIRREL3,CPS1,NCOA2,PRKCH,ACACB,DLC1,GFRA1,SLC24A3,RAB18,ETV1,TEX11,SIK1,KAT2B,WLS,MEF2D,CDK5RAP2,FLVCR1,ATF3,NPHP1,FBN2,SRI,ARNT,LDLRAD4,TFCP2L1,SEMA3E,SCIN,MACO1,ADAMTS9,STIM1,CDH2,PPARG,RORB,CDK6,TFAP2D,TTC39C,SIPA1L3,SFRP4,FBN1,IQCB1,BMPER,FUT10,CLASP2,ANGPT1,SEMA6A,EIF4G1,ROCK1,PML,AFF2,PRKG1,ELAVL4,UMODL1,MAGI2,NIN,FNDC3A,CALCRL,SLC20A2,STK4,PRLR,TTN,HHIP,KCNE2,EYA4,SEMA5A,TTPA,METTL3,KRT85,ATP8B1,SEMA3C,SMYD1,VPS13B,SPART*
GO:0034329	cell junction assembly	0.008056860933140508	*LRRN1,ARHGEF9,CHRNB2,CLDN6,CDH8,SDK2,EPB41L3,CDH11,SHANK3,NLGN4X,CDH7,MUSK,JAM3,PCLO,PKP4,IL1RAPL2,EPHA3,ADGRB3,LARGE1,PCDHB16,GPM6B,EPB41L5,APP,PTK2,CLSTN2,EPHA7,WNT5A,CTNNB1,GPC4,EFNA5,IKBKB,LINGO2,GRID2,GABRB3,KIRREL3,GABRG2,PRKCH,DLC1,LRTM2,ARHGEF7,NPHP1,BDNF,CDH2,IL1RAP,CLASP2,EIF4G1,ROCK1,CDH9,ASIC2,PTPRA,PARD3*
GO:0007166	cell surface receptor signaling pathway	0.009386087381898738	*FSTL1,CDK14,EPN2,OSBPL8,NFIA,PPM1A,ARL13B,LIMD1,CHST11,SPTBN1,MAPK10,FGF1,CHRNB2,SEZ6,RBPMS,DOK4,HBP1,KALRN,NOTCH2,NPNT,SHANK3,NR4A3,FOXO1,STK39,SMURF2,ADAMTS12,DAAM2,PLXNA4,PRKN,NLGN4X,FAM83B,IFT172,CDON,NLK,MAPKAPK3,LRP8,CNTN1,STAT5B,UBE2O,CPEB4,CALCR,CHRFAM7A,ERBIN,CD247,MCC,ADAR,CDC37,FGF10,COL4A6,NCK1,NREP,BRAF,COL1A2,ALPK2,CHRNA7,HECW1,ZNF304,HIVEP1,TGFB2,MDFIC,LAMA2,TRIO,SMARCC1,NDST1,MUSK,NUP93,EPHA5,SKOR2,*

			UBR5,SOX30,ANKS1B,SLIT3,ROBO1,PAK3,DOK6,MLLT3,TNFRSF12A,OASL,CXCR5,FOSL2,IL1RAPL2,ADGRD1,EPHA3,LATS2,LYN,ADGRB3,GRIK1,PPM1L,NOS3,ERCC6,TGFBR2,ITGA11,TCF7L2,SHISA2,ULK3,GKAP1,ADGRV1,DAPK1,TDP2,INSYN2A,APP,GATA4,SMAD5,GSKIP,ADAM12,PELI2,TLR1,PTK2,TICAM2,SH2B2,PIP4K2B,LRRK2,ITFG2,VTCN1,PJA2,TIAM1,CSRP3,ACVR2A,RABGEF1,KIT,EPHA7,WNT5A,TRABD2B,CTNNB1,TNFRSF19,ITGB8,BCAR3,WWOX,VWC2L,DOCK1,DIAPH3,GPC4,EFNA5,NGEF,TNFSF11,PTPRF,IKBKB,TRBV10-2,ARHGEF28,ATF2,CHRNB3,ACVR1,PID1,GRID2,ZNF423,RB1,IFNLR1,PDE4B,ADIPOR1,PLPP3,HNF1B,PRKCH,GFRA1,BMX,GRIA4,MGRN1,WLS,ANKS1A,ARHGEF7,SAMD12,ATF3,FBN2,ARNT,BDNF,LDLRAD4,SEMA3E,TLR6,TMEM237,KIF16B,RAB11FIP2,CDH2,LAG3,PPARG,PAPPA,CHRNB4,USP9X,IL13RA2,CDK6,SFRP4,IL1RAP,JCAD,VAV2,GRIA3,FBN1,MAP3K1,CLEC6A,BMPER,ANGPT1,SEMA6A,VIPR2,ITGA4,TAX1BP1,PML,AFAP1L2,SP100,MAGI2,SPG21,SLC30A10,PRKD1,CALCRL,USP34,STK4,PRLR,PTPRA,NMI,CR2,HHIP,EYA4,SEMA5A,PMEPA1,METTL3,KCNN4,SEMA3C,VAV1,GRIA2,SPART
GO:0007409	axonogenesis	0.009578449410619576	*APBB2,MAP3K13,CHRNB2,KALRN,NOTCH2,MAP6,CDH11,PLXNA4,TIAM2,CNTN1,BRAF,LAMA2,TRIO,MUSK,EPHA5,ENAH,CHL1,SLIT3,ROBO1,PAK3,EPHA3,KLF7,DIP2B,MATN2,APP,PALLD,PTK2,TIAM1,KIT,EPHA7,WNT5A,LMX1A,EFNA5,TRPC5,ARHGEF28,NTNG1,ETV1,LRTM2,BDNF,SEMA3E,CDH2,USP9X,CLASP2,SEMA6A,ITGA4,PRKG1,NIN,SEMA5A,PARD3,RNF6,SEMA3C,SPART*
GO:0045937	positive regulation of phosphate metabolic process	0.009639793961300738	*SMYD3,MAP3K13,TF,FGF1,DIPK2A,CDON,LRP8,PTH,FGF10,MTCP1,BRAF,CHRNA7,TGFB2,MUSK,MAP3K5,STRADA,MAPRE3,CACUL1,ROBO1,PRKAG2,SPDYE2B,ERP29,LYN,NOS3,ERCC6,TGFBR2,UNC119,SPDYE2,APP,PELI2,PIK3R5,AVPR1B,PTK2,LRRK2,ACVR2A,KIT,EPHA7,WNT5A,BCAR3,TPD52L1,NEK10,EFNA5,TRPC5,FNIP2,TNFSF11,FXR2,CEMIP,ATF2,ACVR1,PID1,PLPP3,XRCC5,GFRA1,KAT2B,ARNT,BDNF,TLR6,TENM1,SPDYE16,TFRC,SPDYE6,CLEC6A,ANGPT1,EIF4G1,ROCK1,PRKD1,STK4,PRLR*
GO:0010562	positive regulation of phosphorus metabolic process	0.009639793961300738	*SMYD3,MAP3K13,TF,FGF1,DIPK2A,CDON,LRP8,PTH,FGF10,MTCP1,BRAF,CHRNA7,TGFB2,MUSK,MAP3K5,STRADA,MAPRE3,CACUL1,ROBO1,PRKAG2,SPDYE2B,ERP29,LYN,NOS3,ERCC6,TGFBR2,UNC119,SPDYE2,APP,PELI2,PIK3R5,AVPR1B,PTK2,LRRK2,ACVR2A,KIT,EPHA7,WNT5A,BCAR3,TPD52L1,NEK10,EFNA5,TRPC5,FNIP2,TNFSF11,FXR2,CEMIP,ATF2,ACVR1,PID1,PLPP3,XRCC5,GFRA1,KAT2B,ARNT,BDNF,TLR6,TENM1,SPDYE16,TFRC,SPDYE6,CLEC6A,ANGPT1,EIF4G1,ROCK1,PRKD1,STK4,PRLR*
GO:0080090	regulation of primary metabolic process	0.010746894576039234	*APBB2,RTN1,RGN,NOSTRIN,ING3,MED13L,NFIA,PHF20,FOXK2,SMYD3,MAP3K13,PPM1A,SAMSN1,LIMD1,TF,THRB,ATF7,FGF1,CHRNB2,AGTPBP1,ATF7-*

			NPFF,RBPMS,SPINK5,OPA3,GPBP1L1,BRD4,ZNF264,HBP1,SDR16C5,NOTCH2,HMBOX1,ZNF551,NPNT,MORC1,NR4A3,ARID1B,E2F7,FOXO1,ZNF611,SMURF2,YBX1,DIPK2A,ARB2A,PRKN,KDM7A,CHD7,PTBP3,CDON,NLK,MRPL13,ZNF10,LRP8,TAGLN3,MEOX2,STAT5B,CPEB4,EIF4ENIF1,CALCR,ERBIN,ZNF121,MBTPS2,PTH,ADAR,TM9SF2,TOX2,CDC37,FGF10,MECOM,HERC5,ILDR2,NCK1,CD84,FAM168A,NONO,MTCP1,KDM2B,ZNF404,WRNIP1,RSF1,NCOA7,CIDEB,MCPH1,BRAF,ZNF214,GEMIN5,MRTFB,CHRNA7,HMGB1,ZNF304,AFF3,HIVEP1,ATRX,TGFB2,MDFIC,ZNF85,HOXC13,SMARCC1,MUSK,KCNIP3,SFPQ,MAP3K5,ALKBH1,ZCCHC4,PRKAR2A,RBM33,CTIF,RBM23,ZNF154,SLC15A4,MBTPS1,IGFBP7,EPHA5,SKOR2,RUNX1,STRADA,HUWE1,ZNF595,ASPH,SH3BGRL,GTF2F2,SIM1,SOX30,SPOCK3,GSN,SPON1,MAPRE3,TLR8,CACUL1,NLRP7,PHC2,ROBO1,ZNF578,KLF12,PRKAG2,MLLT3,CCNT2,CNOT10,ZNF429,FOSL2,LATS2,SPDYE2B,ERP29,CDYL2,UVRAG,LYN,KLF7,SUMO2,MTA3,DIP2B,FIGNL1,PUS7,LDB2,MYRFL,FMO5,RNASEL,DBF4B,DCP2,NOS3,VSX1,ZNF280B,TNRC6C,PRAMEF27,MLLT10,ERCC6,NOS1AP,EIF2AK4,MAGEL2,TGFBR2,TCF7L2,ZNF800,ZNF462,E2F6,ZFHX3,CUX1,UNC119,SPDYE2,SERBP1,WAC,DAPK1,TDP2,ZNF678,APP,GATA4,ATF7IP2,SMAD5,IGF2BP1,MAMLD1,GSKIP,PELI2,SP140L,CHRM5,FRY,ELOVL5,PIK3R5,RIPK4,AVPR1B,PTK2,USP7,SCP2,ATP1B4,ZNF41,CELF1,POGZ,LRRK2,INO80D,ZNF395,IKZF3,ARIH1,PLD1,PKHD1,TENT5C,RARB,TCF4,PAEP,COP1,CSRP3,MLIP,ACVR2A,TIPIN,MEIS1,NEDD4L,BRCC3,RABGEF1,KIT,EPHA7,SPAG9,WNT5A,TRABD2B,CTNNB1,ANKRD17,RUNX1T1,PRKDC,IPO5,ESR1,ZNF479,BCAR3,TPD52L1,L3MBTL4,PPM1E,ZXDC,LMX1A,WWOX,NEK10,ZNF215,BBX,PKIG,EFNA5,IGF2BP2,ZNF722,CDC14B,HECTD1,TRPC5,NEUROD2,FNIP2,AASS,KAT14,TNFSF11,IKBKB,FXR2,USF3,RAB3GAP2,TASOR,CCPG1,POU6F2,CEMIP,ATF2,ZNF426,HDAC8,ACVR1,PID1,EBF2,ZNF423,ZCCHC17,RB1,NSMCE1,MPV17,ADIPOR1,ZNF143,ZSCAN23,PLPP3,HNF1B,ZNF615,NCOA2,PRKCH,ACACB,XRCC5,TBRG4,GFRA1,SHLD2,ZNF418,TAF15,ZNF860,POU2F1,ETV1,MID2,SIK1,ZNF850,KAT2B,SUPV3L1,MEF2D,GTF2A1,CDK5RAP2,RFC3,DACH1,ATF3,IKZF1,ARNT,BDNF,TFCP2L1,BCLAF3,MALRD1,TENT4B,ZNF665,TLR6,EEFSEC,PPARG,USP9X,NEK7,ZNF80,TENM1,RORB,CDK6,AVIL,TFAP2D,USP25,ZNF286A-TBC1D26,SFRP4,SPDYE16,TFRC,SPDYE6,ZFP82,CLEC6A,ZNF292,CLTC,ZNF521,ZNF761,ANGPT1,EIF4G1,ZNF618,ROCK1,TAX1BP1,ZNF701,NUP98,IKZF2,PML,FOXO3B,AFF2,TNFSF4,AFAP1L2,SP100,PRKG1,ELAVL4,ZNF286A,ZNF69,KHDRBS3,HSPBP1,PRKD1,STK4,PRLR,ZNF569,ZNF180,NMI,YLPM1,ZNF470,CTBP2,TTN,DYRK1A,KCNE2,CUL4A,EYA4,ZNF888,ZNF736,ELL2,PVT1,GATAD2B,METTL3,PARD3,ZNF33A,CP,NPAS3,BMAL2,ATP8B1,RNF6,SMYD1,FANCB
GO:1901	regulation	0.01175826	*LRRN1,CHRNB2,MUSK,IL1RAPL2,EPHA3,ADGRB*

888	of cell junction assembly	3237350294	*3,GPM6B,EPB41L5,APP,PTK2,CLSTN2,EPHA7,WNT5A,CTNNB1,GPC4,EFNA5,IKBKB,LINGO2,GRID2,PRKCH,DLC1,LRTM2,NPHP1,BDNF,IL1RAP,CLASP2,EIF4G1,ROCK1,ASIC2,PTPRA*
GO:0051239	regulation of multicellular organismal process	0.014343532119487724	*RGN,EPN2,LRRN1,ISM1,MAP3K13,SAMSN1,TF,THRB,SPTBN1,CACNB1,FGF1,CHRNB2,SPINK5,BRD4,CAMK2B,DCT,ABCG8,KALRN,NOTCH2,NPNT,MAP6,SHANK3,NR4A3,ARID1B,FOXO1,STK39,SMURF2,ADAMTS12,DAAM2,PLXNA4,CACNA1C,IDO1,NLGN4X,CHD7,CDON,TIAM2,CNMD,LRP8,STAT5B,CALCR,ERBIN,MCC,PTH,RHOJ,FGF10,TSPAN18,PLG,ILDR2,NCK1,CD84,CHRM3,KDM2B,BRAF,MRTFB,ALPK2,CHRNA7,HMGB1,ZNF304,RFLNA,TGFB2,LAMA2,ADD3,SMARCC1,SLC15A4,PRKG2,RUNX1,ASPH,PLS1,TLR8,NLRP7,ROBO1,ABI3BP,HAVCR1,CPT2,FOSL2,LATS2,LYN,ADGRB3,KLF7,DIP2B,ALDH1A1,CORIN,SGMS2,NOS3,NOS1AP,EIF2AK4,TGFBR2,TCF7L2,ZFHX3,CUX1,GPM6B,GSDMD,ADGRV1,EPB41L5,PRUNE1,APP,GATA4,IGF2BP1,RNLS,ADAM12,CACNA2D1,HOOK3,TLR1,AVPR1B,PTK2,TICAM2,CELF1,LRRK2,INO80D,IKZF3,CLSTN2,VTCN1,RARB,PJA2,PAEP,TIAM1,CSRP3,MLIP,ACVR2A,MEIS1,RABGEF1,KIT,EPHA7,WNT5A,ARFGEF2,CTNNB1,CTSK,PRKDC,ESR1,ITGB8,PTGER4,DOCK1,EFNA5,IGF2BP2,TRPC5,KAT14,TNFSF11,IKBKB,AKAP6,USF3,APOH,LINGO2,ATF2,KCNJ12,ACVR1,HLA-DQA2,EBF2,GRID2,ZNF423,KCNJ3,RB1,PDE4B,ADIPOR1,HNF1B,NCOA2,PRKCH,ACACB,XRCC5,SHLD2,KCND2,LRTM2,KAT2B,MYL4,FLVCR1,TRDN,FBN2,SRI,ATP1B1,ARNT,BDNF,LDLRAD4,SEMA3E,SCIN,TLR6,ADAMTS9,STIM1,TMEM161B,LAG3,PPARG,ASB3,CHRNB4,CDK6,SH3KBP1,SFRP4,BLTP1,IL1RAP,JCAD,TFRC,FBN1,CLEC6A,BMPER,CLASP2,ANGPT1,SEMA6A,ITGA4,ROCK1,PML,MGLL,TNFSF4,AFAP1L2,SP100,PRKG1,NIN,ASIC2,PRKD1,SLC20A2,STK4,TNNI3K,PRLR,NMI,IL16,KCNE2,CUL4A,SEMA5A,LAMA4,TTPA,METTL3,UAP1,PARD3,RNF6,SEMA3C,VAV1,SPART*
GO:0032879	regulation of localization	0.014742757748158733	*OSBPL8,STX8,PPM1A,TF,SPTBN1,CACNB1,CHRNB2,FHL1,KCNJ6,CAMK2B,XPO4,KALRN,SHANK3,NR4A3,FOXO1,STK39,CACNA1C,PRKN,CHD7,GNAI1,CNTN1,CHRFAM7A,CD247,PTH,FGF10,ZFYVE16,CD84,CHRM3,SESTD1,BRAF,HMGB1,DYNLT2B,TGFB2,MDFIC,KCNJ18,KCNIP3,TBC1D5,ATP4A,GOLPH3,PRKG2,MBTPS1,EPHA5,ATP2C1,STON2,PCLO,HUWE1,ASPH,UBR5,GSN,PLS1,ITPR1,PRKAG2,EPHA3,LATS2,ERP29,PTPRN2,LYN,USP6,KLF7,NUP58,RNASEL,CORIN,DCP2,NOS3,KCNIP4,NOS1AP,KIAA1614,MARK4,TCF7L2,UNC119,GPM6B,EPB41L5,SCG5,DAPK1,SAR1B,APP,CACNA2D1,AVPR1B,USP7,SCP2,LRRK2,NKAIN3,PLD1,TIAM1,CSRP3,IQSEC2,NEDD4L,RABGEF1,WNT5A,CTNNB1,IPO5,PTPN14,SCFD1,ERC2,POFUT2,FRMD4A,BEST3,GPC4,PKIG,EFNA5,PLCH1,TNFSF11,AKAP6,CEMIP,KCNJ12,PID1,KCNJ3,PDE4B,BNIP3L,PRKCH,ACACB,RABGAP1L,SIK1,KAT2B,GOLPH3L,WLS,RYR3,ARHGEF7,TRDN,SRI,ATP1B1,BDNF,SCIN,STIM1,R*

			AB11FIP2,CDH2,PPARG,CHRNB4,TENM1,IL13RA2,MYOF,SFRP4,TFRC,OSBPL6,RAB31,RAPGEF4,BMPER,CLASP2,CLTC,ANGPT1,EIF4G1,DNAJC6,ROCK1,TBC1D4,PML,SP100,PRKG1,NDUFAF2,MAGI2,ANKFY1,ASIC2,ENSA,AIMP1,PRKD1,IL16,TTN,PPFIA1,TMEM30A,MYRIP,KCNE2,KCNN4,ATP8B1
GO:0009893	positive regulation of metabolic process	0.015696313597975783	APBB2,RGN,ING3,NFIA,PHF20,FOXK2,SMYD3,MAP3K13,PPM1A,TF,THRB,SPTBN1,ATF7,FGF1,AGTPBP1,RBPMS,GPBP1L1,BRD4,NOTCH2,HMBOX1,NPNT,NR4A3,ARID1B,E2F7,FOXO1,FER1L6,YBX1,DIPK2A,IDO1,PRKN,KDM7A,CHD7,GPSM1,CDON,LRP8,CNTN1,MEOX2,STAT5B,EIF4ENIF1,CALCR,MBTPS2,PTH,ADAR,TOX2,CLCN3,FGF10,MECOM,NCK1,CD84,FAM168A,MTCP1,RSF1,NCOA7,ZNF804A,BRAF,MRTFB,CHRNA7,HMGB1,ZNF304,HIVEP1,ATRX,TGFB2,MDFIC,HOXC13,SMARCC1,MUSK,SFPQ,MAP3K5,ZCCHC4,CTIF,RBM23,RUNX1,ALDH1A2,PTGFR,STRADA,HUWE1,ASPH,SH3BGRL,UBR5,GTF2F2,SOX30,GSN,SPON1,MAPRE3,TLR8,CACUL1,ROBO1,KLF12,PRKAG2,MLLT3,CCNT2,CNOT10,CPT2,FOSL2,SPDYE2B,ERP29,UVRAG,LYN,KLF7,SUMO2,MTA3,DIP2B,LDB2,MYRFL,RNASEL,DBF4B,DCP2,NOS3,TNRC6C,MLLT10,ERCC6,NOS1AP,EIF2AK4,TGFBR2,TCF7L2,ZNF462,ZFHX3,UNC119,SPDYE2,GSDMD,WAC,WHRN,DAPK1,APP,GATA4,SMAD5,IGF2BP1,PELI2,ELOVL5,PIK3R5,TLR1,AVPR1B,PTK2,TICAM2,SCP2,CELF1,POGZ,PIP4K2B,LRRK2,INO80D,ZNF395,IKZF3,ARIH1,PLD1,VTCN1,TENT5C,RARB,TCF4,PAEP,COP1,CSRP3,MLIP,ACVR2A,MEIS1,NEDD4L,BRCC3,KIT,EPHA7,WNT5A,TRABD2B,ARFGEF2,CTNNB1,PRKDC,ESR1,ITGB8,BCAR3,TPD52L1,ZXDC,LMX1A,WWOX,NEK10,PTGER4,EFNA5,IGF2BP2,CDC14B,HECTD1,TRPC5,NEUROD2,FNIP2,TNFSF11,IKBKB,FXR2,USF3,RAB3GAP2,CCPG1,CEMIP,ATF2,ACVR1,PID1,EBF2,ZNF423,RB1,IFNLR1,PDE4B,ADIPOR1,ZNF143,PLPP3,HNF1B,ZNF615,NCOA2,BNIP3L,XRCC5,GFRA1,SHLD2,SLC24A3,TAF15,POU2F1,ETV1,MID2,ZNF850,KAT2B,SUPV3L1,MEF2D,GTF2A1,SPTLC1,CDK5RAP2,RFC3,ATF3,ARNT,BDNF,TFCP2L1,BCLAF3,TENT4B,TLR6,PPARG,NEK7,TENM1,RORB,CDK6,TFAP2D,SFRP4,SPDYE16,IL1RAP,TFRC,SPDYE6,CLEC6A,ZNF292,ZNF521,ANGPT1,EIF4G1,ROCK1,NUP98,PML,TNFSF4,AFAP1L2,SP100,ELAVL4,HSPBP1,PRKD1,STK4,PRLR,NMI,IL16,CTBP2,TTN,DYRK1A,KCNE2,CUL4A,EYA4,ELL2,PVT1,GATAD2B,METTL3,UAP1,ZNF33A,NPAS3,BMAL2,RNF6,FANCB
GO:0033036	macromolecule localization	0.01592029083015795	ESYT1,OSBPL8,STX8,SMYD3,RAPGEF6,BLTP3B,PPM1A,ARL13B,RN7SL556P,IMMP1L,CHST11,RCSD1,SPTBN1,ATP10A,GRIP1,TLK1,ABCG8,RGPD2,XPO4,USH2A,NPNT,EPB41L3,SHANK3,FOXO1,YBX1,PRKN,EXOC4,XPO6,GNAI1,INTS6-AS1,TRAK2,STAT5B,EIF4ENIF1,ERBIN,CD247,MCC,CEP192,ADAR,TM9SF2,CDC37,FGF10,ZFYVE16,ILDR2,KCNQ3,CHRM3,RGPD3,ANO3,CIDEB,MCPH1,MYO1D,BRAF,XPO7,ATRX,TGFB2,MDFIC,MUSK,JAM3,KCNIP3,NUP93,RBM33,TBC1D

			5,ATP11A,SLC15A4,GOLPH3,PRKG2,PIK3C3,EPHA5,SNX10,ATP2C1,STRADA,PCLO,HUWE1,ASPH,ZDHHC4,UBR5,GSN,PLS1,MAPRE3,ITPR1,DNAAF11,CPT2,VPS8,MTX3,PACRG,EPHA3,LATS2,ERP29,PTPRN2,KLF7,SEC24C,LARGE1,NUP58,PDZD8,SIL1,DCP2,TMED7-TICAM2,KCNIP4,NOMO2,RN7SL738P,XKR6,TGFBR2,MARK4,TCF7L2,UNC119,GPM6B,RRAGD,KPNB1,GSDMD,ADGRV1,EPB41L5,SCG5,WHRN,SAR1B,TTC7B,IGF2BP1,ATP9B,HCN1,HERC2,HOOK3,USE1,AVPR1B,SCP2,HYCC1,LRRK2,GET1-SH3BGR,TIAM1,CSRP3,IQSEC2,NEDD4L,ENSG00000288683,SNX13,RABGEF1,CYB5R4,OSBPL3,SPDL1,WNT5A,WIPI2,PEX26,ARFGEF2,TEX2,CTNNB1,IPO5,ESR1,PTPN14,SCFD1,VPS53,FRMD4A,GPC4,PKIG,EFNA5,IGF2BP2,SYNE3,TRPC5,TNFSF11,IKBKB,AKAP6,APOH,RAB3GAP2,TASOR,CEMIP,ATF2,PID1,CCDC91,GRID2,ZNF423,RB1,KIF13A,GOSR2,SMG7,HNF1B,IMMP2L,LAMP2,BNIP3L,PRKCH,ACACB,RABGAP1L,XRCC5,RAB18,MID2,GOLPH3L,WLS,NPHP1,FBN2,SRI,ATP1B1,BDNF,SCIN,ADAMTS9,RAB11FIP2,CDH2,PPARG,ASB3,MTTP,USP9X,TENM1,JAKMIP1,GDAP1,MYO5A,BLTP1,TFRC,VPS13A,OSBPL6,PNPLA8,FBN1,RAB31,RAPGEF4,BMPER,FUT10,CLASP2,CLTC,ANGPT1,EIF4G1,ITGA4,ROCK1,TAX1BP1,NUP98,PML,SCFD2,SP100,NDUFAF2,MAGI2,NIN,ASIC2,ENSA,AP4S1,PRKD1,CALCRL,STK4,RAB6D,SCAMP1,RAB6C,TTN,PPFIA1,TMEM30A,MYRIP,TTPA,PEX5L,PARD3,KCNN4,ATP8B1,VPS13B
GO:0044093	positive regulation of molecular function	0.015949508291595554	*RGN,RAPGEF6,ABR,MAP3K13,FGF1,HMBOX1,STK39,DIPK2A,CACNA1C,PRKN,GPSM1,TIAM2,LRP8,CALCR,FGF10,CHRNA7,HMGB1,TGFB2,MAP3K5,EPHA5,STRADA,ASPH,MAPRE3,CACUL1,ROBO1,PKP4,PRKAG2,SPDYE2B,ERP29,LYN,NOS3,NOS1AP,TGFBR2,TCF7L2,UNC119,SPDYE2,APP,CACNA2D1,PIK3R5,RIPK4,AVPR1B,PTK2,LRRK2,TIAM1,ASAP2,SNX13,KIT,WNT5A,ESR1,BCAR3,TPD52L1,NEK10,CDC14B,NEUROD2,TNFSF11,IKBKB,AKAP6,APOH,CEMIP,ATF2,RB1,PLPP3,PRKCH,XRCC5,MID2,MYL4,RFC3,ARHGEF7,TRDN,ATP1B1,BDNF,TLR6,STIM1,RAB11FIP2,PPARG,USP9X,TENM1,SPDYE16,TFRC,SPDYE6,ANGPT1,EIF4G1,ZNF618,ROCK1,SP100,PRKD1,STK4,PRLR,SERINC1*
GO:0019220	regulation of phosphate metabolic process	0.01694673352892685	*RGN,SMYD3,MAP3K13,SAMSN1,TF,FGF1,DIPK2A,PRKN,CDON,LRP8,PTH,ADAR,CDC37,FGF10,HERC5,NCK1,MTCP1,BRAF,CHRNA7,TGFB2,MUSK,MAP3K5,PRKAR2A,STRADA,MAPRE3,TLR8,CACUL1,ROBO1,PRKAG2,CCNT2,LATS2,SPDYE2B,ERP29,UVRAG,LYN,LDB2,NOS3,ERCC6,TGFBR2,UNC119,SPDYE2,APP,GSKIP,PELI2,CHRM5,PIK3R5,AVPR1B,PTK2,SCP2,LRRK2,ACVR2A,RABGEF1,KIT,EPHA7,SPAG9,WNT5A,PRKDC,IPO5,BCAR3,TPD52L1,PPM1E,NEK10,PKIG,EFNA5,TRPC5,FNIP2,TNFSF11,IKBKB,FXR2,CEMIP,ATF2,ACVR1,PID1,RB1,SMG7,PLPP3,XRCC5,GFRA1,KAT2B,ARNT,BDNF,TLR6,PPARG,TENM1,SPDYE16,TFRC,SPDYE6,CLEC6A,ANGPT1,EIF4G1,ROCK1,PRKG1,PRKD1,STK4,PRLR,TTN,PARD*

			3
GO:0060560	developmental growth involved in morphogenesis	0.017234176695391377	*MAP3K13,FGF1,PLXNA4,PRKN,FGF10,SLIT3,DIP2B,EIF2AK4,TGFBR2,EPB41L5,APP,TIAM1,NEDD4L,EPHA7,SPAG9,WNT5A,CTNNB1,ESR1,EFNA5,TRPC5,HNF1B,BDNF,SEMA3E,USP9X,CLASP2,SEMA6A,ITGA4,PRKG1,NIN,SEMA5A,RNF6,SEMA3C,SPART*
GO:0051174	regulation of phosphorus metabolic process	0.017613812650786983	*RGN,SMYD3,MAP3K13,SAMSN1,TF,FGF1,DIPK2A,PRKN,CDON,LRP8,PTH,ADAR,CDC37,FGF10,HERC5,NCK1,MTCP1,BRAF,CHRNA7,TGFB2,MUSK,MAP3K5,PRKAR2A,STRADA,MAPRE3,TLR8,CACUL1,ROBO1,PRKAG2,CCNT2,LATS2,SPDYE2B,ERP29,UVRAG,LYN,LDB2,NOS3,ERCC6,TGFBR2,UNC119,SPDYE2,APP,GSKIP,PELI2,CHRM5,PIK3R5,AVPR1B,PTK2,SCP2,LRRK2,ACVR2A,RABGEF1,KIT,EPHA7,SPAG9,WNT5A,PRKDC,IPO5,BCAR3,TPD52L1,PPM1E,NEK10,PKIG,EFNA5,TRPC5,FNIP2,TNFSF11,IKBKB,FXR2,CEMIP,ATF2,ACVR1,PID1,RB1,SMG7,PLPP3,XRCC5,GFRA1,KAT2B,ARNT,BDNF,TLR6,PPARG,TENM1,SPDYE16,TFRC,SPDYE6,CLEC6A,ANGPT1,EIF4G1,ROCK1,PRKG1,PRKD1,STK4,PRLR,TTN,PARD3*
GO:0008104	protein localization	0.018116674905024735	*OSBPL8,STX8,SMYD3,RAPGEF6,PPM1A,ARL13B,RN7SL556P,IMMP1L,RCSD1,SPTBN1,GRIP1,TLK1,RGPD2,XPO4,USH2A,NPNT,EPB41L3,SHANK3,FOXO1,YBX1,PRKN,EXOC4,XPO6,GNAI1,INTS6-AS1,TRAK2,EIF4ENIF1,ERBIN,CD247,MCC,CEP192,ADAR,TM9SF2,CDC37,FGF10,ZFYVE16,ILDR2,KCNQ3,CHRM3,RGPD3,MCPH1,MYO1D,BRAF,XPO7,ATRX,TGFB2,MDFIC,MUSK,JAM3,KCNIP3,NUP93,TBC1D5,SLC15A4,GOLPH3,PRKG2,PIK3C3,EPHA5,SNX10,ATP2C1,STRADA,PCLO,HUWE1,ASPH,ZDHHC4,UBR5,GSN,PLS1,MAPRE3,ITPR1,DNAAF11,VPS8,MTX3,PACRG,EPHA3,LATS2,ERP29,PTPRN2,KLF7,SEC24C,LARGE1,NUP58,SIL1,TMED7-TICAM2,KCNIP4,NOMO2,RN7SL738P,MARK4,TCF7L2,UNC119,GPM6B,RRAGD,KPNB1,GSDMD,ADGRV1,EPB41L5,SCG5,WHRN,SAR1B,TTC7B,HCN1,HERC2,HOOK3,USE1,SCP2,HYCC1,LRRK2,GET1-SH3BGR,TIAM1,CSRP3,IQSEC2,NEDD4L,ENSG00000288683,SNX13,RABGEF1,CYB5R4,SPDL1,WNT5A,WIPI2,PEX26,ARFGEF2,CTNNB1,IPO5,ESR1,PTPN14,SCFD1,VPS53,FRMD4A,GPC4,PKIG,EFNA5,SYNE3,IKBKB,AKAP6,RAB3GAP2,TASOR,CEMIP,ATF2,PID1,CCDC91,GRID2,ZNF423,RB1,KIF13A,GOSR2,HNF1B,IMMP2L,LAMP2,BNIP3L,PRKCH,RABGAP1L,XRCC5,RAB18,MID2,GOLPH3L,WLS,NPHP1,FBN2,SRI,ATP1B1,BDNF,SCIN,ADAMTS9,RAB11FIP2,CDH2,PPARG,ASB3,MTTP,USP9X,TENM1,JAKMIP1,GDAP1,MYO5A,TFRC,VPS13A,FBN1,RAB31,RAPGEF4,BMPER,FUT10,CLASP2,CLTC,ANGPT1,EIF4G1,ITGA4,ROCK1,TAX1BP1,NUP98,PML,SCFD2,SP100,NDUFAF2,MAGI2,NIN,ASIC2,ENSA,AP4S1,PRKD1,CALCRL,STK4,RAB6D,SCAMP1,RAB6C,TTN,PPFIA1,TMEM30A,MYRIP,PEX5L,PARD3,KCNN4,ATP8B1*

GO:0050896	response to stimulus	0.019520552112682225	*FSTL1,APBB2,PFKP,ABCC6,RGN,TRGC2,CDK14,EPN2,MSRB3,PDE1C,NOSTRIN,ING3,MAP3K3,OSBPL8,NFIA,STX8,FOXK2,CPNE4,SMYD3,RAPGEF6,ARHGEF9,TAFA2,ABR,MAOA,TMEM255A,MAP3K13,PPM1A,ARL13B,SAMSN1,LIMD1,TF,CHST11,THRB,RCSD1,SPTBN1,CDC42EP3,MAPK10,CACNB1,USP32,DAPP1,GNG12,FGF1,CHRNB2,SEZ6,CDH8,ARHGAP15,GNB4,RBPMS,SPINK5,OPA3,SKAP2,ATP7B,BRD4,DOK4,CAMK2B,GRIP1,ARHGAP11A-SCG5,TLK1,DDI2,DCT,SLC38A9,HBP1,ABCG8,KALRN,USH2A,MAP4K3,NOTCH2,FKBP5,TMOD2,NPNT,MORC1,SHANK3,NR4A3,ARID1B,E2F7,FOXO1,STYXL1,STK39,SMURF2,ADAMTS12,DAAM2,FER1L6,PLXNA4,URB2,YBX1,STK24,DIPK2A,ARMT1,DCDC1,CACNA1C,FAM3C,ASB4,IDO1,PRKN,NLGN4X,PNPLA3,F13A1,PIK3C2B,FAM83B,IFT172,CHD7,CDON,NLK,GNAI1,MAPKAPK3,TIAM2,LRP8,CNTN1,AGL,STAT5B,ARHGEF4,UBE2O,DDIAS,CPEB4,CALCR,CHRFAM7A,ELMO1,ERBIN,MBTPS2,RGS22,CD247,MCC,CEP192,PTH,ADAR,PDE10A,RHOJ,CDC37,FGF10,ZFYVE16,MECOM,COL4A6,TSPAN18,NLRP8,STK32B,PLG,HERC5,UBE2E2,ILDR2,NCK1,CD84,FAM168A,NONO,NREP,EXO1,GABRA5,KCNQ3,MTCP1,CHRM3,PREX1,PMS1,WRNIP1,ANO3,COL11A1,NCOA7,CIDEB,MARCHF1,MCPH1,BRAF,COL1A2,DTNA,ALPK2,CHRNA7,HECW1,HMGB1,ZNF304,CDIP1,AFF3,HIVEP1,ATRX,TGFB2,MDFIC,LAMA2,ADD3,TRIO,SMARCC1,RAD51B,NDST1,MUSK,JAM3,KCNIP3,SFPQ,MAP3K5,ALKBH1,ARPP21,RGS3,NUP93,RERG,OR5AU1,PRKAR2A,TBC1D5,ATP4A,SLC15A4,GOLPH3,PRKG2,MBTPS1,NCF2,PIK3C3,USP10,HMCN1,IGFBP7,EPHA5,SKOR2,ENSG00000289694,SNX10,ATP2C1,ALDH1A2,PTGFR,IGHV1OR21-1,PCLO,HUWE1,ASPH,RRAS2,UBR5,ABTB2,SOX30,ANKS1B,CHL1,GSN,TCP11L1,TLR8,SLIT3,NLRP7,ROBO1,ITPR1,PAK3,ABI3BP,CYP2A13,DOK6,PRKAG2,MLLT3,MR1,F8,TNFRSF12A,OASL,TAS2R14,CXCR5,NCKAP1,CILK1,FOSL2,PACRG,IL1RAPL2,ADGRD1,EPHA3,LATS2,ERP29,ARHGAP18,UVRAG,PTPRN2,LYN,ADGRB3,KLF7,GRIK1,LARGE1,FIGNL1,ALDH1A1,NMNAT3,FMO5,RNASEL,PPM1L,TMED7-TICAM2,NOS3,VSX1,BPHL,ERCC6,NOS1AP,EIF2AK4,MSRA,RAB12,MATN2,TGFBR2,CMKLR2,ITGA11,MARK4,DDX60L,TCF7L2,CHAF1B,ZFHX3,UNC119,SHISA2,RRAGD,ULK3,GPR156,GKAP1,CACNA2D4,PPP2R5E,GSDMD,ADGRV1,RASGRF2,EPB41L5,SCG5,WAC,WHRN,DAPK1,TDP2,ACSM2B,INSYN2A,APP,GATA4,SMAD5,IGF2BP1,RNLS,GSKIP,ADAM12,PELI2,CACNA2D1,HCN1,CHRM5,PI4KB,RHOT1,HERC2,PIK3R5,TLR1,TAS2R30,BMT2,AVPR1B,PTK2,USP7,TICAM2,SH2B2,CELF1,POGZ,PIP4K2B,LRRK2,INO80D,IKZF3,ARIH1,PLD1,ITFG2,VTCN1,PKHD1,RARB,PJA2,MGST1,COP1,TIAM1,CSRP3,MLIP,IQSEC2,ACVR2A,GNAO1,TIPIN,RASSF4,BRCC3,SNX13,RABGEF1,CYB5R4,KIT,EPHA7,SPAG9,SPDL1,WNT5A,TRABD2B,WIPI2,ARFGEF2,TEX2,CTNNB1,AN*

			KRD17,CTSK,PRKDC,IPO5,ESR1,TNFRSF19,SCFD1,ITGB8,BCAR3,TPD52L1,PPM1E,LMX1A,WWOX,HUNK,NEK10,PTGER4,PDE7B,VWC2L,DOCK1,DIAPH3,GPC4,BPGM,EFNA5,CDC14B,PLCH1,OR1M1,VKORC1L1,NGEF,NEUROD2,FNIP2,JAML,TNFSF11,PTPRF,IKBKB,SH3BP5,AKAP6,TRBV10-2,LYVE1,APOH,ARHGEF28,OR8B8,ATF2,CHRNB3,ACVR1,HLA-DQA2,PID1,GRID2,ZNF423,KCNJ3,RB1,NSMCE1,MPV17,IFNLR1,PDE4B,PKD1L3,NLRP4,ADIPOR1,GABRB3,PLPP3,HNF1B,IMMP2L,LAMP2,GABRG2,F5,CPS1,NCOA2,TMEM120A,BNIP3L,PRKCH,ACACB,DLC1,DENND2B,XRCC5,RGS17,GFRA1,SHLD2,KCND2,INPP4A,BMX,GRIA4,POLD3,RAB18,ETV1,RGS7BP,MGRN1,MID2,LRTM2,SIK1,ERCC6L2,TP53BP2,KAT2B,WLS,ANKS1A,IGKV1-6,RYR3,PRCP,ZPLD1,CDK5RAP2,RFC3,ARHGEF7,SAMD12,DACH1,ATF3,TRDN,NPHP1,FBN2,SRI,ATP1B1,ARNT,BDNF,LDLRAD4,SEMA3E,MACO1,TLR6,ADAMTS9,STIM1,TMEM237,KIF16B,RAB11FIP2,CDH2,LAG3,PPARG,TANC1,PAPPA,ASB3,MTTP,CHRNB4,USP9X,NEK7,TENM1,IL13RA2,RORB,CDK6,GABRB1,MYOF,LTB4R2,SMOX,GDAP1,DDX60,USP25,MYO5A,SIPA1L3,WDFY4,SFRP4,IL1RAP,JCAD,VAV2,TFRC,GRIA3,PNPLA8,FBN1,HAPSTR1,RAB31,MAP3K1,CLEC6A,RAPGEF4,BMPER,FUT10,CLASP2,ANGPT1,SEMA6A,VIPR2,EIF4G1,PHLPP1,ITGA4,ROCK1,TAX1BP1,TBC1D4,APIP,MMP28,PML,MGLL,SCARA5,PLEK2,TNFSF4,AFAP1L2,DGKG,SP100,PRKG1,ELAVL4,NDUFAF2,UMODL1,MAGI2,SPG21,ASIC2,ENSA,AIMP1,SLC30A10,PRKD1,CALCRL,USP34,STK4,SPC25,RNASE11,PRLR,PTPRA,PDE11A,NMI,RAB6C,EYS,IL16,ZSWIM7,EDARADD,GNA14,CR2,LPAR6,CTBP2,TTN,NCALD,HHIP,DYRK1A,PPFIA1,PPP1R1C,KCNE2,CUL4A,KCTD16,TXNRD3,EYA4,SEMA5A,PMEPA1,TTPA,PEX5L,METTL3,UAP1,KCNN4,CP,FUNDC1,BMAL2,RNF6,SEMA3C,VAV1,FANCB,GRIA2,VDAC2,SPART,IGLV4-3
GO:0071495	cellular response to endogenous stimulus	0.019541927938076173	FSTL1,EPN2,OSBPL8,NFIA,SMYD3,PPM1A,CHST11,THRB,FGF1,NOTCH2,NPNT,NR4A3,ARID1B,FOXO1,SMURF2,ADAMTS12,PNPLA3,NLK,LRP8,STAT5B,UBE2O,PTH,FGF10,NCK1,NONO,NREP,BRAF,COL1A2,HIVEP1,TGFB2,SMARCC1,NDST1,IGFBP7,SKOR2,ALDH1A2,PTGFR,UBR5,SLIT3,ROBO1,LATS2,LYN,TGFBR2,SHISA2,GKAP1,EPB41L5,GATA4,SMAD5,AVPR1B,PTK2,SH2B2,PIP4K2B,RARB,CSRP3,ACVR2A,WNT5A,CTNNB1,CTSK,PRKDC,ESR1,ITGB8,BCAR3,WWOX,PTGER4,VWC2L,EFNA5,ATF2,ACVR1,PID1,ZNF423,RB1,ADIPOR1,CPS1,NCOA2,GFRA1,KAT2B,PRCP,FBN2,ARNT,BDNF,LDLRAD4,KIF16B,PPARG,USP9X,MYO5A,SFRP4,JCAD,FBN1,RAB31,BMPER,SEMA6A,ROCK1,TBC1D4,PML,TNFSF4,SP100,ELAVL4,UMODL1,MAGI2,SLC30A10,PRKD1,PRLR,PTPRA,CTBP2,HHIP,PMEPA1,RNF6,SPART
GO:0016192	vesicle-mediated	0.0212302667536253	EPN2,NOSTRIN,STX8,BLTP3B,TRAPPC9,TF,RCSD1,SPTBN1,GRIP1,KALRN,CD93,NR4A3,PRKN

	transport		*,NLGN4X,EXOC4,SLC17A6,MAPKAPK3,LRP8,UBE2O,ELMO1,CLCN3,RHOJ,ZFYVE16,TSPAN18,CD84,KCNQ3,CIDEB,MYO1D,BRAF,HMGB1,TBC1D5,GOLPH3,MBTPS1,NCF2,PIK3C3,SNX10,ATP2C1,STON2,PCLO,SOX30,GSN,HAVCR1,TRAPPC10,VPS8,EPHA3,UVRAG,LYN,USP6,SEC24C,ATP6V1H,TMCC1,XKR6,MAGEL2,SYNJ1,CUX1,UNC119,SAR1B,APP,ATP9B,PI4KB,HOOK3,USE1,CECR2,LMAN1L,PTK2,USP7,TICAM2,PIP4K2B,LRRK2,PLD1,NEDD4L,RABGEF1,KIT,SPAG9,WNT5A,ARFGEF2,CTNNB1,SCFD1,ERC2,VPS53,DOCK1,DIAPH3,LYVE1,CCDC91,KIF13A,GOSR2,PLPP3,RABGAP1L,MGRN1,GOLPH3L,MYO1B,SCIN,ADAMTS9,KIF16B,RAB11FIP2,CDH2,PPARG,IL13RA2,SH3KBP1,MYO5A,SFRP4,BLTP1,VAV2,TFRC,VPS13A,RAB31,RAPGEF4,CLASP2,CLTC,ANGPT1,ATP6V0A4,ITGA4,DNAJC6,ROCK1,TBC1D4,SCFD2,SCARA5,MAGI2,ANKFY1,AP4S1,PRKD1,CALCRL,RAB6D,SCAMP1,RAB6C,CTBP2,NCALD,UNC13C,VAV1,VPS13B*
GO:0019538	protein metabolic process	0.022568736974333092	*C10ORF90,STYK1,RPL19,RTN1,HS3ST3A1,RGN,CDK14,MSRB3,MAP3K3,SMYD3,GARS1,ABR,PREP,MAP3K13,EIF1AX,PPM1A,SAMSN1,LIMD1,IMMP1L,CHST11,USP42,MAPK10,USP32,DAPP1,FGF1,CDC14C,AGTPBP1,SPINK5,MRPS24,PIGL,ATP7B,PPM1H,CAMK2B,TLK1,DDI2,OTUD7A,KALRN,SUMF1,MAP4K3,XXYLT1,FPGT-TNNI3K,GALNT9,FKBP5,WDR26,FOXO1,STYXL1,STK39,SMURF2,ADAMTS12,CDK18,YBX1,STK24,DIPK2A,FAM20B,ASB4,PRKN,RMND5A,F13A1,MARCHF9,IFT172,CBLL2,CDON,NLK,MRPL13,MAPKAPK3,CNMD,LRP8,ENPEP,UBE2O,CPEB4,EIF4ENIF1,MBTPS2,ADAR,TM9SF2,CDC37,FGF10,STK32B,PLG,HERC5,PGPEP1L,UBE2E2,NCK1,CD84,KLHL42,MTCP1,CHRM3,COL11A1,NCOA7,MARCHF1,BRAF,GEMIN5,EEF2KMT,ALPK2,CHRNA7,HECW1,TGFB2,TRIO,SMARCC1,NDST1,MUSK,MAP3K5,ALKBH1,ZCCHC4,PRKAR2A,CTIF,MRPL33,GOLPH3,PRKG2,MBTPS1,PIK3C3,PIGV,USP10,RSKR,EPHA5,PTPN20,RUNX1,PPTC7,STRADA,HUWE1,ASPH,SH3BGRL,UBR5,SPOCK3,GSN,SPON1,MAPRE3,TLR8,CACUL1,NLRP7,ROBO1,CPM,PAK3,NDUFAF7,CAMK1G,PRKAG2,CCNT2,CPQ,CNOT10,F8,CILK1,FOSL2,EPHA3,LATS2,SPDYE2B,MRPL32,ERP29,UVRAG,PTPRN2,LYN,USP6,SUMO2,DIP2B,LARGE1,PUS7,PAMR1,SIL1,MYRFL,RNASEL,CORIN,PPM1L,EIF3L,TNRC6C,ERCC6,NOS1AP,EIF2AK4,MSRA,MAGEL2,TGFBR2,MARK4,TCF7L2,MTRF1,OVCH1,UNC119,ULK3,SPDYE2,ADGRV1,SERBP1,SCG5,WAC,DAPK1,APP,IGF2BP1,PPIL6,GSKIP,RNF38,ADAM12,UBE2G1,PELI2,FRY,HERC2,USE1,PIK3R5,RIPK4,PTK2,USP7,CELF1,LRRK2,ARIH1,PLD1,UBXN7,PJA2,PAEP,COP1,ACVR2A,NEDD4L,BRCC3,DUSP14,PRMT8,RABGEF1,KIT,EPHA7,SPAG9,WNT5A,TRABD2B,CTNNB1,CTSK,PRKDC,IPO5,PTPN14,POFUT2,BCAR3,TPD52L1,PPM1E,TPST2,PARS2,HUNK,NEK10,ANAPC10,USP40,PKIG,EFNA5,IGF2BP2,CDC14B,HECTD1,VKORC1L1,TRPC5,NEUROD2,FNIP2,KAT14,UGGT2,UBE3B,TNFSF11,PTPRF,IKBKB,FXR2,APOH,RAB3GAP2,CE*

			MIP,ATF2,HDAC8,ACVR1,PID1,HYAL4,RB1,NSMCE1,PLPP3,IMMP2L,LAMP2,SPSB1,BNIP3L,PRKCH,XRCC5,GFRA1,BMX,ZNF418,TPST1,TMTC1,NRK,MGRN1,MID2,SIK1,FBXL13,KAT2B,TMTC2,TMPRSS11B,PRCP,UBE2E3,FARSB,ARNT,BDNF,TLR6,NDST4,ADAMTS9,EEFSEC,PPARG,PAPPA,ASB3,MTTP,USP9X,TGM5,NEK7,TENM1,CDK6,MYLK4,USP25,SPDYE16,TFRC,VPS13A,SPDYE6,MAP3K1,CLEC6A,FUT10,ANGPT1,EIF4G1,TMPRSS15,B3GAT2,ROCK1,MMP28,NUP98,PML,SACS,PRKG1,ELAVL4,RFPL4B,PRSS50,AIMP1,HSPBP1,PRKD1,USP34,STK4,TNNI3K,ST6GAL2,RPL27A,ANPEP,PRLR,PTPRA,NMI,TTN,DYRK1A,KCNE2,CUL4A,CHSY3,UBE3C,ATE1,METTL3,PARD3,CP,PGPEP1,RNF6,PEPD,DPY19L1
GO:0042325	regulation of phosphorylation	0.02277124724177408	*RGN,SMYD3,MAP3K13,SAMSN1,TF,FGF1,DIPK2A,PRKN,CDON,LRP8,ADAR,CDC37,FGF10,HERC5,NCK1,MTCP1,BRAF,CHRNA7,TGFB2,MUSK,MAP3K5,PRKAR2A,STRADA,MAPRE3,TLR8,CACUL1,ROBO1,PRKAG2,CCNT2,LATS2,SPDYE2B,ERP29,UVRAG,LYN,LDB2,ERCC6,TGFBR2,UNC119,SPDYE2,APP,GSKIP,PELI2,PIK3R5,PTK2,LRRK2,ACVR2A,RABGEF1,KIT,EPHA7,SPAG9,WNT5A,PRKDC,IPO5,BCAR3,TPD52L1,PPM1E,NEK10,PKIG,EFNA5,TRPC5,FNIP2,TNFSF11,IKBKB,FXR2,CEMIP,ATF2,ACVR1,PID1,RB1,PLPP3,XRCC5,GFRA1,BDNF,TLR6,PPARG,TENM1,SPDYE16,TFRC,SPDYE6,CLEC6A,ANGPT1,EIF4G1,ROCK1,PRKD1,STK4,PRLR,TTN,PARD3*
GO:0070727	cellular macromolecule localization	0.023595531433835172	*OSBPL8,STX8,SMYD3,RAPGEF6,PPM1A,ARL13B,RN7SL556P,IMMP1L,RCSD1,SPTBN1,GRIP1,TLK1,RGPD2,XPO4,USH2A,NPNT,EPB41L3,SHANK3,FOXO1,YBX1,PRKN,EXOC4,XPO6,GNAI1,INTS6-AS1,TRAK2,EIF4ENIF1,ERBIN,CD247,MCC,CEP192,ADAR,TM9SF2,CDC37,FGF10,ZFYVE16,ILDR2,KCNQ3,CHRM3,RGPD3,MCPH1,MYO1D,BRAF,XPO7,ATRX,TGFB2,MDFIC,MUSK,JAM3,KCNIP3,NUP93,TBC1D5,SLC15A4,GOLPH3,PRKG2,PIK3C3,EPHA5,SNX10,ATP2C1,STRADA,PCLO,HUWE1,ASPH,ZDHHC4,UBR5,GSN,PLS1,MAPRE3,ITPR1,DNAAF11,VPS8,MTX3,PACRG,EPHA3,LATS2,ERP29,PTPRN2,KLF7,SEC24C,LARGE1,NUP58,SIL1,TMED7-TICAM2,KCNIP4,NOMO2,RN7SL738P,MARK4,TCF7L2,UNC119,GPM6B,RRAGD,KPNB1,GSDMD,ADGRV1,EPB41L5,SCG5,WHRN,SAR1B,TTC7B,HCN1,HERC2,HOOK3,USE1,SCP2,HYCC1,LRRK2,GET1-SH3BGR,TIAM1,CSRP3,IQSEC2,NEDD4L,ENSG00000288683,SNX13,RABGEF1,CYB5R4,SPDL1,WNT5A,WIPI2,PEX26,ARFGEF2,CTNNB1,IPO5,ESR1,PTPN14,SCFD1,VPS53,FRMD4A,GPC4,PKIG,EFNA5,SYNE3,IKBKB,AKAP6,RAB3GAP2,TASOR,CEMIP,ATF2,PID1,CCDC91,GRID2,ZNF423,RB1,KIF13A,GOSR2,HNF1B,IMMP2L,LAMP2,BNIP3L,PRKCH,RABGAP1L,XRCC5,RAB18,MID2,GOLPH3L,WLS,NPHP1,FBN2,SRI,ATP1B1,BDNF,SCIN,ADAMTS9,RAB11FIP2,CDH2,PPARG,ASB3,MTTP,USP9X,TENM1,JAKMIP1,GDAP1,MYO5A,TFRC,VPS13A,FBN1,RAB31,RAPGEF4,BMPER*

			,FUT10,CLASP2,CLTC,ANGPT1,EIF4G1,ITGA4,ROCK1,TAX1BP1,NUP98,PML,SCFD2,SP100,NDUFAF2,MAGI2,NIN,ASIC2,ENSA,AP4S1,PRKD1,CALCRL,STK4,RAB6D,SCAMP1,RAB6C,TTN,PPFIA1,TMEM30A,MYRIP,PEX5L,PARD3,KCNN4,ATP8B1
GO:0007417	central nervous system development	0.0237215493799523	*RTN1,TRAPPC9,ARL13B,SPTBN1,CHRNB2,SEZ6,GNB4,AGTPBP1,SPINK5,DCT,CDH11,SHANK3,DAAM2,PLXNA4,PRKN,NLGN4X,KDM7A,IFT172,CHD7,CDON,SERPINI1,LRP8,TAGLN3,CNTN1,FGF10,KDM2B,MCPH1,ATRX,NDST1,EPHA5,SKOR2,ALDH1A2,GSN,ROBO1,NCKAP1,IL1RAPL2,LYN,GRIK1,LARGE1,CA10,TGFBR2,GPM6B,WHRN,APP,IGF2BP1,HOOK3,CELF1,LRRK2,RARB,PHACTR1,VIT,SNTG2,MEIS1,EPHA7,WNT5A,CTNNB1,PRKDC,LMX1A,BPGM,NEUROD2,FXR2,ARHGEF28,POU6F2,ATF2,GRID2,ZNF423,SHROOM4,PLPP3,HNF1B,IMMP2L,KIRREL3,DLC1,RAB18,WLS,CDK5RAP2,SEMA3E,SCIN,MACO1,CDH2,PPARG,CDK6,GABRB1,TFAP2D,FUT10,AFF2,PRKG1,ELAVL4,NIN,ASIC2,STK4,SEMA5A,TTPA,METTL3,VPS13B*
GO:0001932	regulation of protein phosphorylation	0.024564671976367605	*RGN,SMYD3,MAP3K13,SAMSN1,FGF1,DIPK2A,PRKN,CDON,LRP8,ADAR,CDC37,FGF10,HERC5,NCK1,MTCP1,BRAF,CHRNA7,TGFB2,MUSK,MAP3K5,PRKAR2A,STRADA,MAPRE3,TLR8,CACUL1,ROBO1,PRKAG2,CCNT2,LATS2,SPDYE2B,ERP29,UVRAG,LYN,ERCC6,TGFBR2,UNC119,SPDYE2,APP,GSKIP,PELI2,PIK3R5,PTK2,LRRK2,ACVR2A,RABGEF1,KIT,EPHA7,SPAG9,WNT5A,PRKDC,IPO5,BCAR3,TPD52L1,PPM1E,NEK10,PKIG,EFNA5,TRPC5,FNIP2,TNFSF11,FXR2,CEMIP,ATF2,ACVR1,PID1,RB1,PLPP3,XRCC5,GFRA1,BDNF,TLR6,PPARG,TENM1,SPDYE16,TFRC,SPDYE6,CLEC6A,ANGPT1,EIF4G1,PRKD1,STK4,PRLR,TTN,PARD3*
GO:0001944	vasculature development	0.02781459393816715	*EPN2,MAP3K3,ISM1,FGF1,SPINK5,NOTCH2,E2F7,FOXO1,ASB4,CHD7,THSD7A,CNMD,MEOX2,ENPEP,RHOJ,FGF10,TSPAN18,PLG,COL1A2,CHRNA7,ZNF304,TGFB2,NDST1,JAM3,RUNX1,ALDH1A2,ROBO1,TNFRSF12A,ADGRB3,LARGE1,NOS3,TGFBR2,TCF7L2,GATA4,ADAM12,PTK2,MEIS1,KIT,ANTXR1,WNT5A,CTNNB1,ANKRD17,PTPN14,ITGB8,HECTD1,APOH,ATF2,ACVR1,PLPP3,IMMP2L,PRCP,FLVCR1,SEMA3E,ADAMTS9,STIM1,CDH2,PPARG,JCAD,VAV2,BMPER,ANGPT1,SEMA6A,ROCK1,PML,SP100,AIMP1,PRKD1,CALCRL,STK4,ANPEP,SEMA5A,LAMA4,SEMA3C*
GO:0050982	detection of mechanical stimulus	0.02812395676195622	*ANO3,COL11A1,ADGRV1,WHRN,PTK2,CSRP3,KIT,CTNNB1,PKD1L3,TMEM120A,CDH2,ASIC2,TTN*
GO:0010557	positive regulation of macromolecule biosynthetic process	0.028780891945650592	*APBB2,ING3,NFIA,PHF20,FOXK2,SMYD3,PPM1A,TF,THRB,SPTBN1,ATF7,FGF1,RBPMS,GPBP1L1,BRD4,NOTCH2,HMBOX1,NPNT,NR4A3,ARID1B,E2F7,FOXO1,FER1L6,YBX1,IDO1,PRKN,KDM7A,CHD7,CDON,CNTN1,MEOX2,STAT5B,EIF4ENIF1,CALCR,MBTPS2,PTH,ADAR,TOX2,FGF10,MECOM,NCK1,CD84,RSF1,NCOA7,ZNF804A,BRAF,MRTFB,HMGB1,ZNF304,HIVEP1,ATRX,TGFB2,*

			MDFIC,HOXC13,SMARCC1,MUSK,SFPQ,MAP3K5,ZCCHC4,CTIF,RBM23,RUNX1,ALDH1A2,PTGFR,ASPH,SH3BGRL,UBR5,GTF2F2,SOX30,GSN,SPON1,MAPRE3,TLR8,ROBO1,KLF12,MLLT3,CCNT2,FOSL2,ERP29,KLF7,SUMO2,MTA3,LDB2,MYRFL,RNASEL,NOS3,MLLT10,ERCC6,NOS1AP,EIF2AK4,TCF7L2,ZNF462,ZFHX3,GSDMD,WAC,WHRN,APP,GATA4,SMAD5,IGF2BP1,TLR1,TICAM2,CELF1,POGZ,LRRK2,INO80D,ZNF395,IKZF3,PLD1,VTCN1,TENT5C,RARB,TCF4,PAEP,CSRP3,MLIP,ACVR2A,MEIS1,KIT,WNT5A,ARFGEF2,CTNNB1,PRKDC,ESR1,ITGB8,ZXDC,LMX1A,WWOX,PTGER4,IGF2BP2,NEUROD2,TNFSF11,IKBKB,FXR2,USF3,CCPG1,ATF2,ACVR1,PID1,EBF2,ZNF423,RB1,PDE4B,ZNF143,HNF1B,ZNF615,NCOA2,XRCC5,SHLD2,SLC24A3,TAF15,POU2F1,ETV1,MID2,ZNF850,KAT2B,MEF2D,GTF2A1,CDK5RAP2,RFC3,ATF3,ARNT,TFCP2L1,BCLAF3,TENT4B,TLR6,PPARG,NEK7,RORB,CDK6,TFAP2D,SFRP4,IL1RAP,TFRC,CLEC6A,ZNF292,ZNF521,ANGPT1,EIF4G1,ROCK1,NUP98,PML,TNFSF4,AFAP1L2,SP100,ELAVL4,PRKD1,IL16,CTBP2,TTN,DYRK1A,ELL2,PVT1,GATAD2B,METTL3,UAP1,ZNF33A,NPAS3,BMAL2,RNF6
GO:0035995	detection of muscle stretch	0.030488070667612298	*PTK2,CSRP3,CTNNB1,CDH2,TTN*
GO:0006811	monoatomic ion transport	0.030501678572565225	*ABCC6,SLC12A8,TF,CACNB1,CHRNB2,KCNQ5,FHL1,ATP10A,ATP7B,KCNJ6,CAMK2B,STK39,CACNA1C,SLC5A7,CHD7,SLC17A6,CNTN1,CHRFAM7A,CLCN3,CD84,GABRA5,KCNQ3,ANO3,SESTD1,KCNS3,CHRNA7,KCNJ18,KCNIP3,ATP4A,SLC15A4,PRKG2,ATP13A4,ATP2C1,ASPH,ANO2,ITPR1,CACNA2D3,LYN,GRIK1,LARGE1,ATP6V1H,SLC12A1,SLC41A2,NOS3,KCNIP4,NOS1AP,CACNA2D4,OCA2,CACNA2D1,HCN1,SLC13A1,TMCO3,ATP1B4,NKAIN3,SLC38A4,NEDD4L,CTNNB1,BEST3,PLCH1,TRPC5,AKAP6,CLCN1,CEMIP,KCNJ12,CHRNB3,GRID2,KCNJ3,PDE4B,PKD1L3,GABRB3,UNC80,GABRG2,TMEM120A,KCND2,SLC24A3,GRIA4,SIK1,RYR3,FLVCR1,TRDN,SLC26A8,CACHD1,SRI,ATP1B1,STIM1,CHRNB4,GABRB1,NALCN,SLC6A13,TFRC,GRIA3,CLTC,ATP6V0A4,KCNT2,PML,SCARA5,SLC5A12,ASIC2,SLC30A10,PRKD1,CALCRL,SLC20A2,IL16,KCNE2,KCNN4,CP,ATP8B1,GRIA2,VDAC2*
GO:0051049	regulation of transport	0.03170399658935262	*OSBPL8,PPM1A,TF,CACNB1,CHRNB2,FHL1,KCNJ6,CAMK2B,XPO4,KALRN,NR4A3,FOXO1,STK39,CACNA1C,PRKN,CHD7,CNTN1,CHRFAM7A,PTH,FGF10,ZFYVE16,CD84,CHRM3,SESTD1,BRAF,HMGB1,DYNLT2B,TGFB2,MDFIC,KCNJ18,KCNIP3,TBC1D5,ATP4A,GOLPH3,PRKG2,MBTPS1,EPHA5,ATP2C1,STON2,PCLO,ASPH,UBR5,ITPR1,PRKAG2,EPHA3,ERP29,PTPRN2,LYN,USP6,KLF7,NUP58,RNASEL,CORIN,NOS3,KCNIP4,NOS1AP,TCF7L2,UNC119,GPM6B,SCG5,DAPK1,SAR1B,APP,CACNA2D1,AVPR1B,USP7,SCP2,LRRK2,NKAIN3,PLD1,TIAM1,NEDD4L,RABGEF1,WNT5A,CTNNB1,IPO5,PTPN14,SCFD1,ERC2,POFUT2,FRMD4A,BEST3,PKIG,EFNA5,TNFSF11,AKAP6,CEMIP,KCNJ12,PID1,KCNJ3,PDE4B,SIK1,GOLPH3*

			L,WLS,ARHGEF7,TRDN,SRI,ATP1B1,SCIN,STIM1,CDH2,PPARG,CHRNB4,TENM1,IL13RA2,MYOF,SFRP4,OSBPL6,RAB31,RAPGEF4,CLASP2,ANGPT1,DNAJC6,ROCK1,TBC1D4,PML,SP100,PRKG1,NDUFAF2,MAGI2,ANKFY1,ASIC2,ENSA,AIMP1,PRKD1,IL16,TTN,TMEM30A,MYRIP,KCNE2,KCNN4,ATP8B1
GO:0045595	regulation of cell differentiation	0.03432761399287378	MAP3K13,LIMD1,SPINK5,BRD4,CAMK2B,DCT,KALRN,USH2A,NOTCH2,NPNT,MAP6,SHANK3,ARID1B,FOXO1,ADAMTS12,DAAM2,PLXNA4,YBX1,ASB4,CHD7,PTBP3,CDON,TIAM2,LRP8,STAT5B,EIF4ENIF1,PTH,FGF10,NREP,BRAF,HMGB1,RFLNA,TGFB2,LAMA2,TRIO,SMARCC1,MAP3K5,ATP11A,PRKG2,RUNX1,ROBO1,ABI3BP,CCNT2,FOSL2,EPHA3,LYN,KLF7,MTA3,DIP2B,PUS7,PRAMEF27,EIF2AK4,TGFBR2,TCF7L2,ZFHX3,CUX1,ADGRV1,ST7,PRUNE1,APP,SMAD5,HOOK3,PTK2,LRRK2,IKZF3,TENT5C,RARB,TCF4,PAEP,TIAM1,CSRP3,ACVR2A,MEIS1,KIT,EPHA7,SPAG9,WNT5A,CTNNB1,ANKRD17,RUNX1T1,PRKDC,POFUT2,LMX1A,VWC2L,EFNA5,TRPC5,NEUROD2,TNFSF11,IKBKB,AKAP6,USF3,ACVR1,RB1,ADIPOR1,PRKCH,XRCC5,SIK1,CDK5RAP2,FBN2,ARNT,BDNF,LDLRAD4,SEMA3E,SCIN,ADAMTS9,LAG3,PPARG,RORB,CDK6,SFRP4,BLTP1,FBN1,IQCB1,CLASP2,SEMA6A,EIF4G1,ROCK1,TNFSF4,NIN,PRKD1,STK4,PRLR,CUL4A,SEMA5A,TTPA,GATAD2B,METTL3,RNF6,SEMA3C,SMYD1,SPART
GO:0048588	developmental cell growth	0.03537535723166448	MAP3K13,PLXNA4,PRKN,SLIT3,DIP2B,EIF2AK4,TGFBR2,APP,GATA4,TIAM1,MEIS1,NEDD4L,EPHA7,SPAG9,WNT5A,CTNNB1,EFNA5,TRPC5,AKAP6,BDNF,SEMA3E,USP9X,CLASP2,SEMA6A,ITGA4,PRKG1,NIN,SEMA5A,RNF6,SEMA3C,SPART
GO:0032535	regulation of cellular component size	0.03696753784375084	SLC12A8,MAP3K13,SPTBN1,CDC42EP3,TMOD2,SHANK3,STK39,DAAM2,PLXNA4,CLCN3,NCK1,PREX1,TMOD3,ADD3,GSN,PLS1,PAK3,NCKAP1,ARHGAP18,DIP2B,SLC12A1,SPTB,EPHA7,WNT5A,EFNA5,TRPC5,CAPZA2,BDNF,SEMA3E,SCIN,TENM1,AVIL,SPTBN5,VAV2,CLASP2,SEMA6A,PRKD1,SEMA5A,KCNN4,RNF6,SEMA3C,VAV1,SPART
GO:0072359	circulatory system development	0.03708822245975397	EPN2,MAP3K3,ISM1,ARL13B,FGF1,SPINK5,NOTCH2,CDH11,E2F7,FOXO1,PLXNA4,DIPK2A,CACNA1C,ASB4,IFT172,CHD7,THSD7A,CNMD,MEOX2,ENPEP,RHOJ,FGF10,TSPAN18,PLG,COL11A1,COL1A2,ALPK2,CHRNA7,ZNF304,TGFB2,NDST1,JAM3,RUNX1,ALDH1A2,SLIT3,ROBO1,ABI3BP,TNFRSF12A,ADGRB3,LARGE1,NOS3,TGFBR2,TCF7L2,GATA4,SMAD5,ADAM12,PTK2,RARB,CSRP3,MEIS1,KIT,ANTXR1,WNT5A,CTNNB1,ANKRD17,PRKDC,PTPN14,ITGB8,GREB1L,HECTD1,AKAP6,APOH,ATF2,ACVR1,RB1,PLPP3,IMMP2L,ACACB,DLC1,SIK1,KAT2B,PRCP,MEF2D,FLVCR1,SRI,SEMA3E,ADAMTS9,STIM1,CDH2,PPARG,JCAD,VAV2,FBN1,BMPER,ANGPT1,SEMA6A,ROCK1,PML,SP100,PRKG1,AIMP1,PRKD1,CALCRL,STK4,ANPEP,TTN,SEMA5A,LAMA4,SEMA3C,SMYD1
GO:0007	cell	0.03725515	FSTL1,NFIA,PPM1A,CHST11,SPTBN1,RBPMS,N

178	surface receptor protein serine/threonine kinase signaling pathway	1562831094	OTCH2,NPNT,SMURF2,NLK,UBE2O,FGF10,NREP,COL1A2,HIVEP1,TGFB2,NUP93,SKOR2,LATS2,PPM1L,TGFBR2,GATA4,SMAD5,PTK2,ACVR2A,WNT5A,ITGB8,VWC2L,ATF2,ACVR1,ZNF423,FBN2,LDLRAD4,PPARG,USP9X,SFRP4,FBN1,BMPER,PML,MAGI2,PMEPA1,SPART
GO:0031399	regulation of protein modification process	0.04236189011710706	RGN,SMYD3,MAP3K13,SAMSN1,FGF1,DIPK2A,PRKN,CDON,LRP8,ADAR,CDC37,FGF10,HERC5,NCK1,MTCP1,NCOA7,BRAF,CHRNA7,TGFB2,MUSK,MAP3K5,PRKAR2A,STRADA,HUWE1,MAPRE3,TLR8,CACUL1,ROBO1,PRKAG2,CCNT2,LATS2,SPDYE2B,ERP29,UVRAG,LYN,DIP2B,ERCC6,NOS1AP,TGFBR2,UNC119,SPDYE2,APP,GSKIP,PELI2,FRY,PIK3R5,PTK2,LRRK2,ACVR2A,RABGEF1,KIT,EPHA7,SPAG9,WNT5A,TRABD2B,CTNNB1,PRKDC,IPO5,BCAR3,TPD52L1,PPM1E,NEK10,PKIG,EFNA5,CDC14B,TRPC5,FNIP2,KAT14,TNFSF11,IKBKB,FXR2,RAB3GAP2,CEMIP,ATF2,HDAC8,ACVR1,PID1,RB1,PLPP3,XRCC5,GFRA1,KAT2B,ARNT,BDNF,TLR6,PPARG,TENM1,SPDYE16,TFRC,SPDYE6,CLEC6A,ANGPT1,EIF4G1,PML,HSPBP1,PRKD1,STK4,PRLR,NMI,TTN,DYRK1A,PARD3
GO:0031401	positive regulation of protein modification process	0.042447178979586234	SMYD3,MAP3K13,FGF1,DIPK2A,PRKN,CDON,LRP8,FGF10,MTCP1,BRAF,CHRNA7,TGFB2,MUSK,MAP3K5,STRADA,HUWE1,MAPRE3,CACUL1,ROBO1,PRKAG2,SPDYE2B,ERP29,LYN,DIP2B,ERCC6,NOS1AP,TGFBR2,UNC119,SPDYE2,APP,PELI2,PIK3R5,PTK2,LRRK2,ACVR2A,KIT,EPHA7,WNT5A,TRABD2B,BCAR3,TPD52L1,NEK10,EFNA5,CDC14B,TRPC5,FNIP2,TNFSF11,FXR2,RAB3GAP2,CEMIP,ATF2,ACVR1,PLPP3,XRCC5,GFRA1,ARNT,BDNF,TLR6,TENM1,SPDYE16,TFRC,SPDYE6,CLEC6A,ANGPT1,EIF4G1,PML,HSPBP1,PRKD1,STK4,PRLR,NMI,DYRK1A
GO:0001568	blood vessel development	0.04407599299502049	EPN2,MAP3K3,ISM1,FGF1,SPINK5,NOTCH2,E2F7,FOXO1,ASB4,CHD7,THSD7A,CNMD,MEOX2,ENPEP,RHOJ,FGF10,TSPAN18,PLG,COL1A2,CHRNA7,ZNF304,TGFB2,NDST1,JAM3,RUNX1,ALDH1A2,ROBO1,TNFRSF12A,ADGRB3,LARGE1,NOS3,TGFBR2,TCF7L2,GATA4,ADAM12,PTK2,MEIS1,ANTXR1,WNT5A,CTNNB1,ANKRD17,ITGB8,HECTD1,APOH,ATF2,ACVR1,PLPP3,PRCP,FLVCR1,SEMA3E,ADAMTS9,STIM1,CDH2,PPARG,JCAD,VAV2,BMPER,ANGPT1,SEMA6A,ROCK1,PML,SP100,AIMP1,PRKD1,CALCRL,STK4,ANPEP,SEMA5A,LAMA4,SEMA3C
GO:0007169	cell surface receptor protein tyrosine kinase signaling pathway	0.04422690147441535	EPN2,OSBPL8,FGF1,DOK4,KALRN,NR4A3,FOXO1,ADAMTS12,FAM83B,MAPKAPK3,STAT5B,ERBIN,FGF10,COL4A6,NCK1,BRAF,SMARCC1,NDST1,MUSK,EPHA5,ANKS1B,ROBO1,PAK3,DOK6,EPHA3,LYN,SHISA2,GKAP1,PTK2,SH2B2,PIP4K2B,TIAM1,CSRP3,RABGEF1,KIT,EPHA7,WNT5A,CTNNB1,BCAR3,EFNA5,NGEF,ARHGEF28,PID1,ADIPOR1,GFRA1,ANKS1A,ARHGEF7,SAMD12,ARNT,BDNF,KIF16B,JCAD,VAV2,ANGPT1,SEMA6A,AFAP1L2,SLC30A10,PRKD1,PRLR,PTPRA,HHIP,VAV1

GO:2000026	regulation of multicellular organismal development	0.04754857849368754	*RGN,EPN2,LRRN1,ISM1,MAP3K13,FGF1,SPINK5,BRD4,CAMK2B,DCT,KALRN,NOTCH2,MAP6,SHANK3,ARID1B,ADAMTS12,DAAM2,PLXNA4,CHD7,CDON,TIAM2,CNMD,LRP8,STAT5B,PTH,RHOJ,FGF10,TSPAN18,BRAF,CHRNA7,HMGB1,ZNF304,RFLNA,TGFB2,LAMA2,SMARCC1,PRKG2,RUNX1,ROBO1,LYN,ADGRB3,KLF7,DIP2B,SGMS2,NOS3,TGFBR2,CUX1,GPM6B,ADGRV1,PRUNE1,GATA4,ADAM12,HOOK3,INO80D,IKZF3,CLSTN2,RARB,TIAM1,ACVR2A,MEIS1,KIT,EPHA7,WNT5A,CTNNB1,CTSK,PRKDC,ITGB8,EFNA5,TRPC5,KAT14,TNFSF11,IKBKB,AKAP6,APOH,LINGO2,ATF2,ACVR1,GRID2,RB1,PRKCH,ACACB,XRCC5,LRTM2,KAT2B,FBN2,ARNT,BDNF,SEMA3E,SCIN,ADAMTS9,STIM1,LAG3,PPARG,CDK6,SFRP4,BLTP1,IL1RAP,JCAD,FBN1,BMPER,CLASP2,SEMA6A,ROCK1,PML,TNFSF4,SP100,NIN,ASIC2,PRKD1,SLC20A2,PRLR,CUL4A,SEMA5A,LAMA4,METTL3,PARD3,RNF6,SEMA3C,SPART*
GO:0001934	positive regulation of protein phosphorylation	0.04928461412771839	*SMYD3,MAP3K13,FGF1,DIPK2A,CDON,LRP8,FGF10,MTCP1,BRAF,CHRNA7,TGFB2,MUSK,MAP3K5,STRADA,MAPRE3,CACUL1,ROBO1,PRKAG2,SPDYE2B,ERP29,LYN,ERCC6,TGFBR2,UNC119,SPDYE2,APP,PELI2,PIK3R5,PTK2,LRRK2,ACVR2A,KIT,EPHA7,WNT5A,BCAR3,TPD52L1,NEK10,EFNA5,TRPC5,FNIP2,TNFSF11,FXR2,CEMIP,ATF2,ACVR1,PLPP3,XRCC5,GFRA1,BDNF,TLR6,TENM1,SPDYE16,TFRC,SPDYE6,CLEC6A,ANGPT1,EIF4G1,PRKD1,STK4,PRLR*
		CC	
GO:0016020	membrane	7.891823410629205e-14	*C10ORF90,APBB2,PFKP,ABCC6,ESYT1,STYK1,RPL19,RTN1,HS3ST3A1,TRGC2,CDK14,EPN2,MREG,NOSTRIN,WSCD1,SLC12A8,OSBPL8,LRRN1,POTED,STX8,PHF20,CPNE4,RAPGEF6,ABR,MAOA,SLC16A5,TMEM255A,PREP,MAP3K13,PPM1A,ARL13B,THADA,LIMD1,IMMP1L,TF,CHST11,SPTBN1,CDC42EP3,MAPK10,CACNB1,USP32,DAPP1,GNG12,CHRNB2,CDC14C,SEZ6,CLDN6,KCNQ5,CDH8,ARHGAP15,GNB4,FHL1,ATP10A,SPINK5,MRPS24,SKAP2,PRND,PIGL,ATP7B,KCNJ6,ZAN,TNRC18,CAMK2B,GRIP1,DCT,DLGAP1,PECR,SLC38A9,SDR16C5,ABCG8,KALRN,USH2A,XXYLT1,GALNT9,NOTCH2,CD93,FKBP5,SLC44A5,SDK2,NPNT,DSC1,EPB41L3,MAP6,PARVB,CDH11,SHANK3,SPTLC3,ARID1B,SLC14A2,STK39,SMURF2,SRL,FER1L6,PLXNA4,YBX1,STK24,DIPK2A,CACNA1C,FAM20B,PRKN,NLGN4X,EXOC4,PNPLA3,PIK3C2B,XPO6,SLC44A3,SLC5A7,MARCHF9,FAM83B,GPSM1,CDON,SLC17A6,THSD7A,GNAI1,MRPL13,NCAM2,PAPOLG,TIAM2,CFAP54,TRAK2,CNMD,LRP8,CNTN1,ENPEP,ARHGEF4,MUC17,EIF4ENIF1,CALCR,CHRFAM7A,ELMO1,ERBIN,MBTPS2,CD247,MCC,ADAR,DNAH10,TM9SF2,CLCN3,RHOJ,FGF10,ZFYVE16,COL4A6,CATSPERB,TSPAN18,PLG,CNTNAP5,CDH7,ILDR2,NCK1,CD84,NONO,RARRES1,EXO1,GABRA5,KCNQ3,CHRM3,PREX1,AIG1,WRNIP1,ANO3,TYW1,GOLGA8R,KCTD3,C2ORF88,SERTM2,ZNF804A,CIDEB,MARCHF1,SLC45A1,MYO1D,SESTD1,BRAF,DTNA,GEMIN5,KCNS3,ALPK2,CHRNA7,HMGB1,CDIP1,LPP,MDFIC,LAMA2,ADD3,TRIO,NDST1,MUSK,*

			KCNJ18,JAM3,KCNIP3,PNPLA4,MAP3K5,RGS3,NUP93,RERG,OR5AU1,PRKAR2A,TBC1D5,ATP11A,MRPL33,RBM23,ATP4A,SLC15A4,PCDHB8,GOLPH3,PRKG2,MBTPS1,NCF2,PIK3C3,PIGV,SND1,HMCN1,EPHA5,PTPN20,ATP13A4,ENAH,SNX10,ATP2C1,TMEM230,STON2,PTGFR,IGHV1OR21-1,FAM171A1,PCLO,HUWE1,ASPH,RRAS2,SH3BGRL,ZDHHC4,UBR5,TMEM132C,ANKS1B,CHL1,GSN,PLS1,ANO2,TLR8,ARMH3,ARHGEF39,ROBO1,ITPR1,PKP4,CPM,PAK3,CYP2A13,TMEM183A,CAMK1G,PRUNE2,PRKAG2,CCNT2,HAVCR1,CNOT10,MR1,F8,TNFRSF12A,OASL,GDPD1,CPT2,TAS2R14,VPS8,MTX3,CACNA2D3,CXCR5,NCKAP1,PRPF40A,CLDN12,CNTN3,MGAM,PACRG,IL1RAPL2,ADGRD1,EPHA3,CHCHD3,MYH14,MRPL32,ERP29,LRRTM4,ARHGAP18,UVRAG,PTPRN2,LYN,ADGRB3,USP6,GRIK1,UBL3,DIP2B,SEC24C,LARGE1,ATP6V1H,NUP58,SLC12A1,PDZD8,LDB2,MYRFL,SPTB,UNC93A,FMO5,PAQR5,CORIN,TMCC1,LRRC7,PCDHB16,PPM1L,SGMS2,TMED7-TICAM2,SLC41A2,NOS3,KCNIP4,EIF3L,NOMO2,BPHL,NOS1AP,KIAA1614,MSRA,SYNPR,RAB12,HMGCLL1,XKR6,MAGEL2,TGFBR2,CMKLR2,ITGA11,MDN1,SYNJ1,CUX1,SHISA2,GPM6B,RRAGD,ULK3,GPR156,CACNA2D4,LHFPL3,KPNB1,GSDMD,ADGRV1,RASGRF2,ST7,SERBP1,QTRT2,EVA1C,EPB41L5,GAD1,WHRN,OCA2,DAPK1,ACSM2B,SAR1B,TTC7B,APP,ADAM12,ATP9B,CACNA2D1,HCN1,CHRM5,SLC13A1,PI4KB,RHOT1,ELOVL5,HERC2,HOOK3,USE1,PIK3R5,PALLD,TLR1,TAS2R30,TMCO3,RIPK4,AVPR1B,LMAN1L,FRRS1,PTK2,TICAM2,SCP2,SH2B2,ATP1B4,AVEN,CELF1,HYCC1,PIP4K2B,UQCC1,LRRK2,NKAIN3,GET1-SH3BGR,SLC38A4,CLSTN2,PLD1,ITFG2,VTCN1,PKHD1,UBXN7,PJA2,MGST1,COP1,TIAM1,MLIP,ACVR2A,GNAO1,ASAP2,ENSG00000288635,SNTG2,NTM,NEDD4L,ENSG00000288683,PRMT8,SNX13,RABGEF1,CYB5R4,KIT,TBC1D8,EPHA7,SPAG9,OSBPL3,ANTXR1,WNT5A,TRABD2B,WIPI2,PEX26,ARFGEF2,TEX2,CTNNB1,ANKRD17,CTSK,PRKDC,IPO5,ESR1,TNFRSF19,SCFD1,ITGB8,ERC2,POFUT2,BCAR3,VPS53,WWOX,TPST2,BTBD8,FILIP1,TMEM62,PTGER4,GREB1L,VWC2L,BEST3,LYPD6B,DOCK1,DIAPH3,CCDC107,GPC4,EFNA5,CDC14B,SYNE3,PLCH1,OR1M1,VKORC1L1,NGEF,TRPC5,FNIP2,JAML,TNFSF11,PTPRF,IKBKB,FXR2,SH3BP5,AKAP6,TRBV10-2,LYVE1,ARHGEF28,OR8B8,CLCN1,RAB3GAP2,STEAP1B,CCPG1,GPRIN1,LINGO2,OPCML,CEMIP,ATF2,KCNJ12,CHRNB3,ACVR1,HLA-DQA2,HYAL4,CCDC91,GRID2,KCNJ3,MPV17,IFNLR1,NTNG1,PDE4B,PKD1L3,KIF13A,ADIPOR1,GABRB3,GOSR2,SHROOM4,PLPP3,UNC80,IMMP2L,LAMP2,SSPN,KIRREL3,GABRG2,F5,CFAP95,CPS1,TMEM120A,BNIP3L,PRKCH,ACACB,AGPAT5,RABGAP1L,DLC1,DENND2B,XRCC5,RGS17,GFRA1,KCND2,INPP4A,BMX,SLC24A3,TPST1,GRIA4,RAB18,TMTC1,NRK,RGS7BP,MGRN1,LRTM2,GOLPH3L,TMTC2,WLS,IGKV1-

			6,RYR3,TMPRSS11B,PRCP,MYO1B,ZPLD1,MGAM2,SPTLC1,CDK5RAP2,FLVCR1,FARSB,ARHGEF7,SAMD12,TRDN,GASK1A,NPHP1,SLC26A8,CAPZA2,SNTG1,CACHD1,SRI,ATP1B1,GREB1,LDLRAD4,TFCP2L1,MALRD1,TENT4B,MACO1,TLR6,NDST4,STIM1,TMEM237,TMEM161B,KIF16B,RAB11FIP2,CDH2,COX7B2,LAG3,ENSG00000290217,ASB3,MTTP,CHRNB4,USP9X,TGM5,TENM1,IL13RA2,GABRB1,MYOF,LTB4R2,JAKMIP1,SH3KBP1,NALCN,SMOX,LPCAT2,GDAP1,MYLK4,KIF2C,MYO5A,SIPA1L3,SLC6A13,LRFN2,SPTBN5,BLTP1,IL1RAP,JCAD,VAV2,TFRC,VPS13A,TES,OSBPL6,GRIA3,PNPLA8,RAB31,COA1,CLEC6A,RAPGEF4,FUT10,CLASP2,CLTC,ANGPT1,SEMA6A,VIPR2,EIF4G1,TMPRSS15,B3GAT2,ATP6V0A4,PHLPP1,ITGA4,DNAJC6,ROCK1,NCAPH,M1AP,KCNT2,NUP98,PML,LAYN,MGLL,SCARA5,SLC5A12,PLEK2,TNFSF4,AFAP1L2,DGKG,SPATA25,PRKG1,PLXDC2,SBF2,CDH9,NDUFAF2,UMODL1,MAGI2,PCDH19,NIN,SPG21,ZNF286A,ANKFY1,ASIC2,FNDC3A,AIMP1,SLC30A10,AP4S1,PRKD1,CALCRL,SLC20A2,PCDH10,TMEM131,ST6GAL2,SCAMP1,PTCHD4,RPL27A,ANPEP,PRLR,PTPRA,NMI,CLVS1,EYS,IL16,DIPK1A,POTEH,GNA14,CR2,LPAR6,NCALD,HHIP,TMEM30A,KCNE2,UNC13C,SERINC1,KCTD16,CHSY3,DLGAP2,MAP7D3,SEMA5A,LAMA4,PMEPA1,ETNK1,PEX5L,UAP1,PARD3,KCNN4,TRDC,SLC25A48,CP,FUNDC1,ATP8B1,RNF6,VAV1,DPY19L1,GRIA2,VDAC2,VPS13B,SPART,IGLV4-3
GO:0005737	cytoplasm	8.163182794450631e-14	FSTL1,C10ORF90,APBB2,PFKP,ABCC6,ESYT1,RPL19,RTN1,HS3ST3A1,RGN,CDK14,EPN2,MSRB3,PDE1C,MREG,NOSTRIN,ENSG00000290149,MAP3K3,NINL,WSCD1,OSBPL8,SUGCT,STX8,PHF20,FOXK2,TXNDC8,SMYD3,GARS1,RAPGEF6,ARHGEF9,TAFA2,ABR,MAOA,BLTP3B,TRAPPC9,PREP,MAP3K13,EIF1AX,PPM1A,ARL13B,SAMSN1,RN7SL556P,THADA,LIMD1,IMMP1L,TF,CHST11,MYBPC1,USP42,IPP,RCSD1,SPTBN1,CDC42EP3,MYO5C,MAPK10,USP32,AHCYL2,DAPP1,ATF7,FGF1,CDC14C,SEZ6,KCNQ5,ARHGAP15,GNB4,AGTPBP1,FHL1,RBPMS,ATP10A,SH3PXD2A,SPINK5,MRPS24,OPA3,SKAP2,PIGL,ATP7B,PPM1H,KCNJ6,DOK4,TNRC18,CAMK2B,GRIP1,ARHGAP11A-SCG5,DDI2,PGM1,DCT,PECR,SLC38A9,SDR16C5,RGPD2,XPO4,OTUD7A,KALRN,SUMF1,USH2A,MAP4K3,XXYLT1,GALNT9,NOTCH2,CD93,FKBP5,TMOD2,HMBOX1,NPNT,DSC1,EPB41L3,MAP6,PARVB,CDH11,SHANK3,WDR26,SPTLC3,ARID1B,FOXO1,STYXL1,STK39,SMURF2,SRL,TBC1D16,CDK18,YBX1,STK24,DIPK2A,CFAP70,DCDC1,CACNA1C,FAM20B,FAM3C,ASB4,IDO1,ARB2A,PRKN,EXOC4,RMND5A,PNPLA3,F13A1,PIK3C2B,XPO6,SLC5A7,MARCHF9,FAM83B,IFT172,GPSM1,CBLL2,SERPINI1,SLC17A6,NLK,SDF4,GNAI1,MRPL13,MAPKAPK3,PAPOLG,TIAM2,CFAP54,INTS6-AS1,TRAK2,AGL,MEOX2,STAT5B,ENPEP,NUDT19,ARHGEF4,UBE2O,MUC17,DDIAS,CPEB4,EIF4ENIF1,CALCR,ELMO1,ERBIN,MBTPS2,RGS22,C

			D247,MCC,CEP192,ADAR,DNAH10,PDE10A,TM9SF2,PDHX,CLCN3,CDC37,FGF10,ZFYVE16,MECOM,RSPH10B,COL4A6,NLRP8,PLG,NEURL4,HERC5,NUDT13,PGPEP1L,ILDR2,NCK1,KLHL42,NREP,GABRA5,NEXMIF,KCNQ3,SH3BGR,MROH6,CHRM3,PREX1,RGPD3,WRNIP1,COL24A1,GOLGA8R,COL11A1,ZNF804A,SEPTIN10,CIDEB,MARCHF1,MCPH1,MYO1D,BRAF,COL1A2,DTNA,TMOD3,GEMIN5,EEF2KMT,XPO7,KCNS3,VWA3B,COL12A1,ABLIM1,RBMS1,HECW1,HMGB1,DYNLT2B,CDIP1,AFF3,RFLNA,LPP,HIVEP1,TGFB2,MDFIC,EPB41L4A,ADD3,TRIO,SMARCC1,NDST1,JAM3,KCNIP3,SFPQ,PNPLA4,MAP3K5,ALKBH1,ARPP21,RGS3,NUP93,ZCCHC4,RERG,PRKAR2A,RBM33,TBC1D5,CTIF,ATP11A,MRPL33,SLC15A4,GOLPH3,PRKG2,MBTPS1,NCF2,PIK3C3,PIGV,USP10,SND1,HMCN1,IGFBP7,EPHA5,PTPN20,SKOR2,CBR4,ATP13A4,ENAH,SNX10,ATP2C1,ALDH1A2,TMEM230,PPTC7,STON2,PTGFR,STRADA,PCLO,HUWE1,ASPH,RRAS2,SH3BGRL,ZDHHC4,UBR5,KIAA1217,SOX30,ANKS1B,GSN,PLS1,SPON1,MAPRE3,TLR8,HPS3,KIF21A,SLIT3,ARMH3,NLRP7,ROBO1,ANKRD11,ITPR1,PKP4,DNAAF11,PAK3,NDUFAF7,KLF12,AXDND1,IPMK,CYP2A13,CAMK1G,PRUNE2,DOK6,PRKAG2,MLLT3,CCNT2,BTG4,TRAPPC10,CPQ,CNOT10,MR1,KRTAP21-2,F8,OASL,GDPD1,CPT2,VPS8,MTX3,NCKAP1,DTD1,MGAM,CILK1,PACRG,ADGRD1,EPHA3,CHCHD3,LATS2,TYW3,MYH14,MRPL32,TPRG1,ERP29,ARHGAP18,UVRAG,PTPRN2,MYOM2,LYN,USP6,KLF7,MTA3,DIP2B,NME7,SEC24C,LARGE1,ATP6V1H,FIGNL1,PDZD8,CCDC33,RRM2,SIL1,MYRFL,SPTB,ALDH1A1,NMNAT3,FMO5,RNASEL,NAV1,DBF4B,TMCC1,LRRC7,DCP2,PPM1L,SGMS2,TMED7-TICAM2,NOS3,KCNIP4,EIF3L,NOMO2,TNRC6C,PRAMEF27,MLLT10,BPHL,NOS1AP,EIF2AK4,RN7SL738P,KIAA1614,MSRA,SYNPR,HMGCLL1,MAGEL2,TGFBR2,CFAP77,MARK4,DDX60L,MDN1,SYNJ1,CHAF1B,MTRF1,ECHDC2,ZFHX3,CUX1,POLR2C,UNC119,SHISA2,RRAGD,ULK3,GKAP1,PPP2R5E,KPNB1,GSDMD,ADGRV1,RASGRF2,SERBP1,QTRT2,EPB41L5,GAD1,SCG5,NUDCD1,WHRN,OCA2,DAPK1,TDP2,ALDH9A1,ACSM2B,PRUNE1,SAR1B,TTC7B,BTG3,APP,SMAD5,IGF2BP1,PPIL6,MAMLD1,GSKIP,UBE2G1,NBPF12,PELI2,ATP9B,CACNA2D1,PI4KB,FRY,RHOT1,ELOVL5,HERC2,HOOK3,USE1,PIK3R5,PALLD,TLR1,BMT2,RIPK4,AVPR1B,LMAN1L,QDPR,PTK2,USP7,TICAM2,SCP2,SH2B2,AVEN,GATD3,CELF1,HYCC1,DNAH12,POGZ,PIP4K2B,UQCC1,LRRK2,ZNF395,IKZF3,GET1-SH3BGR,CLSTN2,ARIH1,CFAP298,PLD1,ITFG2,PKHD1,TENT5C,UBXN7,RARB,PJA2,MGST1,RBM44,ARHGEF38,COP1,TIAM1,CSRP3,PHACTR1,MLIP,IQSEC2,ACVR2A,ASAP2,TIPIN,SNTG2,AMMECR1,NEDD4L,BRCC3,ENSG00000288683,SNX13,RABGEF1,CYB5R4,KIT,SPAG9,OSBPL3,ANTXR1,SPDL1,WNT5A,WIPI2,PEX26,ARFGEF2,TEX2,CTNNB1,ANKRD17,COL19A1,CTSK,PRKDC,IPO5,ESR1,PTPN14,SCFD1,ERC2,POFUT2,DMG

			DH,BCAR3,TPD52L1,PPM1E,VPS53,FRMD4A,DCAF8L1,WWOX,TPST2,FIGN,BTBD8,PARS2,BNIPL,HUNK,FILIP1,ANAPC10,FBXO15,PDE7B,USP40,DOCK1,DIAPH3,GPC4,BPGM,PKIG,IGF2BP2,ACTR3B,CDC14B,SYNE3,PLCH1,VKORC1L1,NGEF,FNIP2,AASS,KAT14,UGGT2,CCNJ,TNFSF11,IKBKB,FXR2,SH3BP5,AKAP6,APOH,ARHGEF28,RAB3GAP2,STEAP1B,EML6,CEMIP,ATF2,HDAC8,HLA-DQA2,PID1,HYAL4,CCDC91,ZCCHC17,RB1,MPV17,PDE4B,KIF13A,NLRP4,GABRB3,GOSR2,PET117,SHROOM4,SMG7,PLPP3,IMMP2L,LAMP2,SSPN,SPSB1,KIRREL3,GABRG2,F5,CFAP95,AK5,CPS1,NCOA2,BNIP3L,PRKCH,ACACB,AGPAT5,RABGAP1L,DLC1,DENND2B,XRCC5,TBRG4,RGS17,GFRA1,SPHKAP,INPP4A,BMX,MPRIP,TAF15,TPST1,GRIA4,POLD3,RAB18,TPH2,POU2F1,TMTC1,RGS7BP,MGRN1,MID2,SIK1,FBXL13,ERCC6L2,PIPOX,TP53BP2,KAT2B,GOLPH3L,TMTC2,WLS,ANKS1A,RYR3,PRCP,SUPV3L1,MEF2D,MYO1B,ZPLD1,MYL4,GTF2A1,SPTLC1,AGR3,CDK5RAP2,FLVCR1,UBE2E3,FARSB,ARHGEF7,DACH1,TRDN,GASK1A,NPHP1,IKZF1,CAPZA2,SNTG1,SRI,ARNT,BDNF,LDLRAD4,TFCP2L1,ACBD6,BCLAF3,SCIN,MALRD1,TENT4B,MACO1,TLR6,NDST4,ADAMTS9,STIM1,HAUS6,KIF16B,RAB11FIP2,CDH2,EEFSEC,COX7B2,PPARG,ENSG00000290217,ASB3,MTTP,CHRNB4,USP9X,TGM5,NEK7,TENM1,CDK6,MYOF,JAKMIP1,SH3KBP1,SMOX,AVIL,LPCAT2,GDAP1,DDX60,USP25,KIF2C,MYO5A,SIPA1L3,WDFY4,SPTBN5,SFRP4,BLTP1,PNRC1,JCAD,VAV2,TFRC,VPS13A,TES,OSBPL6,GRIA3,PNPLA8,FBN1,HAPSTR1,RAB31,MAP3K1,DDX4,IQCB1,COA1,RAPGEF4,FUT10,CLASP2,CLTC,EIF4G1,B3GAT2,ATP6V0A4,ACSM5,PHLPP1,DNAJC6,ROCK1,NCAPH,M1AP,TAX1BP1,TBC1D4,APIP,MMP28,NUP98,PML,FOXO3B,SACS,MGLL,LONRF3,PLEK2,AFAP1L2,DGKG,SP100,PRKG1,ELAVL4,SBF2,FOXRED2,NDUFAF2,UMODL1,RFPL4B,TTC28,TSEN2,MAGI2,GALK2,NIN,SPG21,ANKFY1,PRSS50,ENSA,FNDC3A,AIMP1,SLC30A10,AP4S1,TPK1,HSPBP1,PRKD1,CALCRL,USP34,STK4,TNNI3K,RAB6D,SPC25,ST6GAL2,SCAMP1,RPL27A,ANPEP,PRLR,PDE11A,NMI,RAB6C,PCCA,CLVS1,EYS,IL16,DIPK1A,EDARADD,CTBP2,TTN,NCALD,HHIP,DYRK1A,PPFIA1,TMEM30A,MYRIP,PPP1R1C,KCNE2,UNC13C,CUL4A,SERINC1,CHSY3,TXNRD3,EYA4,DLGAP2,MAP7D3,PMEPA1,ATE1,ETNK1,TTPA,PEX5L,CHAC2,METTL3,FMNL2,KRT85,UAP1,PARD3,KCNN4,SLC25A48,CP,FUNDC1,BMAL2,ATP8B1,PGPEP1,RNF6,VAV1,SMYD1,FANCB,DPY19L1,GRIA2,VDAC2,VPS13B,SPART
GO:0071944	cell periphery	1.4481041177439953e-12	*C10ORF90,ABCC6,ESYT1,STYK1,TRGC2,CDK14,EPN2,MREG,NOSTRIN,OSBPL8,LRRN1,POTED,STX8,CPNE4,RAPGEF6,SLC16A5,PPM1A,ARL13B,LIMD1,TF,SPTBN1,CDC42EP3,MAPK10,CACNB1,DAPP1,GNG12,FGF1,CHRNB2,SEZ6,CLDN6,KCNQ5,CDH8,ARHGAP15,GNB4,FHL1,ATP10A,SPINK5,SKAP2,PRND,ATP7B,KCNJ6,ZAN,GRIP1,DCT,DLGAP1,SDR16C5,ABCG8,USH2A,NOTCH2*

			,CD93,SLC44A5,SDK2,NPNT,DSC1,EPB41L3,PARVB,CDH11,SHANK3,ARID1B,SLC14A2,STK39,SMURF2,ADAMTS12,PLXNA4,YBX1,CACNA1C,NLGN4X,EXOC4,F13A1,PIK3C2B,XPO6,SLC44A3,SLC5A7,GPSM1,CDON,SLC17A6,THSD7A,GNAI1,NCAM2,TRAK2,LRP8,CNTN1,ENPEP,ARHGEF4,MUC17,CALCR,CHRFAM7A,ELMO1,ERBIN,CD247,MCC,TM9SF2,CLCN3,RHOJ,FGF10,COL4A6,CATSPERB,PLG,CDH7,NCK1,CD84,EXO1,GABRA5,KCNQ3,CHRM3,PREX1,AIG1,COL24A1,ANO3,COL11A1,KCTD3,C2ORF88,ZNF804A,SEPTIN10,MARCHF1,MYO1D,BRAF,COL1A2,DTNA,KCNS3,COL12A1,ALPK2,CHRNA7,HMGB1,LPP,TGFB2,MDFIC,LAMA2,ADD3,MUSK,KCNJ18,JAM3,KCNIP3,MAP3K5,RGS3,RERG,OR5AU1,PRKAR2A,TBC1D5,ATP11A,ATP4A,SLC15A4,PCDHB8,GOLPH3,PRKG2,NCF2,HMCN1,IGFBP7,EPHA5,PTPN20,ATP13A4,ENAH,ATP2C1,STON2,PTGFR,IGHV1OR21-1,FAM171A1,PCLO,ASPH,RRAS2,SH3BGRL,ZDHHC4,ANKS1B,CHL1,SPOCK3,GSN,PLS1,SPON1,ANO2,TLR8,ARHGEF39,ROBO1,ITPR1,PKP4,CPM,PAK3,ABI3BP,CAMK1G,CCNT2,HAVCR1,MR1,F8,TNFRSF12A,TAS2R14,CACNA2D3,CXCR5,NCKAP1,CLDN12,CNTN3,MGAM,IL1RAPL2,ADGRD1,EPHA3,LRRTM4,ARHGAP18,PTPRN2,LYN,ADGRB3,USP6,GRIK1,UBL3,LARGE1,ATP6V1H,SLC12A1,LDB2,SPTB,UNC93A,PAQR5,CORIN,LRRC7,PCDHB16,SGMS2,SLC41A2,NOS3,KCNIP4,NOS1AP,KIAA1614,MSRA,IMPG1,XKR6,MATN2,TGFBR2,CMKLR2,ITGA11,SYNJ1,GPM6B,GPR156,CACNA2D4,LHFPL3,GSDMD,ADGRV1,RASGRF2,EPB41L5,GAD1,WHRN,DAPK1,TTC7B,APP,ADAM12,ATP9B,CACNA2D1,HCN1,CHRM5,SLC13A1,FRY,HERC2,PIK3R5,PALLD,TLR1,TAS2R30,AVPR1B,LMAN1L,PTK2,TICAM2,SH2B2,ATP1B4,HYCC1,PIP4K2B,LRRK2,NKAIN3,SLC38A4,CLSTN2,PLD1,VTCN1,PKHD1,PJA2,MGST1,TIAM1,MLIP,ACVR2A,GNAO1,ASAP2,VIT,SNTG2,NTM,NEDD4L,PRMT8,KIT,EPHA7,OSBPL3,ANTXR1,WNT5A,TRABD2B,CTNNB1,COL19A1,CTSK,ESR1,TNFRSF19,SCFD1,ITGB8,ERC2,WWOX,BTBD8,FILIP1,PTGER4,VWC2L,BEST3,LYPD6B,DOCK1,DIAPH3,GPC4,EFNA5,PLCH1,OR1M1,TRPC5,JAML,TNFSF11,PTPRF,IKBKB,AKAP6,TRBV10-2,LYVE1,APOH,ARHGEF28,OR8B8,CLCN1,RAB3GAP2,STEAP1B,GPRIN1,LINGO2,OPCML,CEMIP,KCNJ12,CHRNB3,ACVR1,HLA-DQA2,COL6A5,GRID2,KCNJ3,IFNLR1,NTNG1,PDE4B,PKD1L3,ADIPOR1,GABRB3,SHROOM4,PLPP3,UNC80,LAMP2,SSPN,KIRREL3,GABRG2,F5,CFAP95,CPS1,TMEM120A,PRKCH,DLC1,DENND2B,XRCC5,RGS17,GFRA1,KCND2,INPP4A,BMX,SLC24A3,GRIA4,RAB18,RGS7BP,MGRN1,WLS,IGKV1-6,RYR3,TMPRSS11B,PRCP,MYO1B,FLVCR1,ARHGEF7,SAMD12,TRDN,GASK1A,FBN2,SLC26A8,CAPZA2,SNTG1,CACHD1,SRI,ATP1B1,SCIN,TENT4B,TLR6,ADAMTS9,STIM1,TMEM161B,RAB11FIP2,CDH2,LAG3,ASB3,MTTP,CHRNB4,TGM5,TENM1,IL13RA2,GABRB1,MYOF,LTB4R2,SH3KBP1

			,NALCN,LPCAT2,SIPA1L3,SLC6A13,LRFN2,SPTBN5,BLTP1,IL1RAP,JCAD,VAV2,TFRC,TES,OSBPL6,GRIA3,FBN1,RAB31,CLEC6A,RAPGEF4,BMPER,CLASP2,CLTC,ANGPT1,SEMA6A,VIPR2,ATP6V0A4,PHLPP1,ITGA4,DNAJC6,ROCK1,MMP28,KCNT2,MGLL,SCARA5,SLC5A12,PLEK2,TNFSF4,AFAP1L2,DGKG,PRKG1,CDH9,UMODL1,MAGI2,PCDH19,NIN,ASIC2,SLC30A10,PRKD1,CALCRL,SLC20A2,PCDH10,SCAMP1,ANPEP,PRLR,PTPRA,EYS,IL16,GNA14,CR2,LPAR6,CTBP2,HHIP,TMEM30A,MYRIP,KCNE2,UNC13C,SERINC1,KCTD16,DLGAP2,SEMA5A,LAMA4,PMEPA1,UAP1,PARD3,KCNN4,TRDC,CP,COL6A6,ATP8B1,VAV1,GRIA2,SPART,IGLV4-3
GO:0045202	synapse	4.026514192564256e-11	*APBB2,RPL19,RTN1,ARHGEF9,ABR,EIF1AX,SPTBN1,CACNB1,CHRNB2,SEZ6,KCNQ5,CDH8,PPM1H,CAMK2B,GRIP1,DLGAP1,KALRN,USH2A,TMOD2,SDK2,CDH11,SHANK3,PLXNA4,YBX1,CACNA1C,PRKN,NLGN4X,EXOC4,SLC5A7,SLC17A6,TIAM2,LRP8,CNTN1,CPEB4,CHRFAM7A,ERBIN,CLCN3,PLG,GABRA5,KCNQ3,CHRM3,ZNF804A,BRAF,DTNA,CHRNA7,LAMA2,ADD3,TRIO,MUSK,ENAH,TMEM230,STON2,PCLO,ANKS1B,ANO2,ITPR1,PKP4,PAK3,IL1RAPL2,LRRTM4,PTPRN2,LYN,ADGRB3,GRIK1,LARGE1,ATP6V1H,SPTB,ALDH1A1,PCDHB16,EIF3L,NOS1AP,SYNPR,SYNJ1,UNC119,GAD1,WHRN,DAPK1,INSYN2A,APP,IGF2BP1,HCN1,CHRM5,PALLD,PTK2,LRRK2,CLSTN2,PLD1,PJA2,TIAM1,PHACTR1,IQSEC2,EPHA7,WNT5A,ARFGEF2,CTNNB1,ERC2,BTBD8,FILIP1,PDE7B,VWC2L,DIAPH3,GPC4,EFNA5,FXR2,CHRNB3,GRID2,KCNJ3,NTNG1,PDE4B,GABRB3,SSPN,KIRREL3,GABRG2,RGS17,KCND2,INPP4A,GRIA4,RGS7BP,ARHGEF7,BDNF,MACO1,CDH2,TANC1,CHRNB4,GABRB1,MYOF,SH3KBP1,SLC6A13,LRFN2,BLTP1,IL1RAP,GRIA3,RAPGEF4,EIF4G1,ATP6V0A4,DNAJC6,ROCK1,ELAVL4,CDH9,MAGI2,ASIC2,SCAMP1,RPL27A,PTPRA,CTBP2,PPFIA1,MYRIP,UNC13C,KCTD16,DLGAP2,LAMA4,GRIA2,SPART*
GO:0005886	plasma membrane	1.5672704696600543e-10	*C10ORF90,ABCC6,ESYT1,STYK1,TRGC2,CDK14,EPN2,MREG,NOSTRIN,POTED,STX8,CPNE4,RAPGEF6,SLC16A5,PPM1A,ARL13B,LIMD1,TF,SPTBN1,CDC42EP3,MAPK10,CACNB1,DAPP1,GNG12,CHRNB2,SEZ6,CLDN6,KCNQ5,CDH8,ARHGAP15,GNB4,FHL1,ATP10A,SKAP2,PRND,ATP7B,KCNJ6,ZAN,GRIP1,DCT,DLGAP1,SDR16C5,ABCG8,USH2A,NOTCH2,CD93,SLC44A5,SDK2,DSC1,EPB41L3,PARVB,CDH11,SHANK3,ARID1B,SLC14A2,STK39,SMURF2,PLXNA4,YBX1,CACNA1C,NLGN4X,EXOC4,PIK3C2B,XPO6,SLC44A3,SLC5A7,GPSM1,CDON,SLC17A6,THSD7A,GNAI1,NCAM2,TRAK2,LRP8,CNTN1,ENPEP,ARHGEF4,MUC17,CALCR,CHRFAM7A,ELMO1,ERBIN,CD247,MCC,TM9SF2,CLCN3,RHOJ,FGF10,CATSPERB,PLG,CDH7,NCK1,CD84,EXO1,GABRA5,KCNQ3,CHRM3,PREX1,AIG1,ANO3,KCTD3,C2ORF88,ZNF804A,MARCHF1,MYO1D,BRAF,DTNA,KCNS3,ALPK2,CHRNA7,HMGB1,LPP,MDFIC,LAMA2,ADD3,MUSK,KCNJ18,JAM3,KCNIP3,MAP3K5,RGS3,RERG,OR5AU1,PRKAR2A,TBC1D5,ATP11A,ATP4A,SLC15A4*

			,*PCDHB8,GOLPH3,PRKG2,NCF2,HMCN1,EPHA5,PTPN20,ATP13A4,ENAH,ATP2C1,STON2,PTGFR,IGHV1OR21-1,FAM171A1,ASPH,RRAS2,SH3BGRL,ZDHHC4,ANKS1B,CHL1,GSN,PLS1,ANO2,TLR8,ARHGEF39,ROBO1,ITPR1,PKP4,CPM,PAK3,CAMK1G,CCNT2,HAVCR1,MR1,F8,TNFRSF12A,TAS2R14,CACNA2D3,CXCR5,NCKAP1,CLDN12,CNTN3,MGAM,IL1RAPL2,ADGRD1,EPHA3,LRRTM4,ARHGAP18,PTPRN2,LYN,ADGRB3,USP6,GRIK1,UBL3,LARGE1,ATP6V1H,SLC12A1,LDB2,SPTB,UNC93A,PAQR5,CORIN,LRRC7,PCDHB16,SGMS2,SLC41A2,NOS3,KCNIP4,NOS1AP,KIAA1614,MSRA,XKR6,TGFBR2,CMKLR2,ITGA11,SYNJ1,GPM6B,GPR156,CACNA2D4,LHFPL3,GSDMD,ADGRV1,RASGRF2,EPB41L5,GAD1,WHRN,DAPK1,TTC7B,APP,ADAM12,ATP9B,CACNA2D1,HCN1,CHRM5,SLC13A1,HERC2,PIK3R5,PALLD,TLR1,TAS2R30,AVPR1B,PTK2,TICAM2,SH2B2,ATP1B4,HYCC1,PIP4K2B,LRRK2,NKAIN3,SLC38A4,CLSTN2,PLD1,VTCN1,PKHD1,PJA2,MGST1,TIAM1,MLIP,ACVR2A,GNAO1,ASAP2,SNTG2,NTM,NEDD4L,PRMT8,KIT,EPHA7,OSBPL3,ANTXR1,WNT5A,TRABD2B,CTNNB1,CTSK,ESR1,TNFRSF19,SCFD1,ITGB8,ERC2,WWOX,BTBD8,FILIP1,PTGER4,VWC2L,BEST3,LYPD6B,DOCK1,DIAPH3,GPC4,EFNA5,PLCH1,OR1M1,TRPC5,JAML,TNFSF11,PTPRF,IKBKB,AKAP6,TRBV10-2,LYVE1,ARHGEF28,OR8B8,CLCN1,RAB3GAP2,STEAP1B,GPRIN1,OPCML,CEMIP,KCNJ12,CHRNB3,ACVR1,HLA-DQA2,GRID2,KCNJ3,IFNLR1,NTNG1,PDE4B,PKD1L3,ADIPOR1,GABRB3,SHROOM4,PLPP3,UNC80,LAMP2,SSPN,KIRREL3,GABRG2,F5,CFAP95,CPS1,TMEM120A,PRKCH,DLC1,DENND2B,XRCC5,RGS17,GFRA1,KCND2,INPP4A,BMX,SLC24A3,GRIA4,RAB18,RGS7BP,MGRN1,WLS,IGKV1-6,RYR3,TMPRSS11B,PRCP,MYO1B,FLVCR1,ARHGEF7,SAMD12,TRDN,GASK1A,SLC26A8,SNTG1,CACHD1,SRI,ATP1B1,TENT4B,TLR6,STIM1,TMEM161B,RAB11FIP2,CDH2,LAG3,ASB3,MTTP,CHRNB4,TGM5,TENM1,IL13RA2,GABRB1,MYOF,LTB4R2,SH3KBP1,NALCN,LPCAT2,SIPA1L3,SLC6A13,LRFN2,SPTBN5,BLTP1,IL1RAP,JCAD,VAV2,TFRC,TES,OSBPL6,GRIA3,RAB31,CLEC6A,RAPGEF4,CLASP2,CLTC,ANGPT1,SEMA6A,VIPR2,ATP6V0A4,PHLPP1,ITGA4,DNAJC6,ROCK1,KCNT2,MGLL,SCARA5,SLC5A12,PLEK2,TNFSF4,AFAP1L2,DGKG,PRKG1,CDH9,UMODL1,MAGI2,PCDH19,NIN,ASIC2,SLC30A10,PRKD1,CALCRL,SLC20A2,PCDH10,SCAMP1,ANPEP,PRLR,PTPRA,IL16,GNA14,CR2,LPAR6,HHIP,TMEM30A,KCNE2,UNC13C,SERINC1,KCTD16,DLGAP2,SEMA5A,PMEPA1,UAP1,PARD3,KCNN4,TRDC,CP,ATP8B1,VAV1,GRIA2,SPART,IGLV4-3*
GO:0030054	cell junction	2.1922316622070768e-10	*APBB2,RPL19,RTN1,NFIA,ARHGEF9,ABR,EIF1AX,LIMD1,SPTBN1,CACNB1,CHRNB2,SEZ6,CLDN6,KCNQ5,CDH8,FHL1,SH3PXD2A,PPM1H,CAMK2B,GRIP1,DLGAP1,KALRN,USH2A,TMOD2,SDK2,DSC1,EPB41L3,PARVB,CDH11,SHANK3,PLXNA4,YBX1,CACNA1C,PRKN,NLGN4X,EXOC4,SLC5A*

			7,SLC17A6,TIAM2,LRP8,CNTN1,CPEB4,CHRFAM7A,ERBIN,CLCN3,PLG,CDH7,ILDR2,NCK1,GABRA5,KCNQ3,CHRM3,ZNF804A,BRAF,DTNA,TMOD3,CHRNA7,LPP,LAMA2,ADD3,TRIO,MUSK,JAM3,PRKAR2A,HMCN1,ENAH,TMEM230,STON2,PCLO,RRAS2,ANKS1B,GSN,ANO2,ITPR1,PKP4,PAK3,NCKAP1,CLDN12,IL1RAPL2,LRRTM4,PTPRN2,LYN,ADGRB3,GRIK1,LARGE1,ATP6V1H,SPTB,ALDH1A1,PCDHB16,DCP2,EIF3L,NOS1AP,SYNPR,ITGA11,SYNJ1,UNC119,EPB41L5,GAD1,WHRN,DAPK1,PRUNE1,INSYN2A,APP,IGF2BP1,HCN1,CHRM5,PALLD,PTK2,LRRK2,CLSTN2,PLD1,PJA2,TIAM1,PHACTR1,IQSEC2,KIT,EPHA7,WNT5A,ARFGEF2,CTNNB1,ITGB8,ERC2,BCAR3,FRMD4A,BTBD8,FILIP1,PDE7B,VWC2L,DIAPH3,GPC4,EFNA5,JAML,FXR2,AKAP6,CHRNB3,GRID2,KCNJ3,NTNG1,PDE4B,GABRB3,SHROOM4,PLPP3,SSPN,KIRREL3,GABRG2,PRKCH,DLC1,RGS17,KCND2,INPP4A,MPRIP,GRIA4,RGS7BP,TP53BP2,CDK5RAP2,ARHGEF7,NPHP1,ATP1B1,BDNF,SCIN,MACO1,CDH2,TANC1,CHRNB4,GABRB1,MYOF,SH3KBP1,AVIL,SIPA1L3,SLC6A13,LRFN2,SPTBN5,BLTP1,IL1RAP,JCAD,TES,GRIA3,RAPGEF4,CLASP2,CLTC,EIF4G1,ATP6V0A4,ITGA4,DNAJC6,ROCK1,LAYN,ELAVL4,CDH9,MAGI2,ASIC2,PRKD1,SCAMP1,RPL27A,PTPRA,IL16,CTBP2,PPFIA1,MYRIP,UNC13C,KCTD16,DLGAP2,LAMA4,PARD3,VAV1,GRIA2,SPART*
GO:0043005	neuron projection	5.527434678213096e-10	*APBB2,RTN1,GARS1,ABR,SPTBN1,AHCYL2,CHRNB2,SEZ6,KCNQ5,CDH8,AGTPBP1,CAMK2B,GRIP1,USH2A,EPB41L3,MAP6,SHANK3,CACNA1C,PRKN,NLGN4X,EXOC4,SLC5A7,SLC17A6,NCAM2,TIAM2,TRAK2,LRP8,CNTN1,CPEB4,CALCR,CHRFAM7A,CLCN3,GABRA5,KCNQ3,CHRM3,PREX1,ZNF804A,MYO1D,BRAF,DTNA,CHRNA7,TGFB2,LAMA2,EPHA5,STON2,PCLO,ANKS1B,CHL1,PLS1,ANO2,KIF21A,ROBO1,PACRG,EPHA3,MYH14,DIP2B,ALDH1A1,NMNAT3,NAV1,KCNIP4,SYNPR,IMPG1,CMKLR2,MARK4,SYNJ1,ADGRV1,GAD1,WHRN,APP,IGF2BP1,HCN1,CHRM5,ELOVL5,PALLD,PTK2,HYCC1,LRRK2,CLSTN2,TIAM1,RABGEF1,EPHA7,ARFGEF2,ERC2,BTBD8,NGEF,TRPC5,PTPRF,FXR2,GPRIN1,CHRNB3,GRID2,PDE4B,GABRB3,UNC80,KIRREL3,GABRG2,RGS17,GFRA1,KCND2,GRIA4,TPH2,RGS7BP,ANKS1A,ARHGEF7,NPHP1,BDNF,MACO1,TMEM237,CDH2,TANC1,CHRNB4,USP9X,TENM1,GABRB1,SH3KBP1,AVIL,MYO5A,SPTBN5,GRIA3,IQCB1,CLASP2,SEMA6A,ITGA4,SACS,ELAVL4,SBF2,MAGI2,NIN,ASIC2,EYS,DYRK1A,PPFIA1,MYRIP,UNC13C,PARD3,KCNN4,ATP8B1,RNF6,GRIA2*
GO:0042995	cell projection	9.099267605010038e-9	*APBB2,RTN1,GARS1,ABR,ARL13B,SAMSN1,SPTBN1,AHCYL2,CHRNB2,SEZ6,KCNQ5,CDH8,AGTPBP1,SH3PXD2A,CAMK2B,GRIP1,USH2A,NOTCH2,EPB41L3,MAP6,PARVB,SHANK3,CFAP70,CACNA1C,PRKN,NLGN4X,EXOC4,SLC5A7,IFT172,SLC17A6,THSD7A,NCAM2,TIAM2,CFAP54,TRAK2,LRP8,CNTN1,ARHGEF4,CPEB4,CALCR,CHRFAM7A,MCC,DNAH10,CLCN3,RSPH10B,CATSPERB,GABRA5,KCNQ3,CHRM3,PREX1,ZNF804A,SEPTIN10,MYO1D,BRAF,DTNA,ABLIM1,CHRNA7,DYNLT2*

			B,TGFB2,LAMA2,TRIO,JAM3,PRKAR2A,PIK3C3,EPHA5,ENAH,STON2,PCLO,ANKS1B,CHL1,GSN,PLS1,ANO2,KIF21A,ROBO1,DNAAF11,HAVCR1,NCKAP1,CILK1,PACRG,EPHA3,MYH14,ARHGAP18,DIP2B,NME7,SPTB,ALDH1A1,NMNAT3,NAV1,KCNIP4,NOS1AP,SYNPR,IMPG1,CMKLR2,CFAP77,MARK4,SYNJ1,ULK3,ADGRV1,EPB41L5,GAD1,WHRN,APP,IGF2BP1,RNF38,HCN1,CHRM5,ELOVL5,PALLD,PTK2,TICAM2,SH2B2,HYCC1,DNAH12,LRRK2,SLC38A4,CLSTN2,CFAP298,PKHD1,PJA2,TIAM1,RABGEF1,EPHA7,OSBPL3,ANTXR1,ARFGEF2,CTNNB1,ERC2,WWOX,BTBD8,DIAPH3,ACTR3B,NGEF,TRPC5,PTPRF,FXR2,GPRIN1,CHRNB3,GRID2,PDE4B,GABRB3,UNC80,KIRREL3,GABRG2,CFAP95,RABGAP1L,DLC1,RGS17,GFRA1,KCND2,BMX,GRIA4,TPH2,RGS7BP,FBXL13,ANKS1A,MYO1B,ARHGEF7,NPHP1,SLC26A8,SNTG1,ATP1B1,BDNF,SCIN,MACO1,TMEM237,RAB11FIP2,CDH2,TANC1,MTTP,CHRNB4,USP9X,TENM1,CDK6,GABRB1,SH3KBP1,AVIL,MYO5A,SPTBN5,JCAD,VPS13A,GRIA3,IQCB1,CLASP2,ANGPT1,SEMA6A,ATP6V0A4,ITGA4,ROCK1,SACS,LAYN,PLEK2,ELAVL4,SBF2,MAGI2,NIN,ASIC2,SLC20A2,EYS,HHIP,DYRK1A,PPFIA1,MYRIP,UNC13C,KCTD16,PARD3,KCNN4,ATP8B1,RNF6,GRIA2,VDAC2
GO:0120025	plasma membrane bounded cell projection	3.0945062170175295e-8	*APBB2,RTN1,GARS1,ABR,ARL13B,SAMSN1,SPTBN1,AHCYL2,CHRNB2,SEZ6,KCNQ5,CDH8,AGTPBP1,CAMK2B,GRIP1,USH2A,NOTCH2,EPB41L3,MAP6,PARVB,SHANK3,CFAP70,CACNA1C,PRKN,NLGN4X,EXOC4,SLC5A7,IFT172,SLC17A6,NCAM2,TIAM2,CFAP54,TRAK2,LRP8,CNTN1,ARHGEF4,CPEB4,CALCR,CHRFAM7A,MCC,DNAH10,CLCN3,RSPH10B,CATSPERB,GABRA5,KCNQ3,CHRM3,PREX1,ZNF804A,SEPTIN10,MYO1D,BRAF,DTNA,ABLIM1,CHRNA7,DYNLT2B,TGFB2,LAMA2,JAM3,PRKAR2A,PIK3C3,EPHA5,ENAH,STON2,PCLO,ANKS1B,CHL1,GSN,PLS1,ANO2,KIF21A,ROBO1,DNAAF11,HAVCR1,NCKAP1,CILK1,PACRG,EPHA3,MYH14,ARHGAP18,DIP2B,NME7,ALDH1A1,NMNAT3,NAV1,KCNIP4,NOS1AP,SYNPR,IMPG1,CMKLR2,CFAP77,MARK4,SYNJ1,ULK3,ADGRV1,EPB41L5,GAD1,WHRN,APP,IGF2BP1,RNF38,HCN1,CHRM5,ELOVL5,PALLD,PTK2,SH2B2,HYCC1,DNAH12,LRRK2,SLC38A4,CLSTN2,CFAP298,PKHD1,PJA2,TIAM1,RABGEF1,EPHA7,OSBPL3,ANTXR1,ARFGEF2,CTNNB1,ERC2,WWOX,BTBD8,NGEF,TRPC5,PTPRF,FXR2,GPRIN1,CHRNB3,GRID2,PDE4B,GABRB3,UNC80,KIRREL3,GABRG2,CFAP95,RABGAP1L,DLC1,RGS17,GFRA1,KCND2,BMX,GRIA4,TPH2,RGS7BP,FBXL13,ANKS1A,MYO1B,ARHGEF7,NPHP1,SLC26A8,SNTG1,ATP1B1,BDNF,MACO1,TMEM237,CDH2,TANC1,MTTP,CHRNB4,USP9X,TENM1,CDK6,GABRB1,SH3KBP1,AVIL,MYO5A,SPTBN5,JCAD,VPS13A,GRIA3,IQCB1,CLASP2,ANGPT1,SEMA6A,ATP6V0A4,ITGA4,ROCK1,SACS,LAYN,PLEK2,ELAVL4,SBF2,MAGI2,NIN,ASIC2,SLC20A2,EYS,HHIP,DYRK1A,PPFIA1,MYRIP,UNC13C,PARD3,KCNN4,ATP8B1,RNF6,GRIA2,VDAC2*
GO:0098	postsynapse	2.65016192	*RTN1,ARHGEF9,ABR,SPTBN1,CHRNB2,SEZ6,GR*

794		0326477e-7	IP1,DLGAP1,KALRN,SHANK3,CACNA1C,PRKN,NLGN4X,CNTN1,CPEB4,CHRFAM7A,ERBIN,GABRA5,CHRM3,ZNF804A,CHRNA7,LAMA2,ADD3,TRIO,MUSK,PCLO,ANKS1B,ITPR1,PKP4,PAK3,LRRTM4,LYN,ADGRB3,GRIK1,SPTB,DAPK1,INSYN2A,APP,IGF2BP1,HCN1,CHRM5,PTK2,LRRK2,CLSTN2,PJA2,TIAM1,WNT5A,ARFGEF2,CTNNB1,FXR2,CHRNB3,GRID2,PDE4B,GABRB3,SSPN,GABRG2,KCND2,INPP4A,GRIA4,RGS7BP,ARHGEF7,CDH2,TANC1,CHRNB4,GABRB1,LRFN2,GRIA3,EIF4G1,DNAJC6,ELAVL4,CDH9,MAGI2,ASIC2,PPFIA1,KCTD16,DLGAP2,GRIA2
GO:0005829	cytosol	8.220363903261056e-7	C10ORF90,PFKP,RPL19,CDK14,EPN2,MSRB3,PDE1C,MAP3K3,NINL,OSBPL8,STX8,PHF20,SMYD3,GARS1,RAPGEF6,ARHGEF9,ABR,MAOA,BLTP3B,TRAPPC9,PREP,EIF1AX,PPM1A,ARL13B,SAMSN1,LIMD1,MYBPC1,USP42,RCSD1,SPTBN1,CDC42EP3,MAPK10,USP32,AHCYL2,DAPP1,FGF1,ARHGAP15,GNB4,AGTPBP1,FHL1,RBPMS,SH3PXD2A,SPINK5,SKAP2,DOK4,TNRC18,CAMK2B,GRIP1,DDI2,PGM1,DCT,PECR,XPO4,OTUD7A,KALRN,FKBP5,HMBOX1,EPB41L3,PARVB,WDR26,ARID1B,FOXO1,STK39,SMURF2,TBC1D16,YBX1,STK24,ASB4,IDO1,PRKN,EXOC4,PIK3C2B,XPO6,GPSM1,NLK,MAPKAPK3,PAPOLG,TIAM2,TRAK2,AGL,STAT5B,NUDT19,ARHGEF4,UBE2O,EIF4ENIF1,ELMO1,RGS22,MCC,CEP192,ADAR,PDE10A,CDC37,ZFYVE16,MECOM,HERC5,PGPEP1L,NCK1,KLHL42,GABRA5,NEXMIF,PREX1,CIDEB,MYO1D,BRAF,GEMIN5,EEF2KMT,KCNS3,VWA3B,RBMS1,HECW1,AFF3,LPP,HIVEP1,ADD3,TRIO,KCNIP3,SFPQ,PNPLA4,MAP3K5,RGS3,NUP93,RERG,PRKAR2A,TBC1D5,CTIF,GOLPH3,PRKG2,NCF2,PIK3C3,USP10,SND1,ENAH,ALDH1A2,STON2,STRADA,HUWE1,SH3BGRL,UBR5,SOX30,ANKS1B,GSN,PLS1,HPS3,KIF21A,ARMH3,ANKRD11,DNAAF11,PAK3,KLF12,PRUNE2,DOK6,PRKAG2,MLLT3,CCNT2,TRAPPC10,CNOT10,KRTAP21-2,OASL,NCKAP1,CILK1,PACRG,ADGRD1,EPHA3,LATS2,MYH14,ARHGAP18,LYN,KLF7,NME7,SEC24C,ATP6V1H,RRM2,SPTB,ALDH1A1,FMO5,RNASEL,TMCC1,LRRC7,DCP2,NOS3,KCNIP4,EIF3L,NOMO2,TNRC6C,MLLT10,NOS1AP,EIF2AK4,MSRA,HMGCLL1,MAGEL2,TGFBR2,MARK4,MDN1,SYNJ1,CHAF1B,CUX1,POLR2C,UNC119,RRAGD,ULK3,PPP2R5E,KPNB1,GSDMD,RASGRF2,SERBP1,EPB41L5,NUDCD1,ALDH9A1,PRUNE1,SAR1B,TTC7B,APP,SMAD5,IGF2BP1,UBE2G1,PELI2,PI4KB,HERC2,HOOK3,PIK3R5,PALLD,BMT2,QDPR,PTK2,USP7,SCP2,SH2B2,AVEN,HYCC1,POGZ,PIP4K2B,LRRK2,ZNF395,IKZF3,ARIH1,CFAP298,ITFG2,UBXN7,COP1,TIAM1,CSRP3,PHACTR1,MLIP,ASAP2,NEDD4L,BRCC3,RABGEF1,SPAG9,OSBPL3,SPDL1,WIPI2,PEX26,ARFGEF2,CTNNB1,PRKDC,IPO5,ESR1,SCFD1,VPS53,WWOX,BNIPL,FILIP1,ANAPC10,FBXO15,PDE7B,USP40,DOCK1,DIAPH3,BPGM,IGF2BP2,PLCH1,NGEF,FNIP2,AASS,IKBKB,FXR2,ARHGEF28,RAB3GAP2,CCDC91,ZCCHC17,RB1,MPV17,PDE4B,NLRP4,GOSR2,SMG7,SPSB1,AK5,PRKCH,ACACB,DLC1,XRCC5,INPP4A,BMX,MPRIP,RAB18,TPH2,MGR

			N1,FBXL13,ERCC6L2,PIPOX,TP53BP2,KAT2B,GOLPH3L,WLS,ANKS1A,MYL4,GTF2A1,CDK5RAP2,UBE2E3,FARSB,ARHGEF7,DACH1,TRDN,NPHP1,IKZF1,CAPZA2,SRI,ACBD6,TENT4B,HAUS6,KIF16B,PPARG,ASB3,MTTP,USP9X,CDK6,SH3KBP1,SMOX,GDAP1,DDX60,USP25,KIF2C,MYO5A,SPTBN5,VAV2,VPS13A,TES,OSBPL6,RAB31,MAP3K1,IQCB1,COA1,RAPGEF4,CLASP2,CLTC,EIF4G1,PHLPP1,DNAJC6,ROCK1,NCAPH,TAX1BP1,TBC1D4,APIP,NUP98,PML,FOXO3B,MGLL,AFAP1L2,DGKG,PRKG1,SBF2,RFPL4B,TSEN2,GALK2,SPG21,ANKFY1,FNDC3A,AIMP1,TPK1,PRKD1,USP34,STK4,RAB6D,SPC25,RPL27A,PDE11A,NMI,RAB6C,PCCA,IL16,EDARADD,CTBP2,TTN,NCALD,PPFIA1,TXNRD3,ETNK1,TTPA,PEX5L,CHAC2,METTL3,FMNL2,KRT85,UAP1,PARD3,KCNN4,ATP8B1,PGPEP1,VAV1,FANCB,SPART
GO:0098796	membrane protein complex	9.964202957901845e-7	*TRGC2,EPN2,STX8,IMMP1L,TF,CACNB1,GNG12,CHRNB2,KCNQ5,CDH8,GNB4,KCNJ6,SLC38A9,ABCG8,CDH11,DIPK2A,CACNA1C,PIK3C2B,SLC17A6,GNAI1,CALCR,CHRFAM7A,CD247,CATSPERB,CDH7,GABRA5,KCNQ3,CIDEB,SESTD1,KCNS3,CHRNA7,HMGB1,KCNJ18,KCNIP3,MAP3K5,TBC1D5,ATP4A,NCF2,PIK3C3,STON2,ASPH,ANO2,MR1,VPS8,MTX3,CACNA2D3,CHCHD3,UVRAG,LYN,GRIK1,SEC24C,ATP6V1H,KCNIP4,MAGEL2,TGFBR2,ITGA11,SYNJ1,GPR156,CACNA2D4,SAR1B,CACNA2D1,HCN1,HOOK3,USE1,PIK3R5,TLR1,ATP1B4,UBXN7,ACVR2A,GNAO1,SNTG2,CTNNB1,ITGB8,BTBD8,VWC2L,BEST3,DIAPH3,SYNE3,TRPC5,IKBKB,AKAP6,TRBV10-2,CLCN1,KCNJ12,CHRNB3,ACVR1,HLA-DQA2,GRID2,KCNJ3,IFNLR1,PDE4B,PKD1L3,GABRB3,GOSR2,UNC80,IMMP2L,LAMP2,SSPN,GABRG2,GFRA1,KCND2,GRIA4,RYR3,SNTG1,CACHD1,ATP1B1,TLR6,CDH2,COX7B2,CHRNB4,GABRB1,NALCN,TFRC,GRIA3,CLTC,ATP6V0A4,ITGA4,CDH9,ANKFY1,AP4S1,CALCRL,GNA14,NCALD,KCNE2,KCNN4,GRIA2,VDAC2*
GO:1990351	transporter complex	0.0000010134351523224254	*CACNB1,CHRNB2,KCNQ5,ATP10A,KCNJ6,ABCG8,CACNA1C,SLC17A6,CHRFAM7A,CATSPERB,GABRA5,KCNQ3,SESTD1,KCNS3,CHRNA7,KCNJ18,KCNIP3,ATP11A,ATP4A,ASPH,ANO2,CACNA2D3,GRIK1,ATP6V1H,KCNIP4,CACNA2D4,CACNA2D1,HCN1,ATP1B4,VWC2L,BEST3,TRPC5,AKAP6,CLCN1,KCNJ12,CHRNB3,GRID2,KCNJ3,PDE4B,PKD1L3,GABRB3,UNC80,GABRG2,KCND2,GRIA4,RYR3,CACHD1,ATP1B1,COX7B2,CHRNB4,GABRB1,NALCN,GRIA3,ATP6V0A4,TMEM30A,KCNE2,KCNN4,ATP8B1,GRIA2*
GO:1902495	transmembrane transporter complex	0.000004845552831288936	*CACNB1,CHRNB2,KCNQ5,KCNJ6,ABCG8,CACNA1C,SLC17A6,CHRFAM7A,CATSPERB,GABRA5,KCNQ3,SESTD1,KCNS3,CHRNA7,KCNJ18,KCNIP3,ATP4A,ASPH,ANO2,CACNA2D3,GRIK1,ATP6V1H,KCNIP4,CACNA2D4,CACNA2D1,HCN1,ATP1B4,VWC2L,BEST3,TRPC5,AKAP6,CLCN1,KCNJ12,CHRNB3,GRID2,KCNJ3,PDE4B,PKD1L3,GABRB3,UNC80,GABRG2,KCND2,GRIA4,RYR3,CACHD1,ATP1B1,COX7B2,CHRNB4,GABRB1,NALCN,GRIA3,ATP6V0A4,KCNE2,KCNN4,GRIA2*

GO:0012505	endomembrane system	0.000005853171542060084	*FSTL1,APBB2,ABCC6,ESYT1,RTN1,HS3ST3A1,EPN2,MSRB3,MREG,WSCD1,OSBPL8,STX8,PHF20,TXNDC8,GARS1,BLTP3B,TRAPPC9,THADA,TF,CHST11,RCSD1,CDC42EP3,USP32,AHCYL2,CDC14C,SEZ6,ATP10A,SPINK5,PIGL,ATP7B,KCNJ6,TNRC18,CAMK2B,GRIP1,ARHGAP11A-SCG5,PGM1,SLC38A9,SDR16C5,RGPD2,XPO4,SUMF1,XXYLT1,GALNT9,NOTCH2,CD93,DSC1,MAP6,SPTLC3,SRL,TBC1D16,YBX1,STK24,DIPK2A,FAM20B,FAM3C,ARB2A,PRKN,PNPLA3,F13A1,PIK3C2B,SLC5A7,MARCHF9,GPSM1,SERPINI1,SLC17A6,SDF4,TRAK2,CNMD,AGL,MUC17,CPEB4,CALCR,ERBIN,MBTPS2,CD247,TM9SF2,CLCN3,ZFYVE16,COL4A6,PLG,ILDR2,NCK1,CHRM3,RGPD3,AIG1,COL24A1,GOLGA8R,COL11A1,CIDEB,MARCHF1,MYO1D,COL1A2,XPO7,KCNS3,COL12A1,HMGB1,CDIP1,TGFB2,NDST1,JAM3,KCNIP3,MAP3K5,ALKBH1,NUP93,TBC1D5,ATP11A,SLC15A4,GOLPH3,PRKG2,MBTPS1,NCF2,PIK3C3,PIGV,USP10,IGFBP7,EPHA5,ATP13A4,SNX10,ATP2C1,TMEM230,STON2,HUWE1,ASPH,RRAS2,ZDHHC4,GSN,SPON1,TLR8,HPS3,ARMH3,ITPR1,CYP2A13,CAMK1G,TRAPPC10,CPQ,MR1,F8,GDPD1,VPS8,MGAM,EPHA3,ERP29,UVRAG,PTPRN2,LYN,USP6,SEC24C,LARGE1,ATP6V1H,NUP58,PDZD8,SIL1,MYRFL,FMO5,TMCC1,PPM1L,SGMS2,TMED7-TICAM2,NOS3,KCNIP4,NOMO2,NOS1AP,SYNPR,HMGCLL1,MAGEL2,SYNJ1,CUX1,SHISA2,GKAP1,KPNB1,GSDMD,RASGRF2,SCG5,OCA2,SAR1B,APP,MAMLD1,ATP9B,CACNA2D1,PI4KB,ELOVL5,HOOK3,USE1,TLR1,AVPR1B,LMAN1L,TICAM2,SCP2,ATP1B4,AVEN,PIP4K2B,LRRK2,GET1-SH3BGR,CLSTN2,PLD1,ITFG2,PKHD1,UBXN7,PJA2,MGST1,MLIP,ASAP2,NEDD4L,SNX13,RABGEF1,CYB5R4,KIT,SPAG9,OSBPL3,ANTXR1,WNT5A,ARFGEF2,TEX2,ANKRD17,COL19A1,CTSK,IPO5,ESR1,SCFD1,POFUT2,VPS53,WWOX,TPST2,BTBD8,DIAPH3,GPC4,SYNE3,VKORC1L1,UGGT2,AKAP6,APOH,RAB3GAP2,STEAP1B,CEMIP,HLA-DQA2,CCDC91,PDE4B,KIF13A,GOSR2,PLPP3,LAMP2,SSPN,KIRREL3,F5,TMEM120A,BNIP3L,AGPAT5,RABGAP1L,DLC1,DENND2B,XRCC5,GFRA1,INPP4A,TPST1,RAB18,POU2F1,TMTC1,MGRN1,GOLPH3L,TMTC2,WLS,RYR3,PRCP,MYO1B,SPTLC1,AGR3,CDK5RAP2,DACH1,TRDN,GASK1A,SRI,BDNF,LDLRAD4,MALRD1,MACO1,TLR6,NDST4,ADAMTS9,STIM1,KIF16B,RAB11FIP2,CDH2,ENSG00000290217,MTTP,CHRNB4,TENM1,GABRB1,MYOF,SMOX,LPCAT2,USP25,MYO5A,SIPA1L3,WDFY4,BLTP1,TFRC,VPS13A,OSBPL6,PNPLA8,FBN1,RAB31,FUT10,CLASP2,CLTC,B3GAT2,ATP6V0A4,ROCK1,NUP98,PML,MGLL,PRKG1,ELAVL4,SBF2,FOXRED2,MAGI2,SPG21,ANKFY1,PRSS50,FNDC3A,AIMP1,SLC30A10,AP4S1,HSPBP1,PRKD1,CALCRL,RAB6D,ST6GAL2,SCAMP1,RPL27A,ANPEP,PRLR,RAB6C,CLVS1,DIPK1A,NCALD,PPFIA1,TMEM30A,MYRIP,UNC13C,SERINC1,CHSY3,TXNRD3,PMEPA1,TTPA,METTL3,PARD3,CP,ATP8B1,RNF6,DPY19L1,GRIA2,VDAC2,V*

			PS13B
GO:0030424	axon	0.000008643830210803553	*APBB2,GARS1,ABR,SPTBN1,KCNQ5,CDH8,AGTPBP1,USH2A,EPB41L3,MAP6,EXOC4,SLC5A7,NCAM2,TIAM2,TRAK2,LRP8,CNTN1,CPEB4,CALCR,CLCN3,KCNQ3,PREX1,ZNF804A,MYO1D,DTNA,TGFB2,EPHA5,PCLO,KIF21A,ROBO1,MYH14,DIP2B,ALDH1A1,NMNAT3,NAV1,SYNJ1,GAD1,WHRN,APP,IGF2BP1,HCN1,PALLD,LRRK2,TIAM1,ERC2,BTBD8,NGEF,TRPC5,FXR2,GPRIN1,UNC80,KIRREL3,GABRG2,GFRA1,RGS7BP,BDNF,MACO1,TANC1,USP9X,AVIL,MYO5A,CLASP2,SEMA6A,ITGA4,SACS,ELAVL4,SBF2,NIN,DYRK1A,PPFIA1,UNC13C,PARD3,RNF6*
GO:0034702	monoatomic ion channel complex	0.0000156593994511599 4	*CACNB1,CHRNB2,KCNQ5,KCNJ6,CACNA1C,SLC17A6,CHRFAM7A,CATSPERB,GABRA5,KCNQ3,SESTD1,KCNS3,CHRNA7,KCNJ18,KCNIP3,ASPH,ANO2,CACNA2D3,GRIK1,KCNIP4,CACNA2D4,CACNA2D1,HCN1,VWC2L,BEST3,TRPC5,AKAP6,CLCN1,KCNJ12,CHRNB3,GRID2,KCNJ3,PDE4B,PKD1L3,GABRB3,UNC80,GABRG2,KCND2,GRIA4,RYR3,CACHD1,CHRNB4,GABRB1,NALCN,GRIA3,KCNE2,KCNN4,GRIA2*
GO:0098797	plasma membrane protein complex	0.0000245134973915483 7	*TRGC2,TF,CACNB1,GNG12,CHRNB2,KCNQ5,CDH8,GNB4,KCNJ6,ABCG8,CDH11,CACNA1C,GNAI1,CALCR,CHRFAM7A,CD247,CATSPERB,CDH7,KCNQ3,KCNS3,CHRNA7,HMGB1,KCNIP3,TBC1D5,ATP4A,NCF2,STON2,MR1,CACNA2D3,LYN,GRIK1,KCNIP4,TGFBR2,ITGA11,SYNJ1,GPR156,CACNA2D4,CACNA2D1,HCN1,TLR1,ATP1B4,ACVR2A,GNAO1,SNTG2,CTNNB1,ITGB8,BTBD8,VWC2L,IKBKB,TRBV10-2,CHRNB3,ACVR1,HLA-DQA2,GRID2,KCNJ3,IFNLR1,PDE4B,SSPN,GFRA1,KCND2,GRIA4,SNTG1,CACHD1,ATP1B1,TLR6,CDH2,CHRNB4,TFRC,GRIA3,CLTC,ITGA4,CDH9,CALCRL,GNA14,KCNE2,KCNN4,GRIA2*
GO:0005783	endoplasmic reticulum	0.000110772494409299 4	*FSTL1,APBB2,ABCC6,ESYT1,RTN1,MSRB3,OSBPL8,STX8,TRAPPC9,THADA,TF,AHCYL2,CDC14C,SEZ6,ATP10A,SPINK5,PIGL,CAMK2B,GRIP1,SDR16C5,SUMF1,XXYLT1,NOTCH2,SPTLC3,SRL,YBX1,ARB2A,PRKN,PNPLA3,PIK3C2B,GPSM1,AGL,CPEB4,COL4A6,ILDR2,NCK1,CHRM3,COL24A1,COL11A1,CIDEB,MARCHF1,MYO1D,COL1A2,COL12A1,HMGB1,KCNIP3,MAP3K5,ALKBH1,ATP11A,MBTPS1,PIGV,IGFBP7,EPHA5,ATP13A4,SNX10,ATP2C1,TMEM230,ASPH,RRAS2,ZDHHC4,SPON1,TLR8,ITPR1,CYP2A13,CPQ,MR1,F8,GDPD1,ERP29,UVRAG,SEC24C,PDZD8,SIL1,MYRFL,FMO5,TMCC1,PPM1L,TMED7-TICAM2,KCNIP4,NOMO2,NOS1AP,HMGCLL1,SHISA2,KPNB1,RASGRF2,OCA2,SAR1B,APP,CACNA2D1,PI4KB,ELOVL5,USE1,LMAN1L,TICAM2,SCP2,PIP4K2B,LRRK2,GET1-SH3BGR,CLSTN2,PLD1,PKHD1,UBXN7,PJA2,MGST1,CYB5R4,OSBPL3,WNT5A,TEX2,COL19A1,SCFD1,POFUT2,TPST2,SYNE3,VKORC1L1,UGGT2,AKAP6,RAB3GAP2,CEMIP,HLA-DQA2,GOSR2,PLPP3,F5,BNIP3L,AGPAT5,DLC1,RAB18,POU2F1,TMTC1,MGRN1,TMTC2,WLS,RYR3,SPTLC1,AGR3,TRDN,GASK1A,SRI,BDNF,MACO1,ADAMTS9,STIM1,CDH2,MTTP,TENM1,LPCA*

			T2,USP25,MYO5A,WDFY4,BLTP1,VPS13A,OSBPL6,PNPLA8,FBN1,FUT10,PML,MGLL,FOXRED2,PRSS50,AIMP1,HSPBP1,CALCRL,RPL27A,DIPK1A,TMEM30A,SERINC1,TXNRD3,CP,ATP8B1,DPY19L1,GRIA2
GO:0098588	bounding membrane of organelle	0.00011592877532211668	RTN1,HS3ST3A1,EPN2,MREG,NOSTRIN,WSCD1,MAOA,ARL13B,TF,CHST11,USP32,GNB4,ATP7B,CAMK2B,DCT,PECR,SLC38A9,GALNT9,NOTCH2,CD93,DSC1,MAP6,SRL,STK24,DIPK2A,FAM20B,PRKN,SLC5A7,MARCHF9,GPSM1,SLC17A6,GNAI1,ENPEP,MBTPS2,TM9SF2,CLCN3,ZFYVE16,CIDEB,MARCHF1,CDIP1,NDST1,TBC1D5,ATP11A,SLC15A4,GOLPH3,MBTPS1,PIK3C3,ATP13A4,SNX10,ATP2C1,STON2,HUWE1,ASPH,RRAS2,ZDHHC4,TLR8,ARMH3,ROBO1,ITPR1,CAMK1G,MR1,F8,MTX3,MGAM,CHCHD3,UVRAG,PTPRN2,LYN,SEC24C,LARGE1,ATP6V1H,SGMS2,TMED7-TICAM2,NOS3,BPHL,NOS1AP,SYNPR,CUX1,RRAGD,QTRT2,GAD1,OCA2,SAR1B,PI4KB,RHOT1,TLR1,LMAN1L,TICAM2,LRRK2,CLSTN2,PLD1,ITFG2,PJA2,MGST1,ENSG00000288683,SNX13,RABGEF1,SPAG9,ANTXR1,WNT5A,PEX26,ARFGEF2,SCFD1,VPS53,TPST2,BTBD8,DIAPH3,SYNE3,FNIP2,AKAP6,CEMIP,ATF2,HLA-DQA2,MPV17,KIF13A,GOSR2,PLPP3,LAMP2,F5,BNIP3L,ACACB,AGPAT5,INPP4A,TPST1,GRIA4,RAB18,GOLPH3L,WLS,RYR3,PRCP,MYO1B,TRDN,SRI,LDLRAD4,MACO1,TLR6,NDST4,STIM1,KIF16B,RAB11FIP2,ENSG00000290217,CHRNB4,MYOF,LPCAT2,GDAP1,TFRC,VPS13A,OSBPL6,GRIA3,PNPLA8,RAB31,FUT10,CLTC,B3GAT2,ATP6V0A4,ROCK1,PML,SBF2,SPG21,ANKFY1,FNDC3A,SLC30A10,PRKD1,ST6GAL2,SCAMP1,ANPEP,CLVS1,NCALD,HHIP,TMEM30A,UNC13C,CHSY3,PMEPA1,PEX5L,CP,FUNDC1,GRIA2,VDAC2,VPS13B,SPART
GO:0099572	postsynaptic specialization	0.00018637356072965014	RTN1,ARHGEF9,SPTBN1,CHRNB2,GRIP1,DLGAP1,KALRN,SHANK3,CACNA1C,PRKN,NLGN4X,CPEB4,ERBIN,GABRA5,ADD3,PCLO,ANKS1B,ITPR1,PKP4,PAK3,LYN,ADGRB3,GRIK1,DAPK1,INSYN2A,CLSTN2,PJA2,TIAM1,CTNNB1,FXR2,GRID2,PDE4B,KCND2,INPP4A,GRIA4,RGS7BP,CDH2,TANC1,GABRB1,GRIA3,DNAJC6,MAGI2,DLGAP2,GRIA2
GO:0110165	cellular anatomical structure	0.0001935804551517897	FSTL1,C10ORF90,APBB2,PFKP,ABCC6,ESYT1,STYK1,RPL19,RTN1,HS3ST3A1,RGN,TRGC2,CDK14,ZSWIM1,EPN2,MSRB3,PDE1C,MREG,HNRNPCL4,NOSTRIN,ING3,ENSG00000290149,MAP3K3,NINL,WSCD1,SLC12A8,OSBPL8,LRRN1,MED13L,NFIA,SUGCT,POTED,STX8,PHF20,FOXK2,CPNE4,TXNDC8,RBM17,SMYD3,GARS1,RAPGEF6,ARHGEF9,TAFA2,ABR,ISM1,MAOA,BLTP3B,TRAPPC9,SLC16A5,TMEM255A,FNDC1,PREP,MAP3K13,EIF1AX,PPM1A,ARL13B,SAMSN1,RN7SL556P,THADA,LIMD1,IMMP1L,TF,CHST11,THRB,MYBPC1,USP42,IPP,RCSD1,SPTBN1,CDC42EP3,MYO5C,MAPK10,CACNB1,USP32,AHCYL2,DAPP1,ATF7,GNG12,FGF1,CHRNB2,CDC14C,SEZ6,CLDN6,KCNQ5,CDH8,ARHGAP15,GNB4,AGTPBP1,ATF7-NPFF,FHL1,TRMT6,RBPMS,ATP10A,SH3PXD2A,

			SPINK5,MRPS24,OPA3,SKAP2,PRND,PIGL,ATP7B,GPBP1L1,PPM1H,KCNJ6,BRD4,DOK4,ZAN,TNRC18,CAMK2B,GRIP1,ARHGAP11A-SCG5,TLK1,GPATCH8,DDI2,PGM1,DCT,DLGAP1,PECR,SLC38A9,ZNF264,HBP1,SDR16C5,ABCG8,RGPD2,XPO4,OTUD7A,KALRN,SUMF1,USH2A,MAP4K3,XXYLT1,GALNT9,SNORD114-10,NOTCH2,CD93,FKBP5,TMOD2,SLC44A5,SDK2,HMBOX1,ZNF551,RNU2-49P,NPNT,DSC1,EPB41L3,MAP6,MORC1,PARVB,CDH11,SHANK3,WDR26,NR4A3,SPTLC3,ARID1B,E2F7,SLC14A2,FOXO1,ZNF611,STYXL1,STK39,SMURF2,SRL,ADAMTS12,DAAM2,FER1L6,TBC1D16,PLXNA4,CDK18,URB2,YBX1,STK24,DIPK2A,CFAP70,DCDC1,CACNA1C,FAM20B,FAM3C,ASB4,SNORA5B,IDO1,ARB2A,PRKN,NLGN4X,EXOC4,RMND5A,PNPLA3,F13A1,PIK3C2B,XPO6,KDM7A,SLC44A3,SLC5A7,MARCHF9,FAM83B,IFT172,CHD7,GPSM1,CBLL2,PTBP3,CDON,SERPINI1,SLC17A6,NLK,SDF4,THSD7A,GNAI1,MRPL13,MAPKAPK3,NCAM2,PAPOLG,TIAM2,CFAP54,INTS6-AS1,ZNF10,TRAK2,CNMD,LRP8,TAGLN3,CNTN1,AGL,MEOX2,STAT5B,ENPEP,NUDT19,ARHGEF4,UBE2O,MUC17,DDIAS,CPEB4,EIF4ENIF1,CALCR,CHRFAM7A,ELMO1,ERBIN,ZNF121,MBTPS2,RGS22,CD247,MCC,CEP192,PTH,ADAR,DNAH10,PDE10A,TM9SF2,TOX2,PDHX,CLCN3,RHOJ,CDC37,FGF10,ZFYVE16,MECOM,RSPH10B,COL4A6,CATSPERB,TSPAN18,NLRP8,PLG,NEURL4,HERC5,CNTNAP5,NUDT13,CDH7,PGPEP1L,UBE2E2,ILDR2,NCK1,CD84,NONO,RARRES1,KLHL42,NREP,EXO1,GABRA5,EBNA1BP2,SNORD114-9,NEXMIF,KCNQ3,SH3BGR,MROH6,CHRM3,KDM2B,PREX1,RGPD3,AIG1,ZNF404,PMS1,WRNIP1,COL24A1,ANO3,TYW1,GOLGA8R,COL11A1,RSF1,KCTD3,NCOA7,C2ORF88,RNU2-33P,SERTM2,ZNF804A,SEPTIN10,CIDEB,MARCHF1,SLC45A1,MCPH1,MYO1D,SESTD1,BRAF,ZNF214,COL1A2,DTNA,TMOD3,GEMIN5,EEF2KMT,MRTFB,XPO7,KCNS3,VWA3B,COL12A1,ABLIM1,RBMS1,ALPK2,CHRNA7,HECW1,HMGB1,ZNF304,DYNLT2B,CDIP1,AFF3,RFLNA,LPP,HIVEP1,ATRX,TGFB2,MDFIC,EPB41L4A,LAMA2,ADD3,TRIO,ZNF385D,ZNF85,HOXC13,SMARCC1,RAD51B,NDST1,MUSK,KCNJ18,JAM3,KCNIP3,SFPQ,PNPLA4,MAP3K5,ALKBH1,ARPP21,SNORA5A,RGS3,NUP93,ZCCHC4,RERG,OR5AU1,PRKAR2A,RBM33,TBC1D5,CTIF,ATP11A,MRPL33,RBM23,ZNF154,CSTF3,ATP4A,SLC15A4,PCDHB8,GOLPH3,PRKG2,MBTPS1,NCF2,PIK3C3,PIGV,RNU6-1216P,USP10,SND1,HMCN1,IGFBP7,EPHA5,PTPN20,SKOR2,CBR4,ATP13A4,RUNX1,ENAH,SNX10,ATP2C1,ALDH1A2,TMEM230,PPTC7,STON2,PTGFR,IGHV1OR21-1,FAM171A1,STRADA,PCLO,HUWE1,ZNF595,ASPH,RRAS2,SH3BGRL,ZDHHC4,UBR5,GTF2F2,SIM1,KIAA1217,SOX30,TMEM132C,ANKS1B,CHL1,SPOCK3,GSN,PLS1,TCP11L1,SPON1,MAPRE3,ANO2,TLR8,HPS3,KIF21A,SLIT3,ARMH3,NLRP7,ARHGEF39,PHC2,ROBO1,ZNF578,ANKRD11,I

			TPR1,PKP4,CPM,DNAAF11,PAK3,NDUFAF7,ABI3BP,RNU1-83P,KLF12,LOC124900504,AXDND1,IPMK,CYP2A13,TMEM183A,CAMK1G,PRUNE2,DOK6,PRKAG2,MLLT3,CCNT2,BTG4,HAVCR1,TRAPPC10,CPQ,CNOT10,MR1,KRTAP21-2,F8,TNFRSF12A,OASL,GDPD1,CPT2,TAS2R14,VPS8,MTX3,ISY1,CACNA2D3,ZNF429,CXCR5,NCKAP1,PRPF40A,CLDN12,DTD1,CNTN3,MGAM,CILK1,FOSL2,PACRG,IL1RAPL2,ADGRD1,HNRNPCL3,EPHA3,CHCHD3,LATS2,TYW3,MYH14,MRPL32,TPRG1,ERP29,CDYL2,LRRTM4,ARHGAP18,UVRAG,PTPRN2,MYOM2,LYN,ADGRB3,USP6,KLF7,SUMO2,GRIK1,MTA3,UBL3,DIP2B,NME7,SEC24C,LARGE1,ATP6V1H,NUP58,FIGNL1,SLC12A1,PUS7,PDZD8,PAMR1,CCDC33,RRM2,SIL1,LDB2,MYRFL,SPTB,UNC93A,C21ORF62,ALDH1A1,NMNAT3,FMO5,RNASEL,NAV1,DBF4B,PAQR5,CORIN,TMCC1,LRRC7,PCDHB16,DCP2,PPM1L,SGMS2,TMED7-TICAM2,SLC41A2,NOS3,KCNIP4,VSX1,ZNF280B,EIF3L,NOMO2,TNRC6C,PRAMEF27,MLLT10,BPHL,ERCC6,NOS1AP,EIF2AK4,RN7SL738P,KIAA1614,MSRA,SYNPR,RAB12,IMPG1,HMGCLL1,XKR6,MAGEL2,MATN2,TGFBR2,CMKLR2,ITGA11,CFAP77,MARK4,DDX60L,MDN1,SYNJ1,TCF7L2,ZNF800,CHAF1B,ZNF462,MTRF1,ECHDC2,OVCH1,CRTAC1,E2F6,ZFHX3,CUX1,POLR2C,UNC119,SHISA2,GPM6B,RRAGD,ULK3,GPR156,ZMAT4,GKAP1,CACNA2D4,LHFPL3,PPP2R5E,KPNB1,GSDMD,ADGRV1,RASGRF2,ST7,SERBP1,QTRT2,EVA1C,EPB41L5,GAD1,SCG5,NUDCD1,WAC,WHRN,OCA2,DAPK1,TDP2,ALDH9A1,ZNF678,ACSM2B,PRUNE1,SAR1B,TTC7B,INSYN2A,BTG3,APP,GATA4,ATF7IP2,SMAD5,IGF2BP1,PPIL6,MAMLD1,RNLS,GSKIP,RNF38,ADAM12,UBE2G1,NBPF12,PELI2,ATP9B,CACNA2D1,DHX35,SP140L,HCN1,CHRM5,SLC13A1,PI4KB,FRY,RHOT1,ELOVL5,HERC2,HOOK3,USE1,PIK3R5,LYZL1,PALLD,TLR1,CECR2,TAS2R30,TMCO3,BMT2,RIPK4,AVPR1B,LMAN1L,QDPR,FRRS1,PTK2,NOP9,USP7,TICAM2,SCP2,SH2B2,ATP1B4,ZNF41,AVEN,GATD3,CELF1,HYCC1,DNAH12,POGZ,PIP4K2B,UQCC1,LRRK2,NKAIN3,INO80D,ZNF395,IKZF3,GET1-SH3BGR,SLC38A4,CLSTN2,ARIH1,CFAP298,PLD1,ITFG2,VTCN1,PKHD1,MEG8,TENT5C,UBXN7,RARB,SNORD114-7,PJA2,TCF4,MGST1,RBM44,ARHGEF38,PAEP,COP1,TIAM1,CSRP3,PHACTR1,MLIP,IQSEC2,ACVR2A,GNAO1,ASAP2,TIPIN,VIT,C6ORF58,ENSG00000288635,SNTG2,SNORA5C,NTM,MEIS1,AMMECR1,NEDD4L,BRCC3,ENSG00000288683,PRMT8,SNX13,RABGEF1,CYB5R4,KIT,TBC1D8,EPHA7,SPAG9,OSBPL3,ANTXR1,C19ORF47,SPDL1,WNT5A,TRABD2B,WIPI2,PEX26,ARFGEF2,TEX2,CTNNB1,ANKRD17,COL19A1,RUNX1T1,CTSK,PRKDC,IPO5,ESR1,TNFRSF19,ZNF479,LUZP2,PTPN14,SCFD1,ITGB8,ERC2,POFUT2,DMGDH,BCAR3,TPD52L1,L3MBTL4,PPM1E,ZXDC,VPS53,FRMD4A,LMX1A,DCAF8L1,WWOX,TPST2,FIGN,

			BTBD8,PARS2,BNIPL,HUNK,NEK10,FILIP1,TMEM62,ANAPC10,FBXO15,PTGER4,GREB1L,ZNF215,BBX,PDE7B,USP40,VWC2L,BEST3,LYPD6B,DOCK1,DIAPH3,CCDC107,GPC4,BPGM,PKIG,EFNA5,IGF2BP2,ACTR3B,ZNF722,CDC14B,SYNE3,PLCH1,OR1M1,HECTD1,VKORC1L1,NGEF,TRPC5,NEUROD2,FNIP2,AASS,JAML,KAT14,UGGT2,CCNJ,TNFSF11,PTPRF,IKBKB,FXR2,SH3BP5,AKAP6,TRBV10-2,USF3,LYVE1,APOH,ARHGEF28,OR8B8,CLCN1,RAB3GAP2,TASOR,STEAP1B,CCPG1,GPRIN1,EML6,POU6F2,LINGO2,OPCML,CEMIP,ATF2,RNU6-986P,ZNF426,KCNJ12,HDAC8,CHRNB3,ACVR1,HLA-DQA2,PID1,COL6A5,HYAL4,EBF2,CCDC91,GRID2,ZNF423,ZCCHC17,KCNJ3,RB1,NSMCE1,MPV17,IFNLR1,RNU6-826P,NTNG1,PDE4B,DDX10,PKD1L3,KIF13A,NLRP4,ADIPOR1,LOC124900205,ZNF143,GABRB3,GOSR2,PET117,SHROOM4,ZSCAN23,SMG7,PLPP3,UNC80,HNF1B,RNU6-1311P,IMMP2L,ZNF615,LAMP2,SSPN,SPSB1,KIRREL3,GABRG2,F5,CFAP95,AK5,CPS1,NCOA2,TMEM120A,BNIP3L,PRKCH,ACACB,AGPAT5,RABGAP1L,DLC1,ENSG00000286476,DENND2B,XRCC5,TBRG4,RGS17,GFRA1,SHLD2,SPHKAP,KCND2,INPP4A,BMX,ZNF418,SLC24A3,RAD54L2,MPRIP,TAF15,TPST1,GRIA4,RNA5SP489,POLD3,RAB18,ZNF860,TPH2,POU2F1,PRPF40B,TMTC1,ETV1,NRK,RGS7BP,MGRN1,TEX11,MID2,RBM28,LRTM2,SIK1,FBXL13,ZCWPW2,ERCC6L2,PIPOX,TP53BP2,ZNF850,KAT2B,GOLPH3L,TMTC2,WLS,ACTR3C,ANKS1A,IGKV1-6,RYR3,TMPRSS11B,PRCP,SUPV3L1,MEF2D,MYO1B,ZPLD1,MGAM2,MYL4,GTF2A1,SPTLC1,AGR3,CDK5RAP2,FLVCR1,UBE2E3,RFC3,FARSB,ARHGEF7,SAMD12,DACH1,ATF3,TRDN,GASK1A,NPHP1,FBN2,IKZF1,SLC26A8,CAPZA2,SNTG1,CACHD1,SRI,ATP1B1,GREB1,ADAT2,ARNT,BDNF,LDLRAD4,TFCP2L1,ACBD6,SEMA3E,BCLAF3,SCIN,MALRD1,TENT4B,MACO1,ZNF665,TLR6,NDST4,ADAMTS9,STIM1,TSEN15,TMEM237,TMEM161B,HAUS6,KIF16B,RAB11FIP2,CDH2,EEFSEC,COX7B2,LAG3,PPARG,TANC1,PAPPA,ENSG00000290217,ASB3,MTTP,CHRNB4,USP9X,TGM5,NEK7,INTS15,ZNF80,TENM1,IL13RA2,RORB,CDK6,GABRB1,MYOF,LTB4R2,JAKMIP1,SH3KBP1,NALCN,SMOX,AVIL,LPCAT2,GDAP1,AMY2A,DDX60,TFAP2D,MYLK4,BEND7,USP25,KIF2C,MYO5A,SIPA1L3,SLC6A13,LRFN2,WDFY4,SPTBN5,SFRP4,BLTP1,PNRC1,IL1RAP,JCAD,VAV2,TFRC,VPS13A,TES,OSBPL6,GRIA3,PNPLA8,FBN1,HAPSTR1,RAB31,DIPK2B,MAP3K1,ZFP82,DDX4,IQCB1,COA1,CLEC6A,ZNF292,RAPGEF4,BMPER,FUT10,RNU6-984P,CLASP2,CLTC,ZNF521,RPRD2,ZNF761,ANGPT1,SEMA6A,VIPR2,EIF4G1,TMPRSS15,B3GAT2,ATP6V0A4,ACSM5,PHLPP1,ZNF618,ITGA4,DNAJC6,ROCK1,NCAPH,M1AP,TAX1BP1,TBC1D4,APIP,MMP28,ZNF701,KCNT2,NUP98,CLEC3A

			,IKZF2,PML,FOXO3B,SACS,AFF2,LAYN,MGLL,SCARA5,LONRF3,SLC5A12,PLEK2,TNFSF4,AFAP1L2,DGKG,SPATA25,SP100,RNA5SP260,PRKG1,PLXDC2,ELAVL4,SBF2,CDH9,FOXRED2,NDUFAF2,UMODL1,RFPL4B,TTC28,TSEN2,MAGI2,GALK2,PCDH19,NIN,SPG21,ZNF286A,ANKFY1,ZNF69,PRSS50,ASIC2,ENSA,FNDC3A,KHDRBS3,AIMP1,SLC30A10,AP4S1,TPK1,HSPBP1,PRKD1,CALCRL,SLC20A2,USP34,STK4,PCDH10,TNNI3K,RAB6D,TMEM131,SPC25,TMEM161B-DT,ST6GAL2,RNASE11,SCAMP1,PTCHD4,RPL27A,ANPEP,PRLR,ZNF569,PTPRA,ZNF180,PDE11A,NMI,RAB6C,PCCA,CLVS1,YLPM1,EYS,IL16,DIPK1A,ZSWIM7,ZNF470,POTEH,EDARADD,GNA14,CR2,LPAR6,CTBP2,TTN,NCALD,HHIP,DYRK1A,PPFIA1,TMEM30A,MYRIP,PPP1R1C,KCNE2,UNC13C,CUL4A,SERINC1,KCTD16,CHSY3,TXNRD3,EYA4,ZNF888,DLGAP2,ARL15,ZNF736,MAP7D3,SEMA5A,ELL2,LAMA4,PMEPA1,ATE1,ETNK1,TTPA,PEX5L,PVT1,CHAC2,GATAD2B,METTL3,FMNL2,KRT85,UAP1,PARD3,KCNN4,TRDC,ZNF33A,SLC25A48,CP,FUNDC1,RNU6-849P,NPAS3,COL6A6,BMAL2,ATP8B1,PGPEP1,RNF6,SEMA3C,VAV1,SMYD1,FANCB,PEPD,DPY19L1,GRIA2,VDAC2,VPS13B,SPART,IGLV4-3
GO:0098590	plasma membrane region	0.000219073490512989 73	*ABCC6,MREG,RAPGEF6,ARL13B,TF,SPTBN1,CHRNB2,CLDN6,KCNQ5,CDH8,ATP7B,GRIP1,ABCG8,USH2A,EPB41L3,SLC14A2,STK39,CACNA1C,NLGN4X,SLC5A7,LRP8,CNTN1,ENPEP,ARHGEF4,MUC17,ERBIN,CLCN3,GABRA5,CHRM3,MYO1D,ALPK2,CHRNA7,MUSK,PRKAR2A,ATP4A,PRKG2,HMCN1,ANO2,NCKAP1,MGAM,LRRTM4,ADGRB3,GRIK1,SLC12A1,NOS3,NOS1AP,KIAA1614,TGFBR2,SYNJ1,ADGRV1,EPB41L5,WHRN,HCN1,CHRM5,SLC13A1,LRRK2,SLC38A4,CLSTN2,PLD1,PKHD1,TIAM1,ANTXR1,CTNNB1,CTSK,ERC2,DIAPH3,EFNA5,AKAP6,CHRNB3,GRID2,KCNJ3,NTNG1,GABRB3,SHROOM4,PLPP3,SSPN,GABRG2,DLC1,KCND2,BMX,GRIA4,RAB18,RGS7BP,GASK1A,SNTG1,ATP1B1,STIM1,CDH2,MTTP,CHRNB4,GABRB1,MYOF,SIPA1L3,SLC6A13,LRFN2,JCAD,TFRC,GRIA3,CLASP2,ATP6V0A4,DNAJC6,SLC5A12,PLEK2,CDH9,SLC20A2,PTPRA,HHIP,TMEM30A,KCNE2,UNC13C,KCTD16,PARD3,ATP8B1,GRIA2*
GO:0005794	Golgi apparatus	0.000809069019899502 3	*APBB2,RTN1,HS3ST3A1,WSCD1,STX8,TXNDC8,TRAPPC9,CHST11,USP32,ATP7B,KCNJ6,GALNT9,NOTCH2,MAP6,STK24,DIPK2A,FAM20B,FAM3C,PRKN,MARCHF9,GPSM1,SDF4,MUC17,MBTPS2,CD247,TM9SF2,CLCN3,GOLGA8R,CIDEB,MARCHF1,KCNS3,NDST1,JAM3,KCNIP3,TBC1D5,ATP11A,GOLPH3,MBTPS1,ATP2C1,TMEM230,HUWE1,RRAS2,ZDHHC4,TLR8,ARMH3,CAMK1G,TRAPPC10,CPQ,MR1,F8,LYN,USP6,LARGE1,ATP6V1H,SGMS2,TMED7-TICAM2,NOS3,CUX1,GKAP1,SAR1B,APP,MAMLD1,ATP9B,PI4KB,HOOK3,TLR1,AVPR1B,LMAN1L,TICAM2,LRRK2,CLSTN2,PLD1,ITFG2,PKHD1,PJA2,ASAP2,NEDD4L,WNT5A,ARFGEF2,IPO5,ESR1,SCFD1,POFUT2,VPS53,WWOX,TPST2,GPC4,HLA-*

			DQA2,CCDC91,KIF13A,GOSR2,PLPP3,LAMP2,RABGAP1L,GFRA1,TPST1,RAB18,GOLPH3L,WLS,MYO1B,CDK5RAP2,DACH1,GASK1A,LDLRAD4,MALRD1,TLR6,NDST4,MTTP,TENM1,LPCAT2,SIPA1L3,VPS13A,RAB31,FUT10,CLASP2,CLTC,B3GAT2,ROCK1,PRKG1,SPG21,FNDC3A,AIMP1,SLC30A10,AP4S1,PRKD1,RAB6D,ST6GAL2,SCAMP1,RAB6C,CLVS1,NCALD,TMEM30A,CHSY3,PMEPA1,METTL3,ATP8B1,VPS13B
GO:0032279	asymmetric synapse	0.002681111049774948	*RTN1,ARHGEF9,SPTBN1,GRIP1,DLGAP1,KALRN,SHANK3,PLXNA4,CACNA1C,PRKN,NLGN4X,CPEB4,ADD3,PCLO,ANKS1B,ITPR1,PKP4,PAK3,ADGRB3,GRIK1,DAPK1,INSYN2A,CLSTN2,PJA2,TIAM1,ARFGEF2,CTNNB1,FXR2,GRID2,PDE4B,INPP4A,GRIA4,RGS7BP,CDH2,TANC1,GRIA3,DNAJC6,MAGI2,DLGAP2,GRIA2*
GO:0005938	cell cortex	0.003310124029977625 6	*OSBPL8,SPTBN1,FGF1,SPINK5,STK39,EXOC4,GPSM1,GNAI1,SEPTIN10,MYO1D,ADD3,HMCN1,PCLO,ASPH,GSN,PLS1,SPTB,KIAA1614,GAD1,FRY,PTK2,CTNNB1,ERC2,SHROOM4,DLC1,DENND2B,ARHGEF7,CAPZA2,SCIN,STIM1,CDH2,SPTBN5,CLASP2,PRKD1,CTBP2,MYRIP,UNC13C,PARD3*
GO:0043235	receptor complex	0.003332554131284972	*TRGC2,TF,CHRNB2,ABCG8,NOTCH2,PLXNA4,LRP8,CALCR,CHRFAM7A,CD247,GABRA5,CHRNA7,MUSK,PTPRN2,LYN,GRIK1,TGFBR2,ITGA11,GPR156,ADGRV1,APP,TLR1,ACVR2A,KIT,ITGB8,VWC2L,IKBKB,TRBV10-2,CHRNB3,ACVR1,GRID2,IFNLR1,PKD1L3,GABRB3,GABRG2,GFRA1,GRIA4,ARNT,TLR6,PPARG,MTTP,CHRNB4,IL13RA2,GABRB1,TFRC,GRIA3,ITGA4,CALCRL,PRLR,PTPRA,CR2,KCTD16,PEX5L,BMAL2,GRIA2*
GO:0031090	organelle membrane	0.004466278901453831 5	*ABCC6,ESYT1,RTN1,HS3ST3A1,EPN2,MREG,NOSTRIN,WSCD1,OSBPL8,PHF20,MAOA,ARL13B,THADA,IMMP1L,TF,CHST11,USP32,CDC14C,GNB4,ATP10A,SPINK5,MRPS24,PIGL,ATP7B,TNRC18,CAMK2B,GRIP1,DCT,PECR,SLC38A9,SDR16C5,XXYLT1,GALNT9,NOTCH2,CD93,DSC1,MAP6,SPTLC3,SRL,STK24,DIPK2A,FAM20B,PRKN,PNPLA3,SLC5A7,MARCHF9,GPSM1,SLC17A6,GNAI1,MRPL13,ENPEP,ERBIN,MBTPS2,TM9SF2,CLCN3,ZFYVE16,ILDR2,NCK1,CHRM3,GOLGA8R,CIDEB,MARCHF1,CDIP1,NDST1,MAP3K5,NUP93,TBC1D5,ATP11A,MRPL33,SLC15A4,GOLPH3,PRKG2,MBTPS1,PIK3C3,PIGV,ATP13A4,SNX10,ATP2C1,STON2,HUWE1,ASPH,RRAS2,ZDHHC4,TLR8,ARMH3,ROBO1,ITPR1,CYP2A13,CAMK1G,MR1,F8,GDPD1,CPT2,MTX3,MGAM,EPHA3,CHCHD3,MRPL32,UVRAG,PTPRN2,LYN,SEC24C,LARGE1,ATP6V1H,NUP58,PDZD8,MYRFL,FMO5,TMCC1,PPM1L,SGMS2,TMED7-TICAM2,NOS3,NOMO2,BPHL,NOS1AP,SYNPR,HMGCLL1,SYNJ1,CUX1,SHISA2,RRAGD,KPNB1,GSDMD,RASGRF2,QTRT2,GAD1,OCA2,SAR1B,APP,PI4KB,RHOT1,ELOVL5,USE1,TLR1,LMAN1L,TICAM2,ATP1B4,PIP4K2B,UQCC1,LRRK2,GET1-SH3BGR,CLSTN2,PLD1,ITFG2,UBXN7,PJA2,MGST1,ASAP2,ENSG00000288683,SNX13,RABGEF1,CYB5R4,SPAG9,OSBPL3,ANTXR1,WNT5A,TRA*

			BD2B,PEX26,ARFGEF2,TEX2,ANKRD17,IPO5,SCFD1,POFUT2,VPS53,TPST2,BTBD8,DIAPH3,SYNE3,VKORC1L1,FNIP2,SH3BP5,AKAP6,RAB3GAP2,CEMIP,ATF2,HLA-DQA2,MPV17,KIF13A,GABRB3,GOSR2,PLPP3,IMMP2L,LAMP2,GABRG2,F5,CPS1,TMEM120A,BNIP3L,ACACB,AGPAT5,INPP4A,TPST1,GRIA4,RAB18,GOLPH3L,TMTC2,WLS,RYR3,PRCP,MYO1B,ZPLD1,SPTLC1,FLVCR1,TRDN,SRI,ATP1B1,LDLRAD4,MALRD1,MACO1,TLR6,NDST4,STIM1,KIF16B,RAB11FIP2,COX7B2,ENSG00000290217,CHRNB4,MYOF,SH3KBP1,SMOX,LPCAT2,GDAP1,SPTBN5,BLTP1,TFRC,VPS13A,OSBPL6,GRIA3,PNPLA8,RAB31,COA1,FUT10,CLTC,B3GAT2,ATP6V0A4,ROCK1,NUP98,PML,MGLL,SBF2,NDUFAF2,SPG21,ANKFY1,FNDC3A,SLC30A10,AP4S1,PRKD1,ST6GAL2,SCAMP1,ANPEP,CLVS1,DIPK1A,NCALD,HHIP,TMEM30A,UNC13C,SERINC1,CHSY3,PMEPA1,PEX5L,SLC25A48,CP,FUNDC1,RNF6,DPY19L1,GRIA2,VDAC2,VPS13B,SPART
GO:0014069	postsynaptic density	0.004755271374444474	*RTN1,ARHGEF9,SPTBN1,GRIP1,DLGAP1,KALRN,SHANK3,CACNA1C,PRKN,NLGN4X,CPEB4,ADD3,PCLO,ANKS1B,ITPR1,PKP4,PAK3,ADGRB3,GRIK1,DAPK1,INSYN2A,CLSTN2,PJA2,TIAM1,CTNNB1,FXR2,GRID2,PDE4B,INPP4A,GRIA4,RGS7BP,CDH2,TANC1,GRIA3,DNAJC6,MAGI2,DLGAP2,GRIA2*
GO:0005768	endosome	0.005082850212045622	*APBB2,EPN2,MREG,STX8,BLTP3B,TRAPPC9,TF,RCSD1,ATP7B,SLC38A9,TBC1D16,SLC5A7,SDF4,TRAK2,TM9SF2,CLCN3,ZFYVE16,MARCHF1,MYO1D,HMGB1,CDIP1,TBC1D5,ATP11A,SLC15A4,GOLPH3,PIK3C3,USP10,ATP13A4,SNX10,TMEM230,TLR8,HPS3,TRAPPC10,MR1,VPS8,EPHA3,UVRAG,USP6,ATP6V1H,MAGEL2,OCA2,APP,ATP9B,PI4KB,AVPR1B,TICAM2,LRRK2,PLD1,NEDD4L,SNX13,RABGEF1,ANTXR1,ARFGEF2,CTSK,VPS53,DIAPH3,STEAP1B,HLA-DQA2,KIF13A,GOSR2,LAMP2,RABGAP1L,DENND2B,GFRA1,INPP4A,MGRN1,WLS,MYO1B,LDLRAD4,KIF16B,RAB11FIP2,MYO5A,WDFY4,TFRC,VPS13A,OSBPL6,RAB31,CLTC,ATP6V0A4,PML,SBF2,MAGI2,SPG21,ANKFY1,SLC30A10,AP4S1,CALCRL,SCAMP1,PRLR,CLVS1,TMEM30A,PMEPA1,TTPA,VPS13B*
GO:0030427	site of polarized growth	0.005998213323602621	*APBB2,EXOC4,TIAM2,CPEB4,PREX1,ZNF804A,MYH14,WHRN,APP,IGF2BP1,FRY,PALLD,LRRK2,TIAM1,ERC2,NGEF,TRPC5,FXR2,GPRIN1,USP9X,MYO5A,CLASP2,ITGA4,ELAVL4,NIN*
GO:0005769	early endosome	0.008477722439144825	*APBB2,STX8,BLTP3B,TF,RCSD1,TBC1D16,SLC5A7,TRAK2,CLCN3,ZFYVE16,MARCHF1,MYO1D,ATP11A,SLC15A4,USP10,ATP13A4,TMEM230,HPS3,MR1,VPS8,EPHA3,UVRAG,MAGEL2,APP,TICAM2,SNX13,RABGEF1,RABGAP1L,INPP4A,MGRN1,WLS,MYO1B,LDLRAD4,KIF16B,MYO5A,WDFY4,TFRC,OSBPL6,RAB31,PML,ANKFY1,SLC30A10,CLVS1,TMEM30A,PMEPA1,VPS13B*
GO:0005856	cytoskeleton	0.008892749529719907	*C10ORF90,NOSTRIN,NINL,RAPGEF6,ARL13B,IPP,RCSD1,SPTBN1,CDC42EP3,MYO5C,CDC14C,SH3PXD2A,CAMK2B,KALRN,USH2A,TMOD2,HMBOX1,EPB41L3,MAP6,PARVB,CFAP70,DCDC1,EXO*

			C4,IFT172,GNAI1,CFAP54,LRP8,TAGLN3,RGS22,CEP192,DNAH10,TM9SF2,RSPH10B,NEURL4,KLHL42,ZNF804A,SEPTIN10,MCPH1,MYO1D,SESTD1,DTNA,TMOD3,ABLIM1,DYNLT2B,RFLNA,LPP,EPB41L4A,ADD3,JAM3,NUP93,PRKAR2A,PIK3C3,USP10,PTPN20,ENAH,SNX10,PCLO,GTF2F2,KIAA1217,ANKS1B,GSN,PLS1,TCP11L1,MAPRE3,KIF21A,PKP4,KRTAP21-2,NCKAP1,CILK1,PACRG,EPHA3,LATS2,MYH14,ARHGAP18,UVRAG,NME7,SPTB,NAV1,CORIN,LRRC7,NOS3,NOS1AP,MSRA,CFAP77,MARK4,MDN1,SYNJ1,UNC119,RRAGD,EPB41L5,WHRN,DAPK1,APP,MAMLD1,FRY,HERC2,HOOK3,PIK3R5,PALLD,PTK2,SH2B2,DNAH12,LRRK2,CFAP298,PKHD1,TENT5C,PJA2,TIAM1,CSRP3,SNTG2,BRCC3,SPAG9,SPDL1,ARFGEF2,CTNNB1,PTPN14,ERC2,FRMD4A,FIGN,FILIP1,DIAPH3,IGF2BP2,ACTR3B,CDC14B,FNIP2,KAT14,EML6,RB1,PDE4B,KIF13A,SHROOM4,CFAP95,AK5,DLC1,SHLD2,MPRIP,MID2,FBXL13,ERCC6L2,KAT2B,MYO1B,MYL4,CDK5RAP2,ARHGEF7,NPHP1,CAPZA2,SNTG1,SCIN,STIM1,HAUS6,KIF16B,CDH2,USP9X,NEK7,TENM1,CDK6,MYOF,JAKMIP1,SH3KBP1,AVIL,DDX60,KIF2C,MYO5A,SIPA1L3,SPTBN5,IQCB1,CLASP2,CLTC,ROCK1,PLEK2,DGKG,TTC28,TSEN2,MAGI2,NIN,RAB6C,EYS,TTN,DYRK1A,MYRIP,DLGAP2,MAP7D3,KRT85,PARD3
GO:0098984	neuron to neuron synapse	0.010137993527368033	RTN1,ARHGEF9,SPTBN1,GRIP1,DLGAP1,KALRN,SHANK3,PLXNA4,CACNA1C,PRKN,NLGN4X,CPEB4,ADD3,PCLO,ANKS1B,ITPR1,PKP4,PAK3,ADGRB3,GRIK1,DAPK1,INSYN2A,CLSTN2,PJA2,TIAM1,ARFGEF2,CTNNB1,FXR2,GRID2,PDE4B,INPP4A,GRIA4,RGS7BP,CDH2,TANC1,GRIA3,RAPGEF4,DNAJC6,MAGI2,DLGAP2,GRIA2
GO:0031984	organelle subcompartment	0.010375461389249285	ABCC6,ESYT1,RTN1,OSBPL8,STX8,TRAPPC9,THADA,CDC14C,ATP10A,SPINK5,PIGL,ATP7B,CAMK2B,GRIP1,SDR16C5,XXYLT1,NOTCH2,SPTLC3,SRL,PNPLA3,MARCHF9,GPSM1,ILDR2,CHRM3,GOLGA8R,CIDEB,MARCHF1,NDST1,MAP3K5,ATP11A,GOLPH3,MBTPS1,PIGV,ATP13A4,ATP2C1,TMEM230,ASPH,ZDHHC4,TLR8,ITPR1,CYP2A13,MR1,GDPD1,SEC24C,PDZD8,MYRFL,FMO5,TMCC1,PPM1L,TMED7-TICAM2,NOMO2,NOS1AP,HMGCLL1,SHISA2,KPNB1,RASGRF2,OCA2,SAR1B,APP,ATP9B,PI4KB,ELOVL5,USE1,LMAN1L,PIP4K2B,LRRK2,GET1-SH3BGR,CLSTN2,PLD1,UBXN7,PJA2,MGST1,ASAP2,CYB5R4,OSBPL3,ARFGEF2,TEX2,SCFD1,POFUT2,VPS53,VKORC1L1,AKAP6,RAB3GAP2,HLA-DQA2,CCDC91,KIF13A,GOSR2,PLPP3,LAMP2,AGPAT5,RAB18,GOLPH3L,TMTC2,WLS,RYR3,MYO1B,SPTLC1,TRDN,SRI,MACO1,STIM1,LPCAT2,BLTP1,VPS13A,OSBPL6,PNPLA8,RAB31,FUT10,CLASP2,CLTC,PML,MGLL,AP4S1,PRKD1,ST6GAL2,SCAMP1,CLVS1,DIPK1A,SERINC1,CHSY3,ATP8B1,DPY19L1,GRIA2,VPS13B
GO:0030426	growth cone	0.010669545728160543	APBB2,EXOC4,TIAM2,CPEB4,PREX1,ZNF804A,MYH14,WHRN,APP,IGF2BP1,PALLD,LRRK2,TIAM1,ERC2,NGEF,TRPC5,FXR2,GPRIN1,USP9X,MYO5A,CLASP2,ITGA4,ELAVL4,NIN

GO:0031982	vesicle	0.01502895812202344	*FSTL1,APBB2,PFKP,EPN2,MREG,NOSTRIN,STX8,CPNE4,TXNDC8,GARS1,RAPGEF6,BLTP3B,TRAPPC9,TF,RCSD1,SPTBN1,MYO5C,GNG12,GNB4,SPINK5,ATP7B,CAMK2B,GRIP1,ARHGAP11A-SCG5,PGM1,DCT,SLC38A9,KALRN,CD93,FKBP5,NPNT,DSC1,MAP6,CDH11,DAAM2,TBC1D16,YBX1,STK24,DIPK2A,CFAP70,FAM3C,PRKN,F13A1,PIK3C2B,SLC5A7,IFT172,SERPINI1,SLC17A6,SDF4,GNAI1,TIAM2,TRAK2,CNTN1,AGL,ENPEP,CALCR,TM9SF2,CLCN3,RHOJ,CDC37,ZFYVE16,PLG,NCK1,RARRES1,CIDEB,MARCHF1,MYO1D,COL1A2,COL12A1,HMGB1,CDIP1,TGFB2,PRKAR2A,TBC1D5,ATP11A,SLC15A4,GOLPH3,NCF2,PIK3C3,USP10,SND1,HMCN1,IGFBP7,ATP13A4,SNX10,TMEM230,STON2,PCLO,HUWE1,RRAS2,SH3BGRL,CHL1,GSN,PLS1,TLR8,HPS3,ITPR1,CPM,MLLT3,TRAPPC10,CPQ,MR1,F8,VPS8,NCKAP1,MGAM,PACRG,EPHA3,CHCHD3,MYH14,ERP29,UVRAG,PTPRN2,LYN,USP6,UBL3,DIP2B,SEC24C,ATP6V1H,FIGNL1,SLC12A1,C21ORF62,ALDH1A1,PPM1L,NOS3,MSRA,SYNPR,MAGEL2,SYNJ1,CRTAC1,KPNB1,GSDMD,ADGRV1,SERBP1,GAD1,SCG5,OCA2,ALDH9A1,SAR1B,APP,UBE2G1,ATP9B,CACNA2D1,PI4KB,USE1,TLR1,AVPR1B,LMAN1L,QDPR,TICAM2,UQCC1,LRRK2,PLD1,PKHD1,MGST1,C6ORF58,NEDD4L,SNX13,RABGEF1,KIT,SPAG9,ANTXR1,WNT5A,ARFGEF2,CTNNB1,CTSK,SCFD1,ITGB8,VPS53,BTBD8,DIAPH3,GPC4,BPGM,ACTR3B,PTPRF,SH3BP5,LYVE1,APOH,STEAP1B,CEMIP,HLA-DQA2,HYAL4,PDE4B,PKD1L3,KIF13A,GABRB3,GOSR2,LAMP2,SSPN,KIRREL3,GABRG2,F5,PRKCH,RABGAP1L,DENND2B,XRCC5,GFRA1,INPP4A,GRIA4,RAB18,MGRN1,MID2,WLS,ACTR3C,TMPRSS11B,PRCP,MYO1B,ZPLD1,CDK5RAP2,CAPZA2,SRI,ATP1B1,GREB1,BDNF,LDLRAD4,SCIN,MALRD1,TLR6,KIF16B,RAB11FIP2,ENSG00000290217,MTTP,CHRNB4,MYOF,SH3KBP1,AMY2A,BEND7,MYO5A,SLC6A13,WDFY4,TFRC,VPS13A,OSBPL6,GRIA3,RAB31,IQCB1,CLTC,ANGPT1,ATP6V0A4,ITGA4,DNAJC6,ROCK1,TAX1BP1,TBC1D4,PML,SLC5A12,PRKG1,SBF2,MAGI2,SPG21,ANKFY1,FNDC3A,SLC30A10,AP4S1,CALCRL,SLC20A2,SCAMP1,ANPEP,PRLR,PTPRA,CLVS1,EYS,GNA14,CR2,TTN,NCALD,TMEM30A,MYRIP,UNC13C,ARL15,SEMA5A,LAMA4,PMEPA1,TTPA,KCNN4,CP,SEMA3C,PEPD,GRIA2,VDAC2,VPS13B*
GO:0097060	synaptic membrane	0.015364676095056899	*CHRNB2,KCNQ5,CDH8,GRIP1,CACNA1C,NLGN4X,SLC5A7,CNTN1,GABRA5,CHRM3,CHRNA7,MUSK,ANO2,LRRTM4,ADGRB3,GRIK1,SYNJ1,HCN1,CHRM5,CLSTN2,TIAM1,CTNNB1,ERC2,CHRNB3,GRID2,KCNJ3,NTNG1,GABRB3,SSPN,GABRG2,KCND2,GRIA4,RGS7BP,CHRNB4,GABRB1,LRFN2,GRIA3,DNAJC6,CDH9,PTPRA,UNC13C,KCTD16,GRIA2*
GO:0019898	extrinsic component of membrane	0.022832144020667966	*ESYT1,GNG12,CDH8,GNB4,KALRN,CDH11,PIK3C2B,GNAI1,CDH7,DTNA,TRIO,PIK3C3,SNX10,UVRAG,LYN,ATP6V1H,PIK3R5,TIAM1,GNAO1,WIPI2,CTNNB1,GFRA1,CDH2,VPS13A,DNAJC6,CDH9,GNA14*
GO:0015	actin	0.02885814	*C10ORF90,IPP,RCSD1,SPTBN1,CDC42EP3,MYO*

629	cytoskeleton	4014468068	*5C,SH3PXD2A,KALRN,TMOD2,PARVB,TAGLN3,RGS22,MYO1D,TMOD3,ABLIM1,RFLNA,LPP,JAM3,GSN,PLS1,NCKAP1,EPHA3,MYH14,SPTB,CORIN,NOS1AP,MSRA,WHRN,DAPK1,PALLD,PTK2,SH2B2,FILIP1,DIAPH3,SHROOM4,DLC1,SHLD2,MPRIP,KAT2B,MYO1B,MYL4,CAPZA2,SCIN,CDH2,AVIL,MYO5A,SIPA1L3,SPTBN5,TTN,DYRK1A,MYRIP*
GO:0005802	trans-Golgi network	0.035372650551689845	*STX8,TRAPPC9,ATP7B,MARCHF9,MARCHF1,NDST1,GOLPH3,ATP2C1,TMEM230,APP,ATP9B,LRRK2,ARFGEF2,VPS53,HLA-DQA2,CCDC91,KIF13A,PLPP3,LAMP2,GOLPH3L,WLS,MYO1B,RAB31,CLASP2,CLTC,AP4S1,PRKD1,SCAMP1,CLVS1,ATP8B1,VPS13B*
GO:0045211	postsynaptic membrane	0.040159655295530325	*CHRNB2,GRIP1,CACNA1C,NLGN4X,CNTN1,GABRA5,CHRM3,CHRNA7,MUSK,LRRTM4,ADGRB3,GRIK1,HCN1,CHRM5,CLSTN2,TIAM1,CTNNB1,CHRNB3,GRID2,GABRB3,SSPN,GABRG2,KCND2,GRIA4,RGS7BP,CHRNB4,GABRB1,LRFN2,GRIA3,CDH9,KCTD16,GRIA2*
GO:0031410	cytoplasmic vesicle	0.04030961972906744	*APBB2,EPN2,MREG,NOSTRIN,STX8,GARS1,RAPGEF6,BLTP3B,TRAPPC9,TF,RCSD1,SPINK5,ATP7B,CAMK2B,GRIP1,ARHGAP11A-SCG5,PGM1,DCT,SLC38A9,CD93,DSC1,MAP6,TBC1D16,DIPK2A,FAM3C,PRKN,F13A1,PIK3C2B,SLC5A7,IFT172,SERPINI1,SLC17A6,SDF4,TRAK2,AGL,ENPEP,CALCR,TM9SF2,CLCN3,ZFYVE16,PLG,CIDEB,MARCHF1,MYO1D,HMGB1,CDIP1,TGFB2,TBC1D5,ATP11A,SLC15A4,GOLPH3,NCF2,PIK3C3,USP10,SND1,ATP13A4,SNX10,TMEM230,STON2,HUWE1,GSN,TLR8,HPS3,ITPR1,TRAPPC10,MR1,F8,VPS8,MGAM,EPHA3,ERP29,UVRAG,PTPRN2,LYN,USP6,SEC24C,ATP6V1H,NOS3,SYNPR,MAGEL2,KPNB1,GSDMD,GAD1,SCG5,OCA2,SAR1B,APP,ATP9B,CACNA2D1,PI4KB,USE1,TLR1,AVPR1B,LMAN1L,TICAM2,UQCC1,LRRK2,PLD1,MGST1,NEDD4L,SNX13,RABGEF1,KIT,SPAG9,ANTXR1,WNT5A,ARFGEF2,CTSK,SCFD1,VPS53,BTBD8,DIAPH3,SH3BP5,APOH,STEAP1B,CEMIP,HLA-DQA2,HYAL4,PDE4B,KIF13A,GABRB3,GOSR2,LAMP2,SSPN,KIRREL3,GABRG2,F5,RABGAP1L,DENND2B,XRCC5,GFRA1,INPP4A,GRIA4,RAB18,MGRN1,WLS,PRCP,MYO1B,ZPLD1,SRI,BDNF,LDLRAD4,MALRD1,TLR6,KIF16B,RAB11FIP2,ENSG00000290217,CHRNB4,MYOF,SH3KBP1,MYO5A,WDFY4,TFRC,VPS13A,OSBPL6,GRIA3,RAB31,CLTC,ATP6V0A4,DNAJC6,ROCK1,TAX1BP1,PML,PRKG1,SBF2,MAGI2,SPG21,ANKFY1,FNDC3A,SLC30A10,AP4S1,CALCRL,SCAMP1,ANPEP,PRLR,CLVS1,NCALD,TMEM30A,MYRIP,UNC13C,PMEPA1,TTPA,GRIA2,VDAC2,VPS13B*
GO:0150034	distal axon	0.04295701471018198	*APBB2,KCNQ5,CDH8,USH2A,EXOC4,TIAM2,CPEB4,CLCN3,PREX1,ZNF804A,MYH14,SYNJ1,GAD1,WHRN,APP,IGF2BP1,PALLD,LRRK2,TIAM1,ERC2,NGEF,TRPC5,FXR2,GPRIN1,TANC1,USP9X,MYO5A,CLASP2,ITGA4,ELAVL4,NIN,UNC13C*
GO:0097708	intracellular vesicle	0.045771416132198185	*APBB2,EPN2,MREG,NOSTRIN,STX8,GARS1,RAPGEF6,BLTP3B,TRAPPC9,TF,RCSD1,SPINK5,ATP7B,CAMK2B,GRIP1,ARHGAP11A-*

			SCG5,PGM1,DCT,SLC38A9,CD93,DSC1,MAP6,TBC1D16,DIPK2A,FAM3C,PRKN,F13A1,PIK3C2B,SLC5A7,IFT172,SERPINI1,SLC17A6,SDF4,TRAK2,AGL,ENPEP,CALCR,TM9SF2,CLCN3,ZFYVE16,PLG,CIDEB,MARCHF1,MYO1D,HMGB1,CDIP1,TGFB2,TBC1D5,ATP11A,SLC15A4,GOLPH3,NCF2,PIK3C3,USP10,SND1,ATP13A4,SNX10,TMEM230,STON2,HUWE1,GSN,TLR8,HPS3,ITPR1,TRAPPC10,MR1,F8,VPS8,MGAM,EPHA3,ERP29,UVRAG,PTPRN2,LYN,USP6,SEC24C,ATP6V1H,NOS3,SYNPR,MAGEL2,KPNB1,GSDMD,GAD1,SCG5,OCA2,SAR1B,APP,ATP9B,CACNA2D1,PI4KB,USE1,TLR1,AVPR1B,LMAN1L,TICAM2,UQCC1,LRRK2,PLD1,MGST1,NEDD4L,SNX13,RABGEF1,KIT,SPAG9,ANTXR1,WNT5A,ARFGEF2,CTSK,SCFD1,VPS53,BTBD8,DIAPH3,SH3BP5,APOH,STEAP1B,CEMIP,HLA-DQA2,HYAL4,PDE4B,KIF13A,GABRB3,GOSR2,LAMP2,SSPN,KIRREL3,GABRG2,F5,RABGAP1L,DENND2B,XRCC5,GFRA1,INPP4A,GRIA4,RAB18,MGRN1,WLS,PRCP,MYO1B,ZPLD1,SRI,BDNF,LDLRAD4,MALRD1,TLR6,KIF16B,RAB11FIP2,ENSG00000290217,CHRNB4,MYOF,SH3KBP1,MYO5A,WDFY4,TFRC,VPS13A,OSBPL6,GRIA3,RAB31,CLTC,ATP6V0A4,DNAJC6,ROCK1,TAX1BP1,PML,PRKG1,SBF2,MAGI2,SPG21,ANKFY1,FNDC3A,SLC30A10,AP4S1,CALCRL,SCAMP1,ANPEP,PRLR,CLVS1,NCALD,TMEM30A,MYRIP,UNC13C,PMEPA1,TTPA,GRIA2,VDAC2,VPS13B
GO:0098793	presynapse	0.0496970309437255	*CHRNB2,KCNQ5,CDH8,USH2A,PRKN,NLGN4X,SLC5A7,SLC17A6,CNTN1,CLCN3,GABRA5,ZNF804A,BRAF,TRIO,TMEM230,STON2,PCLO,ANO2,PTPRN2,GRIK1,ATP6V1H,SYNPR,SYNJ1,GAD1,APP,HCN1,LRRK2,ARFGEF2,CTNNB1,ERC2,BTBD8,FXR2,CHRNB3,KCNJ3,NTNG1,PDE4B,KIRREL3,RGS7BP,BDNF,CDH2,TANC1,GABRB1,MYOF,SLC6A13,LRFN2,BLTP1,ATP6V0A4,DNAJC6,CDH9,SCAMP1,CTBP2,PPFIA1,UNC13C,KCTD16*

Table S16. Group of 115 rDNA-contacting genes that are involved in morphogenesis in Mel Z cells grown on Matrigel and are co-expressed with 133 different lincRNAs. The genes belong to the group of genes that exhibited an increase in the number of contacts with rDNA clusters (group of 2527 genes in Figure 4). The search was performed in https://maayanlab.cloud/Enrichr/enrich# for lncHUB lncRNA Co-Expression.

Term	Overlap	Adjusted P-value	Genes
LINC00943	8/100	8.145816288548667E-5	*DNM3;OLFM1;GPM6A;SNAP25;KIF5C;MAP2;SH3GL2;PACSIN1*
LINC00641	8/100	8.145816288548667E-5	*DNM3;GPM6A;KIF5C;MAP2;CTNND2;CDH10;ZDHHC17;SH3GL2*
CADM3-AS1	7/100	5.180865287604976E-4	*DNM3;GPM6A;SNAP25;KIF5C;MAP2;CTNND2;SH3GL2*

LINC02488	7/100	5.180865287604976E-4	*GPM6A;CRB1;PTPRZ1;MAP2;CTNND2;CDH10;EPHB1*
MIR124-2HG	7/100	5.180865287604976E-4	*DNM3;GPM6A;SNAP25;KIF5C;MAP2;CDH10;SH3GL2*
BASP1-AS1	6/100	0.0017707857636734944	*SNAP25;MYO10;KIF5C;STXBP1;SH3GL2;PACSIN1*
ZNF32-AS3	6/100	0.0017707857636734944	*DNM3;GPM6A;MAP2;CDH10;PTPN11;EPHB1*
SRGAP3-AS1	6/100	0.0017707857636734944	*DNM3;GPM6A;KIF5C;MAP2;CTNND2;CDH10*
PTPRD-AS2	6/100	0.0017707857636734944	*OLFM1;SNAP25;SYT1;KIF5C;SH3GL2;PACSIN1*
LINC00945	6/100	0.0017707857636734944	*GPM6A;CRB1;PTPRZ1;MAP2;CTNND2;DISC1*
LINC01677	6/100	0.0017707857636734944	*GPM6A;CRB1;MAP2;CTNND2;CDH10;EPHB1*
ATP13A5-AS1	6/100	0.0017707857636734944	*GPM6A;CRB1;PTPRZ1;MAP2;CTNND2;EPHB1*
LRRC7-AS1	6/100	0.0017707857636734944	*GPM6A;KIF5C;MAP2;CTNND2;CDH10;SH3GL2*
ADGRL3-AS1	6/100	0.0017707857636734944	*DNM3;GPM6A;CRB1;MAP2;CTNND2;CDH10*
OPCML-IT1	6/100	0.0017707857636734944	*GPM6A;CRB1;PTPRZ1;MAP2;TMEM108;EPHB1*
MIR9-3HG	6/100	0.0017707857636734944	*GPM6A;CRB1;MAP2;CTNND2;CDH10;EPHB1*
SEC23A-AS1	6/100	0.0017707857636734944	*ITGB1;NEDD4;PARVA;BVES;FERMT2;CORO1C*
LINC02598	6/100	0.0017707857636734944	*GPM6A;CRB1;MAP2;CTNND2;CDH10;EPHB1*
LINC02588	6/100	0.0017707857636734944	*GPM6A;CRB1;PTPRZ1;MAP2;CTNND2;CDH10*
LINC00928	6/100	0.0017707857636734944	*GPM6A;CRB1;MAP2;CTNND2;CDH10;EPHB1*
LINC00652	5/100	0.007293320097424206	*GPM6A;CRB1;MAP2;CDH10;EPHB1*
VWC2L-IT1	5/100	0.007293320097424206	*DNM3;GPM6A;MAP2;CDH10;SH3GL2*
TMEM72-AS1	5/100	0.007293320097424206	*DNM3;GPM6A;MAP2;CTNND2;SH3GL2*

RORB-AS1	5/100	0.007293320097424206	*SLC23A2;OLFM1;SNAP25;KIF5C;PACSIN1*
LINC00381	5/100	0.007293320097424206	*GPM6A;PTPRZ1;CTNND2;CDH10;GNA12*
NCAM1-AS1	5/100	0.007293320097424206	*DNM3;GPM6A;MAP2;CTNND2;CDH10*
FRMPD3-AS1	5/100	0.007293320097424206	*GPM6A;MAP2;CTNND2;CDH10;EPHB1*
SOX21-AS1	5/100	0.007293320097424206	*GPM6A;CRB1;MAP2;CTNND2;CDH10*
CTNNA2-AS1	5/100	0.007293320097424206	*GPM6A;CRB1;MAP2;CTNND2;CDH10*
LINC01690	5/100	0.007293320097424206	*GPM6A;CRB1;MAP2;CTNND2;CDH10*
TRAF3IP2-AS1	5/100	0.007293320097424206	*GPM6A;MAP2;CTNND2;CDH10;ZDHHC17*
LRRC8C-DT	5/100	0.007293320097424206	*MACF1;MYCBP2;DOCK7;WDPCP;DISC1*
LINC00689	5/100	0.007293320097424206	*GPM6A;CRB1;MAP2;CDH10;EPHB1*
LINC01750	5/100	0.007293320097424206	*GPM6A;ANKRD24;KIF5C;CTNND2;CDH10*
TGFB2-AS1	5/100	0.007293320097424206	*CTNND2;DOCK7;GNA12;ANOS1;MAP4K4*
GSN-AS1	5/100	0.007293320097424206	*MACF1;NRP2;DOCK7;PARVA;MYO9B*
ARHGEF7-AS2	5/100	0.007293320097424206	*DNM3;GPM6A;KIF5C;MAP2;CDH10*
CXXC4-AS1	5/100	0.007293320097424206	*DNM3;GPM6A;MAP2;CTNND2;CDH10*
LINC00461	5/100	0.007293320097424206	*GPM6A;CRB1;MAP2;CTNND2;CDH10*
LINC02283	5/100	0.007293320097424206	*GPM6A;CRB1;MAP2;CDH10;EPHB1*
LINC00290	5/100	0.007293320097424206	*OLFM1;SNAP25;KIF5C;SH3GL2;PACSIN1*
XIRP2-AS1	5/100	0.007293320097424206	*NRP2;MYO10;DOCK7;PACSIN2;PLXNC1*
GRM5-AS1	5/100	0.007293320097424206	*SNAP25;KIF5C;MAP2;SH3GL2;PACSIN1*

LINC00599	5/100	0.0072933200974242 06	*DNM3;GPM6A;MAP2;CDH10;SH3GL2*
FOXG1-AS1	5/100	0.0072933200974242 06	*GPM6A;PTPRZ1;MAP2;CTNND2;CDH10*
LINC02389	5/100	0.0072933200974242 06	*SNAP25;SYT1;KIF5C;SH3GL2;PACSIN1*
LINC01152	5/100	0.0072933200974242 06	*GPM6A;MAP2;CTNND2;DOCK7;WDPCP*
MAPT-AS1	5/100	0.0072933200974242 06	*GPM6A;CRB1;MAP2;CTNND2;CDH10*
LINC02318	5/100	0.0072933200974242 06	*GPM6A;CRB1;CTNND2;EPHB1;SH3GL2*
FBXW7-AS1	5/100	0.0072933200974242 06	*SNAP25;MEF2C;SYT1;MYCBP2;PACSIN1*
LINC00644	5/100	0.0072933200974242 06	*GPM6A;MAP2;CTNND2;CDH10;SH3GL2*
LINC01963	5/100	0.0072933200974242 06	*DNM3;GPM6A;MAP2;CTNND2;CDH10*
LINC02192	5/100	0.0072933200974242 06	*OLFM1;SNAP25;SYT1;SH3GL2;PACSIN1*
LINC01974	5/100	0.0072933200974242 06	*GPM6A;MAP2;CTNND2;CDH10;SH3GL2*
LINC02338	5/100	0.0072933200974242 06	*GPM6A;SNAP25;CTNND2;SH3GL2;PACSIN1*
LINC01651	5/100	0.0072933200974242 06	*GPM6A;CRB1;MAP2;CDH10;EPHB1*
ANKRD62P1-PARP4P3	4/100	0.0291142468988512 7	*DNM3;GPM6A;CRB1;CTNND2*
CYYR1-AS1	4/100	0.0291142468988512 7	*MACF1;MYCBP2;WDPCP;PHIP*
LINC02520	4/100	0.0291142468988512 7	*CRB1;RELN;MAP2;EPHB1*
LINC01310	4/100	0.0291142468988512 7	*GPM6A;KIF5C;CTNND2;CDH10*
LINC02347	4/100	0.0291142468988512 7	*SNAP25;SYT1;STXBP1;PACSIN1*
LY86-AS1	4/100	0.0291142468988512 7	*SNAP25;SYT1;SH3GL2;PACSIN1*
KIRREL3-AS3	4/100	0.0291142468988512 7	*OLFM1;SNAP25;SYT1;PACSIN1*

HTR5A-AS1	4/100	0.0291142468988512 7	*SNAP25;SYT1;SH3GL2;PACSIN1*
MRPS9-AS2	4/100	0.0291142468988512 7	*DNM3;GPM6A;CTNND2;SH3GL2*
LINC00297	4/100	0.0291142468988512 7	*NRP2;MYO10;PACSIN2;PLXNC1*
LINC00320	4/100	0.0291142468988512 7	*DNM3;GPM6A;CTNND2;SH3GL2*
LINC00237	4/100	0.0291142468988512 7	*GPM6A;CRB1;MAP2;CDH10*
MYT1L-AS1	4/100	0.0291142468988512 7	*SNAP25;SYT1;KIF5C;PACSIN1*
PRICKLE2-AS3	4/100	0.0291142468988512 7	*MACF1;PARVA;FERMT2;SYNE1*
SLC39A12-AS1	4/100	0.0291142468988512 7	*GPM6A;CTNND2;SH3GL2;PACSIN1*
LINC00368	4/100	0.0291142468988512 7	*DNM3;GPM6A;MAP2;CDH10*
LINC01907	4/100	0.0291142468988512 7	*GPM6A;CTNND2;GNA12;MAP4K4*
LAMA4-AS1	4/100	0.0291142468988512 7	*ILK;PARVA;BVES;FERMT2*
WDR11-AS1	4/100	0.0291142468988512 7	*GPM6A;CTNND2;CDH10;SH3GL2*
LINC00511	4/100	0.0291142468988512 7	*GPM6A;CRB1;PTPRZ1;MYO10*
LINC00578	4/100	0.0291142468988512 7	*ILK;PARVA;BVES;FERMT2*
LINC02607	4/100	0.0291142468988512 7	*SNAP25;SYT1;SH3GL2;PACSIN1*
LINC00326	4/100	0.0291142468988512 7	*RELN;GDNF;PACSIN2;ARAP1*
LINC00396	4/100	0.0291142468988512 7	*GPM6A;PTPRZ1;CTNND2;GNA12*
GNG12-AS1	4/100	0.0291142468988512 7	*DOCK7;ILK;PARVA;FERMT2*
SLC6A1-AS1	4/100	0.0291142468988512 7	*GPM6A;CRB1;MAP2;CDH10*
GPC5-AS2	4/100	0.0291142468988512 7	*LATS1;MEF2C;PHIP;SYNE1*

GRM3-AS1	4/100	0.02911424689885127	*SNAP25;KIF5C;SH3GL2;PACSIN1*
DSCAM-IT1	4/100	0.02911424689885127	*GPM6A;CRB1;MAP2;EPHB1*
LINC00702	4/100	0.02911424689885127	*ILK;PARVA;BVES;FERMT2*
ETV5-AS1	4/100	0.02911424689885127	*DOCK7;WDPCP;PLXNC1;DISC1*
OBI1-AS1	4/100	0.02911424689885127	*GPM6A;CTNND2;PTPN11;SH3GL2*
ID2-AS1	4/100	0.02911424689885127	*GPM6A;CTNND2;PTPN11;SH3GL2*
L3MBTL2-AS1	4/100	0.02911424689885127	*MYO10;DOCK7;PACSIN2;MYO9B*
LINC00440	4/100	0.02911424689885127	*OLFM1;SNAP25;SYT1;STXBP1*
CPB2-AS1	4/100	0.02911424689885127	*GPM6A;CRB1;MAP2;CDH10*
NEXN-AS1	4/100	0.02911424689885127	*ILK;PARVA;BVES;FERMT2*
MYCBP2-AS1	4/100	0.02911424689885127	*LATS1;MACF1;MYCBP2;PHIP*
KCNMA1-AS1	4/100	0.02911424689885127	*ILK;PARVA;BVES;FERMT2*
NDUFA6-DT	4/100	0.02911424689885127	*GPM6A;ANKRD24;MAP2;CDH10*
NRXN2-AS1	4/100	0.02911424689885127	*GPM6A;MAP2;CTNND2;CDH10*
LINC01351	4/100	0.02911424689885127	*GPM6A;MAP2;CTNND2;CDH10*
CADM2-AS1	4/100	0.02911424689885127	*DNM3;GPM6A;CRB1;CTNND2*
MYLK-AS1	4/100	0.02911424689885127	*ILK;PARVA;BVES;FERMT2*
DENND6A-AS1	4/100	0.02911424689885127	*LATS1;MACF1;MYCBP2;PHIP*
LINC00391	4/100	0.02911424689885127	*GPM6A;MAP2;CTNND2;CDH10*
LINC02008	4/100	0.02911424689885127	*GPM6A;PTPRZ1;MAP2;CDH10*

LINC01168	4/100	0.0291142468988512 7	*DNM3;SNAP25;MAP2;SH3GL2*
FLNC-AS1	4/100	0.0291142468988512 7	*ILK;PARVA;BVES;FERMT2*
LINC01998	4/100	0.0291142468988512 7	*GPM6A;CTNND2;GNA12;SH3GL2*
PPM1K-DT	4/100	0.0291142468988512 7	*GPM6A;CTNND2;CDH10;SH3GL2*
LINC02234	4/100	0.0291142468988512 7	*GPM6A;PTPRZ1;MAP2;CDH10*
LINC02144	4/100	0.0291142468988512 7	*GPM6A;MAP2;CTNND2;CDH10*
HAS2-AS1	4/100	0.0291142468988512 7	*DOCK7;GNA12;CORO1C;MAP4K4*
LINC00500	4/100	0.0291142468988512 7	*GPM6A;CTNND2;SH3GL2;PACSIN1*
EDIL3-DT	4/100	0.0291142468988512 7	*ITGB1;ULK2;FERMT2;CORO1C*
LINC02060	4/100	0.0291142468988512 7	*GPM6A;MAP2;CTNND2;CDH10*
LINC01332	4/100	0.0291142468988512 7	*SNAP25;SYT1;SH3GL2;PACSIN1*
NAV2-AS2	4/100	0.0291142468988512 7	*NRP2;MYO10;DOCK7;PLXNC1*
LINC00499	4/100	0.0291142468988512 7	*GPM6A;CTNND2;SH3GL2;PACSIN1*
CCND2-AS1	4/100	0.0291142468988512 7	*GPM6A;MAP2;CTNND2;IL1RAPL1*
MRGPRF-AS1	4/100	0.0291142468988512 7	*ILK;PARVA;BVES;FERMT2*
ADGRA1-AS1	4/100	0.0291142468988512 7	*DNM3;GPM6A;MAP2;CDH10*
LINC02682	4/100	0.0291142468988512 7	*ULK2;ILK;BVES;FERMT2*
LINC02735	4/100	0.0291142468988512 7	*MYO10;PACSIN2;MYO9B;PLXNC1*
LINC00507	4/100	0.0291142468988512 7	*SNAP25;SYT1;SH3GL2;PACSIN1*
DLGAP1-AS4	4/100	0.0291142468988512 7	*SNAP25;SYT1;SH3GL2;PACSIN1*

LINC00508	4/100	0.02911424689885127	*SNAP25;SYT1;SH3GL2;PACSIN1*
LINC02282	4/100	0.02911424689885127	*GPM6A;PTPRZ1;MAP2;CDH10*
JAKMIP2-AS1	4/100	0.02911424689885127	*GPM6A;MAP2;CTNND2;CDH10*
LINC01581	4/100	0.02911424689885127	*ILK;PARVA;BVES;FERMT2*
LINC02134	4/100	0.02911424689885127	*GPM6A;PTPRZ1;MAP2;CDH10*
LINC01572	4/100	0.02911424689885127	*GPM6A;PTPRZ1;MAP2;CDH10*
LINC01476	4/100	0.02911424689885127	*SNAP25;SYT1;SH3GL2;PACSIN1*
LINC00906	4/100	0.02911424689885127	*GPM6A;CRB1;CTNND2;EPHB1*
LINC01081	4/100	0.02911424689885127	*ILK;PARVA;BVES;FERMT2*
LINC01109	4/100	0.02911424689885127	*GPM6A;PTPRZ1;CTNND2;CDH10*

Table S17. Group of 115 rDNA-contacting genes that are involved in morphogenesis in Mel Z cells grown on Matrigel and are co-expressed with 127 different miRNAs. The genes belong to the group of genes that exhibited an increase in the number of contacts with rDNA clusters (group of 2527 genes in Figure 4).

Term	Overlap	Adjusted P-value	Genes
hsa-miR-208a	27/1772	1.0798250454171075E-4	*GPM6A;STAU2;STXBP1;PTPRO;IQGAP1;NYAP2;TRAK1;PPP3CA;FGD4;SNX2;MAP2;PDPN;PLXNC1;DISC1;MAP4K4;BCL11B;SYT1;MYO10;VDR;UNC5C;PTPN11;DNM3;ARHGAP32;NFASC;CTTN;ULK2;WDPCP*
hsa-miR-208b	27/1772	1.0798250454171075E-4	*GPM6A;STAU2;STXBP1;PTPRO;IQGAP1;NYAP2;TRAK1;PPP3CA;FGD4;SNX2;MAP2;PDPN;PLXNC1;DISC1;MAP4K4;BCL11B;SYT1;MYO10;VDR;UNC5C;PTPN11;DNM3;ARHGAP32;NFASC;CTTN;ULK2;WDPCP*
hsa-miR-3545-5p	26/1750	1.61429313904412E-4	*ITGB1;STAU2;SDC2;TRAK1;FSTL4;FGD4;PARD6B;SNX2;VSIG1;KIF5C;EPHB1;PACSIN1;SYT1;SEMA4D;MYO10;UNC5C;PARVA;SEMA4F;PTPN11;ARHGAP32;NFASC;XK;TBC1D20;GDNF;ULK2;PHIP*
hsa-miR-4661-5p	21/1241	1.94801514952166E-4	*ITGB1;SNAP25;MEF2C;SYT1;UNC5B;STAU2;STXBP1;UNC5C;PARVA;MYO9B;ANK3;NYAP2;ZDHHC17;GLI3;FSTL4;PPP3CA;PARD6B;FGD6;ULK2;DISC1;MET*
hsa-	21/127	2.36541041639373	*SEMA4D;UNC5B;STAU2;RYK;ST8SIA2;MYCBP2;*

miR-548ad	2	37E-4	*LEF1;UNC5C;PTPN11;NYAP2;MAPK14;FGD1;FGD6;TBC1D20;GDNF;GNA12;CDHR3;ULK2;EPHB1;SLC26A5;MAP4K4*
hsa-miR-154	26/1842	2.6111039830259284E-4	*STXBP1;GLI3;FGD4;PARD6B;FGD6;VSIG1;KIF5C;MAP2;TMEM108;DISC1;MAP4K4;SS18;MEF2C;SEMA4D;UNC5B;PARVA;MYO9B;SEMA4F;ANK3;LATS1;NFASC;XK;ULK2;PHIP;FERMT2;FRYL*
hsa-miR-28-3p	24/1654	3.2961178200603496E-4	*SNAP25;SS18;MYO10;STAU2;SEMA3D;CTNND2;SEMA6D;ST8SIA2;UNC5C;PARVA;PTPN11;ZDHHC17;DNM3;OLFM1;ARHGAP32;ADAM17;FGD6;XK;KIF5C;MAP2;NEDD4;GAS2;PHIP;DISC1*
hsa-miR-648	26/1888	3.3367932179978775E-4	*ITGB1;GPM6A;MACF1;STAU2;SEMA3D;ADCY1;FGD4;PARD6B;KIF5C;MAP2;PLEKHG4B;CDH26;BVES;MAP4K4;BCL11B;MYO10;SEMA6D;ST8SIA2;UNC5C;PARVA;LATS1;NFASC;GDNF;CTTN;NEDD4;PHIP*
hsa-miR-3681	24/1673	3.674443909916396E-4	*MACF1;ST8SIA2;MYCBP2;PARVA;IQGAP1;PROX1;ADCY1;GLI3;FSTL4;DNM3;PARD6B;FGD6;XK;CTTN;KIF5C;MAP2;PLEKHG4B;PACSIN2;GAS2;DISC1;BVES;SH3GL2;FRYL;MAP4K4*
hsa-miR-4684-3p	26/1953	4.869081419274838E-4	*ITGB1;SDC2;ADCY1;TRAK1;PARD6B;VSIG1;MAP2;GNA12;PLXNC1;BVES;NEO1;MAP4K4;MEF2C;ANKRD27;SYT1;SEMA4D;MYO10;SEMA6D;PTPN11;ANK3;DNM3;TBC1D20;CDHR3;ULK2;WDPCP;MET*
hsa-miR-3146	23/1682	8.173703280313195E-4	*SNAP25;BCL11B;MYO10;VDR;ST8SIA2;ANK3;IQGAP1;ADCY1;LRP2;GLI3;CORO1C;SYNE1;DNM3;NELL2;PPP3CA;FGD4;NFASC;EFNB3;XK;KIF5C;GNA12;DISC1;BVES*
hsa-miR-142-3p	22/1584	9.299504280756833E-4	*SS18;MEF2C;SEMA4D;VDR;STAU2;SEMA3D;SEMA6D;UNC5C;ANK3;NYAP2;FSTL4;PPP3CA;ADAM17;PARD6B;RELN;XK;CTTN;MAP2;CCDC88C;PLEKHG4B;GAS2;PHIP*
hsa-miR-4774-5p	24/1845	0.0011129098021115972	*SNAP25;CRB1;MEF2C;BCL11B;SYT1;STAU2;CTNND2;SDC2;DOCK7;UNC5C;PARVA;PTPN11;LRP2;FSTL4;CORO1C;DNM3;NELL2;NFASC;GDNF;NEFL;BVES;MET;FERMT2;MAP4K4*
hsa-miR-4506	25/1967	0.0011129098021115972	*PTPRQ;SEMA3D;MYCBP2;PTPRO;LEF1;NYAP2;FGD4;FGD6;VSIG1;PLEKHG4B;PLXNC1;DISC1;BVES;PACSIN1;MYO10;SEMA6D;PARVA;SEMA4F;DNM3;SHTN1;ARHGAP32;NFASC;CDHR3;PHIP;MET*
hsa-miR-3613-5p	21/1519	0.0011954469337505304	*MEF2C;MYO10;PTPRQ;STAU2;SEMA3D;PTPRO;UNC5C;PARVA;ANK3;FSTL4;FGD1;CORO1C;DNM3;NELL2;PARD6B;RELN;XK;GDNF;CDHR3;ULK2;PHIP*
hsa-miR-548al	11/508	0.0013281083478976193	*OLFM1;PPP3CA;SNX2;SEMA6D;PLEKHG4B;UNC5C;PARVA;ADCY1;TRAK1;MAPK14;MET*
hsa-miR-103b	21/1545	0.0013874485232107756	*ITGB1;SNAP25;DBNL;STXBP1;ST8SIA2;MYCBP2;DOCK7;LEF1;UNC5C;SEMA4F;IQGAP1;ADCY1;LRP2;GLI3;GLI2;DNM3;SHTN1;PARD6B;RELN;NEFL;DISC1*
hsa-miR-325	19/1334	0.001577301905677769	*GPM6A;SYT1;STAU2;CTNND2;TRAK1;ZDHHC17;LATS1;DNM3;PPP3CA;FGD4;PARD6B;FGD6;GDNF;KIF5C;MAP2;NEDD4;NEFL;DISC1;SLC26A5*
hsa-miR-4317	23/1852	0.002047854753604591	*SS18;BCL11B;STAU2;ST8SIA2;MYCBP2;PTPRO;UNC5C;PARVA;PROX1;CORO1C;OLFM1;PPP3CA;NFASC;CTTN;CCDC88C;PDPN;CDHR3;NEFL;DISC1;BVES;NEO1;SH3GL2;MAP4K4*

hsa-miR-563	19/1387	0.0020691845201630645	*MEF2C;SEMA4D;VDR;PTPRQ;SEMA6D;ST8SIA2;UNC5C;IQGAP1;ADCY1;MAPK14;CORO1C;NELL2;ARHGAP32;FGD4;NEDD4;NEFL;DISC1;PACSIN1;MAP4K4*
hsa-miR-934	20/1507	0.002121817557287678	*MEF2C;SYT1;SEMA4D;MYO10;SEMA3D;PARVA;PTPN11;ANK3;ADCY1;NYAP2;ZDHHC17;SYNE1;NELL2;SHTN1;NFASC;KIF5C;PDPN;DISC1;NEO1;PACSIN1*
hsa-miR-4704-5p	23/1869	0.0021277713655722336	*SEMA3D;SDC2;UNC5C;PARVA;MYO9B;SEMA4F;IQGAP1;TRAK1;ZDHHC17;GLI3;DNM3;OLFM1;EFNB3;FGD6;PTPRZ1;NEDD4;PLEKHG4B;GNA12;GAS2;PHIP;PLXNC1;MET;FRYL*
hsa-miR-4703-3p	22/1749	0.002144133460205012	*GPM6A;CRB1;MYO10;PTPRO;PARVA;PTPN11;ADCY1;FSTL4;PREX2;DNM3;NELL2;PARD6B;NFASC;PTPRZ1;NEDD4;PLEKHG4B;FAT1;CDHR3;ULK2;PHIP;PLXNC1;DISC1*
hsa-miR-503	23/1875	0.002188625772022606	*MACF1;BCL11B;SEMA4D;UNC5B;SEMA3D;SEMA6D;STXBP1;MYCBP2;PARVA;ANK3;ADCY1;TRAK1;RTN4;FSTL4;ARHGAP32;PARD6B;FGD6;KIF5C;PLEKHG4B;GNA12;PLXNC1;BVES;NEO1*
hsa-miR-4785	10/477	0.0024230846098763728	*FGD4;FGD6;GDNF;KIF5C;MAP2;CCDC88C;PTPRO;PLEKHG4B;MYO9B;PTPN11*
hsa-miR-590-5p	23/1900	0.002489737811679338	*MEF2C;BCL11B;PARVA;TRAK1;ZDHHC17;RTN4;CORO1C;LATS1;NELL2;ARHGAP32;PPP3CA;FGD4;NFASC;FGD6;XK;NEDD4;PLEKHG4B;CDHR3;PHIP;PLXNC1;DISC1;BVES;FERMT2*
hsa-miR-21	23/1900	0.002489737811679338	*MEF2C;BCL11B;PARVA;TRAK1;ZDHHC17;RTN4;CORO1C;LATS1;NELL2;ARHGAP32;PPP3CA;FGD4;NFASC;FGD6;XK;NEDD4;PLEKHG4B;CDHR3;PHIP;PLXNC1;DISC1;BVES;FERMT2*
hsa-miR-190b	22/1779	0.0024908212013114656	*ITGB1;MEF2C;MYO10;STAU2;SEMA3D;MYCBP2;UNC5C;ANK3;ADCY1;LRP2;NELL2;ARHGAP32;PPP3CA;FGD4;NFASC;FGD6;KIF5C;NEDD4;GNA12;PHIP;DISC1;MAP4K4*
hsa-miR-190	22/1779	0.0024908212013114656	*ITGB1;MEF2C;MYO10;STAU2;SEMA3D;MYCBP2;UNC5C;ANK3;ADCY1;LRP2;NELL2;ARHGAP32;PPP3CA;FGD4;NFASC;FGD6;KIF5C;NEDD4;GNA12;PHIP;DISC1;MAP4K4*
hsa-miR-502-3p	23/1907	0.0024994320918285333	*MEF2C;ANKRD27;MYO10;STAU2;SEMA3D;ST8SIA2;PTPRO;UNC5C;PARVA;IQGAP1;ARAP1;TRAK1;GLI3;GLI2;DNM3;SHTN1;PPP3CA;NFASC;XK;TBC1D20;CTTN;PACSIN2;DISC1*
hsa-miR-501-3p	23/1907	0.0024994320918285333	*MEF2C;ANKRD27;MYO10;STAU2;SEMA3D;ST8SIA2;PTPRO;UNC5C;PARVA;IQGAP1;ARAP1;TRAK1;GLI3;GLI2;DNM3;SHTN1;PPP3CA;NFASC;XK;TBC1D20;CTTN;PACSIN2;DISC1*
hsa-miR-4445	22/1826	0.0032345257527068654	*GPM6A;MEF2C;BCL11B;SYT1;MYO10;SEMA3D;STXBP1;ST8SIA2;LEF1;PARVA;PTPN11;NYAP2;CORO1C;ARHGAP32;NFASC;FGD6;KIF5C;NEDD4;PLEKHG4B;ULK2;PLXNC1;DISC1*
hsa-miR-591	22/1831	0.00333102216865896	*VDR;STXBP1;ST8SIA2;PTPRO;UNC5C;PARVA;PTPN11;TRAK1;ZDHHC17;FSTL4;NELL2;OLFM1;FGD4;NFASC;FGD6;TBC1D20;KIF5C;MAP2;PLEKHG4B;DISC1;MET;MAP4K4*
hsa-miR-891a	9/431	0.0039582907714094916	*DNM3;PPP3CA;PARD6B;FGD6;ST8SIA2;CDHR3;BVES;NEO1;RTN4*
hsa-miR-	21/1754	0.00429416026021521	*MEF2C;SEMA4D;UNC5B;STAU2;CTNND2;STXBP1;ST8SIA2;UNC5C;PARVA;ADCY1;PREX2;ARHGA*

3171			*P32;PARD6B;FGD6;XK;TBC1D20;KIF5C;PHIP;DISC1;MET;FRYL*
hsa-miR-770-5p	22/1893	0.004549376442212718	*MACF1;ANKRD27;BCL11B;SEMA4D;STAU2;UNC5C;PARVA;ANK3;ADCY1;TRAK1;GLI2;ARHGAP32;PPP3CA;XK;PLEKHG4B;GAS2;CDHR3;DISC1;EPHB1;MET;PACSIN1;MAP4K4*
hsa-miR-4712-5p	22/1893	0.004549376442212718	*MACF1;ANKRD27;BCL11B;SEMA4D;STAU2;UNC5C;PARVA;ANK3;ADCY1;TRAK1;GLI2;ARHGAP32;PPP3CA;XK;PLEKHG4B;GAS2;CDHR3;DISC1;EPHB1;MET;PACSIN1;MAP4K4*
hsa-miR-4636	8/357	0.004600144158029497	*ARHGAP32;PARD6B;SDC2;PTPRO;PACSIN2;GAS2;ZDHHC17;FSTL4*
hsa-miR-4671-3p	19/1528	0.004715440980420776	*SNAP25;MYO10;STAU2;SEMA3D;ST8SIA2;UNC5C;ADCY1;NYAP2;MAPK14;SYNE1;ARHGAP32;PPP3CA;SNX2;XK;PLEKHG4B;ULK2;MET;FERMT2;MAP4K4*
hsa-miR-4694-5p	22/1906	0.004794071244432018	*GPM6A;UNC5B;SEMA6D;PTPRO;UNC5C;PARVA;ADCY1;TRAK1;GLI3;NELL2;ARHGAP32;FGD4;RELN;TBC1D20;PTPRZ1;MAP2;NEDD4;PDPN;PLEKHG4B;CDHR3;ULK2;NEFL*
hsa-miR-4484	22/1907	0.004794071244432018	*ITGB1;SEMA4D;MYO10;PTPRQ;SEMA3D;UNC5C;PARVA;IQGAP1;PROX1;NYAP2;GLI2;NELL2;ARHGAP32;TBC1D20;GDNF;NEDD4;PDPN;ENPP2;DISC1;BVES;FERMT2;FRYL*
hsa-miR-921	22/1922	0.0052288002744939705	*SEMA4D;MYO10;MYCBP2;UNC5C;PARVA;PTPN11;ANK3;LRP2;TRAK1;GLI3;FSTL4;DNM3;OLFM1;PARD6B;XK;TBC1D20;GDNF;KIF5C;ULK2;NEFL;PLXNC1;DISC1*
hsa-miR-3144-3p	19/1548	0.0052288002744939705	*MACF1;MEF2C;MYO10;VDR;STAU2;SEMA3D;ANK3;NYAP2;TRAK1;SYNE1;DNM3;ARHGAP32;FGD6;NEDD4;PLEKHG4B;PHIP;BVES;SH3GL2;PACSIN1*
hsa-miR-4772-5p	22/1927	0.005325977661279499	*MYO10;PTPRO;UNC5C;PARVA;PTPN11;TRAK1;ZDHHC17;CORO1C;SHTN1;ARHGAP32;PARD6B;TBC1D20;KIF5C;MAP2;NEDD4;PDPN;PLEKHG4B;PLXNC1;DISC1;BVES;SH3GL2;FRYL*
hsa-miR-628-3p	19/1555	0.005413822798958523	*GPM6A;MEF2C;SYT1;STAU2;CTNND2;TRAK1;PPP3CA;FGD4;PARD6B;FGD6;XK;TBC1D20;KIF5C;MAP2;NEDD4;NEFL;DISC1;SLC26A5;MAP4K4*
hsa-miR-296-3p	22/1950	0.005956587812861253	*SYT1;MYO10;CTNND2;SEMA6D;SDC2;ST8SIA2;PTPRO;PARVA;IQGAP1;ADCY1;GLI3;FSTL4;NELL2;NFASC;FGD6;VSIG1;KIF5C;MAP2;CDHR3;ULK2;PHIP;PLXNC1*
hsa-miR-3201	21/1826	0.006033983857487964	*MEF2C;SEMA4D;SEMA3D;UNC5C;NYAP2;DNM3;NELL2;NFASC;EFNB3;FGD6;XK;TBC1D20;KIF5C;NEDD4;PDPN;GNA12;GAS2;PHIP;PLXNC1;DISC1;MAP4K4*
hsa-miR-4791	21/1826	0.006033983857487964	*MEF2C;SEMA4D;SEMA3D;UNC5C;NYAP2;DNM3;NELL2;NFASC;EFNB3;FGD6;XK;TBC1D20;KIF5C;NEDD4;PDPN;GNA12;GAS2;PHIP;PLXNC1;DISC1;MAP4K4*
hsa-miR-3923	17/1335	0.006073693692857485	*GPM6A;MEF2C;SYT1;SEMA6D;MYCBP2;PTPRO;SEMA4F;ADCY1;ZDHHC17;LATS1;DNM3;NELL2;NFASC;GDNF;PHIP;SH3GL2;SLC26A5*
hsa-miR-3618	20/1709	0.006318954717518279	*GPM6A;SYT1;SEMA4D;MYO10;SEMA3D;SEMA6D;PARVA;PTPN11;ANK3;ADCY1;LRP2;NYAP2;ZDHHC17;NELL2;SHTN1;NFASC;KIF5C;NEDD4;PDPN;NEO1*
hsa-	19/158	0.00642147347444	*MEF2C;VDR;STAU2;SEMA6D;ANK3;ADCY1;LRP2*

miR-875-5p	9	71496	;NYAP2;TRAK1;SYNE1;DNM3;NELL2;ARHGAP32;NFASC;FGD6;NEDD4;PHIP;SH3GL2;PACSIN1
hsa-miR-556-5p	21/1841	0.0064214734744471496	DBNL;MYO10;VDR;STXBP1;PTPN11;ANK3;ADCY1;NYAP2;DNM3;NELL2;ARHGAP32;PARD6B;RELN;TBC1D20;KIF5C;MAP2;CDHR3;NEFL;BVES;MET;FRYL
hsa-miR-3177-5p	21/1852	0.0068716208112335276	SS18;MYO10;PTPRQ;SEMA3D;CTNND2;ST8SIA2;MYCBP2;UNC5C;PARVA;SEMA4F;TRAK1;ZDHHC17;PREX2;RELN;CCDC88C;PDPN;GNA12;PHIP;PLXNC1;NEO1;MAP4K4
hsa-miR-4460	19/1601	0.006887246361970675	SYT1;SEMA4D;STAU2;SDC2;PARVA;SEMA4F;IQGAP1;TRAK1;FSTL4;FGD4;PARD6B;NFASC;SNX2;XK;VSIG1;TBC1D20;KIF5C;PLEKHG4B;DISC1
hsa-miR-210	10/584	0.007183640804334111	NELL2;FGD4;MEF2C;ANKRD24;CTTN;PTPRO;PLEKHG4B;TMEM108;ADCY1;CORO1C
hsa-miR-3659	22/1994	0.007241322067497308	SNAP25;MEF2C;ANKRD27;BCL11B;SEMA4D;STAU2;ST8SIA2;ADCY1;NYAP2;TRAK1;GLI3;NFASC;FGD6;XK;PTPRZ1;CCDC88C;PLEKHG4B;GNA12;GAS2;CDHR3;DISC1;MET
hsa-miR-3152-3p	22/1998	0.007241322067497308	SNAP25;SS18;DBNL;SYT1;MYO10;STAU2;SEMA3D;RYK;LEF1;PARVA;NYAP2;TRAK1;PARD6B;NFASC;VSIG1;MAP2;PDPN;GNA12;PHIP;PLXNC1;DISC1;SH3GL2
hsa-miR-4280	19/1624	0.007647767015681702	SS18;ANKRD24;STAU2;DOCK7;UNC5C;ANK3;MAPK14;DNM3;PPP3CA;FGD4;ADAM17;PARD6B;FGD6;TBC1D20;CDHR3;NEFL;PHIP;PLXNC1;MAP4K4
hsa-miR-4714-3p	21/1899	0.008446909727956996	BCL11B;PTPRQ;RYK;PTPN11;ANK3;IQGAP1;ADCY1;TRAK1;GLI3;ARHGAP32;FGD4;XK;MAP2;IL1RAPL1;ULK2;NEFL;PHIP;BVES;EPHB1;PACSIN1;MAP4K4
hsa-miR-508-3p	19/1660	0.009219935021724546	SEMA4D;MYO10;SEMA3D;STXBP1;ST8SIA2;MYCBP2;LEF1;NYAP2;TRAK1;CORO1C;SYNE1;FGD4;FGD6;NEDD4;PLEKHG4B;PLXNC1;DISC1;MET;FERMT2
hsa-miR-184	11/720	0.009325169976975802	NELL2;TBC1D20;SDC2;ST8SIA2;PLEKHG4B;GNA12;ULK2;UNC5C;TRAK1;FSTL4;PACSIN1
hsa-miR-379	19/1664	0.009336081999698716	GPM6A;MACF1;BCL11B;SEMA4D;MYO10;PARVA;GLI3;SYNE1;DNM3;ADAM17;FGD6;XK;GDNF;PTPRZ1;KIF5C;DISC1;NEO1;MET;MAP4K4
hsa-miR-3529	19/1664	0.009336081999698716	GPM6A;MACF1;BCL11B;SEMA4D;MYO10;PARVA;GLI3;SYNE1;DNM3;ADAM17;FGD6;XK;GDNF;PTPRZ1;KIF5C;DISC1;NEO1;MET;MAP4K4
hsa-miR-4732-5p	19/1679	0.01012075455282523	MACF1;SEMA3D;CTNND2;PTPRO;UNC5C;SEMA4F;PTPN11;ANK3;PROX1;GLI3;CORO1C;ARHGAP32;FGD4;PARD6B;EFNB3;GDNF;KIF5C;DISC1;BVES
hsa-miR-3676	10/629	0.010551915754373932	MACF1;FGD6;VSIG1;MYO10;STAU2;CTNND2;STXBP1;PARVA;ADCY1;FSTL4
hsa-miR-324-5p	21/1962	0.011482286154861982	ANKRD27;DBNL;RYK;UNC5C;ANK3;ADCY1;NYAP2;TRAK1;GLI3;FSTL4;SHTN1;ARHGAP32;XK;TBC1D20;GDNF;MAP2;CCDC88C;PLEKHG4B;DISC1;EPHB1;PACSIN1
hsa-miR-4661-	18/1578	0.01161990656537721	SYT1;SEMA4D;MYO10;CTNND2;PARVA;ADCY1;ZDHHC17;FSTL4;NELL2;OLFM1;NFASC;EFNB3;TBC1D20;CTTN;KIF5C;FAT1;ULK2;DISC1

3p			
hsa-miR-4540	16/1329	0.01161990656537721	*BCL11B;UNC5B;STXBP1;MYCBP2;PTPRO;UNC5C;PARVA;TRAK1;CORO1C;ARHGAP32;PPP3CA;FGD4;EFNB3;KIF5C;DISC1;MET*
hsa-miR-4259	21/1971	0.011844601002547916	*SYT1;MYO10;VDR;STXBP1;UNC5C;PTPN11;ARAP1;ADCY1;MAPK14;ZDHHC17;GLI3;FSTL4;NELL2;ARHGAP32;NFASC;FGD6;NEDD4;MAEL;FAT1;CDHR3;DISC1*
hsa-miR-2277-3p	21/1993	0.013011395906315403	*MEF2C;MYO10;SDC2;ST8SIA2;MYCBP2;PTPRO;UNC5C;PARVA;MYO9B;ADCY1;NYAP2;TRAK1;NELL2;SHTN1;GDNF;CTTN;PLEKHG4B;GNA12;WDPCP;PHIP;PACSIN1*
hsa-miR-95	8/452	0.013136427482729146	*DNM3;BCL11B;GDNF;MYO10;PTPN11;IQGAP1;BVES;GLI2*
hsa-miR-3141	12/879	0.01327639504554517	*OLFM1;NFASC;BCL11B;MYO10;KIF5C;STXBP1;LEF1;PLEKHG4B;GNA12;ADCY1;TRAK1;PACSIN1*
hsa-miR-3115	17/1481	0.01327639504554517	*MEF2C;PTPRO;UNC5C;PARVA;ANK3;MAPK14;GLI3;ARHGAP32;FGD4;NFASC;XK;TBC1D20;GDNF;CTTN;KIF5C;PLEKHG4B;CDH26*
hsa-miR-192	19/1740	0.01337739293316085	*MACF1;SEMA4D;MYO10;UNC5B;VDR;PTPRO;UNC5C;PARVA;MYO9B;FSTL4;DNM3;FGD4;NFASC;EFNB3;XK;PLEKHG4B;NEFL;PHIP;DISC1*
hsa-miR-215	19/1740	0.01337739293316085	*MACF1;SEMA4D;MYO10;UNC5B;VDR;PTPRO;UNC5C;PARVA;MYO9B;FSTL4;DNM3;FGD4;NFASC;EFNB3;XK;PLEKHG4B;NEFL;PHIP;DISC1*
hsa-miR-4759	19/1756	0.014571539714409595	*BCL11B;ANK3;GLI3;FSTL4;FGD1;NELL2;ARHGAP32;NFASC;FGD6;VSIG1;GDNF;NEDD4;PLEKHG4B;ENPP2;MAEL;NEFL;CDH26;DISC1;FERMT2*
hsa-miR-4737	8/463	0.014576024140522479	*BCL11B;GDNF;CCDC88C;PLEKHG4B;MYO9B;PLXNC1;MET;FSTL4*
hsa-miR-3935	18/1637	0.015442788733541716	*ITGB1;UNC5C;PARVA;ANK3;NYAP2;TRAK1;MAPK14;DNM3;NELL2;ARHGAP32;FGD4;NFASC;FGD6;VSIG1;ULK2;PHIP;DISC1;FERMT2*
hsa-miR-151-3p	18/1653	0.01674563707308393	*MEF2C;VDR;STAU2;STXBP1;ST8SIA2;PARVA;PTPN11;IQGAP1;ADCY1;TRAK1;ZDHHC17;NFASC;FGD6;PTPRZ1;MAP2;NEFL;PHIP;DISC1*
hsa-miR-4655-5p	8/483	0.01792483485772756	*OLFM1;MEF2C;BCL11B;VDR;STXBP1;CCDC88C;PLEKHG4B;GNA12*
hsa-miR-4800-3p	6/292	0.018030510313784158	*MEF2C;PLEKHG4B;PHIP;PARVA;ANK3;DISC1*
hsa-miR-4633-5p	17/1551	0.01895743130143315	*MEF2C;SEMA4D;STAU2;SEMA6D;MYCBP2;UNC5C;ANK3;ARAP1;NYAP2;TRAK1;GLI3;DNM3;NFASC;XK;WDPCP;BVES;MAP4K4*
hsa-miR-1268	13/1055	0.01934967157192375	*UNC5B;SEMA3D;STXBP1;ADCY1;RTN4;GLI3;VSIG1;TBC1D20;PLEKHG4B;TMEM108;ULK2;NEFL;PACSIN1*
hsa-miR-1268b	13/1055	0.01934967157192375	*UNC5B;SEMA3D;STXBP1;ADCY1;RTN4;GLI3;VSIG1;TBC1D20;PLEKHG4B;TMEM108;ULK2;NEFL;PACSIN1*
hsa-miR-383	20/1953	0.019465125568092115	*MEF2C;UNC5B;STAU2;PTPRO;UNC5C;PARVA;TRAK1;ZDHHC17;CORO1C;SHTN1;NFASC;TBC1D20;GDNF;KIF5C;MAP2;PLEKHG4B;PLXNC1;DISC1;MET;FRYL*

hsa-miR-3677-3p	9/599	0.019525999364577523	*NELL2;ARHGAP32;NFASC;DBNL;ST8SIA2;PLEKHG4B;ADCY1;CORO1C;PACSIN1*
hsa-miR-1225-5p	18/1691	0.01954636261721669	*SEMA4D;MYO10;SEMA6D;ST8SIA2;DOCK7;LEF1;PARVA;GLI2;PPP3CA;NFASC;FGD6;GDNF;MAP2;PDPN;ULK2;PHIP;DISC1;FRYL*
hsa-miR-2964a-5p	19/1825	0.01954636261721669	*SS18;BCL11B;MYCBP2;LEF1;UNC5C;SEMA4F;ADCY1;GLI3;FSTL4;GLI2;DNM3;PPP3CA;FGD4;NEDD4;IL1RAPL1;TMEM108;DISC1;BVES;PACSIN1*
hsa-miR-4764-3p	20/1960	0.019559299924760368	*SEMA4D;STAU2;SEMA3D;CTNND2;SDC2;ST8SIA2;LEF1;UNC5C;SEMA4F;NELL2;SHTN1;FGD4;PARD6B;FGD6;XK;PACSIN2;CDHR3;PHIP;DISC1;BVES*
hsa-miR-601	15/1320	0.020755707367336767	*MEF2C;SEMA6D;UNC5C;LPAR3;PARVA;PTPN11;ZDHHC17;FGD1;SYNE1;NELL2;NFASC;TBC1D20;PLEKHG4B;CDHR3;NEFL*
hsa-miR-3147	20/1986	0.021776573190325228	*SNAP25;SEMA4D;STXBP1;ST8SIA2;ILK;UNC5C;PARVA;IQGAP1;ARAP1;TRAK1;NELL2;ARHGAP32;XK;GDNF;PLEKHG4B;GNA12;MAEL;EPHB1;NEO1;PACSIN1*
hsa-miR-4727-3p	17/1588	0.02189357640639766	*MEF2C;SYT1;SEMA4D;SEMA6D;SDC2;ST8SIA2;PARVA;IQGAP1;GLI3;FSTL4;FGD4;PARD6B;NFASC;VSIG1;NEDD4;PLEKHG4B;DISC1*
hsa-miR-4718	19/1867	0.023002807886096512	*MACF1;SS18;BCL11B;CTNND2;PTPRO;UNC5C;ADCY1;NYAP2;MAPK14;DNM3;NFASC;FGD6;XK;VSIG1;MAP2;GNA12;TMEM108;PLXNC1;DISC1*
hsa-miR-1539	10/740	0.023487895586699774	*NELL2;NFASC;VSIG1;ST8SIA2;PLEKHG4B;GNA12;PACSIN2;ULK2;PLXNC1;ADCY1*
hsa-miR-486-5p	19/1873	0.023487895586699774	*CRB1;BCL11B;MYO10;STAU2;PTPRO;UNC5C;PARVA;TRAK1;MAPK14;GLI2;NELL2;ARHGAP32;FGD6;VSIG1;CDHR3;ULK2;PHIP;DISC1;PACSIN1*
hsa-miR-1258	17/1607	0.023487895586699774	*MEF2C;SYT1;SEMA6D;LEF1;PARVA;SEMA4F;PTPN11;ANK3;MAPK14;FGD6;PLEKHG4B;GNA12;TMEM108;DISC1;BVES;EPHB1;PACSIN1*
hsa-miR-645	18/1740	0.023491877379246476	*MACF1;MEF2C;ANKRD27;STAU2;SDC2;ST8SIA2;MYCBP2;PTPRO;PARVA;PTPN11;ADCY1;FSTL4;XK;TBC1D20;NEDD4;CDHR3;CDH26;BVES*
hsa-miR-662	12/979	0.02401885151345067	*UNC5B;SDC2;PDPN;PLEKHG4B;PACSIN2;PLXNC1;ADCY1;EPHB1;GLI3;FSTL4;FGD1;PACSIN1*
hsa-miR-409-5p	15/1354	0.024110224408084125	*GPM6A;MACF1;SEMA4D;STAU2;UNC5C;PARVA;ANK3;TRAK1;ZDHHC17;DNM3;OLFM1;XK;PDPN;PLEKHG4B;SH3GL2*
hsa-miR-4508	13/1106	0.02462843076677841	*BCL11B;SEMA3D;ST8SIA2;ILK;ADCY1;GLI3;FGD1;ARHGAP32;NFASC;FGD6;GDNF;PLEKHG4B;GNA12*
hsa-miR-126	4/153	0.026127239025231425	*NFASC;SEMA4D;SDC2;PARVA*
hsa-miR-4715-5p	17/1631	0.026158041784001336	*SYT1;MYO10;RYK;UNC5C;NYAP2;MAPK14;ZDHHC17;FSTL4;GLI2;DNM3;FGD6;XK;MAP2;NEDD4;CFL1;FAT1;DISC1*
hsa-	18/176	0.02658836439798	*GPM6A;SDC2;SEMA4F;ZDHHC17;GLI3;LATS1;D*

miR-<br>1244	8	8024	*NM3;NELL2;PARD6B;VSIG1;MAP2;NEDD4;CCDC<br>88C;GNA12;MAEL;FAT1;DISC1;FERMT2*
hsa-<br>miR-<br>3713	12/100<br>3	0.02765970152442<br>9432	*DNM3;ARHGAP32;ADAM17;MYO10;PTPRQ;STAU2<br>;SEMA3D;NEDD4;ULK2;NEFL;DISC1;MET*
hsa-<br>miR-<br>585	9/654	0.02833632020280<br>0712	*TBC1D20;KIF5C;PDPN;PLEKHG4B;GNA12;TMEM<br>108;ADCY1;RTN4;GLI3*
hsa-<br>miR-<br>509-3p	15/138<br>8	0.02833632020280<br>0712	*MEF2C;BCL11B;MYO10;PARVA;ANK3;GLI3;FST<br>L4;FGD6;VSIG1;KIF5C;MAP2;ENPP2;CDHR3;N<br>EO1;MET*
hsa-<br>miR-<br>3619-<br>3p	17/166<br>2	0.02980474819294<br>58	*CRB1;SYT1;VDR;PARVA;ADCY1;RTN4;FSTL4;G<br>LI2;ARHGAP32;NFASC;CTTN;MAP2;PDPN;PLEK<br>HG4B;ULK2;DISC1;SH3GL2*
hsa-<br>miR-<br>496	18/181<br>2	0.03208420919858<br>031	*SS18;SEMA4D;MYO10;STAU2;SEMA3D;PARVA;P<br>TPN11;TRAK1;ZDHHC17;FGD1;DNM3;ARHGAP32<br>;FGD4;VSIG1;NEDD4;PLEKHG4B;DISC1;BVES*
hsa-<br>miR-<br>4536	18/182<br>1	0.03349680689548<br>233	*SYT1;CTNND2;SEMA6D;PTPRO;ILK;ADCY1;GLI<br>3;FGD1;NELL2;SHTN1;ARHGAP32;NFASC;FGD6<br>;XK;TBC1D20;CTTN;PDPN;MAP4K4*
hsa-<br>miR-<br>4637	16/155<br>3	0.03366824721567<br>513	*ITGB1;BCL11B;STAU2;SEMA3D;STXBP1;MYCBP<br>2;UNC5C;GLI3;SHTN1;ARHGAP32;PARD6B;MAP<br>2;CDH10;ENPP2;FAT1;PHIP*
hsa-<br>miR-<br>660	18/182<br>4	0.03380272683080<br>998	*MEF2C;SEMA4D;SEMA6D;SDC2;PTPN11;ANK3;I<br>QGAP1;ADCY1;TRAK1;MAPK14;NFASC;KIF5C;N<br>EDD4;PLEKHG4B;MAEL;ULK2;PHIP;BVES*
hsa-<br>miR-<br>323b-<br>5p	16/155<br>5	0.03383247342581<br>586	*BCL11B;SYT1;SEMA4D;MYO10;UNC5C;MYO9B;P<br>TPN11;ADCY1;TRAK1;OLFM1;PPP3CA;FGD4;GD<br>NF;KIF5C;DISC1;FRYL*
hsa-<br>miR-<br>1278	17/170<br>3	0.03592668998297<br>6184	*MACF1;SEMA6D;UNC5C;PARVA;IQGAP1;ADCY1;<br>LRP2;DNM3;PPP3CA;FGD4;PARD6B;NFASC;XK;<br>PLEKHG4B;DISC1;BVES;MET*
hsa-<br>miR-<br>3186-<br>5p	7/463	0.03592668998297<br>6184	*NELL2;NFASC;SDC2;STXBP1;NYAP2;DISC1;SL<br>C26A5*
hsa-<br>miR-<br>3162-<br>3p	17/174<br>7	0.04350622151930<br>697	*GPM6A;BCL11B;SEMA3D;MYCBP2;PTPRO;UNC5C<br>;PARVA;ADCY1;TRAK1;GLI2;PARD6B;XK;GDNF<br>;CDHR3;PHIP;EPHB1;PACSIN1*
hsa-<br>miR-<br>614	6/379	0.04387845628335<br>729	*MEF2C;BCL11B;VDR;PLEKHG4B;ADCY1;GLI3*
hsa-<br>miR-<br>4466	10/843	0.04523433707512<br>875	*BCL11B;SEMA4D;MYO10;VDR;PTPRO;PACSIN2;<br>PARVA;NYAP2;DISC1;MAP4K4*
hsa-<br>miR-<br>1295	10/843	0.04523433707512<br>875	*EFNB3;XK;VSIG1;SDC2;ULK2;ADCY1;NYAP2;B<br>VES;EPHB1;PACSIN1*
hsa-<br>miR-<br>675	10/843	0.04523433707512<br>875	*BCL11B;SEMA4D;MYO10;VDR;PTPRO;PACSIN2;<br>PARVA;NYAP2;DISC1;MAP4K4*
hsa-<br>miR-<br>4423-<br>3p	18/190<br>0	0.04523433707512<br>875	*ITGB1;MEF2C;VDR;CTNND2;ST8SIA2;PTPRO;P<br>ARVA;ADCY1;TRAK1;PPP3CA;FGD4;NEDD4;GNA<br>12;CDHR3;ULK2;FERMT2;FRYL;MAP4K4*
hsa-<br>miR-	16/162<br>6	0.04547275855896<br>3135	*SNAP25;MEF2C;SYT1;VDR;SDC2;PTPN11;ADCY<br>1;LRP2;RTN4;DNM3;ARHGAP32;NFASC;XK;GAS*

4796-5p			*2;BVES;MET*
hsa-miR-550b	18/1910	0.0469925012309845	*SEMA6D;MYCBP2;PARVA;PTPN11;NYAP2;ZDHHC17;CORO1C;SYNE1;PARD6B;GDNF;KIF5C;CDHR3;ULK2;NEFL;PHIP;DISC1;MET;SH3GL2*
hsa-miR-2909	18/1912	0.04729631007951462	*MEF2C;BCL11B;SEMA4D;MYO10;ST8SIA2;PTPRO;PARVA;SEMA4F;PTPN11;IQGAP1;ADCY1;GLI3;NFASC;KIF5C;PLEKHG4B;CDHR3;PHIP;FRYL*
hsa-miR-4281	18/1918	0.04821610459879617	*GPM6A;VDR;ST8SIA2;MYCBP2;UNC5C;PARVA;IQGAP1;ADCY1;TRAK1;GLI3;CORO1C;FGD4;PARD6B;GDNF;CCDC88C;PLEKHG4B;EPHB1;PACSIN1*
hsa-miR-4444	6/393	0.04896481032979729	*ANKRD27;STAU2;GNA12;TRAK1;ZDHHC17;PACSIN1*
hsa-miR-553	8/618	0.0492350803702228	*LATS1;PPP3CA;FGD4;VDR;SDC2;PDPN;GNA12;DISC1*
